# Neuroplasticity in Post-Stroke Aphasia: A Systematic Review and Meta-Analysis of Functional Imaging Studies of Reorganization of Language Processing

**DOI:** 10.1162/nol_a_00025

**Published:** 2020-12-01

**Authors:** Stephen M. Wilson, Sarah M. Schneck

**Affiliations:** Department of Hearing and Speech Sciences, Vanderbilt University Medical Center, Nashville, TN, USA; Department of Hearing and Speech Sciences, Vanderbilt University Medical Center, Nashville, TN, USA

**Keywords:** aphasia, PET, fMRI, neuroplasticity, systematic review, meta-analysis

## Abstract

Recovery from aphasia is thought to depend on neural plasticity, that is, the functional reorganization of surviving brain regions such that they take on new or expanded roles in language processing. We carried out a systematic review and meta-analysis of all articles published between 1995 and early 2020 that have described functional imaging studies of six or more individuals with post-stroke aphasia, and have reported analyses bearing on neuroplasticity of language processing. Each study was characterized and appraised in detail, with particular attention to three critically important methodological issues: task performance confounds, contrast validity, and correction for multiple comparisons. We identified 86 studies describing a total of 561 relevant analyses. We found that methodological limitations related to task performance confounds, contrast validity, and correction for multiple comparisons have been pervasive. Only a few claims about language processing in individuals with aphasia are strongly supported by the extant literature: First, left hemisphere language regions are less activated in individuals with aphasia than in neurologically normal controls; and second, in cohorts with aphasia, activity in left hemisphere language regions, and possibly a temporal lobe region in the right hemisphere, is positively correlated with language function. There is modest, equivocal evidence for the claim that individuals with aphasia differentially recruit right hemisphere homotopic regions, but no compelling evidence for differential recruitment of additional left hemisphere regions or domain-general networks. There is modest evidence that left hemisphere language regions return to function over time, but no compelling longitudinal evidence for dynamic reorganization of the language network.

## INTRODUCTION

Aphasia is an acquired language impairment caused by damage to language regions of the brain, and is one of the most common and debilitating consequences of stroke. Fortunately, most individuals with post-stroke aphasia experience some degree of recovery of language function over time. The pace of recovery is greatest in the first weeks and months (Kertesz & McCabe, 1977; Swinburn, Porter, & Howard, 2004; Yagata et al., 2017), but clinically meaningful gains in language function are possible even years after stroke (Breitenstein et al., 2017; Holland, Fromm, Forbes, & MacWhinney, 2017). Recovery from aphasia is thought to depend on neural plasticity, that is, the functional reorganization of surviving brain regions such that they take on new or expanded roles in language processing (Hartwigsen & Saur, 2019; Turkeltaub, 2019; Stefaniak, Halai, & Lambon Ralph, 2020).

The nature of this putative process of functional reorganization has been of great interest ever since Broca’s (1865) initial speculations on the question over 150 years ago. Before the development of functional imaging, it was generally believed that right hemisphere regions homotopic to damaged left hemisphere language regions were likely to play an important role in recovery. This idea derived from observations that in patients who had recovered from aphasia, new aphasias could be induced by subsequent right hemisphere strokes (Barlow, 1877; Luria, 1963; Basso, Gardelli, Grassi, & Mariotti, 1989), or transiently by anesthetization of the right hemisphere in the Wada procedure (Kinsbourne, 1971). Language reorganization after aphasia was one of the first questions to be addressed in the earliest metabolic imaging studies (Soh, Larsen, Skinhøj, & Lassen, 1978; Meyer, Sakai, Yamaguchi, Yamamoto, & Shaw, 1980; Knopman, Rubens, Selnes, Klassen, & Meyer, 1984; Demeurisse & Capon, 1987). Although limited by the technology of the time, these pioneering studies suggested a more complex picture in which both left and right hemisphere regions contributed to language processing not only in individuals with aphasia, but also in neurologically normal individuals.

The advent of three-dimensional positron emission tomography (PET) in the early 1990s provided a foundation for substantial progress in understanding patterns of functional reorganization of language processing in post-stroke aphasia. In 1995, a German group published a seminal study with striking images suggesting an expanded role for right hemisphere regions in language processing in six individuals who had recovered from Wernicke’s aphasia (Weiller et al., 1995). However, this right hemisphere reorganization hypothesis was soon sharply challenged by another German group whose functional imaging studies suggested that the most critical determinant of successful recovery was return to function of left hemisphere language regions (Heiss et al., 1997; Karbe et al., 1998; Heiss, Kessler, Thiel, Ghaemi, & Karbe, 1999).

Dozens of studies followed in the next two decades, using PET along with functional magnetic resonance imaging (fMRI). The findings from these studies have been highly variable. Some studies have supported a role for the right hemisphere (Rosen et al., 2000; Blank, Bird, Turkheimer, & Wise, 2003; Crinion & Price, 2005; Turkeltaub, Messing, Norise, & Hamilton, 2011), others have reinforced the importance of residual left hemisphere language areas (Saur et al., 2006; Griffis, Nenert, Allendorfer, Vannest, et al., 2017), while still others have suggested that new left hemisphere regions not previously involved in language function may be recruited (Fridriksson, Richardson, Fillmore, & Cai, 2012). Most recently, several studies have suggested that domain-general networks not specifically related to language may play a role in supporting recovery from aphasia (Fridriksson, Bonilha, Baker, Moser, & Rorden, 2010; Brownsett et al., 2014; Geranmayeh, Brownsett, & Wise, 2014). Researchers generally concur that all of these types of mechanisms are likely to play some role in recovery from aphasia, and that the relative importance of different mechanisms probably depends on the location and extent of the left hemisphere lesion, as well as the phase of recovery. Several recent and authoritative reviews have provided a range of complementary perspectives on this literature (Hartwigsen & Saur, 2019; Turkeltaub, 2019; Stefaniak et al., 2020).

The authors of these recent reviews have, quite reasonably, relied on their own expertise to make implicit decisions about which empirical findings to emphasize and which to minimize. In contrast, our approach in the present study is to systematically appraise the strength of the evidence for each reported finding bearing on the functional reorganization of language processing in post-stroke aphasia. We were motivated by the increased focus in the global scientific community on rigor and reproducibility, which has emerged in recent years in response to a growing awareness that many published findings are not reproducible (Ioannidis, 2005; Open Science Collaboration, 2015). In our appraisal of each relevant study, we focused especially on three aspects of methodology that have recently been argued to be critically important. First, individuals with aphasia are likely to experience difficulty performing language tasks, which may lead to task performance confounds in accuracy and/or reaction time, which can have dramatic effects on activation patterns (Binder, Medler, Desai, Conant, & Liebenthal, 2005; Geranmayeh et al., 2014). Second, the contrasts commonly used to map language regions differ markedly in the extent to which they selectively activate left-lateralized perisylvian language regions; therefore, contrast validity needs to be demonstrated in neurologically normal individuals before a contrast can be used to investigate potential reorganization of the language network (Binder, Swanson, Hammeke, & Sabsevitz, 2008; Wilson, Bautista, Yen, Lauderdale, & Eriksson, 2017; Wilson, Yen, & Eriksson, 2018). Third, the analysis of functional imaging data usually involves simultaneous inferences about signal changes in multiple brain regions; therefore it is critically important to correct appropriately for multiple comparisons (Nichols & Hayasaka, 2003); yet many commonly used approaches do not effectively control the false positive rate (Eklund, Nichols, & Knutsson, 2016).

We carried out a systematic review and meta-analysis of all studies published between 1995 and early 2020 that report analyses bearing on neuroplasticity of language processing in post-stroke aphasia. We extracted numerous data items to characterize and appraise the methodology of each study in detail, including but not limited to the three important issues outlined above. We also coded the findings of each study, and we identified patterns across the reported findings, taking into account the methodological quality of each study.

## METHODS

This systematic review and meta-analysis was conducted under the Preferred Reporting Items for Systematic Reviews and Meta-Analyses (PRISMA) guidelines (Moher, Liberati, Tetzlaff, Altman, & PRISMA Group, 2009). The protocol for the review was preregistered on PROSPERO (CRD42018116295) and can be accessed at https://www.crd.york.ac.uk/prospero/display_record.php?ID=CRD42018116295.

### Inclusion Criteria

Studies were included if they met the following five criteria:

(1)  At least six individuals with adult onset post-stroke aphasia were successfully scanned with PET or fMRI.
(2)  At least one language condition and at least one control condition were included.
(3)  The publication was written in English.
(4)  The study was published between 1995 and April 23, 2020, inclusive.
(5)  The study reported one or more second level analyses (i.e., group analyses) of functional imaging data bearing on the functional reorganization of language processing in post-stroke aphasia, as defined in detail below.

These inclusion criteria are quite broad, capturing cross-sectional as well as longitudinal studies. Longitudinal studies could be observational, or they could include speech-language therapy and/or brain stimulation in between time points. The first criterion excludes case studies and small case series, since we sought to restrict our scope to reported generalizations across individuals. The first criterion also excludes studies using other relevant imaging modalities, such as magnetoencephalography, although such studies certainly have potential to contribute to understanding neuroplasticity in aphasia (Breier et al., 2009; Meltzer, Wagage, Ryder, Solomon, & Braun, 2013). The second criterion rules out resting state studies of functional connectivity, which also have considerable potential to contribute to our understanding of neuroplasticity in aphasia (Siegel et al., 2018; Klingbeil, Wawrzyniak, Stockert, & Saur, 2019). The third criterion rules out publications written in languages other than English, although we are not aware of any such publications that would meet our other criteria. The fourth criterion rules out the earliest PET studies, which were considerably limited technically. Note that one earlier three-dimensional PET study (Heiss, Kessler, Karbe, Fink, & Pawlik, 1993) would have met our first three inclusion criteria; however it would not have met the fifth criterion, because the language and control conditions were never compared.

The fifth and final inclusion criterion limits our scope to studies that report analyses that bear on the functional reorganization of language in post-stroke aphasia, which we now define in detail. At the first level, within the individual participant, a relevant analysis must be based on a contrast comparing one or more conditions entailing language processing (e.g., picture naming, semantic decision, etc.) to one or more conditions not involving language processing (e.g., rest, tone decision, etc.) or involving less language processing (e.g., listening to ambiguous sentences vs. listening to unambiguous sentences). Such contrasts are typically intended to identify language regions: either language regions in general or some specific subset of language regions, such as semantic regions.

At the second level, across participants, we identified eight relevant classes of designs that have the potential to be informative regarding neuroplasticity in aphasia. All eight classes involve comparisons of functional activation for language processing derived from first level analyses. The first four classes of designs are cross-sectional, relying on data from a single point in time:

(1)  Comparisons between individuals with aphasia and neurologically normal participants: Such analyses can show whether individuals with aphasia systematically recruit different brain regions to process language than do neurologically normal individuals.
(2)  Comparisons between two distinct groups of individuals with aphasia, where the two groups are defined by criteria such as aphasia type, lesion location, severity, or treatment group assignment: These kinds of analyses are relevant because it is likely that patterns of functional reorganization depend on factors such as these.
(3)  Correlations within a group of individuals with aphasia, between functional activity and a measure of language function, or another relevant variable (e.g., lesion extent): Such analyses also have the potential to reveal how patterns of functional reorganization differ according to individual circumstances, and whether particular patterns of reorganization are associated with relatively good or relatively poor outcomes.
(4)  Contrasts between successful and unsuccessful processing on individual trials in a group of individuals with aphasia (e.g., correct vs. incorrect picture naming): These types of analyses can reveal brain regions that are necessary for successful language processing in individuals with aphasia. A control group is typically not applicable in these types of analyses, since language processing is essentially always successful in neurologically normal individuals.

Longitudinal studies are more difficult, time-consuming, and expensive to conduct than cross-sectional studies, but they have the potential to provide more direct evidence about reorganization of language processing in post-stroke aphasia. Since reorganization is a dynamic process, an optimal investigation of reorganization will necessarily involve a demonstration of change over time, which is only possible in a longitudinal study. Longitudinal studies can investigate spontaneous recovery, or recovery mediated by behavioral or other treatments. Cross-cutting these two possibilities, we identified four relevant classes of longitudinal designs:

(5)  Comparisons between two or more time points in a group of individuals with aphasia.
(6)  Comparisons of change over time between individuals with aphasia and neurologically normal participants: These longitudinal analyses correspond to the first class of cross-sectional analyses described above.
(7)  Comparisons of change over time between two distinct groups of individuals with aphasia, where the two groups are defined by criteria such as aphasia type, lesion location, severity, or treatment group assignment: These longitudinal analyses correspond to the second class of cross-sectional analyses described above.
(8)  Correlations within a group of individuals with aphasia between change over time and a measure of language function, or another relevant variable: Usually, but not always, the behavioral variables in these analyses are measures of change in language function. These longitudinal analyses correspond to the third class of cross-sectional analyses described above.

Most of the analyses belonging to one of these eight classes of second level designs that have been reported in the literature have been either whole brain voxelwise analyses or analyses of signal change in regions of interest (ROIs). However, we also identified several dozen more complicated types of analyses that fell broadly into one of the eight classes; these will be referred to as “complex analyses.” Complex analyses were included in our review, except for those using dynamic causal modeling or structural equation modeling. We believe that although these approaches have potential, they are most appropriate in situations where a small set of relevant regions and connections relevant to a process of interest has been firmly established (Penny, Stephan, Mechelli, & Friston, 2004), which we do not think is the case for our present level of understanding of language in the brain.

Analyses were included in our review whenever the authors of the study drew an explicit generalization across participants, even if an appropriate statistical test was not carried out to support the generalization.

Minor variants of analyses (e.g., addition of a covariate, exclusion of a participant, etc.) that yielded the same or similar results were excluded. A small number of analyses were excluded because they were not described with sufficient detail or clarity to be coded, or because inconsistent reporting of results made the findings unclear.

### Literature Search

**Figure 1.**
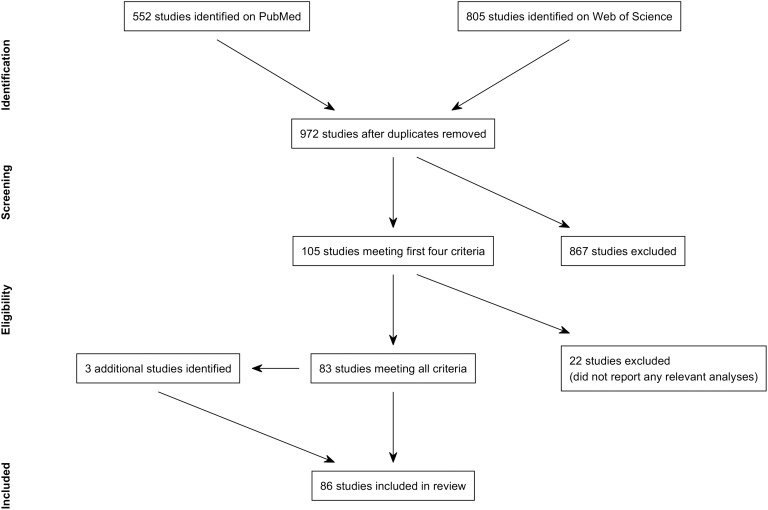
PRISMA flow diagram, modified for our specific procedures.

A PRISMA flow diagram for our review is shown in Figure 1. We searched the PubMed and Web of Science databases for relevant studies on several occasions between February 16, 2018, and April 23, 2020. The search terms for each database are shown in Table 1. The PubMed searches yielded 552 citations and the Web of Science searches yielded 805 citations. The lists were combined and duplicates were removed, yielding 972 citations. We reviewed the titles and abstracts of these citations to determine whether they met the first four criteria; in a few dozen cases, it was necessary to refer also to the full text. We identified 105 studies that met the first four criteria. The full text of these 105 studies was examined in more detail. We determined that 22 studies did not meet the fifth criterion, as follows: neuroimaging used only to localize subsequent brain stimulation (Winhuisen et al., 2005, 2007; Baker, Rorden, & Fridriksson, 2010; Fridriksson, Richardson, Baker, & Rorden, 2011; Abo et al., 2012; Dmochowski et al., 2013); no second level analyses bearing on reorganization (Altamura et al., 2009; Saur et al., 2010; Dietz et al., 2016; Sreedharan, Arun, Sylaja, Kesavadas, & Sitaram, 2019); dynamic causal modeling or structural equation modeling analyses only (Meier, Kapse, & Kiran, 2016; Meier, Johnson, & Kiran, 2018; Meier, Johnson, Pan, & Kiran, 2019; Chu, Meltzer, & Bitan, 2018; Santhanam, Duncan, & Small, 2018); no attempt to generalize across patients (Cherney, Erickson, & Small, 2010; Li & Yang, 2011; Heath et al., 2012; Wilson et al., 2018); described previously reported data without additional analyses that met criteria (Heiss et al., 2013); connectivity analyses only without reference to task (Marcotte, Perlbarg, Marrelec, Benali, & Ansaldo, 2013); psychometric comparisons only (Higgins et al., 2020).

**Table 1.** Search criteria for identifying articles for possible inclusion in the systematic review and meta-analysis

Database	Search criteria
PubMed	(aphasia OR dysphasia OR anomia OR aphasic OR dysphasic OR anomic OR “language impairment” OR “impaired language”) AND (fmri[Title/Abstract] OR “functional mri” OR “functional neuroimaging” OR “functional imaging” OR “functional magnetic resonance imaging” OR “activation” OR “activated” OR pet OR “positron emission tomography”) AND (chronic OR stroke OR post-stroke OR ischemic OR ischemia OR hemorrhage OR hemorrhagic OR vascular) AND “English”[Language] AND (“1995”[Date - Publication]: “2020”[Date - Publication])
Web of Science	(TS=((aphasia OR dysphasia OR anomia OR aphasic OR dysphasic OR anomic OR “language impairment” OR “impaired language”) AND (fmri OR “functional mri” OR “functional neuroimaging” OR “functional imaging” OR “functional magnetic resonance imaging” OR “activation” OR “activated” OR pet OR “positron emission tomography”) AND (chronic OR stroke OR post-stroke OR ischemic OR ischemia OR hemorrhage OR hemorrhagic OR vascular))) AND LANGUAGE: (English) AND DOCUMENT TYPES: (Article) Indexes=SCI-EXPANDED, SSCI, A&HCI, CPCI-S, CPCI-SSH, BKCI-S, BKCI-SSH, ESCI, CCR-EXPANDED, IC Timespan=1995-2020

The remaining 83 studies were included in the review. In the course of evaluating these 83 studies, we identified an additional 3 cited studies that met all criteria (Belin et al., 1996; Blasi et al., 2002; Sharp, Turkheimer, Bose, Scott, & Wise, 2010). Therefore, a total of 86 studies were included in the review (Table 2).

**Table 2.** Studies included in the systematic review and meta-analysis

Author(s)	Year	Title	Journal	DOI
Weiller et al.	1995	Recovery from Wernicke’s aphasia: A positron emission tomographic study	*Annals of Neurology*	10.1002/ana.410370605
Belin et al.	1996	Recovery from nonfluent aphasia after melodic intonation therapy: A PET study	*Neurology*	10.1212/wnl.47.6.1504
Ohyama et al.	1996	Role of the nondominant hemisphere and undamaged area during word repetition in poststroke aphasics: A PET activation study	*Stroke*	10.1161/01.str.27.5.897
Heiss et al.	1997	Speech-induced cerebral metabolic activation reflects recovery from aphasia	*Journal of the Neurological Sciences*	10.1016/s0022-510x(96)00252-3
Karbe et al.	1998	Brain plasticity in poststroke aphasia: What is the contribution of the right hemisphere?	*Brain and Language*	10.1006/brln.1998.1961
Cao, Vikingstad, George, Johnson, & Welch	1999	Cortical language activation in stroke patients recovering from aphasia with functional MRI	*Stroke*	10.1161/01.str.30.11.2331
Heiss et al.	1999	Differential capacity of left and right hemispheric areas for compensation of poststroke aphasia	*Annals of Neurology*	10.1002/1531-8249(199904)45:4<430::aid-ana3>3.0.co;2-p
Kessler, Thiel, Karbe, & Heiss	2000	Piracetam improves activated blood flow and facilitates rehabilitation of poststroke aphasic patients	*Stroke*	10.1161/01.str.31.9.2112
Rosen et al.	2000	Neural correlates of recovery from aphasia after damage to left inferior frontal cortex	*Neurology*	10.1212/wnl.55.12.1883
Blasi et al.	2002	Word retrieval learning modulates right frontal cortex in patients with left frontal damage	*Neuron*	10.1016/s0896-6273(02)00936-4
Leff et al.	2002	A physiological change in the homotopic cortex following left posterior temporal lobe infarction	*Annals of Neurology*	10.1002/ana.10181
Blank et al.	2003	Speech production after stroke: The role of the right pars opercularis	*Annals of Neurology*	10.1002/ana.10656
Cardebat et al.	2003	Behavioral and neurofunctional changes over time in healthy and aphasic subjects: A PET language activation study	*Stroke*	10.1161/01.str.0000099965.99393.83
Sharp et al.	2004	Retrieving meaning after temporal lobe infarction: The role of the basal language area	*Annals of Neurology*	10.1002/ana.20294
Zahn et al.	2004	Recovery of semantic word processing in global aphasia: A functional MRI study	*Cognitive Brain Research*	10.1016/j.cogbrainres.2003.10.021
Crinion & Price	2005	Right anterior superior temporal activation predicts auditory sentence comprehension following aphasic stroke	*Brain*	10.1093/brain/awh659
de Boissezon et al.	2005	Subcortical aphasia: A longitudinal PET study	*Stroke*	10.1161/01.str.0000169947.08972.4f
Connor et al.	2006	Cerebellar activity switches hemispheres with cerebral recovery in aphasia	*Neuropsychologia*	10.1016/j.neuropsychologia.2005.05.019
Crinion et al.	2006	Listening to narrative speech after aphasic stroke: The role of the left anterior temporal lobe	*Cerebral Cortex*	10.1093/cercor/bhj053
Saur et al.	2006	Dynamics of language reorganization after stroke	*Brain*	10.1093/brain/awl090
Meinzer et al.	2008	Functional re-recruitment of dysfunctional brain areas predicts language recovery in chronic aphasia	*NeuroImage*	10.1016/j.neuroimage.2007.10.008
Raboyeau et al.	2008	Right hemisphere activation in recovery from aphasia: Lesion effect or function recruitment?	*Neurology*	10.1212/01.wnl.0000287115.85956.87
Richter et al.	2008	Association between therapy outcome and right-hemispheric activation in chronic aphasia	*Brain*	10.1093/brain/awn043
de Boissezon et al.	2009	Good recovery from aphasia is also supported by right basal ganglia: A longitudinal controlled PET study	*European Journal of Physical & Rehabilitation Medicine*	n/a
Fridriksson et al.	2009	Cortical mapping of naming errors in aphasia	*Human Brain Mapping*	10.1002/hbm.20683
Menke et al.	2009	Imaging short- and long-term training success in chronic aphasia	*BMC Neuroscience*	10.1186/1471-2202-10-118
Specht et al.	2009	Joint independent component analysis of structural and functional images reveals complex patterns of functional reorganisation in stroke aphasia	*NeuroImage*	10.1016/j.neuroimage.2009.06.011
Warren et al.	2009	Anterior temporal lobe connectivity correlates with functional outcome after aphasic stroke	*Brain*	10.1093/brain/awp270
Chau et al.	2010	An fMRI study showing the effect of acupuncture in chronic stage stroke patients with aphasia	*Journal of Acupuncture and Meridian Studies*	10.1016/s2005-2901(10)60009-x
Fridriksson	2010	Preservation and modulation of specific left hemisphere regions is vital for treated recovery from anomia in stroke	*Journal of Neuroscience*	10.1523/jneurosci.2227-10.2010
Fridriksson et al.	2010	Activity in preserved left hemisphere regions predicts anomia severity in aphasia	*Cerebral Cortex*	10.1093/cercor/bhp160
Sharp et al.	2010	Increased frontoparietal integration after stroke and cognitive recovery	*Annals of Neurology*	10.1002/ana.21866
Thompson, den Ouden, Bonakdarpour, Garibaldi, & Parrish	2010	Neural plasticity and treatment-induced recovery of sentence processing in agrammatism	*Neuropsychologia*	10.1016/j.neuropsychologia.2010.06.036
Tyler et al.	2010	Reorganization of syntactic processing following left-hemisphere brain damage: Does right-hemisphere activity preserve function?	*Brain*	10.1093/brain/awq262
van Oers et al.	2010	Contribution of the left and right inferior frontal gyrus in recovery from aphasia: A functional MRI study in stroke patients with preserved hemodynamic responsiveness	*NeuroImage*	10.1016/j.neuroimage.2009.08.057
Papoutsi et al.	2011	Is left fronto-temporal connectivity essential for syntax? Effective connectivity, tractography and performance in left-hemisphere damaged patients	*NeuroImage*	10.1016/j.neuroimage.2011.06.036
Sebastian & Kiran	2011	Task-modulated neural activation patterns in chronic stroke patients with aphasia	*Aphasiology*	10.1080/02687038.2011.557436
Szaflarski et al.	2011	Excitatory repetitive transcranial magnetic stimulation induces improvements in chronic post-stroke aphasia	*Medical Science Monitor*	10.12659/msm.881446
Tyler et al.	2011	Left inferior frontal cortex and syntax: Function, structure and behaviour in patients with left hemisphere damage	*Brain*	10.1093/brain/awq369
Weiduschat et al.	2011	Effects of repetitive transcranial magnetic stimulation in aphasic stroke: A randomized controlled pilot study	*Stroke*	10.1161/strokeaha.110.597864
Allendorfer et al.	2012	Different patterns of language activation in post-stroke aphasia are detected by overt and covert versions of the verb generation fMRI task	*Medical Science Monitor*	10.12659/msm.882518
Fridriksson, Hubbard, et al.	2012	Speech entrainment enables patients with Broca’s aphasia to produce fluent speech	*Brain*	10.1093/brain/aws301
Fridriksson, Richardson, et al.	2012	Left hemisphere plasticity and aphasia recovery	*NeuroImage*	10.1016/j.neuroimage.2011.12.057
Marcotte et al.	2012	Therapy-induced neuroplasticity in chronic aphasia	*Neuropsychologia*	10.1016/j.neuropsychologia.2012.04.001
Schofield et al.	2012	Changes in auditory feedback connections determine the severity of speech processing deficits after stroke	*Journal of Neuroscience*	10.1523/jneurosci.4670-11.2012
Wright et al.	2012	Differentiating hemispheric contributions to syntax and semantics in patients with left-hemisphere lesions	*Journal of Neuroscience*	10.1523/jneurosci.0485-12.2012
Szaflarski et al.	2013	Recovered vs. not-recovered from post-stroke aphasia: The contributions from the dominant and non-dominant hemispheres	*Restorative Neurology and Neuroscience*	10.3233/rnn-120267
Thiel et al.	2013	Effects of noninvasive brain stimulation on language networks and recovery in early poststroke aphasia	*Stroke*	10.1161/strokeaha.111.000574
Abel et al.	2014	Neural underpinnings for model-oriented therapy of aphasic word production	*Neuropsychologia*	10.1016/j.neuropsychologia.2014.03.010
Benjamin et al.	2014	A behavioral manipulation engages right frontal cortex during aphasia therapy	*Neurorehabilitation & Neural Repair*	10.1177/1545968313517754
Brownsett et al.	2014	Cognitive control and its impact on recovery from aphasic stroke	*Brain*	10.1093/brain/awt289
Mattioli et al.	2014	Early aphasia rehabilitation is associated with functional reactivation of the left inferior frontal gyrus: A pilot study	*Stroke*	10.1161/strokeaha.113.003192
Mohr, Difrancesco, Harrington, Evans, & Pulvermüller	2014	Changes of right-hemispheric activation after constraint-induced, intensive language action therapy in chronic aphasia: fMRI evidence from auditory semantic processing	*Frontiers in Human Neuroscience*	10.3389/fnhum.2014.00919
Robson et al.	2014	The anterior temporal lobes support residual comprehension in Wernicke’s aphasia	*Brain*	10.1093/brain/awt373
Szaflarski et al.	2014	Age at stroke determines post-stroke language lateralization	*Restorative Neurology & Neuroscience*	10.3233/rnn-140402
van Hees et al.	2014	Neural activity associated with semantic versus phonological anomia treatments in aphasia	*Brain & Language*	10.1016/j.bandl.2013.12.004
Abel et al.	2015	Therapy-induced brain reorganization patterns in aphasia	*Brain*	10.1093/brain/awv022
Kiran, Meier, Kapse, & Glynn	2015	Changes in task-based effective connectivity in language networks following rehabilitation in post-stroke patients with aphasia	*Frontiers in Human Neuroscience*	10.3389/fnhum.2015.00316
Sandberg, Bohland, & Kiran	2015	Changes in functional connectivity related to direct training and generalization effects of a word finding treatment in chronic aphasia	*Brain & Language*	10.1016/j.bandl.2015.09.002
Geranmayeh et al.	2016	Network dysfunction predicts speech production after left hemisphere stroke	*Neurology*	10.1212/wnl.0000000000002537
Griffis et al.	2016	Interhemispheric plasticity following intermittent theta burst stimulation in chronic poststroke aphasia	*Neural Plasticity*	10.1155/2016/4796906
Sims et al.	2016	The relationships between the amount of spared tissue, percent signal change, and accuracy in semantic processing in aphasia	*Neuropsychologia*	10.1016/j.neuropsychologia.2015.10.019
Darkow et al.	2017	Transcranial direct current stimulation effects on neural processing in post-stroke aphasia	*Human Brain Mapping*	10.1002/hbm.23469
Geranmayeh et al.	2017	Domain-general subregions of the medial prefrontal cortex contribute to recovery of language after stroke	*Brain*	10.1093/brain/awx134
Griffis, Nenert, Allendorfer, & Szaflarski	2017	Linking left hemispheric tissue preservation to fMRI language task activation in chronic stroke patients	*Cortex*	10.1016/j.cortex.2017.08.031
Griffis, Nenert, Allendorfer, Vannest, et al.	2017	The canonical semantic network supports residual language function in chronic post-stroke aphasia	*Human Brain Mapping*	10.1002/hbm.23476
Harvey et al.	2017	Functional reorganization of right prefrontal cortex underlies sustained naming improvements in chronic aphasia via repetitive transcranial magnetic stimulation	*Cognitive & Behavioral Neurology*	10.1097/wnn.0000000000000141
Nardo et al.	2017	Less is more: Neural mechanisms underlying anomia treatment in chronic aphasic patients	*Brain*	10.1093/brain/awx234
Nenert et al.	2017	Neuroimaging correlates of post-stroke aphasia rehabilitation in a pilot randomized trial of constraint-induced aphasia therapy	*Medical Science Monitor*	10.12659/msm.902301
Qiu et al.	2017	Evidence of cortical reorganization of language networks after stroke with subacute Broca’s aphasia: A blood oxygenation level dependent-functional magnetic resonance imaging study	*Neural Regeneration Research*	10.4103/1673-5374.198996
Skipper-Kallal et al.	2017a	Functional activation independently contributes to naming ability and relates to lesion site in post-stroke aphasia	*Human Brain Mapping*	10.1002/hbm.23504
Skipper-Kallal et al.	2017b	Right hemisphere remapping of naming functions depends on lesion size and location in poststroke aphasia	*Neural Plasticity*	10.1155/2017/8740353
Dietz et al.	2018	The feasibility of improving discourse in people with aphasia through AAC: Clinical and functional MRI correlates	*Aphasiology*	10.1080/02687038.2018.1447641
Hallam et al.	2018	Task-based and resting-state fMRI reveal compensatory network changes following damage to left inferior frontal gyrus	*Cortex*	10.1016/j.cortex.2017.10.004
Nenert et al.	2018	Longitudinal fMRI study of language recovery after a left hemispheric ischemic stroke	*Restorative Neurology & Neuroscience*	10.3233/rnn-170767
Pillay et al.	2018	The neural basis of successful word reading in aphasia	*Journal of Cognitive Neuroscience*	10.1162/jocn_a_01214
Szaflarski et al.	2018	A feasibility study of combined intermittent theta burst stimulation and modified constraint-induced aphasia therapy in chronic post-stroke aphasia	*Restorative Neurology & Neuroscience*	10.3233/rnn-180812
van de Sandt-Koenderman, Orellana, van der Meulen, Smits, & Ribbers	2018	Language lateralisation after Melodic Intonation Therapy: An fMRI study in subacute and chronic aphasia	*Aphasiology*	10.1080/02687038.2016.1240353
van Oers et al.	2018	Etiology of language network changes during recovery of aphasia after stroke	*Scientific Reports*	10.1038/s41598-018-19302-4
Barbieri, Mack, Chiappetta, Europa, & Thompson	2019	Recovery of offline and online sentence processing in aphasia: Language and domain-general network neuroplasticity	*Cortex*	10.1016/j.cortex.2019.06.015
Johnson et al.	2019	Treatment-related changes in neural activation vary according to treatment response and extent of spared tissue in patients with chronic aphasia	*Cortex*	10.1016/j.cortex.2019.08.016
Kristinsson et al.	2019	Brain-derived neurotrophic factor genotype-specific differences in cortical activation in chronic aphasia	*Journal of Speech, Language, & Hearing Research*	10.1044/2019_jslhr-l-rsnp-19-0021
Purcell et al.	2019	Re-learning to be different: Increased neural differentiation supports post-stroke language recovery	*NeuroImage*	10.1016/j.neuroimage.2019.116145
Sreedharan, Chandran, et al.	2019	Self-regulation of language areas using real-time functional MRI in stroke patients with expressive aphasia	*Brain Imaging & Behavior*	10.1007/s11682-019-00106-7
Hartwigsen et al.	2020	Short-term modulation of the lesioned language network	*eLife*	10.7554/elife.54277
Stockert et al.	2020	Dynamics of language reorganization after left temporo-parietal and frontal stroke	*Brain*	10.1093/brain/awaa023

### Data Extraction and Appraisal

Five categories of data items were extracted from each study, relating to (1) participants; (2) imaging; (3) conditions; (4) contrasts; and (5) analyses. Data items could be obtained from the article itself, from any supplementary material, and from any source directly referenced in the study (e.g., previous studies describing the same dataset).

We created an interactive relational database for entering and organizing data, using *postgresql*, *python*, and *django*. Both authors independently read and reviewed all 86 studies. For each study, one author read the study first and coded it in the database. The other author then read the study, reviewed the initial coding, and generated a list of potential edits. We then met to discuss the study, resolve any discrepancies, and make all necessary edits. This procedure was started in January, 2018, and completed in July, 2020, with seven studies published in 2019 and the first few months of 2020 being incorporated during the revision process after an initial round of peer review.

Limitations were evaluated with respect to many of the data items in each of the five categories, and were classified as minor, moderate, or major, according to our assessment of their likely impact. Minor limitations were defined as those that would be unlikely to impact the findings of the study. Moderate limitations were defined as those that could potentially limit the interpretation of the findings. Major limitations were defined as those that bring into question the veracity of the findings or preclude the interpretation of the findings with respect to the questions posed by our study.

All limitations were defined with respect to the questions posed by our study, not the aims of the individual studies. Therefore, not all limitations are inherent flaws, because certain study elements may be appropriate for the questions being addressed, even though they may pose limitations with respect to our questions. Furthermore, it is worth noting that it is probably impossible to conduct a study without limitations. For example, it is intrinsically difficult, if not impossible, to avoid task performance confounds when individuals with aphasia are asked to perform language tasks. Therefore, the fact that all studies to date have limitations in this respect does not mean that study designs are flawed, but simply suggests that there are challenges yet to be overcome.

We acknowledge that the appraisal of limitations and their severity is inherently subjective, and we respect that other researchers may have different but well motivated opinions. We have made our complete coding of each included study available (see Supplementary Table S16 in the online supporting information located at https://www.mitpressjournals.org/doi/suppl/10.1162/nol_a_00025), so it should be feasible for other researchers to analyze our dataset in different ways, according to their own views of what is important.

### Participants

We extracted 17 data items to characterize the participants included in each study, the nature of their aphasia, the nature of their strokes and the regions damaged; and to appraise the extent to which this information was provided (Table 3).

**Table 3.** Participants: Data items extracted for characterization and appraisal

Data item	
1	What language did the participants speak?
2	What were the inclusion criteria for the individuals with aphasia? (e.g., lesion location and/or extent; aphasia type and/or severity; preserved functions necessary for task performance)
3	How many individuals with aphasia participated? Were any excluded, and if so, for what reason? How many controls participated?
4	Were any of the participants included in any previous studies?
5	Is age reported for patients and controls, and matched? How old were the patients? (mean, standard deviation, median, range, as available)
6	Is sex reported for patients and controls, and matched? How many of the patients were male and how many were female?
7	Is handedness reported for patients and controls, and matched? How many of the patients were right-handed, left-handed, or something else?
8	Is time post-stroke onset reported and appropriate to the study design? What was the time post-onset? (mean, standard deviation, median, range, as available)
9	To what extent is the nature of the aphasia characterized? (comprehensive battery of scores/severity and type/severity/type/not at all)
10	How was language function evaluated?
11	What was the patients’ aphasia severity?
12	What was the patients’ aphasia type?
13	Did patients have only a single stroke? (yes/no/not stated)
14	What was the etiology of the strokes? (ischemic/hemorrhagic/mixed/not stated)
15	To what extent is the lesion distribution characterized? (individual lesions shown/lesion overlay shown/extent and location/extent/location/not at all)
16	How large were the patients’ lesions? (mean, standard deviation, median, range, as available)
17	Where were the patients’ lesions?

Inclusion criteria were coded only insofar as they entailed a focused study population, that is, inclusion or exclusion based on variables such as lesion location, aphasia type or severity, or specific abilities or deficits. Inclusion criteria that were presumed common to all studies, whether stated or not, were not coded: for instance, that participants were native or fluent speakers of the language under investigation, did not have significant previous neurological history or dementia, were sufficiently medically stable to be scanned, were able to at least minimally follow directions, and so on.

Numerical data items were coded as reported if a measure of central tendency or a range was provided. If individual measures were provided in a table of participants, then we extracted the range from that data.

Limitations were assessed with respect to the nature of the cohort(s) included, and the extent to which participants were adequately characterized. If the number of individuals with aphasia included was at least a dozen but less than two dozen, this was considered a minor limitation, while if there were less than a dozen participants with aphasia, this was considered a moderate limitation. If time post-onset was not fully reported, this was considered a minor limitation, but if participants at different stages of recovery (acute, subacute, chronic) were conflated, this was considered a moderate limitation. Aphasia was considered to be adequately characterized if a comprehensive battery of scores was provided for each patient, documenting performance on language measures typically used for aphasia subtype diagnosis (e.g., spontaneous speech, comprehension, naming, repetition, etc.). In the absence of this, if aphasia severity and aphasia type were reported, this was considered a minor limitation, but if only severity, or only type, were reported, this was counted as two minor limitations, while if neither severity nor type were reported, this was considered a moderate limitation. Lesion location was considered to be satisfactorily characterized if individual lesions were shown, or if a lesion overlay was provided. In the absence of either of these, if extent and location were reported, this was considered a minor limitation, but if only extent, or only location, were reported, this was counted as two minor limitations, while if neither extent nor location were reported, this was considered a moderate limitation. All other limitations pertained to missing information regarding age, sex, handedness, stroke history, or stroke type, or group differences between patients and controls on demographic variables, and were considered to be minor.

### Imaging

We extracted 11 data items to characterize the basic design (i.e., imaging modality, study timing) of each study, and the extent to which data acquisition and basic preprocessing and analysis steps were adequately described and appropriate (Table 4).

**Table 4.** Imaging: Data items extracted for characterization and appraisal

Data item	
1	What is the imaging modality? If PET, what metabolic parameter is estimated?
2	Is the study cross-sectional or longitudinal? If the study is longitudinal, is it a study of spontaneous recovery, a treatment study in the chronic period, or a treatment study in the period during which spontaneous recovery would also be expected?
3	If the study is longitudinal, at what time point(s) were imaging data acquired?
4	If the study is longitudinal, was there any intervention between the time points at which imaging data were acquired?
5	Is the make and model of the scanner described?
6	Is the design blocked or event-related?
7	Is the timing of stimulus presentation (e.g., block length, trials per block) and image acquisition (e.g., number of volumes, repetition time) clearly described and appropriate?
8	Are the imaging acquisition parameters, including coverage, adequately described and appropriate?
9	Is preprocessing and intrasubject coregistration adequately described and appropriate?
10	Is first level model fitting adequately described and appropriate?
11	Is intersubject normalization adequately described and appropriate?

*Note*. PET = positron emission tomography.

Most limitations related to these data items were considered to be minor, generally reflecting missing or incomplete information, or failure to address the potential impact of lesions on intersubject registration (Brett, Leff, Rorden, & Ashburner, 2001). However, some more serious issues were identified with the timing of stimulus presentation and image acquisition, and/or model fitting, which were considered moderate limitations. These specific concerns are described under Imaging in the Results section.

### Conditions

We extracted 6 data items to characterize the conditions included in each study and to appraise their feasibility for individuals with aphasia (Table 5). Conditions were coded even if they were not used in any included analyses.

**Table 5.** Conditions: Data items extracted for characterization and appraisal

Data item	
1	Are the conditions (as a whole) clearly described?
2	For each condition, what is the condition?
3	What type of response is required (button press/word/multiple words/sentence/other/none; overt/covert)?
4	How many times was the condition repeated per scanning session (PET measurements, blocks, or events)?
5	Were all groups at all time points able to perform the task (if any)?
6	Were all individuals at all time points able to perform the task (if any)?

*Note*. PET = positron emission tomography.

If the description of the conditions lacked detail or clarity, this was considered a minor limitation, except in one case where it was considered a moderate limitation, as described under Conditions in the Results section.

For all conditions requiring a response, we attempted to determine whether participants were able to perform the required task. We separately assessed whether each task could be performed by all groups (e.g., patients, controls) at all time points, and whether it could be performed by all individuals at all time points.

For forced-choice tasks, ability to perform the task was defined as performance statistically above chance. For tasks requiring linguistic output, ability to perform the task was defined as production of correct responses on at least 10% of trials. This was based on the reasoning that if patients could perform the task even a small fraction of the time, they were probably engaging in the task as intended. For tasks involving covert responses in the scanner, performance was assessed on the basis of equivalent overt tasks performed outside the scanner, if carried out and reported.

In the absence of sufficient reported behavioral data, statements by authors that all individuals could perform a task, or inclusion criteria requiring ability to perform a task were considered to justify “Yes” answers, but only if other information provided about the participants, such as aphasia subtype diagnoses or an aphasia battery, clearly supported the plausibility of the statement.

If behavioral data showed that not all groups, or not all participants, could perform a task, this was considered a moderate limitation, since it is difficult to interpret imaging data without confirmation that participants were engaged in the intended cognitive-linguistic processes. If there was insufficient information to determine whether all groups, or all participants, could perform a task, this was also considered a moderate limitation, for the same reason. Conditions that did not involve a response (e.g., listening to sentences) were coded as “Not applicable,” which was considered a minor limitation, because although any intended cognitive-linguistic processes could still not be confirmed, at least there was no possibility of overt failure to perform a task.

### Contrasts

We extracted 12 data items to characterize the contrasts computed in each study, and to appraise the effectiveness of their control conditions and their validity in identifying language regions (Table 6). Contrasts were coded only if they were used in one or more included analyses. If the description of the contrast(s) lacked detail or clarity, this was considered a minor limitation in all cases.

**Table 6.** Contrasts: Data items extracted for characterization and appraisal

Data item	
1	Are the contrasts (as a whole) clearly described?
2	What is the language condition?
3	What is the control condition?
4	Are the language and control conditions matched for visual demands?
5	Are the language and control conditions matched for auditory demands?
6	Are the language and control conditions matched for motor demands?
7	Are the language and control conditions matched for cognitive demands?
8	Is accuracy matched between the language and control tasks for all groups at all time points?
9	Is reaction time matched between the language and control tasks for all groups at all time points?
10	Are control data reported in the paper, or in a previous publication that is cited?
11	Does the contrast selectively activate plausible relevant language regions in neurologically normal individuals?
12	Are activations lateralized in neurologically normal individuals?

Contrasts were coded as to whether the language and control conditions were matched for visual, auditory, motor, and cognitive demands. These assessments were made leniently: as long as both conditions made broadly similar demands on the system in question, a contrast was considered matched. For instance, scrambled pictures were considered to be matched in visual demands to pictures of real objects, even though real pictures would entail additional higher-level visual object processing. Mismatches in visual, auditory, motor, or cognitive demands were considered moderate limitations, since contrasts that are not matched for these basic features would necessarily activate sensory, motor, or cognitive regions, in addition to any language regions that may be activated.

We next evaluated whether the language and control conditions were matched in terms of accuracy (or other relevant measures of task performance, such as the number of words produced in an open-ended task). For contrasts involving covert responses in the scanner, any overt responses recorded outside the scanner were considered equivalent, if carried out and reported, otherwise behavioral data were considered to be not reported. The following questions were evaluated in the order stated (because sometimes more than one could apply). (1) If the language and control conditions were incommensurate in their task requirements, in the sense that the control condition was rest, a non-linguistic condition such as finger tapping, or a linguistic condition requiring a different type of response, this was coded as “N/A, tasks not comparable,” which was considered a moderate limitation. (2) If the language condition did not include a task (e.g., listening to narratives), this was coded as “N/A, no behavioral measure,” which was considered a moderate limitation. We think that researchers could reasonably disagree as to whether absence of a task constitutes a limitation, but our position is that it does, because it precludes any assurance that the contrast is balanced for cognitive demands. (3) If the language and control conditions both required comparable responses, but behavioral data were not reported (or were not acquired, in the case of covert tasks), this was coded as “Unknown, not reported,” which was considered a moderate limitation. (4) If behavioral data were reported for both conditions but not compared statistically, this was coded as “Appear similar,” “Appear mismatched,” or “Unknown, no test” depending on our judgment as to whether there was an actual accuracy difference. If the conditions appeared similar, this was considered a minor limitation, otherwise it was considered a moderate limitation. (5) If accuracy was compared across conditions and differed significantly, this was coded as “No, different,” which was considered a moderate limitation. (6) If concrete steps were taken to match accuracy, but accuracy was still not matched, this would have been coded as “No, attempt made” and would have been considered a minor limitation, but this did not occur in any first level analyses (this situation did occur at the second level, as described later). (7) If accuracy was compared across conditions and did not differ, this was coded as “Yes, matched.” (8) Other situations that were considered not to constitute limitations were contrasts limited to correct trials only (“Yes, correct trials only”) and contrasts that were mismatched by design (“No, by design”), such as contrasts between correct and incorrect trials.

The language and control conditions were then compared in terms of reaction time, along much the same lines. The only major difference was that contrasts without tasks (e.g., listening to narrative speech versus listening to reversed speech) were coded as “N/A, no timeable task,” and as long as the language and control conditions were commensurate, this was not considered to be a limitation. Note that contrasts with covert language tasks and incommensurate control tasks (e.g., covert verb generation versus rest) were still coded as “N/A, tasks not comparable,” which was considered a moderate limitation.

The final three data items assessed the validity of each contrast, that is, the extent to which it was demonstrated to activate language regions in neurologically normal individuals (Binder et al., 2008; Wilson et al., 2017; Wilson et al., 2018). First, we asked whether control data for the contrast were reported in the study, or in a previous cited study. A “Yes” answer to this question required control data from at least a dozen participants, with identical methods to those used for the individuals with aphasia, and that the findings be reported in sufficient detail to assess which brain regions were activated by the contrast (usually involving a figure and/or a table). If some control data were provided but these three criteria were not met, then the data item was coded as “Somewhat.” If no control data were provided, the answer was “No.” If the contrast was between successful and unsuccessful language processing (e.g., naming pictures versus failing to name them), then this data item was coded as “Not applicable.” since in most contexts, language processing is essentially always successful in neurologically normal individuals; in these cases, the following two data items were also coded “Not applicable.”

Next, we asked whether the contrast selectively activated plausible relevant language regions in the control group. This would generally be inferior frontal and posterior temporal regions, but the specific regions expected would depend on the particular contrast (Yen, DeMarco, & Wilson, 2019). Activations were required to be selective, that is, language activations should be more prominent than any other activations. This data item was coded as “Yes” when relevant language regions were activated more prominently than any other regions, or as “Somewhat” when some but not all expected language regions were activated, or if activation was not selective. If control data showed that language regions were not selectively activated, the data item was coded as “No.” If there were no control data, or if the control data were insufficient to confirm that language regions were selectively activated, the data item was coded as “Unknown.”

Finally, we asked whether activation in the control group was lateralized to the left hemisphere. While both left and right hemisphere brain regions are involved in language processing, especially for central (semantic) and peripheral (auditory, motor) aspects of language function, it is only left hemisphere damage that reliably results in aphasia, and so paradigms that emphasize lateralized aspects of language processing are much more informative for tracking reorganization in recovery from left hemisphere damage (see Contrast validity in the Discussion section for further discussion). If activations were clearly lateralized (even if there was some right hemisphere activation), this data item was coded as “Yes.” If there was modest asymmetry toward the left hemisphere, the data item was coded as “Somewhat.” If activations were essentially bilateral, the data item was coded “No,” while if there were no control data, or if the control data were insufficient to determine the laterality of the activation, the data item was coded as “Unknown.”

Limitations were assessed simultaneously for the three questions pertaining to contrast validity. If the answer to any of the three questions was “No” or “Unknown,” this was considered a major limitation. In other words, to avoid a major limitation, activation needed (1) to be at least somewhat reported in controls; (2) to at least somewhat activate language regions; and (3) to be at least somewhat lateralized. We think this is a reasonable minimal standard for a contrast to be informative regarding reorganization of language processing. If there were no “No” or “Unknown” answers, then any “Somewhat” answers were counted as moderate limitations; that is, up to three moderate limitations were assessed.

### Analyses

We extracted 20 data items to characterize each reported analysis that met our criteria, and to appraise the second level contrast validity, matching of accuracy and reaction time across the second level contrast, and statistical details, especially the approach taken to correct for multiple comparisons where applicable (Table 7).

**Table 7.** Analyses: Data items extracted for characterization and appraisal

Data item	
1	Are the analyses (as a whole) clearly described?
2	Which first level contrast is the analysis based on?
3	Which of the eight classes of analyses is this?
4	Which group or groups of participants are included?
5	If there is a covariate, what is it?
6	Is the second level contrast valid in terms of the group(s), time point(s), and measures involved?
7	Is accuracy matched across the second level contrast?
8	Is reaction time matched across the second level contrast?
9	Does the analysis involve voxelwise statistics, region(s) of interest (ROI), or something else (Other)?
10	[Voxelwise] What is the search volume?
11	[Voxelwise] How are multiple comparisons across voxels accounted for?
12	[Voxelwise] What software is used for the voxelwise analysis?
13	[Voxelwise] What is the voxelwise *p* threshold?
14	[Voxelwise] What is the cluster extent cutoff?
15	[ROI] Are the ROI(s) anatomical, functional, laterality indices, mixed, or something else?
16	[ROI] How many ROI(s) are there?
17	[ROI] What are the ROI(s)?
18	[ROI] How are the ROI(s) defined?
19	[ROI] If there is more than one ROI, how are the ROIs corrected for multiple comparisons?
20	[Other] Describe the analysis.

If the description of the analyses lacked detail or clarity, this was counted as one or more minor, moderate, or major limitations, depending on the specific concerns, as described under Analyses in the Results section.

We assessed whether second level contrasts were logically constructed to address specific research questions. Issues were identified with some analyses, which were considered moderate or major limitations. These are described under Analyses in the Results section.

We evaluated whether accuracy measures (or other relevant measures of task performance, such as the number of words produced in an open-ended task) were matched across the second level contrast. For comparisons between groups, this means that accuracy should be matched between groups, while for correlational analyses, this means that accuracy should be uncorrelated with the covariate of interest. Matching of accuracy was assessed with the same set of questions described above for first level contrasts, except for the following five differences in assessing matching of accuracy at the second level. (1) For contrasts where both the language and control conditions involve tasks, the relevant variable to be matched at the second level is the *difference* in accuracy between the language and control conditions. Sometimes this could not be evaluated, since control task data were not reported (e.g., Griffis, Nenert, Allendorfer, Vannest, et al., 2017), or behavioral data was combined across language and control conditions (e.g., Saur et al., 2006), in which case we evaluated only the language or combined behavioral data that were reported. (2) Most contrasts involving incommensurate task requirements (e.g., resting or non-linguistic control conditions) could nevertheless be evaluated for matching of accuracy for the language condition at the second level, since the control conditions could be expected to cancel out across participants. (3) There have been many analyses that involved calculating correlations between measures of task performance and functional activity; these were coded as “Accuracy is covariate,” which was not considered to be a limitation. Note that, ideally, accuracy on the control condition should also be reported and considered in this context; however, most studies have not done this, so we set aside this issue. (4) There were many more analyses coded as “Yes, correct trials only” because unlike at the first level, it is possible to carry out such analyses even with incommensurate control conditions (e.g., picture naming, correct trials only, versus rest). (5) There have been several studies in which concrete efforts were made to match accuracy at the second level by using noise-vocoded speech in controls (Sharp, Scott, & Wise, 2004; Raboyeau et al., 2008; Sharp et al., 2010; Brownsett et al., 2014). When these efforts were not entirely successful, this was coded as “No, attempt made” and was considered only a minor limitation.

We next evaluated whether reaction time was matched across the second level contrast. Again, this was largely similar to the first level assessment of matching reaction time, except that first, when both the language and control conditions involve tasks, the *difference* in reaction times between language and control conditions should be matched, and second, contrasts with incommensurate task demands could be assessed at the second level. As for the first level appraisal, contrasts without tasks (e.g., listening to narrative speech versus listening to reversed speech) were coded as “N/A, no timeable task,” which was not considered to be a limitation. However, analyses with covert tasks were coded as “Unknown, not reported” and considered a moderate limitation, unless overt behavioral data were acquired outside the scanner and reported.

Next, specific data items were extracted for voxelwise analyses, ROI analyses, and complex analyses, as described in the following sections.

#### Voxelwise analyses

For voxelwise analyses, we first noted the search volume. Then, we evaluated the most important methodological issue for voxelwise analyses, which is the approach taken to correcting for multiple comparisons. We consider the gold standard approach to be permutation testing (Nichols & Holmes, 2002; Eklund et al., 2016), in which voxelwise or cluster extent-based thresholds are derived from null permutations of the real data. This is the most accurate method, because it makes no assumptions about the spatial structure of the data, unlike all other commonly used approaches (Nichols & Holmes, 2002; Eklund et al., 2016).

Voxelwise thresholds can be derived from Gaussian random field theory (GRFT; Worsley, Evans, Marrett, & Neelin, 1992; Worsley et al., 1996), which offers an effective, albeit overly conservative, means of correcting for multiple comparisons (Eklund et al., 2016). Voxelwise thresholds based on GRFT were not considered to be a limitation. Some studies have used an arbitrary cluster size cutoff in addition to a GRFT-based voxelwise threshold; this was considered a minor limitation, since the additional criterion is arbitrary and unjustified. Small volume correction can be used to investigate effects only in specific brain regions (Worsley et al., 1996). While this is a reasonable approach in principle, we considered small volume correction to constitute a moderate limitation, because there are many degrees of freedom available in terms of specifying the size and location of the correction volume.

Cluster extent thresholds are a commonly used alternative to voxelwise thresholds. In this approach, a prespecified cluster-defining threshold (CDT) is applied, and any resulting suprathreshold clusters are then assessed for statistical significance based on their extent. Most often, the necessary minimum cluster extent is determined using GRFT (Friston, Worsley, Frackowiak, Mazziotta, & Evans, 1994). The validity of this approach has recently been shown to strongly depend on the CDT, such that cluster correction is fairly accurate when the CDT is stringent, but overly liberal, yielding a high proportion of false positives, when the CDT is lenient (Eklund et al., 2016). Based on these findings and other simulation studies (Woo, Krishnan, & Wager, 2014; Cox, Chen, Glen, Reynolds, & Taylor, 2017), we considered clusterwise correction with reference to GRFT to pose no limitation if the CDT was *p* < 0.001 or lower, but to constitute a moderate limitation if the CDT was any higher than 0.001.

Another way to determine the necessary minimum cluster extent is through simulated data (Forman et al., 1995; Slotnick, Moo, Segal, & Hart, 2003). In these approaches, thresholds are derived based on extrema in null data that are generated in a manner intended to match the spatial structure of the real data. The most commonly used implementation of this approach is *3dClustSim* (Forman et al., 1995), which has been shown to be overly lenient, probably because it underestimates the smoothness of real data, and because the simulated data does not have a realistic spatial structure (Eklund et al., 2016). Therefore, the use of *3dClustSim* was considered a moderate limitation. Another implementation of this approach used in a number of aphasia studies is *cluster_threshold_beta* (Slotnick et al., 2003). Although *cluster_threshold_beta* is conceptually similar to *3dClustSim*, it appears to generate even more lenient estimates of necessary minimum cluster extent, as revealed by direct comparisons between the two algorithms (Abel, Weiller, Huber, Willmes, & Specht, 2015). We considered use of the very liberal thresholds derived from *cluster_threshold_beta* to constitute a major limitation. For further discussion, see Slotnick (2017) and Nichols, Eklund, and Knutsson (2017).

Some studies used arbitrary cluster extent thresholds, did not correct for multiple comparisons at all, did not carry out direct statistical comparisons across the second level contrast, or did not describe correction for multiple comparisons in sufficient detail to evaluate. These were all considered to be major limitations. Finally, there were several mixed approaches, which were each assessed on their own merits; all mixed approaches were ultimately considered to involve major limitations.

#### ROI analyses

We first coded whether ROIs were defined anatomically (based on atlases or individual anatomical images) or functionally (based on some functional contrast). Analyses of laterality indices were also treated as ROI analyses, since they are conceptually similar in that patterns of brain activation are reduced to a single number or a few numbers for each participant. Some ROI analyses were mixed, with different ROIs defined in different ways, while others could not be simply classified in these terms. We coded how many ROIs there were, what the ROIs were (generally using the authors’ terminology), and how they were defined.

We then evaluated correction for multiple comparisons across multiple ROIs. Correction for familywise error was considered optimal. Correction for false discovery rate was considered a minor limitation since it is less conservative than correcting for familywise error. When no correction was made for multiple comparisons, this was considered a moderate limitation if there were ten ROIs or fewer, and a major limitation if there were more than ten. Although this cutoff was arbitrary, it was intended to approximately parallel our appraisal of voxelwise analyses in terms of expected degree of inflation of the true false positive rate. Some ROI analyses have been reported in which there was no direct statistical comparison across the second level contrast; this was considered a major limitation.

#### Complex analyses

Analyses other than voxelwise analyses or ROI analyses were inherently varied in their nature. We wrote a brief narrative summary of each complex analysis, and any minor, moderate, or major limitations were identified on a case-by-case basis (see Complex analyses in the Results section). Many complex analyses involved voxelwise analyses with additional complexities. In these cases, correction for multiple comparisons was generally appraised in the same way that more straightforward voxelwise analyses were.

#### Miscellaneous limitations

Some analyses had limitations other than those captured by the specific data items described so far. These miscellaneous limitations were noted under “Statistical details” (see Supplementary Table S16), except for limitations related to unclear or problematic ROI definitions, which were noted in relation to the data item “How are the ROI(s) defined?” (see Supplementary Table S16). Miscellaneous limitations were evaluated as minor, moderate, or major, depending on the particulars of each situation (see Miscellaneous limitations in the Results section).

Finally, cognitive neuroscience studies in general rarely take into account the multiple comparisons that are entailed in reporting multiple analyses per study; however, this can be a significant concern, especially in studies that include many analyses (Alberton, Nichols, Gamba, & Winkler, 2020). Accordingly, we counted it as a minor limitation if a study reported more than one analysis, a moderate limitation if a study reported more than 10 analyses, and an additional moderate limitation for each additional 10 analyses.

### Overall Appraisal

We defined a subset of analyses as “methodologically robust” for further analysis. These were analyses with no major limitations, and no more than 10 moderate limitations. The first of these requirements follows from our definition of major limitations as those that may compromise the veracity of the findings or preclude the interpretation of the findings with respect to our questions of interest. The specific cutoff of 10 moderate limitations in the second requirement is arbitrary, but does allow us to identify and focus on a subset of analyses with relatively few limitations to their interpretation.

We acknowledge that the subset of analyses so identified depends on our identification and appraisal of limitations, which as noted above is to some extent subjective. We also note that some analyses that were not appraised as “methodologically robust” nevertheless yielded findings that we believe to be true. This is discussed further under Limitations of our study in the Discussion section.

When counting limitations, we summated limitations pertaining to the analysis itself, the contrast it was based on, the conditions that entered into the contrast, and the participants and imaging data items relating to the study as a whole. In other words, limitations in any aspect of the study cascaded down to any analyses that were impacted by them. For contrasts that involved more than two conditions, only the condition with the most limitations on each side of the contrast was counted.

### Findings

**Figure 2.**
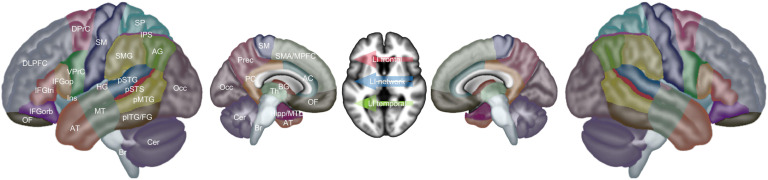
Regions of interest. Descriptions of regions corresponding to each abbreviation are shown in Table 8.

Our included studies spanned over 25 years of research, and as such, findings were reported in many different ways. In order to extract findings from all included studies, we did not limit our analysis to studies that reported Montreal Neurological Institute (MNI) coordinates, or any other specific criterion. Rather, we defined 30 brain regions in each hemisphere (Figure 2, Table 8), and for each analysis we coded all reported activation increases and/or decreases in terms of these regions, based on the best information available in each study. Sometimes this was tables containing MNI coordinates, sometimes figures, sometimes descriptions in the text, and sometimes combinations of these. If activation increases or decreases appeared to span multiple regions, then they were coded in all the regions that they spanned. We focused on the main features of the activation patterns, rather than being concerned with fine details.

**Table 8.** Regions of interest

Abbreviation	Description
IFGop	inferior frontal gyrus pars opercularis
IFGtri	inferior frontal gyrus pars triangularis
IFGorb	inferior frontal gyrus pars orbitalis
Ins	insula
DLPFC	dorsolateral prefrontal cortex
VPrC	ventral precentral (including inferior frontal junction)
DPrC	dorsal precentral
SMA/MPFC	supplementary motor area/medial prefrontal cortex
OF	orbitofrontal
SM	somato-motor
SMG	supramarginal gyrus
AG	angular gyrus
IPS	intraparietal sulcus
SP	superior parietal
Prec	precuneus
pSTG	posterior superior temporal gyrus
pSTS	posterior superior temporal sulcus
pMTG	posterior middle temporal gyrus
HG	Heschl’s gyrus
MT	mid temporal
AT	anterior temporal
pITG/FG	posterior inferior temporal gyrus/fusiform gyrus
Occ	occipital
AC	anterior cingulate
PC	posterior cingulate
Cer	cerebellum
Br	brainstem
Th	thalamus
BG	basal ganglia
Hipp/MTL	hippocampus and related medial temporal lobe structures
LI frontal	lateralization index in frontal language regions
LI temporal	lateralization index in temporal language regions
LI network	lateralization index in language network

Our set of regions was defined manually. Brain regions that are frequently activated in language imaging studies were “oversampled,” that is, perisylvian cortex was parcellated into smaller regions than was the rest of the brain. We also created three combined regions for situations where language activations were larger or less clearly described: (1) inferior frontal gyrus (IFG), comprising the pars opercularis, the pars triangularis, and the pars orbitalis; (2) pSTG/STS/MTG, comprising the posterior superior temporal gyrus (pSTG), the posterior superior temporal sulcus (pSTS), and the posterior middle temporal gyrus (pMTG); and (3) inferior parietal lobule, comprising the supramarginal gyrus and the angular gyrus.

Besides these regions, we also coded increases or decreases in lateralization indices in frontal language areas (LI frontal), temporal language areas (LI temporal), or the whole language network (LI network). Increases in lateralization indices indicated leftward changes in lateralization (i.e., increased left-lateralization, or decreased right-lateralization), while decreases indicated rightward changes.

Occasionally, other findings were reported that could not be described simply in terms of activation increases or decreases in specific brain regions, or changes in lateralization indices. In these cases, we wrote brief narrative descriptions of the findings.

### Statistical Analysis

Color maps were created to indicate how many analyses of each class revealed activation increases or decreases in each brain region. Findings from methodologically robust analyses were plotted, except that when multiple closely related analyses were reported in a study, only a single analysis was plotted. Findings from all analyses, without regard for limitations, were plotted, subject to a restriction that the same finding was counted no more than once per study.

To identify patterns in the findings from all analyses, the relative prevalence of activation increases and decreases was compared and corrected for multiple comparisons across ROIs using permutation testing. Specifically, for each region where activation increases and/or decreases were reported, we determined whether there were more increases than decreases, or vice versa, using the binomial test. Then, 10,000 null datasets were constructed by randomly reassigning the directionality of all reported activation changes, and recording for each iteration the minimum *p* value across the 30 left hemisphere regions, the 30 right hemisphere regions, and the 3 laterality indices. The observed *p* values were corrected with respect to this null distribution. To determine whether there were hemispheric differences in patterns of activation increases and decreases, activation increases and decreases were compared in each pair of homotopic regions using Fisher’s exact test. Then, 10,000 null datasets were constructed by randomly reassigning the hemisphere of all reported activation changes, and recording for each iteration the minimum *p* value across the 30 pairs of regions. The observed *p* values were corrected with respect to this null distribution.

## RESULTS

As described above, we identified 86 studies that met our inclusion criteria. These studies included 287 conditions, and 129 contrasts were computed that were used in one or more relevant analyses. A total of 561 relevant second level analyses were described, of which 383 were cross-sectional and 178 were longitudinal. These analyses yielded a total of 1,455 findings. Our complete coding of each study is provided in Supplementary Table S16. Interactive tables with hyperlinks and tooltips are available at https://langneurosci.org/aphasia-neuroplasticity-review.

### Participants

**Figure 3.**
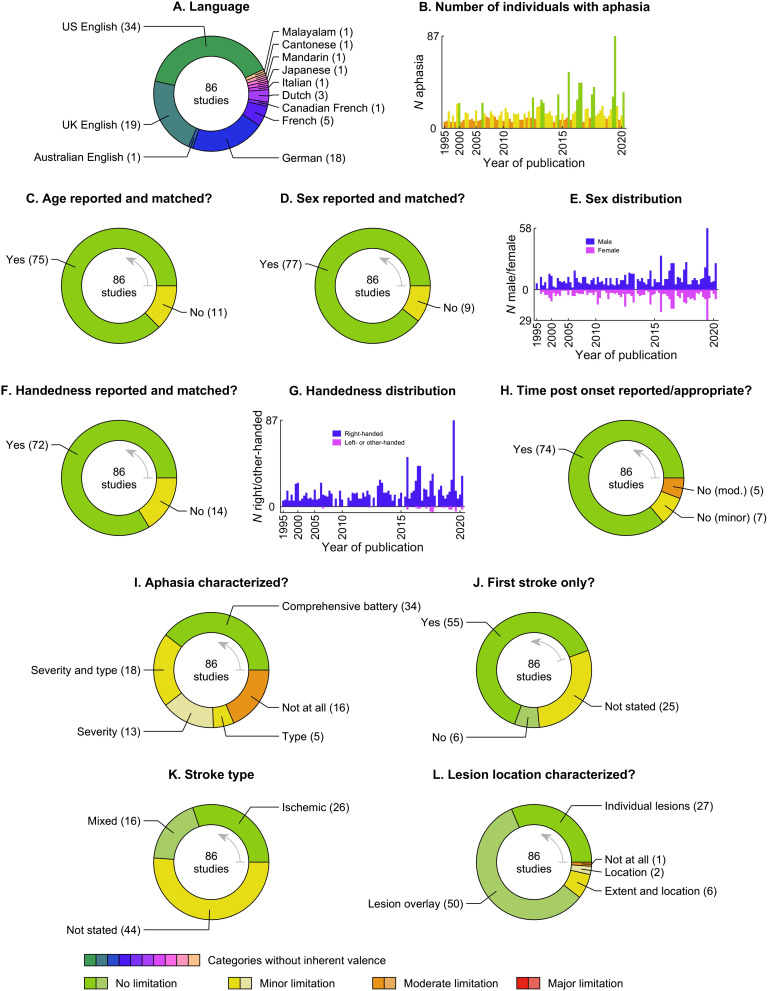
Participants. Donut charts and bar graphs presenting information about the individuals with aphasia who participated in the 86 studies, and our appraisal of the extent to which sufficiently detailed information was provided about the participants. Note that two distinct color scales are used in this and the subsequent five figures: one for categories where there is no inherent valence, and another for those where limitations were assessed. In the latter case, donut plots are arranged with categories in descending order of quality, moving counterclockwise, as indicated by the light gray arrows.

Information about the participants included in each study, and our appraisal of the extent to which sufficiently detailed information has been provided about the participants, is provided in Supplementary Tables S1, S2, S3, and S4, and summarized in Figure 3.

Studies have been performed in nine different languages, most commonly English, followed by German and French (Figure 3A; Supplementary Table S1). About two thirds of studies (59 studies, 69%) have recruited specific cohorts of individuals with aphasia based on variables such as lesion location, aphasia type or severity, or specific abilities or deficits, while the remaining one third of studies have not had any such restrictions (Supplementary Table S1).

The number of individuals with aphasia included in each study has ranged from 6 (required per our inclusion criteria) to 87, with a mean of 16.4 ± 12.1 (*SD*) participants (Figure 3B; Supplementary Table S1). Recent years have seen larger sample sizes; there was a significant correlation between publication year and number of participants with aphasia (*r* = 0.34, *p* = 0.0012). Neurologically normal control participants have been included in 59 studies (69%), and have ranged in number from 4 to 85, with a mean of 17.3 ± 13.0 participants (Supplementary Table S1). At least 16 studies (19%) have included participants from other included studies, and several have been reanalyses of the same datasets (Supplementary Table S1). It was not always possible to determine exactly how much overlap there has been between cohorts and datasets, since this information has often not been clearly stated.

In most studies, age, sex, handedness, and time post onset have been reported, and have been matched between patients and controls where necessary (Figure 3C, 3D, 3F, 3H; Supplementary Table S2). There has been a significant bias toward inclusion of male participants: the mean proportion of males was 0.65 ± 0.16, which is significantly greater than half, *t*(83) = 8.46, *p* < 0.0001, confidence interval = [0.62, 0.69] (Figure 3E; Supplementary Table S2). Most studies have included only right-handed participants, but since 2016, a number of studies have included some non-right-handed participants, roughly consistent with population prevalence of non-right-handedness (Figure 3G; Supplementary Table S2).

The characterization of aphasias has often been somewhat limited (Figure 3I; Supplementary Table S3). Less than half of studies have characterized the nature of aphasia in each individual with a comprehensive battery of scores, with the remaining studies reporting only aphasia severity and/or aphasia type, or in some cases neither.

Most studies have included only first ever strokes, but this information has not always been stated (Figure 3J; Supplementary Table S4). About half of studies have reported stroke type, with about two thirds of studies focused on ischemic stroke, while the remainder recruited mixed ischemic and hemorrhagic cohorts (Figure 3K; Supplementary Table S4). Lesion location has been well characterized in most studies, either by showing individual brain images or lesion overlay maps (Figure 3L; Supplementary Table S4).

### Imaging

**Figure 4.**
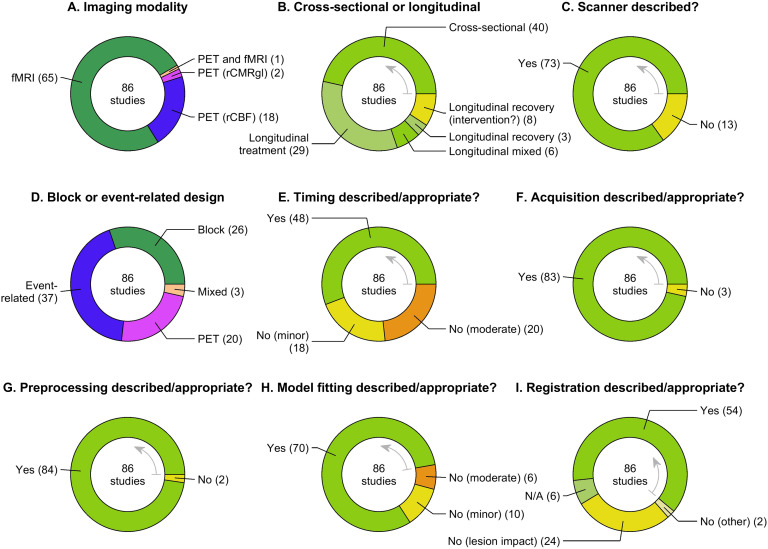
Imaging. Donut charts presenting information about the imaging methods of the 86 studies, and our appraisal of the extent to which methods were described in sufficient detail, and appropriate. See the Figure 3 caption for more information. rCMRgl = region cerebral metabolic rate for glucose; rCBF = regional cerebral blood flow.

Information regarding the basic design of the imaging studies, and the extent to which data acquisition, preprocessing, model fitting, and intersubject registration have been adequately described and appropriate, is provided in Supplementary Tables S5, S6, and S7, and summarized in Figure 4.

About three quarters of studies have used fMRI; and no PET studies have been published since 2013 (Figure 4A; Supplementary Table S5). Studies have been evenly split between cross-sectional and longitudinal designs (Figure 4B; Supplementary Table S5). Most longitudinal studies have been treatment studies in the chronic phase (“Longitudinal treatment”), but a substantial minority have investigated patients in the acute and/or subacute phases of recovery, in the context of usual care (“Longitudinal recovery”) or treatment (“Longitudinal mixed”). The time points and interventions of longitudinal studies are described in Supplementary Table S5. Some studies did not state whether there was any intervention (“Longitudinal recovery (intervention?),” Figure 4B; Supplementary Table S5).

Most studies have described the make and model of the scanner (Figure 4C; Supplementary Table S6), described appropriate imaging acquisition parameters (Figure 4F; Supplementary Table S7), and described appropriate preprocessing and intrasubject coregistration procedures (Figure 4G; Supplementary Table S7). Issues arose more frequently with the remaining three data items in this section.

Event-related and block designs have both been widely used, with the former somewhat more prevalent (Figure 4D; Supplementary Table S6). The timing of stimulus presentation and image acquisition have been clearly described and appropriate in only a little over half of studies (Figure 4E; Supplementary Table S6). In about a quarter of studies, insufficient details or minor inconsistencies regarding timing were considered to be minor limitations. The remaining quarter of studies had more serious issues with timing that were considered to constitute moderate limitations, including acquiring conditions in different runs (Fridriksson, Hubbard, et al., 2012; Robson et al., 2014) or on different days (Karbe et al., 1998), insufficient numbers of blocks (Zahn et al., 2004; Tyler, Wright, Randall, Marslen-Wilson, & Stamatakis, 2010; Wright, Stamatakis, & Tyler, 2012), or systematic timing contingencies between event types that would be likely to limit their separability in the general linear model (Sebastian & Kiran, 2011; Brownsett et al., 2014; Skipper-Kallal, Lacey, Xing, & Turkeltaub, 2017a, 2017b; see Supplementary Table S6 for details).

Most studies have described appropriate first level model fitting procedures (Figure 4H; Supplementary Table S7), but 10 studies (12%) provided insufficient information, which was considered a minor limitation. Five studies (6%) did not explain how model fitting would be able to resolve separate phases of trials, which is problematic because the overlapping hemodynamic responses to adjacent trial phases pose a formidable challenge to this kind of approach (Brownsett et al., 2014; Skipper-Kallal et al., 2017a, 2017b; Sreedharan, Chandran, et al., 2019; Purcell, Wiley, & Rapp, 2019), and one study did not establish that events would be separable from rest (Johnson, Meier, Pan, & Kiran, 2019); these issues were considered moderate limitations.

About two thirds of studies have described appropriate intersubject normalization procedures, with the most common minor limitation being failure to address the impact of lesions on normalization (Figure 4I; Supplementary Table S7).

### Conditions

**Figure 5.**
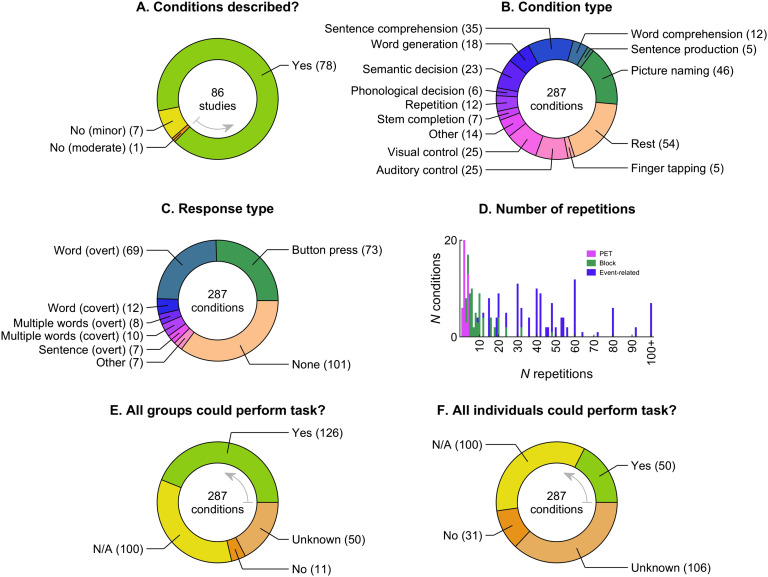
Conditions. Donut charts and a bar graph presenting information about the 287 conditions presented in the 86 studies, and our appraisal of the extent to which the conditions were described in sufficient detail, and whether it was documented that participants could perform any tasks. See the Figure 3 caption for more information.

The conditions included in each study and their feasibility for individuals with aphasia are detailed in Supplementary Table S8 and summarized in Figure 5. The number of conditions has ranged from 2 to 9 (mean = 3.3 ± 1.5), for a total of 287 conditions. Most studies have described these conditions in sufficient detail (Figure 5A), but lack of detail was considered a minor limitation for 7 studies, and a moderate limitation for one study where the nature of the questions posed to patients was not specified (Chau, Fai Cheung, Jiang, Au-Yeung, & Li, 2010). The most frequently used language conditions have been picture naming, sentence/narrative comprehension, semantic decision, and word generation (Figure 5B). The most frequent response types (not including rest conditions) have been overt single words and button presses (Figure 5C). The number of times each condition was repeated (PET measures, blocks, or events) is shown in Figure 5D; any limitations related to this data item were evaluated in relation to timing of stimulus presentation and image acquisition.

We assessed whether all groups were able to perform each task at all time points (Figure 5E). About a third of the conditions required no response. Of the remaining conditions, for about two thirds, data were reported documenting that all groups at all time points were able to perform the task, which was defined, as described above, as above-chance for forced choice tasks, or greater than 10% correct for tasks with open-ended responses. For the remaining third, insufficient data were provided to confirm that the task could be performed by all groups at all time points, or the data that were reported established that the task could not be performed by one or more groups at one or more time points.

We next assessed whether all individuals were able to perform each task at all time points (Figure 5F). Of the conditions requiring a response, for only about a quarter was it documented that all individuals at all time points could perform the task. For the remaining three quarters, insufficient data were provided to confirm that the task could be performed by all individuals at all time points, or the data that were reported established that the task could not be performed by one or more individuals at one or more time points.

### Contrasts

**Figure 6.**
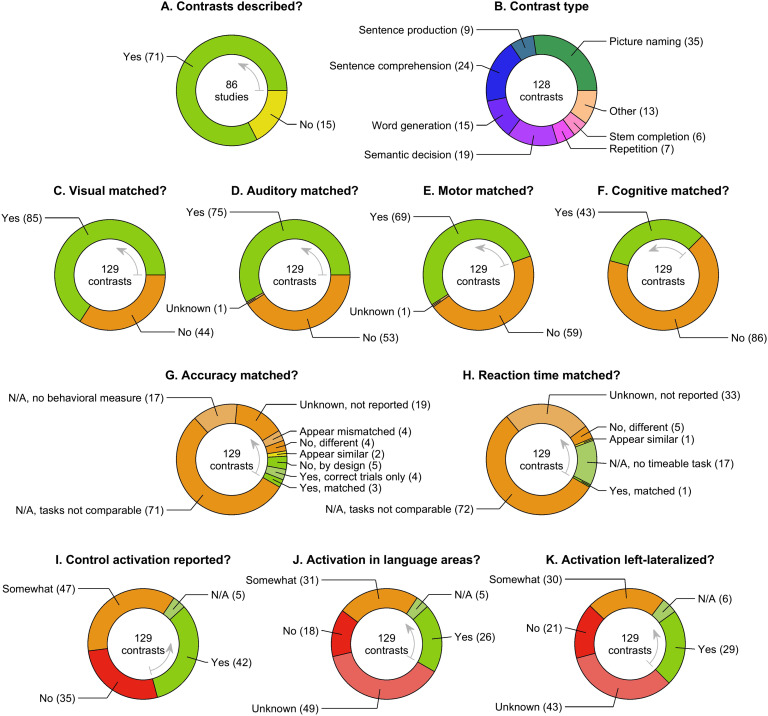
Contrasts. Donut charts presenting information about the 129 contrasts performed in the 86 studies, and our appraisal of the extent to which contrasts were described in sufficient detail, matched between language and control conditions, and whether contrast validity was documented. See Figure 3 caption for more information.

The contrasts computed in each study, and our appraisal of the effectiveness of their control conditions and their validity in identifying language regions, are detailed in Supplementary Table S9 and summarized in Figure 6. The number of contrasts used in relevant analyses has ranged from 1 to 4 (mean = 1.5 ± 0.7), for a total of 129 contrasts. Most studies have described these contrasts in sufficient detail, with 15 exceptions where lack of detail or clarity was considered a minor limitation (Figure 6A; Supplementary Table S9). The most commonly used contrasts have involved picture naming, sentence (or narrative) comprehension, semantic decision, and word generation (Figure 6B; Supplementary Table S9).

Approximately two thirds of contrasts have been matched for visual demands (Figure 6C; Supplementary Table S9), ∼60% have been matched for auditory demands (Figure 6D; Supplementary Table S9), and about half have been matched for motor demands (Figure 6E; Supplementary Table S9). However, only about one third of contrasts have been matched for cognitive demands (Figure 6F; Supplementary Table S9).

Only ∼10% of contrasts have been matched for accuracy in all groups at all time points (statistically, by visual inspection, or by analyzing correct trials only) or mismatched by design (Figure 6G; Supplementary Table S9). For the remaining ∼90% of contrasts, either accuracy was mismatched, accuracy was not reported, there was no behavioral measure, or the language and control conditions had incommensurate task requirements.

Only 2 out of 120 contrasts have been matched for reaction time in all groups at all time points (one statistically, one by visual inspection) (Figure 6H; Supplementary Table S9). Another 17 contrasts did not involve timeable tasks, so matching of reaction time was not applicable. For the remaining ∼85% of contrasts, either reaction times were mismatched, reaction times were not reported, or the language and control conditions had incommensurate task requirements.

Only a minority of contrasts have been demonstrated to selectively activate lateralized language regions in neurologically normal individuals (Figure 6I, 6J, 6K; Supplementary Table S9). Specifically, 15 contrasts (12%) received “Yes” answers on all three data items related to contrast validity, while a further 35 contrasts (27%) received a combination of “Yes” and “Somewhat” answers. Another five contrasts compared successful and unsuccessful language processing and so were not evaluated for contrast validity (see Contrasts in the Methods section). The remaining 74 contrasts (57%) received one or more “No” or “Unknown” answers, meaning that either contrast validity was not established, or that the contrast did not selectively activate lateralized language regions; these were considered to constitute major limitations.

### Analyses

**Figure 7.**
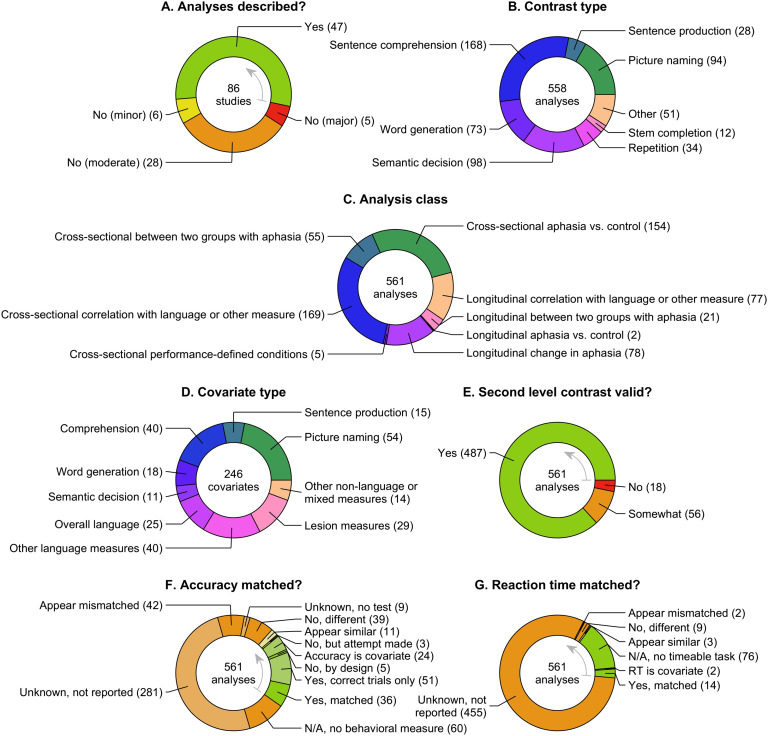
Analyses. Donut charts presenting information about the 561 relevant analyses performed in the 86 studies, and our appraisal of the extent to which analyses were described in sufficient detail, and matched for accuracy and reaction time (RT) at the second level. See Figure 3 caption for more information.

**Figure 8.**
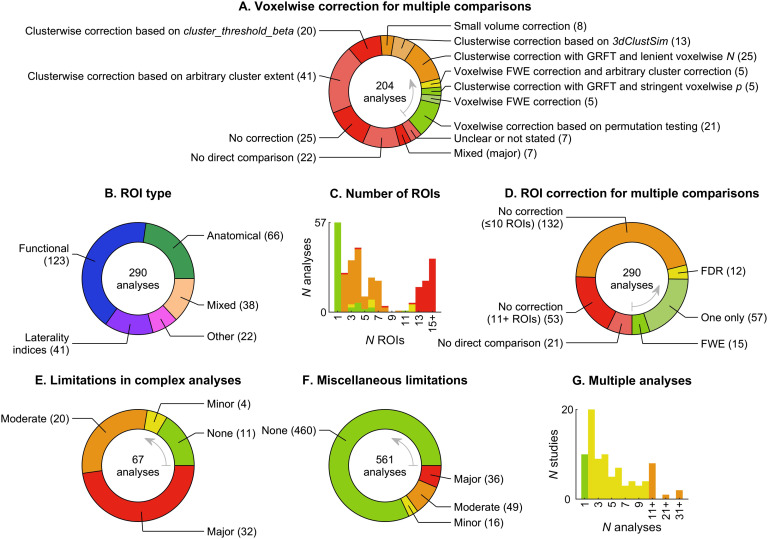
Analyses. Donut charts and bar graphs presenting information about correction for multiple comparisons and other limitations in the 561 relevant analyses. GRFT = Gaussian random field theory; FWE = familywise error; FDR = false discovery rate. See Figure 3 caption for more information.

The analyses included in each study, including the logic of the second level design, matching of accuracy and reaction time across second level contrasts, and statistical details, are described in Supplementary Table S10 and summarized in Figures 7 and 8. Studies have included between 1 and 64 analyses (mean = 6.5 ± 8.6) meeting our criteria, for a total of 561 analyses. Analyses have been clearly described in just over half of the studies, with remaining studies lacking clarity or detail to various extents (Figure 7A; Supplementary Table S10). Analyses have been most commonly based on first level contrasts involving sentence (or narrative) comprehension, semantic decision, picture naming, and word generation (Figure 7B; Supplementary Table S10).

All eight classes of second level designs have been represented (Figure 7C; Supplementary Table S10), but four have been particularly common: cross-sectional comparisons between aphasia and controls, cross-sectional correlations between functional activity and language or other measures, longitudinal comparisons between time points in groups of individuals with aphasia, and longitudinal correlations between change in functional activity and language or other measures (usually measures of change). Covariates of interest have been included in almost half of voxelwise or ROI analyses (Figure 7D; Supplementary Table S10). About five sixths of these covariates have been measures of language function, with the majority of the remainder being lesion-related measures.

Most second level contrasts have been logically constructed to address specific research questions, but issues were identified with some analyses, which were considered moderate or major limitations (Figure 7E; Supplementary Table S10). There were 56 analyses (10%) that had moderate limitations, as follows: comparisons between groups or time points where treatment effects were absent (Chau et al., 2010; Mattioli et al., 2014; Nenert et al., 2017; Sreedharan, Chandran, et al., 2019) or marginal (Dietz et al., 2018); absence of a behavioral difference between stimulation conditions (Darkow, Martin, Würtz, Flöel, & Meinzer, 2017; Hartwigsen et al., 2020); nongeneral measures of aphasia recovery such as semantic fluency (Szaflarski et al., 2011; Griffis, Nenert, Allendorfer, & Szaflarski, 2016), the Token Test (Heiss et al., 1997; Karbe et al., 1998), or task performance (de Boissezon et al., 2009); unclear or inappropriate behavioral measures (Nardo, Holland, Leff, Price, & Crinion, 2017; Purcell et al., 2019); and correlations between activation and subsequent recovery without including contemporaneous behavioral measures in the model (Richter, Miltner, & Straube, 2008; Fridriksson, Richardson, et al., 2012; Marcotte et al., 2012; Abel, Weiller, Huber, & Willmes, 2014; van Hees, McMahon, Angwin, de Zubicaray, & Copland, 2014; Geranmayeh, Chau, Wise, Leech, & Hampshire, 2017; van Oers et al., 2018; Purcell et al., 2019), which is problematic because activations will reflect present language function, which is often correlated with future recovery trajectories, meaning that such analyses are not able to ascribe correlations to subsequent recovery. There were 18 analyses (3%) with major limitations in terms of design logic, as follows: correlations of activations with previous recovery (Karbe et al., 1998; van Oers et al., 2010; Marcotte et al., 2012; van Hees et al., 2014; Geranmayeh et al., 2017), which is not appropriate since activation at any given time point will reflect language function at that time point, which may or may not be related to the extent to which language function improved prior to that time point; correlations between activation changes over time and language outcomes; the logic of the analyses was not clear (Karbe et al., 1998; Menke et al., 2009); and correlations between initial and change values in an ROI that was defined based on exhibiting change (Purcell et al., 2019).

We next examined matching of accuracy and reaction time across the second level contrast, which is important given the extent to which these variables can impact activation patterns. With respect to matching of accuracy, we found that about a quarter of analyses were matched for accuracy across the second level contrast (statistically, by visual inspection, or by analyzing correct trials only), were mismatched by design, treated accuracy as the covariate of interest, or attempted without complete success to match accuracy by manipulating stimulus difficulty (Figure 7F; Supplementary Table S10). For the remaining three quarters of analyses, either accuracy was mismatched, accuracy was not reported, or there was no behavioral measure. Note that correlations between covariates of interest and accuracy have rarely been assessed, resulting in many “Unknown, not reported” codes.

Only 19 out of 561 analyses (3%) have been matched for reaction time across the second level contrast (14 statistically, 3 by visual inspection) or used reaction time as the covariate of interest (Figure 7G; Supplementary Table S10). Another 76 analyses (14%) did not have timeable tasks so this data item was not applicable. For the remaining ∼80% of analyses, reaction times were either mismatched, or much more commonly, not reported.

#### Voxelwise analyses

A total of 204 voxelwise analyses have been reported. The search volumes, software packages used, voxelwise thresholds or CDTs (where applicable), and cluster extent cutoffs (where applicable) for each analysis are detailed in Supplementary Table S10.

The approaches taken to correcting for multiple comparisons are shown in Figure 8A and Supplementary Table S10. Only 36 analyses (18%) used approaches that were satisfactory or posed only minor limitations, and those 36 analyses came from just 7 studies (Crinion & Price, 2005; Crinion, Warburton, Lambon Ralph, Howard, & Wise, 2006; Darkow et al., 2017; Nardo et al., 2017; Nenert et al., 2018; Kristinsson et al., 2019; Hartwigsen et al., 2020), with all 21 permutation analyses coming from a single study (Nenert et al., 2018).

Another 46 analyses (22%) used approaches that are principled, yet are now known to inflate the false positive rate. Of note, 25 out of 30 analyses that corrected for multiple comparisons based on cluster extent and GRFT did so using excessively lenient CDTs, the only exceptions being Darkow et al. (2017) and Hartwigsen et al. (2020).

The remaining ∼60% of voxelwise analyses did not use principled approaches to correcting for multiple comparisons, or did not carry out direct statistical comparisons at the second level. These were considered to constitute major limitations.

#### ROI analyses

A total of 290 ROI analyses have been reported. ROIs have most frequently been defined functionally, but there have also been numerous analyses involving anatomical ROIs, laterality indices, combinations of different types of ROIs, or ROIs defined in other ways (Figure 8B; Supplementary Table S10). The number of ROIs has ranged from 1 to 18 (Figure 8C; Supplementary Table S10). About three quarters of analyses have involved more than one ROI, but only about an eighth of these were corrected for multiple comparisons (Figure 8D; Supplementary Table S10). Of the remaining analyses with more than one ROI, about two thirds involved 10 or less ROIs and appropriate second level group comparisons or correlations. The remaining 74 analyses involved more than 10 ROIs and/or no direct statistical comparison at the second level; these were considered to constitute major limitations.

#### Complex analyses

There were 67 complex analyses (Supplementary Table S10), which comprised group differences in correlations with a behavioral measure (Crinion & Price, 2005); joint independent component analysis (ICA; Specht et al., 2009; Wright et al., 2012; Abel et al., 2015; Griffis, Nenert, Allendorfer, & Szaflarski, 2017); analyses of the relationship between lesion location and functional activation in specific regions using voxel-based lesion-symptom mapping (Warren, Crinion, Lambon Ralph, & Wise, 2009; Fridriksson et al., 2010), voxel-based morphometry (Tyler et al., 2010), support vector regression lesion symptom mapping (Skipper-Kallal et al., 2017a), or other statistical approaches (Sims et al., 2016; Griffis, Nenert, Allendorfer, Vannest, et al., 2017; Johnson et al., 2019; Stockert et al., 2020), psychophysiological interactions between seed regions and the whole brain (Papoutsi, Stamatakis, Griffiths, Marslen-Wilson, & Tyler, 2011; Griffis et al., 2016; Nenert et al., 2018), between pairs of ROIs (Griffis et al., 2016), or between networks (Geranmayeh, Leech, & Wise, 2016), comparisons between patients and controls of activity in networks derived from ICA (Geranmayeh et al., 2016; Darkow et al., 2017), comparisons between patients and controls of correlations between activity in different regions (Sims et al., 2016) or networks (Griffis, Nenert, Allendorfer, Vannest, et al., 2017), investigations of relative activation of networks as a predictor of language function (Geranmayeh et al., 2016) or between patients and controls (Griffis, Nenert, Allendorfer, Vannest, et al., 2017), investigations of associations between local heterogeneity of functional responses and language function (Purcell et al., 2019), and probing the utility of neurofeedback in enhancing recovery (Sreedharan, Chandran, et al., 2019), and correlations between the functional and behavioral effects of cortical stimulation (Hartwigsen et al., 2020).

Most of the complex analyses had one or more limitations (Figure 8E; Supplementary Table S10). Almost all of these limitations related to correction for multiple comparisons of voxelwise maps or ROIs, or failure to quantify apparent differences statistically, and were appraised similarly to the appraisal of multiple comparisons in more conventional analyses.

#### Miscellaneous limitations

About a fifth of the analyses had one or more miscellaneous limitations in addition to those appraised in relation to the data items already described (Figure 8F; Supplementary Table S10). The minor and moderate limitations that were identified mostly involved lack of clarity or incomplete descriptions of methods or reporting of results. Major limitations were identified most commonly when ROI analyses involved aspects of circularity, namely defining ROIs in one group and then comparing that group to another group (Blasi et al., 2002; Saur et al., 2006; Crinion et al., 2006; Connor et al., 2006; Geranmayeh et al., 2016; Darkow et al., 2017; Griffis, Nenert, Allendorfer, Vannest, et al., 2017; Stockert et al., 2020), or defining ROIs based on the same data that were then compared in the ROIs (Richter et al., 2008; Szaflarski, Allendorfer, Banks, Vannest, & Holland, 2013).

As described above, the 86 studies reported between 1 and 64 analyses (Figure 8G). In line with standard practices in cognitive neuroscience, no studies corrected for the multiple comparisons entailed by conducting multiple analyses; therefore, minor or moderate limitations were assessed depending on the number of analyses reported.

### Overall Appraisal

**Figure 9.**
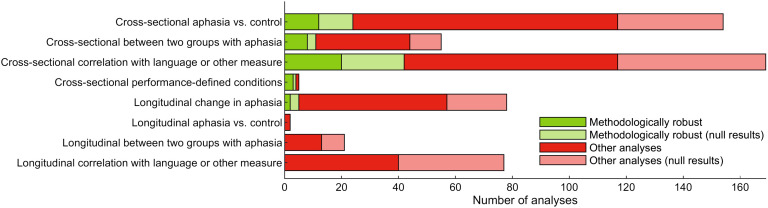
Proportions of analyses that were appraised as methodologically robust, divided according to analysis class.

The numbers of analyses that were appraised as methodologically robust (no major limitations and no more than 10 moderate limitations) are shown for each class of analyses in Figure 9. A total of 84 out of 561 analyses (15%) were appraised as methodologically robust, of which 45 analyses (8% of the total) reported one or more positive findings. Almost all (79) of the methodologically robust analyses were cross-sectional; there were just 5 longitudinal analyses that met our criteria.

### Findings

Of the 561 analyses that have been described, 296 analyses (53%) yielded findings that could be summarized in terms of activation increases and/or decreases in one or more brain regions. In these 296 analyses, the number of brain regions reported ranged from 1 to 20, with a mean of 4.7 ± 4.5. Another 60 analyses (11%; including two that also involved simple findings) involved more idiosyncratic findings that could not be summarized in terms of activation increases and/or decreases (43 of the complex analyses with non-null findings, plus 17 ROI analyses, mostly involving ROI definitions that were dependent on individual lesion locations). The remaining 207 analyses (37%) yielded null results.

In the following four sections, findings from the four most common classes of analyses will be described first, followed by a section describing findings from the remaining four classes of analyses.

#### Cross-sectional aphasia compared to control

There have been 154 analyses comparing individuals with aphasia to neurologically normal controls (Supplementary Table S10), of which 24 (16%) were appraised as methodologically robust (Figure 9; Supplementary Table S11). These 24 analyses came from 12 studies (Leff et al., 2002; Blank et al., 2003; Sharp et al., 2004; Zahn et al., 2004; Crinion & Price, 2005; Crinion et al., 2006; Warren et al., 2009; Fridriksson et al., 2010; van Oers et al., 2010; Allendorfer, Kissela, Holland, & Szaflarski, 2012; Szaflarski et al., 2014; Griffis, Nenert, Allendorfer, Vannest, et al., 2017). Of the 24 analyses, 11 had findings that could be characterized in terms of activation increases and/or decreases in one or more brain regions, one reported a more idiosyncratic finding that could not be summarized in those terms (Griffis, Nenert, Allendorfer, & Szaflarski, 2017), and 12 had null results.

**Figure 10.**
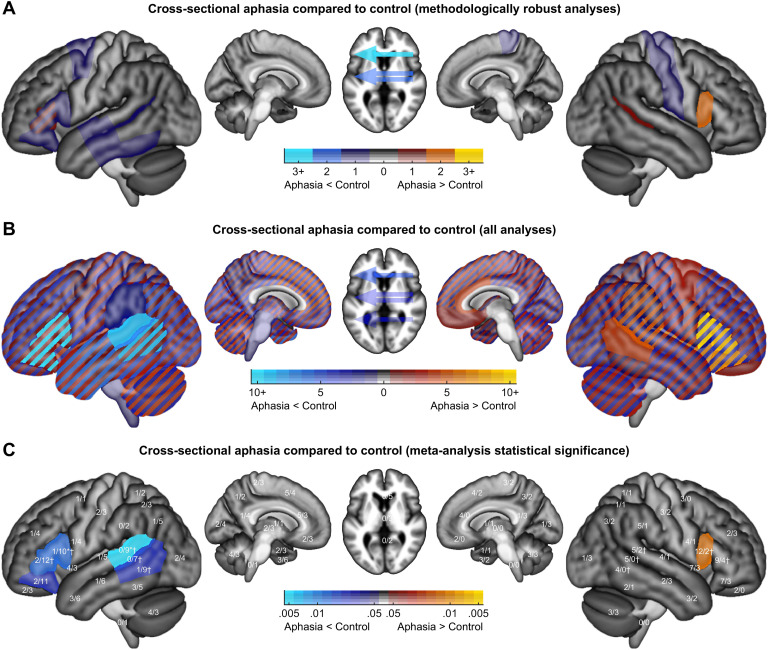
Findings from cross-sectional analyses in which individuals with aphasia were compared to neurologically normal participants. (A) Methodologically robust analyses. For each brain region, hot colors indicate the number of analyses reporting greater activation in aphasia compared to control, while cool colors indicate the number of analyses reporting less activation in aphasia compared to control. (B) All analyses. Colors indicate the number of studies reporting greater or lesser activation in aphasia compared to control. Diagonal stripes are used for brain regions where both increased and decreased activation have been reported. (C) Statistical assessment of all analyses. The two numbers shown for each region indicate the number of activation increases/decreases. Color coding indicates the uncorrected *p* value of binomial tests comparing the number of activation increases to the number of decreases reported in each brain region. * = significant difference in the prevalence of increases and decreases (binomial test, *p* < 0.05 after correction for multiple comparisons). † = significant difference between hemispheres in the prevalence of increases/decreases (Fisher’s exact test, *p* < 0.05 after correction for multiple comparisons).

Of the 11 findings involving activation increases and/or decreases, two analyses from Sharp et al. (2004) were essentially redundant with one another (a voxelwise analysis and an ROI analysis yielded the same finding). After retaining just one of these two, the remaining 10 findings were plotted (Figure 10A). Individuals with aphasia showed reduced activation compared to controls in the left IFG (all three parts, van Oers et al., 2010), left pSTS (Crinion & Price, 2005), left mid temporal region (Crinion & Price, 2005), left posterior inferior temporal gyrus/fusiform gyrus (Sharp et al., 2004), left dorsal precentral region (Crinion & Price, 2005), and right somato-motor cortex (Crinion & Price, 2005), and reduced lateralization indices in patients were reported in three studies (van Oers et al., 2010; Allendorfer et al., 2012; Szaflarski et al., 2014). The one methodologically robust idiosyncratic finding from this class of analyses was also consistent with this general pattern: Griffis, Nenert, Allendorfer, and Szaflarski (2017) reported a joint ICA analysis in which the first component linked damage to the left posterior temporo-parietal region with decreased activation of canonical semantic network nodes including the left angular gyrus and the left IFG. Three studies have reported activation increases in patients: in the left IFG pars triangularis (Warren et al., 2009), right pSTS (Leff et al., 2002), and right IFG pars opercularis (two distinct analyses in Blank et al., 2003).

Next, considering all analyses without regard for limitations, we found that although activation increases and decreases have been reported for almost every brain region (Figure 10B), statistical comparisons between activation increases and decreases revealed some clear patterns (Figure 10C). Decreases were more prevalent than increases throughout the left IFG and left posterior temporal cortex, with these ratios statistically significant after correction for multiple comparisons in the pars opercularis (*p* = 0.0401), pars triangularis (*p* = 0.0491), and pSTG (*p* = 0.0154). In contrast, increases were more prevalent than decreases in right hemisphere homotopic regions, reaching significance in the right IFG pars opercularis (*p* = 0.0491). The relative propensity of increases and decreases differed significantly between hemispheres for the IFG pars opercularis (*p* = 0.0002), IFG pars triangularis (*p* = 0.0255), pSTG (*p* = 0.0218), the pSTS (*p* = 0.0028), and pMTG (*p* = 0.0230).

#### Cross-sectional correlation with language or other measure

There have been 169 analyses in which correlations were computed within a group of individuals with aphasia between functional activity and a measure of language function, or another relevant variable (Supplementary Table S10). Of these, 42 analyses (25%) were appraised as methodologically robust (Figure 9; Supplementary Table S12). These 42 analyses came from 14 studies (Blank et al., 2003; Crinion & Price, 2005; Crinion et al., 2006; Warren et al., 2009; Fridriksson et al., 2010; van Oers et al., 2010; Papoutsi et al., 2011; Sebastian & Kiran, 2011; Tyler et al., 2011; Allendorfer et al., 2012; Griffis, Nenert, Allendorfer, & Szaflarski, 2017; Griffis, Nenert, Allendorfer, Vannest, et al., 2017; Nenert et al., 2018; Hartwigsen et al., 2020). Of the 42 analyses, 16 had findings that could be characterized in terms of activation increases and/or decreases in one or more brain regions, five yielded more idiosyncratic findings that could not be summarized in those terms (Fridriksson et al., 2010; Papoutsi et al., 2011; Griffis, Nenert, Allendorfer, Vannest, et al., 2017; Hartwigsen et al., 2020), and 21 had null results. All of the methodologically robust analyses with positive findings involved measures of language function as covariates (i.e., not other variables such as lesion extent).

**Figure 11.**
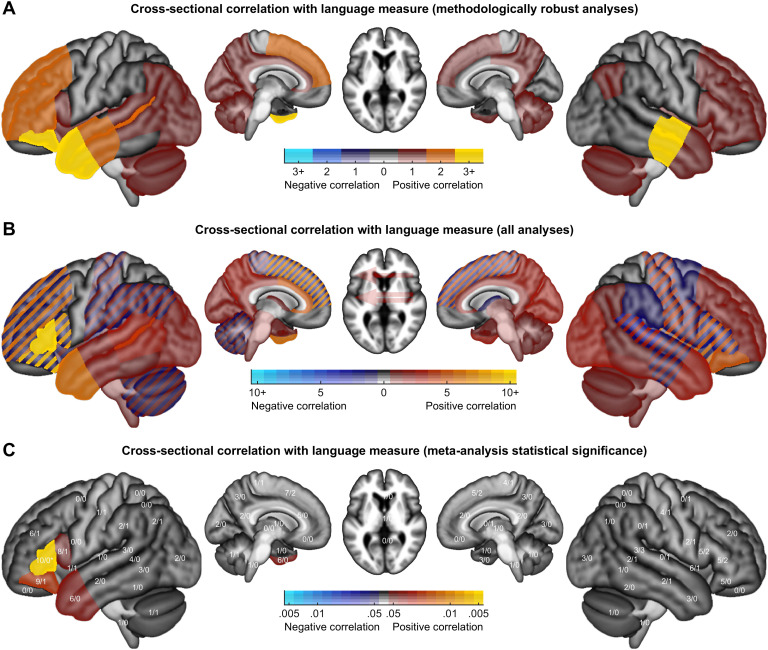
Findings from cross-sectional correlations between activation and language measures in individuals with aphasia. See the Figure 10 caption for details, except that here, hot colors indicate positive correlations, while cool colors indicate negative correlations.

Of the 16 findings involving activation increases and/or decreases, two analyses from Tyler et al. (2011) were quite similar, and six analyses from Griffis, Nenert, Allendorfer, and Szaflarski (2017) and Griffis, Nenert, Allendorfer, Vannest, et al. (2017) were quite similar (these two studies were based on the same dataset). After retaining just one representative finding from each of these sets, the remaining 10 findings were plotted (Figure 11A).

All correlations were positive. Since correlations were reported in many brain regions, we will list only regions that were found in two or more analyses; these were the left anterior temporal lobe (Crinion et al., 2006; Warren et al., 2009; Nenert et al., 2018), left IFG pars orbitalis (Fridriksson et al., 2010; Tyler et al., 2011; Griffis, Nenert, Allendorfer, Vannest, et al., 2017), left IFG pars triangularis (Tyler et al., 2011; Griffis, Nenert, Allendorfer, Vannest, et al., 2017), left dorsolateral prefrontal cortex (Allendorfer et al., 2012; Griffis, Nenert, Allendorfer, Vannest, et al., 2017), supplementary motor area/medial prefrontal cortex (SMA/MPFC; Allendorfer et al., 2012; Griffis, Nenert, Allendorfer, Vannest, et al., 2017), left mid temporal region (Papoutsi et al., 2011; Griffis, Nenert, Allendorfer, Vannest, et al., 2017), left pSTS (Crinion & Price, 2005; Papoutsi et al., 2011), and right mid temporal region (two distinct analyses in Crinion & Price, 2005; Tyler et al., 2011). The methodologically robust idiosyncratic findings were diverse. Papoutsi et al. (2011) found that patients with stronger connectivity between the left IFG and the left pMTG had better syntactic function. Griffis, Nenert, Allendorfer, Vannest, et al. (2017) reported that among right hemisphere regions more activated in patients with larger lesions, in the right SMA, activation was positively correlated with semantic fluency in patients with larger lesions, but negatively correlated in patients with smaller lesions, with a statistically significant interaction. Hartwigsen et al. (2020) observed a positive correlation between the extent of upregulation of the right supramarginal gyrus after stimulation of the left posterior IFG and slowing of reaction times on a phonological task.

We next considered all analyses without regard for limitations. We considered only voxelwise or ROI analyses of correlations with measures of language function (of which there were 133 analyses), and not correlations with lesion extent or other nonlinguistic measures (since these would have fundamentally different interpretations depending on the specifics of each analysis, and cannot easily be aggregated). The majority of correlations reported were positive, though there were a number of brain regions in which both positive and negative correlations were reported (Figure 11B). Positive correlations were more prevalent than negative correlations in the left IFG pars triangularis and orbitalis, and the left anterior temporal lobe, with the difference in prevalence significant after correction for multiple comparisons in the left IFG pars triangularis (*p* < 0.0001; Figure 11C).

#### Longitudinal changes in aphasia

**Figure 12.**
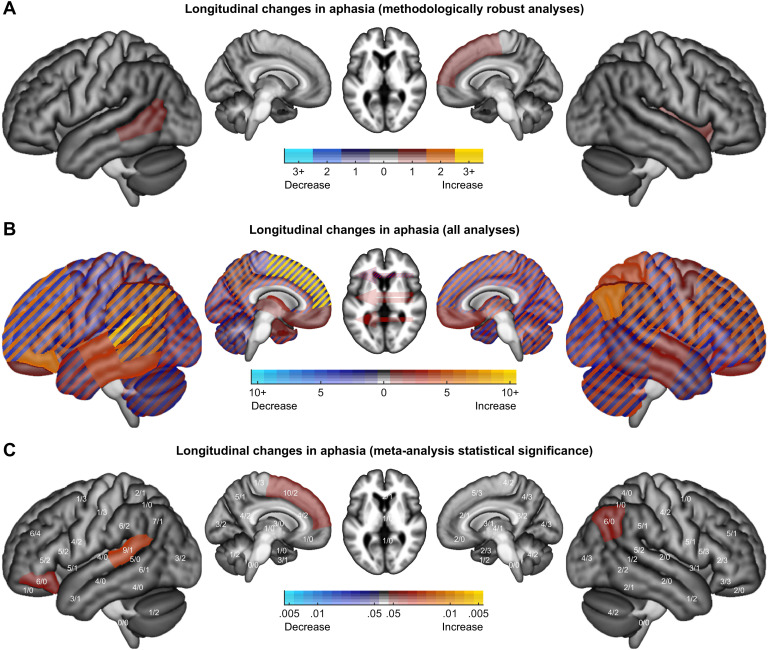
Findings from longitudinal analyses of change over time in individuals with aphasia. See the Figure 10 caption for details, except that here, hot colors indicate increasing activation over time, while cool colors indicate decreasing activation over time.

There have been 78 analyses comparing language activation between two or more time points in groups of individuals with aphasia (Supplementary Table S10). Of these, five analyses (6%) were appraised as methodologically robust (Figure 9; Supplementary Table S13). These five analyses came from three studies (Saur et al., 2006; Nenert et al., 2017; Nenert et al., 2018). Two of the five analyses had findings that could be characterized in terms of activation increases and/or decreases in one or more brain regions, while the other three yielded null results. The two positive findings are plotted in Figure 12A.

Both positive findings were derived from ROI analyses reported by Saur et al. (2006). Activation increased from 2 days post-stroke to 2 weeks post-stroke in the right insula and the right SMA, and activation increased from 2 days post-stroke to 1 year post-stroke in the left pMTG.

Considering all analyses without regard for limitations, we found that activation increases and decreases have been reported for many brain regions (Figure 12B). Increases were most prevalent relative to decreases in the left IFG pars orbitalis, the left pSTG, the left SMA/medial prefrontal cortex, and the right angular gyrus, though none of these patterns were statistically significant after correction for multiple comparisons (Figure 12C).

#### Longitudinal correlation with language or other measure

**Figure 13.**
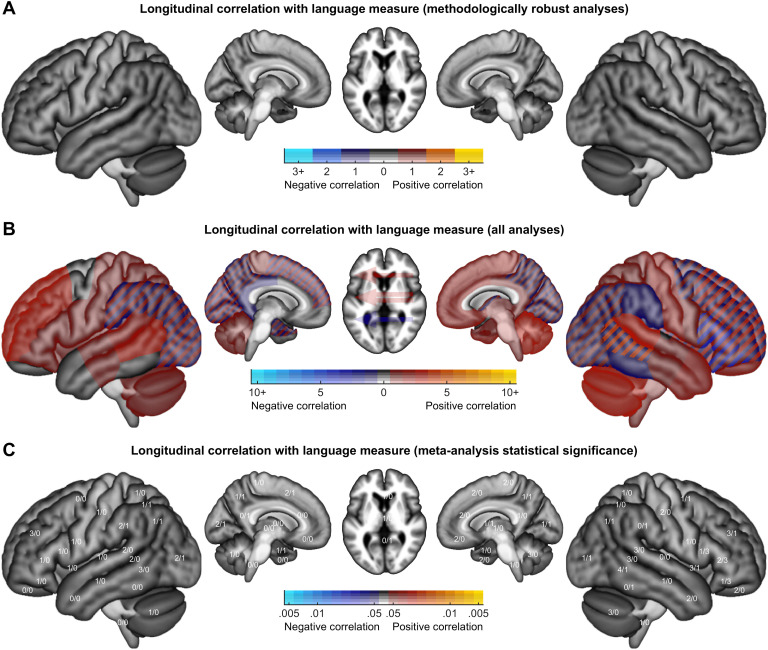
Findings from longitudinal correlations between change in activation and language measures. See the Figure 10 caption for details, except that here, hot colors indicate positive correlations, while cool colors indicate negative correlations. Note that there were no analyses in this class that were appraised as methodologically robust.

There have been 77 analyses in which correlations were computed within a group of individuals with aphasia between *change* in functional activity over time and a measure of language function (usually a measure of change), or another relevant variable (Supplementary Table S10). None of these 77 analyses were appraised as methodologically robust (Figure 9). Accordingly, no brain regions are shown in Figure 13A.

Considering all analyses without regard for limitations, positive correlations have been observed more frequently than negative correlations, but findings have been widely distributed across the brain (Figure 13B) and no patterns were statistically significant (Figure 13C).

#### Other findings

**Figure 14.**
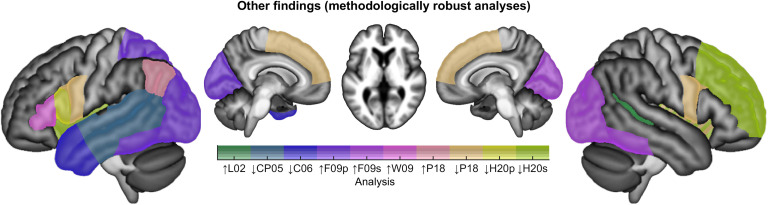
Other findings from methodologically robust analyses. All of these analyses were cross-sectional. ↑L02 = increased activation in Leff et al. (2002), region of interest (ROI) analysis 2; ↓CP05 = decreased activation in Crinion and Price (2005), voxelwise analysis 3; ↓C06 = decreased activation in Crinion et al. (2006), ROI analysis 3; ↑F09p = increased activation in Fridriksson et al. (2009), voxelwise analysis 2; ↑F09s = increased activation in Fridriksson et al. (2009), voxelwise analysis 3; ↑W09 = increased activation in Warren et al. (2009), ROI analysis 11; ↑P18 = increased activation in Pillay et al. (2018), voxelwise analysis 1; ↓P18 = decreased activation in Pillay et al. (2018), voxelwise analysis 1; ↓H20p = decreased activation in Hartwigsen et al. (2020), voxelwise analysis 2; ↓H20s = decreased activation in Hartwigsen et al. (2020), voxelwise analysis 4.

There have been 55 analyses comparing two groups of individuals with aphasia (Supplementary Table S10), of which 11 analyses (20%) were appraised as methodologically robust (Figure 9; Supplementary Table S14). These 11 analyses were described in six studies (Leff et al., 2002; Blank et al., 2003; Crinion & Price, 2005; Crinion et al., 2006; Warren et al., 2009; Hartwigsen et al., 2020). Eight analyses yielded activation increases and/or decreases in one or more brain regions, and three had no significant findings. Six of the eight significant findings are plotted in Figure 14. The findings were as follows: (1) Patients with left pSTS damage showed a greater word rate effect in the right pSTS than those without pSTS damage (Leff et al., 2002); (2) Patients with temporal lobe damage had less posterior temporal activation than those without temporal damage (Crinion & Price, 2005); (3) Patients with posterior temporal damage had reduced activation in the left anterior temporal lobe, compared to those without posterior temporal damage (Crinion et al., 2006); (4) Patients with positive interconnectivity between the anterior temporal lobes had greater activation in the left IFG pars triangularis than those with negative interconnectivity (Warren et al., 2009); (5) Stimulation of the anterior or posterior IFG respectively reduced activation in or adjacent to the stimulated region, as well as several other regions (Hartwigsen et al., 2020); we plotted comparisons between the two stimulation sites, comparisons to sham stimulation were also methodologically robust but are not shown.

There have been five analyses comparing activation in individuals with aphasia between subsets of trials based on performance (Supplementary Table S10), of which four analyses (80%) were appraised as methodologically robust (Figure 9; Supplementary Table S15). This relatively high proportion reflects the fact that these analyses were not required to demonstrate contrast validity in neurologically normal individuals, since people without aphasia do not generally make errors on language tasks. These four analyses were described in three studies (Fridriksson, Baker, & Moser, 2009; Skipper-Kallal et al., 2017a; Pillay et al., 2018). Three analyses yielded activation increases and/or decreases in one or more brain regions, and one reported no significant findings. The three significant findings are plotted in Figure 14. The three findings were as follows: (1) Production of phonological paraphasias was associated with increased activation of left hemisphere posterior extrasylvian regions (Fridriksson et al., 2009); (2) Production of semantic paraphasias involved increased activation of right hemisphere posterior extrasylvian regions (Fridriksson et al., 2009); (3) Correct naming, relative to incorrect naming, was associated with increased activation of the left angular gyrus, and decreased activation of a set of regions in the cingulo-opercular network (Pillay et al., 2018).

There have been two analyses comparing change in language activation over time between individuals with aphasia and neurologically normal participants (Supplementary Table S10), neither of which was appraised as methodologically robust (Figure 9). There have been 21 analyses comparing change in language activation over time between two distinct groups of individuals with aphasia (Supplementary Table S10), none of which were appraised as methodologically robust (Figure 9).

## DISCUSSION

Our systematic review and meta-analysis revealed two main sets of findings. First, we found that most analyses described in the literature to date have been markedly constrained by important limitations, especially related to task performance confounds, contrast validity, and correction for multiple comparisons. Second, we found that only a few claims about language processing in individuals with aphasia are strongly supported by the extant literature, namely, that left hemisphere language regions are less activated in individuals with aphasia than neurologically normal controls, and that left hemisphere language regions, and possibly a temporal lobe region in the right hemisphere, are more activated in individuals with better language function. In contrast, there is only modest, equivocal evidence for the claim that individuals with aphasia differentially recruit right hemisphere homotopic regions, and there is no compelling evidence for differential recruitment of additional left hemisphere regions or domain-general networks. There is modest evidence that left hemisphere language regions return to function over time, but aside from that, there is a striking lack of compelling longitudinal evidence for dynamic reorganization of the language network.

### Methodological Issues

We characterized and appraised the methodology of each study in detail, focusing especially on three major issues that recent work has suggested are critically important: task performance confounds (Geranmayeh et al., 2014), contrast validity (Wilson et al., 2018), and correction for multiple comparisons (Eklund et al., 2016).

#### Task performance confounds

By their very nature, individuals with aphasia are likely to experience difficulty performing language tasks, which may lead to task performance confounds in accuracy and/or reaction time that can have dramatic effects on activation patterns (Binder et al., 2005; Geranmayeh et al., 2014).

We found that for the majority of conditions described, data have not been provided to establish that all groups and individuals could perform the task (Figure 5F). If patients cannot perform a task, then it is difficult to interpret activation maps associated with failure to perform the task (Price & Friston, 1999; Price, Crinion, & Friston, 2006; Wilson et al., 2018). Any differences between patients and controls cannot be readily interpreted as indicative of reorganization, since they may instead reflect failure to engage the language network at all, differences in error detection, other task-related cognitive processes, or feelings of frustration or distress. Other conditions such as rest and passive language comprehension conditions do not require any task to be performed, yet this does not entirely solve the problem, because if there is no task, then there is no way of verifying that participants were engaged in the processes intended.

We found that accuracy and reaction time have rarely been matched between language and control conditions (Figure 6G, 6H), or across participants in second level analyses (Figure 7F, 7G). Task performance confounds have been argued to potentially contribute to or account for many findings in the aphasia neuroplasticity literature (Geranmayeh et al., 2014). Accuracy and reaction time profoundly impact functional signal (Binder et al., 2005; Yarkoni, Barch, Gray, Conturo, & Braver, 2009; Fedorenko, Duncan, & Kanwisher, 2013), with less accurate and/or slower conditions associated with increased bilateral activation of domain general regions in posterior inferior frontal cortex, the anterior insula, inferior parietal cortex, and medial prefrontal cortex, among other brain areas. Many of these regions are immediately adjacent to language regions (Fedorenko, Duncan, & Kanwisher, 2012), and so may be potential loci for reorganization, or may be easily confused with language regions. Moreover, language regions themselves are modulated by task performance in the context of language tasks (Wilson, Isenberg, & Hickok, 2009; Wilson et al., 2014; Wilson et al., 2016). The relationship between task performance and activation may be nonlinear. Taylor, Rastle, and Davis (2014) have proposed that blood oxygen level-dependent signals represent a complex response to engagement and effort. While in their framework, “engagement” refers to the extent to which a given brain area is engaged by a stimulus, “engagement” could also be a relevant concept at the level of the individual participant. For example, an individual with severe aphasia may not “engage” with a linguistic stimulus at all, yielding less activation than normal, while an individual with a milder aphasia may engage with the stimulus but may need to expend additional effort, yielding more activation than normal.

Although task performance confounds are very important, we considered all such limitations to constitute moderate, not major, limitations. There was not a single analysis without at least one task performance-related moderate limitation: therefore, even the findings from analyses that we appraised as methodologically robust need to be carefully interpreted with respect to the potential role of task performance in driving activation patterns.

One simple approach to minimizing task performance confounds is to analyze correct trials only (Price et al., 2006). This is not a panacea, because if processing is more difficult for individuals with aphasia, then even correct trials may have longer reaction times, with attendant consequences for functional activation (Binder et al., 2005; Yarkoni et al., 2009; Wilson et al., 2016). Another potential approach is to use adaptive paradigms in which item difficulty is tailored to individual performance, such that tasks are similarly challenging for all participants, yet within their competence (Wilson et al., 2018; Wilson et al., 2019; Yen et al., 2019).

#### Contrast validity

The functional contrasts commonly used to identify language regions differ markedly in the extent to which they control for nonlinguistic processing and selectively activate left-lateralized perisylvian language regions (Binder et al., 2008; Wilson et al., 2017; Wilson et al., 2018). Therefore, contrast validity needs to be demonstrated empirically in neurologically normal individuals before a contrast can be used to investigate potential reorganization of the language network.

We found that some contrasts have not been matched for visual, auditory, or motor demands (Figure 6C, 6D, 6E), and the majority of contrasts have not been matched for cognitive demands (Figure 6F). Contrasts that are not matched for these basic features would be likely to activate sensory, motor, or cognitive regions, in addition to any language regions that may be activated. For the majority of contrasts, data have not been provided to demonstrate that the contrast selectively activates left-lateralized language regions in neurologically normal individuals (Figure 6I, 6J, 6K).

Although it might seem reasonable to assume that comparing a language condition to a non-language control condition will activate language regions, the brain can be surprisingly indifferent to the logic of subtraction designs. Contrast validity is best assessed in neurologically normal individuals, in whom we have a good understanding of typical language organization (Wilson et al., 2018). In particular, it is established that the great majority of neurologically normal individuals demonstrate three features of language organization: (1) lateralization to the left hemisphere; (2) activation of left inferior frontal cortex; and (3) activation of left posterior temporal cortex (Knecht et al., 2003; Seghier, Kherif, Josse, & Price, 2011; Springer et al., 1999; Tzourio-Mazoyer et al., 2010). Contrasts can be evaluated with respect to whether they are able to consistently reveal these known features of normal language organization.

A potential objection to our perspective might be to claim that most brain regions in both hemispheres are involved in real life language processing: sensory areas, motor areas, domain-general cognitive networks (Fedorenko & Thompson-Schill, 2014), brain regions that encode conceptual representations (Huth, de Heer, Griffiths, Theunissen, & Gallant, 2016), and so on. This is undoubtedly true. However, there is a critically important fact to consider, which is that aphasia results from damage to left hemisphere perisylvian regions, and only rarely from damage to other left hemisphere regions or any right hemisphere regions (Penfield & Roberts, 1959; Selnes, Niccum, Knopman, & Rubens, 1984; Kimura, 1993; Pedersen, Jørgensen, Nakayama, Raaschou, & Olsen, 1995; Croquelois & Bogousslavsky, 2011; Gajardo-Vidal et al., 2018). Accordingly, the central challenge for aphasia neuroplasticity research is to understand how other brain regions can, in some individuals, come to be able to perform the crucial computations that were previously performed by left-lateralized language regions. This entails the necessity of employing language mapping paradigms that reveal *lateralized* aspects of language function. Contrasts that highlight bilateral aspects of language function may be less informative, since any right hemisphere activity observed may be interpreted as the residual component of a bilateral network, rather than bearing on reorganization.

When contrast validity was not established, this was considered a major limitation. This is because activation maps from contrasts that do not activate left-lateralized language regions are likely to reflect other cognitive processes, such as visual, auditory, motor, or cognitive task components, which have often not been matched between conditions. When coupled with the pervasive performance confounds described in the section above, contrasts that load on nonlinguistic processes are likely to reveal between-group differences in visual, auditory, motor, or cognitive processes that may be secondary to task performance confounds.

To ensure contrast validity, paradigms should be selected with reference to the existing literature, and should be psychometrically characterized in neurologically normal participants prior to investigations of individuals with aphasia. Fortunately, because of the important clinical application of fMRI in presurgical language mapping, there is a substantial literature comparing different language mapping paradigms (e.g., Rutten, Ramsey, van Rijen, & van Veelen, 2002; Seghier et al., 2004; Harrington, Buonocore, & Farias, 2006; Binder et al., 2008; Zacà, Nickerson, Deib, & Pillai, 2012; Black et al., 2017; Bradshaw, Thompson, Wilson, Bishop, & Woodhead, 2017; Wilson et al., 2017; Wilson et al., 2018; Yen et al., 2019). Besides validity of the regions activated, it is also important to consider reliability (test–retest reproducibility) and feasibility for individuals with aphasia to perform tasks. One possibility is to use adaptive semantic and phonological matching paradigms, which have been shown to be valid, reliable, and feasible for identifying language regions in individuals with aphasia (Wilson et al., 2018; Wilson et al., 2019; Yen et al., 2019), but many other kinds of paradigms are surely possible, and the field will benefit from a diverse set of approaches.

#### Correction for multiple comparisons

The analysis of functional imaging data usually involves simultaneous inferences about signal changes in multiple brain regions; therefore, it is critically important to correct appropriately for multiple comparisons (Nichols & Hayasaka, 2003; Eklund et al., 2016).

We found that the majority of voxelwise analyses have not been appropriately corrected for multiple comparisons (Figure 8A), and the majority of ROI analyses with more than one ROI have not been corrected for multiple comparisons (Figure 8D). Many of the more complex analyses that have been described also had limitations related to correction for multiple comparisons (Figure 8E).

Voxelwise analyses of whole brain data involve tests over tens or even hundreds of thousands of voxels. A range of approaches have been proposed to control familywise error, most commonly by derivation of voxelwise or cluster extent-based thresholds (Worsley et al., 1992; Worsley et al., 1996; Friston et al., 1994). A recent simulation study showed that while voxelwise thresholds are valid (albeit conservative), the validity of clusterwise approaches strongly depends on the specific details of the procedure (Eklund et al., 2016). In particular, lenient CDTs prior to correction based on cluster extent result in substantial inflation of the nominal false positive rate, so we considered this a moderate limitation, while arbitrary cluster extent cutoffs do not provide any principled control of false positives, which we considered to be a major limitation.

Another common way of analyzing functional imaging data is by defining ROIs. Although ROIs ameliorate the multiple comparisons problem, if there is more than one ROI, it is still necessary to correct for multiple comparisons. In the absence of correction, the actual false positive rate will depend on the number of ROIs and the degree of correlation between them (which is not typically reported). We considered failure to correct for multiple ROIs to constitute a moderate or major limitation depending on how many ROIs there were.

Failure to correct appropriately for multiple comparisons results in findings that reflect random variation in the sample rather than true patterns in the population. Some researchers have argued that lenient thresholds are acceptable because null findings will be “self-erasing,” since they will not be replicated (Nenert et al., 2017). We strongly disagree with this position. The widespread use of excessively lenient approaches has indeed resulted in a failure of most findings to replicate, but the true findings in the literature are very difficult to detect, because they are swamped by a much larger number of findings that are likely to be false positives.

From a technical perspective, correction for multiple comparisons is straightforward. With modern computers, it is now feasible in most cases to perform permutation analyses even on desktop workstations, and we advocate this approach, as implemented in packages such as *SnPM* (Winkler, Ridgway, Webster, Smith, & Nichols, 2014), *randomise* (Winkler et al., 2014), *BROCCOLI* (Eklund, Dufort, Villani, & Laconte, 2014) or *3dttest++* (Cox et al., 2017). ROI analyses also need to be corrected for multiple comparisons. ROI analyses raise an additional concern regarding hidden degrees of freedom in how ROIs are defined, so preregistration should be considered if ROI analyses are planned.

The issue of correction for multiple comparisons leads directly to related issues of sample size and power. Although a number of studies with larger sample sizes have been carried out in the last few years, 32 of the 86 studies we reviewed (37%) included less than a dozen participants, which is often informally considered a minimal sample size for fMRI, and just 15 studies (17%) included two dozen or more participants. Therefore, many of the studies included in our review may be inadequately powered to detect effects of interest (the magnitude of which is essentially unknown). That said, we did not consider small sample size to inherently constitute a major limitation, because even a small study may be capable of convincingly documenting an effect if the effect is large enough. In practice though, we suspect that improper correction for multiple comparisons has often been a response to underpowered studies (Ramus, Altarelli, Jednoróg, Zhao, & Scotto di Covella, 2018), so many smaller studies accrued major limitations that way. As discussed below, we believe that most effects related to neuroplasticity of language processing are likely to be quite subtle and complex, and in the future, large studies with many participants will be necessary to identify functional patterns that will survive correction for multiple comparisons. Collaborative efforts will be essential (Seghier et al., 2016).

### Meta-Analysis of Findings

In evaluating the findings that have been reported in the literature to date, we gave most credence to findings from methodologically robust studies, but we also took note of any patterns that were apparent in the wider set of all analyses, without regard for limitations.

#### Left hemisphere language regions are less activated in individuals with aphasia than in neurologically normal controls

When individuals with aphasia have been compared to neurologically normal controls, most of the findings from methodologically robust analyses have involved activation decreases in individuals with aphasia in left hemisphere language regions or reduced lateralization indices in patients (Sharp et al., 2004; Crinion & Price, 2005; van Oers et al., 2010; Szaflarski et al., 2014; Griffis, Nenert, Allendorfer, & Szaflarski, 2017; Figure 10A: cool colors). Furthermore, consideration of all analyses, without regard for limitations, also revealed many findings of activation decreases in individuals with aphasia in left hemisphere language regions (Figure 10B, 10C: cool colors).

While this general pattern is compelling, it is also not unexpected, since it follows directly from aphasia cohorts having damage to left hemisphere language regions. In many cases, the brain regions in question were wholly or partially destroyed in some or all of the individuals with aphasia. While some studies excluded lesioned voxels from analysis, most did not, so it is inevitable that the brain regions most likely to be damaged in aphasia would show reduced functional activity in patients relative to neurologically normal controls.

A few findings suggest that reduced activation can extend beyond regions that were damaged by stroke, potentially indicative of diaschisis. In particular, Sharp et al. (2004) reported activation decreases in the left fusiform gyrus, a region that was not damaged in any of their participants, and Crinion et al. (2006) showed that patients with posterior temporal damage had reduced activation of undamaged anterior temporal cortex, compared to patients without any temporal damage.

#### Left hemisphere language regions, and possibly a temporal lobe region in the right hemisphere, are more activated in individuals with better language function

Correlations within groups of individuals with aphasia between functional activity and measures of language function have yielded positive relationships in two or more methodologically robust analyses in a number of left hemisphere language regions (Crinion & Price, 2005; Crinion et al., 2006; Warren et al., 2009; Fridriksson et al., 2010; Papoutsi et al., 2011; Tyler et al., 2011; Griffis, Nenert, Allendorfer, Vannest, et al., 2017; Nenert et al., 2018; Figure 11A: warm colors). These correlations have most frequently been reported in semantic regions (the IFG pars orbitalis and the anterior temporal lobe) that are presumably downstream from core language regions. Several more complex methodologically robust analyses have also supported the notion that recruitment of left hemisphere language regions is associated with better language function: (1) Warren et al. (2009) showed that patients with more interconnectivity between their anterior temporal lobes had better language comprehension and more left IFG pars triangularis activation compared to controls, and also compared to patients with less anterior temporal interconnectivity; (2) Papoutsi et al. (2011) found that patients with stronger connectivity between the left IFG and the left pMTG had better syntactic function; (3) Pillay et al. (2018) reported that the left angular gyrus, a key semantic region, showed more activation when reading words correctly compared to when making errors.

Consideration of all correlational analyses, without regard for limitations, yielded broadly similar findings, except that the left IFG pars triangularis was the region with the greatest relative prevalence of positive over negative correlations (Figure 11B, 11C: warm colors). It is not too surprising that activation of left hemisphere language regions should be correlated with better language function. In many cases, these correlations may directly reflect the effect of damage to the regions in question.

Three methodologically robust analyses have yielded positive correlations between right mid temporal activity and language measures (two distinct analyses in Crinion & Price, 2005; Tyler et al., 2011; Figure 11A: warm colors). These correlations are of particular interest, since the right hemisphere was always undamaged. Crinion and Price (2005) observed that the activation of the right mid temporal region did not appear to be compensatory, because the right temporal activation observed was within the normal range observed for the somewhat bilateral contrast employed. They speculated that the correlation may reflect premorbid differences in the capacity of the right temporal lobe for language comprehension.

Indeed, any region performing a compensatory function might not be expected to show a straightforward correlation between activity and language outcome, because patients with the best outcomes may not need to recruit the compensatory region at all (Heiss & Thiel, 2006). Related to this point, Griffis, Nenert, Allendorfer, Vannest, et al.’s (2017) finding that functional activation in the right SMA was correlated with a language measure only in patients with larger lesions is particularly interesting. While this analysis was post-hoc and had a number of limitations including minimal behavioral data and a nonoptimal measure of language function, it was still appraised as methodologically robust, and is a good example of how investigation of complex relationships among structure, function, and behavior will be necessary to move the field forward. Several other studies have investigated these kinds of relationships (Specht et al., 2009; Warren et al., 2009; Fridriksson et al., 2010; Tyler et al., 2010; Wright et al., 2012; Abel et al., 2015; Sims et al., 2016; Griffis, Nenert, Allendorfer, & Szaflarski, 2017; Skipper-Kallal et al., 2017a, 2017b; Stockert et al., 2020), but with a few exceptions (Warren et al., 2009; Griffis, Nenert, Allendorfer, & Szaflarski, 2017), most other structure-function-behavior analyses were not appraised as methodologically robust.

#### Evidence for recruitment of right hemisphere homotopic regions in individuals with aphasia is modest and equivocal

Activation increases in right hemisphere regions homotopic to language regions in individuals with aphasia relative to neurologically normal controls have been observed in three methodologically robust analyses (Leff et al., 2002; two distinct analyses in Blank et al., 2003) (Figure 10A: warm colors). Specifically, Leff et al. (2002) first presented data suggesting that in controls, only the left pSTS showed a linear dependence of activity on word rate, and then showed that six patients with damage to the left pSTS had a steeper dependence of right pSTS activity on word rate compared to eight neurologically normal controls, and also compared to nine patients without left pSTS damage. Although there were no major limitations, the numbers of participants were small, the asymmetry of the word rate dependence effect in controls was modest, and the search region for the critical ROI analysis in the right pSTS was not described. In the other study, Blank et al. (2003) found that seven patients with damage to the left IFG pars opercularis showed more activity in the right IFG pars opercularis than 12 neurologically normal controls for a contrast of propositional speech to rest. The same result was found for another seven patients without damage to the left IFG pars opercularis. Although these analyses had no major limitations, it is noteworthy that the numbers of participants were small, the activation pattern in controls was only somewhat specific to language regions and only somewhat left-lateralized, and small volume correction was used. Furthermore, there were no similar findings when propositional speech was compared to a counting baseline condition. In sum, while Leff et al. (2002) and Blank et al. (2003) are both excellent studies, it is striking that a quarter century of research has produced only three moderately compelling analyses suggesting increased activity in right hemisphere regions homotopic to language regions.

When considering all analyses, without regard to limitations, many more analyses revealed activation increases than activation decreases in right hemisphere homotopic regions, with the difference between increases and decreases almost reaching significance in the right IFG pars opercularis (Figure 10B, 10C: warm colors). This offers some additional support for the possibility that right hemisphere homotopic regions may be differentially recruited in individuals with aphasia.

Taken together with the classical findings that individuals who have recovered from aphasia are vulnerable to subsequent right hemisphere damage or deactivation (Barlow, 1877; Luria, 1963; Kinsbourne, 1971; Basso et al., 1989), the evidence seems to be at least suggestive that the right hemisphere may play an important role in recovery from aphasia, even though this probably does not involve the kind of dramatic large scale reorganization that some early studies suggested (e.g., Weiller et al., 1995).

#### There is minimal evidence for recruitment of additional left hemisphere regions or of domain-general networks

Methodologically robust comparisons between individuals with aphasia and controls have not revealed any recruitment of left hemisphere regions outside the language network, or of domain-general regions (Figure 10A). Most methodologically robust correlational findings have also been in language or semantic regions, with a few notable exceptions (Figure 11A). Fridriksson et al. (2010) reported correlations between activation and naming performance centered on three regions: the left IFG pars orbitalis, the left occipital lobe, and the left anterior cingulate. Patients with better naming showed more activation than controls in these regions. The former two activations could represent expansion of the language network; however, the pars orbitalis activation may alternatively represent semantic processing, while the occipital region is very close to an occipito-temporal activation in the normal controls, and so may reflect visual processing differences between real pictures and abstract pictures. The anterior cingulate activation was interpreted by Fridriksson et al. (2010) as potentially related to attention or error monitoring, that is, recruitment of a domain-general system, but its actual location is ventral to the anterior cingulate regions associated with these functions (e.g., Fedorenko et al., 2013), and so it is more likely to represent a semantic region (Binder, Desai, Graves, & Conant, 2009).

Two other potentially domain-general regions where methodologically robust correlations have been reported between activation and performance are dorsolateral prefrontal cortex (Allendorfer et al., 2012; Griffis, Nenert, Allendorfer, Vannest, et al., 2017) and the SMA (Allendorfer et al., 2012; Griffis, Nenert, Allendorfer, Vannest, et al., 2017). However, the semantic decision and verb generation tasks used in these studies also recruited these regions in controls, so their association with performance in individuals with aphasia does not strongly suggest a compensatory role.

#### There is essentially no evidence for dynamic reorganization of the language network over time

Only 5 out of 132 longitudinal analyses were appraised as methodologically robust, and only 2 of those 5 analyses had positive findings. Specifically, Saur et al. (2006) described ROI analyses in 14 individuals with aphasia showing that activation increased from 2 days post-stroke to 2 weeks post-stroke in the right insula and the right SMA and activation increased from 2 days post-stroke to 1 year post-stroke in the left pMTG (Figure 12A). These analyses did not have major limitations, but as Geranmayeh et al. (2014) discussed in detail, there were dramatic behavioral differences between performance at the three time points in Saur et al.’s (2006) study. We would not interpret Saur et al.’s findings as indicative of reorganization of the language network. Indeed, the authors themselves interpret the right hemisphere increases at 2 weeks as a transient upregulation. The increased activation in the left pMTG at 1 year is more likely to reflect return to function than reorganization, since the left pMTG appears to be a core language region.

When considering all longitudinal analyses, without regard for limitations, there have been more activation increases over time than decreases reported in left hemisphere language regions, especially temporo-parietal regions, and in the right angular gyrus, but none of these ratios were statistically significant after correction for multiple comparisons (Figure 12B, 12C).

The paucity of findings suggests that any macroscopic reorganization of the language network is either very subtle, or is highly variable across individuals. If there is variability across individuals, then it may not be readily accounted for by factors such as aphasia type or lesion location, since many studies have studied subsets of patients defined along such lines, yet still, no compelling findings have emerged.

We do not mean to imply that the language network does not reorganize over time in post-stroke aphasia. Many patients experience substantial recovery (Kertesz & McCabe, 1977; Swinburn et al., 2004; Breitenstein et al., 2017; Holland et al., 2017; Yagata et al., 2017), and behavioral changes can only be explained by neural changes. Our claim is not that functional reorganization does not occur, but only that it has not been observed with the approaches and methodologies that have been used to date.

### Limitations of Our Study

This meta-analysis and systematic review has several noteworthy limitations. First, we must acknowledge that in a project of this scope, errors are inevitable. We made every effort to impartially code and appraise all studies and analyses according to the principles described, but there is no doubt that we will have made some errors. We take full responsibility for any and all misunderstandings or mischaracterizations of the studies that we reviewed.

Second, the manner in which we evaluated the studies, and the specific data items that we defined, were informed by our own perspective on what is important, and we appreciate that other researchers may evaluate studies with respect to different priorities (see, e.g., Crosson et al., 2007; Cocquyt, De Ley, Santens, Van Borsel, & De Letter, 2017), or may elect to aggregate findings without prejudice as to methodological quality (e.g., Schevenels, Price, Zink, De Smedt, & Vandermosten, 2020). Moreover, some of the decisions involved in data extraction were partially subjective. Probably the most important data items with a fair degree of subjectivity were the questions as to whether contrasts selectively activated plausible relevant language regions in neurologically normal individuals, and whether such activations were left-lateralized.

Third, our classification of various kinds of limitations as minor, moderate, or severe could certainly be subject to debate. In particular, we have strong opinions that failure to establish contrast validity (Wilson et al., 2018) and failure to properly correct for multiple comparisons (Eklund et al., 2016) constitute major limitations that fundamentally hinder the interpretation of any resulting findings. While we consider task performance confounds to be vitally important, too (Geranmayeh et al., 2014), we think the inherent challenges of functional imaging of neurological populations make these challenges less tractable (Price et al., 2006; Wilson et al., 2018), so we classified task performance confounds as only moderate limitations. The classification of miscellaneous limitations posed a particular challenge since these limitations were specific to each individual study, so decisions had to be made based on the specific context of the study, rather than applying any systematic rules.

Fourth, in some analyses that did not meet our criteria to be appraised as “methodologically robust,” there were nevertheless mitigating circumstances that may have allowed aspects of the findings to be interpreted. For example, Griffis, Nenert, Allendorfer, Vannest, et al. (2017) is an exceptionally strong study that included many analyses, some of which we appraised as methodologically robust, but others of which we did not. In particular, the first three voxelwise analyses were considered to have major limitations because thresholding was performed with *cluster_threshold_beta*, which certainly does not appropriately control for multiple comparisons. However, importantly, most of the activations that were derived from these analyses were clearly sufficiently extensive that they would have survived a proper correction for multiple comparisons (Griffis, Nenert, Allendorfer, Vannest, et al., 2017, Figure 3A). There are many such analyses throughout the literature that yielded what we believe to be true findings, but that we were unable to confirm as such due to major limitations or large numbers of moderate limitations. It is a drawback of our approach that we had no systematic way of identifying such mitigating factors, and adjusting our appraisal of limitations accordingly. Note that any such procedure would imply assessing limitations at the level of individual findings, rather than analyses (i.e., larger clusters might be considered robust, while smaller clusters might not), which would greatly complicate our analysis.

Fifth, we characterized findings with reference to a few dozen prespecified ROIs. Any brain parcellation scheme is unlikely to capture the actual functional organization of the brain, which is only partly understood; moreover, functional distinctions may be gradient rather than categorical, context-dependent, and subject to individual differences. Moreover, the process of characterizing findings in terms of our ROIs was somewhat subjective.

Sixth, we were able to characterize the findings from most analyses in terms of sets of activation increases and/or decreases in specific brain regions. While this approach successfully captured most of the literature to date, there were several dozen complex analyses with findings that could not be encapsulated in such simple terms, for which we wrote narrative summaries. Although we attempted to integrate these more complex findings as we described each class of findings, narrative summaries are not readily amenable to meta-analysis.

Seventh and finally, we placed little attention on null results. Although analyses that yielded null results were appraised in terms of limitations, and some were classified as “methodologically robust,” we did not attempt to interpret these null findings. This is because, aside from minor or moderate limitations assessed for small sample sizes, we had no way of evaluating power. We believe it is difficult to interpret most if not all of the null results that have been reported, because as mentioned above, sample sizes have often been small, and it has rarely if ever been established that analyses were adequately powered to detect a true effect.

### Future Directions

Progress in understanding neuroplasticity in post-stroke aphasia will depend on developing approaches that address three important methodological issues: task performance confounds (Geranmayeh et al., 2014), contrast validity (Wilson et al., 2018), and correction for multiple comparisons (Eklund et al., 2016). Adaptive language mapping paradigms offer one promising approach, as these paradigms minimize task performance confounds and selectively activate lateralized language regions (Wilson et al., 2018; Wilson et al., 2019; Yen et al., 2019). Correction for multiple comparisons is not technically challenging, but large samples of participants are needed to derive findings that are robust enough to survive correction. We hope that researchers designing and reporting future studies will find Tables 3 through 7 to provide a helpful framework for considering important design issues, and for ensuring that relevant information is reported.

Relatively few robust and replicable findings have emerged from the literature to date. Partly this may reflect methodological limitations, but it may also reflect the heterogeneity of patients with post-stroke aphasia. Future studies that carefully investigate the complex relationships between structural damage, functional activity, and language outcomes may have the most potential for uncovering the neural changes that must surely underlie the recovery trajectories that we observe.

## ACKNOWLEDGMENTS

This research was supported in part by the National Institute on Deafness and Other Communication Disorders. We thank two reviewers for their thoughtful and constructive comments.

## FUNDING INFORMATION

Stephen M. Wilson, National Institute on Deafness and Other Communication Disorders (http://dx.doi.org/10.13039/100000055), Award ID: R01 DC013270. Stephen M. Wilson, National Institute on Deafness and Other Communication Disorders (http://dx.doi.org/10.13039/100000055), Award ID: R21 DC016080.

## AUTHOR CONTRIBUTIONS

Stephen M. Wilson: Conceptualization, Methodology, Data curation, Investigation, Formal analysis, Software, Visualization, Writing—original draft, Funding acquisition. Sarah M. Schneck: Conceptualization, Methodology, Data curation, Investigation, Formal analysis, Writing—review & editing.
