# Supplementary material for: Neuroplasticity in Post-Stroke Aphasia: A Systematic Review and Meta-Analysis of Functional Imaging Studies of Reorganization of Language Processing
Source: Neurobiol Lang (Camb). 2020 Dec 1;2(1):22–82. doi: 10.1162/nol_a_00025 (PMC8057712; doi:10.1162/nol_a_00025)
Supplement: Supplementary file 1 [file nol-2-1-22-s001.pdf]

Neuroplasticity in post-stroke aphasia: A systematic review and meta-analysis of functional imaging studies of reorganization of language processing

Supplementary Tables

Contents

Supplementary Table S1. Participants: Cohorts	2
Supplementary Table S2. Participants: Demographic data	7
Supplementary Table S3. Participants: Characterization of aphasia	12
Supplementary Table S4. Participants: Characterization of neurological status	18
Supplementary Table S5. Imaging: Design	22
Supplementary Table S6. Imaging: Methodology part 1	26
Supplementary Table S7. Imaging: Methodology part 2	30
Supplementary Table S8. Conditions	34
Supplementary Table S9. Contrasts	45
Supplementary Table S10. All analyses	56
Supplementary Table S11. Cross-sectional aphasia compared to control: Methodologically robust analyses	168
Supplementary Table S12. Cross-sectional correlation with language or other measure: Methodologically robust analyses	173
Supplementary Table S13. Longitudinal change in aphasia: Methodologically robust analyses	183
Supplementary Table S14. Cross-sectional between two groups with aphasia: Methodologically robust analyses	184
Supplementary Table S15. Cross-sectional performance-defined conditions: Methodologically robust analyses	186
Supplementary Table S16. Complete coding of all included studies	187

Note

Interactive tables with hyperlinks and tooltips can be accessed at:
https://langneurosci.org/aphasia-neuroplasticity-review

Supplementary Table S1. Participants: Cohorts

Study	Language	Inclusion criteria	N aphasia	N control	Data reuse	Notes
Weiller et al. (1995)	German	Lesion including L pSTG; moderate-to-severe Wernicke's aphasia in the subacute period; now recovered and not aphasic per formal testing; able to perform verb generation task	6	6	N	6 patients were selected from a database of 600 carefully documented cases
Belin et al. (1996)	French	MCA; persistent severe non-fluent aphasia followed by marked improvement with MIT	7	0	N	
Ohyama et al. (1996)	Japanese	Able to repeat single words	16	6	N	
Heiss et al. (1997)	German	—	6	6	N	
Karbe et al. (1998)	German	MCA; able to repeat single words	12	10	N	Only 7 of the 12 patients took part at T2
Cao et al. (1999)	US English	Aphasia with significant recovery over months to years (ADPASS > 70th percentile)	6	37	N	2 additional patients excluded: 1 unable to reliably describe performance post-scan; 1 due to head motion
Heiss et al. (1999)	German	AAT repetition ≥ 50	23	11	N	
Kessler et al. (2000)	German	Mild to moderate aphasia on TT; at least 50 out of 150 on AAT repetition	24	0	N	
Rosen et al. (2000)	US English	L IFG, possibly extending to neighboring regions	6	14	Y	1 participant was reported in a previous case study; of the 14 controls, 6 were studied with PET and 8 with fMRI
Blasi et al. (2002)	US English	L IFG, possibly extending to neighboring regions	8	14	N	
Leff et al. (2002)	UK English	—	15	8	N	
Blank et al. (2003)	UK English	Initial non-fluent aphasia due to anterior perisylvian lesion; subsequently recovered the ability to speak in sentences; patients were divided into those with and without damage to the IFG pars opercularis (POp+: n = 7; POp-: n = 7)	14	12	N	8 of 12 controls included in Blank et al. (2002)
Cardebat et al. (2003)	French	No severe aphasia; no leukoaraiosis	8	6	N	
Sharp et al. (2004)	UK English	Lesion in vicinity of L STG; no extensive frontal damage; no inferior temporal damage; able to perform tasks	9	18	N	
Zahn et al. (2004)	German	Global aphasia in the first three months; some improvement of comprehension within 6-12 months	7	14	N	
Crinion & Price (2005)	UK English	—	17	18	N	
de Boissezon et al. (2005)	French	Subcortical stroke; no severe aphasia	7	0	N	
Connor et al. (2006)	US English	L IFG, possibly extending to neighboring regions	8	14	Y	Re-analysis of data from Blasi et al. (2002)
Crinion et al. (2006)	UK English	—	24	11	N	Results of control participants previously reported in Crinion et al. (2003)
Saur et al. (2006)	German	MCA; age < 70 years; able to distinguish forward vs backward speech outside the scanner; no pronounced small vessel disease	14	14	N	4 additional patients excluded: 1 health problems; 1 scanner noise; 2 did not tolerate fMRI; 198 patients with aphasia were screened
Meinzer et al. (2008)	German	—	11	0	N	

Raboyeau et al. (2008)	French	Naming deficit; good comprehension	10	20	N	
Richter et al. (2008)	German	Main deficits in production rather than comprehension	16	8	N	8 additional patients excluded: 5 completed only one of the two sessions; 3 unable to perform the tasks
de Boissezon et al. (2009)	French	Only part of L MCA; able to perform word generation; no severe aphasia	13	0	Y	7 out of 13 patients appear to represent the same data reported in de Boissezon et al. (2005)
Fridriksson et al. (2009)	US English	—	11	10	N	
Menke et al. (2009)	German	Moderate to severe anomia	8	9	N	
Specht et al. (2009)	German	—	12	12	N	15 controls were scanned but 3 were randomly excluded to match group sizes for jICA.
Warren et al. (2009)	UK English	Comprehension deficit per CAT and TROG (1 patient did not meet this criterion); anterolateral superior temporal cortex spared	16	11	Y	8 additional patients excluded: lesions involved L anterolateral superior temporal cortex; reanalysis of subset of dataset from Crinion et al. (2006)
Chau et al. (2010)	Cantonese	—	7	0	N	
Fridriksson (2010)	US English	—	19	0	Y	7 additional patients excluded: 6 for making fewer than 5 correct responses in one or more sessions; 1 for excessive head motion; "several" patients overlapped with those reported by Fridriksson et al. (2009, 2010); demographic data includes excluded patients
Fridriksson et al. (2010)	US English	—	15	9	N	
Sharp et al. (2010)	UK English	Lesion in vicinity of L STG; no extensive frontal damage; no inferior temporal damage; able to perform tasks	9	18	Y	Additional analysis of same dataset as Sharp et al. (2004)
Thompson et al. (2010)	US English	Agrammatic	6	12	N	
Tyler et al. (2010)	UK English	—	14	10	N	2 of the 14 patients were not stroke, but were post resective surgery
van Oers et al. (2010)	Dutch	MCA; mRS < 3; able to perform at least 2 out of the 3 tasks	13	13	N	
Papoutsi et al. (2011)	UK English	—	14	15	Y	Reanalysis of same dataset from Tyler et al. (2011); 1 patient had post-surgical haematoma rather than stroke (per Tyler et al., 2011)
Sebastian & Kiran (2011)	US English	—	8	8	N	
Szaflarski et al. (2011)	US English	Moderate aphasia, L MCA	8	0	N	3 additional patients excluded: 2 metallic artifact; 1 seizure at time of stroke
Tyler et al. (2011)	UK English	—	14	15	Y	Not stated, but it seems like most of the patients also participated in Tyler et al. (2010); 1 patient had post-surgical haematoma rather than stroke
Weiduschat et al. (2011)	German	Age 55-85	10	0	N	4 additional patients excluded: 3 malfunction of TMS device or claustrophobia; 1 recovered nearly completely prior to intervention
Allendorfer et al. (2012)	US English	MCA; moderate-severe aphasia; mRS ≤ 3	16	32	Y	"Part of a larger ongoing study", may overlap with other studies from this group
Fridriksson, Hubbard, et al. (2012)	US English	Broca's aphasia	10	20	N	3 additional patients excluded: 1 due to a metal implant; 2 for severely non-fluent

						speech; demographic data includes excluded patients
Fridriksson, Richardson, et al. (2012)	US English	—	29	14	Y	1 additional patient excluded: contraindications to MRI; 26 of 30 patients were included in Fridriksson (2010); demographic data includes excluded patient
Marcotte et al. (2012)	Canadian French	Moderate-severe aphasia; anomia	9	0	N	
Schofield et al. (2012)	UK English	Comprehension deficit	20	26	Y	1 additional patient excluded: excessive head motion; patients recruited from database so may have participated in prior studies from this group, but not stated explicitly; demographic data includes excluded patient
Wright et al. (2012)	UK English	—	21	21	Y	Unclear how many, if any, patients were included in previous studies from this group; design is identical to Tyler et al. (2010); 3 of the 21 patients were not stroke, but were post resective surgery
Szaflarski et al. (2013)	US English	—	27	0	N	
Thiel et al. (2013)	German	—	24	0	N	6 additional patients excluded: 4 did not tolerate MRI or PET scans; 2 TMS device was defective
Abel et al. (2014)	German	Anomia; no severe AoS or dysarthria	14	0	N	9 additional patients excluded: 4 for ceiling performance; 5 for technical problems
Benjamin et al. (2014)	US English	"at least minimal evidence of non-fluent output"; lesion including precentral gyrus or underlying white matter	14	0	N	
Brownsett et al. (2014)	UK English	No involvement of ACA territory	16	17	N	3 additional patients excluded: 2 withdrew after attempting first scan; 1 had severe dysarthria
Mattioli et al. (2014)	Italian	L MCA; comprehension mildly impaired	12	10	N	Treated and untreated groups differed in severity at baseline, albeit not significantly
Mohr et al. (2014)	UK English	MCA; mild-moderate non-fluent aphasia; no severe comprehension deficit	6	0	N	6 additional patients excluded: 4 for health risks; 2 for technical problems and data loss; patient numbers in tables 1 and 2 appear not to correspond with patient numbers later in the paper
Robson et al. (2014)	UK English	Wernicke's aphasia (impaired spoken single word comprehension, impaired single word repetition, fluent, sentence-like speech with phonological/neologistic errors)	12	12	N	
Szaflarski et al. (2014)	US English	—	32	32	Y	Some participants included in Allendorfer et al. (2012); one participant was < 18 years old at time of stroke; there was also a perinatal stroke group, not relevant for this review; 3 participants were excluded but it is not stated whether they were adult or perinatal patients.
van Hees et al. (2014)	Australian English	—	8	14	N	
Abel et al. (2015)	German	Anomia; no severe AoS or dysarthria	14	14	Y	9 additional patients excluded: 4 for ceiling performance; 5 for technical problems; same dataset as Abel et al. (2014)
Kiran et al. (2015)	US English	Impaired naming	8	8	N	
Sandberg et al. (2015)	US English	—	10	0	N	

Geranmayeh et al. (2016)	UK English	No severe receptive aphasia	53	24	N	Prior strokes were allowed only if no aphasia resulted
Griffis et al. (2016)	US English	Moderate aphasia, L MCA	8	0	Y	3 additional patients excluded: 2 metallic artifact; 1 seizure at time of stroke; same patients as Szaflarski et al. (2011); different fMRI paradigm acquired in the same sessions
Sims et al. (2016)	US English	Some spared tissue in L IFG	14	8	Y	2 additional patients excluded: 1 had no spared tissue in the L IFG; 1 had a R hemisphere stroke; although not stated, it is apparent that many of the patients were included in Sandberg et al. (2015)
Darkow et al. (2017)	German	L hand motor area spared; mild aphasia	16	16	N	
Geranmayeh et al. (2017)	UK English	—	27	0	Y	Patients are a subset of those in Geranmayeh et al. (2016); 24 control participants are described, but no imaging data from the controls are analyzed in this paper
Griffis, Nenert, Allendorfer, & Szaflarski (2017)	US English	—	43	43	Y	Same dataset as Griffis et al. (2017) Hum Brain Mapp
Griffis, Nenert, Allendorfer, Vannest, et al. (2017)	US English	—	43	43	Y	Data were collected as part of "several separate studies"
Harvey et al. (2017)	US English	Mild-moderate non-fluent aphasia; relatively intact comprehension; able to produce meaningful words and phrases	6	0	N	
Nardo et al. (2017)	UK English	Anomia; good single word comprehension; relatively spared word and nonword repetition; no AoS; spared or partially spared L IFG	18	0	N	
Nenert et al. (2017)	US English	At least mild aphasia per TT	19	38	Y	Patients are a subset of the 24 participants in Szaflarski et al. (2015), a clinical trial on CIAT
Qiu et al. (2017)	Mandarin	Broca's aphasia	10	10	N	
Skipper-Kallal et al. (2017a)	US English	Able to name 20% of pictures correctly in the scanner	32	25	Y	14 additional patients excluded: < 20% accuracy in scanner; 29 of the participants overlap with the other Skipper-Kallal et al. (2017) paper
Skipper-Kallal et al. (2017b)	US English	10% accuracy on scanner task	39	37	Y	10 additional patients excluded: < 10% accuracy in scanner; 29 of the participants overlap with the other Skipper-Kallal et al. (2017) paper
Dietz et al. (2018)	US English	—	12	0	Y	2 additional patients excluded: 1 for illness; 1 for MRI contraindication or personal conflict (inconsistent information provided); same data as Dietz et al. (2016), which is a methodological paper
Hallam et al. (2018)	UK English	Semantic aphasia; left frontal damage (+ other regions, typically)	14	16	N	
Nenert et al. (2018)	US English	Aphasia at acute screening (not necessarily at first study time point)	17	85	N	1 additional patient excluded: significant signal artifacts; presence and severity of aphasia assessed on hospital admission, not at first study time point, so it is not clear that all participants actually had aphasia at first study time point
Pillay et al. (2018)	US English	Residual phonologic retrieval deficit; intact semantic processing	21	0	N	

Szaflarski et al. (2018)	US English	—	12	0	N	1 additional patient excluded: scanned at only 2 out of 3 time points
van de Sandt-Koenderman et al. (2018)	Dutch	Severe non-fluent aphasia (< 50 words/minute); articulation deficits; repetition severely affected; moderate-good auditory comprehension	9	0	N	
van Oers et al. (2018)	Dutch	MRS ≤ 3; ability to perform tasks	12	8	N	
Barbieri et al. (2019)	US English	—	18	23	N	1 additional patient excluded: developed a hematoma between baseline and post-testing; one patient had two strokes within one day, but we would consider that essentially a single stroke
Johnson et al. (2019)	US English	Anomia	30	17	N	5 additional patients excluded: 2 withdrew from non-treatment arm; 3 fMRI acquisition errors; 1 did not complete treatment and post-treatment scanning (but of these latter 4, one must have at least completed the non-treatment arm); there were 26 patients in the treated group and 10 in the untreated group, but 6 patients overlapped between the two groups (they joined the treated group after completing the untreated phase)
Kristinsson et al. (2019)	US English	< 80% on PNT; able to name at least 5 out of 40 items during fMRI; WAB-R spontaneous speech ≥ 2; WAB-R auditory comprehension ≥ 2	87	0	Y	65 were previously included in Fridriksson et al. (2018), a tDCS study
Purcell et al. (2019)	US English	Chronic dysgraphia (acquired impairment in spelling)	21	0	N	4 additional patients excluded: 3 health reasons; 1 data acquisition error
Sreedharan, Chandran, et al. (2019)	Malayalam	Broca's aphasia or anomic aphasia; comprehension relatively preserved; "motivated for speech therapy"	8	4	N	3 additional patients excluded: 2 for claustrophobia; 1 for transportation issues
Hartwigsen et al. (2020)	German	Lesion involving left temporo-parietal cortex and sparing left frontal cortex; relatively well-recovered	12	0	N	2 additional patients excluded: 1 lost to follow-up; 1 did not show any sound-related neural activation in auditory cortex after sham cTBS
Stockert et al. (2020)	German	Lesion localized to frontal or temporal cortex	34	17	Y	50 additional patients excluded: 19 lesions spanned frontal and temporal, or were subcortical, or had persisting large vessel occlusions; 31 not all three timepoints were acquired; 8 patients were included in Saur et al. (2006); there may also be overlap with Saur et al. (2010), a study that did not meet our inclusion criteria; 1630 patients screened for inclusion; frontal patients scanned later than temporal patients at T1 and T2

N aphasia = Number of individuals with aphasia; N control = Number of control participants; Data reuse = Were any of the participants included in any previous studies?; AAT = Aachen Aphasia Test; ACA = anterior cerebral artery; ADPASS = Aphasia Diagnostic Profiles Aphasia Severity Score; AoS = apraxia of speech; CAT = Comprehensive Aphasia Test; CIAT = constraint-induced aphasia therapy; fMRI = functional magnetic resonance imaging; IFG = inferior frontal gyrus; jICA = joint independent components analysis; L = left; MCA = middle cerebral artery; MIT = melodic intonation therapy; mRS = modified Rankin Scale; N = No; PET = positron emission tomography; POp+ = pars opercularis damaged; POp- = pars opercularis spared; pSTG = posterior superior temporal gyrus; R = right; STG = superior temporal gyrus; T1, T2, etc. = first time point, second time point, etc.; TMS = transcranial magnetic stimulation; TROG = Test for Reception of Grammar; TT = Token Test; Y = Yes; Yellow underline = minor limitation; Orange underline = moderate limitation.

Supplementary Table S2. Participants: Demographic data

Study	Age	Sex	Handedness	Time post onset
Weiller et al. (1995)	N (mean 58 years, range 50-66 years; controls were younger: mean 35 years; range 27-50 years)	Y (6 M/0 F)	Y (6 R/0 L)	Y (range 5-117 months)
Belin et al. (1996)	Y (mean 49.7 years, range 40-58 years)	N	Y (7 R/0 L)	Y (range 15-149 months; including MIT for the most recent 1-108 months)
Ohyama et al. (1996)	Y (mean 56.6 ± 11.8 years, range 38-75 years)	Y (12 M/4 F)	Y (16 R/0 L)	N* (mean 15.1 ± 16.7 months, range 1.1-50.3 months; a mix of subacute and chronic participants; 8 of each)
Heiss et al. (1997)	Y (range 33-66 years)	Y (4 M/2 F)	Y (6 R/0 L)	Y (T1: ~4 weeks; T2: ~12-18 months)
Karbe et al. (1998)	N (mean 57 years, range 34-78 years; controls not matched for age)	Y (7 M/5 F); stated to be not matched, but difference not significant	Y (12 R/0 L)	Y (T1: mean 24 ± 11 days, ~3-4 weeks; T2: mean 19 ± 2 months, > 1 year)
Cao et al. (1999)	Y (range 20-56 years)	Y (1 M/5 F)	Y (6 R/0 L)	Y (range 5-32 months)
Heiss et al. (1999)	Y (mean 56 ± 12 years, range 31-77 years; assume patient's age of 5.6 years is a typo for 56 years)	Y (15 M/8 F)	Y (23 R/0 L)	Y (T1: ~2 weeks; T2: ~8 weeks)
Kessler et al. (2000)	Y (piracetam group: mean 57.4 ± 13.5 years; placebo group: mean 56.3 ± 10.0 years)	Y (13 M/11 F)	Y (24 R/0 L)	Y (T1: ~2 weeks; T2: ~8 weeks)
Rosen et al. (2000)	N (mean 47 years, range 32-72 years; control participants not age-matched)	Y (3 M/3 F)	Y (6 R/0 L)	Y (range 0.5-7.6 years)
Blasi et al. (2002)	N (mean 48.6 years; patients and controls not closely matched for age, unclear if difference significant)	Y (2 M/6 F)	Y (8 R/0 L)	N (> 6 months; actual TPO not stated)
Leff et al. (2002)	Y (range 43-76 years)	Y (11 M/4 F)	Y (11 R/0 L)	Y (range 5-76 months)
Blank et al. (2003)	Y (POp+: median 50 years, range 36-72 years; POp-: median 61 years, range 39-70 years)	Y (8 M/6 F)	Y (14 R/0 L)	Y (POp+: median 39 months, range 19-134 months; POp-: median 17 months, range 6-240 months)
Cardebat et al. (2003)	Y (mean 58.4 ± 11.9 years, range 37-73 years)	Y (7 M/1 F)	Y (8 R/0 L)	N* (T1: 58 ± 35 days, range 11-113 days; T2: 11.7 ± 1.6 months, range 320-460 days; T1 varies considerably from early to late subacute)
Sharp et al. (2004)	Y (median 58 years, range 39-72 years)	Y (8 M/1 F)	Y (9 R/0 L)	Y (mean 45 months, range 14-145 months)
Zahn et al. (2004)	Y (range 29-67 years)	Y (6 M/1 F)	Y (7 R/0 L)	Y (range 6 months-4 years)
Crinion & Price (2005)	Y (mean 62 ± 2.7 SEM years, range 34-75 years)	Y (12 M/5 F)	Y (17 R/0 L)	Y (range 4-125 months; aphasia with temporal damage (n=8) mean 41 months; aphasia without temporal damage (n=9) mean 48 months)
de Boissezon et al. (2005)	Y (mean 52.4 ± 13 years, range 31-69 years)	Y (7 M/0 F)	Y (7 R/0 L)	N* (T1: mean 53 ± 35 days, range 11-108 days; T2: mean 12.2 ± 1.4 months; T1 varies considerably from early to late subacute)
Connor et al. (2006)	N (mean 48.6 years; patients and controls not closely matched for age, unclear if difference significant)	Y (2 M/6 F)	Y (8 R/0 L)	N (> 6 months; actual TPO not stated)
Crinion et al. (2006)	Y (range 32-85 years)	Y (18 M/6 F)	Y (24 R/0 L)	N (mean 32 months, range 2-204 months; combines subacute and chronic patients)
Saur et al. (2006)	Y (mean 51.9 ± 14.2 years, range 16-68 years)	Y (11 M/3 F)	Y (12 R/1 L)	Y (T1 acute: mean 1.8 days, range 0-4 days; T2 subacute: mean 12.1 days,

				range 3-16 days; T3 chronic: mean 321 days, range 102-513 days)
Meinzer et al. (2008)	Y (median 51.0 years, range 19-66 years)	Y (7 M/4 F)	Y (11 R/0 L)	Y (median 32 months; range 6-480 months)
Raboyeau et al. (2008)	N (mean 53.8 ± 14.7 years; controls were younger)	Y (6 M/4 F)	Y (10 R/0 L)	Y (range 7-102 months)
Richter et al. (2008)	Y (mean 58.3 years; range 42-73 years)	Y (12 M/4 F)	Y (16 R/0 L)	N (> 12 months; actual TPO not stated)
de Boissezon et al. (2009)	Y (range 31.2-74.2 years)	Y (12 M/1 F)	Y (13 R/0 L)	N* (T1: mean 64 ± 32 days; T2: mean 11.8 ± 1.4 months; T1 varies considerably from early to late subacute)
Fridriksson et al. (2009)	Y (mean 58.8 ± 14.7 years, range 33-78 years)	Y (6 M/5 F)	N	Y (range 10-101 months)
Menke et al. (2009)	Y (range 34-67 years)	Y (5 M/3 F)	Y (8 R/0 L)	Y (range 1.8-6.9 years)
Specht et al. (2009)	N (mean 49 + 14 years, range 30-71 years; controls were younger)	Y (9 M/3 F)	N	N (mean 1.9 ± 1.4 years, range 0.2-3.7 years; one non-chronic patient is included)
Warren et al. (2009)	N (mean 65.8 ± 2.0 SEM years; controls were younger)	Y (11 M/5 F)	Y (16 R/0 L)	N (mean 28.8 ± 9.2 months SEM; minimum time post onset not reported, but some patients in Crinion et al. (2006) were subacute)
Chau et al. (2010)	Y (mean 63 ± 10 years, range 56-79 years)	Y (5 M/2 F)	Y (7 R/0 L)	Y (mean 17 ± 8 months, range 8-28 months)
Fridriksson (2010)	Y (mean 59.7 ± 12.3 years)	Y (12 M/14 F)	N	Y (> 8 months; actual TPO not stated)
Fridriksson et al. (2010)	Y (mean 61.9 years, range 41-81 years)	N (7 M/8 F); not stated for controls	N	Y (mean 29.7 months, > 6 months)
Sharp et al. (2010)	Y (median 58 years, range 39-72 years)	Y (8 M/1 F)	Y (9 R/0 L)	Y (mean 45 months, range 14-145 months)
Thompson et al. (2010)	Y (mean 54 years, range 38-66 years)	Y (5 M/1 F)	Y (6 R/0 L)	Y (range 6-146 months)
Tyler et al. (2010)	Y (mean 54 years, range 33-76 years)	Y (11 M/3 F)	Y (14 R/0 L)	Y (mean 7 years, range 1.4-37.3 years)
van Oers et al. (2010)	Y (mean 53 ± 14 years, range 29-74 years)	Y (4 M/9 F)	N (13 R/0 L); not stated for controls	Y (range 1.3-4.7 years)
Papoutsi et al. (2011)	Y (mean 56 ± 12 years, range 35-77 years)	Y (11 M/3 F)	Y (14 R/0 L)	Y (mean 8 ± 9 years, range 2-40 years)
Sebastian & Kiran (2011)	Y (range 40-79 years)	N (5 M/3 F); control sex not stated, but reported to be matched	Y (8 R/0 L)	Y (mean 48.3 months, range 30-78 months)
Szaflarski et al. (2011)	Y (mean 54.4 ± 12.7 years)	Y (4 M/4 F)	Y (8 R/0 L)	Y (mean 5.3 ± 3.6 years, > 12 months)
Tyler et al. (2011)	Y (mean 56 years, range 34-77 years)	Y (11 M/3 F)	Y (14 R/0 L)	Y (mean 7 years, > 1.5 years)
Weiduschat et al. (2011)	Y (range 59-83 years)	Y (5 M/5 F)	Y (10 R/0 L)	Y (range 18-97 days; patients at different subacute stages of recovery)
Allendorfer et al. (2012)	Y (mean 54.4 ± 9.5 years, range 38-78 years)	Y (9 M/7 F)	Y (16 R/0 L)	Y (mean 3.7 ± 3.5 years, range 0.5-11.4 years)
Fridriksson, Hubbard, et al. (2012)	Y (mean 56.9 ± 9.2 years, range 45-75 years)	N (9 M/4 F); control sex not matched	Y (12 R/1 L)	Y (mean 63.8 ± 64.3 months, range 10-261 months)
Fridriksson, Richardson, et al. (2012)	Y (mean 59.2 years, range 33-81 years)	N (14 M/16 F); not stated for controls	N	Y (mean 51.1 months, range 6-350 months)
Marcotte et al. (2012)	Y (mean 62 ± 6.0 years, range 50-67 years)	Y (5 M/4 F)	Y (9 R/0 L)	Y (mean 110.2 ± 92.5 months, range 50-300 months)

Schofield et al. (2012)	Y (range 35.8-90.3 years)	N (16 M/4 F); control sex not stated	N	Y (mean 3.5 years, range 0.6-8.6 years)
Wright et al. (2012)	Y (mean 57.4 ± 12.5 years)	Y (15 M/6 F)	Y (21 R/0 L)	Y (mean 6.5 ± 7.5 years, > 1.4 years)
Szaflarski et al. (2013)	Y (recovered: mean 50 ± 13 years; non-recovered: mean 51 ± 13 years)	Y (15 M/12 F)	Y (27 R/0 L)	Y (recovered: mean 2.1 ± 2.1 years; non-recovered: mean 4.9 ± 3.1 years)
Thiel et al. (2013)	Y (rTMS group: mean 69.8 ± 8.0 years; sham group: mean 71.2 ± 7.8 years)	N	Y (24 R/0 L)	Y (rTMS group: mean 37.5 ± 18.5 days; sham group: mean 50.6 ± 22.6 days)
Abel et al. (2014)	Y (median 48 years, range 35-74 years)	Y (10 M/4 F)	Y (14 R/0 L)	Y (median 41 months, range 11-72 months)
Benjamin et al. (2014)	Y (intention group: mean 72.1 ± 10.5 years; control group: mean 63.0 ± 9.2 years)	Y (8 M/6 F)	Y (14 R/0 L)	Y (intention group: mean 37.4 ± 33.5 months, range 12-87 months; control group: 38.1 ± 37.4 months, range 10-112 months)
Brownsett et al. (2014)	Y (mean 60 years, range 37-84 years)	Y (11 M/5 F)	Y (16 R/0 L)	Y (mean 4 years, range 6 months-11 years)
Mattioli et al. (2014)	N (range 37-79 years; control ages not reported, though reported to be matched)	N (7 M/5 F); control sex not stated, but reported to be matched	Y (12 R/0 L)	Y (T1: mean 2.2 ± 1.3 days; T2: mean 16.2 ± 1.3 days; T3: mean 190 ± 25.5 days)
Mohr et al. (2014)	Y (range 41-76 years)	Y (5 M/1 F)	Y (6 R/0 L)	Y (range 17-234 months (including excluded patients))
Robson et al. (2014)	Y (mean 70.1 ± 8.7 years, range 59-87 years)	Y (10 M/2 F)	Y (12 R/0 L)	Y (range 7-84 months)
Szaflarski et al. (2014)	Y (mean 51.8 ± 15.1 years)	Y (18 M/14 F)	N	Y (mean 3.2 ± 3.1 years, > 6 months)
van Hees et al. (2014)	Y (mean 56.4 + 9.2 years; range 41-69 years)	Y (3 M/5 F)	Y (8 R/0 L)	Y (mean 52.3 + 49.8 months; range 17-170 months)
Abel et al. (2015)	Y (median 48 years, range 35-74 years)	Y (10 M/4 F)	Y (14 R/0 L)	Y (median 41 months, range 11-72 months)
Kiran et al. (2015)	Y (mean 58 years)	Y (7 M/1 F)	N	Y (range 15-157 months)
Sandberg et al. (2015)	Y (mean 59 years, range 47-75 years)	Y (7 M/3 F)	Y (10 R/0 L)	Y (range 7-134 months)
Geranmayeh et al. (2016)	Y (mean 62 ± 14 years, range 26-83 years)	N (32 M/21 F); controls were mostly female, unlike patients	Y (50 R/3 L)	Y (mean 111 ± 27 days, range 84-200 days)
Griffis et al. (2016)	Y (mean 54.4 ± 12.7 years)	Y (4 M/4 F)	Y (8 R/0 L)	Y (mean 5.3 ± 3.6 years)
Sims et al. (2016)	Y (mean 59.7 years, range 48-75 years)	Y (10 M/4 F)	Y (14 R/0 L)	Y (mean 6 years, range 6 months-13 years)
Darkow et al. (2017)	Y (mean 56.7 ± 10.1 years)	Y (10 M/6 F)	Y (16 R/0 L)	Y (mean 54.3 ± 45.3 months, range 12-169 months)
Geranmayeh et al. (2017)	Y (mean 59.1 ± 10.8 years, range 39-77 years)	Y (18 M/9 F)	Y (26 R/1 L)	Y (T1: 15 ± 7.6 days (range 5-35 days); T2: 108 ± 26 days (range 87-200 days))
Griffis, Nenert, Allendorfer, & Szaflarski (2017)	Y (mean 53 ± 15 years, range 23-90 years)	Y (25 M/18 F)	Y (41 R/2 L)	Y (range 1-14 years)
Griffis, Nenert, Allendorfer, Vannest, et al. (2017)	Y (mean 53 ± 15 years, range 23-90 years)	Y (25 M/18 F)	Y (41 R/2 L)	Y (range 1-14 years)
Harvey et al. (2017)	Y (range 47-75 years)	Y (5 M/1 F)	Y (6 R/0 L)	Y (range 6-102 months)
Nardo et al. (2017)	Y (mean 50 ± 12 years, range 21-67 years)	Y (12 M/6 F)	Y (18 R/0 L)	Y (mean 61 ± 58 months, range 5-264 months)
Nenert et al.	Y (CIAT group: mean 58.0 ± 10.6	Y (11 M/8 F)	N (17 R/0 L); 2	Y (CIAT group: mean 60.2 ± 48.9

(2017)	years; untreated group: mean 50.3 ± 13.3 years)		patients "atypical": unclear whether L or mixed	months; untreated group: mean 41.9 ± 30.0 months; all > 1 year)
Qiu et al. (2017)	Y (mean 55.9 ± 13.4 years, range 40-70 years)	Y (7 M/3 F)	Y (10 R/0 L)	Y (range 1-3 months)
Skipper-Kallal et al. (2017a)	Y (mean 58.8 ± 8.6 years, range 45.7-78.2 years)	Y (19 M/12 F); stated to be not matched, but difference not significant	Y (26 R/3 L)	Y (mean 40.9 ± 36.1 months, 4.9-151.0 months)
Skipper-Kallal et al. (2017b)	Y (mean 59.8 ± 10.0 years)	Y (26 M/13 F)	Y (33 R/4 L); missing for 2 participants	Y (mean 52.9 ± 51.4 months, range 6.3-255.7 months)
Dietz et al. (2018)	Y (AAC group: range 39-63 years; usual care group: range 47-71 years)	Y (5 M/7 F)	Y (11 R/1 L)	Y (AAC group: range 16-170 months; usual care group: range 38-105 months)
Hallam et al. (2018)	Y (mean 61 ± 11 years, range 38-80 years)	Y (5 M/9 F)	N	Y (range 11-264 months)
Nenert et al. (2018)	Y (mean 46 ± 16 years)	Y (9 M/8 F)	N (17 R/0 L); all patients stated to be right handed, but "ambidextrous patients" mentioned on p. 364	Y (T1: ~2 weeks; T2: ~6 weeks; T3: ~12 weeks; T4: ~26 weeks; T5: ~52 weeks)
Pillay et al. (2018)	Y (mean 56.4 ± 12.5 years, range 30-80 years)	Y (11 M/10 F)	Y (21 R/0 L)	Y (mean 1134 ± 1491 days, range 180-6732 days)
Szaflarski et al. (2018)	Y (range 26-66 years)	Y (9 M/3 F)	Y (11 R/1 L)	Y (range 1-12 years)
van de Sandt-Koenderman et al. (2018)	Y (subacute: mean 51.2 years, range 25-61 years; chronic: mean 54.0 years, range 21-66 years)	Y (5 M/4 F)	Y (8 R/0 L)	Y (subacute: range 0.5-3 months; chronic: range 17-40 months)
van Oers et al. (2018)	Y (mean 67.9 ± 11.4 years, range 46-86 years)	Y (10 M/2 F)	Y (12 R/0 L)	N* (T1: within 2 weeks; T2: ~3 months; T3: ~6 months; T4: ~12 months; specific timing of first time point not stated)
Barbieri et al. (2019)	N (range 22-73 years; controls were younger)	Y (11 M/7 F)	N (15 R/3 L); not stated for controls	Y (range 13-107 months)
Johnson et al. (2019)	Y (treated group: mean 62.8 ± 10.2 years, range 42-80 years; untreated group: mean 59.0 ± 11.8 years, range 39-79 years)	Y (21 M/9 F)	Y (27 R/3 L)	Y (treated group: mean 58.3 ± 51.8 months, range 12-170 months; untreated group: mean 85.2 ± 141.9 months, range 10-467 months)
Kristinsson et al. (2019)	Y (typical BDNF genotype group mean 59.6 ± 11.2 years, range 29-77 years; atypical BDNF genotype group mean 57.7 ± 10.9 years, range 30-76 years)	Y (58 M/29 F)	Y (87 R/0 L)	Y (typical BDNF genotype group: mean 44.0 ± 38.7 months; atypical BDNF genotype group: mean 34.5 ± 36.9 months; all participants > 6 months)
Purcell et al. (2019)	Y (range 40-80 years)	Y (13 M/8 F)	Y (16 R/3 L)	Y (range 14-209 months)
Sreedharan, Chandran, et al. (2019)	N (range 18-68 years; controls were younger)	Y (7 M/1 F)	Y (8 R/0 L)	N (6-22 weeks; patients at different subacute stages of recovery)
Hartwigsen et al. (2020)	Y (mean 58.8 years, range 43-72 years)	Y (8 M/4 F)	Y (12 R/0 L)	Y (mean 37.9 ± 34.8 months, range 6-122 months)
Stockert et al. (2020)	Y (frontal group: mean 52.3 ± 18.9 years, range 15-78 years; temporo-parietal group: mean 54.4 ± 12.7 years, range 31-76 years)	Y (25 M/9 F)	N (31 R/2 L); not stated for controls	Y (frontal group: T1 acute: mean 3.2 ± 2.0 days, range 1-7 days; T2 subacute: mean 11.9 ± 2.2 days, range 8-17 days; T3 chronic: mean 272.6 ± 88.5 days, range 181-435 days; temporo-parietal group: T1 acute: mean 1.6 ± 0.8 days, range 1-4 days; T2 subacute: mean 10.1 ± 1.7 days, range 8-13 days; T3 chronic:

	mean 262.5 ± 75.0 days, range 184-394 days)

Age = Is age reported for patients and controls, and matched?; Sex = Is sex reported for patients and controls, and matched?; Handedness = Is handedness reported for patients and controls, and matched?; Time post onset = Is time post stroke onset reported and appropriate to the study design?; AAC = Augmentative and Alternative Communication; CIAT = constraint-induced aphasia therapy; F = female; L = left; M = male; MIT = melodic intonation therapy; N = No; POp+ = pars opercularis damaged; POp- = pars opercularis spared; R = right; rTMS = repetitive transcranial magnatic stimulation; SEM = standard error of the mean; T1, T2, etc. = first time point, second time point, etc.; TPO = time post onset; Y = Yes; Yellow underline = minor limitation; Orange underline/* = moderate limitation.

Supplementary Table S3. Participants: Characterization of aphasia

Study	Aphasia	Language evaluation	Aphasia severity	Aphasia type
Weiller et al. (1995)	Comprehensive battery	AAT	Recovered; not aphasic per formal testing	Recovered, but all had moderate-severe Wernicke's aphasia in the subacute period
Belin et al. (1996)	Severity and type	BDAE	Persistent severe non-fluent aphasia followed by marked improvement with MIT	5 global, 2 Broca's
Ohyama et al. (1996)	Comprehensive battery	WAB	AQ mean 74.3 ± 12.2, range 53.8-92.4	6 anomic, 4 atypical, 4 mild Broca's, 1 mild Wernicke's, 1 transcortical sensory; alternately: 10 fluent, 6 non-fluent
Heiss et al. (1997)	Severity only	Verbal repetition, confrontation naming, oral and written comprehension, reading abilities, TT, phonemic fluency, clinical impression, family interview	T1: TT range 37-48; T2: TT range 3-39 (1 missing)	T1: 5 global, 1 Wernicke's; T2: not stated
Karbe et al. (1998)	Severity and type	TT	T1: 9 severe; 2 mild; 1 not stated; TT range 3-47 errors; T2: not stated	T1: 8 global, 3 anomic, 1 Wernicke's; T2: not stated
Cao et al. (1999)	Severity and type	ADP	ADPASS percentile range 73-99	3 anomic, 1 conduction, 1 recovered, 1 transcortical sensory
Heiss et al. (1999)	Severity and type	AAT, phonemic fluency	T1: subcortical: TT median 8 errors, range 0-17 errors; frontal: TT median 21 errors, range 4-40 errors; temporal: TT median 39 errors, range 1-47 errors; T2: subcortical: TT median 1 error, range 0-14 errors; frontal: TT median 8 errors, range 0-34; temporal: TT median 34 errors, range 0-44 errors	T1: 6 Wernicke's, 5 Broca's, 5 residual aphasia, 4 anomic, 2 transcortical sensory, 1 conduction; T2: not stated
Kessler et al. (2000)	Severity only	AAT	T1: piracetam group: TT 17.16 ± 14.31 errors; placebo group: TT 17.91 ± 15.47 errors; T2: piracetam group: TT 9.66 ± 12.62 errors; placebo group: TT 12.50 ± 16.88 errors	Not stated
Rosen et al. (2000)	Severity and type	WAB (except BDAE in 1 patient), reading pseudowords, word stem completion, verb generation, reading single words	AQ range 74-97 (missing in 1 patient)	3 anomic, 1 Broca's, 1 not stated, 1 recovered
Blasi et al. (2002)	Comprehensive battery	WAB or BDAE	AQ range 66.5-89.0 in 6 participants, BDAE aphasia severity of 4 in 1 participant, no formal evaluation in 1 participant	3 anomic, 3 transcortical motor, 1 Broca's, 1 not stated; most were Broca's or global acutely
Leff et al. (2002)	Not at all	PPT (Dutch), British picture vocabulary scale, Action for Dysphasic Adults lexical decision battery, auditory maximal pairs (an offline phoneme discrimination test)	Not stated	Not stated, but all 6 patients with pSTS damage had single word comprehension deficits acutely
Blank et al. (2003)	Type only	CAT, QPA	Not stated	POp+: 4 non-fluent but not agrammatic, 2 agrammatic, 1 recovered; POp-: 4 non-fluent

				but not agrammatic, 3 recovered
Cardebat et al. (2003)	Not at all	Not stated	Not stated	T1: some prominent symptoms are listed for each patient; T2: not stated
Sharp et al. (2004)	Severity only	Subtests from CAT, subtests from PALPA, Action for dysphasic adults, TROG, PPT	Mild	Not stated
Zahn et al. (2004)	Comprehensive battery	AABT, AAT	TT percentile range 28-63	3 global, 2 Broca's, 2 unclassifiable; all had been global initially
Crinion & Price (2005)	Comprehensive battery	CAT	Not stated	Not stated
de Boissezon et al. (2005)	Type only	Montreal-Toulouse Aphasia Battery	Not stated	T1: 2 Broca's, 2 transcortical sensory, 1 anomic, 1 transcortical motor, 1 Wernicke's; T2: 4 recovered, 1 anomic, 1 transcortical motor; 1 transcortical sensory
Connor et al. (2006)	Comprehensive battery	WAB or BDAE	AQ range 66.5-89.0 in 6 participants, BDAE aphasia severity of 4 in 1 participant, no formal evaluation in 1 participant	3 anomic, 3 transcortical motor, 1 Broca's, 1 not stated; most were Broca's or global acutely
Crinion et al. (2006)	Comprehensive battery	CAT (missing in two participants)	Not stated	Not stated
Saur et al. (2006)	Comprehensive battery	AABT, AAT including TT, analysis of spontaneous speech, CETI, Language Recovery Score (LRS) derived from all these measures plus in-scanner task performance	T1: LRS mean 0.44, range 0.11-0.81; 1 mild, 1 mild-moderate, 7 moderate, 3 moderate-severe, 2 severe per AAT; T2: LRS mean 0.71, range 0.33-0.92; 2 recovered, 2 recovered-mild, 2 mild, 3 mild-moderate, 3 moderate, 2 severe per AAT; T3: LRS mean 0.91, range 0.66-1.00; 8 recovered, 2 recovered-mild, 3 mild, 1 moderate per AAT	T1: 9 non-fluent, 5 fluent; T2: not stated; T3: 6 recovered, 4 minimal language impairment, 3 anomic, 1 global
Meinzer et al. (2008)	Comprehensive battery	AAT, study-specific picture naming test with 150 items	6 moderate, 4 mild, 1 severe	7 Broca's, 2 Wernicke's, 1 global, 1 unclassified
Raboyeau et al. (2008)	Severity and type	Montreal-Toulouse Aphasia Battery	Mild (but had initially been severe)	4 anomic, 3 conduction, 2 Broca's, 1 AoS
Richter et al. (2008)	Comprehensive battery	AAT, two subtests of ANELT	TT range 5-50	7 anomic, 7 Broca's, 2 global; it was an inclusion criterion that the main deficits were in production
de Boissezon et al. (2009)	Comprehensive battery	Montreal-Toulouse Aphasia Battery	Not stated	T1: 3 transcortical motor, 2 anomic, 2 Broca's, 2 transcortical sensory, 2 Wernicke's, 1 conduction, 1 agrammatic; T2: not stated
Fridriksson et al. (2009)	Comprehensive battery	WAB; BNT	AQ range 31.8-91.5	6 anomic, 4 Broca's, 1 transcortical motor; alternatively: 6 fluent, 5 non-fluent
Menke et al. (2009)	Comprehensive battery	AAT	6 moderate-severe, 2 severe	7 Broca's, 1 global
Specht et al. (2009)	Comprehensive battery	AAT	Not stated	3 global, 3 Wernicke's, 2 amnestic, 2 Broca's, 2 unclassified
Warren et al. (2009)	Not at all	CAT, TROG	Not stated	Not stated

Chau et al. (2010)	Severity only	Cantonese Aphasia Battery (modified WAB)	5 patients had AQ > 75, 2 had AQ < 30	Not stated
Fridriksson (2010)	Severity and type	WAB	AQ mean 60.4 ± 25.6 (including excluded patients)	11 anomic, 10 Broca's, 3 conduction, 1 transcortical motor, 1 Wernicke's (including excluded patients)
Fridriksson et al. (2010)	Severity and type	WAB	AQ mean 77.1, range 47.1-93.7	10 anomic, 3 Broca's, 2 conduction
Sharp et al. (2010)	Severity only	Subtests from CAT, subtests from PALPA, Action for dysphasic adults, TROG, PPT	Mild	Not stated
Thompson et al. (2010)	Comprehensive battery	WAB, NAVS, narrative language sample	AQ range 66.8-85.0	All agrammatic; per WAB scores provided: 3 Broca's, 3 unclassified
Tyler et al. (2010)	Not at all	Sentence-picture matching, lexical decision, phonological similarity, word repetition, sentence repetition, morphological similarity, semantic categorization, sentence acceptability	Not stated	Not stated
van Oers et al. (2010)	Comprehensive battery	AAT, BNT, TT	4 moderate, 4 severe, 3 recovered, 2 mild; all had aphasia initially	5 anomic, 4 Broca's, 3 recovered, 1 Wernicke's
Papoutsi et al. (2011)	Not at all	Sentence-picture matching, grammaticality judgment, lexical decision, phonological discrimination, semantic categorization, sentence repetition, word repetition	Not stated	Not stated
Sebastian & Kiran (2011)	Comprehensive battery	WAB, BNT, portions of PALPA, PPT, CLQT	AQ range 74.0-97.8	6 anomic, 2 recovered
Szaflarski et al. (2011)	Severity and type	BNT; phonemic fluency, semantic fluency, complex ideation from BDAE, PPVT, communicative activities log	Moderate	4 Broca's, 3 anomic, 1 anomic/conduction
Tyler et al. (2011)	Not at all	Sentence-picture matching, grammaticality judgment, lexical decision, phonological discrimination, semantic categorization, sentence repetition, word repetition	Not stated	Not stated
Weiduschat et al. (2011)	Type only	AAT	T1: TT range 0-45 errors; T2: TT range 0-44 errors	T1: 5 Wernicke's, 2 Broca's, 2 global, 1 amnestic fluent; T2: not stated
Allendorfer et al. (2012)	Severity and type	TT, PPVT, BNT, semantic and phonemic fluency, complex ideation subtest of BDAE	Moderate-severe; TT mean 25.5 ± 11.3; unclear how to reconcile moderate-severe severity with mostly anomic aphasia	Mostly anomic with some non-fluent
Fridriksson, Hubbard, et al. (2012)	Comprehensive battery	WAB, BNT, AoS from ABA	AQ mean 48.5 ± 20.6, range 20.9-73.5	Broca's
Fridriksson, Richardson, et al. (2012)	Severity and type	WAB	AQ mean 57.9 ± 25.8, range 17.2-95.2	13 Broca's, 10 anomic, 3 conduction, 2 Wernicke's, 1 global, 1 transcortical motor
Marcotte et al. (2012)	Comprehensive battery	Montreal-Toulouse Aphasia Battery, picture naming	Moderate-severe	7 Broca's, 1 Broca's + AoS, 1 Wernicke's + AoS
Schofield et al. (2012)	Severity only	CAT	11 patients (plus one excluded) had moderate comprehension impairments, 9 had severe	Not stated

			comprehension impairments; this distribution was bimodal	
Wright et al. (2012)	Not at all	Sentence-picture matching	Not stated	Not stated
Szaflarski et al. (2013)	Severity only	TT, BNT, semantic fluency, phonemic fluency, PPVT, complex ideation subtest of BDAE	Recovered: TT mean 43 ± 1, ≥ 41; non-recovered: TT mean 23 ± 12, < 41	Not stated
Thiel et al. (2013)	Severity and type	AAT	T1: rTMS group: AAT sum of scores mean 251.5 ± 32.4; sham group: mean 251.1 ± 39.5; T2 not stated	T1: rTMS group: 7 Wernicke's, 3 amnestic, 2 global, 1 Broca's; sham group: 5 Wernicke's, 3 Broca's, 2 global, 1 amnestic; T2: not stated
Abel et al. (2014)	Type only	AAT	Not stated	8 Broca's, 3 Wernicke's, 1 fluent non-classifiable, 1 global, 1 transcortical sensory
Benjamin et al. (2014)	Severity and type	WAB, BNT, PPVT	Intention group: AQ mean 65.5 ± 8.3; control group: AQ mean 71.9 ± 11.9	Intention group: 4 conduction, 2 Broca's, 1 anomic; control group: 4 anomic, 1 Broca's, 1 conduction, 1 transcortical motor
Brownsett et al. (2014)	Not at all	Not stated	Not stated	Not stated, but all had auditory comprehension and repetition deficits, and all could at least attempt to repeat
Mattioli et al. (2014)	Comprehensive battery	AAT, TT	T1: TT range 2-45; T2: TT range 6-48; T3: TT range 21-48	T1: 8 Broca's, 3 anomic, 1 Wernicke's; T2: not stated
Mohr et al. (2014)	Severity only	BDAE, TT	Mild-moderate; T1: TT range 15-49 errors (including 2 excluded patients)	Not stated
Robson et al. (2014)	Comprehensive battery	BDAE, PPT, word-to-picture matching test from Cambridge Semantic Battery, single word reading aloud from PALPA	BDAE comprehension range 6-26 (out of 32); BDAE comprehension scores and percentiles do not seem entirely commensurate	All Wernicke's
Szaflarski et al. (2014)	Not at all	Not stated	"complete or almost complete" recovery in a "substantial proportion" of the patients	Not stated
van Hees et al. (2014)	Comprehensive battery	WAB, BNT, PPT, CAT, picture naming from International Picture Naming Project Database	AQ range 57.3-91.6; 5 mild, 2 moderate, 1 mild-moderate	6 anomic, 2 conduction
Abel et al. (2015)	Type only	AAT	Not stated	8 Broca's, 3 Wernicke's, 1 fluent non-classifiable, 1 global, 1 transcortical sensory
Kiran et al. (2015)	Severity only	WAB, BNT, PPT, CLQT	AQ range 48.0-97.2	Not stated
Sandberg et al. (2015)	Comprehensive battery	WAB, BNT, subtests from PALPA, PPT, CLQT	AQ range 41.7-99.2	6 anomic, 2 conduction, 1 Broca's, 1 transcortical motor
Geranmayeh et al. (2016)	Comprehensive battery	CAT, QPA	"relatively mild stroke"; 17 patients were so mild that they were not aphasic per the CAT	Not stated
Griffis et al. (2016)	Severity and type	BNT; phonemic fluency, semantic fluency, complex ideation from BDAE, PPVT, communicative activities log	Moderate	4 Broca's, 3 anomic, 1 anomic/conduction
Sims et al. (2016)	Severity and type	WAB, BNT, PPT, CLQT	AQ range 48.0-99.2	4 anomic, 2 Broca's, 2 conduction, 2 transcortical motor, 1 anomic or transcortical motor, 1 Broca's or conduction, 1 "N/A", 1 Wernicke's or conduction
Darkow et al. (2017)	Comprehensive battery	AAT	Mild	Not stated

Geranmayeh et al. (2017)	Not at all	CAT, QPA	Not stated	Not stated
Griffis, Nenert, Allendorfer, & Szaflarski (2017)	Not at all	BNT, semantic fluency, phonemic fluency	Not stated	Not stated
Griffis, Nenert, Allendorfer, Vannest, et al. (2017)	Not at all	BNT, semantic fluency, phonemic fluency	Not stated	Not stated
Harvey et al. (2017)	Comprehensive battery	BDAE, BNT	Mild-moderate	All non-fluent
Nardo et al. (2017)	Not at all	BNT, one CAT subtest, two PALPA subtests	Not stated	Not stated
Nenert et al. (2017)	Severity only	TT, PPVT, BNT, semantic fluency, phonemic fluency, communicative activities log	6 mild (2 control, 4 CIAT); 5 moderate (3 control, 2 CIAT); 8 severe (3 control, 5 CIAT)	Not stated
Qiu et al. (2017)	Severity and type	WAB	Moderate-severe	All Broca's
Skipper-Kallal et al. (2017a)	Comprehensive battery	WAB, PNT	AQ mean 77.7 ± 21.0, range 22.8-99.2	21 anomic, 7 Broca's, 3 conduction, 1 transcortical sensory
Skipper-Kallal et al. (2017b)	Comprehensive battery	WAB, PNT	Not stated	23 anomic, 11 Broca's, 3 conduction, 1 transcortical sensory, 1 Wernicke's
Dietz et al. (2018)	Severity and type	WAB, Reading Comprehension Battery for Aphasia	AAC group: AQ range 37.6-82.4; usual care group: AQ range 36.7-89.2	AAC group: 2 Broca's, 1 anomic, 1 conduction, 1 global, 1 Wernicke's; usual care group: 2 anomic, 2 Broca's, 1 conduction, 1 Wernicke's
Hallam et al. (2018)	Comprehensive battery	Cambridge semantic battery, three additional semantic tasks, connected speech words per minute, repetition from PALPA	Not stated	6 anomic, 2 Broca's, 2 global, 2 transcortical sensory, 1 mixed transcortical, 1 not stated
Nenert et al. (2018)	Not at all	PPVT, BNT, phonemic fluency, semantic fluency, complex ideation subtest of BDAE	Not stated for study timepoints, but on admission, aphasia severity was assessed with the TT: 2 no aphasia per cutoff but clinical impression of aphasia, 5 mild, 6 moderate, 4 severe	Not stated
Pillay et al. (2018)	Not at all	Pseudoword rhyme matching, semantic picture matching (similar to PPT-P), picture naming	Not stated	Not stated
Szaflarski et al. (2018)	Comprehensive battery	WAB, BNT, semantic fluency, phonemic fluency	AQ range 10.4-94.6	8 anomic, 2 Broca's, 1 conduction, 1 global
van de Sandt-Koenderman et al. (2018)	Comprehensive battery	AAT, ANELT	T1: subacute: ASRS median 1, range 0-2; ANELT range 10-29; chronic: ASRS median 1.5, range 1-2; ANELT range 20-29; T2: subacute: ASRS range 1-3; ANELT range 10-43; chronic: ASRS range 1-2; ANELT range 22-31	T1: all severe non-fluent; T2: not stated
van Oers et al. (2018)	Comprehensive battery	AAT, BNT	T1: 8 moderate, 2 severe, 2 not stated; T2: 4 moderate, 3 recovered, 2 not stated, 1 mild, 1 severe	T1: 6 Broca's, 3 anomic, 2 Wernicke's, 1 global; T2: 4 anomic, 3 recovered, 2 Broca's, 1 unclassified, 1 Wernicke's
Barbieri et al. (2019)	Comprehensive battery	WAB, Northwestern Assessment of Verbs and Sentences (NAVS), Northwestern Naming Battery (NNB), analysis of spontaneous speech (Cinderella story) using	AQ range 52.8-91.7	Not stated, except that "language deficits were consistent with nonfluent aphasia and agrammatism"

		Northwestern Narrative Language Analysis (NNLA) protocol		
Johnson et al. (2019)	Severity only	WAB, BNT, PPT	Treated group: AQ mean 60.1 ± 24.0, range 11.7-95.2; untreated group: AQ mean 65.8 ± 24.6, range 26.9-91.5	Not stated
Kristinsson et al. (2019)	Severity and type	WAB, PNT, PPT	Typical BDNF genotype group: AQ mean 64.2 ± 20.3; atypical BDNF genotype group: AQ mean 54.3 ± 21.0	Typical BDNF genotype group: 25 Broca's, 12 anomic, 11 conduction, 2 transcortical motor aphasia, 2 Wernicke's, 1 global; atypical BDNF genotype group: 16 Broca's, 6 anomic, 6 conduction, 3 global, 3 Wernicke's
Purcell et al. (2019)	Comprehensive battery	Spelling (PALPA 40 and 54, and other word lists), oral reading (PALPA 35), reading comprehension (PALPA 51), spoken word-picture matching and picture naming tests from Northwestern Naming Battery, PPT-P; note no generic aphasia battery, but fairly complete coverage of language domains	Spelling of untrained items range 51%-94%	4 orthographic working memory deficit, 8 orthographic long-term memory deficit, 9 both types of deficit
Sreedharan, Chandran, et al. (2019)	Severity only	WAB translated into Malayalam	AQ range approximately 50-80	Broca's or anomic
Hartwigsen et al. (2020)	Not at all	AAT	7 mild residual aphasia, 5 recovered	Not stated
Stockert et al. (2020)	Severity only	AAT including TT, comprehension composite (LRScomp) and production composite (LRSprod) were derived	Frontal group: T1 acute: LRScomp mean 0.48 ± 0.26; T2 subacute: LRScomp mean 0.64 ± 0.21; T3 chronic: LRScomp mean 0.91 ± 0.07; temporo-parietal group: T1 acute: LRScomp mean 0.63 ± 0.32; T2 subacute: LRScomp mean 0.79 ± 0.20; T3 chronic: LRScomp mean 0.91 ± 0.13	Not stated

Aphasia [column] = To what extent is the nature of aphasia characterized?; AABT = Aachen Aphasia Bedside Test; AAT = Aachen Aphasia Test; ABA = Apraxia Battery for Adults; ADP = Aphasia Diagnostic Profiles; ADPASS = Aphasia Diagnostic Profiles Aphasia Severity Score; ANELT = Amsterdam-Nijmegen Everyday Language Test; AoS = apraxia of speech; AQ = aphasia quotient; ASRS = Aphasia Severity Rating Scale; BDAE = Boston Diagnostic Aphasia Examination; BNT = Boston Naming Test; CAT = Comprehensive Aphasia Test; CETI = Communicative Effectiveness Index; CIAT = constraint-induced aphasia therapy; CLQT = Cognitive Linguistic Quick Test; LRS = Language Recovery Score; MIT = melodic intonation therapy; NAVS = Northwestern Assessment of Verbs and Sentences; PALPA = Psycholinguistic Assessments of Language Processing in Aphasia; PNT = Philadelphia Naming Test; POp+ = pars opercularis damaged; POp- = pars opercularis spared; PPT = Pyramids and Palm Trees; PPVT = Peabody Picture Vocabulary Test; pSTS = posterior superior temporal sulcus; QPA = Quantitative Production Analysis; rTMS = repetitive transcranial magnetic stimulation; T1, T2, etc. = first time point, second time point, etc.; TROG = Test for Reception of Grammar; TT = Token Test; WAB = Western Aphasia Battery; Yellow underline = minor limitation; Orange underline = moderate limitation.

Supplementary Table S4. Participants: Characterization of neurological status

Study	First stroke	Stroke type	Lesion	Lesion extent	Lesion location
Weiller et al. (1995)	Yes	Ischemic only	Individual lesions	Not stated	Posterior L MCA infarct, lesion to the L posterior STG usually extending to MTG and AG
Belin et al. (1996)	Not stated	Not stated	Individual lesions	Not stated, but note that hypoperfusion greatly exceeded the infarct in all but 1 patient	L MCA; 2 also had ACA
Ohyama et al. (1996)	Yes	Ischemic only	Extent and location	Mean 33.9 ± 26.3 cc, range 8.1-113.2 cc	L perisylvian
Heiss et al. (1997)	Yes	Ischemic only	Individual lesions	Range 27.2-133.2 cc	L MCA; 5 patients had superior temporal damage and 1 had subcortical damage underlying posterior superior temporal cortex
Karbe et al. (1998)	Yes	Ischemic only	Extent and location	Range 2-133 cc	L MCA
Cao et al. (1999)	Yes	Ischemic only	Individual lesions	Extents are reported in three dimensions	4 L MCA, 2 L ICA
Heiss et al. (1999)	Yes	Ischemic only	Extent and location	Range 4.3-154.3 cc (probably; units not stated)	L MCA; 9 subcortical, 7 frontal, 7 temporal
Kessler et al. (2000)	Yes	Ischemic only	Location only	Not stated	10 L frontal, 6 L subcortical, 8 L temporal
Rosen et al. (2000)	Yes	Not stated	Individual lesions	Range 10.7-117.5 cc	L IFG, extending to neighboring areas in most cases
Blasi et al. (2002)	Yes	Ischemic only	Individual lesions	Not stated	L IFG and operculum, extending to adjacent cortex and white matter in several cases
Leff et al. (2002)	Yes	Not stated	Extent and location	Range 0.5-14% of total brain volume	9 L but sparing pSTS, 6 L including pSTS
Blank et al. (2003)	No	Not stated	Individual lesions	Not stated	L frontal, occasionally extending into temporal
Cardebat et al. (2003)	Yes	Mixed etiologies	Individual lesions	Not stated	4 L subcortical, 2 L prerolandic, 2 L postrolandic
Sharp et al. (2004)	Yes	Not stated	Lesion overlay	Not stated	Lesion in vicinity of L STG; no extensive frontal damage; no inferior temporal damage
Zahn et al. (2004)	Yes	Not stated	Lesion overlay	Not stated	L MCA
Crinion & Price (2005)	Yes	Not stated	Lesion overlay	Not stated	L MCA
de Boissezon et al. (2005)	Yes	Mixed etiologies	Individual lesions	Not stated	5 L non-thalamic subcortical, 2 L thalamic
Connor et al. (2006)	Yes	Ischemic only	Individual lesions	Not stated	L IFG and operculum, extending to adjacent cortex and white matter in several cases
Crinion et al. (2006)	Yes	Not stated	Lesion overlay	Not stated	6 L but no temporal damage, 9 L temporal damage excluding anterior temporal cortex, 9 L temporal damage including anterior temporal cortex
Saur et al. (2006)	Yes	Ischemic only	Individual lesions	Not stated	L MCA; 4 frontal (2 extending to temporoparietal); 5 temporoparietal (2 extending to subcortical); 4 striatocapsular (2 extending to cortical); 1 frontoparietal
Meinzer et al. (2008)	Not stated	Mixed etiologies	Lesion overlay	Range 31.0-236.0 cc	L

Raboyeau et al. (2008)	Yes	Not stated	Individual lesions	Range 29.9-195.2 cc	L MCA
Richter et al. (2008)	Not stated	Not stated	Individual lesions	Not stated	L
de Boissezon et al. (2009)	Yes	Mixed etiologies	Lesion overlay	Range 0.9-43.4 cc	L MCA (7 subcortical, 6 cortical)
Fridriksson et al. (2009)	Not stated	Not stated	Lesion overlay	Range 3.0-342.2 cc	L MCA
Menke et al. (2009)	Yes	Mixed etiologies	Individual lesions	Not stated	L
Specht et al. (2009)	Not stated	Not stated	Lesion overlay	Not stated	L MCA, with greatest overlap in the posterior STG
Warren et al. (2009)	Yes	Ischemic only	Lesion overlay	Patients with positive anterior temporal interconnectivity: mean 93.3 ± 24.0 cc; patients with negative anterior temporal interconnectivity: mean 96.1 ± 27.6 cc	L not including anterolateral superior temporal cortex; maximal overlap in posterior superior temporal cortex
Chau et al. (2010)	Yes	Ischemic only	Location only	Not stated	3 L MCA, 2 L frontal, 2 L basal ganglia
Fridriksson (2010)	Yes	Ischemic only	Lesion overlay	Not stated	L MCA
Fridriksson et al. (2010)	Yes	Ischemic only	Lesion overlay	Not stated	L MCA
Sharp et al. (2010)	Yes	Not stated	Lesion overlay	Not stated	Lesion in vicinity of L STG; no extensive frontal damage; no inferior temporal damage
Thompson et al. (2010)	Yes	Not stated	Individual lesions	Not stated	5 L MCA, 1 R MCA with aphasia
Tyler et al. (2010)	Not stated	Mixed etiologies	Lesion overlay	Not stated	L
van Oers et al. (2010)	Yes	Ischemic only	Individual lesions	Range 6.0-167.3 cc	L MCA
Papoutsi et al. (2011)	Not stated	Not stated	Lesion overlay	Not stated	L MCA
Sebastian & Kiran (2011)	Not stated	Mixed etiologies	Individual lesions	Range 23-45 cc	L MCA
Szaflarski et al. (2011)	Not stated	Not stated	Individual lesions	Not stated	L MCA
Tyler et al. (2011)	Not stated	Not stated	Lesion overlay	Not stated	L MCA
Weiduschat et al. (2011)	Yes	Not stated	Extent and location	Range 0.7-88.9 cc	L MCA
Allendorfer et al. (2012)	Not stated	Ischemic only	Individual lesions	Range 2.8-248.9 cc	L MCA
Fridriksson, Hubbard, et al. (2012)	Yes	Not stated	Lesion overlay	Not stated	L MCA
Fridriksson, Richardson, et al. (2012)	Yes	Mixed etiologies	Lesion overlay	Range 7.7-420.5 cc	L MCA
Marcotte et al. (2012)	Yes	Not stated	Lesion overlay	Range 14.6-295.8 cc	L MCA
Schofield et al. (2012)	Yes	Ischemic only	Lesion overlay	Range 24.2-403.6 cc	L MCA
Wright et al. (2012)	Yes	Not stated	Lesion overlay	Not stated	L MCA
Szaflarski et al.	Not stated	Not stated	Lesion	Recovered: median 9.2 cc, range	L MCA

(2013)			overlay	2.2-26.5 cc; non-recovered: median 74 cc, range 5.1-206.0 cc	
Thiel et al. (2013)	Yes	Ischemic only	Individual lesions	RTMS group: 233 ± 197 cc; sham group: 244 ± 243 cc; lesion extent in images appears much smaller than the stated volumes	L MCA
Abel et al. (2014)	Yes	Mixed etiologies	Lesion overlay	Not stated	L MCA; 2 also had ACA
Benjamin et al. (2014)	No	Mixed etiologies	Lesion overlay	Not stated	L MCA, extending frontally at least into the precentral gyrus or underlying white matter
Brownsett et al. (2014)	Not stated	Not stated	Lesion overlay	Not stated	L temporal and parietal cortex; 4 extended into the frontal lobe; no lesions involved ACA territory
Mattioli et al. (2014)	Yes	Not stated	Individual lesions	Range 4.4-158.3 cc (possibly; units stated do not seem correct)	L MCA; lesions seem very small in Supplementary Figure 1, but are described as more extensive in Supplementary Table 1
Mohr et al. (2014)	Yes	Mixed etiologies	Lesion overlay	Not stated	L MCA
Robson et al. (2014)	Yes	Mixed etiologies	Lesion overlay	Not stated	L MCA; all involved STG extending into IPL and temporoparietal junction; 8 extending into MTL; 4 extending into inferior frontal
Szaflarski et al. (2014)	Not stated	Not stated	Lesion overlay	60.1 ± 57.5 cc	L MCA
van Hees et al. (2014)	Yes	Not stated	Lesion overlay	Not stated	L hemisphere
Abel et al. (2015)	Yes	Mixed etiologies	Lesion overlay	Not stated	L MCA; 2 also had ACA
Kiran et al. (2015)	Yes	Not stated	Lesion overlay	24.2-431.6 cc	L MCA except for one patient with R MCA and aphasia
Sandberg et al. (2015)	Not stated	Not stated	Lesion overlay	Range 0.3-256.0 cc	L MCA
Geranmayeh et al. (2016)	No	Not stated	Lesion overlay	Mean 25.4 ± 13.5 cc, range 0.3-168.0 cc	L; modest R involvement in 7 cases
Griffis et al. (2016)	Not stated	Not stated	Individual lesions	Range 1.4-52.5 cc	L MCA
Sims et al. (2016)	Not stated	Not stated	Lesion overlay	Not stated	L MCA
Darkow et al. (2017)	Not stated	Not stated	Lesion overlay	Range 9.7-165.1 cc	L MCA not including hand motor area
Geranmayeh et al. (2017)	No	Not stated	Lesion overlay	Mean 41.4 ± 44.4 cc, range 3.8-173.9 cc	L; modest R involvement in 3 cases
Griffis, Nenert, Allendorfer, & Szaflarski (2017)	Yes	Not stated	Lesion overlay	Mean 105.2 ± 76.3 cc	L
Griffis, Nenert, Allendorfer, Vannest, et al. (2017)	Yes	Not stated	Individual lesions	Mean 105.2 ± 76.3 cc	L
Harvey et al. (2017)	Yes	Ischemic only	Individual lesions	Range 36.6-252.1 cc	L MCA
Nardo et al. (2017)	Yes	Not stated	Lesion overlay	Not stated	L MCA
Nenert et al. (2017)	Yes	Ischemic only	Lesion overlay	Not stated	L MCA
Qiu et al. (2017)	Yes	Mixed etiologies	Not at all	Not stated	L
Skipper-Kallal et	Not stated	Not stated	Lesion	Mean 27.5 ± 22.9 cc	L MCA

al. (2017a)			overlay		
Skipper-Kallal et al. (2017b)	Not stated	Not stated	Lesion overlay	Not stated	L MCA
Dietz et al. (2018)	Yes	Ischemic only	Individual lesions	AAC group: range 7849-30570 voxels; usual care group: 1583-30110 voxels (voxel size not stated)	L MCA
Hallam et al. (2018)	Not stated	Not stated	Lesion overlay	Not stated	L IFG plus other MCA regions; vATL and pMTG spared
Nenert et al. (2018)	No	Ischemic only	Lesion overlay	Not stated	L MCA; mostly posterior per Supplementary Figure 2
Pillay et al. (2018)	Not stated	Ischemic only	Lesion overlay	Mean 73.4 ± 58.6 cc, range 6.7-227.0 cc	17 L MCA, 2 combined L MCA/ACA, combined 2 L MCA/PCA
Szaflarski et al. (2018)	Yes	Not stated	Individual lesions	Not stated	L MCA
van de Sandt-Koenderman et al. (2018)	Not stated	Not stated	Extent and location	Subacute: range 32.4-141.2 cc (no lesion extent was reported for one subacute participant because there was no tissue loss yet); chronic: range 27.4-87.9 cc	8 L MCA, 1 L SMA and R insular-temporoparietal
van Oers et al. (2018)	Yes	Ischemic only	Lesion overlay	Range 9-208 cc	L MCA
Barbieri et al. (2019)	Yes	Mixed etiologies	Lesion overlay	Not stated	Mostly L MCA but some lesions include PCA or ACA territory
Johnson et al. (2019)	Not stated	Not stated	Lesion overlay	Treated group: 136.6 ± 81.1 cc, range 11.7-317.1 cc; untreated group: 112.7 ± 94.6 cc, range 1.6-317.1 cc	Mostly MCA with a few extending into PCA
Kristinsson et al. (2019)	No	Mixed etiologies	Lesion overlay	Typical BDNF genotype group: 121.4 ± 73.2 cc; atypical BDNF genotype group: 142.2 ± 88.4 cc	L MCA
Purcell et al. (2019)	Yes	Not stated	Lesion overlay	Range 7.7-215.0 cc	L MCA with L ventral occipitotemporal cortex mostly intact
Sreedharan, Chandran, et al. (2019)	Not stated	Not stated	Individual lesions	Not stated	7 L MCA, 1 bilateral MCA
Hartwigsen et al. (2020)	Yes	Ischemic only	Lesion overlay	Range 11.9-176.3 cc	Left temporo-parietal cortex; maximal overlap in SMG
Stockert et al. (2020)	Yes	Ischemic only	Lesion overlay	Frontal group: mean 69.3 ± 34.0 cc, range 12.3-76.6 cc; temporo-parietal group: mean 54.8 ± 41.1 cc, range 6.2-108.5 cc	L MCA, frontal (n = 17) or temporo-parietal (n = 17)

First stroke = First stroke only?; Lesion [column] = To what extent is the lesion distribution characterized?; AAC = Augmentative and Alternative Communication; ACA = anterior cerebral artery; AG = angular gyrus; cc = cubic centimeters; ICA = internal carotid artery; IFG = inferior frontal gyrus; IPL = inferior parietal lobule; L = left; MCA = middle cerebral artery; MTG = middle temporal gyrus; MTL = medial temporal lobe; PCA = posterior cerebral artery; pMTG = posterior middle temporal gyrus; pSTS = posterior superior temporal sulcus; R = right; rTMS = repetitive transcranial magnetic stimulation; SMA = supplementary motor area; STG = superior temporal gyrus; vATL = ventral anterior temporal lobe; Yellow underline = minor limitation; Orange underline = moderate limitation.

Supplementary Table S5. Imaging: Design

Study	Modality	Study timing	Time points	Intervention
Weiller et al. (1995)	PET (rCBF)	Cross-sectional	—	—
Belin et al. (1996)	PET (rCBF)	Cross-sectional	—	—
Ohyama et al. (1996)	PET (rCBF)	Cross-sectional	—	—
Heiss et al. (1997)	PET (rCMRgl)	Longitudinal—recovery	T1: ~4 weeks; T2: ~12-18 months	Not stated
Karbe et al. (1998)	PET (rCMRgl)	Longitudinal—recovery	T1: mean 24 ± 11 days, ~3-4 weeks; T2: mean 19 ± 2 months, > 1 year	Not stated
Cao et al. (1999)	fMRI	Cross-sectional	—	—
Heiss et al. (1999)	PET (rCBF)	Longitudinal—recovery	T1: ~2 weeks; T2: ~8 weeks	Not stated
Kessler et al. (2000)	PET (rCBF)	Longitudinal—mixed	T1: pre-treatment, ~2 weeks post onset; T2: post-treatment, ~8 weeks post onset	SLT, 1 hour/day, 5 days/week, 6 weeks; 12 patients received piracetam and 12 received placebo; note that the two groups are not directly compared in any imaging or behavioral analyses
Rosen et al. (2000)	PET and fMRI	Cross-sectional	—	—
Blasi et al. (2002)	fMRI	Cross-sectional	—	—
Leff et al. (2002)	PET (rCBF)	Cross-sectional	—	—
Blank et al. (2003)	PET (rCBF)	Cross-sectional	—	—
Cardebat et al. (2003)	PET (rCBF)	Longitudinal—recovery	T1: 58 ± 35 days, range 11-113 days; T2: 11.7 ± 1.6 months, range 320-460 days; T1 varies considerably from early to late subacute	Not stated
Sharp et al. (2004)	PET (rCBF)	Cross-sectional	—	—
Zahn et al. (2004)	fMRI	Cross-sectional	—	—
Crinion & Price (2005)	fMRI	Cross-sectional	—	—
de Boissezon et al. (2005)	PET (rCBF)	Longitudinal—recovery	T1: mean 53 ± 35 days, range 11-108 days; T2: mean 12.2 ± 1.4 months; T1 varies considerably from early to late subacute	Not stated
Connor et al. (2006)	fMRI	Cross-sectional	—	—
Crinion et al. (2006)	PET (rCBF)	Cross-sectional	—	—
Saur et al. (2006)	fMRI	Longitudinal—recovery	T1 acute: mean 1.8 days, range 0-4 days; T2 subacute: mean 12.1 days, range 3-16 days; T3 chronic: mean 321 days, range 102-513 days	Standard SLT throughout the observation period including at least 3 weeks inpatient
Meinzer et al. (2008)	fMRI	Longitudinal—chronic treatment	T1: pre-treatment/chronic; T2: post-treatment, ~2 weeks later	CIAT, 3 hours/day, 5 days/week, 2 weeks
Raboyeau et al. (2008)	PET (rCBF)	Longitudinal—chronic treatment	T1: pre-treatment/chronic; T2: post-treatment, ~4 weeks later	Lexical training, 15 minutes/day, 5 days/week, 4 weeks; the control group were trained to relearn foreign words that they had learned in school but since mostly forgotten
Richter et al. (2008)	fMRI	Longitudinal—chronic treatment	T1: pre-treatment/chronic; T2: post-treatment, ~2 weeks later	CIAT, 3 hours/day, 10 days
de Boissezon et al. (2009)	PET (rCBF)	Longitudinal—recovery	T1: mean 64 ± 32 days; T2: mean 11.8 ± 1.4 months; T1 varies considerably from early to late subacute	Community SLT; 45 minutes/day, 1-3 days/week
Fridriksson et al. (2009)	fMRI	Cross-sectional	—	—
Menke et al. (2009)	fMRI	Longitudinal—chronic treatment	T1: pre-treatment/chronic; T2: post-treatment, ~2 weeks later; T3: 8 months after the end of treatment	Intensive anomia training; 3 hours/day; 2 weeks
Specht et al. (2009)	PET (rCBF)	Cross-sectional	—	—

Warren et al. (2009)	PET (rCBF)	Cross-sectional	—	—
Chau et al. (2010)	fMRI	Longitudinal—chronic treatment	T1: pre-treatment/chronic; T2: post-treatment, ~10 weeks later	Acupuncture, 3 sessions/week, 8 weeks
Fridriksson (2010)	fMRI	Longitudinal—chronic treatment	T1: pre-treatment/chronic; T2: post-treatment/~4 weeks later; note that there were two separate sessions per time point, as well as another two sessions midway through treatment that are not analyzed in this paper	Anomia treatment using a cueing hierarchy, 3 hours/day, 5 days/week, 2 weeks, with a 1-week gap between the two weeks
Fridriksson et al. (2010)	fMRI	Cross-sectional	—	—
Sharp et al. (2010)	PET (rCBF)	Cross-sectional	—	—
Thompson et al. (2010)	fMRI	Longitudinal—chronic treatment	T1: pre-treatment/chronic; T2: post-treatment, 9-15 weeks later	Treatment of underlying forms
Tyler et al. (2010)	fMRI	Cross-sectional	—	—
van Oers et al. (2010)	fMRI	Cross-sectional	Behavioral data (TT and a naming measure) were also acquired subacutely (mean 26 ± 18 days, range 5-56 days)	—
Papoutsi et al. (2011)	fMRI	Cross-sectional	—	—
Sebastian & Kiran (2011)	fMRI	Cross-sectional	—	—
Szaflarski et al. (2011)	fMRI	Longitudinal—chronic treatment	T1: pre-treatment/chronic; T2: post-treatment, ~2 weeks later	RTMS to residual activation near Broca's area, 5 sessions/week, 2 weeks
Tyler et al. (2011)	fMRI	Cross-sectional	—	—
Weiduschat et al. (2011)	PET (rCBF)	Longitudinal—mixed	T1: pre-treatment/subacute (range 18-97 days post onset); T2: post-treatment, ~2 weeks later	Individualized SLT, 45 minutes/day, 5 days/week, 2 weeks; 6 patients underwent rTMS to the R IFG pars triangularis; 4 received vertex (sham) rTMS
Allendorfer et al. (2012)	fMRI	Cross-sectional	—	—
Fridriksson, Hubbard, et al. (2012)	fMRI	Cross-sectional	—	—
Fridriksson, Richardson, et al. (2012)	fMRI	Longitudinal—chronic treatment	T1: pre-treatment/chronic; T2: post-treatment/~4 weeks later; note that there were two separate sessions per time point, as well as another two sessions midway through treatment that are not analyzed in this paper	Anomia treatment using a cueing hierarchy, 3 hours/day, 5 days/week, 2 weeks, with a 1-week gap between the two weeks
Marcotte et al. (2012)	fMRI	Longitudinal—chronic treatment	T1: pre-treatment/chronic; T2: post-treatment, 3-6 weeks later (after 80% performance on trained items, or 6 weeks)	Semantic feature analysis, 1 hour/day, 3 days/week, 3-6 weeks
Schofield et al. (2012)	fMRI	Cross-sectional	—	—
Wright et al. (2012)	fMRI	Cross-sectional	—	—
Szaflarski et al. (2013)	fMRI	Cross-sectional	—	—
Thiel et al. (2013)	PET (rCBF)	Longitudinal—mixed	T1: pre-treatment/subacute (rTMS group: mean 37.5 ± 18.5 days post onset; sham group: mean 50.6 ± 22.6 days post onset); T2 post-treatment, ~2.5 weeks later	RTMS group: inhibitory rTMS over the R IFG pars triangularis + SLT for 45 minutes/day, 5 days/week, 2 weeks; control group: sham TMS + SLT
Abel et al. (2014)	fMRI	Longitudinal—chronic treatment	T1: pre-treatment/chronic; T2: post-treatment, ~6 weeks later (labeled T2 and T3 in paper)	Lexical therapy, alternating between weeks with phonological and semantic treatment, 4 weeks; 60 out of the 132 items were trained
Benjamin et al. (2014)	fMRI	Longitudinal—chronic treatment	T1: pre-treatment/chronic; T2: post-treatment; T3: 3 months after the end of treatment	Word finding therapy for both groups, but the intention group had to produce complex left hand movements, while the control group did not; note that groups were not directly compared in any imaging analyses

Brownsett et al. (2014)	fMRI	Longitudinal—chronic treatment	Patients: T1: acclimatization/chronic (but used in some analyses); T2: pre-treatment/chronic (not stated how long after T1); T3: post-treatment/~4 weeks later; controls: T1: pre-training; T2: post-training/~2 weeks later	Patients: home-based therapy consisting of auditory discrimination and repetition tasks for 3 or 4 weeks between T2 and T3; control: 2 weeks of similar training using noise vocoded speech
Mattioli et al. (2014)	fMRI	Longitudinal—mixed	T1: pre-treatment, mean 2.2 ± 1.3 days post onset; T2: post-treatment, mean 16.2 ± 1.3 days post onset; T3: mean 190 ± 25.5 days post onset	6 patients were randomized to receive treatment focusing on verbal comprehension and lexical retrieval for 1 hour/day, 5 days/week between T1 and T2; no patient received treatment after T2
Mohr et al. (2014)	fMRI	Longitudinal—chronic treatment	T1: pre-treatment/chronic; T2: post-treatment, ~2 weeks later	CIAT, 3-4 hours/day, 5 days/week, 2 weeks
Robson et al. (2014)	fMRI	Cross-sectional	—	—
Szaflarski et al. (2014)	fMRI	Cross-sectional	—	—
van Hees et al. (2014)	fMRI	Longitudinal—chronic treatment	T1: pre-treatment/chronic; T2: post-treatment, 5-6 weeks later; note that "immediate improvement" was measured at the end of SLT, a week or two prior to T2 scan	SLT with alternating semantic and phonological sessions, 3 days/week, 4 weeks
Abel et al. (2015)	fMRI	Longitudinal—chronic treatment	T1: pre-treatment/chronic; T2: post-treatment, ~6 weeks later (labeled T2 and T3 in paper)	Lexical therapy, alternating between weeks with phonological and semantic treatment, 4 weeks; 60 out of the 132 items were trained
Kiran et al. (2015)	fMRI	Longitudinal—chronic treatment	T1: pre-treatment/chronic; T2: post-treatment, ~10 weeks later	Semantic feature-based treatment, 10 weeks
Sandberg et al. (2015)	fMRI	Longitudinal—chronic treatment	T1: pre-treatment/chronic; T2: post-treatment, up to 10 weeks later	Semantic feature-based treatment, 2 hours/day, 2 days/week, up to 10 weeks (depending on when criterion reached)
Geranmayeh et al. (2016)	fMRI	Cross-sectional	—	—
Griffis et al. (2016)	fMRI	Longitudinal—chronic treatment	T1: pre-treatment/chronic; T2: post-treatment, ~2 weeks later	RTMS to residual activation near Broca's area, 5 sessions/week, 2 weeks
Sims et al. (2016)	fMRI	Cross-sectional	—	—
Darkow et al. (2017)	fMRI	Longitudinal—chronic treatment	T1/T2: chronic; tDCS and sham sessions in randomized order	—
Geranmayeh et al. (2017)	fMRI	Longitudinal—recovery	T1: 15 ± 7.6 days (range 5-35 days); T2: 108 ± 26 days (range 87-200 days)	Variable modest amounts of SLT (range 0-18 hours) reported in Supplementary Table 1
Griffis, Nenert, Allendorfer, & Szaflarski (2017)	fMRI	Cross-sectional	—	—
Griffis, Nenert, Allendorfer, Vannest, et al. (2017)	fMRI	Cross-sectional	—	—
Harvey et al. (2017)	fMRI	Longitudinal—chronic treatment	T1: pre-treatment/chronic; T2: post-treatment, 2 months after treatment; T3: 6 months after treatment (the 2-month time point was not included in analysis because there was no significant behavioral effect at that time)	Inhibitory rTMS to R IFG, 10 days
Nardo et al. (2017)	fMRI	Longitudinal—chronic treatment	T1: pre-treatment/chronic; T2: post-treatment, ~6 weeks later	Anomia treatment (computer-based practice), 2+ hours/day, 6 weeks
Nenert et al. (2017)	fMRI	Longitudinal—chronic treatment	T1: pre-treatment/chronic; T2: post-treatment, ~3 weeks later; T3: 3 months after the end of treatment	CIAT, 4 hours/day, 5 days/week, 2 weeks
Qiu et al. (2017)	fMRI	Cross-sectional	—	—
Skipper-Kallal et al.	fMRI	Cross-sectional	—	—

(2017a)				
Skipper-Kallal et al. (2017b)	fMRI	Cross-sectional	—	—
Dietz et al. (2018)	fMRI	Longitudinal—chronic treatment	T1: pre-treatment/chronic; T2: post-treatment, ~4 weeks later	AAC group: treatment aimed at teaching participants how to utilize AAC to facilitate discourse; usual care group: traditional SLT, not focused on discourse or AAC specifically
Hallam et al. (2018)	fMRI	Cross-sectional	—	—
Nenert et al. (2018)	fMRI	Longitudinal—recovery	T1: ~2 weeks; T2: ~6 weeks; T3: ~12 weeks; T4: ~26 weeks; T5: ~52 weeks	Not stated
Pillay et al. (2018)	fMRI	Cross-sectional	—	—
Szaflarski et al. (2018)	fMRI	Longitudinal—chronic treatment	T1: pre-treatment/chronic (1-2 weeks prior to treatment); T2: post-treatment (within 1 week after end of 2-week treatment); T3: 13-20 weeks after end of treatment	Modified CIAT + intermittent theta burst stimulation to residual left hemispheric language activation, 45 minutes/session, 5 days/week, 2 weeks
van de Sandt-Koenderman et al. (2018)	fMRI	Longitudinal—mixed	T1: pre treatment/subacute or chronic; T2: post-treatment, ~6 weeks later	MIT, 5+ hours/week
van Oers et al. (2018)	fMRI	Longitudinal—recovery	T1: within 2 weeks; T2: ~3 months; T3: ~6 months; T4: ~12 months; specific timing of first time point not stated	Not stated
Barbieri et al. (2019)	fMRI	Longitudinal—chronic treatment	T1: pre-treatment/chronic; T2: post-treatment, ~12 weeks later	13 patients were treated and 5 were not; treatment of underlying forms; 90 minutes/session, 2 sessions/week until 80% accuracy met on weekly probe task, then 1 session/week, 12 weeks except for one patient who demonstrated rapid improvement and completed treatment in 6 weeks
Johnson et al. (2019)	fMRI	Longitudinal—chronic treatment	T1: pre-treatment/chronic; T2: post-treatment, ~12 weeks later	Semantic naming treatment, 2 sessions/week
Kristinsson et al. (2019)	fMRI	Cross-sectional	—	—
Purcell et al. (2019)	fMRI	Longitudinal—chronic treatment	T1: pre-treatment/chronic; T2: post-treatment, 6-24 weeks later	Spelling treatment, 60-80 minutes/day, 2 days/week, range 6-24 weeks
Sreedharan, Chandran, et al. (2019)	fMRI	Longitudinal—mixed	Neurofeedback group: T1: pre-treatment/subacute; T2: 1-5 weeks later; T3: 2-6 weeks after T1; T4: 3-11 weeks after T1; T5: 4-12 weeks after T1; T6: 5-12 weeks after T1; no training group: T1: subacute; T2: 2-12 weeks later; controls: T1: start of study; T2: 1-4 weeks later; T3: 3-5 weeks after T1; T4: 4-8 weeks after T1; T5: 7-37 weeks after T1; T6: 12-43 weeks after T1	4 patients received 4 additional sessions involving neurofeedback training, while 4 patients received treatment as usual
Hartwigsen et al. (2020)	fMRI	Longitudinal—chronic treatment	T1/T2/T3: chronic; sessions consisted of cTBS over left anterior IFG, cTBS over left posterior IFG, or sham; sessions at least 7 days apart in randomized order	CTBS
Stockert et al. (2020)	fMRI	Longitudinal—recovery	T1 acute: 1-7 days; T2 subacute: 8-21 days; T3 chronic: > 6 months	Not stated

Study timing = Is the study cross-sectional or longitudinal?; Time points = If longitudinal, at what time point(s) were imaging data acquired?; Intervention = If longitudinal, was there any intervention between the time points?; AAC = Augmentative and Alternative Communication; CIAT = constraint-induced aphasia therapy; fMRI = functional magnetic resonance imaging; IFG = inferior frontal gyrus; MIT = melodic intonation therapy; PET = positron emission tomography; R = right; rCBF = regional cerebral blood flow; rCMRgl = regional cerebral metabolic rate for glucose; rTMS = repetitive transcranial magnetic stimulation; SLT = speech-language therapy; T1, T2, etc. = first time point, second time point, etc.; tDCS = transcranial direct current stimulation; TT = Token Test; Yellow underline = minor limitation.

Supplementary Table S6. Imaging: Methodology part 1

Study	Scanner	Timing	Design type	Total images
Weiller et al. (1995)	Y (CTI ECAT 953/15)	Y	PET	6
Belin et al. (1996)	Y (CEA LETI-TTV03)	Y	PET	4
Ohyama et al. (1996)	Y (Headtome IV tomograph)	Y	PET	6
Heiss et al. (1997)	Y (Siemens ECAT EXACT HR)	Y	PET	2
Karbe et al. (1998)	Y (CTI-Siemens ECAT EXACT HR)	N* (activation and control images not acquired on the same day; number of acquisitions not clearly described)	PET	8
Cao et al. (1999)	Y (Magnex Scientific 3 Tesla)	Y	Block	40
Heiss et al. (1999)	Y (CTI-Siemens ECAT EXACT HR)	Y	PET	8
Kessler et al. (2000)	Y (CTI-Siemens ECAT EXACT HR)	Y	PET	8
Rosen et al. (2000)	Y (Siemens 961 EXACT HR; Siemens Vision 1.5 Tesla)	N (fMRI timing description is inconsistent)	Mixed	PET: 10; fMRI: 384-768
Blasi et al. (2002)	Y (Siemens Vision 1.5 Tesla)	Y	Event-related	1024
Leff et al. (2002)	Y (CTI-Siemens ECAT EXACT HR++/966)	Y	PET	16
Blank et al. (2003)	Y (CTI-Siemens ECAT EXACT HR++ (966))	Y	PET	15 (patients); 12 (controls)
Cardebat et al. (2003)	Y (Siemens ECAT HR+)	Y	PET	6
Sharp et al. (2004)	Y (Siemens HR++ 966)	Y	PET	16
Zahn et al. (2004)	Y (Philips ACS NT Gyroscan 1.5 Tesla)	N* (insufficient blocks per experimental condition (3) because blocks were too long (44 s))	Block	198
Crinion & Price (2005)	N (Siemens 1.5 Tesla; model not stated)	N (the calculated duration of the stimuli, the calculated duration of the acquisitions, and the stated duration of the acquisitions yield three different numbers)	Block	460
de Boissezon et al. (2005)	Y (CTI-Siemens ECAT EXACT HR+)	Y	PET	6
Connor et al. (2006)	Y (Siemens Vision 1.5 Tesla)	Y	Event-related	1024
Crinion et al. (2006)	Y (CTI-Siemens ECAT EXACT HR++/966 (16 patients and all controls) or GE Advance (8 patients))	Y	PET	12-16
Saur et al. (2006)	Y (Siemens Trio 3 Tesla)	Y	Event-related	660
Meinzer et al. (2008)	Y (Philips Intera 1.5 Tesla)	Y	Block	160
Raboyeau et al. (2008)	Y (Siemens ECAT HR+)	Y	PET	6
Richter et al. (2008)	Y (Siemens Vision plus 1.5 Tesla)	N (minor discrepancies in description of timing)	Block	134
de Boissezon et al. (2009)	Y (CTI-Siemens ECAT EXACT HR+)	Y	PET	6
Fridriksson et al. (2009)	N (not stated)	N (timing of picture presentation not clearly explained)	Event-related	120
Menke et al. (2009)	Y (Philips Intera 3 Tesla)	N (total images acquired not stated)	Event-related	Probably ~360, but not stated
Specht et al. (2009)	Y (CTI-Siemens HR+)	Y	PET	9
Warren et al. (2009)	Y (CTI-Siemens ECAT EXACT HR++/966 (10 patients and all controls) or GE Advance (6 patients))	Y	PET	12-16
Chau et al. (2010)	N (not stated)	N (inconsistent information regarding timing)	Block	90?
Fridriksson (2010)	Y (Siemens Trio 3 Tesla)	N (timing of stimuli within the silent periods	Event-related	120

		is unclear)		
Fridriksson et al. (2010)	Y (Siemens Trio 3 Tesla)	N (exact timing of picture presentation not specified)	Event-related	120
Sharp et al. (2010)	Y (Siemens HR++ 966)	Y	PET	16
Thompson et al. (2010)	Y (Siemens Trio 3 Tesla)	N (total images acquired not stated)	Event-related	Not stated
Tyler et al. (2010)	Y (Siemens Trio 3 Tesla)	N* (there was only one block per condition per run, so condition could be confounded with low frequency drift; also, the length of the sentences is not stated so it is unclear how well the HRF peak aligns with the sparse acquisitions)	Block	69
van Oers et al. (2010)	Y (Philips Achieva 3 Tesla)	Y	Block	3036
Papoutsi et al. (2011)	Y (Siemens Trio 3 Tesla)	N (length of stimuli not described)	Event-related	1059
Sebastian & Kiran (2011)	N (GE 3 Tesla; model not stated)	N* (control events took place in the inter-trial interval between language events, and may have been systematically confounded in timing; the total number of functional images acquired is not stated)	Event-related	Not stated
Szaflarski et al. (2011)	Y (Varian Unity INOVA 4 T)	N (timing not clear, because previous studies cited are not all identical in terms of timing)	Block	Not stated
Tyler et al. (2011)	Y (Siemens Trio 3 Tesla)	N (run length not stated; length of stimuli not described)	Event-related	Not stated but 1059 per Papoutsi et al. (2011)
Weiduschat et al. (2011)	Y (CTI-Siemens ECAT EXACT HR)	Y	PET	8
Allendorfer et al. (2012)	N (Phillips 3 Tesla; model not stated)	Y	Mixed	435
Fridriksson, Hubbard, et al. (2012)	N (Siemens 3 Tesla; model not stated)	N* (it appears that each of the three conditions was presented in a separate run)	Event-related	180?
Fridriksson, Richardson, et al. (2012)	Y (Siemens Trio 3 Tesla)	N (timing of stimuli within the silent periods is unclear)	Event-related	120
Marcotte et al. (2012)	Y (Siemens Trio 3 Tesla)	N (total images acquired not stated)	Event-related	Not stated
Schofield et al. (2012)	Y (Siemens Sonata 1.5 Tesla)	Y	Block	488
Wright et al. (2012)	Y (Siemens Trio 3 Tesla)	N* (there was only one block per condition per run, so condition could be confounded with low frequency drift; also, the length of the sentences is not stated so it is unclear how well the HRF peak aligns with the sparse acquisitions)	Block	69
Szaflarski et al. (2013)	N (Phillips 3 Tesla; model not stated)	Y	Block	330
Thiel et al. (2013)	Y (CTI-Siemens ECAT EXACT HR)	Y	PET	8
Abel et al. (2014)	Y (Philips Achieva 3 Tesla)	N* (trials too close together (~8 s) and insufficient jitter (1-3 s) for event-related design)	Event-related	560
Benjamin et al. (2014)	Y (Philips Achieva 3 Tesla)	N (total images acquired not stated)	Event-related	Not stated
Brownsett et al. (2014)	Y (Philips Intera 3 Tesla)	N* (timing of sentence presentation not described; sparse event-related design, but ITI of only 8 s and consistent linear order of listening and repetition trials could make it difficult to disentangle hemodynamic responses to listening and repeating trials)	Event-related	168 (patients); 280 (controls)
Mattioli et al. (2014)	Y (Siemens Avanto 1.5 Tesla)	N (timing of stimuli not clearly described)	Event-related	504
Mohr et al. (2014)	Y (Siemens Trio 3 Tesla)	Y	Event-related	76
Robson et al. (2014)	Y (Philips Achieva 3 Tesla)	N* (each condition was acquired in a separate run, which is suboptimal)	Block	417

Szaflarski et al. (2014)	Y (Philips Achieva 3 Tesla, except for 1 patient and 1 control on a Bruker 3 Tesla)	Y	Block	165
van Hees et al. (2014)	Y (Bruker MedSpec 4 Tesla)	Y	Event-related	610
Abel et al. (2015)	Y (Philips Achieva 3 Tesla)	N* (trials too close together (~8 s) and insufficient jitter (1-3 s) for event-related design)	Event-related	560
Kiran et al. (2015)	Y (Philips Achieva 3 Tesla)	N* (picture and scrambled conditions have different durations; ITI 2-4 s seems too short; total images acquired not stated)	Event-related	Not stated
Sandberg et al. (2015)	Y (Philips Achieva 3 Tesla)	N* (total images acquired not stated; ITI of 1-3 s seems short)	Event-related	Not stated
Geranmayeh et al. (2016)	Y (Siemens Trio 3 Tesla)	Y	Event-related	213
Griffis et al. (2016)	Y (Varian Unity INOVA 4 Tesla)	Y	Block	140
Sims et al. (2016)	Y (Philips Achieva 3 Tesla)	N (total images acquired not stated)	Event-related	Not stated
Darkow et al. (2017)	Y (Siemens Trio 3 Tesla)	Y	Event-related	100
Geranmayeh et al. (2017)	Y (Siemens Trio 3 Tesla)	Y	Event-related	213
Griffis, Nenert, Allendorfer, & Szaflarski (2017)	N (Siemens Allegra 3 Tesla or Philips 3 Tesla; model not stated)	Y	Block	165
Griffis, Nenert, Allendorfer, Vannest, et al. (2017)	N (Siemens Allegra 3 Tesla or Philips 3 Tesla; model not stated)	Y	Block	165
Harvey et al. (2017)	Y (Siemens Trio 3 Tesla)	Y	Block	200
Nardo et al. (2017)	Y (Siemens Trio 3 Tesla)	Y	Event-related	696
Nenert et al. (2017)	N (Philips 3 Tesla or Siemens 3 Tesla; models not stated)	Y	Block	600
Qiu et al. (2017)	Y (GE Signa 1.5 Tesla)	N* (only three pictures were named per 30-second block)	Block	186
Skipper-Kallal et al. (2017a)	Y (Siemens Trio 3 Tesla)	N* (total images acquired not stated; separation of adjacent events (covert and overt naming) will be limited because of the small amount of jitter in their timing (only 1500 ms))	Event-related	~450 but not stated
Skipper-Kallal et al. (2017b)	Y (Siemens Trio 3 Tesla)	N* (total images acquired not stated; separation of adjacent events (covert and overt naming) will be limited because of the small amount of jitter in their timing (only 1500 ms))	Event-related	~450 but not stated
Dietz et al. (2018)	Y (Philips Achieva 3 Tesla)	Y	Event-related	135
Hallam et al. (2018)	Y (GE Signa HDx 3 Tesla)	Y	Event-related	348
Nenert et al. (2018)	N (Philips 3 Tesla or Siemens 3 Tesla; models not stated)	Y	Block	600
Pillay et al. (2018)	Y (GE Excite 3 Tesla)	N (precise timing of stimuli not stated; total images acquired not stated)	Event-related	Not stated
Szaflarski et al. (2018)	Y (Siemens Allegra 3 Tesla)	Y	Block	330
van de Sandt-Koenderman et al. (2018)	N (GE 3 Tesla; model not stated)	Y	Block	132
van Oers et al. (2018)	Y (Philips Achieva 3 Tesla)	N* (stimulus presentation was self-paced, but the ITI is not reported, nor are the number of trials presented per condition; it is likely that the language and control blocks contained different numbers of trials)	Block	1656
Barbieri et al. (2019)	Y (Siemens Trio 3 Tesla or Siemens Prisma 3 Tesla)	N* (stimulus timing described does not match stated duration of data acquisition;	Block	~482

		timing of language and control trials not matched)		
Johnson et al. (2019)	Y (Siemens Trio 3 Tesla, except for 2 patients on a Siemens Prisma 3 Tesla)	N* (total images not stated; short ITI and minimal jitter)	Event-related	Not stated
Kristinsson et al. (2019)	Y (Siemens Trio 3 Tesla or Siemens Prisma 3 Tesla)	Y	Event-related	60
Purcell et al. (2019)	N (not stated)	Y	Event-related	1232 (four runs distributed over two days)
Sreedharan, Chandran, et al. (2019)	Y (Siemens Avanto 1.5 Tesla)	N* (picture naming events consistently located between blocks)	Mixed	Probably 964
Hartwigsen et al. (2020)	Y (Siemens Verio 3 Tesla)	N* (stimulus timing not described in detail; stated duration of data acquisition substantially outside possible range of duration of stimuli)	Block	740
Stockert et al. (2020)	Y (Siemens Trio 3 Tesla or Siemens Verio 3 Tesla)	Y	Event-related	660 (20 patients; paradigm 1) or 260 (14 patients; paradigm 2)

Scanner = Is the scanner described?; Timing = Is the timing of stimulus presentation and image acquisition clearly described and appropriate?; Total images = Total images acquired; fMRI = functional magnetic resonance imaging; HRF = hemodynamic response function; ITI = inter-trial interval; N = No; PET = positron emission tomography; Y = Yes; Yellow underline = minor limitation; Orange underline/* = moderate limitation.

Supplementary Table S7. Imaging: Methodology part 2

Study	Acquisition	Preprocessing	Model fitting	Registration	Notes
Weiller et al. (1995)	Y (axial; field of view = 5.4 cm; perisylvian only)	Y	Y	Y	
Belin et al. (1996)	Y (7 transaxial slices 12 mm apart)	Y	Y	Y	
Ohyama et al. (1996)	N (91 mm field of view; coverage limitations not stated)	Y	Y	N (lesion impact not addressed)	
Heiss et al. (1997)	Y (whole brain)	Y	Y	N/A	
Karbe et al. (1998)	Y (whole brain)	Y	Y	N/A	
Cao et al. (1999)	Y (axial, perisylvian only)	Y	N (first level cross-correlation analysis unclear)	N/A	
Heiss et al. (1999)	Y (whole brain)	Y	Y	N/A	
Kessler et al. (2000)	Y (whole brain)	Y	Y	N/A	
Rosen et al. (2000)	Y (whole brain)	Y	Y	Y	1 patient scanned on different PET scanner, and not scanned with fMRI; controls had different fMRI sequence to patients
Blasi et al. (2002)	Y (whole brain)	Y	Y	N (not described)	
Leff et al. (2002)	Y (whole brain)	Y	Y	Y	
Blank et al. (2003)	Y (whole brain)	Y	Y	Y	
Cardebat et al. (2003)	Y (whole brain)	Y	Y	N (lesion impact not addressed)	
Sharp et al. (2004)	Y (whole brain)	Y	Y	Y	
Zahn et al. (2004)	Y (whole brain)	Y	Y	N/A	
Crinion & Price (2005)	Y (whole brain)	Y	Y	Y	
de Boissezon et al. (2005)	Y (whole brain)	Y	Y	N (lesion impact not addressed; minimal due to lesions being small and subcortical)	
Connor et al. (2006)	Y (whole brain)	Y	Y	Y	
Crinion et al. (2006)	Y (whole brain)	Y	Y	Y	Two different scanners used for patients, but not for controls
Saur et al. (2006)	Y (whole brain)	Y	Y	Y	
Meinzer et al. (2008)	Y (whole brain)	Y	Y	Y	
Raboyeau et al. (2008)	Y (whole brain)	Y	Y	N (lesion impact not addressed)	
Richter et al. (2008)	Y (whole brain)	Y	Y	N (lesion impact not addressed)	
de Boissezon et al. (2009)	Y (whole brain)	Y	Y	N (lesion impact not addressed)	
Fridriksson et al. (2009)	Y (whole brain)	Y	Y	Y	Sparse sampling
Menke et al. (2009)	Y (whole brain)	Y	Y	Y	
Specht et al. (2009)	Y (whole brain)	Y	Y	Y	
Warren et al. (2009)	Y (whole brain)	Y	Y	Y	Two different scanners used for patients, but not for controls
Chau et al. (2010)	Y (whole brain)	Y	Y	N (lesion impact	

				not addressed)	
Fridriksson (2010)	Y (whole brain)	Y	Y	Y	Sparse sampling
Fridriksson et al. (2010)	Y (whole brain)	Y	Y	Y	Sparse sampling
Sharp et al. (2010)	Y (whole brain)	Y	Y	Y	
Thompson et al. (2010)	Y (whole brain)	Y	Y	Y	
Tyler et al. (2010)	Y (whole brain)	Y	Y	Y	Sparse sampling
van Oers et al. (2010)	Y (whole brain)	Y	Y	Y	Breath holding scan also done to measure hemodynamic responsiveness
Papoutsi et al. (2011)	Y (whole brain)	Y	N (lacks explanation of event durations)	Y	
Sebastian & Kiran (2011)	Y (whole brain)	Y	N (only correct trials are included but it is not stated how incorrect trials were modeled; in general, it is not stated whether the control events were modeled at all)	Y	
Szaflarski et al. (2011)	Y (whole brain)	Y	Y	N (lesion impact not addressed)	
Tyler et al. (2011)	Y (whole brain)	Y	N (lacks explanation of event durations)	Y	
Weiduschat et al. (2011)	Y (whole brain)	Y	Y	Y	
Allendorfer et al. (2012)	Y (whole brain)	Y	N (no description of HRF model, which is important given sparse sampling design)	N (lesion impact not addressed)	Sparse sampling
Fridriksson, Hubbard, et al. (2012)	Y (whole brain)	Y	N (not described clearly)	Y	Sparse sampling
Fridriksson, Richardson, et al. (2012)	Y (whole brain)	Y	Y	Y	Sparse sampling; 26 patients were also scanned with arterial spin labelling
Marcotte et al. (2012)	Y (whole brain)	Y	Y	N (lesion impact not addressed)	
Schofield et al. (2012)	Y (mostly whole brain but convexity or cerebellum excluded in some participants)	Y	Y	Y	
Wright et al. (2012)	Y (whole brain)	Y	Y	Y	Sparse sampling
Szaflarski et al. (2013)	Y (whole brain)	Y	Y	Y	
Thiel et al. (2013)	Y (whole brain)	Y	Y	N (lesion impact not addressed)	
Abel et al. (2014)	Y (whole brain)	Y	Y	N (lesion impact not addressed)	
Benjamin et al. (2014)	Y (whole brain)	N (not described)	N (not described clearly)	N (lesion impact not addressed)	
Brownsett et al. (2014)	Y (whole brain)	Y	N* (consistent linear order of listening and repetition trials could make it difficult to disentangle hemodynamic responses to listening and repeating trials)	Y	Sparse sampling; different task structure in controls (two repetition trials per listening trial) raises concerns about comparisons between groups
Mattioli et al. (2014)	N (unclear; number of	Y	N (model fitting of	Y	

	slices not stated)		noise "bip" not clearly described)		
Mohr et al. (2014)	Y (whole brain)	Y	Y	N (lesion impact not addressed)	Sparse sampling
Robson et al. (2014)	Y (whole brain)	Y	Y	Y	Spin echo fMRI to minimize ATL dropout
Szaflarski et al. (2014)	Y (whole brain)	Y	Y	Y	
van Hees et al. (2014)	Y (whole brain)	Y	Y	N (lesion impact not addressed)	Slow event-related design; sparse sampling
Abel et al. (2015)	Y (whole brain)	Y	Y	N (lesion impact not addressed)	
Kiran et al. (2015)	Y (whole brain)	Y	Y	Y	Controls were run on two different sets of parameters, neither of which was the same as the patients
Sandberg et al. (2015)	Y (whole brain)	Y	Y	Y	
Geranmayeh et al. (2016)	Y (whole brain)	Y	Y	Y	Sparse sampling; mini-blocks of 2-4 trials
Griffis et al. (2016)	Y (whole brain)	Y	Y	N (lesion impact not addressed)	
Sims et al. (2016)	Y (whole brain)	Y	Y	Y	No smoothing
Darkow et al. (2017)	Y (whole brain)	Y	Y	Y	Sparse sampling
Geranmayeh et al. (2017)	Y (whole brain)	Y	Y	Y	Sparse sampling; mini-blocks of 2-4 trials
Griffis, Nenert, Allendorfer, & Szaflarski (2017)	Y (whole brain)	Y	Y	Y	
Griffis, Nenert, Allendorfer, Vannest, et al. (2017)	Y (whole brain)	Y	Y	Y	
Harvey et al. (2017)	Y (whole brain)	Y	Y	N (lesion impact not addressed)	
Nardo et al. (2017)	Y (whole brain)	Y	Y	Y	
Nenert et al. (2017)	Y (whole brain)	Y	Y	N (lesion impact not addressed)	
Qiu et al. (2017)	Y (whole brain)	N (not described)	N (no description of model fitting)	N (not described)	
Skipper-Kallal et al. (2017a)	Y (whole brain)	Y	N* (entire phases where picture was displayed modeled as covert and overt naming; difficult to separate phases due to timing)	Y	
Skipper-Kallal et al. (2017b)	Y (whole brain)	Y	N* (not stated but see Skipper-Kallal et al. (2017b))	Y	At each voxel, individuals with lesions to that voxel were excluded from analysis
Dietz et al. (2018)	Y (whole brain)	Y	N (no description of HRF model, which is important given sparse sampling design)	N (lesion impact not addressed)	Additional methodological details in Dietz et al. (2016)
Hallam et al. (2018)	Y (whole brain)	Y	Y	Y	Interleaved silent steady state imaging
Nenert et al. (2018)	Y (whole brain)	Y	Y	N (lesion impact not addressed)	Scanner identity appropriately included as covariate
Pillay et al. (2018)	Y (whole brain)	Y	Y	Y	

Szaflarski et al. (2018)	Y (whole brain)	Y	Y	Y	
van de Sandt-Koenderman et al. (2018)	Y (whole brain)	Y	Y	N (lesion impact not addressed)	
van Oers et al. (2018)	Y (whole brain)	Y	Y	Y	Not all participants scanned at each time point; the number scanned at each time point is not stated
Barbieri et al. (2019)	Y (whole brain)	Y	Y	Y	2 runs before treatment and 2 runs after treatment; each pair of runs took place on two separate days (1-7 days apart)
Johnson et al. (2019)	Y (whole brain)	Y	N* (unclear whether there was sufficient resting data to allow the key contrast to be computed)	Y	
Kristinsson et al. (2019)	Y (whole brain)	Y	Y	Y	Sparse sampling
Purcell et al. (2019)	Y (cerebellum excluded)	Y	N* (not feasible to separate closely spaced instruction, word, and letter/response, especially when responses will be compared to rest)	Y	
Sreedharan, Chandran, et al. (2019)	Y (whole brain)	Y	N* (event timing will make conditions difficult to disentangle)	N (lesion impact not addressed)	
Hartwigsen et al. (2020)	Y (whole brain)	Y	Y	N (lesion impact not addressed)	
Stockert et al. (2020)	N (whole brain; TE = 96 ms questionable)	Y	Y	Y	

Acquisition = Are the imaging acquisition parameters, including coverage, adequately described and appropriate?; Preprocessing = Is preprocessing and intrasubject coregistration adequately described and appropriate?; Model fitting = Is first level model fitting adequately described and appropriate?; Registration = Is intersubject normalization adequately described and appropriate?; ATL = anterior temporal lobe; fMRI = functional magnetic resonance imaging; HRF = hemodynamic response function; N = No; N/A = N/A—no intersubject normalization.; PET = positron emission tomography; Y = Yes; Yellow underline = minor limitation; Orange underline/* = moderate limitation.

Supplementary Table S8. Conditions

Study	Condition	Response type	Repetitions	All groups could do?	All indivs could do?	Notes
Weiller et al. (1995)	Verb generation	Multiple words (covert)	2	Y	Y	Auditory presentation; pre-scan behavioral data reported
	Pseudoword repetition	Multiple words (covert)	2	Y	Y	
	Rest	None	2	N/A	N/A	
Belin et al. (1996)	Word repetition with MIT-like intonation	Word (overt)	1	Y	U	
	Word repetition	Word (overt)	1	Y	U	
	Listening to words	None	1	N/A	N/A	
	Rest	None	1	N/A	N/A	
Ohyama et al. (1996)	Word repetition	Word (overt)	2	Y	Y	Patients were able to repeat words well, with phonemic errors on no more than 4 out of 48 words; counting condition not analyzed in this paper
	Counting	Multiple words (overt)	2	Y	Y	
	Rest	None	2	N/A	N/A	
Heiss et al. (1997)	Word repetition	Word (overt)	1	U	U	No information about repetition rate, or whether repetition was overt or covert
	Rest	None	1	N/A	N/A	
Karbe et al. (1998)	Word repetition	Word (overt)	4 (?)	U	U	Inability to repeat single words was an exclusion criterion, but many patients had severe aphasia so it is unclear how they would have performed
	Rest	None	4 (?)	N/A	N/A	
Cao et al. (1999)	Picture naming	Word (covert)	4	Y	Y	
	Viewing nonsense drawings	None	4	N/A	N/A	
Heiss et al. (1999)	Noun repetition	Word (overt)	4	U	U	Inclusion criterion would suggest all patients could do the task, but this is not stated
	Rest	None	4	N/A	N/A	
Kessler et al. (2000)	Word repetition	Word (overt)	4	Y	Y	Inclusion criterion was applied to ensure that the task could be performed
	Rest	None	4	N/A	N/A	
Rosen et al. (2000)	Word stem completion (PET)	Word (overt)	4	Y	Y	Pseudoword reading condition not analyzed in this paper
	Reading pseudowords aloud (PET)	Word (overt)	4	Y	N	
	Rest (PET)	None	2	N/A	N/A	
	Word stem completion (fMRI)	Word (covert)	15-30 (?)	Y	Y	
	Rest (fMRI)	None	15-30 (?)	N/A	N/A	
Blasi et al. (2002)	Word stem completion (novel items)	Word (covert)	196	Y	U	Novel items were presented in runs 1, 6, 7, and 8; repeated items were presented in runs 2, 3, 4, and 5; of the four repeated runs, only run 5 was analyzed.
	Word stem completion (repeated items)	Word (covert)	196	Y	U	
	Rest	None	Implicit baseline	N/A	N/A	
Leff et al. (2002)	Listening to words at 10 wpm	None	2	N/A	N/A	
	Listening to words at 35 wpm	None	2	N/A	N/A	
	Listening to words at 55 wpm	None	2	N/A	N/A	
	Listening to words at 70 wpm	None	2	N/A	N/A	
	Listening to words at 85 wpm	None	2	N/A	N/A	
	Listening to words at 95 wpm	None	2	N/A	N/A	
	Listening to words at 115 wpm	None	2	N/A	N/A	

	Listening to words at 130 wpm	None	2	N/A	N/A	
Blank et al. (2003)	Propositional speech production	Sentence (overt)	Aphasia: 5; control: 4	Y	Y	Alertness maintained in rest by asking participants to listen to environmental sounds that were presented before and after data acquisition; speech was recorded and rate was measured, also QPA was done of a separate speech sample outside the scanner
	Counting	Multiple words (overt)	Aphasia: 5; control: 4	Y	Y	
	Rest	None	Aphasia: 5; control: 4	N/A	N/A	
Cardebat et al. (2003)	Word generation	Word (overt)	4	Y	U	Participants were asked to generate words that were semantically related to binaurally presented stimuli; 2 runs involved nouns and 2 involved verbs
	Rest	None	2	N/A	N/A	
Sharp et al. (2004)	Semantic decision	Word (overt)	Aphasia: 8; control: 4	Y	Y	Seems the response was a spoken word, but this is not stated explicitly; assuming all individuals could do the tasks because this was an inclusion criterion and behavioral data supports
	Syllable count decision	Word (overt)	Aphasia: 8; control: 4	Y	Y	
	Semantic decision (noise vocoded) (control only)	Word (overt)	4 (control)	Y	Y	
	Syllable count decision (noise vocoded) (control only)	Word (overt)	4 (control)	Y	Y	
Zahn et al. (2004)	Phonetic decision (reversed words vs sounds)	Button press	3	Y	N	
	Lexical decision (words vs reversed words)	Button press	3	Y	Y	
	Semantic decision	Button press	3	Y	N	
	Rest	None	9	N/A	N/A	
Crinion & Price (2005)	Listening to narrative speech	None	32	N/A	N/A	A post-scan surprise recognition test asked whether or not 38 phrases had occurred in any story; patients answered 12-33 of these questions correctly; controls answered 24-37 correctly; also note that all patients performed above chance on CAT auditory sentence comprehension (73%+ accuracy)
	Listening to reversed speech	None	8	N/A	N/A	
de Boissezon et al. (2005)	Word generation	Word (overt)	4	Y	Y	Nouns in two runs, verbs in two runs, combined here because they were combined in analysis
	Rest	None	2	N/A	N/A	
Connor et al. (2006)	Word stem completion (novel items)	Word (covert)	196	Y	U	Novel items were presented in runs 1, 6, 7, and 8; repeated items were presented in runs 2, 3, 4, and 5; of the four repeated runs, only run 5 was analyzed.
	Word stem completion (repeated items)	Word (covert)	196	Y	U	
	Rest	None	Implicit baseline	N/A	N/A	
Crinion et al. (2006)	Listening to narrative speech	None	6-8	N/A	N/A	
	Listening to reversed speech	None	6-8	N/A	N/A	
Saur et al. (2006)	Listening to sentences and making a plausibility	Button press	92	U	N	In the auditory sentence comprehension condition,

	judgment					participants had to press a button to semantically anomalous sentences; in the reversed speech condition, they had to always press the button; the behavioral scores provided are not explained in the paper, but per a personal communication cited by Geranmayeh et al. (2014), 10% of the score reflects discrimination between intelligible and reversed speech, while 90% reflects semantic anomaly judgment; our coding of behavior is based on this limited information
	Listening to reversed speech	Button press	92	Y	U	
	Rest	None	Implicit baseline	N/A	N/A	
Meinzer et al. (2008)	Picture naming (trained items)	Word (overt)	8	Y	N	One participant was < 10% on trained and untrained items at T1
	Picture naming (untrained items)	Word (overt)	8	Y	N	
	Rest	None	16	N/A	N/A	
Raboyeau et al. (2008)	Picture naming (native language)	Word (overt)	Aphasia: 4; control: 2	Y	U	Picture naming in native language in controls not analyzed in this paper
	Picture naming (relearned foreign language) (controls only)	Word (overt)	2	Y	U	
	Rest	None	2	N/A	N/A	
Richter et al. (2008)	Reading words silently	Word (covert)	4	Y	U	Preliminary data on the tasks suggests that patients would have been able to perform them, and patients were interviewed regarding the tasks after each fMRI session, however the outcomes of these interviews are not reported
	Word stem completion	Word (covert)	4	Y	U	
	Rest	None	10 (?)	N/A	N/A	
de Boissezon et al. (2009)	Word generation	Word (overt)	4	Y	Y	
	Rest	None	2	N/A	N/A	
Fridriksson et al. (2009)	Picture naming	Word (overt)	80	Y	N	
	Viewing scrambled images	None	40	N/A	N/A	
Menke et al. (2009)	Picture naming (trained items)	Word (overt)	30	N	N	Patients could not name trained and untrained items at baseline
	Picture naming (untrained items)	Word (overt)	30	N	N	
	Picture naming (already known items)	Word (overt)	30	Y	U	
	Rest	None	Implicit baseline	N/A	N/A	
Specht et al. (2009)	Lexical decision (words vs pseudowords)	Button press	3	Y	Y	Behavioral data was lost, but it is clearly stated that all participants could perform all tasks above chance; the tone decision task is not described in sufficient detail, but since it is not used in any contrast of interest, the conditions are
	Lexical decision (words vs reversed foreign words)	Button press	3	Y	Y	

	Tone decision	Button press	3	Y	Y	coded as being clearly described
Warren et al. (2009)	Listening to narrative speech	None	6-8	N/A	N/A	
	Listening to reversed speech	None	6-8	N/A	N/A	
Chau et al. (2010)	Answering questions from Cantonese Aphasia Battery	Button press	3	U	U	Nature of questions not described in detail; responses involved raising left or right finger (not button press per se)
	Visual decision	Button press	3	U	U	
Fridriksson (2010)	Picture naming	Word (overt)	80	Y	U	Patients with fewer than 5 correct responses in any session were excluded; there were probably some patients who made 5 or more correct responses but less than 10%, but this is not reported
	Viewing abstract pictures	None	40	N/A	N/A	
Fridriksson et al. (2010)	Picture naming	Word (overt)	80	Y	Y	
	Viewing abstract pictures	None	40	N/A	N/A	
Sharp et al. (2010)	Semantic decision	Word (overt)	Aphasia: 8; control: 4	Y	Y	Seems the response was a spoken word, but this is not stated explicitly; assuming all individuals could do the semantic task because this was an inclusion criterion and behavioral data (PPT) supports, but not sure about the phonological task
	Syllable count decision	Word (overt)	Aphasia: 8; control: 4	Y	U	
	Semantic decision (noise vocoded) (control only)	Word (overt)	4 (control)	Y	Y	
	Syllable count decision (noise vocoded) (control only)	Word (overt)	4 (control)	Y	Y	
Thompson et al. (2010)	Auditory sentence-picture matching (auditory; object cleft)	Button press	60	N	N	
	Auditory sentence-picture matching (subject cleft)	Button press	60	Y	Y	
	Auditory sentence-picture matching (simple past tense active)	Button press	60	Y	N	
	Rest	None	Implicit baseline	N/A	N/A	
Tyler et al. (2010)	Listening to normal sentences and detecting a target word	Button press	2	Y	U	Auditory presentation; target detection task with early and late targets; 12-15 trials per block with single sparse acquisition each, but only one block per run, in fixed order; task can apparently be performed by patients with brain damage, but accuracy is not reported
	Listening to grammatical but meaningless sentences and detecting a target word	Button press	2	Y	U	
	Listening to scrambled sentences and detecting a target word	Button press	2	Y	U	
	Listening to "musical rain" and detecting a period of white noise	Button press	2	Y	U	
	Rest	None	2	N/A	N/A	
van Oers et al. (2010)	Written word-picture matching	Button press	6	Y	Y	Patients who could not do tasks were excluded from analyses of those tasks (1 patient from semantic decision; 3 patients from verb generation); wording is somewhat unclear regarding exclusion of patients who
	Semantic decision	Button press	6	Y	Y	
	Verb generation	Word (covert)	8	Y	Y	

	Visual decision	Button press	12	U	U	could not perform verb generation, but we assume they were excluded
	Rest	None	20	N/A	N/A	
Papoutsi et al. (2011)	Listening to unambiguous sentences ("unambiguous")	None	42	N/A	N/A	
	Listening to ambiguous sentences with dominant resolution ("dominant")	None	42	N/A	N/A	
	Listening to ambiguous sentences with subordinate resolution ("subordinate")	None	42	N/A	N/A	
	Listening to filler sentences	None	126	N/A	N/A	
	Listening to "musical rain"	None	42	N/A	N/A	
	Rest	None	Implicit baseline	N/A	N/A	
Sebastian & Kiran (2011)	Picture naming	Word (overt)	60	Y	Y	
	Viewing scrambled images and saying "pass"	Word (overt)	60	U	U	
	Semantic decision	Button press	48	Y	Y	
	Visual decision	Button press	48	U	U	
Szaflarski et al. (2011)	Semantic decision	Button press	Not stated	U	N	Based on Binder et al. (1997), but details not reported; group only just above chance, unclear whether significantly better; clearly some individuals were at chance
	Tone decision	Button press	Not stated	U	N	
Tyler et al. (2011)	Listening to unambiguous sentences ("unambiguous")	None	42	N/A	N/A	
	Listening to ambiguous sentences with dominant resolution ("dominant")	None	42	N/A	N/A	
	Listening to ambiguous sentences with subordinate resolution ("subordinate")	None	42	N/A	N/A	
	Listening to filler sentences	None	126	N/A	N/A	
	Listening to "musical rain"	None	42	N/A	N/A	
	Rest	None	Implicit baseline	N/A	N/A	
Weiduschat et al. (2011)	Verb generation	Word (covert)	4	U	U	
	Rest	None	4	N/A	N/A	
Allendorfer et al. (2012)	Verb generation (overt, event-related)	Multiple words (overt)	15	Y	U	Given the means and standard deviations presented, it is likely that some patients could not perform some tasks; post-scan recognition tests not considered to quantify performance
	Verb generation (covert, event-related)	Multiple words (covert)	15	U	U	
	Noun repetition (event-related)	Multiple words (overt)	15	Y	U	
	Verb generation (covert, block)	Multiple words (covert)	10	U	U	
	Finger tapping (block)	Other	10	U	U	
Fridriksson, Hubbard, et al. (2012)	Listening to/watching audiovisual sentences, while producing the same sentences in unison (speech entrainment)	Sentence (overt)	30 (?)	Y	U	Rest condition implied but not described
	Listening to reversed sentences and viewing a mouth speaking, while	Sentence (overt)	30 (?)	Y	U	

	producing unrelated sentences					
	Listening to/watching audiovisual sentences and viewing a mouth	None	30 (?)	N/A	N/A	
	Rest	None	Implicit baseline	N/A	N/A	
Fridriksson, Richardson, et al. (2012)	Picture naming	Word (overt)	80	Y	U	
	Viewing abstract pictures	None	40	N/A	N/A	
Marcotte et al. (2012)	Picture naming (already known items)	Word (overt)	20	Y	Y	
	Picture naming (trained items)	Word (overt)	20	N	N	
	Picture naming (untrained items)	Word (overt)	40	N	N	
	Viewing scrambled images and saying "baba"	Word (overt)	20	Y	Y	
	Rest	None	Implicit baseline	N/A	N/A	
Schofield et al. (2012)	Listening to word pairs, speaker gender judgment	Button press	18	Y	U	
	Listening to reversed word pairs, speaker gender judgment	Button press	18	Y	U	
	Rest	None	40 (?)	N/A	N/A	
Wright et al. (2012)	Listening to normal sentences and detecting a target word	Button press	2	Y	Y	Auditory presentation; target detection task with early and late targets; 12-15 trials per block with single sparse acquisition each, but only one block of each condition per run, in fixed order
	Listening to grammatical but meaningless sentences and detecting a target word	Button press	2	Y	Y	
	Listening to scrambled sentences and detecting a target word	Button press	2	Y	Y	
	Listening to "musical rain" and detecting a period of white noise	Button press	2	Y	Y	
	Rest	None	2	N/A	N/A	
Szaflarski et al. (2013)	Semantic decision	Button press	10	N	N	
	Tone decision	Button press	12	N	N	
Thiel et al. (2013)	Verb generation	Word (overt)	4	U	U	
	Rest	None	4	N/A	N/A	
Abel et al. (2014)	Picture naming (semantic trained items)	Word (overt)	30	Y	U	
	Picture naming (phonological trained items)	Word (overt)	30	Y	U	
	Picture naming (untrained items)	Word (overt)	30	Y	U	
	Picture naming (already known items)	Word (overt)	42	Y	U	
	Rest	None	Implicit baseline	N/A	N/A	
Benjamin et al. (2014)	Word generation	Word (overt)	60	U	U	
	Rest	None	Implicit baseline	N/A	N/A	
Brownsett et al. (2014)	Listening to sentences	None	Aphasia: not	N/A	N/A	Paradigm was different in patients and controls, and is

			stated; control: 40			not described in sufficient detail for patients; in two patients, only single words were produced
	Repeating sentences (sentence from previous trial)	Sentence (overt)	Aphasia: not stated; control: 40	Y	N	
	Listening to noise vocoded sentences (control only)	None	40 (control)	N/A	N/A	
	Repeating noise vocoded sentences (control only)	Sentence (overt)	80 (control)	Y	U	
	Listening to segmented white noise	None	Aphasia: not stated; control: 40	N/A	N/A	
Mattioli et al. (2014)	Listening to sentences and making a plausibility judgment	Button press	56	Y	U	There is also mention of a noise "bip" that preceded each sentence but details are lacking; half of the sentences were semantically anomalous
	Listening to reversed speech	None	56	N/A	N/A	
Mohr et al. (2014)	Listening to high ambiguity sentences	None	19	N/A	N/A	
	Listening to low ambiguity sentences	None	19	N/A	N/A	
	Listening to signal-correlated noise	None	19	N/A	N/A	
	Rest	None	19	N/A	N/A	
Robson et al. (2014)	Semantic decision (written word)	Button press	16	Y	N	
	Semantic decision (picture)	Button press	16	Y	N	
	Visual decision	Button press	16	Y	N	
	Rest	None	48	N/A	N/A	
Szaflarski et al. (2014)	Verb generation	Multiple words (covert)	5	Y	U	
	Finger tapping	Other	6	Y	Y	
van Hees et al. (2014)	Picture naming (phonological trained items)	Word (overt)	30	Y	N	Some patients named < 10% correct at T1
	Picture naming (semantic trained items)	Word (overt)	30	Y	N	
	Picture naming (known items)	Word (overt)	30	Y	Y	
	Viewing scrambled images	None	30	N/A	N/A	
Abel et al. (2015)	Picture naming	Word (overt)	132	Y	Y	
	Rest	None	Implicit baseline	N/A	N/A	
Kiran et al. (2015)	Picture naming (trained)	Word (overt)	40	U	U	
	Picture naming (untrained)	Word (overt)	40	U	U	
	Viewing scrambled images and saying "skip"	Word (overt)	80	U	U	
	Semantic feature decision	Button press	40	U	U	
	Visual decision	Button press	40	U	U	
Sandberg et al. (2015)	Concreteness judgment (abstract words)	Button press	60	Y	N	2 patients below chance on abstract words per supplementary table 2
	Concreteness judgment (concrete words)	Button press	60	Y	Y	
	Letter string judgment	Button press	60	U	U	
	Rest	None	Implicit	N/A	N/A	

			baseline			
Geranmayeh et al. (2016)	Propositional speech production	Sentence (overt)	60	Y	N	
	Counting	Multiple words (overt)	48	Y	U	
	Target decision	Button press	48	Y	U	
	Rest	None	45	N/A	N/A	
Griffis et al. (2016)	Verb generation	Multiple words (covert)	7	Y	Y	
	Finger tapping	Other	7	U	U	
Sims et al. (2016)	Semantic feature decision	Button press	64	Y	U	Number of visual decision trials not reported
	Visual decision	Button press	Not stated	Y	U	
	Semantic relatedness decision	Button press	50	Y	U	
	Pseudoword identity decision	Button press	50	Y	U	
	Rest	None	Implicit baseline	N/A	N/A	
Darkow et al. (2017)	Picture naming	Word (overt)	80	Y	Y	
	Rest	None	20	N/A	N/A	
Geranmayeh et al. (2017)	Propositional speech production	Sentence (overt)	60	Y	Y	All participants could do the target decision task except for one who was at chance
	Counting	Multiple words (overt)	48	Y	U	
	Target decision	Button press	48	Y	N	
	Rest	None	45	N/A	N/A	
Griffis, Nenert, Allendorfer, & Szaflarski (2017)	Semantic decision	Button press	5	N	N	Group performance below chance; several patients at 0 which is difficult to understand in a 2AFC task
	Tone decision	Button press	6	U	U	
Griffis, Nenert, Allendorfer, Vannest, et al. (2017)	Semantic decision	Button press	5	N	N	Group performance below chance; several patients at 0 which is difficult to understand in a 2AFC task
	Tone decision	Button press	6	U	U	
Harvey et al. (2017)	Picture naming	Word (overt)	20	Y	Y	Assume all individuals could do based on inclusion criterion and BNT scores
	Viewing patterns	None	20	N/A	N/A	
Nardo et al. (2017)	Picture naming (untrained items, word cue)	Word (overt)	54	Y	U	Spectrally rotated noise vocoded auditory stimulus in no-cue conditions; one patient had a BNT of 1/60 so it is unclear whether that patient could do the task
	Picture naming (untrained items, initial phonemes cue)	Word (overt)	54	Y	U	
	Picture naming (untrained items, final phonemes cue)	Word (overt)	54	Y	U	
	Picture naming (untrained items, no cue)	Word (overt)	54	Y	U	
	Picture naming (trained items, word cue)	Word (overt)	53	Y	U	
	Picture naming (trained items, initial phonemes cue)	Word (overt)	53	Y	U	
	Picture naming (trained items, final phonemes cue)	Word (overt)	53	Y	U	
	Picture naming (trained items, no cue)	Word (overt)	53	Y	U	
	Rest	None	Implicit baseline	N/A	N/A	
Nenert et al. (2017)	Semantic decision	Button press	10	U	U	Behavioral data are provided for the semantic decision and tone decision tasks, but the
	Tone decision	Button press	10	U	U	

	Verb generation	Multiple words (covert)	10	U	U	denominator is unclear; a post-scan recognition test for verb generation is reported, but this cannot confirm verb generation performance
	Finger tapping	Other	10	U	U	
Qiu et al. (2017)	Picture naming	Word (overt)	9	U	U	
	Rest	None	9	N/A	N/A	
Skipper-Kallal et al. (2017a)	Picture naming (silently name)	Word (covert)	32	Y	Y	Covert and overt naming were modeled as two phases of each trial (there was a cue to produce the name after 7500-9000 ms); 5 participants who were more impaired were given easier pictures to name; patients who named less than 20% of items correctly were excluded
	Picture naming (produce the name)	Word (overt)	32	Y	Y	
	Rest	None	Implicit baseline	N/A	N/A	
Skipper-Kallal et al. (2017b)	Picture naming (prepare to name)	Word (covert)	32	Y	Y	Covert and overt naming were modeled as two phases of each trial (there was a cue to produce the name after 7500-9000 ms); 14 participants who were more impaired were given easier pictures to name; patients who named less than 10% of items correctly were excluded
	Picture naming (produce the name)	Word (overt)	32	Y	Y	
	Rest	None	Implicit baseline	N/A	N/A	
Dietz et al. (2018)	Verb generation (covert)	Multiple words (covert)	15	U	U	Evidence for task performance from Dietz et al. (2016)
	Verb generation (overt)	Multiple words (overt)	15	Y	U	
	Noun repetition	Multiple words (overt)	15	Y	U	
Hallam et al. (2018)	Listening to high ambiguity sentences	None	24	N/A	N/A	All but one patient had good single word comprehension, which was argued to support sentence comprehension
	Listening to low ambiguity sentences	None	24	N/A	N/A	
	Listening to spectrally rotated speech	None	24	N/A	N/A	
	Pressing a button to a visual cue	Button press	9	U	U	
	Rest	None	12	N/A	N/A	
Nenert et al. (2018)	Semantic decision	Button press	5	N	N	Assume semantic decision is out of 25, so chance is 12.5 and 95% CI below chance at T2; post-scan recognition test for verb generation not considered to quantify task performance
	Tone decision	Button press	5	Y	U	
	Verb generation	Multiple words (covert)	5	U	U	
	Finger tapping	Other	5	U	U	
Pillay et al. (2018)	Reading nouns aloud	Word (overt)	72	Y	N	Some participants had < 10% accuracy, but this is appropriately addressed in the analysis
	Rest	None	Implicit baseline	N/A	N/A	
Szaflarski et al. (2018)	Semantic decision	Button press	5	U	U	
	Tone decision	Button press	6	U	U	
van de Sandt-Koenderman et al. (2018)	Listening to narrative speech	None	6	N/A	N/A	
	Listening to reversed speech	None	6	N/A	N/A	
van Oers et al. (2018)	Written word-picture matching	Button press	6	U	U	

	Semantic decision	Button press	6	U	U	
	Visual decision	Button press	12	U	U	
	Rest	None	12	N/A	N/A	
Barbieri et al. (2019)	Auditory sentence-picture verification	Button press	32	U	U	Based on the behavioral data obtained outside the scanner, it is likely that many patients were at chance on the language task
	Listening to reversed speech and viewing scrambled pictures	Button press	8	U	U	
Johnson et al. (2019)	Picture naming (trained items)	Word (overt)	36	U	U	The untrained group were not actually trained on "trained items"; no accuracy data for untrained group (except for lack of change between T1 and T2)
	Picture naming (untrained items, from control category)	Word (overt)	36	U	U	
	Picture naming (untrained items, from experimental categories)	Word (overt)	36	U	U	
	Viewing scrambled images and saying "skip"	Word (overt)	36	U	U	
	Rest	None	Implicit baseline	N/A	N/A	
Kristinsson et al. (2019)	Picture naming	Word (overt)	40	Y	U	
	Viewing abstract pictures	None	20	N/A	N/A	
Purcell et al. (2019)	Spelling probe (training items)	Button press	60	Y	U	Condition 3 not used in any contrasts
	Spelling probe (known items)	Button press	60	Y	U	
	Case verification	Button press	60	Y	U	
	Rest	None	Implicit baseline	N/A	N/A	
Sreedharan, Chandran, et al. (2019)	Neurofeedback (try to activate language areas)	Other	24	U	U	Suggested strategies to activate language areas included "making a speech, having a conversation, reciting a poem or any other form of language activity performed covertly"; picture naming task involved covert word response and button press; picture naming task not used in any contrast; word generation task used only to generate ROIs
	Rest	None	24	N/A	N/A	
	Picture naming	Other	First and last timepoints: 48; other timepoints: 0	N	N	
	Word generation	Multiple words (covert)	5	U	U	
Hartwigsen et al. (2020)	Syllable count decision	Button press	10	Y	Y	Extent of recovery supports the assertion that all individuals could do the tasks
	Semantic decision	Button press	10	Y	Y	
	Rest	None	20	N/A	N/A	
Stockert et al. (2020)	Listening to normal sentences and making a plausibility judgment (paradigm 1)	None	46	U	U	Description implies that paradigm 2 did not include a semantically anomalous condition, but previous papers indicate that it did; conditions 2, 5, and 6 were not used, and condition 7 was effectively contrasted out; reported behavioral data collapses across conditions and paradigms and so does not establish performance on any specific condition, but the data suggest that at least the conditions where no language-related decisions were
	Listening to semantically anomalous sentences and making a plausibility judgment (paradigm 1)	Button press	46	U	U	
	Listening to reversed speech	Button press	Paradigm 1: 92; paradigm 2: 30	Y	U	
	Listening to normal sentences (paradigm 2)	Button press	15	Y	U	
	Listening to semantically anomalous sentences	Button press	15	Y	U	

	(paradigm 2)					required could have been performed by all groups
	Listening to pseudoword speech (paradigm 2)	Button press	30	Y	U	
	Rest	None	Implicit baseline	N/A	N/A	

Repetitions = Number of times the condition was repeated per scanning session (PET measurements, blocks, or events); All groups could do? = Were all groups at all time points able to perform the task (if any)?; All indivs could do = Were all individuals at all time points able to perform the task (if any)?; 2AFC = two-alternative forced choice; BNT = Boston Naming Test; CAT = Comprehensive Aphasia Test; fMRI = functional magnetic resonance imaging; MIT = melodic intonation therapy; N = No; N/A = not applicable (no task); PET = positron emission tomography; PPT = Pyramids and Palm Trees; QPA = Quantitative Production Analysis; T1, T2, etc. = first time point, second time point, etc.; U = Unknown; wpm = words per minute; Y = Yes; Yellow underline = minor limitation; Orange underline = moderate limitation.

Supplementary Table S9. Contrasts

Contrast	Language condition	Control condition	Matched for						Ctrl activation			Notes
			Vis	Aud	Mot	Cog	Acc	RT	Rep	Lang	Lat	
Weiller et al. (1995): Contrast 1	Verb generation	Rest	Y	N	Y	N	NANC	NANC	S	Y	Y	L posterior temporal, IFG and ventral precentral gyrus, much smaller activations in the R hemisphere
Weiller et al. (1995): Contrast 2	Pseudoword repetition	Rest	Y	N	Y	N	NANC	NANC	S	S	S	L posterior temporal only; similar but less extensive activation in the R hemisphere
Belin et al. (1996): Contrast 1	Word repetition with MIT-like intonation	Word repetition	Y	Y	Y	Y	NBD	UNR	N/A	N/A	N/A	
Ohyama et al. (1996): Contrast 1	Word repetition	Rest	Y	N	N	N	NANC	NANC	S	S	N	Bilateral auditory and motor activations are prominent, only slightly L-lateralized
Heiss et al. (1997): Contrast 1	Word repetition	Rest	Y	N	N	N	NANC	NANC	S	U	N	The only control data is extent of activation and mean signal increase in L and R superior temporal cortex; both of these measures were slightly L-lateralized
Karbe et al. (1998): Contrast 1	Word repetition	Rest	Y	N	N	N	NANC	NANC	S	N	N	ROIs only; negligible evidence of lateralization
Cao et al. (1999): Contrast 1	Picture naming	Viewing nonsense drawings	Y	Y	Y	N	NANC	NANC	S	U	S	Insufficient data to assess the control activation pattern
Heiss et al. (1999): Contrast 1	Noun repetition	Rest	Y	N	N	N	NANC	NANC	S	S	S	L frontal and bilateral temporal
Kessler et al. (2000): Contrast 1	Word repetition	Rest	Y	N	N	N	NANC	NANC	N	U	U	No control data are reported or cited, however the same task was used in several previous studies by this group
Rosen et al. (2000): Contrast 1	Word stem completion (PET)	Rest (PET)	N	N	N	N	NANC	NANC	S	S	Y	L IFG, L ITG, L anterior fusiform
Rosen et al. (2000): Contrast 2	Word stem completion (fMRI)	Rest (fMRI)	N	Y	Y	N	NANC	NANC	S	S	Y	L IFG, L intraparietal sulcus
Blasi et al. (2002): Contrast 1	Word stem completion (novel items)	Rest	N	Y	Y	N	NANC	NANC	Y	S	S	Activation of language areas but also other areas; frontal activation is somewhat lateralized
Blasi et al. (2002): Contrast 2	Word stem completion (novel items)	Word stem completion (repeated items)	Y	Y	Y	Y	Y	N	S	U	S	No whole brain analysis of this contrast, but somewhat lateralized in the sense that L but not R frontal areas showed a learning effect
Leff et al. (2002): Contrast 1	Higher word rates	Lower word rates	Y	N	Y	Y	NANB	NANT	S	S	S	Control activation is bilateral in primary auditory cortex and the lateral STG (Fig. 1, labels 1 and 2), but there is a left-lateralized activation in the pSTS (label 3); the scatter plots in Fig. 1 show activity-word rate curves for peak pSTS voxels in individual subjects; slopes were steeper in the left hemisphere ($p < 0.05$), however,

												the identification of these voxels is not described in sufficient detail (i.e. what was the search region?)
Blank et al. (2003): Contrast 1	Propositional speech production	Rest	Y	N	N	N	NANC	NANC	Y	S	S	Much bilateral activation due to overt speech but pars opercularis and supratemporal plane L-lateralized
Blank et al. (2003): Contrast 2	Propositional speech production	Counting	Y	Y	Y	N	NANC	NANC	Y	S	S	Extrasylvian; somewhat L-lateralized
Cardebat et al. (2003): Contrast 1	Word generation	Rest	Y	N	N	N	NANC	NANC	S	S	N	Bilateral fronto-temporal and some other regions per text
Sharp et al. (2004): Contrast 1	Semantic decision	Syllable count decision	Y	Y	Y	Y	N	N	S	S	Y	The control data provided also include the noise vocoded conditions; only ventral temporal activations are shown, which are L-lateralized
Zahn et al. (2004): Contrast 1	Semantic decision	Phonetic decision and lexical decision (conjunction)	Y	Y	Y	Y	AS	UNR	Y	Y	Y	L-lateralized frontal activation, as well as temporal and parietal to a lesser extent; conjunction of baseline conditions not described in sufficient detail
Crinion & Price (2005): Contrast 1	Listening to narrative speech	Listening to reversed speech	Y	Y	Y	Y	NANB	NANT	Y	Y	S	Bilateral (L > R) temporal, L IFG and L dorsal precentral
de Boissezon et al. (2005): Contrast 1	Word generation	Rest	Y	N	N	N	NANC	NANC	N	U	U	
Connor et al. (2006): Contrast 1	Word stem completion (novel items)	Word stem completion (repeated items)	Y	Y	Y	Y	Y	N	S	U	S	No whole brain analysis of this contrast, but somewhat lateralized in the sense that L but not R frontal areas showed a learning effect; the only contrast analyzed in this paper is the "learning" contrast which corresponds to contrast 2 in Blasi et al. (2002)
Crinion et al. (2006): Contrast 1	Listening to narrative speech	Listening to reversed speech	Y	Y	Y	Y	NANB	NANT	S	Y	S	11 participants; L-lateralized posterior temporal, bilateral anterior temporal, no frontal
Saur et al. (2006): Contrast 1	Listening to sentences and making a plausibility judgment	Listening to reversed speech	Y	Y	N	N	UNR	UNR	Y	Y	Y	L temporal and L > R frontal
Meinzer et al. (2008): Contrast 1	Picture naming (trained items)	Rest	N	N	N	N	NANC	NANC	N	U	U	
Meinzer et al. (2008): Contrast 2	Picture naming (untrained items)	Rest	N	N	N	N	NANC	NANC	N	U	U	
Raboyeau et al. (2008): Contrast 1	Picture naming (native in patients; relearned foreign in controls)	Rest	N	N	N	N	NANC	NANC	N	U	U	Presumably only the relearned foreign condition was used in controls (not the native condition), but this is not stated explicitly
Richter et al. (2008): Contrast 1	Reading words silently	Rest	N	Y	Y	N	NANC	NANC	S	U	U	Appears to be somewhat L-lateralized frontal, but not well visualized
Richter et al.	Word stem	Rest	N	Y	Y	N	NANC	NANC	S	U	N	Bilateral frontal; other regions not

(2008): Contrast 2	completion											well visualized
de Boissezon et al. (2009): Contrast 1	Word generation	Rest	Y	N	N	N	NANC	NANC	S	S	N	Control data in Cardebat et al. (2003); bilateral fronto-temporal and some other regions per text
Fridriksson et al. (2009): Contrast 1	Picture naming (correct trials)	Viewing scrambled images	Y	N	N	N	NANC	NANC	S	N	S	Control data in Fridriksson et al. (2007); motor activations are prominent; there is some L frontal activation but little temporal activation in either hemisphere
Fridriksson et al. (2009): Contrast 2	Picture naming (phonemic paraphasias)	Picture naming (correct trials)	Y	Y	Y	Y	NBD	UNR	N/A	N/A	N/A	Control data N/A because controls do not typically make errors
Fridriksson et al. (2009): Contrast 3	Picture naming (semantic paraphasias)	Picture naming (correct trials)	Y	Y	Y	Y	NBD	UNR	N/A	N/A	N/A	Control data N/A because controls do not typically make errors
Menke et al. (2009): Contrast 1	Picture naming (trained items)	Rest	N	N	N	N	NANC	NANC	S	U	U	Table of coordinates only
Menke et al. (2009): Contrast 2	Picture naming (untrained items)	Rest	N	N	N	N	NANC	NANC	S	U	U	Table of coordinates only
Specht et al. (2009): Contrast 1	Lexical decision (words vs pseudowords)	Lexical decision (words vs reversed foreign words)	Y	Y	Y	Y	UNR	UNR	Y	S	Y	The contrast activated a ventral part of the L IFG, along with L anterior cingulate and L DLPFC
Warren et al. (2009): Contrast 1	Listening to narrative speech	Listening to reversed speech	Y	Y	Y	Y	NANB	NANT	S	Y	S	11 participants; L-lateralized posterior temporal, bilateral anterior temporal, no frontal
Chau et al. (2010): Contrast 1	Answering questions from Cantonese Aphasia Battery	Visual decision	N	N	Y	N	NANC	NANC	N	U	U	
Fridriksson (2010): Contrast 1	Picture naming (correct trials)	Viewing abstract pictures	Y	N	N	N	NANC	NANC	S	N	S	Control data in Fridriksson et al. (2007); motor activations are prominent; there is some L frontal activation but little temporal activation in either hemisphere.
Fridriksson et al. (2010): Contrast 1	Picture naming (correct trials)	Viewing abstract pictures	Y	N	N	N	NANC	NANC	S	S	S	L-lateralized frontal and temporal activations, but also bilateral visual, motor and auditory
Sharp et al. (2010): Contrast 1	Semantic decision (clear in patients; average of clear and noise vocoded in controls)	Syllable count decision (clear in patients; average of clear and noise vocoded in controls)	Y	Y	Y	Y	N	N	S	S	Y	Not stated exactly what contrast was used in controls
Thompson et al. (2010): Contrast 1	Auditory sentence-picture matching (all three sentence types)	Rest	N	N	N	N	NANC	NANC	N	U	U	
Tyler et al. (2010): Contrast 1	Listening to grammatical but meaningless sentences and	Listening to scrambled sentences and detecting a target word	Y	Y	Y	Y	UNR	AS	S	Y	N	There are more control participants in another paper (Tyler et al., 2010, Cereb Cortex), but the relevant contrast does not seem to be shown in that paper; the contrast is intended to

	detecting a target word												identify regions involved in syntactic processing, however it seems possible that there are semantic differences between these conditions also
van Oers et al. (2010): Contrast 1	Written word-picture matching	Visual decision	N	Y	Y	N	UNR	UNR	Y	S	S		Not clearly stated that language tasks were contrasted only with arrow decision task and not rest for the first two contrasts, but this can be inferred
van Oers et al. (2010): Contrast 2	Semantic decision	Visual decision	N	Y	Y	N	UNR	UNR	Y	S	S		Not clearly stated that language tasks were contrasted only with arrow decision task and not rest for the first two contrasts, but this can be inferred
van Oers et al. (2010): Contrast 3	Verb generation	Rest	N	Y	Y	N	NANC	NANC	Y	S	S		
Papoutsi et al. (2011): Contrast 1	Listening to ambiguous sentences with subordinate resolution ("subordinate")	Listening to ambiguous sentences with dominant resolution ("dominant")	Y	Y	Y	Y	NANB	NANT	Y	Y	Y		Control data in Tyler et al. (2011); L frontal and temporal
Sebastian & Kiran (2011): Contrast 1	Picture naming (correct trials)	Viewing scrambled images and saying "pass"	Y	Y	Y	N	UNR	UNR	S	S	N		Reporting is selective, but appears mostly bilateral with slight L-lateralization of language areas
Sebastian & Kiran (2011): Contrast 2	Semantic decision (correct trials)	Visual decision	Y	Y	Y	Y	UNR	UNR	S	S	Y		Clearly lateralized frontal activation, but very modest temporal activation
Szaflarski et al. (2011): Contrast 1	Semantic decision	Tone decision	Y	Y	Y	Y	AS	UNR	Y	Y	Y		Control data in Kim et al. (2011) and Szaflarski et al. (2008); L frontal and temporal, plus other semantic regions
Tyler et al. (2011): Contrast 1	Listening to ambiguous sentences (dominant and subordinate)	Listening to unambiguous sentences ("unambiguous")	Y	Y	Y	Y	NANB	NANT	Y	S	Y		L frontal and parietal; R frontal (but L > R); no L temporal
Tyler et al. (2011): Contrast 2	Listening to ambiguous sentences with dominant resolution ("dominant")	Listening to unambiguous sentences ("unambiguous")	Y	Y	Y	Y	NANB	NANT	Y	S	Y		L frontal and parietal; no L temporal
Tyler et al. (2011): Contrast 3	Listening to ambiguous sentences with subordinate resolution ("subordinate")	Listening to unambiguous sentences ("unambiguous")	Y	Y	Y	Y	NANB	NANT	Y	Y	Y		L frontal, temporal and parietal, R frontal (but L > R)
Tyler et al. (2011): Contrast 4	Listening to ambiguous sentences with subordinate resolution ("subordinate")	Listening to ambiguous sentences with dominant resolution ("dominant")	Y	Y	Y	Y	NANB	NANT	Y	Y	Y		L frontal and temporal
Weiduschat et al. (2011): Contrast 1	Verb generation	Rest	Y	N	Y	N	NANC	NANC	S	U	U		Control data in Herholz et al. (1996); insufficient to fully validate the contrast

Allendorfer et al. (2012): Contrast 1	Verb generation (covert, block)	Finger tapping (block)	Y	Y	N	N	NANC	NANC	Y	Y	Y	Strongly lateralized frontal and temporal activation
Allendorfer et al. (2012): Contrast 2	Verb generation (overt, event-related)	Noun repetition (event-related)	Y	Y	Y	N	AM	UNR	Y	S	S	Somewhat L-lateralized frontal, temporal and parietal activations, but also extensive midline activation
Allendorfer et al. (2012): Contrast 3	Verb generation (overt, event-related)	Verb generation (covert, event-related)	Y	N	N	Y	NANC	NANC	Y	S	N/A	Bilateral speech motor activations, but also extensive midline activation
Fridriksson, Hubbard, et al. (2012): Contrast 1	Listening to/watching audiovisual sentences, while producing the same sentences in unison (speech entrainment)	Listening to reversed sentences and viewing a mouth speaking, while producing unrelated sentences	Y	Y	Y	Y	UNR	UNR	S	N	N	Control and patient data are combined; this contrast activates bilateral anterior insula and posterior MTG, slightly more extensive on the L
Fridriksson, Hubbard, et al. (2012): Contrast 2	Listening to/watching audiovisual sentences, while producing the same sentences in unison (speech entrainment)	Rest	N	N	N	N	NANC	NANC	N	U	U	Rest condition implied but not explicitly described
Fridriksson, Hubbard, et al. (2012): Contrast 3	Listening to reversed sentences and viewing a mouth speaking, while producing unrelated sentences	Rest	N	N	N	N	NANC	NANC	N	U	U	Rest condition implied but not explicitly described
Fridriksson, Hubbard, et al. (2012): Contrast 4	Listening to/watching audiovisual sentences and viewing a mouth	Rest	N	N	N	N	NANB	NANT	N	U	U	Rest condition implied but not explicitly described
Fridriksson, Richardson, et al. (2012): Contrast 1	Picture naming	Viewing abstract pictures	Y	N	N	N	NANC	NANC	S	N	S	Control data in Fridriksson et al. (2007); motor activations are prominent; there is some L frontal activation but little temporal activation in either hemisphere
Marcotte et al. (2012): Contrast 1	Picture naming (T1: known items; T2: trained items; correct trials)	Viewing scrambled images and saying "baba"	Y	Y	Y	N	YCT	UNR	N	U	U	Different contrasts at different time points not clearly explained
Marcotte et al. (2012): Contrast 2	Picture naming (known items, correct trials)	Viewing scrambled images and saying "baba"	Y	Y	Y	N	YCT	UNR	N	U	U	Different contrasts at different time points not clearly explained
Marcotte et al. (2012): Contrast 3	Picture naming (trained items, correct trials)	Viewing scrambled images and saying "baba"	Y	Y	Y	N	YCT	UNR	N	U	U	Different contrasts at different time points not clearly explained

Schofield et al. (2012): Contrast 1	Listening to word pairs or reversed word pairs, speaker gender judgment	Rest	N	N	N	N	NANC	NANC	Y	N	N	Control data in Leff et al. (2008); auditory contrast, not intended to be language contrast
Schofield et al. (2012): Contrast 2	Listening to word pairs, speaker gender judgment	Listening to reversed word pairs, speaker gender judgment	Y	Y	Y	Y	UNR	UNR	Y	S	Y	Control data in Leff et al. (2008); L-lateralized activation of posterior STS
Wright et al. (2012): Contrast 1	Listening to normal sentences and detecting a target word	Rest	N	N	N	N	NANC	NANC	Y	N	N	Bilateral superior temporal, sensorimotor and visual
Wright et al. (2012): Contrast 2	Listening to grammatical but meaningless sentences and detecting a target word	Rest	N	N	N	N	NANC	NANC	N	U	U	
Szaflarski et al. (2013): Contrast 1	Semantic decision	Tone decision	Y	Y	Y	Y	AM	UNR	Y	Y	Y	Control data in Kim et al. (2011) and Szaflarski et al. (2008); L frontal and temporal, plus other semantic regions
Thiel et al. (2013): Contrast 1	Verb generation	Rest	Y	N	N	N	NANC	NANC	S	U	U	Cites Weiduschat et al. (2011) which in turn cites Herholz et al. (1996) which provides some minimal control data
Abel et al. (2014): Contrast 1	Picture naming (all conditions)	Rest	N	N	N	N	NANC	NANC	N	U	U	But see control data reported in a subsequent paper (Abel et al., 2015)
Abel et al. (2014): Contrast 2	Picture naming (trained items)	Picture naming (untrained items)	Y	Y	Y	Y	N	UNR	N	U	U	
Abel et al. (2014): Contrast 3	Picture naming (semantic trained items)	Picture naming (phonological trained items)	Y	Y	Y	Y	Y	UNR	N	U	U	
Benjamin et al. (2014): Contrast 1	Word generation	Rest	N	N	N	N	NANC	NANC	N	U	U	Contrast not described explicitly but there is only one possible contrast
Brownsett et al. (2014): Contrast 1	Listening to sentences	Listening to segmented white noise	Y	Y	Y	Y	NANB	NANT	N	U	U	
Brownsett et al. (2014): Contrast 2	Listening to sentences (patients) or listening to noise vocoded sentences (controls)	Listening to segmented white noise	Y	Y	Y	Y	NANB	NANT	N	U	U	
Mattioli et al. (2014): Contrast 1	Listening to sentences and making a plausibility judgment	Listening to reversed speech	Y	Y	N	N	NANC	NANC	S	S	Y	10 participants; quite lateralized activity centered on the anterior Sylvian fissure; it is mentioned that "noise" was also included on the negative side of the contrast; it is unclear if this refers to the

												noise "bip", which would be inappropriate
Mohr et al. (2014): Contrast 1	Listening to sentences (high and low ambiguity)	Listening to signal-correlated noise	Y	Y	Y	Y	NANB	NANT	N	U	U	Some control data in Rodd et al. (2005), but half of the participants were performing a probe judgment task, unlike in the present study
Mohr et al. (2014): Contrast 2	Listening to high ambiguity sentences	Listening to low ambiguity sentences	Y	Y	Y	Y	NANB	NANT	N	U	U	Some control data in Rodd et al. (2005), but half of the participants were performing a probe judgment task, unlike in the present study
Robson et al. (2014): Contrast 1	Semantic decision (written word and picture)	Visual decision and rest	N	Y	N	N	NANC	NANC	S	S	N	Control data are provided in Table 6 for contrasts of written word semantic decision vs dual baseline, and picture semantic decision vs dual baseline, but not for the main effect of semantic decision; these data suggest that the contrast activates ventral temporal regions bilaterally; two contrasts are described: (1) written word judgment versus a dual baseline of visual judgment and rest; (2) picture judgment versus a dual baseline of visual judgment and rest; these two primary contrasts are reported in patients and controls separately, but no between-group contrasts are reported, so these contrasts are excluded from our review; rather, the between-groups analyses in the paper take the form of ANOVAs; the main effect of group in these ANOVAs collapses across the two described contrasts, therefore we have coded the contrast as the average of the two described contrasts; the exact nature of the computation of dual baseline contrasts is not described
Szaflarski et al. (2014): Contrast 1	Verb generation	Finger tapping	Y	Y	N	N	NANC	NANC	Y	Y	S	Control data in Szaflarski et al. (2008); frontal activation L-lateralized, temporal less so
van Hees et al. (2014): Contrast 1	Picture naming (phonological trained items, correct trials)	Viewing scrambled images	Y	N	N	N	NANC	NANC	S	U	U	Control data are described for naming untrained items; the data are reported only briefly in the text; it is notable that no speech motor, visual, or auditory activations are reported, as might be expected in a picture naming task; correct and incorrect trials were apparently modeled separately, but this is not clearly stated, nor are the criteria for deciding whether trials were correct; it is generally not clear which contrasts exactly were run
van Hees et al.	Picture naming	Viewing	Y	N	N	N	NANC	NANC	S	U	U	Control data are described for

(2014): Contrast 2	(semantic trained items, correct trials)	scrambled images										naming untrained items; the data are reported only briefly in the text; it is notable that no speech motor, visual, or auditory activations are reported, as might be expected in a picture naming task; correct and incorrect trials were apparently modeled separately, but this is not clearly stated, nor are the criteria for deciding whether trials were correct; it is generally not clear which contrasts exactly were run
Abel et al. (2015): Contrast 1	Picture naming	Rest	N	N	N	N	NANC	NANC	Y	N	N	Bilateral somato-motor, auditory and to a lesser extent higher level visual regions; finite impulse analysis only
Kiran et al. (2015): Contrast 1	Picture naming (trained)	Viewing scrambled images and saying "skip"	Y	Y	Y	N	UNR	UNR	S	N	S	Overlap of individual participant activation maps; somewhat lateralized frontal and temporal, but also bilateral occipito-temporal
Kiran et al. (2015): Contrast 2	Semantic feature decision	Visual decision	Y	Y	Y	N	UNR	UNR	S	N	S	Overlap of individual participant activation maps; somewhat lateralized frontal and temporal, but also bilateral occipito-temporal; this contrast inferred but not described
Sandberg et al. (2015): Contrast 1	Concreteness judgment (abstract words, correct trials)	Rest	N	Y	N	N	NANC	NANC	N	U	U	The concreteness judgment task was compared to the letter string judgment task to define ROIs for connectivity analysis, but the group analysis meeting criteria for this review appears to be based only on comparisons between time points on the concreteness judgment conditions
Sandberg et al. (2015): Contrast 2	Concreteness judgment (concrete words, correct trials)	Rest	N	Y	N	N	NANC	NANC	N	U	U	The concreteness judgment task was compared to the letter string judgment task to define ROIs for connectivity analysis, but the group analysis meeting criteria for this review appears to be based only on comparisons between time points on the concreteness judgment conditions
Geranmayeh et al. (2016): Contrast 1	Propositional speech production	Rest	N	N	N	N	NANC	NANC	S	S	N	Control data for univariate analysis in Geranmayeh et al. (2014), but note that the present paper does not describe a univariate analysis; control activations reflect speech rather than language
Geranmayeh et al. (2016): Contrast 2	Propositional speech production	Counting	N	Y	Y	N	NANC	NANC	S	Y	Y	Control data for univariate analysis in Geranmayeh et al. (2014), but note that the present paper does not describe a univariate analysis; control

												activations are L frontal, L pSTS, L SMA, L > R occipito-temporal
Geranmayeh et al. (2016): Contrast 3	Propositional speech production	Target decision	N	N	N	N	NANC	NANC	N	U	U	
Griffis et al. (2016): Contrast 1	Verb generation	Finger tapping	Y	Y	N	N	NANC	NANC	Y	Y	S	Control data in Szaflarski et al. (2008); frontal activation L-lateralized, temporal less so
Sims et al. (2016): Contrast 1	Semantic feature decision (6 patients, 4 controls) or semantic relatedness decision (8 patients, 4 controls)	Visual decision or pseudoword identity decision	Y	Y	Y	Y	N	UNR	N	U	U	8 patients and 4 controls performed one paradigm, while 6 patients and 4 controls performed another; the data were combined based on the assumption that similar processes were implicated by the two contrasts
Darkow et al. (2017): Contrast 1	Picture naming	Rest	N	N	N	N	NANC	NANC	N	U	U	
Geranmayeh et al. (2017): Contrast 1	Propositional speech production	Rest	N	N	N	N	NANC	NANC	Y	N	N	Control data in Geranmayeh et al. (2014); speech not language; relevant activations are bilateral; not entirely clear that the whole brain analysis is indeed propositional speech production vs rest; a contrast of target decision vs mean of propositional speech and counting is also used to define the preSMA/dACC ROI
Griffis, Nenert, Allendorfer, & Szaflarski (2017): Contrast 1	Semantic decision	Tone decision	Y	Y	Y	Y	UNR	UNR	Y	Y	Y	Temporal activation is mid MTG and AG rather than pSTS
Griffis, Nenert, Allendorfer, Vannest, et al. (2017): Contrast 1	Semantic decision	Tone decision	Y	Y	Y	Y	UNR	UNR	Y	Y	Y	Temporal activation is mid MTG and AG rather than pSTS
Harvey et al. (2017): Contrast 1	Picture naming	Viewing patterns	Y	N	N	N	NANC	NANC	N	U	U	
Nardo et al. (2017): Contrast 1	Picture naming (all conditions, correct trials)	Rest	N	N	N	N	NANC	NANC	N	U	U	It is difficult to determine exactly what contrasts were employed
Nardo et al. (2017): Contrast 2	Picture naming (untrained items, no cue, correct trials)	Picture naming (trained items, no cue, correct trials)	Y	Y	Y	Y	YCT	N	N	U	U	It is difficult to determine exactly what contrasts were employed
Nenert et al. (2017): Contrast 1	Semantic decision	Tone decision	Y	Y	Y	Y	AM	UNR	Y	Y	Y	Lateralized frontal, temporal, and parietal
Nenert et al. (2017): Contrast 2	Verb generation	Finger tapping	Y	Y	N	N	NANC	NANC	Y	Y	S	Control data in Szaflarski et al. (2008); frontal activation L-lateralized, temporal less so
Qiu et al. (2017): Contrast 1	Picture naming	Rest	N	N	N	N	NANC	NANC	S	N	S	Somewhat L-lateralized frontal and anterior temporal language activations, but the majority of activation is in unexpected regions
Skipper-Kallal et al. (2017a): Contrast 1	Picture naming (silently name, correct trials)	Rest	N	Y	Y	N	NANC	NANC	Y	N	N	Bilateral frontal and occipito-temporal, but not posterior temporal
Skipper-Kallal et	Picture naming	Rest	N	N	N	N	NANC	NANC	Y	N	N	Bilateral frontal and occipito-

al. (2017a): Contrast 2	(produce the name, correct trials)										temporal, but not posterior temporal; speech motor activation not readily apparent
Skipper-Kallal et al. (2017a): Contrast 3	Picture naming (both phases, correct trials)	Picture naming (both phases, incorrect trials)	Y	U	U	Y	NBD	UNR	N/A	N/A	N/A	Control data N/A because controls do not typically make errors; it is unclear whether there were no-response trials and whether they were modeled as incorrect
Skipper-Kallal et al. (2017b): Contrast 1	Picture naming (prepare to name, correct trials)	Rest	N	Y	Y	N	NANC	NANC	Y	N	N	Bilateral frontal and occipito-temporal, but not posterior temporal
Skipper-Kallal et al. (2017b): Contrast 2	Picture naming (produce the name, correct trials)	Rest	N	N	N	N	NANC	NANC	Y	N	N	Bilateral frontal and occipito-temporal, but not posterior temporal; speech motor activation not readily apparent
Dietz et al. (2018): Contrast 1	Verb generation (overt)	Noun repetition	Y	Y	Y	N	UNR	UNR	Y	S	S	Control data in Allendorfer et al. (2012); somewhat L-lateralized frontal, temporal and parietal activations, but also extensive midline activation
Hallam et al. (2018): Contrast 1	Listening to high or low ambiguity sentences	Listening to spectrally rotated speech	Y	Y	Y	Y	NANB	NANT	S	U	U	Hard to evaluate contrast because a "semantic mask" is used but is not described in detail
Hallam et al. (2018): Contrast 2	Listening to high ambiguity sentences	Listening to low ambiguity sentences	Y	Y	Y	Y	NANB	NANT	N	U	U	
Nenert et al. (2018): Contrast 1	Semantic decision	Tone decision	Y	Y	Y	Y	AM	UNR	Y	Y	Y	L lateral and medial frontal and AG, strongly lateralized
Nenert et al. (2018): Contrast 2	Verb generation	Finger tapping	Y	Y	N	N	NANC	NANC	Y	Y	Y	L lateral and medial frontal and mid temporal, strongly lateralized
Pillay et al. (2018): Contrast 1	Reading nouns aloud (correct trials)	Reading nouns aloud (incorrect trials)	Y	Y	Y	Y	NBD	Y	N/A	N/A	N/A	Control data N/A because controls do not typically make errors
Szaflarski et al. (2018): Contrast 1	Semantic decision	Tone decision	Y	Y	Y	Y	UNR	UNR	Y	Y	Y	L frontal and temporal, plus other semantic regions
van de Sandt-Koenderman et al. (2018): Contrast 1	Listening to narrative speech	Listening to reversed speech	Y	Y	Y	Y	NANB	NANT	N	U	U	
van Oers et al. (2018): Contrast 1	Written word-picture matching	Visual decision	N	Y	Y	N	UNR	UNR	S	N	S	Primarily bilateral visual activations; frontal activation is L-lateralized
van Oers et al. (2018): Contrast 2	Semantic decision	Visual decision	N	Y	Y	N	UNR	UNR	S	S	Y	L frontal, L posterior ITG, L superior parietal
Barbieri et al. (2019): Contrast 1	Auditory sentence-picture verification	Listening to reversed speech and viewing scrambled pictures	Y	Y	Y	N	UNR	UNR	Y	S	S	L-lateralized inferior frontal and posterior temporal, but also bilateral posterior inferior temporal and lateral occipital activations; contrast described as "passive > control" but seems to involve active and passive sentences
Johnson et al. (2019): Contrast 1	Picture naming (trained items)	Rest	N	N	N	N	NANC	NANC	S	N	N	Most ROIs deactivated in controls
Kristinsson et al. (2019): Contrast 1	Picture naming	Viewing abstract pictures	Y	N	N	N	NANC	NANC	N	U	U	

Purcell et al. (2019): Contrast 1	Spelling probe (training items)	Rest	N	N	N	N	NANC	NANC	N	U	U	Task comes from Rapp and Lipka (2011), who report lateralized activations for the contrast of spelling probes to case verification, but do not report results relative to fixation baseline
Purcell et al. (2019): Contrast 2	Spelling probe (known items)	Rest	N	N	N	N	NANC	NANC	N	U	U	Task comes from Rapp and Lipka (2011), who report lateralized activations for the contrast of spelling probes to case verification, but do not report results relative to fixation baseline
Sreedharan, Chandran, et al. (2019): Contrast 1	Neurofeedback (try to activate language areas)	Rest	N	Y	Y	N	NANC	NANC	S	U	N	Task activated L IFG and L STG in controls (Fig. 8c), but no data on other regions, and language activations were not lateralized (Fig. 9d)
Hartwigsen et al. (2020): Contrast 1	Syllable count decision	Rest	Y	N	N	N	NANC	NANC	Y	Y	S	Control data in Hartwigsen et al. (2017); L-lateralized IFG but bilateral SMG
Hartwigsen et al. (2020): Contrast 2	Semantic decision	Rest	Y	N	N	N	NANC	NANC	Y	Y	Y	Control data in Hartwigsen et al. (2017); L-lateralized IFG and AG most prominent
Stockert et al. (2020): Contrast 1	Listening to normal sentences and making a plausibility judgment (paradigm 1) or listening to normal sentences (paradigm 2)	Listening to reversed speech	Y	Y	N	N	UNR	NANC	S	Y	Y	Not stated which of the two paradigms controls were run on, but clearly L-lateralized frontal and temporal activation; bilateral MD network activation also noted; 20 patients performed paradigm 1 and 14 patients performed paradigm 2; data were combined despite some differences; unclear whether all reversed speech was included, or only reversed speech derived from plausible sentences

Vis = Are the language and control conditions matched for visual demands?; Aud = Are the language and control conditions matched for auditory demands?; Mot = Are the language and control conditions matched for motor demands?; Cog = Are the language and control conditions matched for cognitive demands?; Acc = Is accuracy matched between the language and control tasks for all groups at all time points?; RT = Is reaction time matched between the language and control tasks for all groups at all time points?; Rep = Are control data reported in the paper, or in a previous publication that is cited?; Lang = Does the contrast selectively activate plausible relevant language regions in neurologically normal individuals?; Lat = Are activations lateralized in neurologically normal individuals?; AG = angular gyrus; AM = Appear mismatched; ANOVA = analysis of variance; AS = Appear similar; C = Accuracy or RT is covariate; DLPFC = dorsolateral prefrontal cortex; fMRI = functional magnetic resonance imaging; IFG = inferior frontal gyrus; ITG = inferior temporal gyrus; L = left; MIT = melodic intonation therapy; MTG = middle temporal gyrus; N = No; N/A = not applicable; NAM = No, but attempt made; NANB = Not applicable, no behavioral measure; NANC = Not applicable, tasks not comparable.; NANT = Not applicable, no timeable task; NBD = No, by design; PET = positron emission tomography; pSTS = posterior superior temporal sulcus; R = right; ROI = region of interest; S = Somewhat; SMA = supplementary motor area; STG = superior temporal gyrus; STS = superior temporal sulcus; T1, T2, etc. = first time point, second time point, etc.; U = Unknown; UNR = Unknown, not reported; UNT = Unknown, no test; Y = Yes; YCT = Yes, correct trials only; Yellow underline = minor limitation; Orange underline = moderate limitation; Red underline = major limitation.

Supplementary Table S10. All analyses

Analysis	First level contrast	Second level contrast	Matched for Acc	Matched for RT	Stats	Notes	Findings
Weiller et al. (1995): Vox 1	Verb generation vs rest	CAC<br>Aphasia vs control	AM	UNR	Vox<br>NDC	Behavioral data notes: in practice trials, patients produced 1.5 words on average per prompt, not all of which were verbs, while controls 2.3 words on average per prompt, almost all of which were verbs; search volume: perisylvian; software: SPM; qualitative comparison on p. 729 (the word "significant" is used)	↑ R IFG<br>↑ R posterior STG/STS/MTG<br>↓ L posterior STG/STS/MTG<br>notes: based more on Figure 2 than the text
Weiller et al. (1995): Vox 2	Pseudoword repetition vs rest	CAC<br>Aphasia vs control	AS	UNR	Vox<br>NDC	Behavioral data notes: all participants are reported to have had no difficulties in performing the repetition task; search volume: perisylvian; software: SPM; qualitative comparison on p. 729 (the word "significant" is used)	↑ L ventral precentral/inferior frontal junction<br>↑ R IFG<br>↑ R posterior STG/STS/MTG<br>↓ L posterior STG/STS/MTG<br>notes: based more on Figure 2 than the text
Belin et al. (1996): ROI 1	Word repetition with MIT-like intonation vs word repetition	CB<br>Aphasia	NBD	UNR	ROI<br>Anat<br>NC	Behavioral data notes: more words were correctly repeated with MIT (16.3 ± 8) than without (12.4 ± 8; p < 0.03); number of ROIs: 18; ROIs: (1) L Broca's area; (2) L prefrontal; (3) L sensorimotor mouth; (4) L parietal; (5) L Wernicke's area; (6) L Heschl's gyrus; (7) L anterior STG; (8) L MTG; (9) L temporal pole; (10-18) homotopic counterparts; how ROIs defined: individual anatomical images; activation quantified as mean rCBF, not including any intersection of the infarct with the ROI; three left hemisphere ROIs were excluded (3, 6, 9) because they were completely infarcted in 4 or more patients	↑ L IFG<br>↑ L dorsolateral prefrontal cortex<br>↓ R posterior STG
Ohyama et al. (1996): ROI 1	Word repetition vs rest	CAC<br>Aphasia vs control	UNR	UNR	ROI<br>Func<br>NC	Behavioral data notes: some of the patients made a few errors, so as a group they may have been less accurate than controls; number of ROIs: 7; ROIs: (1) L posterior inferior frontal; (2) R posterior inferior frontal; (3) L posterior superior temporal; (4) R posterior superior temporal; (5) L rolandic; (6) R rolandic; (7) SMA; how ROIs defined: spheres around control peaks; the rCBF increase in R PIF was also significant at p < 0.005 for nonfluent patients with Fisher's protected least-significant difference	↑ R IFG<br>↑ R posterior STG/STS/MTG
Ohyama et al. (1996): ROI 2	Word repetition vs rest	CAA<br>Aphasia fluent (n = 10) vs non-fluent (n = 6)	UNR	UNR	ROI<br>Func<br>NC	Number of ROIs: 7; ROIs: (1) L posterior inferior frontal; (2) R posterior inferior frontal; (3) L posterior superior temporal; (4) R posterior superior temporal; (5) L	↓ R IFG

							rolandic; (6) R rolandic; (7) SMA; how ROIs defined: spheres around control peaks	
Ohyama et al. (1996): ROI 3	Word repetition vs rest	CC<br>Aphasia<br>Covariate: spontaneous speech (WAB)	UNR	UNR	ROI<br>Func<br>NC	Number of ROIs: 7; ROIs: (1) L posterior inferior frontal; (2) R posterior inferior frontal; (3) L posterior superior temporal; (4) R posterior superior temporal; (5) L rolandic; (6) R rolandic; (7) SMA; how ROIs defined: spheres around control peaks; no correction for multiple comparisons across WAB subscores	↑ L IFG
Ohyama et al. (1996): ROI 4	Word repetition vs rest	CC<br>Aphasia<br>Covariate: comprehension (WAB)	UNR	UNR	ROI<br>Func<br>NC	Number of ROIs: 7; ROIs: (1) L posterior inferior frontal; (2) R posterior inferior frontal; (3) L posterior superior temporal; (4) R posterior superior temporal; (5) L rolandic; (6) R rolandic; (7) SMA; how ROIs defined: spheres around control peaks; this non-significant finding is implied but not stated explicitly	None
Ohyama et al. (1996): ROI 5	Word repetition vs rest	CC<br>Aphasia<br>Covariate: repetition (WAB)	UNR	UNR	ROI<br>Func<br>NC	Number of ROIs: 7; ROIs: (1) L posterior inferior frontal; (2) R posterior inferior frontal; (3) L posterior superior temporal; (4) R posterior superior temporal; (5) L rolandic; (6) R rolandic; (7) SMA; how ROIs defined: spheres around control peaks; this non-significant finding is implied but not stated explicitly	None
Ohyama et al. (1996): ROI 6	Word repetition vs rest	CC<br>Aphasia<br>Covariate: naming (WAB)	UNR	UNR	ROI<br>Func<br>NC	Number of ROIs: 7; ROIs: (1) L posterior inferior frontal; (2) R posterior inferior frontal; (3) L posterior superior temporal; (4) R posterior superior temporal; (5) L rolandic; (6) R rolandic; (7) SMA; how ROIs defined: spheres around control peaks; this non-significant finding is implied but not stated explicitly	None
Heiss et al. (1997): Vox 1	Word repetition vs rest	LAA<br>(Aphasia with good recovery (n = 3) T2 vs T1) vs (aphasia with poor recovery (n = 3) T2 vs T1)<br>Somewhat valid (TT not optimal measure of overall language function)	UNR	UNR	Vox<br>NDC	Search volume: whole brain; software: not stated; qualitative generalization across individuals on pp. 214-6	↑ L posterior STG/STS/MTG<br>↓ R posterior STG/STS/MTG<br>notes: the consistent aspects of the findings were that there was an emergence of L posterior temporal activation in patients with better recovery, and R posterior temporal activation in patients with worse recovery
Heiss et al. (1997): ROI 1	Word repetition vs rest	LAA<br>(Aphasia with good recovery (n = 3) T2 vs	UNR	UNR	ROI<br>Anat<br>NDC	Number of ROIs: 2; ROIs: (1) L superior temporal cortex; (2) R superior temporal cortex; how ROIs defined:	↑ L posterior STG/STS/MTG<br>↑ L Heschl's gyrus

		T1) vs (aphasia with poor recovery (n = 3) T2 vs T1)<br>Somewhat valid (TT not optimal measure of overall language function)				individual anatomical images; activation quantified in terms of extent exceeding 10% signal change, and mean % increase over the activation; qualitative generalization across individuals on pp. 214, 216	
Karbe et al. (1998): ROI 1	Word repetition vs rest	CAC<br>Aphasia T1 vs control	UNR	UNR	ROI Anat NDC	Number of ROIs: 8; ROIs: (1) L IFG; (2) L STG/HG; (3) L SMA; (4) L ventral precentral; (5-8) homotopic counterparts; how ROIs defined: individual anatomical images; qualitative comparison on p. 219, but only the L SMA comparison is explicitly quantified	↑ L SMA/medial prefrontal<br>↑ R SMA/medial prefrontal<br>↓ L posterior STG<br>↓ L Heschl's gyrus
Karbe et al. (1998): ROI 2	Word repetition vs rest	CC<br>Aphasia (subset who returned for follow-up) T1 (n = 7)<br>Covariate: TT T1<br>Somewhat valid (TT not optimal measure of overall language function)	UNR	UNR	ROI Anat NC	Number of ROIs: 8; ROIs: (1) L IFG; (2) L STG/HG; (3) L SMA; (4) L ventral precentral; (5-8) homotopic counterparts; how ROIs defined: individual anatomical images	None
Karbe et al. (1998): ROI 3	Word repetition vs rest	CC<br>Aphasia (subset who returned for follow-up) T2 (n = 7)<br>Covariate: TT T2<br>Somewhat valid (TT not optimal measure of overall language function)	UNR	UNR	ROI Anat NC	Number of ROIs: 8; ROIs: (1) L IFG; (2) L STG/HG; (3) L SMA; (4) L ventral precentral; (5-8) homotopic counterparts; how ROIs defined: individual anatomical images	↓ L SMA/medial prefrontal<br>↓ R ventral precentral/inferior frontal junction<br>↓ R SMA/medial prefrontal<br>↓ R posterior STG<br>↓ R Heschl's gyrus<br>notes: more activation in patients with more severe aphasia per TT
Karbe et al. (1998): ROI 4	Word repetition vs rest	LC<br>Aphasia (subset who returned for follow-up) (n = 7) T2 vs T1<br>Covariate: subsequent outcome (T2) TT<br>Not valid (the logic behind correlating activation changes and language outcome is unclear; TT not optimal measure of overall language function)	UNR	UNR	ROI Anat One	Number of ROIs: 1; ROI: L STG/HG; how ROI defined: individual anatomical images	↑ L posterior STG<br>↑ L Heschl's gyrus<br>notes: increase in activation for repetition was correlated with better aphasia outcome per TT
Karbe et al. (1998): ROI 5	Word repetition vs rest	CC<br>Aphasia (subset who returned for follow-up) T2 (n = 7)<br>Covariate: previous Δ (T2 vs T1) activation in L STG/HG<br>Not valid (logically problematic because	UNR	UNR	ROI Anat NC	Number of ROIs: 4; ROIs: (1) R IFG; (2) R STG/HG; (3) R SMA; (4) R ventral precentral; how ROIs defined: individual anatomical images	↓ R IFG<br>↓ R ventral precentral/inferior frontal junction<br>↓ R SMA/medial prefrontal<br>↓ R posterior STG<br>↓ R Heschl's gyrus<br>notes: patients

		patients with less severe initial aphasia would also be expected to show little L temporal increase, but would not be expected to show R temporal recruitment)					with more increase in L STG/HG activation showed less activation of R hemisphere regions at T2
Cao et al. (1999): ROI 1	Picture naming vs viewing nonsense drawings	CAC<br>Aphasia vs control	UNR	UNR	ROI<br>Mix<br>NC	Number of ROIs: 6; ROIs: (1) L IFG and MFG; (2) L pSTG, AG and SMG; (3) R IFG and MFG; (4) R pSTG, AG and SMG; (5) frontal LI; (6) temporal LI; how ROIs defined: (1-4) individual anatomical images; activation quantified in terms of extent	↑ R IFG<br>↑ R dorsolateral prefrontal cortex<br>↑ R supramarginal gyrus<br>↑ R angular gyrus<br>↑ R posterior STG<br>↓ LI (frontal)<br>↓ LI (temporal)
Cao et al. (1999): ROI 2	Picture naming vs viewing nonsense drawings	CC<br>Aphasia<br>Covariate: picture naming (outside scanner)	UNR	UNR	ROI<br>Mix<br>NC	Number of ROIs: 6; ROIs: (1) L IFG and MFG; (2) L pSTG, AG and SMG; (3) R IFG and MFG; (4) R pSTG, AG and SMG; (5) frontal LI; (6) temporal LI; how ROIs defined: (1-4) individual anatomical images; activation quantified in terms of extent	↑ LI (frontal)
Heiss et al. (1999): ROI 1	Noun repetition vs rest	LA<br>Aphasia with subcortical damage (n = 9) T2 vs T1	UNR	UNR	ROI<br>Anat<br>NDC	Number of ROIs: 14; ROIs: (1) L IFG pars opercularis; (2) L IFG pars triangularis; (3) L ventral precentral gyrus; (4) L Heschl's gyrus; (5) L temporal plane (posterior to HG, coded as posterior STG); (6) L posterior STG (coded as mid STG per Fig. 2); (7) L SMA; (8-14) homotopic counterparts; how ROIs defined: individual anatomical images; qualitative comparison on p. 434	↑ L mid temporal<br>↑ R Heschl's gyrus<br>↓ R IFG pars opercularis
Heiss et al. (1999): ROI 2	Noun repetition vs rest	LA<br>Aphasia with frontal damage (n = 7) T2 vs T1	UNR	UNR	ROI<br>Anat<br>NDC	Number of ROIs: 14; ROIs: (1) L IFG pars opercularis; (2) L IFG pars triangularis; (3) L ventral precentral gyrus; (4) L Heschl's gyrus; (5) L temporal plane (posterior to HG, coded as posterior STG); (6) L posterior STG (coded as mid STG per Fig. 2); (7) L SMA; (8-14) homotopic counterparts; how ROIs defined: individual anatomical images; qualitative comparison on p. 434	↑ L posterior STG<br>↑ L mid temporal<br>↑ R Heschl's gyrus<br>↓ R IFG pars opercularis
Heiss et al. (1999): ROI 3	Noun repetition vs rest	LA<br>Aphasia with temporal damage (n = 7) T2 vs T1	UNR	UNR	ROI<br>Anat<br>NDC	Number of ROIs: 14; ROIs: (1) L IFG pars opercularis; (2) L IFG pars triangularis; (3) L ventral precentral gyrus; (4) L Heschl's gyrus; (5) L temporal plane (posterior to HG, coded as posterior STG); (6) L posterior STG (coded as mid STG per Fig. 2); (7) L SMA; (8-14) homotopic counterparts; how ROIs defined: individual anatomical images; qualitative comparison on p. 434	↑ L ventral precentral/inferior frontal junction<br>↑ L SMA/medial prefrontal<br>↑ R ventral precentral/inferior frontal junction<br>↑ R mid temporal<br>↓ R SMA/medial prefrontal
Heiss et al. (1999): ROI 4	Noun repetition vs rest	CAA<br>Aphasia with temporal damage T1	UNR	UNR	ROI<br>Anat<br>NDC	Number of ROIs: 14; ROIs: (1) L IFG pars opercularis; (2) L IFG pars triangularis; (3) L ventral precentral	↑ L IFG pars opercularis<br>↑ R SMA/medial

		(n = 7) vs with subcortical damage T1 (n = 9)				gyrus; (4) L Heschl's gyrus; (5) L temporal plane (posterior to HG, coded as posterior STG); (6) L posterior STG (coded as mid STG per Fig. 2); (7) L SMA; (8-14) homotopic counterparts; how ROIs defined: individual anatomical images; qualitative comparison on p. 434	prefrontal<br>↓ L posterior STG<br>↓ R IFG pars opercularis<br>↓ R posterior STG<br>↓ R mid temporal
Heiss et al. (1999): ROI 5	Noun repetition vs rest	CAA<br>Aphasia with temporal damage T1 (n = 7) vs with frontal damage T1 (n = 7)	UNR	UNR	ROI Anat NDC	Number of ROIs: 14; ROIs: (1) L IFG pars opercularis; (2) L IFG pars triangularis; (3) L ventral precentral gyrus; (4) L Heschl's gyrus; (5) L temporal plane (posterior to HG, coded as posterior STG); (6) L posterior STG (coded as mid STG per Fig. 2); (7) L SMA; (8-14) homotopic counterparts; how ROIs defined: individual anatomical images; qualitative comparison on p. 434	↑ L IFG pars opercularis<br>↑ R SMA/medial prefrontal<br>↓ R IFG pars opercularis<br>↓ R posterior STG<br>↓ R mid temporal
Heiss et al. (1999): ROI 6	Noun repetition vs rest	CAA<br>Aphasia with temporal damage T2 (n = 7) vs with subcortical damage T2 (n = 9)	UNR	UNR	ROI Anat NDC	Number of ROIs: 14; ROIs: (1) L IFG pars opercularis; (2) L IFG pars triangularis; (3) L ventral precentral gyrus; (4) L Heschl's gyrus; (5) L temporal plane (posterior to HG, coded as posterior STG); (6) L posterior STG (coded as mid STG per Fig. 2); (7) L SMA; (8-14) homotopic counterparts; how ROIs defined: individual anatomical images; qualitative comparison on p. 434	↑ L IFG pars opercularis<br>↑ L ventral precentral/inferior frontal junction<br>↑ L SMA/medial prefrontal<br>↑ R ventral precentral/inferior frontal junction<br>↓ L posterior STG<br>↓ L mid temporal<br>↓ R posterior STG<br>↓ R Heschl's gyrus
Heiss et al. (1999): ROI 7	Noun repetition vs rest	CAA<br>Aphasia with temporal damage T2 (n = 7) vs with frontal damage T2 (n = 7)	UNR	UNR	ROI Anat NDC	Number of ROIs: 14; ROIs: (1) L IFG pars opercularis; (2) L IFG pars triangularis; (3) L ventral precentral gyrus; (4) L Heschl's gyrus; (5) L temporal plane (posterior to HG, coded as posterior STG); (6) L posterior STG (coded as mid STG per Fig. 2); (7) L SMA; (8-14) homotopic counterparts; how ROIs defined: individual anatomical images; qualitative comparison on p. 434	↑ L IFG pars opercularis<br>↑ L ventral precentral/inferior frontal junction<br>↑ L SMA/medial prefrontal<br>↑ R ventral precentral/inferior frontal junction<br>↓ L posterior STG<br>↓ L mid temporal<br>↓ R posterior STG<br>↓ R Heschl's gyrus
Heiss et al. (1999): ROI 8	Noun repetition vs rest	CAC<br>Aphasia with subcortical damage T1 (n = 9) vs control	UNR	UNR	ROI Anat NDC	Number of ROIs: 14; ROIs: (1) L IFG pars opercularis; (2) L IFG pars triangularis; (3) L ventral precentral gyrus; (4) L Heschl's gyrus; (5) L temporal plane (posterior to HG, coded as posterior STG); (6) L posterior STG (coded as mid STG per Fig. 2); (7) L SMA; (8-14) homotopic counterparts; how ROIs defined: individual anatomical images; qualitative comparison on p. 434	↑ R IFG pars opercularis<br>↓ L IFG<br>↓ L ventral precentral/inferior frontal junction<br>↓ L Heschl's gyrus<br>↓ L mid temporal<br>↓ R Heschl's gyrus
Heiss et al. (1999): ROI 9	Noun repetition vs rest	CAC<br>Aphasia with frontal damage T1 (n = 7) vs control	UNR	UNR	ROI Anat NDC	Number of ROIs: 14; ROIs: (1) L IFG pars opercularis; (2) L IFG pars triangularis; (3) L ventral precentral gyrus; (4) L Heschl's gyrus; (5) L	↑ R IFG pars opercularis<br>↓ L IFG pars opercularis

						temporal plane (posterior to HG, coded as posterior STG); (6) L posterior STG (coded as mid STG per Fig. 2); (7) L SMA; (8-14) homotopic counterparts; how ROIs defined: individual anatomical images; qualitative comparison on p. 434	↓ L ventral precentral/inferior frontal junction<br>↓ L posterior STG/STS/MTG<br>↓ L Heschl's gyrus<br>↓ L mid temporal<br>↓ R Heschl's gyrus
Heiss et al. (1999): ROI 10	Noun repetition vs rest	CAC<br>Aphasia with temporal damage T1 (n = 7) vs control	UNR	UNR	ROI Anat NDC	Number of ROIs: 14; ROIs: (1) L IFG pars opercularis; (2) L IFG pars triangularis; (3) L ventral precentral gyrus; (4) L Heschl's gyrus; (5) L temporal plane (posterior to HG, coded as posterior STG); (6) L posterior STG (coded as mid STG per Fig. 2); (7) L SMA; (8-14) homotopic counterparts; how ROIs defined: individual anatomical images; qualitative comparison on p. 434; L IFG pars opercularis noted as different in text despite being significant in both groups	↑ L IFG pars opercularis<br>↑ R SMA/medial prefrontal<br>↓ L ventral precentral/inferior frontal junction<br>↓ L posterior STG<br>↓ L Heschl's gyrus<br>↓ L mid temporal<br>↓ R posterior STG<br>↓ R Heschl's gyrus<br>↓ R mid temporal
Heiss et al. (1999): ROI 11	Noun repetition vs rest	CAC<br>Aphasia with subcortical damage T2 (n = 9) vs control	UNR	UNR	ROI Anat NDC	Number of ROIs: 14; ROIs: (1) L IFG pars opercularis; (2) L IFG pars triangularis; (3) L ventral precentral gyrus; (4) L Heschl's gyrus; (5) L temporal plane (posterior to HG, coded as posterior STG); (6) L posterior STG (coded as mid STG per Fig. 2); (7) L SMA; (8-14) homotopic counterparts; how ROIs defined: individual anatomical images; qualitative comparison on p. 434	↓ L IFG pars opercularis<br>↓ L ventral precentral/inferior frontal junction<br>↓ L Heschl's gyrus
Heiss et al. (1999): ROI 12	Noun repetition vs rest	CAC<br>Aphasia with frontal damage T2 (n = 7) vs control	UNR	UNR	ROI Anat NDC	Number of ROIs: 14; ROIs: (1) L IFG pars opercularis; (2) L IFG pars triangularis; (3) L ventral precentral gyrus; (4) L Heschl's gyrus; (5) L temporal plane (posterior to HG, coded as posterior STG); (6) L posterior STG (coded as mid STG per Fig. 2); (7) L SMA; (8-14) homotopic counterparts; how ROIs defined: individual anatomical images; qualitative comparison on p. 434	↓ L IFG pars opercularis<br>↓ L ventral precentral/inferior frontal junction<br>↓ L Heschl's gyrus
Heiss et al. (1999): ROI 13	Noun repetition vs rest	CAC<br>Aphasia with temporal damage T2 (n = 7) vs control	UNR	UNR	ROI Anat NDC	Number of ROIs: 14; ROIs: (1) L IFG pars opercularis; (2) L IFG pars triangularis; (3) L ventral precentral gyrus; (4) L Heschl's gyrus; (5) L temporal plane (posterior to HG, coded as posterior STG); (6) L posterior STG (coded as mid STG per Fig. 2); (7) L SMA; (8-14) homotopic counterparts; how ROIs defined: individual anatomical images; qualitative comparison on p. 434	↑ L IFG pars opercularis<br>↑ L SMA/medial prefrontal<br>↑ R ventral precentral/inferior frontal junction<br>↓ L posterior STG<br>↓ L Heschl's gyrus<br>↓ L mid temporal<br>↓ R posterior STG<br>↓ R Heschl's gyrus
Heiss et al. (1999): ROI 14	Noun repetition vs rest	LA<br>Aphasia with subcortical or frontal damage and good recovery (n = 11) T2 vs T1	UNR	UNR	ROI Anat NDC	Number of ROIs: 14; ROIs: (1) L IFG pars opercularis; (2) L IFG pars triangularis; (3) L ventral precentral gyrus; (4) L Heschl's gyrus; (5) L temporal plane (posterior to HG, coded as posterior STG); (6) L	↑ L SMA/medial prefrontal<br>↑ L Heschl's gyrus<br>↑ R ventral precentral/inferior frontal junction

						posterior STG (coded as mid STG per Fig. 2); (7) L SMA; (8-14) homotopic counterparts; how ROIs defined: individual anatomical images; qualitative comparison on pp. 434-5	↑ R SMA/medial prefrontal<br>↑ R Heschl's gyrus<br>↓ R IFG pars opercularis
Heiss et al. (1999): ROI 15	Noun repetition vs rest	LA<br>Aphasia with subcortical or frontal damage and poor recovery (n = 5) T2 vs T1	UNR	UNR	ROI<br>Anat<br>NDC	Number of ROIs: 14; ROIs: (1) L IFG pars opercularis; (2) L IFG pars triangularis; (3) L ventral precentral gyrus; (4) L Heschl's gyrus; (5) L temporal plane (posterior to HG, coded as posterior STG); (6) L posterior STG (coded as mid STG per Fig. 2); (7) L SMA; (8-14) homotopic counterparts; how ROIs defined: individual anatomical images; qualitative comparison on pp. 434-5	↑ L ventral precentral/inferior frontal junction<br>↑ R Heschl's gyrus<br>↓ R IFG pars opercularis
Heiss et al. (1999): ROI 16	Noun repetition vs rest	CAA<br>Aphasia with subcortical and frontal damage and good recovery T1 (n = 11) vs with subcortical and frontal damage and poor recovery T1 (n = 5)	UNR	UNR	ROI<br>Anat<br>NDC	Number of ROIs: 14; ROIs: (1) L IFG pars opercularis; (2) L IFG pars triangularis; (3) L ventral precentral gyrus; (4) L Heschl's gyrus; (5) L temporal plane (posterior to HG, coded as posterior STG); (6) L posterior STG (coded as mid STG per Fig. 2); (7) L SMA; (8-14) homotopic counterparts; how ROIs defined: individual anatomical images; qualitative comparison on p. 435	↑ L posterior STG<br>↑ L mid temporal
Heiss et al. (1999): ROI 17	Noun repetition vs rest	CAA<br>Aphasia with subcortical and frontal damage and good recovery T2 (n = 11) vs with subcortical and frontal damage and poor recovery T2 (n = 5)	UNR	UNR	ROI<br>Anat<br>NDC	Number of ROIs: 14; ROIs: (1) L IFG pars opercularis; (2) L IFG pars triangularis; (3) L ventral precentral gyrus; (4) L Heschl's gyrus; (5) L temporal plane (posterior to HG, coded as posterior STG); (6) L posterior STG (coded as mid STG per Fig. 2); (7) L SMA; (8-14) homotopic counterparts; how ROIs defined: individual anatomical images; qualitative comparison on p. 435	↑ L SMA/medial prefrontal<br>↑ L posterior STG<br>↑ L Heschl's gyrus<br>↑ L mid temporal<br>↑ R ventral precentral/inferior frontal junction<br>↑ R SMA/medial prefrontal<br>↓ L ventral precentral/inferior frontal junction
Kessler et al. (2000): ROI 1	Word repetition vs rest	LA<br>Aphasia treated with pirecetam (n = 12) T2 vs T1	UNR	UNR	ROI<br>Anat<br>NC	Number of ROIs: 14; ROIs: (1) L BA 44; (2) L BA 45; (3) L ventral PrCG; (4) L HG; (5) L BA 41 and 42; (6) L BA 22; (7) L SMA; (8-14) homotopic counterparts; how ROIs defined: individual anatomical images	↑ L IFG pars triangularis<br>↑ L posterior STG<br>↑ L Heschl's gyrus
Kessler et al. (2000): ROI 2	Word repetition vs rest	LA<br>Aphasia treated with placebo (n = 12) T2 vs T1	UNR	UNR	ROI<br>Anat<br>NC	Number of ROIs: 14; ROIs: (1) L BA 44; (2) L BA 45; (3) L ventral PrCG; (4) L HG; (5) L BA 41 and 42; (6) L BA 22; (7) L SMA; (8-14) homotopic counterparts; how ROIs defined: individual anatomical images	↑ L ventral precentral/inferior frontal junction
Rosen et al. (2000): Vox 1	Word stem completion (PET) vs rest (PET)	CAC<br>Aphasia vs control	N	Y	Vox<br>U	Search volume: whole brain; software: not stated; correction for multiple comparisons unclear; there may be circularity in only correcting for the number of regions that seemed to show differences	↑ L SMA/medial prefrontal<br>↑ R IFG<br>↑ R Heschl's gyrus<br>↓ L IFG
Rosen et al. (2000): Vox 2	Word stem completion (fMRI) vs rest (fMRI)	CAC<br>Aphasia (n = 5) vs control	UNR	UNR	Vox<br>NDC	Search volume: whole brain; software: not stated; qualitative comparison on p. 1888	↑ R IFG<br>↓ L IFG

Rosen et al. (2000): ROI 1	Word stem completion (fMRI) vs rest (fMRI)	CAC Aphasia (n = 5) vs control	UNR	UNR	ROI Func NC	Number of ROIs: 2; ROIs: (1) R IFG; (2) SMA; how ROIs defined: not stated but seem to be functional; possibly circular because not clear how ROIs defined	↑ R IFG
Blasi et al. (2002): Vox 1	Word stem completion (novel items) vs rest	CAC Aphasia vs control	N	N	Vox U	Behavioral data notes: covert task but overt data acquired separately; patients less accurate and slower than controls; search volume: whole brain; software: not stated; voxelwise p: ~.001 (z > 3); cluster extent cutoff: 45 voxels (size not stated); Monte Carlo analysis not described in detail; rather than fitting a HRF, the authors looked at the shape of the signal in the 8 volumes following each stimulus	↑ R IFG pars opercularis<br>↑ R IFG pars triangularis<br>↑ R insula<br>↑ R ventral precentral/inferior frontal junction<br>↑ R dorsal precentral<br>↓ L IFG pars opercularis<br>↓ L ventral precentral/inferior frontal junction<br>notes: labels based on coordinates reported
Blasi et al. (2002): ROI 1	Word stem completion (novel items) vs word stem completion (repeated items)	CAC Aphasia vs control	Y	Y	ROI Func NC	Behavioral data notes: covert task but overt data acquired separately; no interaction of group by practice for accuracy or RT; number of ROIs: 14; ROIs: (1) L dorsal IFG; (2) L ventral IFG; (3) R MFG; (4) L anterior fusiform; (5) R anterior fusiform; (6) R posterior fusiform; (7) R lateral occipital; (8) R lateral cerebellum; (9) L SMA; (10) R dorsal IFG; (11) R posterior fusiform; (12) R lateral occipital; (13) R lingual; (14) L MTG; how ROIs defined: regions that were active for the main effect of word stem completion (irrespective of practice) in either group and modulated by practice in that group; circular because ROIs defined in one group or the other; the L ROIs showed repetition suppression in controls but not in patients, and this difference is interpreted by the authors, but not supported statistically	↑ R ventral precentral/inferior frontal junction<br>↑ R posterior inferior temporal gyrus/fusiform gyrus<br>↓ L IFG<br>↓ L ventral precentral/inferior frontal junction<br>↓ L posterior inferior temporal gyrus/fusiform gyrus<br>notes: labels based on coordinates reported
Leff et al. (2002): Vox 1	Higher word rates vs lower word rates	CAC Aphasia with pSTS damage (n = 6) vs control	NANB	NANT	Vox NDC	Search volume: whole brain; software: SPM99; qualitative comparison on p. 555; a FWE-corrected SPM is reported of the relationship in the 6 patients with L pSTS damage (Fig. 2), however it is masked in a way that is not explained (see figure caption), and there is no direct comparison between patients with L pSTS damage and controls	↑ R posterior STS
Leff et al. (2002): Vox 2	Higher word rates vs lower word rates	CAA Aphasia with pSTS (n = 6) damage vs without pSTS damage (n = 9)	NANB	NANT	Vox NDC	Search volume: whole brain; software: SPM99; qualitative comparison on p. 555; a FWE-corrected SPM is reported of the relationship in the 6 patients with L pSTS damage (Fig. 2), however it is masked in a way that is not	↑ R posterior STS

						explained (see figure caption), and there is no direct comparison between patients with L pSTS damage and patients with R pSTS damage	
Leff et al. (2002): ROI 1	Higher word rates vs lower word rates	CAC Aphasia with pSTS damage (n = 6) vs control (n = 8)	NANB	NANT	ROI Func One	Number of ROIs: 1; ROI: R pSTS; how ROI defined: the peak voxel for the contrast in the R pSTS from each subject's individual analysis, but the search region is not stated; the controls and patients without pSTS damage were combined, however it is stated in the caption to Figure 2 that the patients with pSTS damage were significantly different to both	↑ R posterior STS
Leff et al. (2002): ROI 2	Higher word rates vs lower word rates	CAA Aphasia with pSTS damage (n = 6) vs aphasia without pSTS damage (n = 9)	NANB	NANT	ROI Func One	Number of ROIs: 1; ROI: R pSTS; how ROI defined: the peak voxel for the contrast in the R pSTS from each subject's individual analysis, but the search region is not stated; the controls and patients without pSTS damage were combined, however it is stated in the caption to Figure 2 that the patients with pSTS damage were significantly different to both	↑ R posterior STS
Blank et al. (2003): Vox 1	Propositional speech production vs rest	CAC Aphasia with IFG POp damage (n = 7) vs control	N	NANT	Vox SVC	Behavioral data notes: word rates not reported, but offline speech sample differed; search volume: voxels spared in all patients; software: SPM99; voxelwise p: FWE p < .05 with SVC in R pars opercularis	↑ R IFG pars opercularis notes: no voxels survived FWE correction without SVC
Blank et al. (2003): Vox 2	Propositional speech production vs rest	CAC Aphasia without IFG POp damage (n = 7) vs control	N	NANT	Vox SVC	Behavioral data notes: word rates not reported, but offline speech sample differed; search volume: voxels spared in all patients; software: SPM99; voxelwise p: FWE p < .05 with SVC in R pars opercularis	↑ R IFG pars opercularis
Blank et al. (2003): Vox 3	Propositional speech production vs rest	CAA Aphasia with IFG POp damage (n = 7) vs without IFG POp damage (n = 7)	N	NANT	Vox SVC	Behavioral data notes: word rates not reported, but offline speech sample differed; search volume: voxels spared in all patients; software: SPM99; voxelwise p: FWE p < .05 with SVC in R pars opercularis	None notes: patients with L IFG POp damage showed numerically more signal in the R IFG POp
Blank et al. (2003): Vox 4	Propositional speech production vs counting	CAC Aphasia with IFG POp damage (n = 7) vs control	N	NANT	Vox SVC	Behavioral data notes: word rates not reported, but offline speech sample differed; search volume: voxels spared in all patients; software: SPM99; voxelwise p: FWE p < .05 with SVC in R pars opercularis	None
Blank et al. (2003): Vox 5	Propositional speech production vs counting	CAC Aphasia without IFG POp damage (n = 7) vs control	N	NANT	Vox SVC	Behavioral data notes: word rates not reported, but offline speech sample differed; search volume: voxels spared in all patients; software: SPM99; voxelwise p: FWE p < .05 with SVC in R pars opercularis	None
Blank et al. (2003): Vox 6	Propositional speech production vs counting	CAA Aphasia with IFG POp damage (n = 7) vs without IFG POp damage (n = 7)	N	NANT	Vox SVC	Behavioral data notes: word rates not reported, but offline speech sample differed; search volume: voxels spared in all patients; software: SPM99; voxelwise p: FWE p < .05 with SVC in R pars opercularis	None

Blank et al. (2003): ROI 1	Propositional speech production vs rest	CC<br>Aphasia with IFG POp damage (n = 7)<br>Covariate: speech rate during scan	UNR	NANT	ROI Func One	Number of ROIs: 1; ROI: R IFG pars opercularis; how ROI defined: defined by flipping L IFG pars opercularis activation in controls	None
Blank et al. (2003): ROI 2	Propositional speech production vs rest	CC<br>Aphasia without IFG POp damage (n = 7)<br>Covariate: speech rate during scan	UNR	NANT	ROI Func One	Number of ROIs: 1; ROI: R IFG pars opercularis; how ROI defined: defined by flipping L IFG pars opercularis activation in controls	None
Blank et al. (2003): ROI 3	Propositional speech production vs rest	CC<br>Aphasia with IFG POp damage (n = 7)<br>Covariate: four different QPA measures	UNR	NANT	ROI Func One	Number of ROIs: 1; ROI: R IFG pars opercularis; how ROI defined: defined by flipping L IFG pars opercularis activation in controls	None
Cardebat et al. (2003): Vox 1	Word generation vs rest	LA<br>Aphasia T2 vs T1	N	UNR	Vox CA	Search volume: whole brain; software: SPM99; voxelwise p: .05; cluster extent cutoff: 50 voxels (size not stated); nature of inclusive masks unclear	↑ L dorsolateral prefrontal cortex<br>↑ L SMA/medial prefrontal<br>↑ L somato-motor<br>↑ L posterior STG/STS/MTG<br>↑ L cerebellum<br>↑ R IFG pars opercularis<br>↑ R dorsolateral prefrontal cortex<br>↑ R SMA/medial prefrontal<br>↑ R somato-motor<br>↑ R posterior STG/STS/MTG<br>↑ R cerebellum<br>notes: based on Figure 2
Cardebat et al. (2003): Vox 2	Word generation vs rest	LC<br>Aphasia T2 vs T1<br>Covariate: Δ word generation accuracy	C	UNR	Vox CA	Search volume: whole brain; software: SPM99; voxelwise p: .001; cluster extent cutoff: 100 voxels (size not stated); nature of inclusive masks unclear	↑ L posterior STG/STS/MTG<br>↑ R posterior STG/STS/MTG<br>↑ R cerebellum<br>↓ L occipital<br>↓ L hippocampus/MTL<br>↓ R dorsolateral prefrontal cortex<br>↓ R occipital
Sharp et al. (2004): Vox 1	Semantic decision vs syllable count decision	CAC<br>Aphasia vs control (clear speech)	AM	Y	Vox SVC	Behavioral data notes: interaction of group by task not reported for accuracy; search volume: whole brain; software: SPM99; voxelwise p: FWE p < .05 with SVC in fusiform gyri, temporal poles, L IFG, L orbitofrontal and L SFG	↓ L posterior inferior temporal gyrus/fusiform gyrus
Sharp et al. (2004): Vox 2	Semantic decision vs syllable count decision	CC<br>Aphasia<br>Covariate: semantic decision accuracy	C	UNR	Vox SVC	Search volume: whole brain; software: SPM99; voxelwise p: FWE p < .05 with SVC in fusiform gyri, temporal poles, L IFG, L orbitofrontal and L SFG; fixed effects; this analysis is not clearly described	↑ R posterior inferior temporal gyrus/fusiform gyrus<br>notes: patients who were more accurate had more activity in R

							anterior fusiform gyrus
Sharp et al. (2004): ROI 1	Semantic decision vs syllable count decision	CAC<br>Aphasia vs control (clear speech)	AM	Y	ROI<br>Anat<br>One	Behavioral data notes: interaction of group by task not reported for accuracy; number of ROIs: 1; ROI: L fusiform gyrus; how ROI defined: probabilistic brain atlas	↓ L posterior inferior temporal gyrus/fusiform gyrus
Sharp et al. (2004): ROI 2	Semantic decision vs syllable count decision	CAC<br>Aphasia vs control (noise vocoded)	NAM	Y	ROI<br>Anat<br>One	Behavioral data notes: patients were more accurate on semantic decisions than syllable decisions, whereas controls were less accurate on noise vocoded semantic decisions than clear syllable decisions (which were the baseline for this analysis); number of ROIs: 1; ROI: L fusiform gyrus; how ROI defined: probabilistic brain atlas	None<br>notes: this analysis suggests that the difference between groups in the L fusiform gyrus disappears when the controls perform a semantic task that is similarly challenging
Zahn et al. (2004): ROI 1	Semantic decision vs phonetic decision and lexical decision (conjunction)	CAC<br>Aphasia vs control	UNT	UNR	ROI<br>LI<br>One	Behavioral data notes: relative performance on language and control tasks unclear; number of ROIs: 1; ROI: language network LI; conjunction analyses not clearly described; in two patients, a different conjunction was used (lexical decision vs phonetic decision & semantic decision vs phonetic decision)	None<br>notes: LI > 0 in 12 out of 14 controls and 5 out of 7 patients; no significant difference
Crinion & Price (2005): Vox 1	Listening to narrative speech vs listening to reversed speech	CAC<br>Aphasia without temporal lobe damage (n = 9) vs control	NANB	NANT	Vox<br>VFWC	Search volume: whole brain; software: SPM2; voxelwise p: FWE p < .05; cluster extent cutoff: 5 voxels (size not stated)	↓ L dorsal precentral<br>↓ R somato-motor
Crinion & Price (2005): Vox 2	Listening to narrative speech vs listening to reversed speech	CAC<br>Aphasia with temporal lobe damage (n = 8) vs control	NANB	NANT	Vox<br>VFWC	Search volume: whole brain; software: SPM2; voxelwise p: FWE p < .05; cluster extent cutoff: 5 voxels (size not stated)	↓ L posterior STS<br>↓ L mid temporal
Crinion & Price (2005): Vox 3	Listening to narrative speech vs listening to reversed speech	CAA<br>Aphasia with temporal lobe damage (n = 8) vs without temporal lobe damage (n = 9)	NANB	NANT	Vox<br>VFWC	Search volume: whole brain; software: SPM2; voxelwise p: FWE p < .05; cluster extent cutoff: 5 voxels (size not stated)	↓ L posterior STG/STS/MTG<br>↓ L mid temporal
Crinion & Price (2005): Vox 4	Listening to narrative speech vs listening to reversed speech	CC<br>Aphasia without temporal lobe damage (n = 9)<br>Covariate: sentence comprehension (CAT)	NANB	NANT	Vox<br>VFWC	Search volume: whole brain; software: SPM2; voxelwise p: FWE p < .05; cluster extent cutoff: 5 voxels (size not stated); conjunction with main effect of story comprehension (details hard to follow); this was a multiple regression also involving patients with temporal lobe damage	↑ L posterior STS<br>↑ R mid temporal<br>notes: patients with better sentence comprehension had more activation in the L posterior STS and R mid STS
Crinion & Price (2005): Vox 5	Listening to narrative speech vs listening to reversed speech	CC<br>Aphasia with temporal lobe damage (n = 8)<br>Covariate: sentence comprehension (CAT)	NANB	NANT	Vox<br>VFWC	Search volume: whole brain; software: SPM2; voxelwise p: FWE p < .05; cluster extent cutoff: 5 voxels (size not stated); conjunction with main effect of story comprehension (details hard to follow); this was a multiple	↑ R mid temporal<br>notes: patients with better sentence comprehension had more

						regression also involving patients without temporal lobe damage	activation in the R mid STS
Crinion & Price (2005): Cplx 1	Listening to narrative speech vs listening to reversed speech	CAA<br>Aphasia with temporal damage (n = 8) vs without temporal damage (n = 9)	NANB	NANT	Cplx	Correlations were computed between activity in each voxel, and the sentence comprehension measure from the CAT, and were compared between the two aphasia groups, in regions with a main effect of story comprehension. The voxelwise threshold was p < .001, uncorrected for multiple comparisons.	Other:<br>Activity in the L posterior STS was positively correlated with sentence comprehension in patients without temporal lobe damage, but not in patients with temporal lobe damage
Crinion & Price (2005): Cplx 2	Listening to narrative speech vs listening to reversed speech	CAC<br>Aphasia without temporal damage (n = 9) vs control	NANB	NANT	Cplx	Correlations were computed between activity in each voxel, and post-scan story recall, and were compared between patients without temporal damage and controls, in regions with a main effect of story comprehension. The threshold was p < 0.05 corrected, plus a minimum cluster size of 5 voxels.	None
Crinion & Price (2005): Cplx 3	Listening to narrative speech vs listening to reversed speech	CAC<br>Aphasia with temporal damage (n = 8) vs control	NANB	NANT	Cplx	Correlations were computed between activity in each voxel, and post-scan story recall, and were compared between patients with temporal damage and controls, in regions with a main effect of story comprehension. The threshold was p < 0.05 corrected, plus a minimum cluster size of 5 voxels.	None
Crinion & Price (2005): Cplx 4	Listening to narrative speech vs listening to reversed speech	CAA<br>Aphasia with temporal damage (n = 8) vs without temporal damage (n = 9)	NANB	NANT	Cplx	Correlations were computed between activity in each voxel, and post-scan story recall, and were compared between the two aphasia groups, in regions with a main effect of story comprehension. The threshold was p < 0.05 corrected, plus a minimum cluster size of 5 voxels.	None
de Boissezon et al. (2005): Vox 1	Word generation vs rest	CC<br>Aphasia T1<br>Covariate: time post onset	Y	UNR	Vox CA	Behavioral data notes: no significant correlation between time post onset and accuracy; search volume: whole brain; software: SPM2; voxelwise p: .01; cluster extent cutoff: 50 voxels (size not stated)	↑ L orbitofrontal<br>↑ L anterior temporal<br>↑ L occipital<br>↑ L anterior cingulate<br>↑ L cerebellum<br>↑ R anterior temporal<br>↑ R occipital<br>notes: more activity with longer time post onset; based on coordinates in Table 3a
de Boissezon et al.	Word generation vs rest	CC<br>Aphasia T1<br>Covariate: word	C	UNR	Vox CA	Search volume: whole brain; software: SPM2; voxelwise p: .01; cluster extent cutoff: 50 voxels (size not stated)	↑ L IFG pars triangularis<br>↑ L dorsolateral prefrontal cortex

(2005): Vox 2		generation accuracy T1					↑ L precuneus<br>↑ L Heschl's gyrus<br>↑ L anterior temporal<br>↑ R insula<br>↑ R posterior STG<br>notes: based on coordinates in Table 3b
de Boissezon et al. (2005): Vox 3	Word generation vs rest	LA<br>Aphasia T2 vs T1	N	UNR	Vox<br>CA	Search volume: whole brain; software: SPM2; voxelwise p: .001; cluster extent cutoff: 100 voxels (size not stated); description of masking unclear, but seems to be inclusively masked with T1, which seems inappropriate	↑ L insula<br>↑ L posterior STG<br>↑ R orbitofrontal<br>↑ R posterior STG<br>↑ R cerebellum<br>notes: based on coordinates in Table 2
de Boissezon et al. (2005): Vox 4	Word generation vs rest	LC<br>Aphasia T2 vs T1<br>Covariate: Δ word generation accuracy	C	UNR	Vox<br>CA	Search volume: whole brain; software: SPM2; voxelwise p: .01; cluster extent cutoff: 20 voxels (size not stated)	↑ L mid temporal<br>↑ R anterior temporal<br>↑ R cerebellum<br>notes: based on coordinates in Table 3c
Connor et al. (2006): Vox 1	Word stem completion (novel items) vs word stem completion (repeated items)	CAC<br>Aphasia vs control	Y	Y	Vox<br>NDC	Behavioral data notes: covert task but overt data acquired separately; no interaction of group by practice for accuracy or RT; search volume: cerebellum; software: not stated; qualitative comparison on p. 174; Monte Carlo-based thresholding not described; rather than fitting a HRF, the authors looked at the shape of the signal in the 8 volumes following each stimulus	↑ L cerebellum<br>↓ R cerebellum
Connor et al. (2006): ROI 1	Word stem completion (novel items) vs word stem completion (repeated items)	CAC<br>Aphasia vs control	Y	Y	ROI<br>Func<br>One	Behavioral data notes: covert task but overt data acquired separately; no interaction of group by practice for accuracy or RT; number of ROIs: 1; ROI: L cerebellum; how ROI defined: L cerebellar region with a learning effect in the patients; circular because ROIs defined in one group; rather than fitting a HRF, the authors looked at the shape of the signal in the 8 volumes following each stimulus	↑ L cerebellum
Crinion et al. (2006): Vox 1	Listening to narrative speech vs listening to reversed speech	CAC<br>Aphasia vs control	NANB	NANT	Vox<br>VFWE	Search volume: voxels spared in all patients; software: SPM99; voxelwise p: FWE p < .05	None
Crinion et al. (2006): Vox 2	Listening to narrative speech vs listening to reversed speech	CAC<br>Aphasia without temporal lobe damage (n = 6) vs control	NANB	NANT	Vox<br>VFWE	Search volume: voxels spared in all included patients; software: SPM99; voxelwise p: FWE p < .05	None
Crinion et al. (2006): Vox 3	Listening to narrative speech vs listening to reversed speech	CAC<br>Aphasia with temporal lobe damage (n = 18) vs control	NANB	NANT	Vox<br>VFWE	Search volume: voxels spared in all included patients; software: SPM99; voxelwise p: FWE p < .05	None
Crinion et al	Listening to	CC	NANB	NANT	ROI	Number of ROIs: 1; ROI: L ATL; how	↑ L anterior

al. (2006): ROI 1	narrative speech vs listening to reversed speech	Aphasia with no temporal damage (excluding 1 with missing behavioral data and 1 outlier) or posterior temporal damage sparing anterior temporal cortex (n = 13)<br>Covariate: auditory sentence comprehension (CAT)			Func<br>One	ROI defined: activation in the control group; same result obtained with or without excluding one outlier; two other ROIs are described in the methods, but never used in any analyses	temporal<br>notes: more activity in patients with better auditory sentence comprehension
Crinion et al. (2006): ROI 2	Listening to narrative speech vs listening to reversed speech	CC<br>Aphasia with no temporal damage (excluding 1 with missing behavioral data and 1 outlier) or posterior temporal damage sparing anterior temporal cortex (n = 13)<br>Covariate: time post onset	NANB	NANT	ROI<br>Func<br>One	Number of ROIs: 1; ROI: L ATL; how ROI defined: activation in the control group; two other ROIs are described in the methods, but never used in any analyses	None
Crinion et al. (2006): ROI 3	Listening to narrative speech vs listening to reversed speech	CAA<br>Aphasia with temporal damage excluding anterior temporal cortex (n = 9) vs with no temporal lobe damage (excluding 1 with missing behavioral data and 1 outlier) (n = 4)	NANB	NANT	ROI<br>Func<br>One	Number of ROIs: 1; ROI: L ATL; how ROI defined: activation in the control group; two other ROIs are described in the methods, but never used in any analyses	↓ L anterior temporal<br>notes: patients with posterior temporal damage had less signal change
Crinion et al. (2006): ROI 4	Listening to narrative speech vs listening to reversed speech	CAC<br>Aphasia with temporal damage excluding anterior temporal cortex (n = 9) vs control	NANB	NANT	ROI<br>Func<br>One	Number of ROIs: 1; ROI: L ATL; how ROI defined: activation in the control group; circular because ROI defined in one group; two other ROIs are described in the methods, but never used in any analyses	↓ L anterior temporal<br>notes: large difference 2.7 ± 0.8 (patients) vs 6.3 ± 1.4 (controls) makes finding suggestive even in light of the circularity
Crinion et al. (2006): ROI 5	Listening to narrative speech vs listening to reversed speech	CC<br>Aphasia with no temporal damage (excluding 1 with missing behavioral data and 1 outlier) or posterior temporal damage sparing anterior temporal cortex (n = 13)<br>Covariate: auditory single word comprehension (CAT)	NANB	NANT	ROI<br>Func<br>One	Number of ROIs: 1; ROI: L ATL; how ROI defined: activation in the control group; two other ROIs are described in the methods, but never used in any analyses	None<br>notes: r = 0.39; p > 0.1; seems to be a clear trend so lack of significance may reflect only lack of power
Saur et al. (2006): Vox 1	Listening to sentences and making a plausibility	LA<br>Aphasia T2 vs T1	AM	UNR	Vox<br>NC	Behavioral data notes: accuracy combines language and control conditions; search volume: whole	↑ L insula<br>↑ R IFG pars orbitalis<br>↑ R insula

	judgment vs listening to reversed speech					brain; software: SPM2; voxelwise p: .001; cluster extent cutoff: none	↑ R SMA/medial prefrontal<br>notes: R IFG/insula activation noted to survive FWE correction at p < .05
Saur et al. (2006): Vox 2	Listening to sentences and making a plausibility judgment vs listening to reversed speech	LA<br>Aphasia T3 vs T2	AM	UNR	Vox<br>NC	Behavioral data notes: accuracy combines language and control conditions; search volume: whole brain; software: SPM2; voxelwise p: .005; cluster extent cutoff: none; threshold was lowered to reveal the R frontal change in activation	↓ R IFG pars orbitalis<br>↓ R occipital
Saur et al. (2006): Vox 3	Listening to sentences and making a plausibility judgment vs listening to reversed speech	LA<br>Aphasia T3 vs T1	AM	UNR	Vox<br>NC	Behavioral data notes: accuracy combines language and control conditions; search volume: whole brain; software: SPM2; voxelwise p: .001; cluster extent cutoff: none	↑ L IFG pars orbitalis<br>↑ L SMA/medial prefrontal<br>↑ L posterior inferior temporal gyrus/fusiform gyrus<br>↑ R IFG pars orbitalis<br>↑ R insula
Saur et al. (2006): Vox 4	Listening to sentences and making a plausibility judgment vs listening to reversed speech	CAC<br>Aphasia T1 vs control	AM	UNR	Vox<br>NC	Behavioral data notes: accuracy combines language and control conditions; search volume: whole brain; software: SPM2; voxelwise p: .001; cluster extent cutoff: none	↓ L IFG pars triangularis<br>↓ L IFG pars orbitalis<br>↓ L insula<br>↓ L posterior MTG<br>↓ L posterior inferior temporal gyrus/fusiform gyrus<br>↓ R IFG pars orbitalis<br>↓ R insula<br>notes: L STG in table is actually MTG based on coordinates
Saur et al. (2006): Vox 5	Listening to sentences and making a plausibility judgment vs listening to reversed speech	CAC<br>Aphasia T2 vs control	AM	UNR	Vox<br>NC	Behavioral data notes: accuracy combines language and control conditions; search volume: whole brain; software: SPM2; voxelwise p: .005; cluster extent cutoff: none; threshold was lowered to reveal L IFG	↑ L IFG pars orbitalis<br>↑ L insula<br>↑ L SMA/medial prefrontal<br>↑ R IFG
Saur et al. (2006): Vox 6	Listening to sentences and making a plausibility judgment vs listening to reversed speech	CAC<br>Aphasia T3 vs control	AS	UNR	Vox<br>NC	Behavioral data notes: accuracy combines language and control conditions; search volume: whole brain; software: SPM2; voxelwise p: .001; cluster extent cutoff: none	None
Saur et al. (2006): Vox 7	Listening to sentences and making a plausibility judgment vs	CC<br>Aphasia T1<br>Covariate: language recovery score T1	AM	UNR	Vox<br>NC	Behavioral data notes: accuracy combines language and control conditions; search volume: whole brain; software: SPM2; voxelwise p: .001; cluster extent cutoff: none	↑ L IFG<br>↑ L SMA/medial prefrontal<br>↑ R IFG pars triangularis

	listening to reversed speech						
Saur et al. (2006): Vox 8	Listening to sentences and making a plausibility judgment vs listening to reversed speech	CC<br>Aphasia T2<br>Covariate: language recovery score T2	UNT	UNR	Vox<br>NC	Behavioral data notes: accuracy combines language and control conditions; search volume: whole brain; software: SPM2; voxelwise p: .001; cluster extent cutoff: none	None
Saur et al. (2006): Vox 9	Listening to sentences and making a plausibility judgment vs listening to reversed speech	CC<br>Aphasia T3<br>Covariate: language recovery score T3	UNT	UNR	Vox<br>NC	Behavioral data notes: accuracy combines language and control conditions; search volume: whole brain; software: SPM2; voxelwise p: .001; cluster extent cutoff: none	None
Saur et al. (2006): Vox 10	Listening to sentences and making a plausibility judgment vs listening to reversed speech	LC<br>Aphasia T2 vs T1<br>Covariate: % change in language recovery score	UNT	UNR	Vox<br>NC	Behavioral data notes: accuracy combines language and control conditions; search volume: whole brain; software: SPM2; voxelwise p: .001; cluster extent cutoff: none	↑ L SMA/medial prefrontal<br>↑ R insula<br>↑ R SMA/medial prefrontal
Saur et al. (2006): Vox 11	Listening to sentences and making a plausibility judgment vs listening to reversed speech	LC<br>Aphasia T3 vs T2<br>Covariate: % change in language recovery score	UNT	UNR	Vox<br>NC	Behavioral data notes: accuracy combines language and control conditions; search volume: whole brain; software: SPM2; voxelwise p: .001; cluster extent cutoff: none	None
Saur et al. (2006): Vox 12	Listening to sentences and making a plausibility judgment vs listening to reversed speech	LC<br>Aphasia T3 vs T1<br>Covariate: % change in language recovery score	UNT	UNR	Vox<br>NC	Behavioral data notes: accuracy combines language and control conditions; search volume: whole brain; software: SPM2; voxelwise p: .001; cluster extent cutoff: none	None
Saur et al. (2006): ROI 1	Listening to sentences and making a plausibility judgment vs listening to reversed speech	LA<br>Aphasia T2 vs T1	AM	UNR	ROI<br>Func<br>FWE	Behavioral data notes: accuracy combines language and control conditions; number of ROIs: 6; ROIs: (1) L IFG pars orbitalis; (2) L IFG pars triangularis; (3) L MTG; (4) R insula; (5) R IFG pars triangularis; (6) R SMA; how ROIs defined: peak voxels of overall activation map based on all three time points in patients	↑ R insula<br>↑ R SMA/medial prefrontal<br>notes: some other ROIs also significant prior to correction for multiple comparisons; n.b. performance confound
Saur et al. (2006): ROI 2	Listening to sentences and making a plausibility judgment vs listening to reversed speech	LA<br>Aphasia T3 vs T2	AM	UNR	ROI<br>Func<br>FWE	Behavioral data notes: accuracy combines language and control conditions; number of ROIs: 6; ROIs: (1) L IFG pars orbitalis; (2) L IFG pars triangularis; (3) L MTG; (4) R insula; (5) R IFG pars triangularis; (6) R SMA; how ROIs defined: peak voxels of overall activation map based on all three time points in patients	None<br>notes: some other ROIs also significant prior to correction for multiple comparisons; n.b. performance confound
Saur et al. (2006): ROI 3	Listening to sentences and making a plausibility	LA<br>Aphasia T3 vs T1	AM	UNR	ROI<br>Func<br>FWE	Behavioral data notes: accuracy combines language and control conditions; number of ROIs: 6; ROIs: (1) L IFG pars orbitalis; (2) L IFG pars	↑ L posterior MTG<br>notes: some other ROIs also significant prior to

	judgment vs listening to reversed speech					triangularis; (3) L MTG; (4) R insula; (5) R IFG pars triangularis; (6) R SMA; how ROIs defined: peak voxels of overall activation map based on all three time points in patients	correction for multiple comparisons; n.b. performance confound
Saur et al. (2006): ROI 4	Listening to sentences and making a plausibility judgment vs listening to reversed speech	CAC<br>Aphasia T1 vs control	AM	UNR	ROI<br>Func<br>NC	Behavioral data notes: accuracy combines language and control conditions; number of ROIs: 6; ROIs: (1) L IFG pars orbitalis; (2) L IFG pars triangularis; (3) L MTG; (4) R insula; (5) R IFG pars triangularis; (6) R SMA; how ROIs defined: peak voxels of overall activation map based on all three time points in patients; circular because ROIs defined in one group	↓ L posterior MTG<br>↓ R IFG pars triangularis<br>notes: R IFG difference described in text but not table
Saur et al. (2006): ROI 5	Listening to sentences and making a plausibility judgment vs listening to reversed speech	CAC<br>Aphasia T2 vs control	AM	UNR	ROI<br>Func<br>NC	Behavioral data notes: accuracy combines language and control conditions; number of ROIs: 6; ROIs: (1) L IFG pars orbitalis; (2) L IFG pars triangularis; (3) L MTG; (4) R insula; (5) R IFG pars triangularis; (6) R SMA; how ROIs defined: peak voxels of overall activation map based on all three time points in patients; circular because ROIs defined in one group	None
Saur et al. (2006): ROI 6	Listening to sentences and making a plausibility judgment vs listening to reversed speech	CAC<br>Aphasia T3 vs control	AS	UNR	ROI<br>Func<br>NC	Behavioral data notes: accuracy combines language and control conditions; number of ROIs: 6; ROIs: (1) L IFG pars orbitalis; (2) L IFG pars triangularis; (3) L MTG; (4) R insula; (5) R IFG pars triangularis; (6) R SMA; how ROIs defined: peak voxels of overall activation map based on all three time points in patients; circular because ROIs defined in one group	None
Meinzer et al. (2008): ROI 1	Picture naming (trained items) vs rest	LC<br>Aphasia T2 vs T1<br>Covariate: Δ picture naming (trained items)	C	UNR	ROI<br>Oth<br>NC	Behavioral data notes: picture naming score (trained items) increased from 51.7 ± 24.8 to 78.8 ± 22.1, which was statistically significant ($p < 0.0001$); number of ROIs: 4; ROIs: (1) perilesional area of slow wave activity determined with MEG; (2) right hemisphere homotopic to lesion; (3) right hemisphere homotopic to slow wave area; (4) remainder of left hemisphere; for one patient, maximal slow wave activity was in the right hemisphere and it is not clear how this was handled; how ROIs defined: the dependent measure was the number of voxels in each ROI exceeding certain thresholds that differed across subjects depending on their strength of activation; it appears that increases and decreases may have been summed, though the description is hard to follow; 2 of the 11 patients were classified as outliers and excluded from analyses, however no plots are provided to justify their status as outliers	Other: improved picture naming of trained items was correlated with increased signal in 3 of the 4 ROIs, the exception being the right hemisphere ROI homotopic to the slow wave area; after removing the two outliers, only the correlation in the left hemisphere area of slow wave activity remained significant
Meinzer et	Picture naming	LC	C	UNR	ROI	Behavioral data notes: picture naming	Other:

al. (2008): ROI 2	(untrained items) vs rest	Aphasia T2 vs T1<br>Covariate: Δ picture naming (untrained items)			Oth<br>NC	score (untrained items) increased from 54.0 ± 24.3 to 70.5 ± 26.7, which was statistically significant (p= 0.002); number of ROIs: 4; ROIs: (1) perilesional area of slow wave activity determined with MEG; (2) right hemisphere homotopic to lesion; (3) right hemisphere homotopic to slow wave area; (4) remainder of left hemisphere; for one patient, maximal slow wave activity was in the right hemisphere and it is not clear how this was handled; how ROIs defined: the dependent measure was the number of voxels in each ROI exceeding certain thresholds that differed across subjects depending on their strength of activation; it appears that increases and decreases may have been summed, though the description is hard to follow; 2 of the 11 patients were classified as outliers and excluded from analyses, however no plots are provided to justify their status as outliers	improved picture naming of untrained items was correlated with increased signal in all 4 ROIs; after removing the two outliers, none of the correlations remained significant
Raboyeau et al. (2008): Vox 1	Picture naming (native in patients; relearned foreign in controls) vs rest	LAC<br>(Aphasia T2 vs T1) vs (control T2 vs T1)	NAM	UNR	Vox<br>CA	Behavioral data notes: relearned foreign language was an attempt to equate to recovery in patients; still, patients improved less than controls, as shown by a significant interaction of group by time (p < .0001); search volume: whole brain; software: SPM2; voxelwise p: .01; cluster extent cutoff: 30 voxels (size not stated); nature of control contrast not clear; negative tail of contrast was masked to exclude lesioned areas, but the mask may have been more extensive than that	↑ L orbitofrontal
Raboyeau et al. (2008): Vox 2	Picture naming (native in patients; relearned foreign in controls) vs rest	LC<br>Aphasia T2 vs T1<br>Covariate: Δ picture naming accuracy	C	UNR	Vox<br>CA	Search volume: whole brain; software: SPM2; voxelwise p: .01; cluster extent cutoff: 30 voxels (size not stated); nature of control contrast not clear	↑ R insula<br>↑ R SMA/medial prefrontal<br>↑ R orbitofrontal<br>↑ R anterior cingulate<br>↓ L intraparietal sulcus<br>↓ L precuneus<br>↓ L posterior cingulate<br>↓ R dorsal precentral<br>↓ R precuneus
Richter et al. (2008): Vox 1	Reading words silently vs rest	CAC<br>Aphasia T1 vs control	UNR	UNR	Vox<br>M**	Search volume: R hemisphere; software: BrainVoyager QX 1.7; voxelwise p: R IFG/R insula ROI: .005; elsewhere: .001; cluster extent cutoff: R IFG/R insula ROI: 0.108 cc; elsewhere: none	↑ R IFG<br>↑ R insula
Richter et al. (2008): Vox 2	Word stem completion vs rest	CAC<br>Aphasia T1 vs control	UNR	UNR	Vox<br>M**	Search volume: R hemisphere; software: BrainVoyager QX 1.7; voxelwise p: R IFG/R insula ROI: .005; elsewhere: .001; cluster extent cutoff:	↑ R dorsal precentral

						R IFG/R insula ROI: 0.108 cc; elsewhere: none	
Richter et al. (2008): Vox 3	Reading words silently vs rest	CC<br>Aphasia T1<br>Covariate: subsequent Δ (T2 vs T1) overall language measure (composite measure of AAT spontaneous speech, token test, ANELT auditory comprehensibility, ANELT semantic comprehensibility)<br>Somewhat valid (T1 behavioral measure should be included in model)	UNR	UNR	Vox<br>NC	Search volume: R hemisphere; software: BrainVoyager QX 1.7; voxelwise p: .05; cluster extent cutoff: none; nature of thresholding not entirely clear, so coded according to best guess	↑ R IFG<br>↑ R insula<br>↑ R ventral precentral/inferior frontal junction<br>↑ R posterior MTG<br>notes: increased activity correlated with more behavioral improvement
Richter et al. (2008): Vox 4	Word stem completion vs rest	CC<br>Aphasia T1<br>Covariate: subsequent Δ (T2 vs T1) overall language measure (composite measure of AAT spontaneous speech, token test, ANELT auditory comprehensibility, ANELT semantic comprehensibility)<br>Somewhat valid (T1 behavioral measure should be included in model)	UNR	UNR	Vox<br>NC	Search volume: R hemisphere; software: BrainVoyager QX 1.7; voxelwise p: .05; cluster extent cutoff: none; nature of thresholding not entirely clear, so coded according to best guess	↑ R IFG<br>↑ R insula<br>notes: increased activity correlated with more behavioral improvement
Richter et al. (2008): Vox 5	Reading words silently vs rest	LA<br>Aphasia T2 vs T1	UNR	UNR	Vox<br>M**	Search volume: R hemisphere; software: BrainVoyager QX 1.7; voxelwise p: R IFG/R insula ROI: .005; elsewhere: .001; cluster extent cutoff: R IFG/R insula ROI: 0.108 cc; elsewhere: none	None
Richter et al. (2008): Vox 6	Word stem completion vs rest	LA<br>Aphasia T2 vs T1	UNR	UNR	Vox<br>M**	Search volume: R hemisphere; software: BrainVoyager QX 1.7; voxelwise p: R IFG/R insula ROI: .005; elsewhere: .001; cluster extent cutoff: R IFG/R insula ROI: 0.108 cc; elsewhere: none	None
Richter et al. (2008): ROI 1	Reading words silently vs rest	CC<br>Aphasia T1<br>Covariate: subsequent Δ (T2 vs T1) overall language measure (composite measure of AAT spontaneous speech, token test, ANELT auditory comprehensibility, ANELT semantic comprehensibility)<br>Somewhat valid (T1	UNR	UNR	ROI<br>Func<br>One	Number of ROIs: 1; ROI: L IFG/insula or L perilesional; how ROI defined: peak activations in individual patients in L IFG/insula or L perilesional regions (somewhat unclear)	None

		behavioral measure should be included in model)					
Richter et al. (2008): ROI 2	Word stem completion vs rest	CC<br>Aphasia T1<br>Covariate: subsequent Δ (T2 vs T1) overall language measure (composite measure of AAT spontaneous speech, token test, ANELT auditory comprehensibility, ANELT semantic comprehensibility)<br>Somewhat valid (T1 behavioral measure should be included in model)	UNR	UNR	ROI Func One	Number of ROIs: 1; ROI: L IFG/insula or L perilesional; how ROI defined: peak activations in individual patients in L IFG/insula or L perilesional regions (somewhat unclear)	None
Richter et al. (2008): ROI 3	Reading words silently vs rest	LC<br>Aphasia T2 vs T1<br>Covariate: Δ overall language measure (composite measure of AAT spontaneous speech, token test, ANELT auditory comprehensibility, ANELT semantic comprehensibility)	UNR	UNR	ROI Func NC	Number of ROIs: 4; ROIs: (1) R IFG/insula; (2) R precentral; (3) R MTG; (4) L IFG/insula or L perilesional; how ROIs defined: regions where T1 activation was correlated with subsequent improvement, along with the previously defined left hemisphere ROI; circular because functional ROIs based on related contrast on same data	↓ R posterior MTG<br>notes: decreased activity over time correlated with more behavioral improvement
Richter et al. (2008): ROI 4	Word stem completion vs rest	LC<br>Aphasia T2 vs T1<br>Covariate: Δ overall language measure (composite measure of AAT spontaneous speech, token test, ANELT auditory comprehensibility, ANELT semantic comprehensibility)	UNR	UNR	ROI Func NC	Number of ROIs: 3; ROIs: (1, 2) two clusters within R IFG/insula ROI; (3) L IFG/insula or L perilesional; how ROIs defined: regions where T1 activation was correlated with subsequent improvement, along with the previously defined left hemisphere ROI; circular because functional ROIs based on related contrast on same data	↓ R IFG<br>↓ R insula<br>notes: decreased activity over time correlated with more behavioral improvement
de Boissezon et al. (2009): Vox 1	Word generation vs rest	LA<br>Aphasia with "good recovery" (n = 6) T2 vs T1<br>Somewhat valid (the "good recovery" group showed more improvement than the "poor recovery" group in terms of accuracy on the task, but the distinction was not borne out in behavioral data more generally)	Y	UNR	Vox CA	Behavioral data notes: p = 0.07; search volume: whole brain; software: SPM2; voxelwise p: .001; cluster extent cutoff: 100 voxels (size not stated); contrast may not have included resting condition; inappropriate masking	↑ L ventral precentral/inferior frontal junction<br>↑ L SMA/medial prefrontal<br>↑ L posterior STG/STS/MTG<br>↑ R dorsolateral prefrontal cortex<br>↑ R SMA/medial prefrontal<br>↑ R angular gyrus<br>↑ R occipital<br>↑ R thalamus<br>↑ R basal ganglia<br>↓ L cerebellum<br>notes: based on coordinates in Table 5
de	Word generation	LA	Y	UNR	Vox	Search volume: whole brain; software:	↑ L ventral

Boissezon et al. (2009): Vox 2	vs rest	Aphasia with "poor recovery" (n = 7) T2 vs T1<br>Somewhat valid (the "poor recovery" group showed less improvement than the "good recovery" group in terms of accuracy on the task, but the distinction was not borne out in behavioral data more generally)			CA	SPM2; voxelwise p: .001; cluster extent cutoff: 100 voxels (size not stated); contrast may not have included resting condition; inappropriate masking	precentral/inferior frontal junction<br>↑ R somato-motor<br>↑ R cerebellum<br>↓ R basal ganglia
de Boissezon et al. (2009): Vox 3	Word generation vs rest	CC<br>Aphasia<br>Covariate: word generation accuracy	C	UNR	Vox<br>CA	Search volume: whole brain; software: SPM2; voxelwise p: .01; cluster extent cutoff: 100 voxels (size not stated); each patient's two sessions may be entered into the model without accounting for the dependence between them	↑ L supramarginal gyrus<br>↑ L occipital<br>↑ L anterior cingulate<br>↑ R insula<br>↑ R SMA/medial prefrontal<br>↑ R posterior STG<br>↑ R anterior temporal<br>↑ R occipital<br>↓ L cerebellum
Fridriksson et al. (2009): Vox 1	Picture naming (correct trials) vs viewing scrambled images	CAC<br>Aphasia vs control	YCT	UNR	Vox<br>C-	Search volume: voxels spared in all patients; software: FSL (FEAT 5.4); voxelwise p: ~.01 (z > 2.3); cluster extent cutoff: based on GRFT	None
Fridriksson et al. (2009): Vox 2	Picture naming (phonemic paraphasias) vs picture naming (correct trials)	CB<br>Aphasia	NBD	UNR	Vox<br>C-	Search volume: voxels spared in all patients; software: FSL (FEAT 5.4); voxelwise p: ~.01 (z > 2.3); cluster extent cutoff: based on GRFT	↑ L superior parietal<br>↑ L posterior inferior temporal gyrus/fusiform gyrus<br>↑ L occipital
Fridriksson et al. (2009): Vox 3	Picture naming (semantic paraphasias) vs picture naming (correct trials)	CB<br>Aphasia	NBD	UNR	Vox<br>C-	Search volume: voxels spared in all patients; software: FSL (FEAT 5.4); voxelwise p: ~.01 (z > 2.3); cluster extent cutoff: based on GRFT	↑ R posterior inferior temporal gyrus/fusiform gyrus<br>↑ R occipital
Fridriksson et al. (2009): ROI 1	Picture naming (correct trials) vs viewing scrambled images	CC<br>Aphasia<br>Covariate: picture naming accuracy	YCT	UNR	ROI<br>Func<br>NC	Number of ROIs: 5; ROIs: (1) R IFG/insula; (2) R motor/premotor; (3) R SMA; (4) R inferior parietal; (5) R superior temporal; how ROIs defined: regions activated for picture naming vs viewing scrambled images in aphasia	↑ R IFG<br>↑ R insula<br>notes: R IFG showed more activation in patients who produced more correct responses
Menke et al. (2009): Vox 1	Picture naming (trained items) vs rest	LC<br>Aphasia T2 vs T1<br>Covariate: subsequent outcome (T2) picture naming of trained items outside the scanner<br>Not valid (the logic behind correlating activation changes	UNT	UNR	Vox<br>M**	Search volume: whole brain; software: SPM2; voxelwise p: .05, but at least one voxel in the cluster had to be p < .001; cluster extent cutoff: 0.270 cc; there was an exclusive mask based on activation changes for untrained pictures, but it is unclear what the behavioral covariate was for the mask generation, nor were the regions in the mask reported	↑ L occipital<br>↑ L hippocampus/MTL<br>↑ R precuneus<br>↑ R occipital<br>↑ R posterior cingulate<br>↑ R hippocampus/MTL

		and language outcome is unclear)					
Menke et al. (2009): Vox 2	Picture naming (untrained items) vs rest	LC<br>Aphasia T3 vs T1<br>Covariate: subsequent outcome (T3) picture naming of trained items outside the scanner<br>Not valid (the logic behind correlating activation changes and language outcome is unclear)	UNT	UNR	Vox M**	Search volume: whole brain; software: SPM2; voxelwise p: .05, but at least one voxel in the cluster had to be p < .001; cluster extent cutoff: 0.270 cc; there was an exclusive mask based on activation changes for untrained pictures, but it is unclear what the behavioral covariate was for the mask generation, nor were the regions in the mask reported	↑ R posterior STG/STS/MTG<br>↓ L SMA/medial prefrontal<br>↓ R inferior parietal lobule<br>↓ R posterior inferior temporal gyrus/fusiform gyrus<br>↓ R basal ganglia
Specht et al. (2009): Vox 1	Lexical decision (words vs pseudowords) vs lexical decision (words vs reversed foreign words)	CAC<br>Aphasia vs control	UNR	UNR	Vox CA	Search volume: whole brain; software: SPM5; voxelwise p: .001; cluster extent cutoff: 0.64 cc	↑ R posterior STG<br>↑ R Heschl's gyrus<br>notes: activation is 1105 voxels (> 8 cc) so quite convincing, but when the contrast was examined in the patient group, this region was not activated.
Specht et al. (2009): Cplx 1	Lexical decision (words vs pseudowords) vs lexical decision (words vs reversed foreign words)	CAC<br>Aphasia vs control	UNR	UNR	Cplx	Joint ICA was performed on structural and functional contrast images using FIT 1.1b. Only 1 of the 8 components differed between groups in its loadings and was interpretable. The structural part of this component related to the patients' lesions. The functional part was thresholded at voxelwise p < .001 (CDT), arbitrary minimum cluster extent = 0.64 cc.	Other:<br>The component that differed between groups showed more activation for patients than controls in the L anterior temporal lobe, L cerebellum, R posterior STG, R anterior temporal lobe, R posterior inferior temporal gyrus/fusiform gyrus, R cerebellum, and R brainstem, and less activation in patients than controls in the L IFG, L anterior temporal lobe, L occipital lobe, L anterior cingulate, L cerebellum, L thalamus, and R IFG.
Warren et al. (2009): ROI 1	Listening to narrative speech vs listening to reversed speech	CAC<br>Aphasia vs control	NANB	NANT	ROI Anat NC	Number of ROIs: 6; ROIs: (1) L anterior superior temporal cortex; (2) L basal temporal language area; (3) L IFG pars triangularis; (4-6) homotopic counterparts; how ROIs defined: ROIs were defined anatomically in regions that were functionally connected with L anterior superior temporal cortex in controls (1-4) or homotopic to these	None<br>notes: L IFG pars triangularis almost reached significance (p = .053) for more activation in patients

						(5-6); somewhat circular because ROIs were defined only in regions where controls showed significant connectivity (even though ROIs were anatomical)	
Warren et al. (2009): ROI 2	Listening to narrative speech vs listening to reversed speech	CC<br>Aphasia<br>Covariate: auditory sentence comprehension	NANB	NANT	ROI<br>Anat<br>NC	Number of ROIs: 6; ROIs: (1) L anterior superior temporal cortex; (2) L basal temporal language area; (3) L IFG pars triangularis; (4-6) homotopic counterparts; how ROIs defined: ROIs were defined anatomically in regions that were functionally connected with L anterior superior temporal cortex in controls (1-4) or homotopic to these (5-6)	↑ L anterior temporal
Warren et al. (2009): ROI 3	Listening to narrative speech vs listening to reversed speech	CC<br>Aphasia<br>Covariate: written sentence comprehension	NANB	NANT	ROI<br>Anat<br>NC	Number of ROIs: 6; ROIs: (1) L anterior superior temporal cortex; (2) L basal temporal language area; (3) L IFG pars triangularis; (4-6) homotopic counterparts; how ROIs defined: ROIs were defined anatomically in regions that were functionally connected with L anterior superior temporal cortex in controls (1-4) or homotopic to these (5-6)	None
Warren et al. (2009): ROI 4	Listening to narrative speech vs listening to reversed speech	CC<br>Aphasia<br>Covariate: auditory single word comprehension	NANB	NANT	ROI<br>Anat<br>NC	Number of ROIs: 6; ROIs: (1) L anterior superior temporal cortex; (2) L basal temporal language area; (3) L IFG pars triangularis; (4-6) homotopic counterparts; how ROIs defined: ROIs were defined anatomically in regions that were functionally connected with L anterior superior temporal cortex in controls (1-4) or homotopic to these (5-6)	None<br>notes: L anterior temporal p = .08
Warren et al. (2009): ROI 5	Listening to narrative speech vs listening to reversed speech	CC<br>Aphasia<br>Covariate: auditory syntactic comprehension	NANB	NANT	ROI<br>Anat<br>NC	Number of ROIs: 6; ROIs: (1) L anterior superior temporal cortex; (2) L basal temporal language area; (3) L IFG pars triangularis; (4-6) homotopic counterparts; how ROIs defined: ROIs were defined anatomically in regions that were functionally connected with L anterior superior temporal cortex in controls (1-4) or homotopic to these (5-6)	None<br>notes: L anterior temporal p = .09
Warren et al. (2009): ROI 6	Listening to narrative speech vs listening to reversed speech	CC<br>Aphasia<br>Covariate: connectivity between L and R ATL	NANB	NANT	ROI<br>Anat<br>NC	Number of ROIs: 2; ROIs: (1) L anterior superior temporal cortex; (2) R anterior superior temporal cortex; how ROIs defined: ROIs were defined anatomically in regions that were functionally connected with L anterior superior temporal cortex in controls (1-4) or homotopic to these (5-6)	None
Warren et al. (2009): ROI 7	Listening to narrative speech vs listening to reversed speech	CC<br>Aphasia<br>Covariate: time post onset	NANB	NANT	ROI<br>Anat<br>One	Number of ROIs: 1; ROI: L anterior superior temporal cortex; how ROI defined: ROIs were defined anatomically in regions that were functionally connected with L anterior superior temporal cortex in controls (1-4) or homotopic to these (5-6)	None
Warren et	Listening to	CC	NANB	NANT	ROI	Number of ROIs: 1; ROI: L anterior	None

al. (2009): ROI 8	narrative speech vs listening to reversed speech	Aphasia Covariate: lesion volume			Anat One	superior temporal cortex; how ROI defined: ROIs were defined anatomically in regions that were functionally connected with L anterior superior temporal cortex in controls (1-4) or homotopic to these (5-6)	
Warren et al. (2009): ROI 9	Listening to narrative speech vs listening to reversed speech	CAC Aphasia with positive anterior temporal interconnectivity (n = 8) vs control	NANB	NANT	ROI Anat NC	Number of ROIs: 6; ROIs: (1) L anterior superior temporal cortex; (2) L basal temporal language area; (3) L IFG pars triangularis; (4-6) homotopic counterparts; how ROIs defined: ROIs were defined anatomically in regions that were functionally connected with L anterior superior temporal cortex in controls (1-4) or homotopic to these (5-6); somewhat circular because ROIs were defined only in regions where controls showed significant connectivity (even though ROIs were anatomical); excluded 3 patients with L IFG damage	↑ L IFG pars triangularis
Warren et al. (2009): ROI 10	Listening to narrative speech vs listening to reversed speech	CAC Aphasia with negative anterior temporal interconnectivity (n = 8) vs control	NANB	NANT	ROI Anat NC	Number of ROIs: 6; ROIs: (1) L anterior superior temporal cortex; (2) L basal temporal language area; (3) L IFG pars triangularis; (4-6) homotopic counterparts; how ROIs defined: ROIs were defined anatomically in regions that were functionally connected with L anterior superior temporal cortex in controls (1-4) or homotopic to these (5-6); somewhat circular because ROIs were defined only in regions where controls showed significant connectivity (even though ROIs were anatomical); excluded 1 patient with L IFG damage	None
Warren et al. (2009): ROI 11	Listening to narrative speech vs listening to reversed speech	CAA Aphasia with positive anterior temporal interconnectivity (n = 8) vs with negative anterior temporal interconnectivity (n = 8)	NANB	NANT	ROI Anat NC	Number of ROIs: 6; ROIs: (1) L anterior superior temporal cortex; (2) L basal temporal language area; (3) L IFG pars triangularis; (4-6) homotopic counterparts; how ROIs defined: ROIs were defined anatomically in regions that were functionally connected with L anterior superior temporal cortex in controls (1-4) or homotopic to these (5-6); excluded 4 patients with L IFG damage	↑ L IFG pars triangularis
Warren et al. (2009): Cplx 1	Listening to narrative speech vs listening to reversed speech	CC Aphasia Covariate: lesion status of each voxel	NANB	NANT	Cplx	VLSM with FDR correction was used to identify any regions in which damage was predictive of L anterior temporal activation.	None
Chau et al. (2010): Vox 1	Answering questions from Cantonese Aphasia Battery vs visual decision	LC Aphasia T2 vs T1 Covariate: Δ WAB AQ Somewhat valid (no treatment effect)	UNR	UNR	Vox U	Search volume: whole brain; software: SPM2; stated to be corrected $p < 0.05$, but the nature of correction is not described; it is not entirely clear whether the functional measure was the difference between T1 and T2 (we assume it is); it is also not clear whether or not 2 patients with low AQ were excluded (we assume not)	↑ L posterior MTG notes: finding based on table; additional small activations are shown in figure but not table
Fridriksson	Picture naming	LC	YCT	UNR	Vox	Search volume: whole brain; software:	↑ L dorsolateral

(2010): Vox 1	(correct trials) vs viewing abstract pictures	Aphasia T2 vs T1 Covariate: Δ picture naming accuracy			C-	FSL 4.1; voxelwise p: ~.01 (z > 2.3); cluster extent cutoff: based on GRFT	prefrontal cortex<br>↑ L ventral precentral/inferior frontal junction<br>↑ L supramarginal gyrus<br>↑ L intraparietal sulcus<br>↑ L superior parietal<br>↑ L precuneus<br>notes: activated regions were on the borders on the lesion distribution in the 19 included patients
Fridriksson et al. (2010): Vox 1	Picture naming (correct trials) vs viewing abstract pictures	CC Aphasia Covariate: picture naming accuracy	YCT	UNR	Vox C-	Search volume: whole brain; software: FSL 4.1; voxelwise p: ~.02 (z > 2); cluster extent cutoff: based on GRFT	↑ L IFG pars orbitalis<br>↑ L occipital<br>↑ L anterior cingulate<br>notes: greater activation was associated with better picture naming; L IFG pars orbitalis activation classified as middle frontal gyrus in the paper, but coordinates suggest otherwise
Fridriksson et al. (2010): Vox 2	Picture naming (correct trials) vs viewing abstract pictures	CAC Aphasia vs control	YCT	UNR	Vox C-	Search volume: whole brain; software: FSL 4.1; voxelwise p: ~.02 (z > 2); cluster extent cutoff: based on GRFT	None
Fridriksson et al. (2010): ROI 1	Picture naming (correct trials) vs viewing abstract pictures	CC Aphasia Covariate: picture naming accuracy	YCT	UNR	ROI Func One	Number of ROIs: 1; ROI: a single ROI comprising 3 regions where activation in patients was correlated with picture naming accuracy: the L IFG pars orbitalis, occipital lobe, and anterior cingulate; how ROI defined: based on SPM analysis 1; the purpose of this analysis was to determine whether these regions were recruited in the patients with better naming, or not activated in the patients with worse naming, relative to the control mean	Other:<br>patients with better naming showed greater activation than controls, while the patients with poorer naming showed less activation than controls.
Fridriksson et al. (2010): Cplx 1	Picture naming (correct trials) vs viewing abstract pictures	CC Aphasia Covariate: lesion status of each voxel	YCT	UNR	Cplx	VLSM was used to identify any regions in which damage was predictive of activation in the regions identified in SPM analysis 1, considered as a single ROI. There was no correction for multiple comparisons, and the analysis is appropriately presented as exploratory.	Other:<br>Only in the L IFG pars opercularis was damage predictive of reduced activation in the potentially compensatory network.
Sharp et al. (2010): ROI 1	Semantic decision (clear in patients; average of clear and noise vocoded	CAC Aphasia vs control	NAM	AS	ROI Oth NDC	Behavioral data notes: accuracy and RT were not significantly different for the semantic task; statistics are not reported for the syllable counting task,	Other:<br>patients showed greater connectivity

	in controls) vs syllable count decision (clear in patients; average of clear and noise vocoded in controls)					but the data provided suggest that accuracy was probably not matched, while RT probably was; number of ROIs: 12; ROIs: functional connectivity between pairs of spared nodes of the L hemisphere semantic network and R hemisphere homotopic regions: (1) L SFG-L AG; (2) L SFG-L IFG; (3) L SFG-L IT; (4) L AG-L IFG; (5) L AG-L IT; (6) L IFG-L IT; (7-12) homotopic counterparts; how ROIs defined: partial correlations between nodes	between L SFG and L AG than controls while performing the semantic task; this was not the case for the syllable counting task, however connectivity during performance of the two tasks was not compared directly
Thompson et al. (2010): ROI 1	Auditory sentence-picture matching (all three sentence types) vs rest	LA<br>Aphasia T2 vs T1	AS	AS	ROI<br>Anat<br>NC	Number of ROIs: 18; ROIs: (1) L BA 7; (2) L BA 9; (3) L BA 13; (4) L BA 21; (5) L BA 22; (6) L BA 39; (7) L BA 40; (8) L BA 44; (9) L BA 45; (10-18) homotopic counterparts; how ROIs defined: WFU pickatlas; proportion of patients who showed increases and decreases in (parts of) each ROI in individual fixed effects SPM analyses	↑ L angular gyrus<br>↑ L superior parietal<br>↑ L mid temporal<br>↑ R supramarginal gyrus<br>↑ R superior parietal<br>↓ L insula<br>↓ L posterior STG<br>notes: these are the regions involved in what the authors interpret as a "general shift"
Tyler et al. (2010): Vox 1	Listening to grammatical but meaningless sentences and detecting a target word vs listening to scrambled sentences and detecting a target word	CAC<br>Aphasia vs control	UNR	AS	Vox<br>NDC	Behavioral data notes: the two groups showed similar differences between RTs in the two conditions of the contrast; search volume: whole brain; software: SPM5; qualitative comparison on pp. 3402-3; each group is presented at voxelwise p < .005 (CDT), cluster-corrected p < .05 with GRFT	↑ R IFG pars triangularis<br>↑ R IFG pars orbitalis<br>↓ L posterior MTG<br>notes: several other potential differences are apparent in the figure, but only the differences tabulated are interpreted in the text
Tyler et al. (2010): ROI 1	Listening to grammatical but meaningless sentences and detecting a target word vs listening to scrambled sentences and detecting a target word	CC<br>Aphasia<br>Covariate: RT difference between early and late targets on grammatical but meaningless sentences (a measure of syntactic processing)	UNR	UNR	ROI<br>Func<br>One	Behavioral data notes: analyses focuses on RT differences between early and late targets, not on mean RT per se; number of ROIs: 1; ROI: L IFG pars triangularis and orbitalis; how ROI defined: activated for the same contrast	↑ L IFG pars triangularis<br>↑ L IFG pars orbitalis<br>notes: L IFG showed more activation in patients that had a larger target position effect (indicative of better syntactic processing)
Tyler et al. (2010): ROI 2	Listening to grammatical but meaningless sentences and	CC<br>Aphasia<br>Covariate: RT difference between	UNR	UNR	ROI<br>Func<br>One	Number of ROIs: 1; ROI: L IFG pars triangularis and orbitalis; how ROI defined: activated for the same contrast	None

	detecting a target word vs listening to scrambled sentences and detecting a target word	early and late targets on normal sentences					
Tyler et al. (2010): ROI 3	Listening to grammatical but meaningless sentences and detecting a target word vs listening to scrambled sentences and detecting a target word	CC<br>Aphasia<br>Covariate: RT difference between early and late targets on scrambled sentences	UNR	UNR	ROI<br>Func<br>One	Number of ROIs: 1; ROI: L IFG pars triangularis and orbitalis; how ROI defined: activated for the same contrast	None
Tyler et al. (2010): ROI 4	Listening to grammatical but meaningless sentences and detecting a target word vs listening to scrambled sentences and detecting a target word	CC<br>Aphasia<br>Covariate: damage to L IFG, estimated from T1 signal	UNR	UNR	ROI<br>Func<br>One	Number of ROIs: 1; ROI: R IFG pars triangularis and orbitalis; how ROI defined: activated for the same contrast	None<br>notes: no correlation (p = .57)
Tyler et al. (2010): ROI 5	Listening to grammatical but meaningless sentences and detecting a target word vs listening to scrambled sentences and detecting a target word	CC<br>Aphasia<br>Covariate: syntactic processing (presumably the target position effect, though this is not stated)	UNR	UNR	ROI<br>Func<br>One	Number of ROIs: 1; ROI: R IFG pars triangularis and orbitalis; how ROI defined: activated for the same contrast	None<br>notes: no correlation (p = .41)
Tyler et al. (2010): Cplx 1	Listening to grammatical but meaningless sentences and detecting a target word vs listening to scrambled sentences and detecting a target word	CC<br>Aphasia<br>Covariate: lesion status of each voxel	UNR	UNR	Cplx	VBM was used to identify any regions where damage was predictive of activation in the L IFG pars triangularis and orbitalis. Tissue integrity was quantified in terms of T1 signal. Clusterwise correction was used, which is not appropriate for VBM.	Other:<br>Only in the L IFG itself was damage predictive of reduced activation in the L IFG.
van Oers et al. (2010): ROI 1	Written word-picture matching vs visual decision	CAC<br>Aphasia vs control	UNR	UNR	ROI<br>Mix<br>NC	Behavioral data notes: accuracy not reported for control condition; number of ROIs: 7; ROIs: (1) L anterior language region (IFG); (2) L posterior language region (AG, SMG, STG, MTG); (3) R anterior language region (IFG); (4) R posterior language region (AG, SMG, STG, MTG); (5) frontal LI; (6) temporal LI; (7) whole network LI; how ROIs defined: WFU pickatlas	↓ L IFG<br>↓ LI (language network)<br>↓ LI (frontal)
van Oers et al. (2010): ROI 2	Semantic decision vs visual decision	CAC<br>Aphasia vs control	UNR	UNR	ROI<br>Mix<br>NC	Behavioral data notes: accuracy not reported for control condition; number of ROIs: 7; ROIs: (1) L anterior language region (IFG); (2) L posterior language region (AG, SMG, STG, MTG);	↓ L IFG<br>↓ LI (language network)<br>↓ LI (frontal)

						(3) R anterior language region (IFG); (4) R posterior language region (AG, SMG, STG, MTG); (5) frontal LI; (6) temporal LI; (7) whole network LI; how ROIs defined: WFU pickatlas	
van Oers et al. (2010): ROI 3	Verb generation vs rest	CAC<br>Aphasia vs control	UNR	UNR	ROI<br>Mix<br>NC	Number of ROIs: 7; ROIs: (1) L anterior language region (IFG); (2) L posterior language region (AG, SMG, STG, MTG); (3) R anterior language region (IFG); (4) R posterior language region (AG, SMG, STG, MTG); (5) frontal LI; (6) temporal LI; (7) whole network LI; how ROIs defined: WFU pickatlas	↓ L IFG<br>↓ LI (language network)<br>↓ LI (frontal)
van Oers et al. (2010): ROI 4	Written word-picture matching vs visual decision	CC<br>Aphasia<br>Covariate: picture-word matching accuracy	C	UNR	ROI<br>Mix<br>NC	Number of ROIs: 7; ROIs: (1) L anterior language region (IFG); (2) L posterior language region (AG, SMG, STG, MTG); (3) R anterior language region (IFG); (4) R posterior language region (AG, SMG, STG, MTG); (5) frontal LI; (6) temporal LI; (7) whole network LI; how ROIs defined: WFU pickatlas	None
van Oers et al. (2010): ROI 5	Semantic decision vs visual decision	CC<br>Aphasia<br>Covariate: semantic decision accuracy	C	UNR	ROI<br>Mix<br>NC	Number of ROIs: 7; ROIs: (1) L anterior language region (IFG); (2) L posterior language region (AG, SMG, STG, MTG); (3) R anterior language region (IFG); (4) R posterior language region (AG, SMG, STG, MTG); (5) frontal LI; (6) temporal LI; (7) whole network LI; how ROIs defined: WFU pickatlas	None
van Oers et al. (2010): ROI 6	Written word-picture matching vs visual decision	CC<br>Aphasia<br>Covariate: overall language measure	UNR	UNR	ROI<br>Mix<br>NC	Number of ROIs: 7; ROIs: (1) L anterior language region (IFG); (2) L posterior language region (AG, SMG, STG, MTG); (3) R anterior language region (IFG); (4) R posterior language region (AG, SMG, STG, MTG); (5) frontal LI; (6) temporal LI; (7) whole network LI; how ROIs defined: WFU pickatlas	None
van Oers et al. (2010): ROI 7	Semantic decision vs visual decision	CC<br>Aphasia<br>Covariate: overall language measure	UNR	UNR	ROI<br>Mix<br>NC	Number of ROIs: 7; ROIs: (1) L anterior language region (IFG); (2) L posterior language region (AG, SMG, STG, MTG); (3) R anterior language region (IFG); (4) R posterior language region (AG, SMG, STG, MTG); (5) frontal LI; (6) temporal LI; (7) whole network LI; how ROIs defined: WFU pickatlas; not clear if it was LI for whole language network	↑ LI (language network)
van Oers et al. (2010): ROI 8	Verb generation vs rest	CC<br>Aphasia<br>Covariate: overall language measure	UNR	UNR	ROI<br>Mix<br>NC	Number of ROIs: 7; ROIs: (1) L anterior language region (IFG); (2) L posterior language region (AG, SMG, STG, MTG); (3) R anterior language region (IFG); (4) R posterior language region (AG, SMG, STG, MTG); (5) frontal LI; (6) temporal LI; (7) whole network LI; how ROIs defined: WFU pickatlas	None
van Oers et al. (2010): ROI 9	Written word-picture matching vs visual decision	CC<br>Aphasia<br>Covariate: lesion volume	UNR	UNR	ROI<br>Anat<br>NC	Number of ROIs: 2; ROIs: (1) R anterior language region (IFG); (2) R posterior language region (AG, SMG, STG, MTG); how ROIs defined: WFU pickatlas	None
van Oers et al. (2010): ROI 10	Semantic decision vs visual decision	CC<br>Aphasia	UNR	UNR	ROI<br>Anat<br>NC	Number of ROIs: 2; ROIs: (1) R anterior language region (IFG); (2) R posterior	None

		Covariate: lesion volume				language region (AG, SMG, STG, MTG); how ROIs defined: WFU pickatlas	
van Oers et al. (2010): ROI 11	Verb generation vs rest	CC<br>Aphasia<br>Covariate: lesion volume	UNR	UNR	ROI<br>Anat<br>NC	Number of ROIs: 2; ROIs: (1) R anterior language region (IFG); (2) R posterior language region (AG, SMG, STG, MTG); how ROIs defined: WFU pickatlas	None
van Oers et al. (2010): ROI 12	Written word-picture matching vs visual decision	CC<br>Aphasia<br>Covariate: damage to L hemisphere language regions	UNR	UNR	ROI<br>Anat<br>NC	Number of ROIs: 2; ROIs: (1) R anterior language region (IFG); (2) R posterior language region (AG, SMG, STG, MTG); how ROIs defined: WFU pickatlas	None
van Oers et al. (2010): ROI 13	Semantic decision vs visual decision	CC<br>Aphasia<br>Covariate: damage to L hemisphere language regions	UNR	UNR	ROI<br>Anat<br>NC	Number of ROIs: 2; ROIs: (1) R anterior language region (IFG); (2) R posterior language region (AG, SMG, STG, MTG); how ROIs defined: WFU pickatlas	None
van Oers et al. (2010): ROI 14	Verb generation vs rest	CC<br>Aphasia<br>Covariate: damage to L hemisphere language regions	UNR	UNR	ROI<br>Anat<br>NC	Number of ROIs: 2; ROIs: (1) R anterior language region (IFG); (2) R posterior language region (AG, SMG, STG, MTG); how ROIs defined: WFU pickatlas	None
van Oers et al. (2010): ROI 15	Written word-picture matching vs visual decision	CC<br>Aphasia<br>Covariate: previous (current vs subacute) Δ naming<br>Not valid (current activation will reflect not just prior recovery, but also current language function)	UNR	UNR	ROI<br>Anat<br>NC	Number of ROIs: 7; ROIs: (1) L anterior language region (IFG); (2) L posterior language region (AG, SMG, STG, MTG); (3) R anterior language region (IFG); (4) R posterior language region (AG, SMG, STG, MTG); (5) frontal LI; (6) temporal LI; (7) whole network LI; how ROIs defined: WFU pickatlas	None
van Oers et al. (2010): ROI 16	Semantic decision vs visual decision	CC<br>Aphasia<br>Covariate: previous (current vs subacute) Δ naming<br>Not valid (current activation will reflect not just prior recovery, but also current language function)	UNR	UNR	ROI<br>Anat<br>NC	Number of ROIs: 7; ROIs: (1) L anterior language region (IFG); (2) L posterior language region (AG, SMG, STG, MTG); (3) R anterior language region (IFG); (4) R posterior language region (AG, SMG, STG, MTG); (5) frontal LI; (6) temporal LI; (7) whole network LI; how ROIs defined: WFU pickatlas	↑ L IFG
van Oers et al. (2010): ROI 17	Verb generation vs rest	CC<br>Aphasia<br>Covariate: previous (current vs subacute) Δ naming<br>Not valid (current activation will reflect not just prior recovery, but also current language function)	UNR	UNR	ROI<br>Anat<br>NC	Number of ROIs: 7; ROIs: (1) L anterior language region (IFG); (2) L posterior language region (AG, SMG, STG, MTG); (3) R anterior language region (IFG); (4) R posterior language region (AG, SMG, STG, MTG); (5) frontal LI; (6) temporal LI; (7) whole network LI; how ROIs defined: WFU pickatlas	↑ L IFG
van Oers et al. (2010): ROI 18	Written word-picture matching vs visual decision	CC<br>Aphasia<br>Covariate: previous (current vs subacute) Δ TT<br>Not valid (current activation will reflect	UNR	UNR	ROI<br>Anat<br>NC	Number of ROIs: 7; ROIs: (1) L anterior language region (IFG); (2) L posterior language region (AG, SMG, STG, MTG); (3) R anterior language region (IFG); (4) R posterior language region (AG, SMG, STG, MTG); (5) frontal LI; (6) temporal	None

		not just prior recovery, but also current language function; TT not optimal measure of overall language function)				LI; (7) whole network LI; how ROIs defined: WFU pickatlas	
van Oers et al. (2010): ROI 19	Semantic decision vs visual decision	CC<br>Aphasia<br>Covariate: previous (current vs subacute) Δ TT<br>Not valid (current activation will reflect not just prior recovery, but also current language function; TT not optimal measure of overall language function)	UNR	UNR	ROI<br>Anat<br>NC	Number of ROIs: 7; ROIs: (1) L anterior language region (IFG); (2) L posterior language region (AG, SMG, STG, MTG); (3) R anterior language region (IFG); (4) R posterior language region (AG, SMG, STG, MTG); (5) frontal LI; (6) temporal LI; (7) whole network LI; how ROIs defined: WFU pickatlas	↑ L IFG<br>↑ R IFG
van Oers et al. (2010): ROI 20	Verb generation vs rest	CC<br>Aphasia<br>Covariate: previous (current vs subacute) Δ TT<br>Not valid (current activation will reflect not just prior recovery, but also current language function; TT not optimal measure of overall language function)	UNR	UNR	ROI<br>Anat<br>NC	Number of ROIs: 7; ROIs: (1) L anterior language region (IFG); (2) L posterior language region (AG, SMG, STG, MTG); (3) R anterior language region (IFG); (4) R posterior language region (AG, SMG, STG, MTG); (5) frontal LI; (6) temporal LI; (7) whole network LI; how ROIs defined: WFU pickatlas	↑ L IFG<br>↑ R IFG
Papoutsi et al. (2011): Vox 1	Listening to ambiguous sentences with subordinate resolution ("subordinate") vs listening to ambiguous sentences with dominant resolution ("dominant")	CC<br>Aphasia<br>Covariate: difference in percent of unacceptable judgments between subordinate and dominant sentences (dominance effect)	NANB	NANT	Vox<br>C-	Search volume: whole brain; software: SPM8; voxelwise p: .01; cluster extent cutoff: based on GRFT	↑ L insula<br>↑ L posterior STG/STS/MTG<br>↑ L mid temporal
Papoutsi et al. (2011): Cplx 1	Listening to ambiguous sentences with subordinate resolution ("subordinate") vs listening to ambiguous sentences with dominant resolution ("dominant")	CC<br>Aphasia<br>Covariate: modulation of L IFG connectivity by dominance effect	NANB	NANT	Cplx	A PPI analysis was carried out with the L IFG as the seed region. Correlations were computed between voxelwise modulation of connectivity with this region, and a behavioral measure of syntactic processing, which was the dominance effect: the difference in percent of unacceptable judgments between subordinate and dominant sentences. The resultant SPM was thresholded at voxelwise p < .01 (CDT), then corrected for multiple corrections based on cluster extent and GRFT using SPM8.	Other: patients with better syntactic performance had more connectivity from the L IFG seed region to L pMTG and adjacent areas (including the insula); pMTG also significant at voxelwise p < .001 in Figure 2B, corrected for

							multiple comparisons with GRFT
Papoutsi et al. (2011): Cplx 2	Listening to ambiguous sentences with subordinate resolution ("subordinate") vs listening to ambiguous sentences with dominant resolution ("dominant")	CC<br>Aphasia<br>Covariate: modulation of L pMTG connectivity by dominance effect	NANB	NANT	Cplx	A similar PPI analysis was carried out with the L pMTG as the seed region. Thresholding was the same as in the previous analysis.	None
Sebastian & Kiran (2011): ROI 1	Picture naming (correct trials) vs viewing scrambled images and saying "pass"	CC<br>Aphasia<br>Covariate: lesion volume	YCT	UNR	ROI<br>Mix<br>NC	Number of ROIs: 4; ROIs: (1) L IFG (oper/tri); (2) L posterior perisylvian (pSTG, pMTG, AG, SMG); (3) R IFG (oper/tri); (4) R posterior perisylvian (pSTG, pMTG, AG, SMG); (5) language network LI; how ROIs defined: Harvard–Oxford atlas	↑ R supramarginal gyrus<br>↑ R angular gyrus<br>↑ R posterior STG/STS/MTG<br>↓ LI (language network)<br>notes: larger lesions were associated with more R posterior perisylvian activation
Sebastian & Kiran (2011): ROI 2	Semantic decision (correct trials) vs visual decision	CC<br>Aphasia<br>Covariate: lesion volume	YCT	UNR	ROI<br>Mix<br>NC	Number of ROIs: 4; ROIs: (1) L IFG (oper/tri); (2) L posterior perisylvian (pSTG, pMTG, AG, SMG); (3) R IFG (oper/tri); (4) R posterior perisylvian (pSTG, pMTG, AG, SMG); (5) language network LI; how ROIs defined: Harvard–Oxford atlas	None
Szaflarski et al. (2011): Vox 1	Semantic decision vs tone decision	LA<br>Aphasia T2 vs T1<br>Somewhat valid (patients improved only on semantic fluency)	Y	UNR	Vox<br>NC	Behavioral data notes: language and control tasks both matched; search volume: whole brain; software: in-house; voxelwise p: .05; cluster extent cutoff: none; the figure shows a cutoff of z > 10, which would not correspond to p < .05; increases and decreases in Figure 3 do not accord with the data from T1 and T2 in Figure 2, raising concerns about the implementation of the analyses; there is no explicit description of the second level analysis	↑ L IFG<br>↑ L SMA/medial prefrontal<br>↑ L orbitofrontal<br>↑ L inferior parietal lobule<br>↑ L supramarginal gyrus<br>↑ L angular gyrus<br>↑ L precuneus<br>↑ L occipital<br>↑ L anterior cingulate<br>↑ L basal ganglia<br>↑ L hippocampus/MTL<br>↑ R dorsal precentral<br>↑ R precuneus<br>↑ R occipital<br>↑ R basal ganglia<br>↑ R hippocampus/MTL<br>↓ R insula<br>↓ R supramarginal gyrus<br>↓ R posterior STG

							notes: based on a combination of coordinates in Table 2, and Figure 3
Szaflarski et al. (2011): ROI 1	Semantic decision vs tone decision	LA<br>Aphasia T2 vs T1<br>Somewhat valid (patients improved only on semantic fluency)	Y	UNR	ROI Func<br>NC	Behavioral data notes: language and control tasks both matched; number of ROIs: 3; ROIs: (1) frontal LI; (2) temporal LI; (3) language network LI; T1 LI (temporal) is reported to be negative, which does not accord with the voxelwise analysis in Figure 2; increases and decreases in Figure 3 do not accord with the data from T1 and T2 in Figure 2, raising concerns about the implementation of the analyses	↑ LI (language network)<br>↑ LI (frontal)<br>↑ LI (temporal)
Tyler et al. (2011): Vox 1	Listening to ambiguous sentences (dominant and subordinate) vs listening to unambiguous sentences ("unambiguous")	CAC<br>Aphasia vs control	NANB	NANT	Vox<br>NDC	Search volume: plausible fronto-temporo-parietal language regions; software: SPM5; qualitative comparison on p. 423	↓ L IFG
Tyler et al. (2011): Vox 2	Listening to ambiguous sentences with dominant resolution ("dominant") vs listening to unambiguous sentences ("unambiguous")	CAC<br>Aphasia vs control	NANB	NANT	Vox<br>NDC	Search volume: plausible fronto-temporo-parietal language regions; software: SPM5; qualitative comparison on p. 423	↓ L IFG
Tyler et al. (2011): Vox 3	Listening to ambiguous sentences with subordinate resolution ("subordinate") vs listening to unambiguous sentences ("unambiguous")	CAC<br>Aphasia vs control	NANB	NANT	Vox<br>NDC	Search volume: plausible fronto-temporo-parietal language regions; software: SPM5; qualitative comparison on p. 423	↓ L IFG<br>notes: lack of patient activation in pMTG implied in text, but this activation looks fairly similar in patients and controls (c.f. Figure 3C vs 2C)
Tyler et al. (2011): Vox 4	Listening to ambiguous sentences with subordinate resolution ("subordinate") vs listening to ambiguous sentences with dominant resolution ("dominant")	CAC<br>Aphasia vs control	NANB	NANT	Vox<br>NDC	Search volume: plausible fronto-temporo-parietal language regions; software: SPM5; qualitative comparison on p. 423	↓ L IFG<br>↓ L posterior MTG
Tyler et al. (2011): Vox 5	Listening to ambiguous sentences (dominant and	CC<br>Aphasia<br>Covariate: performance on	NANB	NANT	Vox<br>C-	Search volume: plausible fronto-temporo-parietal language regions; software: SPM5; voxelwise p: .01; cluster extent cutoff: based on GRFT	↑ L IFG pars triangularis<br>↑ L IFG pars orbitalis

	subordinate) vs listening to unambiguous sentences ("unambiguous")	acceptability judgment task (difference in percent of unacceptable judgments between ambiguous and unambiguous sentences)					↑ R insula<br>↑ R mid temporal<br>notes: also L pMTG but this did not reach significance
Tyler et al. (2011): Vox 6	Listening to ambiguous sentences (dominant and subordinate) vs listening to unambiguous sentences ("unambiguous")	CC<br>Aphasia<br>Covariate: performance on sentence-picture matching task	NANB	NANT	Vox<br>CA	Search volume: plausible fronto-temporo-parietal language regions; software: SPM5; voxelwise p: .01; cluster extent cutoff: 30 (units not stated)	↑ L IFG pars orbitalis<br>↑ L posterior MTG<br>↑ R insula<br>↑ R posterior STG<br>↑ R mid temporal
Tyler et al. (2011): Vox 7	Listening to ambiguous sentences (dominant and subordinate) vs listening to unambiguous sentences ("unambiguous")	CC<br>Aphasia<br>Covariate: performance on word monitoring task	NANB	NANT	Vox<br>CA	Search volume: plausible fronto-temporo-parietal language regions; software: SPM5; voxelwise p: .05; cluster extent cutoff: 10 (units not stated)	↑ L IFG pars orbitalis<br>↑ L posterior MTG<br>↑ R insula<br>↑ R mid temporal
Tyler et al. (2011): Vox 8	Listening to ambiguous sentences (dominant and subordinate) vs listening to unambiguous sentences ("unambiguous")	CC<br>Aphasia<br>Covariate: difference in percent of unacceptable judgments between subordinate and dominant sentences (dominance effect)	NANB	NANT	Vox<br>C-	Search volume: plausible fronto-temporo-parietal language regions; software: SPM5; voxelwise p: .01; cluster extent cutoff: based on GRFT	None
Tyler et al. (2011): ROI 1	Listening to ambiguous sentences (dominant and subordinate) vs listening to unambiguous sentences ("unambiguous")	CC<br>Aphasia<br>Covariate: performance on acceptability judgment task (difference in percent of unacceptable judgments between ambiguous and unambiguous sentences)	NANB	NANT	ROI<br>Anat<br>NC	Number of ROIs: 3; ROIs: (1) IFG pars opercularis; (2) IFG pars triangularis; (3) IFG pars orbitalis; how ROIs defined: AAL	↑ L IFG pars triangularis<br>↑ L IFG pars orbitalis
Tyler et al. (2011): ROI 2	Listening to ambiguous sentences (dominant and subordinate) vs listening to unambiguous sentences ("unambiguous")	CC<br>Aphasia<br>Covariate: difference in percentage of unacceptable judgments between subordinate and dominant sentences (dominance effect)	NANB	NANT	ROI<br>Anat<br>NC	Number of ROIs: 3; ROIs: (1) IFG pars opercularis; (2) IFG pars triangularis; (3) IFG pars orbitalis; how ROIs defined: AAL	None
Weiduschat et al. (2011): ROI 1	Verb generation vs rest	LA<br>Aphasia T2 vs T1 (regardless of rTMS)	UNR	UNR	ROI<br>LI<br>NC	Number of ROIs: 3; ROIs: (1) IFG LI; (2) superior temporal LI; (3) SMA LI	None
Weiduschat	Verb generation vs	LA	UNR	UNR	ROI	Number of ROIs: 3; ROIs: (1) IFG LI; (2)	None

et al. (2011): ROI 2	rest	Aphasia treated with rTMS (n = 6) T2 vs T1			LI NC	superior temporal LI; (3) SMA LI	
Weiduschat et al. (2011): ROI 3	Verb generation vs rest	LAA (Aphasia with R IFG rTMS (n = 6) T2 vs T1) vs (with sham rTMS (n = 4) T2 vs T1)	UNR	UNR	ROI LI NC	Number of ROIs: 3; ROIs: (1) IFG LI; (2) superior temporal LI; (3) SMA LI	↑ LI (frontal) notes: IFG LI was stable in the stimulation group, but shifted to the R in the sham group, yielding a significant difference between groups
Weiduschat et al. (2011): ROI 4	Verb generation vs rest	LC Aphasia T2 vs T1 (regardless of rTMS) Covariate: Δ AAT total score	UNR	UNR	ROI LI One	Number of ROIs: 1; ROI: IFG LI	None
Allendorfer et al. (2012): ROI 1	Verb generation (covert, block) vs finger tapping (block)	CAC Aphasia vs control	UNR	UNR	ROI LI NC	Number of ROIs: 2; ROIs: (1) frontal LI; (2) temporal LI	↓ LI (temporal)
Allendorfer et al. (2012): ROI 2	Verb generation (overt, event-related) vs noun repetition (event-related)	CAC Aphasia vs control	N	UNR	ROI LI NC	Behavioral data notes: patients less accurate and produced less responses on both conditions, but the difference between groups was greater for verb generation; number of ROIs: 2; ROIs: (1) frontal LI; (2) temporal LI	↓ LI (frontal)
Allendorfer et al. (2012): ROI 3	Verb generation (overt, event-related) vs verb generation (covert, event-related)	CAC Aphasia vs control	N	UNR	ROI LI NC	Behavioral data notes: overt performance differed, so covert performance probably did too; number of ROIs: 2; ROIs: (1) frontal LI; (2) temporal LI	None notes: lack of lateralization in controls makes this analysis difficult to interpret
Allendorfer et al. (2012): ROI 4	Verb generation (overt, event-related) vs noun repetition (event-related)	CC Aphasia Covariate: overt verb generation accuracy	C	UNR	ROI Func NC	Number of ROIs: 3; ROIs: (1) L MTG; (2) L SFG/CG; (3) left MFG; how ROIs defined: regions activated by the contrast of overt verb generation vs noun repetition in patients	↑ L dorsolateral prefrontal cortex ↑ L SMA/medial prefrontal
Allendorfer et al. (2012): ROI 5	Verb generation (overt, event-related) vs verb generation (covert, event-related)	CC Aphasia Covariate: overt verb generation accuracy	C	UNR	ROI Func NC	Number of ROIs: 2; ROIs: (1) R insula/IFG; (2) R STG; how ROIs defined: prominent R hemisphere activations for the contrast of overt and covert verb generation in patients	None
Fridriksson, Hubbard, et al. (2012): Vox 1	Listening to/watching audiovisual sentences, while producing the same sentences in unison (speech entrainment) vs listening to reversed sentences and viewing a mouth speaking, while producing unrelated sentences	CAC Aphasia T1 vs control	UNR	NANT	Vox U	Search volume: whole brain; software: FSL (FEAT 5.98); thresholding not stated	↑ L angular gyrus ↓ L anterior temporal notes: based on coordinates in Table 2

Fridriksson, Hubbard, et al. (2012): Vox 2	Listening to/watching audiovisual sentences, while producing the same sentences in unison (speech entrainment) vs rest	LA<br>Aphasia T2 vs T1	UNR	NANT	Vox<br>U	Search volume: whole brain; software: FSL (FEAT 5.98); thresholding not stated	↑ L SMA/medial prefrontal<br>↑ L anterior cingulate<br>↑ R precuneus<br>↑ R occipital<br>↑ R hippocampus/MTL<br>↓ L supramarginal gyrus<br>notes: some labels changed based on coordinates
Fridriksson, Hubbard, et al. (2012): Vox 3	Listening to reversed sentences and viewing a mouth speaking, while producing unrelated sentences vs rest	LA<br>Aphasia T2 vs T1	UNR	NANT	Vox<br>U	Search volume: whole brain; software: FSL (FEAT 5.98); thresholding not stated	None
Fridriksson, Hubbard, et al. (2012): Vox 4	Listening to/watching audiovisual sentences and viewing a mouth vs rest	LA<br>Aphasia T2 vs T1	NANB	NANT	Vox<br>U	Search volume: whole brain; software: FSL (FEAT 5.98); thresholding not stated	None
Fridriksson, Hubbard, et al. (2012): ROI 1	Listening to/watching audiovisual sentences, while producing the same sentences in unison (speech entrainment) vs listening to reversed sentences and viewing a mouth speaking, while producing unrelated sentences	CAC<br>Aphasia T1 vs control	UNR	NANT	ROI<br>Func<br>NC	Number of ROIs: 6; ROIs: (1) L anterior insula/IFG pars orbitalis; (2) R anterior insula/IFG pars orbitalis; (3) Broca's area; (4) L MTG; (5) L BA 37; (6) R BA 37; how ROIs defined: regions activated in both groups considered together; there were no interactions of group by condition; two regions showed main effects of group but this is not pertinent to the contrast	None
Fridriksson, Richardson, et al. (2012): ROI 1	Picture naming vs viewing abstract pictures	LC<br>Aphasia T2 vs T1<br>Covariate: Δ picture naming accuracy	C	UNR	ROI<br>Oth<br>NC	Number of ROIs: 3; ROIs: (1) perilesional L hemisphere language regions; (2) perilesional L hemisphere non-language regions; (3) undamaged non-perilesional L hemisphere language regions; how ROIs defined: based on individual lesions and control activation for picture naming	Other:<br>change in perilesional non-language regions positively correlated with improvement in accuracy
Fridriksson, Richardson, et al. (2012): ROI 2	Picture naming vs viewing abstract pictures	LC<br>Aphasia T2 vs T1<br>Covariate: Δ (decrease in) semantic errors	UNR	UNR	ROI<br>Oth<br>NC	Number of ROIs: 3; ROIs: (1) perilesional L hemisphere language regions; (2) perilesional L hemisphere non-language regions; (3) undamaged non-perilesional L hemisphere language regions; how ROIs defined: based on individual lesions and control activation for picture naming	Other:<br>change in undamaged non-perilesional language regions negatively correlated with decrease in semantic errors
Fridriksson,	Picture naming vs	LC	UNR	UNR	ROI	Number of ROIs: 3; ROIs: (1)	Other:

Richardson, et al. (2012): ROI 3	viewing abstract pictures	Aphasia T2 vs T1 Covariate: Δ (decrease in) phonological paraphasias			Oth NC	perilesional L hemisphere language regions; (2) perilesional L hemisphere non-language regions; (3) undamaged non-perilesional L hemisphere language regions; how ROIs defined: based on individual lesions and control activation for picture naming	change in perilesional language regions, and change in undamaged non-perilesional language regions, negatively correlated with decrease in phonological paraphasias
Fridriksson, Richardson, et al. (2012): ROI 4	Picture naming vs viewing abstract pictures	CC Aphasia T1 Covariate: subsequent Δ (T2 vs T1) picture naming accuracy Somewhat valid (T1 behavioral measure should be included in model)	UNR	UNR	ROI Oth NC	Number of ROIs: 3; ROIs: (1) perilesional L hemisphere language regions; (2) perilesional L hemisphere non-language regions; (3) undamaged non-perilesional L hemisphere language regions; how ROIs defined: based on individual lesions and control activation for picture naming	None
Fridriksson, Richardson, et al. (2012): ROI 5	Picture naming vs viewing abstract pictures	CC Aphasia T1 Covariate: subsequent Δ (T2 vs T1, decrease in) semantic errors Somewhat valid (T1 behavioral measure should be included in model)	UNR	UNR	ROI Oth NC	Number of ROIs: 3; ROIs: (1) perilesional L hemisphere language regions; (2) perilesional L hemisphere non-language regions; (3) undamaged non-perilesional L hemisphere language regions; how ROIs defined: based on individual lesions and control activation for picture naming	Other: change in perilesional language regions correlated with decrease in phonological paraphasias
Fridriksson, Richardson, et al. (2012): ROI 6	Picture naming vs viewing abstract pictures	CC Aphasia T1 Covariate: subsequent Δ (T2 vs T1, decrease in) phonological paraphasias Somewhat valid (T1 behavioral measure should be included in model)	UNR	UNR	ROI Oth NC	Number of ROIs: 3; ROIs: (1) perilesional L hemisphere language regions; (2) perilesional L hemisphere non-language regions; (3) undamaged non-perilesional L hemisphere language regions; how ROIs defined: based on individual lesions and control activation for picture naming	None
Marcotte et al. (2012): Vox 1	Picture naming (T1: known items; T2: trained items; correct trials) vs viewing scrambled images and saying "baba"	LA Aphasia T2 vs T1	YCT	UNR	Vox NDC	Search volume: whole brain; software: SPM5; qualitative comparison on p. 1780; different contrasts at different time points not clearly explained	↑ L supramarginal gyrus<br>↓ L dorsal precentral<br>↓ L posterior MTG<br>notes: labels based on figures rather than text
Marcotte et al. (2012): Vox 2	Picture naming (known items, correct trials) vs viewing scrambled images and saying "baba"	CC Aphasia T1 Covariate: subsequent Δ (T2 vs T1) naming of trained items Somewhat valid (T1 behavioral measure should be included in model)	YCT	UNR	Vox CA	Search volume: whole brain; software: SPM5; voxelwise p: .005; cluster extent cutoff: 10 voxels (size not stated); different contrasts at different time points not clearly explained	↑ L dorsolateral prefrontal cortex<br>↑ L SMA/medial prefrontal<br>↑ L somato-motor<br>↑ L anterior cingulate<br>↑ R dorsolateral prefrontal cortex<br>↑ R somato-motor<br>↑ R thalamus

							notes: labels based on figures and text
Marcotte et al. (2012): Vox 3	Picture naming (trained items, correct trials) vs viewing scrambled images and saying "baba"	CC<br>Aphasia T2<br>Covariate: previous Δ (T2 vs T1) naming of trained items<br>Not valid (T2 activation not an appropriate measure of treatment-induced recovery because it reflects T2 performance)	YCT	UNR	Vox<br>CA	Search volume: whole brain; software: SPM5; voxelwise p: .005; cluster extent cutoff: 10 voxels (size not stated); different contrasts at different time points not clearly explained	↑ L somato-motor<br>notes: label based on figure
Schofield et al. (2012): Vox 1	Listening to word pairs or reversed word pairs, speaker gender judgment vs rest	CAC<br>Moderate aphasia (n = 11) vs control	UNR	UNR	Vox<br>NC	Search volume: whole brain; software: SPM8; voxelwise p: .001; cluster extent cutoff: none	↓ L Heschl's gyrus<br>notes: structurally, HG was not significantly damaged in this group
Schofield et al. (2012): Vox 2	Listening to word pairs or reversed word pairs, speaker gender judgment vs rest	CAC<br>Severe aphasia (n = 9) vs control	UNR	UNR	Vox<br>M**	Search volume: whole brain; software: SPM8; voxelwise p: MGB: SVC; elsewhere: .001; cluster extent cutoff: none	↓ L posterior STG<br>↓ L Heschl's gyrus<br>↓ L thalamus<br>notes: specifically: PT, HG and MGB; structurally, the PT and HG were significantly damaged, but not the MGB
Schofield et al. (2012): Vox 3	Listening to word pairs or reversed word pairs, speaker gender judgment vs rest	CAA<br>Severe (n = 9) vs moderate (n = 11) aphasia	UNR	UNR	Vox<br>NC	Search volume: whole brain; software: SPM8; voxelwise p: .001; cluster extent cutoff: none	↓ L posterior STG<br>notes: specifically, PT; structurally, severe patients had more damage in HG and PT
Wright et al. (2012): Vox 1	Listening to normal sentences and detecting a target word vs rest	CAC<br>Aphasia vs control	Y	UNR	Vox<br>NC	Search volume: whole brain; software: SPM5; voxelwise p: .01	↓ L posterior STG/STS/MTG<br>↓ L Heschl's gyrus<br>↓ L mid temporal<br>notes: at a more stringent threshold of p < .001, with correction for multiple comparisons based on GRFT and cluster extent, only L HG showed reduced activity in patients
Wright et al. (2012): Cplx 1	Listening to normal sentences and detecting a target word vs rest	CC<br>Aphasia<br>Covariate: see statistical details	UNR	UNR	Cplx	Joint ICA was performed on structural and functional contrast images for each of the two contrasts using FIT 2.0b. Seven components were derived, of which 2 were further investigated since their loadings correlated with relevant behavioral measures. Functional components were	Other:<br>Contrast 1 loaded primarily on the R STG for component 1 (the "semantics component") and on the L ITG for

						thresholded at p < .001, cluster-corrected for multiple comparisons, minimum cluster extent = 1.27 cc. Component 1 was considered a "semantics component" because it correlated with the semantic behavioral measure and not with either of the two syntactic measures. This component did not have any anatomical aspect to it. Component 2 was considered a "syntax component" because it correlated with both syntactic behavioral measures and not with the semantic measure. This conceptualization seems somewhat speculative, given that WPE NP and WPE AP are rather indirect measures of syntactic and semantic processing. Component 2 involved damage to left frontal and insular cortex, and underlying dorsal white matter.	component 2 (the "syntax component").
Wright et al. (2012): Cplx 2	Listening to grammatical but meaningless sentences and detecting a target word vs rest	CC<br>Aphasia<br>Covariate: see statistical details	UNR	UNR	Cplx	Joint ICA was performed on structural and functional contrast images for each of the two contrasts using FIT 2.0b. Seven components were derived, of which 2 were further investigated since their loadings correlated with relevant behavioral measures. Functional components were thresholded at p < .001, cluster-corrected for multiple comparisons, minimum cluster extent = 1.27 cc. Component 1 was considered a "semantics component" because it correlated with the semantic behavioral measure and not with either of the two syntactic measures. This component did not have any anatomical aspect to it. Component 2 was considered a "syntax component" because it correlated with both syntactic behavioral measures and not with the semantic measure. This conceptualization seems somewhat speculative, given that WPE NP and WPE AP are rather indirect measures of syntactic and semantic processing. Component 2 involved damage to left frontal and insular cortex, and underlying dorsal white matter.	Other:<br>Contrast 2 loaded primarily on the R posterior STG for component 1 (the "semantics component") and on the L posterior STG and L IFG for component 2 (the "syntax component").
Szaflarski et al. (2013): Vox 1	Semantic decision vs tone decision	CAA<br>Aphasia not recovered (n = 18) vs recovered (n = 9)	AM	UNR	Vox<br>CCS	Behavioral data notes: interaction of group by condition not reported; non-recovered patients were significantly less accurate only on the semantic decision condition, but they actually showed a smaller difference between conditions than the recovered patients; search volume: whole brain; software: AFNI; voxelwise p: .05; cluster extent cutoff: 4.16 cc; cluster-defining threshold (CDT) p < 0.05 too lenient	↑ L dorsolateral prefrontal cortex<br>↑ L superior parietal<br>↑ L cerebellum<br>↑ R cerebellum<br>↓ R posterior STG

Szaflarski et al. (2013): ROI 1	Semantic decision vs tone decision	CC<br>Aphasia (recovered and non-recovered)<br>Covariate: BNT	UNR	UNR	ROI Func FWE	Number of ROIs: 4; ROIs: (1) bilateral cerebellum; (2) R pSTG; (3) L superior parietal lobule; (4) L superior frontal gyrus; how ROIs defined: regions that were differentially recruited between recovered and non-recovered patients; average t scores from individual SPMs; circular because defined based on recovered status	↑ L dorsolateral prefrontal cortex
Szaflarski et al. (2013): ROI 2	Semantic decision vs tone decision	CC<br>Aphasia (recovered and non-recovered)<br>Covariate: semantic fluency	UNR	UNR	ROI Func FWE	Number of ROIs: 4; ROIs: (1) bilateral cerebellum; (2) R pSTG; (3) L superior parietal lobule; (4) L superior frontal gyrus; how ROIs defined: regions that were differentially recruited between recovered and non-recovered patients; average t scores from individual SPMs; circular because defined based on recovered status	↑ L dorsolateral prefrontal cortex
Szaflarski et al. (2013): ROI 3	Semantic decision vs tone decision	CC<br>Aphasia (recovered and non-recovered)<br>Covariate: single word comprehension (PPVT)	UNR	UNR	ROI Func FWE	Number of ROIs: 4; ROIs: (1) bilateral cerebellum; (2) R pSTG; (3) L superior parietal lobule; (4) L superior frontal gyrus; how ROIs defined: regions that were differentially recruited between recovered and non-recovered patients; average t scores from individual SPMs; circular because defined based on recovered status	↑ L dorsolateral prefrontal cortex
Szaflarski et al. (2013): ROI 4	Semantic decision vs tone decision	CC<br>Aphasia (recovered and non-recovered)<br>Covariate: BDAE complex ideation subtest	UNR	UNR	ROI Func FWE	Number of ROIs: 4; ROIs: (1) bilateral cerebellum; (2) R pSTG; (3) L superior parietal lobule; (4) L superior frontal gyrus; how ROIs defined: regions that were differentially recruited between recovered and non-recovered patients; average t scores from individual SPMs; circular because defined based on recovered status	↑ L dorsolateral prefrontal cortex
Szaflarski et al. (2013): ROI 5	Semantic decision vs tone decision	CC<br>Aphasia (recovered and non-recovered)<br>Covariate: phonemic fluency	UNR	UNR	ROI Func FWE	Number of ROIs: 4; ROIs: (1) bilateral cerebellum; (2) R pSTG; (3) L superior parietal lobule; (4) L superior frontal gyrus; how ROIs defined: regions that were differentially recruited between recovered and non-recovered patients; average t scores from individual SPMs; circular because defined based on recovered status	↓ R posterior STG
Szaflarski et al. (2013): ROI 6	Semantic decision vs tone decision	CC<br>Aphasia (recovered and non-recovered)<br>Covariate: semantic decision accuracy	C	UNR	ROI Func FWE	Number of ROIs: 4; ROIs: (1) bilateral cerebellum; (2) R pSTG; (3) L superior parietal lobule; (4) L superior frontal gyrus; how ROIs defined: regions that were differentially recruited between recovered and non-recovered patients; average t scores from individual SPMs; circular because defined based on recovered status	None
Thiel et al. (2013): Vox 1	Verb generation vs rest	LAA<br>(Aphasia with rTMS (n = 13) T2 vs T1) vs (aphasia with sham (n = 11) T2 vs T1)	UNR	UNR	Vox NDC	Search volume: whole brain; software: SPM8; qualitative comparison on p. 2244	↑ L IFG<br>↑ L posterior STG/STS/MTG<br>↓ R IFG<br>↓ R posterior STG/STS/MTG<br>notes:

							approximate interpretation of qualitative patterns shown in Figure 3; T1 R lateralization surprising relative to other findings from this group
Thiel et al. (2013): ROI 1	Verb generation vs rest	LAA (Aphasia with rTMS (n = 13) T2 vs T1) vs (aphasia with sham (n = 11) T2 vs T1)	UNR	UNR	ROI LI One	Number of ROIs: 1; ROI: language network LI; actual LIs are not reported, only change in LI	↑ LI (language network) notes: T1 R lateralization surprising relative to other findings from this group
Thiel et al. (2013): ROI 2	Verb generation vs rest	LC Aphasia T2 vs T1 Covariate: Δ AAT total score	UNR	UNR	ROI LI One	Number of ROIs: 1; ROI: language network LI; model did not include treatment group (rTMS vs sham)	↑ LI (language network) notes: patients who improved more showed a greater leftward shift of activation; T1 R lateralization surprising relative to other findings from this group
Abel et al. (2014): Vox 1	Picture naming (all conditions) vs rest	CC Aphasia T1 Covariate: subsequent Δ (T2 vs T1) picture naming Somewhat valid (T1 behavioral measure should be included in model)	C	UNR	Vox CCTB	Search volume: whole brain; software: SPM8; voxelwise p: .01; cluster extent cutoff: 11 voxels (size not stated)	↑ L IFG pars opercularis<br>↓ R basal ganglia
Abel et al. (2014): Vox 2	Picture naming (all conditions) vs rest	LC Aphasia T2 vs T1 Covariate: Δ picture naming accuracy	C	UNR	Vox CCTB	Search volume: whole brain; software: SPM8; voxelwise p: .01; cluster extent cutoff: 11 voxels (size not stated)	↑ L somato-motor<br>↑ L inferior parietal lobule<br>↑ L supramarginal gyrus<br>↑ L posterior STS<br>↑ L posterior MTG<br>↑ L occipital
Abel et al. (2014): Vox 3	Picture naming (trained items) vs picture naming (untrained items)	LA Aphasia T2 vs T1	N	UNR	Vox CCTB	Behavioral data notes: trained items improved more than untrained items; search volume: whole brain; software: SPM8; voxelwise p: .01; cluster extent cutoff: 11 voxels (size not stated)	↑ L precuneus<br>↑ L posterior STG<br>↑ L Heschl's gyrus<br>↑ L mid temporal<br>↑ L posterior cingulate<br>↑ L thalamus<br>↑ R ventral precentral/inferior frontal junction<br>↑ R somato-motor<br>↑ R Heschl's gyrus<br>↑ R posterior cingulate<br>↑ R thalamus<br>↑ R basal ganglia
Abel et al.	Picture naming	LA	Y	UNR	Vox	Behavioral data notes: no differential	↑ R superior

(2014): Vox 4	(semantic trained items) vs picture naming (phonological trained items)	Aphasia T2 vs T1			CCTB	effects for semantic vs phonological trained items; search volume: whole brain; software: SPM8; voxelwise p: .01; cluster extent cutoff: 11 voxels (size not stated)	parietal<br>↓ L dorsolateral prefrontal cortex<br>↓ L somato-motor<br>↓ L occipital<br>↓ L anterior cingulate<br>↓ L posterior cingulate<br>↓ R precuneus<br>↓ R occipital<br>↓ R anterior cingulate<br>↓ R posterior cingulate<br>↓ R hippocampus/MTL
Abel et al. (2014): Vox 5	Picture naming (all conditions) vs rest	CAA<br>Aphasia with semantic impairment T1 (n = 8) vs with phonological impairment T1 (n = 6)	UNR	UNR	Vox CCTB	Search volume: whole brain; software: SPM8; voxelwise p: .01; cluster extent cutoff: 11 voxels (size not stated)	↑ R IFG pars triangularis<br>↑ R dorsolateral prefrontal cortex
Abel et al. (2014): Vox 6	Picture naming (all conditions) vs rest	LAA<br>(Aphasia with semantic impairment (n = 8) T2 vs T1) vs (aphasia with phonological impairment (n = 6) T2 vs T1)	N	UNR	Vox CCTB	Behavioral data notes: phonological patients showed more improvement on trained items; search volume: whole brain; software: SPM8; voxelwise p: .01; cluster extent cutoff: 11 voxels (size not stated)	↑ L somato-motor<br>↑ L Heschl's gyrus<br>↑ L anterior temporal<br>↑ L occipital<br>↑ L thalamus<br>↑ L basal ganglia<br>↑ R somato-motor<br>↓ L IFG pars opercularis
Abel et al. (2014): Vox 7	Picture naming (all conditions) vs rest	LA<br>Aphasia with semantic impairment (n = 8) T2 vs T1	N	UNR	Vox CCTB	Search volume: whole brain; software: SPM8; voxelwise p: .01; cluster extent cutoff: 11 voxels (size not stated)	↑ L basal ganglia
Abel et al. (2014): Vox 8	Picture naming (all conditions) vs rest	LA<br>Aphasia with phonological impairment (n = 6) T2 vs T1	N	UNR	Vox CCTB	Search volume: whole brain; software: SPM8; voxelwise p: .01; cluster extent cutoff: 11 voxels (size not stated)	None
Benjamin et al. (2014): ROI 1	Word generation vs rest	LA<br>Aphasia with intention treatment (n = 7) T2 vs T1	UNR	UNR	ROI LI NC	Number of ROIs: 3; ROIs: (1) lateral frontal LI; (2) medial frontal LI; (3) posterior perisylvian LI	↓ LI (frontal) notes: laterality shift for lateral frontal LI, not medial frontal LI
Benjamin et al. (2014): ROI 2	Word generation vs rest	LA<br>Aphasia with intention treatment (n = 6) T3 vs T1	UNR	UNR	ROI LI NC	Number of ROIs: 3; ROIs: (1) lateral frontal LI; (2) medial frontal LI; (3) posterior perisylvian LI	↓ LI (frontal) notes: laterality shift for both lateral and medial frontal LIs
Benjamin et al. (2014): ROI 3	Word generation vs rest	LA<br>Aphasia with control treatment (n = 7) T2 vs T1	UNR	UNR	ROI LI NC	Number of ROIs: 3; ROIs: (1) lateral frontal LI; (2) medial frontal LI; (3) posterior perisylvian LI	None
Benjamin et al. (2014): ROI 4	Word generation vs rest	LA<br>Aphasia with control treatment (n = 7) T3 vs T1	UNR	UNR	ROI LI NC	Number of ROIs: 3; ROIs: (1) lateral frontal LI; (2) medial frontal LI; (3) posterior perisylvian LI	None
Benjamin	Word generation	LC	UNR	UNR	ROI	Number of ROIs: 3; ROIs: (1) lateral	↓ LI (temporal)

et al. (2014): ROI 5	vs rest	Aphasia with intention treatment (n = 7) T2 vs T1<br>Covariate: Δ category-member generation probe performance			LI NC	frontal LI; (2) medial frontal LI; (3) posterior perisylvian LI	
Benjamin et al. (2014): ROI 6	Word generation vs rest	LC<br>Aphasia with control treatment (n = 7) T2 vs T1<br>Covariate: Δ category-member generation probe performance	UNR	UNR	ROI LI NC	Number of ROIs: 3; ROIs: (1) lateral frontal LI; (2) medial frontal LI; (3) posterior perisylvian LI	None
Benjamin et al. (2014): ROI 7	Word generation vs rest	LC<br>Aphasia with intention treatment (n = 7) T2 vs T1<br>Covariate: Δ picture naming probe performance	UNR	UNR	ROI LI NC	Number of ROIs: 3; ROIs: (1) lateral frontal LI; (2) medial frontal LI; (3) posterior perisylvian LI	None
Benjamin et al. (2014): ROI 8	Word generation vs rest	LC<br>Aphasia with control treatment (n = 7) T2 vs T1<br>Covariate: Δ picture naming probe performance	UNR	UNR	ROI LI NC	Number of ROIs: 3; ROIs: (1) lateral frontal LI; (2) medial frontal LI; (3) posterior perisylvian LI	None
Brownsett et al. (2014): Vox 1	Listening to sentences vs listening to segmented white noise	CAC<br>Aphasia (T2 and T3) vs control (T1 and T2)	N	NANT	Vox C-	Behavioral data notes: significant difference in accuracy of subsequent repetition; search volume: whole brain; software: FSL (FEAT 5.98); voxelwise p: ~.01 (z > 2.3); cluster extent cutoff: based on GRFT	↑ L insula<br>↑ L anterior cingulate<br>↑ R insula<br>↑ R anterior cingulate<br>↓ L SMA/medial prefrontal<br>↓ L precuneus<br>↓ L posterior cingulate<br>↓ R SMA/medial prefrontal<br>↓ R precuneus<br>↓ R posterior cingulate<br>notes: findings are approximate since description is partially in terms of networks; at the earlier time point only, patients also showed reduced activity in left ventral prefrontal cortex and right medial planum temporale
Brownsett et al. (2014): Vox 2	Listening to sentences (patients) or listening to noise vocoded sentences (controls) vs	CAC<br>Aphasia (T2 and T3) vs control (T1 and T2)	Y	NANT	Vox C-	Behavioral data notes: no significant difference in accuracy of subsequent repetition; search volume: whole brain; software: FSL (FEAT 5.98); voxelwise p: ~.01 (z > 2.3); cluster extent cutoff: based on GRFT	None

	listening to segmented white noise						
Brownsett et al. (2014): ROI 1	Listening to sentences vs listening to segmented white noise	CC<br>Aphasia mean of T1, T2, T3<br>Covariate: picture description score (CAT), mean of T1, T2, T3	UNR	NANT	ROI Func One	Behavioral data notes: referring to accuracy of subsequent repetition; correlation with picture description is not reported; number of ROIs: 1; ROI: dorsal anterior cingulate cortex/midline superior frontal gyrus; how ROI defined: contrast of listening to vocoded speech and listening to normal speech in controls; same result obtained with age and lesion volume included in the model	↑ L SMA/medial prefrontal<br>↑ L anterior cingulate<br>↑ R SMA/medial prefrontal<br>↑ R anterior cingulate<br>notes: increased activation of dACC/SFG was correlated with higher scores on picture description
Mattioli et al. (2014): Vox 1	Listening to sentences and making a plausibility judgment vs listening to reversed speech	CAA<br>Aphasia treated T2 (n = 6) vs untreated T2 (n = 6)<br>Somewhat valid (groups were different but not due to treatment)	Y	UNR	Vox CA	Search volume: whole brain; software: BrainVoyager QX 1.9; voxelwise p: .001; cluster extent cutoff: 0.16 cc; methods report cluster extent threshold (we assume this was done), but figure caption states uncorrected	↑ L IFG pars opercularis<br>↑ L IFG pars triangularis<br>↑ L SMA/medial prefrontal<br>↑ L angular gyrus<br>↑ R ventral precentral/inferior frontal junction<br>↑ R supramarginal gyrus<br>notes: based on coordinates in Table 2
Mattioli et al. (2014): Vox 2	Listening to sentences and making a plausibility judgment vs listening to reversed speech	CAA<br>Aphasia treated T3 (n = 6) vs untreated T3 (n = 6)<br>Somewhat valid (groups were different but not due to treatment)	Y	UNR	Vox CA	Search volume: whole brain; software: BrainVoyager QX 1.9; voxelwise p: .001; cluster extent cutoff: 0.16 cc; methods report cluster extent threshold (we assume this was done), but figure caption states uncorrected	↑ L IFG pars triangularis<br>↑ L insula<br>↑ L supramarginal gyrus<br>notes: based on coordinates in Table 2; also increases in R IFG and R supramarginal gyrus but only uncorrected
Mattioli et al. (2014): Vox 3	Listening to sentences and making a plausibility judgment vs listening to reversed speech	LAA<br>(Aphasia treated (n = 6) T2 vs T1) vs (untreated (n = 6) T2 vs T1)<br>Somewhat valid (no treatment effect)	Y	UNR	Vox NDC	Search volume: whole brain; software: BrainVoyager QX 1.9; qualitative comparison on p. 548	↑ L IFG<br>↑ R posterior STG<br>↓ L inferior parietal lobule<br>↓ R IFG<br>notes: treated patients showed increases in L IFG and R STG, while untreated patients showed increases in L IPL and R IFG
Mattioli et al. (2014): Vox 4	Listening to sentences and making a plausibility judgment vs	LAA<br>(Aphasia treated (n = 6) T3 vs T2) vs (untreated (n = 6) T3 vs T2)	Y	UNR	Vox NDC	Search volume: whole brain; software: BrainVoyager QX 1.9; qualitative comparison on p. 548	None<br>notes: the two groups were reported to have comparable

	listening to reversed speech	Somewhat valid (no treatment effect)					increases in L hemisphere language areas
Mattioli et al. (2014): Vox 5	Listening to sentences and making a plausibility judgment vs listening to reversed speech	LA<br>Aphasia treated (n = 6) T2 vs T1	Y	UNR	Vox<br>NC	Search volume: whole brain; software: BrainVoyager QX 1.9; voxelwise p: .005; cluster extent cutoff: none	↑ L IFG pars opercularis<br>↑ R posterior STG
Mattioli et al. (2014): Vox 6	Listening to sentences and making a plausibility judgment vs listening to reversed speech	LA<br>Aphasia untreated (n = 6) T2 vs T1	Y	UNR	Vox<br>NC	Search volume: whole brain; software: BrainVoyager QX 1.9; voxelwise p: .005; cluster extent cutoff: none	↑ L inferior parietal lobule<br>↑ R insula
Mattioli et al. (2014): Vox 7	Listening to sentences and making a plausibility judgment vs listening to reversed speech	LA<br>Aphasia treated (n = 6) T3 vs T2	Y	UNR	Vox<br>NC	Search volume: whole brain; software: BrainVoyager QX 1.9; voxelwise p: .005; cluster extent cutoff: none	↑ L IFG<br>↑ L insula<br>↑ L inferior parietal lobule<br>↑ L anterior temporal<br>↑ R insula
Mattioli et al. (2014): Vox 8	Listening to sentences and making a plausibility judgment vs listening to reversed speech	LA<br>Aphasia untreated (n = 6) T3 vs T2	Y	UNR	Vox<br>NC	Search volume: whole brain; software: BrainVoyager QX 1.9; voxelwise p: .005; cluster extent cutoff: none	↑ L IFG pars opercularis<br>↑ L IFG pars triangularis<br>↑ L IFG pars orbitalis<br>↑ L angular gyrus<br>↑ L superior parietal<br>↑ L posterior STG/STS/MTG<br>↑ R IFG pars opercularis<br>↑ R angular gyrus
Mattioli et al. (2014): ROI 1	Listening to sentences and making a plausibility judgment vs listening to reversed speech	LAA<br>(Aphasia treated (n = 6) T1 ≠ T2 ≠ T3) vs (untreated (n = 6) T1 ≠ T2 ≠ T3)<br>Somewhat valid (no treatment effect)	Y	UNR	ROI<br>Func<br>NC	Number of ROIs: 4; ROIs: (1) L IFG; (2) R IFG; (3) L STG; (4) R STG; how ROIs defined: based on functional data from patients and controls, but details not stated; a different set of ROIs are mentioned in the results so it is not really clear which set were actually used	↑ L IFG<br>notes: interaction of time by treatment: treated group showed greater L IFG activity at T2
Mattioli et al. (2014): ROI 2	Listening to sentences and making a plausibility judgment vs listening to reversed speech	LC<br>Aphasia treated (n = 6) T2 vs T1<br>Covariate: Δ written language (AAT)	Y	UNR	ROI<br>Func<br>NC	Number of ROIs: 4; ROIs: (1) L IFG; (2) R IFG; (3) L STG; (4) R STG; how ROIs defined: based on functional data from patients and controls, but details not stated; a different set of ROIs are mentioned in the results so it is not really clear which set were actually used	None
Mattioli et al. (2014): ROI 3	Listening to sentences and making a plausibility judgment vs listening to reversed speech	LC<br>Aphasia treated (n = 6) T2 vs T1<br>Covariate: Δ naming (AAT)	Y	UNR	ROI<br>Func<br>NC	Number of ROIs: 4; ROIs: (1) L IFG; (2) R IFG; (3) L STG; (4) R STG; how ROIs defined: based on functional data from patients and controls, but details not stated; a different set of ROIs are mentioned in the results so it is not	↑ L IFG

						really clear which set were actually used	
Mattioli et al. (2014): ROI 4	Listening to sentences and making a plausibility judgment vs listening to reversed speech	LC<br>Aphasia untreated (n = 6) T2 vs T1<br>Covariate: Δ written language (AAT)	Y	UNR	ROI Func NC	Number of ROIs: 4; ROIs: (1) L IFG; (2) R IFG; (3) L STG; (4) R STG; how ROIs defined: based on functional data from patients and controls, but details not stated; a different set of ROIs are mentioned in the results so it is not really clear which set were actually used	None
Mattioli et al. (2014): ROI 5	Listening to sentences and making a plausibility judgment vs listening to reversed speech	LC<br>Aphasia untreated (n = 6) T2 vs T1<br>Covariate: Δ naming (AAT)	Y	UNR	ROI Func NC	Number of ROIs: 4; ROIs: (1) L IFG; (2) R IFG; (3) L STG; (4) R STG; how ROIs defined: based on functional data from patients and controls, but details not stated; a different set of ROIs are mentioned in the results so it is not really clear which set were actually used	↑ R IFG
Mohr et al. (2014): Vox 1	Listening to sentences (high and low ambiguity) vs listening to signal-correlated noise	LA<br>Aphasia T2 vs T1	NANB	NANT	Vox NDC	Search volume: whole brain; software: SPM8; qualitative generalization across individuals on pp. 8-9	None
Mohr et al. (2014): ROI 1	Listening to high ambiguity sentences vs listening to low ambiguity sentences	LA<br>Aphasia T2 vs T1	NANB	NANT	ROI Func NC	Number of ROIs: 4; ROIs: (1) L IFG; (2) R IFG; (3) L ITG; (4) R ITG; the temporal ROIs are described as STG but they seem to be in the ITG; how ROIs defined: defined based on control data from Rodd et al. (2005) but the coordinates do not match so it is not clear exactly how they were defined; ANOVA of timepoint by hemisphere by site, with a significant interaction of timepoint by hemisphere	↑ R IFG<br>↑ R posterior inferior temporal gyrus/fusiform gyrus<br>notes: all signal changes were negative (i.e. less activation for ambiguous sentences), making interpretation challenging
Robson et al. (2014): Vox 1	Semantic decision (written word and picture) vs visual decision and rest	CAC<br>Aphasia vs control	N	N	Vox CA	Behavioral data notes: patients also less accurate on control condition, but control condition includes rest so coded based on language condition only; search volume: whole brain; software: SPM8; voxelwise p: .005; cluster extent cutoff: 4 voxels (size not stated); dual baseline computation not explained	↑ L IFG pars orbitalis<br>↑ L mid temporal<br>↑ L anterior temporal<br>↑ L cerebellum<br>↑ L hippocampus/MTL<br>↑ R mid temporal<br>↑ R anterior temporal<br>↑ R posterior inferior temporal gyrus/fusiform gyrus<br>↑ R cerebellum<br>↑ R hippocampus/MTL<br>↓ R posterior cingulate
Robson et al. (2014): ROI 1	Semantic decision (written word and	CAC<br>Aphasia vs control	N	N	ROI Func NC	Behavioral data notes: patients also less accurate on control condition, but control condition includes rest so	↑ L anterior temporal<br>↑ L posterior

	picture) vs visual decision and rest					coded based on language condition only; number of ROIs: 10; ROIs: (1) L anterior fusiform gyrus; (2) L temporal pole; (3) L anterior STS; (4) L IFG; (5) L ventral occipito-temporal; (6-10) homotopic counterparts; how ROIs defined: spheres around functional peaks from literature; dual baseline computation not explained	inferior temporal gyrus/fusiform gyrus<br>↑ R posterior inferior temporal gyrus/fusiform gyrus
Szaflarski et al. (2014): Vox 1	Verb generation vs finger tapping	CAC<br>Aphasia vs control	UNR	UNR	Vox<br>NDC	Search volume: whole brain; software: CCHIPS; qualitative comparison on pp. 5-6 (page numbers refer to PMC author manuscript)	↓ L inferior parietal lobule<br>↓ L superior parietal<br>↓ L posterior STG/STS/MTG<br>↓ L occipital<br>↓ R occipital
Szaflarski et al. (2014): ROI 1	Verb generation vs finger tapping	CAC<br>Aphasia vs control	UNR	UNR	ROI<br>LI<br>NC	Number of ROIs: 3; ROIs: (1) frontal LI; (2) temporal LI; (3) language network LI	↓ LI (language network)<br>↓ LI (frontal)<br>notes: temporal LI was also marginally significantly reduced (p = .08)
van Hees et al. (2014): Vox 1	Picture naming (phonological trained items, correct trials) vs viewing scrambled images	CC<br>Aphasia T1<br>Covariate: subsequent Δ (T2 vs T1) picture naming (phonological treated items)<br>Somewhat valid (T1 behavioral measure should be included in model)	YCT	UNR	Vox<br>CCS	Search volume: whole brain; software: AFNI; voxelwise p: .005; cluster extent cutoff: 0.999 cc	None
van Hees et al. (2014): Vox 2	Picture naming (semantic trained items, correct trials) vs viewing scrambled images	CC<br>Aphasia T1<br>Covariate: subsequent Δ (T2 vs T1) picture naming (semantic treated items)<br>Somewhat valid (T1 behavioral measure should be included in model)	YCT	UNR	Vox<br>CCS	Search volume: whole brain; software: AFNI; voxelwise p: .005; cluster extent cutoff: 0.999 cc	↑ L basal ganglia
van Hees et al. (2014): Vox 3	Picture naming (phonological trained items, correct trials) vs viewing scrambled images	CC<br>Aphasia T2<br>Covariate: previous Δ (T2 vs T1) picture naming (phonological treated items)<br>Not valid (T2 activation not an appropriate measure of treatment-induced recovery because it reflects T2 performance)	YCT	UNR	Vox<br>CCS	Search volume: whole brain; software: AFNI; voxelwise p: .005; cluster extent cutoff: 0.999 cc	↑ L supramarginal gyrus<br>↑ R precuneus
van Hees et	Picture naming	CC	YCT	UNR	Vox	Search volume: whole brain; software:	None

al. (2014): Vox 4	(semantic trained items, correct trials) vs viewing scrambled images	Aphasia T2<br>Covariate: previous Δ (T2 vs T1) picture naming (semantic treated items)<br>Not valid (T2 activation not an appropriate measure of treatment-induced recovery because it reflects T2 performance)			CCS	AFNI; voxelwise p: .005; cluster extent cutoff: 0.999 cc	
van Hees et al. (2014): Vox 5	Picture naming (phonological trained items, correct trials) vs viewing scrambled images	CC<br>Aphasia T1<br>Covariate: subsequent outcome (T2) picture naming<br>Not valid (not appropriate to correlate T1 imaging with T2 behavior without T1 behavior in model)	YCT	UNR	Vox<br>CCS	Search volume: whole brain; software: AFNI; voxelwise p: .005; cluster extent cutoff: 0.999 cc	None
van Hees et al. (2014): Vox 6	Picture naming (semantic trained items, correct trials) vs viewing scrambled images	CC<br>Aphasia T1<br>Covariate: subsequent outcome (T2) picture naming<br>Not valid (not appropriate to correlate T1 imaging with T2 behavior without T1 behavior in model)	YCT	UNR	Vox<br>CCS	Search volume: whole brain; software: AFNI; voxelwise p: .005; cluster extent cutoff: 0.999 cc	None
van Hees et al. (2014): Vox 7	Picture naming (phonological trained items, correct trials) vs viewing scrambled images	CC<br>Aphasia T2<br>Covariate: picture naming T2	YCT	UNR	Vox<br>CCS	Search volume: whole brain; software: AFNI; voxelwise p: .005; cluster extent cutoff: 0.999 cc	None
van Hees et al. (2014): Vox 8	Picture naming (semantic trained items, correct trials) vs viewing scrambled images	CC<br>Aphasia T2<br>Covariate: picture naming T2	YCT	UNR	Vox<br>CCS	Search volume: whole brain; software: AFNI; voxelwise p: .005; cluster extent cutoff: 0.999 cc	None
Abel et al. (2015): Vox 1	Picture naming vs rest	LA<br>Aphasia T2 vs T1	N	N	Vox<br>CCTB	Behavioral data notes: RT shorter at T2; search volume: whole brain; software: SPM8; voxelwise p: .01; cluster extent cutoff: 11 voxels (size not stated)	↓ L IFG pars triangularis<br>↓ L dorsolateral prefrontal cortex<br>↓ L ventral precentral/inferior frontal junction<br>↓ L dorsal precentral<br>↓ L SMA/medial prefrontal<br>↓ L somato-motor<br>↓ L inferior parietal lobule<br>↓ L precuneus<br>↓ L posterior cingulate

							↓ L cerebellum<br>↓ R SMA/medial prefrontal<br>↓ R somato-motor<br>↓ R precuneus<br>↓ R posterior STS<br>↓ R posterior MTG<br>↓ R posterior cingulate<br>↓ R cerebellum<br>↓ R thalamus<br>↓ R hippocampus/MTL
Abel et al. (2015): Vox 2	Picture naming vs rest	CAC<br>Aphasia T1 vs control T1	AM	N	Vox<br>CCTB	Behavioral data notes: controls responded more quickly; search volume: whole brain; software: SPM8; voxelwise p: .01; cluster extent cutoff: 11 voxels (size not stated)	↑ R precuneus<br>↓ L somato-motor<br>↓ L Heschl's gyrus<br>↓ L anterior cingulate<br>↓ L posterior cingulate<br>↓ L thalamus<br>↓ L basal ganglia<br>↓ R insula<br>↓ R somato-motor<br>↓ R mid temporal
Abel et al. (2015): Vox 3	Picture naming vs rest	LAC<br>(Aphasia T2 vs T1) vs (control T2 vs T1)	AM	UNR	Vox<br>CCTB	Behavioral data notes: RT not reported for controls; search volume: whole brain; software: SPM8; voxelwise p: .01; cluster extent cutoff: 11 voxels (size not stated)	↓ L precuneus<br>↓ L anterior cingulate<br>↓ L posterior cingulate<br>↓ L basal ganglia<br>↓ R precuneus<br>↓ R posterior STS<br>↓ R posterior MTG<br>↓ R posterior cingulate<br>↓ R thalamus<br>↓ R hippocampus/MTL
Abel et al. (2015): Vox 4	Picture naming vs rest	CAC<br>Aphasia T1 vs control T1	AM	UNR	Vox<br>NDC	Behavioral data notes: RT not reported for controls; search volume: whole brain; software: SPM8; qualitative comparison between activation in the first 5 TRs after each stimulus on p. 1101	None<br>notes: the time course of response is stated to be similar in patients and controls, however the response in patients appears like it could be a couple of seconds slower
Abel et al. (2015): Cplx 1	Picture naming vs rest	CAC<br>Aphasia vs control	N	UNR	Cplx	Behavioral data notes: RT not reported for controls; Joint ICA was performed on structural and functional contrast images using FIT 1.2c. Three of the 7 components differed between groups in their loadings. Components were thresholded at z > 3.09, not corrected for multiple comparisons.	Other:<br>Three structural-functional components are described in Figure 5 and Table 4. Functional activations are generally small and do not obviously relate to

							language processing. It is mentioned in the supplementary results that "the lesion maps may dominate estimation of the mixing parameter" (p. 10).
Kiran et al. (2015): Vox 1	Picture naming (trained) vs viewing scrambled images and saying "skip"	LA<br>Aphasia T2 vs T1	UNR	UNR	Vox NDC	Search volume: whole brain; software: SPM8; analyses were carried out in individual patients at p < .001, uncorrected; regions were considered activated when they were found in 6 or more (out of 8) patients	↑ L IFG<br>↑ L dorsolateral prefrontal cortex<br>↑ L ventral precentral/inferior frontal junction<br>↑ L dorsal precentral<br>↑ L SMA/medial prefrontal<br>↑ L supramarginal gyrus<br>↑ L angular gyrus<br>↑ L posterior MTG<br>↑ R IFG<br>↑ R dorsolateral prefrontal cortex<br>↑ R SMA/medial prefrontal<br>↑ R supramarginal gyrus<br>↑ R posterior STG<br>↑ R posterior MTG<br>↑ R posterior inferior temporal gyrus/fusiform gyrus<br>notes: regions are approximate since only broad regions are described in Table 6
Kiran et al. (2015): Vox 2	Semantic feature decision vs visual decision	LA<br>Aphasia T2 vs T1	UNR	UNR	Vox NDC	Search volume: whole brain; software: SPM8; analyses were carried out in individual patients at p < .001, uncorrected; regions were considered activated when they were found in 6 or more (out of 8) patients	↑ L ventral precentral/inferior frontal junction<br>↑ L dorsal precentral<br>↑ L posterior MTG<br>↑ R IFG<br>↑ R dorsolateral prefrontal cortex<br>↑ R SMA/medial prefrontal<br>↑ R angular gyrus<br>↑ R posterior STG<br>↑ R posterior MTG<br>notes: regions are approximate since only broad regions are described in Table 7
Sandberg	Concreteness	LA	Y	Y	Vox	Search volume: whole brain; software:	↑ L IFG pars

et al. (2015): Vox 1	judgment (abstract words, correct trials) vs rest	Aphasia with response to treatment (n = 9) T2 vs T1			NC	SPM8; voxelwise p: .001; cluster extent cutoff: none; images show peaks instead of activations	opercularis<br>↑ L dorsolateral prefrontal cortex<br>↑ L SMA/medial prefrontal<br>↑ L inferior parietal lobule<br>↑ L supramarginal gyrus<br>↑ L angular gyrus<br>↑ L precuneus<br>↑ L posterior inferior temporal gyrus/fusiform gyrus<br>↑ L posterior cingulate<br>↑ L basal ganglia<br>↑ R orbitofrontal<br>↑ R supramarginal gyrus<br>↑ R angular gyrus<br>↑ R anterior temporal<br>↑ R occipital
Sandberg et al. (2015): Vox 2	Concreteness judgment (concrete words, correct trials) vs rest	LA<br>Aphasia with generalization of treatment effects to concrete words (n = 7) T2 vs T1	Y	Y	Vox<br>NC	Search volume: whole brain; software: SPM8; voxelwise p: .001; cluster extent cutoff: none; images show peaks instead of activations	↑ L insula<br>↑ L inferior parietal lobule<br>↑ L supramarginal gyrus<br>↑ L precuneus<br>↑ L occipital<br>↑ R dorsolateral prefrontal cortex<br>↑ R ventral precentral/inferior frontal junction<br>↑ R posterior STG<br>↑ R posterior cingulate
Geranmayeh et al. (2016): ROI 1	Propositional speech production vs rest	CAC<br>Aphasia vs control	N	UNR	ROI<br>Func<br>NC	Behavioral data notes: difference in AICW/trial; number of ROIs: 4; ROIs: (1) L fronto-temporo-parietal network; (2) R fronto-temporo-parietal network; (3) cingulo-opercular network; (4) default mode network; how ROIs defined: identified using ICA in controls; circular because ROIs defined in one group	↑ L insula<br>↑ L anterior cingulate<br>↑ R insula<br>↑ R anterior cingulate
Geranmayeh et al. (2016): ROI 2	Propositional speech production vs counting	CAC<br>Aphasia vs control	N	UNR	ROI<br>Func<br>NC	Behavioral data notes: difference in AICW/trial; number of ROIs: 4; ROIs: (1) L fronto-temporo-parietal network; (2) R fronto-temporo-parietal network; (3) cingulo-opercular network; (4) default mode network; how ROIs defined: identified using ICA in controls; circular because ROIs defined in one group	↑ L insula<br>↑ L anterior cingulate<br>↑ R insula<br>↑ R anterior cingulate<br>↓ L IFG<br>↓ L inferior parietal lobule<br>↓ L posterior inferior temporal gyrus/fusiform gyrus

Geranmayeh et al. (2016): ROI 3	Propositional speech production vs target decision	CAC Aphasia vs control	N	UNR	ROI Func NC	Behavioral data notes: difference in AICW/trial; number of ROIs: 4; ROIs: (1) L fronto-temporo-parietal network; (2) R fronto-temporo-parietal network; (3) cingulo-opercular network; (4) default mode network; how ROIs defined: identified using ICA in controls; circular because ROIs defined in one group	None
Geranmayeh et al. (2016): Cplx 1	Propositional speech production vs rest	CAC Aphasia vs control	N	UNR	Cplx	Behavioral data notes: difference in AICW/trial; Activity was compared between pairs of ICA-derived networks. However, circularity was introduced because the networks were defined based on the control group.	Other: Patients showed greater differential activation than controls between (1) L fronto-temporo-parietal network and the DMN; (2) R fronto-temporo-parietal network and the DMN; (3) cingulo-opercular network and the DMN.
Geranmayeh et al. (2016): Cplx 2	Propositional speech production vs rest	CC Aphasia Covariate: appropriate information-carrying words	C	UNR	Cplx	Multiple regression was used to determine whether differential activation between networks was predictive of the behavioral measure: appropriate information-carrying words. There is no issue of circularity with this analysis since it involved only individuals with aphasia.	Other: Differential activation between L fronto-temporo-parietal network and the DMN was positively correlated with AICW. Differential activation between R fronto-temporo-parietal network and the DMN was negatively correlated with AICW.
Geranmayeh et al. (2016): Cplx 3	Propositional speech production vs rest	CAC Aphasia vs control	N	UNR	Cplx	Behavioral data notes: difference in AICW/trial; PPI analyses were used to investigate how the speech condition modulated functional connectivity between (1) L fronto-temporo-parietal network and the DMN; (2) R fronto-temporo-parietal network and the DMN. However, circularity was introduced because the networks were defined based on the control group.	Other: In controls, the L FTP network reduced connectivity with the DMN during speech, while the R FTP network increased connectivity with the DMN during speech. Both of these interactions were significantly decreased in patients. This was also true for contrasts 2 and 3.
Griffis et al. (2016): Vox 1	Verb generation vs finger tapping	LA Aphasia T2 vs T1 Somewhat valid (patients improved	UNR	UNR	Vox NC	Search volume: whole brain; software: SPM12; voxelwise p: .001; cluster extent cutoff: none	↑ L IFG pars opercularis ↑ R cerebellum ↑ R thalamus ↓ R anterior

		only on semantic fluency)					temporal<br>↓ R cerebellum<br>notes: based on description in text; it is noted that no regions survived FDR correction
Griffis et al. (2016): ROI 1	Verb generation vs finger tapping	LA<br>Aphasia T2 vs T1<br>Somewhat valid (patients improved only on semantic fluency)	UNR	UNR	ROI<br>Mix<br>FDR	Number of ROIs: 3; ROIs: (1) L IFG; (2) R IFG; (3) frontal LI; how ROIs defined: first principal component of 8 mm spheres defined based on previously reported control peaks; lesion volume included in model	↑ L IFG<br>↓ R IFG<br>↑ LI (frontal)
Griffis et al. (2016): ROI 2	Verb generation vs finger tapping	LC<br>Aphasia T2 vs T1<br>Covariate: Δ semantic fluency<br>Somewhat valid (patients improved only on semantic fluency)	UNR	UNR	ROI<br>Mix<br>FDR	Number of ROIs: 3; ROIs: (1) L IFG; (2) R IFG; (3) frontal LI; how ROIs defined: first principal component of 8 mm spheres defined based on previously reported control peaks; lesion volume included in model	↓ R IFG<br>notes: decreased R IFG activation was correlated with improved semantic fluency
Griffis et al. (2016): Cplx 1	Verb generation vs finger tapping	LA<br>Aphasia T2 vs T1<br>Somewhat valid (patients improved only on semantic fluency)	UNR	UNR	Cplx	PPI analyses were used to investigate change over time in modulation by verb generation of functional connectivity between L IFG and R IFG.	Other:<br>There was a significant decrease in modulation by verb generation of functional connectivity between L IFG and R IFG (p = 0.03). Prior to TMS, connectivity increased during verb generation compared to finger tapping, while after TMS, connectivity decreased during verb generation compared to finger tapping.
Griffis et al. (2016): Cplx 2	Verb generation vs finger tapping	LC<br>Aphasia T2 vs T1<br>Covariate: Δ semantic fluency in association with modulation of interhemispheric IFG connectivity by verb generation<br>Somewhat valid (patients improved only on semantic fluency)	UNR	UNR	Cplx	PPI analyses were used to investigate whether change over time in modulation by verb generation of functional connectivity between L IFG and R IFG was associated with changes in semantic fluency scores, which are limited as a measure of language improvement.	None
Griffis et al. (2016): Cplx 3	Verb generation vs finger tapping	LA<br>Aphasia T2 vs T1<br>Somewhat valid (patients improved only on semantic fluency)	UNR	UNR	Cplx	PPI analyses were used to investigate change over time in modulation by verb generation of functional connectivity between R IFG and all other brain regions. Voxelwise p < .001, not corrected for multiple comparisons.	Other:<br>Reduced connectivity was observed in the L IFG pars opercularis, L anterior temporal

							lobe, L occipital lobe, L basal ganglia, R SMA and pre-SMA, R somato-motor cortex, R posterior MTG, and R cerebellum. It is noted that no regions survived FDR correction.
Sims et al. (2016): ROI 1	Semantic feature decision (6 patients, 4 controls) or semantic relatedness decision (8 patients, 4 controls) vs visual decision or pseudoword identity decision	CC Aphasia Covariate: semantic feature decision accuracy	C	UNR	ROI Anat NC	Number of ROIs: 16; ROIs: (1) L IFG pars orbitalis; (2) L IFG pars opercularis; (3) L IFG pars triangularis; (4) L SFG; (5) L MFG; (6) L MTG; (7) L AG/SMG; (8) L ACC; (9-16) homotopic counterparts; how ROIs defined: AAL	↑ L IFG pars opercularis ↑ L IFG pars triangularis
Sims et al. (2016): ROI 2	Semantic feature decision (6 patients, 4 controls) or semantic relatedness decision (8 patients, 4 controls) vs visual decision or pseudoword identity decision	CC Aphasia Covariate: WAB AQ	UNR	UNR	ROI Anat NC	Number of ROIs: 16; ROIs: (1) L IFG pars orbitalis; (2) L IFG pars opercularis; (3) L IFG pars triangularis; (4) L SFG; (5) L MFG; (6) L MTG; (7) L AG/SMG; (8) L ACC; (9-16) homotopic counterparts; how ROIs defined: AAL	None
Sims et al. (2016): ROI 3	Semantic feature decision (6 patients, 4 controls) or semantic relatedness decision (8 patients, 4 controls) vs visual decision or pseudoword identity decision	CC Aphasia Covariate: BNT	UNR	UNR	ROI Anat NC	Number of ROIs: 16; ROIs: (1) L IFG pars orbitalis; (2) L IFG pars opercularis; (3) L IFG pars triangularis; (4) L SFG; (5) L MFG; (6) L MTG; (7) L AG/SMG; (8) L ACC; (9-16) homotopic counterparts; how ROIs defined: AAL	None
Sims et al. (2016): ROI 4	Semantic feature decision (6 patients, 4 controls) or semantic relatedness decision (8 patients, 4 controls) vs visual decision or pseudoword identity decision	CC Aphasia Covariate: PPT	UNR	UNR	ROI Anat NC	Number of ROIs: 16; ROIs: (1) L IFG pars orbitalis; (2) L IFG pars opercularis; (3) L IFG pars triangularis; (4) L SFG; (5) L MFG; (6) L MTG; (7) L AG/SMG; (8) L ACC; (9-16) homotopic counterparts; how ROIs defined: AAL	None
Sims et al. (2016): ROI 5	Semantic feature decision (6 patients, 4	CC Aphasia	Y	UNR	ROI Anat NC	Behavioral data notes: no correlation between lesion volume and accuracy, not clear whether control condition	↑ R supramarginal gyrus ↑ R angular gyrus

	controls) or semantic relatedness decision (8 patients, 4 controls) vs visual decision or pseudoword identity decision	Covariate: lesion volume				accuracy was also tested; number of ROIs: 8; ROIs: as above but only in the R hemisphere; how ROIs defined: AAL	↑ R posterior MTG notes: MTG included anterior too; SMG/AG was single ROI
Sims et al. (2016): Cplx 1	Semantic feature decision (6 patients, 4 controls) or semantic relatedness decision (8 patients, 4 controls) vs visual decision or pseudoword identity decision	CC<br>Aphasia<br>Covariate: lesion status of 8 ROIs	UNR	UNR	Cplx	Multivariate mixed-effects linear regression analyses were used to identify relationships between structural damage to 8 regions, and functional activation in 16 regions. Results were corrected for multiple comparisons based on FDR. This analysis was not described in sufficient detail.	Other:<br>Sparing of the L ACC and L SFG was associated with more functional activation in many regions, however this is difficult to interpret since these regions were largely or completely spared in many patients. Damage to the L IFG pars orbitalis, L MTG and L AG/SMG was associated with activation of the L ACC, L SFG (and other regions) potentially indicative of compensatory processing.
Sims et al. (2016): Cplx 2	Semantic feature decision (6 patients, 4 controls) or semantic relatedness decision (8 patients, 4 controls) vs visual decision or pseudoword identity decision	CAC<br>Aphasia vs control	UNR	UNR	Cplx	Correlations were computed between functional activation in 16 regions, and qualitatively compared between patients and controls (p. 123). There was no correction for multiple comparisons.	Other:<br>In controls, all regions were generally correlated with one another. This was largely true in patients too, with the exception of the R IFG pars orbitalis, which was negatively correlated with the L IFG.
Darkow et al. (2017): Vox 1	Picture naming vs rest	CAA<br>Aphasia after tDCS (n = 16) vs aphasia after sham stimulation (n = 16); same patients, order counterbalanced, repeated measures<br>Somewhat valid (no behavioral difference)	Y	Y	Vox C+	Search volume: whole brain; software: SPM8; voxelwise p: .001; cluster extent cutoff: based on GRFT; repeated measures	↓ L insula<br>↓ L anterior cingulate<br>↓ R occipital<br>↓ R anterior cingulate
Darkow et al. (2017): ROI 1	Picture naming vs rest	CAC<br>Aphasia after sham stimulation (n = 16) vs control	AS	UNR	ROI Func NC	Behavioral data notes: patients named > 90% correctly in all sessions; control RT not reported; number of ROIs: 3; ROIs: (1) bilateral anterior cingulate;	↑ L insula<br>↑ L anterior cingulate

						(2) L insula; (3) R lingual gyrus; how ROIs defined: regions that were less active in patients with tDCS vs sham; circular because ROIs defined in one group	↑ R anterior cingulate
Darkow et al. (2017): ROI 2	Picture naming vs rest	CAC Aphasia after tDCS (n = 16) vs control	AS	UNR	ROI Func NC	Behavioral data notes: patients named > 90% correctly in all sessions; control RT not reported; number of ROIs: 3; ROIs: (1) bilateral anterior cingulate; (2) L insula; (3) R lingual gyrus; how ROIs defined: regions that were less active in patients with tDCS vs sham; circular because ROIs defined in one group	None
Darkow et al. (2017): Cplx 1	Picture naming vs rest	CAA Aphasia after tDCS (n = 16) vs aphasia after sham stimulation (n = 16); same patients, order counterbalanced, repeated measures Somewhat valid (no behavioral difference)	Y	Y	Cplx	ICA was used to derive three task-relevant components: language, motor and visual. Thresholding of the functional maps is not described, but they appear to reflect coherent components of a picture naming network. These components were compared between stimulation conditions in terms of mean activity and power in three frequency bins. It should be noted that the language component is left-lateralized, unlike the model-based picture naming contrast.	Other: Activity in the language component was greater in the tDCS condition. In the frequency domain, the tDCS condition showed reduced power in the highest frequency bin, and increased power in the lowest frequency bin.
Darkow et al. (2017): Cplx 2	Picture naming vs rest	CAC Aphasia after sham stimulation (n = 16) vs control	UNR	UNR	Cplx	ICA was used to derive three task-relevant components: language, motor and visual. Thresholding of the functional maps is not described, but they appear to reflect coherent components of a picture naming network. These components were compared between stimulation conditions in terms of mean activity and power in three frequency bins. It should be noted that the language component is left-lateralized, unlike the model-based picture naming contrast.	Other: Mean activity of these components did not differ between patients and controls. However, patients showed increased power in the middle frequency bin of the visual component.
Darkow et al. (2017): Cplx 3	Picture naming vs rest	CAC Aphasia after tDCS (n = 16) vs control	UNR	UNR	Cplx	ICA was used to derive three task-relevant components: language, motor and visual. Thresholding of the functional maps is not described, but they appear to reflect coherent components of a picture naming network. These components were compared between stimulation conditions in terms of mean activity and power in three frequency bins. It should be noted that the language component is left-lateralized, unlike the model-based picture naming contrast.	None
Geranmayeh et al. (2017): Vox 1	Propositional speech production vs rest	CC Aphasia mean of T1, T2 Covariate: simultaneous Δ (T2 vs	AM	UNR	Vox CA	Behavioral data notes: T1 AICW correlated with change in AICW, but not stated whether T2 AICW correlated with change in AICW; search volume: voxels spared in all	↑ L SMA/medial prefrontal ↑ L anterior cingulate ↑ R SMA/medial

		T1) number of appropriate information-carrying words<br>Somewhat valid (potentially confounded by T1 and T2 language function; language function at T1 was predictive of change in language function)				patients; software: FSL; voxelwise p: .05; cluster extent cutoff: 1.6 cc	prefrontal<br>↑ R somato-motor<br>↑ R posterior STS<br>↑ R anterior cingulate<br>notes: findings based on figures and coordinates; the pre-SMA/dACC peak noted to survive FWE correction at p < .001
Geranmayeh et al. (2017): ROI 1	Propositional speech production vs rest	LA<br>Aphasia T2 vs T1	N	UNR	ROI Func One	Behavioral data notes: number of AICW increased; number of ROIs: 1; ROI: L pre-SMA; how ROI defined: peak voxel of the contrast of target decision vs mean of propositional speech and counting in people with aphasia; no main effect of session in session by language recovery ANOVA	None
Geranmayeh et al. (2017): ROI 2	Propositional speech production vs rest	LC<br>Aphasia T2 vs T1<br>Covariate: Δ number of appropriate information-carrying words	UNR	UNR	ROI Func One	Number of ROIs: 1; ROI: L pre-SMA; how ROI defined: peak voxel of the contrast of target decision vs mean of propositional speech and counting in people with aphasia; no interaction of session by language recovery in ANOVA	None
Geranmayeh et al. (2017): ROI 3	Propositional speech production vs rest	CC<br>Aphasia mean of T1, T2<br>Covariate: simultaneous Δ (T2 vs T1) number of appropriate information-carrying words<br>Somewhat valid (potentially confounded by T1 and T2 language function; language function at T1 was predictive of change in language function)	AM	UNR	ROI Func One	Behavioral data notes: T1 AICW correlated with change in AICW, but not stated whether T2 AICW correlated with change in AICW; number of ROIs: 1; ROI: L pre-SMA; how ROI defined: peak voxel of the contrast of target decision vs mean of propositional speech and counting in people with aphasia	↑ L SMA/medial prefrontal<br>notes: patients with more pre-SMA activity improved more
Geranmayeh et al. (2017): ROI 4	Propositional speech production vs rest	CC<br>Aphasia mean of T1, T2<br>Covariate: simultaneous Δ (T2 vs T1) number of appropriate information-carrying words<br>Somewhat valid (potentially confounded by T1 and T2 language function; language function at T1 was predictive of change in language function)	AM	UNR	ROI Func One	Behavioral data notes: T1 AICW correlated with change in AICW, but not stated whether T2 AICW correlated with change in AICW; number of ROIs: 1; ROI: L pre-SMA; how ROI defined: peak voxel of the contrast of target decision vs mean of propositional speech and counting in people with aphasia; lesion size covariate	↑ L SMA/medial prefrontal<br>notes: patients with more pre-SMA activity improved more

Geranmayeh et al. (2017): ROI 5	Propositional speech production vs rest	CC<br>Aphasia mean of T1, T2<br>Covariate: simultaneous Δ (T2 vs T1) number of appropriate information-carrying words	AM	UNR	ROI Func One	Behavioral data notes: T1 AICW correlated with change in AICW, but not stated whether T2 AICW correlated with change in AICW; number of ROIs: 1; ROI: L pre-SMA; how ROI defined: peak voxel of the contrast of target decision vs mean of propositional speech and counting in people with aphasia; lesion size, T1 performance, and age covariates	↑ L SMA/medial prefrontal<br>notes: patients with more pre-SMA activity improved more
Geranmayeh et al. (2017): ROI 6	Propositional speech production vs rest	CC<br>Aphasia mean of T1, T2<br>Covariate: subsequent outcome (T2) number of appropriate information-carrying words<br>Not valid (mathematically equivalent to the previous analysis, because of the inclusion of T1 performance as a covariate)	AM	UNR	ROI Func One	Behavioral data notes: T1 AICW correlated with change in AICW, but not stated whether T2 AICW correlated with change in AICW; number of ROIs: 1; ROI: L pre-SMA; how ROI defined: peak voxel of the contrast of target decision vs mean of propositional speech and counting in people with aphasia; lesion size, T1 performance, and age covariates	↑ L SMA/medial prefrontal
Geranmayeh et al. (2017): ROI 7	Propositional speech production vs rest	CC<br>Aphasia T1<br>Covariate: subsequent Δ (T2 vs T1) number of appropriate information-carrying words<br>Somewhat valid (potentially confounded by T1 language function; language function at T1 was predictive of change in language function)	N	UNR	ROI Func One	Behavioral data notes: T1 AICW correlated with change in AICW; number of ROIs: 1; ROI: L pre-SMA; how ROI defined: peak voxel of the contrast of target decision vs mean of propositional speech and counting in people with aphasia	↑ L SMA/medial prefrontal
Geranmayeh et al. (2017): ROI 8	Propositional speech production vs rest	CC<br>Aphasia T2<br>Covariate: previous Δ (T2 vs T1) number of appropriate information-carrying words<br>Not valid (the logic behind correlating activation changes and language outcome is unclear)	AM	UNR	ROI Func One	Behavioral data notes: T1 AICW correlated with change in AICW, but not stated whether T2 AICW correlated with change in AICW; number of ROIs: 1; ROI: L pre-SMA; how ROI defined: peak voxel of the contrast of target decision vs mean of propositional speech and counting in people with aphasia	↑ L SMA/medial prefrontal
Griffis, Nenert, Allendorfer, & Szaflarski (2017): ROI 1	Semantic decision vs tone decision	CC<br>Aphasia<br>Covariate: semantic decision accuracy	C	UNR	ROI Oth FWE	Number of ROIs: 3; ROIs: (1) L AG and bilateral midline components of the canonical semantic network, along with reduced activity in R frontal, temporal and parietal regions; (2) bilateral IFG pars orbitalis; (3) L IFG and DLPFC along with bilateral midline	↑ L IFG<br>↑ L dorsolateral prefrontal cortex<br>↑ L SMA/medial prefrontal<br>↑ L angular gyrus<br>↑ L precuneus

						regions; how ROIs defined: ROIs are mixing coefficients of functional networks arising from mCCA + jICA that were differently represented in the patient and control groups	↑ L posterior cingulate<br>↑ R IFG pars orbitalis<br>↑ R SMA/medial prefrontal<br>↑ R precuneus<br>↑ R posterior cingulate<br>↓ L insula<br>↓ R IFG pars opercularis<br>↓ R IFG pars triangularis<br>↓ R insula<br>↓ R dorsal precentral<br>↓ R supramarginal gyrus<br>↓ R posterior STG<br>↓ R mid temporal<br>notes: all 3 networks were significantly correlated; analysis of networks so involvement of each individual region cannot be assured
Griffis, Nenert, Allendorfer, & Szaflarski (2017):<br>ROI 2	Semantic decision vs tone decision	CC<br>Aphasia<br>Covariate: average of semantic and phonemic fluency	UNR	UNR	ROI<br>Oth<br>FWE	Number of ROIs: 3; ROIs: (1) L AG and bilateral midline components of the canonical semantic network, along with reduced activity in R frontal, temporal and parietal regions; (2) bilateral IFG pars orbitalis; (3) L IFG and DLPFC along with bilateral midline regions; how ROIs defined: ROIs are mixing coefficients of functional networks arising from mCCA + jICA that were differently represented in the patient and control groups	↑ L IFG<br>↑ L dorsolateral prefrontal cortex<br>↑ L SMA/medial prefrontal<br>↑ L angular gyrus<br>↑ L precuneus<br>↑ L posterior cingulate<br>↑ R SMA/medial prefrontal<br>↑ R precuneus<br>↑ R posterior cingulate<br>↓ L insula<br>↓ R IFG pars opercularis<br>↓ R IFG pars triangularis<br>↓ R insula<br>↓ R dorsal precentral<br>↓ R supramarginal gyrus<br>↓ R posterior STG<br>↓ R mid temporal<br>notes: networks 1 and 3 were significantly correlated; analysis of

							networks so involvement of each individual region cannot be assured
Griffis, Nenert, Allendorfer, & Szaflarski (2017): ROI 3	Semantic decision vs tone decision	CC<br>Aphasia<br>Covariate: BNT	UNR	UNR	ROI<br>Oth<br>FWE	Number of ROIs: 3; ROIs: (1) L AG and bilateral midline components of the canonical semantic network, along with reduced activity in R frontal, temporal and parietal regions; (2) bilateral IFG pars orbitalis; (3) L IFG and DLPFC along with bilateral midline regions; how ROIs defined: ROIs are mixing coefficients of functional networks arising from mCCA + jICA that were differently represented in the patient and control groups	↑ L IFG<br>↑ L dorsolateral prefrontal cortex<br>↑ L SMA/medial prefrontal<br>↑ L angular gyrus<br>↑ L precuneus<br>↑ L posterior cingulate<br>↑ R SMA/medial prefrontal<br>↑ R precuneus<br>↑ R posterior cingulate<br>↓ L insula<br>↓ R IFG pars opercularis<br>↓ R IFG pars triangularis<br>↓ R insula<br>↓ R dorsal precentral<br>↓ R supramarginal gyrus<br>↓ R posterior STG<br>↓ R mid temporal<br>notes: networks 1 and 3 were significantly correlated; analysis of networks so involvement of each individual region cannot be assured
Griffis, Nenert, Allendorfer, & Szaflarski (2017): Cplx 1	Semantic decision vs tone decision	CAC<br>Aphasia vs control	N	UNR	Cplx	Behavioral data notes: semantic decision accuracy not matched, but tone decision accuracy not reported; Multimodal canonical correlation analysis (mCCA) and joint ICA were used to identify 3 joint ICs (structural/functional) that were differently represented in the patient and control groups. Although there was no correction for multiple comparisons when the functional maps were thresholded, the maps for the three networks each appeared to relate to coherent parts of the semantic network.	Other:<br>The first joint IC comprised preservation of tissue in L posterior temporo-parietal region, activity in the L AG and bilateral midline components of the canonical semantic network, and reduced activity in R frontal, temporal and parietal regions. The second joint IC comprised preservation of

							tissue in the the L basal ganglia/insula region, and activity predominantly in the IFG pars orbitalis bilaterally. The third joint IC comprised preservation of the L IFG and activity in the L IFG and DLPFC along with bilateral midline regions. The first joint IC was considered to provide more robust evidence for structure-function relationships than the other two, because it was the only one where individual structural and functional mixing coefficients remained correlated even when lesion volume was included as a covariate.
Griffis, Nenert, Allendorfer, Vannest, et al. (2017): Vox 1	Semantic decision vs tone decision	CC<br>Aphasia<br>Covariate: semantic decision accuracy	C	UNR	Vox<br>CCTB	Search volume: whole brain; software: SPM12/in-house; voxelwise p: .01; cluster extent cutoff: 126 voxels (size not stated); lesion volume covariate	↑ L dorsolateral prefrontal cortex<br>↑ L angular gyrus<br>↑ L precuneus<br>↑ L mid temporal<br>↑ L anterior temporal<br>↑ L posterior cingulate<br>↑ L cerebellum<br>↑ L brainstem<br>↑ L hippocampus/MTL<br>↑ R IFG pars orbitalis<br>↑ R angular gyrus<br>↑ R precuneus<br>↑ R anterior temporal<br>↑ R occipital<br>↑ R brainstem<br>↑ R hippocampus/MTL<br>↓ L somato-motor<br>notes: based on figure and table; larger activations

							are compelling; smaller activations are not due to lenient correction approach
Griffis, Nenert, Allendorfer, Vannest, et al. (2017): Vox 2	Semantic decision vs tone decision	CC<br>Aphasia<br>Covariate: average of semantic and phonemic fluency	UNR	UNR	Vox<br>CCTB	Search volume: whole brain; software: SPM12/in-house; voxelwise p: .01; cluster extent cutoff: 126 voxels (size not stated); lesion volume covariate	↑ L IFG<br>↑ L dorsolateral prefrontal cortex<br>↑ L SMA/medial prefrontal<br>↑ L angular gyrus<br>↑ L precuneus<br>↑ L posterior STS<br>↑ L mid temporal<br>↑ L anterior temporal<br>↑ L posterior cingulate<br>↑ L brainstem<br>↑ L hippocampus/MTL<br>↑ R SMA/medial prefrontal<br>↑ R precuneus<br>↑ R anterior temporal<br>↑ R occipital<br>↑ R posterior cingulate<br>↑ R hippocampus/MTL<br>↓ R posterior STS<br>notes: based on figure and table; larger activations are compelling; smaller activations are not due to lenient correction approach
Griffis, Nenert, Allendorfer, Vannest, et al. (2017): Vox 3	Semantic decision vs tone decision	CC<br>Aphasia<br>Covariate: BNT	UNR	UNR	Vox<br>CCTB	Search volume: whole brain; software: SPM12/in-house; voxelwise p: .01; cluster extent cutoff: 126 voxels (size not stated); lesion volume covariate	↑ L IFG pars orbitalis<br>↑ L SMA/medial prefrontal<br>↑ L angular gyrus<br>↑ L precuneus<br>↑ L posterior cingulate<br>↑ L hippocampus/MTL<br>↑ R IFG pars orbitalis<br>↑ R SMA/medial prefrontal<br>↑ R precuneus<br>↑ R anterior temporal<br>↑ R posterior cingulate<br>↑ R cerebellum<br>notes: based on figure and table;

							larger activations are compelling; smaller activations are not due to lenient correction approach
Griffis, Nenert, Allendorfer, Vannest, et al. (2017): Vox 4	Semantic decision vs tone decision	CC<br>Aphasia<br>Covariate: lesion volume	UNR	UNR	Vox<br>CCTB	Search volume: R hemisphere; software: SPM12/in-house; voxelwise p: .01; cluster extent cutoff: 126 voxels (size not stated)	↑ R IFG pars opercularis<br>↑ R dorsolateral prefrontal cortex<br>↑ R dorsal precentral<br>↑ R SMA/medial prefrontal<br>↓ R orbitofrontal<br>↓ R anterior temporal<br>↓ R cerebellum<br>↓ R thalamus<br>notes: based on figure and table; larger activations are compelling; smaller activations are not due to lenient correction approach
Griffis, Nenert, Allendorfer, Vannest, et al. (2017): ROI 1	Semantic decision vs tone decision	CAC<br>Aphasia vs control	N	UNR	ROI<br>Func<br>FWE	Behavioral data notes: semantic decision accuracy not matched, but tone decision accuracy not reported; number of ROIs: 5; ROIs: (1) overall canonical semantic network (CSN); (2) L CSN; (3) R CSN; (4) mirror L CSN in R; (5) out-of-network CSN in R; how ROIs defined: control data; circular because ROI defined in one group	↓ L IFG<br>↓ L dorsolateral prefrontal cortex<br>↓ L SMA/medial prefrontal<br>↓ L angular gyrus<br>↓ L precuneus<br>↓ L mid temporal<br>↓ L anterior temporal<br>↓ L occipital<br>↓ L posterior cingulate<br>↓ L cerebellum<br>↓ R IFG<br>↓ R dorsolateral prefrontal cortex<br>↓ R SMA/medial prefrontal<br>↓ R angular gyrus<br>↓ R precuneus<br>↓ R anterior temporal<br>↓ R occipital<br>↓ R posterior cingulate<br>↓ R cerebellum<br>notes: results are for whole networks of regions, so individual regions cannot be assured; out-of-network R regions

							not listed since they were not significant in ROI 5 (only in ROI 4)
Griffis, Nenert, Allendorfer, Vannest, et al. (2017): ROI 2	Semantic decision vs tone decision	CC<br>Aphasia<br>Covariate: lesion volume	UNR	UNR	ROI Func FWE	Number of ROIs: 5; ROIs: (1) overall canonical semantic network (CSN); (2) L CSN; (3) R CSN; (4) mirror L CSN in R; (5) out-of-network CSN in R; how ROIs defined: control data	None
Griffis, Nenert, Allendorfer, Vannest, et al. (2017): ROI 3	Semantic decision vs tone decision	CC<br>Aphasia<br>Covariate: semantic decision accuracy	C	UNR	ROI Func One	Number of ROIs: 1; ROI: CSN; how ROI defined: control data; lesion volume covariate	↑ L IFG<br>↑ L dorsolateral prefrontal cortex<br>↑ L SMA/medial prefrontal<br>↑ L angular gyrus<br>↑ L precuneus<br>↑ L mid temporal<br>↑ L anterior temporal<br>↑ L posterior cingulate<br>↑ L cerebellum<br>↑ R IFG<br>↑ R dorsolateral prefrontal cortex<br>↑ R SMA/medial prefrontal<br>↑ R angular gyrus<br>↑ R precuneus<br>↑ R anterior temporal<br>↑ R posterior cingulate<br>↑ R cerebellum<br>notes: correlation calculated for the whole network of regions, so correlation of individual regions cannot be assured
Griffis, Nenert, Allendorfer, Vannest, et al. (2017): ROI 4	Semantic decision vs tone decision	CC<br>Aphasia<br>Covariate: average of semantic and phonemic fluency	UNR	UNR	ROI Func One	Number of ROIs: 1; ROI: CSN; how ROI defined: control data; lesion volume covariate	↑ L IFG<br>↑ L dorsolateral prefrontal cortex<br>↑ L SMA/medial prefrontal<br>↑ L angular gyrus<br>↑ L precuneus<br>↑ L mid temporal<br>↑ L anterior temporal<br>↑ L posterior cingulate<br>↑ L cerebellum<br>↑ R IFG<br>↑ R dorsolateral prefrontal cortex<br>↑ R SMA/medial prefrontal<br>↑ R angular gyrus<br>↑ R precuneus

							↑ R anterior temporal<br>↑ R posterior cingulate<br>↑ R cerebellum<br>notes: correlation calculated for the whole network of regions, so correlation of individual regions cannot be assured
Griffis, Nenert, Allendorfer, Vannest, et al. (2017): ROI 5	Semantic decision vs tone decision	CC<br>Aphasia<br>Covariate: BNT	UNR	UNR	ROI Func One	Number of ROIs: 1; ROI: CSN; how ROI defined: control data; lesion volume covariate	↑ L IFG<br>↑ L dorsolateral prefrontal cortex<br>↑ L SMA/medial prefrontal<br>↑ L angular gyrus<br>↑ L precuneus<br>↑ L mid temporal<br>↑ L anterior temporal<br>↑ L posterior cingulate<br>↑ L cerebellum<br>↑ R IFG<br>↑ R dorsolateral prefrontal cortex<br>↑ R SMA/medial prefrontal<br>↑ R angular gyrus<br>↑ R precuneus<br>↑ R anterior temporal<br>↑ R posterior cingulate<br>↑ R cerebellum<br>notes: correlation calculated for the whole network of regions, so correlation of individual regions cannot be assured
Griffis, Nenert, Allendorfer, Vannest, et al. (2017): Cplx 1	Semantic decision vs tone decision	CAC<br>Aphasia vs control	N	UNR	Cplx	Behavioral data notes: semantic decision accuracy not matched, but tone decision accuracy not reported; Correlations between activation magnitudes in the L and R canonical semantic network (CSN) were compared between groups. However, this analysis is circular because the CSN ROIs were defined based on controls only.	None
Griffis, Nenert, Allendorfer, Vannest, et al. (2017): Cplx 2	Semantic decision vs tone decision	CAC<br>Aphasia vs control	N	UNR	Cplx	Behavioral data notes: semantic decision accuracy not matched, but tone decision accuracy not reported; Correlations between activation magnitudes in the L CSN and R mirrored CSN were compared between groups. However, this analysis is circular because the CSN	Other: Correlations between activations in the L CSN and the mirrored L CSN in the R hemisphere were stronger in

						ROIs were defined based on controls only.	patients than controls.
Griffis, Nenert, Allendorfer, Vannest, et al. (2017): Cplx 3	Semantic decision vs tone decision	CAC Aphasia vs control	N	UNR	Cplx	Behavioral data notes: semantic decision accuracy not matched, but tone decision accuracy not reported; Correlations between activation magnitudes in the L CSN and R out-of-network homotopic regions were compared between groups. However, this analysis is circular because the CSN ROIs were defined based on controls only.	Other: Correlations between activations in the L CSN and R out-of-network homotopic regions were stronger in patients than controls.
Griffis, Nenert, Allendorfer, Vannest, et al. (2017): Cplx 4	Semantic decision vs tone decision	CAC Aphasia vs control	N	UNR	Cplx	Behavioral data notes: semantic decision accuracy not matched, but tone decision accuracy not reported; The difference in activation between the L CSN and R CSN was compared between patients and controls. However, this analysis is circular because the CSN ROIs were defined based on controls only.	None
Griffis, Nenert, Allendorfer, Vannest, et al. (2017): Cplx 5	Semantic decision vs tone decision	CAC Aphasia vs control	N	UNR	Cplx	Behavioral data notes: semantic decision accuracy not matched, but tone decision accuracy not reported; The difference in activation between the L CSN and mirror L CSN in the R was compared between patients and controls. However, this analysis is circular because the CSN ROIs were defined based on controls only.	Other: The difference was smaller in patients.
Griffis, Nenert, Allendorfer, Vannest, et al. (2017): Cplx 6	Semantic decision vs tone decision	CAC Aphasia vs control	N	UNR	Cplx	Behavioral data notes: semantic decision accuracy not matched, but tone decision accuracy not reported; The difference in activation between the R CSN and out-of-network homotopic regions in the R was compared between patients and controls. However, this analysis is circular because the CSN ROIs were defined based on controls only.	Other: The difference was smaller in patients.
Griffis, Nenert, Allendorfer, Vannest, et al. (2017): Cplx 7	Semantic decision vs tone decision	CC Aphasia Covariate: interactions of semantic fluency and naming measures by lesion size	UNR	UNR	Cplx	For the 4 R hemisphere regions that were more activated in patients with larger lesions (SPM analysis 4), analyses were carried out to determine whether the semantic fluency or naming measures were differentially impacted by activation depending on whether lesions were larger or smaller.	Other: For 1 of the 4 regions (R SMA), there were significant interactions such that in patients with larger lesions, more activation was associated with higher semantic fluency scores and higher BNT scores, while in patients with smaller lesions, more activation was associated with lower fluency and BNT scores. There was a similar

							relationship with semantic fluency in the R IFG pars opercularis but only at p(FDR) = 0.07.
Harvey et al. (2017): Vox 1	Picture naming vs viewing patterns	LA<br>Aphasia T3 vs T1	UNR	UNR	Vox<br>NDC	Search volume: voxels spared in all patients; software: SPM8; qualitative comparison on pp. 138-9	↑ L SMA/medial prefrontal<br>↑ L posterior inferior temporal gyrus/fusiform gyrus<br>↑ L occipital<br>↑ L anterior cingulate<br>↑ R IFG pars opercularis<br>↑ R ventral precentral/inferior frontal junction<br>↓ L dorsolateral prefrontal cortex<br>↓ R IFG pars triangularis<br>↓ R posterior inferior temporal gyrus/fusiform gyrus<br>↓ R occipital<br>↓ R hippocampus/MTL<br>notes: based on Figure 5 and Table 4
Nardo et al. (2017): Vox 1	Picture naming (all conditions, correct trials) vs rest	LA<br>Aphasia T2 vs T1	YCT	N	Vox<br>VFWE	Behavioral data notes: RT faster at T2; search volume: whole brain; software: SPM12; voxelwise p: FWE p < .05	None
Nardo et al. (2017): ROI 1	Picture naming (untrained items, no cue, correct trials) vs picture naming (trained items, no cue, correct trials)	CC<br>Aphasia T2<br>Covariate: "a change in un-cued naming RT" (exact measure unclear)<br>Somewhat valid (unclear whether behavioral measure is longitudinal)	YCT	UNR	ROI<br>Func<br>NC	Number of ROIs: 4; ROIs: (1) R anterior insula; (2) R IFG; (3) dorsal anterior cingulate; (4) L premotor cortex; how ROIs defined: peaks (only with SVC) for the main effect of untrained (4 conditions) vs trained (4 conditions) in T2 aphasia; unclear what the behavioral measure was exactly	↑ R IFG pars opercularis<br>↑ R insula
Nenert et al. (2017): Vox 1	Semantic decision vs tone decision	CAA<br>Aphasia CIAT T2 (n = 11) vs untreated T2 (n = 8)<br>Somewhat valid (no treatment effect)	AS	UNR	Vox<br>CA	Search volume: voxels spared in all patients; software: SPM12; voxelwise p: .01; cluster extent cutoff: 50 voxels (size not stated)	↑ L somato-motor<br>↑ L superior parietal<br>↑ L brainstem<br>↑ R ventral precentral/inferior frontal junction<br>↑ R somato-motor<br>↑ R superior parietal<br>notes: based on coordinates in Table 4
Nenert et al. (2017):	Semantic decision vs tone decision	CAA<br>Aphasia CIAT T3 (n =	UNT	UNR	Vox<br>CA	Search volume: voxels spared in all patients; software: SPM12; voxelwise	↑ L superior parietal

Vox 2		11) vs untreated T3 (n = 8)<br>Somewhat valid (no treatment effect)				p: .01; cluster extent cutoff: 50 voxels (size not stated)	↑ L anterior temporal<br>↑ L hippocampus/MTL<br>↑ R orbitofrontal<br>↓ L dorsolateral prefrontal cortex<br>↓ L posterior inferior temporal gyrus/fusiform gyrus<br>↓ R IFG pars orbitalis<br>↓ R ventral precentral/inferior frontal junction<br>↓ R posterior STS<br>notes: based on coordinates in Table 4
Nenert et al. (2017): Vox 3	Verb generation vs finger tapping	CAA<br>Aphasia CIAT T2 (n = 11) vs untreated T2 (n = 8)<br>Somewhat valid (no treatment effect)	UNR	UNR	Vox CA	Search volume: voxels spared in all patients; software: SPM12; voxelwise p: .01; cluster extent cutoff: 50 voxels (size not stated)	↓ L precuneus<br>↓ R dorsolateral prefrontal cortex<br>↓ R posterior STS<br>↓ R anterior temporal<br>↓ R posterior inferior temporal gyrus/fusiform gyrus<br>notes: based on coordinates in Table 4
Nenert et al. (2017): Vox 4	Verb generation vs finger tapping	CAA<br>Aphasia CIAT T3 (n = 11) vs untreated T3 (n = 8)<br>Somewhat valid (no treatment effect)	UNR	UNR	Vox CA	Search volume: voxels spared in all patients; software: SPM12; voxelwise p: .01; cluster extent cutoff: 50 voxels (size not stated)	↑ L SMA/medial prefrontal<br>↑ R basal ganglia<br>↓ L anterior temporal<br>↓ R posterior STS<br>↓ R Heschl's gyrus<br>↓ R posterior inferior temporal gyrus/fusiform gyrus
Nenert et al. (2017): Vox 5	Semantic decision vs tone decision	CAC<br>Aphasia CIAT T1 (n = 11) vs control	AM	UNR	Vox CA	Behavioral data notes: patients less accurate than controls on both tasks, but more so on the tone decision task; search volume: voxels spared in all patients; software: SPM12; voxelwise p: .01; cluster extent cutoff: 50 voxels (size not stated)	↑ L orbitofrontal<br>↑ L hippocampus/MTL<br>↑ R IFG pars opercularis<br>↑ R SMA/medial prefrontal<br>↑ R supramarginal gyrus<br>↑ R posterior STG/STS/MTG<br>↑ R anterior temporal<br>↑ R anterior cingulate<br>↓ R dorsolateral prefrontal cortex
Nenert et	Semantic decision	CAC	AM	UNR	Vox	Behavioral data notes: patients less	↑ L anterior

al. (2017): Vox 6	vs tone decision	Aphasia CIAT T2 (n = 11) vs control			CA	accurate than controls on both tasks, but more so on the tone decision task; search volume: voxels spared in all patients; software: SPM12; voxelwise p: .01; cluster extent cutoff: 50 voxels (size not stated)	cingulate<br>↑ R IFG pars opercularis<br>↑ R insula<br>↑ R ventral precentral/inferior frontal junction<br>↑ R supramarginal gyrus<br>↑ R Heschl's gyrus<br>↓ L dorsolateral prefrontal cortex<br>↓ L SMA/medial prefrontal<br>↓ L cerebellum<br>↓ R dorsolateral prefrontal cortex
Nenert et al. (2017): Vox 7	Semantic decision vs tone decision	CAC<br>Aphasia CIAT T3 (n = 11) vs control	AM	UNR	Vox CA	Behavioral data notes: patients less accurate than controls on both tasks, but more so on the tone decision task; search volume: voxels spared in all patients; software: SPM12; voxelwise p: .01; cluster extent cutoff: 50 voxels (size not stated)	↑ L orbitofrontal<br>↑ L anterior cingulate<br>↑ L hippocampus/MTL<br>↑ R superior parietal<br>↓ L cerebellum<br>↓ R dorsolateral prefrontal cortex<br>↓ R anterior temporal<br>↓ R cerebellum
Nenert et al. (2017): Vox 8	Semantic decision vs tone decision	CAC<br>Aphasia untreated T1 (n = 8) vs control	AM	UNR	Vox CA	Behavioral data notes: patients less accurate than controls on both tasks, but more so on the tone decision task; search volume: voxels spared in all patients; software: SPM12; voxelwise p: .01; cluster extent cutoff: 50 voxels (size not stated)	↑ L dorsolateral prefrontal cortex<br>↑ R dorsolateral prefrontal cortex<br>↑ R SMA/medial prefrontal<br>↑ R somato-motor<br>↓ L IFG pars orbitalis<br>↓ L dorsolateral prefrontal cortex<br>↓ L SMA/medial prefrontal<br>↓ L angular gyrus<br>↓ L mid temporal<br>↓ L anterior temporal<br>↓ R IFG pars orbitalis<br>↓ R angular gyrus<br>↓ R anterior temporal<br>↓ R posterior inferior temporal gyrus/fusiform gyrus
Nenert et al. (2017): Vox 9	Semantic decision vs tone decision	CAC<br>Aphasia untreated T2 (n = 8) vs control	AM	UNR	Vox CA	Behavioral data notes: patients less accurate than controls on both tasks, but more so on the tone decision task; search volume: voxels spared in all patients; software: SPM12; voxelwise	↑ L posterior inferior temporal gyrus/fusiform gyrus<br>↑ R dorsolateral prefrontal cortex

						p: .01; cluster extent cutoff: 50 voxels (size not stated)	↑ R orbitofrontal<br>↑ R mid temporal<br>↓ L IFG pars orbitalis<br>↓ L SMA/medial prefrontal<br>↓ L orbitofrontal<br>↓ L intraparietal sulcus<br>↓ L superior parietal<br>↓ L anterior cingulate<br>↓ L brainstem<br>↓ R IFG pars orbitalis<br>↓ R dorsolateral prefrontal cortex<br>↓ R inferior parietal lobule<br>↓ R supramarginal gyrus<br>↓ R anterior temporal<br>↓ R posterior inferior temporal gyrus/fusiform gyrus<br>↓ R hippocampus/MTL
Nenert et al. (2017): Vox 10	Semantic decision vs tone decision	CAC<br>Aphasia untreated T3 (n = 8) vs control	AM	UNR	Vox CA	Behavioral data notes: patients less accurate than controls on both tasks, but not significantly for the semantic decision task, and more so on the tone decision task; search volume: voxels spared in all patients; software: SPM12; voxelwise p: .01; cluster extent cutoff: 50 voxels (size not stated)	↑ L dorsolateral prefrontal cortex<br>↑ R dorsolateral prefrontal cortex<br>↑ R SMA/medial prefrontal<br>↑ R orbitofrontal<br>↑ R superior parietal<br>↑ R cerebellum<br>↓ L orbitofrontal<br>↓ L mid temporal<br>↓ L anterior temporal<br>↓ L posterior cingulate<br>↓ L cerebellum<br>↓ L hippocampus/MTL<br>↓ R angular gyrus<br>↓ R anterior temporal
Nenert et al. (2017): Vox 11	Verb generation vs finger tapping	CAC<br>Aphasia CIAT T1 (n = 11) vs control	UNR	UNR	Vox CA	Search volume: voxels spared in all patients; software: SPM12; voxelwise p: .01; cluster extent cutoff: 50 voxels (size not stated)	↑ L dorsal precentral<br>↑ L superior parietal<br>↑ R cerebellum<br>↓ L dorsolateral prefrontal cortex<br>↓ L SMA/medial prefrontal<br>↓ R posterior

							inferior temporal gyrus/fusiform gyrus
Nenert et al. (2017): Vox 12	Verb generation vs finger tapping	CAC<br>Aphasia CIAT T2 (n = 11) vs control	UNR	UNR	Vox CA	Search volume: voxels spared in all patients; software: SPM12; voxelwise p: .01; cluster extent cutoff: 50 voxels (size not stated)	↑ L dorsal precentral<br>↑ L anterior cingulate<br>↓ L IFG pars orbitalis<br>↓ L dorsolateral prefrontal cortex<br>↓ L SMA/medial prefrontal<br>↓ L superior parietal<br>↓ L posterior inferior temporal gyrus/fusiform gyrus<br>↓ L occipital<br>↓ R IFG pars orbitalis
Nenert et al. (2017): Vox 13	Verb generation vs finger tapping	CAC<br>Aphasia CIAT T3 (n = 11) vs control	UNR	UNR	Vox CA	Search volume: voxels spared in all patients; software: SPM12; voxelwise p: .01; cluster extent cutoff: 50 voxels (size not stated)	↑ L somato-motor<br>↑ L anterior cingulate<br>↑ L posterior cingulate<br>↓ L IFG pars orbitalis<br>↓ L dorsolateral prefrontal cortex<br>↓ L superior parietal<br>↓ L posterior inferior temporal gyrus/fusiform gyrus<br>↓ R dorsolateral prefrontal cortex<br>↓ R mid temporal
Nenert et al. (2017): Vox 14	Verb generation vs finger tapping	CAC<br>Aphasia untreated T1 (n = 8) vs control	UNR	UNR	Vox CA	Search volume: voxels spared in all patients; software: SPM12; voxelwise p: .01; cluster extent cutoff: 50 voxels (size not stated)	↑ L superior parietal<br>↑ L occipital<br>↑ L cerebellum<br>↑ R dorsolateral prefrontal cortex<br>↑ R cerebellum<br>↓ L IFG pars orbitalis<br>↓ L SMA/medial prefrontal<br>↓ L posterior inferior temporal gyrus/fusiform gyrus<br>↓ L cerebellum<br>↓ R superior parietal
Nenert et al. (2017): Vox 15	Verb generation vs finger tapping	CAC<br>Aphasia untreated T2 (n = 8) vs control	UNR	UNR	Vox CA	Search volume: voxels spared in all patients; software: SPM12; voxelwise p: .01; cluster extent cutoff: 50 voxels (size not stated)	↑ L dorsolateral prefrontal cortex<br>↑ R SMA/medial prefrontal

							↑ R angular gyrus<br>↑ R posterior STG<br>↑ R posterior cingulate<br>↑ R cerebellum<br>↓ L dorsolateral prefrontal cortex<br>↓ L SMA/medial prefrontal<br>↓ L superior parietal<br>↓ L anterior temporal<br>↓ L posterior inferior temporal gyrus/fusiform gyrus<br>↓ L occipital<br>↓ R superior parietal<br>↓ R occipital<br>↓ R cerebellum
Nenert et al. (2017): Vox 16	Verb generation vs finger tapping	CAC<br>Aphasia untreated T3 (n = 8) vs control	UNR	UNR	Vox<br>CA	Search volume: voxels spared in all patients; software: SPM12; voxelwise p: .01; cluster extent cutoff: 50 voxels (size not stated)	↑ L superior parietal<br>↑ L anterior temporal<br>↑ L occipital<br>↑ R insula<br>↑ R ventral precentral/inferior frontal junction<br>↑ R orbitofrontal<br>↑ R occipital<br>↑ R cerebellum<br>↓ L IFG pars orbitalis<br>↓ L SMA/medial prefrontal<br>↓ L superior parietal<br>↓ L occipital<br>↓ R insula<br>↓ R dorsolateral prefrontal cortex<br>↓ R cerebellum<br>↓ R basal ganglia
Nenert et al. (2017): Vox 17	Semantic decision vs tone decision	LC<br>Aphasia T2 vs T1<br>Covariate: Δ BNT	UNR	UNR	Vox<br>CA	Search volume: voxels spared in all patients; software: SPM12; voxelwise p: .01; cluster extent cutoff: 50 voxels (size not stated)	↑ R insula<br>↑ R anterior cingulate<br>↑ R cerebellum<br>↑ R brainstem<br>↑ R basal ganglia
Nenert et al. (2017): Vox 18	Semantic decision vs tone decision	LC<br>Aphasia T3 vs T2<br>Covariate: Δ BNT<br>Somewhat valid (no treatment effect)	UNR	UNR	Vox<br>CA	Search volume: voxels spared in all patients; software: SPM12; voxelwise p: .01; cluster extent cutoff: 50 voxels (size not stated)	↑ R somato-motor<br>↑ R posterior MTG<br>↑ R thalamus
Nenert et al. (2017): Vox 19	Verb generation vs finger tapping	LC<br>Aphasia T2 vs T1<br>Covariate: Δ BNT	UNR	UNR	Vox<br>CA	Search volume: voxels spared in all patients; software: SPM12; voxelwise p: .01; cluster extent cutoff: 50 voxels (size not stated)	↑ R orbitofrontal<br>↑ R mid temporal
Nenert et al	Verb generation vs	LC	UNR	UNR	Vox	Search volume: voxels spared in all	↑ L dorsolateral

al. (2017): Vox 20	finger tapping	Aphasia T3 vs T2<br>Covariate: Δ BNT<br>Somewhat valid (no treatment effect)			CA	patients; software: SPM12; voxelwise p: .01; cluster extent cutoff: 50 voxels (size not stated)	prefrontal cortex<br>↑ R dorsolateral prefrontal cortex<br>↑ R orbitofrontal
Nenert et al. (2017): ROI 1	Semantic decision vs tone decision	LA<br>Aphasia ANOVA including T1, T2, T3	AS	UNR	ROI LI NC	Number of ROIs: 5; ROIs: (1) frontal LI; (2) temporo-parietal LI; (3) cerebellar LI; (4) fronto-parietal LI; (5) Broca's LI	None
Nenert et al. (2017): ROI 2	Semantic decision vs tone decision	LAA<br>(Aphasia CIAT (n = 11) T1 ≠ T2 ≠ T3) vs (untreated (n = 8) T1 ≠ T2 ≠ T3)<br>Somewhat valid (no treatment effect)	AS	UNR	ROI LI NC	Number of ROIs: 5; ROIs: (1) frontal LI; (2) temporo-parietal LI; (3) cerebellar LI; (4) fronto-parietal LI; (5) Broca's LI	None
Nenert et al. (2017): ROI 3	Verb generation vs finger tapping	LA<br>Aphasia ANOVA including T1, T2, T3	UNR	UNR	ROI LI NC	Number of ROIs: 5; ROIs: (1) frontal LI; (2) temporo-parietal LI; (3) cerebellar LI; (4) fronto-parietal LI; (5) Broca's LI	None
Nenert et al. (2017): ROI 4	Verb generation vs finger tapping	LAA<br>(Aphasia CIAT (n = 11) T1 ≠ T2 ≠ T3) vs (untreated (n = 8) T1 ≠ T2 ≠ T3)<br>Somewhat valid (no treatment effect)	UNR	UNR	ROI LI NC	Number of ROIs: 5; ROIs: (1) frontal LI; (2) temporo-parietal LI; (3) cerebellar LI; (4) fronto-parietal LI; (5) Broca's LI	None
Qiu et al. (2017): Vox 1	Picture naming vs rest	CAC<br>Aphasia vs control	UNR	UNR	Vox CA	Search volume: whole brain; software: SPM8; voxelwise p: .05; cluster extent cutoff: 10 voxels (size not stated); in the footnote to Table 2, there is a reference to FWE correction with Monte Carlo simulation, but this is not described in the text, and the values in the table appear to be inconsistent with that	↑ L intraparietal sulcus<br>↑ L posterior inferior temporal gyrus/fusiform gyrus<br>↑ L occipital<br>↑ L thalamus<br>↑ R inferior parietal lobule<br>↑ R intraparietal sulcus<br>↑ R precuneus<br>↑ R anterior temporal<br>↓ L IFG<br>↓ L orbitofrontal<br>↓ L somato-motor<br>↓ R ventral precentral/inferior frontal junction<br>notes: findings are based on coordinates, which in many cases do not match the labels assigned in the paper
Skipper-Kallal et al. (2017a): Vox 1	Picture naming (silently name, correct trials) vs rest	CAC<br>Aphasia vs control	YCT	UNR	Vox C-	Behavioral data notes: covert phase but accuracy derived from overt phase; search volume: whole brain gray matter; software: FSL 5.0.6; voxelwise p: ~.01 (z > 2.3); cluster extent cutoff: based on GRFT; threshold of z > 3.1 mentioned in results, but presume 2.3 based on methods and figure	↑ R precuneus<br>↓ L occipital

Skipper-Kallal et al. (2017a): Vox 2	Picture naming (produce the name, correct trials) vs rest	CAC<br>Aphasia vs control	YCT	UNR	Vox C-	Search volume: whole brain gray matter; software: FSL 5.0.6; voxelwise p: ~.01 (z > 2.3); cluster extent cutoff: based on GRFT; threshold of z > 3.1 mentioned in results, but presume 2.3 based on methods and figure	↑ L SMA/medial prefrontal<br>↑ L orbitofrontal<br>↑ L precuneus<br>↑ R insula<br>↑ R ventral precentral/inferior frontal junction<br>↑ R SMA/medial prefrontal<br>↑ R orbitofrontal<br>↑ R somato-motor<br>↑ R supramarginal gyrus<br>↑ R posterior STS<br>↓ L IFG<br>↓ L insula<br>↓ L ventral precentral/inferior frontal junction<br>↓ L intraparietal sulcus<br>↓ L anterior temporal<br>↓ L hippocampus/MTL<br>↓ R intraparietal sulcus<br>notes: labels based largely on text with some adjustments based on figures; overall pattern of decreased L activity and increased R activity is quite convincing
Skipper-Kallal et al. (2017a): Vox 3	Picture naming (silently name, correct trials) vs rest	CC<br>Aphasia<br>Covariate: PNT	YCT	UNR	Vox C-	Behavioral data notes: covert phase but accuracy derived from overt phase; search volume: whole brain gray matter; software: FSL 5.0.6; voxelwise p: ~.01 (z > 2.3); cluster extent cutoff: based on GRFT	↑ L anterior temporal<br>↓ L SMA/medial prefrontal<br>↓ L supramarginal gyrus<br>↓ R SMA/medial prefrontal<br>↓ R somato-motor<br>notes: L anterior temporal correlation remained significant after accounting for lesion load and other factors
Skipper-Kallal et al. (2017a): Vox 4	Picture naming (produce the name, correct trials) vs rest	CC<br>Aphasia<br>Covariate: PNT	YCT	UNR	Vox C-	Search volume: whole brain gray matter; software: FSL 5.0.6; voxelwise p: ~.01 (z > 2.3); cluster extent cutoff: based on GRFT	↑ L posterior STG<br>↑ R somato-motor<br>↑ R posterior STS<br>↑ R occipital<br>↓ L IFG pars orbitalis

							↓ L dorsolateral prefrontal cortex<br>↓ L angular gyrus<br>notes: L IFG pars orbitalis, R pSTS, and R somato-motor correlations remained remained significant after accounting for lesion load and other factors; note that the pars orbitalis region is described as frontal pole in the paper but the coordinates and image support pars orbitalis
Skipper-Kallal et al. (2017a): Vox 5	Picture naming (both phases, correct trials) vs picture naming (both phases, incorrect trials)	CB<br>Aphasia with naming < 80% (n = 24)	NBD	UNR	Vox<br>C-	Search volume: whole brain gray matter; software: FSL 5.0.6; voxelwise p: ~.01 (z > 2.3); cluster extent cutoff: based on GRFT	None
Skipper-Kallal et al. (2017a): ROI 1	Picture naming (produce the name, correct trials) vs rest	CC<br>Aphasia<br>Covariate: PNT	YCT	UNR	ROI<br>Func<br>FWE	Number of ROIs: 11; ROIs: (1) right IPS; (2) left IPS; (3) left PTr; (4) left dPOp; (5) right superior motor cortex; (6) right ventral motor cortex; (7) right supramarginal sulcus; (8) left medial SMA; (9) right marginal sulcus; (10) left dorsal motor cortex; (11) right STS; how ROIs defined: regions that were activated for control > aphasia (ROIs 1-4) or aphasia > control (ROIs 5-11)	↑ R ventral precentral/inferior frontal junction<br>↑ R posterior STS<br>↓ L IFG pars opercularis<br>notes: the L IFG pars opercularis and the R posterior STS also contributed to predicting PNT scores even when lesion load on critical areas for picture naming, and several other variables, were included in multiple regression models
Skipper-Kallal et al. (2017a): ROI 2	Picture naming (silently name, correct trials) vs rest	CAC<br>Aphasia vs control	YCT	UNR	ROI<br>Func<br>One	Number of ROIs: 1; ROI: L anterior temporal; how ROI defined: activity for covert naming correlated with naming ability in patients, after controlling for lesion and demographic factors	None
Skipper-Kallal et al. (2017a): ROI 3	Picture naming (produce the name, correct trials) vs rest	CAC<br>Aphasia vs control	YCT	UNR	ROI<br>Func<br>NC	Number of ROIs: 3; ROIs: (1) L frontal pole; (2) R postcentral gyrus; (3) R STS; how ROIs defined: activity for overt naming correlated with naming ability in patients, after controlling for lesion and demographic factors	↑ R somato-motor<br>↑ R posterior STS
Skipper-Kallal et al.	Picture naming (produce the	CC<br>Aphasia<br>Covariate: lesion	YCT	UNR	Cplx	SVR-LSM was used to identify regions of damage associated with activation of R pSTS ROI (defined based on SPM	Other:<br>Damage to the L IFG pars

(2017a): Cplx 1	name, correct trials) vs rest	patterns identified with SVR-LSM				analysis 2). The results were thresholded at voxelwise p < .01 (CDT), cluster extent > 500 voxels.	opercularis was associated with more activity in the R pSTS. Damage to the L pSTS was associated with less activity in the R pSTS.
Skipper-Kallal et al. (2017a): Cplx 2	Picture naming (produce the name, correct trials) vs rest	CC<br>Aphasia without IFG POp damage (n = 26)<br>Covariate: lesion patterns identified with SVR-LSM	YCT	UNR	Cplx	SVR-LSM was used to identify regions of damage associated with activation of L IFG pars opercularis ROI (defined based on SPM analysis 2). The results were thresholded at voxelwise p < .01 (CDT), cluster extent > 500 voxels.	Other:<br>Damage to the L pSTG, L pSTS, and white matter underlying the L precuneus was associated with more activity in the L IFG pars opercularis. There were no regions associated with less activity.
Skipper-Kallal et al. (2017b): Vox 1	Picture naming (prepare to name, correct trials) vs rest	CAC<br>Aphasia vs control	YCT	UNR	Vox C-	Behavioral data notes: covert phase but accuracy derived from overt phase; search volume: whole brain; software: FSL 5.0.6; voxelwise p: .01; cluster extent cutoff: based on GRFT	↑ L cerebellum<br>↑ L thalamus<br>↑ L basal ganglia<br>↑ R IFG pars opercularis<br>↑ R insula<br>↑ R cerebellum<br>↑ R basal ganglia<br>↓ L dorsolateral prefrontal cortex<br>↓ L orbitofrontal<br>↓ L intraparietal sulcus<br>↓ L anterior cingulate<br>↓ R dorsolateral prefrontal cortex<br>notes: based on Table 2
Skipper-Kallal et al. (2017b): Vox 2	Picture naming (produce the name, correct trials) vs rest	CAC<br>Aphasia vs control	YCT	UNR	Vox C-	Search volume: whole brain; software: FSL 5.0.6; voxelwise p: .01; cluster extent cutoff: based on GRFT	↑ L somato-motor<br>↑ L intraparietal sulcus<br>↑ L anterior cingulate<br>↑ R insula<br>↑ R dorsal precentral<br>↑ R somato-motor<br>↑ R supramarginal gyrus<br>↑ R posterior MTG<br>↑ R Heschl's gyrus<br>↓ L ventral precentral/inferior frontal junction<br>↓ L somato-motor<br>↓ L posterior STG/STS/MTG<br>↓ L mid temporal<br>↓ L anterior

							temporal<br>↓ L cerebellum<br>↓ L thalamus<br>↓ L hippocampus/MTL<br>notes: based on Table 3
Skipper-Kallal et al. (2017b): Vox 3	Picture naming (prepare to name, correct trials) vs rest	CC<br>Aphasia<br>Covariate: lesion volume	YCT	UNR	Vox<br>C-	Behavioral data notes: covert phase but accuracy derived from overt phase; search volume: whole brain; software: FSL 5.0.6; voxelwise p: .01; cluster extent cutoff: based on GRFT	↑ L ventral precentral/inferior frontal junction<br>↑ L intraparietal sulcus<br>↑ L superior parietal<br>↑ L occipital<br>↑ L basal ganglia<br>↑ R IFG<br>↑ R insula<br>↑ R ventral precentral/inferior frontal junction<br>↑ R SMA/medial prefrontal<br>↑ R somato-motor<br>↑ R intraparietal sulcus<br>↑ R occipital<br>↑ R cerebellum<br>↑ R brainstem<br>↑ R basal ganglia<br>notes: based on Table 4, except for R frontal activations which are missing from the table, and were added based on the figure
Skipper-Kallal et al. (2017b): Vox 4	Picture naming (produce the name, correct trials) vs rest	CC<br>Aphasia<br>Covariate: lesion volume	YCT	UNR	Vox<br>C-	Search volume: whole brain; software: FSL 5.0.6; voxelwise p: .01; cluster extent cutoff: based on GRFT	↑ L somato-motor<br>↑ L precuneus<br>↑ L occipital<br>↑ L cerebellum<br>↑ R IFG pars triangularis<br>↑ R insula<br>↑ R ventral precentral/inferior frontal junction<br>↑ R SMA/medial prefrontal<br>↑ R posterior STG/STS/MTG<br>↑ R mid temporal<br>↑ R occipital<br>↑ R cerebellum<br>↑ R basal ganglia<br>↑ R hippocampus/MTL<br>notes: based on Table 4, except for bilateral occipital activations which

							are missing from the table, and were added based on the figure
Skipper-Kallal et al. (2017b): Vox 5	Picture naming (prepare to name, correct trials) vs rest	CAA<br>Aphasia with IPS damage (n not stated) vs without IPS damage (n not stated)	YCT	UNR	Vox C-	Behavioral data notes: covert phase but accuracy derived from overt phase; search volume: whole brain; software: FSL 5.0.6; voxelwise p: .01; cluster extent cutoff: based on GRFT; lesion volume covariate	None
Skipper-Kallal et al. (2017b): Vox 6	Picture naming (prepare to name, correct trials) vs rest	CAA<br>Aphasia with insula damage (n = 18) vs without insula damage (n = 21)	YCT	UNR	Vox C-	Behavioral data notes: covert phase but accuracy derived from overt phase; search volume: whole brain; software: FSL 5.0.6; voxelwise p: .01; cluster extent cutoff: based on GRFT; lesion volume covariate	↓ R IFG pars triangularis<br>↓ R dorsolateral prefrontal cortex
Skipper-Kallal et al. (2017b): Vox 7	Picture naming (prepare to name, correct trials) vs rest	CAA<br>Aphasia with IFG POp damage (n = 16) vs without IFG POp damage (n = 23)	YCT	UNR	Vox C-	Behavioral data notes: covert phase but accuracy derived from overt phase; search volume: whole brain; software: FSL 5.0.6; voxelwise p: .01; cluster extent cutoff: based on GRFT; lesion volume covariate	↓ R IFG pars triangularis<br>↓ R dorsolateral prefrontal cortex
Skipper-Kallal et al. (2017b): Vox 8	Picture naming (produce the name, correct trials) vs rest	CAA<br>Aphasia with motor cortex damage (n = 24) vs without motor cortex damage (n = 15)	YCT	UNR	Vox C-	Search volume: whole brain; software: FSL 5.0.6; voxelwise p: .01; cluster extent cutoff: based on GRFT; lesion volume covariate	None
Skipper-Kallal et al. (2017b): Vox 9	Picture naming (produce the name, correct trials) vs rest	CAA<br>Aphasia with STS damage (n not stated) vs without STS damage (n not stated)	YCT	UNR	Vox C-	Search volume: whole brain; software: FSL 5.0.6; voxelwise p: .01; cluster extent cutoff: based on GRFT; lesion volume covariate	None
Skipper-Kallal et al. (2017b): ROI 1	Picture naming (prepare to name, correct trials) vs rest	CC<br>Aphasia with IFG POp damage (n = 16)<br>Covariate: PNT	YCT	UNR	ROI Func One	Behavioral data notes: covert phase but accuracy derived from overt phase; number of ROIs: 1; ROI: R DLPFC; how ROI defined: peak location for decreased activation for patients with left insula and left POp lesions compared to patients without said damage; lesion volume covariate	None
Skipper-Kallal et al. (2017b): ROI 2	Picture naming (prepare to name, correct trials) vs rest	CC<br>Aphasia without IFG POp damage (n = 23)<br>Covariate: PNT	YCT	UNR	ROI Func One	Behavioral data notes: covert phase but accuracy derived from overt phase; number of ROIs: 1; ROI: R DLPFC; how ROI defined: peak location for decreased activation for patients with left insula and left POp lesions compared to patients without said damage; lesion volume covariate	None
Skipper-Kallal et al. (2017b): ROI 3	Picture naming (prepare to name, correct trials) vs rest	CC<br>Aphasia with insula damage (n = 18)<br>Covariate: PNT	YCT	UNR	ROI Func One	Behavioral data notes: covert phase but accuracy derived from overt phase; number of ROIs: 1; ROI: R DLPFC; how ROI defined: peak location for decreased activation for patients with left insula and left POp lesions compared to patients without said damage; lesion volume covariate	None
Skipper-Kallal et al. (2017b): ROI 4	Picture naming (prepare to name, correct trials) vs rest	CC<br>Aphasia without insula damage (n =	YCT	UNR	ROI Func One	Behavioral data notes: covert phase but accuracy derived from overt phase; number of ROIs: 1; ROI: R DLPFC; how ROI defined: peak location	None

		21)<br>Covariate: PNT				for decreased activation for patients with left insula and left POp lesions compared to patients without said damage; lesion volume covariate	
Skipper-Kallal et al. (2017b): ROI 5	Picture naming (prepare to name, correct trials) vs rest	CAA<br>Aphasia with IPS damage (n not stated) vs without IPS damage (n not stated)	YCT	UNR	ROI<br>Func<br>NC	Behavioral data notes: covert phase but accuracy derived from overt phase; number of ROIs: 5; ROIs: (1) L IPS; (2) L insula; (3) L IFG pars opercularis; (4) R IPS; (5) R insula; how ROIs defined: 5 mm spheres around control peaks; lesion volume covariate	None
Skipper-Kallal et al. (2017b): ROI 6	Picture naming (prepare to name, correct trials) vs rest	CAA<br>Aphasia with insula damage (n = 18) vs without insula damage (n = 21)	YCT	UNR	ROI<br>Func<br>NC	Behavioral data notes: covert phase but accuracy derived from overt phase; number of ROIs: 5; ROIs: (1) L IPS; (2) L insula; (3) L IFG pars opercularis; (4) R IPS; (5) R insula; how ROIs defined: 5 mm spheres around control peaks; lesion volume covariate	None
Skipper-Kallal et al. (2017b): ROI 7	Picture naming (prepare to name, correct trials) vs rest	CAA<br>Aphasia with IFG POp damage (n = 16) vs without IFG POp damage (n = 23)	YCT	UNR	ROI<br>Func<br>NC	Behavioral data notes: covert phase but accuracy derived from overt phase; number of ROIs: 5; ROIs: (1) L IPS; (2) L insula; (3) L IFG pars opercularis; (4) R IPS; (5) R insula; how ROIs defined: 5 mm spheres around control peaks; lesion volume covariate	None
Skipper-Kallal et al. (2017b): ROI 8	Picture naming (produce the name, correct trials) vs rest	CAA<br>Aphasia with motor cortex damage (n = 24) vs without motor cortex damage (n = 15)	YCT	UNR	ROI<br>Func<br>NC	Number of ROIs: 4; ROIs: (1) L motor; (2) L pSTS; (3) R motor; (4) R pSTS; how ROIs defined: 5 mm spheres around control peaks; lesion volume covariate	↑ R somato-motor
Skipper-Kallal et al. (2017b): ROI 9	Picture naming (produce the name, correct trials) vs rest	CAA<br>Aphasia with STS damage (n not stated) vs without STS damage (n not stated)	YCT	UNR	ROI<br>Func<br>NC	Number of ROIs: 4; ROIs: (1) L motor; (2) L pSTS; (3) R motor; (4) R pSTS; how ROIs defined: 5 mm spheres around control peaks; lesion volume covariate	↓ R somato-motor
Skipper-Kallal et al. (2017b): ROI 10	Picture naming (produce the name, correct trials) vs rest	CC<br>Aphasia without motor cortex damage (n = 15)<br>Covariate: PNT	YCT	UNR	ROI<br>Func<br>One	Number of ROIs: 1; ROI: R motor; how ROI defined: 5 mm sphere around control peak; lesion volume covariate	None
Skipper-Kallal et al. (2017b): ROI 11	Picture naming (produce the name, correct trials) vs rest	CC<br>Aphasia with motor cortex damage (n = 24)<br>Covariate: PNT	YCT	UNR	ROI<br>Func<br>One	Number of ROIs: 1; ROI: R motor; how ROI defined: 5 mm sphere around control peak; lesion volume covariate	↑ R somato-motor
Dietz et al. (2018): ROI 1	Verb generation (overt) vs noun repetition	CAA<br>Aphasia with AAC treatment (n = 6) T2 vs usual care T2 (n = 6)<br>Somewhat valid (marginal treatment effect)	UNR	UNR	ROI<br>LI<br>One	Number of ROIs: 1; ROI: frontal LI; temporal LI calculated but not reported	None
Dietz et al. (2018): ROI 2	Verb generation (overt) vs noun repetition	LC<br>Aphasia (both groups) T2 vs T1<br>Covariate: Δ WAB AQ<br>Somewhat valid (gain in AQ not tested for significance)	UNR	UNR	ROI<br>LI<br>One	Number of ROIs: 1; ROI: frontal LI; temporal LI calculated but not reported	↑ LI (frontal)

Hallam et al. (2018): ROI 1	Listening to high or low ambiguity sentences vs listening to spectrally rotated speech	CAC<br>Aphasia vs control	NANB	NANT	ROI<br>Func<br>NC	Number of ROIs: 2; ROIs: (1) L vATL; (2) L pMTG; how ROIs defined: functional coordinates in literature; ANOVA revealed main effect of group (patient vs control), confirmed in follow-up tests for each ROI	↑ L posterior MTG<br>↑ L anterior temporal
Hallam et al. (2018): ROI 2	Listening to high ambiguity sentences vs listening to low ambiguity sentences	CAC<br>Aphasia vs control	NANB	NANT	ROI<br>Func<br>NC	Number of ROIs: 2; ROIs: (1) L vATL; (2) L pMTG; how ROIs defined: functional coordinates in literature; no interaction of group by condition	None
Hallam et al. (2018): Cplx 1	Listening to high ambiguity sentences vs listening to low ambiguity sentences	CAC<br>Aphasia (subset with resting state data, n = 10) vs control (subset with resting state data, n = 10)	NANB	NANT	Cplx	A whole brain analysis was carried out to identify regions where the groups differed in the extent to which the strength of functional connectivity at rest from L pMTG was associated with the difference in signal between the high ambiguity and low ambiguity conditions in the same ROI. Thresholding is not described and cluster extent is not reported.	Other:<br>There was a functional activation by group interaction in the L aSTG. For controls, there was a positive association between L pMTG activity and functional connectivity to aSTG, while for the patients, there was a negative association.
Hallam et al. (2018): Cplx 2	Listening to high ambiguity sentences vs listening to low ambiguity sentences	CAC<br>Aphasia (subset with resting state data, n = 10) vs control (subset with resting state data, n = 10)	NANB	NANT	Cplx	A whole brain analysis was carried out to identify regions where the groups differed in the extent to which the strength of functional connectivity at rest from L pMTG was associated with the difference in signal between the high ambiguity and low ambiguity conditions in the same ROI. Thresholding is not described.	None<br>notes: no interaction is reported; both groups showed a correlation between L vATL activity and functional connectivity to a ventral IFG region
Nenert et al. (2018): Vox 1	Semantic decision vs tone decision	CAC<br>Aphasia T1 vs control	AM	UNR	Vox<br>VP	Behavioral data notes: patients less accurate than controls on both tasks, but more so on the tone decision task; search volume: whole brain; software: SPM12/SnPM13; voxelwise p: FWE p < .05	↑ L Heschl's gyrus
Nenert et al. (2018): Vox 2	Semantic decision vs tone decision	CAC<br>Aphasia T2 vs control	AM	UNR	Vox<br>VP	Behavioral data notes: patients less accurate than controls on both tasks, but more so on the tone decision task; search volume: whole brain; software: SPM12/SnPM13; voxelwise p: FWE p < .05	None
Nenert et al. (2018): Vox 3	Semantic decision vs tone decision	CAC<br>Aphasia T3 vs control	AM	UNR	Vox<br>VP	Behavioral data notes: patients less accurate than controls on both tasks, but more so on the tone decision task; search volume: whole brain; software: SPM12/SnPM13; voxelwise p: FWE p < .05	None
Nenert et al. (2018): Vox 4	Semantic decision vs tone decision	CAC<br>Aphasia T4 vs control	AM	UNR	Vox<br>VP	Behavioral data notes: patients less accurate than controls on both tasks, but more so on the tone decision task;	None

						search volume: whole brain; software: SPM12/SnPM13; voxelwise p: FWE p < .05	
Nenert et al. (2018): Vox 5	Semantic decision vs tone decision	CAC<br>Aphasia T5 vs control	AM	UNR	Vox VP	Behavioral data notes: patients less accurate than controls on both tasks, but more so on the tone decision task; search volume: whole brain; software: SPM12/SnPM13; voxelwise p: FWE p < .05	None
Nenert et al. (2018): Vox 6	Verb generation vs finger tapping	CAC<br>Aphasia T1 vs control	UNR	UNR	Vox VP	Search volume: whole brain; software: SPM12/SnPM13; voxelwise p: FWE p < .05	None
Nenert et al. (2018): Vox 7	Verb generation vs finger tapping	CAC<br>Aphasia T2 vs control	UNR	UNR	Vox VP	Search volume: whole brain; software: SPM12/SnPM13; voxelwise p: FWE p < .05	None
Nenert et al. (2018): Vox 8	Verb generation vs finger tapping	CAC<br>Aphasia T3 vs control	UNR	UNR	Vox VP	Search volume: whole brain; software: SPM12/SnPM13; voxelwise p: FWE p < .05	None
Nenert et al. (2018): Vox 9	Verb generation vs finger tapping	CAC<br>Aphasia T4 vs control	UNR	UNR	Vox VP	Search volume: whole brain; software: SPM12/SnPM13; voxelwise p: FWE p < .05	None
Nenert et al. (2018): Vox 10	Verb generation vs finger tapping	CAC<br>Aphasia T5 vs control	UNR	UNR	Vox VP	Search volume: whole brain; software: SPM12/SnPM13; voxelwise p: FWE p < .05	None
Nenert et al. (2018): Vox 11	Semantic decision vs tone decision	CC<br>Aphasia T1<br>Covariate: semantic decision accuracy	C	UNR	Vox VP	Search volume: whole brain; software: SPM12/SnPM13; voxelwise p: FWE p < .05	↑ L anterior temporal<br>notes: unclear why this type of analysis was run only for semantic task, and only at T1
Nenert et al. (2018): Vox 12	Semantic decision vs tone decision	LC<br>Aphasia T4 vs aphasia T1<br>Covariate: Δ BNT	UNR	UNR	Vox VP	Search volume: whole brain; software: SPM12/SnPM13; voxelwise p: FWE p < .05	None
Nenert et al. (2018): Vox 13	Semantic decision vs tone decision	LC<br>Aphasia T4 vs aphasia T1<br>Covariate: Δ semantic fluency	UNR	UNR	Vox VP	Search volume: whole brain; software: SPM12/SnPM13; voxelwise p: FWE p < .05	None
Nenert et al. (2018): Vox 14	Semantic decision vs tone decision	LC<br>Aphasia T4 vs aphasia T1<br>Covariate: Δ PPVT	UNR	UNR	Vox VP	Search volume: whole brain; software: SPM12/SnPM13; voxelwise p: FWE p < .05	None
Nenert et al. (2018): Vox 15	Semantic decision vs tone decision	LC<br>Aphasia T4 vs aphasia T1<br>Covariate: Δ phonemic fluency	UNR	UNR	Vox VP	Search volume: whole brain; software: SPM12/SnPM13; voxelwise p: FWE p < .05	None
Nenert et al. (2018): Vox 16	Semantic decision vs tone decision	LC<br>Aphasia T4 vs aphasia T1<br>Covariate: Δ BDAE complex ideation subtest	UNR	UNR	Vox VP	Search volume: whole brain; software: SPM12/SnPM13; voxelwise p: FWE p < .05	None
Nenert et al. (2018): Vox 17	Verb generation vs finger tapping	LC<br>Aphasia T4 vs aphasia T1<br>Covariate: Δ BNT	UNR	UNR	Vox VP	Search volume: whole brain; software: SPM12/SnPM13; voxelwise p: FWE p < .05	None

Nenert et al. (2018): Vox 18	Verb generation vs finger tapping	LC<br>Aphasia T4 vs aphasia T1<br>Covariate: Δ semantic fluency	UNR	UNR	Vox<br>VP	Search volume: whole brain; software: SPM12/SnPM13; voxelwise p: FWE p < .05	↑ L dorsolateral prefrontal cortex<br>↑ L SMA/medial prefrontal<br>↑ R somato-motor<br>↑ R anterior temporal
Nenert et al. (2018): Vox 19	Verb generation vs finger tapping	LC<br>Aphasia T4 vs aphasia T1<br>Covariate: Δ PPVT	UNR	UNR	Vox<br>VP	Search volume: whole brain; software: SPM12/SnPM13; voxelwise p: FWE p < .05	None
Nenert et al. (2018): Vox 20	Verb generation vs finger tapping	LC<br>Aphasia T4 vs aphasia T1<br>Covariate: Δ phonemic fluency	UNR	UNR	Vox<br>VP	Search volume: whole brain; software: SPM12/SnPM13; voxelwise p: FWE p < .05	↑ L cerebellum
Nenert et al. (2018): Vox 21	Verb generation vs finger tapping	LC<br>Aphasia T4 vs aphasia T1<br>Covariate: Δ BDAE complex ideation subtest	UNR	UNR	Vox<br>VP	Search volume: whole brain; software: SPM12/SnPM13; voxelwise p: FWE p < .05	None
Nenert et al. (2018): ROI 1	Semantic decision vs tone decision	LA<br>Aphasia (comparisons between all pairs of time points)	AS	UNR	ROI<br>LI<br>NC	Number of ROIs: 4; ROIs: (1) frontal LI; (2) temporo-parietal LI; (3) language network LI; (4) cerebellar LI	None
Nenert et al. (2018): ROI 2	Verb generation vs finger tapping	LA<br>Aphasia (comparisons between all pairs of time points)	UNR	UNR	ROI<br>LI<br>NC	Number of ROIs: 4; ROIs: (1) frontal LI; (2) temporo-parietal LI; (3) language network LI; (4) cerebellar LI	None
Nenert et al. (2018): ROI 3	Semantic decision vs tone decision	CAC<br>Aphasia T1 vs control	AM	UNR	ROI<br>LI<br>NC	Behavioral data notes: patients less accurate than controls on both tasks, but more so on the tone decision task; number of ROIs: 4; ROIs: (1) frontal LI; (2) temporo-parietal LI; (3) language network LI; (4) cerebellar LI	None
Nenert et al. (2018): ROI 4	Semantic decision vs tone decision	CAC<br>Aphasia T2 vs control	AM	UNR	ROI<br>LI<br>NC	Behavioral data notes: patients less accurate than controls on both tasks, but more so on the tone decision task; number of ROIs: 4; ROIs: (1) frontal LI; (2) temporo-parietal LI; (3) language network LI; (4) cerebellar LI	None
Nenert et al. (2018): ROI 5	Semantic decision vs tone decision	CAC<br>Aphasia T3 vs control	AM	UNR	ROI<br>LI<br>NC	Behavioral data notes: patients less accurate than controls on both tasks, but more so on the tone decision task; number of ROIs: 4; ROIs: (1) frontal LI; (2) temporo-parietal LI; (3) language network LI; (4) cerebellar LI	None
Nenert et al. (2018): ROI 6	Semantic decision vs tone decision	CAC<br>Aphasia T4 vs control	AM	UNR	ROI<br>LI<br>NC	Behavioral data notes: patients less accurate than controls on both tasks, but more so on the tone decision task; number of ROIs: 4; ROIs: (1) frontal LI; (2) temporo-parietal LI; (3) language network LI; (4) cerebellar LI	None
Nenert et al. (2018): ROI 7	Semantic decision vs tone decision	CAC<br>Aphasia T5 vs control	AM	UNR	ROI<br>LI<br>NC	Behavioral data notes: patients less accurate than controls on both tasks, but more so on the tone decision task; number of ROIs: 4; ROIs: (1) frontal LI; (2) temporo-parietal LI; (3) language network LI; (4) cerebellar LI	None
Nenert et	Verb generation vs	CAC	UNR	UNR	ROI	Number of ROIs: 4; ROIs: (1) frontal LI;	None

al. (2018): ROI 8	finger tapping	Aphasia T1 vs control			LI NC	(2) temporo-parietal LI; (3) language network LI; (4) cerebellar LI	
Nenert et al. (2018): ROI 9	Verb generation vs finger tapping	CAC Aphasia T2 vs control	UNR	UNR	ROI LI NC	Number of ROIs: 4; ROIs: (1) frontal LI; (2) temporo-parietal LI; (3) language network LI; (4) cerebellar LI	↓ LI (language network)<br>↓ LI (frontal)
Nenert et al. (2018): ROI 10	Verb generation vs finger tapping	CAC Aphasia T3 vs control	UNR	UNR	ROI LI NC	Number of ROIs: 4; ROIs: (1) frontal LI; (2) temporo-parietal LI; (3) language network LI; (4) cerebellar LI	↓ LI (language network)<br>↓ LI (frontal)
Nenert et al. (2018): ROI 11	Verb generation vs finger tapping	CAC Aphasia T4 vs control	UNR	UNR	ROI LI NC	Number of ROIs: 4; ROIs: (1) frontal LI; (2) temporo-parietal LI; (3) language network LI; (4) cerebellar LI	None
Nenert et al. (2018): ROI 12	Verb generation vs finger tapping	CAC Aphasia T5 vs control	UNR	UNR	ROI LI NC	Number of ROIs: 4; ROIs: (1) frontal LI; (2) temporo-parietal LI; (3) language network LI; (4) cerebellar LI	None
Nenert et al. (2018): Cplx 1	Semantic decision vs tone decision	LA Aphasia (comparisons between all pairs of time points)	AS	UNR	Cplx	PPI analyses were carried out to investigate potential changes over time in how connectivity from L and R IFG was modulated by the semantic decision task. The resultant SPM was thresholded at FWE p < .05 using permutation testing implemented in SnPM 13.	None
Nenert et al. (2018): Cplx 2	Verb generation vs finger tapping	LA Aphasia (comparisons between all pairs of time points)	UNR	UNR	Cplx	PPI analyses were carried out to investigate potential changes over time in how connectivity from L and R IFG was modulated by the verb generation task. The resultant SPM was thresholded at FWE p < .05 using permutation testing implemented in SnPM 13.	None
Pillay et al. (2018): Vox 1	Reading nouns aloud (correct trials) vs reading nouns aloud (incorrect trials)	CB Aphasia	NBD	Y	Vox CCS	Search volume: whole brain; software: AFNI; voxelwise p: .01; cluster extent cutoff: 1.609 cc; regarding correction for multiple comparisons, addition of monoexponential function reduces but does not eliminate inflation of p values (Cox et al., 2017)	↑ L angular gyrus<br>↓ L ventral precentral/inferior frontal junction<br>↓ L SMA/medial prefrontal<br>↓ R insula<br>↓ R ventral precentral/inferior frontal junction<br>↓ R SMA/medial prefrontal<br>notes: positive region (L AG) was part of the semantic network, while many negative regions were positively modulated by reaction time in the aphasia group
Szaflarski et al. (2018): Vox 1	Semantic decision vs tone decision	LA Aphasia T2 vs T1	UNR	UNR	Vox CCTB	Search volume: whole brain; software: SPM12; voxelwise p: .05; cluster extent cutoff: 0.928 cc	↑ L supramarginal gyrus<br>↑ L intraparietal sulcus<br>↑ L precuneus<br>↑ L posterior STG<br>↑ L Heschl's gyrus<br>↑ L mid temporal<br>↑ L anterior

							temporal<br>↑ R supramarginal gyrus<br>↑ R superior parietal<br>↑ R precuneus<br>↑ R mid temporal<br>↑ R anterior cingulate<br>↓ L IFG pars opercularis<br>↓ L dorsolateral prefrontal cortex<br>↓ L ventral precentral/inferior frontal junction<br>↓ L dorsal precentral<br>↓ L SMA/medial prefrontal<br>↓ L somato-motor<br>↓ L superior parietal<br>↓ L occipital
Szaflarski et al. (2018): Vox 2	Semantic decision vs tone decision	LA<br>Aphasia T3 vs T2	UNR	UNR	Vox<br>CCTB	Search volume: whole brain; software: SPM12; voxelwise p: .05; cluster extent cutoff: 0.928 cc	↑ L dorsolateral prefrontal cortex<br>↑ L angular gyrus<br>↑ L precuneus<br>↑ L posterior STS<br>↓ L SMA/medial prefrontal<br>↓ L anterior temporal<br>↓ L anterior cingulate<br>↓ R IFG<br>↓ R dorsolateral prefrontal cortex<br>↓ R ventral precentral/inferior frontal junction<br>↓ R SMA/medial prefrontal<br>↓ R somato-motor<br>↓ R precuneus<br>↓ R posterior STG/STS/MTG<br>↓ R anterior temporal
Szaflarski et al. (2018): Vox 3	Semantic decision vs tone decision	LA<br>Aphasia T3 vs T1	UNR	UNR	Vox<br>CCTB	Search volume: whole brain; software: SPM12; voxelwise p: .05; cluster extent cutoff: 0.928 cc	↑ L supramarginal gyrus<br>↑ L angular gyrus<br>↑ L precuneus<br>↑ L posterior STG<br>↑ L mid temporal<br>↑ L anterior temporal<br>↑ L posterior cingulate<br>↓ L somato-motor<br>↓ R dorsolateral prefrontal cortex

Szaflarski et al. (2018): Vox 4	Semantic decision vs tone decision	LC<br>Aphasia T3 vs aphasia T2<br>Covariate: Δ WAB AQ	UNR	UNR	Vox CCTB	Search volume: whole brain; software: SPM12; voxelwise p: .05; cluster extent cutoff: 0.928 cc; inclusive mask of voxels that differed between T2 and T3	↓ L inferior parietal lobule
Szaflarski et al. (2018): Vox 5	Semantic decision vs tone decision	LC<br>Aphasia T3 vs aphasia T1<br>Covariate: Δ BNT	UNR	UNR	Vox CCTB	Search volume: whole brain; software: SPM12; voxelwise p: .05; cluster extent cutoff: 0.928 cc; inclusive mask of voxels that differed between T1 and T3	↓ R IFG
van de Sandt-Koenderman et al. (2018): ROI 1	Listening to narrative speech vs listening to reversed speech	CC<br>Aphasia T1<br>Covariate: lesion volume	NANB	NANT	ROI LI One	Number of ROIs: 1; ROI: language network LI; how ROI defined: activations that were "not clearly related to known language areas" were excluded, but the basis for this determination is not clear	None
van de Sandt-Koenderman et al. (2018): ROI 2	Listening to narrative speech vs listening to reversed speech	LC<br>Aphasia T2 vs T1<br>Covariate: lesion volume	NANB	NANT	ROI LI One	Number of ROIs: 1; ROI: language network LI; how ROI defined: activations that were "not clearly related to known language areas" were excluded, but the basis for this determination is not clear	None
van de Sandt-Koenderman et al. (2018): ROI 3	Listening to narrative speech vs listening to reversed speech	LC<br>Aphasia T2 vs T1<br>Covariate: Δ AAT repetition score	NANB	NANT	ROI LI One	Number of ROIs: 1; ROI: language network LI; how ROI defined: activations that were "not clearly related to known language areas" were excluded, but the basis for this determination is not clear	None
van de Sandt-Koenderman et al. (2018): ROI 4	Listening to narrative speech vs listening to reversed speech	LC<br>Aphasia T2 vs T1<br>Covariate: Δ ANELT	NANB	NANT	ROI LI One	Number of ROIs: 1; ROI: language network LI; how ROI defined: activations that were "not clearly related to known language areas" were excluded, but the basis for this determination is not clear	None
van Oers et al. (2018): ROI 1	Written word-picture matching vs visual decision	CC<br>Aphasia (subset who returned for follow-up) T1 (n = 10)<br>Covariate: subsequent outcome (T4) overall language measure (average of AAT measures)	UNR	UNR	ROI Func FDR	Number of ROIs: 12; ROIs: (1) bilateral dorsal anterior cingulate; (2) L angular gyrus; (3) L IFG pars opercularis and triangularis; (4) L thalamus; (5) L MFG; (6) L posterior ITG; (7) R angular gyrus; (8) R IFG pars triangularis; (9) R thalamus; (10) R posterior ITG; (11) R IFG pars opercularis and triangularis; (12) R MFG; how ROIs defined: control activations and their homotopic counterparts in the R hemisphere; activation measured as count of voxels activated at p < 0.001, uncorrected	↑ L posterior inferior temporal gyrus/fusiform gyrus<br>notes: activation predicted later outcome even when initial language performance was included in the model
van Oers et al. (2018): ROI 2	Written word-picture matching vs visual decision	CC<br>Aphasia (all time points)<br>Covariate: overall language measure (average of AAT measures) all time points	UNR	UNR	ROI Func FDR	Number of ROIs: 12; ROIs: (1) bilateral dorsal anterior cingulate; (2) L angular gyrus; (3) L IFG pars opercularis and triangularis; (4) L thalamus; (5) L MFG; (6) L posterior ITG; (7) R angular gyrus; (8) R IFG pars triangularis; (9) R thalamus; (10) R posterior ITG; (11) R IFG pars opercularis and triangularis; (12) R MFG; how ROIs defined: control activations and their homotopic counterparts in the R hemisphere; activation measured as count of voxels activated at p < 0.001,	↑ L posterior inferior temporal gyrus/fusiform gyrus

						uncorrected; mixed model; minimal detail provided	
van Oers et al. (2018): ROI 3	Written word-picture matching vs visual decision	CC<br>Aphasia (all time points)<br>Covariate: average of AAT comprehension score and BNT, all time points	UNR	UNR	ROI Func FDR	Number of ROIs: 12; ROIs: (1) bilateral dorsal anterior cingulate; (2) L angular gyrus; (3) L IFG pars opercularis and triangularis; (4) L thalamus; (5) L MFG; (6) L posterior ITG; (7) R angular gyrus; (8) R IFG pars triangularis; (9) R thalamus; (10) R posterior ITG; (11) R IFG pars opercularis and triangularis; (12) R MFG; how ROIs defined: control activations and their homotopic counterparts in the R hemisphere; activation measured as count of voxels activated at p < 0.001, uncorrected; mixed model; minimal detail provided	↓ R IFG pars opercularis<br>↓ R IFG pars triangularis
van Oers et al. (2018): ROI 4	Written word-picture matching vs visual decision	CC<br>Aphasia (all time points)<br>Covariate: picture-word matching accuracy, all time points	C	UNR	ROI Func FDR	Number of ROIs: 12; ROIs: (1) bilateral dorsal anterior cingulate; (2) L angular gyrus; (3) L IFG pars opercularis and triangularis; (4) L thalamus; (5) L MFG; (6) L posterior ITG; (7) R angular gyrus; (8) R IFG pars triangularis; (9) R thalamus; (10) R posterior ITG; (11) R IFG pars opercularis and triangularis; (12) R MFG; how ROIs defined: control activations and their homotopic counterparts in the R hemisphere; activation measured as count of voxels activated at p < 0.001, uncorrected; mixed model; minimal detail provided	↑ R posterior inferior temporal gyrus/fusiform gyrus
van Oers et al. (2018): ROI 5	Written word-picture matching vs visual decision	LA<br>Aphasia: linear effect of time	UNR	UNR	ROI Func FDR	Number of ROIs: 12; ROIs: (1) bilateral dorsal anterior cingulate; (2) L angular gyrus; (3) L IFG pars opercularis and triangularis; (4) L thalamus; (5) L MFG; (6) L posterior ITG; (7) R angular gyrus; (8) R IFG pars triangularis; (9) R thalamus; (10) R posterior ITG; (11) R IFG pars opercularis and triangularis; (12) R MFG; how ROIs defined: control activations and their homotopic counterparts in the R hemisphere; activation measured as count of voxels activated at p < 0.001, uncorrected; mixed model; minimal detail provided	↑ L dorsolateral prefrontal cortex<br>↑ L angular gyrus<br>↑ L posterior inferior temporal gyrus/fusiform gyrus<br>↑ L anterior cingulate<br>↑ R dorsolateral prefrontal cortex<br>↑ R angular gyrus<br>↑ R anterior cingulate<br>↑ R thalamus<br>↓ L IFG pars opercularis<br>↓ L IFG pars triangularis<br>notes: similar numbers of findings are reported for controls
van Oers et al. (2018): ROI 6	Semantic decision vs visual decision	CC<br>Aphasia (subset who returned for follow-up) T1 (n = 10)<br>Covariate: subsequent outcome	UNR	UNR	ROI Func FDR	Number of ROIs: 6; ROIs: (1) L angular gyrus; (2) L IFG pars opercularis and triangularis; (3) L posterior ITG; (4) R angular gyrus; (5) R IFG pars opercularis and triangularis; (6) R posterior ITG; how ROIs defined:	None

		(T4) overall language measure (average of AAT measures)<br>Somewhat valid (not appropriate to correlate T1 imaging with T4 behavior without T1 behavior in model)				control activations and their homotopic counterparts in the R hemisphere; activation measured as count of voxels activated at p < 0.001, uncorrected	
van Oers et al. (2018): ROI 7	Semantic decision vs visual decision	CC<br>Aphasia (all time points)<br>Covariate: overall language measure (average of AAT measures) all time points	UNR	UNR	ROI Func FDR	Number of ROIs: 6; ROIs: (1) L angular gyrus; (2) L IFG pars opercularis and triangularis; (3) L posterior ITG; (4) R angular gyrus; (5) R IFG pars opercularis and triangularis; (6) R posterior ITG; how ROIs defined: control activations and their homotopic counterparts in the R hemisphere; activation measured as count of voxels activated at p < 0.001, uncorrected; mixed model; minimal detail provided	None
van Oers et al. (2018): ROI 8	Semantic decision vs visual decision	CC<br>Aphasia (all time points)<br>Covariate: average of AAT comprehension score and BNT, all time points	UNR	UNR	ROI Func FDR	Number of ROIs: 6; ROIs: (1) L angular gyrus; (2) L IFG pars opercularis and triangularis; (3) L posterior ITG; (4) R angular gyrus; (5) R IFG pars opercularis and triangularis; (6) R posterior ITG; how ROIs defined: control activations and their homotopic counterparts in the R hemisphere; activation measured as count of voxels activated at p < 0.001, uncorrected; mixed model; minimal detail provided	None
van Oers et al. (2018): ROI 9	Semantic decision vs visual decision	CC<br>Aphasia (all time points)<br>Covariate: semantic decision accuracy, all time points	C	UNR	ROI Func FDR	Number of ROIs: 6; ROIs: (1) L angular gyrus; (2) L IFG pars opercularis and triangularis; (3) L posterior ITG; (4) R angular gyrus; (5) R IFG pars opercularis and triangularis; (6) R posterior ITG; how ROIs defined: control activations and their homotopic counterparts in the R hemisphere; activation measured as count of voxels activated at p < 0.001, uncorrected; mixed model; minimal detail provided	None
van Oers et al. (2018): ROI 10	Semantic decision vs visual decision	LA<br>Aphasia: linear effect of time	UNR	UNR	ROI Func FDR	Number of ROIs: 6; ROIs: (1) L angular gyrus; (2) L IFG pars opercularis and triangularis; (3) L posterior ITG; (4) R angular gyrus; (5) R IFG pars opercularis and triangularis; (6) R posterior ITG; how ROIs defined: control activations and their homotopic counterparts in the R hemisphere; activation measured as count of voxels activated at p < 0.001, uncorrected; mixed model; minimal detail provided	↑ L posterior inferior temporal gyrus/fusiform gyrus<br>↑ R angular gyrus<br>↓ L IFG pars opercularis<br>↓ L IFG pars triangularis<br>notes: similar numbers of findings are reported for controls
Barbieri et al. (2019): Vox 1	Auditory sentence-picture verification vs listening to	LA<br>Aphasia treated (n = 13) T2 vs T1	UNR	UNR	Vox CCS	Behavioral data notes: out-of-scanner performance on passive sentences improved; software: SPM8; voxelwise	↑ L precuneus<br>↑ R ventral precentral/inferior

	reversed speech and viewing scrambled pictures					p: .001; cluster extent cutoff: 37 voxels (size not stated)	frontal junction<br>↑ R somato-motor<br>↑ R supramarginal gyrus<br>↑ R intraparietal sulcus<br>↑ R superior parietal<br>↑ R precuneus<br>notes: based on Table 7 and Figure 8
Barbieri et al. (2019): Vox 2	Auditory sentence-picture verification vs listening to reversed speech and viewing scrambled pictures	LA<br>Aphasia natural history (n = 5) T2 vs T1	UNR	UNR	Vox<br>CCS	Software: SPM8; voxelwise p: .001; cluster extent cutoff: 37 voxels (size not stated)	None
Barbieri et al. (2019): ROI 1	Auditory sentence-picture verification vs listening to reversed speech and viewing scrambled pictures	LAA<br>(Aphasia treated (n=13) T2 vs T1) vs (aphasia natural history (n=5) T2 vs T1)	UNR	UNR	ROI<br>Anat<br>NC	Number of ROIs: 4; ROIs: (1) L hemisphere sentence processing network (IFGpt, pMTG, pSTG, AG); (2) R hemisphere homotopic regions; (3) L dorsal attention network (MFG, PrCG, SPL, sLOC); (4) R dorsal attention network (same regions); how ROIs defined: sentence processing network based on Walenski et al. (2019); dorsal attention network based on Corbetta et al. (2008) and Vincent et al. (2008); ROIs were defined based on Harvard-Oxford atlas which would align imperfectly with these functional networks; dependent variable was number of active voxels (p < .001, uncorrected) divided by number of intact voxels; derivation of dependent measures from ROIs difficulty to follow, but it seems that ROIs with less than 5 voxels upregulated were excluded and deactivations were not considered, meaning that estimates of change may be biased	↑ L dorsolateral prefrontal cortex<br>↑ L ventral precentral/inferior frontal junction<br>↑ L dorsal precentral<br>↑ L angular gyrus<br>↑ L intraparietal sulcus<br>↑ L superior parietal<br>↑ R dorsolateral prefrontal cortex<br>↑ R ventral precentral/inferior frontal junction<br>↑ R dorsal precentral<br>↑ R angular gyrus<br>↑ R intraparietal sulcus<br>↑ R superior parietal<br>notes: bilateral dorsal attention network; findings were for networks as a whole; regions coded correspond to atlas ROIs
Barbieri et al. (2019): ROI 2	Auditory sentence-picture verification vs listening to reversed speech and viewing scrambled pictures	LC<br>Aphasia T2 vs T1<br>Covariate: ∆ offline comprehension composite	UNR	UNR	ROI<br>Anat<br>NC	Number of ROIs: 4; ROIs: (1) L hemisphere sentence processing network (IFGpt, pMTG, pSTG, AG); (2) R hemisphere homotopic regions; (3) L dorsal attention network (MFG, PrCG, SPL, sLOC); (4) R dorsal attention network (same regions); how ROIs defined: sentence processing network based on Walenski et al. (2019); dorsal attention network based on Corbetta et al. (2008) and Vincent et al. (2008); ROIs were defined based on Harvard-	↑ R IFG pars triangularis<br>↑ R dorsolateral prefrontal cortex<br>↑ R ventral precentral/inferior frontal junction<br>↑ R dorsal precentral<br>↑ R angular gyrus<br>↑ R intraparietal sulcus

						Oxford atlas which would align imperfectly with these functional networks; dependent variable was number of active voxels (p < .001, uncorrected) divided by number of intact voxels; derivation of dependent measures from ROIs difficulty to follow, but it seems that ROIs with less than 5 voxels upregulated were excluded and deactivations were not considered, meaning that estimates of change may be biased	↑ R superior parietal<br>↑ R posterior STG/STS/MTG<br>notes: R homotopic sentence processing network and R dorsal attention network; findings were for networks as a whole; regions coded correspond to atlas ROIs
Barbieri et al. (2019): ROI 3	Auditory sentence-picture verification vs listening to reversed speech and viewing scrambled pictures	LC<br>Aphasia participants with eye tracking data (n = 16) T2 vs T1<br>Covariate: ∆ decrease in eye tracking online thematic prediction score	UNR	UNR	ROI Anat NC	Number of ROIs: 4; ROIs: (1) L hemisphere sentence processing network (IFGpt, pMTG, pSTG, AG); (2) R hemisphere homotopic regions; (3) L dorsal attention network (MFG, PrCG, SPL, sLOC); (4) R dorsal attention network (same regions); how ROIs defined: sentence processing network based on Walenski et al. (2019); dorsal attention network based on Corbetta et al. (2008) and Vincent et al. (2008); ROIs were defined based on Harvard-Oxford atlas which would align imperfectly with these functional networks; dependent variable was number of active voxels (p < .001, uncorrected) divided by number of intact voxels; derivation of dependent measures from ROIs difficulty to follow, but it seems that ROIs with less than 5 voxels upregulated were excluded and deactivations were not considered, meaning that estimates of change may be biased	↑ R IFG pars triangularis<br>↑ R angular gyrus<br>↑ R posterior STG/STS/MTG<br>notes: R homotopic sentence processing network; findings were for networks as a whole; regions coded correspond to atlas ROIs
Barbieri et al. (2019): ROI 4	Auditory sentence-picture verification vs listening to reversed speech and viewing scrambled pictures	LC<br>Aphasia participants with eye tracking data (n = 16) T2 vs T1<br>Covariate: ∆ eye tracking online thematic integragration score	UNR	UNR	ROI Anat NC	Number of ROIs: 4; ROIs: (1) L hemisphere sentence processing network (IFGpt, pMTG, pSTG, AG); (2) R hemisphere homotopic regions; (3) L dorsal attention network (MFG, PrCG, SPL, sLOC); (4) R dorsal attention network (same regions); how ROIs defined: sentence processing network based on Walenski et al. (2019); dorsal attention network based on Corbetta et al. (2008) and Vincent et al. (2008); ROIs were defined based on Harvard-Oxford atlas which would align imperfectly with these functional networks; dependent variable was number of active voxels (p < .001, uncorrected) divided by number of intact voxels; derivation of dependent measures from ROIs difficulty to follow, but it seems that ROIs with less than 5 voxels upregulated were excluded and deactivations were not	↑ R dorsolateral prefrontal cortex<br>↑ R ventral precentral/inferior frontal junction<br>↑ R dorsal precentral<br>↑ R angular gyrus<br>↑ R intraparietal sulcus<br>↑ R superior parietal<br>notes: R dorsal attention network; findings were for networks as a whole; regions coded correspond to atlas ROIs

						considered, meaning that estimates of change may be biased	
Johnson et al. (2019): ROI 1	Picture naming (trained items) vs rest	CAC Aphasia treated T1 (n = 26) vs control	N	UNR	ROI Anat NC	Number of ROIs: 16; ROIs: (1) L IFGorb; (2) L IFGtri; (3) L IFGop; (4) L MFG; (5) L PrCG; (6) L MTG; (7) L SMG; (8) L AG; (9-16) homotopic counterparts; how ROIs defined: AAL but lesioned voxels were excluded from ROIs on an individual basis	↑ L IFG pars triangularis<br>↑ R IFG pars triangularis<br>↓ L angular gyrus<br>notes: significant interaction of ROI by group
Johnson et al. (2019): ROI 2	Picture naming (trained items) vs rest	CAC Aphasia treated T2 (n = 26) vs control	N	UNR	ROI Anat NC	Number of ROIs: 16; ROIs: (1) L IFGorb; (2) L IFGtri; (3) L IFGop; (4) L MFG; (5) L PrCG; (6) L MTG; (7) L SMG; (8) L AG; (9-16) homotopic counterparts; how ROIs defined: AAL but lesioned voxels were excluded from ROIs on an individual basis	↑ L IFG pars triangularis<br>↑ R IFG pars opercularis<br>↑ R IFG pars triangularis<br>notes: significant interaction of ROI by group; patients also showed more activity than controls across the average of all ROIs
Johnson et al. (2019): ROI 3	Picture naming (trained items) vs rest	LA Aphasia untreated (n = 10) T2 vs T1	Y	UNR	ROI Anat NC	Number of ROIs: 16; ROIs: (1) L IFGorb; (2) L IFGtri; (3) L IFGop; (4) L MFG; (5) L PrCG; (6) L MTG; (7) L SMG; (8) L AG; (9-16) homotopic counterparts; how ROIs defined: AAL but lesioned voxels were excluded from ROIs on an individual basis	None<br>notes: no main effect of time or interaction of time by ROI
Johnson et al. (2019): Cplx 1	Picture naming (trained items) vs rest	LA Aphasia treated (n = 26) T2 vs T1	N	UNR	Cplx	A linear model was constructed to examine the relationship between proportion of spared tissue in each L hemisphere ROI and changes in activation over time. The model is not described in sufficient detail.	Other:<br>There was a significant 3-way interaction of time by ROI by spared tissue, such that in some regions (AG, MFG, IFG orb, SMG), less spared tissue was associated with greater increases in activation, while in others (PrCG, IFG op, IFG tri), less spared tissue was associated with greater decreases in activation.
Kristinsson et al. (2019): Vox 1	Picture naming vs viewing abstract pictures	CAA Aphasia with typical genotype (n = 53) vs atypical genotype (n = 34)	Y	UNR	Vox VFWE	Software: SPM12	None
Purcell et al. (2019): Vox 1	Spelling probe (training items) vs rest	LA Aphasia with both timepoints (n = 20) T2 vs T1	AM	AM	Vox CCS	Behavioral data notes: see section S2, but main effects include known items also; search volume: appears to be restricted to voxels spared in all patients; software: BrainVoyager QX 2.4 or SPM12; voxelwise p: .01; cluster	↑ L posterior cingulate<br>↑ R angular gyrus<br>↑ R posterior cingulate

						extent cutoff: 49 voxels (size not stated)	
Purcell et al. (2019): ROI 1	Spelling probe (training items) vs rest	LC<br>Aphasia with both timepoints (n = 20) T2 vs T1<br>Covariate: Δ spelling accuracy on training items	UNR	UNR	ROI Func NC	Number of ROIs: 3; ROIs: (1) R AG; (2) L PCC; (3) R PCC; how ROIs defined: regions activated in SPM analysis 1	None
Purcell et al. (2019): ROI 2	Spelling probe (training items) vs rest	LC<br>Aphasia with both timepoints (n = 20) T2 vs T1<br>Covariate: Δ spelling accuracy on untrained items	UNR	UNR	ROI Func NC	Number of ROIs: 3; ROIs: (1) R AG; (2) L PCC; (3) R PCC; how ROIs defined: regions activated in SPM analysis 1	None
Purcell et al. (2019): ROI 3	Spelling probe (training items) vs rest	CC<br>Aphasia T1<br>Covariate: subsequent Δ spelling accuracy on training items (T2 vs T1)<br>Somewhat valid (T1 behavioral measure should be included in model)	UNR	UNR	ROI Func One	Number of ROIs: 1; ROI: L ventral occipitotemporal cortex; how ROI defined: the region that showed an increase in Local-Hreg from T1 to T2	None
Purcell et al. (2019): ROI 4	Spelling probe (training items) vs rest	CC<br>Aphasia with both timepoints T1 (n = 20)<br>Covariate: subsequent Δ spelling accuracy on untrained items (T2 vs T1)<br>Somewhat valid (T1 behavioral measure should be included in model)	UNR	UNR	ROI Func One	Number of ROIs: 1; ROI: L ventral occipitotemporal cortex; how ROI defined: the region that showed an increase in Local-Hreg from T1 to T2	None
Purcell et al. (2019): ROI 5	Spelling probe (training items) vs rest	LC<br>Aphasia with both timepoints (n = 20) T2 vs T1<br>Covariate: Δ spelling accuracy on training items	UNR	UNR	ROI Func One	Number of ROIs: 1; ROI: L ventral occipitotemporal cortex; how ROI defined: the region that showed an increase in Local-Hreg from T1 to T2	None
Purcell et al. (2019): ROI 6	Spelling probe (training items) vs rest	LC<br>Aphasia with both timepoints (n = 20) T2 vs T1<br>Covariate: Δ spelling accuracy on untrained items	UNR	UNR	ROI Func One	Number of ROIs: 1; ROI: L ventral occipitotemporal cortex; how ROI defined: the region that showed an increase in Local-Hreg from T1 to T2	None
Purcell et al. (2019): Cplx 1	Spelling probe (training items) vs rest	LA<br>Aphasia with both timepoints (n = 20) T2 vs T1	AM	AM	Cplx	Behavioral data notes: see section S2, where Figures S1 and S2 appear to show differences; the main effects of time were not significant for accuracy or RT, but those analyses included known items also, which had smaller effects; Local Heterogeneity Regression Analysis (Local-Hreg) was used to identify brain regions where the heterogeneity of timecourses	Other: Only in L ventral occipitotemporal cortex, there was a significant increase in Local-Hreg from T1 to T2 (p = 0.028, corrected).

						between neighboring voxels, specifically for the trained condition, increased from T1 to T2. A voxelwise threshold of $p < 0.05$ was applied, followed by cluster correction based on permutation testing. The analysis appears to have been restricted to brain regions not damaged in any patients.	
Purcell et al. (2019): Cplx 2	Spelling probe (known items) vs rest	LA<br>Aphasia with both timepoints (n = 20) T2 vs T1	Y	Y	Cplx	Behavioral data notes: see section S2, main effects were not significant and effects appear smaller for known than trained; Local Heterogeneity Regression Analysis (Local-Hreg) was used to identify brain regions where the heterogeneity of timecourses between neighboring voxels, specifically for the known condition, increased from T1 to T2. A voxelwise threshold of $p < 0.05$ was applied, followed by cluster correction based on permutation testing. The analysis appears to have been restricted to brain regions not damaged in any patients.	None
Purcell et al. (2019): Cplx 3	Spelling probe (training items) vs rest	CC<br>Aphasia T1<br>Covariate: T1 spelling accuracy on training items<br>Somewhat valid (training items were selected for individual patients, so training item accuracy is not an appropriate measure of spelling ability)	UNR	UNR	Cplx	A linear mixed effects model was used to investigate the relationship between Local-Hreg at T1 in the L ventral occipitotemporal region previously identified and T1 spelling accuracy of training items. A complex model was used in which every voxel for every patient was considered an observation, with random effects of voxel and patient, but this is not described in detail.	Other:<br>There was a significant positive relationship between T1 Local-Hreg and T1 spelling accuracy on training items.
Purcell et al. (2019): Cplx 4	Spelling probe (training items) vs rest	CC<br>Aphasia T1<br>Covariate: subsequent Δ spelling accuracy on training items (T2 vs T1)<br>Somewhat valid (T1 behavioral measure should be included in model)	UNR	UNR	Cplx	A linear mixed effects model was used to investigate the relationship between Local-Hreg at T1 in the L ventral occipitotemporal region previously identified and subsequent improvement in spelling accuracy of training items from T1 to T2. A complex model was used in which every voxel for every patient was considered an observation, with random effects of voxel and patient, but this is not described in detail.	Other:<br>There was a significant positive relationship between T1 Local-Hreg and subsequent improvement in spelling accuracy on training items from T1 to T2.
Purcell et al. (2019): Cplx 5	Spelling probe (training items) vs rest	CC<br>Aphasia with both timepoints T1 (n = 20)<br>Covariate: subsequent Δ spelling accuracy on untrained items (T2 vs T1)<br>Somewhat valid (T1 behavioral measure should be included in model)	UNR	UNR	Cplx	A linear mixed effects model was used to investigate the relationship between Local-Hreg at T1 in the L ventral occipitotemporal region previously identified and subsequent improvement in spelling accuracy of untrained items from T1 to T2. A complex model was used in which every voxel for every patient was considered an observation, with random effects of voxel and patient, but this is not described in detail.	Other:<br>There was a significant positive relationship between T1 Local-Hreg and subsequent improvement in spelling accuracy on untrained items from T1 to T2.

Purcell et al. (2019): Cplx 6	Spelling probe (training items) vs rest	LC<br>Aphasia with both timepoints (n = 20) T2 vs T1<br>Covariate: Δ spelling accuracy on training items	UNR	UNR	Cplx	A linear mixed effects model was used to investigate the relationship between change in Local-Hreg in the L ventral occipitotemporal region previously identified and change in spelling accuracy of training items. A complex model was used in which every voxel for every patient was considered an observation, with random effects of voxel and patient, but this is not described in detail.	Other:<br>There was a significant negative relationship between change in Local-Hreg and change in spelling accuracy on training items.
Purcell et al. (2019): Cplx 7	Spelling probe (training items) vs rest	LC<br>Aphasia with both timepoints (n = 20) T2 vs T1<br>Covariate: Δ spelling accuracy on untrained items	UNR	UNR	Cplx	A linear mixed effects model was used to investigate the relationship between change in Local-Hreg in the L ventral occipitotemporal region previously identified and change in spelling accuracy of untrained items. A complex model was used in which every voxel for every patient was considered an observation, with random effects of voxel and patient, but this is not described in detail.	Other:<br>There was a significant negative relationship between change in Local-Hreg and change in spelling accuracy on untrained items.
Purcell et al. (2019): Cplx 8	Spelling probe (training items) vs rest	CC<br>Aphasia with both timepoints T2 (n = 20)<br>Covariate: T2 spelling accuracy on training items	UNR	UNR	Cplx	A linear mixed effects model was used to investigate the relationship between Local-Hreg at T2 in the L ventral occipitotemporal region previously identified and T2 spelling accuracy of training items. A complex model was used in which every voxel for every patient was considered an observation, with random effects of voxel and patient, but this is not described in detail.	None
Purcell et al. (2019): Cplx 9	Spelling probe (training items) vs rest	LC<br>Aphasia with both timepoints (n = 20) T2 vs T1<br>Covariate: previous T1 Local-Hreg in L ventral occipitotemporal ROI<br>Not valid (the ROI was defined based on change in Local-Hreg, so spurious findings could arise in the absence of a real effect)	UNR	UNR	Cplx	A linear mixed effects model was used to investigate the relationship between change in Local-Hreg in the L ventral occipitotemporal region previously identified and T1 Local-Hreg. A complex model was used in which every voxel for every patient was considered an observation, with random effects of voxel and patient, but this is not described in detail.	Other:<br>There was a significant negative relationship between change in Local-Hreg and T1 Local-Hreg.
Purcell et al. (2019): Cplx 10	Spelling probe (training items) vs rest	LC<br>Aphasia with both timepoints (n = 20) T2 vs T1<br>Covariate: Δ spelling accuracy on training items	UNR	UNR	Cplx	A linear mixed effects model was used to investigate the relationship between change in Local-Hreg in the R AG, L PCC, and R PCC and change in spelling accuracy of training items. A complex model was used in which every voxel for every patient was considered an observation, with random effects of voxel and patient, but this is not described in detail.	None
Purcell et al. (2019): Cplx 11	Spelling probe (training items) vs rest	LC<br>Aphasia with both timepoints (n = 20) T2 vs T1<br>Covariate: Δ spelling	UNR	UNR	Cplx	A linear mixed effects model was used to investigate the relationship between change in Local-Hreg in the R AG, L PCC, and R PCC and change in spelling accuracy of untrained items. A	None

		accuracy on untrained items				complex model was used in which every voxel for every patient was considered an observation, with random effects of voxel and patient, but this is not described in detail.	
Sreedharan, Chandran, et al. (2019): ROI 1	Neurofeedback (try to activate language areas) vs rest	CAC Aphasia mean of T1, T2, T3, T4, T5, T6 (neurofeedback patients) or T1, T2 (no training patients) vs control mean	NANB	NANT	ROI Func NDC	Number of ROIs: 4; ROIs: (1) L Broca's area (IFG pars opercularis and triangularis); (2) L Wernicke's area (pSTG); (3-4) homotopic counterparts; how ROIs defined: individual activations within AAL ROIs on a separate word generation localizer	↓ L IFG pars opercularis ↓ L IFG pars triangularis ↓ L posterior STG ↓ R IFG pars opercularis ↓ R IFG pars triangularis ↓ R posterior STG
Sreedharan, Chandran, et al. (2019): ROI 2	Neurofeedback (try to activate language areas) vs rest	CAA Aphasia with neurofeedback training (n = 4) mean of T4, T5, T6 vs no training (n = 4) T2 Somewhat valid (no treatment effect; second half measures rather than measures of change)	NANB	NANT	ROI Func NC	Number of ROIs: 15; ROIs: (1) L Broca's area (IFG pars opercularis and triangularis); (2) L Wernicke's area (pSTG); (3-4) homotopic counterparts; (5) L MFG; (6) L PrCG; (7) L Rolandic operculum; (8) L insula; (9) L IFG pars orbitalis; (10) L MFG orbital; (11) L SMG; (12) L MTG; (13) L PoCG; (14) L AG; (15) L HG; how ROIs defined: (1-4) individual activations within AAL ROIs on a separate word generation localizer; (5-15) AAL	↑ L ventral precentral/inferior frontal junction ↑ L somato-motor
Sreedharan, Chandran, et al. (2019): Cplx 1	Neurofeedback (try to activate language areas) vs rest	CAC Aphasia mean of T1, T2, T3, T4, T5, T6 (neurofeedback patients) or T1, T2 (no training patients) vs control mean	NANB	NANT	Cplx	Signal change in L IFG and L pSTG ROIs was computed, along with functional connectivity between these ROIs. Neurofeedback values were calculated based on signal change as well as correlation between the ROIs. Group differences in neurofeedback values were compared, but not quantified statistically.	Other: Patients received lower neurofeedback values than controls, due to lower signal changes and lower functional connectivity.
Hartwigsen et al. (2020): Vox 1	Syllable count decision vs rest	CAA Aphasia after cTBS to posterior IFG vs sham; same patients, repeated measures	Y	N	Vox C+	Behavioral data notes: significantly slower response times when cTBS was applied over pIFG relative to when sham cTBS was applied; search volume: voxels spared in all patients; software: SPM12; voxelwise p: .001; cluster extent cutoff: based on GRFT	↓ L IFG pars opercularis ↓ L SMA/medial prefrontal ↓ R SMA/medial prefrontal ↓ R basal ganglia notes: based on Figure 4A and Table 3
Hartwigsen et al. (2020): Vox 2	Syllable count decision vs rest	CAA Aphasia after cTBS to posterior IFG vs after cTBS to anterior IFG; same patients, repeated measures	Y	N	Vox C+	Behavioral data notes: significantly slower response times when cTBS was applied over pIFG relative to when cTBS was applied over aIFG; search volume: voxels spared in all patients; software: SPM12; voxelwise p: .001; cluster extent cutoff: based on GRFT	↓ L IFG pars opercularis notes: based on Table 3
Hartwigsen et al. (2020): Vox 3	Semantic decision vs rest	CAA Aphasia after cTBS to anterior IFG vs sham; same patients, repeated measures Somewhat valid (no behavioral difference)	Y	Y	Vox C+	Behavioral data notes: difference in reaction time did not survive correction; search volume: voxels spared in all patients; software: SPM12; voxelwise p: .001; cluster extent cutoff: based on GRFT	↓ L insula ↓ L dorsolateral prefrontal cortex ↓ R insula ↓ R dorsolateral prefrontal cortex ↓ R SMA/medial

							prefrontal<br>notes: based on Figure 4B and Table 3
Hartwigsen et al. (2020): Vox 4	Semantic decision vs rest	CAA<br>Aphasia after cTBS to anterior IFG vs after cTBS to posterior IFG ; same patients, repeated measures	Y	N	Vox C+	Behavioral data notes: significantly slower response times when cTBS was applied over aIFG relative to when cTBS was applied over pIFG; search volume: voxels spared in all patients; software: SPM12; voxelwise p: .001; cluster extent cutoff: based on GRFT	↓ L insula<br>↓ R insula<br>↓ R dorsolateral prefrontal cortex<br>notes: based on Table 3
Hartwigsen et al. (2020): Cplx 1	Syllable count decision vs rest	CC<br>Aphasia after cTBS to posterior IFG vs sham; same patients, repeated measures<br>Covariate: Δ RT for syllable decision (cTBS to posterior IFG timepoint vs sham timepoint)	UNR	C	Cplx	Whole brain correlations were computed between the difference in functional activity after cTBS to posterior IFG versus sham stimulation, and the difference in reaction times on the syllable counting task under these two conditions. The resulting SPM was thresholded at voxelwise p < .001 (CDT) followed by correction for multiple comparisons based on cluster extent and GRFT using SPM12.	Other: Upregulation of the R supramarginal gyrus after cTBS was significantly associated with slowing of RT after cTBS. This finding remained significant after including lesion volume as covariate.
Hartwigsen et al. (2020): Cplx 2	Semantic decision vs rest	CC<br>Aphasia after cTBS to anterior IFG vs sham; same patients, repeated measures<br>Covariate: Δ RT for semantic decision (cTBS to posterior IFG timepoint vs sham timepoint)	UNR	C	Cplx	Whole brain correlations were computed between the difference in functional activity after cTBS to anterior IFG versus sham stimulation, and the difference in reaction times on the semantic decision task under these two conditions. The resulting SPM was thresholded at voxelwise p < .001 (CDT) followed by correction for multiple comparisons based on cluster extent and GRFT using SPM12.	None
Stockert et al. (2020): ROI 1	Listening to normal sentences and making a plausibility judgment (paradigm 1) or listening to normal sentences (paradigm 2) vs listening to reversed speech	LA<br>Aphasia T2 vs T1	UNR	UNR	ROI Func NC	Behavioral data notes: no differences in proportion of expected button presses by group or time, but behavioral data pooled across conditions; number of ROIs: 13; ROIs: (1) L IFG orb; (2) L IFG tri; (3) L IFG op; (4) L DLPFC; (5) L insula; (6) L ATL; (7) L PTL; (8) L SMA/dACC; (9) R L IFG orb; (10) R IFG tri; (11) R insula; (12) R DLPFC; (13) R ATL; how ROIs defined: spheres around peaks of whole brain analysis of all patients collapsing across groups and timepoints; post-hoc tests comparing 2 out of the 3 time points were corrected using the Bonferroni-Holm procedure, but there is no indication that that multiple comparisons across ROIs were accounted for	↑ L IFG pars orbitalis<br>↑ L insula<br>↑ L dorsolateral prefrontal cortex<br>↑ L SMA/medial prefrontal<br>↑ R insula<br>notes: based on Figure 3; several additional regions are mentioned in text and/or Table 1
Stockert et al. (2020): ROI 2	Listening to normal sentences and making a plausibility judgment (paradigm 1) or listening to normal	LA<br>Aphasia T3 vs T1	UNR	UNR	ROI Func NC	Behavioral data notes: no differences in proportion of expected button presses by group or time, but behavioral data pooled across conditions; number of ROIs: 13; ROIs: (1) L IFG orb; (2) L IFG tri; (3) L IFG op; (4) L DLPFC; (5) L insula; (6) L ATL; (7) L	↑ L IFG pars orbitalis<br>↑ L dorsolateral prefrontal cortex<br>↑ L posterior STG/STS/MTG<br>↑ L anterior

	sentences (paradigm 2) vs listening to reversed speech					PTL; (8) L SMA/dACC; (9) R L IFG orb; (10) R IFG tri; (11) R insula; (12) R DLPFC; (13) R ATL; how ROIs defined: spheres around peaks of whole brain analysis of all patients collapsing across groups and timepoints; post-hoc tests comparing 2 out of the 3 time points were corrected using the Bonferroni-Holm procedure, but there is no indication that that multiple comparisons across ROIs were accounted for	temporal<br>notes: based on Figure 3; several additional regions are mentioned in text and/or Table 1
Stockert et al. (2020): ROI 3	Listening to normal sentences and making a plausibility judgment (paradigm 1) or listening to normal sentences (paradigm 2) vs listening to reversed speech	LA<br>Aphasia T3 vs T2	UNR	UNR	ROI Func NC	Behavioral data notes: no differences in proportion of expected button presses by group or time, but behavioral data pooled across conditions; number of ROIs: 13; ROIs: (1) L IFG orb; (2) L IFG tri; (3) L IFG op; (4) L DLPFC; (5) L insula; (6) L ATL; (7) L PTL; (8) L SMA/dACC; (9) R L IFG orb; (10) R IFG tri; (11) R insula; (12) R DLPFC; (13) R ATL; how ROIs defined: spheres around peaks of whole brain analysis of all patients collapsing across groups and timepoints; post-hoc tests comparing 2 out of the 3 time points were corrected using the Bonferroni-Holm procedure, but there is no indication that that multiple comparisons across ROIs were accounted for	None<br>notes: based on Figure 3; several additional regions are mentioned in text and/or Table 1
Stockert et al. (2020): ROI 4	Listening to normal sentences and making a plausibility judgment (paradigm 1) or listening to normal sentences (paradigm 2) vs listening to reversed speech	CAA<br>Aphasia frontal mean of T1, T2, T3 (n = 17) vs temporo-parietal mean of T1, T2, T3 (n = 17)	UNR	UNR	ROI Func NC	Behavioral data notes: no differences in proportion of expected button presses by group or time, but behavioral data pooled across conditions; number of ROIs: 13; ROIs: (1) L IFG orb; (2) L IFG tri; (3) L IFG op; (4) L DLPFC; (5) L insula; (6) L ATL; (7) L PTL; (8) L SMA/dACC; (9) R L IFG orb; (10) R IFG tri; (11) R insula; (12) R DLPFC; (13) R ATL; how ROIs defined: spheres around peaks of whole brain analysis of all patients collapsing across groups and timepoints	↑ L posterior STG/STS/MTG<br>↑ R IFG pars orbitalis<br>↑ R anterior temporal<br>↓ L IFG pars opercularis<br>↓ L IFG pars triangularis<br>↓ L dorsolateral prefrontal cortex<br>notes: based on Table 1
Stockert et al. (2020): ROI 5	Listening to normal sentences and making a plausibility judgment (paradigm 1) or listening to normal sentences (paradigm 2) vs listening to reversed speech	LAA<br>(Aphasia frontal (n = 17) T2 vs T1) vs (temporo-parietal (n = 17) T2 vs T1)	UNR	UNR	ROI Func NC	Behavioral data notes: no differences in proportion of expected button presses by group or time, but behavioral data pooled across conditions; number of ROIs: 13; ROIs: (1) L IFG orb; (2) L IFG tri; (3) L IFG op; (4) L DLPFC; (5) L insula; (6) L ATL; (7) L PTL; (8) L SMA/dACC; (9) R L IFG orb; (10) R IFG tri; (11) R insula; (12) R DLPFC; (13) R ATL; how ROIs defined: spheres around peaks of whole brain analysis of all patients collapsing across groups and timepoints; interactions were significant in model with all 3 time points; post-hoc sub-interactions not reported but the patterns appear clear	↓ L IFG pars opercularis<br>↓ L IFG pars triangularis<br>↓ R IFG pars triangularis<br>↓ R dorsolateral prefrontal cortex

Stockert et al. (2020): ROI 6	Listening to normal sentences and making a plausibility judgment (paradigm 1) or listening to normal sentences (paradigm 2) vs listening to reversed speech	LAA (Aphasia frontal (n = 17) T3 vs T1) vs (temporo-parietal (n = 17) T3 vs T1)	UNR	UNR	ROI Func NC	Behavioral data notes: no differences in proportion of expected button presses by group or time, but behavioral data pooled across conditions; number of ROIs: 13; ROIs: (1) L IFG orb; (2) L IFG tri; (3) L IFG op; (4) L DLPFC; (5) L insula; (6) L ATL; (7) L PTL; (8) L SMA/dACC; (9) R L IFG orb; (10) R IFG tri; (11) R insula; (12) R DLPFC; (13) R ATL; how ROIs defined: spheres around peaks of whole brain analysis of all patients collapsing across groups and timepoints; interactions were significant in model with all 3 time points; post-hoc sub-interactions not reported and patterns are not clear	↓ L IFG pars opercularis<br>↓ L IFG pars triangularis<br>↓ R IFG pars triangularis<br>↓ R dorsolateral prefrontal cortex
Stockert et al. (2020): ROI 7	Listening to normal sentences and making a plausibility judgment (paradigm 1) or listening to normal sentences (paradigm 2) vs listening to reversed speech	LAA (Aphasia frontal (n = 17) T3 vs T2) vs (temporo-parietal (n = 17) T3 vs T2)	UNR	UNR	ROI Func NC	Behavioral data notes: no differences in proportion of expected button presses by group or time, but behavioral data pooled across conditions; number of ROIs: 13; ROIs: (1) L IFG orb; (2) L IFG tri; (3) L IFG op; (4) L DLPFC; (5) L insula; (6) L ATL; (7) L PTL; (8) L SMA/dACC; (9) R L IFG orb; (10) R IFG tri; (11) R insula; (12) R DLPFC; (13) R ATL; how ROIs defined: spheres around peaks of whole brain analysis of all patients collapsing across groups and timepoints; post-hoc sub-interactions not reported but there do not appear to be any T2/T3 effects	None
Stockert et al. (2020): ROI 8	Listening to normal sentences and making a plausibility judgment (paradigm 1) or listening to normal sentences (paradigm 2) vs listening to reversed speech	LA Aphasia T2 vs T1	UNR	UNR	ROI Oth NC	Behavioral data notes: no differences in proportion of expected button presses by group or time, but behavioral data pooled across conditions; number of ROIs: 2; ROIs: (1) perilesional tissue; (2) regions homotopic to lesions; each unique to individuals; how ROIs defined: (1) perilesional ROIs were voxels 3-15 mm from the lesion that were located in frontal or temporal regions activated by the language contrast in controls; (2) homotopic ROIs were flipped lesions; test of group by time interaction not reported	Other: there was a significant increase in activation in perilesional ROIs
Stockert et al. (2020): ROI 9	Listening to normal sentences and making a plausibility judgment (paradigm 1) or listening to normal sentences (paradigm 2) vs listening to reversed speech	LA Aphasia T3 vs T1	UNR	UNR	ROI Oth NC	Behavioral data notes: no differences in proportion of expected button presses by group or time, but behavioral data pooled across conditions; number of ROIs: 2; ROIs: (1) perilesional tissue; (2) regions homotopic to lesions; each unique to individuals; how ROIs defined: (1) perilesional ROIs were voxels 3-15 mm from the lesion that were located in frontal or temporal regions activated by the language contrast in controls; (2) homotopic ROIs were flipped	Other: there was a significant increase in activation in perilesional ROIs

						lesions; test of group by time interaction not reported	
Stockert et al. (2020): ROI 10	Listening to normal sentences and making a plausibility judgment (paradigm 1) or listening to normal sentences (paradigm 2) vs listening to reversed speech	LA<br>Aphasia T3 vs T2	UNR	UNR	ROI Oth NC	Behavioral data notes: no differences in proportion of expected button presses by group or time, but behavioral data pooled across conditions; number of ROIs: 2; ROIs: (1) perilesional tissue; (2) regions homotopic to lesions; each unique to individuals; how ROIs defined: (1) perilesional ROIs were voxels 3-15 mm from the lesion that were located in frontal or temporal regions activated by the language contrast in controls; (2) homotopic ROIs were flipped lesions; test of group by time interaction not reported	None
Stockert et al. (2020): ROI 11	Listening to normal sentences and making a plausibility judgment (paradigm 1) or listening to normal sentences (paradigm 2) vs listening to reversed speech	CAA<br>Aphasia frontal mean of T1, T2, T3 (n = 17) vs temporo-parietal mean of T1, T2, T3 (n = 17)	UNR	UNR	ROI Oth NC	Behavioral data notes: no differences in proportion of expected button presses by group or time, but behavioral data pooled across conditions; number of ROIs: 2; ROIs: (1) perilesional tissue; (2) regions homotopic to lesions; each unique to individuals; how ROIs defined: (1) perilesional ROIs were voxels 3-15 mm from the lesion that were located in frontal or temporal regions activated by the language contrast in controls; (2) homotopic ROIs were flipped lesions; test of group by time interaction not reported; this comparison is somewhat questionable given the differing extent to which frontal and temporal regions are activated in controls	Other: frontal patients showed relatively greater activation in regions homotopic to their lesions
Stockert et al. (2020): ROI 12	Listening to normal sentences and making a plausibility judgment (paradigm 1) or listening to normal sentences (paradigm 2) vs listening to reversed speech	CAC<br>Aphasia frontal T1 (n = 17) vs control	UNR	UNR	ROI Func NC	Behavioral data notes: no differences in proportion of expected button presses by group or time, but behavioral data pooled across conditions; number of ROIs: 13; ROIs: (1) L IFG orb; (2) L IFG tri; (3) L IFG op; (4) L DLPFC; (5) L insula; (6) L ATL; (7) L PTL; (8) L SMA/dACC; (9) R L IFG orb; (10) R IFG tri; (11) R insula; (12) R DLPFC; (13) R ATL; how ROIs defined: spheres around peaks of whole brain analysis of all patients collapsing across groups and timepoints; circular because patients but not controls used to define ROIs	↓ L IFG pars triangularis<br>↓ L insula<br>↓ L dorsolateral prefrontal cortex
Stockert et al. (2020): ROI 13	Listening to normal sentences and making a plausibility judgment (paradigm 1) or listening to normal sentences (paradigm 2) vs listening to reversed speech	CAC<br>Aphasia temporo-parietal T1 (n = 17) vs control	UNR	UNR	ROI Func NC	Behavioral data notes: no differences in proportion of expected button presses by group or time, but behavioral data pooled across conditions; number of ROIs: 13; ROIs: (1) L IFG orb; (2) L IFG tri; (3) L IFG op; (4) L DLPFC; (5) L insula; (6) L ATL; (7) L PTL; (8) L SMA/dACC; (9) R L IFG orb; (10) R IFG tri; (11) R insula; (12) R DLPFC; (13) R ATL; how ROIs defined: spheres around peaks of whole brain	↓ L IFG pars triangularis<br>↓ L insula<br>↓ L dorsolateral prefrontal cortex<br>↓ L SMA/medial prefrontal<br>↓ L posterior STG/STS/MTG<br>↓ R IFG pars triangularis

						analysis of all patients collapsing across groups and timepoints; circular because patients but not controls used to define ROIs	
Stockert et al. (2020): ROI 14	Listening to normal sentences and making a plausibility judgment (paradigm 1) or listening to normal sentences (paradigm 2) vs listening to reversed speech	CAA Aphasia frontal T1 (n = 17) vs temporo-parietal T1 (n = 17)	UNR	UNR	ROI Func NC	Behavioral data notes: no differences in proportion of expected button presses by group or time, but behavioral data pooled across conditions; number of ROIs: 13; ROIs: (1) L IFG orb; (2) L IFG tri; (3) L IFG op; (4) L DLPFC; (5) L insula; (6) L ATL; (7) L PTL; (8) L SMA/dACC; (9) R L IFG orb; (10) R IFG tri; (11) R insula; (12) R DLPFC; (13) R ATL; how ROIs defined: spheres around peaks of whole brain analysis of all patients collapsing across groups and timepoints	↑ L anterior temporal<br>↑ R IFG pars triangularis<br>↑ R anterior temporal
Stockert et al. (2020): ROI 15	Listening to normal sentences and making a plausibility judgment (paradigm 1) or listening to normal sentences (paradigm 2) vs listening to reversed speech	CAC Aphasia frontal T2 (n = 17) vs control	UNR	UNR	ROI Func NC	Behavioral data notes: no differences in proportion of expected button presses by group or time, but behavioral data pooled across conditions; number of ROIs: 13; ROIs: (1) L IFG orb; (2) L IFG tri; (3) L IFG op; (4) L DLPFC; (5) L insula; (6) L ATL; (7) L PTL; (8) L SMA/dACC; (9) R L IFG orb; (10) R IFG tri; (11) R insula; (12) R DLPFC; (13) R ATL; how ROIs defined: spheres around peaks of whole brain analysis of all patients collapsing across groups and timepoints; circular because patients but not controls used to define ROIs	↓ L IFG pars triangularis
Stockert et al. (2020): ROI 16	Listening to normal sentences and making a plausibility judgment (paradigm 1) or listening to normal sentences (paradigm 2) vs listening to reversed speech	CAC Aphasia temporo-parietal T2 (n = 17) vs control	UNR	UNR	ROI Func NC	Behavioral data notes: no differences in proportion of expected button presses by group or time, but behavioral data pooled across conditions; number of ROIs: 13; ROIs: (1) L IFG orb; (2) L IFG tri; (3) L IFG op; (4) L DLPFC; (5) L insula; (6) L ATL; (7) L PTL; (8) L SMA/dACC; (9) R L IFG orb; (10) R IFG tri; (11) R insula; (12) R DLPFC; (13) R ATL; how ROIs defined: spheres around peaks of whole brain analysis of all patients collapsing across groups and timepoints; circular because patients but not controls used to define ROIs	None
Stockert et al. (2020): ROI 17	Listening to normal sentences and making a plausibility judgment (paradigm 1) or listening to normal sentences (paradigm 2) vs listening to reversed speech	CAA Aphasia frontal T2 (n = 17) vs temporo-parietal T2 (n = 17)	UNR	UNR	ROI Func NC	Behavioral data notes: no differences in proportion of expected button presses by group or time, but behavioral data pooled across conditions; number of ROIs: 13; ROIs: (1) L IFG orb; (2) L IFG tri; (3) L IFG op; (4) L DLPFC; (5) L insula; (6) L ATL; (7) L PTL; (8) L SMA/dACC; (9) R L IFG orb; (10) R IFG tri; (11) R insula; (12) R DLPFC; (13) R ATL; how ROIs defined: spheres around peaks of whole brain analysis of all patients collapsing across groups and timepoints	↓ L IFG pars opercularis<br>↓ L IFG pars triangularis<br>↓ L dorsolateral prefrontal cortex
Stockert et al. (2020):	Listening to normal sentences	CAC Aphasia frontal T3 (n	UNR	UNR	ROI Func	Behavioral data notes: no differences in proportion of expected button	↓ L IFG pars triangularis

ROI 18	and making a plausibility judgment (paradigm 1) or listening to normal sentences (paradigm 2) vs listening to reversed speech	= 17) vs control			NC	presses by group or time, but behavioral data pooled across conditions; number of ROIs: 13; ROIs: (1) L IFG orb; (2) L IFG tri; (3) L IFG op; (4) L DLPFC; (5) L insula; (6) L ATL; (7) L PTL; (8) L SMA/dACC; (9) R L IFG orb; (10) R IFG tri; (11) R insula; (12) R DLPFC; (13) R ATL; how ROIs defined: spheres around peaks of whole brain analysis of all patients collapsing across groups and timepoints; circular because patients but not controls used to define ROIs	↓ L insula
Stockert et al. (2020): ROI 19	Listening to normal sentences and making a plausibility judgment (paradigm 1) or listening to normal sentences (paradigm 2) vs listening to reversed speech	CAC Aphasia temporo-parietal T3 (n = 17) vs control	UNR	UNR	ROI Func NC	Behavioral data notes: no differences in proportion of expected button presses by group or time, but behavioral data pooled across conditions; number of ROIs: 13; ROIs: (1) L IFG orb; (2) L IFG tri; (3) L IFG op; (4) L DLPFC; (5) L insula; (6) L ATL; (7) L PTL; (8) L SMA/dACC; (9) R L IFG orb; (10) R IFG tri; (11) R insula; (12) R DLPFC; (13) R ATL; how ROIs defined: spheres around peaks of whole brain analysis of all patients collapsing across groups and timepoints; circular because patients but not controls used to define ROIs	None
Stockert et al. (2020): ROI 20	Listening to normal sentences and making a plausibility judgment (paradigm 1) or listening to normal sentences (paradigm 2) vs listening to reversed speech	CAA Aphasia frontal T3 (n = 17) vs temporo-parietal T3 (n = 17)	UNR	UNR	ROI Func NC	Behavioral data notes: no differences in proportion of expected button presses by group or time, but behavioral data pooled across conditions; number of ROIs: 13; ROIs: (1) L IFG orb; (2) L IFG tri; (3) L IFG op; (4) L DLPFC; (5) L insula; (6) L ATL; (7) L PTL; (8) L SMA/dACC; (9) R L IFG orb; (10) R IFG tri; (11) R insula; (12) R DLPFC; (13) R ATL; how ROIs defined: spheres around peaks of whole brain analysis of all patients collapsing across groups and timepoints	↓ L IFG pars opercularis<br>↓ L IFG pars triangularis<br>↓ L IFG pars orbitalis<br>↓ L dorsolateral prefrontal cortex
Stockert et al. (2020): ROI 21	Listening to normal sentences and making a plausibility judgment (paradigm 1) or listening to normal sentences (paradigm 2) vs listening to reversed speech	CAC Aphasia frontal T1 (n = 17) vs control	UNR	UNR	ROI Oth NC	Behavioral data notes: no differences in proportion of expected button presses by group or time, but behavioral data pooled across conditions; number of ROIs: 2; ROIs: (1) perilesional tissue; (2) regions homotopic to lesions; each unique to individuals; how ROIs defined: (1) perilesional ROIs were voxels 3-15 mm from the lesion that were located in frontal or temporal regions activated by the language contrast in controls; (2) homotopic ROIs were flipped lesions	Other: frontal patients showed reduced activation in perilesional tissue
Stockert et al. (2020): ROI 22	Listening to normal sentences and making a plausibility judgment (paradigm 1) or listening to normal	CAC Aphasia frontal T2 (n = 17) vs control	UNR	UNR	ROI Oth NC	Behavioral data notes: no differences in proportion of expected button presses by group or time, but behavioral data pooled across conditions; number of ROIs: 2; ROIs: (1) perilesional tissue; (2) regions homotopic to lesions; each unique to	Other: frontal patients showed reduced activation in perilesional tissue

	sentences (paradigm 2) vs listening to reversed speech					individuals; how ROIs defined: (1) perilesional ROIs were voxels 3-15 mm from the lesion that were located in frontal or temporal regions activated by the language contrast in controls; (2) homotopic ROIs were flipped lesions	
Stockert et al. (2020): ROI 23	Listening to normal sentences and making a plausibility judgment (paradigm 1) or listening to normal sentences (paradigm 2) vs listening to reversed speech	CAC Aphasia frontal T3 (n = 17) vs control	UNR	UNR	ROI Oth NC	Behavioral data notes: no differences in proportion of expected button presses by group or time, but behavioral data pooled across conditions; number of ROIs: 2; ROIs: (1) perilesional tissue; (2) regions homotopic to lesions; each unique to individuals; how ROIs defined: (1) perilesional ROIs were voxels 3-15 mm from the lesion that were located in frontal or temporal regions activated by the language contrast in controls; (2) homotopic ROIs were flipped lesions	Other: frontal patients showed reduced activation in perilesional tissue
Stockert et al. (2020): ROI 24	Listening to normal sentences and making a plausibility judgment (paradigm 1) or listening to normal sentences (paradigm 2) vs listening to reversed speech	CAC Aphasia temporo-parietal T1 (n = 17) vs control	UNR	UNR	ROI Oth NC	Behavioral data notes: no differences in proportion of expected button presses by group or time, but behavioral data pooled across conditions; number of ROIs: 2; ROIs: (1) perilesional tissue; (2) regions homotopic to lesions; each unique to individuals; how ROIs defined: (1) perilesional ROIs were voxels 3-15 mm from the lesion that were located in frontal or temporal regions activated by the language contrast in controls; (2) homotopic ROIs were flipped lesions	Other: temporal patients showed reduced activation in perilesional tissue and in regions homotopic to their lesions
Stockert et al. (2020): ROI 25	Listening to normal sentences and making a plausibility judgment (paradigm 1) or listening to normal sentences (paradigm 2) vs listening to reversed speech	CAC Aphasia temporo-parietal T2 (n = 17) vs control	UNR	UNR	ROI Oth NC	Behavioral data notes: no differences in proportion of expected button presses by group or time, but behavioral data pooled across conditions; number of ROIs: 2; ROIs: (1) perilesional tissue; (2) regions homotopic to lesions; each unique to individuals; how ROIs defined: (1) perilesional ROIs were voxels 3-15 mm from the lesion that were located in frontal or temporal regions activated by the language contrast in controls; (2) homotopic ROIs were flipped lesions	None
Stockert et al. (2020): ROI 26	Listening to normal sentences and making a plausibility judgment (paradigm 1) or listening to normal sentences (paradigm 2) vs listening to reversed speech	CAC Aphasia temporo-parietal T3 (n = 17) vs control	UNR	UNR	ROI Oth NC	Behavioral data notes: no differences in proportion of expected button presses by group or time, but behavioral data pooled across conditions; number of ROIs: 2; ROIs: (1) perilesional tissue; (2) regions homotopic to lesions; each unique to individuals; how ROIs defined: (1) perilesional ROIs were voxels 3-15 mm from the lesion that were located in frontal or temporal regions activated by the language contrast in controls;	None

						(2) homotopic ROIs were flipped lesions	
Stockert et al. (2020): ROI 27	Listening to normal sentences and making a plausibility judgment (paradigm 1) or listening to normal sentences (paradigm 2) vs listening to reversed speech	CC Aphasia T1 Covariate: comprehension composite	UNR	UNR	ROI Mix NC	Behavioral data notes: no differences in proportion of expected button presses by group or time, but behavioral data pooled across conditions; number of ROIs: 15; ROIs: (1) L IFG orb; (2) L IFG tri; (3) L IFG op; (4) L DLPFC; (5) L insula; (6) L ATL; (7) L PTL; (8) L SMA/dACC; (9) R L IFG orb; (10) R IFG tri; (11) R insula; (12) R DLPFC; (13) R ATL; (14) perilesional tissue; (15) regions homotopic to lesions; how ROIs defined: (1-13) spheres around peaks of whole brain analysis of all patients collapsing across groups and timepoints; (14) perilesional ROIs were voxels 3-15 mm from the lesion that were located in frontal or temporal regions activated by the language contrast in controls; (15) homotopic ROIs were flipped lesions	↑ L IFG pars opercularis<br>↑ L IFG pars triangularis<br>↑ L IFG pars orbitalis<br>other:<br>L IFG pars opercularis and orbitalis did not remain significant when lesion volume was included as a covariate; there was a significant correlation between perilesional activation and LRScomp; this did not remain significant when lesion volume was included as a covariate
Stockert et al. (2020): ROI 28	Listening to normal sentences and making a plausibility judgment (paradigm 1) or listening to normal sentences (paradigm 2) vs listening to reversed speech	CC Aphasia T2 Covariate: comprehension composite	UNR	UNR	ROI Mix NC	Behavioral data notes: no differences in proportion of expected button presses by group or time, but behavioral data pooled across conditions; number of ROIs: 15; ROIs: (1) L IFG orb; (2) L IFG tri; (3) L IFG op; (4) L DLPFC; (5) L insula; (6) L ATL; (7) L PTL; (8) L SMA/dACC; (9) R L IFG orb; (10) R IFG tri; (11) R insula; (12) R DLPFC; (13) R ATL; (14) perilesional tissue; (15) regions homotopic to lesions; how ROIs defined: (1-13) spheres around peaks of whole brain analysis of all patients collapsing across groups and timepoints; (14) perilesional ROIs were voxels 3-15 mm from the lesion that were located in frontal or temporal regions activated by the language contrast in controls; (15) homotopic ROIs were flipped lesions	↑ L IFG pars triangularis<br>other:<br>there was a significant correlation between perilesional activation and LRScomp
Stockert et al. (2020): ROI 29	Listening to normal sentences and making a plausibility judgment (paradigm 1) or listening to normal sentences (paradigm 2) vs listening to reversed speech	CC Aphasia T3 Covariate: comprehension composite	UNR	UNR	ROI Mix NC	Behavioral data notes: no differences in proportion of expected button presses by group or time, but behavioral data pooled across conditions; number of ROIs: 15; ROIs: (1) L IFG orb; (2) L IFG tri; (3) L IFG op; (4) L DLPFC; (5) L insula; (6) L ATL; (7) L PTL; (8) L SMA/dACC; (9) R L IFG orb; (10) R IFG tri; (11) R insula; (12) R DLPFC; (13) R ATL; (14) perilesional tissue; (15) regions homotopic to lesions; how ROIs defined: (1-13) spheres around peaks of whole brain	↑ L IFG pars triangularis<br>notes: did not remain significant when lesion volume was included as a covariate

						analysis of all patients collapsing across groups and timepoints; (14) perilesional ROIs were voxels 3-15 mm from the lesion that were located in frontal or temporal regions activated by the language contrast in controls; (15) homotopic ROIs were flipped lesions	
Stockert et al. (2020): ROI 30	Listening to normal sentences and making a plausibility judgment (paradigm 1) or listening to normal sentences (paradigm 2) vs listening to reversed speech	LC Aphasia T2 vs T1 Covariate: Δ comprehension composite	UNR	UNR	ROI Mix NC	Behavioral data notes: no differences in proportion of expected button presses by group or time, but behavioral data pooled across conditions; number of ROIs: 15; ROIs: (1) L IFG orb; (2) L IFG tri; (3) L IFG op; (4) L DLPFC; (5) L insula; (6) L ATL; (7) L PTL; (8) L SMA/dACC; (9) R L IFG orb; (10) R IFG tri; (11) R insula; (12) R DLPFC; (13) R ATL; (14) perilesional tissue; (15) regions homotopic to lesions; how ROIs defined: (1-13) spheres around peaks of whole brain analysis of all patients collapsing across groups and timepoints; (14) perilesional ROIs were voxels 3-15 mm from the lesion that were located in frontal or temporal regions activated by the language contrast in controls; (15) homotopic ROIs were flipped lesions	↑ L insula ↑ R dorsolateral prefrontal cortex notes: R dorsolateral prefrontal cortex did not remain significant when lesion volume was included as a covariate
Stockert et al. (2020): ROI 31	Listening to normal sentences and making a plausibility judgment (paradigm 1) or listening to normal sentences (paradigm 2) vs listening to reversed speech	LC Aphasia T3 vs T1 Covariate: Δ comprehension composite	UNR	UNR	ROI Mix NC	Behavioral data notes: no differences in proportion of expected button presses by group or time, but behavioral data pooled across conditions; number of ROIs: 15; ROIs: (1) L IFG orb; (2) L IFG tri; (3) L IFG op; (4) L DLPFC; (5) L insula; (6) L ATL; (7) L PTL; (8) L SMA/dACC; (9) R L IFG orb; (10) R IFG tri; (11) R insula; (12) R DLPFC; (13) R ATL; (14) perilesional tissue; (15) regions homotopic to lesions; how ROIs defined: (1-13) spheres around peaks of whole brain analysis of all patients collapsing across groups and timepoints; (14) perilesional ROIs were voxels 3-15 mm from the lesion that were located in frontal or temporal regions activated by the language contrast in controls; (15) homotopic ROIs were flipped lesions	None
Stockert et al. (2020): ROI 32	Listening to normal sentences and making a plausibility judgment (paradigm 1) or listening to normal sentences (paradigm 2) vs listening to reversed speech	LC Aphasia T3 vs T2 Covariate: Δ comprehension composite	UNR	UNR	ROI Mix NC	Behavioral data notes: no differences in proportion of expected button presses by group or time, but behavioral data pooled across conditions; number of ROIs: 15; ROIs: (1) L IFG orb; (2) L IFG tri; (3) L IFG op; (4) L DLPFC; (5) L insula; (6) L ATL; (7) L PTL; (8) L SMA/dACC; (9) R L IFG orb; (10) R IFG tri; (11) R insula; (12) R DLPFC; (13) R ATL; (14) perilesional tissue; (15) regions homotopic to lesions; how ROIs defined: (1-13)	None

						spheres around peaks of whole brain analysis of all patients collapsing across groups and timepoints; (14) perilesional ROIs were voxels 3-15 mm from the lesion that were located in frontal or temporal regions activated by the language contrast in controls; (15) homotopic ROIs were flipped lesions	
Stockert et al. (2020): ROI 33	Listening to normal sentences and making a plausibility judgment (paradigm 1) or listening to normal sentences (paradigm 2) vs listening to reversed speech	CC<br>Aphasia frontal T1 (n = 17)<br>Covariate: comprehension composite	UNR	UNR	ROI<br>Mix<br>NC	Behavioral data notes: no differences in proportion of expected button presses by group or time, but behavioral data pooled across conditions; number of ROIs: 15; ROIs: (1) L IFG orb; (2) L IFG tri; (3) L IFG op; (4) L DLPFC; (5) L insula; (6) L ATL; (7) L PTL; (8) L SMA/dACC; (9) R L IFG orb; (10) R IFG tri; (11) R insula; (12) R DLPFC; (13) R ATL; (14) perilesional tissue; (15) regions homotopic to lesions; how ROIs defined: (1-13) spheres around peaks of whole brain analysis of all patients collapsing across groups and timepoints; (14) perilesional ROIs were voxels 3-15 mm from the lesion that were located in frontal or temporal regions activated by the language contrast in controls; (15) homotopic ROIs were flipped lesions	None
Stockert et al. (2020): ROI 34	Listening to normal sentences and making a plausibility judgment (paradigm 1) or listening to normal sentences (paradigm 2) vs listening to reversed speech	CC<br>Aphasia frontal T2 (n = 17)<br>Covariate: comprehension composite	UNR	UNR	ROI<br>Mix<br>NC	Behavioral data notes: no differences in proportion of expected button presses by group or time, but behavioral data pooled across conditions; number of ROIs: 15; ROIs: (1) L IFG orb; (2) L IFG tri; (3) L IFG op; (4) L DLPFC; (5) L insula; (6) L ATL; (7) L PTL; (8) L SMA/dACC; (9) R L IFG orb; (10) R IFG tri; (11) R insula; (12) R DLPFC; (13) R ATL; (14) perilesional tissue; (15) regions homotopic to lesions; how ROIs defined: (1-13) spheres around peaks of whole brain analysis of all patients collapsing across groups and timepoints; (14) perilesional ROIs were voxels 3-15 mm from the lesion that were located in frontal or temporal regions activated by the language contrast in controls; (15) homotopic ROIs were flipped lesions	None
Stockert et al. (2020): ROI 35	Listening to normal sentences and making a plausibility judgment (paradigm 1) or listening to normal sentences (paradigm 2) vs listening to reversed speech	CC<br>Aphasia frontal T3 (n = 17)<br>Covariate: comprehension composite	UNR	UNR	ROI<br>Mix<br>NC	Behavioral data notes: no differences in proportion of expected button presses by group or time, but behavioral data pooled across conditions; number of ROIs: 15; ROIs: (1) L IFG orb; (2) L IFG tri; (3) L IFG op; (4) L DLPFC; (5) L insula; (6) L ATL; (7) L PTL; (8) L SMA/dACC; (9) R L IFG orb; (10) R IFG tri; (11) R insula; (12) R DLPFC; (13) R ATL; (14) perilesional tissue; (15) regions homotopic to	None

						lesions; how ROIs defined: (1-13) spheres around peaks of whole brain analysis of all patients collapsing across groups and timepoints; (14) perilesional ROIs were voxels 3-15 mm from the lesion that were located in frontal or temporal regions activated by the language contrast in controls; (15) homotopic ROIs were flipped lesions	
Stockert et al. (2020): ROI 36	Listening to normal sentences and making a plausibility judgment (paradigm 1) or listening to normal sentences (paradigm 2) vs listening to reversed speech	LC<br>Aphasia frontal (n = 17) T2 vs T1<br>Covariate: Δ comprehension composite	UNR	UNR	ROI<br>Mix<br>NC	Behavioral data notes: no differences in proportion of expected button presses by group or time, but behavioral data pooled across conditions; number of ROIs: 15; ROIs: (1) L IFG orb; (2) L IFG tri; (3) L IFG op; (4) L DLPFC; (5) L insula; (6) L ATL; (7) L PTL; (8) L SMA/dACC; (9) R L IFG orb; (10) R IFG tri; (11) R insula; (12) R DLPFC; (13) R ATL; (14) perilesional tissue; (15) regions homotopic to lesions; how ROIs defined: (1-13) spheres around peaks of whole brain analysis of all patients collapsing across groups and timepoints; (14) perilesional ROIs were voxels 3-15 mm from the lesion that were located in frontal or temporal regions activated by the language contrast in controls; (15) homotopic ROIs were flipped lesions	None
Stockert et al. (2020): ROI 37	Listening to normal sentences and making a plausibility judgment (paradigm 1) or listening to normal sentences (paradigm 2) vs listening to reversed speech	LC<br>Aphasia frontal (n = 17) T3 vs T1<br>Covariate: Δ comprehension composite	UNR	UNR	ROI<br>Mix<br>NC	Behavioral data notes: no differences in proportion of expected button presses by group or time, but behavioral data pooled across conditions; number of ROIs: 15; ROIs: (1) L IFG orb; (2) L IFG tri; (3) L IFG op; (4) L DLPFC; (5) L insula; (6) L ATL; (7) L PTL; (8) L SMA/dACC; (9) R L IFG orb; (10) R IFG tri; (11) R insula; (12) R DLPFC; (13) R ATL; (14) perilesional tissue; (15) regions homotopic to lesions; how ROIs defined: (1-13) spheres around peaks of whole brain analysis of all patients collapsing across groups and timepoints; (14) perilesional ROIs were voxels 3-15 mm from the lesion that were located in frontal or temporal regions activated by the language contrast in controls; (15) homotopic ROIs were flipped lesions	None
Stockert et al. (2020): ROI 38	Listening to normal sentences and making a plausibility judgment (paradigm 1) or listening to normal sentences (paradigm 2) vs	LC<br>Aphasia frontal (n = 17) T3 vs T2<br>Covariate: Δ comprehension composite	UNR	UNR	ROI<br>Mix<br>NC	Behavioral data notes: no differences in proportion of expected button presses by group or time, but behavioral data pooled across conditions; number of ROIs: 15; ROIs: (1) L IFG orb; (2) L IFG tri; (3) L IFG op; (4) L DLPFC; (5) L insula; (6) L ATL; (7) L PTL; (8) L SMA/dACC; (9) R L IFG orb; (10) R IFG tri; (11) R insula; (12) R DLPFC; (13) R ATL; (14) perilesional	None

	listening to reversed speech					tissue; (15) regions homotopic to lesions; how ROIs defined: (1-13) spheres around peaks of whole brain analysis of all patients collapsing across groups and timepoints; (14) perilesional ROIs were voxels 3-15 mm from the lesion that were located in frontal or temporal regions activated by the language contrast in controls; (15) homotopic ROIs were flipped lesions	
Stockert et al. (2020): ROI 39	Listening to normal sentences and making a plausibility judgment (paradigm 1) or listening to normal sentences (paradigm 2) vs listening to reversed speech	CC Aphasia temporo-parietal T1 (n = 17) Covariate: comprehension composite	UNR	UNR	ROI Mix NC	Behavioral data notes: no differences in proportion of expected button presses by group or time, but behavioral data pooled across conditions; number of ROIs: 15; ROIs: (1) L IFG orb; (2) L IFG tri; (3) L IFG op; (4) L DLPFC; (5) L insula; (6) L ATL; (7) L PTL; (8) L SMA/dACC; (9) R L IFG orb; (10) R IFG tri; (11) R insula; (12) R DLPFC; (13) R ATL; (14) perilesional tissue; (15) regions homotopic to lesions; how ROIs defined: (1-13) spheres around peaks of whole brain analysis of all patients collapsing across groups and timepoints; (14) perilesional ROIs were voxels 3-15 mm from the lesion that were located in frontal or temporal regions activated by the language contrast in controls; (15) homotopic ROIs were flipped lesions	↑ R anterior temporal
Stockert et al. (2020): ROI 40	Listening to normal sentences and making a plausibility judgment (paradigm 1) or listening to normal sentences (paradigm 2) vs listening to reversed speech	CC Aphasia temporo-parietal T2 (n = 17) Covariate: comprehension composite	UNR	UNR	ROI Mix NC	Behavioral data notes: no differences in proportion of expected button presses by group or time, but behavioral data pooled across conditions; number of ROIs: 15; ROIs: (1) L IFG orb; (2) L IFG tri; (3) L IFG op; (4) L DLPFC; (5) L insula; (6) L ATL; (7) L PTL; (8) L SMA/dACC; (9) R L IFG orb; (10) R IFG tri; (11) R insula; (12) R DLPFC; (13) R ATL; (14) perilesional tissue; (15) regions homotopic to lesions; how ROIs defined: (1-13) spheres around peaks of whole brain analysis of all patients collapsing across groups and timepoints; (14) perilesional ROIs were voxels 3-15 mm from the lesion that were located in frontal or temporal regions activated by the language contrast in controls; (15) homotopic ROIs were flipped lesions	↑ L IFG pars opercularis ↑ L posterior STG/STS/MTG
Stockert et al. (2020): ROI 41	Listening to normal sentences and making a plausibility judgment (paradigm 1) or listening to normal sentences (paradigm 2) vs	CC Aphasia temporo-parietal T3 (n = 17) Covariate: comprehension composite	UNR	UNR	ROI Mix NC	Behavioral data notes: no differences in proportion of expected button presses by group or time, but behavioral data pooled across conditions; number of ROIs: 15; ROIs: (1) L IFG orb; (2) L IFG tri; (3) L IFG op; (4) L DLPFC; (5) L insula; (6) L ATL; (7) L PTL; (8) L SMA/dACC; (9) R L IFG orb; (10) R IFG tri; (11) R insula; (12) R	None

	listening to reversed speech					DLPFC; (13) R ATL; (14) perilesional tissue; (15) regions homotopic to lesions; how ROIs defined: (1-13) spheres around peaks of whole brain analysis of all patients collapsing across groups and timepoints; (14) perilesional ROIs were voxels 3-15 mm from the lesion that were located in frontal or temporal regions activated by the language contrast in controls; (15) homotopic ROIs were flipped lesions	
Stockert et al. (2020): ROI 42	Listening to normal sentences and making a plausibility judgment (paradigm 1) or listening to normal sentences (paradigm 2) vs listening to reversed speech	LC<br>Aphasia temporo-parietal (n = 17) T2 vs T1<br>Covariate: Δ comprehension composite	UNR	UNR	ROI<br>Mix<br>NC	Behavioral data notes: no differences in proportion of expected button presses by group or time, but behavioral data pooled across conditions; number of ROIs: 15; ROIs: (1) L IFG orb; (2) L IFG tri; (3) L IFG op; (4) L DLPFC; (5) L insula; (6) L ATL; (7) L PTL; (8) L SMA/dACC; (9) R L IFG orb; (10) R IFG tri; (11) R insula; (12) R DLPFC; (13) R ATL; (14) perilesional tissue; (15) regions homotopic to lesions; how ROIs defined: (1-13) spheres around peaks of whole brain analysis of all patients collapsing across groups and timepoints; (14) perilesional ROIs were voxels 3-15 mm from the lesion that were located in frontal or temporal regions activated by the language contrast in controls; (15) homotopic ROIs were flipped lesions	↑ L insula
Stockert et al. (2020): ROI 43	Listening to normal sentences and making a plausibility judgment (paradigm 1) or listening to normal sentences (paradigm 2) vs listening to reversed speech	LC<br>Aphasia temporo-parietal (n = 17) T3 vs T1<br>Covariate: Δ comprehension composite	UNR	UNR	ROI<br>Mix<br>NC	Behavioral data notes: no differences in proportion of expected button presses by group or time, but behavioral data pooled across conditions; number of ROIs: 15; ROIs: (1) L IFG orb; (2) L IFG tri; (3) L IFG op; (4) L DLPFC; (5) L insula; (6) L ATL; (7) L PTL; (8) L SMA/dACC; (9) R L IFG orb; (10) R IFG tri; (11) R insula; (12) R DLPFC; (13) R ATL; (14) perilesional tissue; (15) regions homotopic to lesions; how ROIs defined: (1-13) spheres around peaks of whole brain analysis of all patients collapsing across groups and timepoints; (14) perilesional ROIs were voxels 3-15 mm from the lesion that were located in frontal or temporal regions activated by the language contrast in controls; (15) homotopic ROIs were flipped lesions	None
Stockert et al. (2020): ROI 44	Listening to normal sentences and making a plausibility judgment (paradigm 1) or listening to normal sentences	LC<br>Aphasia temporo-parietal (n = 17) T3 vs T2<br>Covariate: Δ comprehension composite	UNR	UNR	ROI<br>Mix<br>NC	Behavioral data notes: no differences in proportion of expected button presses by group or time, but behavioral data pooled across conditions; number of ROIs: 15; ROIs: (1) L IFG orb; (2) L IFG tri; (3) L IFG op; (4) L DLPFC; (5) L insula; (6) L ATL; (7) L PTL; (8) L SMA/dACC; (9) R L IFG orb;	None

	(paradigm 2) vs listening to reversed speech					(10) R IFG tri; (11) R insula; (12) R DLPFC; (13) R ATL; (14) perilesional tissue; (15) regions homotopic to lesions; how ROIs defined: (1-13) spheres around peaks of whole brain analysis of all patients collapsing across groups and timepoints; (14) perilesional ROIs were voxels 3-15 mm from the lesion that were located in frontal or temporal regions activated by the language contrast in controls; (15) homotopic ROIs were flipped lesions	
Stockert et al. (2020): ROI 45	Listening to normal sentences and making a plausibility judgment (paradigm 1) or listening to normal sentences (paradigm 2) vs listening to reversed speech	CC<br>Aphasia T1<br>Covariate: lesion volume	UNR	UNR	ROI<br>Mix<br>NC	Behavioral data notes: no differences in proportion of expected button presses by group or time, but behavioral data pooled across conditions; number of ROIs: 15; ROIs: (1) L IFG orb; (2) L IFG tri; (3) L IFG op; (4) L DLPFC; (5) L insula; (6) L ATL; (7) L PTL; (8) L SMA/dACC; (9) R L IFG orb; (10) R IFG tri; (11) R insula; (12) R DLPFC; (13) R ATL; (14) perilesional tissue; (15) regions homotopic to lesions; how ROIs defined: (1-13) spheres around peaks of whole brain analysis of all patients collapsing across groups and timepoints; (14) perilesional ROIs were voxels 3-15 mm from the lesion that were located in frontal or temporal regions activated by the language contrast in controls; (15) homotopic ROIs were flipped lesions	↓ L IFG pars triangularis notes: lesion volume negatively correlated with activation
Stockert et al. (2020): ROI 46	Listening to normal sentences and making a plausibility judgment (paradigm 1) or listening to normal sentences (paradigm 2) vs listening to reversed speech	CC<br>Aphasia T2<br>Covariate: lesion volume	UNR	UNR	ROI<br>Mix<br>NC	Behavioral data notes: no differences in proportion of expected button presses by group or time, but behavioral data pooled across conditions; number of ROIs: 15; ROIs: (1) L IFG orb; (2) L IFG tri; (3) L IFG op; (4) L DLPFC; (5) L insula; (6) L ATL; (7) L PTL; (8) L SMA/dACC; (9) R L IFG orb; (10) R IFG tri; (11) R insula; (12) R DLPFC; (13) R ATL; (14) perilesional tissue; (15) regions homotopic to lesions; how ROIs defined: (1-13) spheres around peaks of whole brain analysis of all patients collapsing across groups and timepoints; (14) perilesional ROIs were voxels 3-15 mm from the lesion that were located in frontal or temporal regions activated by the language contrast in controls; (15) homotopic ROIs were flipped lesions	None
Stockert et al. (2020): ROI 47	Listening to normal sentences and making a plausibility judgment (paradigm 1) or listening to normal	CC<br>Aphasia T3<br>Covariate: lesion volume	UNR	UNR	ROI<br>Mix<br>NC	Behavioral data notes: no differences in proportion of expected button presses by group or time, but behavioral data pooled across conditions; number of ROIs: 15; ROIs: (1) L IFG orb; (2) L IFG tri; (3) L IFG op; (4) L DLPFC; (5) L insula; (6) L ATL; (7) L	None

	sentences (paradigm 2) vs listening to reversed speech					PTL; (8) L SMA/dACC; (9) R L IFG orb; (10) R IFG tri; (11) R insula; (12) R DLPFC; (13) R ATL; (14) perilesional tissue; (15) regions homotopic to lesions; how ROIs defined: (1-13) spheres around peaks of whole brain analysis of all patients collapsing across groups and timepoints; (14) perilesional ROIs were voxels 3-15 mm from the lesion that were located in frontal or temporal regions activated by the language contrast in controls; (15) homotopic ROIs were flipped lesions	
Stockert et al. (2020): ROI 48	Listening to normal sentences and making a plausibility judgment (paradigm 1) or listening to normal sentences (paradigm 2) vs listening to reversed speech	LC<br>Aphasia T2 vs T1<br>Covariate: lesion volume	UNR	UNR	ROI<br>Mix<br>NC	Behavioral data notes: no differences in proportion of expected button presses by group or time, but behavioral data pooled across conditions; number of ROIs: 15; ROIs: (1) L IFG orb; (2) L IFG tri; (3) L IFG op; (4) L DLPFC; (5) L insula; (6) L ATL; (7) L PTL; (8) L SMA/dACC; (9) R L IFG orb; (10) R IFG tri; (11) R insula; (12) R DLPFC; (13) R ATL; (14) perilesional tissue; (15) regions homotopic to lesions; how ROIs defined: (1-13) spheres around peaks of whole brain analysis of all patients collapsing across groups and timepoints; (14) perilesional ROIs were voxels 3-15 mm from the lesion that were located in frontal or temporal regions activated by the language contrast in controls; (15) homotopic ROIs were flipped lesions	None
Stockert et al. (2020): ROI 49	Listening to normal sentences and making a plausibility judgment (paradigm 1) or listening to normal sentences (paradigm 2) vs listening to reversed speech	LC<br>Aphasia T3 vs T1<br>Covariate: lesion volume	UNR	UNR	ROI<br>Mix<br>NC	Behavioral data notes: no differences in proportion of expected button presses by group or time, but behavioral data pooled across conditions; number of ROIs: 15; ROIs: (1) L IFG orb; (2) L IFG tri; (3) L IFG op; (4) L DLPFC; (5) L insula; (6) L ATL; (7) L PTL; (8) L SMA/dACC; (9) R L IFG orb; (10) R IFG tri; (11) R insula; (12) R DLPFC; (13) R ATL; (14) perilesional tissue; (15) regions homotopic to lesions; how ROIs defined: (1-13) spheres around peaks of whole brain analysis of all patients collapsing across groups and timepoints; (14) perilesional ROIs were voxels 3-15 mm from the lesion that were located in frontal or temporal regions activated by the language contrast in controls; (15) homotopic ROIs were flipped lesions	None
Stockert et al. (2020): ROI 50	Listening to normal sentences and making a plausibility judgment (paradigm 1) or	LC<br>Aphasia T3 vs T2<br>Covariate: lesion volume	UNR	UNR	ROI<br>Mix<br>NC	Behavioral data notes: no differences in proportion of expected button presses by group or time, but behavioral data pooled across conditions; number of ROIs: 15; ROIs: (1) L IFG orb; (2) L IFG tri; (3) L IFG op;	None

	listening to normal sentences (paradigm 2) vs listening to reversed speech					(4) L DLPFC; (5) L insula; (6) L ATL; (7) L PTL; (8) L SMA/dACC; (9) R L IFG orb; (10) R IFG tri; (11) R insula; (12) R DLPFC; (13) R ATL; (14) perilesional tissue; (15) regions homotopic to lesions; how ROIs defined: (1-13) spheres around peaks of whole brain analysis of all patients collapsing across groups and timepoints; (14) perilesional ROIs were voxels 3-15 mm from the lesion that were located in frontal or temporal regions activated by the language contrast in controls; (15) homotopic ROIs were flipped lesions	
Stockert et al. (2020): Cplx 1	Listening to normal sentences and making a plausibility judgment (paradigm 1) or listening to normal sentences (paradigm 2) vs listening to reversed speech	CAA Aphasia frontal T1 (n = 17) vs temporo-parietal T1 (n = 17)	UNR	UNR	Cplx	Behavioral data notes: no differences in proportion of expected button presses by group or time, but behavioral data pooled across conditions; Correlations between activity in 15 ROIs and LRScomp were compared between patients with frontal and temporal lesions, using interaction terms as well as the Fisher r-to-z transformation. There was no correction for multiple comparisons across the 15 ROIs.	Other: Correlations were higher in the temporal group in the R ATL.
Stockert et al. (2020): Cplx 2	Listening to normal sentences and making a plausibility judgment (paradigm 1) or listening to normal sentences (paradigm 2) vs listening to reversed speech	CAA Aphasia frontal T2 (n = 17) vs temporo-parietal T2 (n = 17)	UNR	UNR	Cplx	Behavioral data notes: no differences in proportion of expected button presses by group or time, but behavioral data pooled across conditions; Correlations between activity in 15 ROIs and LRScomp were compared between patients with frontal and temporal lesions, using interaction terms as well as the Fisher r-to-z transformation. There was no correction for multiple comparisons across the 15 ROIs.	Other: Correlations were higher in the temporal group in L posterior temporal cortex and L IFG op.
Stockert et al. (2020): Cplx 3	Listening to normal sentences and making a plausibility judgment (paradigm 1) or listening to normal sentences (paradigm 2) vs listening to reversed speech	CAA Aphasia frontal T3 (n = 17) vs temporo-parietal T3 (n = 17)	UNR	UNR	Cplx	Behavioral data notes: no differences in proportion of expected button presses by group or time, but behavioral data pooled across conditions; Correlations between activity in 15 ROIs and LRScomp were compared between patients with frontal and temporal lesions, using interaction terms. There was no correction for multiple comparisons across the 15 ROIs.	Other: Correlations were different between groups in the R ATL, but the correlation is not reported as significant in the temporo-parietal group alone.
Stockert et al. (2020): Cplx 4	Listening to normal sentences and making a plausibility judgment (paradigm 1) or listening to normal sentences (paradigm 2) vs listening to reversed speech	LAA (Aphasia frontal (n = 17) T2 vs T1) vs (aphasia temporo-parietal (n = 17) T2 vs T1)	UNR	UNR	Cplx	Behavioral data notes: no differences in proportion of expected button presses by group or time, but behavioral data pooled across conditions; Correlations between changes in activity in 15 ROIs and changes in LRScomp were compared between patients with frontal and temporal lesions, using interaction terms as well as the Fisher r-to-z transformation. There was no	Other: In the L insula, the temporo-parietal group showed a stronger correlation than the frontal group between changes in activation and changes in LRScomp.

						correction for multiple comparisons across the 15 ROIs.	
Stockert et al. (2020): Cplx 5	Listening to normal sentences and making a plausibility judgment (paradigm 1) or listening to normal sentences (paradigm 2) vs listening to reversed speech	LAA (Aphasia frontal (n = 17) T3 vs T1) vs (temporo-parietal (n = 17) T3 vs T1)	UNR	UNR	Cplx	Behavioral data notes: no differences in proportion of expected button presses by group or time, but behavioral data pooled across conditions; Correlations between changes in activity in 15 ROIs and changes in LRScomp were compared between patients with frontal and temporal lesions, using interaction terms as well as the Fisher r-to-z transformation. There was no correction for multiple comparisons across the 15 ROIs.	Other: In the L insula, the temporo-parietal group showed a stronger correlation than the frontal group between changes in activation and changes in LRScomp.
Stockert et al. (2020): Cplx 6	Listening to normal sentences and making a plausibility judgment (paradigm 1) or listening to normal sentences (paradigm 2) vs listening to reversed speech	LAA (Aphasia frontal (n = 17) T3 vs T2) vs (temporo-parietal (n = 17) T3 vs T2)	UNR	UNR	Cplx	Behavioral data notes: no differences in proportion of expected button presses by group or time, but behavioral data pooled across conditions; Correlations between changes in activity in 15 ROIs and changes in LRScomp were compared between patients with frontal and temporal lesions, using interaction terms as well as the Fisher r-to-z transformation. There was no correction for multiple comparisons across the 15 ROIs.	None
Stockert et al. (2020): Cplx 7	Listening to normal sentences and making a plausibility judgment (paradigm 1) or listening to normal sentences (paradigm 2) vs listening to reversed speech	CAA Aphasia frontal T1 (n = 17) vs temporo-parietal T1 (n = 17)	UNR	UNR	Cplx	Behavioral data notes: no differences in proportion of expected button presses by group or time, but behavioral data pooled across conditions; Correlations between activity in 15 ROIs and lesion extent were compared between patients with frontal and temporal lesions. There was no correction for multiple comparisons across the 15 ROIs.	None
Stockert et al. (2020): Cplx 8	Listening to normal sentences and making a plausibility judgment (paradigm 1) or listening to normal sentences (paradigm 2) vs listening to reversed speech	CAA Aphasia frontal T2 (n = 17) vs temporo-parietal T2 (n = 17)	UNR	UNR	Cplx	Behavioral data notes: no differences in proportion of expected button presses by group or time, but behavioral data pooled across conditions; Correlations between activity in 15 ROIs and lesion extent were compared between patients with frontal and temporal lesions. There was no correction for multiple comparisons across the 15 ROIs.	None
Stockert et al. (2020): Cplx 9	Listening to normal sentences and making a plausibility judgment (paradigm 1) or listening to normal sentences (paradigm 2) vs listening to reversed speech	CAA Aphasia frontal T3 (n = 17) vs temporo-parietal T3 (n = 17)	UNR	UNR	Cplx	Behavioral data notes: no differences in proportion of expected button presses by group or time, but behavioral data pooled across conditions; Correlations between activity in 15 ROIs and lesion extent were compared between patients with frontal and temporal lesions. There was no correction for multiple comparisons across the 15 ROIs.	None

Stockert et al. (2020): Cplx 10	Listening to normal sentences and making a plausibility judgment (paradigm 1) or listening to normal sentences (paradigm 2) vs listening to reversed speech	LAA (Aphasia frontal (n = 17) T2 vs T1) vs (temporo-parietal (n = 17) T2 vs T1)	UNR	UNR	Cplx	Behavioral data notes: no differences in proportion of expected button presses by group or time, but behavioral data pooled across conditions; Correlations between changes in activity in 15 ROIs and lesion extent were compared between patients with frontal and temporal lesions. There was no correction for multiple comparisons across the 15 ROIs.	None
Stockert et al. (2020): Cplx 11	Listening to normal sentences and making a plausibility judgment (paradigm 1) or listening to normal sentences (paradigm 2) vs listening to reversed speech	LAA (Aphasia frontal (n = 17) T3 vs T1) vs (temporo-parietal (n = 17) T3 vs T1)	UNR	UNR	Cplx	Behavioral data notes: no differences in proportion of expected button presses by group or time, but behavioral data pooled across conditions; Correlations between changes in activity in 15 ROIs and lesion extent were compared between patients with frontal and temporal lesions. There was no correction for multiple comparisons across the 15 ROIs.	None
Stockert et al. (2020): Cplx 12	Listening to normal sentences and making a plausibility judgment (paradigm 1) or listening to normal sentences (paradigm 2) vs listening to reversed speech	LAA (Aphasia frontal (n = 17) T3 vs T2) vs (temporo-parietal (n = 17) T3 vs T2)	UNR	UNR	Cplx	Behavioral data notes: no differences in proportion of expected button presses by group or time, but behavioral data pooled across conditions; Correlations between changes in activity in 15 ROIs and lesion extent were compared between patients with frontal and temporal lesions. There was no correction for multiple comparisons across the 15 ROIs.	None
Stockert et al. (2020): Cplx 13	Listening to normal sentences and making a plausibility judgment (paradigm 1) or listening to normal sentences (paradigm 2) vs listening to reversed speech	CC Aphasia T1 Covariate: interaction of comprehension composite by lesion size	UNR	UNR	Cplx	Behavioral data notes: no differences in proportion of expected button presses by group or time, but behavioral data pooled across conditions; To investigate why some activation-behavior relationships did not remain significant when lesion extent was included as a covariate, models were constructed looking at the relationship between activation and behavior in patients with larger and smaller lesions.	Other: The three regions where this applied at T1, namely perilesional cortex, L IFG op, and L IFG orb, all showed positive correlations between activation and LRScomp in patients with larger lesions, but no correlations in patients with smaller lesions.
Stockert et al. (2020): Cplx 14	Listening to normal sentences and making a plausibility judgment (paradigm 1) or listening to normal sentences (paradigm 2) vs listening to reversed speech	LC Aphasia T2 vs T1 Covariate: interaction of Δ comprehension composite by lesion size	UNR	UNR	Cplx	Behavioral data notes: no differences in proportion of expected button presses by group or time, but behavioral data pooled across conditions; To investigate why some activation-behavior relationships did not remain significant when lesion extent was included as a covariate, models were constructed looking at the relationship between activation and behavior in patients with larger and smaller lesions.	Other: This applied to the R DLPFC in the T2 vs T1 analysis. This region showed a positive correlation between activation and LRScomp in patients with larger lesions, but no correlation in

	patients with smaller lesions.

Second level contrast = Which of the 8 relevant classes of analyses is this? Which group or groups of participants are included? If there is a covariate, what is it?; Acc = Is accuracy matched across the second level contrast?; RT = Is reaction time matched across the second level contrast?; Stats = Does the analysis involve voxelwise statistics, region(s) of interest (ROI), or something else (complex)? If voxelwise, how are multiple comparisons across voxels accounted for? If ROI, were the ROI(s) anatomical, functional, laterality indices, mixed, or something else? If there was more than one ROI, how were the ROIs corrected for multiple comparions?; Yellow underline = minor limitation; Orange underline = moderate limitation; Red underline = major limitation; CAC = Cross-sectional aphasia vs control; CAA = Cross-sectional between two groups with aphasia; CC = Cross-sectional correlation with language or other measure; CB = Cross-sectional performance-defined conditions; LA = Longitudinal change in aphasia; LAC = Longitudinal aphasia vs control; LAA = Longitudinal between two groups with aphasia; LC = Longitudinal correlation with language or other measure; Y = Yes, matched; YCT = Yes, correct trials only; NBD = No, by design; NAM = No, but attempt made; N = No, different; C = Accuracy or RT is covariate; UNT = Unknown, no test; AS = Appear similar; AM = Appear mismatched; UNR = Unknown, not reported; NANB = N/A, no behavioral measure; NANT = N/A, no timeable task; Vox = Voxelwise; VP = Voxelwise correction based on permutation testing; VFWE = Voxelwise FWE correction; C+ = Clusterwise correction with with GRFT and stringent voxelwise p; VFWC = Voxelwise FWE correction and additional arbitrary cluster correction; C- = Clusterwise correction with with GRFT and lenient voxelwise p; CCS = Clusterwise correction based on 3dClustSim; SVC = Small volume correction; CCTB = Clusterwise correction based on cluster_threshold_beta; CA = Clusterwise correction based on arbitrary cluster extent; NC = No correction; NDC = No direct comparison; M** = Mixed** (major limitation); U = Unclear or not stated; ROI = Region(s) of interest; Anat = Anatomical; Func = Functional; Oth = Other; LI = Laterality indi(ces); Mix = Mixed; FWE = Familywise error (FWE); FDR = False discovery rate (FDR); NC = No correction; One = One only; NDC = No direct comparison; Cplx = Complex.

Supplementary Table S11. Cross-sectional aphasia compared to control: Methodologically robust analyses

Analysis	First level contrast	Second level contrast	Matched for		Stats	Notes	Findings
			Acc	RT			
Leff et al. (2002): ROI 1	Higher word rates vs lower word rates	CAC Aphasia with pSTS damage (n = 6) vs control (n = 8)	NANB	NANT	ROI Func One	Number of ROIs: 1; ROI: R pSTS; how ROI defined: the peak voxel for the contrast in the R pSTS from each subject's individual analysis, but the search region is not stated; the controls and patients without pSTS damage were combined, however it is stated in the caption to Figure 2 that the patients with pSTS damage were significantly different to both	↑ R posterior STS
Blank et al. (2003): Vox 1	Propositional speech production vs rest	CAC Aphasia with IFG POp damage (n = 7) vs control	N	NANT	Vox SVC	Behavioral data notes: word rates not reported, but offline speech sample differed; search volume: voxels spared in all patients; software: SPM99; voxelwise p: FWE p < .05 with SVC in R pars opercularis	↑ R IFG pars opercularis notes: no voxels survived FWE correction without SVC
Blank et al. (2003): Vox 2	Propositional speech production vs rest	CAC Aphasia without IFG POp damage (n = 7) vs control	N	NANT	Vox SVC	Behavioral data notes: word rates not reported, but offline speech sample differed; search volume: voxels spared in all patients; software: SPM99; voxelwise p: FWE p < .05 with SVC in R pars opercularis	↑ R IFG pars opercularis
Blank et al. (2003): Vox 4	Propositional speech production vs counting	CAC Aphasia with IFG POp damage (n = 7) vs control	N	NANT	Vox SVC	Behavioral data notes: word rates not reported, but offline speech sample differed; search volume: voxels spared in all patients; software: SPM99; voxelwise p: FWE p < .05 with SVC in R pars opercularis	None
Blank et al. (2003): Vox 5	Propositional speech production vs counting	CAC Aphasia without IFG POp damage (n = 7) vs control	N	NANT	Vox SVC	Behavioral data notes: word rates not reported, but offline speech sample differed; search volume: voxels spared in all patients; software: SPM99; voxelwise p: FWE p < .05 with SVC in R pars opercularis	None
Sharp et al. (2004): Vox 1	Semantic decision vs syllable count decision	CAC Aphasia vs control (clear speech)	AM	Y	Vox SVC	Behavioral data notes: interaction of group by task not reported for accuracy; search volume: whole brain; software: SPM99; voxelwise p: FWE p < .05 with SVC in fusiform gyri, temporal poles, L IFG, L orbitofrontal and L SFG	↓ L posterior inferior temporal gyrus/fusiform gyrus
Sharp et al. (2004): ROI 1	Semantic decision vs syllable count decision	CAC Aphasia vs control (clear speech)	AM	Y	ROI Anat One	Behavioral data notes: interaction of group by task not reported for accuracy; number of ROIs: 1; ROI: L fusiform gyrus; how ROI defined: probabilistic brain atlas	↓ L posterior inferior temporal gyrus/fusiform gyrus
Sharp et al. (2004): ROI 2	Semantic decision vs syllable count decision	CAC Aphasia vs control (noise vocoded)	NAM	Y	ROI Anat One	Behavioral data notes: patients were more accurate on semantic decisions than syllable decisions, whereas controls were less accurate on noise vocoded semantic decisions than clear syllable decisions (which were the baseline for this analysis); number of ROIs: 1; ROI: L fusiform gyrus; how ROI defined: probabilistic brain atlas	None notes: this analysis suggests that the difference between groups in the L fusiform gyrus disappears when the controls perform a

							semantic task that is similarly challenging
Zahn et al. (2004): ROI 1	Semantic decision vs phonetic decision and lexical decision (conjunction)	CAC Aphasia vs control	UNT	UNR	ROI LI One	Behavioral data notes: relative performance on language and control tasks unclear; number of ROIs: 1; ROI: language network LI; conjunction analyses not clearly described; in two patients, a different conjunction was used (lexical decision vs phonetic decision & semantic decision vs phonetic decision)	None notes: LI > 0 in 12 out of 14 controls and 5 out of 7 patients; no significant difference
Crinion & Price (2005): Vox 1	Listening to narrative speech vs listening to reversed speech	CAC Aphasia without temporal lobe damage (n = 9) vs control	NANB	NANT	Vox VFWC	Search volume: whole brain; software: SPM2; voxelwise p: FWE p < .05; cluster extent cutoff: 5 voxels (size not stated)	↓ L dorsal precentral ↓ R somato-motor
Crinion & Price (2005): Vox 2	Listening to narrative speech vs listening to reversed speech	CAC Aphasia with temporal lobe damage (n = 8) vs control	NANB	NANT	Vox VFWC	Search volume: whole brain; software: SPM2; voxelwise p: FWE p < .05; cluster extent cutoff: 5 voxels (size not stated)	↓ L posterior STS ↓ L mid temporal
Crinion & Price (2005): Cplx 2	Listening to narrative speech vs listening to reversed speech	CAC Aphasia without temporal damage (n = 9) vs control	NANB	NANT	Cplx	Correlations were computed between activity in each voxel, and post-scan story recall, and were compared between patients without temporal damage and controls, in regions with a main effect of story comprehension. The threshold was p < 0.05 corrected, plus a minimum cluster size of 5 voxels.	None
Crinion & Price (2005): Cplx 3	Listening to narrative speech vs listening to reversed speech	CAC Aphasia with temporal damage (n = 8) vs control	NANB	NANT	Cplx	Correlations were computed between activity in each voxel, and post-scan story recall, and were compared between patients with temporal damage and controls, in regions with a main effect of story comprehension. The threshold was p < 0.05 corrected, plus a minimum cluster size of 5 voxels.	None
Crinion et al. (2006): Vox 1	Listening to narrative speech vs listening to reversed speech	CAC Aphasia vs control	NANB	NANT	Vox VFWE	Search volume: voxels spared in all patients; software: SPM99; voxelwise p: FWE p < .05	None
Crinion et al. (2006): Vox 2	Listening to narrative speech vs listening to reversed speech	CAC Aphasia without temporal lobe damage (n = 6) vs control	NANB	NANT	Vox VFWE	Search volume: voxels spared in all included patients; software: SPM99; voxelwise p: FWE p < .05	None
Crinion et al. (2006): Vox 3	Listening to narrative speech vs listening to reversed speech	CAC Aphasia with temporal lobe damage (n = 18) vs control	NANB	NANT	Vox VFWE	Search volume: voxels spared in all included patients; software: SPM99; voxelwise p: FWE p < .05	None
Warren et al. (2009): ROI 1	Listening to narrative speech vs listening to reversed speech	CAC Aphasia vs control	NANB	NANT	ROI Anat NC	Number of ROIs: 6; ROIs: (1) L anterior superior temporal cortex; (2) L basal temporal language area; (3) L IFG pars triangularis; (4-6) homotopic counterparts; how ROIs defined: ROIs were defined anatomically in regions that were functionally connected with	None notes: L IFG pars triangularis almost reached significance (p = .053) for more

						L anterior superior temporal cortex in controls (1-4) or homotopic to these (5-6); somewhat circular because ROIs were defined only in regions where controls showed significant connectivity (even though ROIs were anatomical)	activation in patients
Warren et al. (2009): ROI 9	Listening to narrative speech vs listening to reversed speech	CAC Aphasia with positive anterior temporal interconnectivity (n = 8) vs control	NANB	NANT	ROI Anat NC	Number of ROIs: 6; ROIs: (1) L anterior superior temporal cortex; (2) L basal temporal language area; (3) L IFG pars triangularis; (4-6) homotopic counterparts; how ROIs defined: ROIs were defined anatomically in regions that were functionally connected with L anterior superior temporal cortex in controls (1-4) or homotopic to these (5-6); somewhat circular because ROIs were defined only in regions where controls showed significant connectivity (even though ROIs were anatomical); excluded 3 patients with L IFG damage	↑ L IFG pars triangularis
Warren et al. (2009): ROI 10	Listening to narrative speech vs listening to reversed speech	CAC Aphasia with negative anterior temporal interconnectivity (n = 8) vs control	NANB	NANT	ROI Anat NC	Number of ROIs: 6; ROIs: (1) L anterior superior temporal cortex; (2) L basal temporal language area; (3) L IFG pars triangularis; (4-6) homotopic counterparts; how ROIs defined: ROIs were defined anatomically in regions that were functionally connected with L anterior superior temporal cortex in controls (1-4) or homotopic to these (5-6); somewhat circular because ROIs were defined only in regions where controls showed significant connectivity (even though ROIs were anatomical); excluded 1 patient with L IFG damage	None
Fridriksson et al. (2010): Vox 2	Picture naming (correct trials) vs viewing abstract pictures	CAC Aphasia vs control	YCT	UNR	Vox C-	Search volume: whole brain; software: FSL 4.1; voxelwise p: ~.02 (z > 2); cluster extent cutoff: based on GRFT	None
van Oers et al. (2010): ROI 3	Verb generation vs rest	CAC Aphasia vs control	UNR	UNR	ROI Mix NC	Number of ROIs: 7; ROIs: (1) L anterior language region (IFG); (2) L posterior language region (AG, SMG, STG, MTG); (3) R anterior language region (IFG); (4) R posterior language region (AG, SMG, STG, MTG); (5) frontal LI; (6) temporal LI; (7) whole network LI; how ROIs defined: WFU pickatlas	↓ L IFG<br>↓ LI (language network)<br>↓ LI (frontal)
Allendorfer et al. (2012): ROI 2	Verb generation (overt, event-related) vs noun repetition (event-related)	CAC Aphasia vs control	N	UNR	ROI LI NC	Behavioral data notes: patients less accurate and produced less responses on both conditions, but the difference between groups was greater for verb generation; number of ROIs: 2; ROIs: (1) frontal LI; (2) temporal LI	↓ LI (frontal)
Szaflarski et al. (2014): ROI 1	Verb generation vs finger tapping	CAC Aphasia vs control	UNR	UNR	ROI LI NC	Number of ROIs: 3; ROIs: (1) frontal LI; (2) temporal LI; (3) language network LI	↓ LI (language network)<br>↓ LI (frontal) notes: temporal LI was also marginally

							significantly reduced (p = .08)
Griffis, Nenert, Allendorfer, & Szaflarski (2017): Cplx 1	Semantic decision vs tone decision	CAC Aphasia vs control	N	UNR	Cplx	Behavioral data notes: semantic decision accuracy not matched, but tone decision accuracy not reported; Multimodal canonical correlation analysis (mCCA) and joint ICA were used to identify 3 joint ICs (structural/functional) that were differently represented in the patient and control groups. Although there was no correction for multiple comparisons when the functional maps were thresholded, the maps for the three networks each appeared to relate to coherent parts of the semantic network.	Other: The first joint IC comprised preservation of tissue in L posterior temporo-parietal region, activity in the L AG and bilateral midline components of the canonical semantic network, and reduced activity in R frontal, temporal and parietal regions. The second joint IC comprised preservation of tissue in the the L basal ganglia/insula region, and activity predominantly in the IFG pars orbitalis bilaterally. The third joint IC comprised preservation of the L IFG and activity in the L IFG and DLPFC along with bilateral midline regions. The first joint IC was considered to provide more robust evidence for structure-function relationships than the other two, because it was the only one where individual structural and functional mixing coefficients remained correlated even when lesion volume was included as a covariate.

Second level contrast = Which of the 8 relevant classes of analyses is this? Which group or groups of participants are included? If there is a covariate, what is it?; Acc = Is accuracy matched across the second level contrast?; RT = Is reaction time matched across the second level contrast?; Stats = Does the analysis involve voxelwise statistics, region(s) of interest (ROI), or something else (complex)? If voxelwise, how are multiple comparisons across

voxels accounted for? If ROI, were the ROI(s) anatomical, functional, laterality indices, mixed, or something else? If there was more than one ROI, how were the ROIs corrected for multiple comparions?; Yellow underline = minor limitation; Orange underline = moderate limitation; Red underline = major limitation; CAC = Cross-sectional aphasia vs control; Y = Yes, matched; YCT = Yes, correct trials only; NAM = No, but attempt made; N = No, different; UNT = Unknown, no test; AM = Appear mismatched; UNR = Unknown, not reported; NANB = N/A, no behavioral measure; NANT = N/A, no timeable task; Vox = Voxelwise; VFWE = Voxelwise FWE correction; VFWC = Voxelwise FWE correction and additional arbitrary cluster correction; C- = Clusterwise correction with with GRFT and lenient voxelwise p; SVC = Small volume correction; ROI = Region(s) of interest; Anat = Anatomical; Func = Functional; LI = Laterality indi(ces); Mix = Mixed; NC = No correction; One = One only; Cplx = Complex.

Supplementary Table S12. Cross-sectional correlation with language or other measure: Methodologically robust analyses

Analysis	First level contrast	Second level contrast	Matched for		Stats	Notes	Findings
			Acc	RT			
Blank et al. (2003): ROI 1	Propositional speech production vs rest	CC<br>Aphasia with IFG POp damage (n = 7)<br>Covariate: speech rate during scan	UNR	NANT	ROI Func One	Number of ROIs: 1; ROI: R IFG pars opercularis; how ROI defined: defined by flipping L IFG pars opercularis activation in controls	None
Blank et al. (2003): ROI 2	Propositional speech production vs rest	CC<br>Aphasia without IFG POp damage (n = 7)<br>Covariate: speech rate during scan	UNR	NANT	ROI Func One	Number of ROIs: 1; ROI: R IFG pars opercularis; how ROI defined: defined by flipping L IFG pars opercularis activation in controls	None
Blank et al. (2003): ROI 3	Propositional speech production vs rest	CC<br>Aphasia with IFG POp damage (n = 7)<br>Covariate: four different QPA measures	UNR	NANT	ROI Func One	Number of ROIs: 1; ROI: R IFG pars opercularis; how ROI defined: defined by flipping L IFG pars opercularis activation in controls	None
Crinion & Price (2005): Vox 4	Listening to narrative speech vs listening to reversed speech	CC<br>Aphasia without temporal lobe damage (n = 9)<br>Covariate: sentence comprehension (CAT)	NANB	NANT	Vox VFWC	Search volume: whole brain; software: SPM2; voxelwise p: FWE p < .05; cluster extent cutoff: 5 voxels (size not stated); conjunction with main effect of story comprehension (details hard to follow); this was a multiple regression also involving patients with temporal lobe damage	↑ L posterior STS<br>↑ R mid temporal<br>notes: patients with better sentence comprehension had more activation in the L posterior STS and R mid STS
Crinion & Price (2005): Vox 5	Listening to narrative speech vs listening to reversed speech	CC<br>Aphasia with temporal lobe damage (n = 8)<br>Covariate: sentence comprehension (CAT)	NANB	NANT	Vox VFWC	Search volume: whole brain; software: SPM2; voxelwise p: FWE p < .05; cluster extent cutoff: 5 voxels (size not stated); conjunction with main effect of story comprehension (details hard to follow); this was a multiple regression also involving patients without temporal lobe damage	↑ R mid temporal<br>notes: patients with better sentence comprehension had more activation in the R mid STS
Crinion et al. (2006): ROI 1	Listening to narrative speech vs listening to reversed speech	CC<br>Aphasia with no temporal damage (excluding 1 with missing behavioral data and 1 outlier) or posterior temporal damage sparing anterior temporal cortex (n = 13)<br>Covariate: auditory sentence comprehension (CAT)	NANB	NANT	ROI Func One	Number of ROIs: 1; ROI: L ATL; how ROI defined: activation in the control group; same result obtained with or without excluding one outlier; two other ROIs are described in the methods, but never used in any analyses	↑ L anterior temporal<br>notes: more activity in patients with better auditory sentence comprehension
Crinion et al. (2006): ROI 2	Listening to narrative speech vs listening to reversed speech	CC<br>Aphasia with no temporal damage (excluding 1 with missing behavioral data and 1 outlier) or posterior temporal	NANB	NANT	ROI Func One	Number of ROIs: 1; ROI: L ATL; how ROI defined: activation in the control group; two other ROIs are described in the methods, but never used in any analyses	None

		damage sparing anterior temporal cortex (n = 13)<br>Covariate: time post onset					
Crinion et al. (2006): ROI 5	Listening to narrative speech vs listening to reversed speech	CC<br>Aphasia with no temporal damage (excluding 1 with missing behavioral data and 1 outlier) or posterior temporal damage sparing anterior temporal cortex (n = 13)<br>Covariate: auditory single word comprehension (CAT)	NANB	NANT	ROI<br>Func<br>One	Number of ROIs: 1; ROI: L ATL; how ROI defined: activation in the control group; two other ROIs are described in the methods, but never used in any analyses	None<br>notes: r = 0.39; p > 0.1; seems to be a clear trend so lack of significance may reflect only lack of power
Warren et al. (2009): ROI 2	Listening to narrative speech vs listening to reversed speech	CC<br>Aphasia<br>Covariate: auditory sentence comprehension	NANB	NANT	ROI<br>Anat<br>NC	Number of ROIs: 6; ROIs: (1) L anterior superior temporal cortex; (2) L basal temporal language area; (3) L IFG pars triangularis; (4-6) homotopic counterparts; how ROIs defined: ROIs were defined anatomically in regions that were functionally connected with L anterior superior temporal cortex in controls (1-4) or homotopic to these (5-6)	↑ L anterior temporal
Warren et al. (2009): ROI 3	Listening to narrative speech vs listening to reversed speech	CC<br>Aphasia<br>Covariate: written sentence comprehension	NANB	NANT	ROI<br>Anat<br>NC	Number of ROIs: 6; ROIs: (1) L anterior superior temporal cortex; (2) L basal temporal language area; (3) L IFG pars triangularis; (4-6) homotopic counterparts; how ROIs defined: ROIs were defined anatomically in regions that were functionally connected with L anterior superior temporal cortex in controls (1-4) or homotopic to these (5-6)	None
Warren et al. (2009): ROI 4	Listening to narrative speech vs listening to reversed speech	CC<br>Aphasia<br>Covariate: auditory single word comprehension	NANB	NANT	ROI<br>Anat<br>NC	Number of ROIs: 6; ROIs: (1) L anterior superior temporal cortex; (2) L basal temporal language area; (3) L IFG pars triangularis; (4-6) homotopic counterparts; how ROIs defined: ROIs were defined anatomically in regions that were functionally connected with L anterior superior temporal cortex in controls (1-4) or homotopic to these (5-6)	None<br>notes: L anterior temporal p = .08
Warren et al. (2009): ROI 5	Listening to narrative speech vs listening to reversed speech	CC<br>Aphasia<br>Covariate: auditory syntactic comprehension	NANB	NANT	ROI<br>Anat<br>NC	Number of ROIs: 6; ROIs: (1) L anterior superior temporal cortex; (2) L basal temporal language area; (3) L IFG pars triangularis; (4-6) homotopic counterparts; how ROIs defined: ROIs were defined anatomically in regions that were functionally connected with L anterior superior temporal cortex in controls (1-4) or homotopic to these (5-6)	None<br>notes: L anterior temporal p = .09
Warren et al. (2009): ROI 6	Listening to narrative speech	CC<br>Aphasia<br>Covariate:	NANB	NANT	ROI<br>Anat<br>NC	Number of ROIs: 2; ROIs: (1) L anterior superior temporal cortex; (2) R anterior superior temporal cortex;	None

	vs listening to reversed speech	connectivity between L and R ATL				how ROIs defined: ROIs were defined anatomically in regions that were functionally connected with L anterior superior temporal cortex in controls (1-4) or homotopic to these (5-6)	
Warren et al. (2009): ROI 7	Listening to narrative speech vs listening to reversed speech	CC Aphasia Covariate: time post onset	NANB	NANT	ROI Anat One	Number of ROIs: 1; ROI: L anterior superior temporal cortex; how ROI defined: ROIs were defined anatomically in regions that were functionally connected with L anterior superior temporal cortex in controls (1-4) or homotopic to these (5-6)	None
Warren et al. (2009): ROI 8	Listening to narrative speech vs listening to reversed speech	CC Aphasia Covariate: lesion volume	NANB	NANT	ROI Anat One	Number of ROIs: 1; ROI: L anterior superior temporal cortex; how ROI defined: ROIs were defined anatomically in regions that were functionally connected with L anterior superior temporal cortex in controls (1-4) or homotopic to these (5-6)	None
Warren et al. (2009): Cplx 1	Listening to narrative speech vs listening to reversed speech	CC Aphasia Covariate: lesion status of each voxel	NANB	NANT	Cplx	VLSM with FDR correction was used to identify any regions in which damage was predictive of L anterior temporal activation.	None
Fridriksson et al. (2010): Vox 1	Picture naming (correct trials) vs viewing abstract pictures	CC Aphasia Covariate: picture naming accuracy	YCT	UNR	Vox C-	Search volume: whole brain; software: FSL 4.1; voxelwise p: ~.02 (z > 2); cluster extent cutoff: based on GRFT	↑ L IFG pars orbitalis<br>↑ L occipital<br>↑ L anterior cingulate<br>notes: greater activation was associated with better picture naming; L IFG pars orbitalis activation classified as middle frontal gyrus in the paper, but coordinates suggest otherwise
Fridriksson et al. (2010): ROI 1	Picture naming (correct trials) vs viewing abstract pictures	CC Aphasia Covariate: picture naming accuracy	YCT	UNR	ROI Func One	Number of ROIs: 1; ROI: a single ROI comprising 3 regions where activation in patients was correlated with picture naming accuracy: the L IFG pars orbitalis, occipital lobe, and anterior cingulate; how ROI defined: based on SPM analysis 1; the purpose of this analysis was to determine whether these regions were recruited in the patients with better naming, or not activated in the patients with worse naming, relative to the control mean	Other: patients with better naming showed greater activation than controls, while the patients with poorer naming showed less activation than controls.
van Oers et al. (2010): ROI 4	Written word-picture matching vs visual decision	CC Aphasia Covariate: picture-word matching accuracy	C	UNR	ROI Mix NC	Number of ROIs: 7; ROIs: (1) L anterior language region (IFG); (2) L posterior language region (AG, SMG, STG, MTG); (3) R anterior language region (IFG); (4) R posterior language region (AG, SMG, STG, MTG); (5) frontal LI; (6) temporal LI; (7) whole network LI; how ROIs defined: WFU pickatlas	None
van Oers et al. (2010):	Semantic decision vs visual decision	CC Aphasia	C	UNR	ROI Mix	Number of ROIs: 7; ROIs: (1) L anterior language region (IFG); (2) L posterior	None

ROI 5		Covariate: semantic decision accuracy			NC	language region (AG, SMG, STG, MTG); (3) R anterior language region (IFG); (4) R posterior language region (AG, SMG, STG, MTG); (5) frontal LI; (6) temporal LI; (7) whole network LI; how ROIs defined: WFU pickatlas	
van Oers et al. (2010): ROI 8	Verb generation vs rest	CC<br>Aphasia<br>Covariate: overall language measure	UNR	UNR	ROI<br>Mix<br>NC	Number of ROIs: 7; ROIs: (1) L anterior language region (IFG); (2) L posterior language region (AG, SMG, STG, MTG); (3) R anterior language region (IFG); (4) R posterior language region (AG, SMG, STG, MTG); (5) frontal LI; (6) temporal LI; (7) whole network LI; how ROIs defined: WFU pickatlas	None
van Oers et al. (2010): ROI 11	Verb generation vs rest	CC<br>Aphasia<br>Covariate: lesion volume	UNR	UNR	ROI<br>Anat<br>NC	Number of ROIs: 2; ROIs: (1) R anterior language region (IFG); (2) R posterior language region (AG, SMG, STG, MTG); how ROIs defined: WFU pickatlas	None
van Oers et al. (2010): ROI 14	Verb generation vs rest	CC<br>Aphasia<br>Covariate: damage to L hemisphere language regions	UNR	UNR	ROI<br>Anat<br>NC	Number of ROIs: 2; ROIs: (1) R anterior language region (IFG); (2) R posterior language region (AG, SMG, STG, MTG); how ROIs defined: WFU pickatlas	None
Papoutsi et al. (2011): Vox 1	Listening to ambiguous sentences with subordinate resolution ("subordinate") vs listening to ambiguous sentences with dominant resolution ("dominant")	CC<br>Aphasia<br>Covariate: difference in percent of unacceptable judgments between subordinate and dominant sentences (dominance effect)	NANB	NANT	Vox<br>C-	Search volume: whole brain; software: SPM8; voxelwise p: .01; cluster extent cutoff: based on GRFT	↑ L insula<br>↑ L posterior STG/STS/MTG<br>↑ L mid temporal
Papoutsi et al. (2011): Cplx 1	Listening to ambiguous sentences with subordinate resolution ("subordinate") vs listening to ambiguous sentences with dominant resolution ("dominant")	CC<br>Aphasia<br>Covariate: modulation of L IFG connectivity by dominance effect	NANB	NANT	Cplx	A PPI analysis was carried out with the L IFG as the seed region. Correlations were computed between voxelwise modulation of connectivity with this region, and a behavioral measure of syntactic processing, which was the dominance effect: the difference in percent of unacceptable judgments between subordinate and dominant sentences. The resultant SPM was thresholded at voxelwise p < .01 (CDT), then corrected for multiple corrections based on cluster extent and GRFT using SPM8.	Other: patients with better syntactic performance had more connectivity from the L IFG seed region to L pMTG and adjacent areas (including the insula); pMTG also significant at voxelwise p < .001 in Figure 2B, corrected for multiple comparisons with GRFT
Papoutsi et al. (2011): Cplx 2	Listening to ambiguous sentences with subordinate resolution ("subordinate") vs listening to ambiguous	CC<br>Aphasia<br>Covariate: modulation of L pMTG connectivity by dominance effect	NANB	NANT	Cplx	A similar PPI analysis was carried out with the L pMTG as the seed region. Thresholding was the same as in the previous analysis.	None

	sentences with dominant resolution ("dominant")						
Sebastian & Kiran (2011): ROI 2	Semantic decision (correct trials) vs visual decision	CC Aphasia Covariate: lesion volume	YCT	UNR	ROI Mix NC	Number of ROIs: 4; ROIs: (1) L IFG (oper/tri); (2) L posterior perisylvian (pSTG, pMTG, AG, SMG); (3) R IFG (oper/tri); (4) R posterior perisylvian (pSTG, pMTG, AG, SMG); (5) language network LI; how ROIs defined: Harvard–Oxford atlas	None
Tyler et al. (2011): Vox 5	Listening to ambiguous sentences (dominant and subordinate) vs listening to unambiguous sentences ("unambiguous")	CC Aphasia Covariate: performance on acceptability judgment task (difference in percent of unacceptable judgments between ambiguous and unambiguous sentences)	NANB	NANT	Vox C-	Search volume: plausible fronto-temporo-parietal language regions; software: SPM5; voxelwise p: .01; cluster extent cutoff: based on GRFT	↑ L IFG pars triangularis ↑ L IFG pars orbitalis ↑ R insula ↑ R mid temporal notes: also L pMTG but this did not reach significance
Tyler et al. (2011): Vox 8	Listening to ambiguous sentences (dominant and subordinate) vs listening to unambiguous sentences ("unambiguous")	CC Aphasia Covariate: difference in percent of unacceptable judgments between subordinate and dominant sentences (dominance effect)	NANB	NANT	Vox C-	Search volume: plausible fronto-temporo-parietal language regions; software: SPM5; voxelwise p: .01; cluster extent cutoff: based on GRFT	None
Tyler et al. (2011): ROI 1	Listening to ambiguous sentences (dominant and subordinate) vs listening to unambiguous sentences ("unambiguous")	CC Aphasia Covariate: performance on acceptability judgment task (difference in percent of unacceptable judgments between ambiguous and unambiguous sentences)	NANB	NANT	ROI Anat NC	Number of ROIs: 3; ROIs: (1) IFG pars opercularis; (2) IFG pars triangularis; (3) IFG pars orbitalis; how ROIs defined: AAL	↑ L IFG pars triangularis ↑ L IFG pars orbitalis
Tyler et al. (2011): ROI 2	Listening to ambiguous sentences (dominant and subordinate) vs listening to unambiguous sentences ("unambiguous")	CC Aphasia Covariate: difference in percentage of unacceptable judgments between subordinate and dominant sentences (dominance effect)	NANB	NANT	ROI Anat NC	Number of ROIs: 3; ROIs: (1) IFG pars opercularis; (2) IFG pars triangularis; (3) IFG pars orbitalis; how ROIs defined: AAL	None
Allendorfer et al. (2012): ROI 4	Verb generation (overt, event-related) vs noun repetition (event-related)	CC Aphasia Covariate: overt verb generation accuracy	C	UNR	ROI Func NC	Number of ROIs: 3; ROIs: (1) L MTG; (2) L SFG/CG; (3) left MFG; how ROIs defined: regions activated by the contrast of overt verb generation vs noun repetition in patients	↑ L dorsolateral prefrontal cortex ↑ L SMA/medial prefrontal
Allendorfer et al.	Verb generation (overt, event-related) vs verb	CC Aphasia	C	UNR	ROI Func NC	Number of ROIs: 2; ROIs: (1) R insula/IFG; (2) R STG; how ROIs defined: prominent R hemisphere	None

(2012):<br>ROI 5	generation (covert, event-related)	Covariate: overt verb generation accuracy				activations for the contrast of overt and covert verb generation in patients	
Griffis, Nenert, Allendorfer, & Szaflarski (2017):<br>ROI 1	Semantic decision vs tone decision	CC<br>Aphasia<br>Covariate: semantic decision accuracy	C	UNR	ROI<br>Oth<br>FWE	Number of ROIs: 3; ROIs: (1) L AG and bilateral midline components of the canonical semantic network, along with reduced activity in R frontal, temporal and parietal regions; (2) bilateral IFG pars orbitalis; (3) L IFG and DLPFC along with bilateral midline regions; how ROIs defined: ROIs are mixing coefficients of functional networks arising from mCCA + jICA that were differently represented in the patient and control groups	↑ L IFG<br>↑ L dorsolateral prefrontal cortex<br>↑ L SMA/medial prefrontal<br>↑ L angular gyrus<br>↑ L precuneus<br>↑ L posterior cingulate<br>↑ R IFG pars orbitalis<br>↑ R SMA/medial prefrontal<br>↑ R precuneus<br>↑ R posterior cingulate<br>↓ L insula<br>↓ R IFG pars opercularis<br>↓ R IFG pars triangularis<br>↓ R insula<br>↓ R dorsal precentral<br>↓ R supramarginal gyrus<br>↓ R posterior STG<br>↓ R mid temporal<br>notes: all 3 networks were significantly correlated; analysis of networks so involvement of each individual region cannot be assured
Griffis, Nenert, Allendorfer, & Szaflarski (2017):<br>ROI 2	Semantic decision vs tone decision	CC<br>Aphasia<br>Covariate: average of semantic and phonemic fluency	UNR	UNR	ROI<br>Oth<br>FWE	Number of ROIs: 3; ROIs: (1) L AG and bilateral midline components of the canonical semantic network, along with reduced activity in R frontal, temporal and parietal regions; (2) bilateral IFG pars orbitalis; (3) L IFG and DLPFC along with bilateral midline regions; how ROIs defined: ROIs are mixing coefficients of functional networks arising from mCCA + jICA that were differently represented in the patient and control groups	↑ L IFG<br>↑ L dorsolateral prefrontal cortex<br>↑ L SMA/medial prefrontal<br>↑ L angular gyrus<br>↑ L precuneus<br>↑ L posterior cingulate<br>↑ R SMA/medial prefrontal<br>↑ R precuneus<br>↑ R posterior cingulate<br>↓ L insula<br>↓ R IFG pars opercularis<br>↓ R IFG pars triangularis<br>↓ R insula<br>↓ R dorsal precentral

							↓ R supramarginal gyrus<br>↓ R posterior STG<br>↓ R mid temporal<br>notes: networks 1 and 3 were significantly correlated; analysis of networks so involvement of each individual region cannot be assured
Griffis, Nenert, Allendorfer, & Szaflarski (2017): ROI 3	Semantic decision vs tone decision	CC<br>Aphasia<br>Covariate: BNT	UNR	UNR	ROI<br>Oth<br>FWE	Number of ROIs: 3; ROIs: (1) L AG and bilateral midline components of the canonical semantic network, along with reduced activity in R frontal, temporal and parietal regions; (2) bilateral IFG pars orbitalis; (3) L IFG and DLPFC along with bilateral midline regions; how ROIs defined: ROIs are mixing coefficients of functional networks arising from mCCA + jICA that were differently represented in the patient and control groups	↑ L IFG<br>↑ L dorsolateral prefrontal cortex<br>↑ L SMA/medial prefrontal<br>↑ L angular gyrus<br>↑ L precuneus<br>↑ L posterior cingulate<br>↑ R SMA/medial prefrontal<br>↑ R precuneus<br>↑ R posterior cingulate<br>↓ L insula<br>↓ R IFG pars opercularis<br>↓ R IFG pars triangularis<br>↓ R insula<br>↓ R dorsal precentral<br>↓ R supramarginal gyrus<br>↓ R posterior STG<br>↓ R mid temporal<br>notes: networks 1 and 3 were significantly correlated; analysis of networks so involvement of each individual region cannot be assured
Griffis, Nenert, Allendorfer, Vannest, et al. (2017): ROI 2	Semantic decision vs tone decision	CC<br>Aphasia<br>Covariate: lesion volume	UNR	UNR	ROI<br>Func<br>FWE	Number of ROIs: 5; ROIs: (1) overall canonical semantic network (CSN); (2) L CSN; (3) R CSN; (4) mirror L CSN in R; (5) out-of-network CSN in R; how ROIs defined: control data	None
Griffis, Nenert, Allendorfer, Vannest, et al. (2017): ROI 3	Semantic decision vs tone decision	CC<br>Aphasia<br>Covariate: semantic decision accuracy	C	UNR	ROI<br>Func<br>One	Number of ROIs: 1; ROI: CSN; how ROI defined: control data; lesion volume covariate	↑ L IFG<br>↑ L dorsolateral prefrontal cortex<br>↑ L SMA/medial prefrontal<br>↑ L angular gyrus

							↑ L precuneus<br>↑ L mid temporal<br>↑ L anterior temporal<br>↑ L posterior cingulate<br>↑ L cerebellum<br>↑ R IFG<br>↑ R dorsolateral prefrontal cortex<br>↑ R SMA/medial prefrontal<br>↑ R angular gyrus<br>↑ R precuneus<br>↑ R anterior temporal<br>↑ R posterior cingulate<br>↑ R cerebellum<br>notes: correlation calculated for the whole network of regions, so correlation of individual regions cannot be assured
Griffis, Nenert, Allendorfer, Vannest, et al. (2017): ROI 4	Semantic decision vs tone decision	CC<br>Aphasia<br>Covariate: average of semantic and phonemic fluency	UNR	UNR	ROI<br>Func<br>One	Number of ROIs: 1; ROI: CSN; how ROI defined: control data; lesion volume covariate	↑ L IFG<br>↑ L dorsolateral prefrontal cortex<br>↑ L SMA/medial prefrontal<br>↑ L angular gyrus<br>↑ L precuneus<br>↑ L mid temporal<br>↑ L anterior temporal<br>↑ L posterior cingulate<br>↑ L cerebellum<br>↑ R IFG<br>↑ R dorsolateral prefrontal cortex<br>↑ R SMA/medial prefrontal<br>↑ R angular gyrus<br>↑ R precuneus<br>↑ R anterior temporal<br>↑ R posterior cingulate<br>↑ R cerebellum<br>notes: correlation calculated for the whole network of regions, so correlation of individual regions cannot be assured
Griffis, Nenert, Allendorfer, Vannest, et	Semantic decision vs tone decision	CC<br>Aphasia<br>Covariate: BNT	UNR	UNR	ROI<br>Func<br>One	Number of ROIs: 1; ROI: CSN; how ROI defined: control data; lesion volume covariate	↑ L IFG<br>↑ L dorsolateral prefrontal cortex<br>↑ L SMA/medial

al. (2017): ROI 5							prefrontal<br>↑ L angular gyrus<br>↑ L precuneus<br>↑ L mid temporal<br>↑ L anterior temporal<br>↑ L posterior cingulate<br>↑ L cerebellum<br>↑ R IFG<br>↑ R dorsolateral prefrontal cortex<br>↑ R SMA/medial prefrontal<br>↑ R angular gyrus<br>↑ R precuneus<br>↑ R anterior temporal<br>↑ R posterior cingulate<br>↑ R cerebellum<br>notes: correlation calculated for the whole network of regions, so correlation of individual regions cannot be assured
Griffis, Nenert, Allendorfer, Vannest, et al. (2017): Cplx 7	Semantic decision vs tone decision	CC<br>Aphasia<br>Covariate: interactions of semantic fluency and naming measures by lesion size	UNR	UNR	Cplx	For the 4 R hemisphere regions that were more activated in patients with larger lesions (SPM analysis 4), analyses were carried out to determine whether the semantic fluency or naming measures were differentially impacted by activation depending on whether lesions were larger or smaller.	Other:<br>For 1 of the 4 regions (R SMA), there were significant interactions such that in patients with larger lesions, more activation was associated with higher semantic fluency scores and higher BNT scores, while in patients with smaller lesions, more activation was associated with lower fluency and BNT scores. There was a similar relationship with semantic fluency in the R IFG pars opercularis but only at p(FDR) = 0.07.
Nenert et al. (2018): Vox 11	Semantic decision vs tone decision	CC<br>Aphasia T1<br>Covariate: semantic decision accuracy	C	UNR	Vox VP	Search volume: whole brain; software: SPM12/SnPM13; voxelwise p: FWE p < .05	↑ L anterior temporal<br>notes: unclear why this type of analysis was run only for semantic

							task, and only at T1
Hartwigsen et al. (2020): Cplx 1	Syllable count decision vs rest	CC<br>Aphasia after cTBS to posterior IFG vs sham; same patients, repeated measures<br>Covariate: Δ RT for syllable decision (cTBS to posterior IFG timepoint vs sham timepoint)	UNR	C	Cplx	Whole brain correlations were computed between the difference in functional activity after cTBS to posterior IFG versus sham stimulation, and the difference in reaction times on the syllable counting task under these two conditions. The resulting SPM was thresholded at voxelwise p < .001 (CDT) followed by correction for multiple comparisons based on cluster extent and GRFT using SPM12.	Other: Upregulation of the R supramarginal gyrus after cTBS was significantly associated with slowing of RT after cTBS. This finding remained significant after including lesion volume as covariate.
Hartwigsen et al. (2020): Cplx 2	Semantic decision vs rest	CC<br>Aphasia after cTBS to anterior IFG vs sham; same patients, repeated measures<br>Covariate: Δ RT for semantic decision (cTBS to posterior IFG timepoint vs sham timepoint)	UNR	C	Cplx	Whole brain correlations were computed between the difference in functional activity after cTBS to anterior IFG versus sham stimulation, and the difference in reaction times on the semantic decision task under these two conditions. The resulting SPM was thresholded at voxelwise p < .001 (CDT) followed by correction for multiple comparisons based on cluster extent and GRFT using SPM12.	None

Second level contrast = Which of the 8 relevant classes of analyses is this? Which group or groups of participants are included? If there is a covariate, what is it?; Acc = Is accuracy matched across the second level contrast?; RT = Is reaction time matched across the second level contrast?; Stats = Does the analysis involve voxelwise statistics, region(s) of interest (ROI), or something else (complex)? If voxelwise, how are multiple comparisons across voxels accounted for? If ROI, were the ROI(s) anatomical, functional, laterality indices, mixed, or something else? If there was more than one ROI, how were the ROIs corrected for multiple comparions?; Yellow underline = minor limitation; Orange underline = moderate limitation; Red underline = major limitation; CC = Cross-sectional correlation with language or other measure; YCT = Yes, correct trials only; C = Accuracy or RT is covariate; UNR = Unknown, not reported; NANB = N/A, no behavioral measure; NANT = N/A, no timeable task; Vox = Voxelwise; VP = Voxelwise correction based on permutation testing; VFWC = Voxelwise FWE correction and additional arbitrary cluster correction; C- = Clusterwise correction with with GRFT and lenient voxelwise p; ROI = Region(s) of interest; Anat = Anatomical; Func = Functional; Oth = Other; Mix = Mixed; FWE = Familywise error (FWE); NC = No correction; One = One only; Cplx = Complex.

Supplementary Table S13. Longitudinal change in aphasia: Methodologically robust analyses

Analysis	First level contrast	Second level contrast	Matched for		Stats	Notes	Findings
			Acc	RT			
Saur et al. (2006): ROI 1	Listening to sentences and making a plausibility judgment vs listening to reversed speech	LA<br>Aphasia T2 vs T1	AM	UNR	ROI<br>Func<br>FWE	Behavioral data notes: accuracy combines language and control conditions; number of ROIs: 6; ROIs: (1) L IFG pars orbitalis; (2) L IFG pars triangularis; (3) L MTG; (4) R insula; (5) R IFG pars triangularis; (6) R SMA; how ROIs defined: peak voxels of overall activation map based on all three time points in patients	↑ R insula<br>↑ R SMA/medial prefrontal<br>notes: some other ROIs also significant prior to correction for multiple comparisons; n.b. performance confound
Saur et al. (2006): ROI 2	Listening to sentences and making a plausibility judgment vs listening to reversed speech	LA<br>Aphasia T3 vs T2	AM	UNR	ROI<br>Func<br>FWE	Behavioral data notes: accuracy combines language and control conditions; number of ROIs: 6; ROIs: (1) L IFG pars orbitalis; (2) L IFG pars triangularis; (3) L MTG; (4) R insula; (5) R IFG pars triangularis; (6) R SMA; how ROIs defined: peak voxels of overall activation map based on all three time points in patients	None<br>notes: some other ROIs also significant prior to correction for multiple comparisons; n.b. performance confound
Saur et al. (2006): ROI 3	Listening to sentences and making a plausibility judgment vs listening to reversed speech	LA<br>Aphasia T3 vs T1	AM	UNR	ROI<br>Func<br>FWE	Behavioral data notes: accuracy combines language and control conditions; number of ROIs: 6; ROIs: (1) L IFG pars orbitalis; (2) L IFG pars triangularis; (3) L MTG; (4) R insula; (5) R IFG pars triangularis; (6) R SMA; how ROIs defined: peak voxels of overall activation map based on all three time points in patients	↑ L posterior MTG<br>notes: some other ROIs also significant prior to correction for multiple comparisons; n.b. performance confound
Nenert et al. (2017): ROI 1	Semantic decision vs tone decision	LA<br>Aphasia ANOVA including T1, T2, T3	AS	UNR	ROI<br>LI<br>NC	Number of ROIs: 5; ROIs: (1) frontal LI; (2) temporo-parietal LI; (3) cerebellar LI; (4) fronto-parietal LI; (5) Broca's LI	None
Nenert et al. (2018): Cplx 1	Semantic decision vs tone decision	LA<br>Aphasia (comparisons between all pairs of time points)	AS	UNR	Cplx	PPI analyses were carried out to investigate potential changes over time in how connectivity from L and R IFG was modulated by the semantic decision task. The resultant SPM was thresholded at FWE p < .05 using permutation testing implemented in SnPM 13.	None

Second level contrast = Which of the 8 relevant classes of analyses is this? Which group or groups of participants are included? If there is a covariate, what is it?; Acc = Is accuracy matched across the second level contrast?; RT = Is reaction time matched across the second level contrast?; Stats = Does the analysis involve voxelwise statistics, region(s) of interest (ROI), or something else (complex)? If voxelwise, how are multiple comparisons across voxels accounted for? If ROI, were the ROI(s) anatomical, functional, laterality indices, mixed, or something else? If there was more than one ROI, how were the ROIs corrected for multiple comparions?; Yellow underline = minor limitation; Orange underline = moderate limitation; Red underline = major limitation; LA = Longitudinal change in aphasia; AS = Appear similar; AM = Appear mismatched; UNR = Unknown, not reported; ROI = Region(s) of interest; Func = Functional; LI = Laterality indi(ces); FWE = Familywise error (FWE); NC = No correction; Cplx = Complex.

Supplementary Table S14. Cross-sectional between two groups with aphasia: Methodologically robust analyses

Analysis	First level contrast	Second level contrast	Matched for		Stats	Notes	Findings
			Acc	RT			
Leff et al. (2002): ROI 2	Higher word rates vs lower word rates	CAA Aphasia with pSTS damage (n = 6) vs aphasia without pSTS damage (n = 9)	NANB	NANT	ROI Func One	Number of ROIs: 1; ROI: R pSTS; how ROI defined: the peak voxel for the contrast in the R pSTS from each subject's individual analysis, but the search region is not stated; the controls and patients without pSTS damage were combined, however it is stated in the caption to Figure 2 that the patients with pSTS damage were significantly different to both	↑ R posterior STS
Blank et al. (2003): Vox 3	Propositional speech production vs rest	CAA Aphasia with IFG POp damage (n = 7) vs without IFG POp damage (n = 7)	N	NANT	Vox SVC	Behavioral data notes: word rates not reported, but offline speech sample differed; search volume: voxels spared in all patients; software: SPM99; voxelwise p: FWE p < .05 with SVC in R pars opercularis	None notes: patients with L IFG POp damage showed numerically more signal in the R IFG POp
Blank et al. (2003): Vox 6	Propositional speech production vs counting	CAA Aphasia with IFG POp damage (n = 7) vs without IFG POp damage (n = 7)	N	NANT	Vox SVC	Behavioral data notes: word rates not reported, but offline speech sample differed; search volume: voxels spared in all patients; software: SPM99; voxelwise p: FWE p < .05 with SVC in R pars opercularis	None
Crinion & Price (2005): Vox 3	Listening to narrative speech vs listening to reversed speech	CAA Aphasia with temporal lobe damage (n = 8) vs without temporal lobe damage (n = 9)	NANB	NANT	Vox VFWC	Search volume: whole brain; software: SPM2; voxelwise p: FWE p < .05; cluster extent cutoff: 5 voxels (size not stated)	↓ L posterior STG/STS/MTG ↓ L mid temporal
Crinion & Price (2005): Cplx 4	Listening to narrative speech vs listening to reversed speech	CAA Aphasia with temporal damage (n = 8) vs without temporal damage (n = 9)	NANB	NANT	Cplx	Correlations were computed between activity in each voxel, and post-scan story recall, and were compared between the two aphasia groups, in regions with a main effect of story comprehension. The threshold was p < 0.05 corrected, plus a minimum cluster size of 5 voxels.	None
Crinion et al. (2006): ROI 3	Listening to narrative speech vs listening to reversed speech	CAA Aphasia with temporal damage excluding anterior temporal cortex (n = 9) vs with no temporal lobe damage (excluding 1 with missing behavioral data and 1 outlier) (n = 4)	NANB	NANT	ROI Func One	Number of ROIs: 1; ROI: L ATL; how ROI defined: activation in the control group; two other ROIs are described in the methods, but never used in any analyses	↓ L anterior temporal notes: patients with posterior temporal damage had less signal change
Warren et al. (2009): ROI 11	Listening to narrative speech vs listening to reversed speech	CAA Aphasia with positive anterior temporal interconnectivity (n = 8) vs with negative anterior temporal	NANB	NANT	ROI Anat NC	Number of ROIs: 6; ROIs: (1) L anterior superior temporal cortex; (2) L basal temporal language area; (3) L IFG pars triangularis; (4-6) homotopic counterparts; how ROIs defined: ROIs were defined anatomically in regions	↑ L IFG pars triangularis

		interconnectivity (n = 8)				that were functionally connected with L anterior superior temporal cortex in controls (1-4) or homotopic to these (5-6); excluded 4 patients with L IFG damage	
Hartwigsen et al. (2020): Vox 1	Syllable count decision vs rest	CAA Aphasia after cTBS to posterior IFG vs sham; same patients, repeated measures	Y	N	Vox C+	Behavioral data notes: significantly slower response times when cTBS was applied over pIFG relative to when sham cTBS was applied; search volume: voxels spared in all patients; software: SPM12; voxelwise p: .001; cluster extent cutoff: based on GRFT	↓ L IFG pars opercularis ↓ L SMA/medial prefrontal ↓ R SMA/medial prefrontal ↓ R basal ganglia notes: based on Figure 4A and Table 3
Hartwigsen et al. (2020): Vox 2	Syllable count decision vs rest	CAA Aphasia after cTBS to posterior IFG vs after cTBS to anterior IFG; same patients, repeated measures	Y	N	Vox C+	Behavioral data notes: significantly slower response times when cTBS was applied over pIFG relative to when cTBS was applied over aIFG; search volume: voxels spared in all patients; software: SPM12; voxelwise p: .001; cluster extent cutoff: based on GRFT	↓ L IFG pars opercularis notes: based on Table 3
Hartwigsen et al. (2020): Vox 3	Semantic decision vs rest	CAA Aphasia after cTBS to anterior IFG vs sham; same patients, repeated measures Somewhat valid (no behavioral difference)	Y	Y	Vox C+	Behavioral data notes: difference in reaction time did not survive correction; search volume: voxels spared in all patients; software: SPM12; voxelwise p: .001; cluster extent cutoff: based on GRFT	↓ L insula ↓ L dorsolateral prefrontal cortex ↓ R insula ↓ R dorsolateral prefrontal cortex ↓ R SMA/medial prefrontal notes: based on Figure 4B and Table 3
Hartwigsen et al. (2020): Vox 4	Semantic decision vs rest	CAA Aphasia after cTBS to anterior IFG vs after cTBS to posterior IFG ; same patients, repeated measures	Y	N	Vox C+	Behavioral data notes: significantly slower response times when cTBS was applied over aIFG relative to when cTBS was applied over pIFG; search volume: voxels spared in all patients; software: SPM12; voxelwise p: .001; cluster extent cutoff: based on GRFT	↓ L insula ↓ R insula ↓ R dorsolateral prefrontal cortex notes: based on Table 3

Second level contrast = Which of the 8 relevant classes of analyses is this? Which group or groups of participants are included? If there is a covariate, what is it?; Acc = Is accuracy matched across the second level contrast?; RT = Is reaction time matched across the second level contrast?; Stats = Does the analysis involve voxelwise statistics, region(s) of interest (ROI), or something else (complex)? If voxelwise, how are multiple comparisons across voxels accounted for? If ROI, were the ROI(s) anatomical, functional, laterality indices, mixed, or something else? If there was more than one ROI, how were the ROIs corrected for multiple comparions?; Yellow underline = minor limitation; Orange underline = moderate limitation; Red underline = major limitation; CAA = Cross-sectional between two groups with aphasia; Y = Yes, matched; N = No, different; NANB = N/A, no behavioral measure; NANT = N/A, no timeable task; Vox = Voxelwise; C+ = Clusterwise correction with with GRFT and stringent voxelwise p; VFWC = Voxelwise FWE correction and additional arbitrary cluster correction; SVC = Small volume correction; ROI = Region(s) of interest; Anat = Anatomical; Func = Functional; NC = No correction; One = One only; Cplx = Complex.

Supplementary Table S15. Cross-sectional performance-defined conditions: Methodologically robust analyses

Analysis	First level contrast	Second level contrast	Matched for		Stats	Notes	Findings
			Acc	RT			
Fridriksson et al. (2009): Vox 2	Picture naming (phonemic paraphasias) vs picture naming (correct trials)	CB<br>Aphasia	NBD	UNR	Vox<br>C-	Search volume: voxels spared in all patients; software: FSL (FEAT 5.4); voxelwise p: ~.01 (z > 2.3); cluster extent cutoff: based on GRFT	↑ L superior parietal<br>↑ L posterior inferior temporal gyrus/fusiform gyrus<br>↑ L occipital
Fridriksson et al. (2009): Vox 3	Picture naming (semantic paraphasias) vs picture naming (correct trials)	CB<br>Aphasia	NBD	UNR	Vox<br>C-	Search volume: voxels spared in all patients; software: FSL (FEAT 5.4); voxelwise p: ~.01 (z > 2.3); cluster extent cutoff: based on GRFT	↑ R posterior inferior temporal gyrus/fusiform gyrus<br>↑ R occipital
Skipper-Kallal et al. (2017a): Vox 5	Picture naming (both phases, correct trials) vs picture naming (both phases, incorrect trials)	CB<br>Aphasia with naming < 80% (n = 24)	NBD	UNR	Vox<br>C-	Search volume: whole brain gray matter; software: FSL 5.0.6; voxelwise p: ~.01 (z > 2.3); cluster extent cutoff: based on GRFT	None
Pillay et al. (2018): Vox 1	Reading nouns aloud (correct trials) vs reading nouns aloud (incorrect trials)	CB<br>Aphasia	NBD	Y	Vox<br>CCS	Search volume: whole brain; software: AFNI; voxelwise p: .01; cluster extent cutoff: 1.609 cc; regarding correction for multiple comparisons, addition of monoexponential function reduces but does not eliminate inflation of p values (Cox et al., 2017)	↑ L angular gyrus<br>↓ L ventral precentral/inferior frontal junction<br>↓ L SMA/medial prefrontal<br>↓ R insula<br>↓ R ventral precentral/inferior frontal junction<br>↓ R SMA/medial prefrontal<br>notes: positive region (L AG) was part of the semantic network, while many negative regions were positively modulated by reaction time in the aphasia group

Second level contrast = Which of the 8 relevant classes of analyses is this? Which group or groups of participants are included? If there is a covariate, what is it?; Acc = Is accuracy matched across the second level contrast?; RT = Is reaction time matched across the second level contrast?; Stats = Does the analysis involve voxelwise statistics, region(s) of interest (ROI), or something else (complex)? If voxelwise, how are multiple comparisons across voxels accounted for? If ROI, were the ROI(s) anatomical, functional, laterality indices, mixed, or something else? If there was more than one ROI, how were the ROIs corrected for multiple comparions?; Yellow underline = minor limitation; Orange underline = moderate limitation; Red underline = major limitation; CB = Cross-sectional performance-defined conditions; Y = Yes, matched; NBD = No, by design; UNR = Unknown, not reported; Vox = Voxelwise; C- = Clusterwise correction with with GRFT and lenient voxelwise p; CCS = Clusterwise correction based on 3dClustSim.

Supplementary Table S16: Complete coding of all included studies

Weiller et al. (1995)

Reference

Authors	Weiller C, Isensee C, Rijntjes M, Huber W, Müller S, Bier D, Dutschka K, Woods RP, Noth J, Diener HC
Title	Recovery from Wernicke's aphasia: a positron emission tomographic study
Reference	*Ann Neurol* 1995; 37: 723-732
PMID	7778845
DOI	10.1002/ana.410370605

Participants

Language	German
Inclusion criteria	Lesion including L pSTG; moderate-to-severe Wernicke's aphasia in the subacute period; now recovered and not aphasic per formal testing; able to perform verb generation task
Number of individuals with aphasia	6
Number of control participants	6
Were any of the participants included in any previous studies?	No
Is age reported for patients and controls, and matched?	No (mean 58 years, range 50-66 years; controls were younger: mean 35 years; range 27-50 years)
Is sex reported for patients and controls, and matched?	Yes (males: 6; females: 0)
Is handedness reported for patients and controls, and matched?	Yes (right: 6; left: 0)
Is time post stroke onset reported and appropriate to the study design?	Yes (range 5-117 months)
To what extent is the nature of aphasia characterized?	Comprehensive battery
Language evaluation	AAT
Aphasia severity	Recovered; not aphasic per formal testing
Aphasia type	Recovered, but all had moderate-severe Wernicke's aphasia in the subacute period
First stroke only?	Yes
Stroke type	Ischemic only
To what extent is the lesion distribution characterized?	Individual lesions
Lesion extent	Not stated
Lesion location	Posterior L MCA infarct, lesion to the L posterior STG usually extending to MTG and AG
Participants notes	6 patients were selected from a database of 600 carefully documented cases

Imaging

Modality	PET (rCBF)
Is the study cross-sectional or longitudinal?	Cross-sectional
If longitudinal, at what time point(s) were imaging data acquired?	—
If longitudinal, was there any intervention between the time points?	—
Is the scanner described?	Yes (CTI ECAT 953/15)
Is the timing of stimulus presentation and image acquisition clearly described and appropriate?	Yes
Design type	PET
Total images acquired	6
Are the imaging acquisition parameters, including coverage, adequately described and appropriate?	Yes (axial; field of view = 5.4 cm; perisylvian only)

Is preprocessing and intrasubject coregistration adequately described and appropriate?	Yes
Is first level model fitting adequately described and appropriate?	Yes
Is intersubject normalization adequately described and appropriate?	Yes
Imaging notes	—

Conditions

Are the conditions clearly described?	Yes

Condition	Response type	Repetitions	All groups could do?	All individuals could do?
verb generation	Multiple words (covert)	2	Yes	Yes
pseudoword repetition	Multiple words (covert)	2	Yes	Yes
rest	None	2	N/A	N/A

Conditions notes	Auditory presentation; pre-scan behavioral data reported

Contrasts

Are the contrasts clearly described?	Yes

Contrast 1: verb generation vs rest

Language condition	Verb generation
Control condition	Rest
Are the conditions matched for visual demands?	Yes
Are the conditions matched for auditory demands?	No
Are the conditions matched for motor demands?	Yes
Are the conditions matched for cognitive/executive demands?	No
Is accuracy matched between the language and control tasks for all relevant groups?	N/A, tasks not comparable
Is reaction time matched between the language and control tasks for all relevant groups?	N/A, tasks not comparable
Behavioral data notes	—
Are control data reported in this paper or another that is referenced?	Somewhat
Does the contrast selectively activate plausible relevant language regions in the control group?	Yes
Are activations lateralized in the control data?	Yes
Control activation notes	L posterior temporal, IFG and ventral precentral gyrus, much smaller activations in the R hemisphere
Contrast notes	—

Contrast 2: pseudoword repetition vs rest

Language condition	Pseudoword repetition
Control condition	Rest
Are the conditions matched for visual demands?	Yes
Are the conditions matched for auditory demands?	No
Are the conditions matched for motor demands?	Yes
Are the conditions matched for cognitive/executive demands?	No
Is accuracy matched between the language and control tasks for all relevant groups?	N/A, tasks not comparable
Is reaction time matched between the language and control tasks for all relevant groups?	N/A, tasks not comparable
Behavioral data notes	—

Are control data reported in this paper or another that is referenced?	Somewhat
Does the contrast selectively activate plausible relevant language regions in the control group?	Somewhat
Are activations lateralized in the control data?	Somewhat
Control activation notes	L posterior temporal only; similar but less extensive activation in the R hemisphere
Contrast notes	—

Analyses

Are the analyses clearly described?	Yes

Voxelwise analysis 1

First level contrast	Verb generation vs rest
Analysis class	Cross-sectional aphasia vs control
Group(s)	Aphasia vs control
Covariate	—
Is the second level contrast valid in terms of the group(s), time point(s), and measures involved?	Yes
Is accuracy matched across the second level contrast?	Appear mismatched
Is reaction time matched across the second level contrast?	Unknown, not reported
Behavioral data notes	In practice trials, patients produced 1.5 words on average per prompt, not all of which were verbs, while controls 2.3 words on average per prompt, almost all of which were verbs
Type of analysis	Voxelwise
Search volume	Perisylvian
Correction for multiple comparisons	No direct comparison
Software	SPM
Voxelwise p	—
Cluster extent	—
Statistical details	Qualitative comparison on p. 729 (the word "significant" is used)
Findings	↑ R IFG<br>↑ R posterior STG/STS/MTG<br>↓ L posterior STG/STS/MTG
Findings notes	Based more on Figure 2 than the text

Voxelwise analysis 2

First level contrast	Pseudoword repetition vs rest
Analysis class	Cross-sectional aphasia vs control
Group(s)	Aphasia vs control
Covariate	—
Is the second level contrast valid in terms of the group(s), time point(s), and measures involved?	Yes
Is accuracy matched across the second level contrast?	Appear similar
Is reaction time matched across the second level contrast?	Unknown, not reported
Behavioral data notes	All participants are reported to have had no difficulties in performing the repetition task
Type of analysis	Voxelwise
Search volume	Perisylvian
Correction for multiple comparisons	No direct comparison
Software	SPM
Voxelwise p	—
Cluster extent	—
Statistical details	Qualitative comparison on p. 729 (the word "significant" is used)
Findings	↑ L ventral precentral/inferior frontal junction

	↑ R IFG<br>↑ R posterior STG/STS/MTG<br>↓ L posterior STG/STS/MTG
Findings notes	Based more on Figure 2 than the text

Notes

Excluded analyses	—

Belin et al. (1996)

Reference

Authors	Belin P, Van Eeckhout P, Zilbovicius M, Remy P, François C, Guillaume S, Chain F, Rancurel G, Samson Y
Title	Recovery from nonfluent aphasia after melodic intonation therapy: a PET study
Reference	*Neurology* 1996; 47: 1504-1511
PMID	8960735
DOI	10.1212/wnl.47.6.1504

Participants

Language	French
Inclusion criteria	MCA; persistent severe non-fluent aphasia followed by marked improvement with MIT
Number of individuals with aphasia	7
Number of control participants	0
Were any of the participants included in any previous studies?	No
Is age reported for patients and controls, and matched?	Yes (mean 49.7 years, range 40-58 years)
Is sex reported for patients and controls, and matched?	No
Is handedness reported for patients and controls, and matched?	Yes (right: 7; left: 0)
Is time post stroke onset reported and appropriate to the study design?	Yes (range 15-149 months; including MIT for the most recent 1-108 months)
To what extent is the nature of aphasia characterized?	Severity and type
Language evaluation	BDAE
Aphasia severity	Persistent severe non-fluent aphasia followed by marked improvement with MIT
Aphasia type	5 global, 2 Broca's
First stroke only?	Not stated
Stroke type	Not stated
To what extent is the lesion distribution characterized?	Individual lesions
Lesion extent	Not stated, but note that hypoperfusion greatly exceeded the infarct in all but 1 patient
Lesion location	L MCA; 2 also had ACA
Participants notes	—

Imaging

Modality	PET (rCBF)
Is the study cross-sectional or longitudinal?	Cross-sectional
If longitudinal, at what time point(s) were imaging data acquired?	—
If longitudinal, was there any intervention between the time points?	—

Is the scanner described?	Yes (CEA LETI-TTV03)
Is the timing of stimulus presentation and image acquisition clearly described and appropriate?	Yes
Design type	PET
Total images acquired	4
Are the imaging acquisition parameters, including coverage, adequately described and appropriate?	Yes (7 transaxial slices 12 mm apart)
Is preprocessing and intrasubject coregistration adequately described and appropriate?	Yes
Is first level model fitting adequately described and appropriate?	Yes
Is intersubject normalization adequately described and appropriate?	Yes
Imaging notes	—

Conditions

Are the conditions clearly described?	Yes

Condition	Response type	Repetitions	All groups could do?	All individuals could do?
word repetition with MIT-like intonation	Word (overt)	1	Yes	Unknown
word repetition	Word (overt)	1	Yes	Unknown
listening to words	None	1	N/A	N/A
rest	None	1	N/A	N/A

Conditions notes	—

Contrasts

Are the contrasts clearly described?	Yes

Contrast 1: word repetition with MIT-like intonation vs word repetition

Language condition	Word repetition with MIT-like intonation
Control condition	Word repetition
Are the conditions matched for visual demands?	Yes
Are the conditions matched for auditory demands?	Yes
Are the conditions matched for motor demands?	Yes
Are the conditions matched for cognitive/executive demands?	Yes
Is accuracy matched between the language and control tasks for all relevant groups?	No, by design
Is reaction time matched between the language and control tasks for all relevant groups?	Unknown, not reported
Behavioral data notes	More words were correctly repeated with MIT (16.3 ± 8) than without (12.4 ± 8; $p < 0.03$)
Are control data reported in this paper or another that is referenced?	N/A
Does the contrast selectively activate plausible relevant language regions in the control group?	N/A
Are activations lateralized in the control data?	N/A
Control activation notes	—
Contrast notes	—

Analyses

Are the analyses clearly described?	Yes

ROI analysis 1

First level contrast	Word repetition with MIT-like intonation vs word repetition
Analysis class	Cross-sectional performance-defined conditions

Group(s)	Aphasia
Covariate	—
Is the second level contrast valid in terms of the group(s), time point(s), and measures involved?	Yes
Is accuracy matched across the second level contrast?	No, by design
Is reaction time matched across the second level contrast?	Unknown, not reported
Behavioral data notes	More words were correctly repeated with MIT (16.3 ± 8) than without (12.4 ± 8; $p < 0.03$)
Type of analysis	Regions of interest (ROI)
ROI type	Anatomical
How many ROIs are there?	18
What are the ROI(s)?	(1) L Broca's area; (2) L prefrontal; (3) L sensorimotor mouth; (4) L parietal; (5) L Wernicke's area; (6) L Heschl's gyrus; (7) L anterior STG; (8) L MTG; (9) L temporal pole; (10-18) homotopic counterparts
How are the ROI(s) defined?	Individual anatomical images; activation quantified as mean rCBF, not including any intersection of the infarct with the ROI
Correction for multiple comparisons	No correction
Statistical details	Three left hemisphere ROIs were excluded (3, 6, 9) because they were completely infarcted in 4 or more patients
Findings	↑ L IFG<br>↑ L dorsolateral prefrontal cortex<br>↓ R posterior STG
Findings notes	—

Notes

Excluded analyses	Two other contrasts are also reported, but do not fall within the scope of this review

Ohyama et al. (1996)

Reference

Authors	Ohyama M, Senda M, Kitamura S, Ishii K, Mishina M, Terashi A
Title	Role of the nondominant hemisphere and undamaged area during word repetition in poststroke aphasics: a PET activation study
Reference	*Stroke* 1996; 27: 897-903
PMID	8623110
DOI	10.1161/01.str.27.5.897

Participants

Language	Japanese
Inclusion criteria	Able to repeat single words
Number of individuals with aphasia	16
Number of control participants	6
Were any of the participants included in any previous studies?	No
Is age reported for patients and controls, and matched?	Yes (mean 56.6 ± 11.8 years, range 38-75 years)
Is sex reported for patients and controls, and matched?	Yes (males: 12; females: 4)
Is handedness reported for patients and controls, and matched?	Yes (right: 16; left: 0)
Is time post stroke onset reported and appropriate to the study design?	No* (moderate limitation) (mean 15.1 ± 16.7 months, range 1.1-50.3 months; a mix of subacute and chronic participants; 8 of each)

To what extent is the nature of aphasia characterized?	Comprehensive battery
Language evaluation	WAB
Aphasia severity	AQ mean 74.3 ± 12.2, range 53.8-92.4
Aphasia type	6 anomic, 4 atypical, 4 mild Broca's, 1 mild Wernicke's, 1 transcortical sensory; alternately: 10 fluent, 6 non-fluent
First stroke only?	Yes
Stroke type	Ischemic only
To what extent is the lesion distribution characterized?	Extent and location
Lesion extent	Mean 33.9 ± 26.3 cc, range 8.1-113.2 cc
Lesion location	L perisylvian
Participants notes	—

Imaging

Modality	PET (rCBF)
Is the study cross-sectional or longitudinal?	Cross-sectional
If longitudinal, at what time point(s) were imaging data acquired?	—
If longitudinal, was there any intervention between the time points?	—
Is the scanner described?	Yes (Headtome IV tomograph)
Is the timing of stimulus presentation and image acquisition clearly described and appropriate?	Yes
Design type	PET
Total images acquired	6
Are the imaging acquisition parameters, including coverage, adequately described and appropriate?	No (91 mm field of view; coverage limitations not stated)
Is preprocessing and intrasubject coregistration adequately described and appropriate?	Yes
Is first level model fitting adequately described and appropriate?	Yes
Is intersubject normalization adequately described and appropriate?	No (lesion impact not addressed)
Imaging notes	—

Conditions

Are the conditions clearly described?	Yes

Condition	Response type	Repetitions	All groups could do?	All individuals could do?
word repetition	Word (overt)	2	Yes	Yes
counting	Multiple words (overt)	2	Yes	Yes
rest	None	2	N/A	N/A

Conditions notes	Patients were able to repeat words well, with phonemic errors on no more than 4 out of 48 words; counting condition not analyzed in this paper

Contrasts

Are the contrasts clearly described?	Yes

Contrast 1: word repetition vs rest

Language condition	Word repetition
Control condition	Rest
Are the conditions matched for visual demands?	Yes
Are the conditions matched for auditory demands?	No
Are the conditions matched for motor demands?	No

Are the conditions matched for cognitive/executive demands?	No
Is accuracy matched between the language and control tasks for all relevant groups?	N/A, tasks not comparable
Is reaction time matched between the language and control tasks for all relevant groups?	N/A, tasks not comparable
Behavioral data notes	—
Are control data reported in this paper or another that is referenced?	Somewhat
Does the contrast selectively activate plausible relevant language regions in the control group?	Somewhat
Are activations lateralized in the control data?	No
Control activation notes	Bilateral auditory and motor activations are prominent, only slightly L-lateralized
Contrast notes	—

Analyses

Are the analyses clearly described?	No (see specific limitation(s) below)

ROI analysis 1

First level contrast	Word repetition vs rest
Analysis class	Cross-sectional aphasia vs control
Group(s)	Aphasia vs control
Covariate	—
Is the second level contrast valid in terms of the group(s), time point(s), and measures involved?	Yes
Is accuracy matched across the second level contrast?	Unknown, not reported
Is reaction time matched across the second level contrast?	Unknown, not reported
Behavioral data notes	Some of the patients made a few errors, so as a group they may have been less accurate than controls
Type of analysis	Regions of interest (ROI)
ROI type	Functional
How many ROIs are there?	7
What are the ROI(s)?	(1) L posterior inferior frontal; (2) R posterior inferior frontal; (3) L posterior superior temporal; (4) R posterior superior temporal; (5) L rolandic; (6) R rolandic; (7) SMA
How are the ROI(s) defined?	Spheres around control peaks
Correction for multiple comparisons	No correction
Statistical details	The rCBF increase in R PIF was also significant at $p < 0.005$ for nonfluent patients with Fisher's protected least-significant difference
Findings	↑ R IFG<br>↑ R posterior STG/STS/MTG
Findings notes	—

ROI analysis 2

First level contrast	Word repetition vs rest
Analysis class	Cross-sectional between two groups with aphasia
Group(s)	Aphasia fluent (n = 10) vs non-fluent (n = 6)
Covariate	—
Is the second level contrast valid in terms of the group(s), time point(s), and measures involved?	Yes
Is accuracy matched across the second level contrast?	Unknown, not reported
Is reaction time matched across the second level contrast?	Unknown, not reported
Behavioral data notes	—

Type of analysis	Regions of interest (ROI)
ROI type	Functional
How many ROIs are there?	7
What are the ROI(s)?	(1) L posterior inferior frontal; (2) R posterior inferior frontal; (3) L posterior superior temporal; (4) R posterior superior temporal; (5) L rolandic; (6) R rolandic; (7) SMA
How are the ROI(s) defined?	Spheres around control peaks
Correction for multiple comparisons	No correction
Statistical details	—
Findings	↓ R IFG
Findings notes	—

ROI analysis 3

First level contrast	Word repetition vs rest
Analysis class	Cross-sectional correlation with language or other measure
Group(s)	Aphasia
Covariate	Spontaneous speech (WAB)
Is the second level contrast valid in terms of the group(s), time point(s), and measures involved?	Yes
Is accuracy matched across the second level contrast?	Unknown, not reported
Is reaction time matched across the second level contrast?	Unknown, not reported
Behavioral data notes	—
Type of analysis	Regions of interest (ROI)
ROI type	Functional
How many ROIs are there?	7
What are the ROI(s)?	(1) L posterior inferior frontal; (2) R posterior inferior frontal; (3) L posterior superior temporal; (4) R posterior superior temporal; (5) L rolandic; (6) R rolandic; (7) SMA
How are the ROI(s) defined?	Spheres around control peaks
Correction for multiple comparisons	No correction
Statistical details	No correction for multiple comparisons across WAB subscores
Findings	↑ L IFG
Findings notes	—

ROI analysis 4

First level contrast	Word repetition vs rest
Analysis class	Cross-sectional correlation with language or other measure
Group(s)	Aphasia
Covariate	Comprehension (WAB)
Is the second level contrast valid in terms of the group(s), time point(s), and measures involved?	Yes
Is accuracy matched across the second level contrast?	Unknown, not reported
Is reaction time matched across the second level contrast?	Unknown, not reported
Behavioral data notes	—
Type of analysis	Regions of interest (ROI)
ROI type	Functional
How many ROIs are there?	7
What are the ROI(s)?	(1) L posterior inferior frontal; (2) R posterior inferior frontal; (3) L posterior superior temporal; (4) R posterior superior temporal; (5) L rolandic; (6) R rolandic; (7) SMA
How are the ROI(s) defined?	Spheres around control peaks
Correction for multiple comparisons	No correction
Statistical details	This non-significant finding is implied but not stated explicitly
Findings	None

Findings notes	—

ROI analysis 5

First level contrast	Word repetition vs rest
Analysis class	Cross-sectional correlation with language or other measure
Group(s)	Aphasia
Covariate	Repetition (WAB)
Is the second level contrast valid in terms of the group(s), time point(s), and measures involved?	Yes
Is accuracy matched across the second level contrast?	Unknown, not reported
Is reaction time matched across the second level contrast?	Unknown, not reported
Behavioral data notes	—
Type of analysis	Regions of interest (ROI)
ROI type	Functional
How many ROIs are there?	7
What are the ROI(s)?	(1) L posterior inferior frontal; (2) R posterior inferior frontal; (3) L posterior superior temporal; (4) R posterior superior temporal; (5) L rolandic; (6) R rolandic; (7) SMA
How are the ROI(s) defined?	Spheres around control peaks
Correction for multiple comparisons	No correction
Statistical details	This non-significant finding is implied but not stated explicitly
Findings	None
Findings notes	—

ROI analysis 6

First level contrast	Word repetition vs rest
Analysis class	Cross-sectional correlation with language or other measure
Group(s)	Aphasia
Covariate	Naming (WAB)
Is the second level contrast valid in terms of the group(s), time point(s), and measures involved?	Yes
Is accuracy matched across the second level contrast?	Unknown, not reported
Is reaction time matched across the second level contrast?	Unknown, not reported
Behavioral data notes	—
Type of analysis	Regions of interest (ROI)
ROI type	Functional
How many ROIs are there?	7
What are the ROI(s)?	(1) L posterior inferior frontal; (2) R posterior inferior frontal; (3) L posterior superior temporal; (4) R posterior superior temporal; (5) L rolandic; (6) R rolandic; (7) SMA
How are the ROI(s) defined?	Spheres around control peaks
Correction for multiple comparisons	No correction
Statistical details	This non-significant finding is implied but not stated explicitly
Findings	None
Findings notes	—

Notes

Excluded analyses	Separate analyses for fluent and non-fluent patients revealed essentially similar results

Heiss et al. (1997)

Reference

Authors	Heiss WD, Kessler J, Karbe H, Fink GR, Pawlik G
Title	Speech-induced cerebral metabolic activation reflects recovery from aphasia
Reference	*J Neurol Sci* 1997; 145: 213-217
PMID	9094051
DOI	10.1016/s0022-510x(96)00252-3

Participants

Language	German
Inclusion criteria	—
Number of individuals with aphasia	6
Number of control participants	6
Were any of the participants included in any previous studies?	No
Is age reported for patients and controls, and matched?	Yes (range 33-66 years)
Is sex reported for patients and controls, and matched?	Yes (males: 4; females: 2)
Is handedness reported for patients and controls, and matched?	Yes (right: 6; left: 0)
Is time post stroke onset reported and appropriate to the study design?	Yes (T1: ~4 weeks; T2: ~12-18 months)
To what extent is the nature of aphasia characterized?	Severity only
Language evaluation	Verbal repetition, confrontation naming, oral and written comprehension, reading abilities, TT, phonemic fluency, clinical impression, family interview
Aphasia severity	T1: TT range 37-48; T2: TT range 3-39 (1 missing)
Aphasia type	T1: 5 global, 1 Wernicke's; T2: not stated
First stroke only?	Yes
Stroke type	Ischemic only
To what extent is the lesion distribution characterized?	Individual lesions
Lesion extent	Range 27.2-133.2 cc
Lesion location	L MCA; 5 patients had superior temporal damage and 1 had subcortical damage underlying posterior superior temporal cortex
Participants notes	—

Imaging

Modality	PET (rCMRgl)
Is the study cross-sectional or longitudinal?	Longitudinal—recovery
If longitudinal, at what time point(s) were imaging data acquired?	T1: ~4 weeks; T2: ~12-18 months
If longitudinal, was there any intervention between the time points?	Not stated
Is the scanner described?	Yes (Siemens ECAT EXACT HR)
Is the timing of stimulus presentation and image acquisition clearly described and appropriate?	Yes
Design type	PET
Total images acquired	2
Are the imaging acquisition parameters, including coverage, adequately described and appropriate?	Yes (whole brain)
Is preprocessing and intrasubject coregistration adequately described and appropriate?	Yes
Is first level model fitting adequately described and appropriate?	Yes
Is intersubject normalization adequately described	N/A—no intersubject normalization

and appropriate?	
Imaging notes	—

Conditions

Are the conditions clearly described?	No (no information about repetition rate, or whether repetition was overt or covert)

Condition	Response type	Repetitions	All groups could do?	All individuals could do?
word repetition	Word (overt)	1	Unknown	Unknown
rest	None	1	N/A	N/A

Conditions notes	—

Contrasts

Are the contrasts clearly described?	Yes

Contrast 1: word repetition vs rest

Language condition	Word repetition
Control condition	Rest
Are the conditions matched for visual demands?	Yes
Are the conditions matched for auditory demands?	No
Are the conditions matched for motor demands?	No
Are the conditions matched for cognitive/executive demands?	No
Is accuracy matched between the language and control tasks for all relevant groups?	N/A, tasks not comparable
Is reaction time matched between the language and control tasks for all relevant groups?	N/A, tasks not comparable
Behavioral data notes	—
Are control data reported in this paper or another that is referenced?	Somewhat
Does the contrast selectively activate plausible relevant language regions in the control group?	Unknown
Are activations lateralized in the control data?	No
Control activation notes	The only control data is extent of activation and mean signal increase in L and R superior temporal cortex; both of these measures were slightly L-lateralized
Contrast notes	—

Analyses

Are the analyses clearly described?	Yes

Voxelwise analysis 1

First level contrast	Word repetition vs rest
Analysis class	Longitudinal between two groups with aphasia
Group(s)	(Aphasia with good recovery (n = 3) T2 vs T1) vs (aphasia with poor recovery (n = 3) T2 vs T1)
Covariate	—
Is the second level contrast valid in terms of the group(s), time point(s), and measures involved?	Somewhat (TT not optimal measure of overall language function)
Is accuracy matched across the second level contrast?	Unknown, not reported
Is reaction time matched across the second level contrast?	Unknown, not reported
Behavioral data notes	—
Type of analysis	Voxelwise
Search volume	Whole brain
Correction for multiple comparisons	No direct comparison

Software	not stated
Voxelwise p	—
Cluster extent	—
Statistical details	Qualitative generalization across individuals on pp. 214-6
Findings	↑ L posterior STG/STS/MTG<br>↓ R posterior STG/STS/MTG
Findings notes	The consistent aspects of the findings were that there was an emergence of L posterior temporal activation in patients with better recovery, and R posterior temporal activation in patients with worse recovery

ROI analysis 1

First level contrast	Word repetition vs rest
Analysis class	Longitudinal between two groups with aphasia
Group(s)	(Aphasia with good recovery (n = 3) T2 vs T1) vs (aphasia with poor recovery (n = 3) T2 vs T1)
Covariate	—
Is the second level contrast valid in terms of the group(s), time point(s), and measures involved?	Somewhat (TT not optimal measure of overall language function)
Is accuracy matched across the second level contrast?	Unknown, not reported
Is reaction time matched across the second level contrast?	Unknown, not reported
Behavioral data notes	—
Type of analysis	Regions of interest (ROI)
ROI type	Anatomical
How many ROIs are there?	2
What are the ROI(s)?	(1) L superior temporal cortex; (2) R superior temporal cortex
How are the ROI(s) defined?	Individual anatomical images; activation quantified in terms of extent exceeding 10% signal change, and mean % increase over the activation
Correction for multiple comparisons	No direct comparison
Statistical details	Qualitative generalization across individuals on pp. 214, 216
Findings	↑ L posterior STG/STS/MTG<br>↑ L Heschl's gyrus
Findings notes	—

Notes

Excluded analyses	—

Karbe et al. (1998)

Reference

Authors	Karbe H, Thiel A, Weber-Luxenburger G, Herholz K, Kessler J, Heiss WD
Title	Brain plasticity in poststroke aphasia: what is the contribution of the right hemisphere?
Reference	*Brain Lang* 1998; 64: 215-230
PMID	9710490
DOI	10.1006/brln.1998.1961

Participants

Language	German
Inclusion criteria	MCA; able to repeat single words
Number of individuals with aphasia	12
Number of control participants	10
Were any of the participants included in any previous studies?	No

Is age reported for patients and controls, and matched?	No (mean 57 years, range 34-78 years; controls not matched for age)
Is sex reported for patients and controls, and matched?	Yes (males: 7; females: 5; stated to be not matched, but difference not significant)
Is handedness reported for patients and controls, and matched?	Yes (right: 12; left: 0)
Is time post stroke onset reported and appropriate to the study design?	Yes (T1: mean 24 ± 11 days, ~3-4 weeks; T2: mean 19 ± 2 months, > 1 year)
To what extent is the nature of aphasia characterized?	Severity and type
Language evaluation	TT
Aphasia severity	T1: 9 severe; 2 mild; 1 not stated; TT range 3-47 errors; T2: not stated
Aphasia type	T1: 8 global, 3 anomic, 1 Wernicke's; T2: not stated
First stroke only?	Yes
Stroke type	Ischemic only
To what extent is the lesion distribution characterized?	Extent and location
Lesion extent	Range 2-133 cc
Lesion location	L MCA
Participants notes	Only 7 of the 12 patients took part at T2

Imaging

Modality	PET (rCMRgl)
Is the study cross-sectional or longitudinal?	Longitudinal—recovery
If longitudinal, at what time point(s) were imaging data acquired?	T1: mean 24 ± 11 days, ~3-4 weeks; T2: mean 19 ± 2 months, > 1 year
If longitudinal, was there any intervention between the time points?	Not stated
Is the scanner described?	Yes (CTI-Siemens ECAT EXACT HR)
Is the timing of stimulus presentation and image acquisition clearly described and appropriate?	No* (moderate limitation) (activation and control images not acquired on the same day; number of acquisitions not clearly described)
Design type	PET
Total images acquired	8
Are the imaging acquisition parameters, including coverage, adequately described and appropriate?	Yes (whole brain)
Is preprocessing and intrasubject coregistration adequately described and appropriate?	Yes
Is first level model fitting adequately described and appropriate?	Yes
Is intersubject normalization adequately described and appropriate?	N/A—no intersubject normalization
Imaging notes	—

Conditions

Are the conditions clearly described?	Yes

Condition	Response type	Repetitions	All groups could do?	All individuals could do?
word repetition	Word (overt)	4 (?)	Unknown	Unknown
rest	None	4 (?)	N/A	N/A

Conditions notes	Inability to repeat single words was an exclusion criterion, but many patients had severe aphasia so it is unclear how they would have performed

Contrasts

Are the contrasts clearly described?	Yes

Contrast 1: word repetition vs rest

Language condition	Word repetition
Control condition	Rest
Are the conditions matched for visual demands?	Yes
Are the conditions matched for auditory demands?	No
Are the conditions matched for motor demands?	No
Are the conditions matched for cognitive/executive demands?	No
Is accuracy matched between the language and control tasks for all relevant groups?	N/A, tasks not comparable
Is reaction time matched between the language and control tasks for all relevant groups?	N/A, tasks not comparable
Behavioral data notes	—
Are control data reported in this paper or another that is referenced?	Somewhat
Does the contrast selectively activate plausible relevant language regions in the control group?	No
Are activations lateralized in the control data?	No
Control activation notes	ROIs only; negligible evidence of lateralization
Contrast notes	—

Analyses

Are the analyses clearly described?	No* (moderate limitation) (see specific limitation(s) below)

ROI analysis 1

First level contrast	Word repetition vs rest
Analysis class	Cross-sectional aphasia vs control
Group(s)	Aphasia T1 vs control
Covariate	—
Is the second level contrast valid in terms of the group(s), time point(s), and measures involved?	Yes
Is accuracy matched across the second level contrast?	Unknown, not reported
Is reaction time matched across the second level contrast?	Unknown, not reported
Behavioral data notes	—
Type of analysis	Regions of interest (ROI)
ROI type	Anatomical
How many ROIs are there?	8
What are the ROI(s)?	(1) L IFG; (2) L STG/HG; (3) L SMA; (4) L ventral precentral; (5-8) homotopic counterparts
How are the ROI(s) defined?	Individual anatomical images
Correction for multiple comparisons	No direct comparison
Statistical details	Qualitative comparison on p. 219, but only the L SMA comparison is explicitly quantified
Findings	↑ L SMA/medial prefrontal<br>↑ R SMA/medial prefrontal<br>↓ L posterior STG<br>↓ L Heschl's gyrus
Findings notes	—

ROI analysis 2

First level contrast	Word repetition vs rest
Analysis class	Cross-sectional correlation with language or other measure
Group(s)	Aphasia (subset who returned for follow-up) T1 (n = 7)
Covariate	TT T1
Is the second level contrast valid in terms of the	Somewhat (TT not optimal measure of overall language function)

group(s), time point(s), and measures involved?	
Is accuracy matched across the second level contrast?	Unknown, not reported
Is reaction time matched across the second level contrast?	Unknown, not reported
Behavioral data notes	—
Type of analysis	Regions of interest (ROI)
ROI type	Anatomical
How many ROIs are there?	8
What are the ROI(s)?	(1) L IFG; (2) L STG/HG; (3) L SMA; (4) L ventral precentral; (5-8) homotopic counterparts
How are the ROI(s) defined?	Individual anatomical images
Correction for multiple comparisons	No correction
Statistical details	—
Findings	None
Findings notes	—

ROI analysis 3

First level contrast	Word repetition vs rest
Analysis class	Cross-sectional correlation with language or other measure
Group(s)	Aphasia (subset who returned for follow-up) T2 (n = 7)
Covariate	TT T2
Is the second level contrast valid in terms of the group(s), time point(s), and measures involved?	Somewhat (TT not optimal measure of overall language function)
Is accuracy matched across the second level contrast?	Unknown, not reported
Is reaction time matched across the second level contrast?	Unknown, not reported
Behavioral data notes	—
Type of analysis	Regions of interest (ROI)
ROI type	Anatomical
How many ROIs are there?	8
What are the ROI(s)?	(1) L IFG; (2) L STG/HG; (3) L SMA; (4) L ventral precentral; (5-8) homotopic counterparts
How are the ROI(s) defined?	Individual anatomical images
Correction for multiple comparisons	No correction
Statistical details	—
Findings	↓ L SMA/medial prefrontal<br>↓ R ventral precentral/inferior frontal junction<br>↓ R SMA/medial prefrontal<br>↓ R posterior STG<br>↓ R Heschl's gyrus
Findings notes	More activation in patients with more severe aphasia per TT

ROI analysis 4

First level contrast	Word repetition vs rest
Analysis class	Longitudinal correlation with language or other measure
Group(s)	Aphasia (subset who returned for follow-up) (n = 7) T2 vs T1
Covariate	Subsequent outcome (T2) TT
Is the second level contrast valid in terms of the group(s), time point(s), and measures involved?	No (the logic behind correlating activation changes and language outcome is unclear; TT not optimal measure of overall language function)
Is accuracy matched across the second level contrast?	Unknown, not reported
Is reaction time matched across the second level contrast?	Unknown, not reported
Behavioral data notes	—
Type of analysis	Region of interest (ROI)
ROI type	Anatomical

How many ROIs are there?	1
What are the ROI(s)?	L STG/HG
How are the ROI(s) defined?	Individual anatomical images
Correction for multiple comparisons	One only
Statistical details	—
Findings	↑ L posterior STG<br>↑ L Heschl's gyrus
Findings notes	Increase in activation for repetition was correlated with better aphasia outcome per TT

ROI analysis 5

First level contrast	Word repetition vs rest
Analysis class	Cross-sectional correlation with language or other measure
Group(s)	Aphasia (subset who returned for follow-up) T2 (n = 7)
Covariate	Previous Δ (T2 vs T1) activation in L STG/HG
Is the second level contrast valid in terms of the group(s), time point(s), and measures involved?	No (logically problematic because patients with less severe initial aphasia would also be expected to show little L temporal increase, but would not be expected to show R temporal recruitment)
Is accuracy matched across the second level contrast?	Unknown, not reported
Is reaction time matched across the second level contrast?	Unknown, not reported
Behavioral data notes	—
Type of analysis	Regions of interest (ROI)
ROI type	Anatomical
How many ROIs are there?	4
What are the ROI(s)?	(1) R IFG; (2) R STG/HG; (3) R SMA; (4) R ventral precentral
How are the ROI(s) defined?	Individual anatomical images
Correction for multiple comparisons	No correction
Statistical details	—
Findings	↓ R IFG<br>↓ R ventral precentral/inferior frontal junction<br>↓ R SMA/medial prefrontal<br>↓ R posterior STG<br>↓ R Heschl's gyrus
Findings notes	Patients with more increase in L STG/HG activation showed less activation of R hemisphere regions at T2

Notes

Excluded analyses	The "Initial study" columns of table 3, because they are not described in the text and it is not clear exactly what is being correlated with what

Cao et al. (1999)

Reference

Authors	Cao Y, Vikingstad EM, George KP, Johnson AF, Welch KM
Title	Cortical language activation in stroke patients recovering from aphasia with functional MRI
Reference	*Stroke* 1999; 30: 2331-2340
PMID	10548667
DOI	10.1161/01.str.30.11.2331

Participants

Language	US English
Inclusion criteria	Aphasia with significant recovery over months to years (ADPASS > 70th percentile)

Number of individuals with aphasia	6 (plus 2 excluded: 1 unable to reliably describe performance post-scan; 1 due to head motion)
Number of control participants	37
Were any of the participants included in any previous studies?	No
Is age reported for patients and controls, and matched?	Yes (range 20-56 years)
Is sex reported for patients and controls, and matched?	Yes (males: 1; females: 5)
Is handedness reported for patients and controls, and matched?	Yes (right: 6; left: 0)
Is time post stroke onset reported and appropriate to the study design?	Yes (range 5-32 months)
To what extent is the nature of aphasia characterized?	Severity and type
Language evaluation	ADP
Aphasia severity	ADPASS percentile range 73-99
Aphasia type	3 anomic, 1 conduction, 1 recovered, 1 transcortical sensory
First stroke only?	Yes
Stroke type	Ischemic only
To what extent is the lesion distribution characterized?	Individual lesions
Lesion extent	Extents are reported in three dimensions
Lesion location	4 L MCA, 2 L ICA
Participants notes	—

Imaging

Modality	fMRI
Is the study cross-sectional or longitudinal?	Cross-sectional
If longitudinal, at what time point(s) were imaging data acquired?	—
If longitudinal, was there any intervention between the time points?	—
Is the scanner described?	Yes (Magnex Scientific 3 Tesla)
Is the timing of stimulus presentation and image acquisition clearly described and appropriate?	Yes
Design type	Block
Total images acquired	40
Are the imaging acquisition parameters, including coverage, adequately described and appropriate?	Yes (axial, perisylvian only)
Is preprocessing and intrasubject coregistration adequately described and appropriate?	Yes
Is first level model fitting adequately described and appropriate?	No (first level cross-correlation analysis unclear)
Is intersubject normalization adequately described and appropriate?	N/A—no intersubject normalization
Imaging notes	—

Conditions

Are the conditions clearly described?	Yes

Condition	Response type	Repetitions	All groups could do?	All individuals could do?
picture naming	Word (covert)	4	Yes	Yes
viewing nonsense drawings	None	4	N/A	N/A

Conditions notes	—

Contrasts

Are the contrasts clearly described?	Yes

Contrast 1: picture naming vs viewing nonsense drawings

Language condition	Picture naming
Control condition	Viewing nonsense drawings
Are the conditions matched for visual demands?	Yes
Are the conditions matched for auditory demands?	Yes
Are the conditions matched for motor demands?	Yes
Are the conditions matched for cognitive/executive demands?	No
Is accuracy matched between the language and control tasks for all relevant groups?	N/A, tasks not comparable
Is reaction time matched between the language and control tasks for all relevant groups?	N/A, tasks not comparable
Behavioral data notes	—
Are control data reported in this paper or another that is referenced?	Somewhat
Does the contrast selectively activate plausible relevant language regions in the control group?	Unknown
Are activations lateralized in the control data?	Somewhat
Control activation notes	Insufficient data to assess the control activation pattern
Contrast notes	—

Analyses

Are the analyses clearly described?	Yes

ROI analysis 1

First level contrast	Picture naming vs viewing nonsense drawings
Analysis class	Cross-sectional aphasia vs control
Group(s)	Aphasia vs control
Covariate	—
Is the second level contrast valid in terms of the group(s), time point(s), and measures involved?	Yes
Is accuracy matched across the second level contrast?	Unknown, not reported
Is reaction time matched across the second level contrast?	Unknown, not reported
Behavioral data notes	—
Type of analysis	Regions of interest (ROI)
ROI type	Mixed
How many ROIs are there?	6
What are the ROI(s)?	(1) L IFG and MFG; (2) L pSTG, AG and SMG; (3) R IFG and MFG; (4) R pSTG, AG and SMG; (5) frontal LI; (6) temporal LI
How are the ROI(s) defined?	(1-4) individual anatomical images; activation quantified in terms of extent
Correction for multiple comparisons	No correction
Statistical details	—
Findings	↑ R IFG<br>↑ R dorsolateral prefrontal cortex<br>↑ R supramarginal gyrus<br>↑ R angular gyrus<br>↑ R posterior STG<br>↓ LI (frontal)<br>↓ LI (temporal)
Findings notes	—

ROI analysis 2

First level contrast	Picture naming vs viewing nonsense drawings
Analysis class	Cross-sectional correlation with language or other measure
Group(s)	Aphasia
Covariate	Picture naming (outside scanner)
Is the second level contrast valid in terms of the group(s), time point(s), and measures involved?	Yes
Is accuracy matched across the second level contrast?	Unknown, not reported
Is reaction time matched across the second level contrast?	Unknown, not reported
Behavioral data notes	—
Type of analysis	Regions of interest (ROI)
ROI type	Mixed
How many ROIs are there?	6
What are the ROI(s)?	(1) L IFG and MFG; (2) L pSTG, AG and SMG; (3) R IFG and MFG; (4) R pSTG, AG and SMG; (5) frontal LI; (6) temporal LI
How are the ROI(s) defined?	(1-4) individual anatomical images; activation quantified in terms of extent
Correction for multiple comparisons	No correction
Statistical details	—
Findings	↑ LI (frontal)
Findings notes	—

Notes

Excluded analyses	(1) verb generation study with n = 4 patients; (2) individual patient results; (3) whole brain and whole hemisphere activation measures

Heiss et al. (1999)

Reference

Authors	Heiss WD, Kessler J, Thiel A, Ghaemi M, Karbe H
Title	Differential capacity of left and right hemispheric areas for compensation of poststroke aphasia
Reference	*Ann Neurol* 1999; 45: 430-438
PMID	10211466
DOI	10.1002/1531-8249(199904)45:4<430::aid-ana3>3.0.co;2-p

Participants

Language	German
Inclusion criteria	AAT repetition ≥ 50
Number of individuals with aphasia	23
Number of control participants	11
Were any of the participants included in any previous studies?	No
Is age reported for patients and controls, and matched?	Yes (mean 56 ± 12 years, range 31-77 years; assume patient's age of 5.6 years is a typo for 56 years)
Is sex reported for patients and controls, and matched?	Yes (males: 15; females: 8)
Is handedness reported for patients and controls, and matched?	Yes (right: 23; left: 0)
Is time post stroke onset reported and appropriate to the study design?	Yes (T1: ~2 weeks; T2: ~8 weeks)
To what extent is the nature of aphasia	Severity and type

characterized?	
Language evaluation	AAT, phonemic fluency
Aphasia severity	T1: subcortical: TT median 8 errors, range 0-17 errors; frontal: TT median 21 errors, range 4-40 errors; temporal: TT median 39 errors, range 1-47 errors; T2: subcortical: TT median 1 error, range 0-14 errors; frontal: TT median 8 errors, range 0-34; temporal: TT median 34 errors, range 0-44 errors
Aphasia type	T1: 6 Wernicke's, 5 Broca's, 5 residual aphasia, 4 anomic, 2 transcortical sensory, 1 conduction; T2: not stated
First stroke only?	Yes
Stroke type	Ischemic only
To what extent is the lesion distribution characterized?	Extent and location
Lesion extent	Range 4.3-154.3 cc (probably; units not stated)
Lesion location	L MCA; 9 subcortical, 7 frontal, 7 temporal
Participants notes	—

Imaging

Modality	PET (rCBF)
Is the study cross-sectional or longitudinal?	Longitudinal—recovery
If longitudinal, at what time point(s) were imaging data acquired?	T1: ~2 weeks; T2: ~8 weeks
If longitudinal, was there any intervention between the time points?	Not stated
Is the scanner described?	Yes (CTI-Siemens ECAT EXACT HR)
Is the timing of stimulus presentation and image acquisition clearly described and appropriate?	Yes
Design type	PET
Total images acquired	8
Are the imaging acquisition parameters, including coverage, adequately described and appropriate?	Yes (whole brain)
Is preprocessing and intrasubject coregistration adequately described and appropriate?	Yes
Is first level model fitting adequately described and appropriate?	Yes
Is intersubject normalization adequately described and appropriate?	N/A—no intersubject normalization
Imaging notes	—

Conditions

Are the conditions clearly described?	Yes

Condition	Response type	Repetitions	All groups could do?	All individuals could do?
noun repetition	Word (overt)	4	Unknown	Unknown
rest	None	4	N/A	N/A

Conditions notes	Inclusion criterion would suggest all patients could do the task, but this is not stated

Contrasts

Are the contrasts clearly described?	Yes

Contrast 1: noun repetition vs rest

Language condition	Noun repetition
Control condition	Rest
Are the conditions matched for visual demands?	Yes
Are the conditions matched for auditory demands?	No
Are the conditions matched for motor demands?	No

Are the conditions matched for cognitive/executive demands?	No
Is accuracy matched between the language and control tasks for all relevant groups?	N/A, tasks not comparable
Is reaction time matched between the language and control tasks for all relevant groups?	N/A, tasks not comparable
Behavioral data notes	—
Are control data reported in this paper or another that is referenced?	Somewhat
Does the contrast selectively activate plausible relevant language regions in the control group?	Somewhat
Are activations lateralized in the control data?	Somewhat
Control activation notes	L frontal and bilateral temporal
Contrast notes	—

Analyses

Are the analyses clearly described?	Yes

ROI analysis 1

First level contrast	Noun repetition vs rest
Analysis class	Longitudinal change in aphasia
Group(s)	Aphasia with subcortical damage (n = 9) T2 vs T1
Covariate	—
Is the second level contrast valid in terms of the group(s), time point(s), and measures involved?	Yes
Is accuracy matched across the second level contrast?	Unknown, not reported
Is reaction time matched across the second level contrast?	Unknown, not reported
Behavioral data notes	—
Type of analysis	Regions of interest (ROI)
ROI type	Anatomical
How many ROIs are there?	14
What are the ROI(s)?	(1) L IFG pars opercularis; (2) L IFG pars triangularis; (3) L ventral precentral gyrus; (4) L Heschl's gyrus; (5) L temporal plane (posterior to HG, coded as posterior STG); (6) L posterior STG (coded as mid STG per Fig. 2); (7) L SMA; (8-14) homotopic counterparts
How are the ROI(s) defined?	Individual anatomical images
Correction for multiple comparisons	No direct comparison
Statistical details	Qualitative comparison on p. 434
Findings	↑ L mid temporal<br>↑ R Heschl's gyrus<br>↓ R IFG pars opercularis
Findings notes	—

ROI analysis 2

First level contrast	Noun repetition vs rest
Analysis class	Longitudinal change in aphasia
Group(s)	Aphasia with frontal damage (n = 7) T2 vs T1
Covariate	—
Is the second level contrast valid in terms of the group(s), time point(s), and measures involved?	Yes
Is accuracy matched across the second level contrast?	Unknown, not reported
Is reaction time matched across the second level contrast?	Unknown, not reported
Behavioral data notes	—

Type of analysis	Regions of interest (ROI)
ROI type	Anatomical
How many ROIs are there?	14
What are the ROI(s)?	(1) L IFG pars opercularis; (2) L IFG pars triangularis; (3) L ventral precentral gyrus; (4) L Heschl's gyrus; (5) L temporal plane (posterior to HG, coded as posterior STG); (6) L posterior STG (coded as mid STG per Fig. 2); (7) L SMA; (8-14) homotopic counterparts
How are the ROI(s) defined?	Individual anatomical images
Correction for multiple comparisons	No direct comparison
Statistical details	Qualitative comparison on p. 434
Findings	↑ L posterior STG<br>↑ L mid temporal<br>↑ R Heschl's gyrus<br>↓ R IFG pars opercularis
Findings notes	—

ROI analysis 3

First level contrast	Noun repetition vs rest
Analysis class	Longitudinal change in aphasia
Group(s)	Aphasia with temporal damage (n = 7) T2 vs T1
Covariate	—
Is the second level contrast valid in terms of the group(s), time point(s), and measures involved?	Yes
Is accuracy matched across the second level contrast?	Unknown, not reported
Is reaction time matched across the second level contrast?	Unknown, not reported
Behavioral data notes	—
Type of analysis	Regions of interest (ROI)
ROI type	Anatomical
How many ROIs are there?	14
What are the ROI(s)?	(1) L IFG pars opercularis; (2) L IFG pars triangularis; (3) L ventral precentral gyrus; (4) L Heschl's gyrus; (5) L temporal plane (posterior to HG, coded as posterior STG); (6) L posterior STG (coded as mid STG per Fig. 2); (7) L SMA; (8-14) homotopic counterparts
How are the ROI(s) defined?	Individual anatomical images
Correction for multiple comparisons	No direct comparison
Statistical details	Qualitative comparison on p. 434
Findings	↑ L ventral precentral/inferior frontal junction<br>↑ L SMA/medial prefrontal<br>↑ R ventral precentral/inferior frontal junction<br>↑ R mid temporal<br>↓ R SMA/medial prefrontal
Findings notes	—

ROI analysis 4

First level contrast	Noun repetition vs rest
Analysis class	Cross-sectional between two groups with aphasia
Group(s)	Aphasia with temporal damage T1 (n = 7) vs with subcortical damage T1 (n = 9)
Covariate	—
Is the second level contrast valid in terms of the group(s), time point(s), and measures involved?	Yes
Is accuracy matched across the second level contrast?	Unknown, not reported
Is reaction time matched across the second level contrast?	Unknown, not reported
Behavioral data notes	—
Type of analysis	Regions of interest (ROI)

ROI type	Anatomical
How many ROIs are there?	14
What are the ROI(s)?	(1) L IFG pars opercularis; (2) L IFG pars triangularis; (3) L ventral precentral gyrus; (4) L Heschl's gyrus; (5) L temporal plane (posterior to HG, coded as posterior STG); (6) L posterior STG (coded as mid STG per Fig. 2); (7) L SMA; (8-14) homotopic counterparts
How are the ROI(s) defined?	Individual anatomical images
Correction for multiple comparisons	No direct comparison
Statistical details	Qualitative comparison on p. 434
Findings	↑ L IFG pars opercularis<br>↑ R SMA/medial prefrontal<br>↓ L posterior STG<br>↓ R IFG pars opercularis<br>↓ R posterior STG<br>↓ R mid temporal
Findings notes	—

ROI analysis 5

First level contrast	Noun repetition vs rest
Analysis class	Cross-sectional between two groups with aphasia
Group(s)	Aphasia with temporal damage T1 (n = 7) vs with frontal damage T1 (n = 7)
Covariate	—
Is the second level contrast valid in terms of the group(s), time point(s), and measures involved?	Yes
Is accuracy matched across the second level contrast?	Unknown, not reported
Is reaction time matched across the second level contrast?	Unknown, not reported
Behavioral data notes	—
Type of analysis	Regions of interest (ROI)
ROI type	Anatomical
How many ROIs are there?	14
What are the ROI(s)?	(1) L IFG pars opercularis; (2) L IFG pars triangularis; (3) L ventral precentral gyrus; (4) L Heschl's gyrus; (5) L temporal plane (posterior to HG, coded as posterior STG); (6) L posterior STG (coded as mid STG per Fig. 2); (7) L SMA; (8-14) homotopic counterparts
How are the ROI(s) defined?	Individual anatomical images
Correction for multiple comparisons	No direct comparison
Statistical details	Qualitative comparison on p. 434
Findings	↑ L IFG pars opercularis<br>↑ R SMA/medial prefrontal<br>↓ R IFG pars opercularis<br>↓ R posterior STG<br>↓ R mid temporal
Findings notes	—

ROI analysis 6

First level contrast	Noun repetition vs rest
Analysis class	Cross-sectional between two groups with aphasia
Group(s)	Aphasia with temporal damage T2 (n = 7) vs with subcortical damage T2 (n = 9)
Covariate	—
Is the second level contrast valid in terms of the group(s), time point(s), and measures involved?	Yes
Is accuracy matched across the second level contrast?	Unknown, not reported
Is reaction time matched across the second level contrast?	Unknown, not reported
Behavioral data notes	—
Type of analysis	Regions of interest (ROI)

ROI type	Anatomical
How many ROIs are there?	14
What are the ROI(s)?	(1) L IFG pars opercularis; (2) L IFG pars triangularis; (3) L ventral precentral gyrus; (4) L Heschl's gyrus; (5) L temporal plane (posterior to HG, coded as posterior STG); (6) L posterior STG (coded as mid STG per Fig. 2); (7) L SMA; (8-14) homotopic counterparts
How are the ROI(s) defined?	Individual anatomical images
Correction for multiple comparisons	No direct comparison
Statistical details	Qualitative comparison on p. 434
Findings	↑ L IFG pars opercularis<br>↑ L ventral precentral/inferior frontal junction<br>↑ L SMA/medial prefrontal<br>↑ R ventral precentral/inferior frontal junction<br>↓ L posterior STG<br>↓ L mid temporal<br>↓ R posterior STG<br>↓ R Heschl's gyrus
Findings notes	—

ROI analysis 7

First level contrast	Noun repetition vs rest
Analysis class	Cross-sectional between two groups with aphasia
Group(s)	Aphasia with temporal damage T2 (n = 7) vs with frontal damage T2 (n = 7)
Covariate	—
Is the second level contrast valid in terms of the group(s), time point(s), and measures involved?	Yes
Is accuracy matched across the second level contrast?	Unknown, not reported
Is reaction time matched across the second level contrast?	Unknown, not reported
Behavioral data notes	—
Type of analysis	Regions of interest (ROI)
ROI type	Anatomical
How many ROIs are there?	14
What are the ROI(s)?	(1) L IFG pars opercularis; (2) L IFG pars triangularis; (3) L ventral precentral gyrus; (4) L Heschl's gyrus; (5) L temporal plane (posterior to HG, coded as posterior STG); (6) L posterior STG (coded as mid STG per Fig. 2); (7) L SMA; (8-14) homotopic counterparts
How are the ROI(s) defined?	Individual anatomical images
Correction for multiple comparisons	No direct comparison
Statistical details	Qualitative comparison on p. 434
Findings	↑ L IFG pars opercularis<br>↑ L ventral precentral/inferior frontal junction<br>↑ L SMA/medial prefrontal<br>↑ R ventral precentral/inferior frontal junction<br>↓ L posterior STG<br>↓ L mid temporal<br>↓ R posterior STG<br>↓ R Heschl's gyrus
Findings notes	—

ROI analysis 8

First level contrast	Noun repetition vs rest
Analysis class	Cross-sectional aphasia vs control
Group(s)	Aphasia with subcortical damage T1 (n = 9) vs control
Covariate	—
Is the second level contrast valid in terms of the group(s), time point(s), and measures involved?	Yes
Is accuracy matched across the second level	Unknown, not reported

contrast?	
Is reaction time matched across the second level contrast?	Unknown, not reported
Behavioral data notes	—
Type of analysis	Regions of interest (ROI)
ROI type	Anatomical
How many ROIs are there?	14
What are the ROI(s)?	(1) L IFG pars opercularis; (2) L IFG pars triangularis; (3) L ventral precentral gyrus; (4) L Heschl's gyrus; (5) L temporal plane (posterior to HG, coded as posterior STG); (6) L posterior STG (coded as mid STG per Fig. 2); (7) L SMA; (8-14) homotopic counterparts
How are the ROI(s) defined?	Individual anatomical images
Correction for multiple comparisons	No direct comparison
Statistical details	Qualitative comparison on p. 434
Findings	↑ R IFG pars opercularis<br>↓ L IFG<br>↓ L ventral precentral/inferior frontal junction<br>↓ L Heschl's gyrus<br>↓ L mid temporal<br>↓ R Heschl's gyrus
Findings notes	—

ROI analysis 9

First level contrast	Noun repetition vs rest
Analysis class	Cross-sectional aphasia vs control
Group(s)	Aphasia with frontal damage T1 (n = 7) vs control
Covariate	—
Is the second level contrast valid in terms of the group(s), time point(s), and measures involved?	Yes
Is accuracy matched across the second level contrast?	Unknown, not reported
Is reaction time matched across the second level contrast?	Unknown, not reported
Behavioral data notes	—
Type of analysis	Regions of interest (ROI)
ROI type	Anatomical
How many ROIs are there?	14
What are the ROI(s)?	(1) L IFG pars opercularis; (2) L IFG pars triangularis; (3) L ventral precentral gyrus; (4) L Heschl's gyrus; (5) L temporal plane (posterior to HG, coded as posterior STG); (6) L posterior STG (coded as mid STG per Fig. 2); (7) L SMA; (8-14) homotopic counterparts
How are the ROI(s) defined?	Individual anatomical images
Correction for multiple comparisons	No direct comparison
Statistical details	Qualitative comparison on p. 434
Findings	↑ R IFG pars opercularis<br>↓ L IFG pars opercularis<br>↓ L ventral precentral/inferior frontal junction<br>↓ L posterior STG/STS/MTG<br>↓ L Heschl's gyrus<br>↓ L mid temporal<br>↓ R Heschl's gyrus
Findings notes	—

ROI analysis 10

First level contrast	Noun repetition vs rest
Analysis class	Cross-sectional aphasia vs control
Group(s)	Aphasia with temporal damage T1 (n = 7) vs control
Covariate	—
Is the second level contrast valid in terms of the	Yes

group(s), time point(s), and measures involved?	
Is accuracy matched across the second level contrast?	Unknown, not reported
Is reaction time matched across the second level contrast?	Unknown, not reported
Behavioral data notes	—
Type of analysis	Regions of interest (ROI)
ROI type	Anatomical
How many ROIs are there?	14
What are the ROI(s)?	(1) L IFG pars opercularis; (2) L IFG pars triangularis; (3) L ventral precentral gyrus; (4) L Heschl's gyrus; (5) L temporal plane (posterior to HG, coded as posterior STG); (6) L posterior STG (coded as mid STG per Fig. 2); (7) L SMA; (8-14) homotopic counterparts
How are the ROI(s) defined?	Individual anatomical images
Correction for multiple comparisons	No direct comparison
Statistical details	Qualitative comparison on p. 434; L IFG pars opercularis noted as different in text despite being significant in both groups
Findings	↑ L IFG pars opercularis<br>↑ R SMA/medial prefrontal<br>↓ L ventral precentral/inferior frontal junction<br>↓ L posterior STG<br>↓ L Heschl's gyrus<br>↓ L mid temporal<br>↓ R posterior STG<br>↓ R Heschl's gyrus<br>↓ R mid temporal
Findings notes	—

ROI analysis 11

First level contrast	Noun repetition vs rest
Analysis class	Cross-sectional aphasia vs control
Group(s)	Aphasia with subcortical damage T2 (n = 9) vs control
Covariate	—
Is the second level contrast valid in terms of the group(s), time point(s), and measures involved?	Yes
Is accuracy matched across the second level contrast?	Unknown, not reported
Is reaction time matched across the second level contrast?	Unknown, not reported
Behavioral data notes	—
Type of analysis	Regions of interest (ROI)
ROI type	Anatomical
How many ROIs are there?	14
What are the ROI(s)?	(1) L IFG pars opercularis; (2) L IFG pars triangularis; (3) L ventral precentral gyrus; (4) L Heschl's gyrus; (5) L temporal plane (posterior to HG, coded as posterior STG); (6) L posterior STG (coded as mid STG per Fig. 2); (7) L SMA; (8-14) homotopic counterparts
How are the ROI(s) defined?	Individual anatomical images
Correction for multiple comparisons	No direct comparison
Statistical details	Qualitative comparison on p. 434
Findings	↓ L IFG pars opercularis<br>↓ L ventral precentral/inferior frontal junction<br>↓ L Heschl's gyrus
Findings notes	—

ROI analysis 12

First level contrast	Noun repetition vs rest
Analysis class	Cross-sectional aphasia vs control
Group(s)	Aphasia with frontal damage T2 (n = 7) vs control

Covariate	—
Is the second level contrast valid in terms of the group(s), time point(s), and measures involved?	Yes
Is accuracy matched across the second level contrast?	Unknown, not reported
Is reaction time matched across the second level contrast?	Unknown, not reported
Behavioral data notes	—
Type of analysis	Regions of interest (ROI)
ROI type	Anatomical
How many ROIs are there?	14
What are the ROI(s)?	(1) L IFG pars opercularis; (2) L IFG pars triangularis; (3) L ventral precentral gyrus; (4) L Heschl's gyrus; (5) L temporal plane (posterior to HG, coded as posterior STG); (6) L posterior STG (coded as mid STG per Fig. 2); (7) L SMA; (8-14) homotopic counterparts
How are the ROI(s) defined?	Individual anatomical images
Correction for multiple comparisons	No direct comparison
Statistical details	Qualitative comparison on p. 434
Findings	↓ L IFG pars opercularis<br>↓ L ventral precentral/inferior frontal junction<br>↓ L Heschl's gyrus
Findings notes	—

ROI analysis 13

First level contrast	Noun repetition vs rest
Analysis class	Cross-sectional aphasia vs control
Group(s)	Aphasia with temporal damage T2 (n = 7) vs control
Covariate	—
Is the second level contrast valid in terms of the group(s), time point(s), and measures involved?	Yes
Is accuracy matched across the second level contrast?	Unknown, not reported
Is reaction time matched across the second level contrast?	Unknown, not reported
Behavioral data notes	—
Type of analysis	Regions of interest (ROI)
ROI type	Anatomical
How many ROIs are there?	14
What are the ROI(s)?	(1) L IFG pars opercularis; (2) L IFG pars triangularis; (3) L ventral precentral gyrus; (4) L Heschl's gyrus; (5) L temporal plane (posterior to HG, coded as posterior STG); (6) L posterior STG (coded as mid STG per Fig. 2); (7) L SMA; (8-14) homotopic counterparts
How are the ROI(s) defined?	Individual anatomical images
Correction for multiple comparisons	No direct comparison
Statistical details	Qualitative comparison on p. 434
Findings	↑ L IFG pars opercularis<br>↑ L SMA/medial prefrontal<br>↑ R ventral precentral/inferior frontal junction<br>↓ L posterior STG<br>↓ L Heschl's gyrus<br>↓ L mid temporal<br>↓ R posterior STG<br>↓ R Heschl's gyrus
Findings notes	—

ROI analysis 14

First level contrast	Noun repetition vs rest
Analysis class	Longitudinal change in aphasia
Group(s)	Aphasia with subcortical or frontal damage and good recovery (n = 11) T2 vs T1

Covariate	—
Is the second level contrast valid in terms of the group(s), time point(s), and measures involved?	Yes
Is accuracy matched across the second level contrast?	Unknown, not reported
Is reaction time matched across the second level contrast?	Unknown, not reported
Behavioral data notes	—
Type of analysis	Regions of interest (ROI)
ROI type	Anatomical
How many ROIs are there?	14
What are the ROI(s)?	(1) L IFG pars opercularis; (2) L IFG pars triangularis; (3) L ventral precentral gyrus; (4) L Heschl's gyrus; (5) L temporal plane (posterior to HG, coded as posterior STG); (6) L posterior STG (coded as mid STG per Fig. 2); (7) L SMA; (8-14) homotopic counterparts
How are the ROI(s) defined?	Individual anatomical images
Correction for multiple comparisons	No direct comparison
Statistical details	Qualitative comparison on pp. 434-5
Findings	↑ L SMA/medial prefrontal<br>↑ L Heschl's gyrus<br>↑ R ventral precentral/inferior frontal junction<br>↑ R SMA/medial prefrontal<br>↑ R Heschl's gyrus<br>↓ R IFG pars opercularis
Findings notes	—

ROI analysis 15

First level contrast	Noun repetition vs rest
Analysis class	Longitudinal change in aphasia
Group(s)	Aphasia with subcortical or frontal damage and poor recovery (n = 5) T2 vs T1
Covariate	—
Is the second level contrast valid in terms of the group(s), time point(s), and measures involved?	Yes
Is accuracy matched across the second level contrast?	Unknown, not reported
Is reaction time matched across the second level contrast?	Unknown, not reported
Behavioral data notes	—
Type of analysis	Regions of interest (ROI)
ROI type	Anatomical
How many ROIs are there?	14
What are the ROI(s)?	(1) L IFG pars opercularis; (2) L IFG pars triangularis; (3) L ventral precentral gyrus; (4) L Heschl's gyrus; (5) L temporal plane (posterior to HG, coded as posterior STG); (6) L posterior STG (coded as mid STG per Fig. 2); (7) L SMA; (8-14) homotopic counterparts
How are the ROI(s) defined?	Individual anatomical images
Correction for multiple comparisons	No direct comparison
Statistical details	Qualitative comparison on pp. 434-5
Findings	↑ L ventral precentral/inferior frontal junction<br>↑ R Heschl's gyrus<br>↓ R IFG pars opercularis
Findings notes	—

ROI analysis 16

First level contrast	Noun repetition vs rest
Analysis class	Cross-sectional between two groups with aphasia
Group(s)	Aphasia with subcortical and frontal damage and good recovery T1 (n = 11) vs with subcortical and frontal damage and poor recovery T1 (n = 5)

Covariate	—
Is the second level contrast valid in terms of the group(s), time point(s), and measures involved?	Yes
Is accuracy matched across the second level contrast?	Unknown, not reported
Is reaction time matched across the second level contrast?	Unknown, not reported
Behavioral data notes	—
Type of analysis	Regions of interest (ROI)
ROI type	Anatomical
How many ROIs are there?	14
What are the ROI(s)?	(1) L IFG pars opercularis; (2) L IFG pars triangularis; (3) L ventral precentral gyrus; (4) L Heschl's gyrus; (5) L temporal plane (posterior to HG, coded as posterior STG); (6) L posterior STG (coded as mid STG per Fig. 2); (7) L SMA; (8-14) homotopic counterparts
How are the ROI(s) defined?	Individual anatomical images
Correction for multiple comparisons	No direct comparison
Statistical details	Qualitative comparison on p. 435
Findings	↑ L posterior STG<br>↑ L mid temporal
Findings notes	—

ROI analysis 17

First level contrast	Noun repetition vs rest
Analysis class	Cross-sectional between two groups with aphasia
Group(s)	Aphasia with subcortical and frontal damage and good recovery T2 (n = 11) vs with subcortical and frontal damage and poor recovery T2 (n = 5)
Covariate	—
Is the second level contrast valid in terms of the group(s), time point(s), and measures involved?	Yes
Is accuracy matched across the second level contrast?	Unknown, not reported
Is reaction time matched across the second level contrast?	Unknown, not reported
Behavioral data notes	—
Type of analysis	Regions of interest (ROI)
ROI type	Anatomical
How many ROIs are there?	14
What are the ROI(s)?	(1) L IFG pars opercularis; (2) L IFG pars triangularis; (3) L ventral precentral gyrus; (4) L Heschl's gyrus; (5) L temporal plane (posterior to HG, coded as posterior STG); (6) L posterior STG (coded as mid STG per Fig. 2); (7) L SMA; (8-14) homotopic counterparts
How are the ROI(s) defined?	Individual anatomical images
Correction for multiple comparisons	No direct comparison
Statistical details	Qualitative comparison on p. 435
Findings	↑ L SMA/medial prefrontal<br>↑ L posterior STG<br>↑ L Heschl's gyrus<br>↑ L mid temporal<br>↑ R ventral precentral/inferior frontal junction<br>↑ R SMA/medial prefrontal<br>↓ L ventral precentral/inferior frontal junction
Findings notes	—

Notes

Excluded analyses	—

Kessler et al. (2000)

Reference

Authors	Kessler J, Thiel A, Karbe H, Heiss WD
Title	Piracetam improves activated blood flow and facilitates rehabilitation of poststroke aphasic patients
Reference	*Stroke* 2000; 31: 2112-2116
PMID	10978039
DOI	10.1161/01.str.31.9.2112

Participants

Language	German
Inclusion criteria	Mild to moderate aphasia on TT; at least 50 out of 150 on AAT repetition
Number of individuals with aphasia	24
Number of control participants	0
Were any of the participants included in any previous studies?	No
Is age reported for patients and controls, and matched?	Yes (piracetam group: mean 57.4 ± 13.5 years; placebo group: mean 56.3 ± 10.0 years)
Is sex reported for patients and controls, and matched?	Yes (males: 13; females: 11)
Is handedness reported for patients and controls, and matched?	Yes (right: 24; left: 0)
Is time post stroke onset reported and appropriate to the study design?	Yes (T1: ~2 weeks; T2: ~8 weeks)
To what extent is the nature of aphasia characterized?	Severity only
Language evaluation	AAT
Aphasia severity	T1: piracetam group: TT 17.16 ± 14.31 errors; placebo group: TT 17.91 ± 15.47 errors; T2: piracetam group: TT 9.66 ± 12.62 errors; placebo group: TT 12.50 ± 16.88 errors
Aphasia type	Not stated
First stroke only?	Yes
Stroke type	Ischemic only
To what extent is the lesion distribution characterized?	Location only
Lesion extent	Not stated
Lesion location	10 L frontal, 6 L subcortical, 8 L temporal
Participants notes	—

Imaging

Modality	PET (rCBF)
Is the study cross-sectional or longitudinal?	Longitudinal—mixed
If longitudinal, at what time point(s) were imaging data acquired?	T1: pre-treatment, ~2 weeks post onset; T2: post-treatment, ~8 weeks post onset
If longitudinal, was there any intervention between the time points?	SLT, 1 hour/day, 5 days/week, 6 weeks; 12 patients received piracetam and 12 received placebo; note that the two groups are not directly compared in any imaging or behavioral analyses
Is the scanner described?	Yes (CTI-Siemens ECAT EXACT HR)
Is the timing of stimulus presentation and image acquisition clearly described and appropriate?	Yes
Design type	PET
Total images acquired	8
Are the imaging acquisition parameters, including coverage, adequately described and appropriate?	Yes (whole brain)
Is preprocessing and intrasubject coregistration adequately described and appropriate?	Yes

Is first level model fitting adequately described and appropriate?	Yes
Is intersubject normalization adequately described and appropriate?	N/A—no intersubject normalization
Imaging notes	—

Conditions

Are the conditions clearly described?	Yes

Condition	Response type	Repetitions	All groups could do?	All individuals could do?
word repetition	Word (overt)	4	Yes	Yes
rest	None	4	N/A	N/A

Conditions notes	Inclusion criterion was applied to ensure that the task could be performed

Contrasts

Are the contrasts clearly described?	Yes

Contrast 1: word repetition vs rest

Language condition	Word repetition
Control condition	Rest
Are the conditions matched for visual demands?	Yes
Are the conditions matched for auditory demands?	No
Are the conditions matched for motor demands?	No
Are the conditions matched for cognitive/executive demands?	No
Is accuracy matched between the language and control tasks for all relevant groups?	N/A, tasks not comparable
Is reaction time matched between the language and control tasks for all relevant groups?	N/A, tasks not comparable
Behavioral data notes	—
Are control data reported in this paper or another that is referenced?	No
Does the contrast selectively activate plausible relevant language regions in the control group?	Unknown
Are activations lateralized in the control data?	Unknown
Control activation notes	No control data are reported or cited, however the same task was used in several previous studies by this group
Contrast notes	—

Analyses

Are the analyses clearly described?	Yes

ROI analysis 1

First level contrast	Word repetition vs rest
Analysis class	Longitudinal change in aphasia
Group(s)	Aphasia treated with pirecetam (n = 12) T2 vs T1
Covariate	—
Is the second level contrast valid in terms of the group(s), time point(s), and measures involved?	Yes
Is accuracy matched across the second level contrast?	Unknown, not reported
Is reaction time matched across the second level contrast?	Unknown, not reported
Behavioral data notes	—
Type of analysis	Regions of interest (ROI)

ROI type	Anatomical
How many ROIs are there?	14
What are the ROI(s)?	(1) L BA 44; (2) L BA 45; (3) L ventral PrCG; (4) L HG; (5) L BA 41 and 42; (6) L BA 22; (7) L SMA; (8-14) homotopic counterparts
How are the ROI(s) defined?	Individual anatomical images
Correction for multiple comparisons	No correction
Statistical details	—
Findings	↑ L IFG pars triangularis<br>↑ L posterior STG<br>↑ L Heschl's gyrus
Findings notes	—

ROI analysis 2

First level contrast	Word repetition vs rest
Analysis class	Longitudinal change in aphasia
Group(s)	Aphasia treated with placebo (n = 12) T2 vs T1
Covariate	—
Is the second level contrast valid in terms of the group(s), time point(s), and measures involved?	Yes
Is accuracy matched across the second level contrast?	Unknown, not reported
Is reaction time matched across the second level contrast?	Unknown, not reported
Behavioral data notes	—
Type of analysis	Regions of interest (ROI)
ROI type	Anatomical
How many ROIs are there?	14
What are the ROI(s)?	(1) L BA 44; (2) L BA 45; (3) L ventral PrCG; (4) L HG; (5) L BA 41 and 42; (6) L BA 22; (7) L SMA; (8-14) homotopic counterparts
How are the ROI(s) defined?	Individual anatomical images
Correction for multiple comparisons	No correction
Statistical details	—
Findings	↑ L ventral precentral/inferior frontal junction
Findings notes	—

Notes

Excluded analyses	—

Rosen et al. (2000)

Reference

Authors	Rosen HJ, Petersen SE, Linenweber MR, Snyder AZ, White DA, Chapman L, Dromerick AW, Fiez JA, Corbetta M
Title	Neural correlates of recovery from aphasia after damage to left inferior frontal cortex
Reference	*Neurology* 2000; 55: 1883-1894
PMID	11134389
DOI	10.1212/wnl.55.12.1883

Participants

Language	US English
Inclusion criteria	L IFG, possibly extending to neighboring regions
Number of individuals with aphasia	6
Number of control participants	14

Were any of the participants included in any previous studies?	Yes (1 participant was reported in a previous case study)
Is age reported for patients and controls, and matched?	No (mean 47 years, range 32-72 years; control participants not age-matched)
Is sex reported for patients and controls, and matched?	Yes (males: 3; females: 3)
Is handedness reported for patients and controls, and matched?	Yes (right: 6; left: 0)
Is time post stroke onset reported and appropriate to the study design?	Yes (range 0.5-7.6 years)
To what extent is the nature of aphasia characterized?	Severity and type
Language evaluation	WAB (except BDAE in 1 patient), reading pseudowords, word stem completion, verb generation, reading single words
Aphasia severity	AQ range 74-97 (missing in 1 patient)
Aphasia type	3 anomic, 1 Broca's, 1 not stated, 1 recovered
First stroke only?	Yes
Stroke type	Not stated
To what extent is the lesion distribution characterized?	Individual lesions
Lesion extent	Range 10.7-117.5 cc
Lesion location	L IFG, extending to neighboring areas in most cases
Participants notes	Of the 14 controls, 6 were studied with PET and 8 with fMRI

Imaging

Modality	PET and fMRI
Is the study cross-sectional or longitudinal?	Cross-sectional
If longitudinal, at what time point(s) were imaging data acquired?	—
If longitudinal, was there any intervention between the time points?	—
Is the scanner described?	Yes (Siemens 961 EXACT HR; Siemens Vision 1.5 Tesla)
Is the timing of stimulus presentation and image acquisition clearly described and appropriate?	No (fMRI timing description is inconsistent)
Design type	Mixed
Total images acquired	PET: 10; fMRI: 384-768
Are the imaging acquisition parameters, including coverage, adequately described and appropriate?	Yes (whole brain)
Is preprocessing and intrasubject coregistration adequately described and appropriate?	Yes
Is first level model fitting adequately described and appropriate?	Yes
Is intersubject normalization adequately described and appropriate?	Yes
Imaging notes	1 patient scanned on different PET scanner, and not scanned with fMRI; controls had different fMRI sequence to patients

Conditions

Are the conditions clearly described?	Yes

Condition	Response type	Repetitions	All groups could do?	All individuals could do?
word stem completion (PET)	Word (overt)	4	Yes	Yes
reading pseudowords aloud (PET)	Word (overt)	4	Yes	No
rest (PET)	None	2	N/A	N/A
word stem completion (fMRI)	Word (covert)	15-30 (?)	Yes	Yes
rest (fMRI)	None	15-30 (?)	N/A	N/A

Conditions notes	Pseudoword reading condition not analyzed in this paper

Contrasts

Are the contrasts clearly described?	Yes

Contrast 1: word stem completion (PET) vs rest (PET)

Language condition	Word stem completion (PET)
Control condition	Rest (PET)
Are the conditions matched for visual demands?	No
Are the conditions matched for auditory demands?	No
Are the conditions matched for motor demands?	No
Are the conditions matched for cognitive/executive demands?	No
Is accuracy matched between the language and control tasks for all relevant groups?	N/A, tasks not comparable
Is reaction time matched between the language and control tasks for all relevant groups?	N/A, tasks not comparable
Behavioral data notes	—
Are control data reported in this paper or another that is referenced?	Somewhat
Does the contrast selectively activate plausible relevant language regions in the control group?	Somewhat
Are activations lateralized in the control data?	Yes
Control activation notes	L IFG, L ITG, L anterior fusiform
Contrast notes	—

Contrast 2: word stem completion (fMRI) vs rest (fMRI)

Language condition	Word stem completion (fMRI)
Control condition	Rest (fMRI)
Are the conditions matched for visual demands?	No
Are the conditions matched for auditory demands?	Yes
Are the conditions matched for motor demands?	Yes
Are the conditions matched for cognitive/executive demands?	No
Is accuracy matched between the language and control tasks for all relevant groups?	N/A, tasks not comparable
Is reaction time matched between the language and control tasks for all relevant groups?	N/A, tasks not comparable
Behavioral data notes	—
Are control data reported in this paper or another that is referenced?	Somewhat
Does the contrast selectively activate plausible relevant language regions in the control group?	Somewhat
Are activations lateralized in the control data?	Yes
Control activation notes	L IFG, L intraparietal sulcus
Contrast notes	—

Analyses

Are the analyses clearly described?	No* (moderate limitation) (see specific limitation(s) below)

Voxelwise analysis 1

First level contrast	Word stem completion (PET) vs rest (PET)
Analysis class	Cross-sectional aphasia vs control
Group(s)	Aphasia vs control
Covariate	—

Is the second level contrast valid in terms of the group(s), time point(s), and measures involved?	Yes
Is accuracy matched across the second level contrast?	No, different
Is reaction time matched across the second level contrast?	Yes, matched
Behavioral data notes	—
Type of analysis	Voxelwise
Search volume	Whole brain
Correction for multiple comparisons	Unclear or not stated
Software	not stated
Voxelwise p	—
Cluster extent	—
Statistical details	Correction for multiple comparisons unclear; there may be circularity in only correcting for the number of regions that seemed to show differences
Findings	↑ L SMA/medial prefrontal<br>↑ R IFG<br>↑ R Heschl's gyrus<br>↓ L IFG
Findings notes	—

Voxelwise analysis 2

First level contrast	Word stem completion (fMRI) vs rest (fMRI)
Analysis class	Cross-sectional aphasia vs control
Group(s)	Aphasia (n = 5) vs control
Covariate	—
Is the second level contrast valid in terms of the group(s), time point(s), and measures involved?	Yes
Is accuracy matched across the second level contrast?	Unknown, not reported
Is reaction time matched across the second level contrast?	Unknown, not reported
Behavioral data notes	—
Type of analysis	Voxelwise
Search volume	Whole brain
Correction for multiple comparisons	No direct comparison
Software	not stated
Voxelwise p	—
Cluster extent	—
Statistical details	Qualitative comparison on p. 1888
Findings	↑ R IFG<br>↓ L IFG
Findings notes	—

ROI analysis 1

First level contrast	Word stem completion (fMRI) vs rest (fMRI)
Analysis class	Cross-sectional aphasia vs control
Group(s)	Aphasia (n = 5) vs control
Covariate	—
Is the second level contrast valid in terms of the group(s), time point(s), and measures involved?	Yes
Is accuracy matched across the second level contrast?	Unknown, not reported
Is reaction time matched across the second level contrast?	Unknown, not reported
Behavioral data notes	—

Type of analysis	Regions of interest (ROI)
ROI type	Functional
How many ROIs are there?	2
What are the ROI(s)?	(1) R IFG; (2) SMA
How are the ROI(s) defined?	Not stated but seem to be functional
Correction for multiple comparisons	No correction
Statistical details	Possibly circular because not clear how ROIs defined
Findings	↑ R IFG
Findings notes	—

Notes

Excluded analyses	(1) the authors also observe that the two patients with the best language outcomes retained perilesional activation in the L IFG; (2) two non-significant correlational analyses involving only 5 patients, but note that the main fMRI analyses have been included even though n = 5

Blasi et al. (2002)

Reference

Authors	Blasi V, Young AC, Tansy AP, Petersen SE, Snyder AZ, Corbetta M
Title	Word retrieval learning modulates right frontal cortex in patients with left frontal damage
Reference	*Neuron* 2002; 36: 159-170
PMID	12367514
DOI	10.1016/s0896-6273(02)00936-4

Participants

Language	US English
Inclusion criteria	L IFG, possibly extending to neighboring regions
Number of individuals with aphasia	8
Number of control participants	14
Were any of the participants included in any previous studies?	No
Is age reported for patients and controls, and matched?	No (mean 48.6 years; patients and controls not closely matched for age, unclear if difference significant)
Is sex reported for patients and controls, and matched?	Yes (males: 2; females: 6)
Is handedness reported for patients and controls, and matched?	Yes (right: 8; left: 0)
Is time post stroke onset reported and appropriate to the study design?	No (> 6 months; actual TPO not stated)
To what extent is the nature of aphasia characterized?	Comprehensive battery
Language evaluation	WAB or BDAE
Aphasia severity	AQ range 66.5-89.0 in 6 participants, BDAE aphasia severity of 4 in 1 participant, no formal evaluation in 1 participant
Aphasia type	3 anomic, 3 transcortical motor, 1 Broca's, 1 not stated; most were Broca's or global acutely
First stroke only?	Yes
Stroke type	Ischemic only
To what extent is the lesion distribution characterized?	Individual lesions
Lesion extent	Not stated
Lesion location	L IFG and operculum, extending to adjacent cortex and white matter in several cases
Participants notes	—

Imaging

Modality	fMRI
Is the study cross-sectional or longitudinal?	Cross-sectional
If longitudinal, at what time point(s) were imaging data acquired?	—
If longitudinal, was there any intervention between the time points?	—
Is the scanner described?	Yes (Siemens Vision 1.5 Tesla)
Is the timing of stimulus presentation and image acquisition clearly described and appropriate?	Yes
Design type	Event-related
Total images acquired	1024
Are the imaging acquisition parameters, including coverage, adequately described and appropriate?	Yes (whole brain)
Is preprocessing and intrasubject coregistration adequately described and appropriate?	Yes
Is first level model fitting adequately described and appropriate?	Yes
Is intersubject normalization adequately described and appropriate?	No (not described)
Imaging notes	—

Conditions

Are the conditions clearly described?	Yes

Condition	Response type	Repetitions	All groups could do?	All individuals could do?
word stem completion (novel items)	Word (covert)	196	Yes	Unknown
word stem completion (repeated items)	Word (covert)	196	Yes	Unknown
rest	None	implicit baseline	N/A	N/A

Conditions notes	Novel items were presented in runs 1, 6, 7, and 8; repeated items were presented in runs 2, 3, 4, and 5; of the four repeated runs, only run 5 was analyzed.

Contrasts

Are the contrasts clearly described?	Yes

Contrast 1: word stem completion (novel items) vs rest

Language condition	Word stem completion (novel items)
Control condition	Rest
Are the conditions matched for visual demands?	No
Are the conditions matched for auditory demands?	Yes
Are the conditions matched for motor demands?	Yes
Are the conditions matched for cognitive/executive demands?	No
Is accuracy matched between the language and control tasks for all relevant groups?	N/A, tasks not comparable
Is reaction time matched between the language and control tasks for all relevant groups?	N/A, tasks not comparable
Behavioral data notes	—
Are control data reported in this paper or another that is referenced?	Yes
Does the contrast selectively activate plausible relevant language regions in the control group?	Somewhat
Are activations lateralized in the control data?	Somewhat
Control activation notes	Activation of language areas but also other areas; frontal activation is somewhat lateralized

Contrast notes	—

Contrast 2: word stem completion (novel items) vs word stem completion (repeated items)

Language condition	Word stem completion (novel items)
Control condition	Word stem completion (repeated items)
Are the conditions matched for visual demands?	Yes
Are the conditions matched for auditory demands?	Yes
Are the conditions matched for motor demands?	Yes
Are the conditions matched for cognitive/executive demands?	Yes
Is accuracy matched between the language and control tasks for all relevant groups?	Yes, matched
Is reaction time matched between the language and control tasks for all relevant groups?	No, different
Behavioral data notes	—
Are control data reported in this paper or another that is referenced?	Somewhat
Does the contrast selectively activate plausible relevant language regions in the control group?	Unknown
Are activations lateralized in the control data?	Somewhat
Control activation notes	No whole brain analysis of this contrast, but somewhat lateralized in the sense that L but not R frontal areas showed a learning effect
Contrast notes	—

Analyses

Are the analyses clearly described?	No** (major limitation) (see specific limitation(s) below)

Voxelwise analysis 1

First level contrast	Word stem completion (novel items) vs rest
Analysis class	Cross-sectional aphasia vs control
Group(s)	Aphasia vs control
Covariate	—
Is the second level contrast valid in terms of the group(s), time point(s), and measures involved?	Yes
Is accuracy matched across the second level contrast?	No, different
Is reaction time matched across the second level contrast?	No, different
Behavioral data notes	Covert task but overt data acquired separately; patients less accurate and slower than controls
Type of analysis	Voxelwise
Search volume	Whole brain
Correction for multiple comparisons	Unclear or not stated
Software	not stated
Voxelwise p	~.001 (z > 3)
Cluster extent	45 voxels (size not stated)
Statistical details	Monte Carlo analysis not described in detail; rather than fitting a HRF, the authors looked at the shape of the signal in the 8 volumes following each stimulus
Findings	↑ R IFG pars opercularis<br>↑ R IFG pars triangularis<br>↑ R insula<br>↑ R ventral precentral/inferior frontal junction<br>↑ R dorsal precentral<br>↓ L IFG pars opercularis<br>↓ L ventral precentral/inferior frontal junction
Findings notes	Labels based on coordinates reported

ROI analysis 1

First level contrast	Word stem completion (novel items) vs word stem completion (repeated items)
Analysis class	Cross-sectional aphasia vs control
Group(s)	Aphasia vs control
Covariate	—
Is the second level contrast valid in terms of the group(s), time point(s), and measures involved?	Yes
Is accuracy matched across the second level contrast?	Yes, matched
Is reaction time matched across the second level contrast?	Yes, matched
Behavioral data notes	Covert task but overt data acquired separately; no interaction of group by practice for accuracy or RT
Type of analysis	Regions of interest (ROI)
ROI type	Functional
How many ROIs are there?	14
What are the ROI(s)?	(1) L dorsal IFG; (2) L ventral IFG; (3) R MFG; (4) L anterior fusiform; (5) R anterior fusiform; (6) R posterior fusiform; (7) R lateral occipital; (8) R lateral cerebellum; (9) L SMA; (10) R dorsal IFG; (11) R posterior fusiform; (12) R lateral occipital; (13) R lingual; (14) L MTG
How are the ROI(s) defined?	Regions that were active for the main effect of word stem completion (irrespective of practice) in either group and modulated by practice in that group
Correction for multiple comparisons	No correction
Statistical details	Circular because ROIs defined in one group or the other; the L ROIs showed repetition suppression in controls but not in patients, and this difference is interpreted by the authors, but not supported statistically
Findings	↑ R ventral precentral/inferior frontal junction<br>↑ R posterior inferior temporal gyrus/fusiform gyrus<br>↓ L IFG<br>↓ L ventral precentral/inferior frontal junction<br>↓ L posterior inferior temporal gyrus/fusiform gyrus
Findings notes	Labels based on coordinates reported

Notes

Excluded analyses	(1) the ROI results were replicated in a whole brain SPM analysis, but that analysis is not reported; (2) the authors observe that patients with smaller L frontal lesions, and perilesional activation, performed better on word stem completion overall, but did not differ in rate of learning

Leff et al. (2002)

Reference

Authors	Leff A, Crinion J, Scott S, Turkheimer F, Howard D, Wise R
Title	A physiological change in the homotopic cortex following left posterior temporal lobe infarction
Reference	*Ann Neurol* 2002; 51: 553-558
PMID	12112100
DOI	10.1002/ana.10181

Participants

Language	UK English
Inclusion criteria	—
Number of individuals with aphasia	15
Number of control participants	8
Were any of the participants included in any	No

previous studies?	
Is age reported for patients and controls, and matched?	Yes (range 43-76 years)
Is sex reported for patients and controls, and matched?	Yes (males: 11; females: 4)
Is handedness reported for patients and controls, and matched?	Yes (right: 11; left: 0)
Is time post stroke onset reported and appropriate to the study design?	Yes (range 5-76 months)
To what extent is the nature of aphasia characterized?	Not at all
Language evaluation	PPT (Dutch), British picture vocabulary scale, Action for Dysphasic Adults lexical decision battery, auditory maximal pairs (an offline phoneme discrimination test)
Aphasia severity	Not stated
Aphasia type	Not stated, but all 6 patients with pSTS damage had single word comprehension deficits acutely
First stroke only?	Yes
Stroke type	Not stated
To what extent is the lesion distribution characterized?	Extent and location
Lesion extent	Range 0.5-14% of total brain volume
Lesion location	9 L but sparing pSTS, 6 L including pSTS
Participants notes	—

Imaging

Modality	PET (rCBF)
Is the study cross-sectional or longitudinal?	Cross-sectional
If longitudinal, at what time point(s) were imaging data acquired?	—
If longitudinal, was there any intervention between the time points?	—
Is the scanner described?	Yes (CTI-Siemens ECAT EXACT HR++/966)
Is the timing of stimulus presentation and image acquisition clearly described and appropriate?	Yes
Design type	PET
Total images acquired	16
Are the imaging acquisition parameters, including coverage, adequately described and appropriate?	Yes (whole brain)
Is preprocessing and intrasubject coregistration adequately described and appropriate?	Yes
Is first level model fitting adequately described and appropriate?	Yes
Is intersubject normalization adequately described and appropriate?	Yes
Imaging notes	—

Conditions

Are the conditions clearly described?	Yes

Condition	Response type	Repetitions	All groups could do?	All individuals could do?
listening to words at 10 wpm	None	2	N/A	N/A
listening to words at 35 wpm	None	2	N/A	N/A
listening to words at 55 wpm	None	2	N/A	N/A
listening to words at 70 wpm	None	2	N/A	N/A
listening to words at 85 wpm	None	2	N/A	N/A
listening to words at 95 wpm	None	2	N/A	N/A

listening to words at 115 wpm	None	2	N/A	N/A
listening to words at 130 wpm	None	2	N/A	N/A

Conditions notes	—

Contrasts

Are the contrasts clearly described?	Yes

Contrast 1: higher word rates vs lower word rates

Language condition	Higher word rates
Control condition	Lower word rates
Are the conditions matched for visual demands?	Yes
Are the conditions matched for auditory demands?	No
Are the conditions matched for motor demands?	Yes
Are the conditions matched for cognitive/executive demands?	Yes
Is accuracy matched between the language and control tasks for all relevant groups?	N/A, no behavioral measure
Is reaction time matched between the language and control tasks for all relevant groups?	N/A, no timeable task
Behavioral data notes	—
Are control data reported in this paper or another that is referenced?	Somewhat
Does the contrast selectively activate plausible relevant language regions in the control group?	Somewhat
Are activations lateralized in the control data?	Somewhat
Control activation notes	Control activation is bilateral in primary auditory cortex and the lateral STG (Fig. 1, labels 1 and 2), but there is a left-lateralized activation in the pSTS (label 3); the scatter plots in Fig. 1 show activity-word rate curves for peak pSTS voxels in individual subjects; slopes were steeper in the left hemisphere ($p < 0.05$), however, the identification of these voxels is not described in sufficient detail (i.e. what was the search region?)
Contrast notes	—

Analyses

Are the analyses clearly described?	No* (moderate limitation) (see specific limitation(s) below)

Voxelwise analysis 1

First level contrast	Higher word rates vs lower word rates
Analysis class	Cross-sectional aphasia vs control
Group(s)	Aphasia with pSTS damage (n = 6) vs control
Covariate	—
Is the second level contrast valid in terms of the group(s), time point(s), and measures involved?	Yes
Is accuracy matched across the second level contrast?	N/A, no behavioral measure
Is reaction time matched across the second level contrast?	N/A, no timeable task
Behavioral data notes	—
Type of analysis	Voxelwise
Search volume	Whole brain
Correction for multiple comparisons	No direct comparison
Software	SPM99
Voxelwise p	—
Cluster extent	—
Statistical details	Qualitative comparison on p. 555; a FWE-corrected SPM is reported of the relationship in the 6 patients with L pSTS damage (Fig. 2), however it is masked in a way that is not explained (see

	figure caption), and there is no direct comparison between patients with L pSTS damage and controls
Findings	↑ R posterior STS
Findings notes	—

Voxelwise analysis 2

First level contrast	Higher word rates vs lower word rates
Analysis class	Cross-sectional between two groups with aphasia
Group(s)	Aphasia with pSTS (n = 6) damage vs without pSTS damage (n = 9)
Covariate	—
Is the second level contrast valid in terms of the group(s), time point(s), and measures involved?	Yes
Is accuracy matched across the second level contrast?	N/A, no behavioral measure
Is reaction time matched across the second level contrast?	N/A, no timeable task
Behavioral data notes	—
Type of analysis	Voxelwise
Search volume	Whole brain
Correction for multiple comparisons	No direct comparison
Software	SPM99
Voxelwise p	—
Cluster extent	—
Statistical details	Qualitative comparison on p. 555; a FWE-corrected SPM is reported of the relationship in the 6 patients with L pSTS damage (Fig. 2), however it is masked in a way that is not explained (see figure caption), and there is no direct comparison between patients with L pSTS damage and patients with R pSTS damage
Findings	↑ R posterior STS
Findings notes	—

ROI analysis 1

First level contrast	Higher word rates vs lower word rates
Analysis class	Cross-sectional aphasia vs control
Group(s)	Aphasia with pSTS damage (n = 6) vs control (n = 8)
Covariate	—
Is the second level contrast valid in terms of the group(s), time point(s), and measures involved?	Yes
Is accuracy matched across the second level contrast?	N/A, no behavioral measure
Is reaction time matched across the second level contrast?	N/A, no timeable task
Behavioral data notes	—
Type of analysis	Region of interest (ROI)
ROI type	Functional
How many ROIs are there?	1
What are the ROI(s)?	R pSTS
How are the ROI(s) defined?	The peak voxel for the contrast in the R pSTS from each subject's individual analysis, but the search region is not stated
Correction for multiple comparisons	One only
Statistical details	The controls and patients without pSTS damage were combined, however it is stated in the caption to Figure 2 that the patients with pSTS damage were significantly different to both
Findings	↑ R posterior STS
Findings notes	—

ROI analysis 2

First level contrast	Higher word rates vs lower word rates

Analysis class	Cross-sectional between two groups with aphasia
Group(s)	Aphasia with pSTS damage (n = 6) vs aphasia without pSTS damage (n = 9)
Covariate	—
Is the second level contrast valid in terms of the group(s), time point(s), and measures involved?	Yes
Is accuracy matched across the second level contrast?	N/A, no behavioral measure
Is reaction time matched across the second level contrast?	N/A, no timeable task
Behavioral data notes	—
Type of analysis	Region of interest (ROI)
ROI type	Functional
How many ROIs are there?	1
What are the ROI(s)?	R pSTS
How are the ROI(s) defined?	The peak voxel for the contrast in the R pSTS from each subject's individual analysis, but the search region is not stated
Correction for multiple comparisons	One only
Statistical details	The controls and patients without pSTS damage were combined, however it is stated in the caption to Figure 2 that the patients with pSTS damage were significantly different to both
Findings	↑ R posterior STS
Findings notes	—

Notes

Excluded analyses	—

Blank et al. (2003)

Reference

Authors	Blank SC, Bird H, Turkheimer F, Wise RJ
Title	Speech production after stroke: the role of the right pars opercularis
Reference	*Ann Neurol* 2003; 54: 310-320
PMID	12953263
DOI	10.1002/ana.10656

Participants

Language	UK English
Inclusion criteria	Initial non-fluent aphasia due to anterior perisylvian lesion; subsequently recovered the ability to speak in sentences; patients were divided into those with and without damage to the IFG pars opercularis (POp+: n = 7; POp-: n = 7)
Number of individuals with aphasia	14
Number of control participants	12
Were any of the participants included in any previous studies?	No
Is age reported for patients and controls, and matched?	Yes (POp+: median 50 years, range 36-72 years; POp-: median 61 years, range 39-70 years)
Is sex reported for patients and controls, and matched?	Yes (males: 8; females: 6)
Is handedness reported for patients and controls, and matched?	Yes (right: 14; left: 0)
Is time post stroke onset reported and appropriate to the study design?	Yes (POp+: median 39 months, range 19-134 months; POp-: median 17 months, range 6-240 months)
To what extent is the nature of aphasia characterized?	Type only

Language evaluation	CAT, QPA
Aphasia severity	Not stated
Aphasia type	POp+: 4 non-fluent but not agrammatic, 2 agrammatic, 1 recovered; POp-: 4 non-fluent but not agrammatic, 3 recovered
First stroke only?	No
Stroke type	Not stated
To what extent is the lesion distribution characterized?	Individual lesions
Lesion extent	Not stated
Lesion location	L frontal, occasionally extending into temporal
Participants notes	8 of 12 controls included in Blank et al. (2002)

Imaging

Modality	PET (rCBF)
Is the study cross-sectional or longitudinal?	Cross-sectional
If longitudinal, at what time point(s) were imaging data acquired?	—
If longitudinal, was there any intervention between the time points?	—
Is the scanner described?	Yes (CTI-Siemens ECAT EXACT HR++ (966))
Is the timing of stimulus presentation and image acquisition clearly described and appropriate?	Yes
Design type	PET
Total images acquired	15 (patients); 12 (controls)
Are the imaging acquisition parameters, including coverage, adequately described and appropriate?	Yes (whole brain)
Is preprocessing and intrasubject coregistration adequately described and appropriate?	Yes
Is first level model fitting adequately described and appropriate?	Yes
Is intersubject normalization adequately described and appropriate?	Yes
Imaging notes	—

Conditions

Are the conditions clearly described?	Yes

Condition	Response type	Repetitions	All groups could do?	All individuals could do?
propositional speech production	Sentence (overt)	aphasia: 5; control: 4	Yes	Yes
counting	Multiple words (overt)	aphasia: 5; control: 4	Yes	Yes
rest	None	aphasia: 5; control: 4	N/A	N/A

Conditions notes	Alertness maintained in rest by asking participants to listen to environmental sounds that were presented before and after data acquisition; speech was recorded and rate was measured, also QPA was done of a separate speech sample outside the scanner

Contrasts

Are the contrasts clearly described?	Yes

Contrast 1: propositional speech production vs rest

Language condition	Propositional speech production
Control condition	Rest
Are the conditions matched for visual demands?	Yes

Are the conditions matched for auditory demands?	No
Are the conditions matched for motor demands?	No
Are the conditions matched for cognitive/executive demands?	No
Is accuracy matched between the language and control tasks for all relevant groups?	N/A, tasks not comparable
Is reaction time matched between the language and control tasks for all relevant groups?	N/A, tasks not comparable
Behavioral data notes	—
Are control data reported in this paper or another that is referenced?	Yes
Does the contrast selectively activate plausible relevant language regions in the control group?	Somewhat
Are activations lateralized in the control data?	Somewhat
Control activation notes	Much bilateral activation due to overt speech but pars opercularis and supratemporal plane L-lateralized
Contrast notes	—

Contrast 2: propositional speech production vs counting

Language condition	Propositional speech production
Control condition	Counting
Are the conditions matched for visual demands?	Yes
Are the conditions matched for auditory demands?	Yes
Are the conditions matched for motor demands?	Yes
Are the conditions matched for cognitive/executive demands?	No
Is accuracy matched between the language and control tasks for all relevant groups?	N/A, tasks not comparable
Is reaction time matched between the language and control tasks for all relevant groups?	N/A, tasks not comparable
Behavioral data notes	—
Are control data reported in this paper or another that is referenced?	Yes
Does the contrast selectively activate plausible relevant language regions in the control group?	Somewhat
Are activations lateralized in the control data?	Somewhat
Control activation notes	Extrasylvian; somewhat L-lateralized
Contrast notes	—

Analyses

Are the analyses clearly described?	Yes

Voxelwise analysis 1

First level contrast	Propositional speech production vs rest
Analysis class	Cross-sectional aphasia vs control
Group(s)	Aphasia with IFG POp damage (n = 7) vs control
Covariate	—
Is the second level contrast valid in terms of the group(s), time point(s), and measures involved?	Yes
Is accuracy matched across the second level contrast?	No, different
Is reaction time matched across the second level contrast?	N/A, no timeable task
Behavioral data notes	Word rates not reported, but offline speech sample differed
Type of analysis	Voxelwise
Search volume	Voxels spared in all patients

Correction for multiple comparisons	Small volume correction
Software	SPM99
Voxelwise p	FWE p < .05 with SVC in R pars opercularis
Cluster extent	—
Statistical details	—
Findings	↑ R IFG pars opercularis
Findings notes	No voxels survived FWE correction without SVC

Voxelwise analysis 2

First level contrast	Propositional speech production vs rest
Analysis class	Cross-sectional aphasia vs control
Group(s)	Aphasia without IFG POp damage (n = 7) vs control
Covariate	—
Is the second level contrast valid in terms of the group(s), time point(s), and measures involved?	Yes
Is accuracy matched across the second level contrast?	No, different
Is reaction time matched across the second level contrast?	N/A, no timeable task
Behavioral data notes	Word rates not reported, but offline speech sample differed
Type of analysis	Voxelwise
Search volume	Voxels spared in all patients
Correction for multiple comparisons	Small volume correction
Software	SPM99
Voxelwise p	FWE p < .05 with SVC in R pars opercularis
Cluster extent	—
Statistical details	—
Findings	↑ R IFG pars opercularis
Findings notes	—

Voxelwise analysis 3

First level contrast	Propositional speech production vs rest
Analysis class	Cross-sectional between two groups with aphasia
Group(s)	Aphasia with IFG POp damage (n = 7) vs without IFG POp damage (n = 7)
Covariate	—
Is the second level contrast valid in terms of the group(s), time point(s), and measures involved?	Yes
Is accuracy matched across the second level contrast?	No, different
Is reaction time matched across the second level contrast?	N/A, no timeable task
Behavioral data notes	Word rates not reported, but offline speech sample differed
Type of analysis	Voxelwise
Search volume	Voxels spared in all patients
Correction for multiple comparisons	Small volume correction
Software	SPM99
Voxelwise p	FWE p < .05 with SVC in R pars opercularis
Cluster extent	—
Statistical details	—
Findings	None
Findings notes	Patients with L IFG POp damage showed numerically more signal in the R IFG POp

Voxelwise analysis 4

First level contrast	Propositional speech production vs counting
Analysis class	Cross-sectional aphasia vs control

Group(s)	Aphasia with IFG POp damage (n = 7) vs control
Covariate	—
Is the second level contrast valid in terms of the group(s), time point(s), and measures involved?	Yes
Is accuracy matched across the second level contrast?	No, different
Is reaction time matched across the second level contrast?	N/A, no timeable task
Behavioral data notes	Word rates not reported, but offline speech sample differed
Type of analysis	Voxelwise
Search volume	Voxels spared in all patients
Correction for multiple comparisons	Small volume correction
Software	SPM99
Voxelwise p	FWE p < .05 with SVC in R pars opercularis
Cluster extent	—
Statistical details	—
Findings	None
Findings notes	—

Voxelwise analysis 5

First level contrast	Propositional speech production vs counting
Analysis class	Cross-sectional aphasia vs control
Group(s)	Aphasia without IFG POp damage (n = 7) vs control
Covariate	—
Is the second level contrast valid in terms of the group(s), time point(s), and measures involved?	Yes
Is accuracy matched across the second level contrast?	No, different
Is reaction time matched across the second level contrast?	N/A, no timeable task
Behavioral data notes	Word rates not reported, but offline speech sample differed
Type of analysis	Voxelwise
Search volume	Voxels spared in all patients
Correction for multiple comparisons	Small volume correction
Software	SPM99
Voxelwise p	FWE p < .05 with SVC in R pars opercularis
Cluster extent	—
Statistical details	—
Findings	None
Findings notes	—

Voxelwise analysis 6

First level contrast	Propositional speech production vs counting
Analysis class	Cross-sectional between two groups with aphasia
Group(s)	Aphasia with IFG POp damage (n = 7) vs without IFG POp damage (n = 7)
Covariate	—
Is the second level contrast valid in terms of the group(s), time point(s), and measures involved?	Yes
Is accuracy matched across the second level contrast?	No, different
Is reaction time matched across the second level contrast?	N/A, no timeable task
Behavioral data notes	Word rates not reported, but offline speech sample differed
Type of analysis	Voxelwise
Search volume	Voxels spared in all patients

Correction for multiple comparisons	Small volume correction
Software	SPM99
Voxelwise p	FWE p < .05 with SVC in R pars opercularis
Cluster extent	—
Statistical details	—
Findings	None
Findings notes	—

ROI analysis 1

First level contrast	Propositional speech production vs rest
Analysis class	Cross-sectional correlation with language or other measure
Group(s)	Aphasia with IFG POp damage (n = 7)
Covariate	Speech rate during scan
Is the second level contrast valid in terms of the group(s), time point(s), and measures involved?	Yes
Is accuracy matched across the second level contrast?	Unknown, not reported
Is reaction time matched across the second level contrast?	N/A, no timeable task
Behavioral data notes	—
Type of analysis	Region of interest (ROI)
ROI type	Functional
How many ROIs are there?	1
What are the ROI(s)?	R IFG pars opercularis
How are the ROI(s) defined?	Defined by flipping L IFG pars opercularis activation in controls
Correction for multiple comparisons	One only
Statistical details	—
Findings	None
Findings notes	—

ROI analysis 2

First level contrast	Propositional speech production vs rest
Analysis class	Cross-sectional correlation with language or other measure
Group(s)	Aphasia without IFG POp damage (n = 7)
Covariate	Speech rate during scan
Is the second level contrast valid in terms of the group(s), time point(s), and measures involved?	Yes
Is accuracy matched across the second level contrast?	Unknown, not reported
Is reaction time matched across the second level contrast?	N/A, no timeable task
Behavioral data notes	—
Type of analysis	Region of interest (ROI)
ROI type	Functional
How many ROIs are there?	1
What are the ROI(s)?	R IFG pars opercularis
How are the ROI(s) defined?	Defined by flipping L IFG pars opercularis activation in controls
Correction for multiple comparisons	One only
Statistical details	—
Findings	None
Findings notes	—

ROI analysis 3

First level contrast	Propositional speech production vs rest
Analysis class	Cross-sectional correlation with language or other measure

Group(s)	Aphasia with IFG POp damage (n = 7)
Covariate	Four different QPA measures
Is the second level contrast valid in terms of the group(s), time point(s), and measures involved?	Yes
Is accuracy matched across the second level contrast?	Unknown, not reported
Is reaction time matched across the second level contrast?	N/A, no timeable task
Behavioral data notes	—
Type of analysis	Region of interest (ROI)
ROI type	Functional
How many ROIs are there?	1
What are the ROI(s)?	R IFG pars opercularis
How are the ROI(s) defined?	Defined by flipping L IFG pars opercularis activation in controls
Correction for multiple comparisons	One only
Statistical details	—
Findings	None
Findings notes	—

Notes

Excluded analyses	ROI analyses may have been carried out for both contrasts, but this is not stated

Cardebat et al. (2003)

Reference

Authors	Cardebat D, Démonet JF, De Boissezon X, Marie N, Marié RM, Lambert J, Baron JC, Puel M
Title	Behavioral and neurofunctional changes over time in healthy and aphasic subjects: a PET language activation study
Reference	*Stroke* 2003; 34: 2900-2906
PMID	14615626
DOI	10.1161/01.str.0000099965.99393.83

Participants

Language	French
Inclusion criteria	No severe aphasia; no leukoaraiosis
Number of individuals with aphasia	8
Number of control participants	6
Were any of the participants included in any previous studies?	No
Is age reported for patients and controls, and matched?	Yes (mean 58.4 ± 11.9 years, range 37-73 years)
Is sex reported for patients and controls, and matched?	Yes (males: 7; females: 1)
Is handedness reported for patients and controls, and matched?	Yes (right: 8; left: 0)
Is time post stroke onset reported and appropriate to the study design?	No* (moderate limitation) (T1: 58 ± 35 days, range 11-113 days; T2: 11.7 ± 1.6 months, range 320-460 days; T1 varies considerably from early to late subacute)
To what extent is the nature of aphasia characterized?	Not at all
Language evaluation	Not stated
Aphasia severity	Not stated
Aphasia type	T1: some prominent symptoms are listed for each patient; T2: not stated
First stroke only?	Yes

Stroke type	Mixed etiologies
To what extent is the lesion distribution characterized?	Individual lesions
Lesion extent	Not stated
Lesion location	4 L subcortical, 2 L prerolandic, 2 L postrolandic
Participants notes	—

Imaging

Modality	PET (rCBF)
Is the study cross-sectional or longitudinal?	Longitudinal—recovery
If longitudinal, at what time point(s) were imaging data acquired?	T1: 58 ± 35 days, range 11-113 days; T2: 11.7 ± 1.6 months, range 320-460 days; T1 varies considerably from early to late subacute
If longitudinal, was there any intervention between the time points?	Not stated
Is the scanner described?	Yes (Siemens ECAT HR+)
Is the timing of stimulus presentation and image acquisition clearly described and appropriate?	Yes
Design type	PET
Total images acquired	6
Are the imaging acquisition parameters, including coverage, adequately described and appropriate?	Yes (whole brain)
Is preprocessing and intrasubject coregistration adequately described and appropriate?	Yes
Is first level model fitting adequately described and appropriate?	Yes
Is intersubject normalization adequately described and appropriate?	No (lesion impact not addressed)
Imaging notes	—

Conditions

Are the conditions clearly described?	Yes

Condition	Response type	Repetitions	All groups could do?	All individuals could do?
word generation	Word (overt)	4	Yes	Unknown
rest	None	2	N/A	N/A

Conditions notes	Participants were asked to generate words that were semantically related to binaurally presented stimuli; 2 runs involved nouns and 2 involved verbs

Contrasts

Are the contrasts clearly described?	Yes

Contrast 1: word generation vs rest

Language condition	Word generation
Control condition	Rest
Are the conditions matched for visual demands?	Yes
Are the conditions matched for auditory demands?	No
Are the conditions matched for motor demands?	No
Are the conditions matched for cognitive/executive demands?	No
Is accuracy matched between the language and control tasks for all relevant groups?	N/A, tasks not comparable
Is reaction time matched between the language and control tasks for all relevant groups?	N/A, tasks not comparable
Behavioral data notes	—
Are control data reported in this paper or another	Somewhat

that is referenced?	
Does the contrast selectively activate plausible relevant language regions in the control group?	Somewhat
Are activations lateralized in the control data?	No
Control activation notes	Bilateral fronto-temporal and some other regions per text
Contrast notes	—

Analyses

Are the analyses clearly described?	No* (moderate limitation) (see specific limitation(s) below)

Voxelwise analysis 1

First level contrast	Word generation vs rest
Analysis class	Longitudinal change in aphasia
Group(s)	Aphasia T2 vs T1
Covariate	—
Is the second level contrast valid in terms of the group(s), time point(s), and measures involved?	Yes
Is accuracy matched across the second level contrast?	No, different
Is reaction time matched across the second level contrast?	Unknown, not reported
Behavioral data notes	—
Type of analysis	Voxelwise
Search volume	Whole brain
Correction for multiple comparisons	Clusterwise correction based on arbitrary cluster extent
Software	SPM99
Voxelwise p	.05
Cluster extent	50 voxels (size not stated)
Statistical details	Nature of inclusive masks unclear
Findings	↑ L dorsolateral prefrontal cortex<br>↑ L SMA/medial prefrontal<br>↑ L somato-motor<br>↑ L posterior STG/STS/MTG<br>↑ L cerebellum<br>↑ R IFG pars opercularis<br>↑ R dorsolateral prefrontal cortex<br>↑ R SMA/medial prefrontal<br>↑ R somato-motor<br>↑ R posterior STG/STS/MTG<br>↑ R cerebellum
Findings notes	Based on Figure 2

Voxelwise analysis 2

First level contrast	Word generation vs rest
Analysis class	Longitudinal correlation with language or other measure
Group(s)	Aphasia T2 vs T1
Covariate	Δ word generation accuracy
Is the second level contrast valid in terms of the group(s), time point(s), and measures involved?	Yes
Is accuracy matched across the second level contrast?	Accuracy is covariate
Is reaction time matched across the second level contrast?	Unknown, not reported
Behavioral data notes	—
Type of analysis	Voxelwise
Search volume	Whole brain

Correction for multiple comparisons	Clusterwise correction based on arbitrary cluster extent
Software	SPM99
Voxelwise p	.001
Cluster extent	100 voxels (size not stated)
Statistical details	Nature of inclusive masks unclear
Findings	↑ L posterior STG/STS/MTG<br>↑ R posterior STG/STS/MTG<br>↑ R cerebellum<br>↓ L occipital<br>↓ L hippocampus/MTL<br>↓ R dorsolateral prefrontal cortex<br>↓ R occipital
Findings notes	—

Notes

Excluded analyses	Aphasia vs control SPM analyses at each time point, because they are not reported in sufficient detail to determine activated regions

Sharp et al. (2004)

Reference

Authors	Sharp DJ, Scott SK, Wise RJ
Title	Retrieving meaning after temporal lobe infarction: the role of the basal language area
Reference	*Ann Neurol* 2004; 56: 836-846
PMID	15514975
DOI	10.1002/ana.20294

Participants

Language	UK English
Inclusion criteria	Lesion in vicinity of L STG; no extensive frontal damage; no inferior temporal damage; able to perform tasks
Number of individuals with aphasia	9
Number of control participants	18
Were any of the participants included in any previous studies?	No
Is age reported for patients and controls, and matched?	Yes (median 58 years, range 39-72 years)
Is sex reported for patients and controls, and matched?	Yes (males: 8; females: 1)
Is handedness reported for patients and controls, and matched?	Yes (right: 9; left: 0)
Is time post stroke onset reported and appropriate to the study design?	Yes (mean 45 months, range 14-145 months)
To what extent is the nature of aphasia characterized?	Severity only
Language evaluation	Subtests from CAT, subtests from PALPA, Action for dysphasic adults, TROG, PPT
Aphasia severity	Mild
Aphasia type	Not stated
First stroke only?	Yes
Stroke type	Not stated
To what extent is the lesion distribution characterized?	Lesion overlay
Lesion extent	Not stated
Lesion location	Lesion in vicinity of L STG; no extensive frontal damage; no inferior temporal damage

Participants notes	—

Imaging

Modality	PET (rCBF)
Is the study cross-sectional or longitudinal?	Cross-sectional
If longitudinal, at what time point(s) were imaging data acquired?	—
If longitudinal, was there any intervention between the time points?	—
Is the scanner described?	Yes (Siemens HR++ 966)
Is the timing of stimulus presentation and image acquisition clearly described and appropriate?	Yes
Design type	PET
Total images acquired	16
Are the imaging acquisition parameters, including coverage, adequately described and appropriate?	Yes (whole brain)
Is preprocessing and intrasubject coregistration adequately described and appropriate?	Yes
Is first level model fitting adequately described and appropriate?	Yes
Is intersubject normalization adequately described and appropriate?	Yes
Imaging notes	—

Conditions

Are the conditions clearly described?	Yes

Condition	Response type	Repetitions	All groups could do?	All individuals could do?
semantic decision	Word (overt)	aphasia: 8; control: 4	Yes	Yes
syllable count decision	Word (overt)	aphasia: 8; control: 4	Yes	Yes
semantic decision (noise vocoded) (control only)	Word (overt)	4 (control)	Yes	Yes
syllable count decision (noise vocoded) (control only)	Word (overt)	4 (control)	Yes	Yes

Conditions notes	Seems the response was a spoken word, but this is not stated explicitly; assuming all individuals could do the tasks because this was an inclusion criterion and behavioral data supports

Contrasts

Are the contrasts clearly described?	Yes

Contrast 1: semantic decision vs syllable count decision

Language condition	Semantic decision
Control condition	Syllable count decision
Are the conditions matched for visual demands?	Yes
Are the conditions matched for auditory demands?	Yes
Are the conditions matched for motor demands?	Yes
Are the conditions matched for cognitive/executive demands?	Yes
Is accuracy matched between the language and control tasks for all relevant groups?	No, different
Is reaction time matched between the language and control tasks for all relevant groups?	No, different
Behavioral data notes	—

Are control data reported in this paper or another that is referenced?	Somewhat
Does the contrast selectively activate plausible relevant language regions in the control group?	Somewhat
Are activations lateralized in the control data?	Yes
Control activation notes	The control data provided also include the noise vocoded conditions; only ventral temporal activations are shown, which are L-lateralized
Contrast notes	—

Analyses

Are the analyses clearly described?	No* (moderate limitation) (see specific limitation(s) below)

Voxelwise analysis 1

First level contrast	Semantic decision vs syllable count decision
Analysis class	Cross-sectional aphasia vs control
Group(s)	Aphasia vs control (clear speech)
Covariate	—
Is the second level contrast valid in terms of the group(s), time point(s), and measures involved?	Yes
Is accuracy matched across the second level contrast?	Appear mismatched
Is reaction time matched across the second level contrast?	Yes, matched
Behavioral data notes	Interaction of group by task not reported for accuracy
Type of analysis	Voxelwise
Search volume	Whole brain
Correction for multiple comparisons	Small volume correction
Software	SPM99
Voxelwise p	FWE $p < .05$ with SVC in fusiform gyri, temporal poles, L IFG, L orbitofrontal and L SFG
Cluster extent	—
Statistical details	—
Findings	↓ L posterior inferior temporal gyrus/fusiform gyrus
Findings notes	—

Voxelwise analysis 2

First level contrast	Semantic decision vs syllable count decision
Analysis class	Cross-sectional correlation with language or other measure
Group(s)	Aphasia
Covariate	Semantic decision accuracy
Is the second level contrast valid in terms of the group(s), time point(s), and measures involved?	Yes
Is accuracy matched across the second level contrast?	Accuracy is covariate
Is reaction time matched across the second level contrast?	Unknown, not reported
Behavioral data notes	—
Type of analysis	Voxelwise
Search volume	Whole brain
Correction for multiple comparisons	Small volume correction
Software	SPM99
Voxelwise p	FWE $p < .05$ with SVC in fusiform gyri, temporal poles, L IFG, L orbitofrontal and L SFG
Cluster extent	—
Statistical details	Fixed effects; this analysis is not clearly described
Findings	↑ R posterior inferior temporal gyrus/fusiform gyrus
Findings notes	Patients who were more accurate had more activity in R anterior fusiform gyrus

ROI analysis 1

First level contrast	Semantic decision vs syllable count decision
Analysis class	Cross-sectional aphasia vs control
Group(s)	Aphasia vs control (clear speech)
Covariate	—
Is the second level contrast valid in terms of the group(s), time point(s), and measures involved?	Yes
Is accuracy matched across the second level contrast?	Appear mismatched
Is reaction time matched across the second level contrast?	Yes, matched
Behavioral data notes	Interaction of group by task not reported for accuracy
Type of analysis	Region of interest (ROI)
ROI type	Anatomical
How many ROIs are there?	1
What are the ROI(s)?	L fusiform gyrus
How are the ROI(s) defined?	Probabilistic brain atlas
Correction for multiple comparisons	One only
Statistical details	—
Findings	↓ L posterior inferior temporal gyrus/fusiform gyrus
Findings notes	—

ROI analysis 2

First level contrast	Semantic decision vs syllable count decision
Analysis class	Cross-sectional aphasia vs control
Group(s)	Aphasia vs control (noise vocoded)
Covariate	—
Is the second level contrast valid in terms of the group(s), time point(s), and measures involved?	Yes
Is accuracy matched across the second level contrast?	No, but attempt made
Is reaction time matched across the second level contrast?	Yes, matched
Behavioral data notes	Patients were more accurate on semantic decisions than syllable decisions, whereas controls were less accurate on noise vocoded semantic decisions than clear syllable decisions (which were the baseline for this analysis)
Type of analysis	Region of interest (ROI)
ROI type	Anatomical
How many ROIs are there?	1
What are the ROI(s)?	L fusiform gyrus
How are the ROI(s) defined?	Probabilistic brain atlas
Correction for multiple comparisons	One only
Statistical details	—
Findings	None
Findings notes	This analysis suggests that the difference between groups in the L fusiform gyrus disappears when the controls perform a semantic task that is similarly challenging

Notes

Excluded analyses	(1) combined analysis of patients and controls (Figure 4); (2) correlation with syllable decision making not described in sufficient detail

Zahn et al. (2004)

Reference

Authors	Zahn R, Drews E, Specht K, Kemeny S, Reith W, Willmes K, Schwarz M, Huber W
Title	Recovery of semantic word processing in global aphasia: a functional MRI study
Reference	*Cogn Brain Res* 2004; 18: 322-336
PMID	14741318
DOI	10.1016/j.cogbrainres.2003.10.021

Participants

Language	German
Inclusion criteria	Global aphasia in the first three months; some improvement of comprehension within 6-12 months
Number of individuals with aphasia	7
Number of control participants	14
Were any of the participants included in any previous studies?	No
Is age reported for patients and controls, and matched?	Yes (range 29-67 years)
Is sex reported for patients and controls, and matched?	Yes (males: 6; females: 1)
Is handedness reported for patients and controls, and matched?	Yes (right: 7; left: 0)
Is time post stroke onset reported and appropriate to the study design?	Yes (range 6 months-4 years)
To what extent is the nature of aphasia characterized?	Comprehensive battery
Language evaluation	AABT, AAT
Aphasia severity	TT percentile range 28-63
Aphasia type	3 global, 2 Broca's, 2 unclassifiable; all had been global initially
First stroke only?	Yes
Stroke type	Not stated
To what extent is the lesion distribution characterized?	Lesion overlay
Lesion extent	Not stated
Lesion location	L MCA
Participants notes	—

Imaging

Modality	fMRI
Is the study cross-sectional or longitudinal?	Cross-sectional
If longitudinal, at what time point(s) were imaging data acquired?	—
If longitudinal, was there any intervention between the time points?	—
Is the scanner described?	Yes (Philips ACS NT Gyroscan 1.5 Tesla)
Is the timing of stimulus presentation and image acquisition clearly described and appropriate?	No* (moderate limitation) (insufficient blocks per experimental condition (3) because blocks were too long (44 s))
Design type	Block
Total images acquired	198
Are the imaging acquisition parameters, including coverage, adequately described and appropriate?	Yes (whole brain)
Is preprocessing and intrasubject coregistration adequately described and appropriate?	Yes
Is first level model fitting adequately described and appropriate?	Yes
Is intersubject normalization adequately described and appropriate?	N/A—no intersubject normalization

Imaging notes	—

Conditions

Are the conditions clearly described?	Yes

Condition	Response type	Repetitions	All groups could do?	All individuals could do?
phonetic decision (reversed words vs sounds)	Button press	3	Yes	No
lexical decision (words vs reversed words)	Button press	3	Yes	Yes
semantic decision	Button press	3	Yes	No
rest	None	9	N/A	N/A

Conditions notes	—

Contrasts

Are the contrasts clearly described?	No (see specific limitation(s) below)

Contrast 1: semantic decision vs phonetic decision and lexical decision (conjunction)

Language condition	Semantic decision
Control condition	Phonetic decision and lexical decision (conjunction)
Are the conditions matched for visual demands?	Yes
Are the conditions matched for auditory demands?	Yes
Are the conditions matched for motor demands?	Yes
Are the conditions matched for cognitive/executive demands?	Yes
Is accuracy matched between the language and control tasks for all relevant groups?	Appear similar
Is reaction time matched between the language and control tasks for all relevant groups?	Unknown, not reported
Behavioral data notes	Tasks were matched in controls, but no statistics reported for patients
Are control data reported in this paper or another that is referenced?	Yes
Does the contrast selectively activate plausible relevant language regions in the control group?	Yes
Are activations lateralized in the control data?	Yes
Control activation notes	L-lateralized frontal activation, as well as temporal and parietal to a lesser extent
Contrast notes	Conjunction of baseline conditions not described in sufficient detail

Analyses

Are the analyses clearly described?	No* (moderate limitation) (see specific limitation(s) below)

ROI analysis 1

First level contrast	Semantic decision vs phonetic decision and lexical decision (conjunction)
Analysis class	Cross-sectional aphasia vs control
Group(s)	Aphasia vs control
Covariate	—
Is the second level contrast valid in terms of the group(s), time point(s), and measures involved?	Yes
Is accuracy matched across the second level contrast?	Unknown, no test
Is reaction time matched across the second level contrast?	Unknown, not reported
Behavioral data notes	Relative performance on language and control tasks unclear
Type of analysis	Region of interest (ROI)
ROI type	Laterality indi(ces)
How many ROIs are there?	1

What are the ROI(s)?	Language network LI
How are the ROI(s) defined?	
Correction for multiple comparisons	One only
Statistical details	Conjunction analyses not clearly described; in two patients, a different conjunction was used (lexical decision vs phonetic decision & semantic decision vs phonetic decision)
Findings	None
Findings notes	LI > 0 in 12 out of 14 controls and 5 out of 7 patients; no significant difference

Notes

Excluded analyses	Individual patient analyses

Crinion & Price (2005)

Reference

Authors	Crinion J, Price CJ
Title	Right anterior superior temporal activation predicts auditory sentence comprehension following aphasic stroke
Reference	*Brain* 2005; 128: 2858-2871
PMID	16234297
DOI	10.1093/brain/awh659

Participants

Language	UK English
Inclusion criteria	—
Number of individuals with aphasia	17
Number of control participants	18
Were any of the participants included in any previous studies?	No
Is age reported for patients and controls, and matched?	Yes (mean 62 ± 2.7 SEM years, range 34-75 years)
Is sex reported for patients and controls, and matched?	Yes (males: 12; females: 5)
Is handedness reported for patients and controls, and matched?	Yes (right: 17; left: 0)
Is time post stroke onset reported and appropriate to the study design?	Yes (range 4-125 months; aphasia with temporal damage (n=8) mean 41 months; aphasia without temporal damage (n=9) mean 48 months)
To what extent is the nature of aphasia characterized?	Comprehensive battery
Language evaluation	CAT
Aphasia severity	Not stated
Aphasia type	Not stated
First stroke only?	Yes
Stroke type	Not stated
To what extent is the lesion distribution characterized?	Lesion overlay
Lesion extent	Not stated
Lesion location	L MCA
Participants notes	—

Imaging

Modality	fMRI
Is the study cross-sectional or longitudinal?	Cross-sectional
If longitudinal, at what time point(s) were imaging	—

data acquired?	
If longitudinal, was there any intervention between the time points?	—
Is the scanner described?	No (Siemens 1.5 Tesla; model not stated)
Is the timing of stimulus presentation and image acquisition clearly described and appropriate?	No (the calculated duration of the stimuli, the calculated duration of the acquisitions, and the stated duration of the acquisitions yield three different numbers)
Design type	Block
Total images acquired	460
Are the imaging acquisition parameters, including coverage, adequately described and appropriate?	Yes (whole brain)
Is preprocessing and intrasubject coregistration adequately described and appropriate?	Yes
Is first level model fitting adequately described and appropriate?	Yes
Is intersubject normalization adequately described and appropriate?	Yes
Imaging notes	—

Conditions

Are the conditions clearly described?	Yes

Condition	Response type	Repetitions	All groups could do?	All individuals could do?
listening to narrative speech	None	32	N/A	N/A
listening to reversed speech	None	8	N/A	N/A

Conditions notes	A post-scan surprise recognition test asked whether or not 38 phrases had occurred in any story; patients answered 12-33 of these questions correctly; controls answered 24-37 correctly; also note that all patients performed above chance on CAT auditory sentence comprehension (73%+ accuracy)

Contrasts

Are the contrasts clearly described?	Yes

Contrast 1: listening to narrative speech vs listening to reversed speech

Language condition	Listening to narrative speech
Control condition	Listening to reversed speech
Are the conditions matched for visual demands?	Yes
Are the conditions matched for auditory demands?	Yes
Are the conditions matched for motor demands?	Yes
Are the conditions matched for cognitive/executive demands?	Yes
Is accuracy matched between the language and control tasks for all relevant groups?	N/A, no behavioral measure
Is reaction time matched between the language and control tasks for all relevant groups?	N/A, no timeable task
Behavioral data notes	—
Are control data reported in this paper or another that is referenced?	Yes
Does the contrast selectively activate plausible relevant language regions in the control group?	Yes
Are activations lateralized in the control data?	Somewhat
Control activation notes	Bilateral (L > R) temporal, L IFG and L dorsal precentral
Contrast notes	—

Analyses

Are the analyses clearly described?	Yes

Voxelwise analysis 1

First level contrast	Listening to narrative speech vs listening to reversed speech
Analysis class	Cross-sectional aphasia vs control
Group(s)	Aphasia without temporal lobe damage (n = 9) vs control
Covariate	—
Is the second level contrast valid in terms of the group(s), time point(s), and measures involved?	Yes
Is accuracy matched across the second level contrast?	N/A, no behavioral measure
Is reaction time matched across the second level contrast?	N/A, no timeable task
Behavioral data notes	—
Type of analysis	Voxelwise
Search volume	Whole brain
Correction for multiple comparisons	Voxelwise FWE correction and additional arbitrary cluster correction
Software	SPM2
Voxelwise p	FWE p < .05
Cluster extent	5 voxels (size not stated)
Statistical details	—
Findings	↓ L dorsal precentral<br>↓ R somato-motor
Findings notes	—

Voxelwise analysis 2

First level contrast	Listening to narrative speech vs listening to reversed speech
Analysis class	Cross-sectional aphasia vs control
Group(s)	Aphasia with temporal lobe damage (n = 8) vs control
Covariate	—
Is the second level contrast valid in terms of the group(s), time point(s), and measures involved?	Yes
Is accuracy matched across the second level contrast?	N/A, no behavioral measure
Is reaction time matched across the second level contrast?	N/A, no timeable task
Behavioral data notes	—
Type of analysis	Voxelwise
Search volume	Whole brain
Correction for multiple comparisons	Voxelwise FWE correction and additional arbitrary cluster correction
Software	SPM2
Voxelwise p	FWE p < .05
Cluster extent	5 voxels (size not stated)
Statistical details	—
Findings	↓ L posterior STS<br>↓ L mid temporal
Findings notes	—

Voxelwise analysis 3

First level contrast	Listening to narrative speech vs listening to reversed speech
Analysis class	Cross-sectional between two groups with aphasia
Group(s)	Aphasia with temporal lobe damage (n = 8) vs without temporal lobe damage (n = 9)
Covariate	—
Is the second level contrast valid in terms of the group(s), time point(s), and measures involved?	Yes
Is accuracy matched across the second level contrast?	N/A, no behavioral measure

Is reaction time matched across the second level contrast?	N/A, no timeable task
Behavioral data notes	—
Type of analysis	Voxelwise
Search volume	Whole brain
Correction for multiple comparisons	Voxelwise FWE correction and additional arbitrary cluster correction
Software	SPM2
Voxelwise p	FWE p < .05
Cluster extent	5 voxels (size not stated)
Statistical details	—
Findings	↓ L posterior STG/STS/MTG<br>↓ L mid temporal
Findings notes	—

Voxelwise analysis 4

First level contrast	Listening to narrative speech vs listening to reversed speech
Analysis class	Cross-sectional correlation with language or other measure
Group(s)	Aphasia without temporal lobe damage (n = 9)
Covariate	Sentence comprehension (CAT)
Is the second level contrast valid in terms of the group(s), time point(s), and measures involved?	Yes
Is accuracy matched across the second level contrast?	N/A, no behavioral measure
Is reaction time matched across the second level contrast?	N/A, no timeable task
Behavioral data notes	—
Type of analysis	Voxelwise
Search volume	Whole brain
Correction for multiple comparisons	Voxelwise FWE correction and additional arbitrary cluster correction
Software	SPM2
Voxelwise p	FWE p < .05
Cluster extent	5 voxels (size not stated)
Statistical details	Conjunction with main effect of story comprehension (details hard to follow); this was a multiple regression also involving patients with temporal lobe damage
Findings	↑ L posterior STS<br>↑ R mid temporal
Findings notes	Patients with better sentence comprehension had more activation in the L posterior STS and R mid STS

Voxelwise analysis 5

First level contrast	Listening to narrative speech vs listening to reversed speech
Analysis class	Cross-sectional correlation with language or other measure
Group(s)	Aphasia with temporal lobe damage (n = 8)
Covariate	Sentence comprehension (CAT)
Is the second level contrast valid in terms of the group(s), time point(s), and measures involved?	Yes
Is accuracy matched across the second level contrast?	N/A, no behavioral measure
Is reaction time matched across the second level contrast?	N/A, no timeable task
Behavioral data notes	—
Type of analysis	Voxelwise
Search volume	Whole brain
Correction for multiple comparisons	Voxelwise FWE correction and additional arbitrary cluster correction
Software	SPM2
Voxelwise p	FWE p < .05

Cluster extent	5 voxels (size not stated)
Statistical details	Conjunction with main effect of story comprehension (details hard to follow); this was a multiple regression also involving patients without temporal lobe damage
Findings	↑ R mid temporal
Findings notes	Patients with better sentence comprehension had more activation in the R mid STS

Complex analysis 1

First level contrast	Listening to narrative speech vs listening to reversed speech
Analysis class	Cross-sectional between two groups with aphasia
Group(s)	Aphasia with temporal damage (n = 8) vs without temporal damage (n = 9)
Covariate	—
Is the second level contrast valid in terms of the group(s), time point(s), and measures involved?	Yes
Is accuracy matched across the second level contrast?	N/A, no behavioral measure
Is reaction time matched across the second level contrast?	N/A, no timeable task
Behavioral data notes	—
Type of analysis	Complex
Statistical details	Correlations were computed between activity in each voxel, and the sentence comprehension measure from the CAT, and were compared between the two aphasia groups, in regions with a main effect of story comprehension. The voxelwise threshold was $p < .001$, uncorrected for multiple comparisons.
Findings	Other
Findings notes	Activity in the L posterior STS was positively correlated with sentence comprehension in patients without temporal lobe damage, but not in patients with temporal lobe damage

Complex analysis 2

First level contrast	Listening to narrative speech vs listening to reversed speech
Analysis class	Cross-sectional aphasia vs control
Group(s)	Aphasia without temporal damage (n = 9) vs control
Covariate	—
Is the second level contrast valid in terms of the group(s), time point(s), and measures involved?	Yes
Is accuracy matched across the second level contrast?	N/A, no behavioral measure
Is reaction time matched across the second level contrast?	N/A, no timeable task
Behavioral data notes	—
Type of analysis	Complex
Statistical details	Correlations were computed between activity in each voxel, and post-scan story recall, and were compared between patients without temporal damage and controls, in regions with a main effect of story comprehension. The threshold was $p < 0.05$ corrected, plus a minimum cluster size of 5 voxels.
Findings	None
Findings notes	—

Complex analysis 3

First level contrast	Listening to narrative speech vs listening to reversed speech
Analysis class	Cross-sectional aphasia vs control
Group(s)	Aphasia with temporal damage (n = 8) vs control
Covariate	—
Is the second level contrast valid in terms of the group(s), time point(s), and measures involved?	Yes
Is accuracy matched across the second level contrast?	N/A, no behavioral measure

Is reaction time matched across the second level contrast?	N/A, no timeable task
Behavioral data notes	—
Type of analysis	Complex
Statistical details	Correlations were computed between activity in each voxel, and post-scan story recall, and were compared between patients with temporal damage and controls, in regions with a main effect of story comprehension. The threshold was $p < 0.05$ corrected, plus a minimum cluster size of 5 voxels.
Findings	None
Findings notes	—

Complex analysis 4

First level contrast	Listening to narrative speech vs listening to reversed speech
Analysis class	Cross-sectional between two groups with aphasia
Group(s)	Aphasia with temporal damage (n = 8) vs without temporal damage (n = 9)
Covariate	—
Is the second level contrast valid in terms of the group(s), time point(s), and measures involved?	Yes
Is accuracy matched across the second level contrast?	N/A, no behavioral measure
Is reaction time matched across the second level contrast?	N/A, no timeable task
Behavioral data notes	—
Type of analysis	Complex
Statistical details	Correlations were computed between activity in each voxel, and post-scan story recall, and were compared between the two aphasia groups, in regions with a main effect of story comprehension. The threshold was $p < 0.05$ corrected, plus a minimum cluster size of 5 voxels.
Findings	None
Findings notes	—

Notes

Excluded analyses	An analysis involving associations between activations and story recognition memory because it included both controls and patients

de Boissezon et al. (2005)

Reference

Authors	de Boissezon X, Démonet JF, Puel M, Marie N, Raboyeau G, Albucher JF, Chollet F, Cardebat D
Title	Subcortical aphasia: a longitudinal PET study
Reference	*Stroke* 2005; 36: 1467-1473
PMID	15933252
DOI	10.1161/01.str.0000169947.08972.4f

Participants

Language	French
Inclusion criteria	Subcortical stroke; no severe aphasia
Number of individuals with aphasia	7
Number of control participants	0
Were any of the participants included in any previous studies?	No
Is age reported for patients and controls, and matched?	Yes (mean 52.4 ± 13 years, range 31-69 years)

Is sex reported for patients and controls, and matched?	Yes (males: 7; females: 0)
Is handedness reported for patients and controls, and matched?	Yes (right: 7; left: 0)
Is time post stroke onset reported and appropriate to the study design?	No* (moderate limitation) (T1: mean 53 ± 35 days, range 11-108 days; T2: mean 12.2 ± 1.4 months; T1 varies considerably from early to late subacute)
To what extent is the nature of aphasia characterized?	Type only
Language evaluation	Montreal-Toulouse Aphasia Battery
Aphasia severity	Not stated
Aphasia type	T1: 2 Broca's, 2 transcortical sensory, 1 anomic, 1 transcortical motor, 1 Wernicke's; T2: 4 recovered, 1 anomic, 1 transcortical motor; 1 transcortical sensory
First stroke only?	Yes
Stroke type	Mixed etiologies
To what extent is the lesion distribution characterized?	Individual lesions
Lesion extent	Not stated
Lesion location	5 L non-thalamic subcortical, 2 L thalamic
Participants notes	—

Imaging

Modality	PET (rCBF)
Is the study cross-sectional or longitudinal?	Longitudinal—recovery
If longitudinal, at what time point(s) were imaging data acquired?	T1: mean 53 ± 35 days, range 11-108 days; T2: mean 12.2 ± 1.4 months; T1 varies considerably from early to late subacute
If longitudinal, was there any intervention between the time points?	Not stated
Is the scanner described?	Yes (CTI-Siemens ECAT EXACT HR+)
Is the timing of stimulus presentation and image acquisition clearly described and appropriate?	Yes
Design type	PET
Total images acquired	6
Are the imaging acquisition parameters, including coverage, adequately described and appropriate?	Yes (whole brain)
Is preprocessing and intrasubject coregistration adequately described and appropriate?	Yes
Is first level model fitting adequately described and appropriate?	Yes
Is intersubject normalization adequately described and appropriate?	No (lesion impact not addressed; minimal due to lesions being small and subcortical)
Imaging notes	—

Conditions

Are the conditions clearly described?	Yes

Condition	Response type	Repetitions	All groups could do?	All individuals could do?
word generation	Word (overt)	4	Yes	Yes
rest	None	2	N/A	N/A

Conditions notes	Nouns in two runs, verbs in two runs, combined here because they were combined in analysis

Contrasts

Are the contrasts clearly described?	Yes

Contrast 1: word generation vs rest

Language condition	Word generation

Control condition	Rest
Are the conditions matched for visual demands?	Yes
Are the conditions matched for auditory demands?	No
Are the conditions matched for motor demands?	No
Are the conditions matched for cognitive/executive demands?	No
Is accuracy matched between the language and control tasks for all relevant groups?	N/A, tasks not comparable
Is reaction time matched between the language and control tasks for all relevant groups?	N/A, tasks not comparable
Behavioral data notes	—
Are control data reported in this paper or another that is referenced?	No
Does the contrast selectively activate plausible relevant language regions in the control group?	Unknown
Are activations lateralized in the control data?	Unknown
Control activation notes	—
Contrast notes	—

Analyses

Are the analyses clearly described?	No* (moderate limitation) (see specific limitation(s) below)

Voxelwise analysis 1

First level contrast	Word generation vs rest
Analysis class	Cross-sectional correlation with language or other measure
Group(s)	Aphasia T1
Covariate	Time post onset
Is the second level contrast valid in terms of the group(s), time point(s), and measures involved?	Yes
Is accuracy matched across the second level contrast?	Yes, matched
Is reaction time matched across the second level contrast?	Unknown, not reported
Behavioral data notes	No significant correlation between time post onset and accuracy
Type of analysis	Voxelwise
Search volume	Whole brain
Correction for multiple comparisons	Clusterwise correction based on arbitrary cluster extent
Software	SPM2
Voxelwise p	.01
Cluster extent	50 voxels (size not stated)
Statistical details	—
Findings	↑ L orbitofrontal<br>↑ L anterior temporal<br>↑ L occipital<br>↑ L anterior cingulate<br>↑ L cerebellum<br>↑ R anterior temporal<br>↑ R occipital
Findings notes	More activity with longer time post onset; based on coordinates in Table 3a

Voxelwise analysis 2

First level contrast	Word generation vs rest
Analysis class	Cross-sectional correlation with language or other measure
Group(s)	Aphasia T1
Covariate	Word generation accuracy T1
Is the second level contrast valid in terms of the	Yes

group(s), time point(s), and measures involved?	
Is accuracy matched across the second level contrast?	Accuracy is covariate
Is reaction time matched across the second level contrast?	Unknown, not reported
Behavioral data notes	—
Type of analysis	Voxelwise
Search volume	Whole brain
Correction for multiple comparisons	Clusterwise correction based on arbitrary cluster extent
Software	SPM2
Voxelwise p	.01
Cluster extent	50 voxels (size not stated)
Statistical details	—
Findings	↑ L IFG pars triangularis<br>↑ L dorsolateral prefrontal cortex<br>↑ L precuneus<br>↑ L Heschl's gyrus<br>↑ L anterior temporal<br>↑ R insula<br>↑ R posterior STG
Findings notes	Based on coordinates in Table 3b

Voxelwise analysis 3

First level contrast	Word generation vs rest
Analysis class	Longitudinal change in aphasia
Group(s)	Aphasia T2 vs T1
Covariate	—
Is the second level contrast valid in terms of the group(s), time point(s), and measures involved?	Yes
Is accuracy matched across the second level contrast?	No, different
Is reaction time matched across the second level contrast?	Unknown, not reported
Behavioral data notes	—
Type of analysis	Voxelwise
Search volume	Whole brain
Correction for multiple comparisons	Clusterwise correction based on arbitrary cluster extent
Software	SPM2
Voxelwise p	.001
Cluster extent	100 voxels (size not stated)
Statistical details	Description of masking unclear, but seems to be inclusively masked with T1, which seems inappropriate
Findings	↑ L insula<br>↑ L posterior STG<br>↑ R orbitofrontal<br>↑ R posterior STG<br>↑ R cerebellum
Findings notes	Based on coordinates in Table 2

Voxelwise analysis 4

First level contrast	Word generation vs rest
Analysis class	Longitudinal correlation with language or other measure
Group(s)	Aphasia T2 vs T1
Covariate	Δ word generation accuracy
Is the second level contrast valid in terms of the group(s), time point(s), and measures involved?	Yes

Is accuracy matched across the second level contrast?	Accuracy is covariate
Is reaction time matched across the second level contrast?	Unknown, not reported
Behavioral data notes	—
Type of analysis	Voxelwise
Search volume	Whole brain
Correction for multiple comparisons	Clusterwise correction based on arbitrary cluster extent
Software	SPM2
Voxelwise p	.01
Cluster extent	20 voxels (size not stated)
Statistical details	—
Findings	↑ L mid temporal<br>↑ R anterior temporal<br>↑ R cerebellum
Findings notes	Based on coordinates in Table 3c

Notes

Excluded analyses	—

Connor et al. (2006)

Reference

Authors	Connor LT, DeShazo Braby T, Snyder AZ, Lewis C, Blasi V, Corbetta M
Title	Cerebellar activity switches hemispheres with cerebral recovery in aphasia
Reference	*Neuropsychologia* 2006; 44: 171-177
PMID	16019040
DOI	10.1016/j.neuropsychologia.2005.05.019

Participants

Language	US English
Inclusion criteria	L IFG, possibly extending to neighboring regions
Number of individuals with aphasia	8
Number of control participants	14
Were any of the participants included in any previous studies?	Yes (re-analysis of data from Blasi et al. (2002))
Is age reported for patients and controls, and matched?	No (mean 48.6 years; patients and controls not closely matched for age, unclear if difference significant)
Is sex reported for patients and controls, and matched?	Yes (males: 2; females: 6)
Is handedness reported for patients and controls, and matched?	Yes (right: 8; left: 0)
Is time post stroke onset reported and appropriate to the study design?	No (> 6 months; actual TPO not stated)
To what extent is the nature of aphasia characterized?	Comprehensive battery
Language evaluation	WAB or BDAE
Aphasia severity	AQ range 66.5-89.0 in 6 participants, BDAE aphasia severity of 4 in 1 participant, no formal evaluation in 1 participant
Aphasia type	3 anomic, 3 transcortical motor, 1 Broca's, 1 not stated; most were Broca's or global acutely
First stroke only?	Yes
Stroke type	Ischemic only
To what extent is the lesion distribution	Individual lesions

characterized?	
Lesion extent	Not stated
Lesion location	L IFG and operculum, extending to adjacent cortex and white matter in several cases
Participants notes	—

Imaging

Modality	fMRI
Is the study cross-sectional or longitudinal?	Cross-sectional
If longitudinal, at what time point(s) were imaging data acquired?	—
If longitudinal, was there any intervention between the time points?	—
Is the scanner described?	Yes (Siemens Vision 1.5 Tesla)
Is the timing of stimulus presentation and image acquisition clearly described and appropriate?	Yes
Design type	Event-related
Total images acquired	1024
Are the imaging acquisition parameters, including coverage, adequately described and appropriate?	Yes (whole brain)
Is preprocessing and intrasubject coregistration adequately described and appropriate?	Yes
Is first level model fitting adequately described and appropriate?	Yes
Is intersubject normalization adequately described and appropriate?	Yes
Imaging notes	—

Conditions

Are the conditions clearly described?	Yes

Condition	Response type	Repetitions	All groups could do?	All individuals could do?
word stem completion (novel items)	Word (covert)	196	Yes	Unknown
word stem completion (repeated items)	Word (covert)	196	Yes	Unknown
rest	None	implicit baseline	N/A	N/A

Conditions notes	Novel items were presented in runs 1, 6, 7, and 8; repeated items were presented in runs 2, 3, 4, and 5; of the four repeated runs, only run 5 was analyzed.

Contrasts

Are the contrasts clearly described?	Yes

Contrast 1: word stem completion (novel items) vs word stem completion (repeated items)

Language condition	Word stem completion (novel items)
Control condition	Word stem completion (repeated items)
Are the conditions matched for visual demands?	Yes
Are the conditions matched for auditory demands?	Yes
Are the conditions matched for motor demands?	Yes
Are the conditions matched for cognitive/executive demands?	Yes
Is accuracy matched between the language and control tasks for all relevant groups?	Yes, matched
Is reaction time matched between the language and control tasks for all relevant groups?	No, different
Behavioral data notes	—
Are control data reported in this paper or another	Somewhat

that is referenced?	
Does the contrast selectively activate plausible relevant language regions in the control group?	Unknown
Are activations lateralized in the control data?	Somewhat
Control activation notes	No whole brain analysis of this contrast, but somewhat lateralized in the sense that L but not R frontal areas showed a learning effect
Contrast notes	The only contrast analyzed in this paper is the "learning" contrast which corresponds to contrast 2 in Blasi et al. (2002)

Analyses

Are the analyses clearly described?	No* (moderate limitation) (see specific limitation(s) below)

Voxelwise analysis 1

First level contrast	Word stem completion (novel items) vs word stem completion (repeated items)
Analysis class	Cross-sectional aphasia vs control
Group(s)	Aphasia vs control
Covariate	—
Is the second level contrast valid in terms of the group(s), time point(s), and measures involved?	Yes
Is accuracy matched across the second level contrast?	Yes, matched
Is reaction time matched across the second level contrast?	Yes, matched
Behavioral data notes	Covert task but overt data acquired separately; no interaction of group by practice for accuracy or RT
Type of analysis	Voxelwise
Search volume	Cerebellum
Correction for multiple comparisons	No direct comparison
Software	not stated
Voxelwise p	—
Cluster extent	—
Statistical details	Qualitative comparison on p. 174; Monte Carlo-based thresholding not described; rather than fitting a HRF, the authors looked at the shape of the signal in the 8 volumes following each stimulus
Findings	↑ L cerebellum<br>↓ R cerebellum
Findings notes	—

ROI analysis 1

First level contrast	Word stem completion (novel items) vs word stem completion (repeated items)
Analysis class	Cross-sectional aphasia vs control
Group(s)	Aphasia vs control
Covariate	—
Is the second level contrast valid in terms of the group(s), time point(s), and measures involved?	Yes
Is accuracy matched across the second level contrast?	Yes, matched
Is reaction time matched across the second level contrast?	Yes, matched
Behavioral data notes	Covert task but overt data acquired separately; no interaction of group by practice for accuracy or RT
Type of analysis	Region of interest (ROI)
ROI type	Functional
How many ROIs are there?	1
What are the ROI(s)?	L cerebellum
How are the ROI(s) defined?	L cerebellar region with a learning effect in the patients

Correction for multiple comparisons	One only
Statistical details	Circular because ROIs defined in one group; rather than fitting a HRF, the authors looked at the shape of the signal in the 8 volumes following each stimulus
Findings	↑ L cerebellum
Findings notes	—

Notes

Excluded analyses	(1) analysis of frontal changes is excluded since it appears to be identical to Blasi et al. (2002); (2) the analyses involving mirrored cerebellar regions are excluded since the groups were not compared directly

Crinion et al. (2006)

Reference

Authors	Crinion JT, Warburton EA, Lambon-Ralph MA, Howard D, Wise RJ
Title	Listening to narrative speech after aphasic stroke: the role of the left anterior temporal lobe
Reference	*Cereb Cortex* 2006; 16: 1116-1125
PMID	16251507
DOI	10.1093/cercor/bhj053

Participants

Language	UK English
Inclusion criteria	—
Number of individuals with aphasia	24
Number of control participants	11
Were any of the participants included in any previous studies?	No
Is age reported for patients and controls, and matched?	Yes (range 32-85 years)
Is sex reported for patients and controls, and matched?	Yes (males: 18; females: 6)
Is handedness reported for patients and controls, and matched?	Yes (right: 24; left: 0)
Is time post stroke onset reported and appropriate to the study design?	No (mean 32 months, range 2-204 months; combines subacute and chronic patients)
To what extent is the nature of aphasia characterized?	Comprehensive battery
Language evaluation	CAT (missing in two participants)
Aphasia severity	Not stated
Aphasia type	Not stated
First stroke only?	Yes
Stroke type	Not stated
To what extent is the lesion distribution characterized?	Lesion overlay
Lesion extent	Not stated
Lesion location	6 L but no temporal damage, 9 L temporal damage excluding anterior temporal cortex, 9 L temporal damage including anterior temporal cortex
Participants notes	Results of control participants previously reported in Crinion et al. (2003)

Imaging

Modality	PET (rCBF)
Is the study cross-sectional or longitudinal?	Cross-sectional
If longitudinal, at what time point(s) were imaging	—

data acquired?	
If longitudinal, was there any intervention between the time points?	—
Is the scanner described?	Yes (CTI-Siemens ECAT EXACT HR++/966 (16 patients and all controls) or GE Advance (8 patients))
Is the timing of stimulus presentation and image acquisition clearly described and appropriate?	Yes
Design type	PET
Total images acquired	12-16
Are the imaging acquisition parameters, including coverage, adequately described and appropriate?	Yes (whole brain)
Is preprocessing and intrasubject coregistration adequately described and appropriate?	Yes
Is first level model fitting adequately described and appropriate?	Yes
Is intersubject normalization adequately described and appropriate?	Yes
Imaging notes	two different scanners used for patients, but not for controls

Conditions

Are the conditions clearly described?	Yes

Condition	Response type	Repetitions	All groups could do?	All individuals could do?
listening to narrative speech	None	6-8	N/A	N/A
listening to reversed speech	None	6-8	N/A	N/A

Conditions notes	—

Contrasts

Are the contrasts clearly described?	Yes

Contrast 1: listening to narrative speech vs listening to reversed speech

Language condition	Listening to narrative speech
Control condition	Listening to reversed speech
Are the conditions matched for visual demands?	Yes
Are the conditions matched for auditory demands?	Yes
Are the conditions matched for motor demands?	Yes
Are the conditions matched for cognitive/executive demands?	Yes
Is accuracy matched between the language and control tasks for all relevant groups?	N/A, no behavioral measure
Is reaction time matched between the language and control tasks for all relevant groups?	N/A, no timeable task
Behavioral data notes	—
Are control data reported in this paper or another that is referenced?	Somewhat
Does the contrast selectively activate plausible relevant language regions in the control group?	Yes
Are activations lateralized in the control data?	Somewhat
Control activation notes	11 participants; L-lateralized posterior temporal, bilateral anterior temporal, no frontal
Contrast notes	—

Analyses

Are the analyses clearly described?	Yes

Voxelwise analysis 1

First level contrast	Listening to narrative speech vs listening to reversed speech
Analysis class	Cross-sectional aphasia vs control
Group(s)	Aphasia vs control
Covariate	—
Is the second level contrast valid in terms of the group(s), time point(s), and measures involved?	Yes
Is accuracy matched across the second level contrast?	N/A, no behavioral measure
Is reaction time matched across the second level contrast?	N/A, no timeable task
Behavioral data notes	—
Type of analysis	Voxelwise
Search volume	Voxels spared in all patients
Correction for multiple comparisons	Voxelwise FWE correction
Software	SPM99
Voxelwise p	FWE p < .05
Cluster extent	—
Statistical details	—
Findings	None
Findings notes	—

Voxelwise analysis 2

First level contrast	Listening to narrative speech vs listening to reversed speech
Analysis class	Cross-sectional aphasia vs control
Group(s)	Aphasia without temporal lobe damage (n = 6) vs control
Covariate	—
Is the second level contrast valid in terms of the group(s), time point(s), and measures involved?	Yes
Is accuracy matched across the second level contrast?	N/A, no behavioral measure
Is reaction time matched across the second level contrast?	N/A, no timeable task
Behavioral data notes	—
Type of analysis	Voxelwise
Search volume	Voxels spared in all included patients
Correction for multiple comparisons	Voxelwise FWE correction
Software	SPM99
Voxelwise p	FWE p < .05
Cluster extent	—
Statistical details	—
Findings	None
Findings notes	—

Voxelwise analysis 3

First level contrast	Listening to narrative speech vs listening to reversed speech
Analysis class	Cross-sectional aphasia vs control
Group(s)	Aphasia with temporal lobe damage (n = 18) vs control
Covariate	—
Is the second level contrast valid in terms of the group(s), time point(s), and measures involved?	Yes
Is accuracy matched across the second level contrast?	N/A, no behavioral measure
Is reaction time matched across the second level contrast?	N/A, no timeable task
Behavioral data notes	—

Type of analysis	Voxelwise
Search volume	Voxels spared in all included patients
Correction for multiple comparisons	Voxelwise FWE correction
Software	SPM99
Voxelwise p	FWE $p < .05$
Cluster extent	—
Statistical details	—
Findings	None
Findings notes	—

ROI analysis 1

First level contrast	Listening to narrative speech vs listening to reversed speech
Analysis class	Cross-sectional correlation with language or other measure
Group(s)	Aphasia with no temporal damage (excluding 1 with missing behavioral data and 1 outlier) or posterior temporal damage sparing anterior temporal cortex (n = 13)
Covariate	Auditory sentence comprehension (CAT)
Is the second level contrast valid in terms of the group(s), time point(s), and measures involved?	Yes
Is accuracy matched across the second level contrast?	N/A, no behavioral measure
Is reaction time matched across the second level contrast?	N/A, no timeable task
Behavioral data notes	—
Type of analysis	Region of interest (ROI)
ROI type	Functional
How many ROIs are there?	1
What are the ROI(s)?	L ATL
How are the ROI(s) defined?	Activation in the control group
Correction for multiple comparisons	One only
Statistical details	Same result obtained with or without excluding one outlier; two other ROIs are described in the methods, but never used in any analyses
Findings	↑ L anterior temporal
Findings notes	More activity in patients with better auditory sentence comprehension

ROI analysis 2

First level contrast	Listening to narrative speech vs listening to reversed speech
Analysis class	Cross-sectional correlation with language or other measure
Group(s)	Aphasia with no temporal damage (excluding 1 with missing behavioral data and 1 outlier) or posterior temporal damage sparing anterior temporal cortex (n = 13)
Covariate	Time post onset
Is the second level contrast valid in terms of the group(s), time point(s), and measures involved?	Yes
Is accuracy matched across the second level contrast?	N/A, no behavioral measure
Is reaction time matched across the second level contrast?	N/A, no timeable task
Behavioral data notes	—
Type of analysis	Region of interest (ROI)
ROI type	Functional
How many ROIs are there?	1
What are the ROI(s)?	L ATL
How are the ROI(s) defined?	Activation in the control group
Correction for multiple comparisons	One only
Statistical details	Two other ROIs are described in the methods, but never used in any analyses
Findings	None

Findings notes	—

ROI analysis 3

First level contrast	Listening to narrative speech vs listening to reversed speech
Analysis class	Cross-sectional between two groups with aphasia
Group(s)	Aphasia with temporal damage excluding anterior temporal cortex (n = 9) vs with no temporal lobe damage (excluding 1 with missing behavioral data and 1 outlier) (n = 4)
Covariate	—
Is the second level contrast valid in terms of the group(s), time point(s), and measures involved?	Yes
Is accuracy matched across the second level contrast?	N/A, no behavioral measure
Is reaction time matched across the second level contrast?	N/A, no timeable task
Behavioral data notes	—
Type of analysis	Region of interest (ROI)
ROI type	Functional
How many ROIs are there?	1
What are the ROI(s)?	L ATL
How are the ROI(s) defined?	Activation in the control group
Correction for multiple comparisons	One only
Statistical details	Two other ROIs are described in the methods, but never used in any analyses
Findings	↓ L anterior temporal
Findings notes	Patients with posterior temporal damage had less signal change

ROI analysis 4

First level contrast	Listening to narrative speech vs listening to reversed speech
Analysis class	Cross-sectional aphasia vs control
Group(s)	Aphasia with temporal damage excluding anterior temporal cortex (n = 9) vs control
Covariate	—
Is the second level contrast valid in terms of the group(s), time point(s), and measures involved?	Yes
Is accuracy matched across the second level contrast?	N/A, no behavioral measure
Is reaction time matched across the second level contrast?	N/A, no timeable task
Behavioral data notes	—
Type of analysis	Region of interest (ROI)
ROI type	Functional
How many ROIs are there?	1
What are the ROI(s)?	L ATL
How are the ROI(s) defined?	Activation in the control group
Correction for multiple comparisons	One only
Statistical details	Circular because ROI defined in one group; two other ROIs are described in the methods, but never used in any analyses
Findings	↓ L anterior temporal
Findings notes	Large difference 2.7 ± 0.8 (patients) vs 6.3 ± 1.4 (controls) makes finding suggestive even in light of the circularity

ROI analysis 5

First level contrast	Listening to narrative speech vs listening to reversed speech
Analysis class	Cross-sectional correlation with language or other measure
Group(s)	Aphasia with no temporal damage (excluding 1 with missing behavioral data and 1 outlier) or posterior temporal damage sparing anterior temporal cortex (n = 13)
Covariate	Auditory single word comprehension (CAT)

Is the second level contrast valid in terms of the group(s), time point(s), and measures involved?	Yes
Is accuracy matched across the second level contrast?	N/A, no behavioral measure
Is reaction time matched across the second level contrast?	N/A, no timeable task
Behavioral data notes	—
Type of analysis	Region of interest (ROI)
ROI type	Functional
How many ROIs are there?	1
What are the ROI(s)?	L ATL
How are the ROI(s) defined?	Activation in the control group
Correction for multiple comparisons	One only
Statistical details	Two other ROIs are described in the methods, but never used in any analyses
Findings	None
Findings notes	R = 0.39; p > 0.1; seems to be a clear trend so lack of significance may reflect only lack of power

Notes

Excluded analyses	—

Saur et al. (2006)

Reference

Authors	Saur D, Lange R, Baumgaertner A, Schraknepper V, Willmes K, Rijntjes M, Weiller C
Title	Dynamics of language reorganization after stroke
Reference	*Brain* 2006; 129: 1371-1384
PMID	16638796
DOI	10.1093/brain/awl090

Participants

Language	German
Inclusion criteria	MCA; age < 70 years; able to distinguish forward vs backward speech outside the scanner; no pronounced small vessel disease
Number of individuals with aphasia	14 (plus 4 excluded: 1 health problems; 1 scanner noise; 2 did not tolerate fMRI)
Number of control participants	14
Were any of the participants included in any previous studies?	No
Is age reported for patients and controls, and matched?	Yes (mean 51.9 ± 14.2 years, range 16-68 years)
Is sex reported for patients and controls, and matched?	Yes (males: 11; females: 3)
Is handedness reported for patients and controls, and matched?	Yes (right: 12; left: 1; other: 1)
Is time post stroke onset reported and appropriate to the study design?	Yes (T1 acute: mean 1.8 days, range 0-4 days; T2 subacute: mean 12.1 days, range 3-16 days; T3 chronic: mean 321 days, range 102-513 days)
To what extent is the nature of aphasia characterized?	Comprehensive battery
Language evaluation	AABT, AAT including TT, analysis of spontaneous speech, CETI, Language Recovery Score (LRS) derived from all these measures plus in-scanner task performance
Aphasia severity	T1: LRS mean 0.44, range 0.11-0.81; 1 mild, 1 mild-moderate, 7 moderate, 3 moderate-severe, 2 severe per AAT; T2: LRS mean 0.71, range 0.33-0.92; 2 recovered, 2 recovered-mild, 2 mild, 3 mild-moderate, 3 moderate, 2 severe per AAT; T3: LRS mean 0.91, range 0.66-1.00; 8 recovered, 2 recovered-mild, 3 mild, 1 moderate per AAT

Aphasia type	T1: 9 non-fluent, 5 fluent; T2: not stated; T3: 6 recovered, 4 minimal language impairment, 3 anomic, 1 global
First stroke only?	Yes
Stroke type	Ischemic only
To what extent is the lesion distribution characterized?	Individual lesions
Lesion extent	Not stated
Lesion location	L MCA; 4 frontal (2 extending to temporoparietal); 5 temporoparietal (2 extending to subcortical); 4 striatocapsular (2 extending to cortical); 1 frontoparietal
Participants notes	198 patients with aphasia were screened

Imaging

Modality	fMRI
Is the study cross-sectional or longitudinal?	Longitudinal—recovery
If longitudinal, at what time point(s) were imaging data acquired?	T1 acute: mean 1.8 days, range 0-4 days; T2 subacute: mean 12.1 days, range 3-16 days; T3 chronic: mean 321 days, range 102-513 days
If longitudinal, was there any intervention between the time points?	Standard SLT throughout the observation period including at least 3 weeks inpatient
Is the scanner described?	Yes (Siemens Trio 3 Tesla)
Is the timing of stimulus presentation and image acquisition clearly described and appropriate?	Yes
Design type	Event-related
Total images acquired	660
Are the imaging acquisition parameters, including coverage, adequately described and appropriate?	Yes (whole brain)
Is preprocessing and intrasubject coregistration adequately described and appropriate?	Yes
Is first level model fitting adequately described and appropriate?	Yes
Is intersubject normalization adequately described and appropriate?	Yes
Imaging notes	—

Conditions

Are the conditions clearly described?	Yes

Condition	Response type	Repetitions	All groups could do?	All individuals could do?
listening to sentences and making a plausibility judgment	Button press	92	Unknown	No
listening to reversed speech	Button press	92	Yes	Unknown
rest	None	implicit baseline	N/A	N/A

Conditions notes	In the auditory sentence comprehension condition, participants had to press a button to semantically anomalous sentences; in the reversed speech condition, they had to always press the button; the behavioral scores provided are not explained in the paper, but per a personal communication cited by Geranmayeh et al. (2014), 10% of the score reflects discrimination between intelligible and reversed speech, while 90% reflects semantic anomaly judgment; our coding of behavior is based on this limited information

Contrasts

Are the contrasts clearly described?	Yes

Contrast 1: listening to sentences and making a plausibility judgment vs listening to reversed speech

Language condition	Listening to sentences and making a plausibility judgment
Control condition	Listening to reversed speech

Are the conditions matched for visual demands?	Yes
Are the conditions matched for auditory demands?	Yes
Are the conditions matched for motor demands?	No
Are the conditions matched for cognitive/executive demands?	No
Is accuracy matched between the language and control tasks for all relevant groups?	Unknown, not reported
Is reaction time matched between the language and control tasks for all relevant groups?	Unknown, not reported
Behavioral data notes	Reported accuracy combines the two conditions in a way that is not explained
Are control data reported in this paper or another that is referenced?	Yes
Does the contrast selectively activate plausible relevant language regions in the control group?	Yes
Are activations lateralized in the control data?	Yes
Control activation notes	L temporal and L > R frontal
Contrast notes	—

Analyses

Are the analyses clearly described?	Yes

Voxelwise analysis 1

First level contrast	Listening to sentences and making a plausibility judgment vs listening to reversed speech
Analysis class	Longitudinal change in aphasia
Group(s)	Aphasia T2 vs T1
Covariate	—
Is the second level contrast valid in terms of the group(s), time point(s), and measures involved?	Yes
Is accuracy matched across the second level contrast?	Appear mismatched
Is reaction time matched across the second level contrast?	Unknown, not reported
Behavioral data notes	Accuracy combines language and control conditions
Type of analysis	Voxelwise
Search volume	Whole brain
Correction for multiple comparisons	No correction
Software	SPM2
Voxelwise p	.001
Cluster extent	None
Statistical details	—
Findings	↑ L insula<br>↑ R IFG pars orbitalis<br>↑ R insula<br>↑ R SMA/medial prefrontal
Findings notes	R IFG/insula activation noted to survive FWE correction at p < .05

Voxelwise analysis 2

First level contrast	Listening to sentences and making a plausibility judgment vs listening to reversed speech
Analysis class	Longitudinal change in aphasia
Group(s)	Aphasia T3 vs T2
Covariate	—
Is the second level contrast valid in terms of the group(s), time point(s), and measures involved?	Yes
Is accuracy matched across the second level contrast?	Appear mismatched
Is reaction time matched across the second level	Unknown, not reported

contrast?	
Behavioral data notes	Accuracy combines language and control conditions
Type of analysis	Voxelwise
Search volume	Whole brain
Correction for multiple comparisons	No correction
Software	SPM2
Voxelwise p	.005
Cluster extent	None
Statistical details	Threshold was lowered to reveal the R frontal change in activation
Findings	↓ R IFG pars orbitalis<br>↓ R occipital
Findings notes	—

Voxelwise analysis 3

First level contrast	Listening to sentences and making a plausibility judgment vs listening to reversed speech
Analysis class	Longitudinal change in aphasia
Group(s)	Aphasia T3 vs T1
Covariate	—
Is the second level contrast valid in terms of the group(s), time point(s), and measures involved?	Yes
Is accuracy matched across the second level contrast?	Appear mismatched
Is reaction time matched across the second level contrast?	Unknown, not reported
Behavioral data notes	Accuracy combines language and control conditions
Type of analysis	Voxelwise
Search volume	Whole brain
Correction for multiple comparisons	No correction
Software	SPM2
Voxelwise p	.001
Cluster extent	None
Statistical details	—
Findings	↑ L IFG pars orbitalis<br>↑ L SMA/medial prefrontal<br>↑ L posterior inferior temporal gyrus/fusiform gyrus<br>↑ R IFG pars orbitalis<br>↑ R insula
Findings notes	—

Voxelwise analysis 4

First level contrast	Listening to sentences and making a plausibility judgment vs listening to reversed speech
Analysis class	Cross-sectional aphasia vs control
Group(s)	Aphasia T1 vs control
Covariate	—
Is the second level contrast valid in terms of the group(s), time point(s), and measures involved?	Yes
Is accuracy matched across the second level contrast?	Appear mismatched
Is reaction time matched across the second level contrast?	Unknown, not reported
Behavioral data notes	Accuracy combines language and control conditions
Type of analysis	Voxelwise
Search volume	Whole brain
Correction for multiple comparisons	No correction
Software	SPM2
Voxelwise p	.001

Cluster extent	None
Statistical details	—
Findings	↓ L IFG pars triangularis<br>↓ L IFG pars orbitalis<br>↓ L insula<br>↓ L posterior MTG<br>↓ L posterior inferior temporal gyrus/fusiform gyrus<br>↓ R IFG pars orbitalis<br>↓ R insula
Findings notes	L STG in table is actually MTG based on coordinates

Voxelwise analysis 5

First level contrast	Listening to sentences and making a plausibility judgment vs listening to reversed speech
Analysis class	Cross-sectional aphasia vs control
Group(s)	Aphasia T2 vs control
Covariate	—
Is the second level contrast valid in terms of the group(s), time point(s), and measures involved?	Yes
Is accuracy matched across the second level contrast?	Appear mismatched
Is reaction time matched across the second level contrast?	Unknown, not reported
Behavioral data notes	Accuracy combines language and control conditions
Type of analysis	Voxelwise
Search volume	Whole brain
Correction for multiple comparisons	No correction
Software	SPM2
Voxelwise p	.005
Cluster extent	None
Statistical details	Threshold was lowered to reveal L IFG
Findings	↑ L IFG pars orbitalis<br>↑ L insula<br>↑ L SMA/medial prefrontal<br>↑ R IFG
Findings notes	—

Voxelwise analysis 6

First level contrast	Listening to sentences and making a plausibility judgment vs listening to reversed speech
Analysis class	Cross-sectional aphasia vs control
Group(s)	Aphasia T3 vs control
Covariate	—
Is the second level contrast valid in terms of the group(s), time point(s), and measures involved?	Yes
Is accuracy matched across the second level contrast?	Appear similar
Is reaction time matched across the second level contrast?	Unknown, not reported
Behavioral data notes	Accuracy combines language and control conditions
Type of analysis	Voxelwise
Search volume	Whole brain
Correction for multiple comparisons	No correction
Software	SPM2
Voxelwise p	.001
Cluster extent	None
Statistical details	—
Findings	None

Findings notes	—

Voxelwise analysis 7

First level contrast	Listening to sentences and making a plausibility judgment vs listening to reversed speech
Analysis class	Cross-sectional correlation with language or other measure
Group(s)	Aphasia T1
Covariate	Language recovery score T1
Is the second level contrast valid in terms of the group(s), time point(s), and measures involved?	Yes
Is accuracy matched across the second level contrast?	Appear mismatched
Is reaction time matched across the second level contrast?	Unknown, not reported
Behavioral data notes	Accuracy combines language and control conditions
Type of analysis	Voxelwise
Search volume	Whole brain
Correction for multiple comparisons	No correction
Software	SPM2
Voxelwise p	.001
Cluster extent	None
Statistical details	—
Findings	↑ L IFG<br>↑ L SMA/medial prefrontal<br>↑ R IFG pars triangularis
Findings notes	—

Voxelwise analysis 8

First level contrast	Listening to sentences and making a plausibility judgment vs listening to reversed speech
Analysis class	Cross-sectional correlation with language or other measure
Group(s)	Aphasia T2
Covariate	Language recovery score T2
Is the second level contrast valid in terms of the group(s), time point(s), and measures involved?	Yes
Is accuracy matched across the second level contrast?	Unknown, no test
Is reaction time matched across the second level contrast?	Unknown, not reported
Behavioral data notes	Accuracy combines language and control conditions
Type of analysis	Voxelwise
Search volume	Whole brain
Correction for multiple comparisons	No correction
Software	SPM2
Voxelwise p	.001
Cluster extent	None
Statistical details	—
Findings	None
Findings notes	—

Voxelwise analysis 9

First level contrast	Listening to sentences and making a plausibility judgment vs listening to reversed speech
Analysis class	Cross-sectional correlation with language or other measure
Group(s)	Aphasia T3
Covariate	Language recovery score T3
Is the second level contrast valid in terms of the group(s), time point(s), and measures involved?	Yes

Is accuracy matched across the second level contrast?	Unknown, no test
Is reaction time matched across the second level contrast?	Unknown, not reported
Behavioral data notes	Accuracy combines language and control conditions
Type of analysis	Voxelwise
Search volume	Whole brain
Correction for multiple comparisons	No correction
Software	SPM2
Voxelwise p	.001
Cluster extent	None
Statistical details	—
Findings	None
Findings notes	—

Voxelwise analysis 10

First level contrast	Listening to sentences and making a plausibility judgment vs listening to reversed speech
Analysis class	Longitudinal correlation with language or other measure
Group(s)	Aphasia T2 vs T1
Covariate	% change in language recovery score
Is the second level contrast valid in terms of the group(s), time point(s), and measures involved?	Yes
Is accuracy matched across the second level contrast?	Unknown, no test
Is reaction time matched across the second level contrast?	Unknown, not reported
Behavioral data notes	Accuracy combines language and control conditions
Type of analysis	Voxelwise
Search volume	Whole brain
Correction for multiple comparisons	No correction
Software	SPM2
Voxelwise p	.001
Cluster extent	None
Statistical details	—
Findings	↑ L SMA/medial prefrontal<br>↑ R insula<br>↑ R SMA/medial prefrontal
Findings notes	—

Voxelwise analysis 11

First level contrast	Listening to sentences and making a plausibility judgment vs listening to reversed speech
Analysis class	Longitudinal correlation with language or other measure
Group(s)	Aphasia T3 vs T2
Covariate	% change in language recovery score
Is the second level contrast valid in terms of the group(s), time point(s), and measures involved?	Yes
Is accuracy matched across the second level contrast?	Unknown, no test
Is reaction time matched across the second level contrast?	Unknown, not reported
Behavioral data notes	Accuracy combines language and control conditions
Type of analysis	Voxelwise
Search volume	Whole brain
Correction for multiple comparisons	No correction
Software	SPM2

Voxelwise p	.001
Cluster extent	None
Statistical details	—
Findings	None
Findings notes	—

Voxelwise analysis 12

First level contrast	Listening to sentences and making a plausibility judgment vs listening to reversed speech
Analysis class	Longitudinal correlation with language or other measure
Group(s)	Aphasia T3 vs T1
Covariate	% change in language recovery score
Is the second level contrast valid in terms of the group(s), time point(s), and measures involved?	Yes
Is accuracy matched across the second level contrast?	Unknown, no test
Is reaction time matched across the second level contrast?	Unknown, not reported
Behavioral data notes	Accuracy combines language and control conditions
Type of analysis	Voxelwise
Search volume	Whole brain
Correction for multiple comparisons	No correction
Software	SPM2
Voxelwise p	.001
Cluster extent	None
Statistical details	—
Findings	None
Findings notes	—

ROI analysis 1

First level contrast	Listening to sentences and making a plausibility judgment vs listening to reversed speech
Analysis class	Longitudinal change in aphasia
Group(s)	Aphasia T2 vs T1
Covariate	—
Is the second level contrast valid in terms of the group(s), time point(s), and measures involved?	Yes
Is accuracy matched across the second level contrast?	Appear mismatched
Is reaction time matched across the second level contrast?	Unknown, not reported
Behavioral data notes	Accuracy combines language and control conditions
Type of analysis	Regions of interest (ROI)
ROI type	Functional
How many ROIs are there?	6
What are the ROI(s)?	(1) L IFG pars orbitalis; (2) L IFG pars triangularis; (3) L MTG; (4) R insula; (5) R IFG pars triangularis; (6) R SMA
How are the ROI(s) defined?	Peak voxels of overall activation map based on all three time points in patients
Correction for multiple comparisons	Familywise error (FWE)
Statistical details	—
Findings	↑ R insula<br>↑ R SMA/medial prefrontal
Findings notes	Some other ROIs also significant prior to correction for multiple comparisons; n.b. performance confound

ROI analysis 2

First level contrast	Listening to sentences and making a plausibility judgment vs listening to reversed speech

Analysis class	Longitudinal change in aphasia
Group(s)	Aphasia T3 vs T2
Covariate	—
Is the second level contrast valid in terms of the group(s), time point(s), and measures involved?	Yes
Is accuracy matched across the second level contrast?	Appear mismatched
Is reaction time matched across the second level contrast?	Unknown, not reported
Behavioral data notes	Accuracy combines language and control conditions
Type of analysis	Regions of interest (ROI)
ROI type	Functional
How many ROIs are there?	6
What are the ROI(s)?	(1) L IFG pars orbitalis; (2) L IFG pars triangularis; (3) L MTG; (4) R insula; (5) R IFG pars triangularis; (6) R SMA
How are the ROI(s) defined?	Peak voxels of overall activation map based on all three time points in patients
Correction for multiple comparisons	Familywise error (FWE)
Statistical details	—
Findings	None
Findings notes	Some other ROIs also significant prior to correction for multiple comparisons; n.b. performance confound

ROI analysis 3

First level contrast	Listening to sentences and making a plausibility judgment vs listening to reversed speech
Analysis class	Longitudinal change in aphasia
Group(s)	Aphasia T3 vs T1
Covariate	—
Is the second level contrast valid in terms of the group(s), time point(s), and measures involved?	Yes
Is accuracy matched across the second level contrast?	Appear mismatched
Is reaction time matched across the second level contrast?	Unknown, not reported
Behavioral data notes	Accuracy combines language and control conditions
Type of analysis	Regions of interest (ROI)
ROI type	Functional
How many ROIs are there?	6
What are the ROI(s)?	(1) L IFG pars orbitalis; (2) L IFG pars triangularis; (3) L MTG; (4) R insula; (5) R IFG pars triangularis; (6) R SMA
How are the ROI(s) defined?	Peak voxels of overall activation map based on all three time points in patients
Correction for multiple comparisons	Familywise error (FWE)
Statistical details	—
Findings	↑ L posterior MTG
Findings notes	Some other ROIs also significant prior to correction for multiple comparisons; n.b. performance confound

ROI analysis 4

First level contrast	Listening to sentences and making a plausibility judgment vs listening to reversed speech
Analysis class	Cross-sectional aphasia vs control
Group(s)	Aphasia T1 vs control
Covariate	—
Is the second level contrast valid in terms of the group(s), time point(s), and measures involved?	Yes
Is accuracy matched across the second level contrast?	Appear mismatched
Is reaction time matched across the second level	Unknown, not reported

contrast?	
Behavioral data notes	Accuracy combines language and control conditions
Type of analysis	Regions of interest (ROI)
ROI type	Functional
How many ROIs are there?	6
What are the ROI(s)?	(1) L IFG pars orbitalis; (2) L IFG pars triangularis; (3) L MTG; (4) R insula; (5) R IFG pars triangularis; (6) R SMA
How are the ROI(s) defined?	Peak voxels of overall activation map based on all three time points in patients
Correction for multiple comparisons	No correction
Statistical details	Circular because ROIs defined in one group
Findings	↓ L posterior MTG<br>↓ R IFG pars triangularis
Findings notes	R IFG difference described in text but not table

ROI analysis 5

First level contrast	Listening to sentences and making a plausibility judgment vs listening to reversed speech
Analysis class	Cross-sectional aphasia vs control
Group(s)	Aphasia T2 vs control
Covariate	—
Is the second level contrast valid in terms of the group(s), time point(s), and measures involved?	Yes
Is accuracy matched across the second level contrast?	Appear mismatched
Is reaction time matched across the second level contrast?	Unknown, not reported
Behavioral data notes	Accuracy combines language and control conditions
Type of analysis	Regions of interest (ROI)
ROI type	Functional
How many ROIs are there?	6
What are the ROI(s)?	(1) L IFG pars orbitalis; (2) L IFG pars triangularis; (3) L MTG; (4) R insula; (5) R IFG pars triangularis; (6) R SMA
How are the ROI(s) defined?	Peak voxels of overall activation map based on all three time points in patients
Correction for multiple comparisons	No correction
Statistical details	Circular because ROIs defined in one group
Findings	None
Findings notes	—

ROI analysis 6

First level contrast	Listening to sentences and making a plausibility judgment vs listening to reversed speech
Analysis class	Cross-sectional aphasia vs control
Group(s)	Aphasia T3 vs control
Covariate	—
Is the second level contrast valid in terms of the group(s), time point(s), and measures involved?	Yes
Is accuracy matched across the second level contrast?	Appear similar
Is reaction time matched across the second level contrast?	Unknown, not reported
Behavioral data notes	Accuracy combines language and control conditions
Type of analysis	Regions of interest (ROI)
ROI type	Functional
How many ROIs are there?	6
What are the ROI(s)?	(1) L IFG pars orbitalis; (2) L IFG pars triangularis; (3) L MTG; (4) R insula; (5) R IFG pars triangularis; (6) R SMA
How are the ROI(s) defined?	Peak voxels of overall activation map based on all three time points in patients

Correction for multiple comparisons	No correction
Statistical details	Circular because ROIs defined in one group
Findings	None
Findings notes	—

Notes

Excluded analyses	Additional analyses using absolute improvements in LRS instead of proportional improvements

Meinzer et al. (2008)

Reference

Authors	Meinzer M, Flaisch T, Breitenstein C, Wienbruch C, Elbert T, Rockstroh B
Title	Functional re-recruitment of dysfunctional brain areas predicts language recovery in chronic aphasia
Reference	*NeuroImage* 2008; 39: 2038-2046
PMID	18096407
DOI	10.1016/j.neuroimage.2007.10.008

Participants

Language	German
Inclusion criteria	—
Number of individuals with aphasia	11
Number of control participants	0
Were any of the participants included in any previous studies?	No
Is age reported for patients and controls, and matched?	Yes (median 51.0 years, range 19-66 years)
Is sex reported for patients and controls, and matched?	Yes (males: 7; females: 4)
Is handedness reported for patients and controls, and matched?	Yes (right: 11; left: 0)
Is time post stroke onset reported and appropriate to the study design?	Yes (median 32 months; range 6-480 months)
To what extent is the nature of aphasia characterized?	Comprehensive battery
Language evaluation	AAT, study-specific picture naming test with 150 items
Aphasia severity	6 moderate, 4 mild, 1 severe
Aphasia type	7 Broca's, 2 Wernicke's, 1 global, 1 unclassified
First stroke only?	Not stated
Stroke type	Mixed etiologies
To what extent is the lesion distribution characterized?	Lesion overlay
Lesion extent	Range 31.0-236.0 cc
Lesion location	L
Participants notes	—

Imaging

Modality	fMRI
Is the study cross-sectional or longitudinal?	Longitudinal—chronic treatment
If longitudinal, at what time point(s) were imaging data acquired?	T1: pre-treatment/chronic; T2: post-treatment, ~2 weeks later
If longitudinal, was there any intervention between	CIAT, 3 hours/day, 5 days/week, 2 weeks

the time points?	
Is the scanner described?	Yes (Philips Intera 1.5 Tesla)
Is the timing of stimulus presentation and image acquisition clearly described and appropriate?	Yes
Design type	Block
Total images acquired	160
Are the imaging acquisition parameters, including coverage, adequately described and appropriate?	Yes (whole brain)
Is preprocessing and intrasubject coregistration adequately described and appropriate?	Yes
Is first level model fitting adequately described and appropriate?	Yes
Is intersubject normalization adequately described and appropriate?	Yes
Imaging notes	—

Conditions

Are the conditions clearly described?	Yes

Condition	Response type	Repetitions	All groups could do?	All individuals could do?
picture naming (trained items)	Word (overt)	8	Yes	No
picture naming (untrained items)	Word (overt)	8	Yes	No
rest	None	16	N/A	N/A

Conditions notes	One participant was < 10% on trained and untrained items at T1

Contrasts

Are the contrasts clearly described?	Yes

Contrast 1: picture naming (trained items) vs rest

Language condition	Picture naming (trained items)
Control condition	Rest
Are the conditions matched for visual demands?	No
Are the conditions matched for auditory demands?	No
Are the conditions matched for motor demands?	No
Are the conditions matched for cognitive/executive demands?	No
Is accuracy matched between the language and control tasks for all relevant groups?	N/A, tasks not comparable
Is reaction time matched between the language and control tasks for all relevant groups?	N/A, tasks not comparable
Behavioral data notes	—
Are control data reported in this paper or another that is referenced?	No
Does the contrast selectively activate plausible relevant language regions in the control group?	Unknown
Are activations lateralized in the control data?	Unknown
Control activation notes	—
Contrast notes	—

Contrast 2: picture naming (untrained items) vs rest

Language condition	Picture naming (untrained items)
Control condition	Rest
Are the conditions matched for visual demands?	No
Are the conditions matched for auditory demands?	No

Are the conditions matched for motor demands?	No
Are the conditions matched for cognitive/executive demands?	No
Is accuracy matched between the language and control tasks for all relevant groups?	N/A, tasks not comparable
Is reaction time matched between the language and control tasks for all relevant groups?	N/A, tasks not comparable
Behavioral data notes	—
Are control data reported in this paper or another that is referenced?	No
Does the contrast selectively activate plausible relevant language regions in the control group?	Unknown
Are activations lateralized in the control data?	Unknown
Control activation notes	—
Contrast notes	—

Analyses

Are the analyses clearly described?	No* (moderate limitation) (see specific limitation(s) below)

ROI analysis 1

First level contrast	Picture naming (trained items) vs rest
Analysis class	Longitudinal correlation with language or other measure
Group(s)	Aphasia T2 vs T1
Covariate	Δ picture naming (trained items)
Is the second level contrast valid in terms of the group(s), time point(s), and measures involved?	Yes
Is accuracy matched across the second level contrast?	Accuracy is covariate
Is reaction time matched across the second level contrast?	Unknown, not reported
Behavioral data notes	Picture naming score (trained items) increased from 51.7 ± 24.8 to 78.8 ± 22.1, which was statistically significant ($p < 0.0001$)
Type of analysis	Regions of interest (ROI)
ROI type	Other
How many ROIs are there?	4
What are the ROI(s)?	(1) perilesional area of slow wave activity determined with MEG; (2) right hemisphere homotopic to lesion; (3) right hemisphere homotopic to slow wave area; (4) remainder of left hemisphere; for one patient, maximal slow wave activity was in the right hemisphere and it is not clear how this was handled
How are the ROI(s) defined?	The dependent measure was the number of voxels in each ROI exceeding certain thresholds that differed across subjects depending on their strength of activation; it appears that increases and decreases may have been summed, though the description is hard to follow
Correction for multiple comparisons	No correction
Statistical details	2 of the 11 patients were classified as outliers and excluded from analyses, however no plots are provided to justify their status as outliers
Findings	Other
Findings notes	Improved picture naming of trained items was correlated with increased signal in 3 of the 4 ROIs, the exception being the right hemisphere ROI homotopic to the slow wave area; after removing the two outliers, only the correlation in the left hemisphere area of slow wave activity remained significant

ROI analysis 2

First level contrast	Picture naming (untrained items) vs rest
Analysis class	Longitudinal correlation with language or other measure
Group(s)	Aphasia T2 vs T1
Covariate	Δ picture naming (untrained items)

Is the second level contrast valid in terms of the group(s), time point(s), and measures involved?	Yes
Is accuracy matched across the second level contrast?	Accuracy is covariate
Is reaction time matched across the second level contrast?	Unknown, not reported
Behavioral data notes	Picture naming score (untrained items) increased from 54.0 ± 24.3 to 70.5 ± 26.7, which was statistically significant (p= 0.002)
Type of analysis	Regions of interest (ROI)
ROI type	Other
How many ROIs are there?	4
What are the ROI(s)?	(1) perilesional area of slow wave activity determined with MEG; (2) right hemisphere homotopic to lesion; (3) right hemisphere homotopic to slow wave area; (4) remainder of left hemisphere; for one patient, maximal slow wave activity was in the right hemisphere and it is not clear how this was handled
How are the ROI(s) defined?	The dependent measure was the number of voxels in each ROI exceeding certain thresholds that differed across subjects depending on their strength of activation; it appears that increases and decreases may have been summed, though the description is hard to follow
Correction for multiple comparisons	No correction
Statistical details	2 of the 11 patients were classified as outliers and excluded from analyses, however no plots are provided to justify their status as outliers
Findings	Other
Findings notes	Improved picture naming of untrained items was correlated with increased signal in all 4 ROIs; after removing the two outliers, none of the correlations remained significant

Notes

Excluded analyses	Additional analyses correlating functional changes in the "delta ROI" with ROI extent, initial severity, duration of aphasia, overall speech activity, since limited detail is provided and only one ROI is reported

Raboyeau et al. (2008)

Reference

Authors	Raboyeau G, De Boissezon X, Marie N, Balduyck S, Puel M, Bézy C, Démonet JF, Cardebat D
Title	Right hemisphere activation in recovery from aphasia: lesion effect or function recruitment?
Reference	*Neurology* 2008; 70: 2900-298
PMID	18209203
DOI	10.1212/01.wnl.0000287115.85956.87

Participants

Language	French
Inclusion criteria	Naming deficit; good comprehension
Number of individuals with aphasia	10
Number of control participants	20
Were any of the participants included in any previous studies?	No
Is age reported for patients and controls, and matched?	No (mean 53.8 ± 14.7 years; controls were younger)
Is sex reported for patients and controls, and matched?	Yes (males: 6; females: 4)
Is handedness reported for patients and controls, and matched?	Yes (right: 10; left: 0)
Is time post stroke onset reported and appropriate to the study design?	Yes (range 7-102 months)

To what extent is the nature of aphasia characterized?	Severity and type
Language evaluation	Montreal-Toulouse Aphasia Battery
Aphasia severity	Mild (but had initially been severe)
Aphasia type	4 anomic, 3 conduction, 2 Broca's, 1 AoS
First stroke only?	Yes
Stroke type	Not stated
To what extent is the lesion distribution characterized?	Individual lesions
Lesion extent	Range 29.9-195.2 cc
Lesion location	L MCA
Participants notes	—

Imaging

Modality	PET (rCBF)
Is the study cross-sectional or longitudinal?	Longitudinal—chronic treatment
If longitudinal, at what time point(s) were imaging data acquired?	T1: pre-treatment/chronic; T2: post-treatment, ~4 weeks later
If longitudinal, was there any intervention between the time points?	Lexical training, 15 minutes/day, 5 days/week, 4 weeks; the control group were trained to relearn foreign words that they had learned in school but since mostly forgotten
Is the scanner described?	Yes (Siemens ECAT HR+)
Is the timing of stimulus presentation and image acquisition clearly described and appropriate?	Yes
Design type	PET
Total images acquired	6
Are the imaging acquisition parameters, including coverage, adequately described and appropriate?	Yes (whole brain)
Is preprocessing and intrasubject coregistration adequately described and appropriate?	Yes
Is first level model fitting adequately described and appropriate?	Yes
Is intersubject normalization adequately described and appropriate?	No (lesion impact not addressed)
Imaging notes	—

Conditions

Are the conditions clearly described?	Yes

Condition	Response type	Repetitions	All groups could do?	All individuals could do?
picture naming (native language)	Word (overt)	aphasia: 4; control: 2	Yes	Unknown
picture naming (relearned foreign language) (controls only)	Word (overt)	2	Yes	Unknown
rest	None	2	N/A	N/A

Conditions notes	Picture naming in native language in controls not analyzed in this paper

Contrasts

Are the contrasts clearly described?	No (see specific limitation(s) below)

Contrast 1: picture naming (native in patients; relearned foreign in controls) vs rest

Language condition	Picture naming (native in patients; relearned foreign in controls)
Control condition	Rest
Are the conditions matched for visual demands?	No
Are the conditions matched for auditory demands?	No
Are the conditions matched for motor demands?	No

Are the conditions matched for cognitive/executive demands?	No
Is accuracy matched between the language and control tasks for all relevant groups?	N/A, tasks not comparable
Is reaction time matched between the language and control tasks for all relevant groups?	N/A, tasks not comparable
Behavioral data notes	—
Are control data reported in this paper or another that is referenced?	No
Does the contrast selectively activate plausible relevant language regions in the control group?	Unknown
Are activations lateralized in the control data?	Unknown
Control activation notes	—
Contrast notes	Presumably only the relearned foreign condition was used in controls (not the native condition), but this is not stated explicitly

Analyses

Are the analyses clearly described?	No (see specific limitation(s) below)

Voxelwise analysis 1

First level contrast	Picture naming (native in patients; relearned foreign in controls) vs rest
Analysis class	Longitudinal aphasia vs control
Group(s)	(Aphasia T2 vs T1) vs (control T2 vs T1)
Covariate	—
Is the second level contrast valid in terms of the group(s), time point(s), and measures involved?	Yes
Is accuracy matched across the second level contrast?	No, but attempt made
Is reaction time matched across the second level contrast?	Unknown, not reported
Behavioral data notes	Relearned foreign language was an attempt to equate to recovery in patients; still, patients improved less than controls, as shown by a significant interaction of group by time ($p < .0001$)
Type of analysis	Voxelwise
Search volume	Whole brain
Correction for multiple comparisons	Clusterwise correction based on arbitrary cluster extent
Software	SPM2
Voxelwise p	.01
Cluster extent	30 voxels (size not stated)
Statistical details	Nature of control contrast not clear; negative tail of contrast was masked to exclude lesioned areas, but the mask may have been more extensive than that
Findings	↑ L orbitofrontal
Findings notes	—

Voxelwise analysis 2

First level contrast	Picture naming (native in patients; relearned foreign in controls) vs rest
Analysis class	Longitudinal correlation with language or other measure
Group(s)	Aphasia T2 vs T1
Covariate	Δ picture naming accuracy
Is the second level contrast valid in terms of the group(s), time point(s), and measures involved?	Yes
Is accuracy matched across the second level contrast?	Accuracy is covariate
Is reaction time matched across the second level contrast?	Unknown, not reported
Behavioral data notes	—
Type of analysis	Voxelwise

Search volume	Whole brain
Correction for multiple comparisons	Clusterwise correction based on arbitrary cluster extent
Software	SPM2
Voxelwise p	.01
Cluster extent	30 voxels (size not stated)
Statistical details	Nature of control contrast not clear
Findings	↑ R insula<br>↑ R SMA/medial prefrontal<br>↑ R orbitofrontal<br>↑ R anterior cingulate<br>↓ L intraparietal sulcus<br>↓ L precuneus<br>↓ L posterior cingulate<br>↓ R dorsal precentral<br>↓ R precuneus
Findings notes	—

Notes

Excluded analyses	Conjunction analysis, because it collapsed across patients and controls

Richter et al. (2008)

Reference

Authors	Richter M, Miltner WH, Straube T
Title	Association between therapy outcome and right-hemispheric activation in chronic aphasia
Reference	*Brain* 2008; 131: 1391-1401
PMID	18349055
DOI	10.1093/brain/awn043

Participants

Language	German
Inclusion criteria	Main deficits in production rather than comprehension
Number of individuals with aphasia	16 (plus 8 excluded: 5 completed only one of the two sessions; 3 unable to perform the tasks)
Number of control participants	8
Were any of the participants included in any previous studies?	No
Is age reported for patients and controls, and matched?	Yes (mean 58.3 years; range 42-73 years)
Is sex reported for patients and controls, and matched?	Yes (males: 12; females: 4)
Is handedness reported for patients and controls, and matched?	Yes (right: 16; left: 0)
Is time post stroke onset reported and appropriate to the study design?	No (> 12 months; actual TPO not stated)
To what extent is the nature of aphasia characterized?	Comprehensive battery
Language evaluation	AAT, two subtests of ANELT
Aphasia severity	TT range 5-50
Aphasia type	7 anomic, 7 Broca's, 2 global; it was an inclusion criterion that the main deficits were in production
First stroke only?	Not stated
Stroke type	Not stated
To what extent is the lesion distribution characterized?	Individual lesions

Lesion extent	Not stated
Lesion location	L
Participants notes	—

Imaging

Modality	fMRI
Is the study cross-sectional or longitudinal?	Longitudinal—chronic treatment
If longitudinal, at what time point(s) were imaging data acquired?	T1: pre-treatment/chronic; T2: post-treatment, ~2 weeks later
If longitudinal, was there any intervention between the time points?	CIAT, 3 hours/day, 10 days
Is the scanner described?	Yes (Siemens Vision plus 1.5 Tesla)
Is the timing of stimulus presentation and image acquisition clearly described and appropriate?	No (minor discrepancies in description of timing)
Design type	Block
Total images acquired	134
Are the imaging acquisition parameters, including coverage, adequately described and appropriate?	Yes (whole brain)
Is preprocessing and intrasubject coregistration adequately described and appropriate?	Yes
Is first level model fitting adequately described and appropriate?	Yes
Is intersubject normalization adequately described and appropriate?	No (lesion impact not addressed)
Imaging notes	—

Conditions

Are the conditions clearly described?	Yes

Condition	Response type	Repetitions	All groups could do?	All individuals could do?
reading words silently	Word (covert)	4	Yes	Unknown
word stem completion	Word (covert)	4	Yes	Unknown
rest	None	10 (?)	N/A	N/A

Conditions notes	Preliminary data on the tasks suggests that patients would have been able to perform them, and patients were interviewed regarding the tasks after each fMRI session, however the outcomes of these interviews are not reported

Contrasts

Are the contrasts clearly described?	Yes

Contrast 1: reading words silently vs rest

Language condition	Reading words silently
Control condition	Rest
Are the conditions matched for visual demands?	No
Are the conditions matched for auditory demands?	Yes
Are the conditions matched for motor demands?	Yes
Are the conditions matched for cognitive/executive demands?	No
Is accuracy matched between the language and control tasks for all relevant groups?	N/A, tasks not comparable
Is reaction time matched between the language and control tasks for all relevant groups?	N/A, tasks not comparable
Behavioral data notes	—
Are control data reported in this paper or another that is referenced?	Somewhat

Does the contrast selectively activate plausible relevant language regions in the control group?	Unknown
Are activations lateralized in the control data?	Unknown
Control activation notes	Appears to be somewhat L-lateralized frontal, but not well visualized
Contrast notes	—

Contrast 2: word stem completion vs rest

Language condition	Word stem completion
Control condition	Rest
Are the conditions matched for visual demands?	No
Are the conditions matched for auditory demands?	Yes
Are the conditions matched for motor demands?	Yes
Are the conditions matched for cognitive/executive demands?	No
Is accuracy matched between the language and control tasks for all relevant groups?	N/A, tasks not comparable
Is reaction time matched between the language and control tasks for all relevant groups?	N/A, tasks not comparable
Behavioral data notes	—
Are control data reported in this paper or another that is referenced?	Somewhat
Does the contrast selectively activate plausible relevant language regions in the control group?	Unknown
Are activations lateralized in the control data?	No
Control activation notes	Bilateral frontal; other regions not well visualized
Contrast notes	—

Analyses

Are the analyses clearly described?	No* (moderate limitation) (see specific limitation(s) below)

Voxelwise analysis 1

First level contrast	Reading words silently vs rest
Analysis class	Cross-sectional aphasia vs control
Group(s)	Aphasia T1 vs control
Covariate	—
Is the second level contrast valid in terms of the group(s), time point(s), and measures involved?	Yes
Is accuracy matched across the second level contrast?	Unknown, not reported
Is reaction time matched across the second level contrast?	Unknown, not reported
Behavioral data notes	—
Type of analysis	Voxelwise
Search volume	R hemisphere
Correction for multiple comparisons	Mixed** (major limitation)
Software	BrainVoyager QX 1.7
Voxelwise p	R IFG/R insula ROI: .005; elsewhere: .001
Cluster extent	R IFG/R insula ROI: 0.108 cc; elsewhere: none
Statistical details	—
Findings	↑ R IFG<br>↑ R insula
Findings notes	—

Voxelwise analysis 2

First level contrast	Word stem completion vs rest

Analysis class	Cross-sectional aphasia vs control
Group(s)	Aphasia T1 vs control
Covariate	—
Is the second level contrast valid in terms of the group(s), time point(s), and measures involved?	Yes
Is accuracy matched across the second level contrast?	Unknown, not reported
Is reaction time matched across the second level contrast?	Unknown, not reported
Behavioral data notes	—
Type of analysis	Voxelwise
Search volume	R hemisphere
Correction for multiple comparisons	Mixed** (major limitation)
Software	BrainVoyager QX 1.7
Voxelwise p	R IFG/R insula ROI: .005; elsewhere: .001
Cluster extent	R IFG/R insula ROI: 0.108 cc; elsewhere: none
Statistical details	—
Findings	↑ R dorsal precentral
Findings notes	—

Voxelwise analysis 3

First level contrast	Reading words silently vs rest
Analysis class	Cross-sectional correlation with language or other measure
Group(s)	Aphasia T1
Covariate	Subsequent Δ (T2 vs T1) overall language measure (composite measure of AAT spontaneous speech, token test, ANELT auditory comprehensibility, ANELT semantic comprehensibility)
Is the second level contrast valid in terms of the group(s), time point(s), and measures involved?	Somewhat (T1 behavioral measure should be included in model)
Is accuracy matched across the second level contrast?	Unknown, not reported
Is reaction time matched across the second level contrast?	Unknown, not reported
Behavioral data notes	—
Type of analysis	Voxelwise
Search volume	R hemisphere
Correction for multiple comparisons	No correction
Software	BrainVoyager QX 1.7
Voxelwise p	.05
Cluster extent	None
Statistical details	Nature of thresholding not entirely clear, so coded according to best guess
Findings	↑ R IFG<br>↑ R insula<br>↑ R ventral precentral/inferior frontal junction<br>↑ R posterior MTG
Findings notes	Increased activity correlated with more behavioral improvement

Voxelwise analysis 4

First level contrast	Word stem completion vs rest
Analysis class	Cross-sectional correlation with language or other measure
Group(s)	Aphasia T1
Covariate	Subsequent Δ (T2 vs T1) overall language measure (composite measure of AAT spontaneous speech, token test, ANELT auditory comprehensibility, ANELT semantic comprehensibility)
Is the second level contrast valid in terms of the group(s), time point(s), and measures involved?	Somewhat (T1 behavioral measure should be included in model)
Is accuracy matched across the second level contrast?	Unknown, not reported

Is reaction time matched across the second level contrast?	Unknown, not reported
Behavioral data notes	—
Type of analysis	Voxelwise
Search volume	R hemisphere
Correction for multiple comparisons	No correction
Software	BrainVoyager QX 1.7
Voxelwise p	.05
Cluster extent	None
Statistical details	Nature of thresholding not entirely clear, so coded according to best guess
Findings	↑ R IFG<br>↑ R insula
Findings notes	Increased activity correlated with more behavioral improvement

Voxelwise analysis 5

First level contrast	Reading words silently vs rest
Analysis class	Longitudinal change in aphasia
Group(s)	Aphasia T2 vs T1
Covariate	—
Is the second level contrast valid in terms of the group(s), time point(s), and measures involved?	Yes
Is accuracy matched across the second level contrast?	Unknown, not reported
Is reaction time matched across the second level contrast?	Unknown, not reported
Behavioral data notes	—
Type of analysis	Voxelwise
Search volume	R hemisphere
Correction for multiple comparisons	Mixed** (major limitation)
Software	BrainVoyager QX 1.7
Voxelwise p	R IFG/R insula ROI: .005; elsewhere: .001
Cluster extent	R IFG/R insula ROI: 0.108 cc; elsewhere: none
Statistical details	—
Findings	None
Findings notes	—

Voxelwise analysis 6

First level contrast	Word stem completion vs rest
Analysis class	Longitudinal change in aphasia
Group(s)	Aphasia T2 vs T1
Covariate	—
Is the second level contrast valid in terms of the group(s), time point(s), and measures involved?	Yes
Is accuracy matched across the second level contrast?	Unknown, not reported
Is reaction time matched across the second level contrast?	Unknown, not reported
Behavioral data notes	—
Type of analysis	Voxelwise
Search volume	R hemisphere
Correction for multiple comparisons	Mixed** (major limitation)
Software	BrainVoyager QX 1.7
Voxelwise p	R IFG/R insula ROI: .005; elsewhere: .001
Cluster extent	R IFG/R insula ROI: 0.108 cc; elsewhere: none
Statistical details	—

Findings	None
Findings notes	—

ROI analysis 1

First level contrast	Reading words silently vs rest
Analysis class	Cross-sectional correlation with language or other measure
Group(s)	Aphasia T1
Covariate	Subsequent Δ (T2 vs T1) overall language measure (composite measure of AAT spontaneous speech, token test, ANELT auditory comprehensibility, ANELT semantic comprehensibility)
Is the second level contrast valid in terms of the group(s), time point(s), and measures involved?	Somewhat (T1 behavioral measure should be included in model)
Is accuracy matched across the second level contrast?	Unknown, not reported
Is reaction time matched across the second level contrast?	Unknown, not reported
Behavioral data notes	—
Type of analysis	Region of interest (ROI)
ROI type	Functional
How many ROIs are there?	1
What are the ROI(s)?	L IFG/insula or L perilesional
How are the ROI(s) defined?	Peak activations in individual patients in L IFG/insula or L perilesional regions (somewhat unclear)
Correction for multiple comparisons	One only
Statistical details	—
Findings	None
Findings notes	—

ROI analysis 2

First level contrast	Word stem completion vs rest
Analysis class	Cross-sectional correlation with language or other measure
Group(s)	Aphasia T1
Covariate	Subsequent Δ (T2 vs T1) overall language measure (composite measure of AAT spontaneous speech, token test, ANELT auditory comprehensibility, ANELT semantic comprehensibility)
Is the second level contrast valid in terms of the group(s), time point(s), and measures involved?	Somewhat (T1 behavioral measure should be included in model)
Is accuracy matched across the second level contrast?	Unknown, not reported
Is reaction time matched across the second level contrast?	Unknown, not reported
Behavioral data notes	—
Type of analysis	Region of interest (ROI)
ROI type	Functional
How many ROIs are there?	1
What are the ROI(s)?	L IFG/insula or L perilesional
How are the ROI(s) defined?	Peak activations in individual patients in L IFG/insula or L perilesional regions (somewhat unclear)
Correction for multiple comparisons	One only
Statistical details	—
Findings	None
Findings notes	—

ROI analysis 3

First level contrast	Reading words silently vs rest
Analysis class	Longitudinal correlation with language or other measure
Group(s)	Aphasia T2 vs T1

Covariate	Δ overall language measure (composite measure of AAT spontaneous speech, token test, ANELT auditory comprehensibility, ANELT semantic comprehensibility)
Is the second level contrast valid in terms of the group(s), time point(s), and measures involved?	Yes
Is accuracy matched across the second level contrast?	Unknown, not reported
Is reaction time matched across the second level contrast?	Unknown, not reported
Behavioral data notes	—
Type of analysis	Regions of interest (ROI)
ROI type	Functional
How many ROIs are there?	4
What are the ROI(s)?	(1) R IFG/insula; (2) R precentral; (3) R MTG; (4) L IFG/insula or L perilesional
How are the ROI(s) defined?	Regions where T1 activation was correlated with subsequent improvement, along with the previously defined left hemisphere ROI
Correction for multiple comparisons	No correction
Statistical details	Circular because functional ROIs based on related contrast on same data
Findings	↓ R posterior MTG
Findings notes	Decreased activity over time correlated with more behavioral improvement

ROI analysis 4

First level contrast	Word stem completion vs rest
Analysis class	Longitudinal correlation with language or other measure
Group(s)	Aphasia T2 vs T1
Covariate	Δ overall language measure (composite measure of AAT spontaneous speech, token test, ANELT auditory comprehensibility, ANELT semantic comprehensibility)
Is the second level contrast valid in terms of the group(s), time point(s), and measures involved?	Yes
Is accuracy matched across the second level contrast?	Unknown, not reported
Is reaction time matched across the second level contrast?	Unknown, not reported
Behavioral data notes	—
Type of analysis	Regions of interest (ROI)
ROI type	Functional
How many ROIs are there?	3
What are the ROI(s)?	(1, 2) two clusters within R IFG/insula ROI; (3) L IFG/insula or L perilesional
How are the ROI(s) defined?	Regions where T1 activation was correlated with subsequent improvement, along with the previously defined left hemisphere ROI
Correction for multiple comparisons	No correction
Statistical details	Circular because functional ROIs based on related contrast on same data
Findings	↓ R IFG<br>↓ R insula
Findings notes	Decreased activity over time correlated with more behavioral improvement

Notes

Excluded analyses	—

de Boissezon et al. (2009)

Reference

Authors	de Boissezon X, Marie N, Castel-Lacanal E, Marque P, Bezy C, Gros H, Lotterie JA, Cardebat D, Puel M, Demonet JF

Title	Good recovery from aphasia is also supported by right basal ganglia: a longitudinal controlled PET study
Reference	*Eur J Phys Rehabil Med* 2009; 45: 547-558
PMID	20032914
DOI	N/A

Participants

Language	French
Inclusion criteria	Only part of L MCA; able to perform word generation; no severe aphasia
Number of individuals with aphasia	13
Number of control participants	0
Were any of the participants included in any previous studies?	Yes (7 out of 13 patients appear to represent the same data reported in de Boissezon et al. (2005))
Is age reported for patients and controls, and matched?	Yes (range 31.2-74.2 years)
Is sex reported for patients and controls, and matched?	Yes (males: 12; females: 1)
Is handedness reported for patients and controls, and matched?	Yes (right: 13; left: 0)
Is time post stroke onset reported and appropriate to the study design?	No* (moderate limitation) (T1: mean 64 ± 32 days; T2: mean 11.8 ± 1.4 months; T1 varies considerably from early to late subacute)
To what extent is the nature of aphasia characterized?	Comprehensive battery
Language evaluation	Montreal-Toulouse Aphasia Battery
Aphasia severity	Not stated
Aphasia type	T1: 3 transcortical motor, 2 anomic, 2 Broca's, 2 transcortical sensory, 2 Wernicke's, 1 conduction, 1 agrammatic; T2: not stated
First stroke only?	Yes
Stroke type	Mixed etiologies
To what extent is the lesion distribution characterized?	Lesion overlay
Lesion extent	Range 0.9-43.4 cc
Lesion location	L MCA (7 subcortical, 6 cortical)
Participants notes	—

Imaging

Modality	PET (rCBF)
Is the study cross-sectional or longitudinal?	Longitudinal—recovery
If longitudinal, at what time point(s) were imaging data acquired?	T1: mean 64 ± 32 days; T2: mean 11.8 ± 1.4 months; T1 varies considerably from early to late subacute
If longitudinal, was there any intervention between the time points?	Community SLT; 45 minutes/day, 1-3 days/week
Is the scanner described?	Yes (CTI-Siemens ECAT EXACT HR+)
Is the timing of stimulus presentation and image acquisition clearly described and appropriate?	Yes
Design type	PET
Total images acquired	6
Are the imaging acquisition parameters, including coverage, adequately described and appropriate?	Yes (whole brain)
Is preprocessing and intrasubject coregistration adequately described and appropriate?	Yes
Is first level model fitting adequately described and appropriate?	Yes
Is intersubject normalization adequately described and appropriate?	No (lesion impact not addressed)
Imaging notes	—

Conditions

Are the conditions clearly described?	Yes

Condition	Response type	Repetitions	All groups could do?	All individuals could do?
word generation	Word (overt)	4	Yes	Yes
rest	None	2	N/A	N/A

Conditions notes	—

Contrasts

Are the contrasts clearly described?	Yes

Contrast 1: word generation vs rest

Language condition	Word generation
Control condition	Rest
Are the conditions matched for visual demands?	Yes
Are the conditions matched for auditory demands?	No
Are the conditions matched for motor demands?	No
Are the conditions matched for cognitive/executive demands?	No
Is accuracy matched between the language and control tasks for all relevant groups?	N/A, tasks not comparable
Is reaction time matched between the language and control tasks for all relevant groups?	N/A, tasks not comparable
Behavioral data notes	—
Are control data reported in this paper or another that is referenced?	Somewhat
Does the contrast selectively activate plausible relevant language regions in the control group?	Somewhat
Are activations lateralized in the control data?	No
Control activation notes	Control data in Cardebat et al. (2003); bilateral fronto-temporal and some other regions per text
Contrast notes	—

Analyses

Are the analyses clearly described?	No* (moderate limitation) (see specific limitation(s) below)

Voxelwise analysis 1

First level contrast	Word generation vs rest
Analysis class	Longitudinal change in aphasia
Group(s)	Aphasia with "good recovery" (n = 6) T2 vs T1
Covariate	—
Is the second level contrast valid in terms of the group(s), time point(s), and measures involved?	Somewhat (the "good recovery" group showed more improvement than the "poor recovery" group in terms of accuracy on the task, but the distinction was not borne out in behavioral data more generally)
Is accuracy matched across the second level contrast?	Yes, matched
Is reaction time matched across the second level contrast?	Unknown, not reported
Behavioral data notes	P = 0.07
Type of analysis	Voxelwise
Search volume	Whole brain
Correction for multiple comparisons	Clusterwise correction based on arbitrary cluster extent
Software	SPM2
Voxelwise p	.001

Cluster extent	100 voxels (size not stated)
Statistical details	Contrast may not have included resting condition; inappropriate masking
Findings	↑ L ventral precentral/inferior frontal junction<br>↑ L SMA/medial prefrontal<br>↑ L posterior STG/STS/MTG<br>↑ R dorsolateral prefrontal cortex<br>↑ R SMA/medial prefrontal<br>↑ R angular gyrus<br>↑ R occipital<br>↑ R thalamus<br>↑ R basal ganglia<br>↓ L cerebellum
Findings notes	Based on coordinates in Table 5

Voxelwise analysis 2

First level contrast	Word generation vs rest
Analysis class	Longitudinal change in aphasia
Group(s)	Aphasia with "poor recovery" (n = 7) T2 vs T1
Covariate	—
Is the second level contrast valid in terms of the group(s), time point(s), and measures involved?	Somewhat (the "poor recovery" group showed less improvement than the "good recovery" group in terms of accuracy on the task, but the distinction was not borne out in behavioral data more generally)
Is accuracy matched across the second level contrast?	Yes, matched
Is reaction time matched across the second level contrast?	Unknown, not reported
Behavioral data notes	—
Type of analysis	Voxelwise
Search volume	Whole brain
Correction for multiple comparisons	Clusterwise correction based on arbitrary cluster extent
Software	SPM2
Voxelwise p	.001
Cluster extent	100 voxels (size not stated)
Statistical details	Contrast may not have included resting condition; inappropriate masking
Findings	↑ L ventral precentral/inferior frontal junction<br>↑ R somato-motor<br>↑ R cerebellum<br>↓ R basal ganglia
Findings notes	—

Voxelwise analysis 3

First level contrast	Word generation vs rest
Analysis class	Cross-sectional correlation with language or other measure
Group(s)	Aphasia
Covariate	Word generation accuracy
Is the second level contrast valid in terms of the group(s), time point(s), and measures involved?	Yes
Is accuracy matched across the second level contrast?	Accuracy is covariate
Is reaction time matched across the second level contrast?	Unknown, not reported
Behavioral data notes	—
Type of analysis	Voxelwise
Search volume	Whole brain
Correction for multiple comparisons	Clusterwise correction based on arbitrary cluster extent
Software	SPM2
Voxelwise p	.01

Cluster extent	100 voxels (size not stated)
Statistical details	Each patient's two sessions may be entered into the model without accounting for the dependence between them
Findings	↑ L supramarginal gyrus<br>↑ L occipital<br>↑ L anterior cingulate<br>↑ R insula<br>↑ R SMA/medial prefrontal<br>↑ R posterior STG<br>↑ R anterior temporal<br>↑ R occipital<br>↓ L cerebellum
Findings notes	—

Notes

Excluded analyses	—

Fridriksson et al. (2009)

Reference

Authors	Fridriksson J, Baker JM, Moser D
Title	Cortical mapping of naming errors in aphasia
Reference	*Hum Brain Mapp* 2009; 30: 2487-2498
PMID	19294641
DOI	10.1002/hbm.20683

Participants

Language	US English
Inclusion criteria	—
Number of individuals with aphasia	11
Number of control participants	10
Were any of the participants included in any previous studies?	No
Is age reported for patients and controls, and matched?	Yes (mean 58.8 ± 14.7 years, range 33-78 years)
Is sex reported for patients and controls, and matched?	Yes (males: 6; females: 5)
Is handedness reported for patients and controls, and matched?	No
Is time post stroke onset reported and appropriate to the study design?	Yes (range 10-101 months)
To what extent is the nature of aphasia characterized?	Comprehensive battery
Language evaluation	WAB; BNT
Aphasia severity	AQ range 31.8-91.5
Aphasia type	6 anomic, 4 Broca's, 1 transcortical motor; alternatively: 6 fluent, 5 non-fluent
First stroke only?	Not stated
Stroke type	Not stated
To what extent is the lesion distribution characterized?	Lesion overlay
Lesion extent	Range 3.0-342.2 cc
Lesion location	L MCA
Participants notes	—

Imaging

Modality	fMRI
Is the study cross-sectional or longitudinal?	Cross-sectional
If longitudinal, at what time point(s) were imaging data acquired?	—
If longitudinal, was there any intervention between the time points?	—
Is the scanner described?	No (not stated)
Is the timing of stimulus presentation and image acquisition clearly described and appropriate?	No (timing of picture presentation not clearly explained)
Design type	Event-related
Total images acquired	120
Are the imaging acquisition parameters, including coverage, adequately described and appropriate?	Yes (whole brain)
Is preprocessing and intrasubject coregistration adequately described and appropriate?	Yes
Is first level model fitting adequately described and appropriate?	Yes
Is intersubject normalization adequately described and appropriate?	Yes
Imaging notes	sparse sampling

Conditions

Are the conditions clearly described?	Yes

Condition	Response type	Repetitions	All groups could do?	All individuals could do?
picture naming	Word (overt)	80	Yes	No
viewing scrambled images	None	40	N/A	N/A

Conditions notes	—

Contrasts

Are the contrasts clearly described?	Yes

Contrast 1: picture naming (correct trials) vs viewing scrambled images

Language condition	Picture naming (correct trials)
Control condition	Viewing scrambled images
Are the conditions matched for visual demands?	Yes
Are the conditions matched for auditory demands?	No
Are the conditions matched for motor demands?	No
Are the conditions matched for cognitive/executive demands?	No
Is accuracy matched between the language and control tasks for all relevant groups?	N/A, tasks not comparable
Is reaction time matched between the language and control tasks for all relevant groups?	N/A, tasks not comparable
Behavioral data notes	—
Are control data reported in this paper or another that is referenced?	Somewhat
Does the contrast selectively activate plausible relevant language regions in the control group?	No
Are activations lateralized in the control data?	Somewhat
Control activation notes	Control data in Fridriksson et al. (2007); motor activations are prominent; there is some L frontal activation but little temporal activation in either hemisphere
Contrast notes	—

Contrast 2: picture naming (phonemic paraphasias) vs picture naming (correct trials)

Language condition	Picture naming (phonemic paraphasias)
Control condition	Picture naming (correct trials)
Are the conditions matched for visual demands?	Yes
Are the conditions matched for auditory demands?	Yes
Are the conditions matched for motor demands?	Yes
Are the conditions matched for cognitive/executive demands?	Yes
Is accuracy matched between the language and control tasks for all relevant groups?	No, by design
Is reaction time matched between the language and control tasks for all relevant groups?	Unknown, not reported
Behavioral data notes	—
Are control data reported in this paper or another that is referenced?	N/A
Does the contrast selectively activate plausible relevant language regions in the control group?	N/A
Are activations lateralized in the control data?	N/A
Control activation notes	Control data N/A because controls do not typically make errors
Contrast notes	—

Contrast 3: picture naming (semantic paraphasias) vs picture naming (correct trials)

Language condition	Picture naming (semantic paraphasias)
Control condition	Picture naming (correct trials)
Are the conditions matched for visual demands?	Yes
Are the conditions matched for auditory demands?	Yes
Are the conditions matched for motor demands?	Yes
Are the conditions matched for cognitive/executive demands?	Yes
Is accuracy matched between the language and control tasks for all relevant groups?	No, by design
Is reaction time matched between the language and control tasks for all relevant groups?	Unknown, not reported
Behavioral data notes	—
Are control data reported in this paper or another that is referenced?	N/A
Does the contrast selectively activate plausible relevant language regions in the control group?	N/A
Are activations lateralized in the control data?	N/A
Control activation notes	Control data N/A because controls do not typically make errors
Contrast notes	—

Analyses

Are the analyses clearly described?	Yes

Voxelwise analysis 1

First level contrast	Picture naming (correct trials) vs viewing scrambled images
Analysis class	Cross-sectional aphasia vs control
Group(s)	Aphasia vs control
Covariate	—
Is the second level contrast valid in terms of the group(s), time point(s), and measures involved?	Yes
Is accuracy matched across the second level contrast?	Yes, correct trials only
Is reaction time matched across the second level contrast?	Unknown, not reported

Behavioral data notes	—
Type of analysis	Voxelwise
Search volume	Voxels spared in all patients
Correction for multiple comparisons	Clusterwise correction with with GRFT and lenient voxelwise p
Software	FSL (FEAT 5.4)
Voxelwise p	~.01 (z > 2.3)
Cluster extent	Based on GRFT
Statistical details	—
Findings	None
Findings notes	—

Voxelwise analysis 2

First level contrast	Picture naming (phonemic paraphasias) vs picture naming (correct trials)
Analysis class	Cross-sectional performance-defined conditions
Group(s)	Aphasia
Covariate	—
Is the second level contrast valid in terms of the group(s), time point(s), and measures involved?	Yes
Is accuracy matched across the second level contrast?	No, by design
Is reaction time matched across the second level contrast?	Unknown, not reported
Behavioral data notes	—
Type of analysis	Voxelwise
Search volume	Voxels spared in all patients
Correction for multiple comparisons	Clusterwise correction with with GRFT and lenient voxelwise p
Software	FSL (FEAT 5.4)
Voxelwise p	~.01 (z > 2.3)
Cluster extent	Based on GRFT
Statistical details	—
Findings	↑ L superior parietal<br>↑ L posterior inferior temporal gyrus/fusiform gyrus<br>↑ L occipital
Findings notes	—

Voxelwise analysis 3

First level contrast	Picture naming (semantic paraphasias) vs picture naming (correct trials)
Analysis class	Cross-sectional performance-defined conditions
Group(s)	Aphasia
Covariate	—
Is the second level contrast valid in terms of the group(s), time point(s), and measures involved?	Yes
Is accuracy matched across the second level contrast?	No, by design
Is reaction time matched across the second level contrast?	Unknown, not reported
Behavioral data notes	—
Type of analysis	Voxelwise
Search volume	Voxels spared in all patients
Correction for multiple comparisons	Clusterwise correction with with GRFT and lenient voxelwise p
Software	FSL (FEAT 5.4)
Voxelwise p	~.01 (z > 2.3)
Cluster extent	Based on GRFT
Statistical details	—
Findings	↑ R posterior inferior temporal gyrus/fusiform gyrus

	↑ R occipital
Findings notes	—

ROI analysis 1

First level contrast	Picture naming (correct trials) vs viewing scrambled images
Analysis class	Cross-sectional correlation with language or other measure
Group(s)	Aphasia
Covariate	Picture naming accuracy
Is the second level contrast valid in terms of the group(s), time point(s), and measures involved?	Yes
Is accuracy matched across the second level contrast?	Yes, correct trials only
Is reaction time matched across the second level contrast?	Unknown, not reported
Behavioral data notes	—
Type of analysis	Regions of interest (ROI)
ROI type	Functional
How many ROIs are there?	5
What are the ROI(s)?	(1) R IFG/insula; (2) R motor/premotor; (3) R SMA; (4) R inferior parietal; (5) R superior temporal
How are the ROI(s) defined?	Regions activated for picture naming vs viewing scrambled images in aphasia
Correction for multiple comparisons	No correction
Statistical details	—
Findings	↑ R IFG<br>↑ R insula
Findings notes	R IFG showed more activation in patients who produced more correct responses

Notes

Excluded analyses	—

Menke et al. (2009)

Reference

Authors	Menke R, Meinzer M, Kugel H, Deppe M, Baumgärtner A, Schiffbauer H, Thomas M, Kramer K, Lohmann H, Flöel A, Knecht S, Breitenstein C
Title	Imaging short- and long-term training success in chronic aphasia
Reference	*BMC Neurosci* 2009; 10: 118
PMID	19772660
DOI	10.1186/1471-2202-10-118

Participants

Language	German
Inclusion criteria	Moderate to severe anomia
Number of individuals with aphasia	8
Number of control participants	9
Were any of the participants included in any previous studies?	No
Is age reported for patients and controls, and matched?	Yes (range 34-67 years)
Is sex reported for patients and controls, and matched?	Yes (males: 5; females: 3)
Is handedness reported for patients and controls, and matched?	Yes (right: 8; left: 0)
Is time post stroke onset reported and appropriate	Yes (range 1.8-6.9 years)

to the study design?	
To what extent is the nature of aphasia characterized?	Comprehensive battery
Language evaluation	AAT
Aphasia severity	6 moderate-severe, 2 severe
Aphasia type	7 Broca's, 1 global
First stroke only?	Yes
Stroke type	Mixed etiologies
To what extent is the lesion distribution characterized?	Individual lesions
Lesion extent	Not stated
Lesion location	L
Participants notes	—

Imaging

Modality	fMRI
Is the study cross-sectional or longitudinal?	Longitudinal—chronic treatment
If longitudinal, at what time point(s) were imaging data acquired?	T1: pre-treatment/chronic; T2: post-treatment, ~2 weeks later; T3: 8 months after the end of treatment
If longitudinal, was there any intervention between the time points?	Intensive anomia training; 3 hours/day; 2 weeks
Is the scanner described?	Yes (Philips Intera 3 Tesla)
Is the timing of stimulus presentation and image acquisition clearly described and appropriate?	No (total images acquired not stated)
Design type	Event-related
Total images acquired	probably ~360, but not stated
Are the imaging acquisition parameters, including coverage, adequately described and appropriate?	Yes (whole brain)
Is preprocessing and intrasubject coregistration adequately described and appropriate?	Yes
Is first level model fitting adequately described and appropriate?	Yes
Is intersubject normalization adequately described and appropriate?	Yes
Imaging notes	—

Conditions

Are the conditions clearly described?	Yes

Condition	Response type	Repetitions	All groups could do?	All individuals could do?
picture naming (trained items)	Word (overt)	30	No	No
picture naming (untrained items)	Word (overt)	30	No	No
picture naming (already known items)	Word (overt)	30	Yes	Unknown
rest	None	implicit baseline	N/A	N/A

Conditions notes	Patients could not name trained and untrained items at baseline

Contrasts

Are the contrasts clearly described?	Yes

Contrast 1: picture naming (trained items) vs rest

Language condition	Picture naming (trained items)
Control condition	Rest
Are the conditions matched for visual demands?	No

Are the conditions matched for auditory demands?	No
Are the conditions matched for motor demands?	No
Are the conditions matched for cognitive/executive demands?	No
Is accuracy matched between the language and control tasks for all relevant groups?	N/A, tasks not comparable
Is reaction time matched between the language and control tasks for all relevant groups?	N/A, tasks not comparable
Behavioral data notes	—
Are control data reported in this paper or another that is referenced?	Somewhat
Does the contrast selectively activate plausible relevant language regions in the control group?	Unknown
Are activations lateralized in the control data?	Unknown
Control activation notes	Table of coordinates only
Contrast notes	—

Contrast 2: picture naming (untrained items) vs rest

Language condition	Picture naming (untrained items)
Control condition	Rest
Are the conditions matched for visual demands?	No
Are the conditions matched for auditory demands?	No
Are the conditions matched for motor demands?	No
Are the conditions matched for cognitive/executive demands?	No
Is accuracy matched between the language and control tasks for all relevant groups?	N/A, tasks not comparable
Is reaction time matched between the language and control tasks for all relevant groups?	N/A, tasks not comparable
Behavioral data notes	—
Are control data reported in this paper or another that is referenced?	Somewhat
Does the contrast selectively activate plausible relevant language regions in the control group?	Unknown
Are activations lateralized in the control data?	Unknown
Control activation notes	Table of coordinates only
Contrast notes	—

Analyses

Are the analyses clearly described?	No* (moderate limitation) (see specific limitation(s) below)

Voxelwise analysis 1

First level contrast	Picture naming (trained items) vs rest
Analysis class	Longitudinal correlation with language or other measure
Group(s)	Aphasia T2 vs T1
Covariate	Subsequent outcome (T2) picture naming of trained items outside the scanner
Is the second level contrast valid in terms of the group(s), time point(s), and measures involved?	No (the logic behind correlating activation changes and language outcome is unclear)
Is accuracy matched across the second level contrast?	Unknown, no test
Is reaction time matched across the second level contrast?	Unknown, not reported
Behavioral data notes	—
Type of analysis	Voxelwise
Search volume	Whole brain
Correction for multiple comparisons	Mixed** (major limitation)

Software	SPM2
Voxelwise p	.05, but at least one voxel in the cluster had to be p < .001
Cluster extent	0.270 cc
Statistical details	There was an exclusive mask based on activation changes for untrained pictures, but it is unclear what the behavioral covariate was for the mask generation, nor were the regions in the mask reported
Findings	↑ L occipital<br>↑ L hippocampus/MTL<br>↑ R precuneus<br>↑ R occipital<br>↑ R posterior cingulate<br>↑ R hippocampus/MTL
Findings notes	—

Voxelwise analysis 2

First level contrast	Picture naming (untrained items) vs rest
Analysis class	Longitudinal correlation with language or other measure
Group(s)	Aphasia T3 vs T1
Covariate	Subsequent outcome (T3) picture naming of trained items outside the scanner
Is the second level contrast valid in terms of the group(s), time point(s), and measures involved?	No (the logic behind correlating activation changes and language outcome is unclear)
Is accuracy matched across the second level contrast?	Unknown, no test
Is reaction time matched across the second level contrast?	Unknown, not reported
Behavioral data notes	—
Type of analysis	Voxelwise
Search volume	Whole brain
Correction for multiple comparisons	Mixed** (major limitation)
Software	SPM2
Voxelwise p	.05, but at least one voxel in the cluster had to be p < .001
Cluster extent	0.270 cc
Statistical details	There was an exclusive mask based on activation changes for untrained pictures, but it is unclear what the behavioral covariate was for the mask generation, nor were the regions in the mask reported
Findings	↑ R posterior STG/STS/MTG<br>↓ L SMA/medial prefrontal<br>↓ R inferior parietal lobule<br>↓ R posterior inferior temporal gyrus/fusiform gyrus<br>↓ R basal ganglia
Findings notes	—

Notes

Excluded analyses	—

Specht et al. (2009)

Reference

Authors	Specht K, Zahn R, Willmes K, Weis S, Holtel C, Krause BJ, Herzog H, Huber W
Title	Joint independent component analysis of structural and functional images reveals complex patterns of functional reorganisation in stroke aphasia
Reference	*NeuroImage* 2009; 47: 2057-2063
PMID	19524049
DOI	10.1016/j.neuroimage.2009.06.011

Participants

Language	German
Inclusion criteria	—
Number of individuals with aphasia	12
Number of control participants	12
Were any of the participants included in any previous studies?	No
Is age reported for patients and controls, and matched?	No (mean 49 + 14 years, range 30-71 years; controls were younger)
Is sex reported for patients and controls, and matched?	Yes (males: 9; females: 3)
Is handedness reported for patients and controls, and matched?	No
Is time post stroke onset reported and appropriate to the study design?	No (mean 1.9 ± 1.4 years, range 0.2-3.7 years; one non-chronic patient is included)
To what extent is the nature of aphasia characterized?	Comprehensive battery
Language evaluation	AAT
Aphasia severity	Not stated
Aphasia type	3 global, 3 Wernicke's, 2 amnestic, 2 Broca's, 2 unclassified
First stroke only?	Not stated
Stroke type	Not stated
To what extent is the lesion distribution characterized?	Lesion overlay
Lesion extent	Not stated
Lesion location	L MCA, with greatest overlap in the posterior STG
Participants notes	15 controls were scanned but 3 were randomly excluded to match group sizes for jICA.

Imaging

Modality	PET (rCBF)
Is the study cross-sectional or longitudinal?	Cross-sectional
If longitudinal, at what time point(s) were imaging data acquired?	—
If longitudinal, was there any intervention between the time points?	—
Is the scanner described?	Yes (CTI-Siemens HR+)
Is the timing of stimulus presentation and image acquisition clearly described and appropriate?	Yes
Design type	PET
Total images acquired	9
Are the imaging acquisition parameters, including coverage, adequately described and appropriate?	Yes (whole brain)
Is preprocessing and intrasubject coregistration adequately described and appropriate?	Yes
Is first level model fitting adequately described and appropriate?	Yes
Is intersubject normalization adequately described and appropriate?	Yes
Imaging notes	—

Conditions

Are the conditions clearly described?	Yes

Condition	Response type	Repetitions	All groups could do?	All individuals could do?
lexical decision (words vs pseudowords)	Button press	3	Yes	Yes
lexical decision (words vs reversed foreign words)	Button press	3	Yes	Yes

tone decision	Button press	3	Yes	Yes

Conditions notes	Behavioral data was lost, but it is clearly stated that all participants could perform all tasks above chance; the tone decision task is not described in sufficient detail, but since it is not used in any contrast of interest, the conditions are coded as being clearly described

Contrasts

Are the contrasts clearly described?	Yes

Contrast 1: lexical decision (words vs pseudowords) vs lexical decision (words vs reversed foreign words)

Language condition	Lexical decision (words vs pseudowords)
Control condition	Lexical decision (words vs reversed foreign words)
Are the conditions matched for visual demands?	Yes
Are the conditions matched for auditory demands?	Yes
Are the conditions matched for motor demands?	Yes
Are the conditions matched for cognitive/executive demands?	Yes
Is accuracy matched between the language and control tasks for all relevant groups?	Unknown, not reported
Is reaction time matched between the language and control tasks for all relevant groups?	Unknown, not reported
Behavioral data notes	—
Are control data reported in this paper or another that is referenced?	Yes
Does the contrast selectively activate plausible relevant language regions in the control group?	Somewhat
Are activations lateralized in the control data?	Yes
Control activation notes	The contrast activated a ventral part of the L IFG, along with L anterior cingulate and L DLPFC
Contrast notes	—

Analyses

Are the analyses clearly described?	Yes

Voxelwise analysis 1

First level contrast	Lexical decision (words vs pseudowords) vs lexical decision (words vs reversed foreign words)
Analysis class	Cross-sectional aphasia vs control
Group(s)	Aphasia vs control
Covariate	—
Is the second level contrast valid in terms of the group(s), time point(s), and measures involved?	Yes
Is accuracy matched across the second level contrast?	Unknown, not reported
Is reaction time matched across the second level contrast?	Unknown, not reported
Behavioral data notes	—
Type of analysis	Voxelwise
Search volume	Whole brain
Correction for multiple comparisons	Clusterwise correction based on arbitrary cluster extent
Software	SPM5
Voxelwise p	.001
Cluster extent	0.64 cc
Statistical details	—
Findings	↑ R posterior STG<br>↑ R Heschl's gyrus
Findings notes	Activation is 1105 voxels (> 8 cc) so quite convincing, but when the contrast was examined in the patient group, this region was not activated.

Complex analysis 1

First level contrast	Lexical decision (words vs pseudowords) vs lexical decision (words vs reversed foreign words)
Analysis class	Cross-sectional aphasia vs control
Group(s)	Aphasia vs control
Covariate	—
Is the second level contrast valid in terms of the group(s), time point(s), and measures involved?	Yes
Is accuracy matched across the second level contrast?	Unknown, not reported
Is reaction time matched across the second level contrast?	Unknown, not reported
Behavioral data notes	—
Type of analysis	Complex
Statistical details	Joint ICA was performed on structural and functional contrast images using FIT 1.1b. Only 1 of the 8 components differed between groups in its loadings and was interpretable. The structural part of this component related to the patients' lesions. The functional part was thresholded at voxelwise p < .001 (CDT), arbitrary minimum cluster extent = 0.64 cc.
Findings	Other
Findings notes	The component that differed between groups showed more activation for patients than controls in the L anterior temporal lobe, L cerebellum, R posterior STG, R anterior temporal lobe, R posterior inferior temporal gyrus/fusiform gyrus, R cerebellum, and R brainstem, and less activation in patients than controls in the L IFG, L anterior temporal lobe, L occipital lobe, L anterior cingulate, L cerebellum, L thalamus, and R IFG.

Notes

Excluded analyses	—

Warren et al. (2009)

Reference

Authors	Warren JE, Crinion JT, Lambon Ralph MA, Wise RJ
Title	Anterior temporal lobe connectivity correlates with functional outcome after aphasic stroke
Reference	*Brain* 2009; 132: 3428-3442
PMID	19903736
DOI	10.1093/brain/awp270

Participants

Language	UK English
Inclusion criteria	Comprehension deficit per CAT and TROG (1 patient did not meet this criterion); anterolateral superior temporal cortex spared
Number of individuals with aphasia	16 (plus 8 excluded: lesions involved L anterolateral superior temporal cortex)
Number of control participants	11
Were any of the participants included in any previous studies?	Yes (reanalysis of subset of dataset from Crinion et al. (2006))
Is age reported for patients and controls, and matched?	No (mean 65.8 ± 2.0 SEM years; controls were younger)
Is sex reported for patients and controls, and matched?	Yes (males: 11; females: 5)
Is handedness reported for patients and controls, and matched?	Yes (right: 16; left: 0)
Is time post stroke onset reported and appropriate to the study design?	No (mean 28.8 ± 9.2 months SEM; minimum time post onset not reported, but some patients in Crinion et al. (2006) were subacute)
To what extent is the nature of aphasia characterized?	Not at all

Language evaluation	CAT, TROG
Aphasia severity	Not stated
Aphasia type	Not stated
First stroke only?	Yes
Stroke type	Ischemic only
To what extent is the lesion distribution characterized?	Lesion overlay
Lesion extent	Patients with positive anterior temporal interconnectivity: mean 93.3 ± 24.0 cc; patients with negative anterior temporal interconnectivity: mean 96.1 ± 27.6 cc
Lesion location	L not including anterolateral superior temporal cortex; maximal overlap in posterior superior temporal cortex
Participants notes	—

Imaging

Modality	PET (rCBF)
Is the study cross-sectional or longitudinal?	Cross-sectional
If longitudinal, at what time point(s) were imaging data acquired?	—
If longitudinal, was there any intervention between the time points?	—
Is the scanner described?	Yes (CTI-Siemens ECAT EXACT HR++/966 (10 patients and all controls) or GE Advance (6 patients))
Is the timing of stimulus presentation and image acquisition clearly described and appropriate?	Yes
Design type	PET
Total images acquired	12-16
Are the imaging acquisition parameters, including coverage, adequately described and appropriate?	Yes (whole brain)
Is preprocessing and intrasubject coregistration adequately described and appropriate?	Yes
Is first level model fitting adequately described and appropriate?	Yes
Is intersubject normalization adequately described and appropriate?	Yes
Imaging notes	two different scanners used for patients, but not for controls

Conditions

Are the conditions clearly described?	Yes

Condition	Response type	Repetitions	All groups could do?	All individuals could do?
listening to narrative speech	None	6-8	N/A	N/A
listening to reversed speech	None	6-8	N/A	N/A

Conditions notes	—

Contrasts

Are the contrasts clearly described?	Yes

Contrast 1: listening to narrative speech vs listening to reversed speech

Language condition	Listening to narrative speech
Control condition	Listening to reversed speech
Are the conditions matched for visual demands?	Yes
Are the conditions matched for auditory demands?	Yes
Are the conditions matched for motor demands?	Yes
Are the conditions matched for cognitive/executive demands?	Yes

Is accuracy matched between the language and control tasks for all relevant groups?	N/A, no behavioral measure
Is reaction time matched between the language and control tasks for all relevant groups?	N/A, no timeable task
Behavioral data notes	—
Are control data reported in this paper or another that is referenced?	Somewhat
Does the contrast selectively activate plausible relevant language regions in the control group?	Yes
Are activations lateralized in the control data?	Somewhat
Control activation notes	11 participants; L-lateralized posterior temporal, bilateral anterior temporal, no frontal
Contrast notes	—

Analyses

Are the analyses clearly described?	Yes

ROI analysis 1

First level contrast	Listening to narrative speech vs listening to reversed speech
Analysis class	Cross-sectional aphasia vs control
Group(s)	Aphasia vs control
Covariate	—
Is the second level contrast valid in terms of the group(s), time point(s), and measures involved?	Yes
Is accuracy matched across the second level contrast?	N/A, no behavioral measure
Is reaction time matched across the second level contrast?	N/A, no timeable task
Behavioral data notes	—
Type of analysis	Regions of interest (ROI)
ROI type	Anatomical
How many ROIs are there?	6
What are the ROI(s)?	(1) L anterior superior temporal cortex; (2) L basal temporal language area; (3) L IFG pars triangularis; (4-6) homotopic counterparts
How are the ROI(s) defined?	ROIs were defined anatomically in regions that were functionally connected with L anterior superior temporal cortex in controls (1-4) or homotopic to these (5-6)
Correction for multiple comparisons	No correction
Statistical details	Somewhat circular because ROIs were defined only in regions where controls showed significant connectivity (even though ROIs were anatomical)
Findings	None
Findings notes	L IFG pars triangularis almost reached significance ($p = .053$) for more activation in patients

ROI analysis 2

First level contrast	Listening to narrative speech vs listening to reversed speech
Analysis class	Cross-sectional correlation with language or other measure
Group(s)	Aphasia
Covariate	Auditory sentence comprehension
Is the second level contrast valid in terms of the group(s), time point(s), and measures involved?	Yes
Is accuracy matched across the second level contrast?	N/A, no behavioral measure
Is reaction time matched across the second level contrast?	N/A, no timeable task
Behavioral data notes	—
Type of analysis	Regions of interest (ROI)
ROI type	Anatomical
How many ROIs are there?	6

What are the ROI(s)?	(1) L anterior superior temporal cortex; (2) L basal temporal language area; (3) L IFG pars triangularis; (4-6) homotopic counterparts
How are the ROI(s) defined?	ROIs were defined anatomically in regions that were functionally connected with L anterior superior temporal cortex in controls (1-4) or homotopic to these (5-6)
Correction for multiple comparisons	No correction
Statistical details	—
Findings	↑ L anterior temporal
Findings notes	—

ROI analysis 3

First level contrast	Listening to narrative speech vs listening to reversed speech
Analysis class	Cross-sectional correlation with language or other measure
Group(s)	Aphasia
Covariate	Written sentence comprehension
Is the second level contrast valid in terms of the group(s), time point(s), and measures involved?	Yes
Is accuracy matched across the second level contrast?	N/A, no behavioral measure
Is reaction time matched across the second level contrast?	N/A, no timeable task
Behavioral data notes	—
Type of analysis	Regions of interest (ROI)
ROI type	Anatomical
How many ROIs are there?	6
What are the ROI(s)?	(1) L anterior superior temporal cortex; (2) L basal temporal language area; (3) L IFG pars triangularis; (4-6) homotopic counterparts
How are the ROI(s) defined?	ROIs were defined anatomically in regions that were functionally connected with L anterior superior temporal cortex in controls (1-4) or homotopic to these (5-6)
Correction for multiple comparisons	No correction
Statistical details	—
Findings	None
Findings notes	—

ROI analysis 4

First level contrast	Listening to narrative speech vs listening to reversed speech
Analysis class	Cross-sectional correlation with language or other measure
Group(s)	Aphasia
Covariate	Auditory single word comprehension
Is the second level contrast valid in terms of the group(s), time point(s), and measures involved?	Yes
Is accuracy matched across the second level contrast?	N/A, no behavioral measure
Is reaction time matched across the second level contrast?	N/A, no timeable task
Behavioral data notes	—
Type of analysis	Regions of interest (ROI)
ROI type	Anatomical
How many ROIs are there?	6
What are the ROI(s)?	(1) L anterior superior temporal cortex; (2) L basal temporal language area; (3) L IFG pars triangularis; (4-6) homotopic counterparts
How are the ROI(s) defined?	ROIs were defined anatomically in regions that were functionally connected with L anterior superior temporal cortex in controls (1-4) or homotopic to these (5-6)
Correction for multiple comparisons	No correction
Statistical details	—
Findings	None
Findings notes	L anterior temporal $p = .08$

ROI analysis 5

First level contrast	Listening to narrative speech vs listening to reversed speech
Analysis class	Cross-sectional correlation with language or other measure
Group(s)	Aphasia
Covariate	Auditory syntactic comprehension
Is the second level contrast valid in terms of the group(s), time point(s), and measures involved?	Yes
Is accuracy matched across the second level contrast?	N/A, no behavioral measure
Is reaction time matched across the second level contrast?	N/A, no timeable task
Behavioral data notes	—
Type of analysis	Regions of interest (ROI)
ROI type	Anatomical
How many ROIs are there?	6
What are the ROI(s)?	(1) L anterior superior temporal cortex; (2) L basal temporal language area; (3) L IFG pars triangularis; (4-6) homotopic counterparts
How are the ROI(s) defined?	ROIs were defined anatomically in regions that were functionally connected with L anterior superior temporal cortex in controls (1-4) or homotopic to these (5-6)
Correction for multiple comparisons	No correction
Statistical details	—
Findings	None
Findings notes	L anterior temporal p = .09

ROI analysis 6

First level contrast	Listening to narrative speech vs listening to reversed speech
Analysis class	Cross-sectional correlation with language or other measure
Group(s)	Aphasia
Covariate	Connectivity between L and R ATL
Is the second level contrast valid in terms of the group(s), time point(s), and measures involved?	Yes
Is accuracy matched across the second level contrast?	N/A, no behavioral measure
Is reaction time matched across the second level contrast?	N/A, no timeable task
Behavioral data notes	—
Type of analysis	Regions of interest (ROI)
ROI type	Anatomical
How many ROIs are there?	2
What are the ROI(s)?	(1) L anterior superior temporal cortex; (2) R anterior superior temporal cortex
How are the ROI(s) defined?	ROIs were defined anatomically in regions that were functionally connected with L anterior superior temporal cortex in controls (1-4) or homotopic to these (5-6)
Correction for multiple comparisons	No correction
Statistical details	—
Findings	None
Findings notes	—

ROI analysis 7

First level contrast	Listening to narrative speech vs listening to reversed speech
Analysis class	Cross-sectional correlation with language or other measure
Group(s)	Aphasia
Covariate	Time post onset
Is the second level contrast valid in terms of the group(s), time point(s), and measures involved?	Yes
Is accuracy matched across the second level	N/A, no behavioral measure

contrast?	
Is reaction time matched across the second level contrast?	N/A, no timeable task
Behavioral data notes	—
Type of analysis	Region of interest (ROI)
ROI type	Anatomical
How many ROIs are there?	1
What are the ROI(s)?	L anterior superior temporal cortex
How are the ROI(s) defined?	ROIs were defined anatomically in regions that were functionally connected with L anterior superior temporal cortex in controls (1-4) or homotopic to these (5-6)
Correction for multiple comparisons	One only
Statistical details	—
Findings	None
Findings notes	—

ROI analysis 8

First level contrast	Listening to narrative speech vs listening to reversed speech
Analysis class	Cross-sectional correlation with language or other measure
Group(s)	Aphasia
Covariate	Lesion volume
Is the second level contrast valid in terms of the group(s), time point(s), and measures involved?	Yes
Is accuracy matched across the second level contrast?	N/A, no behavioral measure
Is reaction time matched across the second level contrast?	N/A, no timeable task
Behavioral data notes	—
Type of analysis	Region of interest (ROI)
ROI type	Anatomical
How many ROIs are there?	1
What are the ROI(s)?	L anterior superior temporal cortex
How are the ROI(s) defined?	ROIs were defined anatomically in regions that were functionally connected with L anterior superior temporal cortex in controls (1-4) or homotopic to these (5-6)
Correction for multiple comparisons	One only
Statistical details	—
Findings	None
Findings notes	—

ROI analysis 9

First level contrast	Listening to narrative speech vs listening to reversed speech
Analysis class	Cross-sectional aphasia vs control
Group(s)	Aphasia with positive anterior temporal interconnectivity (n = 8) vs control
Covariate	—
Is the second level contrast valid in terms of the group(s), time point(s), and measures involved?	Yes
Is accuracy matched across the second level contrast?	N/A, no behavioral measure
Is reaction time matched across the second level contrast?	N/A, no timeable task
Behavioral data notes	—
Type of analysis	Regions of interest (ROI)
ROI type	Anatomical
How many ROIs are there?	6
What are the ROI(s)?	(1) L anterior superior temporal cortex; (2) L basal temporal language area; (3) L IFG pars triangularis; (4-6) homotopic counterparts

How are the ROI(s) defined?	ROIs were defined anatomically in regions that were functionally connected with L anterior superior temporal cortex in controls (1-4) or homotopic to these (5-6)
Correction for multiple comparisons	No correction
Statistical details	Somewhat circular because ROIs were defined only in regions where controls showed significant connectivity (even though ROIs were anatomical); excluded 3 patients with L IFG damage
Findings	↑ L IFG pars triangularis
Findings notes	—

ROI analysis 10

First level contrast	Listening to narrative speech vs listening to reversed speech
Analysis class	Cross-sectional aphasia vs control
Group(s)	Aphasia with negative anterior temporal interconnectivity (n = 8) vs control
Covariate	—
Is the second level contrast valid in terms of the group(s), time point(s), and measures involved?	Yes
Is accuracy matched across the second level contrast?	N/A, no behavioral measure
Is reaction time matched across the second level contrast?	N/A, no timeable task
Behavioral data notes	—
Type of analysis	Regions of interest (ROI)
ROI type	Anatomical
How many ROIs are there?	6
What are the ROI(s)?	(1) L anterior superior temporal cortex; (2) L basal temporal language area; (3) L IFG pars triangularis; (4-6) homotopic counterparts
How are the ROI(s) defined?	ROIs were defined anatomically in regions that were functionally connected with L anterior superior temporal cortex in controls (1-4) or homotopic to these (5-6)
Correction for multiple comparisons	No correction
Statistical details	Somewhat circular because ROIs were defined only in regions where controls showed significant connectivity (even though ROIs were anatomical); excluded 1 patient with L IFG damage
Findings	None
Findings notes	—

ROI analysis 11

First level contrast	Listening to narrative speech vs listening to reversed speech
Analysis class	Cross-sectional between two groups with aphasia
Group(s)	Aphasia with positive anterior temporal interconnectivity (n = 8) vs with negative anterior temporal interconnectivity (n = 8)
Covariate	—
Is the second level contrast valid in terms of the group(s), time point(s), and measures involved?	Yes
Is accuracy matched across the second level contrast?	N/A, no behavioral measure
Is reaction time matched across the second level contrast?	N/A, no timeable task
Behavioral data notes	—
Type of analysis	Regions of interest (ROI)
ROI type	Anatomical
How many ROIs are there?	6
What are the ROI(s)?	(1) L anterior superior temporal cortex; (2) L basal temporal language area; (3) L IFG pars triangularis; (4-6) homotopic counterparts
How are the ROI(s) defined?	ROIs were defined anatomically in regions that were functionally connected with L anterior superior temporal cortex in controls (1-4) or homotopic to these (5-6)
Correction for multiple comparisons	No correction

Statistical details	Excluded 4 patients with L IFG damage
Findings	↑ L IFG pars triangularis
Findings notes	—

Complex analysis 1

First level contrast	Listening to narrative speech vs listening to reversed speech
Analysis class	Cross-sectional correlation with language or other measure
Group(s)	Aphasia
Covariate	Lesion status of each voxel
Is the second level contrast valid in terms of the group(s), time point(s), and measures involved?	Yes
Is accuracy matched across the second level contrast?	N/A, no behavioral measure
Is reaction time matched across the second level contrast?	N/A, no timeable task
Behavioral data notes	—
Type of analysis	Complex
Statistical details	VLSM with FDR correction was used to identify any regions in which damage was predictive of L anterior temporal activation.
Findings	None
Findings notes	—

Notes

Excluded analyses	(1) all connectivity analyses because they were based on either both conditions (whole brain analysis) or only the narrative condition (ROI analyses), except where connectivity was investigated in relation to task-based activation differences; (2) correlation with age (covariate not language-related)

Chau et al. (2010)

Reference

Authors	Chau AC, Fai Cheung RT, Jiang X, Au-Yeung PK, Li LS
Title	An fMRI study showing the effect of acupuncture in chronic stage stroke patients with aphasia
Reference	*J Acupunct Meridian Stud* 2010; 30: 53-57
PMID	20633517
DOI	10.1016/s2005-2901(10)60009-x

Participants

Language	Cantonese
Inclusion criteria	—
Number of individuals with aphasia	7
Number of control participants	0
Were any of the participants included in any previous studies?	No
Is age reported for patients and controls, and matched?	Yes (mean 63 ± 10 years, range 56-79 years)
Is sex reported for patients and controls, and matched?	Yes (males: 5; females: 2)
Is handedness reported for patients and controls, and matched?	Yes (right: 7; left: 0)
Is time post stroke onset reported and appropriate to the study design?	Yes (mean 17 ± 8 months, range 8-28 months)
To what extent is the nature of aphasia	Severity only

characterized?	
Language evaluation	Cantonese Aphasia Battery (modified WAB)
Aphasia severity	5 patients had AQ > 75, 2 had AQ < 30
Aphasia type	Not stated
First stroke only?	Yes
Stroke type	Ischemic only
To what extent is the lesion distribution characterized?	Location only
Lesion extent	Not stated
Lesion location	3 L MCA, 2 L frontal, 2 L basal ganglia
Participants notes	—

Imaging

Modality	fMRI
Is the study cross-sectional or longitudinal?	Longitudinal—chronic treatment
If longitudinal, at what time point(s) were imaging data acquired?	T1: pre-treatment/chronic; T2: post-treatment, ~10 weeks later
If longitudinal, was there any intervention between the time points?	Acupuncture, 3 sessions/week, 8 weeks
Is the scanner described?	No (not stated)
Is the timing of stimulus presentation and image acquisition clearly described and appropriate?	No (inconsistent information regarding timing)
Design type	Block
Total images acquired	90?
Are the imaging acquisition parameters, including coverage, adequately described and appropriate?	Yes (whole brain)
Is preprocessing and intrasubject coregistration adequately described and appropriate?	Yes
Is first level model fitting adequately described and appropriate?	Yes
Is intersubject normalization adequately described and appropriate?	No (lesion impact not addressed)
Imaging notes	—

Conditions

Are the conditions clearly described?	No* (moderate limitation) (nature of questions not described in detail)

Condition	Response type	Repetitions	All groups could do?	All individuals could do?
answering questions from Cantonese Aphasia Battery	Button press	3	Unknown	Unknown
visual decision	Button press	3	Unknown	Unknown

Conditions notes	Responses involved raising left or right finger (not button press per se)

Contrasts

Are the contrasts clearly described?	Yes

Contrast 1: answering questions from Cantonese Aphasia Battery vs visual decision

Language condition	Answering questions from Cantonese Aphasia Battery
Control condition	Visual decision
Are the conditions matched for visual demands?	No
Are the conditions matched for auditory demands?	No
Are the conditions matched for motor demands?	Yes
Are the conditions matched for cognitive/executive demands?	No

Is accuracy matched between the language and control tasks for all relevant groups?	N/A, tasks not comparable
Is reaction time matched between the language and control tasks for all relevant groups?	N/A, tasks not comparable
Behavioral data notes	—
Are control data reported in this paper or another that is referenced?	No
Does the contrast selectively activate plausible relevant language regions in the control group?	Unknown
Are activations lateralized in the control data?	Unknown
Control activation notes	—
Contrast notes	—

Analyses

Are the analyses clearly described?	No* (moderate limitation) (see specific limitation(s) below)

Voxelwise analysis 1

First level contrast	Answering questions from Cantonese Aphasia Battery vs visual decision
Analysis class	Longitudinal correlation with language or other measure
Group(s)	Aphasia T2 vs T1
Covariate	Δ WAB AQ
Is the second level contrast valid in terms of the group(s), time point(s), and measures involved?	Somewhat (no treatment effect)
Is accuracy matched across the second level contrast?	Unknown, not reported
Is reaction time matched across the second level contrast?	Unknown, not reported
Behavioral data notes	—
Type of analysis	Voxelwise
Search volume	Whole brain
Correction for multiple comparisons	Unclear or not stated
Software	SPM2
Voxelwise p	—
Cluster extent	—
Statistical details	Stated to be corrected $p < 0.05$, but the nature of correction is not described; it is not entirely clear whether the functional measure was the difference between T1 and T2 (we assume it is); it is also not clear whether or not 2 patients with low AQ were excluded (we assume not)
Findings	↑ L posterior MTG
Findings notes	Finding based on table; additional small activations are shown in figure but not table

Notes

Excluded analyses	—

Fridriksson (2010)

Reference

Authors	Fridriksson J
Title	Preservation and modulation of specific left hemisphere regions is vital for treated recovery from anomia in stroke
Reference	*J Neurosci* 2010; 30: 11558-11564
PMID	20810877
DOI	10.1523/jneurosci.2227-10.2010

Participants

Language	US English
Inclusion criteria	—
Number of individuals with aphasia	19 (plus 7 excluded: 6 for making fewer than 5 correct responses in one or more sessions; 1 for excessive head motion)
Number of control participants	0
Were any of the participants included in any previous studies?	Yes ("several" patients overlapped with those reported by Fridriksson et al. (2009, 2010))
Is age reported for patients and controls, and matched?	Yes (mean 59.7 ± 12.3 years)
Is sex reported for patients and controls, and matched?	Yes (males: 12; females: 14)
Is handedness reported for patients and controls, and matched?	No
Is time post stroke onset reported and appropriate to the study design?	Yes (> 8 months; actual TPO not stated)
To what extent is the nature of aphasia characterized?	Severity and type
Language evaluation	WAB
Aphasia severity	AQ mean 60.4 ± 25.6 (including excluded patients)
Aphasia type	11 anomic, 10 Broca's, 3 conduction, 1 transcortical motor, 1 Wernicke's (including excluded patients)
First stroke only?	Yes
Stroke type	Ischemic only
To what extent is the lesion distribution characterized?	Lesion overlay
Lesion extent	Not stated
Lesion location	L MCA
Participants notes	Demographic data includes excluded patients

Imaging

Modality	fMRI
Is the study cross-sectional or longitudinal?	Longitudinal—chronic treatment
If longitudinal, at what time point(s) were imaging data acquired?	T1: pre-treatment/chronic; T2: post-treatment/~4 weeks later; note that there were two separate sessions per time point, as well as another two sessions midway through treatment that are not analyzed in this paper
If longitudinal, was there any intervention between the time points?	Anomia treatment using a cueing hierarchy, 3 hours/day, 5 days/week, 2 weeks, with a 1-week gap between the two weeks
Is the scanner described?	Yes (Siemens Trio 3 Tesla)
Is the timing of stimulus presentation and image acquisition clearly described and appropriate?	No (timing of stimuli within the silent periods is unclear)
Design type	Event-related
Total images acquired	120
Are the imaging acquisition parameters, including coverage, adequately described and appropriate?	Yes (whole brain)
Is preprocessing and intrasubject coregistration adequately described and appropriate?	Yes
Is first level model fitting adequately described and appropriate?	Yes
Is intersubject normalization adequately described and appropriate?	Yes
Imaging notes	sparse sampling

Conditions

Are the conditions clearly described?	Yes

Condition	Response type	Repetitions	All groups could do?	All individuals could do?
picture naming	Word (overt)	80	Yes	Unknown
viewing abstract pictures	None	40	N/A	N/A

Conditions notes	Patients with fewer than 5 correct responses in any session were excluded; there were probably some patients who made 5 or more correct responses but less than 10%, but this is not reported

Contrasts

Are the contrasts clearly described?	Yes

Contrast 1: picture naming (correct trials) vs viewing abstract pictures

Language condition	Picture naming (correct trials)
Control condition	Viewing abstract pictures
Are the conditions matched for visual demands?	Yes
Are the conditions matched for auditory demands?	No
Are the conditions matched for motor demands?	No
Are the conditions matched for cognitive/executive demands?	No
Is accuracy matched between the language and control tasks for all relevant groups?	N/A, tasks not comparable
Is reaction time matched between the language and control tasks for all relevant groups?	N/A, tasks not comparable
Behavioral data notes	—
Are control data reported in this paper or another that is referenced?	Somewhat
Does the contrast selectively activate plausible relevant language regions in the control group?	No
Are activations lateralized in the control data?	Somewhat
Control activation notes	Control data in Fridriksson et al. (2007); motor activations are prominent; there is some L frontal activation but little temporal activation in either hemisphere.
Contrast notes	—

Analyses

Are the analyses clearly described?	Yes

Voxelwise analysis 1

First level contrast	Picture naming (correct trials) vs viewing abstract pictures
Analysis class	Longitudinal correlation with language or other measure
Group(s)	Aphasia T2 vs T1
Covariate	Δ picture naming accuracy
Is the second level contrast valid in terms of the group(s), time point(s), and measures involved?	Yes
Is accuracy matched across the second level contrast?	Yes, correct trials only
Is reaction time matched across the second level contrast?	Unknown, not reported
Behavioral data notes	—
Type of analysis	Voxelwise
Search volume	Whole brain
Correction for multiple comparisons	Clusterwise correction with with GRFT and lenient voxelwise p
Software	FSL 4.1
Voxelwise p	~.01 (z > 2.3)
Cluster extent	Based on GRFT
Statistical details	—
Findings	↑ L dorsolateral prefrontal cortex

	↑ L ventral precentral/inferior frontal junction<br>↑ L supramarginal gyrus<br>↑ L intraparietal sulcus<br>↑ L superior parietal<br>↑ L precuneus
Findings notes	Activated regions were on the borders on the lesion distribution in the 19 included patients

Notes

Excluded analyses	—

Fridriksson et al. (2010)

Reference

Authors	Fridriksson J, Bonilha L, Baker JM, Moser D, Rorden C
Title	Activity in preserved left hemisphere regions predicts anomia severity in aphasia
Reference	*Cereb Cortex* 2010; 20: 1013-1019
PMID	19687294
DOI	10.1093/cercor/bhp160

Participants

Language	US English
Inclusion criteria	—
Number of individuals with aphasia	15
Number of control participants	9
Were any of the participants included in any previous studies?	No
Is age reported for patients and controls, and matched?	Yes (mean 61.9 years, range 41-81 years)
Is sex reported for patients and controls, and matched?	No (males: 7; females: 8; not stated for controls)
Is handedness reported for patients and controls, and matched?	No
Is time post stroke onset reported and appropriate to the study design?	Yes (mean 29.7 months, > 6 months)
To what extent is the nature of aphasia characterized?	Severity and type
Language evaluation	WAB
Aphasia severity	AQ mean 77.1, range 47.1-93.7
Aphasia type	10 anomic, 3 Broca's, 2 conduction
First stroke only?	Yes
Stroke type	Ischemic only
To what extent is the lesion distribution characterized?	Lesion overlay
Lesion extent	Not stated
Lesion location	L MCA
Participants notes	—

Imaging

Modality	fMRI
Is the study cross-sectional or longitudinal?	Cross-sectional
If longitudinal, at what time point(s) were imaging data acquired?	—
If longitudinal, was there any intervention between	—

the time points?	
Is the scanner described?	Yes (Siemens Trio 3 Tesla)
Is the timing of stimulus presentation and image acquisition clearly described and appropriate?	No (exact timing of picture presentation not specified)
Design type	Event-related
Total images acquired	120
Are the imaging acquisition parameters, including coverage, adequately described and appropriate?	Yes (whole brain)
Is preprocessing and intrasubject coregistration adequately described and appropriate?	Yes
Is first level model fitting adequately described and appropriate?	Yes
Is intersubject normalization adequately described and appropriate?	Yes
Imaging notes	sparse sampling

Conditions

Are the conditions clearly described?	Yes

Condition	Response type	Repetitions	All groups could do?	All individuals could do?
picture naming	Word (overt)	80	Yes	Yes
viewing abstract pictures	None	40	N/A	N/A

Conditions notes	—

Contrasts

Are the contrasts clearly described?	Yes

Contrast 1: picture naming (correct trials) vs viewing abstract pictures

Language condition	Picture naming (correct trials)
Control condition	Viewing abstract pictures
Are the conditions matched for visual demands?	Yes
Are the conditions matched for auditory demands?	No
Are the conditions matched for motor demands?	No
Are the conditions matched for cognitive/executive demands?	No
Is accuracy matched between the language and control tasks for all relevant groups?	N/A, tasks not comparable
Is reaction time matched between the language and control tasks for all relevant groups?	N/A, tasks not comparable
Behavioral data notes	—
Are control data reported in this paper or another that is referenced?	Somewhat
Does the contrast selectively activate plausible relevant language regions in the control group?	Somewhat
Are activations lateralized in the control data?	Somewhat
Control activation notes	L-lateralized frontal and temporal activations, but also bilateral visual, motor and auditory
Contrast notes	—

Analyses

Are the analyses clearly described?	Yes

Voxelwise analysis 1

First level contrast	Picture naming (correct trials) vs viewing abstract pictures
Analysis class	Cross-sectional correlation with language or other measure
Group(s)	Aphasia

Covariate	Picture naming accuracy
Is the second level contrast valid in terms of the group(s), time point(s), and measures involved?	Yes
Is accuracy matched across the second level contrast?	Yes, correct trials only
Is reaction time matched across the second level contrast?	Unknown, not reported
Behavioral data notes	—
Type of analysis	Voxelwise
Search volume	Whole brain
Correction for multiple comparisons	Clusterwise correction with with GRFT and lenient voxelwise p
Software	FSL 4.1
Voxelwise p	~.02 (z > 2)
Cluster extent	Based on GRFT
Statistical details	—
Findings	↑ L IFG pars orbitalis<br>↑ L occipital<br>↑ L anterior cingulate
Findings notes	Greater activation was associated with better picture naming; L IFG pars orbitalis activation classified as middle frontal gyrus in the paper, but coordinates suggest otherwise

Voxelwise analysis 2

First level contrast	Picture naming (correct trials) vs viewing abstract pictures
Analysis class	Cross-sectional aphasia vs control
Group(s)	Aphasia vs control
Covariate	—
Is the second level contrast valid in terms of the group(s), time point(s), and measures involved?	Yes
Is accuracy matched across the second level contrast?	Yes, correct trials only
Is reaction time matched across the second level contrast?	Unknown, not reported
Behavioral data notes	—
Type of analysis	Voxelwise
Search volume	Whole brain
Correction for multiple comparisons	Clusterwise correction with with GRFT and lenient voxelwise p
Software	FSL 4.1
Voxelwise p	~.02 (z > 2)
Cluster extent	Based on GRFT
Statistical details	—
Findings	None
Findings notes	—

ROI analysis 1

First level contrast	Picture naming (correct trials) vs viewing abstract pictures
Analysis class	Cross-sectional correlation with language or other measure
Group(s)	Aphasia
Covariate	Picture naming accuracy
Is the second level contrast valid in terms of the group(s), time point(s), and measures involved?	Yes
Is accuracy matched across the second level contrast?	Yes, correct trials only
Is reaction time matched across the second level contrast?	Unknown, not reported
Behavioral data notes	—
Type of analysis	Region of interest (ROI)

ROI type	Functional
How many ROIs are there?	1
What are the ROI(s)?	A single ROI comprising 3 regions where activation in patients was correlated with picture naming accuracy: the L IFG pars orbitalis, occipital lobe, and anterior cingulate
How are the ROI(s) defined?	Based on SPM analysis 1
Correction for multiple comparisons	One only
Statistical details	The purpose of this analysis was to determine whether these regions were recruited in the patients with better naming, or not activated in the patients with worse naming, relative to the control mean
Findings	Other
Findings notes	Patients with better naming showed greater activation than controls, while the patients with poorer naming showed less activation than controls.

Complex analysis 1

First level contrast	Picture naming (correct trials) vs viewing abstract pictures
Analysis class	Cross-sectional correlation with language or other measure
Group(s)	Aphasia
Covariate	Lesion status of each voxel
Is the second level contrast valid in terms of the group(s), time point(s), and measures involved?	Yes
Is accuracy matched across the second level contrast?	Yes, correct trials only
Is reaction time matched across the second level contrast?	Unknown, not reported
Behavioral data notes	—
Type of analysis	Complex
Statistical details	VLSM was used to identify any regions in which damage was predictive of activation in the regions identified in SPM analysis 1, considered as a single ROI. There was no correction for multiple comparisons, and the analysis is appropriately presented as exploratory.
Findings	Other
Findings notes	Only in the L IFG pars opercularis was damage predictive of reduced activation in the potentially compensatory network.

Notes

Excluded analyses	—

Sharp et al. (2010)

Reference

Authors	Sharp DJ, Turkheimer FE, Bose SK, Scott SK, Wise RJ
Title	Increased frontoparietal integration after stroke and cognitive recovery
Reference	*Ann Neurol* 2010; 68: 753-756
PMID	20687116
DOI	10.1002/ana.21866

Participants

Language	UK English
Inclusion criteria	Lesion in vicinity of L STG; no extensive frontal damage; no inferior temporal damage; able to perform tasks
Number of individuals with aphasia	9
Number of control participants	18
Were any of the participants included in any previous studies?	Yes (additional analysis of same dataset as Sharp et al. (2004))

Is age reported for patients and controls, and matched?	Yes (median 58 years, range 39-72 years)
Is sex reported for patients and controls, and matched?	Yes (males: 8; females: 1)
Is handedness reported for patients and controls, and matched?	Yes (right: 9; left: 0)
Is time post stroke onset reported and appropriate to the study design?	Yes (mean 45 months, range 14-145 months)
To what extent is the nature of aphasia characterized?	Severity only
Language evaluation	Subtests from CAT, subtests from PALPA, Action for dysphasic adults, TROG, PPT
Aphasia severity	Mild
Aphasia type	Not stated
First stroke only?	Yes
Stroke type	Not stated
To what extent is the lesion distribution characterized?	Lesion overlay
Lesion extent	Not stated
Lesion location	Lesion in vicinity of L STG; no extensive frontal damage; no inferior temporal damage
Participants notes	—

Imaging

Modality	PET (rCBF)
Is the study cross-sectional or longitudinal?	Cross-sectional
If longitudinal, at what time point(s) were imaging data acquired?	—
If longitudinal, was there any intervention between the time points?	—
Is the scanner described?	Yes (Siemens HR++ 966)
Is the timing of stimulus presentation and image acquisition clearly described and appropriate?	Yes
Design type	PET
Total images acquired	16
Are the imaging acquisition parameters, including coverage, adequately described and appropriate?	Yes (whole brain)
Is preprocessing and intrasubject coregistration adequately described and appropriate?	Yes
Is first level model fitting adequately described and appropriate?	Yes
Is intersubject normalization adequately described and appropriate?	Yes
Imaging notes	—

Conditions

Are the conditions clearly described?	Yes

Condition	Response type	Repetitions	All groups could do?	All individuals could do?
semantic decision	Word (overt)	aphasia: 8; control: 4	Yes	Yes
syllable count decision	Word (overt)	aphasia: 8; control: 4	Yes	Unknown
semantic decision (noise vocoded) (control only)	Word (overt)	4 (control)	Yes	Yes
syllable count decision (noise vocoded) (control only)	Word (overt)	4 (control)	Yes	Yes

Conditions notes	Seems the response was a spoken word, but this is not stated explicitly; assuming all

	individuals could do the semantic task because this was an inclusion criterion and behavioral data (PPT) supports, but not sure about the phonological task

Contrasts

Are the contrasts clearly described?	Yes

Contrast 1: semantic decision (clear in patients; average of clear and noise vocoded in controls) vs syllable count decision (clear in patients; average of clear and noise vocoded in controls)

Language condition	Semantic decision (clear in patients; average of clear and noise vocoded in controls)
Control condition	Syllable count decision (clear in patients; average of clear and noise vocoded in controls)
Are the conditions matched for visual demands?	Yes
Are the conditions matched for auditory demands?	Yes
Are the conditions matched for motor demands?	Yes
Are the conditions matched for cognitive/executive demands?	Yes
Is accuracy matched between the language and control tasks for all relevant groups?	No, different
Is reaction time matched between the language and control tasks for all relevant groups?	No, different
Behavioral data notes	Significant differences per Sharp et al. (2004)
Are control data reported in this paper or another that is referenced?	Somewhat
Does the contrast selectively activate plausible relevant language regions in the control group?	Somewhat
Are activations lateralized in the control data?	Yes
Control activation notes	Not stated exactly what contrast was used in controls
Contrast notes	—

Analyses

Are the analyses clearly described?	Yes

ROI analysis 1

First level contrast	Semantic decision (clear in patients; average of clear and noise vocoded in controls) vs syllable count decision (clear in patients; average of clear and noise vocoded in controls)
Analysis class	Cross-sectional aphasia vs control
Group(s)	Aphasia vs control
Covariate	—
Is the second level contrast valid in terms of the group(s), time point(s), and measures involved?	Yes
Is accuracy matched across the second level contrast?	No, but attempt made
Is reaction time matched across the second level contrast?	Appear similar
Behavioral data notes	Accuracy and RT were not significantly different for the semantic task; statistics are not reported for the syllable counting task, but the data provided suggest that accuracy was probably not matched, while RT probably was
Type of analysis	Regions of interest (ROI)
ROI type	Other
How many ROIs are there?	12
What are the ROI(s)?	Functional connectivity between pairs of spared nodes of the L hemisphere semantic network and R hemisphere homotopic regions: (1) L SFG-L AG; (2) L SFG-L IFG; (3) L SFG-L IT; (4) L AG-L IFG; (5) L AG-L IT; (6) L IFG-L IT; (7-12) homotopic counterparts
How are the ROI(s) defined?	Partial correlations between nodes
Correction for multiple comparisons	No direct comparison
Statistical details	—
Findings	Other

Findings notes	Patients showed greater connectivity between L SFG and L AG than controls while performing the semantic task; this was not the case for the syllable counting task, however connectivity during performance of the two tasks was not compared directly

Notes

Excluded analyses	(1) correlations between connection strength of AG-IT and language performance, because there was no functional control condition; (2) controls showed greater connectivity between L SFG and L AG while performing the semantic task with noise vocoded speech relative to clear speech, supporting the interpretation that greater connectivity reflects effortful processing

Thompson et al. (2010)

Reference

Authors	Thompson CK, den Ouden DB, Bonakdarpour B, Garibaldi K, Parrish TB
Title	Neural plasticity and treatment-induced recovery of sentence processing in agrammatism
Reference	*Neuropsychologia* 2010; 48: 3211-3227
PMID	20603138
DOI	10.1016/j.neuropsychologia.2010.06.036

Participants

Language	US English
Inclusion criteria	Agrammatic
Number of individuals with aphasia	6
Number of control participants	12
Were any of the participants included in any previous studies?	No
Is age reported for patients and controls, and matched?	Yes (mean 54 years, range 38-66 years)
Is sex reported for patients and controls, and matched?	Yes (males: 5; females: 1)
Is handedness reported for patients and controls, and matched?	Yes (right: 6; left: 0)
Is time post stroke onset reported and appropriate to the study design?	Yes (range 6-146 months)
To what extent is the nature of aphasia characterized?	Comprehensive battery
Language evaluation	WAB, NAVS, narrative language sample
Aphasia severity	AQ range 66.8-85.0
Aphasia type	All agrammatic; per WAB scores provided: 3 Broca's, 3 unclassified
First stroke only?	Yes
Stroke type	Not stated
To what extent is the lesion distribution characterized?	Individual lesions
Lesion extent	Not stated
Lesion location	5 L MCA, 1 R MCA with aphasia
Participants notes	—

Imaging

Modality	fMRI
Is the study cross-sectional or longitudinal?	Longitudinal—chronic treatment
If longitudinal, at what time point(s) were imaging data acquired?	T1: pre-treatment/chronic; T2: post-treatment, 9-15 weeks later
If longitudinal, was there any intervention between	Treatment of underlying forms

the time points?	
Is the scanner described?	Yes (Siemens Trio 3 Tesla)
Is the timing of stimulus presentation and image acquisition clearly described and appropriate?	No (total images acquired not stated)
Design type	Event-related
Total images acquired	not stated
Are the imaging acquisition parameters, including coverage, adequately described and appropriate?	Yes (whole brain)
Is preprocessing and intrasubject coregistration adequately described and appropriate?	Yes
Is first level model fitting adequately described and appropriate?	Yes
Is intersubject normalization adequately described and appropriate?	Yes
Imaging notes	—

Conditions

Are the conditions clearly described?	Yes

Condition	Response type	Repetitions	All groups could do?	All individuals could do?
auditory sentence-picture matching (auditory; object cleft)	Button press	60	No	No
auditory sentence-picture matching (subject cleft)	Button press	60	Yes	Yes
auditory sentence-picture matching (simple past tense active)	Button press	60	Yes	No
rest	None	implicit baseline	N/A	N/A

Conditions notes	—

Contrasts

Are the contrasts clearly described?	Yes

Contrast 1: auditory sentence-picture matching (all three sentence types) vs rest

Language condition	Auditory sentence-picture matching (all three sentence types)
Control condition	Rest
Are the conditions matched for visual demands?	No
Are the conditions matched for auditory demands?	No
Are the conditions matched for motor demands?	No
Are the conditions matched for cognitive/executive demands?	No
Is accuracy matched between the language and control tasks for all relevant groups?	N/A, tasks not comparable
Is reaction time matched between the language and control tasks for all relevant groups?	N/A, tasks not comparable
Behavioral data notes	—
Are control data reported in this paper or another that is referenced?	No
Does the contrast selectively activate plausible relevant language regions in the control group?	Unknown
Are activations lateralized in the control data?	Unknown
Control activation notes	—
Contrast notes	—

Analyses

Are the analyses clearly described?	Yes

ROI analysis 1

First level contrast	Auditory sentence-picture matching (all three sentence types) vs rest
Analysis class	Longitudinal change in aphasia
Group(s)	Aphasia T2 vs T1
Covariate	—
Is the second level contrast valid in terms of the group(s), time point(s), and measures involved?	Yes
Is accuracy matched across the second level contrast?	Appear similar
Is reaction time matched across the second level contrast?	Appear similar
Behavioral data notes	—
Type of analysis	Regions of interest (ROI)
ROI type	Anatomical
How many ROIs are there?	18
What are the ROI(s)?	(1) L BA 7; (2) L BA 9; (3) L BA 13; (4) L BA 21; (5) L BA 22; (6) L BA 39; (7) L BA 40; (8) L BA 44; (9) L BA 45; (10-18) homotopic counterparts
How are the ROI(s) defined?	WFU pickatlas; proportion of patients who showed increases and decreases in (parts of) each ROI in individual fixed effects SPM analyses
Correction for multiple comparisons	No correction
Statistical details	—
Findings	↑ L angular gyrus<br>↑ L superior parietal<br>↑ L mid temporal<br>↑ R supramarginal gyrus<br>↑ R superior parietal<br>↓ L insula<br>↓ L posterior STG
Findings notes	These are the regions involved in what the authors interpret as a "general shift"

Notes

Excluded analyses	Individual patient analyses

Tyler et al. (2010)

Reference

Authors	Tyler LK, Wright P, Randall B, Marslen-Wilson WD, Stamatakis EA
Title	Reorganization of syntactic processing following left-hemisphere brain damage: does right-hemisphere activity preserve function?
Reference	*Brain* 2010; 133: 3396-3408
PMID	20870779
DOI	10.1093/brain/awq262

Participants

Language	UK English
Inclusion criteria	—
Number of individuals with aphasia	14
Number of control participants	10
Were any of the participants included in any previous studies?	No
Is age reported for patients and controls, and matched?	Yes (mean 54 years, range 33-76 years)
Is sex reported for patients and controls, and	Yes (males: 11; females: 3)

matched?	
Is handedness reported for patients and controls, and matched?	Yes (right: 14; left: 0)
Is time post stroke onset reported and appropriate to the study design?	Yes (mean 7 years, range 1.4-37.3 years)
To what extent is the nature of aphasia characterized?	Not at all
Language evaluation	Sentence-picture matching, lexical decision, phonological similarity, word repetition, sentence repetition, morphological similarity, semantic categorization, sentence acceptability
Aphasia severity	Not stated
Aphasia type	Not stated
First stroke only?	Not stated
Stroke type	Mixed etiologies
To what extent is the lesion distribution characterized?	Lesion overlay
Lesion extent	Not stated
Lesion location	L
Participants notes	2 of the 14 patients were not stroke, but were post resective surgery

Imaging

Modality	fMRI
Is the study cross-sectional or longitudinal?	Cross-sectional
If longitudinal, at what time point(s) were imaging data acquired?	—
If longitudinal, was there any intervention between the time points?	—
Is the scanner described?	Yes (Siemens Trio 3 Tesla)
Is the timing of stimulus presentation and image acquisition clearly described and appropriate?	No* (moderate limitation) (there was only one block per condition per run, so condition could be confounded with low frequency drift; also, the length of the sentences is not stated so it is unclear how well the HRF peak aligns with the sparse acquisitions)
Design type	Block
Total images acquired	69
Are the imaging acquisition parameters, including coverage, adequately described and appropriate?	Yes (whole brain)
Is preprocessing and intrasubject coregistration adequately described and appropriate?	Yes
Is first level model fitting adequately described and appropriate?	Yes
Is intersubject normalization adequately described and appropriate?	Yes
Imaging notes	sparse sampling

Conditions

Are the conditions clearly described?	Yes

Condition	Response type	Repetitions	All groups could do?	All individuals could do?
listening to normal sentences and detecting a target word	Button press	2	Yes	Unknown
listening to grammatical but meaningless sentences and detecting a target word	Button press	2	Yes	Unknown
listening to scrambled sentences and detecting a target word	Button press	2	Yes	Unknown
listening to "musical rain" and detecting a period of white noise	Button press	2	Yes	Unknown
rest	None	2	N/A	N/A

Conditions notes	Auditory presentation; target detection task with early and late targets; 12-15 trials per block with single sparse acquisition each, but only one block per run, in fixed order; task can apparently be performed by patients with brain damage, but accuracy is not reported

Contrasts

Are the contrasts clearly described?	Yes

Contrast 1: listening to grammatical but meaningless sentences and detecting a target word vs listening to scrambled sentences and detecting a target word

Language condition	Listening to grammatical but meaningless sentences and detecting a target word
Control condition	Listening to scrambled sentences and detecting a target word
Are the conditions matched for visual demands?	Yes
Are the conditions matched for auditory demands?	Yes
Are the conditions matched for motor demands?	Yes
Are the conditions matched for cognitive/executive demands?	Yes
Is accuracy matched between the language and control tasks for all relevant groups?	Unknown, not reported
Is reaction time matched between the language and control tasks for all relevant groups?	Appear similar
Behavioral data notes	There appears to be a small RT difference (control condition slower)
Are control data reported in this paper or another that is referenced?	Somewhat
Does the contrast selectively activate plausible relevant language regions in the control group?	Yes
Are activations lateralized in the control data?	No
Control activation notes	There are more control participants in another paper (Tyler et al., 2010, Cereb Cortex), but the relevant contrast does not seem to be shown in that paper
Contrast notes	The contrast is intended to identify regions involved in syntactic processing, however it seems possible that there are semantic differences between these conditions also

Analyses

Are the analyses clearly described?	Yes

Voxelwise analysis 1

First level contrast	Listening to grammatical but meaningless sentences and detecting a target word vs listening to scrambled sentences and detecting a target word
Analysis class	Cross-sectional aphasia vs control
Group(s)	Aphasia vs control
Covariate	—
Is the second level contrast valid in terms of the group(s), time point(s), and measures involved?	Yes
Is accuracy matched across the second level contrast?	Unknown, not reported
Is reaction time matched across the second level contrast?	Appear similar
Behavioral data notes	The two groups showed similar differences between RTs in the two conditions of the contrast
Type of analysis	Voxelwise
Search volume	Whole brain
Correction for multiple comparisons	No direct comparison
Software	SPM5
Voxelwise p	—
Cluster extent	—
Statistical details	Qualitative comparison on pp. 3402-3; each group is presented at voxelwise p < .005 (CDT), cluster-corrected p < .05 with GRFT
Findings	↑ R IFG pars triangularis

	↑ R IFG pars orbitalis<br>↓ L posterior MTG
Findings notes	Several other potential differences are apparent in the figure, but only the differences tabulated are interpreted in the text

ROI analysis 1

First level contrast	Listening to grammatical but meaningless sentences and detecting a target word vs listening to scrambled sentences and detecting a target word
Analysis class	Cross-sectional correlation with language or other measure
Group(s)	Aphasia
Covariate	RT difference between early and late targets on grammatical but meaningless sentences (a measure of syntactic processing)
Is the second level contrast valid in terms of the group(s), time point(s), and measures involved?	Yes
Is accuracy matched across the second level contrast?	Unknown, not reported
Is reaction time matched across the second level contrast?	Unknown, not reported
Behavioral data notes	Analyses focuses on RT differences between early and late targets, not on mean RT per se
Type of analysis	Region of interest (ROI)
ROI type	Functional
How many ROIs are there?	1
What are the ROI(s)?	L IFG pars triangularis and orbitalis
How are the ROI(s) defined?	Activated for the same contrast
Correction for multiple comparisons	One only
Statistical details	—
Findings	↑ L IFG pars triangularis<br>↑ L IFG pars orbitalis
Findings notes	L IFG showed more activation in patients that had a larger target position effect (indicative of better syntactic processing)

ROI analysis 2

First level contrast	Listening to grammatical but meaningless sentences and detecting a target word vs listening to scrambled sentences and detecting a target word
Analysis class	Cross-sectional correlation with language or other measure
Group(s)	Aphasia
Covariate	RT difference between early and late targets on normal sentences
Is the second level contrast valid in terms of the group(s), time point(s), and measures involved?	Yes
Is accuracy matched across the second level contrast?	Unknown, not reported
Is reaction time matched across the second level contrast?	Unknown, not reported
Behavioral data notes	—
Type of analysis	Region of interest (ROI)
ROI type	Functional
How many ROIs are there?	1
What are the ROI(s)?	L IFG pars triangularis and orbitalis
How are the ROI(s) defined?	Activated for the same contrast
Correction for multiple comparisons	One only
Statistical details	—
Findings	None
Findings notes	—

ROI analysis 3

First level contrast	Listening to grammatical but meaningless sentences and detecting a target word vs listening

	to scrambled sentences and detecting a target word
Analysis class	Cross-sectional correlation with language or other measure
Group(s)	Aphasia
Covariate	RT difference between early and late targets on scrambled sentences
Is the second level contrast valid in terms of the group(s), time point(s), and measures involved?	Yes
Is accuracy matched across the second level contrast?	Unknown, not reported
Is reaction time matched across the second level contrast?	Unknown, not reported
Behavioral data notes	—
Type of analysis	Region of interest (ROI)
ROI type	Functional
How many ROIs are there?	1
What are the ROI(s)?	L IFG pars triangularis and orbitalis
How are the ROI(s) defined?	Activated for the same contrast
Correction for multiple comparisons	One only
Statistical details	—
Findings	None
Findings notes	—

ROI analysis 4

First level contrast	Listening to grammatical but meaningless sentences and detecting a target word vs listening to scrambled sentences and detecting a target word
Analysis class	Cross-sectional correlation with language or other measure
Group(s)	Aphasia
Covariate	Damage to L IFG, estimated from T1 signal
Is the second level contrast valid in terms of the group(s), time point(s), and measures involved?	Yes
Is accuracy matched across the second level contrast?	Unknown, not reported
Is reaction time matched across the second level contrast?	Unknown, not reported
Behavioral data notes	—
Type of analysis	Region of interest (ROI)
ROI type	Functional
How many ROIs are there?	1
What are the ROI(s)?	R IFG pars triangularis and orbitalis
How are the ROI(s) defined?	Activated for the same contrast
Correction for multiple comparisons	One only
Statistical details	—
Findings	None
Findings notes	No correlation ($p = .57$)

ROI analysis 5

First level contrast	Listening to grammatical but meaningless sentences and detecting a target word vs listening to scrambled sentences and detecting a target word
Analysis class	Cross-sectional correlation with language or other measure
Group(s)	Aphasia
Covariate	Syntactic processing (presumably the target position effect, though this is not stated)
Is the second level contrast valid in terms of the group(s), time point(s), and measures involved?	Yes
Is accuracy matched across the second level contrast?	Unknown, not reported
Is reaction time matched across the second level contrast?	Unknown, not reported

Behavioral data notes	—
Type of analysis	Region of interest (ROI)
ROI type	Functional
How many ROIs are there?	1
What are the ROI(s)?	R IFG pars triangularis and orbitalis
How are the ROI(s) defined?	Activated for the same contrast
Correction for multiple comparisons	One only
Statistical details	—
Findings	None
Findings notes	No correlation (p = .41)

Complex analysis 1

First level contrast	Listening to grammatical but meaningless sentences and detecting a target word vs listening to scrambled sentences and detecting a target word
Analysis class	Cross-sectional correlation with language or other measure
Group(s)	Aphasia
Covariate	Lesion status of each voxel
Is the second level contrast valid in terms of the group(s), time point(s), and measures involved?	Yes
Is accuracy matched across the second level contrast?	Unknown, not reported
Is reaction time matched across the second level contrast?	Unknown, not reported
Behavioral data notes	—
Type of analysis	Complex
Statistical details	VBM was used to identify any regions where damage was predictive of activation in the L IFG pars triangularis and orbitalis. Tissue integrity was quantified in terms of T1 signal. Clusterwise correction was used, which is not appropriate for VBM.
Findings	Other
Findings notes	Only in the L IFG itself was damage predictive of reduced activation in the L IFG.

Notes

Excluded analyses	(1) patients, unlike controls, showed a correlation between R IFG and R MTG activity, but the authors do not make much of this, and there is no direct comparison was reported to controls; (2) a nonsignificant correlation between L pMTG activation in patients (lacking at the group level) and tissue integrity in that same region

van Oers et al. (2010)

Reference

Authors	van Oers CA, Vink M, van Zandvoort MJ, van der Worp HB, de Haan EH, Kappelle LJ, Ramsey NF, Dijkhuizen RM
Title	Contribution of the left and right inferior frontal gyrus in recovery from aphasia: a functional MRI study in stroke patients with preserved hemodynamic responsiveness
Reference	*NeuroImage* 2010; 49: 885-893
PMID	19733673
DOI	10.1016/j.neuroimage.2009.08.057

Participants

Language	Dutch
Inclusion criteria	MCA; mRS < 3; able to perform at least 2 out of the 3 tasks
Number of individuals with aphasia	13
Number of control participants	13

Were any of the participants included in any previous studies?	No
Is age reported for patients and controls, and matched?	Yes (mean 53 ± 14 years, range 29-74 years)
Is sex reported for patients and controls, and matched?	Yes (males: 4; females: 9)
Is handedness reported for patients and controls, and matched?	No (right: 13; left: 0; not stated for controls)
Is time post stroke onset reported and appropriate to the study design?	Yes (range 1.3-4.7 years)
To what extent is the nature of aphasia characterized?	Comprehensive battery
Language evaluation	AAT, BNT, TT
Aphasia severity	4 moderate, 4 severe, 3 recovered, 2 mild; all had aphasia initially
Aphasia type	5 anomic, 4 Broca's, 3 recovered, 1 Wernicke's
First stroke only?	Yes
Stroke type	Ischemic only
To what extent is the lesion distribution characterized?	Individual lesions
Lesion extent	Range 6.0-167.3 cc
Lesion location	L MCA
Participants notes	—

Imaging

Modality	fMRI
Is the study cross-sectional or longitudinal?	Cross-sectional
If longitudinal, at what time point(s) were imaging data acquired?	Behavioral data (TT and a naming measure) were also acquired subacutely (mean 26 ± 18 days, range 5-56 days)
If longitudinal, was there any intervention between the time points?	—
Is the scanner described?	Yes (Philips Achieva 3 Tesla)
Is the timing of stimulus presentation and image acquisition clearly described and appropriate?	Yes
Design type	Block
Total images acquired	3036
Are the imaging acquisition parameters, including coverage, adequately described and appropriate?	Yes (whole brain)
Is preprocessing and intrasubject coregistration adequately described and appropriate?	Yes
Is first level model fitting adequately described and appropriate?	Yes
Is intersubject normalization adequately described and appropriate?	Yes
Imaging notes	breath holding scan also done to measure hemodynamic responsiveness

Conditions

Are the conditions clearly described?	Yes

Condition	Response type	Repetitions	All groups could do?	All individuals could do?
written word-picture matching	Button press	6	Yes	Yes
semantic decision	Button press	6	Yes	Yes
verb generation	Word (covert)	8	Yes	Yes
visual decision	Button press	12	Unknown	Unknown
rest	None	20	N/A	N/A

Conditions notes	Patients who could not do tasks were excluded from analyses of those tasks (1 patient from

	semantic decision; 3 patients from verb generation); wording is somewhat unclear regarding exclusion of patients who could not perform verb generation, but we assume they were excluded

Contrasts

Are the contrasts clearly described?	No (see specific limitation(s) below)

Contrast 1: written word-picture matching vs visual decision

Language condition	Written word-picture matching
Control condition	Visual decision
Are the conditions matched for visual demands?	No
Are the conditions matched for auditory demands?	Yes
Are the conditions matched for motor demands?	Yes
Are the conditions matched for cognitive/executive demands?	No
Is accuracy matched between the language and control tasks for all relevant groups?	Unknown, not reported
Is reaction time matched between the language and control tasks for all relevant groups?	Unknown, not reported
Behavioral data notes	Accuracy not reported for control condition
Are control data reported in this paper or another that is referenced?	Yes
Does the contrast selectively activate plausible relevant language regions in the control group?	Somewhat
Are activations lateralized in the control data?	Somewhat
Control activation notes	—
Contrast notes	Not clearly stated that language tasks were contrasted only with arrow decision task and not rest for the first two contrasts, but this can be inferred

Contrast 2: semantic decision vs visual decision

Language condition	Semantic decision
Control condition	Visual decision
Are the conditions matched for visual demands?	No
Are the conditions matched for auditory demands?	Yes
Are the conditions matched for motor demands?	Yes
Are the conditions matched for cognitive/executive demands?	No
Is accuracy matched between the language and control tasks for all relevant groups?	Unknown, not reported
Is reaction time matched between the language and control tasks for all relevant groups?	Unknown, not reported
Behavioral data notes	Accuracy not reported for control condition
Are control data reported in this paper or another that is referenced?	Yes
Does the contrast selectively activate plausible relevant language regions in the control group?	Somewhat
Are activations lateralized in the control data?	Somewhat
Control activation notes	—
Contrast notes	Not clearly stated that language tasks were contrasted only with arrow decision task and not rest for the first two contrasts, but this can be inferred

Contrast 3: verb generation vs rest

Language condition	Verb generation
Control condition	Rest
Are the conditions matched for visual demands?	No
Are the conditions matched for auditory demands?	Yes

Are the conditions matched for motor demands?	Yes
Are the conditions matched for cognitive/executive demands?	No
Is accuracy matched between the language and control tasks for all relevant groups?	N/A, tasks not comparable
Is reaction time matched between the language and control tasks for all relevant groups?	N/A, tasks not comparable
Behavioral data notes	—
Are control data reported in this paper or another that is referenced?	Yes
Does the contrast selectively activate plausible relevant language regions in the control group?	Somewhat
Are activations lateralized in the control data?	Somewhat
Control activation notes	—
Contrast notes	—

Analyses

Are the analyses clearly described?	No (see specific limitation(s) below)

ROI analysis 1

First level contrast	Written word-picture matching vs visual decision
Analysis class	Cross-sectional aphasia vs control
Group(s)	Aphasia vs control
Covariate	—
Is the second level contrast valid in terms of the group(s), time point(s), and measures involved?	Yes
Is accuracy matched across the second level contrast?	Unknown, not reported
Is reaction time matched across the second level contrast?	Unknown, not reported
Behavioral data notes	Accuracy not reported for control condition
Type of analysis	Regions of interest (ROI)
ROI type	Mixed
How many ROIs are there?	7
What are the ROI(s)?	(1) L anterior language region (IFG); (2) L posterior language region (AG, SMG, STG, MTG); (3) R anterior language region (IFG); (4) R posterior language region (AG, SMG, STG, MTG); (5) frontal LI; (6) temporal LI; (7) whole network LI
How are the ROI(s) defined?	WFU pickatlas
Correction for multiple comparisons	No correction
Statistical details	—
Findings	↓ L IFG<br>↓ LI (language network)<br>↓ LI (frontal)
Findings notes	—

ROI analysis 2

First level contrast	Semantic decision vs visual decision
Analysis class	Cross-sectional aphasia vs control
Group(s)	Aphasia vs control
Covariate	—
Is the second level contrast valid in terms of the group(s), time point(s), and measures involved?	Yes
Is accuracy matched across the second level contrast?	Unknown, not reported
Is reaction time matched across the second level contrast?	Unknown, not reported

Behavioral data notes	Accuracy not reported for control condition
Type of analysis	Regions of interest (ROI)
ROI type	Mixed
How many ROIs are there?	7
What are the ROI(s)?	(1) L anterior language region (IFG); (2) L posterior language region (AG, SMG, STG, MTG); (3) R anterior language region (IFG); (4) R posterior language region (AG, SMG, STG, MTG); (5) frontal LI; (6) temporal LI; (7) whole network LI
How are the ROI(s) defined?	WFU pickatlas
Correction for multiple comparisons	No correction
Statistical details	—
Findings	↓ L IFG<br>↓ LI (language network)<br>↓ LI (frontal)
Findings notes	—

ROI analysis 3

First level contrast	Verb generation vs rest
Analysis class	Cross-sectional aphasia vs control
Group(s)	Aphasia vs control
Covariate	—
Is the second level contrast valid in terms of the group(s), time point(s), and measures involved?	Yes
Is accuracy matched across the second level contrast?	Unknown, not reported
Is reaction time matched across the second level contrast?	Unknown, not reported
Behavioral data notes	—
Type of analysis	Regions of interest (ROI)
ROI type	Mixed
How many ROIs are there?	7
What are the ROI(s)?	(1) L anterior language region (IFG); (2) L posterior language region (AG, SMG, STG, MTG); (3) R anterior language region (IFG); (4) R posterior language region (AG, SMG, STG, MTG); (5) frontal LI; (6) temporal LI; (7) whole network LI
How are the ROI(s) defined?	WFU pickatlas
Correction for multiple comparisons	No correction
Statistical details	—
Findings	↓ L IFG<br>↓ LI (language network)<br>↓ LI (frontal)
Findings notes	—

ROI analysis 4

First level contrast	Written word-picture matching vs visual decision
Analysis class	Cross-sectional correlation with language or other measure
Group(s)	Aphasia
Covariate	Picture-word matching accuracy
Is the second level contrast valid in terms of the group(s), time point(s), and measures involved?	Yes
Is accuracy matched across the second level contrast?	Accuracy is covariate
Is reaction time matched across the second level contrast?	Unknown, not reported
Behavioral data notes	—
Type of analysis	Regions of interest (ROI)
ROI type	Mixed
How many ROIs are there?	7

What are the ROI(s)?	(1) L anterior language region (IFG); (2) L posterior language region (AG, SMG, STG, MTG); (3) R anterior language region (IFG); (4) R posterior language region (AG, SMG, STG, MTG); (5) frontal LI; (6) temporal LI; (7) whole network LI
How are the ROI(s) defined?	WFU pickatlas
Correction for multiple comparisons	No correction
Statistical details	—
Findings	None
Findings notes	—

ROI analysis 5

First level contrast	Semantic decision vs visual decision
Analysis class	Cross-sectional correlation with language or other measure
Group(s)	Aphasia
Covariate	Semantic decision accuracy
Is the second level contrast valid in terms of the group(s), time point(s), and measures involved?	Yes
Is accuracy matched across the second level contrast?	Accuracy is covariate
Is reaction time matched across the second level contrast?	Unknown, not reported
Behavioral data notes	—
Type of analysis	Regions of interest (ROI)
ROI type	Mixed
How many ROIs are there?	7
What are the ROI(s)?	(1) L anterior language region (IFG); (2) L posterior language region (AG, SMG, STG, MTG); (3) R anterior language region (IFG); (4) R posterior language region (AG, SMG, STG, MTG); (5) frontal LI; (6) temporal LI; (7) whole network LI
How are the ROI(s) defined?	WFU pickatlas
Correction for multiple comparisons	No correction
Statistical details	—
Findings	None
Findings notes	—

ROI analysis 6

First level contrast	Written word-picture matching vs visual decision
Analysis class	Cross-sectional correlation with language or other measure
Group(s)	Aphasia
Covariate	Overall language measure
Is the second level contrast valid in terms of the group(s), time point(s), and measures involved?	Yes
Is accuracy matched across the second level contrast?	Unknown, not reported
Is reaction time matched across the second level contrast?	Unknown, not reported
Behavioral data notes	—
Type of analysis	Regions of interest (ROI)
ROI type	Mixed
How many ROIs are there?	7
What are the ROI(s)?	(1) L anterior language region (IFG); (2) L posterior language region (AG, SMG, STG, MTG); (3) R anterior language region (IFG); (4) R posterior language region (AG, SMG, STG, MTG); (5) frontal LI; (6) temporal LI; (7) whole network LI
How are the ROI(s) defined?	WFU pickatlas
Correction for multiple comparisons	No correction
Statistical details	—
Findings	None
Findings notes	—

ROI analysis 7

First level contrast	Semantic decision vs visual decision
Analysis class	Cross-sectional correlation with language or other measure
Group(s)	Aphasia
Covariate	Overall language measure
Is the second level contrast valid in terms of the group(s), time point(s), and measures involved?	Yes
Is accuracy matched across the second level contrast?	Unknown, not reported
Is reaction time matched across the second level contrast?	Unknown, not reported
Behavioral data notes	—
Type of analysis	Regions of interest (ROI)
ROI type	Mixed
How many ROIs are there?	7
What are the ROI(s)?	(1) L anterior language region (IFG); (2) L posterior language region (AG, SMG, STG, MTG); (3) R anterior language region (IFG); (4) R posterior language region (AG, SMG, STG, MTG); (5) frontal LI; (6) temporal LI; (7) whole network LI
How are the ROI(s) defined?	WFU pickatlas
Correction for multiple comparisons	No correction
Statistical details	Not clear if it was LI for whole language network
Findings	↑ LI (language network)
Findings notes	—

ROI analysis 8

First level contrast	Verb generation vs rest
Analysis class	Cross-sectional correlation with language or other measure
Group(s)	Aphasia
Covariate	Overall language measure
Is the second level contrast valid in terms of the group(s), time point(s), and measures involved?	Yes
Is accuracy matched across the second level contrast?	Unknown, not reported
Is reaction time matched across the second level contrast?	Unknown, not reported
Behavioral data notes	—
Type of analysis	Regions of interest (ROI)
ROI type	Mixed
How many ROIs are there?	7
What are the ROI(s)?	(1) L anterior language region (IFG); (2) L posterior language region (AG, SMG, STG, MTG); (3) R anterior language region (IFG); (4) R posterior language region (AG, SMG, STG, MTG); (5) frontal LI; (6) temporal LI; (7) whole network LI
How are the ROI(s) defined?	WFU pickatlas
Correction for multiple comparisons	No correction
Statistical details	—
Findings	None
Findings notes	—

ROI analysis 9

First level contrast	Written word-picture matching vs visual decision
Analysis class	Cross-sectional correlation with language or other measure
Group(s)	Aphasia
Covariate	Lesion volume
Is the second level contrast valid in terms of the group(s), time point(s), and measures involved?	Yes

Is accuracy matched across the second level contrast?	Unknown, not reported
Is reaction time matched across the second level contrast?	Unknown, not reported
Behavioral data notes	—
Type of analysis	Regions of interest (ROI)
ROI type	Anatomical
How many ROIs are there?	2
What are the ROI(s)?	(1) R anterior language region (IFG); (2) R posterior language region (AG, SMG, STG, MTG)
How are the ROI(s) defined?	WFU pickatlas
Correction for multiple comparisons	No correction
Statistical details	—
Findings	None
Findings notes	—

ROI analysis 10

First level contrast	Semantic decision vs visual decision
Analysis class	Cross-sectional correlation with language or other measure
Group(s)	Aphasia
Covariate	Lesion volume
Is the second level contrast valid in terms of the group(s), time point(s), and measures involved?	Yes
Is accuracy matched across the second level contrast?	Unknown, not reported
Is reaction time matched across the second level contrast?	Unknown, not reported
Behavioral data notes	—
Type of analysis	Regions of interest (ROI)
ROI type	Anatomical
How many ROIs are there?	2
What are the ROI(s)?	(1) R anterior language region (IFG); (2) R posterior language region (AG, SMG, STG, MTG)
How are the ROI(s) defined?	WFU pickatlas
Correction for multiple comparisons	No correction
Statistical details	—
Findings	None
Findings notes	—

ROI analysis 11

First level contrast	Verb generation vs rest
Analysis class	Cross-sectional correlation with language or other measure
Group(s)	Aphasia
Covariate	Lesion volume
Is the second level contrast valid in terms of the group(s), time point(s), and measures involved?	Yes
Is accuracy matched across the second level contrast?	Unknown, not reported
Is reaction time matched across the second level contrast?	Unknown, not reported
Behavioral data notes	—
Type of analysis	Regions of interest (ROI)
ROI type	Anatomical
How many ROIs are there?	2
What are the ROI(s)?	(1) R anterior language region (IFG); (2) R posterior language region (AG, SMG, STG, MTG)
How are the ROI(s) defined?	WFU pickatlas
Correction for multiple comparisons	No correction

Statistical details	—
Findings	None
Findings notes	—

ROI analysis 12

First level contrast	Written word-picture matching vs visual decision
Analysis class	Cross-sectional correlation with language or other measure
Group(s)	Aphasia
Covariate	Damage to L hemisphere language regions
Is the second level contrast valid in terms of the group(s), time point(s), and measures involved?	Yes
Is accuracy matched across the second level contrast?	Unknown, not reported
Is reaction time matched across the second level contrast?	Unknown, not reported
Behavioral data notes	—
Type of analysis	Regions of interest (ROI)
ROI type	Anatomical
How many ROIs are there?	2
What are the ROI(s)?	(1) R anterior language region (IFG); (2) R posterior language region (AG, SMG, STG, MTG)
How are the ROI(s) defined?	WFU pickatlas
Correction for multiple comparisons	No correction
Statistical details	—
Findings	None
Findings notes	—

ROI analysis 13

First level contrast	Semantic decision vs visual decision
Analysis class	Cross-sectional correlation with language or other measure
Group(s)	Aphasia
Covariate	Damage to L hemisphere language regions
Is the second level contrast valid in terms of the group(s), time point(s), and measures involved?	Yes
Is accuracy matched across the second level contrast?	Unknown, not reported
Is reaction time matched across the second level contrast?	Unknown, not reported
Behavioral data notes	—
Type of analysis	Regions of interest (ROI)
ROI type	Anatomical
How many ROIs are there?	2
What are the ROI(s)?	(1) R anterior language region (IFG); (2) R posterior language region (AG, SMG, STG, MTG)
How are the ROI(s) defined?	WFU pickatlas
Correction for multiple comparisons	No correction
Statistical details	—
Findings	None
Findings notes	—

ROI analysis 14

First level contrast	Verb generation vs rest
Analysis class	Cross-sectional correlation with language or other measure
Group(s)	Aphasia
Covariate	Damage to L hemisphere language regions
Is the second level contrast valid in terms of the group(s), time point(s), and measures involved?	Yes

Is accuracy matched across the second level contrast?	Unknown, not reported
Is reaction time matched across the second level contrast?	Unknown, not reported
Behavioral data notes	—
Type of analysis	Regions of interest (ROI)
ROI type	Anatomical
How many ROIs are there?	2
What are the ROI(s)?	(1) R anterior language region (IFG); (2) R posterior language region (AG, SMG, STG, MTG)
How are the ROI(s) defined?	WFU pickatlas
Correction for multiple comparisons	No correction
Statistical details	—
Findings	None
Findings notes	—

ROI analysis 15

First level contrast	Written word-picture matching vs visual decision
Analysis class	Cross-sectional correlation with language or other measure
Group(s)	Aphasia
Covariate	Previous (current vs subacute) Δ naming
Is the second level contrast valid in terms of the group(s), time point(s), and measures involved?	No (current activation will reflect not just prior recovery, but also current language function)
Is accuracy matched across the second level contrast?	Unknown, not reported
Is reaction time matched across the second level contrast?	Unknown, not reported
Behavioral data notes	—
Type of analysis	Regions of interest (ROI)
ROI type	Anatomical
How many ROIs are there?	7
What are the ROI(s)?	(1) L anterior language region (IFG); (2) L posterior language region (AG, SMG, STG, MTG); (3) R anterior language region (IFG); (4) R posterior language region (AG, SMG, STG, MTG); (5) frontal LI; (6) temporal LI; (7) whole network LI
How are the ROI(s) defined?	WFU pickatlas
Correction for multiple comparisons	No correction
Statistical details	—
Findings	None
Findings notes	—

ROI analysis 16

First level contrast	Semantic decision vs visual decision
Analysis class	Cross-sectional correlation with language or other measure
Group(s)	Aphasia
Covariate	Previous (current vs subacute) Δ naming
Is the second level contrast valid in terms of the group(s), time point(s), and measures involved?	No (current activation will reflect not just prior recovery, but also current language function)
Is accuracy matched across the second level contrast?	Unknown, not reported
Is reaction time matched across the second level contrast?	Unknown, not reported
Behavioral data notes	—
Type of analysis	Regions of interest (ROI)
ROI type	Anatomical
How many ROIs are there?	7
What are the ROI(s)?	(1) L anterior language region (IFG); (2) L posterior language region (AG, SMG, STG, MTG); (3) R anterior language region (IFG); (4) R posterior language region (AG, SMG, STG, MTG); (5) frontal

	LI; (6) temporal LI; (7) whole network LI
How are the ROI(s) defined?	WFU pickatlas
Correction for multiple comparisons	No correction
Statistical details	—
Findings	↑ L IFG
Findings notes	—

ROI analysis 17

First level contrast	Verb generation vs rest
Analysis class	Cross-sectional correlation with language or other measure
Group(s)	Aphasia
Covariate	Previous (current vs subacute) Δ naming
Is the second level contrast valid in terms of the group(s), time point(s), and measures involved?	No (current activation will reflect not just prior recovery, but also current language function)
Is accuracy matched across the second level contrast?	Unknown, not reported
Is reaction time matched across the second level contrast?	Unknown, not reported
Behavioral data notes	—
Type of analysis	Regions of interest (ROI)
ROI type	Anatomical
How many ROIs are there?	7
What are the ROI(s)?	(1) L anterior language region (IFG); (2) L posterior language region (AG, SMG, STG, MTG); (3) R anterior language region (IFG); (4) R posterior language region (AG, SMG, STG, MTG); (5) frontal LI; (6) temporal LI; (7) whole network LI
How are the ROI(s) defined?	WFU pickatlas
Correction for multiple comparisons	No correction
Statistical details	—
Findings	↑ L IFG
Findings notes	—

ROI analysis 18

First level contrast	Written word-picture matching vs visual decision
Analysis class	Cross-sectional correlation with language or other measure
Group(s)	Aphasia
Covariate	Previous (current vs subacute) Δ TT
Is the second level contrast valid in terms of the group(s), time point(s), and measures involved?	No (current activation will reflect not just prior recovery, but also current language function; TT not optimal measure of overall language function)
Is accuracy matched across the second level contrast?	Unknown, not reported
Is reaction time matched across the second level contrast?	Unknown, not reported
Behavioral data notes	—
Type of analysis	Regions of interest (ROI)
ROI type	Anatomical
How many ROIs are there?	7
What are the ROI(s)?	(1) L anterior language region (IFG); (2) L posterior language region (AG, SMG, STG, MTG); (3) R anterior language region (IFG); (4) R posterior language region (AG, SMG, STG, MTG); (5) frontal LI; (6) temporal LI; (7) whole network LI
How are the ROI(s) defined?	WFU pickatlas
Correction for multiple comparisons	No correction
Statistical details	—
Findings	None
Findings notes	—

ROI analysis 19

First level contrast	Semantic decision vs visual decision
Analysis class	Cross-sectional correlation with language or other measure
Group(s)	Aphasia
Covariate	Previous (current vs subacute) Δ TT
Is the second level contrast valid in terms of the group(s), time point(s), and measures involved?	No (current activation will reflect not just prior recovery, but also current language function; TT not optimal measure of overall language function)
Is accuracy matched across the second level contrast?	Unknown, not reported
Is reaction time matched across the second level contrast?	Unknown, not reported
Behavioral data notes	—
Type of analysis	Regions of interest (ROI)
ROI type	Anatomical
How many ROIs are there?	7
What are the ROI(s)?	(1) L anterior language region (IFG); (2) L posterior language region (AG, SMG, STG, MTG); (3) R anterior language region (IFG); (4) R posterior language region (AG, SMG, STG, MTG); (5) frontal LI; (6) temporal LI; (7) whole network LI
How are the ROI(s) defined?	WFU pickatlas
Correction for multiple comparisons	No correction
Statistical details	—
Findings	↑ L IFG<br>↑ R IFG
Findings notes	—

ROI analysis 20

First level contrast	Verb generation vs rest
Analysis class	Cross-sectional correlation with language or other measure
Group(s)	Aphasia
Covariate	Previous (current vs subacute) Δ TT
Is the second level contrast valid in terms of the group(s), time point(s), and measures involved?	No (current activation will reflect not just prior recovery, but also current language function; TT not optimal measure of overall language function)
Is accuracy matched across the second level contrast?	Unknown, not reported
Is reaction time matched across the second level contrast?	Unknown, not reported
Behavioral data notes	—
Type of analysis	Regions of interest (ROI)
ROI type	Anatomical
How many ROIs are there?	7
What are the ROI(s)?	(1) L anterior language region (IFG); (2) L posterior language region (AG, SMG, STG, MTG); (3) R anterior language region (IFG); (4) R posterior language region (AG, SMG, STG, MTG); (5) frontal LI; (6) temporal LI; (7) whole network LI
How are the ROI(s) defined?	WFU pickatlas
Correction for multiple comparisons	No correction
Statistical details	—
Findings	↑ L IFG<br>↑ R IFG
Findings notes	—

Notes

Excluded analyses	—

Papoutsi et al. (2011)

Reference

Authors	Papoutsi M, Stamatakis EA, Griffiths J, Marslen-Wilson WD, Tyler LK
Title	Is left fronto-temporal connectivity essential for syntax? Effective connectivity, tractography and performance in left-hemisphere damaged patients
Reference	*NeuroImage* 2011; 58: 656-664
PMID	21722742
DOI	10.1016/j.neuroimage.2011.06.036

Participants

Language	UK English
Inclusion criteria	—
Number of individuals with aphasia	14
Number of control participants	15
Were any of the participants included in any previous studies?	Yes (reanalysis of same dataset from Tyler et al. (2011))
Is age reported for patients and controls, and matched?	Yes (mean 56 ± 12 years, range 35-77 years)
Is sex reported for patients and controls, and matched?	Yes (males: 11; females: 3)
Is handedness reported for patients and controls, and matched?	Yes (right: 14; left: 0)
Is time post stroke onset reported and appropriate to the study design?	Yes (mean 8 ± 9 years, range 2-40 years)
To what extent is the nature of aphasia characterized?	Not at all
Language evaluation	Sentence-picture matching, grammaticality judgment, lexical decision, phonological discrimination, semantic categorization, sentence repetition, word repetition
Aphasia severity	Not stated
Aphasia type	Not stated
First stroke only?	Not stated
Stroke type	Not stated
To what extent is the lesion distribution characterized?	Lesion overlay
Lesion extent	Not stated
Lesion location	L MCA
Participants notes	1 patient had post-surgical haematoma rather than stroke (per Tyler et al., 2011)

Imaging

Modality	fMRI
Is the study cross-sectional or longitudinal?	Cross-sectional
If longitudinal, at what time point(s) were imaging data acquired?	—
If longitudinal, was there any intervention between the time points?	—
Is the scanner described?	Yes (Siemens Trio 3 Tesla)
Is the timing of stimulus presentation and image acquisition clearly described and appropriate?	No (length of stimuli not described)
Design type	Event-related
Total images acquired	1059
Are the imaging acquisition parameters, including coverage, adequately described and appropriate?	Yes (whole brain)
Is preprocessing and intrasubject coregistration adequately described and appropriate?	Yes
Is first level model fitting adequately described and appropriate?	No (lacks explanation of event durations)
Is intersubject normalization adequately described	Yes

and appropriate?	
Imaging notes	—

Conditions

Are the conditions clearly described?	Yes

Condition	Response type	Repetitions	All groups could do?	All individuals could do?
listening to unambiguous sentences ("unambiguous")	None	42	N/A	N/A
listening to ambiguous sentences with dominant resolution ("dominant")	None	42	N/A	N/A
listening to ambiguous sentences with subordinate resolution ("subordinate")	None	42	N/A	N/A
listening to filler sentences	None	126	N/A	N/A
listening to "musical rain"	None	42	N/A	N/A
rest	None	implicit baseline	N/A	N/A

Conditions notes	—

Contrasts

Are the contrasts clearly described?	Yes

Contrast 1: listening to ambiguous sentences with subordinate resolution ("subordinate") vs listening to ambiguous sentences with dominant resolution ("dominant")

Language condition	Listening to ambiguous sentences with subordinate resolution ("subordinate")
Control condition	Listening to ambiguous sentences with dominant resolution ("dominant")
Are the conditions matched for visual demands?	Yes
Are the conditions matched for auditory demands?	Yes
Are the conditions matched for motor demands?	Yes
Are the conditions matched for cognitive/executive demands?	Yes
Is accuracy matched between the language and control tasks for all relevant groups?	N/A, no behavioral measure
Is reaction time matched between the language and control tasks for all relevant groups?	N/A, no timeable task
Behavioral data notes	—
Are control data reported in this paper or another that is referenced?	Yes
Does the contrast selectively activate plausible relevant language regions in the control group?	Yes
Are activations lateralized in the control data?	Yes
Control activation notes	Control data in Tyler et al. (2011); L frontal and temporal
Contrast notes	—

Analyses

Are the analyses clearly described?	Yes

Voxelwise analysis 1

First level contrast	Listening to ambiguous sentences with subordinate resolution ("subordinate") vs listening to ambiguous sentences with dominant resolution ("dominant")
Analysis class	Cross-sectional correlation with language or other measure
Group(s)	Aphasia
Covariate	Difference in percent of unacceptable judgments between subordinate and dominant sentences (dominance effect)
Is the second level contrast valid in terms of the	Yes

group(s), time point(s), and measures involved?	
Is accuracy matched across the second level contrast?	N/A, no behavioral measure
Is reaction time matched across the second level contrast?	N/A, no timeable task
Behavioral data notes	—
Type of analysis	Voxelwise
Search volume	Whole brain
Correction for multiple comparisons	Clusterwise correction with with GRFT and lenient voxelwise p
Software	SPM8
Voxelwise p	.01
Cluster extent	Based on GRFT
Statistical details	—
Findings	↑ L insula<br>↑ L posterior STG/STS/MTG<br>↑ L mid temporal
Findings notes	—

Complex analysis 1

First level contrast	Listening to ambiguous sentences with subordinate resolution ("subordinate") vs listening to ambiguous sentences with dominant resolution ("dominant")
Analysis class	Cross-sectional correlation with language or other measure
Group(s)	Aphasia
Covariate	Modulation of L IFG connectivity by dominance effect
Is the second level contrast valid in terms of the group(s), time point(s), and measures involved?	Yes
Is accuracy matched across the second level contrast?	N/A, no behavioral measure
Is reaction time matched across the second level contrast?	N/A, no timeable task
Behavioral data notes	—
Type of analysis	Complex
Statistical details	A PPI analysis was carried out with the L IFG as the seed region. Correlations were computed between voxelwise modulation of connectivity with this region, and a behavioral measure of syntactic processing, which was the dominance effect: the difference in percent of unacceptable judgments between subordinate and dominant sentences. The resultant SPM was thresholded at voxelwise p < .01 (CDT), then corrected for multiple corrections based on cluster extent and GRFT using SPM8.
Findings	Other
Findings notes	Patients with better syntactic performance had more connectivity from the L IFG seed region to L pMTG and adjacent areas (including the insula); pMTG also significant at voxelwise p < .001 in Figure 2B, corrected for multiple comparisons with GRFT

Complex analysis 2

First level contrast	Listening to ambiguous sentences with subordinate resolution ("subordinate") vs listening to ambiguous sentences with dominant resolution ("dominant")
Analysis class	Cross-sectional correlation with language or other measure
Group(s)	Aphasia
Covariate	Modulation of L pMTG connectivity by dominance effect
Is the second level contrast valid in terms of the group(s), time point(s), and measures involved?	Yes
Is accuracy matched across the second level contrast?	N/A, no behavioral measure
Is reaction time matched across the second level contrast?	N/A, no timeable task
Behavioral data notes	—
Type of analysis	Complex

Statistical details	A similar PPI analysis was carried out with the L pMTG as the seed region. Thresholding was the same as in the previous analysis.
Findings	None
Findings notes	—

Notes

Excluded analyses	—

Sebastian & Kiran (2011)

Reference

Authors	Sebastian R, Kiran S.
Title	Task-modulated neural activation patterns in chronic stroke patients with aphasia
Reference	*Aphasiology* 2011; 25: 927-951
PMID	N/A
DOI	10.1080/02687038.2011.557436

Participants

Language	US English
Inclusion criteria	—
Number of individuals with aphasia	8
Number of control participants	8
Were any of the participants included in any previous studies?	No
Is age reported for patients and controls, and matched?	Yes (range 40-79 years)
Is sex reported for patients and controls, and matched?	No (males: 5; females: 3; control sex not stated, but reported to be matched)
Is handedness reported for patients and controls, and matched?	Yes (right: 8; left: 0)
Is time post stroke onset reported and appropriate to the study design?	Yes (mean 48.3 months, range 30-78 months)
To what extent is the nature of aphasia characterized?	Comprehensive battery
Language evaluation	WAB, BNT, portions of PALPA, PPT, CLQT
Aphasia severity	AQ range 74.0-97.8
Aphasia type	6 anomic, 2 recovered
First stroke only?	Not stated
Stroke type	Mixed etiologies
To what extent is the lesion distribution characterized?	Individual lesions
Lesion extent	Range 23-45 cc
Lesion location	L MCA
Participants notes	—

Imaging

Modality	fMRI
Is the study cross-sectional or longitudinal?	Cross-sectional
If longitudinal, at what time point(s) were imaging data acquired?	—
If longitudinal, was there any intervention between the time points?	—
Is the scanner described?	No (GE 3 Tesla; model not stated)

Is the timing of stimulus presentation and image acquisition clearly described and appropriate?	No* (moderate limitation) (control events took place in the inter-trial interval between language events, and may have been systematically confounded in timing; the total number of functional images acquired is not stated)
Design type	Event-related
Total images acquired	not stated
Are the imaging acquisition parameters, including coverage, adequately described and appropriate?	Yes (whole brain)
Is preprocessing and intrasubject coregistration adequately described and appropriate?	Yes
Is first level model fitting adequately described and appropriate?	No (only correct trials are included but it is not stated how incorrect trials were modeled; in general, it is not stated whether the control events were modeled at all)
Is intersubject normalization adequately described and appropriate?	Yes
Imaging notes	—

Conditions

Are the conditions clearly described?	Yes

Condition	Response type	Repetitions	All groups could do?	All individuals could do?
picture naming	Word (overt)	60	Yes	Yes
viewing scrambled images and saying "pass"	Word (overt)	60	Unknown	Unknown
semantic decision	Button press	48	Yes	Yes
visual decision	Button press	48	Unknown	Unknown

Conditions notes	—

Contrasts

Are the contrasts clearly described?	Yes

Contrast 1: picture naming (correct trials) vs viewing scrambled images and saying "pass"

Language condition	Picture naming (correct trials)
Control condition	Viewing scrambled images and saying "pass"
Are the conditions matched for visual demands?	Yes
Are the conditions matched for auditory demands?	Yes
Are the conditions matched for motor demands?	Yes
Are the conditions matched for cognitive/executive demands?	No
Is accuracy matched between the language and control tasks for all relevant groups?	Unknown, not reported
Is reaction time matched between the language and control tasks for all relevant groups?	Unknown, not reported
Behavioral data notes	Accuracy/RT not reported for control task
Are control data reported in this paper or another that is referenced?	Somewhat
Does the contrast selectively activate plausible relevant language regions in the control group?	Somewhat
Are activations lateralized in the control data?	No
Control activation notes	Reporting is selective, but appears mostly bilateral with slight L-lateralization of language areas
Contrast notes	—

Contrast 2: semantic decision (correct trials) vs visual decision

Language condition	Semantic decision (correct trials)
Control condition	Visual decision
Are the conditions matched for visual demands?	Yes
Are the conditions matched for auditory demands?	Yes

Are the conditions matched for motor demands?	Yes
Are the conditions matched for cognitive/executive demands?	Yes
Is accuracy matched between the language and control tasks for all relevant groups?	Unknown, not reported
Is reaction time matched between the language and control tasks for all relevant groups?	Unknown, not reported
Behavioral data notes	Accuracy/RT not reported for control task
Are control data reported in this paper or another that is referenced?	Somewhat
Does the contrast selectively activate plausible relevant language regions in the control group?	Somewhat
Are activations lateralized in the control data?	Yes
Control activation notes	Clearly lateralized frontal activation, but very modest temporal activation
Contrast notes	—

Analyses

Are the analyses clearly described?	Yes

ROI analysis 1

First level contrast	Picture naming (correct trials) vs viewing scrambled images and saying "pass"
Analysis class	Cross-sectional correlation with language or other measure
Group(s)	Aphasia
Covariate	Lesion volume
Is the second level contrast valid in terms of the group(s), time point(s), and measures involved?	Yes
Is accuracy matched across the second level contrast?	Yes, correct trials only
Is reaction time matched across the second level contrast?	Unknown, not reported
Behavioral data notes	—
Type of analysis	Regions of interest (ROI)
ROI type	Mixed
How many ROIs are there?	4
What are the ROI(s)?	(1) L IFG (oper/tri); (2) L posterior perisylvian (pSTG, pMTG, AG, SMG); (3) R IFG (oper/tri); (4) R posterior perisylvian (pSTG, pMTG, AG, SMG); (5) language network LI
How are the ROI(s) defined?	Harvard–Oxford atlas
Correction for multiple comparisons	No correction
Statistical details	—
Findings	↑ R supramarginal gyrus<br>↑ R angular gyrus<br>↑ R posterior STG/STS/MTG<br>↓ LI (language network)
Findings notes	Larger lesions were associated with more R posterior perisylvian activation

ROI analysis 2

First level contrast	Semantic decision (correct trials) vs visual decision
Analysis class	Cross-sectional correlation with language or other measure
Group(s)	Aphasia
Covariate	Lesion volume
Is the second level contrast valid in terms of the group(s), time point(s), and measures involved?	Yes
Is accuracy matched across the second level contrast?	Yes, correct trials only
Is reaction time matched across the second level contrast?	Unknown, not reported

Behavioral data notes	—
Type of analysis	Regions of interest (ROI)
ROI type	Mixed
How many ROIs are there?	4
What are the ROI(s)?	(1) L IFG (oper/tri); (2) L posterior perisylvian (pSTG, pMTG, AG, SMG); (3) R IFG (oper/tri); (4) R posterior perisylvian (pSTG, pMTG, AG, SMG); (5) language network LI
How are the ROI(s) defined?	Harvard–Oxford atlas
Correction for multiple comparisons	No correction
Statistical details	—
Findings	None
Findings notes	—

Notes

Excluded analyses	(1) individual patient analyses; (2) comparisons between the two language tasks

Szaflarski et al. (2011)

Reference

Authors	Szaflarski JP, Vannest J, Wu SW, DiFrancesco MW, Banks C, Gilbert DL
Title	Excitatory repetitive transcranial magnetic stimulation induces improvements in chronic post-stroke aphasia
Reference	*Med Sci Monit* 2011; 17: CR132-139
PMID	21358599
DOI	10.12659/msm.881446

Participants

Language	US English
Inclusion criteria	Moderate aphasia, L MCA
Number of individuals with aphasia	8 (plus 3 excluded: 2 metallic artifact; 1 seizure at time of stroke)
Number of control participants	0
Were any of the participants included in any previous studies?	No
Is age reported for patients and controls, and matched?	Yes (mean 54.4 ± 12.7 years)
Is sex reported for patients and controls, and matched?	Yes (males: 4; females: 4)
Is handedness reported for patients and controls, and matched?	Yes (right: 8; left: 0)
Is time post stroke onset reported and appropriate to the study design?	Yes (mean 5.3 ± 3.6 years, > 12 months)
To what extent is the nature of aphasia characterized?	Severity and type
Language evaluation	BNT; phonemic fluency, semantic fluency, complex ideation from BDAE, PPVT, communicative activities log
Aphasia severity	Moderate
Aphasia type	4 Broca's, 3 anomic, 1 anomic/conduction
First stroke only?	Not stated
Stroke type	Not stated
To what extent is the lesion distribution characterized?	Individual lesions
Lesion extent	Not stated
Lesion location	L MCA
Participants notes	—

Imaging

Modality	fMRI
Is the study cross-sectional or longitudinal?	Longitudinal—chronic treatment
If longitudinal, at what time point(s) were imaging data acquired?	T1: pre-treatment/chronic; T2: post-treatment, ~2 weeks later
If longitudinal, was there any intervention between the time points?	RTMS to residual activation near Broca's area, 5 sessions/week, 2 weeks
Is the scanner described?	Yes (Varian Unity INOVA 4 T)
Is the timing of stimulus presentation and image acquisition clearly described and appropriate?	No (timing not clear, because previous studies cited are not all identical in terms of timing)
Design type	Block
Total images acquired	not stated
Are the imaging acquisition parameters, including coverage, adequately described and appropriate?	Yes (whole brain)
Is preprocessing and intrasubject coregistration adequately described and appropriate?	Yes
Is first level model fitting adequately described and appropriate?	Yes
Is intersubject normalization adequately described and appropriate?	No (lesion impact not addressed)
Imaging notes	—

Conditions

Are the conditions clearly described?	No (based on Binder et al. (1997), but details not reported)

Condition	Response type	Repetitions	All groups could do?	All individuals could do?
semantic decision	Button press	not stated	Unknown	No
tone decision	Button press	not stated	Unknown	No

Conditions notes	Group only just above chance, unclear whether significantly better; clearly some individuals were at chance

Contrasts

Are the contrasts clearly described?	Yes

Contrast 1: semantic decision vs tone decision

Language condition	Semantic decision
Control condition	Tone decision
Are the conditions matched for visual demands?	Yes
Are the conditions matched for auditory demands?	Yes
Are the conditions matched for motor demands?	Yes
Are the conditions matched for cognitive/executive demands?	Yes
Is accuracy matched between the language and control tasks for all relevant groups?	Appear similar
Is reaction time matched between the language and control tasks for all relevant groups?	Unknown, not reported
Behavioral data notes	—
Are control data reported in this paper or another that is referenced?	Yes
Does the contrast selectively activate plausible relevant language regions in the control group?	Yes
Are activations lateralized in the control data?	Yes
Control activation notes	Control data in Kim et al. (2011) and Szaflarski et al. (2008); L frontal and temporal, plus other semantic regions
Contrast notes	—

Analyses

Are the analyses clearly described?	No* (moderate limitation) (see specific limitation(s) below)

Voxelwise analysis 1

First level contrast	Semantic decision vs tone decision
Analysis class	Longitudinal change in aphasia
Group(s)	Aphasia T2 vs T1
Covariate	—
Is the second level contrast valid in terms of the group(s), time point(s), and measures involved?	Somewhat (patients improved only on semantic fluency)
Is accuracy matched across the second level contrast?	Yes, matched
Is reaction time matched across the second level contrast?	Unknown, not reported
Behavioral data notes	Language and control tasks both matched
Type of analysis	Voxelwise
Search volume	Whole brain
Correction for multiple comparisons	No correction
Software	in-house
Voxelwise p	.05
Cluster extent	None
Statistical details	The figure shows a cutoff of z > 10, which would not correspond to p < .05; increases and decreases in Figure 3 do not accord with the data from T1 and T2 in Figure 2, raising concerns about the implementation of the analyses; there is no explicit description of the second level analysis
Findings	↑ L IFG<br>↑ L SMA/medial prefrontal<br>↑ L orbitofrontal<br>↑ L inferior parietal lobule<br>↑ L supramarginal gyrus<br>↑ L angular gyrus<br>↑ L precuneus<br>↑ L occipital<br>↑ L anterior cingulate<br>↑ L basal ganglia<br>↑ L hippocampus/MTL<br>↑ R dorsal precentral<br>↑ R precuneus<br>↑ R occipital<br>↑ R basal ganglia<br>↑ R hippocampus/MTL<br>↓ R insula<br>↓ R supramarginal gyrus<br>↓ R posterior STG
Findings notes	Based on a combination of coordinates in Table 2, and Figure 3

ROI analysis 1

First level contrast	Semantic decision vs tone decision
Analysis class	Longitudinal change in aphasia
Group(s)	Aphasia T2 vs T1
Covariate	—
Is the second level contrast valid in terms of the group(s), time point(s), and measures involved?	Somewhat (patients improved only on semantic fluency)
Is accuracy matched across the second level contrast?	Yes, matched
Is reaction time matched across the second level contrast?	Unknown, not reported

Behavioral data notes	Language and control tasks both matched
Type of analysis	Regions of interest (ROI)
ROI type	Functional
How many ROIs are there?	3
What are the ROI(s)?	(1) frontal LI; (2) temporal LI; (3) language network LI
How are the ROI(s) defined?	
Correction for multiple comparisons	No correction
Statistical details	T1 LI (temporal) is reported to be negative, which does not accord with the voxelwise analysis in Figure 2; increases and decreases in Figure 3 do not accord with the data from T1 and T2 in Figure 2, raising concerns about the implementation of the analyses
Findings	↑ LI (language network)<br>↑ LI (frontal)<br>↑ LI (temporal)
Findings notes	—

Notes

Excluded analyses	—

Tyler et al. (2011)

Reference

Authors	Tyler LK, Marslen-Wilson WD, Randall B, Wright P, Devereux BJ, Zhuang J, Papoutsi M, Stamatakis EA
Title	Left inferior frontal cortex and syntax: function, structure and behaviour in patients with left hemisphere damage
Reference	*Brain* 2011; 134: 415-431
PMID	21278407
DOI	10.1093/brain/awq369

Participants

Language	UK English
Inclusion criteria	—
Number of individuals with aphasia	14
Number of control participants	15
Were any of the participants included in any previous studies?	Yes (not stated, but it seems like most of the patients also participated in Tyler et al. (2010))
Is age reported for patients and controls, and matched?	Yes (mean 56 years, range 34-77 years)
Is sex reported for patients and controls, and matched?	Yes (males: 11; females: 3)
Is handedness reported for patients and controls, and matched?	Yes (right: 14; left: 0)
Is time post stroke onset reported and appropriate to the study design?	Yes (mean 7 years, > 1.5 years)
To what extent is the nature of aphasia characterized?	Not at all
Language evaluation	Sentence-picture matching, grammaticality judgment, lexical decision, phonological discrimination, semantic categorization, sentence repetition, word repetition
Aphasia severity	Not stated
Aphasia type	Not stated
First stroke only?	Not stated
Stroke type	Not stated
To what extent is the lesion distribution	Lesion overlay

characterized?	
Lesion extent	Not stated
Lesion location	L MCA
Participants notes	1 patient had post-surgical haematoma rather than stroke

Imaging

Modality	fMRI
Is the study cross-sectional or longitudinal?	Cross-sectional
If longitudinal, at what time point(s) were imaging data acquired?	—
If longitudinal, was there any intervention between the time points?	—
Is the scanner described?	Yes (Siemens Trio 3 Tesla)
Is the timing of stimulus presentation and image acquisition clearly described and appropriate?	No (run length not stated; length of stimuli not described)
Design type	Event-related
Total images acquired	not stated but 1059 per Papoutsi et al. (2011)
Are the imaging acquisition parameters, including coverage, adequately described and appropriate?	Yes (whole brain)
Is preprocessing and intrasubject coregistration adequately described and appropriate?	Yes
Is first level model fitting adequately described and appropriate?	No (lacks explanation of event durations)
Is intersubject normalization adequately described and appropriate?	Yes
Imaging notes	—

Conditions

Are the conditions clearly described?	Yes

Condition	Response type	Repetitions	All groups could do?	All individuals could do?
listening to unambiguous sentences ("unambiguous")	None	42	N/A	N/A
listening to ambiguous sentences with dominant resolution ("dominant")	None	42	N/A	N/A
listening to ambiguous sentences with subordinate resolution ("subordinate")	None	42	N/A	N/A
listening to filler sentences	None	126	N/A	N/A
listening to "musical rain"	None	42	N/A	N/A
rest	None	implicit baseline	N/A	N/A

Conditions notes	—

Contrasts

Are the contrasts clearly described?	Yes

Contrast 1: listening to ambiguous sentences (dominant and subordinate) vs listening to unambiguous sentences ("unambiguous")

Language condition	Listening to ambiguous sentences (dominant and subordinate)
Control condition	Listening to unambiguous sentences ("unambiguous")
Are the conditions matched for visual demands?	Yes
Are the conditions matched for auditory demands?	Yes
Are the conditions matched for motor demands?	Yes
Are the conditions matched for cognitive/executive demands?	Yes
Is accuracy matched between the language and	N/A, no behavioral measure

control tasks for all relevant groups?	
Is reaction time matched between the language and control tasks for all relevant groups?	N/A, no timeable task
Behavioral data notes	—
Are control data reported in this paper or another that is referenced?	Yes
Does the contrast selectively activate plausible relevant language regions in the control group?	Somewhat
Are activations lateralized in the control data?	Yes
Control activation notes	L frontal and parietal; R frontal (but L > R); no L temporal
Contrast notes	—

Contrast 2: listening to ambiguous sentences with dominant resolution ("dominant") vs listening to unambiguous sentences ("unambiguous")

Language condition	Listening to ambiguous sentences with dominant resolution ("dominant")
Control condition	Listening to unambiguous sentences ("unambiguous")
Are the conditions matched for visual demands?	Yes
Are the conditions matched for auditory demands?	Yes
Are the conditions matched for motor demands?	Yes
Are the conditions matched for cognitive/executive demands?	Yes
Is accuracy matched between the language and control tasks for all relevant groups?	N/A, no behavioral measure
Is reaction time matched between the language and control tasks for all relevant groups?	N/A, no timeable task
Behavioral data notes	—
Are control data reported in this paper or another that is referenced?	Yes
Does the contrast selectively activate plausible relevant language regions in the control group?	Somewhat
Are activations lateralized in the control data?	Yes
Control activation notes	L frontal and parietal; no L temporal
Contrast notes	—

Contrast 3: listening to ambiguous sentences with subordinate resolution ("subordinate") vs listening to unambiguous sentences ("unambiguous")

Language condition	Listening to ambiguous sentences with subordinate resolution ("subordinate")
Control condition	Listening to unambiguous sentences ("unambiguous")
Are the conditions matched for visual demands?	Yes
Are the conditions matched for auditory demands?	Yes
Are the conditions matched for motor demands?	Yes
Are the conditions matched for cognitive/executive demands?	Yes
Is accuracy matched between the language and control tasks for all relevant groups?	N/A, no behavioral measure
Is reaction time matched between the language and control tasks for all relevant groups?	N/A, no timeable task
Behavioral data notes	—
Are control data reported in this paper or another that is referenced?	Yes
Does the contrast selectively activate plausible relevant language regions in the control group?	Yes
Are activations lateralized in the control data?	Yes
Control activation notes	L frontal, temporal and parietal, R frontal (but L > R)
Contrast notes	—

Contrast 4: listening to ambiguous sentences with subordinate resolution ("subordinate") vs listening to ambiguous sentences with dominant resolution ("dominant")

Language condition	Listening to ambiguous sentences with subordinate resolution ("subordinate")
Control condition	Listening to ambiguous sentences with dominant resolution ("dominant")
Are the conditions matched for visual demands?	Yes
Are the conditions matched for auditory demands?	Yes
Are the conditions matched for motor demands?	Yes
Are the conditions matched for cognitive/executive demands?	Yes
Is accuracy matched between the language and control tasks for all relevant groups?	N/A, no behavioral measure
Is reaction time matched between the language and control tasks for all relevant groups?	N/A, no timeable task
Behavioral data notes	—
Are control data reported in this paper or another that is referenced?	Yes
Does the contrast selectively activate plausible relevant language regions in the control group?	Yes
Are activations lateralized in the control data?	Yes
Control activation notes	L frontal and temporal
Contrast notes	—

Analyses

Are the analyses clearly described?	Yes

Voxelwise analysis 1

First level contrast	Listening to ambiguous sentences (dominant and subordinate) vs listening to unambiguous sentences ("unambiguous")
Analysis class	Cross-sectional aphasia vs control
Group(s)	Aphasia vs control
Covariate	—
Is the second level contrast valid in terms of the group(s), time point(s), and measures involved?	Yes
Is accuracy matched across the second level contrast?	N/A, no behavioral measure
Is reaction time matched across the second level contrast?	N/A, no timeable task
Behavioral data notes	—
Type of analysis	Voxelwise
Search volume	Plausible fronto-temporo-parietal language regions
Correction for multiple comparisons	No direct comparison
Software	SPM5
Voxelwise p	—
Cluster extent	—
Statistical details	Qualitative comparison on p. 423
Findings	↓ L IFG
Findings notes	—

Voxelwise analysis 2

First level contrast	Listening to ambiguous sentences with dominant resolution ("dominant") vs listening to unambiguous sentences ("unambiguous")
Analysis class	Cross-sectional aphasia vs control
Group(s)	Aphasia vs control
Covariate	—
Is the second level contrast valid in terms of the	Yes

group(s), time point(s), and measures involved?	
Is accuracy matched across the second level contrast?	N/A, no behavioral measure
Is reaction time matched across the second level contrast?	N/A, no timeable task
Behavioral data notes	—
Type of analysis	Voxelwise
Search volume	Plausible fronto-temporo-parietal language regions
Correction for multiple comparisons	No direct comparison
Software	SPM5
Voxelwise p	—
Cluster extent	—
Statistical details	Qualitative comparison on p. 423
Findings	↓ L IFG
Findings notes	—

Voxelwise analysis 3

First level contrast	Listening to ambiguous sentences with subordinate resolution ("subordinate") vs listening to unambiguous sentences ("unambiguous")
Analysis class	Cross-sectional aphasia vs control
Group(s)	Aphasia vs control
Covariate	—
Is the second level contrast valid in terms of the group(s), time point(s), and measures involved?	Yes
Is accuracy matched across the second level contrast?	N/A, no behavioral measure
Is reaction time matched across the second level contrast?	N/A, no timeable task
Behavioral data notes	—
Type of analysis	Voxelwise
Search volume	Plausible fronto-temporo-parietal language regions
Correction for multiple comparisons	No direct comparison
Software	SPM5
Voxelwise p	—
Cluster extent	—
Statistical details	Qualitative comparison on p. 423
Findings	↓ L IFG
Findings notes	Lack of patient activation in pMTG implied in text, but this activation looks fairly similar in patients and controls (c.f. Figure 3C vs 2C)

Voxelwise analysis 4

First level contrast	Listening to ambiguous sentences with subordinate resolution ("subordinate") vs listening to ambiguous sentences with dominant resolution ("dominant")
Analysis class	Cross-sectional aphasia vs control
Group(s)	Aphasia vs control
Covariate	—
Is the second level contrast valid in terms of the group(s), time point(s), and measures involved?	Yes
Is accuracy matched across the second level contrast?	N/A, no behavioral measure
Is reaction time matched across the second level contrast?	N/A, no timeable task
Behavioral data notes	—
Type of analysis	Voxelwise
Search volume	Plausible fronto-temporo-parietal language regions
Correction for multiple comparisons	No direct comparison

Software	SPM5
Voxelwise p	—
Cluster extent	—
Statistical details	Qualitative comparison on p. 423
Findings	↓ L IFG<br>↓ L posterior MTG
Findings notes	—

Voxelwise analysis 5

First level contrast	Listening to ambiguous sentences (dominant and subordinate) vs listening to unambiguous sentences ("unambiguous")
Analysis class	Cross-sectional correlation with language or other measure
Group(s)	Aphasia
Covariate	Performance on acceptability judgment task (difference in percent of unacceptable judgments between ambiguous and unambiguous sentences)
Is the second level contrast valid in terms of the group(s), time point(s), and measures involved?	Yes
Is accuracy matched across the second level contrast?	N/A, no behavioral measure
Is reaction time matched across the second level contrast?	N/A, no timeable task
Behavioral data notes	—
Type of analysis	Voxelwise
Search volume	Plausible fronto-temporo-parietal language regions
Correction for multiple comparisons	Clusterwise correction with with GRFT and lenient voxelwise p
Software	SPM5
Voxelwise p	.01
Cluster extent	Based on GRFT
Statistical details	—
Findings	↑ L IFG pars triangularis<br>↑ L IFG pars orbitalis<br>↑ R insula<br>↑ R mid temporal
Findings notes	Also L pMTG but this did not reach significance

Voxelwise analysis 6

First level contrast	Listening to ambiguous sentences (dominant and subordinate) vs listening to unambiguous sentences ("unambiguous")
Analysis class	Cross-sectional correlation with language or other measure
Group(s)	Aphasia
Covariate	Performance on sentence-picture matching task
Is the second level contrast valid in terms of the group(s), time point(s), and measures involved?	Yes
Is accuracy matched across the second level contrast?	N/A, no behavioral measure
Is reaction time matched across the second level contrast?	N/A, no timeable task
Behavioral data notes	—
Type of analysis	Voxelwise
Search volume	Plausible fronto-temporo-parietal language regions
Correction for multiple comparisons	Clusterwise correction based on arbitrary cluster extent
Software	SPM5
Voxelwise p	.01
Cluster extent	30 (units not stated)
Statistical details	—
Findings	↑ L IFG pars orbitalis

	↑ L posterior MTG<br>↑ R insula<br>↑ R posterior STG<br>↑ R mid temporal
Findings notes	—

Voxelwise analysis 7

First level contrast	Listening to ambiguous sentences (dominant and subordinate) vs listening to unambiguous sentences ("unambiguous")
Analysis class	Cross-sectional correlation with language or other measure
Group(s)	Aphasia
Covariate	Performance on word monitoring task
Is the second level contrast valid in terms of the group(s), time point(s), and measures involved?	Yes
Is accuracy matched across the second level contrast?	N/A, no behavioral measure
Is reaction time matched across the second level contrast?	N/A, no timeable task
Behavioral data notes	—
Type of analysis	Voxelwise
Search volume	Plausible fronto-temporo-parietal language regions
Correction for multiple comparisons	Clusterwise correction based on arbitrary cluster extent
Software	SPM5
Voxelwise p	.05
Cluster extent	10 (units not stated)
Statistical details	—
Findings	↑ L IFG pars orbitalis<br>↑ L posterior MTG<br>↑ R insula<br>↑ R mid temporal
Findings notes	—

Voxelwise analysis 8

First level contrast	Listening to ambiguous sentences (dominant and subordinate) vs listening to unambiguous sentences ("unambiguous")
Analysis class	Cross-sectional correlation with language or other measure
Group(s)	Aphasia
Covariate	Difference in percent of unacceptable judgments between subordinate and dominant sentences (dominance effect)
Is the second level contrast valid in terms of the group(s), time point(s), and measures involved?	Yes
Is accuracy matched across the second level contrast?	N/A, no behavioral measure
Is reaction time matched across the second level contrast?	N/A, no timeable task
Behavioral data notes	—
Type of analysis	Voxelwise
Search volume	Plausible fronto-temporo-parietal language regions
Correction for multiple comparisons	Clusterwise correction with with GRFT and lenient voxelwise p
Software	SPM5
Voxelwise p	.01
Cluster extent	Based on GRFT
Statistical details	—
Findings	None
Findings notes	—

ROI analysis 1

First level contrast	Listening to ambiguous sentences (dominant and subordinate) vs listening to unambiguous sentences ("unambiguous")
Analysis class	Cross-sectional correlation with language or other measure
Group(s)	Aphasia
Covariate	Performance on acceptability judgment task (difference in percent of unacceptable judgments between ambiguous and unambiguous sentences)
Is the second level contrast valid in terms of the group(s), time point(s), and measures involved?	Yes
Is accuracy matched across the second level contrast?	N/A, no behavioral measure
Is reaction time matched across the second level contrast?	N/A, no timeable task
Behavioral data notes	—
Type of analysis	Regions of interest (ROI)
ROI type	Anatomical
How many ROIs are there?	3
What are the ROI(s)?	(1) IFG pars opercularis; (2) IFG pars triangularis; (3) IFG pars orbitalis
How are the ROI(s) defined?	AAL
Correction for multiple comparisons	No correction
Statistical details	—
Findings	↑ L IFG pars triangularis<br>↑ L IFG pars orbitalis
Findings notes	—

ROI analysis 2

First level contrast	Listening to ambiguous sentences (dominant and subordinate) vs listening to unambiguous sentences ("unambiguous")
Analysis class	Cross-sectional correlation with language or other measure
Group(s)	Aphasia
Covariate	Difference in percentage of unacceptable judgments between subordinate and dominant sentences (dominance effect)
Is the second level contrast valid in terms of the group(s), time point(s), and measures involved?	Yes
Is accuracy matched across the second level contrast?	N/A, no behavioral measure
Is reaction time matched across the second level contrast?	N/A, no timeable task
Behavioral data notes	—
Type of analysis	Regions of interest (ROI)
ROI type	Anatomical
How many ROIs are there?	3
What are the ROI(s)?	(1) IFG pars opercularis; (2) IFG pars triangularis; (3) IFG pars orbitalis
How are the ROI(s) defined?	AAL
Correction for multiple comparisons	No correction
Statistical details	—
Findings	None
Findings notes	—

Notes

Excluded analyses	It is mentioned in the supplementary material that there was no correlation between activation and lexical (non-syntactic) errors

Weiduschat et al. (2011)

Reference

Authors	Weiduschat N, Thiel A, Rubi-Fessen I, Hartmann A, Kessler J, Merl P, Kracht L, Rommel T, Heiss WD
Title	Effects of repetitive transcranial magnetic stimulation in aphasic stroke: a randomized controlled pilot study
Reference	*Stroke* 2011; 42: 409-415
PMID	21164121
DOI	10.1161/strokeaha.110.597864

Participants

Language	German
Inclusion criteria	Age 55-85
Number of individuals with aphasia	10 (plus 4 excluded: 3 malfunction of TMS device or claustrophobia; 1 recovered nearly completely prior to intervention)
Number of control participants	0
Were any of the participants included in any previous studies?	No
Is age reported for patients and controls, and matched?	Yes (range 59-83 years)
Is sex reported for patients and controls, and matched?	Yes (males: 5; females: 5)
Is handedness reported for patients and controls, and matched?	Yes (right: 10; left: 0)
Is time post stroke onset reported and appropriate to the study design?	Yes (range 18-97 days; patients at different subacute stages of recovery)
To what extent is the nature of aphasia characterized?	Type only
Language evaluation	AAT
Aphasia severity	T1: TT range 0-45 errors; T2: TT range 0-44 errors
Aphasia type	T1: 5 Wernicke's, 2 Broca's, 2 global, 1 amnestic fluent; T2: not stated
First stroke only?	Yes
Stroke type	Not stated
To what extent is the lesion distribution characterized?	Extent and location
Lesion extent	Range 0.7-88.9 cc
Lesion location	L MCA
Participants notes	—

Imaging

Modality	PET (rCBF)
Is the study cross-sectional or longitudinal?	Longitudinal—mixed
If longitudinal, at what time point(s) were imaging data acquired?	T1: pre-treatment/subacute (range 18-97 days post onset); T2: post-treatment, ~2 weeks later
If longitudinal, was there any intervention between the time points?	Individualized SLT, 45 minutes/day, 5 days/week, 2 weeks; 6 patients underwent rTMS to the R IFG pars triangularis; 4 received vertex (sham) rTMS
Is the scanner described?	Yes (CTI-Siemens ECAT EXACT HR)
Is the timing of stimulus presentation and image acquisition clearly described and appropriate?	Yes
Design type	PET
Total images acquired	8
Are the imaging acquisition parameters, including coverage, adequately described and appropriate?	Yes (whole brain)
Is preprocessing and intrasubject coregistration adequately described and appropriate?	Yes

Is first level model fitting adequately described and appropriate?	Yes
Is intersubject normalization adequately described and appropriate?	Yes
Imaging notes	—

Conditions

Are the conditions clearly described?	Yes

Condition	Response type	Repetitions	All groups could do?	All individuals could do?
verb generation	Word (covert)	4	Unknown	Unknown
rest	None	4	N/A	N/A

Conditions notes	—

Contrasts

Are the contrasts clearly described?	Yes

Contrast 1: verb generation vs rest

Language condition	Verb generation
Control condition	Rest
Are the conditions matched for visual demands?	Yes
Are the conditions matched for auditory demands?	No
Are the conditions matched for motor demands?	Yes
Are the conditions matched for cognitive/executive demands?	No
Is accuracy matched between the language and control tasks for all relevant groups?	N/A, tasks not comparable
Is reaction time matched between the language and control tasks for all relevant groups?	N/A, tasks not comparable
Behavioral data notes	—
Are control data reported in this paper or another that is referenced?	Somewhat
Does the contrast selectively activate plausible relevant language regions in the control group?	Unknown
Are activations lateralized in the control data?	Unknown
Control activation notes	Control data in Herholz et al. (1996); insufficient to fully validate the contrast
Contrast notes	—

Analyses

Are the analyses clearly described?	Yes

ROI analysis 1

First level contrast	Verb generation vs rest
Analysis class	Longitudinal change in aphasia
Group(s)	Aphasia T2 vs T1 (regardless of rTMS)
Covariate	—
Is the second level contrast valid in terms of the group(s), time point(s), and measures involved?	Yes
Is accuracy matched across the second level contrast?	Unknown, not reported
Is reaction time matched across the second level contrast?	Unknown, not reported
Behavioral data notes	—
Type of analysis	Regions of interest (ROI)
ROI type	Laterality indi(ces)

How many ROIs are there?	3
What are the ROI(s)?	(1) IFG LI; (2) superior temporal LI; (3) SMA LI
How are the ROI(s) defined?	
Correction for multiple comparisons	No correction
Statistical details	—
Findings	None
Findings notes	—

ROI analysis 2

First level contrast	Verb generation vs rest
Analysis class	Longitudinal change in aphasia
Group(s)	Aphasia treated with rTMS (n = 6) T2 vs T1
Covariate	—
Is the second level contrast valid in terms of the group(s), time point(s), and measures involved?	Yes
Is accuracy matched across the second level contrast?	Unknown, not reported
Is reaction time matched across the second level contrast?	Unknown, not reported
Behavioral data notes	—
Type of analysis	Regions of interest (ROI)
ROI type	Laterality indi(ces)
How many ROIs are there?	3
What are the ROI(s)?	(1) IFG LI; (2) superior temporal LI; (3) SMA LI
How are the ROI(s) defined?	
Correction for multiple comparisons	No correction
Statistical details	—
Findings	None
Findings notes	—

ROI analysis 3

First level contrast	Verb generation vs rest
Analysis class	Longitudinal between two groups with aphasia
Group(s)	(Aphasia with R IFG rTMS (n = 6) T2 vs T1) vs (with sham rTMS (n = 4) T2 vs T1)
Covariate	—
Is the second level contrast valid in terms of the group(s), time point(s), and measures involved?	Yes
Is accuracy matched across the second level contrast?	Unknown, not reported
Is reaction time matched across the second level contrast?	Unknown, not reported
Behavioral data notes	—
Type of analysis	Regions of interest (ROI)
ROI type	Laterality indi(ces)
How many ROIs are there?	3
What are the ROI(s)?	(1) IFG LI; (2) superior temporal LI; (3) SMA LI
How are the ROI(s) defined?	
Correction for multiple comparisons	No correction
Statistical details	—
Findings	↑ LI (frontal)
Findings notes	IFG LI was stable in the stimulation group, but shifted to the R in the sham group, yielding a significant difference between groups

ROI analysis 4

First level contrast	Verb generation vs rest

Analysis class	Longitudinal correlation with language or other measure
Group(s)	Aphasia T2 vs T1 (regardless of rTMS)
Covariate	Δ AAT total score
Is the second level contrast valid in terms of the group(s), time point(s), and measures involved?	Yes
Is accuracy matched across the second level contrast?	Unknown, not reported
Is reaction time matched across the second level contrast?	Unknown, not reported
Behavioral data notes	—
Type of analysis	Region of interest (ROI)
ROI type	Laterality indi(ces)
How many ROIs are there?	1
What are the ROI(s)?	IFG LI
How are the ROI(s) defined?	
Correction for multiple comparisons	One only
Statistical details	—
Findings	None
Findings notes	—

Notes

Excluded analyses	(1) difference between groups at T1 (pre-treatment); (2) sham group T2 vs T1 (n = 4)

Allendorfer et al. (2012)

Reference

Authors	Allendorfer JB, Kissela BM, Holland SK, Szaflarski JP
Title	Different patterns of language activation in post-stroke aphasia are detected by overt and covert versions of the verb generation fMRI task
Reference	*Med Sci Monit* 2012; 18: CR135-147
PMID	22367124
DOI	10.12659/msm.882518

Participants

Language	US English
Inclusion criteria	MCA; moderate-severe aphasia; mRS ≤ 3
Number of individuals with aphasia	16
Number of control participants	32
Were any of the participants included in any previous studies?	Yes ("part of a larger ongoing study", may overlap with other studies from this group)
Is age reported for patients and controls, and matched?	Yes (mean 54.4 ± 9.5 years, range 38-78 years)
Is sex reported for patients and controls, and matched?	Yes (males: 9; females: 7)
Is handedness reported for patients and controls, and matched?	Yes (right: 16; left: 0)
Is time post stroke onset reported and appropriate to the study design?	Yes (mean 3.7 ± 3.5 years, range 0.5-11.4 years)
To what extent is the nature of aphasia characterized?	Severity and type
Language evaluation	TT, PPVT, BNT, semantic and phonemic fluency, complex ideation subtest of BDAE
Aphasia severity	Moderate-severe; TT mean 25.5 ± 11.3; unclear how to reconcile moderate-severe severity with mostly anomic aphasia

Aphasia type	Mostly anomic with some non-fluent
First stroke only?	Not stated
Stroke type	Ischemic only
To what extent is the lesion distribution characterized?	Individual lesions
Lesion extent	Range 2.8-248.9 cc
Lesion location	L MCA
Participants notes	—

Imaging

Modality	fMRI
Is the study cross-sectional or longitudinal?	Cross-sectional
If longitudinal, at what time point(s) were imaging data acquired?	—
If longitudinal, was there any intervention between the time points?	—
Is the scanner described?	No (Phillips 3 Tesla; model not stated)
Is the timing of stimulus presentation and image acquisition clearly described and appropriate?	Yes
Design type	Mixed
Total images acquired	435
Are the imaging acquisition parameters, including coverage, adequately described and appropriate?	Yes (whole brain)
Is preprocessing and intrasubject coregistration adequately described and appropriate?	Yes
Is first level model fitting adequately described and appropriate?	No (no description of HRF model, which is important given sparse sampling design)
Is intersubject normalization adequately described and appropriate?	No (lesion impact not addressed)
Imaging notes	sparse sampling

Conditions

Are the conditions clearly described?	Yes

Condition	Response type	Repetitions	All groups could do?	All individuals could do?
verb generation (overt, event-related)	Multiple words (overt)	15	Yes	Unknown
verb generation (covert, event-related)	Multiple words (covert)	15	Unknown	Unknown
noun repetition (event-related)	Multiple words (overt)	15	Yes	Unknown
verb generation (covert, block)	Multiple words (covert)	10	Unknown	Unknown
finger tapping (block)	Other	10	Unknown	Unknown

Conditions notes	Given the means and standard deviations presented, it is likely that some patients could not perform some tasks; post-scan recognition tests not considered to quantify performance

Contrasts

Are the contrasts clearly described?	Yes

Contrast 1: verb generation (covert, block) vs finger tapping (block)

Language condition	Verb generation (covert, block)
Control condition	Finger tapping (block)
Are the conditions matched for visual demands?	Yes
Are the conditions matched for auditory demands?	Yes
Are the conditions matched for motor demands?	No
Are the conditions matched for cognitive/executive demands?	No

Is accuracy matched between the language and control tasks for all relevant groups?	N/A, tasks not comparable
Is reaction time matched between the language and control tasks for all relevant groups?	N/A, tasks not comparable
Behavioral data notes	—
Are control data reported in this paper or another that is referenced?	Yes
Does the contrast selectively activate plausible relevant language regions in the control group?	Yes
Are activations lateralized in the control data?	Yes
Control activation notes	Strongly lateralized frontal and temporal activation
Contrast notes	—

Contrast 2: verb generation (overt, event-related) vs noun repetition (event-related)

Language condition	Verb generation (overt, event-related)
Control condition	Noun repetition (event-related)
Are the conditions matched for visual demands?	Yes
Are the conditions matched for auditory demands?	Yes
Are the conditions matched for motor demands?	Yes
Are the conditions matched for cognitive/executive demands?	No
Is accuracy matched between the language and control tasks for all relevant groups?	Appear mismatched
Is reaction time matched between the language and control tasks for all relevant groups?	Unknown, not reported
Behavioral data notes	—
Are control data reported in this paper or another that is referenced?	Yes
Does the contrast selectively activate plausible relevant language regions in the control group?	Somewhat
Are activations lateralized in the control data?	Somewhat
Control activation notes	Somewhat L-lateralized frontal, temporal and parietal activations, but also extensive midline activation
Contrast notes	—

Contrast 3: verb generation (overt, event-related) vs verb generation (covert, event-related)

Language condition	Verb generation (overt, event-related)
Control condition	Verb generation (covert, event-related)
Are the conditions matched for visual demands?	Yes
Are the conditions matched for auditory demands?	No
Are the conditions matched for motor demands?	No
Are the conditions matched for cognitive/executive demands?	Yes
Is accuracy matched between the language and control tasks for all relevant groups?	N/A, tasks not comparable
Is reaction time matched between the language and control tasks for all relevant groups?	N/A, tasks not comparable
Behavioral data notes	—
Are control data reported in this paper or another that is referenced?	Yes
Does the contrast selectively activate plausible relevant language regions in the control group?	Somewhat
Are activations lateralized in the control data?	N/A
Control activation notes	Bilateral speech motor activations, but also extensive midline activation
Contrast notes	—

Analyses

Are the analyses clearly described?	Yes

ROI analysis 1

First level contrast	Verb generation (covert, block) vs finger tapping (block)
Analysis class	Cross-sectional aphasia vs control
Group(s)	Aphasia vs control
Covariate	—
Is the second level contrast valid in terms of the group(s), time point(s), and measures involved?	Yes
Is accuracy matched across the second level contrast?	Unknown, not reported
Is reaction time matched across the second level contrast?	Unknown, not reported
Behavioral data notes	—
Type of analysis	Regions of interest (ROI)
ROI type	Laterality indi(ces)
How many ROIs are there?	2
What are the ROI(s)?	(1) frontal LI; (2) temporal LI
How are the ROI(s) defined?	
Correction for multiple comparisons	No correction
Statistical details	—
Findings	↓ LI (temporal)
Findings notes	—

ROI analysis 2

First level contrast	Verb generation (overt, event-related) vs noun repetition (event-related)
Analysis class	Cross-sectional aphasia vs control
Group(s)	Aphasia vs control
Covariate	—
Is the second level contrast valid in terms of the group(s), time point(s), and measures involved?	Yes
Is accuracy matched across the second level contrast?	No, different
Is reaction time matched across the second level contrast?	Unknown, not reported
Behavioral data notes	Patients less accurate and produced less responses on both conditions, but the difference between groups was greater for verb generation
Type of analysis	Regions of interest (ROI)
ROI type	Laterality indi(ces)
How many ROIs are there?	2
What are the ROI(s)?	(1) frontal LI; (2) temporal LI
How are the ROI(s) defined?	
Correction for multiple comparisons	No correction
Statistical details	—
Findings	↓ LI (frontal)
Findings notes	—

ROI analysis 3

First level contrast	Verb generation (overt, event-related) vs verb generation (covert, event-related)
Analysis class	Cross-sectional aphasia vs control
Group(s)	Aphasia vs control
Covariate	—
Is the second level contrast valid in terms of the group(s), time point(s), and measures involved?	Yes

Is accuracy matched across the second level contrast?	No, different
Is reaction time matched across the second level contrast?	Unknown, not reported
Behavioral data notes	Overt performance differed, so covert performance probably did too
Type of analysis	Regions of interest (ROI)
ROI type	Laterality indi(ces)
How many ROIs are there?	2
What are the ROI(s)?	(1) frontal LI; (2) temporal LI
How are the ROI(s) defined?	
Correction for multiple comparisons	No correction
Statistical details	—
Findings	None
Findings notes	Lack of lateralization in controls makes this analysis difficult to interpret

ROI analysis 4

First level contrast	Verb generation (overt, event-related) vs noun repetition (event-related)
Analysis class	Cross-sectional correlation with language or other measure
Group(s)	Aphasia
Covariate	Overt verb generation accuracy
Is the second level contrast valid in terms of the group(s), time point(s), and measures involved?	Yes
Is accuracy matched across the second level contrast?	Accuracy is covariate
Is reaction time matched across the second level contrast?	Unknown, not reported
Behavioral data notes	—
Type of analysis	Regions of interest (ROI)
ROI type	Functional
How many ROIs are there?	3
What are the ROI(s)?	(1) L MTG; (2) L SFG/CG; (3) left MFG
How are the ROI(s) defined?	Regions activated by the contrast of overt verb generation vs noun repetition in patients
Correction for multiple comparisons	No correction
Statistical details	—
Findings	↑ L dorsolateral prefrontal cortex<br>↑ L SMA/medial prefrontal
Findings notes	—

ROI analysis 5

First level contrast	Verb generation (overt, event-related) vs verb generation (covert, event-related)
Analysis class	Cross-sectional correlation with language or other measure
Group(s)	Aphasia
Covariate	Overt verb generation accuracy
Is the second level contrast valid in terms of the group(s), time point(s), and measures involved?	Yes
Is accuracy matched across the second level contrast?	Accuracy is covariate
Is reaction time matched across the second level contrast?	Unknown, not reported
Behavioral data notes	—
Type of analysis	Regions of interest (ROI)
ROI type	Functional
How many ROIs are there?	2
What are the ROI(s)?	(1) R insula/IFG; (2) R STG
How are the ROI(s) defined?	Prominent R hemisphere activations for the contrast of overt and covert verb generation in

	patients
Correction for multiple comparisons	No correction
Statistical details	—
Findings	None
Findings notes	—

Notes

Excluded analyses	Analysis of LI distribution (left/right/bilateral) yielded similar results

Fridriksson, Hubbard, et al. (2012)

Reference

Authors	Fridriksson J, Hubbard HI, Hudspeth SG, Holland AL, Bonilha L, Fromm D, Rorden C
Title	Speech entrainment enables patients with Broca's aphasia to produce fluent speech
Reference	*Brain* 2012; 135: 3815-3829
PMID	23250889
DOI	10.1093/brain/aws301

Participants

Language	US English
Inclusion criteria	Broca's aphasia
Number of individuals with aphasia	10 (plus 3 excluded: 1 due to a metal implant; 2 for severely non-fluent speech)
Number of control participants	20
Were any of the participants included in any previous studies?	No
Is age reported for patients and controls, and matched?	Yes (mean 56.9 ± 9.2 years, range 45-75 years)
Is sex reported for patients and controls, and matched?	No (males: 9; females: 4; control sex not matched)
Is handedness reported for patients and controls, and matched?	Yes (right: 12; left: 1)
Is time post stroke onset reported and appropriate to the study design?	Yes (mean 63.8 ± 64.3 months, range 10-261 months)
To what extent is the nature of aphasia characterized?	Comprehensive battery
Language evaluation	WAB, BNT, AoS from ABA
Aphasia severity	AQ mean 48.5 ± 20.6, range 20.9-73.5
Aphasia type	Broca's
First stroke only?	Yes
Stroke type	Not stated
To what extent is the lesion distribution characterized?	Lesion overlay
Lesion extent	Not stated
Lesion location	L MCA
Participants notes	Demographic data includes excluded patients

Imaging

Modality	fMRI
Is the study cross-sectional or longitudinal?	Cross-sectional
If longitudinal, at what time point(s) were imaging data acquired?	—
If longitudinal, was there any intervention between the time points?	—

Is the scanner described?	No (Siemens 3 Tesla; model not stated)
Is the timing of stimulus presentation and image acquisition clearly described and appropriate?	No* (moderate limitation) (it appears that each of the three conditions was presented in a separate run)
Design type	Event-related
Total images acquired	180?
Are the imaging acquisition parameters, including coverage, adequately described and appropriate?	Yes (whole brain)
Is preprocessing and intrasubject coregistration adequately described and appropriate?	Yes
Is first level model fitting adequately described and appropriate?	No (not described clearly)
Is intersubject normalization adequately described and appropriate?	Yes
Imaging notes	sparse sampling

Conditions

Are the conditions clearly described?	No (rest condition implied but not described)

Condition	Response type	Repetitions	All groups could do?	All individuals could do?
listening to/watching audiovisual sentences, while producing the same sentences in unison (speech entrainment)	Sentence (overt)	30 (?)	Yes	Unknown
listening to reversed sentences and viewing a mouth speaking, while producing unrelated sentences	Sentence (overt)	30 (?)	Yes	Unknown
listening to/watching audiovisual sentences and viewing a mouth	None	30 (?)	N/A	N/A
rest	None	implicit baseline	N/A	N/A

Conditions notes	—

Contrasts

Are the contrasts clearly described?	No (see specific limitation(s) below)

Contrast 1: listening to/watching audiovisual sentences, while producing the same sentences in unison (speech entrainment) vs listening to reversed sentences and viewing a mouth speaking, while producing unrelated sentences

Language condition	Listening to/watching audiovisual sentences, while producing the same sentences in unison (speech entrainment)
Control condition	Listening to reversed sentences and viewing a mouth speaking, while producing unrelated sentences
Are the conditions matched for visual demands?	Yes
Are the conditions matched for auditory demands?	Yes
Are the conditions matched for motor demands?	Yes
Are the conditions matched for cognitive/executive demands?	Yes
Is accuracy matched between the language and control tasks for all relevant groups?	Unknown, not reported
Is reaction time matched between the language and control tasks for all relevant groups?	Unknown, not reported
Behavioral data notes	Behavioral data outside the scanner suggest not matched, but in-scanner behavioral data not reported
Are control data reported in this paper or another that is referenced?	Somewhat
Does the contrast selectively activate plausible relevant language regions in the control group?	No
Are activations lateralized in the control data?	No

Control activation notes	Control and patient data are combined; this contrast activates bilateral anterior insula and posterior MTG, slightly more extensive on the L
Contrast notes	—

Contrast 2: listening to/watching audiovisual sentences, while producing the same sentences in unison (speech entrainment) vs rest

Language condition	Listening to/watching audiovisual sentences, while producing the same sentences in unison (speech entrainment)
Control condition	Rest
Are the conditions matched for visual demands?	No
Are the conditions matched for auditory demands?	No
Are the conditions matched for motor demands?	No
Are the conditions matched for cognitive/executive demands?	No
Is accuracy matched between the language and control tasks for all relevant groups?	N/A, tasks not comparable
Is reaction time matched between the language and control tasks for all relevant groups?	N/A, tasks not comparable
Behavioral data notes	—
Are control data reported in this paper or another that is referenced?	No
Does the contrast selectively activate plausible relevant language regions in the control group?	Unknown
Are activations lateralized in the control data?	Unknown
Control activation notes	—
Contrast notes	Rest condition implied but not explicitly described

Contrast 3: listening to reversed sentences and viewing a mouth speaking, while producing unrelated sentences vs rest

Language condition	Listening to reversed sentences and viewing a mouth speaking, while producing unrelated sentences
Control condition	Rest
Are the conditions matched for visual demands?	No
Are the conditions matched for auditory demands?	No
Are the conditions matched for motor demands?	No
Are the conditions matched for cognitive/executive demands?	No
Is accuracy matched between the language and control tasks for all relevant groups?	N/A, tasks not comparable
Is reaction time matched between the language and control tasks for all relevant groups?	N/A, tasks not comparable
Behavioral data notes	—
Are control data reported in this paper or another that is referenced?	No
Does the contrast selectively activate plausible relevant language regions in the control group?	Unknown
Are activations lateralized in the control data?	Unknown
Control activation notes	—
Contrast notes	Rest condition implied but not explicitly described

Contrast 4: listening to/watching audiovisual sentences and viewing a mouth vs rest

Language condition	Listening to/watching audiovisual sentences and viewing a mouth
Control condition	Rest
Are the conditions matched for visual demands?	No
Are the conditions matched for auditory demands?	No
Are the conditions matched for motor demands?	No
Are the conditions matched for cognitive/executive demands?	No

Is accuracy matched between the language and control tasks for all relevant groups?	N/A, no behavioral measure
Is reaction time matched between the language and control tasks for all relevant groups?	N/A, no timeable task
Behavioral data notes	—
Are control data reported in this paper or another that is referenced?	No
Does the contrast selectively activate plausible relevant language regions in the control group?	Unknown
Are activations lateralized in the control data?	Unknown
Control activation notes	—
Contrast notes	Rest condition implied but not explicitly described

Analyses

Are the analyses clearly described?	No** (major limitation) (see specific limitation(s) below)

Voxelwise analysis 1

First level contrast	Listening to/watching audiovisual sentences, while producing the same sentences in unison (speech entrainment) vs listening to reversed sentences and viewing a mouth speaking, while producing unrelated sentences
Analysis class	Cross-sectional aphasia vs control
Group(s)	Aphasia T1 vs control
Covariate	—
Is the second level contrast valid in terms of the group(s), time point(s), and measures involved?	Yes
Is accuracy matched across the second level contrast?	Unknown, not reported
Is reaction time matched across the second level contrast?	N/A, no timeable task
Behavioral data notes	—
Type of analysis	Voxelwise
Search volume	Whole brain
Correction for multiple comparisons	Unclear or not stated
Software	FSL (FEAT 5.98)
Voxelwise p	—
Cluster extent	—
Statistical details	Thresholding not stated
Findings	↑ L angular gyrus<br>↓ L anterior temporal
Findings notes	Based on coordinates in Table 2

Voxelwise analysis 2

First level contrast	Listening to/watching audiovisual sentences, while producing the same sentences in unison (speech entrainment) vs rest
Analysis class	Longitudinal change in aphasia
Group(s)	Aphasia T2 vs T1
Covariate	—
Is the second level contrast valid in terms of the group(s), time point(s), and measures involved?	Yes
Is accuracy matched across the second level contrast?	Unknown, not reported
Is reaction time matched across the second level contrast?	N/A, no timeable task
Behavioral data notes	—
Type of analysis	Voxelwise
Search volume	Whole brain

Correction for multiple comparisons	Unclear or not stated
Software	FSL (FEAT 5.98)
Voxelwise p	—
Cluster extent	—
Statistical details	Thresholding not stated
Findings	↑ L SMA/medial prefrontal<br>↑ L anterior cingulate<br>↑ R precuneus<br>↑ R occipital<br>↑ R hippocampus/MTL<br>↓ L supramarginal gyrus
Findings notes	Some labels changed based on coordinates

Voxelwise analysis 3

First level contrast	Listening to reversed sentences and viewing a mouth speaking, while producing unrelated sentences vs rest
Analysis class	Longitudinal change in aphasia
Group(s)	Aphasia T2 vs T1
Covariate	—
Is the second level contrast valid in terms of the group(s), time point(s), and measures involved?	Yes
Is accuracy matched across the second level contrast?	Unknown, not reported
Is reaction time matched across the second level contrast?	N/A, no timeable task
Behavioral data notes	—
Type of analysis	Voxelwise
Search volume	Whole brain
Correction for multiple comparisons	Unclear or not stated
Software	FSL (FEAT 5.98)
Voxelwise p	—
Cluster extent	—
Statistical details	Thresholding not stated
Findings	None
Findings notes	—

Voxelwise analysis 4

First level contrast	Listening to/watching audiovisual sentences and viewing a mouth vs rest
Analysis class	Longitudinal change in aphasia
Group(s)	Aphasia T2 vs T1
Covariate	—
Is the second level contrast valid in terms of the group(s), time point(s), and measures involved?	Yes
Is accuracy matched across the second level contrast?	N/A, no behavioral measure
Is reaction time matched across the second level contrast?	N/A, no timeable task
Behavioral data notes	—
Type of analysis	Voxelwise
Search volume	Whole brain
Correction for multiple comparisons	Unclear or not stated
Software	FSL (FEAT 5.98)
Voxelwise p	—
Cluster extent	—
Statistical details	Thresholding not stated
Findings	None

Findings notes	—

ROI analysis 1

First level contrast	Listening to/watching audiovisual sentences, while producing the same sentences in unison (speech entrainment) vs listening to reversed sentences and viewing a mouth speaking, while producing unrelated sentences
Analysis class	Cross-sectional aphasia vs control
Group(s)	Aphasia T1 vs control
Covariate	—
Is the second level contrast valid in terms of the group(s), time point(s), and measures involved?	Yes
Is accuracy matched across the second level contrast?	Unknown, not reported
Is reaction time matched across the second level contrast?	N/A, no timeable task
Behavioral data notes	—
Type of analysis	Regions of interest (ROI)
ROI type	Functional
How many ROIs are there?	6
What are the ROI(s)?	(1) L anterior insula/IFG pars orbitalis; (2) R anterior insula/IFG pars orbitalis; (3) Broca's area; (4) L MTG; (5) L BA 37; (6) R BA 37
How are the ROI(s) defined?	Regions activated in both groups considered together
Correction for multiple comparisons	No correction
Statistical details	There were no interactions of group by condition; two regions showed main effects of group but this is not pertinent to the contrast
Findings	None
Findings notes	—

Notes

Excluded analyses	—

Fridriksson, Richardson, et al. (2012)

Reference

Authors	Fridriksson J, Richardson JD, Fillmore P, Cai B
Title	Left hemisphere plasticity and aphasia recovery
Reference	*NeuroImage* 2012; 60: 854-863
PMID	22227052
DOI	10.1016/j.neuroimage.2011.12.057

Participants

Language	US English
Inclusion criteria	—
Number of individuals with aphasia	29 (plus 1 excluded: contraindications to MRI)
Number of control participants	14
Were any of the participants included in any previous studies?	Yes (26 of 30 patients were included in Fridriksson (2010))
Is age reported for patients and controls, and matched?	Yes (mean 59.2 years, range 33-81 years)
Is sex reported for patients and controls, and matched?	No (males: 14; females: 16; not stated for controls)
Is handedness reported for patients and controls, and matched?	No

Is time post stroke onset reported and appropriate to the study design?	Yes (mean 51.1 months, range 6-350 months)
To what extent is the nature of aphasia characterized?	Severity and type
Language evaluation	WAB
Aphasia severity	AQ mean 57.9 ± 25.8, range 17.2-95.2
Aphasia type	13 Broca's, 10 anomic, 3 conduction, 2 Wernicke's, 1 global, 1 transcortical motor
First stroke only?	Yes
Stroke type	Mixed etiologies
To what extent is the lesion distribution characterized?	Lesion overlay
Lesion extent	Range 7.7-420.5 cc
Lesion location	L MCA
Participants notes	Demographic data includes excluded patient

Imaging

Modality	fMRI
Is the study cross-sectional or longitudinal?	Longitudinal—chronic treatment
If longitudinal, at what time point(s) were imaging data acquired?	T1: pre-treatment/chronic; T2: post-treatment/~4 weeks later; note that there were two separate sessions per time point, as well as another two sessions midway through treatment that are not analyzed in this paper
If longitudinal, was there any intervention between the time points?	Anomia treatment using a cueing hierarchy, 3 hours/day, 5 days/week, 2 weeks, with a 1-week gap between the two weeks
Is the scanner described?	Yes (Siemens Trio 3 Tesla)
Is the timing of stimulus presentation and image acquisition clearly described and appropriate?	No (timing of stimuli within the silent periods is unclear)
Design type	Event-related
Total images acquired	120
Are the imaging acquisition parameters, including coverage, adequately described and appropriate?	Yes (whole brain)
Is preprocessing and intrasubject coregistration adequately described and appropriate?	Yes
Is first level model fitting adequately described and appropriate?	Yes
Is intersubject normalization adequately described and appropriate?	Yes
Imaging notes	sparse sampling; 26 patients were also scanned with arterial spin labelling

Conditions

Are the conditions clearly described?	Yes

Condition	Response type	Repetitions	All groups could do?	All individuals could do?
picture naming	Word (overt)	80	Yes	Unknown
viewing abstract pictures	None	40	N/A	N/A

Conditions notes	—

Contrasts

Are the contrasts clearly described?	Yes

Contrast 1: picture naming vs viewing abstract pictures

Language condition	Picture naming
Control condition	Viewing abstract pictures
Are the conditions matched for visual demands?	Yes
Are the conditions matched for auditory demands?	No
Are the conditions matched for motor demands?	No

Are the conditions matched for cognitive/executive demands?	No
Is accuracy matched between the language and control tasks for all relevant groups?	N/A, tasks not comparable
Is reaction time matched between the language and control tasks for all relevant groups?	N/A, tasks not comparable
Behavioral data notes	—
Are control data reported in this paper or another that is referenced?	Somewhat
Does the contrast selectively activate plausible relevant language regions in the control group?	No
Are activations lateralized in the control data?	Somewhat
Control activation notes	Control data in Fridriksson et al. (2007); motor activations are prominent; there is some L frontal activation but little temporal activation in either hemisphere
Contrast notes	—

Analyses

Are the analyses clearly described?	No* (moderate limitation) (see specific limitation(s) below)

ROI analysis 1

First level contrast	Picture naming vs viewing abstract pictures
Analysis class	Longitudinal correlation with language or other measure
Group(s)	Aphasia T2 vs T1
Covariate	Δ picture naming accuracy
Is the second level contrast valid in terms of the group(s), time point(s), and measures involved?	Yes
Is accuracy matched across the second level contrast?	Accuracy is covariate
Is reaction time matched across the second level contrast?	Unknown, not reported
Behavioral data notes	—
Type of analysis	Regions of interest (ROI)
ROI type	Other
How many ROIs are there?	3
What are the ROI(s)?	(1) perilesional L hemisphere language regions; (2) perilesional L hemisphere non-language regions; (3) undamaged non-perilesional L hemisphere language regions
How are the ROI(s) defined?	Based on individual lesions and control activation for picture naming
Correction for multiple comparisons	No correction
Statistical details	—
Findings	Other
Findings notes	Change in perilesional non-language regions positively correlated with improvement in accuracy

ROI analysis 2

First level contrast	Picture naming vs viewing abstract pictures
Analysis class	Longitudinal correlation with language or other measure
Group(s)	Aphasia T2 vs T1
Covariate	Δ (decrease in) semantic errors
Is the second level contrast valid in terms of the group(s), time point(s), and measures involved?	Yes
Is accuracy matched across the second level contrast?	Unknown, not reported
Is reaction time matched across the second level contrast?	Unknown, not reported
Behavioral data notes	—
Type of analysis	Regions of interest (ROI)

ROI type	Other
How many ROIs are there?	3
What are the ROI(s)?	(1) perilesional L hemisphere language regions; (2) perilesional L hemisphere non-language regions; (3) undamaged non-perilesional L hemisphere language regions
How are the ROI(s) defined?	Based on individual lesions and control activation for picture naming
Correction for multiple comparisons	No correction
Statistical details	—
Findings	Other
Findings notes	Change in undamaged non-perilesional language regions negatively correlated with decrease in semantic errors

ROI analysis 3

First level contrast	Picture naming vs viewing abstract pictures
Analysis class	Longitudinal correlation with language or other measure
Group(s)	Aphasia T2 vs T1
Covariate	Δ (decrease in) phonological paraphasias
Is the second level contrast valid in terms of the group(s), time point(s), and measures involved?	Yes
Is accuracy matched across the second level contrast?	Unknown, not reported
Is reaction time matched across the second level contrast?	Unknown, not reported
Behavioral data notes	—
Type of analysis	Regions of interest (ROI)
ROI type	Other
How many ROIs are there?	3
What are the ROI(s)?	(1) perilesional L hemisphere language regions; (2) perilesional L hemisphere non-language regions; (3) undamaged non-perilesional L hemisphere language regions
How are the ROI(s) defined?	Based on individual lesions and control activation for picture naming
Correction for multiple comparisons	No correction
Statistical details	—
Findings	Other
Findings notes	Change in perilesional language regions, and change in undamaged non-perilesional language regions, negatively correlated with decrease in phonological paraphasias

ROI analysis 4

First level contrast	Picture naming vs viewing abstract pictures
Analysis class	Cross-sectional correlation with language or other measure
Group(s)	Aphasia T1
Covariate	Subsequent Δ (T2 vs T1) picture naming accuracy
Is the second level contrast valid in terms of the group(s), time point(s), and measures involved?	Somewhat (T1 behavioral measure should be included in model)
Is accuracy matched across the second level contrast?	Unknown, not reported
Is reaction time matched across the second level contrast?	Unknown, not reported
Behavioral data notes	—
Type of analysis	Regions of interest (ROI)
ROI type	Other
How many ROIs are there?	3
What are the ROI(s)?	(1) perilesional L hemisphere language regions; (2) perilesional L hemisphere non-language regions; (3) undamaged non-perilesional L hemisphere language regions
How are the ROI(s) defined?	Based on individual lesions and control activation for picture naming
Correction for multiple comparisons	No correction
Statistical details	—
Findings	None

Findings notes	—

ROI analysis 5

First level contrast	Picture naming vs viewing abstract pictures
Analysis class	Cross-sectional correlation with language or other measure
Group(s)	Aphasia T1
Covariate	Subsequent Δ (T2 vs T1, decrease in) semantic errors
Is the second level contrast valid in terms of the group(s), time point(s), and measures involved?	Somewhat (T1 behavioral measure should be included in model)
Is accuracy matched across the second level contrast?	Unknown, not reported
Is reaction time matched across the second level contrast?	Unknown, not reported
Behavioral data notes	—
Type of analysis	Regions of interest (ROI)
ROI type	Other
How many ROIs are there?	3
What are the ROI(s)?	(1) perilesional L hemisphere language regions; (2) perilesional L hemisphere non-language regions; (3) undamaged non-perilesional L hemisphere language regions
How are the ROI(s) defined?	Based on individual lesions and control activation for picture naming
Correction for multiple comparisons	No correction
Statistical details	—
Findings	Other
Findings notes	Change in perilesional language regions correlated with decrease in phonological paraphasias

ROI analysis 6

First level contrast	Picture naming vs viewing abstract pictures
Analysis class	Cross-sectional correlation with language or other measure
Group(s)	Aphasia T1
Covariate	Subsequent Δ (T2 vs T1, decrease in) phonological paraphasias
Is the second level contrast valid in terms of the group(s), time point(s), and measures involved?	Somewhat (T1 behavioral measure should be included in model)
Is accuracy matched across the second level contrast?	Unknown, not reported
Is reaction time matched across the second level contrast?	Unknown, not reported
Behavioral data notes	—
Type of analysis	Regions of interest (ROI)
ROI type	Other
How many ROIs are there?	3
What are the ROI(s)?	(1) perilesional L hemisphere language regions; (2) perilesional L hemisphere non-language regions; (3) undamaged non-perilesional L hemisphere language regions
How are the ROI(s) defined?	Based on individual lesions and control activation for picture naming
Correction for multiple comparisons	No correction
Statistical details	—
Findings	None
Findings notes	—

Notes

Excluded analyses	(1) breakdown of frontal, temporal and parietal components of masks, because stepwise regression not described in sufficient detail; (2) pASL rCBF predictors not task-based; (3) ancillary analyses based on total naming responses instead of accuracy; (4) ancillary analyses after excluding one patient

Marcotte et al. (2012)

Reference

Authors	Marcotte K, Adrover-Roig D, Damien B, de Préaumont M, Généreux S, Hubert M, Ansaldo AI
Title	Therapy-induced neuroplasticity in chronic aphasia
Reference	*Neuropsychologia* 2012; 50: 1776-1786
PMID	22564481
DOI	10.1016/j.neuropsychologia.2012.04.001

Participants

Language	Canadian French
Inclusion criteria	Moderate-severe aphasia; anomia
Number of individuals with aphasia	9
Number of control participants	0
Were any of the participants included in any previous studies?	No
Is age reported for patients and controls, and matched?	Yes (mean 62 ± 6.0 years, range 50-67 years)
Is sex reported for patients and controls, and matched?	Yes (males: 5; females: 4)
Is handedness reported for patients and controls, and matched?	Yes (right: 9; left: 0)
Is time post stroke onset reported and appropriate to the study design?	Yes (mean 110.2 ± 92.5 months, range 50-300 months)
To what extent is the nature of aphasia characterized?	Comprehensive battery
Language evaluation	Montreal-Toulouse Aphasia Battery, picture naming
Aphasia severity	Moderate-severe
Aphasia type	7 Broca's, 1 Broca's + AoS, 1 Wernicke's + AoS
First stroke only?	Yes
Stroke type	Not stated
To what extent is the lesion distribution characterized?	Lesion overlay
Lesion extent	Range 14.6-295.8 cc
Lesion location	L MCA
Participants notes	—

Imaging

Modality	fMRI
Is the study cross-sectional or longitudinal?	Longitudinal—chronic treatment
If longitudinal, at what time point(s) were imaging data acquired?	T1: pre-treatment/chronic; T2: post-treatment, 3-6 weeks later (after 80% performance on trained items, or 6 weeks)
If longitudinal, was there any intervention between the time points?	Semantic feature analysis, 1 hour/day, 3 days/week, 3-6 weeks
Is the scanner described?	Yes (Siemens Trio 3 Tesla)
Is the timing of stimulus presentation and image acquisition clearly described and appropriate?	No (total images acquired not stated)
Design type	Event-related
Total images acquired	not stated
Are the imaging acquisition parameters, including coverage, adequately described and appropriate?	Yes (whole brain)
Is preprocessing and intrasubject coregistration adequately described and appropriate?	Yes
Is first level model fitting adequately described and appropriate?	Yes
Is intersubject normalization adequately described	No (lesion impact not addressed)

and appropriate?	
Imaging notes	—

Conditions

Are the conditions clearly described?	Yes

Condition	Response type	Repetitions	All groups could do?	All individuals could do?
picture naming (already known items)	Word (overt)	20	Yes	Yes
picture naming (trained items)	Word (overt)	20	No	No
picture naming (untrained items)	Word (overt)	40	No	No
viewing scrambled images and saying "baba"	Word (overt)	20	Yes	Yes
rest	None	implicit baseline	N/A	N/A

Conditions notes	—

Contrasts

Are the contrasts clearly described?	No (see specific limitation(s) below)

Contrast 1: picture naming (T1: known items; T2: trained items; correct trials) vs viewing scrambled images and saying "baba"

Language condition	Picture naming (T1: known items; T2: trained items; correct trials)
Control condition	Viewing scrambled images and saying "baba"
Are the conditions matched for visual demands?	Yes
Are the conditions matched for auditory demands?	Yes
Are the conditions matched for motor demands?	Yes
Are the conditions matched for cognitive/executive demands?	No
Is accuracy matched between the language and control tasks for all relevant groups?	Yes, correct trials only
Is reaction time matched between the language and control tasks for all relevant groups?	Unknown, not reported
Behavioral data notes	—
Are control data reported in this paper or another that is referenced?	No
Does the contrast selectively activate plausible relevant language regions in the control group?	Unknown
Are activations lateralized in the control data?	Unknown
Control activation notes	—
Contrast notes	Different contrasts at different time points not clearly explained

Contrast 2: picture naming (known items, correct trials) vs viewing scrambled images and saying "baba"

Language condition	Picture naming (known items, correct trials)
Control condition	Viewing scrambled images and saying "baba"
Are the conditions matched for visual demands?	Yes
Are the conditions matched for auditory demands?	Yes
Are the conditions matched for motor demands?	Yes
Are the conditions matched for cognitive/executive demands?	No
Is accuracy matched between the language and control tasks for all relevant groups?	Yes, correct trials only
Is reaction time matched between the language and control tasks for all relevant groups?	Unknown, not reported
Behavioral data notes	—
Are control data reported in this paper or another that is referenced?	No

Does the contrast selectively activate plausible relevant language regions in the control group?	Unknown
Are activations lateralized in the control data?	Unknown
Control activation notes	—
Contrast notes	Different contrasts at different time points not clearly explained

Contrast 3: picture naming (trained items, correct trials) vs viewing scrambled images and saying "baba"

Language condition	Picture naming (trained items, correct trials)
Control condition	Viewing scrambled images and saying "baba"
Are the conditions matched for visual demands?	Yes
Are the conditions matched for auditory demands?	Yes
Are the conditions matched for motor demands?	Yes
Are the conditions matched for cognitive/executive demands?	No
Is accuracy matched between the language and control tasks for all relevant groups?	Yes, correct trials only
Is reaction time matched between the language and control tasks for all relevant groups?	Unknown, not reported
Behavioral data notes	—
Are control data reported in this paper or another that is referenced?	No
Does the contrast selectively activate plausible relevant language regions in the control group?	Unknown
Are activations lateralized in the control data?	Unknown
Control activation notes	—
Contrast notes	Different contrasts at different time points not clearly explained

Analyses

Are the analyses clearly described?	No (see specific limitation(s) below)

Voxelwise analysis 1

First level contrast	Picture naming (T1: known items; T2: trained items; correct trials) vs viewing scrambled images and saying "baba"
Analysis class	Longitudinal change in aphasia
Group(s)	Aphasia T2 vs T1
Covariate	—
Is the second level contrast valid in terms of the group(s), time point(s), and measures involved?	Yes
Is accuracy matched across the second level contrast?	Yes, correct trials only
Is reaction time matched across the second level contrast?	Unknown, not reported
Behavioral data notes	—
Type of analysis	Voxelwise
Search volume	Whole brain
Correction for multiple comparisons	No direct comparison
Software	SPM5
Voxelwise p	—
Cluster extent	—
Statistical details	Qualitative comparison on p. 1780; different contrasts at different time points not clearly explained
Findings	↑ L supramarginal gyrus<br>↓ L dorsal precentral<br>↓ L posterior MTG
Findings notes	Labels based on figures rather than text

Voxelwise analysis 2

First level contrast	Picture naming (known items, correct trials) vs viewing scrambled images and saying "baba"
Analysis class	Cross-sectional correlation with language or other measure
Group(s)	Aphasia T1
Covariate	Subsequent Δ (T2 vs T1) naming of trained items
Is the second level contrast valid in terms of the group(s), time point(s), and measures involved?	Somewhat (T1 behavioral measure should be included in model)
Is accuracy matched across the second level contrast?	Yes, correct trials only
Is reaction time matched across the second level contrast?	Unknown, not reported
Behavioral data notes	—
Type of analysis	Voxelwise
Search volume	Whole brain
Correction for multiple comparisons	Clusterwise correction based on arbitrary cluster extent
Software	SPM5
Voxelwise p	.005
Cluster extent	10 voxels (size not stated)
Statistical details	Different contrasts at different time points not clearly explained
Findings	↑ L dorsolateral prefrontal cortex<br>↑ L SMA/medial prefrontal<br>↑ L somato-motor<br>↑ L anterior cingulate<br>↑ R dorsolateral prefrontal cortex<br>↑ R somato-motor<br>↑ R thalamus
Findings notes	Labels based on figures and text

Voxelwise analysis 3

First level contrast	Picture naming (trained items, correct trials) vs viewing scrambled images and saying "baba"
Analysis class	Cross-sectional correlation with language or other measure
Group(s)	Aphasia T2
Covariate	Previous Δ (T2 vs T1) naming of trained items
Is the second level contrast valid in terms of the group(s), time point(s), and measures involved?	No (T2 activation not an appropriate measure of treatment-induced recovery because it reflects T2 performance)
Is accuracy matched across the second level contrast?	Yes, correct trials only
Is reaction time matched across the second level contrast?	Unknown, not reported
Behavioral data notes	—
Type of analysis	Voxelwise
Search volume	Whole brain
Correction for multiple comparisons	Clusterwise correction based on arbitrary cluster extent
Software	SPM5
Voxelwise p	.005
Cluster extent	10 voxels (size not stated)
Statistical details	Different contrasts at different time points not clearly explained
Findings	↑ L somato-motor
Findings notes	Label based on figure

Notes

Excluded analyses	Individual analyses of participants with more and less successful recovery

Schofield et al. (2012)

Reference

Authors	Schofield TM, Penny WD, Stephan KE, Crinion JT, Thompson AJ, Price CJ, Leff AP
Title	Changes in auditory feedback connections determine the severity of speech processing deficits after stroke
Reference	*J Neurosci* 2012; 32: 4260-4270
PMID	22442088
DOI	10.1523/jneurosci.4670-11.2012

Participants

Language	UK English
Inclusion criteria	Comprehension deficit
Number of individuals with aphasia	20 (plus 1 excluded: excessive head motion)
Number of control participants	26
Were any of the participants included in any previous studies?	Yes (patients recruited from database so may have participated in prior studies from this group, but not stated explicitly)
Is age reported for patients and controls, and matched?	Yes (range 35.8-90.3 years)
Is sex reported for patients and controls, and matched?	No (males: 16; females: 4; control sex not stated)
Is handedness reported for patients and controls, and matched?	No
Is time post stroke onset reported and appropriate to the study design?	Yes (mean 3.5 years, range 0.6-8.6 years)
To what extent is the nature of aphasia characterized?	Severity only
Language evaluation	CAT
Aphasia severity	11 patients (plus one excluded) had moderate comprehension impairments, 9 had severe comprehension impairments; this distribution was bimodal
Aphasia type	Not stated
First stroke only?	Yes
Stroke type	Ischemic only
To what extent is the lesion distribution characterized?	Lesion overlay
Lesion extent	Range 24.2-403.6 cc
Lesion location	L MCA
Participants notes	Demographic data includes excluded patient

Imaging

Modality	fMRI
Is the study cross-sectional or longitudinal?	Cross-sectional
If longitudinal, at what time point(s) were imaging data acquired?	—
If longitudinal, was there any intervention between the time points?	—
Is the scanner described?	Yes (Siemens Sonata 1.5 Tesla)
Is the timing of stimulus presentation and image acquisition clearly described and appropriate?	Yes
Design type	Block
Total images acquired	488
Are the imaging acquisition parameters, including coverage, adequately described and appropriate?	Yes (mostly whole brain but convexity or cerebellum excluded in some participants)
Is preprocessing and intrasubject coregistration adequately described and appropriate?	Yes
Is first level model fitting adequately described and	Yes

appropriate?	
Is intersubject normalization adequately described and appropriate?	Yes
Imaging notes	—

Conditions

Are the conditions clearly described?	Yes

Condition	Response type	Repetitions	All groups could do?	All individuals could do?
listening to word pairs, speaker gender judgment	Button press	18	Yes	Unknown
listening to reversed word pairs, speaker gender judgment	Button press	18	Yes	Unknown
rest	None	40 (?)	N/A	N/A

Conditions notes	—

Contrasts

Are the contrasts clearly described?	Yes

Contrast 1: listening to word pairs or reversed word pairs, speaker gender judgment vs rest

Language condition	Listening to word pairs or reversed word pairs, speaker gender judgment
Control condition	Rest
Are the conditions matched for visual demands?	No
Are the conditions matched for auditory demands?	No
Are the conditions matched for motor demands?	No
Are the conditions matched for cognitive/executive demands?	No
Is accuracy matched between the language and control tasks for all relevant groups?	N/A, tasks not comparable
Is reaction time matched between the language and control tasks for all relevant groups?	N/A, tasks not comparable
Behavioral data notes	—
Are control data reported in this paper or another that is referenced?	Yes
Does the contrast selectively activate plausible relevant language regions in the control group?	No
Are activations lateralized in the control data?	No
Control activation notes	Control data in Leff et al. (2008); auditory contrast, not intended to be language contrast
Contrast notes	—

Contrast 2: listening to word pairs, speaker gender judgment vs listening to reversed word pairs, speaker gender judgment

Language condition	Listening to word pairs, speaker gender judgment
Control condition	Listening to reversed word pairs, speaker gender judgment
Are the conditions matched for visual demands?	Yes
Are the conditions matched for auditory demands?	Yes
Are the conditions matched for motor demands?	Yes
Are the conditions matched for cognitive/executive demands?	Yes
Is accuracy matched between the language and control tasks for all relevant groups?	Unknown, not reported
Is reaction time matched between the language and control tasks for all relevant groups?	Unknown, not reported
Behavioral data notes	Behavioral data not separated by condition
Are control data reported in this paper or another that is referenced?	Yes
Does the contrast selectively activate plausible	Somewhat

relevant language regions in the control group?	
Are activations lateralized in the control data?	Yes
Control activation notes	Control data in Leff et al. (2008); L-lateralized activation of posterior STS
Contrast notes	—

Analyses

Are the analyses clearly described?	No** (major limitation) (see specific limitation(s) below)

Voxelwise analysis 1

First level contrast	Listening to word pairs or reversed word pairs, speaker gender judgment vs rest
Analysis class	Cross-sectional aphasia vs control
Group(s)	Moderate aphasia (n = 11) vs control
Covariate	—
Is the second level contrast valid in terms of the group(s), time point(s), and measures involved?	Yes
Is accuracy matched across the second level contrast?	Unknown, not reported
Is reaction time matched across the second level contrast?	Unknown, not reported
Behavioral data notes	—
Type of analysis	Voxelwise
Search volume	Whole brain
Correction for multiple comparisons	No correction
Software	SPM8
Voxelwise p	.001
Cluster extent	None
Statistical details	—
Findings	↓ L Heschl's gyrus
Findings notes	Structurally, HG was not significantly damaged in this group

Voxelwise analysis 2

First level contrast	Listening to word pairs or reversed word pairs, speaker gender judgment vs rest
Analysis class	Cross-sectional aphasia vs control
Group(s)	Severe aphasia (n = 9) vs control
Covariate	—
Is the second level contrast valid in terms of the group(s), time point(s), and measures involved?	Yes
Is accuracy matched across the second level contrast?	Unknown, not reported
Is reaction time matched across the second level contrast?	Unknown, not reported
Behavioral data notes	—
Type of analysis	Voxelwise
Search volume	Whole brain
Correction for multiple comparisons	Mixed** (major limitation)
Software	SPM8
Voxelwise p	MGB: SVC; elsewhere: .001
Cluster extent	None
Statistical details	—
Findings	↓ L posterior STG<br>↓ L Heschl's gyrus<br>↓ L thalamus
Findings notes	Specifically: PT, HG and MGB; structurally, the PT and HG were significantly damaged, but not the MGB

Voxelwise analysis 3

First level contrast	Listening to word pairs or reversed word pairs, speaker gender judgment vs rest
Analysis class	Cross-sectional between two groups with aphasia
Group(s)	Severe (n = 9) vs moderate (n = 11) aphasia
Covariate	—
Is the second level contrast valid in terms of the group(s), time point(s), and measures involved?	Yes
Is accuracy matched across the second level contrast?	Unknown, not reported
Is reaction time matched across the second level contrast?	Unknown, not reported
Behavioral data notes	—
Type of analysis	Voxelwise
Search volume	Whole brain
Correction for multiple comparisons	No correction
Software	SPM8
Voxelwise p	.001
Cluster extent	None
Statistical details	—
Findings	↓ L posterior STG
Findings notes	Specifically, PT; structurally, severe patients had more damage in HG and PT

Notes

Excluded analyses	Intelligibility contrasts, because findings are unclear: statements of significance in the text do not match Table 5; DCM analyses (which are the main focus of the paper)

Wright et al. (2012)

Reference

Authors	Wright P, Stamatakis EA, Tyler LK
Title	Differentiating hemispheric contributions to syntax and semantics in patients with left-hemisphere lesions
Reference	*J Neurosci* 2012; 32: 8149-8157
PMID	22699896
DOI	10.1523/jneurosci.0485-12.2012

Participants

Language	UK English
Inclusion criteria	—
Number of individuals with aphasia	21
Number of control participants	21
Were any of the participants included in any previous studies?	Yes (unclear how many, if any, patients were included in previous studies from this group; design is identical to Tyler et al. (2010))
Is age reported for patients and controls, and matched?	Yes (mean 57.4 ± 12.5 years)
Is sex reported for patients and controls, and matched?	Yes (males: 15; females: 6)
Is handedness reported for patients and controls, and matched?	Yes (right: 21; left: 0)
Is time post stroke onset reported and appropriate to the study design?	Yes (mean 6.5 ± 7.5 years, > 1.4 years)
To what extent is the nature of aphasia characterized?	Not at all
Language evaluation	Sentence-picture matching

Aphasia severity	Not stated
Aphasia type	Not stated
First stroke only?	Yes
Stroke type	Not stated
To what extent is the lesion distribution characterized?	Lesion overlay
Lesion extent	Not stated
Lesion location	L MCA
Participants notes	3 of the 21 patients were not stroke, but were post resective surgery

Imaging

Modality	fMRI
Is the study cross-sectional or longitudinal?	Cross-sectional
If longitudinal, at what time point(s) were imaging data acquired?	—
If longitudinal, was there any intervention between the time points?	—
Is the scanner described?	Yes (Siemens Trio 3 Tesla)
Is the timing of stimulus presentation and image acquisition clearly described and appropriate?	No* (moderate limitation) (there was only one block per condition per run, so condition could be confounded with low frequency drift; also, the length of the sentences is not stated so it is unclear how well the HRF peak aligns with the sparse acquisitions)
Design type	Block
Total images acquired	69
Are the imaging acquisition parameters, including coverage, adequately described and appropriate?	Yes (whole brain)
Is preprocessing and intrasubject coregistration adequately described and appropriate?	Yes
Is first level model fitting adequately described and appropriate?	Yes
Is intersubject normalization adequately described and appropriate?	Yes
Imaging notes	sparse sampling

Conditions

Are the conditions clearly described?	Yes

Condition	Response type	Repetitions	All groups could do?	All individuals could do?
listening to normal sentences and detecting a target word	Button press	2	Yes	Yes
listening to grammatical but meaningless sentences and detecting a target word	Button press	2	Yes	Yes
listening to scrambled sentences and detecting a target word	Button press	2	Yes	Yes
listening to "musical rain" and detecting a period of white noise	Button press	2	Yes	Yes
rest	None	2	N/A	N/A

Conditions notes	Auditory presentation; target detection task with early and late targets; 12-15 trials per block with single sparse acquisition each, but only one block of each condition per run, in fixed order

Contrasts

Are the contrasts clearly described?	Yes

Contrast 1: listening to normal sentences and detecting a target word vs rest

Language condition	Listening to normal sentences and detecting a target word

Control condition	Rest
Are the conditions matched for visual demands?	No
Are the conditions matched for auditory demands?	No
Are the conditions matched for motor demands?	No
Are the conditions matched for cognitive/executive demands?	No
Is accuracy matched between the language and control tasks for all relevant groups?	N/A, tasks not comparable
Is reaction time matched between the language and control tasks for all relevant groups?	N/A, tasks not comparable
Behavioral data notes	—
Are control data reported in this paper or another that is referenced?	Yes
Does the contrast selectively activate plausible relevant language regions in the control group?	No
Are activations lateralized in the control data?	No
Control activation notes	Bilateral superior temporal, sensorimotor and visual
Contrast notes	—

Contrast 2: listening to grammatical but meaningless sentences and detecting a target word vs rest

Language condition	Listening to grammatical but meaningless sentences and detecting a target word
Control condition	Rest
Are the conditions matched for visual demands?	No
Are the conditions matched for auditory demands?	No
Are the conditions matched for motor demands?	No
Are the conditions matched for cognitive/executive demands?	No
Is accuracy matched between the language and control tasks for all relevant groups?	N/A, tasks not comparable
Is reaction time matched between the language and control tasks for all relevant groups?	N/A, tasks not comparable
Behavioral data notes	—
Are control data reported in this paper or another that is referenced?	No
Does the contrast selectively activate plausible relevant language regions in the control group?	Unknown
Are activations lateralized in the control data?	Unknown
Control activation notes	—
Contrast notes	—

Analyses

Are the analyses clearly described?	Yes

Voxelwise analysis 1

First level contrast	Listening to normal sentences and detecting a target word vs rest
Analysis class	Cross-sectional aphasia vs control
Group(s)	Aphasia vs control
Covariate	—
Is the second level contrast valid in terms of the group(s), time point(s), and measures involved?	Yes
Is accuracy matched across the second level contrast?	Yes, matched
Is reaction time matched across the second level contrast?	Unknown, not reported
Behavioral data notes	—
Type of analysis	Voxelwise

Search volume	Whole brain
Correction for multiple comparisons	No correction
Software	SPM5
Voxelwise p	.01
Cluster extent	—
Statistical details	—
Findings	↓ L posterior STG/STS/MTG<br>↓ L Heschl's gyrus<br>↓ L mid temporal
Findings notes	At a more stringent threshold of p < .001, with correction for multiple comparisons based on GRFT and cluster extent, only L HG showed reduced activity in patients

Complex analysis 1

First level contrast	Listening to normal sentences and detecting a target word vs rest
Analysis class	Cross-sectional correlation with language or other measure
Group(s)	Aphasia
Covariate	See statistical details
Is the second level contrast valid in terms of the group(s), time point(s), and measures involved?	Yes
Is accuracy matched across the second level contrast?	Unknown, not reported
Is reaction time matched across the second level contrast?	Unknown, not reported
Behavioral data notes	—
Type of analysis	Complex
Statistical details	Joint ICA was performed on structural and functional contrast images for each of the two contrasts using FIT 2.0b. Seven components were derived, of which 2 were further investigated since their loadings correlated with relevant behavioral measures. Functional components were thresholded at p < .001, cluster-corrected for multiple comparisons, minimum cluster extent = 1.27 cc. Component 1 was considered a "semantics component" because it correlated with the semantic behavioral measure and not with either of the two syntactic measures. This component did not have any anatomical aspect to it. Component 2 was considered a "syntax component" because it correlated with both syntactic behavioral measures and not with the semantic measure. This conceptualization seems somewhat speculative, given that WPE NP and WPE AP are rather indirect measures of syntactic and semantic processing. Component 2 involved damage to left frontal and insular cortex, and underlying dorsal white matter.
Findings	Other
Findings notes	Contrast 1 loaded primarily on the R STG for component 1 (the "semantics component") and on the L ITG for component 2 (the "syntax component").

Complex analysis 2

First level contrast	Listening to grammatical but meaningless sentences and detecting a target word vs rest
Analysis class	Cross-sectional correlation with language or other measure
Group(s)	Aphasia
Covariate	See statistical details
Is the second level contrast valid in terms of the group(s), time point(s), and measures involved?	Yes
Is accuracy matched across the second level contrast?	Unknown, not reported
Is reaction time matched across the second level contrast?	Unknown, not reported
Behavioral data notes	—
Type of analysis	Complex
Statistical details	Joint ICA was performed on structural and functional contrast images for each of the two contrasts using FIT 2.0b. Seven components were derived, of which 2 were further investigated since their loadings correlated with relevant behavioral measures. Functional

	components were thresholded at p < .001, cluster-corrected for multiple comparisons, minimum cluster extent = 1.27 cc. Component 1 was considered a "semantics component" because it correlated with the semantic behavioral measure and not with either of the two syntactic measures. This component did not have any anatomical aspect to it. Component 2 was considered a "syntax component" because it correlated with both syntactic behavioral measures and not with the semantic measure. This conceptualization seems somewhat speculative, given that WPE NP and WPE AP are rather indirect measures of syntactic and semantic processing. Component 2 involved damage to left frontal and insular cortex, and underlying dorsal white matter.
Findings	Other
Findings notes	Contrast 2 loaded primarily on the R posterior STG for component 1 (the "semantics component") and on the L posterior STG and L IFG for component 2 (the "syntax component").

Notes

Excluded analyses	—

Szaflarski et al. (2013)

Reference

Authors	Szaflarski JP, Allendorfer JB, Banks C, Vannest J, Holland SK
Title	Recovered vs. not-recovered from post-stroke aphasia: the contributions from the dominant and non-dominant hemispheres
Reference	*Restor Neurol Neurosci* 2013; 31: 347-360
PMID	23482065
DOI	10.3233/rnn-120267

Participants

Language	US English
Inclusion criteria	—
Number of individuals with aphasia	27
Number of control participants	0
Were any of the participants included in any previous studies?	No
Is age reported for patients and controls, and matched?	Yes (recovered: mean 50 ± 13 years; non-recovered: mean 51 ± 13 years)
Is sex reported for patients and controls, and matched?	Yes (males: 15; females: 12)
Is handedness reported for patients and controls, and matched?	Yes (right: 27; left: 0)
Is time post stroke onset reported and appropriate to the study design?	Yes (recovered: mean 2.1 ± 2.1 years; non-recovered: mean 4.9 ± 3.1 years)
To what extent is the nature of aphasia characterized?	Severity only
Language evaluation	TT, BNT, semantic fluency, phonemic fluency, PPVT, complex ideation subtest of BDAE
Aphasia severity	Recovered: TT mean 43 ± 1, ≥ 41; non-recovered: TT mean 23 ± 12, < 41
Aphasia type	Not stated
First stroke only?	Not stated
Stroke type	Not stated
To what extent is the lesion distribution characterized?	Lesion overlay
Lesion extent	Recovered: median 9.2 cc, range 2.2-26.5 cc; non-recovered: median 74 cc, range 5.1-206.0 cc
Lesion location	L MCA
Participants notes	—

Imaging

Modality	fMRI
Is the study cross-sectional or longitudinal?	Cross-sectional
If longitudinal, at what time point(s) were imaging data acquired?	—
If longitudinal, was there any intervention between the time points?	—
Is the scanner described?	No (Phillips 3 Tesla; model not stated)
Is the timing of stimulus presentation and image acquisition clearly described and appropriate?	Yes
Design type	Block
Total images acquired	330
Are the imaging acquisition parameters, including coverage, adequately described and appropriate?	Yes (whole brain)
Is preprocessing and intrasubject coregistration adequately described and appropriate?	Yes
Is first level model fitting adequately described and appropriate?	Yes
Is intersubject normalization adequately described and appropriate?	Yes
Imaging notes	—

Conditions

Are the conditions clearly described?	Yes

Condition	Response type	Repetitions	All groups could do?	All individuals could do?
semantic decision	Button press	10	No	No
tone decision	Button press	12	No	No

Conditions notes	—

Contrasts

Are the contrasts clearly described?	Yes

Contrast 1: semantic decision vs tone decision

Language condition	Semantic decision
Control condition	Tone decision
Are the conditions matched for visual demands?	Yes
Are the conditions matched for auditory demands?	Yes
Are the conditions matched for motor demands?	Yes
Are the conditions matched for cognitive/executive demands?	Yes
Is accuracy matched between the language and control tasks for all relevant groups?	Appear mismatched
Is reaction time matched between the language and control tasks for all relevant groups?	Unknown, not reported
Behavioral data notes	Accuracy appears similar in the non-recovered group, but not in the recovered group
Are control data reported in this paper or another that is referenced?	Yes
Does the contrast selectively activate plausible relevant language regions in the control group?	Yes
Are activations lateralized in the control data?	Yes
Control activation notes	Control data in Kim et al. (2011) and Szaflarski et al. (2008); L frontal and temporal, plus other semantic regions
Contrast notes	—

Analyses

Are the analyses clearly described?	Yes

Voxelwise analysis 1

First level contrast	Semantic decision vs tone decision
Analysis class	Cross-sectional between two groups with aphasia
Group(s)	Aphasia not recovered (n = 18) vs recovered (n = 9)
Covariate	—
Is the second level contrast valid in terms of the group(s), time point(s), and measures involved?	Yes
Is accuracy matched across the second level contrast?	Appear mismatched
Is reaction time matched across the second level contrast?	Unknown, not reported
Behavioral data notes	Interaction of group by condition not reported; non-recovered patients were significantly less accurate only on the semantic decision condition, but they actually showed a smaller difference between conditions than the recovered patients
Type of analysis	Voxelwise
Search volume	Whole brain
Correction for multiple comparisons	Clusterwise correction based on 3dClustSim
Software	AFNI
Voxelwise p	.05
Cluster extent	4.16 cc
Statistical details	Cluster-defining threshold (CDT) p < 0.05 too lenient
Findings	↑ L dorsolateral prefrontal cortex<br>↑ L superior parietal<br>↑ L cerebellum<br>↑ R cerebellum<br>↓ R posterior STG
Findings notes	—

ROI analysis 1

First level contrast	Semantic decision vs tone decision
Analysis class	Cross-sectional correlation with language or other measure
Group(s)	Aphasia (recovered and non-recovered)
Covariate	BNT
Is the second level contrast valid in terms of the group(s), time point(s), and measures involved?	Yes
Is accuracy matched across the second level contrast?	Unknown, not reported
Is reaction time matched across the second level contrast?	Unknown, not reported
Behavioral data notes	—
Type of analysis	Regions of interest (ROI)
ROI type	Functional
How many ROIs are there?	4
What are the ROI(s)?	(1) bilateral cerebellum; (2) R pSTG; (3) L superior parietal lobule; (4) L superior frontal gyrus
How are the ROI(s) defined?	Regions that were differentially recruited between recovered and non-recovered patients; average t scores from individual SPMs
Correction for multiple comparisons	Familywise error (FWE)
Statistical details	Circular because defined based on recovered status
Findings	↑ L dorsolateral prefrontal cortex
Findings notes	—

ROI analysis 2

First level contrast	Semantic decision vs tone decision

Analysis class	Cross-sectional correlation with language or other measure
Group(s)	Aphasia (recovered and non-recovered)
Covariate	Semantic fluency
Is the second level contrast valid in terms of the group(s), time point(s), and measures involved?	Yes
Is accuracy matched across the second level contrast?	Unknown, not reported
Is reaction time matched across the second level contrast?	Unknown, not reported
Behavioral data notes	—
Type of analysis	Regions of interest (ROI)
ROI type	Functional
How many ROIs are there?	4
What are the ROI(s)?	(1) bilateral cerebellum; (2) R pSTG; (3) L superior parietal lobule; (4) L superior frontal gyrus
How are the ROI(s) defined?	Regions that were differentially recruited between recovered and non-recovered patients; average t scores from individual SPMs
Correction for multiple comparisons	Familywise error (FWE)
Statistical details	Circular because defined based on recovered status
Findings	↑ L dorsolateral prefrontal cortex
Findings notes	—

ROI analysis 3

First level contrast	Semantic decision vs tone decision
Analysis class	Cross-sectional correlation with language or other measure
Group(s)	Aphasia (recovered and non-recovered)
Covariate	Single word comprehension (PPVT)
Is the second level contrast valid in terms of the group(s), time point(s), and measures involved?	Yes
Is accuracy matched across the second level contrast?	Unknown, not reported
Is reaction time matched across the second level contrast?	Unknown, not reported
Behavioral data notes	—
Type of analysis	Regions of interest (ROI)
ROI type	Functional
How many ROIs are there?	4
What are the ROI(s)?	(1) bilateral cerebellum; (2) R pSTG; (3) L superior parietal lobule; (4) L superior frontal gyrus
How are the ROI(s) defined?	Regions that were differentially recruited between recovered and non-recovered patients; average t scores from individual SPMs
Correction for multiple comparisons	Familywise error (FWE)
Statistical details	Circular because defined based on recovered status
Findings	↑ L dorsolateral prefrontal cortex
Findings notes	—

ROI analysis 4

First level contrast	Semantic decision vs tone decision
Analysis class	Cross-sectional correlation with language or other measure
Group(s)	Aphasia (recovered and non-recovered)
Covariate	BDAE complex ideation subtest
Is the second level contrast valid in terms of the group(s), time point(s), and measures involved?	Yes
Is accuracy matched across the second level contrast?	Unknown, not reported
Is reaction time matched across the second level contrast?	Unknown, not reported

Behavioral data notes	—
Type of analysis	Regions of interest (ROI)
ROI type	Functional
How many ROIs are there?	4
What are the ROI(s)?	(1) bilateral cerebellum; (2) R pSTG; (3) L superior parietal lobule; (4) L superior frontal gyrus
How are the ROI(s) defined?	Regions that were differentially recruited between recovered and non-recovered patients; average t scores from individual SPMs
Correction for multiple comparisons	Familywise error (FWE)
Statistical details	Circular because defined based on recovered status
Findings	↑ L dorsolateral prefrontal cortex
Findings notes	—

ROI analysis 5

First level contrast	Semantic decision vs tone decision
Analysis class	Cross-sectional correlation with language or other measure
Group(s)	Aphasia (recovered and non-recovered)
Covariate	Phonemic fluency
Is the second level contrast valid in terms of the group(s), time point(s), and measures involved?	Yes
Is accuracy matched across the second level contrast?	Unknown, not reported
Is reaction time matched across the second level contrast?	Unknown, not reported
Behavioral data notes	—
Type of analysis	Regions of interest (ROI)
ROI type	Functional
How many ROIs are there?	4
What are the ROI(s)?	(1) bilateral cerebellum; (2) R pSTG; (3) L superior parietal lobule; (4) L superior frontal gyrus
How are the ROI(s) defined?	Regions that were differentially recruited between recovered and non-recovered patients; average t scores from individual SPMs
Correction for multiple comparisons	Familywise error (FWE)
Statistical details	Circular because defined based on recovered status
Findings	↓ R posterior STG
Findings notes	—

ROI analysis 6

First level contrast	Semantic decision vs tone decision
Analysis class	Cross-sectional correlation with language or other measure
Group(s)	Aphasia (recovered and non-recovered)
Covariate	Semantic decision accuracy
Is the second level contrast valid in terms of the group(s), time point(s), and measures involved?	Yes
Is accuracy matched across the second level contrast?	Accuracy is covariate
Is reaction time matched across the second level contrast?	Unknown, not reported
Behavioral data notes	—
Type of analysis	Regions of interest (ROI)
ROI type	Functional
How many ROIs are there?	4
What are the ROI(s)?	(1) bilateral cerebellum; (2) R pSTG; (3) L superior parietal lobule; (4) L superior frontal gyrus
How are the ROI(s) defined?	Regions that were differentially recruited between recovered and non-recovered patients; average t scores from individual SPMs
Correction for multiple comparisons	Familywise error (FWE)
Statistical details	Circular because defined based on recovered status

Findings	None
Findings notes	—

Notes

Excluded analyses	—

Thiel et al. (2013)

Reference

Authors	Thiel A, Hartmann A, Rubi-Fessen I, Anglade C, Kracht L, Weiduschat N, Kessler J, Rommel T, Heiss WD
Title	Effects of noninvasive brain stimulation on language networks and recovery in early poststroke aphasia
Reference	*Stroke* 2013; 44: 2240-2246
PMID	23813984
DOI	10.1161/strokeaha.111.000574

Participants

Language	German
Inclusion criteria	—
Number of individuals with aphasia	24 (plus 6 excluded: 4 did not tolerate MRI or PET scans; 2 TMS device was defective)
Number of control participants	0
Were any of the participants included in any previous studies?	No
Is age reported for patients and controls, and matched?	Yes (rTMS group: mean 69.8 ± 8.0 years; sham group: mean 71.2 ± 7.8 years)
Is sex reported for patients and controls, and matched?	No
Is handedness reported for patients and controls, and matched?	Yes (right: 24; left: 0)
Is time post stroke onset reported and appropriate to the study design?	Yes (rTMS group: mean 37.5 ± 18.5 days; sham group: mean 50.6 ± 22.6 days)
To what extent is the nature of aphasia characterized?	Severity and type
Language evaluation	AAT
Aphasia severity	T1: rTMS group: AAT sum of scores mean 251.5 ± 32.4; sham group: mean 251.1 ± 39.5; T2 not stated
Aphasia type	T1: rTMS group: 7 Wernicke's, 3 amnestic, 2 global, 1 Broca's; sham group: 5 Wernicke's, 3 Broca's, 2 global, 1 amnestic; T2: not stated
First stroke only?	Yes
Stroke type	Ischemic only
To what extent is the lesion distribution characterized?	Individual lesions
Lesion extent	RTMS group: 233 ± 197 cc; sham group: 244 ± 243 cc; lesion extent in images appears much smaller than the stated volumes
Lesion location	L MCA
Participants notes	—

Imaging

Modality	PET (rCBF)
Is the study cross-sectional or longitudinal?	Longitudinal—mixed
If longitudinal, at what time point(s) were imaging data acquired?	T1: pre-treatment/subacute (rTMS group: mean 37.5 ± 18.5 days post onset; sham group: mean 50.6 ± 22.6 days post onset); T2 post-treatment, ~2.5 weeks later

If longitudinal, was there any intervention between the time points?	RTMS group: inhibitory rTMS over the R IFG pars triangularis + SLT for 45 minutes/day, 5 days/week, 2 weeks; control group: sham TMS + SLT
Is the scanner described?	Yes (CTI-Siemens ECAT EXACT HR)
Is the timing of stimulus presentation and image acquisition clearly described and appropriate?	Yes
Design type	PET
Total images acquired	8
Are the imaging acquisition parameters, including coverage, adequately described and appropriate?	Yes (whole brain)
Is preprocessing and intrasubject coregistration adequately described and appropriate?	Yes
Is first level model fitting adequately described and appropriate?	Yes
Is intersubject normalization adequately described and appropriate?	No (lesion impact not addressed)
Imaging notes	—

Conditions

Are the conditions clearly described?	Yes

Condition	Response type	Repetitions	All groups could do?	All individuals could do?
verb generation	Word (overt)	4	Unknown	Unknown
rest	None	4	N/A	N/A

Conditions notes	—

Contrasts

Are the contrasts clearly described?	Yes

Contrast 1: verb generation vs rest

Language condition	Verb generation
Control condition	Rest
Are the conditions matched for visual demands?	Yes
Are the conditions matched for auditory demands?	No
Are the conditions matched for motor demands?	No
Are the conditions matched for cognitive/executive demands?	No
Is accuracy matched between the language and control tasks for all relevant groups?	N/A, tasks not comparable
Is reaction time matched between the language and control tasks for all relevant groups?	N/A, tasks not comparable
Behavioral data notes	—
Are control data reported in this paper or another that is referenced?	Somewhat
Does the contrast selectively activate plausible relevant language regions in the control group?	Unknown
Are activations lateralized in the control data?	Unknown
Control activation notes	Cites Weiduschat et al. (2011) which in turn cites Herholz et al. (1996) which provides some minimal control data
Contrast notes	—

Analyses

Are the analyses clearly described?	Yes

Voxelwise analysis 1

First level contrast	Verb generation vs rest

Analysis class	Longitudinal between two groups with aphasia
Group(s)	(Aphasia with rTMS (n = 13) T2 vs T1) vs (aphasia with sham (n = 11) T2 vs T1)
Covariate	—
Is the second level contrast valid in terms of the group(s), time point(s), and measures involved?	Yes
Is accuracy matched across the second level contrast?	Unknown, not reported
Is reaction time matched across the second level contrast?	Unknown, not reported
Behavioral data notes	—
Type of analysis	Voxelwise
Search volume	Whole brain
Correction for multiple comparisons	No direct comparison
Software	SPM8
Voxelwise p	—
Cluster extent	—
Statistical details	Qualitative comparison on p. 2244
Findings	↑ L IFG<br>↑ L posterior STG/STS/MTG<br>↓ R IFG<br>↓ R posterior STG/STS/MTG
Findings notes	Approximate interpretation of qualitative patterns shown in Figure 3; T1 R lateralization surprising relative to other findings from this group

ROI analysis 1

First level contrast	Verb generation vs rest
Analysis class	Longitudinal between two groups with aphasia
Group(s)	(Aphasia with rTMS (n = 13) T2 vs T1) vs (aphasia with sham (n = 11) T2 vs T1)
Covariate	—
Is the second level contrast valid in terms of the group(s), time point(s), and measures involved?	Yes
Is accuracy matched across the second level contrast?	Unknown, not reported
Is reaction time matched across the second level contrast?	Unknown, not reported
Behavioral data notes	—
Type of analysis	Region of interest (ROI)
ROI type	Laterality indi(ces)
How many ROIs are there?	1
What are the ROI(s)?	Language network LI
How are the ROI(s) defined?	
Correction for multiple comparisons	One only
Statistical details	Actual LIs are not reported, only change in LI
Findings	↑ LI (language network)
Findings notes	T1 R lateralization surprising relative to other findings from this group

ROI analysis 2

First level contrast	Verb generation vs rest
Analysis class	Longitudinal correlation with language or other measure
Group(s)	Aphasia T2 vs T1
Covariate	Δ AAT total score
Is the second level contrast valid in terms of the group(s), time point(s), and measures involved?	Yes
Is accuracy matched across the second level contrast?	Unknown, not reported
Is reaction time matched across the second level	Unknown, not reported

contrast?	
Behavioral data notes	—
Type of analysis	Region of interest (ROI)
ROI type	Laterality indi(ces)
How many ROIs are there?	1
What are the ROI(s)?	Language network LI
How are the ROI(s) defined?	
Correction for multiple comparisons	One only
Statistical details	Model did not include treatment group (rTMS vs sham)
Findings	↑ LI (language network)
Findings notes	Patients who improved more showed a greater leftward shift of activation; T1 R lateralization surprising relative to other findings from this group

Notes

Excluded analyses	—

Abel et al. (2014)

Reference

Authors	Abel S, Weiller C, Huber W, Willmes K
Title	Neural underpinnings for model-oriented therapy of aphasic word production
Reference	*Neuropsychologia* 2014; 57: 154-165
PMID	24686092
DOI	10.1016/j.neuropsychologia.2014.03.010

Participants

Language	German
Inclusion criteria	Anomia; no severe AoS or dysarthria
Number of individuals with aphasia	14 (plus 9 excluded: 4 for ceiling performance; 5 for technical problems)
Number of control participants	0
Were any of the participants included in any previous studies?	No
Is age reported for patients and controls, and matched?	Yes (median 48 years, range 35-74 years)
Is sex reported for patients and controls, and matched?	Yes (males: 10; females: 4)
Is handedness reported for patients and controls, and matched?	Yes (right: 14; left: 0)
Is time post stroke onset reported and appropriate to the study design?	Yes (median 41 months, range 11-72 months)
To what extent is the nature of aphasia characterized?	Type only
Language evaluation	AAT
Aphasia severity	Not stated
Aphasia type	8 Broca's, 3 Wernicke's, 1 fluent non-classifiable, 1 global, 1 transcortical sensory
First stroke only?	Yes
Stroke type	Mixed etiologies
To what extent is the lesion distribution characterized?	Lesion overlay
Lesion extent	Not stated
Lesion location	L MCA; 2 also had ACA
Participants notes	—

Imaging

Modality	fMRI
Is the study cross-sectional or longitudinal?	Longitudinal—chronic treatment
If longitudinal, at what time point(s) were imaging data acquired?	T1: pre-treatment/chronic; T2: post-treatment, ~6 weeks later (labeled T2 and T3 in paper)
If longitudinal, was there any intervention between the time points?	Lexical therapy, alternating between weeks with phonological and semantic treatment, 4 weeks; 60 out of the 132 items were trained
Is the scanner described?	Yes (Philips Achieva 3 Tesla)
Is the timing of stimulus presentation and image acquisition clearly described and appropriate?	No* (moderate limitation) (trials too close together (~8 s) and insufficient jitter (1-3 s) for event-related design)
Design type	Event-related
Total images acquired	560
Are the imaging acquisition parameters, including coverage, adequately described and appropriate?	Yes (whole brain)
Is preprocessing and intrasubject coregistration adequately described and appropriate?	Yes
Is first level model fitting adequately described and appropriate?	Yes
Is intersubject normalization adequately described and appropriate?	No (lesion impact not addressed)
Imaging notes	—

Conditions

Are the conditions clearly described?	Yes

Condition	Response type	Repetitions	All groups could do?	All individuals could do?
picture naming (semantic trained items)	Word (overt)	30	Yes	Unknown
picture naming (phonological trained items)	Word (overt)	30	Yes	Unknown
picture naming (untrained items)	Word (overt)	30	Yes	Unknown
picture naming (already known items)	Word (overt)	42	Yes	Unknown
rest	None	implicit baseline	N/A	N/A

Conditions notes	—

Contrasts

Are the contrasts clearly described?	Yes

Contrast 1: picture naming (all conditions) vs rest

Language condition	Picture naming (all conditions)
Control condition	Rest
Are the conditions matched for visual demands?	No
Are the conditions matched for auditory demands?	No
Are the conditions matched for motor demands?	No
Are the conditions matched for cognitive/executive demands?	No
Is accuracy matched between the language and control tasks for all relevant groups?	N/A, tasks not comparable
Is reaction time matched between the language and control tasks for all relevant groups?	N/A, tasks not comparable
Behavioral data notes	—
Are control data reported in this paper or another that is referenced?	No
Does the contrast selectively activate plausible relevant language regions in the control group?	Unknown
Are activations lateralized in the control data?	Unknown

Control activation notes	But see control data reported in a subsequent paper (Abel et al., 2015)
Contrast notes	—

Contrast 2: picture naming (trained items) vs picture naming (untrained items)

Language condition	Picture naming (trained items)
Control condition	Picture naming (untrained items)
Are the conditions matched for visual demands?	Yes
Are the conditions matched for auditory demands?	Yes
Are the conditions matched for motor demands?	Yes
Are the conditions matched for cognitive/executive demands?	Yes
Is accuracy matched between the language and control tasks for all relevant groups?	No, different
Is reaction time matched between the language and control tasks for all relevant groups?	Unknown, not reported
Behavioral data notes	Trained items improved more than untrained items
Are control data reported in this paper or another that is referenced?	No
Does the contrast selectively activate plausible relevant language regions in the control group?	Unknown
Are activations lateralized in the control data?	Unknown
Control activation notes	—
Contrast notes	—

Contrast 3: picture naming (semantic trained items) vs picture naming (phonological trained items)

Language condition	Picture naming (semantic trained items)
Control condition	Picture naming (phonological trained items)
Are the conditions matched for visual demands?	Yes
Are the conditions matched for auditory demands?	Yes
Are the conditions matched for motor demands?	Yes
Are the conditions matched for cognitive/executive demands?	Yes
Is accuracy matched between the language and control tasks for all relevant groups?	Yes, matched
Is reaction time matched between the language and control tasks for all relevant groups?	Unknown, not reported
Behavioral data notes	—
Are control data reported in this paper or another that is referenced?	No
Does the contrast selectively activate plausible relevant language regions in the control group?	Unknown
Are activations lateralized in the control data?	Unknown
Control activation notes	—
Contrast notes	—

Analyses

Are the analyses clearly described?	Yes

Voxelwise analysis 1

First level contrast	Picture naming (all conditions) vs rest
Analysis class	Cross-sectional correlation with language or other measure
Group(s)	Aphasia T1
Covariate	Subsequent Δ (T2 vs T1) picture naming
Is the second level contrast valid in terms of the group(s), time point(s), and measures involved?	Somewhat (T1 behavioral measure should be included in model)

Is accuracy matched across the second level contrast?	Accuracy is covariate
Is reaction time matched across the second level contrast?	Unknown, not reported
Behavioral data notes	—
Type of analysis	Voxelwise
Search volume	Whole brain
Correction for multiple comparisons	Clusterwise correction based on cluster_threshold_beta
Software	SPM8
Voxelwise p	.01
Cluster extent	11 voxels (size not stated)
Statistical details	—
Findings	↑ L IFG pars opercularis<br>↓ R basal ganglia
Findings notes	—

Voxelwise analysis 2

First level contrast	Picture naming (all conditions) vs rest
Analysis class	Longitudinal correlation with language or other measure
Group(s)	Aphasia T2 vs T1
Covariate	Δ picture naming accuracy
Is the second level contrast valid in terms of the group(s), time point(s), and measures involved?	Yes
Is accuracy matched across the second level contrast?	Accuracy is covariate
Is reaction time matched across the second level contrast?	Unknown, not reported
Behavioral data notes	—
Type of analysis	Voxelwise
Search volume	Whole brain
Correction for multiple comparisons	Clusterwise correction based on cluster_threshold_beta
Software	SPM8
Voxelwise p	.01
Cluster extent	11 voxels (size not stated)
Statistical details	—
Findings	↑ L somato-motor<br>↑ L inferior parietal lobule<br>↑ L supramarginal gyrus<br>↑ L posterior STS<br>↑ L posterior MTG<br>↑ L occipital
Findings notes	—

Voxelwise analysis 3

First level contrast	Picture naming (trained items) vs picture naming (untrained items)
Analysis class	Longitudinal change in aphasia
Group(s)	Aphasia T2 vs T1
Covariate	—
Is the second level contrast valid in terms of the group(s), time point(s), and measures involved?	Yes
Is accuracy matched across the second level contrast?	No, different
Is reaction time matched across the second level contrast?	Unknown, not reported
Behavioral data notes	Trained items improved more than untrained items
Type of analysis	Voxelwise

Search volume	Whole brain
Correction for multiple comparisons	Clusterwise correction based on cluster_threshold_beta
Software	SPM8
Voxelwise p	.01
Cluster extent	11 voxels (size not stated)
Statistical details	—
Findings	↑ L precuneus<br>↑ L posterior STG<br>↑ L Heschl's gyrus<br>↑ L mid temporal<br>↑ L posterior cingulate<br>↑ L thalamus<br>↑ R ventral precentral/inferior frontal junction<br>↑ R somato-motor<br>↑ R Heschl's gyrus<br>↑ R posterior cingulate<br>↑ R thalamus<br>↑ R basal ganglia
Findings notes	—

Voxelwise analysis 4

First level contrast	Picture naming (semantic trained items) vs picture naming (phonological trained items)
Analysis class	Longitudinal change in aphasia
Group(s)	Aphasia T2 vs T1
Covariate	—
Is the second level contrast valid in terms of the group(s), time point(s), and measures involved?	Yes
Is accuracy matched across the second level contrast?	Yes, matched
Is reaction time matched across the second level contrast?	Unknown, not reported
Behavioral data notes	No differential effects for semantic vs phonological trained items
Type of analysis	Voxelwise
Search volume	Whole brain
Correction for multiple comparisons	Clusterwise correction based on cluster_threshold_beta
Software	SPM8
Voxelwise p	.01
Cluster extent	11 voxels (size not stated)
Statistical details	—
Findings	↑ R superior parietal<br>↓ L dorsolateral prefrontal cortex<br>↓ L somato-motor<br>↓ L occipital<br>↓ L anterior cingulate<br>↓ L posterior cingulate<br>↓ R precuneus<br>↓ R occipital<br>↓ R anterior cingulate<br>↓ R posterior cingulate<br>↓ R hippocampus/MTL
Findings notes	—

Voxelwise analysis 5

First level contrast	Picture naming (all conditions) vs rest
Analysis class	Cross-sectional between two groups with aphasia
Group(s)	Aphasia with semantic impairment T1 (n = 8) vs with phonological impairment T1 (n = 6)
Covariate	—

Is the second level contrast valid in terms of the group(s), time point(s), and measures involved?	Yes
Is accuracy matched across the second level contrast?	Unknown, not reported
Is reaction time matched across the second level contrast?	Unknown, not reported
Behavioral data notes	—
Type of analysis	Voxelwise
Search volume	Whole brain
Correction for multiple comparisons	Clusterwise correction based on cluster_threshold_beta
Software	SPM8
Voxelwise p	.01
Cluster extent	11 voxels (size not stated)
Statistical details	—
Findings	↑ R IFG pars triangularis<br>↑ R dorsolateral prefrontal cortex
Findings notes	—

Voxelwise analysis 6

First level contrast	Picture naming (all conditions) vs rest
Analysis class	Longitudinal between two groups with aphasia
Group(s)	(Aphasia with semantic impairment (n = 8) T2 vs T1) vs (aphasia with phonological impairment (n = 6) T2 vs T1)
Covariate	—
Is the second level contrast valid in terms of the group(s), time point(s), and measures involved?	Yes
Is accuracy matched across the second level contrast?	No, different
Is reaction time matched across the second level contrast?	Unknown, not reported
Behavioral data notes	Phonological patients showed more improvement on trained items
Type of analysis	Voxelwise
Search volume	Whole brain
Correction for multiple comparisons	Clusterwise correction based on cluster_threshold_beta
Software	SPM8
Voxelwise p	.01
Cluster extent	11 voxels (size not stated)
Statistical details	—
Findings	↑ L somato-motor<br>↑ L Heschl's gyrus<br>↑ L anterior temporal<br>↑ L occipital<br>↑ L thalamus<br>↑ L basal ganglia<br>↑ R somato-motor<br>↓ L IFG pars opercularis
Findings notes	—

Voxelwise analysis 7

First level contrast	Picture naming (all conditions) vs rest
Analysis class	Longitudinal change in aphasia
Group(s)	Aphasia with semantic impairment (n = 8) T2 vs T1
Covariate	—
Is the second level contrast valid in terms of the group(s), time point(s), and measures involved?	Yes
Is accuracy matched across the second level	No, different

contrast?	
Is reaction time matched across the second level contrast?	Unknown, not reported
Behavioral data notes	—
Type of analysis	Voxelwise
Search volume	Whole brain
Correction for multiple comparisons	Clusterwise correction based on cluster_threshold_beta
Software	SPM8
Voxelwise p	.01
Cluster extent	11 voxels (size not stated)
Statistical details	—
Findings	↑ L basal ganglia
Findings notes	—

Voxelwise analysis 8

First level contrast	Picture naming (all conditions) vs rest
Analysis class	Longitudinal change in aphasia
Group(s)	Aphasia with phonological impairment (n = 6) T2 vs T1
Covariate	—
Is the second level contrast valid in terms of the group(s), time point(s), and measures involved?	Yes
Is accuracy matched across the second level contrast?	No, different
Is reaction time matched across the second level contrast?	Unknown, not reported
Behavioral data notes	—
Type of analysis	Voxelwise
Search volume	Whole brain
Correction for multiple comparisons	Clusterwise correction based on cluster_threshold_beta
Software	SPM8
Voxelwise p	.01
Cluster extent	11 voxels (size not stated)
Statistical details	—
Findings	None
Findings notes	—

Notes

Excluded analyses	—

Benjamin et al. (2014)

Reference

Authors	Benjamin ML, Towler S, Garcia A, Park H, Sudhyadhom A, Harnish SM, McGregor KM, Zlatar Z, Reilly JJ, Rosenbek JC, Gonzalez LJ, Crosson B
Title	A behavioral manipulation engages right frontal cortex during aphasia therapy
Reference	*Neurorehabil Neural Repair* 2014; 28: 545-553
PMID	24407914
DOI	10.1177/1545968313517754

Participants

Language	US English
Inclusion criteria	"at least minimal evidence of non-fluent output"; lesion including precentral gyrus or

	underlying white matter
Number of individuals with aphasia	14
Number of control participants	0
Were any of the participants included in any previous studies?	No
Is age reported for patients and controls, and matched?	Yes (intention group: mean 72.1 ± 10.5 years; control group: mean 63.0 ± 9.2 years)
Is sex reported for patients and controls, and matched?	Yes (males: 8; females: 6)
Is handedness reported for patients and controls, and matched?	Yes (right: 14; left: 0)
Is time post stroke onset reported and appropriate to the study design?	Yes (intention group: mean 37.4 ± 33.5 months, range 12-87 months; control group: 38.1 ± 37.4 months, range 10-112 months)
To what extent is the nature of aphasia characterized?	Severity and type
Language evaluation	WAB, BNT, PPVT
Aphasia severity	Intention group: AQ mean 65.5 ± 8.3; control group: AQ mean 71.9 ± 11.9
Aphasia type	Intention group: 4 conduction, 2 Broca's, 1 anomic; control group: 4 anomic, 1 Broca's, 1 conduction, 1 transcortical motor
First stroke only?	No
Stroke type	Mixed etiologies
To what extent is the lesion distribution characterized?	Lesion overlay
Lesion extent	Not stated
Lesion location	L MCA, extending frontally at least into the precentral gyrus or underlying white matter
Participants notes	—

Imaging

Modality	fMRI
Is the study cross-sectional or longitudinal?	Longitudinal—chronic treatment
If longitudinal, at what time point(s) were imaging data acquired?	T1: pre-treatment/chronic; T2: post-treatment; T3: 3 months after the end of treatment
If longitudinal, was there any intervention between the time points?	Word finding therapy for both groups, but the intention group had to produce complex left hand movements, while the control group did not; note that groups were not directly compared in any imaging analyses
Is the scanner described?	Yes (Philips Achieva 3 Tesla)
Is the timing of stimulus presentation and image acquisition clearly described and appropriate?	No (total images acquired not stated)
Design type	Event-related
Total images acquired	not stated
Are the imaging acquisition parameters, including coverage, adequately described and appropriate?	Yes (whole brain)
Is preprocessing and intrasubject coregistration adequately described and appropriate?	No (not described)
Is first level model fitting adequately described and appropriate?	No (not described clearly)
Is intersubject normalization adequately described and appropriate?	No (lesion impact not addressed)
Imaging notes	—

Conditions

Are the conditions clearly described?	Yes

Condition	Response type	Repetitions	All groups could do?	All individuals could do?
word generation	Word (overt)	60	Unknown	Unknown
rest	None	implicit	N/A	N/A

	baseline

Conditions notes	—

Contrasts

Are the contrasts clearly described?	Yes

Contrast 1: word generation vs rest

Language condition	Word generation
Control condition	Rest
Are the conditions matched for visual demands?	No
Are the conditions matched for auditory demands?	No
Are the conditions matched for motor demands?	No
Are the conditions matched for cognitive/executive demands?	No
Is accuracy matched between the language and control tasks for all relevant groups?	N/A, tasks not comparable
Is reaction time matched between the language and control tasks for all relevant groups?	N/A, tasks not comparable
Behavioral data notes	—
Are control data reported in this paper or another that is referenced?	No
Does the contrast selectively activate plausible relevant language regions in the control group?	Unknown
Are activations lateralized in the control data?	Unknown
Control activation notes	—
Contrast notes	Contrast not described explicitly but there is only one possible contrast

Analyses

Are the analyses clearly described?	Yes

ROI analysis 1

First level contrast	Word generation vs rest
Analysis class	Longitudinal change in aphasia
Group(s)	Aphasia with intention treatment (n = 7) T2 vs T1
Covariate	—
Is the second level contrast valid in terms of the group(s), time point(s), and measures involved?	Yes
Is accuracy matched across the second level contrast?	Unknown, not reported
Is reaction time matched across the second level contrast?	Unknown, not reported
Behavioral data notes	—
Type of analysis	Regions of interest (ROI)
ROI type	Laterality indi(ces)
How many ROIs are there?	3
What are the ROI(s)?	(1) lateral frontal LI; (2) medial frontal LI; (3) posterior perisylvian LI
How are the ROI(s) defined?	
Correction for multiple comparisons	No correction
Statistical details	—
Findings	↓ LI (frontal)
Findings notes	Laterality shift for lateral frontal LI, not medial frontal LI

ROI analysis 2

First level contrast	Word generation vs rest

Analysis class	Longitudinal change in aphasia
Group(s)	Aphasia with intention treatment (n = 6) T3 vs T1
Covariate	—
Is the second level contrast valid in terms of the group(s), time point(s), and measures involved?	Yes
Is accuracy matched across the second level contrast?	Unknown, not reported
Is reaction time matched across the second level contrast?	Unknown, not reported
Behavioral data notes	—
Type of analysis	Regions of interest (ROI)
ROI type	Laterality indi(ces)
How many ROIs are there?	3
What are the ROI(s)?	(1) lateral frontal LI; (2) medial frontal LI; (3) posterior perisylvian LI
How are the ROI(s) defined?	
Correction for multiple comparisons	No correction
Statistical details	—
Findings	↓ LI (frontal)
Findings notes	Laterality shift for both lateral and medial frontal LIs

ROI analysis 3

First level contrast	Word generation vs rest
Analysis class	Longitudinal change in aphasia
Group(s)	Aphasia with control treatment (n = 7) T2 vs T1
Covariate	—
Is the second level contrast valid in terms of the group(s), time point(s), and measures involved?	Yes
Is accuracy matched across the second level contrast?	Unknown, not reported
Is reaction time matched across the second level contrast?	Unknown, not reported
Behavioral data notes	—
Type of analysis	Regions of interest (ROI)
ROI type	Laterality indi(ces)
How many ROIs are there?	3
What are the ROI(s)?	(1) lateral frontal LI; (2) medial frontal LI; (3) posterior perisylvian LI
How are the ROI(s) defined?	
Correction for multiple comparisons	No correction
Statistical details	—
Findings	None
Findings notes	—

ROI analysis 4

First level contrast	Word generation vs rest
Analysis class	Longitudinal change in aphasia
Group(s)	Aphasia with control treatment (n = 7) T3 vs T1
Covariate	—
Is the second level contrast valid in terms of the group(s), time point(s), and measures involved?	Yes
Is accuracy matched across the second level contrast?	Unknown, not reported
Is reaction time matched across the second level contrast?	Unknown, not reported
Behavioral data notes	—
Type of analysis	Regions of interest (ROI)

ROI type	Laterality indi(ces)
How many ROIs are there?	3
What are the ROI(s)?	(1) lateral frontal LI; (2) medial frontal LI; (3) posterior perisylvian LI
How are the ROI(s) defined?	
Correction for multiple comparisons	No correction
Statistical details	—
Findings	None
Findings notes	—

ROI analysis 5

First level contrast	Word generation vs rest
Analysis class	Longitudinal correlation with language or other measure
Group(s)	Aphasia with intention treatment (n = 7) T2 vs T1
Covariate	Δ category-member generation probe performance
Is the second level contrast valid in terms of the group(s), time point(s), and measures involved?	Yes
Is accuracy matched across the second level contrast?	Unknown, not reported
Is reaction time matched across the second level contrast?	Unknown, not reported
Behavioral data notes	—
Type of analysis	Regions of interest (ROI)
ROI type	Laterality indi(ces)
How many ROIs are there?	3
What are the ROI(s)?	(1) lateral frontal LI; (2) medial frontal LI; (3) posterior perisylvian LI
How are the ROI(s) defined?	
Correction for multiple comparisons	No correction
Statistical details	—
Findings	↓ LI (temporal)
Findings notes	—

ROI analysis 6

First level contrast	Word generation vs rest
Analysis class	Longitudinal correlation with language or other measure
Group(s)	Aphasia with control treatment (n = 7) T2 vs T1
Covariate	Δ category-member generation probe performance
Is the second level contrast valid in terms of the group(s), time point(s), and measures involved?	Yes
Is accuracy matched across the second level contrast?	Unknown, not reported
Is reaction time matched across the second level contrast?	Unknown, not reported
Behavioral data notes	—
Type of analysis	Regions of interest (ROI)
ROI type	Laterality indi(ces)
How many ROIs are there?	3
What are the ROI(s)?	(1) lateral frontal LI; (2) medial frontal LI; (3) posterior perisylvian LI
How are the ROI(s) defined?	
Correction for multiple comparisons	No correction
Statistical details	—
Findings	None
Findings notes	—

ROI analysis 7

First level contrast	Word generation vs rest

Analysis class	Longitudinal correlation with language or other measure
Group(s)	Aphasia with intention treatment (n = 7) T2 vs T1
Covariate	Δ picture naming probe performance
Is the second level contrast valid in terms of the group(s), time point(s), and measures involved?	Yes
Is accuracy matched across the second level contrast?	Unknown, not reported
Is reaction time matched across the second level contrast?	Unknown, not reported
Behavioral data notes	—
Type of analysis	Regions of interest (ROI)
ROI type	Laterality indi(ces)
How many ROIs are there?	3
What are the ROI(s)?	(1) lateral frontal LI; (2) medial frontal LI; (3) posterior perisylvian LI
How are the ROI(s) defined?	
Correction for multiple comparisons	No correction
Statistical details	—
Findings	None
Findings notes	—

ROI analysis 8

First level contrast	Word generation vs rest
Analysis class	Longitudinal correlation with language or other measure
Group(s)	Aphasia with control treatment (n = 7) T2 vs T1
Covariate	Δ picture naming probe performance
Is the second level contrast valid in terms of the group(s), time point(s), and measures involved?	Yes
Is accuracy matched across the second level contrast?	Unknown, not reported
Is reaction time matched across the second level contrast?	Unknown, not reported
Behavioral data notes	—
Type of analysis	Regions of interest (ROI)
ROI type	Laterality indi(ces)
How many ROIs are there?	3
What are the ROI(s)?	(1) lateral frontal LI; (2) medial frontal LI; (3) posterior perisylvian LI
How are the ROI(s) defined?	
Correction for multiple comparisons	No correction
Statistical details	—
Findings	None
Findings notes	—

Notes

Excluded analyses	SPM analysis in Figure 3, because the authors do not attempt to interpret it

Brownsett et al. (2014)

Reference

Authors	Brownsett SL, Warren JE, Geranmayeh F, Woodhead Z, Leech R, Wise RJ
Title	Cognitive control and its impact on recovery from aphasic stroke
Reference	*Brain* 2014; 137: 242-254
PMID	24163248

DOI	10.1093/brain/awt289

Participants

Language	UK English
Inclusion criteria	No involvement of ACA territory
Number of individuals with aphasia	16 (plus 3 excluded: 2 withdrew after attempting first scan; 1 had severe dysarthria)
Number of control participants	17
Were any of the participants included in any previous studies?	No
Is age reported for patients and controls, and matched?	Yes (mean 60 years, range 37-84 years)
Is sex reported for patients and controls, and matched?	Yes (males: 11; females: 5)
Is handedness reported for patients and controls, and matched?	Yes (right: 16; left: 0)
Is time post stroke onset reported and appropriate to the study design?	Yes (mean 4 years, range 6 months-11 years)
To what extent is the nature of aphasia characterized?	Not at all
Language evaluation	Not stated
Aphasia severity	Not stated
Aphasia type	Not stated, but all had auditory comprehension and repetition deficits, and all could at least attempt to repeat
First stroke only?	Not stated
Stroke type	Not stated
To what extent is the lesion distribution characterized?	Lesion overlay
Lesion extent	Not stated
Lesion location	L temporal and parietal cortex; 4 extended into the frontal lobe; no lesions involved ACA territory
Participants notes	—

Imaging

Modality	fMRI
Is the study cross-sectional or longitudinal?	Longitudinal—chronic treatment
If longitudinal, at what time point(s) were imaging data acquired?	Patients: T1: acclimatization/chronic (but used in some analyses); T2: pre-treatment/chronic (not stated how long after T1); T3: post-treatment/~4 weeks later; controls: T1: pre-training; T2: post-training/~2 weeks later
If longitudinal, was there any intervention between the time points?	Patients: home-based therapy consisting of auditory discrimination and repetition tasks for 3 or 4 weeks between T2 and T3; control: 2 weeks of similar training using noise vocoded speech
Is the scanner described?	Yes (Philips Intera 3 Tesla)
Is the timing of stimulus presentation and image acquisition clearly described and appropriate?	No* (moderate limitation) (timing of sentence presentation not described; sparse event-related design, but ITI of only 8 s and consistent linear order of listening and repetition trials could make it difficult to disentangle hemodynamic responses to listening and repeating trials)
Design type	Event-related
Total images acquired	168 (patients); 280 (controls)
Are the imaging acquisition parameters, including coverage, adequately described and appropriate?	Yes (whole brain)
Is preprocessing and intrasubject coregistration adequately described and appropriate?	Yes
Is first level model fitting adequately described and appropriate?	No* (moderate limitation) (consistent linear order of listening and repetition trials could make it difficult to disentangle hemodynamic responses to listening and repeating trials)
Is intersubject normalization adequately described and appropriate?	Yes
Imaging notes	sparse sampling; different task structure in controls (two repetition trials per listening trial) raises concerns about comparisons between groups

Conditions

Are the conditions clearly described?	No (paradigm was different in patients and controls, and is not described in sufficient detail for patients)

Condition	Response type	Repetitions	All groups could do?	All individuals could do?
listening to sentences	None	aphasia: not stated; control: 40	N/A	N/A
repeating sentences (sentence from previous trial)	Sentence (overt)	aphasia: not stated; control: 40	Yes	No
listening to noise vocoded sentences (control only)	None	40 (control)	N/A	N/A
repeating noise vocoded sentences (control only)	Sentence (overt)	80 (control)	Yes	Unknown
listening to segmented white noise	None	aphasia: not stated; control: 40	N/A	N/A

Conditions notes	In two patients, only single words were produced

Contrasts

Are the contrasts clearly described?	Yes

Contrast 1: listening to sentences vs listening to segmented white noise

Language condition	Listening to sentences
Control condition	Listening to segmented white noise
Are the conditions matched for visual demands?	Yes
Are the conditions matched for auditory demands?	Yes
Are the conditions matched for motor demands?	Yes
Are the conditions matched for cognitive/executive demands?	Yes
Is accuracy matched between the language and control tasks for all relevant groups?	N/A, no behavioral measure
Is reaction time matched between the language and control tasks for all relevant groups?	N/A, no timeable task
Behavioral data notes	—
Are control data reported in this paper or another that is referenced?	No
Does the contrast selectively activate plausible relevant language regions in the control group?	Unknown
Are activations lateralized in the control data?	Unknown
Control activation notes	—
Contrast notes	—

Contrast 2: listening to sentences (patients) or listening to noise vocoded sentences (controls) vs listening to segmented white noise

Language condition	Listening to sentences (patients) or listening to noise vocoded sentences (controls)
Control condition	Listening to segmented white noise
Are the conditions matched for visual demands?	Yes
Are the conditions matched for auditory demands?	Yes
Are the conditions matched for motor demands?	Yes
Are the conditions matched for cognitive/executive demands?	Yes
Is accuracy matched between the language and control tasks for all relevant groups?	N/A, no behavioral measure
Is reaction time matched between the language and control tasks for all relevant groups?	N/A, no timeable task

Behavioral data notes	—
Are control data reported in this paper or another that is referenced?	No
Does the contrast selectively activate plausible relevant language regions in the control group?	Unknown
Are activations lateralized in the control data?	Unknown
Control activation notes	—
Contrast notes	—

Analyses

Are the analyses clearly described?	Yes

Voxelwise analysis 1

First level contrast	Listening to sentences vs listening to segmented white noise
Analysis class	Cross-sectional aphasia vs control
Group(s)	Aphasia (T2 and T3) vs control (T1 and T2)
Covariate	—
Is the second level contrast valid in terms of the group(s), time point(s), and measures involved?	Yes
Is accuracy matched across the second level contrast?	No, different
Is reaction time matched across the second level contrast?	N/A, no timeable task
Behavioral data notes	Significant difference in accuracy of subsequent repetition
Type of analysis	Voxelwise
Search volume	Whole brain
Correction for multiple comparisons	Clusterwise correction with with GRFT and lenient voxelwise p
Software	FSL (FEAT 5.98)
Voxelwise p	~.01 (z > 2.3)
Cluster extent	Based on GRFT
Statistical details	—
Findings	↑ L insula<br>↑ L anterior cingulate<br>↑ R insula<br>↑ R anterior cingulate<br>↓ L SMA/medial prefrontal<br>↓ L precuneus<br>↓ L posterior cingulate<br>↓ R SMA/medial prefrontal<br>↓ R precuneus<br>↓ R posterior cingulate
Findings notes	Findings are approximate since description is partially in terms of networks; at the earlier time point only, patients also showed reduced activity in left ventral prefrontal cortex and right medial planum temporale

Voxelwise analysis 2

First level contrast	Listening to sentences (patients) or listening to noise vocoded sentences (controls) vs listening to segmented white noise
Analysis class	Cross-sectional aphasia vs control
Group(s)	Aphasia (T2 and T3) vs control (T1 and T2)
Covariate	—
Is the second level contrast valid in terms of the group(s), time point(s), and measures involved?	Yes
Is accuracy matched across the second level contrast?	Yes, matched
Is reaction time matched across the second level contrast?	N/A, no timeable task

Behavioral data notes	No significant difference in accuracy of subsequent repetition
Type of analysis	Voxelwise
Search volume	Whole brain
Correction for multiple comparisons	Clusterwise correction with with GRFT and lenient voxelwise p
Software	FSL (FEAT 5.98)
Voxelwise p	~.01 (z > 2.3)
Cluster extent	Based on GRFT
Statistical details	—
Findings	None
Findings notes	—

ROI analysis 1

First level contrast	Listening to sentences vs listening to segmented white noise
Analysis class	Cross-sectional correlation with language or other measure
Group(s)	Aphasia mean of T1, T2, T3
Covariate	Picture description score (CAT), mean of T1, T2, T3
Is the second level contrast valid in terms of the group(s), time point(s), and measures involved?	Yes
Is accuracy matched across the second level contrast?	Unknown, not reported
Is reaction time matched across the second level contrast?	N/A, no timeable task
Behavioral data notes	Referring to accuracy of subsequent repetition; correlation with picture description is not reported
Type of analysis	Region of interest (ROI)
ROI type	Functional
How many ROIs are there?	1
What are the ROI(s)?	Dorsal anterior cingulate cortex/midline superior frontal gyrus
How are the ROI(s) defined?	Contrast of listening to vocoded speech and listening to normal speech in controls
Correction for multiple comparisons	One only
Statistical details	Same result obtained with age and lesion volume included in the model
Findings	↑ L SMA/medial prefrontal<br>↑ L anterior cingulate<br>↑ R SMA/medial prefrontal<br>↑ R anterior cingulate
Findings notes	Increased activation of dACC/SFG was correlated with higher scores on picture description

Notes

Excluded analyses	Longitudinal analyses, since these were null findings that were not the focus of this paper

Mattioli et al. (2014)

Reference

Authors	Mattioli F, Ambrosi C, Mascaro L, Scarpazza C, Pasquali P, Frugoni M, Magoni M, Biagi L, Gasparotti R
Title	Early aphasia rehabilitation is associated with functional reactivation of the left inferior frontal gyrus: a pilot study
Reference	*Stroke* 2014; 45: 545-552
PMID	24309584
DOI	10.1161/strokeaha.113.003192

Participants

Language	Italian

Inclusion criteria	L MCA; comprehension mildly impaired
Number of individuals with aphasia	12
Number of control participants	10
Were any of the participants included in any previous studies?	No
Is age reported for patients and controls, and matched?	No (range 37-79 years; control ages not reported, though reported to be matched)
Is sex reported for patients and controls, and matched?	No (males: 7; females: 5; control sex not stated, but reported to be matched)
Is handedness reported for patients and controls, and matched?	Yes (right: 12; left: 0)
Is time post stroke onset reported and appropriate to the study design?	Yes (T1: mean 2.2 ± 1.3 days; T2: mean 16.2 ± 1.3 days; T3: mean 190 ± 25.5 days)
To what extent is the nature of aphasia characterized?	Comprehensive battery
Language evaluation	AAT, TT
Aphasia severity	T1: TT range 2-45; T2: TT range 6-48; T3: TT range 21-48
Aphasia type	T1: 8 Broca's, 3 anomic, 1 Wernicke's; T2: not stated
First stroke only?	Yes
Stroke type	Not stated
To what extent is the lesion distribution characterized?	Individual lesions
Lesion extent	Range 4.4-158.3 cc (possibly; units stated do not seem correct)
Lesion location	L MCA; lesions seem very small in Supplementary Figure 1, but are described as more extensive in Supplementary Table 1
Participants notes	Treated and untreated groups differed in severity at baseline, albeit not significantly

Imaging

Modality	fMRI
Is the study cross-sectional or longitudinal?	Longitudinal—mixed
If longitudinal, at what time point(s) were imaging data acquired?	T1: pre-treatment, mean 2.2 ± 1.3 days post onset; T2: post-treatment, mean 16.2 ± 1.3 days post onset; T3: mean 190 ± 25.5 days post onset
If longitudinal, was there any intervention between the time points?	6 patients were randomized to receive treatment focusing on verbal comprehension and lexical retrieval for 1 hour/day, 5 days/week between T1 and T2; no patient received treatment after T2
Is the scanner described?	Yes (Siemens Avanto 1.5 Tesla)
Is the timing of stimulus presentation and image acquisition clearly described and appropriate?	No (timing of stimuli not clearly described)
Design type	Event-related
Total images acquired	504
Are the imaging acquisition parameters, including coverage, adequately described and appropriate?	No (unclear; number of slices not stated)
Is preprocessing and intrasubject coregistration adequately described and appropriate?	Yes
Is first level model fitting adequately described and appropriate?	No (model fitting of noise "bip" not clearly described)
Is intersubject normalization adequately described and appropriate?	Yes
Imaging notes	—

Conditions

Are the conditions clearly described?	No (there is also mention of a noise "bip" that preceded each sentence but details are lacking)

Condition	Response type	Repetitions	All groups could do?	All individuals could do?
listening to sentences and making a plausibility judgment	Button press	56	Yes	Unknown

listening to reversed speech	None	56	N/A	N/A

Conditions notes	Half of the sentences were semantically anomalous

Contrasts

Are the contrasts clearly described?	No (see specific limitation(s) below)

Contrast 1: listening to sentences and making a plausibility judgment vs listening to reversed speech

Language condition	Listening to sentences and making a plausibility judgment
Control condition	Listening to reversed speech
Are the conditions matched for visual demands?	Yes
Are the conditions matched for auditory demands?	Yes
Are the conditions matched for motor demands?	No
Are the conditions matched for cognitive/executive demands?	No
Is accuracy matched between the language and control tasks for all relevant groups?	N/A, tasks not comparable
Is reaction time matched between the language and control tasks for all relevant groups?	N/A, tasks not comparable
Behavioral data notes	—
Are control data reported in this paper or another that is referenced?	Somewhat
Does the contrast selectively activate plausible relevant language regions in the control group?	Somewhat
Are activations lateralized in the control data?	Yes
Control activation notes	10 participants; quite lateralized activity centered on the anterior Sylvian fissure
Contrast notes	It is mentioned that "noise" was also included on the negative side of the contrast; it is unclear if this refers to the noise "bip", which would be inappropriate

Analyses

Are the analyses clearly described?	No* (moderate limitation) (see specific limitation(s) below)

Voxelwise analysis 1

First level contrast	Listening to sentences and making a plausibility judgment vs listening to reversed speech
Analysis class	Cross-sectional between two groups with aphasia
Group(s)	Aphasia treated T2 (n = 6) vs untreated T2 (n = 6)
Covariate	—
Is the second level contrast valid in terms of the group(s), time point(s), and measures involved?	Somewhat (groups were different but not due to treatment)
Is accuracy matched across the second level contrast?	Yes, matched
Is reaction time matched across the second level contrast?	Unknown, not reported
Behavioral data notes	—
Type of analysis	Voxelwise
Search volume	Whole brain
Correction for multiple comparisons	Clusterwise correction based on arbitrary cluster extent
Software	BrainVoyager QX 1.9
Voxelwise p	.001
Cluster extent	0.16 cc
Statistical details	Methods report cluster extent threshold (we assume this was done), but figure caption states uncorrected
Findings	↑ L IFG pars opercularis<br>↑ L IFG pars triangularis<br>↑ L SMA/medial prefrontal<br>↑ L angular gyrus

	↑ R ventral precentral/inferior frontal junction<br>↑ R supramarginal gyrus
Findings notes	Based on coordinates in Table 2

Voxelwise analysis 2

First level contrast	Listening to sentences and making a plausibility judgment vs listening to reversed speech
Analysis class	Cross-sectional between two groups with aphasia
Group(s)	Aphasia treated T3 (n = 6) vs untreated T3 (n = 6)
Covariate	—
Is the second level contrast valid in terms of the group(s), time point(s), and measures involved?	Somewhat (groups were different but not due to treatment)
Is accuracy matched across the second level contrast?	Yes, matched
Is reaction time matched across the second level contrast?	Unknown, not reported
Behavioral data notes	—
Type of analysis	Voxelwise
Search volume	Whole brain
Correction for multiple comparisons	Clusterwise correction based on arbitrary cluster extent
Software	BrainVoyager QX 1.9
Voxelwise p	.001
Cluster extent	0.16 cc
Statistical details	Methods report cluster extent threshold (we assume this was done), but figure caption states uncorrected
Findings	↑ L IFG pars triangularis<br>↑ L insula<br>↑ L supramarginal gyrus
Findings notes	Based on coordinates in Table 2; also increases in R IFG and R supramarginal gyrus but only uncorrected

Voxelwise analysis 3

First level contrast	Listening to sentences and making a plausibility judgment vs listening to reversed speech
Analysis class	Longitudinal between two groups with aphasia
Group(s)	(Aphasia treated (n = 6) T2 vs T1) vs (untreated (n = 6) T2 vs T1)
Covariate	—
Is the second level contrast valid in terms of the group(s), time point(s), and measures involved?	Somewhat (no treatment effect)
Is accuracy matched across the second level contrast?	Yes, matched
Is reaction time matched across the second level contrast?	Unknown, not reported
Behavioral data notes	—
Type of analysis	Voxelwise
Search volume	Whole brain
Correction for multiple comparisons	No direct comparison
Software	BrainVoyager QX 1.9
Voxelwise p	—
Cluster extent	—
Statistical details	Qualitative comparison on p. 548
Findings	↑ L IFG<br>↑ R posterior STG<br>↓ L inferior parietal lobule<br>↓ R IFG
Findings notes	Treated patients showed increases in L IFG and R STG, while untreated patients showed increases in L IPL and R IFG

Voxelwise analysis 4

First level contrast	Listening to sentences and making a plausibility judgment vs listening to reversed speech
Analysis class	Longitudinal between two groups with aphasia
Group(s)	(Aphasia treated (n = 6) T3 vs T2) vs (untreated (n = 6) T3 vs T2)
Covariate	—
Is the second level contrast valid in terms of the group(s), time point(s), and measures involved?	Somewhat (no treatment effect)
Is accuracy matched across the second level contrast?	Yes, matched
Is reaction time matched across the second level contrast?	Unknown, not reported
Behavioral data notes	—
Type of analysis	Voxelwise
Search volume	Whole brain
Correction for multiple comparisons	No direct comparison
Software	BrainVoyager QX 1.9
Voxelwise p	—
Cluster extent	—
Statistical details	Qualitative comparison on p. 548
Findings	None
Findings notes	The two groups were reported to have comparable increases in L hemisphere language areas

Voxelwise analysis 5

First level contrast	Listening to sentences and making a plausibility judgment vs listening to reversed speech
Analysis class	Longitudinal change in aphasia
Group(s)	Aphasia treated (n = 6) T2 vs T1
Covariate	—
Is the second level contrast valid in terms of the group(s), time point(s), and measures involved?	Yes
Is accuracy matched across the second level contrast?	Yes, matched
Is reaction time matched across the second level contrast?	Unknown, not reported
Behavioral data notes	—
Type of analysis	Voxelwise
Search volume	Whole brain
Correction for multiple comparisons	No correction
Software	BrainVoyager QX 1.9
Voxelwise p	.005
Cluster extent	None
Statistical details	—
Findings	↑ L IFG pars opercularis<br>↑ R posterior STG
Findings notes	—

Voxelwise analysis 6

First level contrast	Listening to sentences and making a plausibility judgment vs listening to reversed speech
Analysis class	Longitudinal change in aphasia
Group(s)	Aphasia untreated (n = 6) T2 vs T1
Covariate	—
Is the second level contrast valid in terms of the group(s), time point(s), and measures involved?	Yes
Is accuracy matched across the second level contrast?	Yes, matched
Is reaction time matched across the second level	Unknown, not reported

contrast?	
Behavioral data notes	—
Type of analysis	Voxelwise
Search volume	Whole brain
Correction for multiple comparisons	No correction
Software	BrainVoyager QX 1.9
Voxelwise p	.005
Cluster extent	None
Statistical details	—
Findings	↑ L inferior parietal lobule<br>↑ R insula
Findings notes	—

Voxelwise analysis 7

First level contrast	Listening to sentences and making a plausibility judgment vs listening to reversed speech
Analysis class	Longitudinal change in aphasia
Group(s)	Aphasia treated (n = 6) T3 vs T2
Covariate	—
Is the second level contrast valid in terms of the group(s), time point(s), and measures involved?	Yes
Is accuracy matched across the second level contrast?	Yes, matched
Is reaction time matched across the second level contrast?	Unknown, not reported
Behavioral data notes	—
Type of analysis	Voxelwise
Search volume	Whole brain
Correction for multiple comparisons	No correction
Software	BrainVoyager QX 1.9
Voxelwise p	.005
Cluster extent	None
Statistical details	—
Findings	↑ L IFG<br>↑ L insula<br>↑ L inferior parietal lobule<br>↑ L anterior temporal<br>↑ R insula
Findings notes	—

Voxelwise analysis 8

First level contrast	Listening to sentences and making a plausibility judgment vs listening to reversed speech
Analysis class	Longitudinal change in aphasia
Group(s)	Aphasia untreated (n = 6) T3 vs T2
Covariate	—
Is the second level contrast valid in terms of the group(s), time point(s), and measures involved?	Yes
Is accuracy matched across the second level contrast?	Yes, matched
Is reaction time matched across the second level contrast?	Unknown, not reported
Behavioral data notes	—
Type of analysis	Voxelwise
Search volume	Whole brain
Correction for multiple comparisons	No correction
Software	BrainVoyager QX 1.9
Voxelwise p	.005

Cluster extent	None
Statistical details	—
Findings	↑ L IFG pars opercularis<br>↑ L IFG pars triangularis<br>↑ L IFG pars orbitalis<br>↑ L angular gyrus<br>↑ L superior parietal<br>↑ L posterior STG/STS/MTG<br>↑ R IFG pars opercularis<br>↑ R angular gyrus
Findings notes	—

ROI analysis 1

First level contrast	Listening to sentences and making a plausibility judgment vs listening to reversed speech
Analysis class	Longitudinal between two groups with aphasia
Group(s)	(Aphasia treated (n = 6) T1 ≠ T2 ≠ T3) vs (untreated (n = 6) T1 ≠ T2 ≠ T3)
Covariate	—
Is the second level contrast valid in terms of the group(s), time point(s), and measures involved?	Somewhat (no treatment effect)
Is accuracy matched across the second level contrast?	Yes, matched
Is reaction time matched across the second level contrast?	Unknown, not reported
Behavioral data notes	—
Type of analysis	Regions of interest (ROI)
ROI type	Functional
How many ROIs are there?	4
What are the ROI(s)?	(1) L IFG; (2) R IFG; (3) L STG; (4) R STG
How are the ROI(s) defined?	Based on functional data from patients and controls, but details not stated; a different set of ROIs are mentioned in the results so it is not really clear which set were actually used
Correction for multiple comparisons	No correction
Statistical details	—
Findings	↑ L IFG
Findings notes	Interaction of time by treatment: treated group showed greater L IFG activity at T2

ROI analysis 2

First level contrast	Listening to sentences and making a plausibility judgment vs listening to reversed speech
Analysis class	Longitudinal correlation with language or other measure
Group(s)	Aphasia treated (n = 6) T2 vs T1
Covariate	Δ written language (AAT)
Is the second level contrast valid in terms of the group(s), time point(s), and measures involved?	Yes
Is accuracy matched across the second level contrast?	Yes, matched
Is reaction time matched across the second level contrast?	Unknown, not reported
Behavioral data notes	—
Type of analysis	Regions of interest (ROI)
ROI type	Functional
How many ROIs are there?	4
What are the ROI(s)?	(1) L IFG; (2) R IFG; (3) L STG; (4) R STG
How are the ROI(s) defined?	Based on functional data from patients and controls, but details not stated; a different set of ROIs are mentioned in the results so it is not really clear which set were actually used
Correction for multiple comparisons	No correction
Statistical details	—
Findings	None

Findings notes	—

ROI analysis 3

First level contrast	Listening to sentences and making a plausibility judgment vs listening to reversed speech
Analysis class	Longitudinal correlation with language or other measure
Group(s)	Aphasia treated (n = 6) T2 vs T1
Covariate	Δ naming (AAT)
Is the second level contrast valid in terms of the group(s), time point(s), and measures involved?	Yes
Is accuracy matched across the second level contrast?	Yes, matched
Is reaction time matched across the second level contrast?	Unknown, not reported
Behavioral data notes	—
Type of analysis	Regions of interest (ROI)
ROI type	Functional
How many ROIs are there?	4
What are the ROI(s)?	(1) L IFG; (2) R IFG; (3) L STG; (4) R STG
How are the ROI(s) defined?	Based on functional data from patients and controls, but details not stated; a different set of ROIs are mentioned in the results so it is not really clear which set were actually used
Correction for multiple comparisons	No correction
Statistical details	—
Findings	↑ L IFG
Findings notes	—

ROI analysis 4

First level contrast	Listening to sentences and making a plausibility judgment vs listening to reversed speech
Analysis class	Longitudinal correlation with language or other measure
Group(s)	Aphasia untreated (n = 6) T2 vs T1
Covariate	Δ written language (AAT)
Is the second level contrast valid in terms of the group(s), time point(s), and measures involved?	Yes
Is accuracy matched across the second level contrast?	Yes, matched
Is reaction time matched across the second level contrast?	Unknown, not reported
Behavioral data notes	—
Type of analysis	Regions of interest (ROI)
ROI type	Functional
How many ROIs are there?	4
What are the ROI(s)?	(1) L IFG; (2) R IFG; (3) L STG; (4) R STG
How are the ROI(s) defined?	Based on functional data from patients and controls, but details not stated; a different set of ROIs are mentioned in the results so it is not really clear which set were actually used
Correction for multiple comparisons	No correction
Statistical details	—
Findings	None
Findings notes	—

ROI analysis 5

First level contrast	Listening to sentences and making a plausibility judgment vs listening to reversed speech
Analysis class	Longitudinal correlation with language or other measure
Group(s)	Aphasia untreated (n = 6) T2 vs T1
Covariate	Δ naming (AAT)
Is the second level contrast valid in terms of the group(s), time point(s), and measures involved?	Yes

Is accuracy matched across the second level contrast?	Yes, matched
Is reaction time matched across the second level contrast?	Unknown, not reported
Behavioral data notes	—
Type of analysis	Regions of interest (ROI)
ROI type	Functional
How many ROIs are there?	4
What are the ROI(s)?	(1) L IFG; (2) R IFG; (3) L STG; (4) R STG
How are the ROI(s) defined?	Based on functional data from patients and controls, but details not stated; a different set of ROIs are mentioned in the results so it is not really clear which set were actually used
Correction for multiple comparisons	No correction
Statistical details	—
Findings	↑ R IFG
Findings notes	—

Notes

Excluded analyses	(1) a visual comparison between all patients at T1, and controls, because there are no specific claims apart from "markedly reduced cortical activation" in patients; (2) pre-treatment comparison between treated and untreated groups

Mohr et al. (2014)

Reference

Authors	Mohr B, Difrancesco S, Harrington K, Evans S, Pulvermüller F
Title	Changes of right-hemispheric activation after constraint-induced, intensive language action therapy in chronic aphasia: fMRI evidence from auditory semantic processing
Reference	*Front Hum Neurosci* 2014; 8: 919
PMID	25452721
DOI	10.3389/fnhum.2014.00919

Participants

Language	UK English
Inclusion criteria	MCA; mild-moderate non-fluent aphasia; no severe comprehension deficit
Number of individuals with aphasia	6 (plus 6 excluded: 4 for health risks; 2 for technical problems and data loss)
Number of control participants	0
Were any of the participants included in any previous studies?	No
Is age reported for patients and controls, and matched?	Yes (range 41-76 years)
Is sex reported for patients and controls, and matched?	Yes (males: 5; females: 1)
Is handedness reported for patients and controls, and matched?	Yes (right: 6; left: 0)
Is time post stroke onset reported and appropriate to the study design?	Yes (range 17-234 months (including excluded patients))
To what extent is the nature of aphasia characterized?	Severity only
Language evaluation	BDAE, TT
Aphasia severity	Mild-moderate; T1: TT range 15-49 errors (including 2 excluded patients)
Aphasia type	Not stated
First stroke only?	Yes
Stroke type	Mixed etiologies

To what extent is the lesion distribution characterized?	Lesion overlay
Lesion extent	Not stated
Lesion location	L MCA
Participants notes	Patient numbers in tables 1 and 2 appear not to correspond with patient numbers later in the paper

Imaging

Modality	fMRI
Is the study cross-sectional or longitudinal?	Longitudinal—chronic treatment
If longitudinal, at what time point(s) were imaging data acquired?	T1: pre-treatment/chronic; T2: post-treatment, ~2 weeks later
If longitudinal, was there any intervention between the time points?	CIAT, 3-4 hours/day, 5 days/week, 2 weeks
Is the scanner described?	Yes (Siemens Trio 3 Tesla)
Is the timing of stimulus presentation and image acquisition clearly described and appropriate?	Yes
Design type	Event-related
Total images acquired	76
Are the imaging acquisition parameters, including coverage, adequately described and appropriate?	Yes (whole brain)
Is preprocessing and intrasubject coregistration adequately described and appropriate?	Yes
Is first level model fitting adequately described and appropriate?	Yes
Is intersubject normalization adequately described and appropriate?	No (lesion impact not addressed)
Imaging notes	sparse sampling

Conditions

Are the conditions clearly described?	Yes

Condition	Response type	Repetitions	All groups could do?	All individuals could do?
listening to high ambiguity sentences	None	19	N/A	N/A
listening to low ambiguity sentences	None	19	N/A	N/A
listening to signal-correlated noise	None	19	N/A	N/A
rest	None	19	N/A	N/A

Conditions notes	—

Contrasts

Are the contrasts clearly described?	Yes

Contrast 1: listening to sentences (high and low ambiguity) vs listening to signal-correlated noise

Language condition	Listening to sentences (high and low ambiguity)
Control condition	Listening to signal-correlated noise
Are the conditions matched for visual demands?	Yes
Are the conditions matched for auditory demands?	Yes
Are the conditions matched for motor demands?	Yes
Are the conditions matched for cognitive/executive demands?	Yes
Is accuracy matched between the language and control tasks for all relevant groups?	N/A, no behavioral measure
Is reaction time matched between the language and control tasks for all relevant groups?	N/A, no timeable task
Behavioral data notes	—

Are control data reported in this paper or another that is referenced?	No
Does the contrast selectively activate plausible relevant language regions in the control group?	Unknown
Are activations lateralized in the control data?	Unknown
Control activation notes	Some control data in Rodd et al. (2005), but half of the participants were performing a probe judgment task, unlike in the present study
Contrast notes	—

Contrast 2: listening to high ambiguity sentences vs listening to low ambiguity sentences

Language condition	Listening to high ambiguity sentences
Control condition	Listening to low ambiguity sentences
Are the conditions matched for visual demands?	Yes
Are the conditions matched for auditory demands?	Yes
Are the conditions matched for motor demands?	Yes
Are the conditions matched for cognitive/executive demands?	Yes
Is accuracy matched between the language and control tasks for all relevant groups?	N/A, no behavioral measure
Is reaction time matched between the language and control tasks for all relevant groups?	N/A, no timeable task
Behavioral data notes	—
Are control data reported in this paper or another that is referenced?	No
Does the contrast selectively activate plausible relevant language regions in the control group?	Unknown
Are activations lateralized in the control data?	Unknown
Control activation notes	Some control data in Rodd et al. (2005), but half of the participants were performing a probe judgment task, unlike in the present study
Contrast notes	—

Analyses

Are the analyses clearly described?	No* (moderate limitation) (see specific limitation(s) below)

Voxelwise analysis 1

First level contrast	Listening to sentences (high and low ambiguity) vs listening to signal-correlated noise
Analysis class	Longitudinal change in aphasia
Group(s)	Aphasia T2 vs T1
Covariate	—
Is the second level contrast valid in terms of the group(s), time point(s), and measures involved?	Yes
Is accuracy matched across the second level contrast?	N/A, no behavioral measure
Is reaction time matched across the second level contrast?	N/A, no timeable task
Behavioral data notes	—
Type of analysis	Voxelwise
Search volume	Whole brain
Correction for multiple comparisons	No direct comparison
Software	SPM8
Voxelwise p	—
Cluster extent	—
Statistical details	Qualitative generalization across individuals on pp. 8-9
Findings	None
Findings notes	—

ROI analysis 1

First level contrast	Listening to high ambiguity sentences vs listening to low ambiguity sentences
Analysis class	Longitudinal change in aphasia
Group(s)	Aphasia T2 vs T1
Covariate	—
Is the second level contrast valid in terms of the group(s), time point(s), and measures involved?	Yes
Is accuracy matched across the second level contrast?	N/A, no behavioral measure
Is reaction time matched across the second level contrast?	N/A, no timeable task
Behavioral data notes	—
Type of analysis	Regions of interest (ROI)
ROI type	Functional
How many ROIs are there?	4
What are the ROI(s)?	(1) L IFG; (2) R IFG; (3) L ITG; (4) R ITG; the temporal ROIs are described as STG but they seem to be in the ITG
How are the ROI(s) defined?	Defined based on control data from Rodd et al. (2005) but the coordinates do not match so it is not clear exactly how they were defined
Correction for multiple comparisons	No correction
Statistical details	ANOVA of timepoint by hemisphere by site, with a significant interaction of timepoint by hemisphere
Findings	↑ R IFG<br>↑ R posterior inferior temporal gyrus/fusiform gyrus
Findings notes	All signal changes were negative (i.e. less activation for ambiguous sentences), making interpretation challenging

Notes

Excluded analyses	Noise vs rest (not language); individual patient analyses

Robson et al. (2014)

Reference

Authors	Robson H, Zahn R, Keidel JL, Binney RJ, Sage K, Lambon Ralph MA
Title	The anterior temporal lobes support residual comprehension in Wernicke's aphasia
Reference	*Brain* 2014; 137: 931-943
PMID	24519979
DOI	10.1093/brain/awt373

Participants

Language	UK English
Inclusion criteria	Wernicke's aphasia (impaired spoken single word comprehension, impaired single word repetition, fluent, sentence-like speech with phonological/neologistic errors)
Number of individuals with aphasia	12
Number of control participants	12
Were any of the participants included in any previous studies?	No
Is age reported for patients and controls, and matched?	Yes (mean 70.1 ± 8.7 years, range 59-87 years)
Is sex reported for patients and controls, and matched?	Yes (males: 10; females: 2)
Is handedness reported for patients and controls, and matched?	Yes (right: 12; left: 0)

Is time post stroke onset reported and appropriate to the study design?	Yes (range 7-84 months)
To what extent is the nature of aphasia characterized?	Comprehensive battery
Language evaluation	BDAE, PPT, word-to-picture matching test from Cambridge Semantic Battery, single word reading aloud from PALPA
Aphasia severity	BDAE comprehension range 6-26 (out of 32); BDAE comprehension scores and percentiles do not seem entirely commensurate
Aphasia type	All Wernicke's
First stroke only?	Yes
Stroke type	Mixed etiologies
To what extent is the lesion distribution characterized?	Lesion overlay
Lesion extent	Not stated
Lesion location	L MCA; all involved STG extending into IPL and temporoparietal junction; 8 extending into MTL; 4 extending into inferior frontal
Participants notes	—

Imaging

Modality	fMRI
Is the study cross-sectional or longitudinal?	Cross-sectional
If longitudinal, at what time point(s) were imaging data acquired?	—
If longitudinal, was there any intervention between the time points?	—
Is the scanner described?	Yes (Philips Achieva 3 Tesla)
Is the timing of stimulus presentation and image acquisition clearly described and appropriate?	No* (moderate limitation) (each condition was acquired in a separate run, which is suboptimal)
Design type	Block
Total images acquired	417
Are the imaging acquisition parameters, including coverage, adequately described and appropriate?	Yes (whole brain)
Is preprocessing and intrasubject coregistration adequately described and appropriate?	Yes
Is first level model fitting adequately described and appropriate?	Yes
Is intersubject normalization adequately described and appropriate?	Yes
Imaging notes	spin echo fMRI to minimize ATL dropout

Conditions

Are the conditions clearly described?	Yes

Condition	Response type	Repetitions	All groups could do?	All individuals could do?
semantic decision (written word)	Button press	16	Yes	No
semantic decision (picture)	Button press	16	Yes	No
visual decision	Button press	16	Yes	No
rest	None	48	N/A	N/A

Conditions notes	—

Contrasts

Are the contrasts clearly described?	No (see specific limitation(s) below)

Contrast 1: semantic decision (written word and picture) vs visual decision and rest

Language condition	Semantic decision (written word and picture)

Control condition	Visual decision and rest
Are the conditions matched for visual demands?	No
Are the conditions matched for auditory demands?	Yes
Are the conditions matched for motor demands?	No
Are the conditions matched for cognitive/executive demands?	No
Is accuracy matched between the language and control tasks for all relevant groups?	N/A, tasks not comparable
Is reaction time matched between the language and control tasks for all relevant groups?	N/A, tasks not comparable
Behavioral data notes	Not comparable because the control condition includes rest
Are control data reported in this paper or another that is referenced?	Somewhat
Does the contrast selectively activate plausible relevant language regions in the control group?	Somewhat
Are activations lateralized in the control data?	No
Control activation notes	Control data are provided in Table 6 for contrasts of written word semantic decision vs dual baseline, and picture semantic decision vs dual baseline, but not for the main effect of semantic decision; these data suggest that the contrast activates ventral temporal regions bilaterally
Contrast notes	Two contrasts are described: (1) written word judgment versus a dual baseline of visual judgment and rest; (2) picture judgment versus a dual baseline of visual judgment and rest; these two primary contrasts are reported in patients and controls separately, but no between-group contrasts are reported, so these contrasts are excluded from our review; rather, the between-groups analyses in the paper take the form of ANOVAs; the main effect of group in these ANOVAs collapses across the two described contrasts, therefore we have coded the contrast as the average of the two described contrasts; the exact nature of the computation of dual baseline contrasts is not described

Analyses

Are the analyses clearly described?	No* (moderate limitation) (see specific limitation(s) below)

Voxelwise analysis 1

First level contrast	Semantic decision (written word and picture) vs visual decision and rest
Analysis class	Cross-sectional aphasia vs control
Group(s)	Aphasia vs control
Covariate	—
Is the second level contrast valid in terms of the group(s), time point(s), and measures involved?	Yes
Is accuracy matched across the second level contrast?	No, different
Is reaction time matched across the second level contrast?	No, different
Behavioral data notes	Patients also less accurate on control condition, but control condition includes rest so coded based on language condition only
Type of analysis	Voxelwise
Search volume	Whole brain
Correction for multiple comparisons	Clusterwise correction based on arbitrary cluster extent
Software	SPM8
Voxelwise p	.005
Cluster extent	4 voxels (size not stated)
Statistical details	Dual baseline computation not explained
Findings	↑ L IFG pars orbitalis<br>↑ L mid temporal<br>↑ L anterior temporal<br>↑ L cerebellum<br>↑ L hippocampus/MTL

	↑ R mid temporal<br>↑ R anterior temporal<br>↑ R posterior inferior temporal gyrus/fusiform gyrus<br>↑ R cerebellum<br>↑ R hippocampus/MTL<br>↓ R posterior cingulate
Findings notes	—

ROI analysis 1

First level contrast	Semantic decision (written word and picture) vs visual decision and rest
Analysis class	Cross-sectional aphasia vs control
Group(s)	Aphasia vs control
Covariate	—
Is the second level contrast valid in terms of the group(s), time point(s), and measures involved?	Yes
Is accuracy matched across the second level contrast?	No, different
Is reaction time matched across the second level contrast?	No, different
Behavioral data notes	Patients also less accurate on control condition, but control condition includes rest so coded based on language condition only
Type of analysis	Regions of interest (ROI)
ROI type	Functional
How many ROIs are there?	10
What are the ROI(s)?	(1) L anterior fusiform gyrus; (2) L temporal pole; (3) L anterior STS; (4) L IFG; (5) L ventral occipito-temporal; (6-10) homotopic counterparts
How are the ROI(s) defined?	Spheres around functional peaks from literature
Correction for multiple comparisons	No correction
Statistical details	Dual baseline computation not explained
Findings	↑ L anterior temporal<br>↑ L posterior inferior temporal gyrus/fusiform gyrus<br>↑ R posterior inferior temporal gyrus/fusiform gyrus
Findings notes	—

Notes

Excluded analyses	(1) main effect of condition (written words vs pictures); (2) interactions of condition by group (all of which were non-significant); (3) additional analyses were run including only participants who performed above chance, and only correct responses from all participants, but these gave essentially similar results

Szaflarski et al. (2014)

Reference

Authors	Szaflarski JP, Allendorfer JB, Byars AW, Vannest J, Dietz A, Hernando KA, Holland SK
Title	Age at stroke determines post-stroke language lateralization
Reference	*Restor Neurol Neurosci* 2014; 32: 733-742
PMID	25159870
DOI	10.3233/rnn-140402

Participants

Language	US English
Inclusion criteria	—
Number of individuals with aphasia	32
Number of control participants	32

Were any of the participants included in any previous studies?	Yes (some participants included in Allendorfer et al. (2012))
Is age reported for patients and controls, and matched?	Yes (mean 51.8 ± 15.1 years)
Is sex reported for patients and controls, and matched?	Yes (males: 18; females: 14)
Is handedness reported for patients and controls, and matched?	No
Is time post stroke onset reported and appropriate to the study design?	Yes (mean 3.2 ± 3.1 years, > 6 months)
To what extent is the nature of aphasia characterized?	Not at all
Language evaluation	Not stated
Aphasia severity	"complete or almost complete" recovery in a "substantial proportion" of the patients
Aphasia type	Not stated
First stroke only?	Not stated
Stroke type	Not stated
To what extent is the lesion distribution characterized?	Lesion overlay
Lesion extent	60.1 ± 57.5 cc
Lesion location	L MCA
Participants notes	One participant was < 18 years old at time of stroke; there was also a perinatal stroke group, not relevant for this review; 3 participants were excluded but it is not stated whether they were adult or perinatal patients.

Imaging

Modality	fMRI
Is the study cross-sectional or longitudinal?	Cross-sectional
If longitudinal, at what time point(s) were imaging data acquired?	—
If longitudinal, was there any intervention between the time points?	—
Is the scanner described?	Yes (Philips Achieva 3 Tesla, except for 1 patient and 1 control on a Bruker 3 Tesla)
Is the timing of stimulus presentation and image acquisition clearly described and appropriate?	Yes
Design type	Block
Total images acquired	165
Are the imaging acquisition parameters, including coverage, adequately described and appropriate?	Yes (whole brain)
Is preprocessing and intrasubject coregistration adequately described and appropriate?	Yes
Is first level model fitting adequately described and appropriate?	Yes
Is intersubject normalization adequately described and appropriate?	Yes
Imaging notes	—

Conditions

Are the conditions clearly described?	Yes

Condition	Response type	Repetitions	All groups could do?	All individuals could do?
verb generation	Multiple words (covert)	5	Yes	Unknown
finger tapping	Other	6	Yes	Yes

Conditions notes	—

Contrasts

Are the contrasts clearly described?	Yes

Contrast 1: verb generation vs finger tapping

Language condition	Verb generation
Control condition	Finger tapping
Are the conditions matched for visual demands?	Yes
Are the conditions matched for auditory demands?	Yes
Are the conditions matched for motor demands?	No
Are the conditions matched for cognitive/executive demands?	No
Is accuracy matched between the language and control tasks for all relevant groups?	N/A, tasks not comparable
Is reaction time matched between the language and control tasks for all relevant groups?	N/A, tasks not comparable
Behavioral data notes	—
Are control data reported in this paper or another that is referenced?	Yes
Does the contrast selectively activate plausible relevant language regions in the control group?	Yes
Are activations lateralized in the control data?	Somewhat
Control activation notes	Control data in Szaflarski et al. (2008); frontal activation L-lateralized, temporal less so
Contrast notes	—

Analyses

Are the analyses clearly described?	Yes

Voxelwise analysis 1

First level contrast	Verb generation vs finger tapping
Analysis class	Cross-sectional aphasia vs control
Group(s)	Aphasia vs control
Covariate	—
Is the second level contrast valid in terms of the group(s), time point(s), and measures involved?	Yes
Is accuracy matched across the second level contrast?	Unknown, not reported
Is reaction time matched across the second level contrast?	Unknown, not reported
Behavioral data notes	—
Type of analysis	Voxelwise
Search volume	Whole brain
Correction for multiple comparisons	No direct comparison
Software	CCHIPS
Voxelwise p	—
Cluster extent	—
Statistical details	Qualitative comparison on pp. 5-6 (page numbers refer to PMC author manuscript)
Findings	↓ L inferior parietal lobule<br>↓ L superior parietal<br>↓ L posterior STG/STS/MTG<br>↓ L occipital<br>↓ R occipital
Findings notes	—

ROI analysis 1

First level contrast	Verb generation vs finger tapping

Analysis class	Cross-sectional aphasia vs control
Group(s)	Aphasia vs control
Covariate	—
Is the second level contrast valid in terms of the group(s), time point(s), and measures involved?	Yes
Is accuracy matched across the second level contrast?	Unknown, not reported
Is reaction time matched across the second level contrast?	Unknown, not reported
Behavioral data notes	—
Type of analysis	Regions of interest (ROI)
ROI type	Laterality indi(ces)
How many ROIs are there?	3
What are the ROI(s)?	(1) frontal LI; (2) temporal LI; (3) language network LI
How are the ROI(s) defined?	
Correction for multiple comparisons	No correction
Statistical details	—
Findings	↓ LI (language network)<br>↓ LI (frontal)
Findings notes	Temporal LI was also marginally significantly reduced (p = .08)

Notes

Excluded analyses	All analyses involving perinatal stroke group; distribution of language lateralization categories (derived from LI) also differed between patients and controls

van Hees et al. (2014)

Reference

Authors	van Hees S, McMahon K, Angwin A, de Zubicaray G, Copland DA
Title	Neural activity associated with semantic versus phonological anomia treatments in aphasia
Reference	*Brain Lang* 2014; 129: 47-57
PMID	24556337
DOI	10.1016/j.bandl.2013.12.004

Participants

Language	Australian English
Inclusion criteria	—
Number of individuals with aphasia	8
Number of control participants	14
Were any of the participants included in any previous studies?	No
Is age reported for patients and controls, and matched?	Yes (mean 56.4 + 9.2 years; range 41-69 years)
Is sex reported for patients and controls, and matched?	Yes (males: 3; females: 5)
Is handedness reported for patients and controls, and matched?	Yes (right: 8; left: 0)
Is time post stroke onset reported and appropriate to the study design?	Yes (mean 52.3 + 49.8 months; range 17-170 months)
To what extent is the nature of aphasia characterized?	Comprehensive battery
Language evaluation	WAB, BNT, PPT, CAT, picture naming from International Picture Naming Project Database
Aphasia severity	AQ range 57.3-91.6; 5 mild, 2 moderate, 1 mild-moderate

Aphasia type	6 anomic, 2 conduction
First stroke only?	Yes
Stroke type	Not stated
To what extent is the lesion distribution characterized?	Lesion overlay
Lesion extent	Not stated
Lesion location	L hemisphere
Participants notes	—

Imaging

Modality	fMRI
Is the study cross-sectional or longitudinal?	Longitudinal—chronic treatment
If longitudinal, at what time point(s) were imaging data acquired?	T1: pre-treatment/chronic; T2: post-treatment, 5-6 weeks later; note that "immediate improvement" was measured at the end of SLT, a week or two prior to T2 scan
If longitudinal, was there any intervention between the time points?	SLT with alternating semantic and phonological sessions, 3 days/week, 4 weeks
Is the scanner described?	Yes (Bruker MedSpec 4 Tesla)
Is the timing of stimulus presentation and image acquisition clearly described and appropriate?	Yes
Design type	Event-related
Total images acquired	610
Are the imaging acquisition parameters, including coverage, adequately described and appropriate?	Yes (whole brain)
Is preprocessing and intrasubject coregistration adequately described and appropriate?	Yes
Is first level model fitting adequately described and appropriate?	Yes
Is intersubject normalization adequately described and appropriate?	No (lesion impact not addressed)
Imaging notes	slow event-related design; sparse sampling

Conditions

Are the conditions clearly described?	Yes

Condition	Response type	Repetitions	All groups could do?	All individuals could do?
picture naming (phonological trained items)	Word (overt)	30	Yes	No
picture naming (semantic trained items)	Word (overt)	30	Yes	No
picture naming (known items)	Word (overt)	30	Yes	Yes
viewing scrambled images	None	30	N/A	N/A

Conditions notes	Some patients named < 10% correct at T1

Contrasts

Are the contrasts clearly described?	No (see specific limitation(s) below)

Contrast 1: picture naming (phonological trained items, correct trials) vs viewing scrambled images

Language condition	Picture naming (phonological trained items, correct trials)
Control condition	Viewing scrambled images
Are the conditions matched for visual demands?	Yes
Are the conditions matched for auditory demands?	No
Are the conditions matched for motor demands?	No
Are the conditions matched for cognitive/executive demands?	No
Is accuracy matched between the language and control tasks for all relevant groups?	N/A, tasks not comparable

Is reaction time matched between the language and control tasks for all relevant groups?	N/A, tasks not comparable
Behavioral data notes	—
Are control data reported in this paper or another that is referenced?	Somewhat
Does the contrast selectively activate plausible relevant language regions in the control group?	Unknown
Are activations lateralized in the control data?	Unknown
Control activation notes	Control data are described for naming untrained items; the data are reported only briefly in the text; it is notable that no speech motor, visual, or auditory activations are reported, as might be expected in a picture naming task
Contrast notes	Correct and incorrect trials were apparently modeled separately, but this is not clearly stated, nor are the criteria for deciding whether trials were correct; it is generally not clear which contrasts exactly were run

Contrast 2: picture naming (semantic trained items, correct trials) vs viewing scrambled images

Language condition	Picture naming (semantic trained items, correct trials)
Control condition	Viewing scrambled images
Are the conditions matched for visual demands?	Yes
Are the conditions matched for auditory demands?	No
Are the conditions matched for motor demands?	No
Are the conditions matched for cognitive/executive demands?	No
Is accuracy matched between the language and control tasks for all relevant groups?	N/A, tasks not comparable
Is reaction time matched between the language and control tasks for all relevant groups?	N/A, tasks not comparable
Behavioral data notes	—
Are control data reported in this paper or another that is referenced?	Somewhat
Does the contrast selectively activate plausible relevant language regions in the control group?	Unknown
Are activations lateralized in the control data?	Unknown
Control activation notes	Control data are described for naming untrained items; the data are reported only briefly in the text; it is notable that no speech motor, visual, or auditory activations are reported, as might be expected in a picture naming task
Contrast notes	Correct and incorrect trials were apparently modeled separately, but this is not clearly stated, nor are the criteria for deciding whether trials were correct; it is generally not clear which contrasts exactly were run

Analyses

Are the analyses clearly described?	Yes

Voxelwise analysis 1

First level contrast	Picture naming (phonological trained items, correct trials) vs viewing scrambled images
Analysis class	Cross-sectional correlation with language or other measure
Group(s)	Aphasia T1
Covariate	Subsequent Δ (T2 vs T1) picture naming (phonological treated items)
Is the second level contrast valid in terms of the group(s), time point(s), and measures involved?	Somewhat (T1 behavioral measure should be included in model)
Is accuracy matched across the second level contrast?	Yes, correct trials only
Is reaction time matched across the second level contrast?	Unknown, not reported
Behavioral data notes	—
Type of analysis	Voxelwise
Search volume	Whole brain

Correction for multiple comparisons	Clusterwise correction based on 3dClustSim
Software	AFNI
Voxelwise p	.005
Cluster extent	0.999 cc
Statistical details	—
Findings	None
Findings notes	—

Voxelwise analysis 2

First level contrast	Picture naming (semantic trained items, correct trials) vs viewing scrambled images
Analysis class	Cross-sectional correlation with language or other measure
Group(s)	Aphasia T1
Covariate	Subsequent Δ (T2 vs T1) picture naming (semantic treated items)
Is the second level contrast valid in terms of the group(s), time point(s), and measures involved?	Somewhat (T1 behavioral measure should be included in model)
Is accuracy matched across the second level contrast?	Yes, correct trials only
Is reaction time matched across the second level contrast?	Unknown, not reported
Behavioral data notes	—
Type of analysis	Voxelwise
Search volume	Whole brain
Correction for multiple comparisons	Clusterwise correction based on 3dClustSim
Software	AFNI
Voxelwise p	.005
Cluster extent	0.999 cc
Statistical details	—
Findings	↑ L basal ganglia
Findings notes	—

Voxelwise analysis 3

First level contrast	Picture naming (phonological trained items, correct trials) vs viewing scrambled images
Analysis class	Cross-sectional correlation with language or other measure
Group(s)	Aphasia T2
Covariate	Previous Δ (T2 vs T1) picture naming (phonological treated items)
Is the second level contrast valid in terms of the group(s), time point(s), and measures involved?	No (T2 activation not an appropriate measure of treatment-induced recovery because it reflects T2 performance)
Is accuracy matched across the second level contrast?	Yes, correct trials only
Is reaction time matched across the second level contrast?	Unknown, not reported
Behavioral data notes	—
Type of analysis	Voxelwise
Search volume	Whole brain
Correction for multiple comparisons	Clusterwise correction based on 3dClustSim
Software	AFNI
Voxelwise p	.005
Cluster extent	0.999 cc
Statistical details	—
Findings	↑ L supramarginal gyrus<br>↑ R precuneus
Findings notes	—

Voxelwise analysis 4

First level contrast	Picture naming (semantic trained items, correct trials) vs viewing scrambled images

Analysis class	Cross-sectional correlation with language or other measure
Group(s)	Aphasia T2
Covariate	Previous Δ (T2 vs T1) picture naming (semantic treated items)
Is the second level contrast valid in terms of the group(s), time point(s), and measures involved?	No (T2 activation not an appropriate measure of treatment-induced recovery because it reflects T2 performance)
Is accuracy matched across the second level contrast?	Yes, correct trials only
Is reaction time matched across the second level contrast?	Unknown, not reported
Behavioral data notes	—
Type of analysis	Voxelwise
Search volume	Whole brain
Correction for multiple comparisons	Clusterwise correction based on 3dClustSim
Software	AFNI
Voxelwise p	.005
Cluster extent	0.999 cc
Statistical details	—
Findings	None
Findings notes	—

Voxelwise analysis 5

First level contrast	Picture naming (phonological trained items, correct trials) vs viewing scrambled images
Analysis class	Cross-sectional correlation with language or other measure
Group(s)	Aphasia T1
Covariate	Subsequent outcome (T2) picture naming
Is the second level contrast valid in terms of the group(s), time point(s), and measures involved?	No (not appropriate to correlate T1 imaging with T2 behavior without T1 behavior in model)
Is accuracy matched across the second level contrast?	Yes, correct trials only
Is reaction time matched across the second level contrast?	Unknown, not reported
Behavioral data notes	—
Type of analysis	Voxelwise
Search volume	Whole brain
Correction for multiple comparisons	Clusterwise correction based on 3dClustSim
Software	AFNI
Voxelwise p	.005
Cluster extent	0.999 cc
Statistical details	—
Findings	None
Findings notes	—

Voxelwise analysis 6

First level contrast	Picture naming (semantic trained items, correct trials) vs viewing scrambled images
Analysis class	Cross-sectional correlation with language or other measure
Group(s)	Aphasia T1
Covariate	Subsequent outcome (T2) picture naming
Is the second level contrast valid in terms of the group(s), time point(s), and measures involved?	No (not appropriate to correlate T1 imaging with T2 behavior without T1 behavior in model)
Is accuracy matched across the second level contrast?	Yes, correct trials only
Is reaction time matched across the second level contrast?	Unknown, not reported
Behavioral data notes	—
Type of analysis	Voxelwise

Search volume	Whole brain
Correction for multiple comparisons	Clusterwise correction based on 3dClustSim
Software	AFNI
Voxelwise p	.005
Cluster extent	0.999 cc
Statistical details	—
Findings	None
Findings notes	—

Voxelwise analysis 7

First level contrast	Picture naming (phonological trained items, correct trials) vs viewing scrambled images
Analysis class	Cross-sectional correlation with language or other measure
Group(s)	Aphasia T2
Covariate	Picture naming T2
Is the second level contrast valid in terms of the group(s), time point(s), and measures involved?	Yes
Is accuracy matched across the second level contrast?	Yes, correct trials only
Is reaction time matched across the second level contrast?	Unknown, not reported
Behavioral data notes	—
Type of analysis	Voxelwise
Search volume	Whole brain
Correction for multiple comparisons	Clusterwise correction based on 3dClustSim
Software	AFNI
Voxelwise p	.005
Cluster extent	0.999 cc
Statistical details	—
Findings	None
Findings notes	—

Voxelwise analysis 8

First level contrast	Picture naming (semantic trained items, correct trials) vs viewing scrambled images
Analysis class	Cross-sectional correlation with language or other measure
Group(s)	Aphasia T2
Covariate	Picture naming T2
Is the second level contrast valid in terms of the group(s), time point(s), and measures involved?	Yes
Is accuracy matched across the second level contrast?	Yes, correct trials only
Is reaction time matched across the second level contrast?	Unknown, not reported
Behavioral data notes	—
Type of analysis	Voxelwise
Search volume	Whole brain
Correction for multiple comparisons	Clusterwise correction based on 3dClustSim
Software	AFNI
Voxelwise p	.005
Cluster extent	0.999 cc
Statistical details	—
Findings	None
Findings notes	—

Notes

Excluded analyses	Individual patient analyses

Abel et al. (2015)

Reference

Authors	Abel S, Weiller C, Huber W, Willmes K, Specht K
Title	Therapy-induced brain reorganization patterns in aphasia
Reference	*Brain* 2015; 138: 1097-1112
PMID	25688082
DOI	10.1093/brain/awv022

Participants

Language	German
Inclusion criteria	Anomia; no severe AoS or dysarthria
Number of individuals with aphasia	14 (plus 9 excluded: 4 for ceiling performance; 5 for technical problems)
Number of control participants	14
Were any of the participants included in any previous studies?	Yes (same dataset as Abel et al. (2014))
Is age reported for patients and controls, and matched?	Yes (median 48 years, range 35-74 years)
Is sex reported for patients and controls, and matched?	Yes (males: 10; females: 4)
Is handedness reported for patients and controls, and matched?	Yes (right: 14; left: 0)
Is time post stroke onset reported and appropriate to the study design?	Yes (median 41 months, range 11-72 months)
To what extent is the nature of aphasia characterized?	Type only
Language evaluation	AAT
Aphasia severity	Not stated
Aphasia type	8 Broca's, 3 Wernicke's, 1 fluent non-classifiable, 1 global, 1 transcortical sensory
First stroke only?	Yes
Stroke type	Mixed etiologies
To what extent is the lesion distribution characterized?	Lesion overlay
Lesion extent	Not stated
Lesion location	L MCA; 2 also had ACA
Participants notes	—

Imaging

Modality	fMRI
Is the study cross-sectional or longitudinal?	Longitudinal—chronic treatment
If longitudinal, at what time point(s) were imaging data acquired?	T1: pre-treatment/chronic; T2: post-treatment, ~6 weeks later (labeled T2 and T3 in paper)
If longitudinal, was there any intervention between the time points?	Lexical therapy, alternating between weeks with phonological and semantic treatment, 4 weeks; 60 out of the 132 items were trained
Is the scanner described?	Yes (Philips Achieva 3 Tesla)
Is the timing of stimulus presentation and image acquisition clearly described and appropriate?	No* (moderate limitation) (trials too close together (~8 s) and insufficient jitter (1-3 s) for event-related design)
Design type	Event-related
Total images acquired	560
Are the imaging acquisition parameters, including coverage, adequately described and appropriate?	Yes (whole brain)
Is preprocessing and intrasubject coregistration adequately described and appropriate?	Yes

Is first level model fitting adequately described and appropriate?	Yes
Is intersubject normalization adequately described and appropriate?	No (lesion impact not addressed)
Imaging notes	—

Conditions

Are the conditions clearly described?	Yes

Condition	Response type	Repetitions	All groups could do?	All individuals could do?
picture naming	Word (overt)	132	Yes	Yes
rest	None	implicit baseline	N/A	N/A

Conditions notes	—

Contrasts

Are the contrasts clearly described?	Yes

Contrast 1: picture naming vs rest

Language condition	Picture naming
Control condition	Rest
Are the conditions matched for visual demands?	No
Are the conditions matched for auditory demands?	No
Are the conditions matched for motor demands?	No
Are the conditions matched for cognitive/executive demands?	No
Is accuracy matched between the language and control tasks for all relevant groups?	N/A, tasks not comparable
Is reaction time matched between the language and control tasks for all relevant groups?	N/A, tasks not comparable
Behavioral data notes	—
Are control data reported in this paper or another that is referenced?	Yes
Does the contrast selectively activate plausible relevant language regions in the control group?	No
Are activations lateralized in the control data?	No
Control activation notes	Bilateral somato-motor, auditory and to a lesser extent higher level visual regions; finite impulse analysis only
Contrast notes	—

Analyses

Are the analyses clearly described?	Yes

Voxelwise analysis 1

First level contrast	Picture naming vs rest
Analysis class	Longitudinal change in aphasia
Group(s)	Aphasia T2 vs T1
Covariate	—
Is the second level contrast valid in terms of the group(s), time point(s), and measures involved?	Yes
Is accuracy matched across the second level contrast?	No, different
Is reaction time matched across the second level contrast?	No, different
Behavioral data notes	RT shorter at T2

Type of analysis	Voxelwise
Search volume	Whole brain
Correction for multiple comparisons	Clusterwise correction based on cluster_threshold_beta
Software	SPM8
Voxelwise p	.01
Cluster extent	11 voxels (size not stated)
Statistical details	—
Findings	↓ L IFG pars triangularis<br>↓ L dorsolateral prefrontal cortex<br>↓ L ventral precentral/inferior frontal junction<br>↓ L dorsal precentral<br>↓ L SMA/medial prefrontal<br>↓ L somato-motor<br>↓ L inferior parietal lobule<br>↓ L precuneus<br>↓ L posterior cingulate<br>↓ L cerebellum<br>↓ R SMA/medial prefrontal<br>↓ R somato-motor<br>↓ R precuneus<br>↓ R posterior STS<br>↓ R posterior MTG<br>↓ R posterior cingulate<br>↓ R cerebellum<br>↓ R thalamus<br>↓ R hippocampus/MTL
Findings notes	—

Voxelwise analysis 2

First level contrast	Picture naming vs rest
Analysis class	Cross-sectional aphasia vs control
Group(s)	Aphasia T1 vs control T1
Covariate	—
Is the second level contrast valid in terms of the group(s), time point(s), and measures involved?	Yes
Is accuracy matched across the second level contrast?	Appear mismatched
Is reaction time matched across the second level contrast?	No, different
Behavioral data notes	Controls responded more quickly
Type of analysis	Voxelwise
Search volume	Whole brain
Correction for multiple comparisons	Clusterwise correction based on cluster_threshold_beta
Software	SPM8
Voxelwise p	.01
Cluster extent	11 voxels (size not stated)
Statistical details	—
Findings	↑ R precuneus<br>↓ L somato-motor<br>↓ L Heschl's gyrus<br>↓ L anterior cingulate<br>↓ L posterior cingulate<br>↓ L thalamus<br>↓ L basal ganglia<br>↓ R insula<br>↓ R somato-motor<br>↓ R mid temporal
Findings notes	—

Voxelwise analysis 3

First level contrast	Picture naming vs rest
Analysis class	Longitudinal aphasia vs control
Group(s)	(Aphasia T2 vs T1) vs (control T2 vs T1)
Covariate	—
Is the second level contrast valid in terms of the group(s), time point(s), and measures involved?	Yes
Is accuracy matched across the second level contrast?	Appear mismatched
Is reaction time matched across the second level contrast?	Unknown, not reported
Behavioral data notes	RT not reported for controls
Type of analysis	Voxelwise
Search volume	Whole brain
Correction for multiple comparisons	Clusterwise correction based on cluster_threshold_beta
Software	SPM8
Voxelwise p	.01
Cluster extent	11 voxels (size not stated)
Statistical details	—
Findings	↓ L precuneus<br>↓ L anterior cingulate<br>↓ L posterior cingulate<br>↓ L basal ganglia<br>↓ R precuneus<br>↓ R posterior STS<br>↓ R posterior MTG<br>↓ R posterior cingulate<br>↓ R thalamus<br>↓ R hippocampus/MTL
Findings notes	—

Voxelwise analysis 4

First level contrast	Picture naming vs rest
Analysis class	Cross-sectional aphasia vs control
Group(s)	Aphasia T1 vs control T1
Covariate	—
Is the second level contrast valid in terms of the group(s), time point(s), and measures involved?	Yes
Is accuracy matched across the second level contrast?	Appear mismatched
Is reaction time matched across the second level contrast?	Unknown, not reported
Behavioral data notes	RT not reported for controls
Type of analysis	Voxelwise
Search volume	Whole brain
Correction for multiple comparisons	No direct comparison
Software	SPM8
Voxelwise p	—
Cluster extent	—
Statistical details	Qualitative comparison between activation in the first 5 TRs after each stimulus on p. 1101
Findings	None
Findings notes	The time course of response is stated to be similar in patients and controls, however the response in patients appears like it could be a couple of seconds slower

Complex analysis 1

First level contrast	Picture naming vs rest

Analysis class	Cross-sectional aphasia vs control
Group(s)	Aphasia vs control
Covariate	—
Is the second level contrast valid in terms of the group(s), time point(s), and measures involved?	Yes
Is accuracy matched across the second level contrast?	No, different
Is reaction time matched across the second level contrast?	Unknown, not reported
Behavioral data notes	RT not reported for controls
Type of analysis	Complex
Statistical details	Joint ICA was performed on structural and functional contrast images using FIT 1.2c. Three of the 7 components differed between groups in their loadings. Components were thresholded at $z > 3.09$, not corrected for multiple comparisons.
Findings	Other
Findings notes	Three structural-functional components are described in Figure 5 and Table 4. Functional activations are generally small and do not obviously relate to language processing. It is mentioned in the supplementary results that "the lesion maps may dominate estimation of the mixing parameter" (p. 10).

Notes

Excluded analyses	—

Kiran et al. (2015)

Reference

Authors	Kiran S, Meier EL, Kapse KJ, Glynn PA
Title	Changes in task-based effective connectivity in language networks following rehabilitation in post-stroke patients with aphasia
Reference	*Front Hum Neurosci* 2015; 9: 316
PMID	26106314
DOI	10.3389/fnhum.2015.00316

Participants

Language	US English
Inclusion criteria	Impaired naming
Number of individuals with aphasia	8
Number of control participants	8
Were any of the participants included in any previous studies?	No
Is age reported for patients and controls, and matched?	Yes (mean 58 years)
Is sex reported for patients and controls, and matched?	Yes (males: 7; females: 1)
Is handedness reported for patients and controls, and matched?	No
Is time post stroke onset reported and appropriate to the study design?	Yes (range 15-157 months)
To what extent is the nature of aphasia characterized?	Severity only
Language evaluation	WAB, BNT, PPT, CLQT
Aphasia severity	AQ range 48.0-97.2
Aphasia type	Not stated
First stroke only?	Yes

Stroke type	Not stated
To what extent is the lesion distribution characterized?	Lesion overlay
Lesion extent	24.2-431.6 cc
Lesion location	L MCA except for one patient with R MCA and aphasia
Participants notes	—

Imaging

Modality	fMRI
Is the study cross-sectional or longitudinal?	Longitudinal—chronic treatment
If longitudinal, at what time point(s) were imaging data acquired?	T1: pre-treatment/chronic; T2: post-treatment, ~10 weeks later
If longitudinal, was there any intervention between the time points?	Semantic feature-based treatment, 10 weeks
Is the scanner described?	Yes (Philips Achieva 3 Tesla)
Is the timing of stimulus presentation and image acquisition clearly described and appropriate?	No* (moderate limitation) (picture and scrambled conditions have different durations; ITI 2-4 s seems too short; total images acquired not stated)
Design type	Event-related
Total images acquired	not stated
Are the imaging acquisition parameters, including coverage, adequately described and appropriate?	Yes (whole brain)
Is preprocessing and intrasubject coregistration adequately described and appropriate?	Yes
Is first level model fitting adequately described and appropriate?	Yes
Is intersubject normalization adequately described and appropriate?	Yes
Imaging notes	controls were run on two different sets of parameters, neither of which was the same as the patients

Conditions

Are the conditions clearly described?	Yes

Condition	Response type	Repetitions	All groups could do?	All individuals could do?
picture naming (trained)	Word (overt)	40	Unknown	Unknown
picture naming (untrained)	Word (overt)	40	Unknown	Unknown
viewing scrambled images and saying "skip"	Word (overt)	80	Unknown	Unknown
semantic feature decision	Button press	40	Unknown	Unknown
visual decision	Button press	40	Unknown	Unknown

Conditions notes	—

Contrasts

Are the contrasts clearly described?	No (see specific limitation(s) below)

Contrast 1: picture naming (trained) vs viewing scrambled images and saying "skip"

Language condition	Picture naming (trained)
Control condition	Viewing scrambled images and saying "skip"
Are the conditions matched for visual demands?	Yes
Are the conditions matched for auditory demands?	Yes
Are the conditions matched for motor demands?	Yes
Are the conditions matched for cognitive/executive demands?	No
Is accuracy matched between the language and control tasks for all relevant groups?	Unknown, not reported
Is reaction time matched between the language	Unknown, not reported

and control tasks for all relevant groups?	
Behavioral data notes	—
Are control data reported in this paper or another that is referenced?	Somewhat
Does the contrast selectively activate plausible relevant language regions in the control group?	No
Are activations lateralized in the control data?	Somewhat
Control activation notes	Overlap of individual participant activation maps; somewhat lateralized frontal and temporal, but also bilateral occipito-temporal
Contrast notes	—

Contrast 2: semantic feature decision vs visual decision

Language condition	Semantic feature decision
Control condition	Visual decision
Are the conditions matched for visual demands?	Yes
Are the conditions matched for auditory demands?	Yes
Are the conditions matched for motor demands?	Yes
Are the conditions matched for cognitive/executive demands?	No
Is accuracy matched between the language and control tasks for all relevant groups?	Unknown, not reported
Is reaction time matched between the language and control tasks for all relevant groups?	Unknown, not reported
Behavioral data notes	—
Are control data reported in this paper or another that is referenced?	Somewhat
Does the contrast selectively activate plausible relevant language regions in the control group?	No
Are activations lateralized in the control data?	Somewhat
Control activation notes	Overlap of individual participant activation maps; somewhat lateralized frontal and temporal, but also bilateral occipito-temporal
Contrast notes	This contrast inferred but not described

Analyses

Are the analyses clearly described?	Yes

Voxelwise analysis 1

First level contrast	Picture naming (trained) vs viewing scrambled images and saying "skip"
Analysis class	Longitudinal change in aphasia
Group(s)	Aphasia T2 vs T1
Covariate	—
Is the second level contrast valid in terms of the group(s), time point(s), and measures involved?	Yes
Is accuracy matched across the second level contrast?	Unknown, not reported
Is reaction time matched across the second level contrast?	Unknown, not reported
Behavioral data notes	—
Type of analysis	Voxelwise
Search volume	Whole brain
Correction for multiple comparisons	No direct comparison
Software	SPM8
Voxelwise p	—
Cluster extent	—
Statistical details	Analyses were carried out in individual patients at $p < .001$, uncorrected; regions were considered activated when they were found in 6 or more (out of 8) patients

Findings	↑ L IFG<br>↑ L dorsolateral prefrontal cortex<br>↑ L ventral precentral/inferior frontal junction<br>↑ L dorsal precentral<br>↑ L SMA/medial prefrontal<br>↑ L supramarginal gyrus<br>↑ L angular gyrus<br>↑ L posterior MTG<br>↑ R IFG<br>↑ R dorsolateral prefrontal cortex<br>↑ R SMA/medial prefrontal<br>↑ R supramarginal gyrus<br>↑ R posterior STG<br>↑ R posterior MTG<br>↑ R posterior inferior temporal gyrus/fusiform gyrus
Findings notes	Regions are approximate since only broad regions are described in Table 6

Voxelwise analysis 2

First level contrast	Semantic feature decision vs visual decision
Analysis class	Longitudinal change in aphasia
Group(s)	Aphasia T2 vs T1
Covariate	—
Is the second level contrast valid in terms of the group(s), time point(s), and measures involved?	Yes
Is accuracy matched across the second level contrast?	Unknown, not reported
Is reaction time matched across the second level contrast?	Unknown, not reported
Behavioral data notes	—
Type of analysis	Voxelwise
Search volume	Whole brain
Correction for multiple comparisons	No direct comparison
Software	SPM8
Voxelwise p	—
Cluster extent	—
Statistical details	Analyses were carried out in individual patients at $p < .001$, uncorrected; regions were considered activated when they were found in 6 or more (out of 8) patients
Findings	↑ L ventral precentral/inferior frontal junction<br>↑ L dorsal precentral<br>↑ L posterior MTG<br>↑ R IFG<br>↑ R dorsolateral prefrontal cortex<br>↑ R SMA/medial prefrontal<br>↑ R angular gyrus<br>↑ R posterior STG<br>↑ R posterior MTG
Findings notes	Regions are approximate since only broad regions are described in Table 7

Notes

Excluded analyses	(1) DCM analyses; (2) activation for untrained categories, since this is reported only for individual patients in supplementary material

Sandberg et al. (2015)

Reference

Authors	Sandberg CW, Bohland JW, Kiran S
Title	Changes in functional connectivity related to direct training and generalization effects of a word finding treatment in chronic aphasia
Reference	*Brain Lang* 2015; 150: 103-116
PMID	26398158
DOI	10.1016/j.bandl.2015.09.002

Participants

Language	US English
Inclusion criteria	—
Number of individuals with aphasia	10
Number of control participants	0
Were any of the participants included in any previous studies?	No
Is age reported for patients and controls, and matched?	Yes (mean 59 years, range 47-75 years)
Is sex reported for patients and controls, and matched?	Yes (males: 7; females: 3)
Is handedness reported for patients and controls, and matched?	Yes (right: 10; left: 0)
Is time post stroke onset reported and appropriate to the study design?	Yes (range 7-134 months)
To what extent is the nature of aphasia characterized?	Comprehensive battery
Language evaluation	WAB, BNT, subtests from PALPA, PPT, CLQT
Aphasia severity	AQ range 41.7-99.2
Aphasia type	6 anomic, 2 conduction, 1 Broca's, 1 transcortical motor
First stroke only?	Not stated
Stroke type	Not stated
To what extent is the lesion distribution characterized?	Lesion overlay
Lesion extent	Range 0.3-256.0 cc
Lesion location	L MCA
Participants notes	—

Imaging

Modality	fMRI
Is the study cross-sectional or longitudinal?	Longitudinal—chronic treatment
If longitudinal, at what time point(s) were imaging data acquired?	T1: pre-treatment/chronic; T2: post-treatment, up to 10 weeks later
If longitudinal, was there any intervention between the time points?	Semantic feature-based treatment, 2 hours/day, 2 days/week, up to 10 weeks (depending on when criterion reached)
Is the scanner described?	Yes (Philips Achieva 3 Tesla)
Is the timing of stimulus presentation and image acquisition clearly described and appropriate?	No* (moderate limitation) (total images acquired not stated; ITI of 1-3 s seems short)
Design type	Event-related
Total images acquired	not stated
Are the imaging acquisition parameters, including coverage, adequately described and appropriate?	Yes (whole brain)
Is preprocessing and intrasubject coregistration adequately described and appropriate?	Yes
Is first level model fitting adequately described and appropriate?	Yes
Is intersubject normalization adequately described and appropriate?	Yes
Imaging notes	—

Conditions

Are the conditions clearly described?	Yes

Condition	Response type	Repetitions	All groups could do?	All individuals could do?
concreteness judgment (abstract words)	Button press	60	Yes	No
concreteness judgment (concrete words)	Button press	60	Yes	Yes
letter string judgment	Button press	60	Unknown	Unknown
rest	None	implicit baseline	N/A	N/A

Conditions notes	2 patients below chance on abstract words per supplementary table 2

Contrasts

Are the contrasts clearly described?	No (see specific limitation(s) below)

Contrast 1: concreteness judgment (abstract words, correct trials) vs rest

Language condition	Concreteness judgment (abstract words, correct trials)
Control condition	Rest
Are the conditions matched for visual demands?	No
Are the conditions matched for auditory demands?	Yes
Are the conditions matched for motor demands?	No
Are the conditions matched for cognitive/executive demands?	No
Is accuracy matched between the language and control tasks for all relevant groups?	N/A, tasks not comparable
Is reaction time matched between the language and control tasks for all relevant groups?	N/A, tasks not comparable
Behavioral data notes	—
Are control data reported in this paper or another that is referenced?	No
Does the contrast selectively activate plausible relevant language regions in the control group?	Unknown
Are activations lateralized in the control data?	Unknown
Control activation notes	—
Contrast notes	The concreteness judgment task was compared to the letter string judgment task to define ROIs for connectivity analysis, but the group analysis meeting criteria for this review appears to be based only on comparisons between time points on the concreteness judgment conditions

Contrast 2: concreteness judgment (concrete words, correct trials) vs rest

Language condition	Concreteness judgment (concrete words, correct trials)
Control condition	Rest
Are the conditions matched for visual demands?	No
Are the conditions matched for auditory demands?	Yes
Are the conditions matched for motor demands?	No
Are the conditions matched for cognitive/executive demands?	No
Is accuracy matched between the language and control tasks for all relevant groups?	N/A, tasks not comparable
Is reaction time matched between the language and control tasks for all relevant groups?	N/A, tasks not comparable
Behavioral data notes	—
Are control data reported in this paper or another that is referenced?	No
Does the contrast selectively activate plausible relevant language regions in the control group?	Unknown

Are activations lateralized in the control data?	Unknown
Control activation notes	—
Contrast notes	The concreteness judgment task was compared to the letter string judgment task to define ROIs for connectivity analysis, but the group analysis meeting criteria for this review appears to be based only on comparisons between time points on the concreteness judgment conditions

Analyses

Are the analyses clearly described?	No** (major limitation) (see specific limitation(s) below)

Voxelwise analysis 1

First level contrast	Concreteness judgment (abstract words, correct trials) vs rest
Analysis class	Longitudinal change in aphasia
Group(s)	Aphasia with response to treatment (n = 9) T2 vs T1
Covariate	—
Is the second level contrast valid in terms of the group(s), time point(s), and measures involved?	Yes
Is accuracy matched across the second level contrast?	Yes, matched
Is reaction time matched across the second level contrast?	Yes, matched
Behavioral data notes	—
Type of analysis	Voxelwise
Search volume	Whole brain
Correction for multiple comparisons	No correction
Software	SPM8
Voxelwise p	.001
Cluster extent	None
Statistical details	Images show peaks instead of activations
Findings	↑ L IFG pars opercularis<br>↑ L dorsolateral prefrontal cortex<br>↑ L SMA/medial prefrontal<br>↑ L inferior parietal lobule<br>↑ L supramarginal gyrus<br>↑ L angular gyrus<br>↑ L precuneus<br>↑ L posterior inferior temporal gyrus/fusiform gyrus<br>↑ L posterior cingulate<br>↑ L basal ganglia<br>↑ R orbitofrontal<br>↑ R supramarginal gyrus<br>↑ R angular gyrus<br>↑ R anterior temporal<br>↑ R occipital
Findings notes	—

Voxelwise analysis 2

First level contrast	Concreteness judgment (concrete words, correct trials) vs rest
Analysis class	Longitudinal change in aphasia
Group(s)	Aphasia with generalization of treatment effects to concrete words (n = 7) T2 vs T1
Covariate	—
Is the second level contrast valid in terms of the group(s), time point(s), and measures involved?	Yes
Is accuracy matched across the second level contrast?	Yes, matched
Is reaction time matched across the second level contrast?	Yes, matched

Behavioral data notes	—
Type of analysis	Voxelwise
Search volume	Whole brain
Correction for multiple comparisons	No correction
Software	SPM8
Voxelwise p	.001
Cluster extent	None
Statistical details	Images show peaks instead of activations
Findings	↑ L insula<br>↑ L inferior parietal lobule<br>↑ L supramarginal gyrus<br>↑ L precuneus<br>↑ L occipital<br>↑ R dorsolateral prefrontal cortex<br>↑ R ventral precentral/inferior frontal junction<br>↑ R posterior STG<br>↑ R posterior cingulate
Findings notes	—

Notes

Excluded analyses	Connectivity analyses due to degree of complexity, which precluded assessment

Geranmayeh et al. (2016)

Reference

Authors	Geranmayeh F, Leech R, Wise RJ
Title	Network dysfunction predicts speech production after left hemisphere stroke
Reference	*Neurology* 2016; 86: 1296-1305
PMID	26962070
DOI	10.1212/wnl.0000000000002537

Participants

Language	UK English
Inclusion criteria	No severe receptive aphasia
Number of individuals with aphasia	53
Number of control participants	24
Were any of the participants included in any previous studies?	No
Is age reported for patients and controls, and matched?	Yes (mean 62 ± 14 years, range 26-83 years)
Is sex reported for patients and controls, and matched?	No (males: 32; females: 21; controls were mostly female, unlike patients)
Is handedness reported for patients and controls, and matched?	Yes (right: 50; left: 3)
Is time post stroke onset reported and appropriate to the study design?	Yes (mean 111 ± 27 days, range 84-200 days)
To what extent is the nature of aphasia characterized?	Comprehensive battery
Language evaluation	CAT, QPA
Aphasia severity	"relatively mild stroke"; 17 patients were so mild that they were not aphasic per the CAT
Aphasia type	Not stated
First stroke only?	No
Stroke type	Not stated

To what extent is the lesion distribution characterized?	Lesion overlay
Lesion extent	Mean 25.4 ± 13.5 cc, range 0.3-168.0 cc
Lesion location	L; modest R involvement in 7 cases
Participants notes	Prior strokes were allowed only if no aphasia resulted

Imaging

Modality	fMRI
Is the study cross-sectional or longitudinal?	Cross-sectional
If longitudinal, at what time point(s) were imaging data acquired?	—
If longitudinal, was there any intervention between the time points?	—
Is the scanner described?	Yes (Siemens Trio 3 Tesla)
Is the timing of stimulus presentation and image acquisition clearly described and appropriate?	Yes
Design type	Event-related
Total images acquired	213
Are the imaging acquisition parameters, including coverage, adequately described and appropriate?	Yes (whole brain)
Is preprocessing and intrasubject coregistration adequately described and appropriate?	Yes
Is first level model fitting adequately described and appropriate?	Yes
Is intersubject normalization adequately described and appropriate?	Yes
Imaging notes	sparse sampling; mini-blocks of 2-4 trials

Conditions

Are the conditions clearly described?	Yes

Condition	Response type	Repetitions	All groups could do?	All individuals could do?
propositional speech production	Sentence (overt)	60	Yes	No
counting	Multiple words (overt)	48	Yes	Unknown
target decision	Button press	48	Yes	Unknown
rest	None	45	N/A	N/A

Conditions notes	—

Contrasts

Are the contrasts clearly described?	Yes

Contrast 1: propositional speech production vs rest

Language condition	Propositional speech production
Control condition	Rest
Are the conditions matched for visual demands?	No
Are the conditions matched for auditory demands?	No
Are the conditions matched for motor demands?	No
Are the conditions matched for cognitive/executive demands?	No
Is accuracy matched between the language and control tasks for all relevant groups?	N/A, tasks not comparable
Is reaction time matched between the language and control tasks for all relevant groups?	N/A, tasks not comparable
Behavioral data notes	—
Are control data reported in this paper or another	Somewhat

that is referenced?	
Does the contrast selectively activate plausible relevant language regions in the control group?	Somewhat
Are activations lateralized in the control data?	No
Control activation notes	Control data for univariate analysis in Geranmayeh et al. (2014), but note that the present paper does not describe a univariate analysis; control activations reflect speech rather than language
Contrast notes	—

Contrast 2: propositional speech production vs counting

Language condition	Propositional speech production
Control condition	Counting
Are the conditions matched for visual demands?	No
Are the conditions matched for auditory demands?	Yes
Are the conditions matched for motor demands?	Yes
Are the conditions matched for cognitive/executive demands?	No
Is accuracy matched between the language and control tasks for all relevant groups?	N/A, tasks not comparable
Is reaction time matched between the language and control tasks for all relevant groups?	N/A, tasks not comparable
Behavioral data notes	—
Are control data reported in this paper or another that is referenced?	Somewhat
Does the contrast selectively activate plausible relevant language regions in the control group?	Yes
Are activations lateralized in the control data?	Yes
Control activation notes	Control data for univariate analysis in Geranmayeh et al. (2014), but note that the present paper does not describe a univariate analysis; control activations are L frontal, L pSTS, L SMA, L > R occipito-temporal
Contrast notes	—

Contrast 3: propositional speech production vs target decision

Language condition	Propositional speech production
Control condition	Target decision
Are the conditions matched for visual demands?	No
Are the conditions matched for auditory demands?	No
Are the conditions matched for motor demands?	No
Are the conditions matched for cognitive/executive demands?	No
Is accuracy matched between the language and control tasks for all relevant groups?	N/A, tasks not comparable
Is reaction time matched between the language and control tasks for all relevant groups?	N/A, tasks not comparable
Behavioral data notes	—
Are control data reported in this paper or another that is referenced?	No
Does the contrast selectively activate plausible relevant language regions in the control group?	Unknown
Are activations lateralized in the control data?	Unknown
Control activation notes	—
Contrast notes	—

Analyses

Are the analyses clearly described?	No (see specific limitation(s) below)

ROI analysis 1

First level contrast	Propositional speech production vs rest
Analysis class	Cross-sectional aphasia vs control
Group(s)	Aphasia vs control
Covariate	—
Is the second level contrast valid in terms of the group(s), time point(s), and measures involved?	Yes
Is accuracy matched across the second level contrast?	No, different
Is reaction time matched across the second level contrast?	Unknown, not reported
Behavioral data notes	Difference in AICW/trial
Type of analysis	Regions of interest (ROI)
ROI type	Functional
How many ROIs are there?	4
What are the ROI(s)?	(1) L fronto-temporo-parietal network; (2) R fronto-temporo-parietal network; (3) cingulo-opercular network; (4) default mode network
How are the ROI(s) defined?	Identified using ICA in controls
Correction for multiple comparisons	No correction
Statistical details	Circular because ROIs defined in one group
Findings	↑ L insula<br>↑ L anterior cingulate<br>↑ R insula<br>↑ R anterior cingulate
Findings notes	—

ROI analysis 2

First level contrast	Propositional speech production vs counting
Analysis class	Cross-sectional aphasia vs control
Group(s)	Aphasia vs control
Covariate	—
Is the second level contrast valid in terms of the group(s), time point(s), and measures involved?	Yes
Is accuracy matched across the second level contrast?	No, different
Is reaction time matched across the second level contrast?	Unknown, not reported
Behavioral data notes	Difference in AICW/trial
Type of analysis	Regions of interest (ROI)
ROI type	Functional
How many ROIs are there?	4
What are the ROI(s)?	(1) L fronto-temporo-parietal network; (2) R fronto-temporo-parietal network; (3) cingulo-opercular network; (4) default mode network
How are the ROI(s) defined?	Identified using ICA in controls
Correction for multiple comparisons	No correction
Statistical details	Circular because ROIs defined in one group
Findings	↑ L insula<br>↑ L anterior cingulate<br>↑ R insula<br>↑ R anterior cingulate<br>↓ L IFG<br>↓ L inferior parietal lobule<br>↓ L posterior inferior temporal gyrus/fusiform gyrus
Findings notes	—

ROI analysis 3

First level contrast	Propositional speech production vs target decision
Analysis class	Cross-sectional aphasia vs control
Group(s)	Aphasia vs control
Covariate	—
Is the second level contrast valid in terms of the group(s), time point(s), and measures involved?	Yes
Is accuracy matched across the second level contrast?	No, different
Is reaction time matched across the second level contrast?	Unknown, not reported
Behavioral data notes	Difference in AICW/trial
Type of analysis	Regions of interest (ROI)
ROI type	Functional
How many ROIs are there?	4
What are the ROI(s)?	(1) L fronto-temporo-parietal network; (2) R fronto-temporo-parietal network; (3) cingulo-opercular network; (4) default mode network
How are the ROI(s) defined?	Identified using ICA in controls
Correction for multiple comparisons	No correction
Statistical details	Circular because ROIs defined in one group
Findings	None
Findings notes	—

Complex analysis 1

First level contrast	Propositional speech production vs rest
Analysis class	Cross-sectional aphasia vs control
Group(s)	Aphasia vs control
Covariate	—
Is the second level contrast valid in terms of the group(s), time point(s), and measures involved?	Yes
Is accuracy matched across the second level contrast?	No, different
Is reaction time matched across the second level contrast?	Unknown, not reported
Behavioral data notes	Difference in AICW/trial
Type of analysis	Complex
Statistical details	Activity was compared between pairs of ICA-derived networks. However, circularity was introduced because the networks were defined based on the control group.
Findings	Other
Findings notes	Patients showed greater differential activation than controls between (1) L fronto-temporo-parietal network and the DMN; (2) R fronto-temporo-parietal network and the DMN; (3) cingulo-opercular network and the DMN.

Complex analysis 2

First level contrast	Propositional speech production vs rest
Analysis class	Cross-sectional correlation with language or other measure
Group(s)	Aphasia
Covariate	Appropriate information-carrying words
Is the second level contrast valid in terms of the group(s), time point(s), and measures involved?	Yes
Is accuracy matched across the second level contrast?	Accuracy is covariate
Is reaction time matched across the second level contrast?	Unknown, not reported
Behavioral data notes	—
Type of analysis	Complex
Statistical details	Multiple regression was used to determine whether differential activation between networks

	was predictive of the behavioral measure: appropriate information-carrying words. There is no issue of circularity with this analysis since it involved only individuals with aphasia.
Findings	Other
Findings notes	Differential activation between L fronto-temporo-parietal network and the DMN was positively correlated with AICW. Differential activation between R fronto-temporo-parietal network and the DMN was negatively correlated with AICW.

Complex analysis 3

First level contrast	Propositional speech production vs rest
Analysis class	Cross-sectional aphasia vs control
Group(s)	Aphasia vs control
Covariate	—
Is the second level contrast valid in terms of the group(s), time point(s), and measures involved?	Yes
Is accuracy matched across the second level contrast?	No, different
Is reaction time matched across the second level contrast?	Unknown, not reported
Behavioral data notes	Difference in AICW/trial
Type of analysis	Complex
Statistical details	PPI analyses were used to investigate how the speech condition modulated functional connectivity between (1) L fronto-temporo-parietal network and the DMN; (2) R fronto-temporo-parietal network and the DMN. However, circularity was introduced because the networks were defined based on the control group.
Findings	Other
Findings notes	In controls, the L FTP network reduced connectivity with the DMN during speech, while the R FTP network increased connectivity with the DMN during speech. Both of these interactions were significantly decreased in patients. This was also true for contrasts 2 and 3.

Notes

Excluded analyses	It is mentioned that LFTP and DMN activation did not correlate with speech performance, but insufficient details are provided regarding this analysis

Griffis et al. (2016)

Reference

Authors	Griffis JC, Nenert R, Allendorfer JB, Szaflarski JP
Title	Interhemispheric plasticity following intermittent theta burst stimulation in chronic poststroke aphasia
Reference	*Neural Plast* 2016; 2016: 4796906
PMID	26881111
DOI	10.1155/2016/4796906

Participants

Language	US English
Inclusion criteria	Moderate aphasia, L MCA
Number of individuals with aphasia	8 (plus 3 excluded: 2 metallic artifact; 1 seizure at time of stroke)
Number of control participants	0
Were any of the participants included in any previous studies?	Yes (same patients as Szaflarski et al. (2011); different fMRI paradigm acquired in the same sessions)
Is age reported for patients and controls, and matched?	Yes (mean 54.4 ± 12.7 years)
Is sex reported for patients and controls, and matched?	Yes (males: 4; females: 4)

Is handedness reported for patients and controls, and matched?	Yes (right: 8; left: 0)
Is time post stroke onset reported and appropriate to the study design?	Yes (mean 5.3 ± 3.6 years)
To what extent is the nature of aphasia characterized?	Severity and type
Language evaluation	BNT; phonemic fluency, semantic fluency, complex ideation from BDAE, PPVT, communicative activities log
Aphasia severity	Moderate
Aphasia type	4 Broca's, 3 anomic, 1 anomic/conduction
First stroke only?	Not stated
Stroke type	Not stated
To what extent is the lesion distribution characterized?	Individual lesions
Lesion extent	Range 1.4-52.5 cc
Lesion location	L MCA
Participants notes	—

Imaging

Modality	fMRI
Is the study cross-sectional or longitudinal?	Longitudinal—chronic treatment
If longitudinal, at what time point(s) were imaging data acquired?	T1: pre-treatment/chronic; T2: post-treatment, ~2 weeks later
If longitudinal, was there any intervention between the time points?	RTMS to residual activation near Broca's area, 5 sessions/week, 2 weeks
Is the scanner described?	Yes (Varian Unity INOVA 4 Tesla)
Is the timing of stimulus presentation and image acquisition clearly described and appropriate?	Yes
Design type	Block
Total images acquired	140
Are the imaging acquisition parameters, including coverage, adequately described and appropriate?	Yes (whole brain)
Is preprocessing and intrasubject coregistration adequately described and appropriate?	Yes
Is first level model fitting adequately described and appropriate?	Yes
Is intersubject normalization adequately described and appropriate?	No (lesion impact not addressed)
Imaging notes	—

Conditions

Are the conditions clearly described?	Yes

Condition	Response type	Repetitions	All groups could do?	All individuals could do?
verb generation	Multiple words (covert)	7	Yes	Yes
finger tapping	Other	7	Unknown	Unknown

Conditions notes	—

Contrasts

Are the contrasts clearly described?	Yes

Contrast 1: verb generation vs finger tapping

Language condition	Verb generation
Control condition	Finger tapping
Are the conditions matched for visual demands?	Yes

Are the conditions matched for auditory demands?	Yes
Are the conditions matched for motor demands?	No
Are the conditions matched for cognitive/executive demands?	No
Is accuracy matched between the language and control tasks for all relevant groups?	N/A, tasks not comparable
Is reaction time matched between the language and control tasks for all relevant groups?	N/A, tasks not comparable
Behavioral data notes	—
Are control data reported in this paper or another that is referenced?	Yes
Does the contrast selectively activate plausible relevant language regions in the control group?	Yes
Are activations lateralized in the control data?	Somewhat
Control activation notes	Control data in Szaflarski et al. (2008); frontal activation L-lateralized, temporal less so
Contrast notes	—

Analyses

Are the analyses clearly described?	No (see specific limitation(s) below)

Voxelwise analysis 1

First level contrast	Verb generation vs finger tapping
Analysis class	Longitudinal change in aphasia
Group(s)	Aphasia T2 vs T1
Covariate	—
Is the second level contrast valid in terms of the group(s), time point(s), and measures involved?	Somewhat (patients improved only on semantic fluency)
Is accuracy matched across the second level contrast?	Unknown, not reported
Is reaction time matched across the second level contrast?	Unknown, not reported
Behavioral data notes	—
Type of analysis	Voxelwise
Search volume	Whole brain
Correction for multiple comparisons	No correction
Software	SPM12
Voxelwise p	.001
Cluster extent	None
Statistical details	—
Findings	↑ L IFG pars opercularis<br>↑ R cerebellum<br>↑ R thalamus<br>↓ R anterior temporal<br>↓ R cerebellum
Findings notes	Based on description in text; it is noted that no regions survived FDR correction

ROI analysis 1

First level contrast	Verb generation vs finger tapping
Analysis class	Longitudinal change in aphasia
Group(s)	Aphasia T2 vs T1
Covariate	—
Is the second level contrast valid in terms of the group(s), time point(s), and measures involved?	Somewhat (patients improved only on semantic fluency)
Is accuracy matched across the second level contrast?	Unknown, not reported
Is reaction time matched across the second level	Unknown, not reported

contrast?	
Behavioral data notes	—
Type of analysis	Regions of interest (ROI)
ROI type	Mixed
How many ROIs are there?	3
What are the ROI(s)?	(1) L IFG; (2) R IFG; (3) frontal LI
How are the ROI(s) defined?	First principal component of 8 mm spheres defined based on previously reported control peaks
Correction for multiple comparisons	False discovery rate (FDR)
Statistical details	Lesion volume included in model
Findings	↑ L IFG<br>↓ R IFG<br>↑ LI (frontal)
Findings notes	—

ROI analysis 2

First level contrast	Verb generation vs finger tapping
Analysis class	Longitudinal correlation with language or other measure
Group(s)	Aphasia T2 vs T1
Covariate	Δ semantic fluency
Is the second level contrast valid in terms of the group(s), time point(s), and measures involved?	Somewhat (patients improved only on semantic fluency)
Is accuracy matched across the second level contrast?	Unknown, not reported
Is reaction time matched across the second level contrast?	Unknown, not reported
Behavioral data notes	—
Type of analysis	Regions of interest (ROI)
ROI type	Mixed
How many ROIs are there?	3
What are the ROI(s)?	(1) L IFG; (2) R IFG; (3) frontal LI
How are the ROI(s) defined?	First principal component of 8 mm spheres defined based on previously reported control peaks
Correction for multiple comparisons	False discovery rate (FDR)
Statistical details	Lesion volume included in model
Findings	↓ R IFG
Findings notes	Decreased R IFG activation was correlated with improved semantic fluency

Complex analysis 1

First level contrast	Verb generation vs finger tapping
Analysis class	Longitudinal change in aphasia
Group(s)	Aphasia T2 vs T1
Covariate	—
Is the second level contrast valid in terms of the group(s), time point(s), and measures involved?	Somewhat (patients improved only on semantic fluency)
Is accuracy matched across the second level contrast?	Unknown, not reported
Is reaction time matched across the second level contrast?	Unknown, not reported
Behavioral data notes	—
Type of analysis	Complex
Statistical details	PPI analyses were used to investigate change over time in modulation by verb generation of functional connectivity between L IFG and R IFG.
Findings	Other
Findings notes	There was a significant decrease in modulation by verb generation of functional connectivity between L IFG and R IFG ($p = 0.03$). Prior to TMS, connectivity increased during verb

	generation compared to finger tapping, while after TMS, connectivity decreased during verb generation compared to finger tapping.

Complex analysis 2

First level contrast	Verb generation vs finger tapping
Analysis class	Longitudinal correlation with language or other measure
Group(s)	Aphasia T2 vs T1
Covariate	Δ semantic fluency in association with modulation of interhemispheric IFG connectivity by verb generation
Is the second level contrast valid in terms of the group(s), time point(s), and measures involved?	Somewhat (patients improved only on semantic fluency)
Is accuracy matched across the second level contrast?	Unknown, not reported
Is reaction time matched across the second level contrast?	Unknown, not reported
Behavioral data notes	—
Type of analysis	Complex
Statistical details	PPI analyses were used to investigate whether change over time in modulation by verb generation of functional connectivity between L IFG and R IFG was associated with changes in semantic fluency scores, which are limited as a measure of language improvement.
Findings	None
Findings notes	—

Complex analysis 3

First level contrast	Verb generation vs finger tapping
Analysis class	Longitudinal change in aphasia
Group(s)	Aphasia T2 vs T1
Covariate	—
Is the second level contrast valid in terms of the group(s), time point(s), and measures involved?	Somewhat (patients improved only on semantic fluency)
Is accuracy matched across the second level contrast?	Unknown, not reported
Is reaction time matched across the second level contrast?	Unknown, not reported
Behavioral data notes	—
Type of analysis	Complex
Statistical details	PPI analyses were used to investigate change over time in modulation by verb generation of functional connectivity between R IFG and all other brain regions. Voxelwise $p < .001$, not corrected for multiple comparisons.
Findings	Other
Findings notes	Reduced connectivity was observed in the L IFG pars opercularis, L anterior temporal lobe, L occipital lobe, L basal ganglia, R SMA and pre-SMA, R somato-motor cortex, R posterior MTG, and R cerebellum. It is noted that no regions survived FDR correction.

Notes

Excluded analyses	(1) correlations between lesion volume and functional measures, not described in sufficient detail; (2) ad hoc analyses in section 3.4

Sims et al. (2016)

Reference

Authors	Sims JA, Kapse K, Glynn P, Sandberg C, Tripodis Y, Kiran S
Title	The relationships between the amount of spared tissue, percent signal change, and accuracy in semantic processing in aphasia

Reference	*Neuropsychologia* 2016; 84: 113-126
PMID	26775192
DOI	10.1016/j.neuropsychologia.2015.10.019

Participants

Language	US English
Inclusion criteria	Some spared tissue in L IFG
Number of individuals with aphasia	14 (plus 2 excluded: 1 had no spared tissue in the L IFG; 1 had a R hemisphere stroke)
Number of control participants	8
Were any of the participants included in any previous studies?	Yes (although not stated, it is apparent that many of the patients were included in Sandberg et al. (2015))
Is age reported for patients and controls, and matched?	Yes (mean 59.7 years, range 48-75 years)
Is sex reported for patients and controls, and matched?	Yes (males: 10; females: 4)
Is handedness reported for patients and controls, and matched?	Yes (right: 14; left: 0)
Is time post stroke onset reported and appropriate to the study design?	Yes (mean 6 years, range 6 months-13 years)
To what extent is the nature of aphasia characterized?	Severity and type
Language evaluation	WAB, BNT, PPT, CLQT
Aphasia severity	AQ range 48.0-99.2
Aphasia type	4 anomic, 2 Broca's, 2 conduction, 2 transcortical motor, 1 anomic or transcortical motor, 1 Broca's or conduction, 1 "N/A", 1 Wernicke's or conduction
First stroke only?	Not stated
Stroke type	Not stated
To what extent is the lesion distribution characterized?	Lesion overlay
Lesion extent	Not stated
Lesion location	L MCA
Participants notes	—

Imaging

Modality	fMRI
Is the study cross-sectional or longitudinal?	Cross-sectional
If longitudinal, at what time point(s) were imaging data acquired?	—
If longitudinal, was there any intervention between the time points?	—
Is the scanner described?	Yes (Philips Achieva 3 Tesla)
Is the timing of stimulus presentation and image acquisition clearly described and appropriate?	No (total images acquired not stated)
Design type	Event-related
Total images acquired	not stated
Are the imaging acquisition parameters, including coverage, adequately described and appropriate?	Yes (whole brain)
Is preprocessing and intrasubject coregistration adequately described and appropriate?	Yes
Is first level model fitting adequately described and appropriate?	Yes
Is intersubject normalization adequately described and appropriate?	Yes
Imaging notes	no smoothing

Conditions

Are the conditions clearly described?	No (number of visual decision trials not reported)

Condition	Response type	Repetitions	All groups could do?	All individuals could do?
semantic feature decision	Button press	64	Yes	Unknown
visual decision	Button press	not stated	Yes	Unknown
semantic relatedness decision	Button press	50	Yes	Unknown
pseudoword identity decision	Button press	50	Yes	Unknown
rest	None	implicit baseline	N/A	N/A

Conditions notes	—

Contrasts

Are the contrasts clearly described?	Yes

Contrast 1: semantic feature decision (6 patients, 4 controls) or semantic relatedness decision (8 patients, 4 controls) vs visual decision or pseudoword identity decision

Language condition	Semantic feature decision (6 patients, 4 controls) or semantic relatedness decision (8 patients, 4 controls)
Control condition	Visual decision or pseudoword identity decision
Are the conditions matched for visual demands?	Yes
Are the conditions matched for auditory demands?	Yes
Are the conditions matched for motor demands?	Yes
Are the conditions matched for cognitive/executive demands?	Yes
Is accuracy matched between the language and control tasks for all relevant groups?	No, different
Is reaction time matched between the language and control tasks for all relevant groups?	Unknown, not reported
Behavioral data notes	—
Are control data reported in this paper or another that is referenced?	No
Does the contrast selectively activate plausible relevant language regions in the control group?	Unknown
Are activations lateralized in the control data?	Unknown
Control activation notes	—
Contrast notes	8 patients and 4 controls performed one paradigm, while 6 patients and 4 controls performed another; the data were combined based on the assumption that similar processes were implicated by the two contrasts

Analyses

Are the analyses clearly described?	No* (moderate limitation) (see specific limitation(s) below)

ROI analysis 1

First level contrast	Semantic feature decision (6 patients, 4 controls) or semantic relatedness decision (8 patients, 4 controls) vs visual decision or pseudoword identity decision
Analysis class	Cross-sectional correlation with language or other measure
Group(s)	Aphasia
Covariate	Semantic feature decision accuracy
Is the second level contrast valid in terms of the group(s), time point(s), and measures involved?	Yes
Is accuracy matched across the second level contrast?	Accuracy is covariate
Is reaction time matched across the second level contrast?	Unknown, not reported
Behavioral data notes	—

Type of analysis	Regions of interest (ROI)
ROI type	Anatomical
How many ROIs are there?	16
What are the ROI(s)?	(1) L IFG pars orbitalis; (2) L IFG pars opercularis; (3) L IFG pars triangularis; (4) L SFG; (5) L MFG; (6) L MTG; (7) L AG/SMG; (8) L ACC; (9-16) homotopic counterparts
How are the ROI(s) defined?	AAL
Correction for multiple comparisons	No correction
Statistical details	—
Findings	↑ L IFG pars opercularis<br>↑ L IFG pars triangularis
Findings notes	—

ROI analysis 2

First level contrast	Semantic feature decision (6 patients, 4 controls) or semantic relatedness decision (8 patients, 4 controls) vs visual decision or pseudoword identity decision
Analysis class	Cross-sectional correlation with language or other measure
Group(s)	Aphasia
Covariate	WAB AQ
Is the second level contrast valid in terms of the group(s), time point(s), and measures involved?	Yes
Is accuracy matched across the second level contrast?	Unknown, not reported
Is reaction time matched across the second level contrast?	Unknown, not reported
Behavioral data notes	—
Type of analysis	Regions of interest (ROI)
ROI type	Anatomical
How many ROIs are there?	16
What are the ROI(s)?	(1) L IFG pars orbitalis; (2) L IFG pars opercularis; (3) L IFG pars triangularis; (4) L SFG; (5) L MFG; (6) L MTG; (7) L AG/SMG; (8) L ACC; (9-16) homotopic counterparts
How are the ROI(s) defined?	AAL
Correction for multiple comparisons	No correction
Statistical details	—
Findings	None
Findings notes	—

ROI analysis 3

First level contrast	Semantic feature decision (6 patients, 4 controls) or semantic relatedness decision (8 patients, 4 controls) vs visual decision or pseudoword identity decision
Analysis class	Cross-sectional correlation with language or other measure
Group(s)	Aphasia
Covariate	BNT
Is the second level contrast valid in terms of the group(s), time point(s), and measures involved?	Yes
Is accuracy matched across the second level contrast?	Unknown, not reported
Is reaction time matched across the second level contrast?	Unknown, not reported
Behavioral data notes	—
Type of analysis	Regions of interest (ROI)
ROI type	Anatomical
How many ROIs are there?	16
What are the ROI(s)?	(1) L IFG pars orbitalis; (2) L IFG pars opercularis; (3) L IFG pars triangularis; (4) L SFG; (5) L MFG; (6) L MTG; (7) L AG/SMG; (8) L ACC; (9-16) homotopic counterparts
How are the ROI(s) defined?	AAL
Correction for multiple comparisons	No correction

Statistical details	—
Findings	None
Findings notes	—

ROI analysis 4

First level contrast	Semantic feature decision (6 patients, 4 controls) or semantic relatedness decision (8 patients, 4 controls) vs visual decision or pseudoword identity decision
Analysis class	Cross-sectional correlation with language or other measure
Group(s)	Aphasia
Covariate	PPT
Is the second level contrast valid in terms of the group(s), time point(s), and measures involved?	Yes
Is accuracy matched across the second level contrast?	Unknown, not reported
Is reaction time matched across the second level contrast?	Unknown, not reported
Behavioral data notes	—
Type of analysis	Regions of interest (ROI)
ROI type	Anatomical
How many ROIs are there?	16
What are the ROI(s)?	(1) L IFG pars orbitalis; (2) L IFG pars opercularis; (3) L IFG pars triangularis; (4) L SFG; (5) L MFG; (6) L MTG; (7) L AG/SMG; (8) L ACC; (9-16) homotopic counterparts
How are the ROI(s) defined?	AAL
Correction for multiple comparisons	No correction
Statistical details	—
Findings	None
Findings notes	—

ROI analysis 5

First level contrast	Semantic feature decision (6 patients, 4 controls) or semantic relatedness decision (8 patients, 4 controls) vs visual decision or pseudoword identity decision
Analysis class	Cross-sectional correlation with language or other measure
Group(s)	Aphasia
Covariate	Lesion volume
Is the second level contrast valid in terms of the group(s), time point(s), and measures involved?	Yes
Is accuracy matched across the second level contrast?	Yes, matched
Is reaction time matched across the second level contrast?	Unknown, not reported
Behavioral data notes	No correlation between lesion volume and accuracy, not clear whether control condition accuracy was also tested
Type of analysis	Regions of interest (ROI)
ROI type	Anatomical
How many ROIs are there?	8
What are the ROI(s)?	As above but only in the R hemisphere
How are the ROI(s) defined?	AAL
Correction for multiple comparisons	No correction
Statistical details	—
Findings	↑ R supramarginal gyrus<br>↑ R angular gyrus<br>↑ R posterior MTG
Findings notes	MTG included anterior too; SMG/AG was single ROI

Complex analysis 1

First level contrast	Semantic feature decision (6 patients, 4 controls) or semantic relatedness decision (8 patients,

	4 controls) vs visual decision or pseudoword identity decision
Analysis class	Cross-sectional correlation with language or other measure
Group(s)	Aphasia
Covariate	Lesion status of 8 ROIs
Is the second level contrast valid in terms of the group(s), time point(s), and measures involved?	Yes
Is accuracy matched across the second level contrast?	Unknown, not reported
Is reaction time matched across the second level contrast?	Unknown, not reported
Behavioral data notes	—
Type of analysis	Complex
Statistical details	Multivariate mixed-effects linear regression analyses were used to identify relationships between structural damage to 8 regions, and functional activation in 16 regions. Results were corrected for multiple comparisons based on FDR. This analysis was not described in sufficient detail.
Findings	Other
Findings notes	Sparing of the L ACC and L SFG was associated with more functional activation in many regions, however this is difficult to interpret since these regions were largely or completely spared in many patients. Damage to the L IFG pars orbitalis, L MTG and L AG/SMG was associated with activation of the L ACC, L SFG (and other regions) potentially indicative of compensatory processing.

Complex analysis 2

First level contrast	Semantic feature decision (6 patients, 4 controls) or semantic relatedness decision (8 patients, 4 controls) vs visual decision or pseudoword identity decision
Analysis class	Cross-sectional aphasia vs control
Group(s)	Aphasia vs control
Covariate	—
Is the second level contrast valid in terms of the group(s), time point(s), and measures involved?	Yes
Is accuracy matched across the second level contrast?	Unknown, not reported
Is reaction time matched across the second level contrast?	Unknown, not reported
Behavioral data notes	—
Type of analysis	Complex
Statistical details	Correlations were computed between functional activation in 16 regions, and qualitatively compared between patients and controls (p. 123). There was no correction for multiple comparisons.
Findings	Other
Findings notes	In controls, all regions were generally correlated with one another. This was largely true in patients too, with the exception of the R IFG pars orbitalis, which was negatively correlated with the L IFG.

Notes

Excluded analyses	PCA analysis (section 3.4.1)

Darkow et al. (2017)

Reference

Authors	Darkow R, Martin A, Würtz A, Flöel A, Meinzer M
Title	Transcranial direct current stimulation effects on neural processing in post-stroke aphasia
Reference	*Hum Brain Mapp* 2017; 38: 1518-1531

PMID	27859982
DOI	10.1002/hbm.23469

Participants

Language	German
Inclusion criteria	L hand motor area spared; mild aphasia
Number of individuals with aphasia	16
Number of control participants	16
Were any of the participants included in any previous studies?	No
Is age reported for patients and controls, and matched?	Yes (mean 56.7 ± 10.1 years)
Is sex reported for patients and controls, and matched?	Yes (males: 10; females: 6)
Is handedness reported for patients and controls, and matched?	Yes (right: 16; left: 0)
Is time post stroke onset reported and appropriate to the study design?	Yes (mean 54.3 ± 45.3 months, range 12-169 months)
To what extent is the nature of aphasia characterized?	Comprehensive battery
Language evaluation	AAT
Aphasia severity	Mild
Aphasia type	Not stated
First stroke only?	Not stated
Stroke type	Not stated
To what extent is the lesion distribution characterized?	Lesion overlay
Lesion extent	Range 9.7-165.1 cc
Lesion location	L MCA not including hand motor area
Participants notes	—

Imaging

Modality	fMRI
Is the study cross-sectional or longitudinal?	Longitudinal—chronic treatment
If longitudinal, at what time point(s) were imaging data acquired?	T1/T2: chronic; tDCS and sham sessions in randomized order
If longitudinal, was there any intervention between the time points?	—
Is the scanner described?	Yes (Siemens Trio 3 Tesla)
Is the timing of stimulus presentation and image acquisition clearly described and appropriate?	Yes
Design type	Event-related
Total images acquired	100
Are the imaging acquisition parameters, including coverage, adequately described and appropriate?	Yes (whole brain)
Is preprocessing and intrasubject coregistration adequately described and appropriate?	Yes
Is first level model fitting adequately described and appropriate?	Yes
Is intersubject normalization adequately described and appropriate?	Yes
Imaging notes	sparse sampling

Conditions

Are the conditions clearly described?	Yes

Condition	Response type	Repetitions	All groups could do?	All individuals could do?
picture naming	Word (overt)	80	Yes	Yes
rest	None	20	N/A	N/A

Conditions notes	—

Contrasts

Are the contrasts clearly described?	Yes

Contrast 1: picture naming vs rest

Language condition	Picture naming
Control condition	Rest
Are the conditions matched for visual demands?	No
Are the conditions matched for auditory demands?	No
Are the conditions matched for motor demands?	No
Are the conditions matched for cognitive/executive demands?	No
Is accuracy matched between the language and control tasks for all relevant groups?	N/A, tasks not comparable
Is reaction time matched between the language and control tasks for all relevant groups?	N/A, tasks not comparable
Behavioral data notes	—
Are control data reported in this paper or another that is referenced?	No
Does the contrast selectively activate plausible relevant language regions in the control group?	Unknown
Are activations lateralized in the control data?	Unknown
Control activation notes	—
Contrast notes	—

Analyses

Are the analyses clearly described?	Yes

Voxelwise analysis 1

First level contrast	Picture naming vs rest
Analysis class	Cross-sectional between two groups with aphasia
Group(s)	Aphasia after tDCS (n = 16) vs aphasia after sham stimulation (n = 16); same patients, order counterbalanced, repeated measures
Covariate	—
Is the second level contrast valid in terms of the group(s), time point(s), and measures involved?	Somewhat (no behavioral difference)
Is accuracy matched across the second level contrast?	Yes, matched
Is reaction time matched across the second level contrast?	Yes, matched
Behavioral data notes	—
Type of analysis	Voxelwise
Search volume	Whole brain
Correction for multiple comparisons	Clusterwise correction with with GRFT and stringent voxelwise p
Software	SPM8
Voxelwise p	.001
Cluster extent	Based on GRFT
Statistical details	Repeated measures
Findings	↓ L insula<br>↓ L anterior cingulate

	↓ R occipital<br>↓ R anterior cingulate
Findings notes	—

ROI analysis 1

First level contrast	Picture naming vs rest
Analysis class	Cross-sectional aphasia vs control
Group(s)	Aphasia after sham stimulation (n = 16) vs control
Covariate	—
Is the second level contrast valid in terms of the group(s), time point(s), and measures involved?	Yes
Is accuracy matched across the second level contrast?	Appear similar
Is reaction time matched across the second level contrast?	Unknown, not reported
Behavioral data notes	Patients named > 90% correctly in all sessions; control RT not reported
Type of analysis	Regions of interest (ROI)
ROI type	Functional
How many ROIs are there?	3
What are the ROI(s)?	(1) bilateral anterior cingulate; (2) L insula; (3) R lingual gyrus
How are the ROI(s) defined?	Regions that were less active in patients with tDCS vs sham
Correction for multiple comparisons	No correction
Statistical details	Circular because ROIs defined in one group
Findings	↑ L insula<br>↑ L anterior cingulate<br>↑ R anterior cingulate
Findings notes	—

ROI analysis 2

First level contrast	Picture naming vs rest
Analysis class	Cross-sectional aphasia vs control
Group(s)	Aphasia after tDCS (n = 16) vs control
Covariate	—
Is the second level contrast valid in terms of the group(s), time point(s), and measures involved?	Yes
Is accuracy matched across the second level contrast?	Appear similar
Is reaction time matched across the second level contrast?	Unknown, not reported
Behavioral data notes	Patients named > 90% correctly in all sessions; control RT not reported
Type of analysis	Regions of interest (ROI)
ROI type	Functional
How many ROIs are there?	3
What are the ROI(s)?	(1) bilateral anterior cingulate; (2) L insula; (3) R lingual gyrus
How are the ROI(s) defined?	Regions that were less active in patients with tDCS vs sham
Correction for multiple comparisons	No correction
Statistical details	Circular because ROIs defined in one group
Findings	None
Findings notes	—

Complex analysis 1

First level contrast	Picture naming vs rest
Analysis class	Cross-sectional between two groups with aphasia
Group(s)	Aphasia after tDCS (n = 16) vs aphasia after sham stimulation (n = 16); same patients, order counterbalanced, repeated measures

Covariate	—
Is the second level contrast valid in terms of the group(s), time point(s), and measures involved?	Somewhat (no behavioral difference)
Is accuracy matched across the second level contrast?	Yes, matched
Is reaction time matched across the second level contrast?	Yes, matched
Behavioral data notes	—
Type of analysis	Complex
Statistical details	ICA was used to derive three task-relevant components: language, motor and visual. Thresholding of the functional maps is not described, but they appear to reflect coherent components of a picture naming network. These components were compared between stimulation conditions in terms of mean activity and power in three frequency bins. It should be noted that the language component is left-lateralized, unlike the model-based picture naming contrast.
Findings	Other
Findings notes	Activity in the language component was greater in the tDCS condition. In the frequency domain, the tDCS condition showed reduced power in the highest frequency bin, and increased power in the lowest frequency bin.

Complex analysis 2

First level contrast	Picture naming vs rest
Analysis class	Cross-sectional aphasia vs control
Group(s)	Aphasia after sham stimulation (n = 16) vs control
Covariate	—
Is the second level contrast valid in terms of the group(s), time point(s), and measures involved?	Yes
Is accuracy matched across the second level contrast?	Unknown, not reported
Is reaction time matched across the second level contrast?	Unknown, not reported
Behavioral data notes	—
Type of analysis	Complex
Statistical details	ICA was used to derive three task-relevant components: language, motor and visual. Thresholding of the functional maps is not described, but they appear to reflect coherent components of a picture naming network. These components were compared between stimulation conditions in terms of mean activity and power in three frequency bins. It should be noted that the language component is left-lateralized, unlike the model-based picture naming contrast.
Findings	Other
Findings notes	Mean activity of these components did not differ between patients and controls. However, patients showed increased power in the middle frequency bin of the visual component.

Complex analysis 3

First level contrast	Picture naming vs rest
Analysis class	Cross-sectional aphasia vs control
Group(s)	Aphasia after tDCS (n = 16) vs control
Covariate	—
Is the second level contrast valid in terms of the group(s), time point(s), and measures involved?	Yes
Is accuracy matched across the second level contrast?	Unknown, not reported
Is reaction time matched across the second level contrast?	Unknown, not reported
Behavioral data notes	—
Type of analysis	Complex
Statistical details	ICA was used to derive three task-relevant components: language, motor and visual.

	Thresholding of the functional maps is not described, but they appear to reflect coherent components of a picture naming network. These components were compared between stimulation conditions in terms of mean activity and power in three frequency bins. It should be noted that the language component is left-lateralized, unlike the model-based picture naming contrast.
Findings	None
Findings notes	—

Notes

Excluded analyses	—

Geranmayeh et al. (2017)

Reference

Authors	Geranmayeh F, Chau TW, Wise RJS, Leech R, Hampshire A
Title	Domain-general subregions of the medial prefrontal cortex contribute to recovery of language after stroke
Reference	*Brain* 2017; 140: 1947-1958
PMID	29177494
DOI	10.1093/brain/awx134

Participants

Language	UK English
Inclusion criteria	—
Number of individuals with aphasia	27
Number of control participants	0
Were any of the participants included in any previous studies?	Yes (patients are a subset of those in Geranmayeh et al. (2016))
Is age reported for patients and controls, and matched?	Yes (mean 59.1 ± 10.8 years, range 39-77 years)
Is sex reported for patients and controls, and matched?	Yes (males: 18; females: 9)
Is handedness reported for patients and controls, and matched?	Yes (right: 26; left: 1)
Is time post stroke onset reported and appropriate to the study design?	Yes (T1: 15 ± 7.6 days (range 5-35 days); T2: 108 ± 26 days (range 87-200 days))
To what extent is the nature of aphasia characterized?	Not at all
Language evaluation	CAT, QPA
Aphasia severity	Not stated
Aphasia type	Not stated
First stroke only?	No
Stroke type	Not stated
To what extent is the lesion distribution characterized?	Lesion overlay
Lesion extent	Mean 41.4 ± 44.4 cc, range 3.8-173.9 cc
Lesion location	L; modest R involvement in 3 cases
Participants notes	24 control participants are described, but no imaging data from the controls are analyzed in this paper

Imaging

Modality	fMRI
Is the study cross-sectional or longitudinal?	Longitudinal—recovery

If longitudinal, at what time point(s) were imaging data acquired?	T1: 15 ± 7.6 days (range 5-35 days); T2: 108 ± 26 days (range 87-200 days)
If longitudinal, was there any intervention between the time points?	Variable modest amounts of SLT (range 0-18 hours) reported in Supplementary Table 1
Is the scanner described?	Yes (Siemens Trio 3 Tesla)
Is the timing of stimulus presentation and image acquisition clearly described and appropriate?	Yes
Design type	Event-related
Total images acquired	213
Are the imaging acquisition parameters, including coverage, adequately described and appropriate?	Yes (whole brain)
Is preprocessing and intrasubject coregistration adequately described and appropriate?	Yes
Is first level model fitting adequately described and appropriate?	Yes
Is intersubject normalization adequately described and appropriate?	Yes
Imaging notes	sparse sampling; mini-blocks of 2-4 trials

Conditions

Are the conditions clearly described?	Yes

Condition	Response type	Repetitions	All groups could do?	All individuals could do?
propositional speech production	Sentence (overt)	60	Yes	Yes
counting	Multiple words (overt)	48	Yes	Unknown
target decision	Button press	48	Yes	No
rest	None	45	N/A	N/A

Conditions notes	All participants could do the target decision task except for one who was at chance

Contrasts

Are the contrasts clearly described?	No (see specific limitation(s) below)

Contrast 1: propositional speech production vs rest

Language condition	Propositional speech production
Control condition	Rest
Are the conditions matched for visual demands?	No
Are the conditions matched for auditory demands?	No
Are the conditions matched for motor demands?	No
Are the conditions matched for cognitive/executive demands?	No
Is accuracy matched between the language and control tasks for all relevant groups?	N/A, tasks not comparable
Is reaction time matched between the language and control tasks for all relevant groups?	N/A, tasks not comparable
Behavioral data notes	—
Are control data reported in this paper or another that is referenced?	Yes
Does the contrast selectively activate plausible relevant language regions in the control group?	No
Are activations lateralized in the control data?	No
Control activation notes	Control data in Geranmayeh et al. (2014); speech not language; relevant activations are bilateral
Contrast notes	Not entirely clear that the whole brain analysis is indeed propositional speech production vs rest; a contrast of target decision vs mean of propositional speech and counting is also used to define the preSMA/dACC ROI

Analyses

Are the analyses clearly described?	Yes

Voxelwise analysis 1

First level contrast	Propositional speech production vs rest
Analysis class	Cross-sectional correlation with language or other measure
Group(s)	Aphasia mean of T1, T2
Covariate	Simultaneous Δ (T2 vs T1) number of appropriate information-carrying words
Is the second level contrast valid in terms of the group(s), time point(s), and measures involved?	Somewhat (potentially confounded by T1 and T2 language function; language function at T1 was predictive of change in language function)
Is accuracy matched across the second level contrast?	Appear mismatched
Is reaction time matched across the second level contrast?	Unknown, not reported
Behavioral data notes	T1 AICW correlated with change in AICW, but not stated whether T2 AICW correlated with change in AICW
Type of analysis	Voxelwise
Search volume	Voxels spared in all patients
Correction for multiple comparisons	Clusterwise correction based on arbitrary cluster extent
Software	FSL
Voxelwise p	.05
Cluster extent	1.6 cc
Statistical details	—
Findings	↑ L SMA/medial prefrontal<br>↑ L anterior cingulate<br>↑ R SMA/medial prefrontal<br>↑ R somato-motor<br>↑ R posterior STS<br>↑ R anterior cingulate
Findings notes	Findings based on figures and coordinates; the pre-SMA/dACC peak noted to survive FWE correction at $p < .001$

ROI analysis 1

First level contrast	Propositional speech production vs rest
Analysis class	Longitudinal change in aphasia
Group(s)	Aphasia T2 vs T1
Covariate	—
Is the second level contrast valid in terms of the group(s), time point(s), and measures involved?	Yes
Is accuracy matched across the second level contrast?	No, different
Is reaction time matched across the second level contrast?	Unknown, not reported
Behavioral data notes	Number of AICW increased
Type of analysis	Region of interest (ROI)
ROI type	Functional
How many ROIs are there?	1
What are the ROI(s)?	L pre-SMA
How are the ROI(s) defined?	Peak voxel of the contrast of target decision vs mean of propositional speech and counting in people with aphasia
Correction for multiple comparisons	One only
Statistical details	No main effect of session in session by language recovery ANOVA
Findings	None
Findings notes	—

ROI analysis 2

First level contrast	Propositional speech production vs rest
Analysis class	Longitudinal correlation with language or other measure
Group(s)	Aphasia T2 vs T1
Covariate	Δ number of appropriate information-carrying words
Is the second level contrast valid in terms of the group(s), time point(s), and measures involved?	Yes
Is accuracy matched across the second level contrast?	Unknown, not reported
Is reaction time matched across the second level contrast?	Unknown, not reported
Behavioral data notes	—
Type of analysis	Region of interest (ROI)
ROI type	Functional
How many ROIs are there?	1
What are the ROI(s)?	L pre-SMA
How are the ROI(s) defined?	Peak voxel of the contrast of target decision vs mean of propositional speech and counting in people with aphasia
Correction for multiple comparisons	One only
Statistical details	No interaction of session by language recovery in ANOVA
Findings	None
Findings notes	—

ROI analysis 3

First level contrast	Propositional speech production vs rest
Analysis class	Cross-sectional correlation with language or other measure
Group(s)	Aphasia mean of T1, T2
Covariate	Simultaneous Δ (T2 vs T1) number of appropriate information-carrying words
Is the second level contrast valid in terms of the group(s), time point(s), and measures involved?	Somewhat (potentially confounded by T1 and T2 language function; language function at T1 was predictive of change in language function)
Is accuracy matched across the second level contrast?	Appear mismatched
Is reaction time matched across the second level contrast?	Unknown, not reported
Behavioral data notes	T1 AICW correlated with change in AICW, but not stated whether T2 AICW correlated with change in AICW
Type of analysis	Region of interest (ROI)
ROI type	Functional
How many ROIs are there?	1
What are the ROI(s)?	L pre-SMA
How are the ROI(s) defined?	Peak voxel of the contrast of target decision vs mean of propositional speech and counting in people with aphasia
Correction for multiple comparisons	One only
Statistical details	—
Findings	↑ L SMA/medial prefrontal
Findings notes	Patients with more pre-SMA activity improved more

ROI analysis 4

First level contrast	Propositional speech production vs rest
Analysis class	Cross-sectional correlation with language or other measure
Group(s)	Aphasia mean of T1, T2
Covariate	Simultaneous Δ (T2 vs T1) number of appropriate information-carrying words
Is the second level contrast valid in terms of the group(s), time point(s), and measures involved?	Somewhat (potentially confounded by T1 and T2 language function; language function at T1 was predictive of change in language function)
Is accuracy matched across the second level contrast?	Appear mismatched
Is reaction time matched across the second level	Unknown, not reported

contrast?	
Behavioral data notes	T1 AICW correlated with change in AICW, but not stated whether T2 AICW correlated with change in AICW
Type of analysis	Region of interest (ROI)
ROI type	Functional
How many ROIs are there?	1
What are the ROI(s)?	L pre-SMA
How are the ROI(s) defined?	Peak voxel of the contrast of target decision vs mean of propositional speech and counting in people with aphasia
Correction for multiple comparisons	One only
Statistical details	Lesion size covariate
Findings	↑ L SMA/medial prefrontal
Findings notes	Patients with more pre-SMA activity improved more

ROI analysis 5

First level contrast	Propositional speech production vs rest
Analysis class	Cross-sectional correlation with language or other measure
Group(s)	Aphasia mean of T1, T2
Covariate	Simultaneous Δ (T2 vs T1) number of appropriate information-carrying words
Is the second level contrast valid in terms of the group(s), time point(s), and measures involved?	Yes (this analysis is appropriate because T1 behavior is included in model)
Is accuracy matched across the second level contrast?	Appear mismatched
Is reaction time matched across the second level contrast?	Unknown, not reported
Behavioral data notes	T1 AICW correlated with change in AICW, but not stated whether T2 AICW correlated with change in AICW
Type of analysis	Region of interest (ROI)
ROI type	Functional
How many ROIs are there?	1
What are the ROI(s)?	L pre-SMA
How are the ROI(s) defined?	Peak voxel of the contrast of target decision vs mean of propositional speech and counting in people with aphasia
Correction for multiple comparisons	One only
Statistical details	Lesion size, T1 performance, and age covariates
Findings	↑ L SMA/medial prefrontal
Findings notes	Patients with more pre-SMA activity improved more

ROI analysis 6

First level contrast	Propositional speech production vs rest
Analysis class	Cross-sectional correlation with language or other measure
Group(s)	Aphasia mean of T1, T2
Covariate	Subsequent outcome (T2) number of appropriate information-carrying words
Is the second level contrast valid in terms of the group(s), time point(s), and measures involved?	No (mathematically equivalent to the previous analysis, because of the inclusion of T1 performance as a covariate)
Is accuracy matched across the second level contrast?	Appear mismatched
Is reaction time matched across the second level contrast?	Unknown, not reported
Behavioral data notes	T1 AICW correlated with change in AICW, but not stated whether T2 AICW correlated with change in AICW
Type of analysis	Region of interest (ROI)
ROI type	Functional
How many ROIs are there?	1
What are the ROI(s)?	L pre-SMA
How are the ROI(s) defined?	Peak voxel of the contrast of target decision vs mean of propositional speech and counting in

	people with aphasia
Correction for multiple comparisons	One only
Statistical details	Lesion size, T1 performance, and age covariates
Findings	↑ L SMA/medial prefrontal
Findings notes	—

ROI analysis 7

First level contrast	Propositional speech production vs rest
Analysis class	Cross-sectional correlation with language or other measure
Group(s)	Aphasia T1
Covariate	Subsequent Δ (T2 vs T1) number of appropriate information-carrying words
Is the second level contrast valid in terms of the group(s), time point(s), and measures involved?	Somewhat (potentially confounded by T1 language function; language function at T1 was predictive of change in language function)
Is accuracy matched across the second level contrast?	No, different
Is reaction time matched across the second level contrast?	Unknown, not reported
Behavioral data notes	T1 AICW correlated with change in AICW
Type of analysis	Region of interest (ROI)
ROI type	Functional
How many ROIs are there?	1
What are the ROI(s)?	L pre-SMA
How are the ROI(s) defined?	Peak voxel of the contrast of target decision vs mean of propositional speech and counting in people with aphasia
Correction for multiple comparisons	One only
Statistical details	—
Findings	↑ L SMA/medial prefrontal
Findings notes	—

ROI analysis 8

First level contrast	Propositional speech production vs rest
Analysis class	Cross-sectional correlation with language or other measure
Group(s)	Aphasia T2
Covariate	Previous Δ (T2 vs T1) number of appropriate information-carrying words
Is the second level contrast valid in terms of the group(s), time point(s), and measures involved?	No (the logic behind correlating activation changes and language outcome is unclear)
Is accuracy matched across the second level contrast?	Appear mismatched
Is reaction time matched across the second level contrast?	Unknown, not reported
Behavioral data notes	T1 AICW correlated with change in AICW, but not stated whether T2 AICW correlated with change in AICW
Type of analysis	Region of interest (ROI)
ROI type	Functional
How many ROIs are there?	1
What are the ROI(s)?	L pre-SMA
How are the ROI(s) defined?	Peak voxel of the contrast of target decision vs mean of propositional speech and counting in people with aphasia
Correction for multiple comparisons	One only
Statistical details	—
Findings	↑ L SMA/medial prefrontal
Findings notes	—

Notes

Excluded analyses	It is mentioned that activity for other tasks did not correlate with language recovery, but no

	details are provided

Griffis, Nenert, Allendorfer, & Szaflarski (2017)

Reference

Authors	Griffis JC, Nenert R, Allendorfer JB, Szaflarski JP
Title	Linking left hemispheric tissue preservation to fMRI language task activation in chronic stroke patients
Reference	*Cortex* 2017; 96: 1-18
PMID	28961522
DOI	10.1016/j.cortex.2017.08.031

Participants

Language	US English
Inclusion criteria	—
Number of individuals with aphasia	43
Number of control participants	43
Were any of the participants included in any previous studies?	Yes (same dataset as Griffis et al. (2017) Hum Brain Mapp)
Is age reported for patients and controls, and matched?	Yes (mean 53 ± 15 years, range 23-90 years)
Is sex reported for patients and controls, and matched?	Yes (males: 25; females: 18)
Is handedness reported for patients and controls, and matched?	Yes (right: 41; left: 2)
Is time post stroke onset reported and appropriate to the study design?	Yes (range 1-14 years)
To what extent is the nature of aphasia characterized?	Not at all
Language evaluation	BNT, semantic fluency, phonemic fluency
Aphasia severity	Not stated
Aphasia type	Not stated
First stroke only?	Yes
Stroke type	Not stated
To what extent is the lesion distribution characterized?	Lesion overlay
Lesion extent	Mean 105.2 ± 76.3 cc
Lesion location	L
Participants notes	—

Imaging

Modality	fMRI
Is the study cross-sectional or longitudinal?	Cross-sectional
If longitudinal, at what time point(s) were imaging data acquired?	—
If longitudinal, was there any intervention between the time points?	—
Is the scanner described?	No (Siemens Allegra 3 Tesla or Philips 3 Tesla; model not stated)
Is the timing of stimulus presentation and image acquisition clearly described and appropriate?	Yes
Design type	Block
Total images acquired	165
Are the imaging acquisition parameters, including	Yes (whole brain)

coverage, adequately described and appropriate?	
Is preprocessing and intrasubject coregistration adequately described and appropriate?	Yes
Is first level model fitting adequately described and appropriate?	Yes
Is intersubject normalization adequately described and appropriate?	Yes
Imaging notes	—

Conditions

Are the conditions clearly described?	Yes

Condition	Response type	Repetitions	All groups could do?	All individuals could do?
semantic decision	Button press	5	No	No
tone decision	Button press	6	Unknown	Unknown

Conditions notes	Group performance below chance; several patients at 0 which is difficult to understand in a 2AFC task

Contrasts

Are the contrasts clearly described?	Yes

Contrast 1: semantic decision vs tone decision

Language condition	Semantic decision
Control condition	Tone decision
Are the conditions matched for visual demands?	Yes
Are the conditions matched for auditory demands?	Yes
Are the conditions matched for motor demands?	Yes
Are the conditions matched for cognitive/executive demands?	Yes
Is accuracy matched between the language and control tasks for all relevant groups?	Unknown, not reported
Is reaction time matched between the language and control tasks for all relevant groups?	Unknown, not reported
Behavioral data notes	Tone decision accuracy not reported
Are control data reported in this paper or another that is referenced?	Yes
Does the contrast selectively activate plausible relevant language regions in the control group?	Yes
Are activations lateralized in the control data?	Yes
Control activation notes	Temporal activation is mid MTG and AG rather than pSTS
Contrast notes	—

Analyses

Are the analyses clearly described?	Yes

ROI analysis 1

First level contrast	Semantic decision vs tone decision
Analysis class	Cross-sectional correlation with language or other measure
Group(s)	Aphasia
Covariate	Semantic decision accuracy
Is the second level contrast valid in terms of the group(s), time point(s), and measures involved?	Yes
Is accuracy matched across the second level contrast?	Accuracy is covariate
Is reaction time matched across the second level	Unknown, not reported

contrast?	
Behavioral data notes	—
Type of analysis	Regions of interest (ROI)
ROI type	Other
How many ROIs are there?	3
What are the ROI(s)?	(1) L AG and bilateral midline components of the canonical semantic network, along with reduced activity in R frontal, temporal and parietal regions; (2) bilateral IFG pars orbitalis; (3) L IFG and DLPFC along with bilateral midline regions
How are the ROI(s) defined?	ROIs are mixing coefficients of functional networks arising from mCCA + jICA that were differently represented in the patient and control groups
Correction for multiple comparisons	Familywise error (FWE)
Statistical details	—
Findings	↑ L IFG<br>↑ L dorsolateral prefrontal cortex<br>↑ L SMA/medial prefrontal<br>↑ L angular gyrus<br>↑ L precuneus<br>↑ L posterior cingulate<br>↑ R IFG pars orbitalis<br>↑ R SMA/medial prefrontal<br>↑ R precuneus<br>↑ R posterior cingulate<br>↓ L insula<br>↓ R IFG pars opercularis<br>↓ R IFG pars triangularis<br>↓ R insula<br>↓ R dorsal precentral<br>↓ R supramarginal gyrus<br>↓ R posterior STG<br>↓ R mid temporal
Findings notes	All 3 networks were significantly correlated; analysis of networks so involvement of each individual region cannot be assured

ROI analysis 2

First level contrast	Semantic decision vs tone decision
Analysis class	Cross-sectional correlation with language or other measure
Group(s)	Aphasia
Covariate	Average of semantic and phonemic fluency
Is the second level contrast valid in terms of the group(s), time point(s), and measures involved?	Yes
Is accuracy matched across the second level contrast?	Unknown, not reported
Is reaction time matched across the second level contrast?	Unknown, not reported
Behavioral data notes	—
Type of analysis	Regions of interest (ROI)
ROI type	Other
How many ROIs are there?	3
What are the ROI(s)?	(1) L AG and bilateral midline components of the canonical semantic network, along with reduced activity in R frontal, temporal and parietal regions; (2) bilateral IFG pars orbitalis; (3) L IFG and DLPFC along with bilateral midline regions
How are the ROI(s) defined?	ROIs are mixing coefficients of functional networks arising from mCCA + jICA that were differently represented in the patient and control groups
Correction for multiple comparisons	Familywise error (FWE)
Statistical details	—
Findings	↑ L IFG<br>↑ L dorsolateral prefrontal cortex<br>↑ L SMA/medial prefrontal

	↑ L angular gyrus<br>↑ L precuneus<br>↑ L posterior cingulate<br>↑ R SMA/medial prefrontal<br>↑ R precuneus<br>↑ R posterior cingulate<br>↓ L insula<br>↓ R IFG pars opercularis<br>↓ R IFG pars triangularis<br>↓ R insula<br>↓ R dorsal precentral<br>↓ R supramarginal gyrus<br>↓ R posterior STG<br>↓ R mid temporal
Findings notes	Networks 1 and 3 were significantly correlated; analysis of networks so involvement of each individual region cannot be assured

ROI analysis 3

First level contrast	Semantic decision vs tone decision
Analysis class	Cross-sectional correlation with language or other measure
Group(s)	Aphasia
Covariate	BNT
Is the second level contrast valid in terms of the group(s), time point(s), and measures involved?	Yes
Is accuracy matched across the second level contrast?	Unknown, not reported
Is reaction time matched across the second level contrast?	Unknown, not reported
Behavioral data notes	—
Type of analysis	Regions of interest (ROI)
ROI type	Other
How many ROIs are there?	3
What are the ROI(s)?	(1) L AG and bilateral midline components of the canonical semantic network, along with reduced activity in R frontal, temporal and parietal regions; (2) bilateral IFG pars orbitalis; (3) L IFG and DLPFC along with bilateral midline regions
How are the ROI(s) defined?	ROIs are mixing coefficients of functional networks arising from mCCA + jICA that were differently represented in the patient and control groups
Correction for multiple comparisons	Familywise error (FWE)
Statistical details	—
Findings	↑ L IFG<br>↑ L dorsolateral prefrontal cortex<br>↑ L SMA/medial prefrontal<br>↑ L angular gyrus<br>↑ L precuneus<br>↑ L posterior cingulate<br>↑ R SMA/medial prefrontal<br>↑ R precuneus<br>↑ R posterior cingulate<br>↓ L insula<br>↓ R IFG pars opercularis<br>↓ R IFG pars triangularis<br>↓ R insula<br>↓ R dorsal precentral<br>↓ R supramarginal gyrus<br>↓ R posterior STG<br>↓ R mid temporal
Findings notes	Networks 1 and 3 were significantly correlated; analysis of networks so involvement of each individual region cannot be assured

Complex analysis 1

First level contrast	Semantic decision vs tone decision
Analysis class	Cross-sectional aphasia vs control
Group(s)	Aphasia vs control
Covariate	—
Is the second level contrast valid in terms of the group(s), time point(s), and measures involved?	Yes
Is accuracy matched across the second level contrast?	No, different
Is reaction time matched across the second level contrast?	Unknown, not reported
Behavioral data notes	Semantic decision accuracy not matched, but tone decision accuracy not reported
Type of analysis	Complex
Statistical details	Multimodal canonical correlation analysis (mCCA) and joint ICA were used to identify 3 joint ICs (structural/functional) that were differently represented in the patient and control groups. Although there was no correction for multiple comparisons when the functional maps were thresholded, the maps for the three networks each appeared to relate to coherent parts of the semantic network.
Findings	Other
Findings notes	The first joint IC comprised preservation of tissue in L posterior temporo-parietal region, activity in the L AG and bilateral midline components of the canonical semantic network, and reduced activity in R frontal, temporal and parietal regions. The second joint IC comprised preservation of tissue in the the L basal ganglia/insula region, and activity predominantly in the IFG pars orbitalis bilaterally. The third joint IC comprised preservation of the L IFG and activity in the L IFG and DLPFC along with bilateral midline regions. The first joint IC was considered to provide more robust evidence for structure-function relationships than the other two, because it was the only one where individual structural and functional mixing coefficients remained correlated even when lesion volume was included as a covariate.

Notes

Excluded analyses	(1) group analyses that were described in a previous paper (Griffis et al., 2017, Hum Brain Mapp); (2) ancillary analysis using different numbers of components per modality; (3) ancillary analysis using lesion masks instead of brain tissue maps; (4) ancillary analysis using multivariate lesion-symptom mapping, because these analyses yielded similar results to the main analysis

Griffis, Nenert, Allendorfer, Vannest, et al. (2017)

Reference

Authors	Griffis JC, Nenert R, Allendorfer JB, Vannest J, Holland S, Dietz A, Szaflarski JP
Title	The canonical semantic network supports residual language function in chronic post-stroke aphasia
Reference	*Hum Brain Mapp* 2017; 38: 1636-1658
PMID	27981674
DOI	10.1002/hbm.23476

Participants

Language	US English
Inclusion criteria	—
Number of individuals with aphasia	43
Number of control participants	43
Were any of the participants included in any previous studies?	Yes (data were collected as part of "several separate studies")
Is age reported for patients and controls, and	Yes (mean 53 ± 15 years, range 23-90 years)

matched?	
Is sex reported for patients and controls, and matched?	Yes (males: 25; females: 18)
Is handedness reported for patients and controls, and matched?	Yes (right: 41; left: 2)
Is time post stroke onset reported and appropriate to the study design?	Yes (range 1-14 years)
To what extent is the nature of aphasia characterized?	Not at all
Language evaluation	BNT, semantic fluency, phonemic fluency
Aphasia severity	Not stated
Aphasia type	Not stated
First stroke only?	Yes
Stroke type	Not stated
To what extent is the lesion distribution characterized?	Individual lesions
Lesion extent	Mean 105.2 ± 76.3 cc
Lesion location	L
Participants notes	—

Imaging

Modality	fMRI
Is the study cross-sectional or longitudinal?	Cross-sectional
If longitudinal, at what time point(s) were imaging data acquired?	—
If longitudinal, was there any intervention between the time points?	—
Is the scanner described?	No (Siemens Allegra 3 Tesla or Philips 3 Tesla; model not stated)
Is the timing of stimulus presentation and image acquisition clearly described and appropriate?	Yes
Design type	Block
Total images acquired	165
Are the imaging acquisition parameters, including coverage, adequately described and appropriate?	Yes (whole brain)
Is preprocessing and intrasubject coregistration adequately described and appropriate?	Yes
Is first level model fitting adequately described and appropriate?	Yes
Is intersubject normalization adequately described and appropriate?	Yes
Imaging notes	—

Conditions

Are the conditions clearly described?	Yes

Condition	Response type	Repetitions	All groups could do?	All individuals could do?
semantic decision	Button press	5	No	No
tone decision	Button press	6	Unknown	Unknown

Conditions notes	Group performance below chance; several patients at 0 which is difficult to understand in a 2AFC task

Contrasts

Are the contrasts clearly described?	Yes

Contrast 1: semantic decision vs tone decision

Language condition	Semantic decision
Control condition	Tone decision
Are the conditions matched for visual demands?	Yes
Are the conditions matched for auditory demands?	Yes
Are the conditions matched for motor demands?	Yes
Are the conditions matched for cognitive/executive demands?	Yes
Is accuracy matched between the language and control tasks for all relevant groups?	Unknown, not reported
Is reaction time matched between the language and control tasks for all relevant groups?	Unknown, not reported
Behavioral data notes	Tone decision accuracy not reported
Are control data reported in this paper or another that is referenced?	Yes
Does the contrast selectively activate plausible relevant language regions in the control group?	Yes
Are activations lateralized in the control data?	Yes
Control activation notes	Temporal activation is mid MTG and AG rather than pSTS
Contrast notes	—

Analyses

Are the analyses clearly described?	Yes

Voxelwise analysis 1

First level contrast	Semantic decision vs tone decision
Analysis class	Cross-sectional correlation with language or other measure
Group(s)	Aphasia
Covariate	Semantic decision accuracy
Is the second level contrast valid in terms of the group(s), time point(s), and measures involved?	Yes
Is accuracy matched across the second level contrast?	Accuracy is covariate
Is reaction time matched across the second level contrast?	Unknown, not reported
Behavioral data notes	—
Type of analysis	Voxelwise
Search volume	Whole brain
Correction for multiple comparisons	Clusterwise correction based on cluster_threshold_beta
Software	SPM12/in-house
Voxelwise p	.01
Cluster extent	126 voxels (size not stated)
Statistical details	Lesion volume covariate
Findings	↑ L dorsolateral prefrontal cortex<br>↑ L angular gyrus<br>↑ L precuneus<br>↑ L mid temporal<br>↑ L anterior temporal<br>↑ L posterior cingulate<br>↑ L cerebellum<br>↑ L brainstem<br>↑ L hippocampus/MTL<br>↑ R IFG pars orbitalis<br>↑ R angular gyrus<br>↑ R precuneus<br>↑ R anterior temporal<br>↑ R occipital<br>↑ R brainstem

	↑ R hippocampus/MTL<br>↓ L somato-motor
Findings notes	Based on figure and table; larger activations are compelling; smaller activations are not due to lenient correction approach

Voxelwise analysis 2

First level contrast	Semantic decision vs tone decision
Analysis class	Cross-sectional correlation with language or other measure
Group(s)	Aphasia
Covariate	Average of semantic and phonemic fluency
Is the second level contrast valid in terms of the group(s), time point(s), and measures involved?	Yes
Is accuracy matched across the second level contrast?	Unknown, not reported
Is reaction time matched across the second level contrast?	Unknown, not reported
Behavioral data notes	—
Type of analysis	Voxelwise
Search volume	Whole brain
Correction for multiple comparisons	Clusterwise correction based on cluster_threshold_beta
Software	SPM12/in-house
Voxelwise p	.01
Cluster extent	126 voxels (size not stated)
Statistical details	Lesion volume covariate
Findings	↑ L IFG<br>↑ L dorsolateral prefrontal cortex<br>↑ L SMA/medial prefrontal<br>↑ L angular gyrus<br>↑ L precuneus<br>↑ L posterior STS<br>↑ L mid temporal<br>↑ L anterior temporal<br>↑ L posterior cingulate<br>↑ L brainstem<br>↑ L hippocampus/MTL<br>↑ R SMA/medial prefrontal<br>↑ R precuneus<br>↑ R anterior temporal<br>↑ R occipital<br>↑ R posterior cingulate<br>↑ R hippocampus/MTL<br>↓ R posterior STS
Findings notes	Based on figure and table; larger activations are compelling; smaller activations are not due to lenient correction approach

Voxelwise analysis 3

First level contrast	Semantic decision vs tone decision
Analysis class	Cross-sectional correlation with language or other measure
Group(s)	Aphasia
Covariate	BNT
Is the second level contrast valid in terms of the group(s), time point(s), and measures involved?	Yes
Is accuracy matched across the second level contrast?	Unknown, not reported
Is reaction time matched across the second level contrast?	Unknown, not reported
Behavioral data notes	—
Type of analysis	Voxelwise

Search volume	Whole brain
Correction for multiple comparisons	Clusterwise correction based on cluster_threshold_beta
Software	SPM12/in-house
Voxelwise p	.01
Cluster extent	126 voxels (size not stated)
Statistical details	Lesion volume covariate
Findings	↑ L IFG pars orbitalis<br>↑ L SMA/medial prefrontal<br>↑ L angular gyrus<br>↑ L precuneus<br>↑ L posterior cingulate<br>↑ L hippocampus/MTL<br>↑ R IFG pars orbitalis<br>↑ R SMA/medial prefrontal<br>↑ R precuneus<br>↑ R anterior temporal<br>↑ R posterior cingulate<br>↑ R cerebellum
Findings notes	Based on figure and table; larger activations are compelling; smaller activations are not due to lenient correction approach

Voxelwise analysis 4

First level contrast	Semantic decision vs tone decision
Analysis class	Cross-sectional correlation with language or other measure
Group(s)	Aphasia
Covariate	Lesion volume
Is the second level contrast valid in terms of the group(s), time point(s), and measures involved?	Yes
Is accuracy matched across the second level contrast?	Unknown, not reported
Is reaction time matched across the second level contrast?	Unknown, not reported
Behavioral data notes	—
Type of analysis	Voxelwise
Search volume	R hemisphere
Correction for multiple comparisons	Clusterwise correction based on cluster_threshold_beta
Software	SPM12/in-house
Voxelwise p	.01
Cluster extent	126 voxels (size not stated)
Statistical details	—
Findings	↑ R IFG pars opercularis<br>↑ R dorsolateral prefrontal cortex<br>↑ R dorsal precentral<br>↑ R SMA/medial prefrontal<br>↓ R orbitofrontal<br>↓ R anterior temporal<br>↓ R cerebellum<br>↓ R thalamus
Findings notes	Based on figure and table; larger activations are compelling; smaller activations are not due to lenient correction approach

ROI analysis 1

First level contrast	Semantic decision vs tone decision
Analysis class	Cross-sectional aphasia vs control
Group(s)	Aphasia vs control
Covariate	—
Is the second level contrast valid in terms of the	Yes

group(s), time point(s), and measures involved?	
Is accuracy matched across the second level contrast?	No, different
Is reaction time matched across the second level contrast?	Unknown, not reported
Behavioral data notes	Semantic decision accuracy not matched, but tone decision accuracy not reported
Type of analysis	Regions of interest (ROI)
ROI type	Functional
How many ROIs are there?	5
What are the ROI(s)?	(1) overall canonical semantic network (CSN); (2) L CSN; (3) R CSN; (4) mirror L CSN in R; (5) out-of-network CSN in R
How are the ROI(s) defined?	Control data
Correction for multiple comparisons	Familywise error (FWE)
Statistical details	Circular because ROI defined in one group
Findings	↓ L IFG<br>↓ L dorsolateral prefrontal cortex<br>↓ L SMA/medial prefrontal<br>↓ L angular gyrus<br>↓ L precuneus<br>↓ L mid temporal<br>↓ L anterior temporal<br>↓ L occipital<br>↓ L posterior cingulate<br>↓ L cerebellum<br>↓ R IFG<br>↓ R dorsolateral prefrontal cortex<br>↓ R SMA/medial prefrontal<br>↓ R angular gyrus<br>↓ R precuneus<br>↓ R anterior temporal<br>↓ R occipital<br>↓ R posterior cingulate<br>↓ R cerebellum
Findings notes	Results are for whole networks of regions, so individual regions cannot be assured; out-of-network R regions not listed since they were not significant in ROI 5 (only in ROI 4)

ROI analysis 2

First level contrast	Semantic decision vs tone decision
Analysis class	Cross-sectional correlation with language or other measure
Group(s)	Aphasia
Covariate	Lesion volume
Is the second level contrast valid in terms of the group(s), time point(s), and measures involved?	Yes
Is accuracy matched across the second level contrast?	Unknown, not reported
Is reaction time matched across the second level contrast?	Unknown, not reported
Behavioral data notes	—
Type of analysis	Regions of interest (ROI)
ROI type	Functional
How many ROIs are there?	5
What are the ROI(s)?	(1) overall canonical semantic network (CSN); (2) L CSN; (3) R CSN; (4) mirror L CSN in R; (5) out-of-network CSN in R
How are the ROI(s) defined?	Control data
Correction for multiple comparisons	Familywise error (FWE)
Statistical details	—
Findings	None
Findings notes	—

ROI analysis 3

First level contrast	Semantic decision vs tone decision
Analysis class	Cross-sectional correlation with language or other measure
Group(s)	Aphasia
Covariate	Semantic decision accuracy
Is the second level contrast valid in terms of the group(s), time point(s), and measures involved?	Yes
Is accuracy matched across the second level contrast?	Accuracy is covariate
Is reaction time matched across the second level contrast?	Unknown, not reported
Behavioral data notes	—
Type of analysis	Region of interest (ROI)
ROI type	Functional
How many ROIs are there?	1
What are the ROI(s)?	CSN
How are the ROI(s) defined?	Control data
Correction for multiple comparisons	One only
Statistical details	Lesion volume covariate
Findings	↑ L IFG<br>↑ L dorsolateral prefrontal cortex<br>↑ L SMA/medial prefrontal<br>↑ L angular gyrus<br>↑ L precuneus<br>↑ L mid temporal<br>↑ L anterior temporal<br>↑ L posterior cingulate<br>↑ L cerebellum<br>↑ R IFG<br>↑ R dorsolateral prefrontal cortex<br>↑ R SMA/medial prefrontal<br>↑ R angular gyrus<br>↑ R precuneus<br>↑ R anterior temporal<br>↑ R posterior cingulate<br>↑ R cerebellum
Findings notes	Correlation calculated for the whole network of regions, so correlation of individual regions cannot be assured

ROI analysis 4

First level contrast	Semantic decision vs tone decision
Analysis class	Cross-sectional correlation with language or other measure
Group(s)	Aphasia
Covariate	Average of semantic and phonemic fluency
Is the second level contrast valid in terms of the group(s), time point(s), and measures involved?	Yes
Is accuracy matched across the second level contrast?	Unknown, not reported
Is reaction time matched across the second level contrast?	Unknown, not reported
Behavioral data notes	—
Type of analysis	Region of interest (ROI)
ROI type	Functional
How many ROIs are there?	1
What are the ROI(s)?	CSN
How are the ROI(s) defined?	Control data

Correction for multiple comparisons	One only
Statistical details	Lesion volume covariate
Findings	↑ L IFG<br>↑ L dorsolateral prefrontal cortex<br>↑ L SMA/medial prefrontal<br>↑ L angular gyrus<br>↑ L precuneus<br>↑ L mid temporal<br>↑ L anterior temporal<br>↑ L posterior cingulate<br>↑ L cerebellum<br>↑ R IFG<br>↑ R dorsolateral prefrontal cortex<br>↑ R SMA/medial prefrontal<br>↑ R angular gyrus<br>↑ R precuneus<br>↑ R anterior temporal<br>↑ R posterior cingulate<br>↑ R cerebellum
Findings notes	Correlation calculated for the whole network of regions, so correlation of individual regions cannot be assured

ROI analysis 5

First level contrast	Semantic decision vs tone decision
Analysis class	Cross-sectional correlation with language or other measure
Group(s)	Aphasia
Covariate	BNT
Is the second level contrast valid in terms of the group(s), time point(s), and measures involved?	Yes
Is accuracy matched across the second level contrast?	Unknown, not reported
Is reaction time matched across the second level contrast?	Unknown, not reported
Behavioral data notes	—
Type of analysis	Region of interest (ROI)
ROI type	Functional
How many ROIs are there?	1
What are the ROI(s)?	CSN
How are the ROI(s) defined?	Control data
Correction for multiple comparisons	One only
Statistical details	Lesion volume covariate
Findings	↑ L IFG<br>↑ L dorsolateral prefrontal cortex<br>↑ L SMA/medial prefrontal<br>↑ L angular gyrus<br>↑ L precuneus<br>↑ L mid temporal<br>↑ L anterior temporal<br>↑ L posterior cingulate<br>↑ L cerebellum<br>↑ R IFG<br>↑ R dorsolateral prefrontal cortex<br>↑ R SMA/medial prefrontal<br>↑ R angular gyrus<br>↑ R precuneus<br>↑ R anterior temporal<br>↑ R posterior cingulate<br>↑ R cerebellum
Findings notes	Correlation calculated for the whole network of regions, so correlation of individual regions

	cannot be assured

Complex analysis 1

First level contrast	Semantic decision vs tone decision
Analysis class	Cross-sectional aphasia vs control
Group(s)	Aphasia vs control
Covariate	—
Is the second level contrast valid in terms of the group(s), time point(s), and measures involved?	Yes
Is accuracy matched across the second level contrast?	No, different
Is reaction time matched across the second level contrast?	Unknown, not reported
Behavioral data notes	Semantic decision accuracy not matched, but tone decision accuracy not reported
Type of analysis	Complex
Statistical details	Correlations between activation magnitudes in the L and R canonical semantic network (CSN) were compared between groups. However, this analysis is circular because the CSN ROIs were defined based on controls only.
Findings	None
Findings notes	—

Complex analysis 2

First level contrast	Semantic decision vs tone decision
Analysis class	Cross-sectional aphasia vs control
Group(s)	Aphasia vs control
Covariate	—
Is the second level contrast valid in terms of the group(s), time point(s), and measures involved?	Yes
Is accuracy matched across the second level contrast?	No, different
Is reaction time matched across the second level contrast?	Unknown, not reported
Behavioral data notes	Semantic decision accuracy not matched, but tone decision accuracy not reported
Type of analysis	Complex
Statistical details	Correlations between activation magnitudes in the L CSN and R mirrored CSN were compared between groups. However, this analysis is circular because the CSN ROIs were defined based on controls only.
Findings	Other
Findings notes	Correlations between activations in the L CSN and the mirrored L CSN in the R hemisphere were stronger in patients than controls.

Complex analysis 3

First level contrast	Semantic decision vs tone decision
Analysis class	Cross-sectional aphasia vs control
Group(s)	Aphasia vs control
Covariate	—
Is the second level contrast valid in terms of the group(s), time point(s), and measures involved?	Yes
Is accuracy matched across the second level contrast?	No, different
Is reaction time matched across the second level contrast?	Unknown, not reported
Behavioral data notes	Semantic decision accuracy not matched, but tone decision accuracy not reported
Type of analysis	Complex
Statistical details	Correlations between activation magnitudes in the L CSN and R out-of-network homotopic regions were compared between groups. However, this analysis is circular because the CSN

	ROIs were defined based on controls only.
Findings	Other
Findings notes	Correlations between activations in the L CSN and R out-of-network homotopic regions were stronger in patients than controls.

Complex analysis 4

First level contrast	Semantic decision vs tone decision
Analysis class	Cross-sectional aphasia vs control
Group(s)	Aphasia vs control
Covariate	—
Is the second level contrast valid in terms of the group(s), time point(s), and measures involved?	Yes
Is accuracy matched across the second level contrast?	No, different
Is reaction time matched across the second level contrast?	Unknown, not reported
Behavioral data notes	Semantic decision accuracy not matched, but tone decision accuracy not reported
Type of analysis	Complex
Statistical details	The difference in activation between the L CSN and R CSN was compared between patients and controls. However, this analysis is circular because the CSN ROIs were defined based on controls only.
Findings	None
Findings notes	—

Complex analysis 5

First level contrast	Semantic decision vs tone decision
Analysis class	Cross-sectional aphasia vs control
Group(s)	Aphasia vs control
Covariate	—
Is the second level contrast valid in terms of the group(s), time point(s), and measures involved?	Yes
Is accuracy matched across the second level contrast?	No, different
Is reaction time matched across the second level contrast?	Unknown, not reported
Behavioral data notes	Semantic decision accuracy not matched, but tone decision accuracy not reported
Type of analysis	Complex
Statistical details	The difference in activation between the L CSN and mirror L CSN in the R was compared between patients and controls. However, this analysis is circular because the CSN ROIs were defined based on controls only.
Findings	Other
Findings notes	The difference was smaller in patients.

Complex analysis 6

First level contrast	Semantic decision vs tone decision
Analysis class	Cross-sectional aphasia vs control
Group(s)	Aphasia vs control
Covariate	—
Is the second level contrast valid in terms of the group(s), time point(s), and measures involved?	Yes
Is accuracy matched across the second level contrast?	No, different
Is reaction time matched across the second level contrast?	Unknown, not reported
Behavioral data notes	Semantic decision accuracy not matched, but tone decision accuracy not reported
Type of analysis	Complex

Statistical details	The difference in activation between the R CSN and out-of-network homotopic regions in the R was compared between patients and controls. However, this analysis is circular because the CSN ROIs were defined based on controls only.
Findings	Other
Findings notes	The difference was smaller in patients.

Complex analysis 7

First level contrast	Semantic decision vs tone decision
Analysis class	Cross-sectional correlation with language or other measure
Group(s)	Aphasia
Covariate	Interactions of semantic fluency and naming measures by lesion size
Is the second level contrast valid in terms of the group(s), time point(s), and measures involved?	Yes
Is accuracy matched across the second level contrast?	Unknown, not reported
Is reaction time matched across the second level contrast?	Unknown, not reported
Behavioral data notes	—
Type of analysis	Complex
Statistical details	For the 4 R hemisphere regions that were more activated in patients with larger lesions (SPM analysis 4), analyses were carried out to determine whether the semantic fluency or naming measures were differentially impacted by activation depending on whether lesions were larger or smaller.
Findings	Other
Findings notes	For 1 of the 4 regions (R SMA), there were significant interactions such that in patients with larger lesions, more activation was associated with higher semantic fluency scores and higher BNT scores, while in patients with smaller lesions, more activation was associated with lower fluency and BNT scores. There was a similar relationship with semantic fluency in the R IFG pars opercularis but only at p(FDR) = 0.07.

Notes

Excluded analyses	Ancillary whole brain analyses without lesion volume covariate (Supporting Figure 3); Figure 3b and 3c, which are derivatives of included analyses

Harvey et al. (2017)

Reference

Authors	Harvey DY, Podell J, Turkeltaub PE, Faseyitan O, Coslett HB, Hamilton RH
Title	Functional reorganization of right prefrontal cortex underlies sustained naming improvements in chronic aphasia via repetitive transcranial magnetic stimulation
Reference	*Cogn Behav Neurol* 2017; 30: 133-144
PMID	29256908
DOI	10.1097/wnn.0000000000000141

Participants

Language	US English
Inclusion criteria	Mild-moderate non-fluent aphasia; relatively intact comprehension; able to produce meaningful words and phrases
Number of individuals with aphasia	6
Number of control participants	0
Were any of the participants included in any previous studies?	No
Is age reported for patients and controls, and matched?	Yes (range 47-75 years)

Is sex reported for patients and controls, and matched?	Yes (males: 5; females: 1)
Is handedness reported for patients and controls, and matched?	Yes (right: 6; left: 0)
Is time post stroke onset reported and appropriate to the study design?	Yes (range 6-102 months)
To what extent is the nature of aphasia characterized?	Comprehensive battery
Language evaluation	BDAE, BNT
Aphasia severity	Mild-moderate
Aphasia type	All non-fluent
First stroke only?	Yes
Stroke type	Ischemic only
To what extent is the lesion distribution characterized?	Individual lesions
Lesion extent	Range 36.6-252.1 cc
Lesion location	L MCA
Participants notes	—

Imaging

Modality	fMRI
Is the study cross-sectional or longitudinal?	Longitudinal—chronic treatment
If longitudinal, at what time point(s) were imaging data acquired?	T1: pre-treatment/chronic; T2: post-treatment, 2 months after treatment; T3: 6 months after treatment (the 2-month time point was not included in analysis because there was no significant behavioral effect at that time)
If longitudinal, was there any intervention between the time points?	Inhibitory rTMS to R IFG, 10 days
Is the scanner described?	Yes (Siemens Trio 3 Tesla)
Is the timing of stimulus presentation and image acquisition clearly described and appropriate?	Yes
Design type	Block
Total images acquired	200
Are the imaging acquisition parameters, including coverage, adequately described and appropriate?	Yes (whole brain)
Is preprocessing and intrasubject coregistration adequately described and appropriate?	Yes
Is first level model fitting adequately described and appropriate?	Yes
Is intersubject normalization adequately described and appropriate?	No (lesion impact not addressed)
Imaging notes	—

Conditions

Are the conditions clearly described?	Yes

Condition	Response type	Repetitions	All groups could do?	All individuals could do?
picture naming	Word (overt)	20	Yes	Yes
viewing patterns	None	20	N/A	N/A

Conditions notes	Assume all individuals could do based on inclusion criterion and BNT scores

Contrasts

Are the contrasts clearly described?	Yes

Contrast 1: picture naming vs viewing patterns

Language condition	Picture naming

Control condition	Viewing patterns
Are the conditions matched for visual demands?	Yes
Are the conditions matched for auditory demands?	No
Are the conditions matched for motor demands?	No
Are the conditions matched for cognitive/executive demands?	No
Is accuracy matched between the language and control tasks for all relevant groups?	N/A, tasks not comparable
Is reaction time matched between the language and control tasks for all relevant groups?	N/A, tasks not comparable
Behavioral data notes	—
Are control data reported in this paper or another that is referenced?	No
Does the contrast selectively activate plausible relevant language regions in the control group?	Unknown
Are activations lateralized in the control data?	Unknown
Control activation notes	—
Contrast notes	—

Analyses

Are the analyses clearly described?	Yes

Voxelwise analysis 1

First level contrast	Picture naming vs viewing patterns
Analysis class	Longitudinal change in aphasia
Group(s)	Aphasia T3 vs T1
Covariate	—
Is the second level contrast valid in terms of the group(s), time point(s), and measures involved?	Yes
Is accuracy matched across the second level contrast?	Unknown, not reported
Is reaction time matched across the second level contrast?	Unknown, not reported
Behavioral data notes	—
Type of analysis	Voxelwise
Search volume	Voxels spared in all patients
Correction for multiple comparisons	No direct comparison
Software	SPM8
Voxelwise p	—
Cluster extent	—
Statistical details	Qualitative comparison on pp. 138-9
Findings	↑ L SMA/medial prefrontal<br>↑ L posterior inferior temporal gyrus/fusiform gyrus<br>↑ L occipital<br>↑ L anterior cingulate<br>↑ R IFG pars opercularis<br>↑ R ventral precentral/inferior frontal junction<br>↓ L dorsolateral prefrontal cortex<br>↓ R IFG pars triangularis<br>↓ R posterior inferior temporal gyrus/fusiform gyrus<br>↓ R occipital<br>↓ R hippocampus/MTL
Findings notes	Based on Figure 5 and Table 4

Notes

Excluded analyses	—

Nardo et al. (2017)

Reference

Authors	Nardo D, Holland R, Leff AP, Price CJ, Crinion JT
Title	Less is more: neural mechanisms underlying anomia treatment in chronic aphasic patients
Reference	*Brain* 2017; 140: 3039-3054
PMID	29053773
DOI	10.1093/brain/awx234

Participants

Language	UK English
Inclusion criteria	Anomia; good single word comprehension; relatively spared word and nonword repetition; no AoS; spared or partially spared L IFG
Number of individuals with aphasia	18
Number of control participants	0
Were any of the participants included in any previous studies?	No
Is age reported for patients and controls, and matched?	Yes (mean 50 ± 12 years, range 21-67 years)
Is sex reported for patients and controls, and matched?	Yes (males: 12; females: 6)
Is handedness reported for patients and controls, and matched?	Yes (right: 18; left: 0)
Is time post stroke onset reported and appropriate to the study design?	Yes (mean 61 ± 58 months, range 5-264 months)
To what extent is the nature of aphasia characterized?	Not at all
Language evaluation	BNT, one CAT subtest, two PALPA subtests
Aphasia severity	Not stated
Aphasia type	Not stated
First stroke only?	Yes
Stroke type	Not stated
To what extent is the lesion distribution characterized?	Lesion overlay
Lesion extent	Not stated
Lesion location	L MCA
Participants notes	—

Imaging

Modality	fMRI
Is the study cross-sectional or longitudinal?	Longitudinal—chronic treatment
If longitudinal, at what time point(s) were imaging data acquired?	T1: pre-treatment/chronic; T2: post-treatment, ~6 weeks later
If longitudinal, was there any intervention between the time points?	Anomia treatment (computer-based practice), 2+ hours/day, 6 weeks
Is the scanner described?	Yes (Siemens Trio 3 Tesla)
Is the timing of stimulus presentation and image acquisition clearly described and appropriate?	Yes
Design type	Event-related
Total images acquired	696
Are the imaging acquisition parameters, including coverage, adequately described and appropriate?	Yes (whole brain)
Is preprocessing and intrasubject coregistration	Yes

adequately described and appropriate?	
Is first level model fitting adequately described and appropriate?	Yes
Is intersubject normalization adequately described and appropriate?	Yes
Imaging notes	—

Conditions

Are the conditions clearly described?	Yes

Condition	Response type	Repetitions	All groups could do?	All individuals could do?
picture naming (untrained items, word cue)	Word (overt)	54	Yes	Unknown
picture naming (untrained items, initial phonemes cue)	Word (overt)	54	Yes	Unknown
picture naming (untrained items, final phonemes cue)	Word (overt)	54	Yes	Unknown
picture naming (untrained items, no cue)	Word (overt)	54	Yes	Unknown
picture naming (trained items, word cue)	Word (overt)	53	Yes	Unknown
picture naming (trained items, initial phonemes cue)	Word (overt)	53	Yes	Unknown
picture naming (trained items, final phonemes cue)	Word (overt)	53	Yes	Unknown
picture naming (trained items, no cue)	Word (overt)	53	Yes	Unknown
rest	None	implicit baseline	N/A	N/A

Conditions notes	Spectrally rotated noise vocoded auditory stimulus in no-cue conditions; one patient had a BNT of 1/60 so it is unclear whether that patient could do the task

Contrasts

Are the contrasts clearly described?	No (see specific limitation(s) below)

Contrast 1: picture naming (all conditions, correct trials) vs rest

Language condition	Picture naming (all conditions, correct trials)
Control condition	Rest
Are the conditions matched for visual demands?	No
Are the conditions matched for auditory demands?	No
Are the conditions matched for motor demands?	No
Are the conditions matched for cognitive/executive demands?	No
Is accuracy matched between the language and control tasks for all relevant groups?	N/A, tasks not comparable
Is reaction time matched between the language and control tasks for all relevant groups?	N/A, tasks not comparable
Behavioral data notes	—
Are control data reported in this paper or another that is referenced?	No
Does the contrast selectively activate plausible relevant language regions in the control group?	Unknown
Are activations lateralized in the control data?	Unknown
Control activation notes	—
Contrast notes	It is difficult to determine exactly what contrasts were employed

Contrast 2: picture naming (untrained items, no cue, correct trials) vs picture naming (trained items, no cue, correct trials)

Language condition	Picture naming (untrained items, no cue, correct trials)
Control condition	Picture naming (trained items, no cue, correct trials)

Are the conditions matched for visual demands?	Yes
Are the conditions matched for auditory demands?	Yes
Are the conditions matched for motor demands?	Yes
Are the conditions matched for cognitive/executive demands?	Yes
Is accuracy matched between the language and control tasks for all relevant groups?	Yes, correct trials only
Is reaction time matched between the language and control tasks for all relevant groups?	No, different
Behavioral data notes	Untrained items significantly slower at T2
Are control data reported in this paper or another that is referenced?	No
Does the contrast selectively activate plausible relevant language regions in the control group?	Unknown
Are activations lateralized in the control data?	Unknown
Control activation notes	—
Contrast notes	It is difficult to determine exactly what contrasts were employed

Analyses

Are the analyses clearly described?	No* (moderate limitation) (see specific limitation(s) below)

Voxelwise analysis 1

First level contrast	Picture naming (all conditions, correct trials) vs rest
Analysis class	Longitudinal change in aphasia
Group(s)	Aphasia T2 vs T1
Covariate	—
Is the second level contrast valid in terms of the group(s), time point(s), and measures involved?	Yes
Is accuracy matched across the second level contrast?	Yes, correct trials only
Is reaction time matched across the second level contrast?	No, different
Behavioral data notes	RT faster at T2
Type of analysis	Voxelwise
Search volume	Whole brain
Correction for multiple comparisons	Voxelwise FWE correction
Software	SPM12
Voxelwise p	FWE $p < .05$
Cluster extent	—
Statistical details	—
Findings	None
Findings notes	—

ROI analysis 1

First level contrast	Picture naming (untrained items, no cue, correct trials) vs picture naming (trained items, no cue, correct trials)
Analysis class	Cross-sectional correlation with language or other measure
Group(s)	Aphasia T2
Covariate	"a change in un-cued naming RT" (exact measure unclear)
Is the second level contrast valid in terms of the group(s), time point(s), and measures involved?	Somewhat (unclear whether behavioral measure is longitudinal)
Is accuracy matched across the second level contrast?	Yes, correct trials only
Is reaction time matched across the second level contrast?	Unknown, not reported
Behavioral data notes	—

Type of analysis	Regions of interest (ROI)
ROI type	Functional
How many ROIs are there?	4
What are the ROI(s)?	(1) R anterior insula; (2) R IFG; (3) dorsal anterior cingulate; (4) L premotor cortex
How are the ROI(s) defined?	Peaks (only with SVC) for the main effect of untrained (4 conditions) vs trained (4 conditions) in T2 aphasia
Correction for multiple comparisons	No correction
Statistical details	Unclear what the behavioral measure was exactly
Findings	↑ R IFG pars opercularis<br>↑ R insula
Findings notes	—

Notes

Excluded analyses	Most analyses were between conditions in people with aphasia, so did not meet criteria for this review

Nenert et al. (2017)

Reference

Authors	Nenert R, Allendorfer JB, Martin AM, Banks C, Ball A, Vannest J, Dietz AR, Szaflarski JP
Title	Neuroimaging correlates of post-stroke aphasia rehabilitation in a pilot randomized trial of constraint-induced aphasia therapy
Reference	*Med Sci Monit* 2017; 23: 3489-3507
PMID	28719572
DOI	10.12659/msm.902301

Participants

Language	US English
Inclusion criteria	At least mild aphasia per TT
Number of individuals with aphasia	19
Number of control participants	38
Were any of the participants included in any previous studies?	Yes (patients are a subset of the 24 participants in Szaflarski et al. (2015), a clinical trial on CIAT)
Is age reported for patients and controls, and matched?	Yes (CIAT group: mean 58.0 ± 10.6 years; untreated group: mean 50.3 ± 13.3 years)
Is sex reported for patients and controls, and matched?	Yes (males: 11; females: 8)
Is handedness reported for patients and controls, and matched?	No (right: 17; left: 0; other: 2; 2 patients "atypical": unclear whether L or mixed)
Is time post stroke onset reported and appropriate to the study design?	Yes (CIAT group: mean 60.2 ± 48.9 months; untreated group: mean 41.9 ± 30.0 months; all > 1 year)
To what extent is the nature of aphasia characterized?	Severity only
Language evaluation	TT, PPVT, BNT, semantic fluency, phonemic fluency, communicative activities log
Aphasia severity	6 mild (2 control, 4 CIAT); 5 moderate (3 control, 2 CIAT); 8 severe (3 control, 5 CIAT)
Aphasia type	Not stated
First stroke only?	Yes
Stroke type	Ischemic only
To what extent is the lesion distribution characterized?	Lesion overlay
Lesion extent	Not stated
Lesion location	L MCA
Participants notes	—

Imaging

Modality	fMRI
Is the study cross-sectional or longitudinal?	Longitudinal—chronic treatment
If longitudinal, at what time point(s) were imaging data acquired?	T1: pre-treatment/chronic; T2: post-treatment, ~3 weeks later; T3: 3 months after the end of treatment
If longitudinal, was there any intervention between the time points?	CIAT, 4 hours/day, 5 days/week, 2 weeks
Is the scanner described?	No (Philips 3 Tesla or Siemens 3 Tesla; models not stated)
Is the timing of stimulus presentation and image acquisition clearly described and appropriate?	Yes
Design type	Block
Total images acquired	600
Are the imaging acquisition parameters, including coverage, adequately described and appropriate?	Yes (whole brain)
Is preprocessing and intrasubject coregistration adequately described and appropriate?	Yes
Is first level model fitting adequately described and appropriate?	Yes
Is intersubject normalization adequately described and appropriate?	No (lesion impact not addressed)
Imaging notes	—

Conditions

Are the conditions clearly described?	Yes

Condition	Response type	Repetitions	All groups could do?	All individuals could do?
semantic decision	Button press	10	Unknown	Unknown
tone decision	Button press	10	Unknown	Unknown
verb generation	Multiple words (covert)	10	Unknown	Unknown
finger tapping	Other	10	Unknown	Unknown

Conditions notes	Behavioral data are provided for the semantic decision and tone decision tasks, but the denominator is unclear; a post-scan recognition test for verb generation is reported, but this cannot confirm verb generation performance

Contrasts

Are the contrasts clearly described?	Yes

Contrast 1: semantic decision vs tone decision

Language condition	Semantic decision
Control condition	Tone decision
Are the conditions matched for visual demands?	Yes
Are the conditions matched for auditory demands?	Yes
Are the conditions matched for motor demands?	Yes
Are the conditions matched for cognitive/executive demands?	Yes
Is accuracy matched between the language and control tasks for all relevant groups?	Appear mismatched
Is reaction time matched between the language and control tasks for all relevant groups?	Unknown, not reported
Behavioral data notes	Appear mismatched at least in healthy controls in Table 3
Are control data reported in this paper or another that is referenced?	Yes
Does the contrast selectively activate plausible relevant language regions in the control group?	Yes
Are activations lateralized in the control data?	Yes

Control activation notes	Lateralized frontal, temporal, and parietal
Contrast notes	—

Contrast 2: verb generation vs finger tapping

Language condition	Verb generation
Control condition	Finger tapping
Are the conditions matched for visual demands?	Yes
Are the conditions matched for auditory demands?	Yes
Are the conditions matched for motor demands?	No
Are the conditions matched for cognitive/executive demands?	No
Is accuracy matched between the language and control tasks for all relevant groups?	N/A, tasks not comparable
Is reaction time matched between the language and control tasks for all relevant groups?	N/A, tasks not comparable
Behavioral data notes	—
Are control data reported in this paper or another that is referenced?	Yes
Does the contrast selectively activate plausible relevant language regions in the control group?	Yes
Are activations lateralized in the control data?	Somewhat
Control activation notes	Control data in Szaflarski et al. (2008); frontal activation L-lateralized, temporal less so
Contrast notes	—

Analyses

Are the analyses clearly described?	Yes

Voxelwise analysis 1

First level contrast	Semantic decision vs tone decision
Analysis class	Cross-sectional between two groups with aphasia
Group(s)	Aphasia CIAT T2 (n = 11) vs untreated T2 (n = 8)
Covariate	—
Is the second level contrast valid in terms of the group(s), time point(s), and measures involved?	Somewhat (no treatment effect)
Is accuracy matched across the second level contrast?	Appear similar
Is reaction time matched across the second level contrast?	Unknown, not reported
Behavioral data notes	—
Type of analysis	Voxelwise
Search volume	Voxels spared in all patients
Correction for multiple comparisons	Clusterwise correction based on arbitrary cluster extent
Software	SPM12
Voxelwise p	.01
Cluster extent	50 voxels (size not stated)
Statistical details	—
Findings	↑ L somato-motor<br>↑ L superior parietal<br>↑ L brainstem<br>↑ R ventral precentral/inferior frontal junction<br>↑ R somato-motor<br>↑ R superior parietal
Findings notes	Based on coordinates in Table 4

Voxelwise analysis 2

First level contrast	Semantic decision vs tone decision

Analysis class	Cross-sectional between two groups with aphasia
Group(s)	Aphasia CIAT T3 (n = 11) vs untreated T3 (n = 8)
Covariate	—
Is the second level contrast valid in terms of the group(s), time point(s), and measures involved?	Somewhat (no treatment effect)
Is accuracy matched across the second level contrast?	Unknown, no test
Is reaction time matched across the second level contrast?	Unknown, not reported
Behavioral data notes	—
Type of analysis	Voxelwise
Search volume	Voxels spared in all patients
Correction for multiple comparisons	Clusterwise correction based on arbitrary cluster extent
Software	SPM12
Voxelwise p	.01
Cluster extent	50 voxels (size not stated)
Statistical details	—
Findings	↑ L superior parietal<br>↑ L anterior temporal<br>↑ L hippocampus/MTL<br>↑ R orbitofrontal<br>↓ L dorsolateral prefrontal cortex<br>↓ L posterior inferior temporal gyrus/fusiform gyrus<br>↓ R IFG pars orbitalis<br>↓ R ventral precentral/inferior frontal junction<br>↓ R posterior STS
Findings notes	Based on coordinates in Table 4

Voxelwise analysis 3

First level contrast	Verb generation vs finger tapping
Analysis class	Cross-sectional between two groups with aphasia
Group(s)	Aphasia CIAT T2 (n = 11) vs untreated T2 (n = 8)
Covariate	—
Is the second level contrast valid in terms of the group(s), time point(s), and measures involved?	Somewhat (no treatment effect)
Is accuracy matched across the second level contrast?	Unknown, not reported
Is reaction time matched across the second level contrast?	Unknown, not reported
Behavioral data notes	—
Type of analysis	Voxelwise
Search volume	Voxels spared in all patients
Correction for multiple comparisons	Clusterwise correction based on arbitrary cluster extent
Software	SPM12
Voxelwise p	.01
Cluster extent	50 voxels (size not stated)
Statistical details	—
Findings	↓ L precuneus<br>↓ R dorsolateral prefrontal cortex<br>↓ R posterior STS<br>↓ R anterior temporal<br>↓ R posterior inferior temporal gyrus/fusiform gyrus
Findings notes	Based on coordinates in Table 4

Voxelwise analysis 4

First level contrast	Verb generation vs finger tapping

Analysis class	Cross-sectional between two groups with aphasia
Group(s)	Aphasia CIAT T3 (n = 11) vs untreated T3 (n = 8)
Covariate	—
Is the second level contrast valid in terms of the group(s), time point(s), and measures involved?	Somewhat (no treatment effect)
Is accuracy matched across the second level contrast?	Unknown, not reported
Is reaction time matched across the second level contrast?	Unknown, not reported
Behavioral data notes	—
Type of analysis	Voxelwise
Search volume	Voxels spared in all patients
Correction for multiple comparisons	Clusterwise correction based on arbitrary cluster extent
Software	SPM12
Voxelwise p	.01
Cluster extent	50 voxels (size not stated)
Statistical details	—
Findings	↑ L SMA/medial prefrontal<br>↑ R basal ganglia<br>↓ L anterior temporal<br>↓ R posterior STS<br>↓ R Heschl's gyrus<br>↓ R posterior inferior temporal gyrus/fusiform gyrus
Findings notes	—

Voxelwise analysis 5

First level contrast	Semantic decision vs tone decision
Analysis class	Cross-sectional aphasia vs control
Group(s)	Aphasia CIAT T1 (n = 11) vs control
Covariate	—
Is the second level contrast valid in terms of the group(s), time point(s), and measures involved?	Yes
Is accuracy matched across the second level contrast?	Appear mismatched
Is reaction time matched across the second level contrast?	Unknown, not reported
Behavioral data notes	Patients less accurate than controls on both tasks, but more so on the tone decision task
Type of analysis	Voxelwise
Search volume	Voxels spared in all patients
Correction for multiple comparisons	Clusterwise correction based on arbitrary cluster extent
Software	SPM12
Voxelwise p	.01
Cluster extent	50 voxels (size not stated)
Statistical details	—
Findings	↑ L orbitofrontal<br>↑ L hippocampus/MTL<br>↑ R IFG pars opercularis<br>↑ R SMA/medial prefrontal<br>↑ R supramarginal gyrus<br>↑ R posterior STG/STS/MTG<br>↑ R anterior temporal<br>↑ R anterior cingulate<br>↓ R dorsolateral prefrontal cortex
Findings notes	—

Voxelwise analysis 6

First level contrast	Semantic decision vs tone decision

Analysis class	Cross-sectional aphasia vs control
Group(s)	Aphasia CIAT T2 (n = 11) vs control
Covariate	—
Is the second level contrast valid in terms of the group(s), time point(s), and measures involved?	Yes
Is accuracy matched across the second level contrast?	Appear mismatched
Is reaction time matched across the second level contrast?	Unknown, not reported
Behavioral data notes	Patients less accurate than controls on both tasks, but more so on the tone decision task
Type of analysis	Voxelwise
Search volume	Voxels spared in all patients
Correction for multiple comparisons	Clusterwise correction based on arbitrary cluster extent
Software	SPM12
Voxelwise p	.01
Cluster extent	50 voxels (size not stated)
Statistical details	—
Findings	↑ L anterior cingulate<br>↑ R IFG pars opercularis<br>↑ R insula<br>↑ R ventral precentral/inferior frontal junction<br>↑ R supramarginal gyrus<br>↑ R Heschl's gyrus<br>↓ L dorsolateral prefrontal cortex<br>↓ L SMA/medial prefrontal<br>↓ L cerebellum<br>↓ R dorsolateral prefrontal cortex
Findings notes	—

Voxelwise analysis 7

First level contrast	Semantic decision vs tone decision
Analysis class	Cross-sectional aphasia vs control
Group(s)	Aphasia CIAT T3 (n = 11) vs control
Covariate	—
Is the second level contrast valid in terms of the group(s), time point(s), and measures involved?	Yes
Is accuracy matched across the second level contrast?	Appear mismatched
Is reaction time matched across the second level contrast?	Unknown, not reported
Behavioral data notes	Patients less accurate than controls on both tasks, but more so on the tone decision task
Type of analysis	Voxelwise
Search volume	Voxels spared in all patients
Correction for multiple comparisons	Clusterwise correction based on arbitrary cluster extent
Software	SPM12
Voxelwise p	.01
Cluster extent	50 voxels (size not stated)
Statistical details	—
Findings	↑ L orbitofrontal<br>↑ L anterior cingulate<br>↑ L hippocampus/MTL<br>↑ R superior parietal<br>↓ L cerebellum<br>↓ R dorsolateral prefrontal cortex<br>↓ R anterior temporal<br>↓ R cerebellum
Findings notes	—

Voxelwise analysis 8

First level contrast	Semantic decision vs tone decision
Analysis class	Cross-sectional aphasia vs control
Group(s)	Aphasia untreated T1 (n = 8) vs control
Covariate	—
Is the second level contrast valid in terms of the group(s), time point(s), and measures involved?	Yes
Is accuracy matched across the second level contrast?	Appear mismatched
Is reaction time matched across the second level contrast?	Unknown, not reported
Behavioral data notes	Patients less accurate than controls on both tasks, but more so on the tone decision task
Type of analysis	Voxelwise
Search volume	Voxels spared in all patients
Correction for multiple comparisons	Clusterwise correction based on arbitrary cluster extent
Software	SPM12
Voxelwise p	.01
Cluster extent	50 voxels (size not stated)
Statistical details	—
Findings	↑ L dorsolateral prefrontal cortex<br>↑ R dorsolateral prefrontal cortex<br>↑ R SMA/medial prefrontal<br>↑ R somato-motor<br>↓ L IFG pars orbitalis<br>↓ L dorsolateral prefrontal cortex<br>↓ L SMA/medial prefrontal<br>↓ L angular gyrus<br>↓ L mid temporal<br>↓ L anterior temporal<br>↓ R IFG pars orbitalis<br>↓ R angular gyrus<br>↓ R anterior temporal<br>↓ R posterior inferior temporal gyrus/fusiform gyrus
Findings notes	—

Voxelwise analysis 9

First level contrast	Semantic decision vs tone decision
Analysis class	Cross-sectional aphasia vs control
Group(s)	Aphasia untreated T2 (n = 8) vs control
Covariate	—
Is the second level contrast valid in terms of the group(s), time point(s), and measures involved?	Yes
Is accuracy matched across the second level contrast?	Appear mismatched
Is reaction time matched across the second level contrast?	Unknown, not reported
Behavioral data notes	Patients less accurate than controls on both tasks, but more so on the tone decision task
Type of analysis	Voxelwise
Search volume	Voxels spared in all patients
Correction for multiple comparisons	Clusterwise correction based on arbitrary cluster extent
Software	SPM12
Voxelwise p	.01
Cluster extent	50 voxels (size not stated)
Statistical details	—
Findings	↑ L posterior inferior temporal gyrus/fusiform gyrus<br>↑ R dorsolateral prefrontal cortex<br>↑ R orbitofrontal

	↑ R mid temporal<br>↓ L IFG pars orbitalis<br>↓ L SMA/medial prefrontal<br>↓ L orbitofrontal<br>↓ L intraparietal sulcus<br>↓ L superior parietal<br>↓ L anterior cingulate<br>↓ L brainstem<br>↓ R IFG pars orbitalis<br>↓ R dorsolateral prefrontal cortex<br>↓ R inferior parietal lobule<br>↓ R supramarginal gyrus<br>↓ R anterior temporal<br>↓ R posterior inferior temporal gyrus/fusiform gyrus<br>↓ R hippocampus/MTL
Findings notes	—

Voxelwise analysis 10

First level contrast	Semantic decision vs tone decision
Analysis class	Cross-sectional aphasia vs control
Group(s)	Aphasia untreated T3 (n = 8) vs control
Covariate	—
Is the second level contrast valid in terms of the group(s), time point(s), and measures involved?	Yes
Is accuracy matched across the second level contrast?	Appear mismatched
Is reaction time matched across the second level contrast?	Unknown, not reported
Behavioral data notes	Patients less accurate than controls on both tasks, but not significantly for the semantic decision task, and more so on the tone decision task
Type of analysis	Voxelwise
Search volume	Voxels spared in all patients
Correction for multiple comparisons	Clusterwise correction based on arbitrary cluster extent
Software	SPM12
Voxelwise p	.01
Cluster extent	50 voxels (size not stated)
Statistical details	—
Findings	↑ L dorsolateral prefrontal cortex<br>↑ R dorsolateral prefrontal cortex<br>↑ R SMA/medial prefrontal<br>↑ R orbitofrontal<br>↑ R superior parietal<br>↑ R cerebellum<br>↓ L orbitofrontal<br>↓ L mid temporal<br>↓ L anterior temporal<br>↓ L posterior cingulate<br>↓ L cerebellum<br>↓ L hippocampus/MTL<br>↓ R angular gyrus<br>↓ R anterior temporal
Findings notes	—

Voxelwise analysis 11

First level contrast	Verb generation vs finger tapping
Analysis class	Cross-sectional aphasia vs control
Group(s)	Aphasia CIAT T1 (n = 11) vs control
Covariate	—

Is the second level contrast valid in terms of the group(s), time point(s), and measures involved?	Yes
Is accuracy matched across the second level contrast?	Unknown, not reported
Is reaction time matched across the second level contrast?	Unknown, not reported
Behavioral data notes	—
Type of analysis	Voxelwise
Search volume	Voxels spared in all patients
Correction for multiple comparisons	Clusterwise correction based on arbitrary cluster extent
Software	SPM12
Voxelwise p	.01
Cluster extent	50 voxels (size not stated)
Statistical details	—
Findings	↑ L dorsal precentral<br>↑ L superior parietal<br>↑ R cerebellum<br>↓ L dorsolateral prefrontal cortex<br>↓ L SMA/medial prefrontal<br>↓ R posterior inferior temporal gyrus/fusiform gyrus
Findings notes	—

Voxelwise analysis 12

First level contrast	Verb generation vs finger tapping
Analysis class	Cross-sectional aphasia vs control
Group(s)	Aphasia CIAT T2 (n = 11) vs control
Covariate	—
Is the second level contrast valid in terms of the group(s), time point(s), and measures involved?	Yes
Is accuracy matched across the second level contrast?	Unknown, not reported
Is reaction time matched across the second level contrast?	Unknown, not reported
Behavioral data notes	—
Type of analysis	Voxelwise
Search volume	Voxels spared in all patients
Correction for multiple comparisons	Clusterwise correction based on arbitrary cluster extent
Software	SPM12
Voxelwise p	.01
Cluster extent	50 voxels (size not stated)
Statistical details	—
Findings	↑ L dorsal precentral<br>↑ L anterior cingulate<br>↓ L IFG pars orbitalis<br>↓ L dorsolateral prefrontal cortex<br>↓ L SMA/medial prefrontal<br>↓ L superior parietal<br>↓ L posterior inferior temporal gyrus/fusiform gyrus<br>↓ L occipital<br>↓ R IFG pars orbitalis
Findings notes	—

Voxelwise analysis 13

First level contrast	Verb generation vs finger tapping
Analysis class	Cross-sectional aphasia vs control
Group(s)	Aphasia CIAT T3 (n = 11) vs control
Covariate	—

Is the second level contrast valid in terms of the group(s), time point(s), and measures involved?	Yes
Is accuracy matched across the second level contrast?	Unknown, not reported
Is reaction time matched across the second level contrast?	Unknown, not reported
Behavioral data notes	—
Type of analysis	Voxelwise
Search volume	Voxels spared in all patients
Correction for multiple comparisons	Clusterwise correction based on arbitrary cluster extent
Software	SPM12
Voxelwise p	.01
Cluster extent	50 voxels (size not stated)
Statistical details	—
Findings	↑ L somato-motor<br>↑ L anterior cingulate<br>↑ L posterior cingulate<br>↓ L IFG pars orbitalis<br>↓ L dorsolateral prefrontal cortex<br>↓ L superior parietal<br>↓ L posterior inferior temporal gyrus/fusiform gyrus<br>↓ R dorsolateral prefrontal cortex<br>↓ R mid temporal
Findings notes	—

Voxelwise analysis 14

First level contrast	Verb generation vs finger tapping
Analysis class	Cross-sectional aphasia vs control
Group(s)	Aphasia untreated T1 (n = 8) vs control
Covariate	—
Is the second level contrast valid in terms of the group(s), time point(s), and measures involved?	Yes
Is accuracy matched across the second level contrast?	Unknown, not reported
Is reaction time matched across the second level contrast?	Unknown, not reported
Behavioral data notes	—
Type of analysis	Voxelwise
Search volume	Voxels spared in all patients
Correction for multiple comparisons	Clusterwise correction based on arbitrary cluster extent
Software	SPM12
Voxelwise p	.01
Cluster extent	50 voxels (size not stated)
Statistical details	—
Findings	↑ L superior parietal<br>↑ L occipital<br>↑ L cerebellum<br>↑ R dorsolateral prefrontal cortex<br>↑ R cerebellum<br>↓ L IFG pars orbitalis<br>↓ L SMA/medial prefrontal<br>↓ L posterior inferior temporal gyrus/fusiform gyrus<br>↓ L cerebellum<br>↓ R superior parietal
Findings notes	—

Voxelwise analysis 15

First level contrast	Verb generation vs finger tapping
Analysis class	Cross-sectional aphasia vs control
Group(s)	Aphasia untreated T2 (n = 8) vs control
Covariate	—
Is the second level contrast valid in terms of the group(s), time point(s), and measures involved?	Yes
Is accuracy matched across the second level contrast?	Unknown, not reported
Is reaction time matched across the second level contrast?	Unknown, not reported
Behavioral data notes	—
Type of analysis	Voxelwise
Search volume	Voxels spared in all patients
Correction for multiple comparisons	Clusterwise correction based on arbitrary cluster extent
Software	SPM12
Voxelwise p	.01
Cluster extent	50 voxels (size not stated)
Statistical details	—
Findings	↑ L dorsolateral prefrontal cortex<br>↑ R SMA/medial prefrontal<br>↑ R angular gyrus<br>↑ R posterior STG<br>↑ R posterior cingulate<br>↑ R cerebellum<br>↓ L dorsolateral prefrontal cortex<br>↓ L SMA/medial prefrontal<br>↓ L superior parietal<br>↓ L anterior temporal<br>↓ L posterior inferior temporal gyrus/fusiform gyrus<br>↓ L occipital<br>↓ R superior parietal<br>↓ R occipital<br>↓ R cerebellum
Findings notes	—

Voxelwise analysis 16

First level contrast	Verb generation vs finger tapping
Analysis class	Cross-sectional aphasia vs control
Group(s)	Aphasia untreated T3 (n = 8) vs control
Covariate	—
Is the second level contrast valid in terms of the group(s), time point(s), and measures involved?	Yes
Is accuracy matched across the second level contrast?	Unknown, not reported
Is reaction time matched across the second level contrast?	Unknown, not reported
Behavioral data notes	—
Type of analysis	Voxelwise
Search volume	Voxels spared in all patients
Correction for multiple comparisons	Clusterwise correction based on arbitrary cluster extent
Software	SPM12
Voxelwise p	.01
Cluster extent	50 voxels (size not stated)
Statistical details	—
Findings	↑ L superior parietal<br>↑ L anterior temporal<br>↑ L occipital

	↑ R insula<br>↑ R ventral precentral/inferior frontal junction<br>↑ R orbitofrontal<br>↑ R occipital<br>↑ R cerebellum<br>↓ L IFG pars orbitalis<br>↓ L SMA/medial prefrontal<br>↓ L superior parietal<br>↓ L occipital<br>↓ R insula<br>↓ R dorsolateral prefrontal cortex<br>↓ R cerebellum<br>↓ R basal ganglia
Findings notes	—

Voxelwise analysis 17

First level contrast	Semantic decision vs tone decision
Analysis class	Longitudinal correlation with language or other measure
Group(s)	Aphasia T2 vs T1
Covariate	Δ BNT
Is the second level contrast valid in terms of the group(s), time point(s), and measures involved?	Yes
Is accuracy matched across the second level contrast?	Unknown, not reported
Is reaction time matched across the second level contrast?	Unknown, not reported
Behavioral data notes	—
Type of analysis	Voxelwise
Search volume	Voxels spared in all patients
Correction for multiple comparisons	Clusterwise correction based on arbitrary cluster extent
Software	SPM12
Voxelwise p	.01
Cluster extent	50 voxels (size not stated)
Statistical details	—
Findings	↑ R insula<br>↑ R anterior cingulate<br>↑ R cerebellum<br>↑ R brainstem<br>↑ R basal ganglia
Findings notes	—

Voxelwise analysis 18

First level contrast	Semantic decision vs tone decision
Analysis class	Longitudinal correlation with language or other measure
Group(s)	Aphasia T3 vs T2
Covariate	Δ BNT
Is the second level contrast valid in terms of the group(s), time point(s), and measures involved?	Somewhat (no treatment effect)
Is accuracy matched across the second level contrast?	Unknown, not reported
Is reaction time matched across the second level contrast?	Unknown, not reported
Behavioral data notes	—
Type of analysis	Voxelwise
Search volume	Voxels spared in all patients
Correction for multiple comparisons	Clusterwise correction based on arbitrary cluster extent
Software	SPM12

Voxelwise p	.01
Cluster extent	50 voxels (size not stated)
Statistical details	—
Findings	↑ R somato-motor<br>↑ R posterior MTG<br>↑ R thalamus
Findings notes	—

Voxelwise analysis 19

First level contrast	Verb generation vs finger tapping
Analysis class	Longitudinal correlation with language or other measure
Group(s)	Aphasia T2 vs T1
Covariate	Δ BNT
Is the second level contrast valid in terms of the group(s), time point(s), and measures involved?	Yes
Is accuracy matched across the second level contrast?	Unknown, not reported
Is reaction time matched across the second level contrast?	Unknown, not reported
Behavioral data notes	—
Type of analysis	Voxelwise
Search volume	Voxels spared in all patients
Correction for multiple comparisons	Clusterwise correction based on arbitrary cluster extent
Software	SPM12
Voxelwise p	.01
Cluster extent	50 voxels (size not stated)
Statistical details	—
Findings	↑ R orbitofrontal<br>↑ R mid temporal
Findings notes	—

Voxelwise analysis 20

First level contrast	Verb generation vs finger tapping
Analysis class	Longitudinal correlation with language or other measure
Group(s)	Aphasia T3 vs T2
Covariate	Δ BNT
Is the second level contrast valid in terms of the group(s), time point(s), and measures involved?	Somewhat (no treatment effect)
Is accuracy matched across the second level contrast?	Unknown, not reported
Is reaction time matched across the second level contrast?	Unknown, not reported
Behavioral data notes	—
Type of analysis	Voxelwise
Search volume	Voxels spared in all patients
Correction for multiple comparisons	Clusterwise correction based on arbitrary cluster extent
Software	SPM12
Voxelwise p	.01
Cluster extent	50 voxels (size not stated)
Statistical details	—
Findings	↑ L dorsolateral prefrontal cortex<br>↑ R dorsolateral prefrontal cortex<br>↑ R orbitofrontal
Findings notes	—

ROI analysis 1

First level contrast	Semantic decision vs tone decision
Analysis class	Longitudinal change in aphasia
Group(s)	Aphasia ANOVA including T1, T2, T3
Covariate	—
Is the second level contrast valid in terms of the group(s), time point(s), and measures involved?	Yes
Is accuracy matched across the second level contrast?	Appear similar
Is reaction time matched across the second level contrast?	Unknown, not reported
Behavioral data notes	—
Type of analysis	Regions of interest (ROI)
ROI type	Laterality indi(ces)
How many ROIs are there?	5
What are the ROI(s)?	(1) frontal LI; (2) temporo-parietal LI; (3) cerebellar LI; (4) fronto-parietal LI; (5) Broca's LI
How are the ROI(s) defined?	
Correction for multiple comparisons	No correction
Statistical details	—
Findings	None
Findings notes	—

ROI analysis 2

First level contrast	Semantic decision vs tone decision
Analysis class	Longitudinal between two groups with aphasia
Group(s)	(Aphasia CIAT (n = 11) T1 ≠ T2 ≠ T3) vs (untreated (n = 8) T1 ≠ T2 ≠ T3)
Covariate	—
Is the second level contrast valid in terms of the group(s), time point(s), and measures involved?	Somewhat (no treatment effect)
Is accuracy matched across the second level contrast?	Appear similar
Is reaction time matched across the second level contrast?	Unknown, not reported
Behavioral data notes	—
Type of analysis	Regions of interest (ROI)
ROI type	Laterality indi(ces)
How many ROIs are there?	5
What are the ROI(s)?	(1) frontal LI; (2) temporo-parietal LI; (3) cerebellar LI; (4) fronto-parietal LI; (5) Broca's LI
How are the ROI(s) defined?	
Correction for multiple comparisons	No correction
Statistical details	—
Findings	None
Findings notes	—

ROI analysis 3

First level contrast	Verb generation vs finger tapping
Analysis class	Longitudinal change in aphasia
Group(s)	Aphasia ANOVA including T1, T2, T3
Covariate	—
Is the second level contrast valid in terms of the group(s), time point(s), and measures involved?	Yes
Is accuracy matched across the second level contrast?	Unknown, not reported
Is reaction time matched across the second level contrast?	Unknown, not reported
Behavioral data notes	—

Type of analysis	Regions of interest (ROI)
ROI type	Laterality indi(ces)
How many ROIs are there?	5
What are the ROI(s)?	(1) frontal LI; (2) temporo-parietal LI; (3) cerebellar LI; (4) fronto-parietal LI; (5) Broca's LI
How are the ROI(s) defined?	
Correction for multiple comparisons	No correction
Statistical details	—
Findings	None
Findings notes	—

ROI analysis 4

First level contrast	Verb generation vs finger tapping
Analysis class	Longitudinal between two groups with aphasia
Group(s)	(Aphasia CIAT (n = 11) T1 ≠ T2 ≠ T3) vs (untreated (n = 8) T1 ≠ T2 ≠ T3)
Covariate	—
Is the second level contrast valid in terms of the group(s), time point(s), and measures involved?	Somewhat (no treatment effect)
Is accuracy matched across the second level contrast?	Unknown, not reported
Is reaction time matched across the second level contrast?	Unknown, not reported
Behavioral data notes	—
Type of analysis	Regions of interest (ROI)
ROI type	Laterality indi(ces)
How many ROIs are there?	5
What are the ROI(s)?	(1) frontal LI; (2) temporo-parietal LI; (3) cerebellar LI; (4) fronto-parietal LI; (5) Broca's LI
How are the ROI(s) defined?	
Correction for multiple comparisons	No correction
Statistical details	—
Findings	None
Findings notes	—

Notes

Excluded analyses	(1) pretreatment comparisons between CIAT and untreated groups; (2) Figure 4 caption states that LI values for control group are different to the aphasia groups, but there is no statistical test in support of this

Qiu et al. (2017)

Reference

Authors	Qiu WH, Wu HX, Yang QL, Kang Z, Chen ZC, Li K, Qiu GR, Xie CQ, Wan GF, Chen SQ
Title	Evidence of cortical reorganization of language networks after stroke with subacute Broca's aphasia: a blood oxygenation level dependent-functional magnetic resonance imaging study
Reference	*Neural Regen Res* 2017; 128: 109-117
PMID	28250756
DOI	10.4103/1673-5374.198996

Participants

Language	Mandarin
Inclusion criteria	Broca's aphasia
Number of individuals with aphasia	10
Number of control participants	10

Were any of the participants included in any previous studies?	No
Is age reported for patients and controls, and matched?	Yes (mean 55.9 ± 13.4 years, range 40-70 years)
Is sex reported for patients and controls, and matched?	Yes (males: 7; females: 3)
Is handedness reported for patients and controls, and matched?	Yes (right: 10; left: 0)
Is time post stroke onset reported and appropriate to the study design?	Yes (range 1-3 months)
To what extent is the nature of aphasia characterized?	Severity and type
Language evaluation	WAB
Aphasia severity	Moderate-severe
Aphasia type	All Broca's
First stroke only?	Yes
Stroke type	Mixed etiologies
To what extent is the lesion distribution characterized?	Not at all
Lesion extent	Not stated
Lesion location	L
Participants notes	—

Imaging

Modality	fMRI
Is the study cross-sectional or longitudinal?	Cross-sectional
If longitudinal, at what time point(s) were imaging data acquired?	—
If longitudinal, was there any intervention between the time points?	—
Is the scanner described?	Yes (GE Signa 1.5 Tesla)
Is the timing of stimulus presentation and image acquisition clearly described and appropriate?	No* (moderate limitation) (only three pictures were named per 30-second block)
Design type	Block
Total images acquired	186
Are the imaging acquisition parameters, including coverage, adequately described and appropriate?	Yes (whole brain)
Is preprocessing and intrasubject coregistration adequately described and appropriate?	No (not described)
Is first level model fitting adequately described and appropriate?	No (no description of model fitting)
Is intersubject normalization adequately described and appropriate?	No (not described)
Imaging notes	—

Conditions

Are the conditions clearly described?	Yes

Condition	Response type	Repetitions	All groups could do?	All individuals could do?
picture naming	Word (overt)	9	Unknown	Unknown
rest	None	9	N/A	N/A

Conditions notes	—

Contrasts

Are the contrasts clearly described?	Yes

Contrast 1: picture naming vs rest

Language condition	Picture naming
Control condition	Rest
Are the conditions matched for visual demands?	No
Are the conditions matched for auditory demands?	No
Are the conditions matched for motor demands?	No
Are the conditions matched for cognitive/executive demands?	No
Is accuracy matched between the language and control tasks for all relevant groups?	N/A, tasks not comparable
Is reaction time matched between the language and control tasks for all relevant groups?	N/A, tasks not comparable
Behavioral data notes	—
Are control data reported in this paper or another that is referenced?	Somewhat
Does the contrast selectively activate plausible relevant language regions in the control group?	No
Are activations lateralized in the control data?	Somewhat
Control activation notes	Somewhat L-lateralized frontal and anterior temporal language activations, but the majority of activation is in unexpected regions
Contrast notes	—

Analyses

Are the analyses clearly described?	No* (moderate limitation) (see specific limitation(s) below)

Voxelwise analysis 1

First level contrast	Picture naming vs rest
Analysis class	Cross-sectional aphasia vs control
Group(s)	Aphasia vs control
Covariate	—
Is the second level contrast valid in terms of the group(s), time point(s), and measures involved?	Yes
Is accuracy matched across the second level contrast?	Unknown, not reported
Is reaction time matched across the second level contrast?	Unknown, not reported
Behavioral data notes	—
Type of analysis	Voxelwise
Search volume	Whole brain
Correction for multiple comparisons	Clusterwise correction based on arbitrary cluster extent
Software	SPM8
Voxelwise p	.05
Cluster extent	10 voxels (size not stated)
Statistical details	In the footnote to Table 2, there is a reference to FWE correction with Monte Carlo simulation, but this is not described in the text, and the values in the table appear to be inconsistent with that
Findings	↑ L intraparietal sulcus<br>↑ L posterior inferior temporal gyrus/fusiform gyrus<br>↑ L occipital<br>↑ L thalamus<br>↑ R inferior parietal lobule<br>↑ R intraparietal sulcus<br>↑ R precuneus<br>↑ R anterior temporal<br>↓ L IFG<br>↓ L orbitofrontal

	↓ L somato-motor<br>↓ R ventral precentral/inferior frontal junction
Findings notes	Findings are based on coordinates, which in many cases do not match the labels assigned in the paper

Notes

Excluded analyses	Comparisons between activation volumes in the left and right hemispheres in the two groups, because not described in sufficient detail

Skipper-Kallal et al. (2017a)

Reference

Authors	Skipper-Kallal LM, Lacey EH, Xing S, Turkeltaub PE
Title	Functional activation independently contributes to naming ability and relates to lesion site in post-stroke aphasia
Reference	*Hum Brain Mapp* 2017a; 38: 2051-2066
PMID	28083891
DOI	10.1002/hbm.23504

Participants

Language	US English
Inclusion criteria	Able to name 20% of pictures correctly in the scanner
Number of individuals with aphasia	32 (plus 14 excluded: < 20% accuracy in scanner)
Number of control participants	25
Were any of the participants included in any previous studies?	Yes (29 of the participants overlap with the other Skipper-Kallal et al. (2017) paper)
Is age reported for patients and controls, and matched?	Yes (mean 58.8 ± 8.6 years, range 45.7-78.2 years)
Is sex reported for patients and controls, and matched?	Yes (males: 19; females: 12; stated to be not matched, but difference not significant)
Is handedness reported for patients and controls, and matched?	Yes (right: 26; left: 3; other: 2)
Is time post stroke onset reported and appropriate to the study design?	Yes (mean 40.9 ± 36.1 months, 4.9-151.0 months)
To what extent is the nature of aphasia characterized?	Comprehensive battery
Language evaluation	WAB, PNT
Aphasia severity	AQ mean 77.7 ± 21.0, range 22.8-99.2
Aphasia type	21 anomic, 7 Broca's, 3 conduction, 1 transcortical sensory
First stroke only?	Not stated
Stroke type	Not stated
To what extent is the lesion distribution characterized?	Lesion overlay
Lesion extent	Mean 27.5 ± 22.9 cc
Lesion location	L MCA
Participants notes	—

Imaging

Modality	fMRI
Is the study cross-sectional or longitudinal?	Cross-sectional
If longitudinal, at what time point(s) were imaging data acquired?	—
If longitudinal, was there any intervention between	—

the time points?	
Is the scanner described?	Yes (Siemens Trio 3 Tesla)
Is the timing of stimulus presentation and image acquisition clearly described and appropriate?	No* (moderate limitation) (total images acquired not stated; separation of adjacent events (covert and overt naming) will be limited because of the small amount of jitter in their timing (only 1500 ms))
Design type	Event-related
Total images acquired	~450 but not stated
Are the imaging acquisition parameters, including coverage, adequately described and appropriate?	Yes (whole brain)
Is preprocessing and intrasubject coregistration adequately described and appropriate?	Yes
Is first level model fitting adequately described and appropriate?	No* (moderate limitation) (entire phases where picture was displayed modeled as covert and overt naming; difficult to separate phases due to timing)
Is intersubject normalization adequately described and appropriate?	Yes
Imaging notes	—

Conditions

Are the conditions clearly described?	Yes

Condition	Response type	Repetitions	All groups could do?	All individuals could do?
picture naming (silently name)	Word (covert)	32	Yes	Yes
picture naming (produce the name)	Word (overt)	32	Yes	Yes
rest	None	implicit baseline	N/A	N/A

Conditions notes	Covert and overt naming were modeled as two phases of each trial (there was a cue to produce the name after 7500-9000 ms); 5 participants who were more impaired were given easier pictures to name; patients who named less than 20% of items correctly were excluded

Contrasts

Are the contrasts clearly described?	No (see specific limitation(s) below)

Contrast 1: picture naming (silently name, correct trials) vs rest

Language condition	Picture naming (silently name, correct trials)
Control condition	Rest
Are the conditions matched for visual demands?	No
Are the conditions matched for auditory demands?	Yes
Are the conditions matched for motor demands?	Yes
Are the conditions matched for cognitive/executive demands?	No
Is accuracy matched between the language and control tasks for all relevant groups?	N/A, tasks not comparable
Is reaction time matched between the language and control tasks for all relevant groups?	N/A, tasks not comparable
Behavioral data notes	—
Are control data reported in this paper or another that is referenced?	Yes
Does the contrast selectively activate plausible relevant language regions in the control group?	No
Are activations lateralized in the control data?	No
Control activation notes	Bilateral frontal and occipito-temporal, but not posterior temporal
Contrast notes	—

Contrast 2: picture naming (produce the name, correct trials) vs rest

Language condition	Picture naming (produce the name, correct trials)

Control condition	Rest
Are the conditions matched for visual demands?	No
Are the conditions matched for auditory demands?	No
Are the conditions matched for motor demands?	No
Are the conditions matched for cognitive/executive demands?	No
Is accuracy matched between the language and control tasks for all relevant groups?	N/A, tasks not comparable
Is reaction time matched between the language and control tasks for all relevant groups?	N/A, tasks not comparable
Behavioral data notes	—
Are control data reported in this paper or another that is referenced?	Yes
Does the contrast selectively activate plausible relevant language regions in the control group?	No
Are activations lateralized in the control data?	No
Control activation notes	Bilateral frontal and occipito-temporal, but not posterior temporal; speech motor activation not readily apparent
Contrast notes	—

Contrast 3: picture naming (both phases, correct trials) vs picture naming (both phases, incorrect trials)

Language condition	Picture naming (both phases, correct trials)
Control condition	Picture naming (both phases, incorrect trials)
Are the conditions matched for visual demands?	Yes
Are the conditions matched for auditory demands?	Unknown
Are the conditions matched for motor demands?	Unknown
Are the conditions matched for cognitive/executive demands?	Yes
Is accuracy matched between the language and control tasks for all relevant groups?	No, by design
Is reaction time matched between the language and control tasks for all relevant groups?	Unknown, not reported
Behavioral data notes	—
Are control data reported in this paper or another that is referenced?	N/A
Does the contrast selectively activate plausible relevant language regions in the control group?	N/A
Are activations lateralized in the control data?	N/A
Control activation notes	Control data N/A because controls do not typically make errors
Contrast notes	It is unclear whether there were no-response trials and whether they were modeled as incorrect

Analyses

Are the analyses clearly described?	Yes

Voxelwise analysis 1

First level contrast	Picture naming (silently name, correct trials) vs rest
Analysis class	Cross-sectional aphasia vs control
Group(s)	Aphasia vs control
Covariate	—
Is the second level contrast valid in terms of the group(s), time point(s), and measures involved?	Yes
Is accuracy matched across the second level contrast?	Yes, correct trials only
Is reaction time matched across the second level contrast?	Unknown, not reported

Behavioral data notes	Covert phase but accuracy derived from overt phase
Type of analysis	Voxelwise
Search volume	Whole brain gray matter
Correction for multiple comparisons	Clusterwise correction with with GRFT and lenient voxelwise p
Software	FSL 5.0.6
Voxelwise p	~.01 (z > 2.3)
Cluster extent	Based on GRFT
Statistical details	Threshold of z > 3.1 mentioned in results, but presume 2.3 based on methods and figure
Findings	↑ R precuneus<br>↓ L occipital
Findings notes	—

Voxelwise analysis 2

First level contrast	Picture naming (produce the name, correct trials) vs rest
Analysis class	Cross-sectional aphasia vs control
Group(s)	Aphasia vs control
Covariate	—
Is the second level contrast valid in terms of the group(s), time point(s), and measures involved?	Yes
Is accuracy matched across the second level contrast?	Yes, correct trials only
Is reaction time matched across the second level contrast?	Unknown, not reported
Behavioral data notes	—
Type of analysis	Voxelwise
Search volume	Whole brain gray matter
Correction for multiple comparisons	Clusterwise correction with with GRFT and lenient voxelwise p
Software	FSL 5.0.6
Voxelwise p	~.01 (z > 2.3)
Cluster extent	Based on GRFT
Statistical details	Threshold of z > 3.1 mentioned in results, but presume 2.3 based on methods and figure
Findings	↑ L SMA/medial prefrontal<br>↑ L orbitofrontal<br>↑ L precuneus<br>↑ R insula<br>↑ R ventral precentral/inferior frontal junction<br>↑ R SMA/medial prefrontal<br>↑ R orbitofrontal<br>↑ R somato-motor<br>↑ R supramarginal gyrus<br>↑ R posterior STS<br>↓ L IFG<br>↓ L insula<br>↓ L ventral precentral/inferior frontal junction<br>↓ L intraparietal sulcus<br>↓ L anterior temporal<br>↓ L hippocampus/MTL<br>↓ R intraparietal sulcus
Findings notes	Labels based largely on text with some adjustments based on figures; overall pattern of decreased L activity and increased R activity is quite convincing

Voxelwise analysis 3

First level contrast	Picture naming (silently name, correct trials) vs rest
Analysis class	Cross-sectional correlation with language or other measure
Group(s)	Aphasia
Covariate	PNT
Is the second level contrast valid in terms of the	Yes

group(s), time point(s), and measures involved?	
Is accuracy matched across the second level contrast?	Yes, correct trials only
Is reaction time matched across the second level contrast?	Unknown, not reported
Behavioral data notes	Covert phase but accuracy derived from overt phase
Type of analysis	Voxelwise
Search volume	Whole brain gray matter
Correction for multiple comparisons	Clusterwise correction with with GRFT and lenient voxelwise p
Software	FSL 5.0.6
Voxelwise p	~.01 (z > 2.3)
Cluster extent	Based on GRFT
Statistical details	—
Findings	↑ L anterior temporal<br>↓ L SMA/medial prefrontal<br>↓ L supramarginal gyrus<br>↓ R SMA/medial prefrontal<br>↓ R somato-motor
Findings notes	L anterior temporal correlation remained significant after accounting for lesion load and other factors

Voxelwise analysis 4

First level contrast	Picture naming (produce the name, correct trials) vs rest
Analysis class	Cross-sectional correlation with language or other measure
Group(s)	Aphasia
Covariate	PNT
Is the second level contrast valid in terms of the group(s), time point(s), and measures involved?	Yes
Is accuracy matched across the second level contrast?	Yes, correct trials only
Is reaction time matched across the second level contrast?	Unknown, not reported
Behavioral data notes	—
Type of analysis	Voxelwise
Search volume	Whole brain gray matter
Correction for multiple comparisons	Clusterwise correction with with GRFT and lenient voxelwise p
Software	FSL 5.0.6
Voxelwise p	~.01 (z > 2.3)
Cluster extent	Based on GRFT
Statistical details	—
Findings	↑ L posterior STG<br>↑ R somato-motor<br>↑ R posterior STS<br>↑ R occipital<br>↓ L IFG pars orbitalis<br>↓ L dorsolateral prefrontal cortex<br>↓ L angular gyrus
Findings notes	L IFG pars orbitalis, R pSTS, and R somato-motor correlations remained remained significant after accounting for lesion load and other factors; note that the pars orbitalis region is described as frontal pole in the paper but the coordinates and image support pars orbitalis

Voxelwise analysis 5

First level contrast	Picture naming (both phases, correct trials) vs picture naming (both phases, incorrect trials)
Analysis class	Cross-sectional performance-defined conditions
Group(s)	Aphasia with naming < 80% (n = 24)
Covariate	—

Is the second level contrast valid in terms of the group(s), time point(s), and measures involved?	Yes
Is accuracy matched across the second level contrast?	No, by design
Is reaction time matched across the second level contrast?	Unknown, not reported
Behavioral data notes	—
Type of analysis	Voxelwise
Search volume	Whole brain gray matter
Correction for multiple comparisons	Clusterwise correction with with GRFT and lenient voxelwise p
Software	FSL 5.0.6
Voxelwise p	~.01 (z > 2.3)
Cluster extent	Based on GRFT
Statistical details	—
Findings	None
Findings notes	—

ROI analysis 1

First level contrast	Picture naming (produce the name, correct trials) vs rest
Analysis class	Cross-sectional correlation with language or other measure
Group(s)	Aphasia
Covariate	PNT
Is the second level contrast valid in terms of the group(s), time point(s), and measures involved?	Yes
Is accuracy matched across the second level contrast?	Yes, correct trials only
Is reaction time matched across the second level contrast?	Unknown, not reported
Behavioral data notes	—
Type of analysis	Regions of interest (ROI)
ROI type	Functional
How many ROIs are there?	11
What are the ROI(s)?	(1) right IPS; (2) left IPS; (3) left PTr; (4) left dPOp; (5) right superior motor cortex; (6) right ventral motor cortex; (7) right supramarginal sulcus; (8) left medial SMA; (9) right marginal sulcus; (10) left dorsal motor cortex; (11) right STS
How are the ROI(s) defined?	Regions that were activated for control > aphasia (ROIs 1-4) or aphasia > control (ROIs 5-11)
Correction for multiple comparisons	Familywise error (FWE)
Statistical details	—
Findings	↑ R ventral precentral/inferior frontal junction<br>↑ R posterior STS<br>↓ L IFG pars opercularis
Findings notes	The L IFG pars opercularis and the R posterior STS also contributed to predicting PNT scores even when lesion load on critical areas for picture naming, and several other variables, were included in multiple regression models

ROI analysis 2

First level contrast	Picture naming (silently name, correct trials) vs rest
Analysis class	Cross-sectional aphasia vs control
Group(s)	Aphasia vs control
Covariate	—
Is the second level contrast valid in terms of the group(s), time point(s), and measures involved?	Yes
Is accuracy matched across the second level contrast?	Yes, correct trials only
Is reaction time matched across the second level contrast?	Unknown, not reported

Behavioral data notes	—
Type of analysis	Region of interest (ROI)
ROI type	Functional
How many ROIs are there?	1
What are the ROI(s)?	L anterior temporal
How are the ROI(s) defined?	Activity for covert naming correlated with naming ability in patients, after controlling for lesion and demographic factors
Correction for multiple comparisons	One only
Statistical details	—
Findings	None
Findings notes	—

ROI analysis 3

First level contrast	Picture naming (produce the name, correct trials) vs rest
Analysis class	Cross-sectional aphasia vs control
Group(s)	Aphasia vs control
Covariate	—
Is the second level contrast valid in terms of the group(s), time point(s), and measures involved?	Yes
Is accuracy matched across the second level contrast?	Yes, correct trials only
Is reaction time matched across the second level contrast?	Unknown, not reported
Behavioral data notes	—
Type of analysis	Regions of interest (ROI)
ROI type	Functional
How many ROIs are there?	3
What are the ROI(s)?	(1) L frontal pole; (2) R postcentral gyrus; (3) R STS
How are the ROI(s) defined?	Activity for overt naming correlated with naming ability in patients, after controlling for lesion and demographic factors
Correction for multiple comparisons	No correction
Statistical details	—
Findings	↑ R somato-motor<br>↑ R posterior STS
Findings notes	—

Complex analysis 1

First level contrast	Picture naming (produce the name, correct trials) vs rest
Analysis class	Cross-sectional correlation with language or other measure
Group(s)	Aphasia
Covariate	Lesion patterns identified with SVR-LSM
Is the second level contrast valid in terms of the group(s), time point(s), and measures involved?	Yes
Is accuracy matched across the second level contrast?	Yes, correct trials only
Is reaction time matched across the second level contrast?	Unknown, not reported
Behavioral data notes	—
Type of analysis	Complex
Statistical details	SVR-LSM was used to identify regions of damage associated with activation of R pSTS ROI (defined based on SPM analysis 2). The results were thresholded at voxelwise $p < .01$ (CDT), cluster extent > 500 voxels.
Findings	Other
Findings notes	Damage to the L IFG pars opercularis was associated with more activity in the R pSTS. Damage to the L pSTS was associated with less activity in the R pSTS.

Complex analysis 2

First level contrast	Picture naming (produce the name, correct trials) vs rest
Analysis class	Cross-sectional correlation with language or other measure
Group(s)	Aphasia without IFG POp damage (n = 26)
Covariate	Lesion patterns identified with SVR-LSM
Is the second level contrast valid in terms of the group(s), time point(s), and measures involved?	Yes
Is accuracy matched across the second level contrast?	Yes, correct trials only
Is reaction time matched across the second level contrast?	Unknown, not reported
Behavioral data notes	—
Type of analysis	Complex
Statistical details	SVR-LSM was used to identify regions of damage associated with activation of L IFG pars opercularis ROI (defined based on SPM analysis 2). The results were thresholded at voxelwise $p < .01$ (CDT), cluster extent > 500 voxels.
Findings	Other
Findings notes	Damage to the L pSTG, L pSTS, and white matter underlying the L precuneus was associated with more activity in the L IFG pars opercularis. There were no regions associated with less activity.

Notes

Excluded analyses	Negative correlation between functional activation in the L IFG pars opercularis and R pSTS

Skipper-Kallal et al. (2017b)

Reference

Authors	Skipper-Kallal LM, Lacey EH, Xing S, Turkeltaub PE
Title	Right hemisphere remapping of naming functions depends on lesion size and location in poststroke aphasia
Reference	*Neural Plast* 2017b; 2017: 8740353
PMID	28168061
DOI	10.1155/2017/8740353

Participants

Language	US English
Inclusion criteria	10% accuracy on scanner task
Number of individuals with aphasia	39 (plus 10 excluded: < 10% accuracy in scanner)
Number of control participants	37
Were any of the participants included in any previous studies?	Yes (29 of the participants overlap with the other Skipper-Kallal et al. (2017) paper)
Is age reported for patients and controls, and matched?	Yes (mean 59.8 ± 10.0 years)
Is sex reported for patients and controls, and matched?	Yes (males: 26; females: 13)
Is handedness reported for patients and controls, and matched?	Yes (right: 33; left: 4; other: 2; missing for 2 participants)
Is time post stroke onset reported and appropriate to the study design?	Yes (mean 52.9 ± 51.4 months, range 6.3-255.7 months)
To what extent is the nature of aphasia characterized?	Comprehensive battery
Language evaluation	WAB, PNT
Aphasia severity	Not stated

Aphasia type	23 anomic, 11 Broca's, 3 conduction, 1 transcortical sensory, 1 Wernicke's
First stroke only?	Not stated
Stroke type	Not stated
To what extent is the lesion distribution characterized?	Lesion overlay
Lesion extent	Not stated
Lesion location	L MCA
Participants notes	—

Imaging

Modality	fMRI
Is the study cross-sectional or longitudinal?	Cross-sectional
If longitudinal, at what time point(s) were imaging data acquired?	—
If longitudinal, was there any intervention between the time points?	—
Is the scanner described?	Yes (Siemens Trio 3 Tesla)
Is the timing of stimulus presentation and image acquisition clearly described and appropriate?	No* (moderate limitation) (total images acquired not stated; separation of adjacent events (covert and overt naming) will be limited because of the small amount of jitter in their timing (only 1500 ms))
Design type	Event-related
Total images acquired	~450 but not stated
Are the imaging acquisition parameters, including coverage, adequately described and appropriate?	Yes (whole brain)
Is preprocessing and intrasubject coregistration adequately described and appropriate?	Yes
Is first level model fitting adequately described and appropriate?	No* (moderate limitation) (not stated but see Skipper-Kallal et al. (2017b))
Is intersubject normalization adequately described and appropriate?	Yes
Imaging notes	at each voxel, individuals with lesions to that voxel were excluded from analysis

Conditions

Are the conditions clearly described?	Yes

Condition	Response type	Repetitions	All groups could do?	All individuals could do?
picture naming (prepare to name)	Word (covert)	32	Yes	Yes
picture naming (produce the name)	Word (overt)	32	Yes	Yes
rest	None	implicit baseline	N/A	N/A

Conditions notes	Covert and overt naming were modeled as two phases of each trial (there was a cue to produce the name after 7500-9000 ms); 14 participants who were more impaired were given easier pictures to name; patients who named less than 10% of items correctly were excluded

Contrasts

Are the contrasts clearly described?	Yes

Contrast 1: picture naming (prepare to name, correct trials) vs rest

Language condition	Picture naming (prepare to name, correct trials)
Control condition	Rest
Are the conditions matched for visual demands?	No
Are the conditions matched for auditory demands?	Yes
Are the conditions matched for motor demands?	Yes
Are the conditions matched for cognitive/executive demands?	No

Is accuracy matched between the language and control tasks for all relevant groups?	N/A, tasks not comparable
Is reaction time matched between the language and control tasks for all relevant groups?	N/A, tasks not comparable
Behavioral data notes	—
Are control data reported in this paper or another that is referenced?	Yes
Does the contrast selectively activate plausible relevant language regions in the control group?	No
Are activations lateralized in the control data?	No
Control activation notes	Bilateral frontal and occipito-temporal, but not posterior temporal
Contrast notes	—

Contrast 2: picture naming (produce the name, correct trials) vs rest

Language condition	Picture naming (produce the name, correct trials)
Control condition	Rest
Are the conditions matched for visual demands?	No
Are the conditions matched for auditory demands?	No
Are the conditions matched for motor demands?	No
Are the conditions matched for cognitive/executive demands?	No
Is accuracy matched between the language and control tasks for all relevant groups?	N/A, tasks not comparable
Is reaction time matched between the language and control tasks for all relevant groups?	N/A, tasks not comparable
Behavioral data notes	—
Are control data reported in this paper or another that is referenced?	Yes
Does the contrast selectively activate plausible relevant language regions in the control group?	No
Are activations lateralized in the control data?	No
Control activation notes	Bilateral frontal and occipito-temporal, but not posterior temporal; speech motor activation not readily apparent
Contrast notes	—

Analyses

Are the analyses clearly described?	Yes

Voxelwise analysis 1

First level contrast	Picture naming (prepare to name, correct trials) vs rest
Analysis class	Cross-sectional aphasia vs control
Group(s)	Aphasia vs control
Covariate	—
Is the second level contrast valid in terms of the group(s), time point(s), and measures involved?	Yes
Is accuracy matched across the second level contrast?	Yes, correct trials only
Is reaction time matched across the second level contrast?	Unknown, not reported
Behavioral data notes	Covert phase but accuracy derived from overt phase
Type of analysis	Voxelwise
Search volume	Whole brain
Correction for multiple comparisons	Clusterwise correction with with GRFT and lenient voxelwise p
Software	FSL 5.0.6
Voxelwise p	.01
Cluster extent	Based on GRFT

Statistical details	—
Findings	↑ L cerebellum<br>↑ L thalamus<br>↑ L basal ganglia<br>↑ R IFG pars opercularis<br>↑ R insula<br>↑ R cerebellum<br>↑ R basal ganglia<br>↓ L dorsolateral prefrontal cortex<br>↓ L orbitofrontal<br>↓ L intraparietal sulcus<br>↓ L anterior cingulate<br>↓ R dorsolateral prefrontal cortex
Findings notes	Based on Table 2

Voxelwise analysis 2

First level contrast	Picture naming (produce the name, correct trials) vs rest
Analysis class	Cross-sectional aphasia vs control
Group(s)	Aphasia vs control
Covariate	—
Is the second level contrast valid in terms of the group(s), time point(s), and measures involved?	Yes
Is accuracy matched across the second level contrast?	Yes, correct trials only
Is reaction time matched across the second level contrast?	Unknown, not reported
Behavioral data notes	—
Type of analysis	Voxelwise
Search volume	Whole brain
Correction for multiple comparisons	Clusterwise correction with with GRFT and lenient voxelwise p
Software	FSL 5.0.6
Voxelwise p	.01
Cluster extent	Based on GRFT
Statistical details	—
Findings	↑ L somato-motor<br>↑ L intraparietal sulcus<br>↑ L anterior cingulate<br>↑ R insula<br>↑ R dorsal precentral<br>↑ R somato-motor<br>↑ R supramarginal gyrus<br>↑ R posterior MTG<br>↑ R Heschl's gyrus<br>↓ L ventral precentral/inferior frontal junction<br>↓ L somato-motor<br>↓ L posterior STG/STS/MTG<br>↓ L mid temporal<br>↓ L anterior temporal<br>↓ L cerebellum<br>↓ L thalamus<br>↓ L hippocampus/MTL
Findings notes	Based on Table 3

Voxelwise analysis 3

First level contrast	Picture naming (prepare to name, correct trials) vs rest
Analysis class	Cross-sectional correlation with language or other measure
Group(s)	Aphasia
Covariate	Lesion volume

Is the second level contrast valid in terms of the group(s), time point(s), and measures involved?	Yes
Is accuracy matched across the second level contrast?	Yes, correct trials only
Is reaction time matched across the second level contrast?	Unknown, not reported
Behavioral data notes	Covert phase but accuracy derived from overt phase
Type of analysis	Voxelwise
Search volume	Whole brain
Correction for multiple comparisons	Clusterwise correction with with GRFT and lenient voxelwise p
Software	FSL 5.0.6
Voxelwise p	.01
Cluster extent	Based on GRFT
Statistical details	—
Findings	↑ L ventral precentral/inferior frontal junction<br>↑ L intraparietal sulcus<br>↑ L superior parietal<br>↑ L occipital<br>↑ L basal ganglia<br>↑ R IFG<br>↑ R insula<br>↑ R ventral precentral/inferior frontal junction<br>↑ R SMA/medial prefrontal<br>↑ R somato-motor<br>↑ R intraparietal sulcus<br>↑ R occipital<br>↑ R cerebellum<br>↑ R brainstem<br>↑ R basal ganglia
Findings notes	Based on Table 4, except for R frontal activations which are missing from the table, and were added based on the figure

Voxelwise analysis 4

First level contrast	Picture naming (produce the name, correct trials) vs rest
Analysis class	Cross-sectional correlation with language or other measure
Group(s)	Aphasia
Covariate	Lesion volume
Is the second level contrast valid in terms of the group(s), time point(s), and measures involved?	Yes
Is accuracy matched across the second level contrast?	Yes, correct trials only
Is reaction time matched across the second level contrast?	Unknown, not reported
Behavioral data notes	—
Type of analysis	Voxelwise
Search volume	Whole brain
Correction for multiple comparisons	Clusterwise correction with with GRFT and lenient voxelwise p
Software	FSL 5.0.6
Voxelwise p	.01
Cluster extent	Based on GRFT
Statistical details	—
Findings	↑ L somato-motor<br>↑ L precuneus<br>↑ L occipital<br>↑ L cerebellum<br>↑ R IFG pars triangularis<br>↑ R insula<br>↑ R ventral precentral/inferior frontal junction

	↑ R SMA/medial prefrontal<br>↑ R posterior STG/STS/MTG<br>↑ R mid temporal<br>↑ R occipital<br>↑ R cerebellum<br>↑ R basal ganglia<br>↑ R hippocampus/MTL
Findings notes	Based on Table 4, except for bilateral occipital activations which are missing from the table, and were added based on the figure

Voxelwise analysis 5

First level contrast	Picture naming (prepare to name, correct trials) vs rest
Analysis class	Cross-sectional between two groups with aphasia
Group(s)	Aphasia with IPS damage (n not stated) vs without IPS damage (n not stated)
Covariate	—
Is the second level contrast valid in terms of the group(s), time point(s), and measures involved?	Yes
Is accuracy matched across the second level contrast?	Yes, correct trials only
Is reaction time matched across the second level contrast?	Unknown, not reported
Behavioral data notes	Covert phase but accuracy derived from overt phase
Type of analysis	Voxelwise
Search volume	Whole brain
Correction for multiple comparisons	Clusterwise correction with with GRFT and lenient voxelwise p
Software	FSL 5.0.6
Voxelwise p	.01
Cluster extent	Based on GRFT
Statistical details	Lesion volume covariate
Findings	None
Findings notes	—

Voxelwise analysis 6

First level contrast	Picture naming (prepare to name, correct trials) vs rest
Analysis class	Cross-sectional between two groups with aphasia
Group(s)	Aphasia with insula damage (n = 18) vs without insula damage (n = 21)
Covariate	—
Is the second level contrast valid in terms of the group(s), time point(s), and measures involved?	Yes
Is accuracy matched across the second level contrast?	Yes, correct trials only
Is reaction time matched across the second level contrast?	Unknown, not reported
Behavioral data notes	Covert phase but accuracy derived from overt phase
Type of analysis	Voxelwise
Search volume	Whole brain
Correction for multiple comparisons	Clusterwise correction with with GRFT and lenient voxelwise p
Software	FSL 5.0.6
Voxelwise p	.01
Cluster extent	Based on GRFT
Statistical details	Lesion volume covariate
Findings	↓ R IFG pars triangularis<br>↓ R dorsolateral prefrontal cortex
Findings notes	—

Voxelwise analysis 7

First level contrast	Picture naming (prepare to name, correct trials) vs rest
Analysis class	Cross-sectional between two groups with aphasia
Group(s)	Aphasia with IFG POp damage (n = 16) vs without IFG POp damage (n = 23)
Covariate	—
Is the second level contrast valid in terms of the group(s), time point(s), and measures involved?	Yes
Is accuracy matched across the second level contrast?	Yes, correct trials only
Is reaction time matched across the second level contrast?	Unknown, not reported
Behavioral data notes	Covert phase but accuracy derived from overt phase
Type of analysis	Voxelwise
Search volume	Whole brain
Correction for multiple comparisons	Clusterwise correction with with GRFT and lenient voxelwise p
Software	FSL 5.0.6
Voxelwise p	.01
Cluster extent	Based on GRFT
Statistical details	Lesion volume covariate
Findings	↓ R IFG pars triangularis<br>↓ R dorsolateral prefrontal cortex
Findings notes	—

Voxelwise analysis 8

First level contrast	Picture naming (produce the name, correct trials) vs rest
Analysis class	Cross-sectional between two groups with aphasia
Group(s)	Aphasia with motor cortex damage (n = 24) vs without motor cortex damage (n = 15)
Covariate	—
Is the second level contrast valid in terms of the group(s), time point(s), and measures involved?	Yes
Is accuracy matched across the second level contrast?	Yes, correct trials only
Is reaction time matched across the second level contrast?	Unknown, not reported
Behavioral data notes	—
Type of analysis	Voxelwise
Search volume	Whole brain
Correction for multiple comparisons	Clusterwise correction with with GRFT and lenient voxelwise p
Software	FSL 5.0.6
Voxelwise p	.01
Cluster extent	Based on GRFT
Statistical details	Lesion volume covariate
Findings	None
Findings notes	—

Voxelwise analysis 9

First level contrast	Picture naming (produce the name, correct trials) vs rest
Analysis class	Cross-sectional between two groups with aphasia
Group(s)	Aphasia with STS damage (n not stated) vs without STS damage (n not stated)
Covariate	—
Is the second level contrast valid in terms of the group(s), time point(s), and measures involved?	Yes
Is accuracy matched across the second level contrast?	Yes, correct trials only
Is reaction time matched across the second level contrast?	Unknown, not reported

Behavioral data notes	—
Type of analysis	Voxelwise
Search volume	Whole brain
Correction for multiple comparisons	Clusterwise correction with with GRFT and lenient voxelwise p
Software	FSL 5.0.6
Voxelwise p	.01
Cluster extent	Based on GRFT
Statistical details	Lesion volume covariate
Findings	None
Findings notes	—

ROI analysis 1

First level contrast	Picture naming (prepare to name, correct trials) vs rest
Analysis class	Cross-sectional correlation with language or other measure
Group(s)	Aphasia with IFG POp damage (n = 16)
Covariate	PNT
Is the second level contrast valid in terms of the group(s), time point(s), and measures involved?	Yes
Is accuracy matched across the second level contrast?	Yes, correct trials only
Is reaction time matched across the second level contrast?	Unknown, not reported
Behavioral data notes	Covert phase but accuracy derived from overt phase
Type of analysis	Region of interest (ROI)
ROI type	Functional
How many ROIs are there?	1
What are the ROI(s)?	R DLPFC
How are the ROI(s) defined?	Peak location for decreased activation for patients with left insula and left POp lesions compared to patients without said damage
Correction for multiple comparisons	One only
Statistical details	Lesion volume covariate
Findings	None
Findings notes	—

ROI analysis 2

First level contrast	Picture naming (prepare to name, correct trials) vs rest
Analysis class	Cross-sectional correlation with language or other measure
Group(s)	Aphasia without IFG POp damage (n = 23)
Covariate	PNT
Is the second level contrast valid in terms of the group(s), time point(s), and measures involved?	Yes
Is accuracy matched across the second level contrast?	Yes, correct trials only
Is reaction time matched across the second level contrast?	Unknown, not reported
Behavioral data notes	Covert phase but accuracy derived from overt phase
Type of analysis	Region of interest (ROI)
ROI type	Functional
How many ROIs are there?	1
What are the ROI(s)?	R DLPFC
How are the ROI(s) defined?	Peak location for decreased activation for patients with left insula and left POp lesions compared to patients without said damage
Correction for multiple comparisons	One only
Statistical details	Lesion volume covariate
Findings	None

Findings notes	—

ROI analysis 3

First level contrast	Picture naming (prepare to name, correct trials) vs rest
Analysis class	Cross-sectional correlation with language or other measure
Group(s)	Aphasia with insula damage (n = 18)
Covariate	PNT
Is the second level contrast valid in terms of the group(s), time point(s), and measures involved?	Yes
Is accuracy matched across the second level contrast?	Yes, correct trials only
Is reaction time matched across the second level contrast?	Unknown, not reported
Behavioral data notes	Covert phase but accuracy derived from overt phase
Type of analysis	Region of interest (ROI)
ROI type	Functional
How many ROIs are there?	1
What are the ROI(s)?	R DLPFC
How are the ROI(s) defined?	Peak location for decreased activation for patients with left insula and left POp lesions compared to patients without said damage
Correction for multiple comparisons	One only
Statistical details	Lesion volume covariate
Findings	None
Findings notes	—

ROI analysis 4

First level contrast	Picture naming (prepare to name, correct trials) vs rest
Analysis class	Cross-sectional correlation with language or other measure
Group(s)	Aphasia without insula damage (n = 21)
Covariate	PNT
Is the second level contrast valid in terms of the group(s), time point(s), and measures involved?	Yes
Is accuracy matched across the second level contrast?	Yes, correct trials only
Is reaction time matched across the second level contrast?	Unknown, not reported
Behavioral data notes	Covert phase but accuracy derived from overt phase
Type of analysis	Region of interest (ROI)
ROI type	Functional
How many ROIs are there?	1
What are the ROI(s)?	R DLPFC
How are the ROI(s) defined?	Peak location for decreased activation for patients with left insula and left POp lesions compared to patients without said damage
Correction for multiple comparisons	One only
Statistical details	Lesion volume covariate
Findings	None
Findings notes	—

ROI analysis 5

First level contrast	Picture naming (prepare to name, correct trials) vs rest
Analysis class	Cross-sectional between two groups with aphasia
Group(s)	Aphasia with IPS damage (n not stated) vs without IPS damage (n not stated)
Covariate	—
Is the second level contrast valid in terms of the group(s), time point(s), and measures involved?	Yes

Is accuracy matched across the second level contrast?	Yes, correct trials only
Is reaction time matched across the second level contrast?	Unknown, not reported
Behavioral data notes	Covert phase but accuracy derived from overt phase
Type of analysis	Regions of interest (ROI)
ROI type	Functional
How many ROIs are there?	5
What are the ROI(s)?	(1) L IPS; (2) L insula; (3) L IFG pars opercularis; (4) R IPS; (5) R insula
How are the ROI(s) defined?	5 mm spheres around control peaks
Correction for multiple comparisons	No correction
Statistical details	Lesion volume covariate
Findings	None
Findings notes	—

ROI analysis 6

First level contrast	Picture naming (prepare to name, correct trials) vs rest
Analysis class	Cross-sectional between two groups with aphasia
Group(s)	Aphasia with insula damage (n = 18) vs without insula damage (n = 21)
Covariate	—
Is the second level contrast valid in terms of the group(s), time point(s), and measures involved?	Yes
Is accuracy matched across the second level contrast?	Yes, correct trials only
Is reaction time matched across the second level contrast?	Unknown, not reported
Behavioral data notes	Covert phase but accuracy derived from overt phase
Type of analysis	Regions of interest (ROI)
ROI type	Functional
How many ROIs are there?	5
What are the ROI(s)?	(1) L IPS; (2) L insula; (3) L IFG pars opercularis; (4) R IPS; (5) R insula
How are the ROI(s) defined?	5 mm spheres around control peaks
Correction for multiple comparisons	No correction
Statistical details	Lesion volume covariate
Findings	None
Findings notes	—

ROI analysis 7

First level contrast	Picture naming (prepare to name, correct trials) vs rest
Analysis class	Cross-sectional between two groups with aphasia
Group(s)	Aphasia with IFG POp damage (n = 16) vs without IFG POp damage (n = 23)
Covariate	—
Is the second level contrast valid in terms of the group(s), time point(s), and measures involved?	Yes
Is accuracy matched across the second level contrast?	Yes, correct trials only
Is reaction time matched across the second level contrast?	Unknown, not reported
Behavioral data notes	Covert phase but accuracy derived from overt phase
Type of analysis	Regions of interest (ROI)
ROI type	Functional
How many ROIs are there?	5
What are the ROI(s)?	(1) L IPS; (2) L insula; (3) L IFG pars opercularis; (4) R IPS; (5) R insula
How are the ROI(s) defined?	5 mm spheres around control peaks
Correction for multiple comparisons	No correction

Statistical details	Lesion volume covariate
Findings	None
Findings notes	—

ROI analysis 8

First level contrast	Picture naming (produce the name, correct trials) vs rest
Analysis class	Cross-sectional between two groups with aphasia
Group(s)	Aphasia with motor cortex damage (n = 24) vs without motor cortex damage (n = 15)
Covariate	—
Is the second level contrast valid in terms of the group(s), time point(s), and measures involved?	Yes
Is accuracy matched across the second level contrast?	Yes, correct trials only
Is reaction time matched across the second level contrast?	Unknown, not reported
Behavioral data notes	—
Type of analysis	Regions of interest (ROI)
ROI type	Functional
How many ROIs are there?	4
What are the ROI(s)?	(1) L motor; (2) L pSTS; (3) R motor; (4) R pSTS
How are the ROI(s) defined?	5 mm spheres around control peaks
Correction for multiple comparisons	No correction
Statistical details	Lesion volume covariate
Findings	↑ R somato-motor
Findings notes	—

ROI analysis 9

First level contrast	Picture naming (produce the name, correct trials) vs rest
Analysis class	Cross-sectional between two groups with aphasia
Group(s)	Aphasia with STS damage (n not stated) vs without STS damage (n not stated)
Covariate	—
Is the second level contrast valid in terms of the group(s), time point(s), and measures involved?	Yes
Is accuracy matched across the second level contrast?	Yes, correct trials only
Is reaction time matched across the second level contrast?	Unknown, not reported
Behavioral data notes	—
Type of analysis	Regions of interest (ROI)
ROI type	Functional
How many ROIs are there?	4
What are the ROI(s)?	(1) L motor; (2) L pSTS; (3) R motor; (4) R pSTS
How are the ROI(s) defined?	5 mm spheres around control peaks
Correction for multiple comparisons	No correction
Statistical details	Lesion volume covariate
Findings	↓ R somato-motor
Findings notes	—

ROI analysis 10

First level contrast	Picture naming (produce the name, correct trials) vs rest
Analysis class	Cross-sectional correlation with language or other measure
Group(s)	Aphasia without motor cortex damage (n = 15)
Covariate	PNT
Is the second level contrast valid in terms of the group(s), time point(s), and measures involved?	Yes

Is accuracy matched across the second level contrast?	Yes, correct trials only
Is reaction time matched across the second level contrast?	Unknown, not reported
Behavioral data notes	—
Type of analysis	Region of interest (ROI)
ROI type	Functional
How many ROIs are there?	1
What are the ROI(s)?	R motor
How are the ROI(s) defined?	5 mm sphere around control peak
Correction for multiple comparisons	One only
Statistical details	Lesion volume covariate
Findings	None
Findings notes	—

ROI analysis 11

First level contrast	Picture naming (produce the name, correct trials) vs rest
Analysis class	Cross-sectional correlation with language or other measure
Group(s)	Aphasia with motor cortex damage (n = 24)
Covariate	PNT
Is the second level contrast valid in terms of the group(s), time point(s), and measures involved?	Yes
Is accuracy matched across the second level contrast?	Yes, correct trials only
Is reaction time matched across the second level contrast?	Unknown, not reported
Behavioral data notes	—
Type of analysis	Region of interest (ROI)
ROI type	Functional
How many ROIs are there?	1
What are the ROI(s)?	R motor
How are the ROI(s) defined?	5 mm sphere around control peak
Correction for multiple comparisons	One only
Statistical details	Lesion volume covariate
Findings	↑ R somato-motor
Findings notes	—

Notes

Excluded analyses	—

Dietz et al. (2018)

Reference

Authors	Dietz A, Vannest J, Maloney T, Altaye M, Holland S, Szaflarski JP
Title	The feasibility of improving discourse in people with aphasia through AAC: clinical and functional MRI correlates
Reference	*Aphasiology* 2018; 32: 693-719
PMID	N/A
DOI	10.1080/02687038.2018.1447641

Participants

Language	US English

Inclusion criteria	—
Number of individuals with aphasia	12 (plus 2 excluded: 1 for illness; 1 for MRI contraindication or personal conflict (inconsistent information provided))
Number of control participants	0
Were any of the participants included in any previous studies?	Yes (same data as Dietz et al. (2016), which is a methodological paper)
Is age reported for patients and controls, and matched?	Yes (AAC group: range 39-63 years; usual care group: range 47-71 years)
Is sex reported for patients and controls, and matched?	Yes (males: 5; females: 7)
Is handedness reported for patients and controls, and matched?	Yes (right: 11; left: 1)
Is time post stroke onset reported and appropriate to the study design?	Yes (AAC group: range 16-170 months; usual care group: range 38-105 months)
To what extent is the nature of aphasia characterized?	Severity and type
Language evaluation	WAB, Reading Comprehension Battery for Aphasia
Aphasia severity	AAC group: AQ range 37.6-82.4; usual care group: AQ range 36.7-89.2
Aphasia type	AAC group: 2 Broca's, 1 anomic, 1 conduction, 1 global, 1 Wernicke's; usual care group: 2 anomic, 2 Broca's, 1 conduction, 1 Wernicke's
First stroke only?	Yes
Stroke type	Ischemic only
To what extent is the lesion distribution characterized?	Individual lesions
Lesion extent	AAC group: range 7849-30570 voxels; usual care group: 1583-30110 voxels (voxel size not stated)
Lesion location	L MCA
Participants notes	—

Imaging

Modality	fMRI
Is the study cross-sectional or longitudinal?	Longitudinal—chronic treatment
If longitudinal, at what time point(s) were imaging data acquired?	T1: pre-treatment/chronic; T2: post-treatment, ~4 weeks later
If longitudinal, was there any intervention between the time points?	AAC group: treatment aimed at teaching participants how to utilize AAC to facilitate discourse; usual care group: traditional SLT, not focused on discourse or AAC specifically
Is the scanner described?	Yes (Philips Achieva 3 Tesla)
Is the timing of stimulus presentation and image acquisition clearly described and appropriate?	Yes
Design type	Event-related
Total images acquired	135
Are the imaging acquisition parameters, including coverage, adequately described and appropriate?	Yes (whole brain)
Is preprocessing and intrasubject coregistration adequately described and appropriate?	Yes
Is first level model fitting adequately described and appropriate?	No (no description of HRF model, which is important given sparse sampling design)
Is intersubject normalization adequately described and appropriate?	No (lesion impact not addressed)
Imaging notes	additional methodological details in Dietz et al. (2016)

Conditions

Are the conditions clearly described?	Yes

Condition	Response type	Repetitions	All groups could do?	All individuals could do?
verb generation (covert)	Multiple words (covert)	15	Unknown	Unknown

verb generation (overt)	Multiple words (overt)	15	Yes	Unknown
noun repetition	Multiple words (overt)	15	Yes	Unknown

Conditions notes	Evidence for task performance from Dietz et al. (2016)

Contrasts

Are the contrasts clearly described?	Yes

Contrast 1: verb generation (overt) vs noun repetition

Language condition	Verb generation (overt)
Control condition	Noun repetition
Are the conditions matched for visual demands?	Yes
Are the conditions matched for auditory demands?	Yes
Are the conditions matched for motor demands?	Yes
Are the conditions matched for cognitive/executive demands?	No
Is accuracy matched between the language and control tasks for all relevant groups?	Unknown, not reported
Is reaction time matched between the language and control tasks for all relevant groups?	Unknown, not reported
Behavioral data notes	—
Are control data reported in this paper or another that is referenced?	Yes
Does the contrast selectively activate plausible relevant language regions in the control group?	Somewhat
Are activations lateralized in the control data?	Somewhat
Control activation notes	Control data in Allendorfer et al. (2012); somewhat L-lateralized frontal, temporal and parietal activations, but also extensive midline activation
Contrast notes	—

Analyses

Are the analyses clearly described?	No* (moderate limitation) (see specific limitation(s) below)

ROI analysis 1

First level contrast	Verb generation (overt) vs noun repetition
Analysis class	Cross-sectional between two groups with aphasia
Group(s)	Aphasia with AAC treatment (n = 6) T2 vs usual care T2 (n = 6)
Covariate	—
Is the second level contrast valid in terms of the group(s), time point(s), and measures involved?	Somewhat (marginal treatment effect)
Is accuracy matched across the second level contrast?	Unknown, not reported
Is reaction time matched across the second level contrast?	Unknown, not reported
Behavioral data notes	—
Type of analysis	Region of interest (ROI)
ROI type	Laterality indi(ces)
How many ROIs are there?	1
What are the ROI(s)?	Frontal LI
How are the ROI(s) defined?	
Correction for multiple comparisons	One only
Statistical details	Temporal LI calculated but not reported
Findings	None
Findings notes	—

ROI analysis 2

First level contrast	Verb generation (overt) vs noun repetition
Analysis class	Longitudinal correlation with language or other measure
Group(s)	Aphasia (both groups) T2 vs T1
Covariate	Δ WAB AQ
Is the second level contrast valid in terms of the group(s), time point(s), and measures involved?	Somewhat (gain in AQ not tested for significance)
Is accuracy matched across the second level contrast?	Unknown, not reported
Is reaction time matched across the second level contrast?	Unknown, not reported
Behavioral data notes	—
Type of analysis	Region of interest (ROI)
ROI type	Laterality indi(ces)
How many ROIs are there?	1
What are the ROI(s)?	Frontal LI
How are the ROI(s) defined?	
Correction for multiple comparisons	One only
Statistical details	Temporal LI calculated but not reported
Findings	↑ LI (frontal)
Findings notes	—

Notes

Excluded analyses	(1) pre-treatment comparison between treated and untreated groups; (2) several other analyses based on LI in different ROIs, because there were no inferential statistics

Hallam et al. (2018)

Reference

Authors	Hallam GP, Thompson HE, Hymers M, Millman RE, Rodd JM, Lambon Ralph MA, Smallwood J, Jefferies E
Title	Task-based and resting-state fMRI reveal compensatory network changes following damage to left inferior frontal gyrus
Reference	*Cortex* 2018; 99: 150-165
PMID	29223933
DOI	10.1016/j.cortex.2017.10.004

Participants

Language	UK English
Inclusion criteria	Semantic aphasia; left frontal damage (+ other regions, typically)
Number of individuals with aphasia	14
Number of control participants	16
Were any of the participants included in any previous studies?	No
Is age reported for patients and controls, and matched?	Yes (mean 61 ± 11 years, range 38-80 years)
Is sex reported for patients and controls, and matched?	Yes (males: 5; females: 9)
Is handedness reported for patients and controls, and matched?	No
Is time post stroke onset reported and appropriate to the study design?	Yes (range 11-264 months)
To what extent is the nature of aphasia characterized?	Comprehensive battery

Language evaluation	Cambridge semantic battery, three additional semantic tasks, connected speech words per minute, repetition from PALPA
Aphasia severity	Not stated
Aphasia type	6 anomic, 2 Broca's, 2 global, 2 transcortical sensory, 1 mixed transcortical, 1 not stated
First stroke only?	Not stated
Stroke type	Not stated
To what extent is the lesion distribution characterized?	Lesion overlay
Lesion extent	Not stated
Lesion location	L IFG plus other MCA regions; vATL and pMTG spared
Participants notes	—

Imaging

Modality	fMRI
Is the study cross-sectional or longitudinal?	Cross-sectional
If longitudinal, at what time point(s) were imaging data acquired?	—
If longitudinal, was there any intervention between the time points?	—
Is the scanner described?	Yes (GE Signa HDx 3 Tesla)
Is the timing of stimulus presentation and image acquisition clearly described and appropriate?	Yes
Design type	Event-related
Total images acquired	348
Are the imaging acquisition parameters, including coverage, adequately described and appropriate?	Yes (whole brain)
Is preprocessing and intrasubject coregistration adequately described and appropriate?	Yes
Is first level model fitting adequately described and appropriate?	Yes
Is intersubject normalization adequately described and appropriate?	Yes
Imaging notes	interleaved silent steady state imaging

Conditions

Are the conditions clearly described?	Yes

Condition	Response type	Repetitions	All groups could do?	All individuals could do?
listening to high ambiguity sentences	None	24	N/A	N/A
listening to low ambiguity sentences	None	24	N/A	N/A
listening to spectrally rotated speech	None	24	N/A	N/A
pressing a button to a visual cue	Button press	9	Unknown	Unknown
rest	None	12	N/A	N/A

Conditions notes	All but one patient had good single word comprehension, which was argued to support sentence comprehension

Contrasts

Are the contrasts clearly described?	Yes

Contrast 1: listening to high or low ambiguity sentences vs listening to spectrally rotated speech

Language condition	Listening to high or low ambiguity sentences
Control condition	Listening to spectrally rotated speech
Are the conditions matched for visual demands?	Yes
Are the conditions matched for auditory demands?	Yes

Are the conditions matched for motor demands?	Yes
Are the conditions matched for cognitive/executive demands?	Yes
Is accuracy matched between the language and control tasks for all relevant groups?	N/A, no behavioral measure
Is reaction time matched between the language and control tasks for all relevant groups?	N/A, no timeable task
Behavioral data notes	—
Are control data reported in this paper or another that is referenced?	Somewhat
Does the contrast selectively activate plausible relevant language regions in the control group?	Unknown
Are activations lateralized in the control data?	Unknown
Control activation notes	Hard to evaluate contrast because a "semantic mask" is used but is not described in detail
Contrast notes	—

Contrast 2: listening to high ambiguity sentences vs listening to low ambiguity sentences

Language condition	Listening to high ambiguity sentences
Control condition	Listening to low ambiguity sentences
Are the conditions matched for visual demands?	Yes
Are the conditions matched for auditory demands?	Yes
Are the conditions matched for motor demands?	Yes
Are the conditions matched for cognitive/executive demands?	Yes
Is accuracy matched between the language and control tasks for all relevant groups?	N/A, no behavioral measure
Is reaction time matched between the language and control tasks for all relevant groups?	N/A, no timeable task
Behavioral data notes	—
Are control data reported in this paper or another that is referenced?	No
Does the contrast selectively activate plausible relevant language regions in the control group?	Unknown
Are activations lateralized in the control data?	Unknown
Control activation notes	—
Contrast notes	—

Analyses

Are the analyses clearly described?	Yes

ROI analysis 1

First level contrast	Listening to high or low ambiguity sentences vs listening to spectrally rotated speech
Analysis class	Cross-sectional aphasia vs control
Group(s)	Aphasia vs control
Covariate	—
Is the second level contrast valid in terms of the group(s), time point(s), and measures involved?	Yes
Is accuracy matched across the second level contrast?	N/A, no behavioral measure
Is reaction time matched across the second level contrast?	N/A, no timeable task
Behavioral data notes	—
Type of analysis	Regions of interest (ROI)
ROI type	Functional
How many ROIs are there?	2
What are the ROI(s)?	(1) L vATL; (2) L pMTG

How are the ROI(s) defined?	Functional coordinates in literature
Correction for multiple comparisons	No correction
Statistical details	ANOVA revealed main effect of group (patient vs control), confirmed in follow-up tests for each ROI
Findings	↑ L posterior MTG<br>↑ L anterior temporal
Findings notes	—

ROI analysis 2

First level contrast	Listening to high ambiguity sentences vs listening to low ambiguity sentences
Analysis class	Cross-sectional aphasia vs control
Group(s)	Aphasia vs control
Covariate	—
Is the second level contrast valid in terms of the group(s), time point(s), and measures involved?	Yes
Is accuracy matched across the second level contrast?	N/A, no behavioral measure
Is reaction time matched across the second level contrast?	N/A, no timeable task
Behavioral data notes	—
Type of analysis	Regions of interest (ROI)
ROI type	Functional
How many ROIs are there?	2
What are the ROI(s)?	(1) L vATL; (2) L pMTG
How are the ROI(s) defined?	Functional coordinates in literature
Correction for multiple comparisons	No correction
Statistical details	No interaction of group by condition
Findings	None
Findings notes	—

Complex analysis 1

First level contrast	Listening to high ambiguity sentences vs listening to low ambiguity sentences
Analysis class	Cross-sectional aphasia vs control
Group(s)	Aphasia (subset with resting state data, n = 10) vs control (subset with resting state data, n = 10)
Covariate	—
Is the second level contrast valid in terms of the group(s), time point(s), and measures involved?	Yes
Is accuracy matched across the second level contrast?	N/A, no behavioral measure
Is reaction time matched across the second level contrast?	N/A, no timeable task
Behavioral data notes	—
Type of analysis	Complex
Statistical details	A whole brain analysis was carried out to identify regions where the groups differed in the extent to which the strength of functional connectivity at rest from L pMTG was associated with the difference in signal between the high ambiguity and low ambiguity conditions in the same ROI. Thresholding is not described and cluster extent is not reported.
Findings	Other
Findings notes	There was a functional activation by group interaction in the L aSTG. For controls, there was a positive association between L pMTG activity and functional connectivity to aSTG, while for the patients, there was a negative association.

Complex analysis 2

First level contrast	Listening to high ambiguity sentences vs listening to low ambiguity sentences
Analysis class	Cross-sectional aphasia vs control

Group(s)	Aphasia (subset with resting state data, n = 10) vs control (subset with resting state data, n = 10)
Covariate	—
Is the second level contrast valid in terms of the group(s), time point(s), and measures involved?	Yes
Is accuracy matched across the second level contrast?	N/A, no behavioral measure
Is reaction time matched across the second level contrast?	N/A, no timeable task
Behavioral data notes	—
Type of analysis	Complex
Statistical details	A whole brain analysis was carried out to identify regions where the groups differed in the extent to which the strength of functional connectivity at rest from L pMTG was associated with the difference in signal between the high ambiguity and low ambiguity conditions in the same ROI. Thresholding is not described.
Findings	None
Findings notes	No interaction is reported; both groups showed a correlation between L vATL activity and functional connectivity to a ventral IFG region

Notes

Excluded analyses	Analyses involving resting state data, except for those that also involved task-based data

Nenert et al. (2018)

Reference

Authors	Nenert R, Allendorfer JB, Martin AM, Banks C, Vannest J, Holland SK, Hart KW, Lindsell CJ, Szaflarski JP
Title	Longitudinal fMRI study of language recovery after a left hemispheric ischemic stroke
Reference	*Restor Neurol Neurosci* 2018; 36: 359-385
PMID	29782329
DOI	10.3233/rnn-170767

Participants

Language	US English
Inclusion criteria	Aphasia at acute screening (not necessarily at first study time point)
Number of individuals with aphasia	17 (plus 1 excluded: significant signal artifacts)
Number of control participants	85
Were any of the participants included in any previous studies?	No
Is age reported for patients and controls, and matched?	Yes (mean 46 ± 16 years)
Is sex reported for patients and controls, and matched?	Yes (males: 9; females: 8)
Is handedness reported for patients and controls, and matched?	No (right: 17; left: 0; all patients stated to be right handed, but "ambidextrous patients" mentioned on p. 364)
Is time post stroke onset reported and appropriate to the study design?	Yes (T1: ~2 weeks; T2: ~6 weeks; T3: ~12 weeks; T4: ~26 weeks; T5: ~52 weeks)
To what extent is the nature of aphasia characterized?	Not at all
Language evaluation	PPVT, BNT, phonemic fluency, semantic fluency, complex ideation subtest of BDAE
Aphasia severity	Not stated for study timepoints, but on admission, aphasia severity was assessed with the TT: 2 no aphasia per cutoff but clinical impression of aphasia, 5 mild, 6 moderate, 4 severe
Aphasia type	Not stated
First stroke only?	No

Stroke type	Ischemic only
To what extent is the lesion distribution characterized?	Lesion overlay
Lesion extent	Not stated
Lesion location	L MCA; mostly posterior per Supplementary Figure 2
Participants notes	Presence and severity of aphasia assessed on hospital admission, not at first study time point, so it is not clear that all participants actually had aphasia at first study time point

Imaging

Modality	fMRI
Is the study cross-sectional or longitudinal?	Longitudinal—recovery
If longitudinal, at what time point(s) were imaging data acquired?	T1: ~2 weeks; T2: ~6 weeks; T3: ~12 weeks; T4: ~26 weeks; T5: ~52 weeks
If longitudinal, was there any intervention between the time points?	Not stated
Is the scanner described?	No (Philips 3 Tesla or Siemens 3 Tesla; models not stated)
Is the timing of stimulus presentation and image acquisition clearly described and appropriate?	Yes
Design type	Block
Total images acquired	600
Are the imaging acquisition parameters, including coverage, adequately described and appropriate?	Yes (whole brain)
Is preprocessing and intrasubject coregistration adequately described and appropriate?	Yes
Is first level model fitting adequately described and appropriate?	Yes
Is intersubject normalization adequately described and appropriate?	No (lesion impact not addressed)
Imaging notes	scanner identity appropriately included as covariate

Conditions

Are the conditions clearly described?	Yes

Condition	Response type	Repetitions	All groups could do?	All individuals could do?
semantic decision	Button press	5	No	No
tone decision	Button press	5	Yes	Unknown
verb generation	Multiple words (covert)	5	Unknown	Unknown
finger tapping	Other	5	Unknown	Unknown

Conditions notes	Assume semantic decision is out of 25, so chance is 12.5 and 95% CI below chance at T2; post-scan recognition test for verb generation not considered to quantify task performance

Contrasts

Are the contrasts clearly described?	Yes

Contrast 1: semantic decision vs tone decision

Language condition	Semantic decision
Control condition	Tone decision
Are the conditions matched for visual demands?	Yes
Are the conditions matched for auditory demands?	Yes
Are the conditions matched for motor demands?	Yes
Are the conditions matched for cognitive/executive demands?	Yes
Is accuracy matched between the language and control tasks for all relevant groups?	Appear mismatched
Is reaction time matched between the language	Unknown, not reported

and control tasks for all relevant groups?	
Behavioral data notes	—
Are control data reported in this paper or another that is referenced?	Yes
Does the contrast selectively activate plausible relevant language regions in the control group?	Yes
Are activations lateralized in the control data?	Yes
Control activation notes	L lateral and medial frontal and AG, strongly lateralized
Contrast notes	—

Contrast 2: verb generation vs finger tapping

Language condition	Verb generation
Control condition	Finger tapping
Are the conditions matched for visual demands?	Yes
Are the conditions matched for auditory demands?	Yes
Are the conditions matched for motor demands?	No
Are the conditions matched for cognitive/executive demands?	No
Is accuracy matched between the language and control tasks for all relevant groups?	N/A, tasks not comparable
Is reaction time matched between the language and control tasks for all relevant groups?	N/A, tasks not comparable
Behavioral data notes	—
Are control data reported in this paper or another that is referenced?	Yes
Does the contrast selectively activate plausible relevant language regions in the control group?	Yes
Are activations lateralized in the control data?	Yes
Control activation notes	L lateral and medial frontal and mid temporal, strongly lateralized
Contrast notes	—

Analyses

Are the analyses clearly described?	No** (major limitation) (see specific limitation(s) below)

Voxelwise analysis 1

First level contrast	Semantic decision vs tone decision
Analysis class	Cross-sectional aphasia vs control
Group(s)	Aphasia T1 vs control
Covariate	—
Is the second level contrast valid in terms of the group(s), time point(s), and measures involved?	Yes
Is accuracy matched across the second level contrast?	Appear mismatched
Is reaction time matched across the second level contrast?	Unknown, not reported
Behavioral data notes	Patients less accurate than controls on both tasks, but more so on the tone decision task
Type of analysis	Voxelwise
Search volume	Whole brain
Correction for multiple comparisons	Voxelwise correction based on permutation testing
Software	SPM12/SnPM13
Voxelwise p	FWE p < .05
Cluster extent	—
Statistical details	—
Findings	↑ L Heschl's gyrus
Findings notes	—

Voxelwise analysis 2

First level contrast	Semantic decision vs tone decision
Analysis class	Cross-sectional aphasia vs control
Group(s)	Aphasia T2 vs control
Covariate	—
Is the second level contrast valid in terms of the group(s), time point(s), and measures involved?	Yes
Is accuracy matched across the second level contrast?	Appear mismatched
Is reaction time matched across the second level contrast?	Unknown, not reported
Behavioral data notes	Patients less accurate than controls on both tasks, but more so on the tone decision task
Type of analysis	Voxelwise
Search volume	Whole brain
Correction for multiple comparisons	Voxelwise correction based on permutation testing
Software	SPM12/SnPM13
Voxelwise p	FWE p < .05
Cluster extent	—
Statistical details	—
Findings	None
Findings notes	—

Voxelwise analysis 3

First level contrast	Semantic decision vs tone decision
Analysis class	Cross-sectional aphasia vs control
Group(s)	Aphasia T3 vs control
Covariate	—
Is the second level contrast valid in terms of the group(s), time point(s), and measures involved?	Yes
Is accuracy matched across the second level contrast?	Appear mismatched
Is reaction time matched across the second level contrast?	Unknown, not reported
Behavioral data notes	Patients less accurate than controls on both tasks, but more so on the tone decision task
Type of analysis	Voxelwise
Search volume	Whole brain
Correction for multiple comparisons	Voxelwise correction based on permutation testing
Software	SPM12/SnPM13
Voxelwise p	FWE p < .05
Cluster extent	—
Statistical details	—
Findings	None
Findings notes	—

Voxelwise analysis 4

First level contrast	Semantic decision vs tone decision
Analysis class	Cross-sectional aphasia vs control
Group(s)	Aphasia T4 vs control
Covariate	—
Is the second level contrast valid in terms of the group(s), time point(s), and measures involved?	Yes
Is accuracy matched across the second level contrast?	Appear mismatched
Is reaction time matched across the second level contrast?	Unknown, not reported

Behavioral data notes	Patients less accurate than controls on both tasks, but more so on the tone decision task
Type of analysis	Voxelwise
Search volume	Whole brain
Correction for multiple comparisons	Voxelwise correction based on permutation testing
Software	SPM12/SnPM13
Voxelwise p	FWE p < .05
Cluster extent	—
Statistical details	—
Findings	None
Findings notes	—

Voxelwise analysis 5

First level contrast	Semantic decision vs tone decision
Analysis class	Cross-sectional aphasia vs control
Group(s)	Aphasia T5 vs control
Covariate	—
Is the second level contrast valid in terms of the group(s), time point(s), and measures involved?	Yes
Is accuracy matched across the second level contrast?	Appear mismatched
Is reaction time matched across the second level contrast?	Unknown, not reported
Behavioral data notes	Patients less accurate than controls on both tasks, but more so on the tone decision task
Type of analysis	Voxelwise
Search volume	Whole brain
Correction for multiple comparisons	Voxelwise correction based on permutation testing
Software	SPM12/SnPM13
Voxelwise p	FWE p < .05
Cluster extent	—
Statistical details	—
Findings	None
Findings notes	—

Voxelwise analysis 6

First level contrast	Verb generation vs finger tapping
Analysis class	Cross-sectional aphasia vs control
Group(s)	Aphasia T1 vs control
Covariate	—
Is the second level contrast valid in terms of the group(s), time point(s), and measures involved?	Yes
Is accuracy matched across the second level contrast?	Unknown, not reported
Is reaction time matched across the second level contrast?	Unknown, not reported
Behavioral data notes	—
Type of analysis	Voxelwise
Search volume	Whole brain
Correction for multiple comparisons	Voxelwise correction based on permutation testing
Software	SPM12/SnPM13
Voxelwise p	FWE p < .05
Cluster extent	—
Statistical details	—
Findings	None
Findings notes	—

Voxelwise analysis 7

First level contrast	Verb generation vs finger tapping
Analysis class	Cross-sectional aphasia vs control
Group(s)	Aphasia T2 vs control
Covariate	—
Is the second level contrast valid in terms of the group(s), time point(s), and measures involved?	Yes
Is accuracy matched across the second level contrast?	Unknown, not reported
Is reaction time matched across the second level contrast?	Unknown, not reported
Behavioral data notes	—
Type of analysis	Voxelwise
Search volume	Whole brain
Correction for multiple comparisons	Voxelwise correction based on permutation testing
Software	SPM12/SnPM13
Voxelwise p	FWE p < .05
Cluster extent	—
Statistical details	—
Findings	None
Findings notes	—

Voxelwise analysis 8

First level contrast	Verb generation vs finger tapping
Analysis class	Cross-sectional aphasia vs control
Group(s)	Aphasia T3 vs control
Covariate	—
Is the second level contrast valid in terms of the group(s), time point(s), and measures involved?	Yes
Is accuracy matched across the second level contrast?	Unknown, not reported
Is reaction time matched across the second level contrast?	Unknown, not reported
Behavioral data notes	—
Type of analysis	Voxelwise
Search volume	Whole brain
Correction for multiple comparisons	Voxelwise correction based on permutation testing
Software	SPM12/SnPM13
Voxelwise p	FWE p < .05
Cluster extent	—
Statistical details	—
Findings	None
Findings notes	—

Voxelwise analysis 9

First level contrast	Verb generation vs finger tapping
Analysis class	Cross-sectional aphasia vs control
Group(s)	Aphasia T4 vs control
Covariate	—
Is the second level contrast valid in terms of the group(s), time point(s), and measures involved?	Yes
Is accuracy matched across the second level contrast?	Unknown, not reported
Is reaction time matched across the second level contrast?	Unknown, not reported

Behavioral data notes	—
Type of analysis	Voxelwise
Search volume	Whole brain
Correction for multiple comparisons	Voxelwise correction based on permutation testing
Software	SPM12/SnPM13
Voxelwise p	FWE p < .05
Cluster extent	—
Statistical details	—
Findings	None
Findings notes	—

Voxelwise analysis 10

First level contrast	Verb generation vs finger tapping
Analysis class	Cross-sectional aphasia vs control
Group(s)	Aphasia T5 vs control
Covariate	—
Is the second level contrast valid in terms of the group(s), time point(s), and measures involved?	Yes
Is accuracy matched across the second level contrast?	Unknown, not reported
Is reaction time matched across the second level contrast?	Unknown, not reported
Behavioral data notes	—
Type of analysis	Voxelwise
Search volume	Whole brain
Correction for multiple comparisons	Voxelwise correction based on permutation testing
Software	SPM12/SnPM13
Voxelwise p	FWE p < .05
Cluster extent	—
Statistical details	—
Findings	None
Findings notes	—

Voxelwise analysis 11

First level contrast	Semantic decision vs tone decision
Analysis class	Cross-sectional correlation with language or other measure
Group(s)	Aphasia T1
Covariate	Semantic decision accuracy
Is the second level contrast valid in terms of the group(s), time point(s), and measures involved?	Yes
Is accuracy matched across the second level contrast?	Accuracy is covariate
Is reaction time matched across the second level contrast?	Unknown, not reported
Behavioral data notes	—
Type of analysis	Voxelwise
Search volume	Whole brain
Correction for multiple comparisons	Voxelwise correction based on permutation testing
Software	SPM12/SnPM13
Voxelwise p	FWE p < .05
Cluster extent	—
Statistical details	—
Findings	↑ L anterior temporal
Findings notes	Unclear why this type of analysis was run only for semantic task, and only at T1

Voxelwise analysis 12

First level contrast	Semantic decision vs tone decision
Analysis class	Longitudinal correlation with language or other measure
Group(s)	Aphasia T4 vs aphasia T1
Covariate	Δ BNT
Is the second level contrast valid in terms of the group(s), time point(s), and measures involved?	Yes
Is accuracy matched across the second level contrast?	Unknown, not reported
Is reaction time matched across the second level contrast?	Unknown, not reported
Behavioral data notes	—
Type of analysis	Voxelwise
Search volume	Whole brain
Correction for multiple comparisons	Voxelwise correction based on permutation testing
Software	SPM12/SnPM13
Voxelwise p	FWE p < .05
Cluster extent	—
Statistical details	—
Findings	None
Findings notes	—

Voxelwise analysis 13

First level contrast	Semantic decision vs tone decision
Analysis class	Longitudinal correlation with language or other measure
Group(s)	Aphasia T4 vs aphasia T1
Covariate	Δ semantic fluency
Is the second level contrast valid in terms of the group(s), time point(s), and measures involved?	Yes
Is accuracy matched across the second level contrast?	Unknown, not reported
Is reaction time matched across the second level contrast?	Unknown, not reported
Behavioral data notes	—
Type of analysis	Voxelwise
Search volume	Whole brain
Correction for multiple comparisons	Voxelwise correction based on permutation testing
Software	SPM12/SnPM13
Voxelwise p	FWE p < .05
Cluster extent	—
Statistical details	—
Findings	None
Findings notes	—

Voxelwise analysis 14

First level contrast	Semantic decision vs tone decision
Analysis class	Longitudinal correlation with language or other measure
Group(s)	Aphasia T4 vs aphasia T1
Covariate	Δ PPVT
Is the second level contrast valid in terms of the group(s), time point(s), and measures involved?	Yes
Is accuracy matched across the second level contrast?	Unknown, not reported
Is reaction time matched across the second level contrast?	Unknown, not reported

Behavioral data notes	—
Type of analysis	Voxelwise
Search volume	Whole brain
Correction for multiple comparisons	Voxelwise correction based on permutation testing
Software	SPM12/SnPM13
Voxelwise p	FWE p < .05
Cluster extent	—
Statistical details	—
Findings	None
Findings notes	—

Voxelwise analysis 15

First level contrast	Semantic decision vs tone decision
Analysis class	Longitudinal correlation with language or other measure
Group(s)	Aphasia T4 vs aphasia T1
Covariate	Δ phonemic fluency
Is the second level contrast valid in terms of the group(s), time point(s), and measures involved?	Yes
Is accuracy matched across the second level contrast?	Unknown, not reported
Is reaction time matched across the second level contrast?	Unknown, not reported
Behavioral data notes	—
Type of analysis	Voxelwise
Search volume	Whole brain
Correction for multiple comparisons	Voxelwise correction based on permutation testing
Software	SPM12/SnPM13
Voxelwise p	FWE p < .05
Cluster extent	—
Statistical details	—
Findings	None
Findings notes	—

Voxelwise analysis 16

First level contrast	Semantic decision vs tone decision
Analysis class	Longitudinal correlation with language or other measure
Group(s)	Aphasia T4 vs aphasia T1
Covariate	Δ BDAE complex ideation subtest
Is the second level contrast valid in terms of the group(s), time point(s), and measures involved?	Yes
Is accuracy matched across the second level contrast?	Unknown, not reported
Is reaction time matched across the second level contrast?	Unknown, not reported
Behavioral data notes	—
Type of analysis	Voxelwise
Search volume	Whole brain
Correction for multiple comparisons	Voxelwise correction based on permutation testing
Software	SPM12/SnPM13
Voxelwise p	FWE p < .05
Cluster extent	—
Statistical details	—
Findings	None
Findings notes	—

Voxelwise analysis 17

First level contrast	Verb generation vs finger tapping
Analysis class	Longitudinal correlation with language or other measure
Group(s)	Aphasia T4 vs aphasia T1
Covariate	Δ BNT
Is the second level contrast valid in terms of the group(s), time point(s), and measures involved?	Yes
Is accuracy matched across the second level contrast?	Unknown, not reported
Is reaction time matched across the second level contrast?	Unknown, not reported
Behavioral data notes	—
Type of analysis	Voxelwise
Search volume	Whole brain
Correction for multiple comparisons	Voxelwise correction based on permutation testing
Software	SPM12/SnPM13
Voxelwise p	FWE p < .05
Cluster extent	—
Statistical details	—
Findings	None
Findings notes	—

Voxelwise analysis 18

First level contrast	Verb generation vs finger tapping
Analysis class	Longitudinal correlation with language or other measure
Group(s)	Aphasia T4 vs aphasia T1
Covariate	Δ semantic fluency
Is the second level contrast valid in terms of the group(s), time point(s), and measures involved?	Yes
Is accuracy matched across the second level contrast?	Unknown, not reported
Is reaction time matched across the second level contrast?	Unknown, not reported
Behavioral data notes	—
Type of analysis	Voxelwise
Search volume	Whole brain
Correction for multiple comparisons	Voxelwise correction based on permutation testing
Software	SPM12/SnPM13
Voxelwise p	FWE p < .05
Cluster extent	—
Statistical details	—
Findings	↑ L dorsolateral prefrontal cortex<br>↑ L SMA/medial prefrontal<br>↑ R somato-motor<br>↑ R anterior temporal
Findings notes	—

Voxelwise analysis 19

First level contrast	Verb generation vs finger tapping
Analysis class	Longitudinal correlation with language or other measure
Group(s)	Aphasia T4 vs aphasia T1
Covariate	Δ PPVT
Is the second level contrast valid in terms of the group(s), time point(s), and measures involved?	Yes
Is accuracy matched across the second level	Unknown, not reported

contrast?	
Is reaction time matched across the second level contrast?	Unknown, not reported
Behavioral data notes	—
Type of analysis	Voxelwise
Search volume	Whole brain
Correction for multiple comparisons	Voxelwise correction based on permutation testing
Software	SPM12/SnPM13
Voxelwise p	FWE p < .05
Cluster extent	—
Statistical details	—
Findings	None
Findings notes	—

Voxelwise analysis 20

First level contrast	Verb generation vs finger tapping
Analysis class	Longitudinal correlation with language or other measure
Group(s)	Aphasia T4 vs aphasia T1
Covariate	Δ phonemic fluency
Is the second level contrast valid in terms of the group(s), time point(s), and measures involved?	Yes
Is accuracy matched across the second level contrast?	Unknown, not reported
Is reaction time matched across the second level contrast?	Unknown, not reported
Behavioral data notes	—
Type of analysis	Voxelwise
Search volume	Whole brain
Correction for multiple comparisons	Voxelwise correction based on permutation testing
Software	SPM12/SnPM13
Voxelwise p	FWE p < .05
Cluster extent	—
Statistical details	—
Findings	↑ L cerebellum
Findings notes	—

Voxelwise analysis 21

First level contrast	Verb generation vs finger tapping
Analysis class	Longitudinal correlation with language or other measure
Group(s)	Aphasia T4 vs aphasia T1
Covariate	Δ BDAE complex ideation subtest
Is the second level contrast valid in terms of the group(s), time point(s), and measures involved?	Yes
Is accuracy matched across the second level contrast?	Unknown, not reported
Is reaction time matched across the second level contrast?	Unknown, not reported
Behavioral data notes	—
Type of analysis	Voxelwise
Search volume	Whole brain
Correction for multiple comparisons	Voxelwise correction based on permutation testing
Software	SPM12/SnPM13
Voxelwise p	FWE p < .05
Cluster extent	—
Statistical details	—

Findings	None
Findings notes	—

ROI analysis 1

First level contrast	Semantic decision vs tone decision
Analysis class	Longitudinal change in aphasia
Group(s)	Aphasia (comparisons between all pairs of time points)
Covariate	—
Is the second level contrast valid in terms of the group(s), time point(s), and measures involved?	Yes
Is accuracy matched across the second level contrast?	Appear similar
Is reaction time matched across the second level contrast?	Unknown, not reported
Behavioral data notes	—
Type of analysis	Regions of interest (ROI)
ROI type	Laterality indi(ces)
How many ROIs are there?	4
What are the ROI(s)?	(1) frontal LI; (2) temporo-parietal LI; (3) language network LI; (4) cerebellar LI
How are the ROI(s) defined?	
Correction for multiple comparisons	No correction
Statistical details	—
Findings	None
Findings notes	—

ROI analysis 2

First level contrast	Verb generation vs finger tapping
Analysis class	Longitudinal change in aphasia
Group(s)	Aphasia (comparisons between all pairs of time points)
Covariate	—
Is the second level contrast valid in terms of the group(s), time point(s), and measures involved?	Yes
Is accuracy matched across the second level contrast?	Unknown, not reported
Is reaction time matched across the second level contrast?	Unknown, not reported
Behavioral data notes	—
Type of analysis	Regions of interest (ROI)
ROI type	Laterality indi(ces)
How many ROIs are there?	4
What are the ROI(s)?	(1) frontal LI; (2) temporo-parietal LI; (3) language network LI; (4) cerebellar LI
How are the ROI(s) defined?	
Correction for multiple comparisons	No correction
Statistical details	—
Findings	None
Findings notes	—

ROI analysis 3

First level contrast	Semantic decision vs tone decision
Analysis class	Cross-sectional aphasia vs control
Group(s)	Aphasia T1 vs control
Covariate	—
Is the second level contrast valid in terms of the group(s), time point(s), and measures involved?	Yes
Is accuracy matched across the second level	Appear mismatched

contrast?	
Is reaction time matched across the second level contrast?	Unknown, not reported
Behavioral data notes	Patients less accurate than controls on both tasks, but more so on the tone decision task
Type of analysis	Regions of interest (ROI)
ROI type	Laterality indi(ces)
How many ROIs are there?	4
What are the ROI(s)?	(1) frontal LI; (2) temporo-parietal LI; (3) language network LI; (4) cerebellar LI
How are the ROI(s) defined?	
Correction for multiple comparisons	No correction
Statistical details	—
Findings	None
Findings notes	—

ROI analysis 4

First level contrast	Semantic decision vs tone decision
Analysis class	Cross-sectional aphasia vs control
Group(s)	Aphasia T2 vs control
Covariate	—
Is the second level contrast valid in terms of the group(s), time point(s), and measures involved?	Yes
Is accuracy matched across the second level contrast?	Appear mismatched
Is reaction time matched across the second level contrast?	Unknown, not reported
Behavioral data notes	Patients less accurate than controls on both tasks, but more so on the tone decision task
Type of analysis	Regions of interest (ROI)
ROI type	Laterality indi(ces)
How many ROIs are there?	4
What are the ROI(s)?	(1) frontal LI; (2) temporo-parietal LI; (3) language network LI; (4) cerebellar LI
How are the ROI(s) defined?	
Correction for multiple comparisons	No correction
Statistical details	—
Findings	None
Findings notes	—

ROI analysis 5

First level contrast	Semantic decision vs tone decision
Analysis class	Cross-sectional aphasia vs control
Group(s)	Aphasia T3 vs control
Covariate	—
Is the second level contrast valid in terms of the group(s), time point(s), and measures involved?	Yes
Is accuracy matched across the second level contrast?	Appear mismatched
Is reaction time matched across the second level contrast?	Unknown, not reported
Behavioral data notes	Patients less accurate than controls on both tasks, but more so on the tone decision task
Type of analysis	Regions of interest (ROI)
ROI type	Laterality indi(ces)
How many ROIs are there?	4
What are the ROI(s)?	(1) frontal LI; (2) temporo-parietal LI; (3) language network LI; (4) cerebellar LI
How are the ROI(s) defined?	
Correction for multiple comparisons	No correction
Statistical details	—

Findings	None
Findings notes	—

ROI analysis 6

First level contrast	Semantic decision vs tone decision
Analysis class	Cross-sectional aphasia vs control
Group(s)	Aphasia T4 vs control
Covariate	—
Is the second level contrast valid in terms of the group(s), time point(s), and measures involved?	Yes
Is accuracy matched across the second level contrast?	Appear mismatched
Is reaction time matched across the second level contrast?	Unknown, not reported
Behavioral data notes	Patients less accurate than controls on both tasks, but more so on the tone decision task
Type of analysis	Regions of interest (ROI)
ROI type	Laterality indi(ces)
How many ROIs are there?	4
What are the ROI(s)?	(1) frontal LI; (2) temporo-parietal LI; (3) language network LI; (4) cerebellar LI
How are the ROI(s) defined?	
Correction for multiple comparisons	No correction
Statistical details	—
Findings	None
Findings notes	—

ROI analysis 7

First level contrast	Semantic decision vs tone decision
Analysis class	Cross-sectional aphasia vs control
Group(s)	Aphasia T5 vs control
Covariate	—
Is the second level contrast valid in terms of the group(s), time point(s), and measures involved?	Yes
Is accuracy matched across the second level contrast?	Appear mismatched
Is reaction time matched across the second level contrast?	Unknown, not reported
Behavioral data notes	Patients less accurate than controls on both tasks, but more so on the tone decision task
Type of analysis	Regions of interest (ROI)
ROI type	Laterality indi(ces)
How many ROIs are there?	4
What are the ROI(s)?	(1) frontal LI; (2) temporo-parietal LI; (3) language network LI; (4) cerebellar LI
How are the ROI(s) defined?	
Correction for multiple comparisons	No correction
Statistical details	—
Findings	None
Findings notes	—

ROI analysis 8

First level contrast	Verb generation vs finger tapping
Analysis class	Cross-sectional aphasia vs control
Group(s)	Aphasia T1 vs control
Covariate	—
Is the second level contrast valid in terms of the group(s), time point(s), and measures involved?	Yes
Is accuracy matched across the second level	Unknown, not reported

contrast?	
Is reaction time matched across the second level contrast?	Unknown, not reported
Behavioral data notes	—
Type of analysis	Regions of interest (ROI)
ROI type	Laterality indi(ces)
How many ROIs are there?	4
What are the ROI(s)?	(1) frontal LI; (2) temporo-parietal LI; (3) language network LI; (4) cerebellar LI
How are the ROI(s) defined?	
Correction for multiple comparisons	No correction
Statistical details	—
Findings	None
Findings notes	—

ROI analysis 9

First level contrast	Verb generation vs finger tapping
Analysis class	Cross-sectional aphasia vs control
Group(s)	Aphasia T2 vs control
Covariate	—
Is the second level contrast valid in terms of the group(s), time point(s), and measures involved?	Yes
Is accuracy matched across the second level contrast?	Unknown, not reported
Is reaction time matched across the second level contrast?	Unknown, not reported
Behavioral data notes	—
Type of analysis	Regions of interest (ROI)
ROI type	Laterality indi(ces)
How many ROIs are there?	4
What are the ROI(s)?	(1) frontal LI; (2) temporo-parietal LI; (3) language network LI; (4) cerebellar LI
How are the ROI(s) defined?	
Correction for multiple comparisons	No correction
Statistical details	—
Findings	↓ LI (language network)<br>↓ LI (frontal)
Findings notes	—

ROI analysis 10

First level contrast	Verb generation vs finger tapping
Analysis class	Cross-sectional aphasia vs control
Group(s)	Aphasia T3 vs control
Covariate	—
Is the second level contrast valid in terms of the group(s), time point(s), and measures involved?	Yes
Is accuracy matched across the second level contrast?	Unknown, not reported
Is reaction time matched across the second level contrast?	Unknown, not reported
Behavioral data notes	—
Type of analysis	Regions of interest (ROI)
ROI type	Laterality indi(ces)
How many ROIs are there?	4
What are the ROI(s)?	(1) frontal LI; (2) temporo-parietal LI; (3) language network LI; (4) cerebellar LI
How are the ROI(s) defined?	
Correction for multiple comparisons	No correction

Statistical details	—
Findings	↓ LI (language network)<br>↓ LI (frontal)
Findings notes	—

ROI analysis 11

First level contrast	Verb generation vs finger tapping
Analysis class	Cross-sectional aphasia vs control
Group(s)	Aphasia T4 vs control
Covariate	—
Is the second level contrast valid in terms of the group(s), time point(s), and measures involved?	Yes
Is accuracy matched across the second level contrast?	Unknown, not reported
Is reaction time matched across the second level contrast?	Unknown, not reported
Behavioral data notes	—
Type of analysis	Regions of interest (ROI)
ROI type	Laterality indi(ces)
How many ROIs are there?	4
What are the ROI(s)?	(1) frontal LI; (2) temporo-parietal LI; (3) language network LI; (4) cerebellar LI
How are the ROI(s) defined?	
Correction for multiple comparisons	No correction
Statistical details	—
Findings	None
Findings notes	—

ROI analysis 12

First level contrast	Verb generation vs finger tapping
Analysis class	Cross-sectional aphasia vs control
Group(s)	Aphasia T5 vs control
Covariate	—
Is the second level contrast valid in terms of the group(s), time point(s), and measures involved?	Yes
Is accuracy matched across the second level contrast?	Unknown, not reported
Is reaction time matched across the second level contrast?	Unknown, not reported
Behavioral data notes	—
Type of analysis	Regions of interest (ROI)
ROI type	Laterality indi(ces)
How many ROIs are there?	4
What are the ROI(s)?	(1) frontal LI; (2) temporo-parietal LI; (3) language network LI; (4) cerebellar LI
How are the ROI(s) defined?	
Correction for multiple comparisons	No correction
Statistical details	—
Findings	None
Findings notes	—

Complex analysis 1

First level contrast	Semantic decision vs tone decision
Analysis class	Longitudinal change in aphasia
Group(s)	Aphasia (comparisons between all pairs of time points)
Covariate	—
Is the second level contrast valid in terms of the	Yes

group(s), time point(s), and measures involved?	
Is accuracy matched across the second level contrast?	Appear similar
Is reaction time matched across the second level contrast?	Unknown, not reported
Behavioral data notes	—
Type of analysis	Complex
Statistical details	PPI analyses were carried out to investigate potential changes over time in how connectivity from L and R IFG was modulated by the semantic decision task. The resultant SPM was thresholded at FWE p < .05 using permutation testing implemented in SnPM 13.
Findings	None
Findings notes	—

Complex analysis 2

First level contrast	Verb generation vs finger tapping
Analysis class	Longitudinal change in aphasia
Group(s)	Aphasia (comparisons between all pairs of time points)
Covariate	—
Is the second level contrast valid in terms of the group(s), time point(s), and measures involved?	Yes
Is accuracy matched across the second level contrast?	Unknown, not reported
Is reaction time matched across the second level contrast?	Unknown, not reported
Behavioral data notes	—
Type of analysis	Complex
Statistical details	PPI analyses were carried out to investigate potential changes over time in how connectivity from L and R IFG was modulated by the verb generation task. The resultant SPM was thresholded at FWE p < .05 using permutation testing implemented in SnPM 13.
Findings	None
Findings notes	—

Notes

Excluded analyses	Longitudinal analyses in people with aphasia, because of contradictory and unclear reporting of findings

Pillay et al. (2018)

Reference

Authors	Pillay SB, Gross WL, Graves WW, Humphries C, Book DS, Binder JR
Title	The neural basis of successful word reading in aphasia
Reference	*J Cogn Neurosci* 2018; 30: 514-525
PMID	29211656
DOI	10.1162/jocn_a_01214

Participants

Language	US English
Inclusion criteria	Residual phonologic retrieval deficit; intact semantic processing
Number of individuals with aphasia	21
Number of control participants	0
Were any of the participants included in any previous studies?	No
Is age reported for patients and controls, and	Yes (mean 56.4 ± 12.5 years, range 30-80 years)

matched?	
Is sex reported for patients and controls, and matched?	Yes (males: 11; females: 10)
Is handedness reported for patients and controls, and matched?	Yes (right: 21; left: 0)
Is time post stroke onset reported and appropriate to the study design?	Yes (mean 1134 ± 1491 days, range 180-6732 days)
To what extent is the nature of aphasia characterized?	Not at all
Language evaluation	Pseudoword rhyme matching, semantic picture matching (similar to PPT-P), picture naming
Aphasia severity	Not stated
Aphasia type	Not stated
First stroke only?	Not stated
Stroke type	Ischemic only
To what extent is the lesion distribution characterized?	Lesion overlay
Lesion extent	Mean 73.4 ± 58.6 cc, range 6.7-227.0 cc
Lesion location	17 L MCA, 2 combined L MCA/ACA, combined 2 L MCA/PCA
Participants notes	—

Imaging

Modality	fMRI
Is the study cross-sectional or longitudinal?	Cross-sectional
If longitudinal, at what time point(s) were imaging data acquired?	—
If longitudinal, was there any intervention between the time points?	—
Is the scanner described?	Yes (GE Excite 3 Tesla)
Is the timing of stimulus presentation and image acquisition clearly described and appropriate?	No (precise timing of stimuli not stated; total images acquired not stated)
Design type	Event-related
Total images acquired	not stated
Are the imaging acquisition parameters, including coverage, adequately described and appropriate?	Yes (whole brain)
Is preprocessing and intrasubject coregistration adequately described and appropriate?	Yes
Is first level model fitting adequately described and appropriate?	Yes
Is intersubject normalization adequately described and appropriate?	Yes
Imaging notes	—

Conditions

Are the conditions clearly described?	Yes

Condition	Response type	Repetitions	All groups could do?	All individuals could do?
reading nouns aloud	Word (overt)	72	Yes	No
rest	None	implicit baseline	N/A	N/A

Conditions notes	Some participants had < 10% accuracy, but this is appropriately addressed in the analysis

Contrasts

Are the contrasts clearly described?	Yes

Contrast 1: reading nouns aloud (correct trials) vs reading nouns aloud (incorrect trials)

Language condition	Reading nouns aloud (correct trials)
Control condition	Reading nouns aloud (incorrect trials)
Are the conditions matched for visual demands?	Yes
Are the conditions matched for auditory demands?	Yes
Are the conditions matched for motor demands?	Yes
Are the conditions matched for cognitive/executive demands?	Yes
Is accuracy matched between the language and control tasks for all relevant groups?	No, by design
Is reaction time matched between the language and control tasks for all relevant groups?	Yes, matched
Behavioral data notes	—
Are control data reported in this paper or another that is referenced?	N/A
Does the contrast selectively activate plausible relevant language regions in the control group?	N/A
Are activations lateralized in the control data?	N/A
Control activation notes	Control data N/A because controls do not typically make errors
Contrast notes	—

Analyses

Are the analyses clearly described?	Yes

Voxelwise analysis 1

First level contrast	Reading nouns aloud (correct trials) vs reading nouns aloud (incorrect trials)
Analysis class	Cross-sectional performance-defined conditions
Group(s)	Aphasia
Covariate	—
Is the second level contrast valid in terms of the group(s), time point(s), and measures involved?	Yes
Is accuracy matched across the second level contrast?	No, by design
Is reaction time matched across the second level contrast?	Yes, matched
Behavioral data notes	—
Type of analysis	Voxelwise
Search volume	Whole brain
Correction for multiple comparisons	Clusterwise correction based on 3dClustSim
Software	AFNI
Voxelwise p	.01
Cluster extent	1.609 cc
Statistical details	Regarding correction for multiple comparisons, addition of monoexponential function reduces but does not eliminate inflation of p values (Cox et al., 2017)
Findings	↑ L angular gyrus<br>↓ L ventral precentral/inferior frontal junction<br>↓ L SMA/medial prefrontal<br>↓ R insula<br>↓ R ventral precentral/inferior frontal junction<br>↓ R SMA/medial prefrontal
Findings notes	Positive region (L AG) was part of the semantic network, while many negative regions were positively modulated by reaction time in the aphasia group

Notes

Excluded analyses	(1) ancillary analysis in which similar findings were obtained when phonological impairment was included as a covariate; (2) ancillary analysis in which similar findings were obtained when lesioned patients were excluded at each voxel; (3) analysis of modulation by reaction time (while informative, this analysis does not meet our inclusion criteria)

Szaflarski et al. (2018)

Reference

Authors	Szaflarski JP, Griffis J, Vannest J, Allendorfer JB, Nenert R, Amara AW, Sung V, Walker HC, Martin AN, Mark VW, Zhou X
Title	A feasibility study of combined intermittent theta burst stimulation and modified constraint-induced aphasia therapy in chronic post-stroke aphasia
Reference	*Restor Neurol Neurosci* 2018; 36: 503-518
PMID	29889086
DOI	10.3233/rnn-180812

Participants

Language	US English
Inclusion criteria	—
Number of individuals with aphasia	12 (plus 1 excluded: scanned at only 2 out of 3 time points)
Number of control participants	0
Were any of the participants included in any previous studies?	No
Is age reported for patients and controls, and matched?	Yes (range 26-66 years)
Is sex reported for patients and controls, and matched?	Yes (males: 9; females: 3)
Is handedness reported for patients and controls, and matched?	Yes (right: 11; left: 1)
Is time post stroke onset reported and appropriate to the study design?	Yes (range 1-12 years)
To what extent is the nature of aphasia characterized?	Comprehensive battery
Language evaluation	WAB, BNT, semantic fluency, phonemic fluency
Aphasia severity	AQ range 10.4-94.6
Aphasia type	8 anomic, 2 Broca's, 1 conduction, 1 global
First stroke only?	Yes
Stroke type	Not stated
To what extent is the lesion distribution characterized?	Individual lesions
Lesion extent	Not stated
Lesion location	L MCA
Participants notes	—

Imaging

Modality	fMRI
Is the study cross-sectional or longitudinal?	Longitudinal—chronic treatment
If longitudinal, at what time point(s) were imaging data acquired?	T1: pre-treatment/chronic (1-2 weeks prior to treatment); T2: post-treatment (within 1 week after end of 2-week treatment); T3: 13-20 weeks after end of treatment
If longitudinal, was there any intervention between the time points?	Modified CIAT + intermittent theta burst stimulation to residual left hemispheric language activation, 45 minutes/session, 5 days/week, 2 weeks
Is the scanner described?	Yes (Siemens Allegra 3 Tesla)
Is the timing of stimulus presentation and image acquisition clearly described and appropriate?	Yes
Design type	Block
Total images acquired	330
Are the imaging acquisition parameters, including	Yes (whole brain)

coverage, adequately described and appropriate?	
Is preprocessing and intrasubject coregistration adequately described and appropriate?	Yes
Is first level model fitting adequately described and appropriate?	Yes
Is intersubject normalization adequately described and appropriate?	Yes
Imaging notes	—

Conditions

Are the conditions clearly described?	Yes

Condition	Response type	Repetitions	All groups could do?	All individuals could do?
semantic decision	Button press	5	Unknown	Unknown
tone decision	Button press	6	Unknown	Unknown

Conditions notes	—

Contrasts

Are the contrasts clearly described?	Yes

Contrast 1: semantic decision vs tone decision

Language condition	Semantic decision
Control condition	Tone decision
Are the conditions matched for visual demands?	Yes
Are the conditions matched for auditory demands?	Yes
Are the conditions matched for motor demands?	Yes
Are the conditions matched for cognitive/executive demands?	Yes
Is accuracy matched between the language and control tasks for all relevant groups?	Unknown, not reported
Is reaction time matched between the language and control tasks for all relevant groups?	Unknown, not reported
Behavioral data notes	—
Are control data reported in this paper or another that is referenced?	Yes
Does the contrast selectively activate plausible relevant language regions in the control group?	Yes
Are activations lateralized in the control data?	Yes
Control activation notes	L frontal and temporal, plus other semantic regions
Contrast notes	—

Analyses

Are the analyses clearly described?	Yes

Voxelwise analysis 1

First level contrast	Semantic decision vs tone decision
Analysis class	Longitudinal change in aphasia
Group(s)	Aphasia T2 vs T1
Covariate	—
Is the second level contrast valid in terms of the group(s), time point(s), and measures involved?	Yes
Is accuracy matched across the second level contrast?	Unknown, not reported
Is reaction time matched across the second level contrast?	Unknown, not reported

Behavioral data notes	—
Type of analysis	Voxelwise
Search volume	Whole brain
Correction for multiple comparisons	Clusterwise correction based on cluster_threshold_beta
Software	SPM12
Voxelwise p	.05
Cluster extent	0.928 cc
Statistical details	—
Findings	↑ L supramarginal gyrus<br>↑ L intraparietal sulcus<br>↑ L precuneus<br>↑ L posterior STG<br>↑ L Heschl's gyrus<br>↑ L mid temporal<br>↑ L anterior temporal<br>↑ R supramarginal gyrus<br>↑ R superior parietal<br>↑ R precuneus<br>↑ R mid temporal<br>↑ R anterior cingulate<br>↓ L IFG pars opercularis<br>↓ L dorsolateral prefrontal cortex<br>↓ L ventral precentral/inferior frontal junction<br>↓ L dorsal precentral<br>↓ L SMA/medial prefrontal<br>↓ L somato-motor<br>↓ L superior parietal<br>↓ L occipital
Findings notes	—

Voxelwise analysis 2

First level contrast	Semantic decision vs tone decision
Analysis class	Longitudinal change in aphasia
Group(s)	Aphasia T3 vs T2
Covariate	—
Is the second level contrast valid in terms of the group(s), time point(s), and measures involved?	Yes
Is accuracy matched across the second level contrast?	Unknown, not reported
Is reaction time matched across the second level contrast?	Unknown, not reported
Behavioral data notes	—
Type of analysis	Voxelwise
Search volume	Whole brain
Correction for multiple comparisons	Clusterwise correction based on cluster_threshold_beta
Software	SPM12
Voxelwise p	.05
Cluster extent	0.928 cc
Statistical details	—
Findings	↑ L dorsolateral prefrontal cortex<br>↑ L angular gyrus<br>↑ L precuneus<br>↑ L posterior STS<br>↓ L SMA/medial prefrontal<br>↓ L anterior temporal<br>↓ L anterior cingulate<br>↓ R IFG<br>↓ R dorsolateral prefrontal cortex

	↓ R ventral precentral/inferior frontal junction<br>↓ R SMA/medial prefrontal<br>↓ R somato-motor<br>↓ R precuneus<br>↓ R posterior STG/STS/MTG<br>↓ R anterior temporal
Findings notes	—

Voxelwise analysis 3

First level contrast	Semantic decision vs tone decision
Analysis class	Longitudinal change in aphasia
Group(s)	Aphasia T3 vs T1
Covariate	—
Is the second level contrast valid in terms of the group(s), time point(s), and measures involved?	Yes
Is accuracy matched across the second level contrast?	Unknown, not reported
Is reaction time matched across the second level contrast?	Unknown, not reported
Behavioral data notes	—
Type of analysis	Voxelwise
Search volume	Whole brain
Correction for multiple comparisons	Clusterwise correction based on cluster_threshold_beta
Software	SPM12
Voxelwise p	.05
Cluster extent	0.928 cc
Statistical details	—
Findings	↑ L supramarginal gyrus<br>↑ L angular gyrus<br>↑ L precuneus<br>↑ L posterior STG<br>↑ L mid temporal<br>↑ L anterior temporal<br>↑ L posterior cingulate<br>↓ L somato-motor<br>↓ R dorsolateral prefrontal cortex
Findings notes	—

Voxelwise analysis 4

First level contrast	Semantic decision vs tone decision
Analysis class	Longitudinal correlation with language or other measure
Group(s)	Aphasia T3 vs aphasia T2
Covariate	Δ WAB AQ
Is the second level contrast valid in terms of the group(s), time point(s), and measures involved?	Yes
Is accuracy matched across the second level contrast?	Unknown, not reported
Is reaction time matched across the second level contrast?	Unknown, not reported
Behavioral data notes	—
Type of analysis	Voxelwise
Search volume	Whole brain
Correction for multiple comparisons	Clusterwise correction based on cluster_threshold_beta
Software	SPM12
Voxelwise p	.05
Cluster extent	0.928 cc
Statistical details	Inclusive mask of voxels that differed between T2 and T3

Findings	↓ L inferior parietal lobule
Findings notes	—

Voxelwise analysis 5

First level contrast	Semantic decision vs tone decision
Analysis class	Longitudinal correlation with language or other measure
Group(s)	Aphasia T3 vs aphasia T1
Covariate	Δ BNT
Is the second level contrast valid in terms of the group(s), time point(s), and measures involved?	Yes
Is accuracy matched across the second level contrast?	Unknown, not reported
Is reaction time matched across the second level contrast?	Unknown, not reported
Behavioral data notes	—
Type of analysis	Voxelwise
Search volume	Whole brain
Correction for multiple comparisons	Clusterwise correction based on cluster_threshold_beta
Software	SPM12
Voxelwise p	.05
Cluster extent	0.928 cc
Statistical details	Inclusive mask of voxels that differed between T1 and T3
Findings	↓ R IFG
Findings notes	—

Notes

Excluded analyses	—

van de Sandt-Koenderman et al. (2018)

Reference

Authors	van de Sandt-Koenderman, MWME; Orellana, CPM; van der Meulen, I; Smits, M; Ribbers, GM
Title	Language lateralisation after Melodic Intonation Therapy: an fMRI study in subacute and chronic aphasia
Reference	*Aphasiology* 2018; 32: 765-783
PMID	N/A
DOI	10.1080/02687038.2016.1240353

Participants

Language	Dutch
Inclusion criteria	Severe non-fluent aphasia (< 50 words/minute); articulation deficits; repetition severely affected; moderate-good auditory comprehension
Number of individuals with aphasia	9
Number of control participants	0
Were any of the participants included in any previous studies?	No
Is age reported for patients and controls, and matched?	Yes (subacute: mean 51.2 years, range 25-61 years; chronic: mean 54.0 years, range 21-66 years)
Is sex reported for patients and controls, and matched?	Yes (males: 5; females: 4)
Is handedness reported for patients and controls, and matched?	Yes (right: 8; left: 0; other: 1)
Is time post stroke onset reported and appropriate	Yes (subacute: range 0.5-3 months; chronic: range 17-40 months)

to the study design?	
To what extent is the nature of aphasia characterized?	Comprehensive battery
Language evaluation	AAT, ANELT
Aphasia severity	T1: subacute: ASRS median 1, range 0-2; ANELT range 10-29; chronic: ASRS median 1.5, range 1-2; ANELT range 20-29; T2: subacute: ASRS range 1-3; ANELT range 10-43; chronic: ASRS range 1-2; ANELT range 22-31
Aphasia type	T1: all severe non-fluent; T2: not stated
First stroke only?	Not stated
Stroke type	Not stated
To what extent is the lesion distribution characterized?	Extent and location
Lesion extent	Subacute: range 32.4-141.2 cc (no lesion extent was reported for one subacute participant because there was no tissue loss yet); chronic: range 27.4-87.9 cc
Lesion location	8 L MCA, 1 L SMA and R insular-temporoparietal
Participants notes	—

Imaging

Modality	fMRI
Is the study cross-sectional or longitudinal?	Longitudinal—mixed
If longitudinal, at what time point(s) were imaging data acquired?	T1: pre treatment/subacute or chronic; T2: post-treatment, ~6 weeks later
If longitudinal, was there any intervention between the time points?	MIT, 5+ hours/week
Is the scanner described?	No (GE 3 Tesla; model not stated)
Is the timing of stimulus presentation and image acquisition clearly described and appropriate?	Yes
Design type	Block
Total images acquired	132
Are the imaging acquisition parameters, including coverage, adequately described and appropriate?	Yes (whole brain)
Is preprocessing and intrasubject coregistration adequately described and appropriate?	Yes
Is first level model fitting adequately described and appropriate?	Yes
Is intersubject normalization adequately described and appropriate?	No (lesion impact not addressed)
Imaging notes	—

Conditions

Are the conditions clearly described?	Yes

Condition	Response type	Repetitions	All groups could do?	All individuals could do?
listening to narrative speech	None	6	N/A	N/A
listening to reversed speech	None	6	N/A	N/A

Conditions notes	—

Contrasts

Are the contrasts clearly described?	Yes

Contrast 1: listening to narrative speech vs listening to reversed speech

Language condition	Listening to narrative speech
Control condition	Listening to reversed speech
Are the conditions matched for visual demands?	Yes
Are the conditions matched for auditory demands?	Yes

Are the conditions matched for motor demands?	Yes
Are the conditions matched for cognitive/executive demands?	Yes
Is accuracy matched between the language and control tasks for all relevant groups?	N/A, no behavioral measure
Is reaction time matched between the language and control tasks for all relevant groups?	N/A, no timeable task
Behavioral data notes	—
Are control data reported in this paper or another that is referenced?	No
Does the contrast selectively activate plausible relevant language regions in the control group?	Unknown
Are activations lateralized in the control data?	Unknown
Control activation notes	—
Contrast notes	—

Analyses

Are the analyses clearly described?	No* (moderate limitation) (see specific limitation(s) below)

ROI analysis 1

First level contrast	Listening to narrative speech vs listening to reversed speech
Analysis class	Cross-sectional correlation with language or other measure
Group(s)	Aphasia T1
Covariate	Lesion volume
Is the second level contrast valid in terms of the group(s), time point(s), and measures involved?	Yes
Is accuracy matched across the second level contrast?	N/A, no behavioral measure
Is reaction time matched across the second level contrast?	N/A, no timeable task
Behavioral data notes	—
Type of analysis	Region of interest (ROI)
ROI type	Laterality indi(ces)
How many ROIs are there?	1
What are the ROI(s)?	Language network LI
How are the ROI(s) defined?	Activations that were "not clearly related to known language areas" were excluded, but the basis for this determination is not clear
Correction for multiple comparisons	One only
Statistical details	—
Findings	None
Findings notes	—

ROI analysis 2

First level contrast	Listening to narrative speech vs listening to reversed speech
Analysis class	Longitudinal correlation with language or other measure
Group(s)	Aphasia T2 vs T1
Covariate	Lesion volume
Is the second level contrast valid in terms of the group(s), time point(s), and measures involved?	Yes
Is accuracy matched across the second level contrast?	N/A, no behavioral measure
Is reaction time matched across the second level contrast?	N/A, no timeable task
Behavioral data notes	—
Type of analysis	Region of interest (ROI)
ROI type	Laterality indi(ces)

How many ROIs are there?	1
What are the ROI(s)?	Language network LI
How are the ROI(s) defined?	Activations that were "not clearly related to known language areas" were excluded, but the basis for this determination is not clear
Correction for multiple comparisons	One only
Statistical details	—
Findings	None
Findings notes	—

ROI analysis 3

First level contrast	Listening to narrative speech vs listening to reversed speech
Analysis class	Longitudinal correlation with language or other measure
Group(s)	Aphasia T2 vs T1
Covariate	Δ AAT repetition score
Is the second level contrast valid in terms of the group(s), time point(s), and measures involved?	Yes
Is accuracy matched across the second level contrast?	N/A, no behavioral measure
Is reaction time matched across the second level contrast?	N/A, no timeable task
Behavioral data notes	—
Type of analysis	Region of interest (ROI)
ROI type	Laterality indi(ces)
How many ROIs are there?	1
What are the ROI(s)?	Language network LI
How are the ROI(s) defined?	Activations that were "not clearly related to known language areas" were excluded, but the basis for this determination is not clear
Correction for multiple comparisons	One only
Statistical details	—
Findings	None
Findings notes	—

ROI analysis 4

First level contrast	Listening to narrative speech vs listening to reversed speech
Analysis class	Longitudinal correlation with language or other measure
Group(s)	Aphasia T2 vs T1
Covariate	Δ ANELT
Is the second level contrast valid in terms of the group(s), time point(s), and measures involved?	Yes
Is accuracy matched across the second level contrast?	N/A, no behavioral measure
Is reaction time matched across the second level contrast?	N/A, no timeable task
Behavioral data notes	—
Type of analysis	Region of interest (ROI)
ROI type	Laterality indi(ces)
How many ROIs are there?	1
What are the ROI(s)?	Language network LI
How are the ROI(s) defined?	Activations that were "not clearly related to known language areas" were excluded, but the basis for this determination is not clear
Correction for multiple comparisons	One only
Statistical details	—
Findings	None
Findings notes	—

Notes

Excluded analyses	Individual participant LIs and activation maps

van Oers et al. (2018)

Reference

Authors	van Oers CAMM, van der Worp HB, Kappelle LJ, Raemaekers MAH, Otte WM, Dijkhuizen RM
Title	Etiology of language network changes during recovery of aphasia after stroke
Reference	*Sci Rep* 2018; 8: 856
PMID	29339771
DOI	10.1038/s41598-018-19302-4

Participants

Language	Dutch
Inclusion criteria	MRS ≤ 3; ability to perform tasks
Number of individuals with aphasia	12
Number of control participants	8
Were any of the participants included in any previous studies?	No
Is age reported for patients and controls, and matched?	Yes (mean 67.9 ± 11.4 years, range 46-86 years)
Is sex reported for patients and controls, and matched?	Yes (males: 10; females: 2)
Is handedness reported for patients and controls, and matched?	Yes (right: 12; left: 0)
Is time post stroke onset reported and appropriate to the study design?	No* (moderate limitation) (T1: within 2 weeks; T2: ~3 months; T3: ~6 months; T4: ~12 months; specific timing of first time point not stated)
To what extent is the nature of aphasia characterized?	Comprehensive battery
Language evaluation	AAT, BNT
Aphasia severity	T1: 8 moderate, 2 severe, 2 not stated; T2: 4 moderate, 3 recovered, 2 not stated, 1 mild, 1 severe
Aphasia type	T1: 6 Broca's, 3 anomic, 2 Wernicke's, 1 global; T2: 4 anomic, 3 recovered, 2 Broca's, 1 unclassified, 1 Wernicke's
First stroke only?	Yes
Stroke type	Ischemic only
To what extent is the lesion distribution characterized?	Lesion overlay
Lesion extent	Range 9-208 cc
Lesion location	L MCA
Participants notes	—

Imaging

Modality	fMRI
Is the study cross-sectional or longitudinal?	Longitudinal—recovery
If longitudinal, at what time point(s) were imaging data acquired?	T1: within 2 weeks; T2: ~3 months; T3: ~6 months; T4: ~12 months; specific timing of first time point not stated
If longitudinal, was there any intervention between the time points?	Not stated
Is the scanner described?	Yes (Philips Achieva 3 Tesla)
Is the timing of stimulus presentation and image acquisition clearly described and appropriate?	No* (moderate limitation) (stimulus presentation was self-paced, but the ITI is not reported, nor are the number of trials presented per condition; it is likely that the language and control blocks contained different numbers of trials)
Design type	Block

Total images acquired	1656
Are the imaging acquisition parameters, including coverage, adequately described and appropriate?	Yes (whole brain)
Is preprocessing and intrasubject coregistration adequately described and appropriate?	Yes
Is first level model fitting adequately described and appropriate?	Yes
Is intersubject normalization adequately described and appropriate?	Yes
Imaging notes	not all participants scanned at each time point; the number scanned at each time point is not stated

Conditions

Are the conditions clearly described?	Yes

Condition	Response type	Repetitions	All groups could do?	All individuals could do?
written word-picture matching	Button press	6	Unknown	Unknown
semantic decision	Button press	6	Unknown	Unknown
visual decision	Button press	12	Unknown	Unknown
rest	None	12	N/A	N/A

Conditions notes	—

Contrasts

Are the contrasts clearly described?	Yes

Contrast 1: written word-picture matching vs visual decision

Language condition	Written word-picture matching
Control condition	Visual decision
Are the conditions matched for visual demands?	No
Are the conditions matched for auditory demands?	Yes
Are the conditions matched for motor demands?	Yes
Are the conditions matched for cognitive/executive demands?	No
Is accuracy matched between the language and control tasks for all relevant groups?	Unknown, not reported
Is reaction time matched between the language and control tasks for all relevant groups?	Unknown, not reported
Behavioral data notes	—
Are control data reported in this paper or another that is referenced?	Somewhat
Does the contrast selectively activate plausible relevant language regions in the control group?	No
Are activations lateralized in the control data?	Somewhat
Control activation notes	Primarily bilateral visual activations; frontal activation is L-lateralized
Contrast notes	—

Contrast 2: semantic decision vs visual decision

Language condition	Semantic decision
Control condition	Visual decision
Are the conditions matched for visual demands?	No
Are the conditions matched for auditory demands?	Yes
Are the conditions matched for motor demands?	Yes
Are the conditions matched for cognitive/executive demands?	No
Is accuracy matched between the language and	Unknown, not reported

control tasks for all relevant groups?	
Is reaction time matched between the language and control tasks for all relevant groups?	Unknown, not reported
Behavioral data notes	—
Are control data reported in this paper or another that is referenced?	Somewhat
Does the contrast selectively activate plausible relevant language regions in the control group?	Somewhat
Are activations lateralized in the control data?	Yes
Control activation notes	L frontal, L posterior ITG, L superior parietal
Contrast notes	—

Analyses

Are the analyses clearly described?	No* (moderate limitation) (see specific limitation(s) below)

ROI analysis 1

First level contrast	Written word-picture matching vs visual decision
Analysis class	Cross-sectional correlation with language or other measure
Group(s)	Aphasia (subset who returned for follow-up) T1 (n = 10)
Covariate	Subsequent outcome (T4) overall language measure (average of AAT measures)
Is the second level contrast valid in terms of the group(s), time point(s), and measures involved?	Yes
Is accuracy matched across the second level contrast?	Unknown, not reported
Is reaction time matched across the second level contrast?	Unknown, not reported
Behavioral data notes	—
Type of analysis	Regions of interest (ROI)
ROI type	Functional
How many ROIs are there?	12
What are the ROI(s)?	(1) bilateral dorsal anterior cingulate; (2) L angular gyrus; (3) L IFG pars opercularis and triangularis; (4) L thalamus; (5) L MFG; (6) L posterior ITG; (7) R angular gyrus; (8) R IFG pars triangularis; (9) R thalamus; (10) R posterior ITG; (11) R IFG pars opercularis and triangularis; (12) R MFG
How are the ROI(s) defined?	Control activations and their homotopic counterparts in the R hemisphere; activation measured as count of voxels activated at $p < 0.001$, uncorrected
Correction for multiple comparisons	False discovery rate (FDR)
Statistical details	—
Findings	↑ L posterior inferior temporal gyrus/fusiform gyrus
Findings notes	Activation predicted later outcome even when initial language performance was included in the model

ROI analysis 2

First level contrast	Written word-picture matching vs visual decision
Analysis class	Cross-sectional correlation with language or other measure
Group(s)	Aphasia (all time points)
Covariate	Overall language measure (average of AAT measures) all time points
Is the second level contrast valid in terms of the group(s), time point(s), and measures involved?	Yes
Is accuracy matched across the second level contrast?	Unknown, not reported
Is reaction time matched across the second level contrast?	Unknown, not reported
Behavioral data notes	—
Type of analysis	Regions of interest (ROI)
ROI type	Functional

How many ROIs are there?	12
What are the ROI(s)?	(1) bilateral dorsal anterior cingulate; (2) L angular gyrus; (3) L IFG pars opercularis and triangularis; (4) L thalamus; (5) L MFG; (6) L posterior ITG; (7) R angular gyrus; (8) R IFG pars triangularis; (9) R thalamus; (10) R posterior ITG; (11) R IFG pars opercularis and triangularis; (12) R MFG
How are the ROI(s) defined?	Control activations and their homotopic counterparts in the R hemisphere; activation measured as count of voxels activated at p < 0.001, uncorrected
Correction for multiple comparisons	False discovery rate (FDR)
Statistical details	Mixed model; minimal detail provided
Findings	↑ L posterior inferior temporal gyrus/fusiform gyrus
Findings notes	—

ROI analysis 3

First level contrast	Written word-picture matching vs visual decision
Analysis class	Cross-sectional correlation with language or other measure
Group(s)	Aphasia (all time points)
Covariate	Average of AAT comprehension score and BNT, all time points
Is the second level contrast valid in terms of the group(s), time point(s), and measures involved?	Yes
Is accuracy matched across the second level contrast?	Unknown, not reported
Is reaction time matched across the second level contrast?	Unknown, not reported
Behavioral data notes	—
Type of analysis	Regions of interest (ROI)
ROI type	Functional
How many ROIs are there?	12
What are the ROI(s)?	(1) bilateral dorsal anterior cingulate; (2) L angular gyrus; (3) L IFG pars opercularis and triangularis; (4) L thalamus; (5) L MFG; (6) L posterior ITG; (7) R angular gyrus; (8) R IFG pars triangularis; (9) R thalamus; (10) R posterior ITG; (11) R IFG pars opercularis and triangularis; (12) R MFG
How are the ROI(s) defined?	Control activations and their homotopic counterparts in the R hemisphere; activation measured as count of voxels activated at p < 0.001, uncorrected
Correction for multiple comparisons	False discovery rate (FDR)
Statistical details	Mixed model; minimal detail provided
Findings	↓ R IFG pars opercularis<br>↓ R IFG pars triangularis
Findings notes	—

ROI analysis 4

First level contrast	Written word-picture matching vs visual decision
Analysis class	Cross-sectional correlation with language or other measure
Group(s)	Aphasia (all time points)
Covariate	Picture-word matching accuracy, all time points
Is the second level contrast valid in terms of the group(s), time point(s), and measures involved?	Yes
Is accuracy matched across the second level contrast?	Accuracy is covariate
Is reaction time matched across the second level contrast?	Unknown, not reported
Behavioral data notes	—
Type of analysis	Regions of interest (ROI)
ROI type	Functional
How many ROIs are there?	12
What are the ROI(s)?	(1) bilateral dorsal anterior cingulate; (2) L angular gyrus; (3) L IFG pars opercularis and triangularis; (4) L thalamus; (5) L MFG; (6) L posterior ITG; (7) R angular gyrus; (8) R IFG pars

	triangularis; (9) R thalamus; (10) R posterior ITG; (11) R IFG pars opercularis and triangularis; (12) R MFG
How are the ROI(s) defined?	Control activations and their homotopic counterparts in the R hemisphere; activation measured as count of voxels activated at p < 0.001, uncorrected
Correction for multiple comparisons	False discovery rate (FDR)
Statistical details	Mixed model; minimal detail provided
Findings	↑ R posterior inferior temporal gyrus/fusiform gyrus
Findings notes	—

ROI analysis 5

First level contrast	Written word-picture matching vs visual decision
Analysis class	Longitudinal change in aphasia
Group(s)	Aphasia: linear effect of time
Covariate	—
Is the second level contrast valid in terms of the group(s), time point(s), and measures involved?	Yes
Is accuracy matched across the second level contrast?	Unknown, not reported
Is reaction time matched across the second level contrast?	Unknown, not reported
Behavioral data notes	—
Type of analysis	Regions of interest (ROI)
ROI type	Functional
How many ROIs are there?	12
What are the ROI(s)?	(1) bilateral dorsal anterior cingulate; (2) L angular gyrus; (3) L IFG pars opercularis and triangularis; (4) L thalamus; (5) L MFG; (6) L posterior ITG; (7) R angular gyrus; (8) R IFG pars triangularis; (9) R thalamus; (10) R posterior ITG; (11) R IFG pars opercularis and triangularis; (12) R MFG
How are the ROI(s) defined?	Control activations and their homotopic counterparts in the R hemisphere; activation measured as count of voxels activated at p < 0.001, uncorrected
Correction for multiple comparisons	False discovery rate (FDR)
Statistical details	Mixed model; minimal detail provided
Findings	↑ L dorsolateral prefrontal cortex<br>↑ L angular gyrus<br>↑ L posterior inferior temporal gyrus/fusiform gyrus<br>↑ L anterior cingulate<br>↑ R dorsolateral prefrontal cortex<br>↑ R angular gyrus<br>↑ R anterior cingulate<br>↑ R thalamus<br>↓ L IFG pars opercularis<br>↓ L IFG pars triangularis
Findings notes	Similar numbers of findings are reported for controls

ROI analysis 6

First level contrast	Semantic decision vs visual decision
Analysis class	Cross-sectional correlation with language or other measure
Group(s)	Aphasia (subset who returned for follow-up) T1 (n = 10)
Covariate	Subsequent outcome (T4) overall language measure (average of AAT measures)
Is the second level contrast valid in terms of the group(s), time point(s), and measures involved?	Somewhat (not appropriate to correlate T1 imaging with T4 behavior without T1 behavior in model)
Is accuracy matched across the second level contrast?	Unknown, not reported
Is reaction time matched across the second level contrast?	Unknown, not reported
Behavioral data notes	—
Type of analysis	Regions of interest (ROI)

ROI type	Functional
How many ROIs are there?	6
What are the ROI(s)?	(1) L angular gyrus; (2) L IFG pars opercularis and triangularis; (3) L posterior ITG; (4) R angular gyrus; (5) R IFG pars opercularis and triangularis; (6) R posterior ITG
How are the ROI(s) defined?	Control activations and their homotopic counterparts in the R hemisphere; activation measured as count of voxels activated at $p < 0.001$, uncorrected
Correction for multiple comparisons	False discovery rate (FDR)
Statistical details	—
Findings	None
Findings notes	—

ROI analysis 7

First level contrast	Semantic decision vs visual decision
Analysis class	Cross-sectional correlation with language or other measure
Group(s)	Aphasia (all time points)
Covariate	Overall language measure (average of AAT measures) all time points
Is the second level contrast valid in terms of the group(s), time point(s), and measures involved?	Yes
Is accuracy matched across the second level contrast?	Unknown, not reported
Is reaction time matched across the second level contrast?	Unknown, not reported
Behavioral data notes	—
Type of analysis	Regions of interest (ROI)
ROI type	Functional
How many ROIs are there?	6
What are the ROI(s)?	(1) L angular gyrus; (2) L IFG pars opercularis and triangularis; (3) L posterior ITG; (4) R angular gyrus; (5) R IFG pars opercularis and triangularis; (6) R posterior ITG
How are the ROI(s) defined?	Control activations and their homotopic counterparts in the R hemisphere; activation measured as count of voxels activated at $p < 0.001$, uncorrected
Correction for multiple comparisons	False discovery rate (FDR)
Statistical details	Mixed model; minimal detail provided
Findings	None
Findings notes	—

ROI analysis 8

First level contrast	Semantic decision vs visual decision
Analysis class	Cross-sectional correlation with language or other measure
Group(s)	Aphasia (all time points)
Covariate	Average of AAT comprehension score and BNT, all time points
Is the second level contrast valid in terms of the group(s), time point(s), and measures involved?	Yes
Is accuracy matched across the second level contrast?	Unknown, not reported
Is reaction time matched across the second level contrast?	Unknown, not reported
Behavioral data notes	—
Type of analysis	Regions of interest (ROI)
ROI type	Functional
How many ROIs are there?	6
What are the ROI(s)?	(1) L angular gyrus; (2) L IFG pars opercularis and triangularis; (3) L posterior ITG; (4) R angular gyrus; (5) R IFG pars opercularis and triangularis; (6) R posterior ITG
How are the ROI(s) defined?	Control activations and their homotopic counterparts in the R hemisphere; activation measured as count of voxels activated at $p < 0.001$, uncorrected
Correction for multiple comparisons	False discovery rate (FDR)
Statistical details	Mixed model; minimal detail provided

Findings	None
Findings notes	—

ROI analysis 9

First level contrast	Semantic decision vs visual decision
Analysis class	Cross-sectional correlation with language or other measure
Group(s)	Aphasia (all time points)
Covariate	Semantic decision accuracy, all time points
Is the second level contrast valid in terms of the group(s), time point(s), and measures involved?	Yes
Is accuracy matched across the second level contrast?	Accuracy is covariate
Is reaction time matched across the second level contrast?	Unknown, not reported
Behavioral data notes	—
Type of analysis	Regions of interest (ROI)
ROI type	Functional
How many ROIs are there?	6
What are the ROI(s)?	(1) L angular gyrus; (2) L IFG pars opercularis and triangularis; (3) L posterior ITG; (4) R angular gyrus; (5) R IFG pars opercularis and triangularis; (6) R posterior ITG
How are the ROI(s) defined?	Control activations and their homotopic counterparts in the R hemisphere; activation measured as count of voxels activated at p < 0.001, uncorrected
Correction for multiple comparisons	False discovery rate (FDR)
Statistical details	Mixed model; minimal detail provided
Findings	None
Findings notes	—

ROI analysis 10

First level contrast	Semantic decision vs visual decision
Analysis class	Longitudinal change in aphasia
Group(s)	Aphasia: linear effect of time
Covariate	—
Is the second level contrast valid in terms of the group(s), time point(s), and measures involved?	Yes
Is accuracy matched across the second level contrast?	Unknown, not reported
Is reaction time matched across the second level contrast?	Unknown, not reported
Behavioral data notes	—
Type of analysis	Regions of interest (ROI)
ROI type	Functional
How many ROIs are there?	6
What are the ROI(s)?	(1) L angular gyrus; (2) L IFG pars opercularis and triangularis; (3) L posterior ITG; (4) R angular gyrus; (5) R IFG pars opercularis and triangularis; (6) R posterior ITG
How are the ROI(s) defined?	Control activations and their homotopic counterparts in the R hemisphere; activation measured as count of voxels activated at p < 0.001, uncorrected
Correction for multiple comparisons	False discovery rate (FDR)
Statistical details	Mixed model; minimal detail provided
Findings	↑ L posterior inferior temporal gyrus/fusiform gyrus<br>↑ R angular gyrus<br>↓ L IFG pars opercularis<br>↓ L IFG pars triangularis
Findings notes	Similar numbers of findings are reported for controls

Notes

Excluded analyses	(1) activation maps in patients at each time point (Fig. 2); (2) analyses assessing whether

	outcome can be better predicted by including fMRI data; (3) analyses examining relationships between activations related to breath holding and language tasks (there was little if any evidence that vascular reactivity was abnormal in patients); (4) correlations with ROI activity level instead of counts of activated voxels, which yielded similar but non-significant findings

Barbieri et al. (2019)

Reference

Authors	Barbieri E, Mack J, Chiappetta B, Europa E, Thompson CK
Title	Recovery of offline and online sentence processing in aphasia: Language and domain-general network neuroplasticity
Reference	*Cortex* 2019; 120: 394-418
PMID	31419597
DOI	10.1016/j.cortex.2019.06.015

Participants

Language	US English
Inclusion criteria	—
Number of individuals with aphasia	18 (plus 1 excluded: developed a hematoma between baseline and post-testing)
Number of control participants	23
Were any of the participants included in any previous studies?	No
Is age reported for patients and controls, and matched?	No (range 22-73 years; controls were younger)
Is sex reported for patients and controls, and matched?	Yes (males: 11; females: 7)
Is handedness reported for patients and controls, and matched?	No (right: 15; left: 3; not stated for controls)
Is time post stroke onset reported and appropriate to the study design?	Yes (range 13-107 months)
To what extent is the nature of aphasia characterized?	Comprehensive battery
Language evaluation	WAB, Northwestern Assessment of Verbs and Sentences (NAVS), Northwestern Naming Battery (NNB), analysis of spontaneous speech (Cinderella story) using Northwestern Narrative Language Analysis (NNLA) protocol
Aphasia severity	AQ range 52.8-91.7
Aphasia type	Not stated, except that "language deficits were consistent with nonfluent aphasia and agrammatism"
First stroke only?	Yes
Stroke type	Mixed etiologies
To what extent is the lesion distribution characterized?	Lesion overlay
Lesion extent	Not stated
Lesion location	Mostly L MCA but some lesions include PCA or ACA territory
Participants notes	One patient had two strokes within one day, but we would consider that essentially a single stroke

Imaging

Modality	fMRI
Is the study cross-sectional or longitudinal?	Longitudinal—chronic treatment
If longitudinal, at what time point(s) were imaging data acquired?	T1: pre-treatment/chronic; T2: post-treatment, ~12 weeks later
If longitudinal, was there any intervention between the time points?	13 patients were treated and 5 were not; treatment of underlying forms; 90 minutes/session, 2 sessions/week until 80% accuracy met on weekly probe task, then 1 session/week, 12 weeks

	except for one patient who demonstrated rapid improvement and completed treatment in 6 weeks
Is the scanner described?	Yes (Siemens Trio 3 Tesla or Siemens Prisma 3 Tesla)
Is the timing of stimulus presentation and image acquisition clearly described and appropriate?	No* (moderate limitation) (stimulus timing described does not match stated duration of data acquisition; timing of language and control trials not matched)
Design type	Block
Total images acquired	~482
Are the imaging acquisition parameters, including coverage, adequately described and appropriate?	Yes (whole brain)
Is preprocessing and intrasubject coregistration adequately described and appropriate?	Yes
Is first level model fitting adequately described and appropriate?	Yes
Is intersubject normalization adequately described and appropriate?	Yes
Imaging notes	2 runs before treatment and 2 runs after treatment; each pair of runs took place on two separate days (1-7 days apart)

Conditions

Are the conditions clearly described?	Yes

Condition	Response type	Repetitions	All groups could do?	All individuals could do?
auditory sentence-picture verification	Button press	32	Unknown	Unknown
listening to reversed speech and viewing scrambled pictures	Button press	8	Unknown	Unknown

Conditions notes	Based on the behavioral data obtained outside the scanner, it is likely that many patients were at chance on the language task

Contrasts

Are the contrasts clearly described?	No (see specific limitation(s) below)

Contrast 1: auditory sentence-picture verification vs listening to reversed speech and viewing scrambled pictures

Language condition	Auditory sentence-picture verification
Control condition	Listening to reversed speech and viewing scrambled pictures
Are the conditions matched for visual demands?	Yes
Are the conditions matched for auditory demands?	Yes
Are the conditions matched for motor demands?	Yes
Are the conditions matched for cognitive/executive demands?	No
Is accuracy matched between the language and control tasks for all relevant groups?	Unknown, not reported
Is reaction time matched between the language and control tasks for all relevant groups?	Unknown, not reported
Behavioral data notes	—
Are control data reported in this paper or another that is referenced?	Yes
Does the contrast selectively activate plausible relevant language regions in the control group?	Somewhat
Are activations lateralized in the control data?	Somewhat
Control activation notes	L-lateralized inferior frontal and posterior temporal, but also bilateral posterior inferior temporal and lateral occipital activations
Contrast notes	Contrast described as "passive > control" but seems to involve active and passive sentences

Analyses

Are the analyses clearly described?	No* (moderate limitation) (see specific limitation(s) below)

Voxelwise analysis 1

First level contrast	Auditory sentence-picture verification vs listening to reversed speech and viewing scrambled pictures
Analysis class	Longitudinal change in aphasia
Group(s)	Aphasia treated (n = 13) T2 vs T1
Covariate	—
Is the second level contrast valid in terms of the group(s), time point(s), and measures involved?	Yes
Is accuracy matched across the second level contrast?	Unknown, not reported
Is reaction time matched across the second level contrast?	Unknown, not reported
Behavioral data notes	Out-of-scanner performance on passive sentences improved
Type of analysis	Voxelwise
Search volume	
Correction for multiple comparisons	Clusterwise correction based on 3dClustSim
Software	SPM8
Voxelwise p	.001
Cluster extent	37 voxels (size not stated)
Statistical details	—
Findings	↑ L precuneus<br>↑ R ventral precentral/inferior frontal junction<br>↑ R somato-motor<br>↑ R supramarginal gyrus<br>↑ R intraparietal sulcus<br>↑ R superior parietal<br>↑ R precuneus
Findings notes	Based on Table 7 and Figure 8

Voxelwise analysis 2

First level contrast	Auditory sentence-picture verification vs listening to reversed speech and viewing scrambled pictures
Analysis class	Longitudinal change in aphasia
Group(s)	Aphasia natural history (n = 5) T2 vs T1
Covariate	—
Is the second level contrast valid in terms of the group(s), time point(s), and measures involved?	Yes
Is accuracy matched across the second level contrast?	Unknown, not reported
Is reaction time matched across the second level contrast?	Unknown, not reported
Behavioral data notes	—
Type of analysis	Voxelwise
Search volume	
Correction for multiple comparisons	Clusterwise correction based on 3dClustSim
Software	SPM8
Voxelwise p	.001
Cluster extent	37 voxels (size not stated)
Statistical details	—
Findings	None
Findings notes	—

ROI analysis 1

First level contrast	Auditory sentence-picture verification vs listening to reversed speech and viewing scrambled pictures
Analysis class	Longitudinal between two groups with aphasia

Group(s)	(Aphasia treated (n=13) T2 vs T1) vs (aphasia natural history (n=5) T2 vs T1)
Covariate	—
Is the second level contrast valid in terms of the group(s), time point(s), and measures involved?	Yes
Is accuracy matched across the second level contrast?	Unknown, not reported
Is reaction time matched across the second level contrast?	Unknown, not reported
Behavioral data notes	—
Type of analysis	Regions of interest (ROI)
ROI type	Anatomical
How many ROIs are there?	4
What are the ROI(s)?	(1) L hemisphere sentence processing network (IFGpt, pMTG, pSTG, AG); (2) R hemisphere homotopic regions; (3) L dorsal attention network (MFG, PrCG, SPL, sLOC); (4) R dorsal attention network (same regions)
How are the ROI(s) defined?	Sentence processing network based on Walenski et al. (2019); dorsal attention network based on Corbetta et al. (2008) and Vincent et al. (2008); ROIs were defined based on Harvard-Oxford atlas which would align imperfectly with these functional networks; dependent variable was number of active voxels ($p < .001$, uncorrected) divided by number of intact voxels
Correction for multiple comparisons	No correction
Statistical details	Derivation of dependent measures from ROIs difficulty to follow, but it seems that ROIs with less than 5 voxels upregulated were excluded and deactivations were not considered, meaning that estimates of change may be biased
Findings	↑ L dorsolateral prefrontal cortex<br>↑ L ventral precentral/inferior frontal junction<br>↑ L dorsal precentral<br>↑ L angular gyrus<br>↑ L intraparietal sulcus<br>↑ L superior parietal<br>↑ R dorsolateral prefrontal cortex<br>↑ R ventral precentral/inferior frontal junction<br>↑ R dorsal precentral<br>↑ R angular gyrus<br>↑ R intraparietal sulcus<br>↑ R superior parietal
Findings notes	Bilateral dorsal attention network; findings were for networks as a whole; regions coded correspond to atlas ROIs

ROI analysis 2

First level contrast	Auditory sentence-picture verification vs listening to reversed speech and viewing scrambled pictures
Analysis class	Longitudinal correlation with language or other measure
Group(s)	Aphasia T2 vs T1
Covariate	∆ offline comprehension composite
Is the second level contrast valid in terms of the group(s), time point(s), and measures involved?	Yes
Is accuracy matched across the second level contrast?	Unknown, not reported
Is reaction time matched across the second level contrast?	Unknown, not reported
Behavioral data notes	—
Type of analysis	Regions of interest (ROI)
ROI type	Anatomical
How many ROIs are there?	4
What are the ROI(s)?	(1) L hemisphere sentence processing network (IFGpt, pMTG, pSTG, AG); (2) R hemisphere homotopic regions; (3) L dorsal attention network (MFG, PrCG, SPL, sLOC); (4) R dorsal attention network (same regions)
How are the ROI(s) defined?	Sentence processing network based on Walenski et al. (2019); dorsal attention network based

	on Corbetta et al. (2008) and Vincent et al. (2008); ROIs were defined based on Harvard-Oxford atlas which would align imperfectly with these functional networks; dependent variable was number of active voxels (p < .001, uncorrected) divided by number of intact voxels
Correction for multiple comparisons	No correction
Statistical details	Derivation of dependent measures from ROIs difficulty to follow, but it seems that ROIs with less than 5 voxels upregulated were excluded and deactivations were not considered, meaning that estimates of change may be biased
Findings	↑ R IFG pars triangularis<br>↑ R dorsolateral prefrontal cortex<br>↑ R ventral precentral/inferior frontal junction<br>↑ R dorsal precentral<br>↑ R angular gyrus<br>↑ R intraparietal sulcus<br>↑ R superior parietal<br>↑ R posterior STG/STS/MTG
Findings notes	R homotopic sentence processing network and R dorsal attention network; findings were for networks as a whole; regions coded correspond to atlas ROIs

ROI analysis 3

First level contrast	Auditory sentence-picture verification vs listening to reversed speech and viewing scrambled pictures
Analysis class	Longitudinal correlation with language or other measure
Group(s)	Aphasia participants with eye tracking data (n = 16) T2 vs T1
Covariate	∆ decrease in eye tracking online thematic prediction score
Is the second level contrast valid in terms of the group(s), time point(s), and measures involved?	Yes
Is accuracy matched across the second level contrast?	Unknown, not reported
Is reaction time matched across the second level contrast?	Unknown, not reported
Behavioral data notes	—
Type of analysis	Regions of interest (ROI)
ROI type	Anatomical
How many ROIs are there?	4
What are the ROI(s)?	(1) L hemisphere sentence processing network (IFGpt, pMTG, pSTG, AG); (2) R hemisphere homotopic regions; (3) L dorsal attention network (MFG, PrCG, SPL, sLOC); (4) R dorsal attention network (same regions)
How are the ROI(s) defined?	Sentence processing network based on Walenski et al. (2019); dorsal attention network based on Corbetta et al. (2008) and Vincent et al. (2008); ROIs were defined based on Harvard-Oxford atlas which would align imperfectly with these functional networks; dependent variable was number of active voxels (p < .001, uncorrected) divided by number of intact voxels
Correction for multiple comparisons	No correction
Statistical details	Derivation of dependent measures from ROIs difficulty to follow, but it seems that ROIs with less than 5 voxels upregulated were excluded and deactivations were not considered, meaning that estimates of change may be biased
Findings	↑ R IFG pars triangularis<br>↑ R angular gyrus<br>↑ R posterior STG/STS/MTG
Findings notes	R homotopic sentence processing network; findings were for networks as a whole; regions coded correspond to atlas ROIs

ROI analysis 4

First level contrast	Auditory sentence-picture verification vs listening to reversed speech and viewing scrambled pictures
Analysis class	Longitudinal correlation with language or other measure
Group(s)	Aphasia participants with eye tracking data (n = 16) T2 vs T1
Covariate	∆ eye tracking online thematic integragration score
Is the second level contrast valid in terms of the	Yes

group(s), time point(s), and measures involved?	
Is accuracy matched across the second level contrast?	Unknown, not reported
Is reaction time matched across the second level contrast?	Unknown, not reported
Behavioral data notes	—
Type of analysis	Regions of interest (ROI)
ROI type	Anatomical
How many ROIs are there?	4
What are the ROI(s)?	(1) L hemisphere sentence processing network (IFGpt, pMTG, pSTG, AG); (2) R hemisphere homotopic regions; (3) L dorsal attention network (MFG, PrCG, SPL, sLOC); (4) R dorsal attention network (same regions)
How are the ROI(s) defined?	Sentence processing network based on Walenski et al. (2019); dorsal attention network based on Corbetta et al. (2008) and Vincent et al. (2008); ROIs were defined based on Harvard-Oxford atlas which would align imperfectly with these functional networks; dependent variable was number of active voxels ($p < .001$, uncorrected) divided by number of intact voxels
Correction for multiple comparisons	No correction
Statistical details	Derivation of dependent measures from ROIs difficulty to follow, but it seems that ROIs with less than 5 voxels upregulated were excluded and deactivations were not considered, meaning that estimates of change may be biased
Findings	↑ R dorsolateral prefrontal cortex<br>↑ R ventral precentral/inferior frontal junction<br>↑ R dorsal precentral<br>↑ R angular gyrus<br>↑ R intraparietal sulcus<br>↑ R superior parietal
Findings notes	R dorsal attention network; findings were for networks as a whole; regions coded correspond to atlas ROIs

Notes

Excluded analyses	Analysis of relationship between lesion volume with ROIs and functional changes in ROIs, because L and R hemisphere networks seem to be combined

Johnson et al. (2019)

Reference

Authors	Johnson JP, Meier EL, Pan Y, Kiran S
Title	Treatment-related changes in neural activation vary according to treatment response and extent of spared tissue in patients with chronic aphasia
Reference	*Cortex* 2019; 121: 147-168
PMID	31627014
DOI	10.1016/j.cortex.2019.08.016

Participants

Language	US English
Inclusion criteria	Anomia
Number of individuals with aphasia	30 (plus 5 excluded: 2 withdrew from non-treatment arm; 3 fMRI acquisition errors; 1 did not complete treatment and post-treatment scanning (but of these latter 4, one must have at least completed the non-treatment arm))
Number of control participants	17
Were any of the participants included in any previous studies?	No
Is age reported for patients and controls, and matched?	Yes (treated group: mean 62.8 ± 10.2 years, range 42-80 years; untreated group: mean 59.0 ± 11.8 years, range 39-79 years)

Is sex reported for patients and controls, and matched?	Yes (males: 21; females: 9)
Is handedness reported for patients and controls, and matched?	Yes (right: 27; left: 3)
Is time post stroke onset reported and appropriate to the study design?	Yes (treated group: mean 58.3 ± 51.8 months, range 12-170 months; untreated group: mean 85.2 ± 141.9 months, range 10-467 months)
To what extent is the nature of aphasia characterized?	Severity only
Language evaluation	WAB, BNT, PPT
Aphasia severity	Treated group: AQ mean 60.1 ± 24.0, range 11.7-95.2; untreated group: AQ mean 65.8 ± 24.6, range 26.9-91.5
Aphasia type	Not stated
First stroke only?	Not stated
Stroke type	Not stated
To what extent is the lesion distribution characterized?	Lesion overlay
Lesion extent	Treated group: 136.6 ± 81.1 cc, range 11.7-317.1 cc; untreated group: 112.7 ± 94.6 cc, range 1.6-317.1 cc
Lesion location	Mostly MCA with a few extending into PCA
Participants notes	There were 26 patients in the treated group and 10 in the untreated group, but 6 patients overlapped between the two groups (they joined the treated group after completing the untreated phase)

Imaging

Modality	fMRI
Is the study cross-sectional or longitudinal?	Longitudinal—chronic treatment
If longitudinal, at what time point(s) were imaging data acquired?	T1: pre-treatment/chronic; T2: post-treatment, ~12 weeks later
If longitudinal, was there any intervention between the time points?	Semantic naming treatment, 2 sessions/week
Is the scanner described?	Yes (Siemens Trio 3 Tesla, except for 2 patients on a Siemens Prisma 3 Tesla)
Is the timing of stimulus presentation and image acquisition clearly described and appropriate?	No* (moderate limitation) (total images not stated; short ITI and minimal jitter)
Design type	Event-related
Total images acquired	not stated
Are the imaging acquisition parameters, including coverage, adequately described and appropriate?	Yes (whole brain)
Is preprocessing and intrasubject coregistration adequately described and appropriate?	Yes
Is first level model fitting adequately described and appropriate?	No* (moderate limitation) (unclear whether there was sufficient resting data to allow the key contrast to be computed)
Is intersubject normalization adequately described and appropriate?	Yes
Imaging notes	—

Conditions

Are the conditions clearly described?	Yes

Condition	Response type	Repetitions	All groups could do?	All individuals could do?
picture naming (trained items)	Word (overt)	36	Unknown	Unknown
picture naming (untrained items, from control category)	Word (overt)	36	Unknown	Unknown
picture naming (untrained items, from experimental categories)	Word (overt)	36	Unknown	Unknown
viewing scrambled images and saying "skip"	Word (overt)	36	Unknown	Unknown
rest	None	implicit baseline	N/A	N/A

Conditions notes	The untrained group were not actually trained on "trained items"; no accuracy data for untrained group (except for lack of change between T1 and T2)

Contrasts

Are the contrasts clearly described?	Yes

Contrast 1: picture naming (trained items) vs rest

Language condition	Picture naming (trained items)
Control condition	Rest
Are the conditions matched for visual demands?	No
Are the conditions matched for auditory demands?	No
Are the conditions matched for motor demands?	No
Are the conditions matched for cognitive/executive demands?	No
Is accuracy matched between the language and control tasks for all relevant groups?	N/A, tasks not comparable
Is reaction time matched between the language and control tasks for all relevant groups?	N/A, tasks not comparable
Behavioral data notes	—
Are control data reported in this paper or another that is referenced?	Somewhat
Does the contrast selectively activate plausible relevant language regions in the control group?	No
Are activations lateralized in the control data?	No
Control activation notes	Most ROIs deactivated in controls
Contrast notes	—

Analyses

Are the analyses clearly described?	Yes

ROI analysis 1

First level contrast	Picture naming (trained items) vs rest
Analysis class	Cross-sectional aphasia vs control
Group(s)	Aphasia treated T1 (n = 26) vs control
Covariate	—
Is the second level contrast valid in terms of the group(s), time point(s), and measures involved?	Yes
Is accuracy matched across the second level contrast?	No, different
Is reaction time matched across the second level contrast?	Unknown, not reported
Behavioral data notes	—
Type of analysis	Regions of interest (ROI)
ROI type	Anatomical
How many ROIs are there?	16
What are the ROI(s)?	(1) L IFGorb; (2) L IFGtri; (3) L IFGop; (4) L MFG; (5) L PrCG; (6) L MTG; (7) L SMG; (8) L AG; (9-16) homotopic counterparts
How are the ROI(s) defined?	AAL but lesioned voxels were excluded from ROIs on an individual basis
Correction for multiple comparisons	No correction
Statistical details	—
Findings	↑ L IFG pars triangularis<br>↑ R IFG pars triangularis<br>↓ L angular gyrus
Findings notes	Significant interaction of ROI by group

ROI analysis 2

First level contrast	Picture naming (trained items) vs rest
Analysis class	Cross-sectional aphasia vs control
Group(s)	Aphasia treated T2 (n = 26) vs control
Covariate	—
Is the second level contrast valid in terms of the group(s), time point(s), and measures involved?	Yes
Is accuracy matched across the second level contrast?	No, different
Is reaction time matched across the second level contrast?	Unknown, not reported
Behavioral data notes	—
Type of analysis	Regions of interest (ROI)
ROI type	Anatomical
How many ROIs are there?	16
What are the ROI(s)?	(1) L IFGorb; (2) L IFGtri; (3) L IFGop; (4) L MFG; (5) L PrCG; (6) L MTG; (7) L SMG; (8) L AG; (9-16) homotopic counterparts
How are the ROI(s) defined?	AAL but lesioned voxels were excluded from ROIs on an individual basis
Correction for multiple comparisons	No correction
Statistical details	—
Findings	↑ L IFG pars triangularis<br>↑ R IFG pars opercularis<br>↑ R IFG pars triangularis
Findings notes	Significant interaction of ROI by group; patients also showed more activity than controls across the average of all ROIs

ROI analysis 3

First level contrast	Picture naming (trained items) vs rest
Analysis class	Longitudinal change in aphasia
Group(s)	Aphasia untreated (n = 10) T2 vs T1
Covariate	—
Is the second level contrast valid in terms of the group(s), time point(s), and measures involved?	Yes
Is accuracy matched across the second level contrast?	Yes, matched
Is reaction time matched across the second level contrast?	Unknown, not reported
Behavioral data notes	—
Type of analysis	Regions of interest (ROI)
ROI type	Anatomical
How many ROIs are there?	16
What are the ROI(s)?	(1) L IFGorb; (2) L IFGtri; (3) L IFGop; (4) L MFG; (5) L PrCG; (6) L MTG; (7) L SMG; (8) L AG; (9-16) homotopic counterparts
How are the ROI(s) defined?	AAL but lesioned voxels were excluded from ROIs on an individual basis
Correction for multiple comparisons	No correction
Statistical details	—
Findings	None
Findings notes	No main effect of time or interaction of time by ROI

Complex analysis 1

First level contrast	Picture naming (trained items) vs rest
Analysis class	Longitudinal change in aphasia
Group(s)	Aphasia treated (n = 26) T2 vs T1
Covariate	—
Is the second level contrast valid in terms of the group(s), time point(s), and measures involved?	Yes
Is accuracy matched across the second level	No, different

contrast?	
Is reaction time matched across the second level contrast?	Unknown, not reported
Behavioral data notes	—
Type of analysis	Complex
Statistical details	A linear model was constructed to examine the relationship between proportion of spared tissue in each L hemisphere ROI and changes in activation over time. The model is not described in sufficient detail.
Findings	Other
Findings notes	There was a significant 3-way interaction of time by ROI by spared tissue, such that in some regions (AG, MFG, IFG orb, SMG), less spared tissue was associated with greater increases in activation, while in others (PrCG, IFG op, IFG tri), less spared tissue was associated with greater decreases in activation.

Notes

Excluded analyses	(1) the treated group showed an increase in activation over time averaged across all ROIs, and a near-significant interaction of time by hemisphere such that greater increases were observed in the right hemisphere; (2) "responders" showed an increase in activation over time averaged across all ROIs, while "nonresponders" did not (excluded because not anatomically specific, but also note that the definition of responders vs nonresponders was somewhat arbitrary)

Kristinsson et al. (2019)

Reference

Authors	Kristinsson S, Yourganov G, Xiao F, Bonilha L, Stark BC, Rorden C, Basilakos A, Fridriksson J
Title	Brain-derived neurotrophic factor genotype-specific differences in cortical activation in chronic aphasia
Reference	*J Speech Lang Hear Res* 2019; 62: 3923-3936
PMID	31756156
DOI	10.1044/2019_jslhr-l-rsnp-19-0021

Participants

Language	US English
Inclusion criteria	< 80% on PNT; able to name at least 5 out of 40 items during fMRI; WAB-R spontaneous speech ≥ 2; WAB-R auditory comprehension ≥ 2
Number of individuals with aphasia	87
Number of control participants	0
Were any of the participants included in any previous studies?	Yes (65 were previously included in Fridriksson et al. (2018), a tDCS study)
Is age reported for patients and controls, and matched?	Yes (typical BDNF genotype group mean 59.6 ± 11.2 years, range 29-77 years; atypical BDNF genotype group mean 57.7 ± 10.9 years, range 30-76 years)
Is sex reported for patients and controls, and matched?	Yes (males: 58; females: 29)
Is handedness reported for patients and controls, and matched?	Yes (right: 87; left: 0)
Is time post stroke onset reported and appropriate to the study design?	Yes (typical BDNF genotype group: mean 44.0 ± 38.7 months; atypical BDNF genotype group: mean 34.5 ± 36.9 months; all participants > 6 months)
To what extent is the nature of aphasia characterized?	Severity and type
Language evaluation	WAB, PNT, PPT
Aphasia severity	Typical BDNF genotype group: AQ mean 64.2 ± 20.3; atypical BDNF genotype group: AQ mean 54.3 ± 21.0
Aphasia type	Typical BDNF genotype group: 25 Broca's, 12 anomic, 11 conduction, 2 transcortical motor

	aphasia, 2 Wernicke's, 1 global; atypical BDNF genotype group: 16 Broca's, 6 anomic, 6 conduction, 3 global, 3 Wernicke's
First stroke only?	No
Stroke type	Mixed etiologies
To what extent is the lesion distribution characterized?	Lesion overlay
Lesion extent	Typical BDNF genotype group: 121.4 ± 73.2 cc; atypical BDNF genotype group: 142.2 ± 88.4 cc
Lesion location	L MCA
Participants notes	—

Imaging

Modality	fMRI
Is the study cross-sectional or longitudinal?	Cross-sectional
If longitudinal, at what time point(s) were imaging data acquired?	—
If longitudinal, was there any intervention between the time points?	—
Is the scanner described?	Yes (Siemens Trio 3 Tesla or Siemens Prisma 3 Tesla)
Is the timing of stimulus presentation and image acquisition clearly described and appropriate?	Yes
Design type	Event-related
Total images acquired	60
Are the imaging acquisition parameters, including coverage, adequately described and appropriate?	Yes (whole brain)
Is preprocessing and intrasubject coregistration adequately described and appropriate?	Yes
Is first level model fitting adequately described and appropriate?	Yes
Is intersubject normalization adequately described and appropriate?	Yes
Imaging notes	sparse sampling

Conditions

Are the conditions clearly described?	Yes

Condition	Response type	Repetitions	All groups could do?	All individuals could do?
picture naming	Word (overt)	40	Yes	Unknown
viewing abstract pictures	None	20	N/A	N/A

Conditions notes	—

Contrasts

Are the contrasts clearly described?	Yes

Contrast 1: picture naming vs viewing abstract pictures

Language condition	Picture naming
Control condition	Viewing abstract pictures
Are the conditions matched for visual demands?	Yes
Are the conditions matched for auditory demands?	No
Are the conditions matched for motor demands?	No
Are the conditions matched for cognitive/executive demands?	No
Is accuracy matched between the language and control tasks for all relevant groups?	N/A, tasks not comparable
Is reaction time matched between the language and control tasks for all relevant groups?	N/A, tasks not comparable

Behavioral data notes	—
Are control data reported in this paper or another that is referenced?	No
Does the contrast selectively activate plausible relevant language regions in the control group?	Unknown
Are activations lateralized in the control data?	Unknown
Control activation notes	—
Contrast notes	—

Analyses

Are the analyses clearly described?	No* (moderate limitation) (see specific limitation(s) below)

Voxelwise analysis 1

First level contrast	Picture naming vs viewing abstract pictures
Analysis class	Cross-sectional between two groups with aphasia
Group(s)	Aphasia with typical genotype (n = 53) vs atypical genotype (n = 34)
Covariate	—
Is the second level contrast valid in terms of the group(s), time point(s), and measures involved?	Yes
Is accuracy matched across the second level contrast?	Yes, matched
Is reaction time matched across the second level contrast?	Unknown, not reported
Behavioral data notes	—
Type of analysis	Voxelwise
Search volume	
Correction for multiple comparisons	Voxelwise FWE correction
Software	SPM12
Voxelwise p	—
Cluster extent	—
Statistical details	—
Findings	None
Findings notes	—

Notes

Excluded analyses	Comparisons between numbers of voxels activated, because not regionally specific and not described in sufficient detail

Purcell et al. (2019)

Reference

Authors	Purcell JJ, Wiley RW, Rapp B
Title	Re-learning to be different: Increased neural differentiation supports post-stroke language recovery
Reference	*NeuroImage* 2019; 202: 116145
PMID	31479754
DOI	10.1016/j.neuroimage.2019.116145

Participants

Language	US English
Inclusion criteria	Chronic dysgraphia (acquired impairment in spelling)
Number of individuals with aphasia	21 (plus 4 excluded: 3 health reasons; 1 data acquisition error)
Number of control participants	0

Were any of the participants included in any previous studies?	No
Is age reported for patients and controls, and matched?	Yes (range 40-80 years)
Is sex reported for patients and controls, and matched?	Yes (males: 13; females: 8)
Is handedness reported for patients and controls, and matched?	Yes (right: 16; left: 3; other: 2)
Is time post stroke onset reported and appropriate to the study design?	Yes (range 14-209 months)
To what extent is the nature of aphasia characterized?	Comprehensive battery
Language evaluation	Spelling (PALPA 40 and 54, and other word lists), oral reading (PALPA 35), reading comprehension (PALPA 51), spoken word-picture matching and picture naming tests from Northwestern Naming Battery, PPT-P; note no generic aphasia battery, but fairly complete coverage of language domains
Aphasia severity	Spelling of untrained items range 51%-94%
Aphasia type	4 orthographic working memory deficit, 8 orthographic long-term memory deficit, 9 both types of deficit
First stroke only?	Yes
Stroke type	Not stated
To what extent is the lesion distribution characterized?	Lesion overlay
Lesion extent	Range 7.7-215.0 cc
Lesion location	L MCA with L ventral occipitotemporal cortex mostly intact
Participants notes	—

Imaging

Modality	fMRI
Is the study cross-sectional or longitudinal?	Longitudinal—chronic treatment
If longitudinal, at what time point(s) were imaging data acquired?	T1: pre-treatment/chronic; T2: post-treatment, 6-24 weeks later
If longitudinal, was there any intervention between the time points?	Spelling treatment, 60-80 minutes/day, 2 days/week, range 6-24 weeks
Is the scanner described?	No (not stated)
Is the timing of stimulus presentation and image acquisition clearly described and appropriate?	Yes
Design type	Event-related
Total images acquired	1232 (four runs distributed over two days)
Are the imaging acquisition parameters, including coverage, adequately described and appropriate?	Yes (cerebellum excluded)
Is preprocessing and intrasubject coregistration adequately described and appropriate?	Yes
Is first level model fitting adequately described and appropriate?	No* (moderate limitation) (not feasible to separate closely spaced instruction, word, and letter/response, especially when responses will be compared to rest)
Is intersubject normalization adequately described and appropriate?	Yes
Imaging notes	—

Conditions

Are the conditions clearly described?	Yes

Condition	Response type	Repetitions	All groups could do?	All individuals could do?
spelling probe (training items)	Button press	60	Yes	Unknown
spelling probe (known items)	Button press	60	Yes	Unknown
case verification	Button press	60	Yes	Unknown

rest	None	implicit baseline	N/A	N/A

Conditions notes	Condition 3 not used in any contrasts

Contrasts

Are the contrasts clearly described?	Yes

Contrast 1: spelling probe (training items) vs rest

Language condition	Spelling probe (training items)
Control condition	Rest
Are the conditions matched for visual demands?	No
Are the conditions matched for auditory demands?	No
Are the conditions matched for motor demands?	No
Are the conditions matched for cognitive/executive demands?	No
Is accuracy matched between the language and control tasks for all relevant groups?	N/A, tasks not comparable
Is reaction time matched between the language and control tasks for all relevant groups?	N/A, tasks not comparable
Behavioral data notes	—
Are control data reported in this paper or another that is referenced?	No
Does the contrast selectively activate plausible relevant language regions in the control group?	Unknown
Are activations lateralized in the control data?	Unknown
Control activation notes	Task comes from Rapp and Lipka (2011), who report lateralized activations for the contrast of spelling probes to case verification, but do not report results relative to fixation baseline
Contrast notes	—

Contrast 2: spelling probe (known items) vs rest

Language condition	Spelling probe (known items)
Control condition	Rest
Are the conditions matched for visual demands?	No
Are the conditions matched for auditory demands?	No
Are the conditions matched for motor demands?	No
Are the conditions matched for cognitive/executive demands?	No
Is accuracy matched between the language and control tasks for all relevant groups?	N/A, tasks not comparable
Is reaction time matched between the language and control tasks for all relevant groups?	N/A, tasks not comparable
Behavioral data notes	—
Are control data reported in this paper or another that is referenced?	No
Does the contrast selectively activate plausible relevant language regions in the control group?	Unknown
Are activations lateralized in the control data?	Unknown
Control activation notes	Task comes from Rapp and Lipka (2011), who report lateralized activations for the contrast of spelling probes to case verification, but do not report results relative to fixation baseline
Contrast notes	—

Analyses

Are the analyses clearly described?	No* (moderate limitation) (see specific limitation(s) below)

Voxelwise analysis 1

First level contrast	Spelling probe (training items) vs rest
Analysis class	Longitudinal change in aphasia
Group(s)	Aphasia with both timepoints (n = 20) T2 vs T1
Covariate	—
Is the second level contrast valid in terms of the group(s), time point(s), and measures involved?	Yes
Is accuracy matched across the second level contrast?	Appear mismatched
Is reaction time matched across the second level contrast?	Appear mismatched
Behavioral data notes	See section S2, but main effects include known items also
Type of analysis	Voxelwise
Search volume	Appears to be restricted to voxels spared in all patients
Correction for multiple comparisons	Clusterwise correction based on 3dClustSim
Software	BrainVoyager QX 2.4 or SPM12
Voxelwise p	.01
Cluster extent	49 voxels (size not stated)
Statistical details	—
Findings	↑ L posterior cingulate<br>↑ R angular gyrus<br>↑ R posterior cingulate
Findings notes	—

ROI analysis 1

First level contrast	Spelling probe (training items) vs rest
Analysis class	Longitudinal correlation with language or other measure
Group(s)	Aphasia with both timepoints (n = 20) T2 vs T1
Covariate	Δ spelling accuracy on training items
Is the second level contrast valid in terms of the group(s), time point(s), and measures involved?	Yes
Is accuracy matched across the second level contrast?	Unknown, not reported
Is reaction time matched across the second level contrast?	Unknown, not reported
Behavioral data notes	—
Type of analysis	Regions of interest (ROI)
ROI type	Functional
How many ROIs are there?	3
What are the ROI(s)?	(1) R AG; (2) L PCC; (3) R PCC
How are the ROI(s) defined?	Regions activated in SPM analysis 1
Correction for multiple comparisons	No correction
Statistical details	—
Findings	None
Findings notes	—

ROI analysis 2

First level contrast	Spelling probe (training items) vs rest
Analysis class	Longitudinal correlation with language or other measure
Group(s)	Aphasia with both timepoints (n = 20) T2 vs T1
Covariate	Δ spelling accuracy on untrained items
Is the second level contrast valid in terms of the group(s), time point(s), and measures involved?	Yes
Is accuracy matched across the second level contrast?	Unknown, not reported
Is reaction time matched across the second level contrast?	Unknown, not reported

Behavioral data notes	—
Type of analysis	Regions of interest (ROI)
ROI type	Functional
How many ROIs are there?	3
What are the ROI(s)?	(1) R AG; (2) L PCC; (3) R PCC
How are the ROI(s) defined?	Regions activated in SPM analysis 1
Correction for multiple comparisons	No correction
Statistical details	—
Findings	None
Findings notes	—

ROI analysis 3

First level contrast	Spelling probe (training items) vs rest
Analysis class	Cross-sectional correlation with language or other measure
Group(s)	Aphasia T1
Covariate	Subsequent Δ spelling accuracy on training items (T2 vs T1)
Is the second level contrast valid in terms of the group(s), time point(s), and measures involved?	Somewhat (T1 behavioral measure should be included in model)
Is accuracy matched across the second level contrast?	Unknown, not reported
Is reaction time matched across the second level contrast?	Unknown, not reported
Behavioral data notes	—
Type of analysis	Region of interest (ROI)
ROI type	Functional
How many ROIs are there?	1
What are the ROI(s)?	L ventral occipitotemporal cortex
How are the ROI(s) defined?	The region that showed an increase in Local-Hreg from T1 to T2
Correction for multiple comparisons	One only
Statistical details	—
Findings	None
Findings notes	—

ROI analysis 4

First level contrast	Spelling probe (training items) vs rest
Analysis class	Cross-sectional correlation with language or other measure
Group(s)	Aphasia with both timepoints T1 (n = 20)
Covariate	Subsequent Δ spelling accuracy on untrained items (T2 vs T1)
Is the second level contrast valid in terms of the group(s), time point(s), and measures involved?	Somewhat (T1 behavioral measure should be included in model)
Is accuracy matched across the second level contrast?	Unknown, not reported
Is reaction time matched across the second level contrast?	Unknown, not reported
Behavioral data notes	—
Type of analysis	Region of interest (ROI)
ROI type	Functional
How many ROIs are there?	1
What are the ROI(s)?	L ventral occipitotemporal cortex
How are the ROI(s) defined?	The region that showed an increase in Local-Hreg from T1 to T2
Correction for multiple comparisons	One only
Statistical details	—
Findings	None
Findings notes	—

ROI analysis 5

First level contrast	Spelling probe (training items) vs rest
Analysis class	Longitudinal correlation with language or other measure
Group(s)	Aphasia with both timepoints (n = 20) T2 vs T1
Covariate	Δ spelling accuracy on training items
Is the second level contrast valid in terms of the group(s), time point(s), and measures involved?	Yes
Is accuracy matched across the second level contrast?	Unknown, not reported
Is reaction time matched across the second level contrast?	Unknown, not reported
Behavioral data notes	—
Type of analysis	Region of interest (ROI)
ROI type	Functional
How many ROIs are there?	1
What are the ROI(s)?	L ventral occipitotemporal cortex
How are the ROI(s) defined?	The region that showed an increase in Local-Hreg from T1 to T2
Correction for multiple comparisons	One only
Statistical details	—
Findings	None
Findings notes	—

ROI analysis 6

First level contrast	Spelling probe (training items) vs rest
Analysis class	Longitudinal correlation with language or other measure
Group(s)	Aphasia with both timepoints (n = 20) T2 vs T1
Covariate	Δ spelling accuracy on untrained items
Is the second level contrast valid in terms of the group(s), time point(s), and measures involved?	Yes
Is accuracy matched across the second level contrast?	Unknown, not reported
Is reaction time matched across the second level contrast?	Unknown, not reported
Behavioral data notes	—
Type of analysis	Region of interest (ROI)
ROI type	Functional
How many ROIs are there?	1
What are the ROI(s)?	L ventral occipitotemporal cortex
How are the ROI(s) defined?	The region that showed an increase in Local-Hreg from T1 to T2
Correction for multiple comparisons	One only
Statistical details	—
Findings	None
Findings notes	—

Complex analysis 1

First level contrast	Spelling probe (training items) vs rest
Analysis class	Longitudinal change in aphasia
Group(s)	Aphasia with both timepoints (n = 20) T2 vs T1
Covariate	—
Is the second level contrast valid in terms of the group(s), time point(s), and measures involved?	Yes
Is accuracy matched across the second level contrast?	Appear mismatched
Is reaction time matched across the second level contrast?	Appear mismatched

Behavioral data notes	See section S2, where Figures S1 and S2 appear to show differences; the main effects of time were not significant for accuracy or RT, but those analyses included known items also, which had smaller effects
Type of analysis	Complex
Statistical details	Local Heterogeneity Regression Analysis (Local-Hreg) was used to identify brain regions where the heterogeneity of timecourses between neighboring voxels, specifically for the trained condition, increased from T1 to T2. A voxelwise threshold of $p < 0.05$ was applied, followed by cluster correction based on permutation testing. The analysis appears to have been restricted to brain regions not damaged in any patients.
Findings	Other
Findings notes	Only in L ventral occipitotemporal cortex, there was a significant increase in Local-Hreg from T1 to T2 ($p = 0.028$, corrected).

Complex analysis 2

First level contrast	Spelling probe (known items) vs rest
Analysis class	Longitudinal change in aphasia
Group(s)	Aphasia with both timepoints (n = 20) T2 vs T1
Covariate	—
Is the second level contrast valid in terms of the group(s), time point(s), and measures involved?	Yes
Is accuracy matched across the second level contrast?	Yes, matched
Is reaction time matched across the second level contrast?	Yes, matched
Behavioral data notes	See section S2, main effects were not significant and effects appear smaller for known than trained
Type of analysis	Complex
Statistical details	Local Heterogeneity Regression Analysis (Local-Hreg) was used to identify brain regions where the heterogeneity of timecourses between neighboring voxels, specifically for the known condition, increased from T1 to T2. A voxelwise threshold of $p < 0.05$ was applied, followed by cluster correction based on permutation testing. The analysis appears to have been restricted to brain regions not damaged in any patients.
Findings	None
Findings notes	—

Complex analysis 3

First level contrast	Spelling probe (training items) vs rest
Analysis class	Cross-sectional correlation with language or other measure
Group(s)	Aphasia T1
Covariate	T1 spelling accuracy on training items
Is the second level contrast valid in terms of the group(s), time point(s), and measures involved?	Somewhat (training items were selected for individual patients, so training item accuracy is not an appropriate measure of spelling ability)
Is accuracy matched across the second level contrast?	Unknown, not reported
Is reaction time matched across the second level contrast?	Unknown, not reported
Behavioral data notes	—
Type of analysis	Complex
Statistical details	A linear mixed effects model was used to investigate the relationship between Local-Hreg at T1 in the L ventral occipitotemporal region previously identified and T1 spelling accuracy of training items. A complex model was used in which every voxel for every patient was considered an observation, with random effects of voxel and patient, but this is not described in detail.
Findings	Other
Findings notes	There was a significant positive relationship between T1 Local-Hreg and T1 spelling accuracy on training items.

Complex analysis 4

First level contrast	Spelling probe (training items) vs rest
Analysis class	Cross-sectional correlation with language or other measure
Group(s)	Aphasia T1
Covariate	Subsequent Δ spelling accuracy on training items (T2 vs T1)
Is the second level contrast valid in terms of the group(s), time point(s), and measures involved?	Somewhat (T1 behavioral measure should be included in model)
Is accuracy matched across the second level contrast?	Unknown, not reported
Is reaction time matched across the second level contrast?	Unknown, not reported
Behavioral data notes	—
Type of analysis	Complex
Statistical details	A linear mixed effects model was used to investigate the relationship between Local-Hreg at T1 in the L ventral occipitotemporal region previously identified and subsequent improvement in spelling accuracy of training items from T1 to T2. A complex model was used in which every voxel for every patient was considered an observation, with random effects of voxel and patient, but this is not described in detail.
Findings	Other
Findings notes	There was a significant positive relationship between T1 Local-Hreg and subsequent improvement in spelling accuracy on training items from T1 to T2.

Complex analysis 5

First level contrast	Spelling probe (training items) vs rest
Analysis class	Cross-sectional correlation with language or other measure
Group(s)	Aphasia with both timepoints T1 (n = 20)
Covariate	Subsequent Δ spelling accuracy on untrained items (T2 vs T1)
Is the second level contrast valid in terms of the group(s), time point(s), and measures involved?	Somewhat (T1 behavioral measure should be included in model)
Is accuracy matched across the second level contrast?	Unknown, not reported
Is reaction time matched across the second level contrast?	Unknown, not reported
Behavioral data notes	—
Type of analysis	Complex
Statistical details	A linear mixed effects model was used to investigate the relationship between Local-Hreg at T1 in the L ventral occipitotemporal region previously identified and subsequent improvement in spelling accuracy of untrained items from T1 to T2. A complex model was used in which every voxel for every patient was considered an observation, with random effects of voxel and patient, but this is not described in detail.
Findings	Other
Findings notes	There was a significant positive relationship between T1 Local-Hreg and subsequent improvement in spelling accuracy on untrained items from T1 to T2.

Complex analysis 6

First level contrast	Spelling probe (training items) vs rest
Analysis class	Longitudinal correlation with language or other measure
Group(s)	Aphasia with both timepoints (n = 20) T2 vs T1
Covariate	Δ spelling accuracy on training items
Is the second level contrast valid in terms of the group(s), time point(s), and measures involved?	Yes
Is accuracy matched across the second level contrast?	Unknown, not reported
Is reaction time matched across the second level contrast?	Unknown, not reported
Behavioral data notes	—

Type of analysis	Complex
Statistical details	A linear mixed effects model was used to investigate the relationship between change in Local-Hreg in the L ventral occipitotemporal region previously identified and change in spelling accuracy of training items. A complex model was used in which every voxel for every patient was considered an observation, with random effects of voxel and patient, but this is not described in detail.
Findings	Other
Findings notes	There was a significant negative relationship between change in Local-Hreg and change in spelling accuracy on training items.

Complex analysis 7

First level contrast	Spelling probe (training items) vs rest
Analysis class	Longitudinal correlation with language or other measure
Group(s)	Aphasia with both timepoints (n = 20) T2 vs T1
Covariate	Δ spelling accuracy on untrained items
Is the second level contrast valid in terms of the group(s), time point(s), and measures involved?	Yes
Is accuracy matched across the second level contrast?	Unknown, not reported
Is reaction time matched across the second level contrast?	Unknown, not reported
Behavioral data notes	—
Type of analysis	Complex
Statistical details	A linear mixed effects model was used to investigate the relationship between change in Local-Hreg in the L ventral occipitotemporal region previously identified and change in spelling accuracy of untrained items. A complex model was used in which every voxel for every patient was considered an observation, with random effects of voxel and patient, but this is not described in detail.
Findings	Other
Findings notes	There was a significant negative relationship between change in Local-Hreg and change in spelling accuracy on untrained items.

Complex analysis 8

First level contrast	Spelling probe (training items) vs rest
Analysis class	Cross-sectional correlation with language or other measure
Group(s)	Aphasia with both timepoints T2 (n = 20)
Covariate	T2 spelling accuracy on training items
Is the second level contrast valid in terms of the group(s), time point(s), and measures involved?	Yes
Is accuracy matched across the second level contrast?	Unknown, not reported
Is reaction time matched across the second level contrast?	Unknown, not reported
Behavioral data notes	—
Type of analysis	Complex
Statistical details	A linear mixed effects model was used to investigate the relationship between Local-Hreg at T2 in the L ventral occipitotemporal region previously identified and T2 spelling accuracy of training items. A complex model was used in which every voxel for every patient was considered an observation, with random effects of voxel and patient, but this is not described in detail.
Findings	None
Findings notes	—

Complex analysis 9

First level contrast	Spelling probe (training items) vs rest
Analysis class	Longitudinal correlation with language or other measure
Group(s)	Aphasia with both timepoints (n = 20) T2 vs T1

Covariate	Previous T1 Local-Hreg in L ventral occipitotemporal ROI
Is the second level contrast valid in terms of the group(s), time point(s), and measures involved?	No (the ROI was defined based on change in Local-Hreg, so spurious findings could arise in the absence of a real effect)
Is accuracy matched across the second level contrast?	Unknown, not reported
Is reaction time matched across the second level contrast?	Unknown, not reported
Behavioral data notes	—
Type of analysis	Complex
Statistical details	A linear mixed effects model was used to investigate the relationship between change in Local-Hreg in the L ventral occipitotemporal region previously identified and T1 Local-Hreg. A complex model was used in which every voxel for every patient was considered an observation, with random effects of voxel and patient, but this is not described in detail.
Findings	Other
Findings notes	There was a significant negative relationship between change in Local-Hreg and T1 Local-Hreg.

Complex analysis 10

First level contrast	Spelling probe (training items) vs rest
Analysis class	Longitudinal correlation with language or other measure
Group(s)	Aphasia with both timepoints (n = 20) T2 vs T1
Covariate	Δ spelling accuracy on training items
Is the second level contrast valid in terms of the group(s), time point(s), and measures involved?	Yes
Is accuracy matched across the second level contrast?	Unknown, not reported
Is reaction time matched across the second level contrast?	Unknown, not reported
Behavioral data notes	—
Type of analysis	Complex
Statistical details	A linear mixed effects model was used to investigate the relationship between change in Local-Hreg in the R AG, L PCC, and R PCC and change in spelling accuracy of training items. A complex model was used in which every voxel for every patient was considered an observation, with random effects of voxel and patient, but this is not described in detail.
Findings	None
Findings notes	—

Complex analysis 11

First level contrast	Spelling probe (training items) vs rest
Analysis class	Longitudinal correlation with language or other measure
Group(s)	Aphasia with both timepoints (n = 20) T2 vs T1
Covariate	Δ spelling accuracy on untrained items
Is the second level contrast valid in terms of the group(s), time point(s), and measures involved?	Yes
Is accuracy matched across the second level contrast?	Unknown, not reported
Is reaction time matched across the second level contrast?	Unknown, not reported
Behavioral data notes	—
Type of analysis	Complex
Statistical details	A linear mixed effects model was used to investigate the relationship between change in Local-Hreg in the R AG, L PCC, and R PCC and change in spelling accuracy of untrained items. A complex model was used in which every voxel for every patient was considered an observation, with random effects of voxel and patient, but this is not described in detail.
Findings	None
Findings notes	—

Notes

Excluded analyses	(1) confirmatory voxelwise analyses in section S4.1 and S4.2; (2) additional analyses accounting for spelling deficit type and auditory comprehension deficits described in 3.3.3; (3) relationship between overall BOLD and local heterogeneity described in 3.4.3, because not related to aphasia recovery

Sreedharan, Chandran, et al. (2019)

Reference

Authors	Sreedharan S, Chandran A, Yanamala VR, Sylaja PN, Kesavadas C, Sitaram R
Title	Self-regulation of language areas using real-time functional MRI in stroke patients with expressive aphasia
Reference	*Brain Imaging Behav* 2019; None:
PMID	31089955
DOI	10.1007/s11682-019-00106-7

Participants

Language	Malayalam
Inclusion criteria	Broca's aphasia or anomic aphasia; comprehension relatively preserved; "motivated for speech therapy"
Number of individuals with aphasia	8 (plus 3 excluded: 2 for claustrophobia; 1 for transportation issues)
Number of control participants	4
Were any of the participants included in any previous studies?	No
Is age reported for patients and controls, and matched?	No (range 18-68 years; controls were younger)
Is sex reported for patients and controls, and matched?	Yes (males: 7; females: 1)
Is handedness reported for patients and controls, and matched?	Yes (right: 8; left: 0)
Is time post stroke onset reported and appropriate to the study design?	No (6-22 weeks; patients at different subacute stages of recovery)
To what extent is the nature of aphasia characterized?	Severity only
Language evaluation	WAB translated into Malayalam
Aphasia severity	AQ range approximately 50-80
Aphasia type	Broca's or anomic
First stroke only?	Not stated
Stroke type	Not stated
To what extent is the lesion distribution characterized?	Individual lesions
Lesion extent	Not stated
Lesion location	7 L MCA, 1 bilateral MCA
Participants notes	—

Imaging

Modality	fMRI
Is the study cross-sectional or longitudinal?	Longitudinal—mixed
If longitudinal, at what time point(s) were imaging data acquired?	Neurofeedback group: T1: pre-treatment/subacute; T2: 1-5 weeks later; T3: 2-6 weeks after T1; T4: 3-11 weeks after T1; T5: 4-12 weeks after T1; T6: 5-12 weeks after T1; no training group: T1: subacute; T2: 2-12 weeks later; controls: T1: start of study; T2: 1-4 weeks later; T3: 3-5 weeks after T1; T4: 4-8 weeks after T1; T5: 7-37 weeks after T1; T6: 12-43 weeks after T1
If longitudinal, was there any intervention between the time points?	4 patients received 4 additional sessions involving neurofeedback training, while 4 patients received treatment as usual
Is the scanner described?	Yes (Siemens Avanto 1.5 Tesla)

Is the timing of stimulus presentation and image acquisition clearly described and appropriate?	No* (moderate limitation) (picture naming events consistently located between blocks)
Design type	Mixed
Total images acquired	probably 964
Are the imaging acquisition parameters, including coverage, adequately described and appropriate?	Yes (whole brain)
Is preprocessing and intrasubject coregistration adequately described and appropriate?	Yes
Is first level model fitting adequately described and appropriate?	No* (moderate limitation) (event timing will make conditions difficult to disentangle)
Is intersubject normalization adequately described and appropriate?	No (lesion impact not addressed)
Imaging notes	—

Conditions

Are the conditions clearly described?	Yes

Condition	Response type	Repetitions	All groups could do?	All individuals could do?
neurofeedback (try to activate language areas)	Other	24	Unknown	Unknown
rest	None	24	N/A	N/A
picture naming	Other	first and last timepoints: 48; other timepoints: 0	No	No
word generation	Multiple words (covert)	5	Unknown	Unknown

Conditions notes	Suggested strategies to activate language areas included "making a speech, having a conversation, reciting a poem or any other form of language activity performed covertly"; picture naming task involved covert word response and button press; picture naming task not used in any contrast; word generation task used only to generate ROIs

Contrasts

Are the contrasts clearly described?	Yes

Contrast 1: neurofeedback (try to activate language areas) vs rest

Language condition	Neurofeedback (try to activate language areas)
Control condition	Rest
Are the conditions matched for visual demands?	No
Are the conditions matched for auditory demands?	Yes
Are the conditions matched for motor demands?	Yes
Are the conditions matched for cognitive/executive demands?	No
Is accuracy matched between the language and control tasks for all relevant groups?	N/A, tasks not comparable
Is reaction time matched between the language and control tasks for all relevant groups?	N/A, tasks not comparable
Behavioral data notes	—
Are control data reported in this paper or another that is referenced?	Somewhat
Does the contrast selectively activate plausible relevant language regions in the control group?	Unknown
Are activations lateralized in the control data?	No
Control activation notes	Task activated L IFG and L STG in controls (Fig. 8c), but no data on other regions, and language activations were not lateralized (Fig. 9d)
Contrast notes	—

Analyses

Are the analyses clearly described?	No* (moderate limitation) (see specific limitation(s) below)

ROI analysis 1

First level contrast	Neurofeedback (try to activate language areas) vs rest
Analysis class	Cross-sectional aphasia vs control
Group(s)	Aphasia mean of T1, T2, T3, T4, T5, T6 (neurofeedback patients) or T1, T2 (no training patients) vs control mean
Covariate	—
Is the second level contrast valid in terms of the group(s), time point(s), and measures involved?	Yes
Is accuracy matched across the second level contrast?	N/A, no behavioral measure
Is reaction time matched across the second level contrast?	N/A, no timeable task
Behavioral data notes	—
Type of analysis	Regions of interest (ROI)
ROI type	Functional
How many ROIs are there?	4
What are the ROI(s)?	(1) L Broca's area (IFG pars opercularis and triangularis); (2) L Wernicke's area (pSTG); (3-4) homotopic counterparts
How are the ROI(s) defined?	Individual activations within AAL ROIs on a separate word generation localizer
Correction for multiple comparisons	No direct comparison
Statistical details	—
Findings	↓ L IFG pars opercularis<br>↓ L IFG pars triangularis<br>↓ L posterior STG<br>↓ R IFG pars opercularis<br>↓ R IFG pars triangularis<br>↓ R posterior STG
Findings notes	—

ROI analysis 2

First level contrast	Neurofeedback (try to activate language areas) vs rest
Analysis class	Cross-sectional between two groups with aphasia
Group(s)	Aphasia with neurofeedback training (n = 4) mean of T4, T5, T6 vs no training (n = 4) T2
Covariate	—
Is the second level contrast valid in terms of the group(s), time point(s), and measures involved?	Somewhat (no treatment effect; second half measures rather than measures of change)
Is accuracy matched across the second level contrast?	N/A, no behavioral measure
Is reaction time matched across the second level contrast?	N/A, no timeable task
Behavioral data notes	—
Type of analysis	Regions of interest (ROI)
ROI type	Functional
How many ROIs are there?	15
What are the ROI(s)?	(1) L Broca's area (IFG pars opercularis and triangularis); (2) L Wernicke's area (pSTG); (3-4) homotopic counterparts; (5) L MFG; (6) L PrCG; (7) L Rolandic operculum; (8) L insula; (9) L IFG pars orbitalis; (10) L MFG orbital; (11) L SMG; (12) L MTG; (13) L PoCG; (14) L AG; (15) L HG
How are the ROI(s) defined?	(1-4) individual activations within AAL ROIs on a separate word generation localizer; (5-15) AAL
Correction for multiple comparisons	No correction
Statistical details	—
Findings	↑ L ventral precentral/inferior frontal junction<br>↑ L somato-motor
Findings notes	—

Complex analysis 1

First level contrast	Neurofeedback (try to activate language areas) vs rest
Analysis class	Cross-sectional aphasia vs control
Group(s)	Aphasia mean of T1, T2, T3, T4, T5, T6 (neurofeedback patients) or T1, T2 (no training patients) vs control mean
Covariate	—
Is the second level contrast valid in terms of the group(s), time point(s), and measures involved?	Yes
Is accuracy matched across the second level contrast?	N/A, no behavioral measure
Is reaction time matched across the second level contrast?	N/A, no timeable task
Behavioral data notes	—
Type of analysis	Complex
Statistical details	Signal change in L IFG and L pSTG ROIs was computed, along with functional connectivity between these ROIs. Neurofeedback values were calculated based on signal change as well as correlation between the ROIs. Group differences in neurofeedback values were compared, but not quantified statistically.
Findings	Other
Findings notes	Patients received lower neurofeedback values than controls, due to lower signal changes and lower functional connectivity.

Notes

Excluded analyses	(1) individual participant analyses in Fig. 10; (2) comparisons between groups at each time point (Fig. 11), which yielded similar results to comparisons averaged across time points; (3) vague statements about temporal trends in Figs. 12, 13, and 14

Hartwigsen et al. (2020)

Reference

Authors	Hartwigsen G, Stockert A, Charpentier L, Wawrzyniak M, Klingbeil J, Wrede K, Obrig H, Saur
Title	Short-term modulation of the lesioned language network
Reference	*eLife* 2020; 9: e54277
PMID	32181741
DOI	10.7554/elife.54277

Participants

Language	German
Inclusion criteria	Lesion involving left temporo-parietal cortex and sparing left frontal cortex; relatively well-recovered
Number of individuals with aphasia	12 (plus 2 excluded: 1 lost to follow-up; 1 did not show any sound-related neural activation in auditory cortex after sham cTBS)
Number of control participants	0
Were any of the participants included in any previous studies?	No
Is age reported for patients and controls, and matched?	Yes (mean 58.8 years, range 43-72 years)
Is sex reported for patients and controls, and matched?	Yes (males: 8; females: 4)
Is handedness reported for patients and controls, and matched?	Yes (right: 12; left: 0)
Is time post stroke onset reported and appropriate to the study design?	Yes (mean 37.9 ± 34.8 months, range 6-122 months)
To what extent is the nature of aphasia	Not at all

characterized?	
Language evaluation	AAT
Aphasia severity	7 mild residual aphasia, 5 recovered
Aphasia type	Not stated
First stroke only?	Yes
Stroke type	Ischemic only
To what extent is the lesion distribution characterized?	Lesion overlay
Lesion extent	Range 11.9-176.3 cc
Lesion location	Left temporo-parietal cortex; maximal overlap in SMG
Participants notes	—

Imaging

Modality	fMRI
Is the study cross-sectional or longitudinal?	Longitudinal—chronic treatment
If longitudinal, at what time point(s) were imaging data acquired?	T1/T2/T3: chronic; sessions consisted of cTBS over left anterior IFG, cTBS over left posterior IFG, or sham; sessions at least 7 days apart in randomized order
If longitudinal, was there any intervention between the time points?	CTBS
Is the scanner described?	Yes (Siemens Verio 3 Tesla)
Is the timing of stimulus presentation and image acquisition clearly described and appropriate?	No* (moderate limitation) (stimulus timing not described in detail; stated duration of data acquisition substantially outside possible range of duration of stimuli)
Design type	Block
Total images acquired	740
Are the imaging acquisition parameters, including coverage, adequately described and appropriate?	Yes (whole brain)
Is preprocessing and intrasubject coregistration adequately described and appropriate?	Yes
Is first level model fitting adequately described and appropriate?	Yes
Is intersubject normalization adequately described and appropriate?	No (lesion impact not addressed)
Imaging notes	—

Conditions

Are the conditions clearly described?	Yes

Condition	Response type	Repetitions	All groups could do?	All individuals could do?
syllable count decision	Button press	10	Yes	Yes
semantic decision	Button press	10	Yes	Yes
rest	None	20	N/A	N/A

Conditions notes	Extent of recovery supports the assertion that all individuals could do the tasks

Contrasts

Are the contrasts clearly described?	Yes

Contrast 1: syllable count decision vs rest

Language condition	Syllable count decision
Control condition	Rest
Are the conditions matched for visual demands?	Yes
Are the conditions matched for auditory demands?	No
Are the conditions matched for motor demands?	No
Are the conditions matched for cognitive/executive demands?	No

Is accuracy matched between the language and control tasks for all relevant groups?	N/A, tasks not comparable
Is reaction time matched between the language and control tasks for all relevant groups?	N/A, tasks not comparable
Behavioral data notes	—
Are control data reported in this paper or another that is referenced?	Yes
Does the contrast selectively activate plausible relevant language regions in the control group?	Yes
Are activations lateralized in the control data?	Somewhat
Control activation notes	Control data in Hartwigsen et al. (2017); L-lateralized IFG but bilateral SMG
Contrast notes	—

Contrast 2: semantic decision vs rest

Language condition	Semantic decision
Control condition	Rest
Are the conditions matched for visual demands?	Yes
Are the conditions matched for auditory demands?	No
Are the conditions matched for motor demands?	No
Are the conditions matched for cognitive/executive demands?	No
Is accuracy matched between the language and control tasks for all relevant groups?	N/A, tasks not comparable
Is reaction time matched between the language and control tasks for all relevant groups?	N/A, tasks not comparable
Behavioral data notes	—
Are control data reported in this paper or another that is referenced?	Yes
Does the contrast selectively activate plausible relevant language regions in the control group?	Yes
Are activations lateralized in the control data?	Yes
Control activation notes	Control data in Hartwigsen et al. (2017); L-lateralized IFG and AG most prominent
Contrast notes	—

Analyses

Are the analyses clearly described?	Yes

Voxelwise analysis 1

First level contrast	Syllable count decision vs rest
Analysis class	Cross-sectional between two groups with aphasia
Group(s)	Aphasia after cTBS to posterior IFG vs sham; same patients, repeated measures
Covariate	—
Is the second level contrast valid in terms of the group(s), time point(s), and measures involved?	Yes
Is accuracy matched across the second level contrast?	Yes, matched
Is reaction time matched across the second level contrast?	No, different
Behavioral data notes	Significantly slower response times when cTBS was applied over pIFG relative to when sham cTBS was applied
Type of analysis	Voxelwise
Search volume	Voxels spared in all patients
Correction for multiple comparisons	Clusterwise correction with with GRFT and stringent voxelwise p
Software	SPM12
Voxelwise p	.001
Cluster extent	Based on GRFT

Statistical details	—
Findings	↓ L IFG pars opercularis<br>↓ L SMA/medial prefrontal<br>↓ R SMA/medial prefrontal<br>↓ R basal ganglia
Findings notes	Based on Figure 4A and Table 3

Voxelwise analysis 2

First level contrast	Syllable count decision vs rest
Analysis class	Cross-sectional between two groups with aphasia
Group(s)	Aphasia after cTBS to posterior IFG vs after cTBS to anterior IFG; same patients, repeated measures
Covariate	—
Is the second level contrast valid in terms of the group(s), time point(s), and measures involved?	Yes
Is accuracy matched across the second level contrast?	Yes, matched
Is reaction time matched across the second level contrast?	No, different
Behavioral data notes	Significantly slower response times when cTBS was applied over pIFG relative to when cTBS was applied over aIFG
Type of analysis	Voxelwise
Search volume	Voxels spared in all patients
Correction for multiple comparisons	Clusterwise correction with with GRFT and stringent voxelwise p
Software	SPM12
Voxelwise p	.001
Cluster extent	Based on GRFT
Statistical details	—
Findings	↓ L IFG pars opercularis
Findings notes	Based on Table 3

Voxelwise analysis 3

First level contrast	Semantic decision vs rest
Analysis class	Cross-sectional between two groups with aphasia
Group(s)	Aphasia after cTBS to anterior IFG vs sham; same patients, repeated measures
Covariate	—
Is the second level contrast valid in terms of the group(s), time point(s), and measures involved?	Somewhat (no behavioral difference)
Is accuracy matched across the second level contrast?	Yes, matched
Is reaction time matched across the second level contrast?	Yes, matched
Behavioral data notes	Difference in reaction time did not survive correction
Type of analysis	Voxelwise
Search volume	Voxels spared in all patients
Correction for multiple comparisons	Clusterwise correction with with GRFT and stringent voxelwise p
Software	SPM12
Voxelwise p	.001
Cluster extent	Based on GRFT
Statistical details	—
Findings	↓ L insula<br>↓ L dorsolateral prefrontal cortex<br>↓ R insula<br>↓ R dorsolateral prefrontal cortex<br>↓ R SMA/medial prefrontal
Findings notes	Based on Figure 4B and Table 3

Voxelwise analysis 4

First level contrast	Semantic decision vs rest
Analysis class	Cross-sectional between two groups with aphasia
Group(s)	Aphasia after cTBS to anterior IFG vs after cTBS to posterior IFG ; same patients, repeated measures
Covariate	—
Is the second level contrast valid in terms of the group(s), time point(s), and measures involved?	Yes
Is accuracy matched across the second level contrast?	Yes, matched
Is reaction time matched across the second level contrast?	No, different
Behavioral data notes	Significantly slower response times when cTBS was applied over aIFG relative to when cTBS was applied over pIFG
Type of analysis	Voxelwise
Search volume	Voxels spared in all patients
Correction for multiple comparisons	Clusterwise correction with with GRFT and stringent voxelwise p
Software	SPM12
Voxelwise p	.001
Cluster extent	Based on GRFT
Statistical details	—
Findings	↓ L insula<br>↓ R insula<br>↓ R dorsolateral prefrontal cortex
Findings notes	Based on Table 3

Complex analysis 1

First level contrast	Syllable count decision vs rest
Analysis class	Cross-sectional correlation with language or other measure
Group(s)	Aphasia after cTBS to posterior IFG vs sham; same patients, repeated measures
Covariate	Δ RT for syllable decision (cTBS to posterior IFG timepoint vs sham timepoint)
Is the second level contrast valid in terms of the group(s), time point(s), and measures involved?	Yes
Is accuracy matched across the second level contrast?	Unknown, not reported
Is reaction time matched across the second level contrast?	RT is covariate
Behavioral data notes	—
Type of analysis	Complex
Statistical details	Whole brain correlations were computed between the difference in functional activity after cTBS to posterior IFG versus sham stimulation, and the difference in reaction times on the syllable counting task under these two conditions. The resulting SPM was thresholded at voxelwise $p < .001$ (CDT) followed by correction for multiple comparisons based on cluster extent and GRFT using SPM12.
Findings	Other
Findings notes	Upregulation of the R supramarginal gyrus after cTBS was significantly associated with slowing of RT after cTBS. This finding remained significant after including lesion volume as covariate.

Complex analysis 2

First level contrast	Semantic decision vs rest
Analysis class	Cross-sectional correlation with language or other measure
Group(s)	Aphasia after cTBS to anterior IFG vs sham; same patients, repeated measures
Covariate	Δ RT for semantic decision (cTBS to posterior IFG timepoint vs sham timepoint)
Is the second level contrast valid in terms of the group(s), time point(s), and measures involved?	Yes
Is accuracy matched across the second level	Unknown, not reported

contrast?	
Is reaction time matched across the second level contrast?	RT is covariate
Behavioral data notes	—
Type of analysis	Complex
Statistical details	Whole brain correlations were computed between the difference in functional activity after cTBS to anterior IFG versus sham stimulation, and the difference in reaction times on the semantic decision task under these two conditions. The resulting SPM was thresholded at voxelwise p < .001 (CDT) followed by correction for multiple comparisons based on cluster extent and GRFT using SPM12.
Findings	None
Findings notes	—

Notes

Excluded analyses	—

Stockert et al. (2020)

Reference

Authors	Stockert A, Wawrzyniak M, Klingbeil J, Wrede K, Kümmerer D, Hartwigsen G, Kaller CP, Weiller C, Saur D
Title	Dynamics of language reorganization after left temporo-parietal and frontal stroke
Reference	*Brain* 2020; 143: 844-861
PMID	32068789
DOI	10.1093/brain/awaa023

Participants

Language	German
Inclusion criteria	Lesion localized to frontal or temporal cortex
Number of individuals with aphasia	34 (plus 50 excluded: 19 lesions spanned frontal and temporal, or were subcortical, or had persisting large vessel occlusions; 31 not all three timepoints were acquired)
Number of control participants	17
Were any of the participants included in any previous studies?	Yes (8 patients were included in Saur et al. (2006); there may also be overlap with Saur et al. (2010), a study that did not meet our inclusion criteria)
Is age reported for patients and controls, and matched?	Yes (frontal group: mean 52.3 ± 18.9 years, range 15-78 years; temporo-parietal group: mean 54.4 ± 12.7 years, range 31-76 years)
Is sex reported for patients and controls, and matched?	Yes (males: 25; females: 9)
Is handedness reported for patients and controls, and matched?	No (right: 31; left: 2; other: 1; not stated for controls)
Is time post stroke onset reported and appropriate to the study design?	Yes (frontal group: T1 acute: mean 3.2 ± 2.0 days, range 1-7 days; T2 subacute: mean 11.9 ± 2.2 days, range 8-17 days; T3 chronic: mean 272.6 ± 88.5 days, range 181-435 days; temporo-parietal group: T1 acute: mean 1.6 ± 0.8 days, range 1-4 days; T2 subacute: mean 10.1 ± 1.7 days, range 8-13 days; T3 chronic: mean 262.5 ± 75.0 days, range 184-394 days)
To what extent is the nature of aphasia characterized?	Severity only
Language evaluation	AAT including TT, comprehension composite (LRScomp) and production composite (LRSprod) were derived
Aphasia severity	Frontal group: T1 acute: LRScomp mean 0.48 ± 0.26; T2 subacute: LRScomp mean 0.64 ± 0.21; T3 chronic: LRScomp mean 0.91 ± 0.07; temporo-parietal group: T1 acute: LRScomp mean 0.63 ± 0.32; T2 subacute: LRScomp mean 0.79 ± 0.20; T3 chronic: LRScomp mean 0.91 ± 0.13
Aphasia type	Not stated
First stroke only?	Yes
Stroke type	Ischemic only

To what extent is the lesion distribution characterized?	Lesion overlay
Lesion extent	Frontal group: mean 69.3 ± 34.0 cc, range 12.3-76.6 cc; temporo-parietal group: mean 54.8 ± 41.1 cc, range 6.2-108.5 cc
Lesion location	L MCA, frontal (n = 17) or temporo-parietal (n = 17)
Participants notes	1630 patients screened for inclusion; frontal patients scanned later than temporal patients at T1 and T2

Imaging

Modality	fMRI
Is the study cross-sectional or longitudinal?	Longitudinal—recovery
If longitudinal, at what time point(s) were imaging data acquired?	T1 acute: 1-7 days; T2 subacute: 8-21 days; T3 chronic: > 6 months
If longitudinal, was there any intervention between the time points?	Not stated
Is the scanner described?	Yes (Siemens Trio 3 Tesla or Siemens Verio 3 Tesla)
Is the timing of stimulus presentation and image acquisition clearly described and appropriate?	Yes
Design type	Event-related
Total images acquired	660 (20 patients; paradigm 1) or 260 (14 patients; paradigm 2)
Are the imaging acquisition parameters, including coverage, adequately described and appropriate?	No (whole brain; TE = 96 ms questionable)
Is preprocessing and intrasubject coregistration adequately described and appropriate?	Yes
Is first level model fitting adequately described and appropriate?	Yes
Is intersubject normalization adequately described and appropriate?	Yes
Imaging notes	—

Conditions

Are the conditions clearly described?	No (description implies that paradigm 2 did not include a semantically anomalous condition, but previous papers indicate that it did)

Condition	Response type	Repetitions	All groups could do?	All individuals could do?
listening to normal sentences and making a plausibility judgment (paradigm 1)	None	46	Unknown	Unknown
listening to semantically anomalous sentences and making a plausibility judgment (paradigm 1)	Button press	46	Unknown	Unknown
listening to reversed speech	Button press	paradigm 1: 92; paradigm 2: 30	Yes	Unknown
listening to normal sentences (paradigm 2)	Button press	15	Yes	Unknown
listening to semantically anomalous sentences (paradigm 2)	Button press	15	Yes	Unknown
listening to pseudoword speech (paradigm 2)	Button press	30	Yes	Unknown
rest	None	implicit baseline	N/A	N/A

Conditions notes	Conditions 2, 5, and 6 were not used, and condition 7 was effectively contrasted out; reported behavioral data collapses across conditions and paradigms and so does not establish performance on any specific condition, but the data suggest that at least the conditions where no language-related decisions were required could have been performed by all groups

Contrasts

Are the contrasts clearly described?	No (see specific limitation(s) below)

Contrast 1: listening to normal sentences and making a plausibility judgment (paradigm 1) or listening to normal sentences (paradigm 2) vs listening to reversed speech

Language condition	Listening to normal sentences and making a plausibility judgment (paradigm 1) or listening to normal sentences (paradigm 2)
Control condition	Listening to reversed speech
Are the conditions matched for visual demands?	Yes
Are the conditions matched for auditory demands?	Yes
Are the conditions matched for motor demands?	No
Are the conditions matched for cognitive/executive demands?	No
Is accuracy matched between the language and control tasks for all relevant groups?	Unknown, not reported
Is reaction time matched between the language and control tasks for all relevant groups?	N/A, tasks not comparable
Behavioral data notes	In paradigm 1, responses were required in the language condition but not the control condition, making the tasks not comparable for RT
Are control data reported in this paper or another that is referenced?	Somewhat
Does the contrast selectively activate plausible relevant language regions in the control group?	Yes
Are activations lateralized in the control data?	Yes
Control activation notes	Not stated which of the two paradigms controls were run on, but clearly L-lateralized frontal and temporal activation; bilateral MD network activation also noted
Contrast notes	20 patients performed paradigm 1 and 14 patients performed paradigm 2; data were combined despite some differences; unclear whether all reversed speech was included, or only reversed speech derived from plausible sentences

Analyses

Are the analyses clearly described?	Yes

ROI analysis 1

First level contrast	Listening to normal sentences and making a plausibility judgment (paradigm 1) or listening to normal sentences (paradigm 2) vs listening to reversed speech
Analysis class	Longitudinal change in aphasia
Group(s)	Aphasia T2 vs T1
Covariate	—
Is the second level contrast valid in terms of the group(s), time point(s), and measures involved?	Yes
Is accuracy matched across the second level contrast?	Unknown, not reported
Is reaction time matched across the second level contrast?	Unknown, not reported
Behavioral data notes	No differences in proportion of expected button presses by group or time, but behavioral data pooled across conditions
Type of analysis	Regions of interest (ROI)
ROI type	Functional
How many ROIs are there?	13
What are the ROI(s)?	(1) L IFG orb; (2) L IFG tri; (3) L IFG op; (4) L DLPFC; (5) L insula; (6) L ATL; (7) L PTL; (8) L SMA/dACC; (9) R L IFG orb; (10) R IFG tri; (11) R insula; (12) R DLPFC; (13) R ATL
How are the ROI(s) defined?	Spheres around peaks of whole brain analysis of all patients collapsing across groups and timepoints
Correction for multiple comparisons	No correction
Statistical details	Post-hoc tests comparing 2 out of the 3 time points were corrected using the Bonferroni-Holm procedure, but there is no indication that that multiple comparisons across ROIs were accounted for
Findings	↑ L IFG pars orbitalis

	↑ L insula<br>↑ L dorsolateral prefrontal cortex<br>↑ L SMA/medial prefrontal<br>↑ R insula
Findings notes	Based on Figure 3; several additional regions are mentioned in text and/or Table 1

ROI analysis 2

First level contrast	Listening to normal sentences and making a plausibility judgment (paradigm 1) or listening to normal sentences (paradigm 2) vs listening to reversed speech
Analysis class	Longitudinal change in aphasia
Group(s)	Aphasia T3 vs T1
Covariate	—
Is the second level contrast valid in terms of the group(s), time point(s), and measures involved?	Yes
Is accuracy matched across the second level contrast?	Unknown, not reported
Is reaction time matched across the second level contrast?	Unknown, not reported
Behavioral data notes	No differences in proportion of expected button presses by group or time, but behavioral data pooled across conditions
Type of analysis	Regions of interest (ROI)
ROI type	Functional
How many ROIs are there?	13
What are the ROI(s)?	(1) L IFG orb; (2) L IFG tri; (3) L IFG op; (4) L DLPFC; (5) L insula; (6) L ATL; (7) L PTL; (8) L SMA/dACC; (9) R L IFG orb; (10) R IFG tri; (11) R insula; (12) R DLPFC; (13) R ATL
How are the ROI(s) defined?	Spheres around peaks of whole brain analysis of all patients collapsing across groups and timepoints
Correction for multiple comparisons	No correction
Statistical details	Post-hoc tests comparing 2 out of the 3 time points were corrected using the Bonferroni-Holm procedure, but there is no indication that that multiple comparisons across ROIs were accounted for
Findings	↑ L IFG pars orbitalis<br>↑ L dorsolateral prefrontal cortex<br>↑ L posterior STG/STS/MTG<br>↑ L anterior temporal
Findings notes	Based on Figure 3; several additional regions are mentioned in text and/or Table 1

ROI analysis 3

First level contrast	Listening to normal sentences and making a plausibility judgment (paradigm 1) or listening to normal sentences (paradigm 2) vs listening to reversed speech
Analysis class	Longitudinal change in aphasia
Group(s)	Aphasia T3 vs T2
Covariate	—
Is the second level contrast valid in terms of the group(s), time point(s), and measures involved?	Yes
Is accuracy matched across the second level contrast?	Unknown, not reported
Is reaction time matched across the second level contrast?	Unknown, not reported
Behavioral data notes	No differences in proportion of expected button presses by group or time, but behavioral data pooled across conditions
Type of analysis	Regions of interest (ROI)
ROI type	Functional
How many ROIs are there?	13
What are the ROI(s)?	(1) L IFG orb; (2) L IFG tri; (3) L IFG op; (4) L DLPFC; (5) L insula; (6) L ATL; (7) L PTL; (8) L SMA/dACC; (9) R L IFG orb; (10) R IFG tri; (11) R insula; (12) R DLPFC; (13) R ATL
How are the ROI(s) defined?	Spheres around peaks of whole brain analysis of all patients collapsing across groups and

	timepoints
Correction for multiple comparisons	No correction
Statistical details	Post-hoc tests comparing 2 out of the 3 time points were corrected using the Bonferroni-Holm procedure, but there is no indication that that multiple comparisons across ROIs were accounted for
Findings	None
Findings notes	Based on Figure 3; several additional regions are mentioned in text and/or Table 1

ROI analysis 4

First level contrast	Listening to normal sentences and making a plausibility judgment (paradigm 1) or listening to normal sentences (paradigm 2) vs listening to reversed speech
Analysis class	Cross-sectional between two groups with aphasia
Group(s)	Aphasia frontal mean of T1, T2, T3 (n = 17) vs temporo-parietal mean of T1, T2, T3 (n = 17)
Covariate	—
Is the second level contrast valid in terms of the group(s), time point(s), and measures involved?	Yes
Is accuracy matched across the second level contrast?	Unknown, not reported
Is reaction time matched across the second level contrast?	Unknown, not reported
Behavioral data notes	No differences in proportion of expected button presses by group or time, but behavioral data pooled across conditions
Type of analysis	Regions of interest (ROI)
ROI type	Functional
How many ROIs are there?	13
What are the ROI(s)?	(1) L IFG orb; (2) L IFG tri; (3) L IFG op; (4) L DLPFC; (5) L insula; (6) L ATL; (7) L PTL; (8) L SMA/dACC; (9) R L IFG orb; (10) R IFG tri; (11) R insula; (12) R DLPFC; (13) R ATL
How are the ROI(s) defined?	Spheres around peaks of whole brain analysis of all patients collapsing across groups and timepoints
Correction for multiple comparisons	No correction
Statistical details	—
Findings	↑ L posterior STG/STS/MTG<br>↑ R IFG pars orbitalis<br>↑ R anterior temporal<br>↓ L IFG pars opercularis<br>↓ L IFG pars triangularis<br>↓ L dorsolateral prefrontal cortex
Findings notes	Based on Table 1

ROI analysis 5

First level contrast	Listening to normal sentences and making a plausibility judgment (paradigm 1) or listening to normal sentences (paradigm 2) vs listening to reversed speech
Analysis class	Longitudinal between two groups with aphasia
Group(s)	(Aphasia frontal (n = 17) T2 vs T1) vs (temporo-parietal (n = 17) T2 vs T1)
Covariate	—
Is the second level contrast valid in terms of the group(s), time point(s), and measures involved?	Yes
Is accuracy matched across the second level contrast?	Unknown, not reported
Is reaction time matched across the second level contrast?	Unknown, not reported
Behavioral data notes	No differences in proportion of expected button presses by group or time, but behavioral data pooled across conditions
Type of analysis	Regions of interest (ROI)
ROI type	Functional
How many ROIs are there?	13
What are the ROI(s)?	(1) L IFG orb; (2) L IFG tri; (3) L IFG op; (4) L DLPFC; (5) L insula; (6) L ATL; (7) L PTL; (8) L

	SMA/dACC; (9) R L IFG orb; (10) R IFG tri; (11) R insula; (12) R DLPFC; (13) R ATL
How are the ROI(s) defined?	Spheres around peaks of whole brain analysis of all patients collapsing across groups and timepoints
Correction for multiple comparisons	No correction
Statistical details	Interactions were significant in model with all 3 time points; post-hoc sub-interactions not reported but the patterns appear clear
Findings	↓ L IFG pars opercularis<br>↓ L IFG pars triangularis<br>↓ R IFG pars triangularis<br>↓ R dorsolateral prefrontal cortex
Findings notes	—

ROI analysis 6

First level contrast	Listening to normal sentences and making a plausibility judgment (paradigm 1) or listening to normal sentences (paradigm 2) vs listening to reversed speech
Analysis class	Longitudinal between two groups with aphasia
Group(s)	(Aphasia frontal (n = 17) T3 vs T1) vs (temporo-parietal (n = 17) T3 vs T1)
Covariate	—
Is the second level contrast valid in terms of the group(s), time point(s), and measures involved?	Yes
Is accuracy matched across the second level contrast?	Unknown, not reported
Is reaction time matched across the second level contrast?	Unknown, not reported
Behavioral data notes	No differences in proportion of expected button presses by group or time, but behavioral data pooled across conditions
Type of analysis	Regions of interest (ROI)
ROI type	Functional
How many ROIs are there?	13
What are the ROI(s)?	(1) L IFG orb; (2) L IFG tri; (3) L IFG op; (4) L DLPFC; (5) L insula; (6) L ATL; (7) L PTL; (8) L SMA/dACC; (9) R L IFG orb; (10) R IFG tri; (11) R insula; (12) R DLPFC; (13) R ATL
How are the ROI(s) defined?	Spheres around peaks of whole brain analysis of all patients collapsing across groups and timepoints
Correction for multiple comparisons	No correction
Statistical details	Interactions were significant in model with all 3 time points; post-hoc sub-interactions not reported and patterns are not clear
Findings	↓ L IFG pars opercularis<br>↓ L IFG pars triangularis<br>↓ R IFG pars triangularis<br>↓ R dorsolateral prefrontal cortex
Findings notes	—

ROI analysis 7

First level contrast	Listening to normal sentences and making a plausibility judgment (paradigm 1) or listening to normal sentences (paradigm 2) vs listening to reversed speech
Analysis class	Longitudinal between two groups with aphasia
Group(s)	(Aphasia frontal (n = 17) T3 vs T2) vs (temporo-parietal (n = 17) T3 vs T2)
Covariate	—
Is the second level contrast valid in terms of the group(s), time point(s), and measures involved?	Yes
Is accuracy matched across the second level contrast?	Unknown, not reported
Is reaction time matched across the second level contrast?	Unknown, not reported
Behavioral data notes	No differences in proportion of expected button presses by group or time, but behavioral data pooled across conditions
Type of analysis	Regions of interest (ROI)

ROI type	Functional
How many ROIs are there?	13
What are the ROI(s)?	(1) L IFG orb; (2) L IFG tri; (3) L IFG op; (4) L DLPFC; (5) L insula; (6) L ATL; (7) L PTL; (8) L SMA/dACC; (9) R L IFG orb; (10) R IFG tri; (11) R insula; (12) R DLPFC; (13) R ATL
How are the ROI(s) defined?	Spheres around peaks of whole brain analysis of all patients collapsing across groups and timepoints
Correction for multiple comparisons	No correction
Statistical details	Post-hoc sub-interactions not reported but there do not appear to be any T2/T3 effects
Findings	None
Findings notes	—

ROI analysis 8

First level contrast	Listening to normal sentences and making a plausibility judgment (paradigm 1) or listening to normal sentences (paradigm 2) vs listening to reversed speech
Analysis class	Longitudinal change in aphasia
Group(s)	Aphasia T2 vs T1
Covariate	—
Is the second level contrast valid in terms of the group(s), time point(s), and measures involved?	Yes
Is accuracy matched across the second level contrast?	Unknown, not reported
Is reaction time matched across the second level contrast?	Unknown, not reported
Behavioral data notes	No differences in proportion of expected button presses by group or time, but behavioral data pooled across conditions
Type of analysis	Regions of interest (ROI)
ROI type	Other
How many ROIs are there?	2
What are the ROI(s)?	(1) perilesional tissue; (2) regions homotopic to lesions; each unique to individuals
How are the ROI(s) defined?	(1) perilesional ROIs were voxels 3-15 mm from the lesion that were located in frontal or temporal regions activated by the language contrast in controls; (2) homotopic ROIs were flipped lesions
Correction for multiple comparisons	No correction
Statistical details	Test of group by time interaction not reported
Findings	Other
Findings notes	There was a significant increase in activation in perilesional ROIs

ROI analysis 9

First level contrast	Listening to normal sentences and making a plausibility judgment (paradigm 1) or listening to normal sentences (paradigm 2) vs listening to reversed speech
Analysis class	Longitudinal change in aphasia
Group(s)	Aphasia T3 vs T1
Covariate	—
Is the second level contrast valid in terms of the group(s), time point(s), and measures involved?	Yes
Is accuracy matched across the second level contrast?	Unknown, not reported
Is reaction time matched across the second level contrast?	Unknown, not reported
Behavioral data notes	No differences in proportion of expected button presses by group or time, but behavioral data pooled across conditions
Type of analysis	Regions of interest (ROI)
ROI type	Other
How many ROIs are there?	2
What are the ROI(s)?	(1) perilesional tissue; (2) regions homotopic to lesions; each unique to individuals
How are the ROI(s) defined?	(1) perilesional ROIs were voxels 3-15 mm from the lesion that were located in frontal or

	temporal regions activated by the language contrast in controls; (2) homotopic ROIs were flipped lesions
Correction for multiple comparisons	No correction
Statistical details	Test of group by time interaction not reported
Findings	Other
Findings notes	There was a significant increase in activation in perilesional ROIs

ROI analysis 10

First level contrast	Listening to normal sentences and making a plausibility judgment (paradigm 1) or listening to normal sentences (paradigm 2) vs listening to reversed speech
Analysis class	Longitudinal change in aphasia
Group(s)	Aphasia T3 vs T2
Covariate	—
Is the second level contrast valid in terms of the group(s), time point(s), and measures involved?	Yes
Is accuracy matched across the second level contrast?	Unknown, not reported
Is reaction time matched across the second level contrast?	Unknown, not reported
Behavioral data notes	No differences in proportion of expected button presses by group or time, but behavioral data pooled across conditions
Type of analysis	Regions of interest (ROI)
ROI type	Other
How many ROIs are there?	2
What are the ROI(s)?	(1) perilesional tissue; (2) regions homotopic to lesions; each unique to individuals
How are the ROI(s) defined?	(1) perilesional ROIs were voxels 3-15 mm from the lesion that were located in frontal or temporal regions activated by the language contrast in controls; (2) homotopic ROIs were flipped lesions
Correction for multiple comparisons	No correction
Statistical details	Test of group by time interaction not reported
Findings	None
Findings notes	—

ROI analysis 11

First level contrast	Listening to normal sentences and making a plausibility judgment (paradigm 1) or listening to normal sentences (paradigm 2) vs listening to reversed speech
Analysis class	Cross-sectional between two groups with aphasia
Group(s)	Aphasia frontal mean of T1, T2, T3 (n = 17) vs temporo-parietal mean of T1, T2, T3 (n = 17)
Covariate	—
Is the second level contrast valid in terms of the group(s), time point(s), and measures involved?	Yes
Is accuracy matched across the second level contrast?	Unknown, not reported
Is reaction time matched across the second level contrast?	Unknown, not reported
Behavioral data notes	No differences in proportion of expected button presses by group or time, but behavioral data pooled across conditions
Type of analysis	Regions of interest (ROI)
ROI type	Other
How many ROIs are there?	2
What are the ROI(s)?	(1) perilesional tissue; (2) regions homotopic to lesions; each unique to individuals
How are the ROI(s) defined?	(1) perilesional ROIs were voxels 3-15 mm from the lesion that were located in frontal or temporal regions activated by the language contrast in controls; (2) homotopic ROIs were flipped lesions
Correction for multiple comparisons	No correction
Statistical details	Test of group by time interaction not reported; this comparison is somewhat questionable

	given the differing extent to which frontal and temporal regions are activated in controls
Findings	Other
Findings notes	Frontal patients showed relatively greater activation in regions homotopic to their lesions

ROI analysis 12

First level contrast	Listening to normal sentences and making a plausibility judgment (paradigm 1) or listening to normal sentences (paradigm 2) vs listening to reversed speech
Analysis class	Cross-sectional aphasia vs control
Group(s)	Aphasia frontal T1 (n = 17) vs control
Covariate	—
Is the second level contrast valid in terms of the group(s), time point(s), and measures involved?	Yes
Is accuracy matched across the second level contrast?	Unknown, not reported
Is reaction time matched across the second level contrast?	Unknown, not reported
Behavioral data notes	No differences in proportion of expected button presses by group or time, but behavioral data pooled across conditions
Type of analysis	Regions of interest (ROI)
ROI type	Functional
How many ROIs are there?	13
What are the ROI(s)?	(1) L IFG orb; (2) L IFG tri; (3) L IFG op; (4) L DLPFC; (5) L insula; (6) L ATL; (7) L PTL; (8) L SMA/dACC; (9) R L IFG orb; (10) R IFG tri; (11) R insula; (12) R DLPFC; (13) R ATL
How are the ROI(s) defined?	Spheres around peaks of whole brain analysis of all patients collapsing across groups and timepoints; circular because patients but not controls used to define ROIs
Correction for multiple comparisons	No correction
Statistical details	—
Findings	↓ L IFG pars triangularis<br>↓ L insula<br>↓ L dorsolateral prefrontal cortex
Findings notes	—

ROI analysis 13

First level contrast	Listening to normal sentences and making a plausibility judgment (paradigm 1) or listening to normal sentences (paradigm 2) vs listening to reversed speech
Analysis class	Cross-sectional aphasia vs control
Group(s)	Aphasia temporo-parietal T1 (n = 17) vs control
Covariate	—
Is the second level contrast valid in terms of the group(s), time point(s), and measures involved?	Yes
Is accuracy matched across the second level contrast?	Unknown, not reported
Is reaction time matched across the second level contrast?	Unknown, not reported
Behavioral data notes	No differences in proportion of expected button presses by group or time, but behavioral data pooled across conditions
Type of analysis	Regions of interest (ROI)
ROI type	Functional
How many ROIs are there?	13
What are the ROI(s)?	(1) L IFG orb; (2) L IFG tri; (3) L IFG op; (4) L DLPFC; (5) L insula; (6) L ATL; (7) L PTL; (8) L SMA/dACC; (9) R L IFG orb; (10) R IFG tri; (11) R insula; (12) R DLPFC; (13) R ATL
How are the ROI(s) defined?	Spheres around peaks of whole brain analysis of all patients collapsing across groups and timepoints; circular because patients but not controls used to define ROIs
Correction for multiple comparisons	No correction
Statistical details	—
Findings	↓ L IFG pars triangularis

	↓ L insula<br>↓ L dorsolateral prefrontal cortex<br>↓ L SMA/medial prefrontal<br>↓ L posterior STG/STS/MTG<br>↓ R IFG pars triangularis
Findings notes	—

ROI analysis 14

First level contrast	Listening to normal sentences and making a plausibility judgment (paradigm 1) or listening to normal sentences (paradigm 2) vs listening to reversed speech
Analysis class	Cross-sectional between two groups with aphasia
Group(s)	Aphasia frontal T1 (n = 17) vs temporo-parietal T1 (n = 17)
Covariate	—
Is the second level contrast valid in terms of the group(s), time point(s), and measures involved?	Yes
Is accuracy matched across the second level contrast?	Unknown, not reported
Is reaction time matched across the second level contrast?	Unknown, not reported
Behavioral data notes	No differences in proportion of expected button presses by group or time, but behavioral data pooled across conditions
Type of analysis	Regions of interest (ROI)
ROI type	Functional
How many ROIs are there?	13
What are the ROI(s)?	(1) L IFG orb; (2) L IFG tri; (3) L IFG op; (4) L DLPFC; (5) L insula; (6) L ATL; (7) L PTL; (8) L SMA/dACC; (9) R L IFG orb; (10) R IFG tri; (11) R insula; (12) R DLPFC; (13) R ATL
How are the ROI(s) defined?	Spheres around peaks of whole brain analysis of all patients collapsing across groups and timepoints
Correction for multiple comparisons	No correction
Statistical details	—
Findings	↑ L anterior temporal<br>↑ R IFG pars triangularis<br>↑ R anterior temporal
Findings notes	—

ROI analysis 15

First level contrast	Listening to normal sentences and making a plausibility judgment (paradigm 1) or listening to normal sentences (paradigm 2) vs listening to reversed speech
Analysis class	Cross-sectional aphasia vs control
Group(s)	Aphasia frontal T2 (n = 17) vs control
Covariate	—
Is the second level contrast valid in terms of the group(s), time point(s), and measures involved?	Yes
Is accuracy matched across the second level contrast?	Unknown, not reported
Is reaction time matched across the second level contrast?	Unknown, not reported
Behavioral data notes	No differences in proportion of expected button presses by group or time, but behavioral data pooled across conditions
Type of analysis	Regions of interest (ROI)
ROI type	Functional
How many ROIs are there?	13
What are the ROI(s)?	(1) L IFG orb; (2) L IFG tri; (3) L IFG op; (4) L DLPFC; (5) L insula; (6) L ATL; (7) L PTL; (8) L SMA/dACC; (9) R L IFG orb; (10) R IFG tri; (11) R insula; (12) R DLPFC; (13) R ATL
How are the ROI(s) defined?	Spheres around peaks of whole brain analysis of all patients collapsing across groups and timepoints; circular because patients but not controls used to define ROIs
Correction for multiple comparisons	No correction

Statistical details	—
Findings	↓ L IFG pars triangularis
Findings notes	—

ROI analysis 16

First level contrast	Listening to normal sentences and making a plausibility judgment (paradigm 1) or listening to normal sentences (paradigm 2) vs listening to reversed speech
Analysis class	Cross-sectional aphasia vs control
Group(s)	Aphasia temporo-parietal T2 (n = 17) vs control
Covariate	—
Is the second level contrast valid in terms of the group(s), time point(s), and measures involved?	Yes
Is accuracy matched across the second level contrast?	Unknown, not reported
Is reaction time matched across the second level contrast?	Unknown, not reported
Behavioral data notes	No differences in proportion of expected button presses by group or time, but behavioral data pooled across conditions
Type of analysis	Regions of interest (ROI)
ROI type	Functional
How many ROIs are there?	13
What are the ROI(s)?	(1) L IFG orb; (2) L IFG tri; (3) L IFG op; (4) L DLPFC; (5) L insula; (6) L ATL; (7) L PTL; (8) L SMA/dACC; (9) R L IFG orb; (10) R IFG tri; (11) R insula; (12) R DLPFC; (13) R ATL
How are the ROI(s) defined?	Spheres around peaks of whole brain analysis of all patients collapsing across groups and timepoints; circular because patients but not controls used to define ROIs
Correction for multiple comparisons	No correction
Statistical details	—
Findings	None
Findings notes	—

ROI analysis 17

First level contrast	Listening to normal sentences and making a plausibility judgment (paradigm 1) or listening to normal sentences (paradigm 2) vs listening to reversed speech
Analysis class	Cross-sectional between two groups with aphasia
Group(s)	Aphasia frontal T2 (n = 17) vs temporo-parietal T2 (n = 17)
Covariate	—
Is the second level contrast valid in terms of the group(s), time point(s), and measures involved?	Yes
Is accuracy matched across the second level contrast?	Unknown, not reported
Is reaction time matched across the second level contrast?	Unknown, not reported
Behavioral data notes	No differences in proportion of expected button presses by group or time, but behavioral data pooled across conditions
Type of analysis	Regions of interest (ROI)
ROI type	Functional
How many ROIs are there?	13
What are the ROI(s)?	(1) L IFG orb; (2) L IFG tri; (3) L IFG op; (4) L DLPFC; (5) L insula; (6) L ATL; (7) L PTL; (8) L SMA/dACC; (9) R L IFG orb; (10) R IFG tri; (11) R insula; (12) R DLPFC; (13) R ATL
How are the ROI(s) defined?	Spheres around peaks of whole brain analysis of all patients collapsing across groups and timepoints
Correction for multiple comparisons	No correction
Statistical details	—
Findings	↓ L IFG pars opercularis<br>↓ L IFG pars triangularis<br>↓ L dorsolateral prefrontal cortex

Findings notes	—

ROI analysis 18

First level contrast	Listening to normal sentences and making a plausibility judgment (paradigm 1) or listening to normal sentences (paradigm 2) vs listening to reversed speech
Analysis class	Cross-sectional aphasia vs control
Group(s)	Aphasia frontal T3 (n = 17) vs control
Covariate	—
Is the second level contrast valid in terms of the group(s), time point(s), and measures involved?	Yes
Is accuracy matched across the second level contrast?	Unknown, not reported
Is reaction time matched across the second level contrast?	Unknown, not reported
Behavioral data notes	No differences in proportion of expected button presses by group or time, but behavioral data pooled across conditions
Type of analysis	Regions of interest (ROI)
ROI type	Functional
How many ROIs are there?	13
What are the ROI(s)?	(1) L IFG orb; (2) L IFG tri; (3) L IFG op; (4) L DLPFC; (5) L insula; (6) L ATL; (7) L PTL; (8) L SMA/dACC; (9) R L IFG orb; (10) R IFG tri; (11) R insula; (12) R DLPFC; (13) R ATL
How are the ROI(s) defined?	Spheres around peaks of whole brain analysis of all patients collapsing across groups and timepoints; circular because patients but not controls used to define ROIs
Correction for multiple comparisons	No correction
Statistical details	—
Findings	↓ L IFG pars triangularis<br>↓ L insula
Findings notes	—

ROI analysis 19

First level contrast	Listening to normal sentences and making a plausibility judgment (paradigm 1) or listening to normal sentences (paradigm 2) vs listening to reversed speech
Analysis class	Cross-sectional aphasia vs control
Group(s)	Aphasia temporo-parietal T3 (n = 17) vs control
Covariate	—
Is the second level contrast valid in terms of the group(s), time point(s), and measures involved?	Yes
Is accuracy matched across the second level contrast?	Unknown, not reported
Is reaction time matched across the second level contrast?	Unknown, not reported
Behavioral data notes	No differences in proportion of expected button presses by group or time, but behavioral data pooled across conditions
Type of analysis	Regions of interest (ROI)
ROI type	Functional
How many ROIs are there?	13
What are the ROI(s)?	(1) L IFG orb; (2) L IFG tri; (3) L IFG op; (4) L DLPFC; (5) L insula; (6) L ATL; (7) L PTL; (8) L SMA/dACC; (9) R L IFG orb; (10) R IFG tri; (11) R insula; (12) R DLPFC; (13) R ATL
How are the ROI(s) defined?	Spheres around peaks of whole brain analysis of all patients collapsing across groups and timepoints; circular because patients but not controls used to define ROIs
Correction for multiple comparisons	No correction
Statistical details	—
Findings	None
Findings notes	—

ROI analysis 20

First level contrast	Listening to normal sentences and making a plausibility judgment (paradigm 1) or listening to normal sentences (paradigm 2) vs listening to reversed speech
Analysis class	Cross-sectional between two groups with aphasia
Group(s)	Aphasia frontal T3 (n = 17) vs temporo-parietal T3 (n = 17)
Covariate	—
Is the second level contrast valid in terms of the group(s), time point(s), and measures involved?	Yes
Is accuracy matched across the second level contrast?	Unknown, not reported
Is reaction time matched across the second level contrast?	Unknown, not reported
Behavioral data notes	No differences in proportion of expected button presses by group or time, but behavioral data pooled across conditions
Type of analysis	Regions of interest (ROI)
ROI type	Functional
How many ROIs are there?	13
What are the ROI(s)?	(1) L IFG orb; (2) L IFG tri; (3) L IFG op; (4) L DLPFC; (5) L insula; (6) L ATL; (7) L PTL; (8) L SMA/dACC; (9) R L IFG orb; (10) R IFG tri; (11) R insula; (12) R DLPFC; (13) R ATL
How are the ROI(s) defined?	Spheres around peaks of whole brain analysis of all patients collapsing across groups and timepoints
Correction for multiple comparisons	No correction
Statistical details	—
Findings	↓ L IFG pars opercularis<br>↓ L IFG pars triangularis<br>↓ L IFG pars orbitalis<br>↓ L dorsolateral prefrontal cortex
Findings notes	—

ROI analysis 21

First level contrast	Listening to normal sentences and making a plausibility judgment (paradigm 1) or listening to normal sentences (paradigm 2) vs listening to reversed speech
Analysis class	Cross-sectional aphasia vs control
Group(s)	Aphasia frontal T1 (n = 17) vs control
Covariate	—
Is the second level contrast valid in terms of the group(s), time point(s), and measures involved?	Yes
Is accuracy matched across the second level contrast?	Unknown, not reported
Is reaction time matched across the second level contrast?	Unknown, not reported
Behavioral data notes	No differences in proportion of expected button presses by group or time, but behavioral data pooled across conditions
Type of analysis	Regions of interest (ROI)
ROI type	Other
How many ROIs are there?	2
What are the ROI(s)?	(1) perilesional tissue; (2) regions homotopic to lesions; each unique to individuals
How are the ROI(s) defined?	(1) perilesional ROIs were voxels 3-15 mm from the lesion that were located in frontal or temporal regions activated by the language contrast in controls; (2) homotopic ROIs were flipped lesions
Correction for multiple comparisons	No correction
Statistical details	—
Findings	Other
Findings notes	Frontal patients showed reduced activation in perilesional tissue

ROI analysis 22

First level contrast	Listening to normal sentences and making a plausibility judgment (paradigm 1) or listening to

	normal sentences (paradigm 2) vs listening to reversed speech
Analysis class	Cross-sectional aphasia vs control
Group(s)	Aphasia frontal T2 (n = 17) vs control
Covariate	—
Is the second level contrast valid in terms of the group(s), time point(s), and measures involved?	Yes
Is accuracy matched across the second level contrast?	Unknown, not reported
Is reaction time matched across the second level contrast?	Unknown, not reported
Behavioral data notes	No differences in proportion of expected button presses by group or time, but behavioral data pooled across conditions
Type of analysis	Regions of interest (ROI)
ROI type	Other
How many ROIs are there?	2
What are the ROI(s)?	(1) perilesional tissue; (2) regions homotopic to lesions; each unique to individuals
How are the ROI(s) defined?	(1) perilesional ROIs were voxels 3-15 mm from the lesion that were located in frontal or temporal regions activated by the language contrast in controls; (2) homotopic ROIs were flipped lesions
Correction for multiple comparisons	No correction
Statistical details	—
Findings	Other
Findings notes	Frontal patients showed reduced activation in perilesional tissue

ROI analysis 23

First level contrast	Listening to normal sentences and making a plausibility judgment (paradigm 1) or listening to normal sentences (paradigm 2) vs listening to reversed speech
Analysis class	Cross-sectional aphasia vs control
Group(s)	Aphasia frontal T3 (n = 17) vs control
Covariate	—
Is the second level contrast valid in terms of the group(s), time point(s), and measures involved?	Yes
Is accuracy matched across the second level contrast?	Unknown, not reported
Is reaction time matched across the second level contrast?	Unknown, not reported
Behavioral data notes	No differences in proportion of expected button presses by group or time, but behavioral data pooled across conditions
Type of analysis	Regions of interest (ROI)
ROI type	Other
How many ROIs are there?	2
What are the ROI(s)?	(1) perilesional tissue; (2) regions homotopic to lesions; each unique to individuals
How are the ROI(s) defined?	(1) perilesional ROIs were voxels 3-15 mm from the lesion that were located in frontal or temporal regions activated by the language contrast in controls; (2) homotopic ROIs were flipped lesions
Correction for multiple comparisons	No correction
Statistical details	—
Findings	Other
Findings notes	Frontal patients showed reduced activation in perilesional tissue

ROI analysis 24

First level contrast	Listening to normal sentences and making a plausibility judgment (paradigm 1) or listening to normal sentences (paradigm 2) vs listening to reversed speech
Analysis class	Cross-sectional aphasia vs control
Group(s)	Aphasia temporo-parietal T1 (n = 17) vs control
Covariate	—

Is the second level contrast valid in terms of the group(s), time point(s), and measures involved?	Yes
Is accuracy matched across the second level contrast?	Unknown, not reported
Is reaction time matched across the second level contrast?	Unknown, not reported
Behavioral data notes	No differences in proportion of expected button presses by group or time, but behavioral data pooled across conditions
Type of analysis	Regions of interest (ROI)
ROI type	Other
How many ROIs are there?	2
What are the ROI(s)?	(1) perilesional tissue; (2) regions homotopic to lesions; each unique to individuals
How are the ROI(s) defined?	(1) perilesional ROIs were voxels 3-15 mm from the lesion that were located in frontal or temporal regions activated by the language contrast in controls; (2) homotopic ROIs were flipped lesions
Correction for multiple comparisons	No correction
Statistical details	—
Findings	Other
Findings notes	Temporal patients showed reduced activation in perilesional tissue and in regions homotopic to their lesions

ROI analysis 25

First level contrast	Listening to normal sentences and making a plausibility judgment (paradigm 1) or listening to normal sentences (paradigm 2) vs listening to reversed speech
Analysis class	Cross-sectional aphasia vs control
Group(s)	Aphasia temporo-parietal T2 (n = 17) vs control
Covariate	—
Is the second level contrast valid in terms of the group(s), time point(s), and measures involved?	Yes
Is accuracy matched across the second level contrast?	Unknown, not reported
Is reaction time matched across the second level contrast?	Unknown, not reported
Behavioral data notes	No differences in proportion of expected button presses by group or time, but behavioral data pooled across conditions
Type of analysis	Regions of interest (ROI)
ROI type	Other
How many ROIs are there?	2
What are the ROI(s)?	(1) perilesional tissue; (2) regions homotopic to lesions; each unique to individuals
How are the ROI(s) defined?	(1) perilesional ROIs were voxels 3-15 mm from the lesion that were located in frontal or temporal regions activated by the language contrast in controls; (2) homotopic ROIs were flipped lesions
Correction for multiple comparisons	No correction
Statistical details	—
Findings	None
Findings notes	—

ROI analysis 26

First level contrast	Listening to normal sentences and making a plausibility judgment (paradigm 1) or listening to normal sentences (paradigm 2) vs listening to reversed speech
Analysis class	Cross-sectional aphasia vs control
Group(s)	Aphasia temporo-parietal T3 (n = 17) vs control
Covariate	—
Is the second level contrast valid in terms of the group(s), time point(s), and measures involved?	Yes
Is accuracy matched across the second level	Unknown, not reported

contrast?	
Is reaction time matched across the second level contrast?	Unknown, not reported
Behavioral data notes	No differences in proportion of expected button presses by group or time, but behavioral data pooled across conditions
Type of analysis	Regions of interest (ROI)
ROI type	Other
How many ROIs are there?	2
What are the ROI(s)?	(1) perilesional tissue; (2) regions homotopic to lesions; each unique to individuals
How are the ROI(s) defined?	(1) perilesional ROIs were voxels 3-15 mm from the lesion that were located in frontal or temporal regions activated by the language contrast in controls; (2) homotopic ROIs were flipped lesions
Correction for multiple comparisons	No correction
Statistical details	—
Findings	None
Findings notes	—

ROI analysis 27

First level contrast	Listening to normal sentences and making a plausibility judgment (paradigm 1) or listening to normal sentences (paradigm 2) vs listening to reversed speech
Analysis class	Cross-sectional correlation with language or other measure
Group(s)	Aphasia T1
Covariate	Comprehension composite
Is the second level contrast valid in terms of the group(s), time point(s), and measures involved?	Yes
Is accuracy matched across the second level contrast?	Unknown, not reported
Is reaction time matched across the second level contrast?	Unknown, not reported
Behavioral data notes	No differences in proportion of expected button presses by group or time, but behavioral data pooled across conditions
Type of analysis	Regions of interest (ROI)
ROI type	Mixed
How many ROIs are there?	15
What are the ROI(s)?	(1) L IFG orb; (2) L IFG tri; (3) L IFG op; (4) L DLPFC; (5) L insula; (6) L ATL; (7) L PTL; (8) L SMA/dACC; (9) R L IFG orb; (10) R IFG tri; (11) R insula; (12) R DLPFC; (13) R ATL; (14) perilesional tissue; (15) regions homotopic to lesions
How are the ROI(s) defined?	(1-13) spheres around peaks of whole brain analysis of all patients collapsing across groups and timepoints; (14) perilesional ROIs were voxels 3-15 mm from the lesion that were located in frontal or temporal regions activated by the language contrast in controls; (15) homotopic ROIs were flipped lesions
Correction for multiple comparisons	No correction
Statistical details	—
Findings	↑ L IFG pars opercularis<br>↑ L IFG pars triangularis<br>↑ L IFG pars orbitalis<br>other
Findings notes	L IFG pars opercularis and orbitalis did not remain significant when lesion volume was included as a covariate; there was a significant correlation between perilesional activation and LRScomp; this did not remain significant when lesion volume was included as a covariate

ROI analysis 28

First level contrast	Listening to normal sentences and making a plausibility judgment (paradigm 1) or listening to normal sentences (paradigm 2) vs listening to reversed speech
Analysis class	Cross-sectional correlation with language or other measure
Group(s)	Aphasia T2
Covariate	Comprehension composite

Is the second level contrast valid in terms of the group(s), time point(s), and measures involved?	Yes
Is accuracy matched across the second level contrast?	Unknown, not reported
Is reaction time matched across the second level contrast?	Unknown, not reported
Behavioral data notes	No differences in proportion of expected button presses by group or time, but behavioral data pooled across conditions
Type of analysis	Regions of interest (ROI)
ROI type	Mixed
How many ROIs are there?	15
What are the ROI(s)?	(1) L IFG orb; (2) L IFG tri; (3) L IFG op; (4) L DLPFC; (5) L insula; (6) L ATL; (7) L PTL; (8) L SMA/dACC; (9) R L IFG orb; (10) R IFG tri; (11) R insula; (12) R DLPFC; (13) R ATL; (14) perilesional tissue; (15) regions homotopic to lesions
How are the ROI(s) defined?	(1-13) spheres around peaks of whole brain analysis of all patients collapsing across groups and timepoints; (14) perilesional ROIs were voxels 3-15 mm from the lesion that were located in frontal or temporal regions activated by the language contrast in controls; (15) homotopic ROIs were flipped lesions
Correction for multiple comparisons	No correction
Statistical details	—
Findings	↑ L IFG pars triangularis<br>other
Findings notes	There was a significant correlation between perilesional activation and LRScomp

ROI analysis 29

First level contrast	Listening to normal sentences and making a plausibility judgment (paradigm 1) or listening to normal sentences (paradigm 2) vs listening to reversed speech
Analysis class	Cross-sectional correlation with language or other measure
Group(s)	Aphasia T3
Covariate	Comprehension composite
Is the second level contrast valid in terms of the group(s), time point(s), and measures involved?	Yes
Is accuracy matched across the second level contrast?	Unknown, not reported
Is reaction time matched across the second level contrast?	Unknown, not reported
Behavioral data notes	No differences in proportion of expected button presses by group or time, but behavioral data pooled across conditions
Type of analysis	Regions of interest (ROI)
ROI type	Mixed
How many ROIs are there?	15
What are the ROI(s)?	(1) L IFG orb; (2) L IFG tri; (3) L IFG op; (4) L DLPFC; (5) L insula; (6) L ATL; (7) L PTL; (8) L SMA/dACC; (9) R L IFG orb; (10) R IFG tri; (11) R insula; (12) R DLPFC; (13) R ATL; (14) perilesional tissue; (15) regions homotopic to lesions
How are the ROI(s) defined?	(1-13) spheres around peaks of whole brain analysis of all patients collapsing across groups and timepoints; (14) perilesional ROIs were voxels 3-15 mm from the lesion that were located in frontal or temporal regions activated by the language contrast in controls; (15) homotopic ROIs were flipped lesions
Correction for multiple comparisons	No correction
Statistical details	—
Findings	↑ L IFG pars triangularis
Findings notes	Did not remain significant when lesion volume was included as a covariate

ROI analysis 30

First level contrast	Listening to normal sentences and making a plausibility judgment (paradigm 1) or listening to normal sentences (paradigm 2) vs listening to reversed speech
Analysis class	Longitudinal correlation with language or other measure

Group(s)	Aphasia T2 vs T1
Covariate	Δ comprehension composite
Is the second level contrast valid in terms of the group(s), time point(s), and measures involved?	Yes
Is accuracy matched across the second level contrast?	Unknown, not reported
Is reaction time matched across the second level contrast?	Unknown, not reported
Behavioral data notes	No differences in proportion of expected button presses by group or time, but behavioral data pooled across conditions
Type of analysis	Regions of interest (ROI)
ROI type	Mixed
How many ROIs are there?	15
What are the ROI(s)?	(1) L IFG orb; (2) L IFG tri; (3) L IFG op; (4) L DLPFC; (5) L insula; (6) L ATL; (7) L PTL; (8) L SMA/dACC; (9) R L IFG orb; (10) R IFG tri; (11) R insula; (12) R DLPFC; (13) R ATL; (14) perilesional tissue; (15) regions homotopic to lesions
How are the ROI(s) defined?	(1-13) spheres around peaks of whole brain analysis of all patients collapsing across groups and timepoints; (14) perilesional ROIs were voxels 3-15 mm from the lesion that were located in frontal or temporal regions activated by the language contrast in controls; (15) homotopic ROIs were flipped lesions
Correction for multiple comparisons	No correction
Statistical details	—
Findings	↑ L insula<br>↑ R dorsolateral prefrontal cortex
Findings notes	R dorsolateral prefrontal cortex did not remain significant when lesion volume was included as a covariate

ROI analysis 31

First level contrast	Listening to normal sentences and making a plausibility judgment (paradigm 1) or listening to normal sentences (paradigm 2) vs listening to reversed speech
Analysis class	Longitudinal correlation with language or other measure
Group(s)	Aphasia T3 vs T1
Covariate	Δ comprehension composite
Is the second level contrast valid in terms of the group(s), time point(s), and measures involved?	Yes
Is accuracy matched across the second level contrast?	Unknown, not reported
Is reaction time matched across the second level contrast?	Unknown, not reported
Behavioral data notes	No differences in proportion of expected button presses by group or time, but behavioral data pooled across conditions
Type of analysis	Regions of interest (ROI)
ROI type	Mixed
How many ROIs are there?	15
What are the ROI(s)?	(1) L IFG orb; (2) L IFG tri; (3) L IFG op; (4) L DLPFC; (5) L insula; (6) L ATL; (7) L PTL; (8) L SMA/dACC; (9) R L IFG orb; (10) R IFG tri; (11) R insula; (12) R DLPFC; (13) R ATL; (14) perilesional tissue; (15) regions homotopic to lesions
How are the ROI(s) defined?	(1-13) spheres around peaks of whole brain analysis of all patients collapsing across groups and timepoints; (14) perilesional ROIs were voxels 3-15 mm from the lesion that were located in frontal or temporal regions activated by the language contrast in controls; (15) homotopic ROIs were flipped lesions
Correction for multiple comparisons	No correction
Statistical details	—
Findings	None
Findings notes	—

ROI analysis 32

First level contrast	Listening to normal sentences and making a plausibility judgment (paradigm 1) or listening to normal sentences (paradigm 2) vs listening to reversed speech
Analysis class	Longitudinal correlation with language or other measure
Group(s)	Aphasia T3 vs T2
Covariate	Δ comprehension composite
Is the second level contrast valid in terms of the group(s), time point(s), and measures involved?	Yes
Is accuracy matched across the second level contrast?	Unknown, not reported
Is reaction time matched across the second level contrast?	Unknown, not reported
Behavioral data notes	No differences in proportion of expected button presses by group or time, but behavioral data pooled across conditions
Type of analysis	Regions of interest (ROI)
ROI type	Mixed
How many ROIs are there?	15
What are the ROI(s)?	(1) L IFG orb; (2) L IFG tri; (3) L IFG op; (4) L DLPFC; (5) L insula; (6) L ATL; (7) L PTL; (8) L SMA/dACC; (9) R L IFG orb; (10) R IFG tri; (11) R insula; (12) R DLPFC; (13) R ATL; (14) perilesional tissue; (15) regions homotopic to lesions
How are the ROI(s) defined?	(1-13) spheres around peaks of whole brain analysis of all patients collapsing across groups and timepoints; (14) perilesional ROIs were voxels 3-15 mm from the lesion that were located in frontal or temporal regions activated by the language contrast in controls; (15) homotopic ROIs were flipped lesions
Correction for multiple comparisons	No correction
Statistical details	—
Findings	None
Findings notes	—

ROI analysis 33

First level contrast	Listening to normal sentences and making a plausibility judgment (paradigm 1) or listening to normal sentences (paradigm 2) vs listening to reversed speech
Analysis class	Cross-sectional correlation with language or other measure
Group(s)	Aphasia frontal T1 (n = 17)
Covariate	Comprehension composite
Is the second level contrast valid in terms of the group(s), time point(s), and measures involved?	Yes
Is accuracy matched across the second level contrast?	Unknown, not reported
Is reaction time matched across the second level contrast?	Unknown, not reported
Behavioral data notes	No differences in proportion of expected button presses by group or time, but behavioral data pooled across conditions
Type of analysis	Regions of interest (ROI)
ROI type	Mixed
How many ROIs are there?	15
What are the ROI(s)?	(1) L IFG orb; (2) L IFG tri; (3) L IFG op; (4) L DLPFC; (5) L insula; (6) L ATL; (7) L PTL; (8) L SMA/dACC; (9) R L IFG orb; (10) R IFG tri; (11) R insula; (12) R DLPFC; (13) R ATL; (14) perilesional tissue; (15) regions homotopic to lesions
How are the ROI(s) defined?	(1-13) spheres around peaks of whole brain analysis of all patients collapsing across groups and timepoints; (14) perilesional ROIs were voxels 3-15 mm from the lesion that were located in frontal or temporal regions activated by the language contrast in controls; (15) homotopic ROIs were flipped lesions
Correction for multiple comparisons	No correction
Statistical details	—
Findings	None
Findings notes	—

ROI analysis 34

First level contrast	Listening to normal sentences and making a plausibility judgment (paradigm 1) or listening to normal sentences (paradigm 2) vs listening to reversed speech
Analysis class	Cross-sectional correlation with language or other measure
Group(s)	Aphasia frontal T2 (n = 17)
Covariate	Comprehension composite
Is the second level contrast valid in terms of the group(s), time point(s), and measures involved?	Yes
Is accuracy matched across the second level contrast?	Unknown, not reported
Is reaction time matched across the second level contrast?	Unknown, not reported
Behavioral data notes	No differences in proportion of expected button presses by group or time, but behavioral data pooled across conditions
Type of analysis	Regions of interest (ROI)
ROI type	Mixed
How many ROIs are there?	15
What are the ROI(s)?	(1) L IFG orb; (2) L IFG tri; (3) L IFG op; (4) L DLPFC; (5) L insula; (6) L ATL; (7) L PTL; (8) L SMA/dACC; (9) R L IFG orb; (10) R IFG tri; (11) R insula; (12) R DLPFC; (13) R ATL; (14) perilesional tissue; (15) regions homotopic to lesions
How are the ROI(s) defined?	(1-13) spheres around peaks of whole brain analysis of all patients collapsing across groups and timepoints; (14) perilesional ROIs were voxels 3-15 mm from the lesion that were located in frontal or temporal regions activated by the language contrast in controls; (15) homotopic ROIs were flipped lesions
Correction for multiple comparisons	No correction
Statistical details	—
Findings	None
Findings notes	—

ROI analysis 35

First level contrast	Listening to normal sentences and making a plausibility judgment (paradigm 1) or listening to normal sentences (paradigm 2) vs listening to reversed speech
Analysis class	Cross-sectional correlation with language or other measure
Group(s)	Aphasia frontal T3 (n = 17)
Covariate	Comprehension composite
Is the second level contrast valid in terms of the group(s), time point(s), and measures involved?	Yes
Is accuracy matched across the second level contrast?	Unknown, not reported
Is reaction time matched across the second level contrast?	Unknown, not reported
Behavioral data notes	No differences in proportion of expected button presses by group or time, but behavioral data pooled across conditions
Type of analysis	Regions of interest (ROI)
ROI type	Mixed
How many ROIs are there?	15
What are the ROI(s)?	(1) L IFG orb; (2) L IFG tri; (3) L IFG op; (4) L DLPFC; (5) L insula; (6) L ATL; (7) L PTL; (8) L SMA/dACC; (9) R L IFG orb; (10) R IFG tri; (11) R insula; (12) R DLPFC; (13) R ATL; (14) perilesional tissue; (15) regions homotopic to lesions
How are the ROI(s) defined?	(1-13) spheres around peaks of whole brain analysis of all patients collapsing across groups and timepoints; (14) perilesional ROIs were voxels 3-15 mm from the lesion that were located in frontal or temporal regions activated by the language contrast in controls; (15) homotopic ROIs were flipped lesions
Correction for multiple comparisons	No correction
Statistical details	—
Findings	None

Findings notes	—

ROI analysis 36

First level contrast	Listening to normal sentences and making a plausibility judgment (paradigm 1) or listening to normal sentences (paradigm 2) vs listening to reversed speech
Analysis class	Longitudinal correlation with language or other measure
Group(s)	Aphasia frontal (n = 17) T2 vs T1
Covariate	Δ comprehension composite
Is the second level contrast valid in terms of the group(s), time point(s), and measures involved?	Yes
Is accuracy matched across the second level contrast?	Unknown, not reported
Is reaction time matched across the second level contrast?	Unknown, not reported
Behavioral data notes	No differences in proportion of expected button presses by group or time, but behavioral data pooled across conditions
Type of analysis	Regions of interest (ROI)
ROI type	Mixed
How many ROIs are there?	15
What are the ROI(s)?	(1) L IFG orb; (2) L IFG tri; (3) L IFG op; (4) L DLPFC; (5) L insula; (6) L ATL; (7) L PTL; (8) L SMA/dACC; (9) R L IFG orb; (10) R IFG tri; (11) R insula; (12) R DLPFC; (13) R ATL; (14) perilesional tissue; (15) regions homotopic to lesions
How are the ROI(s) defined?	(1-13) spheres around peaks of whole brain analysis of all patients collapsing across groups and timepoints; (14) perilesional ROIs were voxels 3-15 mm from the lesion that were located in frontal or temporal regions activated by the language contrast in controls; (15) homotopic ROIs were flipped lesions
Correction for multiple comparisons	No correction
Statistical details	—
Findings	None
Findings notes	—

ROI analysis 37

First level contrast	Listening to normal sentences and making a plausibility judgment (paradigm 1) or listening to normal sentences (paradigm 2) vs listening to reversed speech
Analysis class	Longitudinal correlation with language or other measure
Group(s)	Aphasia frontal (n = 17) T3 vs T1
Covariate	Δ comprehension composite
Is the second level contrast valid in terms of the group(s), time point(s), and measures involved?	Yes
Is accuracy matched across the second level contrast?	Unknown, not reported
Is reaction time matched across the second level contrast?	Unknown, not reported
Behavioral data notes	No differences in proportion of expected button presses by group or time, but behavioral data pooled across conditions
Type of analysis	Regions of interest (ROI)
ROI type	Mixed
How many ROIs are there?	15
What are the ROI(s)?	(1) L IFG orb; (2) L IFG tri; (3) L IFG op; (4) L DLPFC; (5) L insula; (6) L ATL; (7) L PTL; (8) L SMA/dACC; (9) R L IFG orb; (10) R IFG tri; (11) R insula; (12) R DLPFC; (13) R ATL; (14) perilesional tissue; (15) regions homotopic to lesions
How are the ROI(s) defined?	(1-13) spheres around peaks of whole brain analysis of all patients collapsing across groups and timepoints; (14) perilesional ROIs were voxels 3-15 mm from the lesion that were located in frontal or temporal regions activated by the language contrast in controls; (15) homotopic ROIs were flipped lesions
Correction for multiple comparisons	No correction
Statistical details	—

Findings	None
Findings notes	—

ROI analysis 38

First level contrast	Listening to normal sentences and making a plausibility judgment (paradigm 1) or listening to normal sentences (paradigm 2) vs listening to reversed speech
Analysis class	Longitudinal correlation with language or other measure
Group(s)	Aphasia frontal (n = 17) T3 vs T2
Covariate	Δ comprehension composite
Is the second level contrast valid in terms of the group(s), time point(s), and measures involved?	Yes
Is accuracy matched across the second level contrast?	Unknown, not reported
Is reaction time matched across the second level contrast?	Unknown, not reported
Behavioral data notes	No differences in proportion of expected button presses by group or time, but behavioral data pooled across conditions
Type of analysis	Regions of interest (ROI)
ROI type	Mixed
How many ROIs are there?	15
What are the ROI(s)?	(1) L IFG orb; (2) L IFG tri; (3) L IFG op; (4) L DLPFC; (5) L insula; (6) L ATL; (7) L PTL; (8) L SMA/dACC; (9) R L IFG orb; (10) R IFG tri; (11) R insula; (12) R DLPFC; (13) R ATL; (14) perilesional tissue; (15) regions homotopic to lesions
How are the ROI(s) defined?	(1-13) spheres around peaks of whole brain analysis of all patients collapsing across groups and timepoints; (14) perilesional ROIs were voxels 3-15 mm from the lesion that were located in frontal or temporal regions activated by the language contrast in controls; (15) homotopic ROIs were flipped lesions
Correction for multiple comparisons	No correction
Statistical details	—
Findings	None
Findings notes	—

ROI analysis 39

First level contrast	Listening to normal sentences and making a plausibility judgment (paradigm 1) or listening to normal sentences (paradigm 2) vs listening to reversed speech
Analysis class	Cross-sectional correlation with language or other measure
Group(s)	Aphasia temporo-parietal T1 (n = 17)
Covariate	Comprehension composite
Is the second level contrast valid in terms of the group(s), time point(s), and measures involved?	Yes
Is accuracy matched across the second level contrast?	Unknown, not reported
Is reaction time matched across the second level contrast?	Unknown, not reported
Behavioral data notes	No differences in proportion of expected button presses by group or time, but behavioral data pooled across conditions
Type of analysis	Regions of interest (ROI)
ROI type	Mixed
How many ROIs are there?	15
What are the ROI(s)?	(1) L IFG orb; (2) L IFG tri; (3) L IFG op; (4) L DLPFC; (5) L insula; (6) L ATL; (7) L PTL; (8) L SMA/dACC; (9) R L IFG orb; (10) R IFG tri; (11) R insula; (12) R DLPFC; (13) R ATL; (14) perilesional tissue; (15) regions homotopic to lesions
How are the ROI(s) defined?	(1-13) spheres around peaks of whole brain analysis of all patients collapsing across groups and timepoints; (14) perilesional ROIs were voxels 3-15 mm from the lesion that were located in frontal or temporal regions activated by the language contrast in controls; (15) homotopic ROIs were flipped lesions
Correction for multiple comparisons	No correction

Statistical details	—
Findings	↑ R anterior temporal
Findings notes	—

ROI analysis 40

First level contrast	Listening to normal sentences and making a plausibility judgment (paradigm 1) or listening to normal sentences (paradigm 2) vs listening to reversed speech
Analysis class	Cross-sectional correlation with language or other measure
Group(s)	Aphasia temporo-parietal T2 (n = 17)
Covariate	Comprehension composite
Is the second level contrast valid in terms of the group(s), time point(s), and measures involved?	Yes
Is accuracy matched across the second level contrast?	Unknown, not reported
Is reaction time matched across the second level contrast?	Unknown, not reported
Behavioral data notes	No differences in proportion of expected button presses by group or time, but behavioral data pooled across conditions
Type of analysis	Regions of interest (ROI)
ROI type	Mixed
How many ROIs are there?	15
What are the ROI(s)?	(1) L IFG orb; (2) L IFG tri; (3) L IFG op; (4) L DLPFC; (5) L insula; (6) L ATL; (7) L PTL; (8) L SMA/dACC; (9) R L IFG orb; (10) R IFG tri; (11) R insula; (12) R DLPFC; (13) R ATL; (14) perilesional tissue; (15) regions homotopic to lesions
How are the ROI(s) defined?	(1-13) spheres around peaks of whole brain analysis of all patients collapsing across groups and timepoints; (14) perilesional ROIs were voxels 3-15 mm from the lesion that were located in frontal or temporal regions activated by the language contrast in controls; (15) homotopic ROIs were flipped lesions
Correction for multiple comparisons	No correction
Statistical details	—
Findings	↑ L IFG pars opercularis<br>↑ L posterior STG/STS/MTG
Findings notes	—

ROI analysis 41

First level contrast	Listening to normal sentences and making a plausibility judgment (paradigm 1) or listening to normal sentences (paradigm 2) vs listening to reversed speech
Analysis class	Cross-sectional correlation with language or other measure
Group(s)	Aphasia temporo-parietal T3 (n = 17)
Covariate	Comprehension composite
Is the second level contrast valid in terms of the group(s), time point(s), and measures involved?	Yes
Is accuracy matched across the second level contrast?	Unknown, not reported
Is reaction time matched across the second level contrast?	Unknown, not reported
Behavioral data notes	No differences in proportion of expected button presses by group or time, but behavioral data pooled across conditions
Type of analysis	Regions of interest (ROI)
ROI type	Mixed
How many ROIs are there?	15
What are the ROI(s)?	(1) L IFG orb; (2) L IFG tri; (3) L IFG op; (4) L DLPFC; (5) L insula; (6) L ATL; (7) L PTL; (8) L SMA/dACC; (9) R L IFG orb; (10) R IFG tri; (11) R insula; (12) R DLPFC; (13) R ATL; (14) perilesional tissue; (15) regions homotopic to lesions
How are the ROI(s) defined?	(1-13) spheres around peaks of whole brain analysis of all patients collapsing across groups and timepoints; (14) perilesional ROIs were voxels 3-15 mm from the lesion that were located

	in frontal or temporal regions activated by the language contrast in controls; (15) homotopic ROIs were flipped lesions
Correction for multiple comparisons	No correction
Statistical details	—
Findings	None
Findings notes	—

ROI analysis 42

First level contrast	Listening to normal sentences and making a plausibility judgment (paradigm 1) or listening to normal sentences (paradigm 2) vs listening to reversed speech
Analysis class	Longitudinal correlation with language or other measure
Group(s)	Aphasia temporo-parietal (n = 17) T2 vs T1
Covariate	Δ comprehension composite
Is the second level contrast valid in terms of the group(s), time point(s), and measures involved?	Yes
Is accuracy matched across the second level contrast?	Unknown, not reported
Is reaction time matched across the second level contrast?	Unknown, not reported
Behavioral data notes	No differences in proportion of expected button presses by group or time, but behavioral data pooled across conditions
Type of analysis	Regions of interest (ROI)
ROI type	Mixed
How many ROIs are there?	15
What are the ROI(s)?	(1) L IFG orb; (2) L IFG tri; (3) L IFG op; (4) L DLPFC; (5) L insula; (6) L ATL; (7) L PTL; (8) L SMA/dACC; (9) R L IFG orb; (10) R IFG tri; (11) R insula; (12) R DLPFC; (13) R ATL; (14) perilesional tissue; (15) regions homotopic to lesions
How are the ROI(s) defined?	(1-13) spheres around peaks of whole brain analysis of all patients collapsing across groups and timepoints; (14) perilesional ROIs were voxels 3-15 mm from the lesion that were located in frontal or temporal regions activated by the language contrast in controls; (15) homotopic ROIs were flipped lesions
Correction for multiple comparisons	No correction
Statistical details	—
Findings	↑ L insula
Findings notes	—

ROI analysis 43

First level contrast	Listening to normal sentences and making a plausibility judgment (paradigm 1) or listening to normal sentences (paradigm 2) vs listening to reversed speech
Analysis class	Longitudinal correlation with language or other measure
Group(s)	Aphasia temporo-parietal (n = 17) T3 vs T1
Covariate	Δ comprehension composite
Is the second level contrast valid in terms of the group(s), time point(s), and measures involved?	Yes
Is accuracy matched across the second level contrast?	Unknown, not reported
Is reaction time matched across the second level contrast?	Unknown, not reported
Behavioral data notes	No differences in proportion of expected button presses by group or time, but behavioral data pooled across conditions
Type of analysis	Regions of interest (ROI)
ROI type	Mixed
How many ROIs are there?	15
What are the ROI(s)?	(1) L IFG orb; (2) L IFG tri; (3) L IFG op; (4) L DLPFC; (5) L insula; (6) L ATL; (7) L PTL; (8) L SMA/dACC; (9) R L IFG orb; (10) R IFG tri; (11) R insula; (12) R DLPFC; (13) R ATL; (14) perilesional tissue; (15) regions homotopic to lesions

How are the ROI(s) defined?	(1-13) spheres around peaks of whole brain analysis of all patients collapsing across groups and timepoints; (14) perilesional ROIs were voxels 3-15 mm from the lesion that were located in frontal or temporal regions activated by the language contrast in controls; (15) homotopic ROIs were flipped lesions
Correction for multiple comparisons	No correction
Statistical details	—
Findings	None
Findings notes	—

ROI analysis 44

First level contrast	Listening to normal sentences and making a plausibility judgment (paradigm 1) or listening to normal sentences (paradigm 2) vs listening to reversed speech
Analysis class	Longitudinal correlation with language or other measure
Group(s)	Aphasia temporo-parietal (n = 17) T3 vs T2
Covariate	Δ comprehension composite
Is the second level contrast valid in terms of the group(s), time point(s), and measures involved?	Yes
Is accuracy matched across the second level contrast?	Unknown, not reported
Is reaction time matched across the second level contrast?	Unknown, not reported
Behavioral data notes	No differences in proportion of expected button presses by group or time, but behavioral data pooled across conditions
Type of analysis	Regions of interest (ROI)
ROI type	Mixed
How many ROIs are there?	15
What are the ROI(s)?	(1) L IFG orb; (2) L IFG tri; (3) L IFG op; (4) L DLPFC; (5) L insula; (6) L ATL; (7) L PTL; (8) L SMA/dACC; (9) R L IFG orb; (10) R IFG tri; (11) R insula; (12) R DLPFC; (13) R ATL; (14) perilesional tissue; (15) regions homotopic to lesions
How are the ROI(s) defined?	(1-13) spheres around peaks of whole brain analysis of all patients collapsing across groups and timepoints; (14) perilesional ROIs were voxels 3-15 mm from the lesion that were located in frontal or temporal regions activated by the language contrast in controls; (15) homotopic ROIs were flipped lesions
Correction for multiple comparisons	No correction
Statistical details	—
Findings	None
Findings notes	—

ROI analysis 45

First level contrast	Listening to normal sentences and making a plausibility judgment (paradigm 1) or listening to normal sentences (paradigm 2) vs listening to reversed speech
Analysis class	Cross-sectional correlation with language or other measure
Group(s)	Aphasia T1
Covariate	Lesion volume
Is the second level contrast valid in terms of the group(s), time point(s), and measures involved?	Yes
Is accuracy matched across the second level contrast?	Unknown, not reported
Is reaction time matched across the second level contrast?	Unknown, not reported
Behavioral data notes	No differences in proportion of expected button presses by group or time, but behavioral data pooled across conditions
Type of analysis	Regions of interest (ROI)
ROI type	Mixed
How many ROIs are there?	15
What are the ROI(s)?	(1) L IFG orb; (2) L IFG tri; (3) L IFG op; (4) L DLPFC; (5) L insula; (6) L ATL; (7) L PTL; (8) L SMA/dACC; (9) R L IFG orb; (10) R IFG tri; (11) R insula; (12) R DLPFC; (13) R ATL; (14) perilesional

	tissue; (15) regions homotopic to lesions
How are the ROI(s) defined?	(1-13) spheres around peaks of whole brain analysis of all patients collapsing across groups and timepoints; (14) perilesional ROIs were voxels 3-15 mm from the lesion that were located in frontal or temporal regions activated by the language contrast in controls; (15) homotopic ROIs were flipped lesions
Correction for multiple comparisons	No correction
Statistical details	—
Findings	↓ L IFG pars triangularis
Findings notes	Lesion volume negatively correlated with activation

ROI analysis 46

First level contrast	Listening to normal sentences and making a plausibility judgment (paradigm 1) or listening to normal sentences (paradigm 2) vs listening to reversed speech
Analysis class	Cross-sectional correlation with language or other measure
Group(s)	Aphasia T2
Covariate	Lesion volume
Is the second level contrast valid in terms of the group(s), time point(s), and measures involved?	Yes
Is accuracy matched across the second level contrast?	Unknown, not reported
Is reaction time matched across the second level contrast?	Unknown, not reported
Behavioral data notes	No differences in proportion of expected button presses by group or time, but behavioral data pooled across conditions
Type of analysis	Regions of interest (ROI)
ROI type	Mixed
How many ROIs are there?	15
What are the ROI(s)?	(1) L IFG orb; (2) L IFG tri; (3) L IFG op; (4) L DLPFC; (5) L insula; (6) L ATL; (7) L PTL; (8) L SMA/dACC; (9) R L IFG orb; (10) R IFG tri; (11) R insula; (12) R DLPFC; (13) R ATL; (14) perilesional tissue; (15) regions homotopic to lesions
How are the ROI(s) defined?	(1-13) spheres around peaks of whole brain analysis of all patients collapsing across groups and timepoints; (14) perilesional ROIs were voxels 3-15 mm from the lesion that were located in frontal or temporal regions activated by the language contrast in controls; (15) homotopic ROIs were flipped lesions
Correction for multiple comparisons	No correction
Statistical details	—
Findings	None
Findings notes	—

ROI analysis 47

First level contrast	Listening to normal sentences and making a plausibility judgment (paradigm 1) or listening to normal sentences (paradigm 2) vs listening to reversed speech
Analysis class	Cross-sectional correlation with language or other measure
Group(s)	Aphasia T3
Covariate	Lesion volume
Is the second level contrast valid in terms of the group(s), time point(s), and measures involved?	Yes
Is accuracy matched across the second level contrast?	Unknown, not reported
Is reaction time matched across the second level contrast?	Unknown, not reported
Behavioral data notes	No differences in proportion of expected button presses by group or time, but behavioral data pooled across conditions
Type of analysis	Regions of interest (ROI)
ROI type	Mixed
How many ROIs are there?	15
What are the ROI(s)?	(1) L IFG orb; (2) L IFG tri; (3) L IFG op; (4) L DLPFC; (5) L insula; (6) L ATL; (7) L PTL; (8) L

	SMA/dACC; (9) R L IFG orb; (10) R IFG tri; (11) R insula; (12) R DLPFC; (13) R ATL; (14) perilesional tissue; (15) regions homotopic to lesions
How are the ROI(s) defined?	(1-13) spheres around peaks of whole brain analysis of all patients collapsing across groups and timepoints; (14) perilesional ROIs were voxels 3-15 mm from the lesion that were located in frontal or temporal regions activated by the language contrast in controls; (15) homotopic ROIs were flipped lesions
Correction for multiple comparisons	No correction
Statistical details	—
Findings	None
Findings notes	—

ROI analysis 48

First level contrast	Listening to normal sentences and making a plausibility judgment (paradigm 1) or listening to normal sentences (paradigm 2) vs listening to reversed speech
Analysis class	Longitudinal correlation with language or other measure
Group(s)	Aphasia T2 vs T1
Covariate	Lesion volume
Is the second level contrast valid in terms of the group(s), time point(s), and measures involved?	Yes
Is accuracy matched across the second level contrast?	Unknown, not reported
Is reaction time matched across the second level contrast?	Unknown, not reported
Behavioral data notes	No differences in proportion of expected button presses by group or time, but behavioral data pooled across conditions
Type of analysis	Regions of interest (ROI)
ROI type	Mixed
How many ROIs are there?	15
What are the ROI(s)?	(1) L IFG orb; (2) L IFG tri; (3) L IFG op; (4) L DLPFC; (5) L insula; (6) L ATL; (7) L PTL; (8) L SMA/dACC; (9) R L IFG orb; (10) R IFG tri; (11) R insula; (12) R DLPFC; (13) R ATL; (14) perilesional tissue; (15) regions homotopic to lesions
How are the ROI(s) defined?	(1-13) spheres around peaks of whole brain analysis of all patients collapsing across groups and timepoints; (14) perilesional ROIs were voxels 3-15 mm from the lesion that were located in frontal or temporal regions activated by the language contrast in controls; (15) homotopic ROIs were flipped lesions
Correction for multiple comparisons	No correction
Statistical details	—
Findings	None
Findings notes	—

ROI analysis 49

First level contrast	Listening to normal sentences and making a plausibility judgment (paradigm 1) or listening to normal sentences (paradigm 2) vs listening to reversed speech
Analysis class	Longitudinal correlation with language or other measure
Group(s)	Aphasia T3 vs T1
Covariate	Lesion volume
Is the second level contrast valid in terms of the group(s), time point(s), and measures involved?	Yes
Is accuracy matched across the second level contrast?	Unknown, not reported
Is reaction time matched across the second level contrast?	Unknown, not reported
Behavioral data notes	No differences in proportion of expected button presses by group or time, but behavioral data pooled across conditions
Type of analysis	Regions of interest (ROI)
ROI type	Mixed
How many ROIs are there?	15

What are the ROI(s)?	(1) L IFG orb; (2) L IFG tri; (3) L IFG op; (4) L DLPFC; (5) L insula; (6) L ATL; (7) L PTL; (8) L SMA/dACC; (9) R L IFG orb; (10) R IFG tri; (11) R insula; (12) R DLPFC; (13) R ATL; (14) perilesional tissue; (15) regions homotopic to lesions
How are the ROI(s) defined?	(1-13) spheres around peaks of whole brain analysis of all patients collapsing across groups and timepoints; (14) perilesional ROIs were voxels 3-15 mm from the lesion that were located in frontal or temporal regions activated by the language contrast in controls; (15) homotopic ROIs were flipped lesions
Correction for multiple comparisons	No correction
Statistical details	—
Findings	None
Findings notes	—

ROI analysis 50

First level contrast	Listening to normal sentences and making a plausibility judgment (paradigm 1) or listening to normal sentences (paradigm 2) vs listening to reversed speech
Analysis class	Longitudinal correlation with language or other measure
Group(s)	Aphasia T3 vs T2
Covariate	Lesion volume
Is the second level contrast valid in terms of the group(s), time point(s), and measures involved?	Yes
Is accuracy matched across the second level contrast?	Unknown, not reported
Is reaction time matched across the second level contrast?	Unknown, not reported
Behavioral data notes	No differences in proportion of expected button presses by group or time, but behavioral data pooled across conditions
Type of analysis	Regions of interest (ROI)
ROI type	Mixed
How many ROIs are there?	15
What are the ROI(s)?	(1) L IFG orb; (2) L IFG tri; (3) L IFG op; (4) L DLPFC; (5) L insula; (6) L ATL; (7) L PTL; (8) L SMA/dACC; (9) R L IFG orb; (10) R IFG tri; (11) R insula; (12) R DLPFC; (13) R ATL; (14) perilesional tissue; (15) regions homotopic to lesions
How are the ROI(s) defined?	(1-13) spheres around peaks of whole brain analysis of all patients collapsing across groups and timepoints; (14) perilesional ROIs were voxels 3-15 mm from the lesion that were located in frontal or temporal regions activated by the language contrast in controls; (15) homotopic ROIs were flipped lesions
Correction for multiple comparisons	No correction
Statistical details	—
Findings	None
Findings notes	—

Complex analysis 1

First level contrast	Listening to normal sentences and making a plausibility judgment (paradigm 1) or listening to normal sentences (paradigm 2) vs listening to reversed speech
Analysis class	Cross-sectional between two groups with aphasia
Group(s)	Aphasia frontal T1 (n = 17) vs temporo-parietal T1 (n = 17)
Covariate	—
Is the second level contrast valid in terms of the group(s), time point(s), and measures involved?	Yes
Is accuracy matched across the second level contrast?	Unknown, not reported
Is reaction time matched across the second level contrast?	Unknown, not reported
Behavioral data notes	No differences in proportion of expected button presses by group or time, but behavioral data pooled across conditions
Type of analysis	Complex
Statistical details	Correlations between activity in 15 ROIs and LRScomp were compared between patients with

	frontal and temporal lesions, using interaction terms as well as the Fisher r-to-z transformation. There was no correction for multiple comparisons across the 15 ROIs.
Findings	Other
Findings notes	Correlations were higher in the temporal group in the R ATL.

Complex analysis 2

First level contrast	Listening to normal sentences and making a plausibility judgment (paradigm 1) or listening to normal sentences (paradigm 2) vs listening to reversed speech
Analysis class	Cross-sectional between two groups with aphasia
Group(s)	Aphasia frontal T2 (n = 17) vs temporo-parietal T2 (n = 17)
Covariate	—
Is the second level contrast valid in terms of the group(s), time point(s), and measures involved?	Yes
Is accuracy matched across the second level contrast?	Unknown, not reported
Is reaction time matched across the second level contrast?	Unknown, not reported
Behavioral data notes	No differences in proportion of expected button presses by group or time, but behavioral data pooled across conditions
Type of analysis	Complex
Statistical details	Correlations between activity in 15 ROIs and LRScomp were compared between patients with frontal and temporal lesions, using interaction terms as well as the Fisher r-to-z transformation. There was no correction for multiple comparisons across the 15 ROIs.
Findings	Other
Findings notes	Correlations were higher in the temporal group in L posterior temporal cortex and L IFG op.

Complex analysis 3

First level contrast	Listening to normal sentences and making a plausibility judgment (paradigm 1) or listening to normal sentences (paradigm 2) vs listening to reversed speech
Analysis class	Cross-sectional between two groups with aphasia
Group(s)	Aphasia frontal T3 (n = 17) vs temporo-parietal T3 (n = 17)
Covariate	—
Is the second level contrast valid in terms of the group(s), time point(s), and measures involved?	Yes
Is accuracy matched across the second level contrast?	Unknown, not reported
Is reaction time matched across the second level contrast?	Unknown, not reported
Behavioral data notes	No differences in proportion of expected button presses by group or time, but behavioral data pooled across conditions
Type of analysis	Complex
Statistical details	Correlations between activity in 15 ROIs and LRScomp were compared between patients with frontal and temporal lesions, using interaction terms. There was no correction for multiple comparisons across the 15 ROIs.
Findings	Other
Findings notes	Correlations were different between groups in the R ATL, but the correlation is not reported as significant in the temporo-parietal group alone.

Complex analysis 4

First level contrast	Listening to normal sentences and making a plausibility judgment (paradigm 1) or listening to normal sentences (paradigm 2) vs listening to reversed speech
Analysis class	Longitudinal between two groups with aphasia
Group(s)	(Aphasia frontal (n = 17) T2 vs T1) vs (aphasia temporo-parietal (n = 17) T2 vs T1)
Covariate	—
Is the second level contrast valid in terms of the group(s), time point(s), and measures involved?	Yes
Is accuracy matched across the second level	Unknown, not reported

contrast?	
Is reaction time matched across the second level contrast?	Unknown, not reported
Behavioral data notes	No differences in proportion of expected button presses by group or time, but behavioral data pooled across conditions
Type of analysis	Complex
Statistical details	Correlations between changes in activity in 15 ROIs and changes in LRScomp were compared between patients with frontal and temporal lesions, using interaction terms as well as the Fisher r-to-z transformation. There was no correction for multiple comparisons across the 15 ROIs.
Findings	Other
Findings notes	In the L insula, the temporo-parietal group showed a stronger correlation than the frontal group between changes in activation and changes in LRScomp.

Complex analysis 5

First level contrast	Listening to normal sentences and making a plausibility judgment (paradigm 1) or listening to normal sentences (paradigm 2) vs listening to reversed speech
Analysis class	Longitudinal between two groups with aphasia
Group(s)	(Aphasia frontal (n = 17) T3 vs T1) vs (temporo-parietal (n = 17) T3 vs T1)
Covariate	—
Is the second level contrast valid in terms of the group(s), time point(s), and measures involved?	Yes
Is accuracy matched across the second level contrast?	Unknown, not reported
Is reaction time matched across the second level contrast?	Unknown, not reported
Behavioral data notes	No differences in proportion of expected button presses by group or time, but behavioral data pooled across conditions
Type of analysis	Complex
Statistical details	Correlations between changes in activity in 15 ROIs and changes in LRScomp were compared between patients with frontal and temporal lesions, using interaction terms as well as the Fisher r-to-z transformation. There was no correction for multiple comparisons across the 15 ROIs.
Findings	Other
Findings notes	In the L insula, the temporo-parietal group showed a stronger correlation than the frontal group between changes in activation and changes in LRScomp.

Complex analysis 6

First level contrast	Listening to normal sentences and making a plausibility judgment (paradigm 1) or listening to normal sentences (paradigm 2) vs listening to reversed speech
Analysis class	Longitudinal between two groups with aphasia
Group(s)	(Aphasia frontal (n = 17) T3 vs T2) vs (temporo-parietal (n = 17) T3 vs T2)
Covariate	—
Is the second level contrast valid in terms of the group(s), time point(s), and measures involved?	Yes
Is accuracy matched across the second level contrast?	Unknown, not reported
Is reaction time matched across the second level contrast?	Unknown, not reported
Behavioral data notes	No differences in proportion of expected button presses by group or time, but behavioral data pooled across conditions
Type of analysis	Complex
Statistical details	Correlations between changes in activity in 15 ROIs and changes in LRScomp were compared between patients with frontal and temporal lesions, using interaction terms as well as the Fisher r-to-z transformation. There was no correction for multiple comparisons across the 15 ROIs.
Findings	None

Findings notes	—

Complex analysis 7

First level contrast	Listening to normal sentences and making a plausibility judgment (paradigm 1) or listening to normal sentences (paradigm 2) vs listening to reversed speech
Analysis class	Cross-sectional between two groups with aphasia
Group(s)	Aphasia frontal T1 (n = 17) vs temporo-parietal T1 (n = 17)
Covariate	—
Is the second level contrast valid in terms of the group(s), time point(s), and measures involved?	Yes
Is accuracy matched across the second level contrast?	Unknown, not reported
Is reaction time matched across the second level contrast?	Unknown, not reported
Behavioral data notes	No differences in proportion of expected button presses by group or time, but behavioral data pooled across conditions
Type of analysis	Complex
Statistical details	Correlations between activity in 15 ROIs and lesion extent were compared between patients with frontal and temporal lesions. There was no correction for multiple comparisons across the 15 ROIs.
Findings	None
Findings notes	—

Complex analysis 8

First level contrast	Listening to normal sentences and making a plausibility judgment (paradigm 1) or listening to normal sentences (paradigm 2) vs listening to reversed speech
Analysis class	Cross-sectional between two groups with aphasia
Group(s)	Aphasia frontal T2 (n = 17) vs temporo-parietal T2 (n = 17)
Covariate	—
Is the second level contrast valid in terms of the group(s), time point(s), and measures involved?	Yes
Is accuracy matched across the second level contrast?	Unknown, not reported
Is reaction time matched across the second level contrast?	Unknown, not reported
Behavioral data notes	No differences in proportion of expected button presses by group or time, but behavioral data pooled across conditions
Type of analysis	Complex
Statistical details	Correlations between activity in 15 ROIs and lesion extent were compared between patients with frontal and temporal lesions. There was no correction for multiple comparisons across the 15 ROIs.
Findings	None
Findings notes	—

Complex analysis 9

First level contrast	Listening to normal sentences and making a plausibility judgment (paradigm 1) or listening to normal sentences (paradigm 2) vs listening to reversed speech
Analysis class	Cross-sectional between two groups with aphasia
Group(s)	Aphasia frontal T3 (n = 17) vs temporo-parietal T3 (n = 17)
Covariate	—
Is the second level contrast valid in terms of the group(s), time point(s), and measures involved?	Yes
Is accuracy matched across the second level contrast?	Unknown, not reported
Is reaction time matched across the second level contrast?	Unknown, not reported
Behavioral data notes	No differences in proportion of expected button presses by group or time, but behavioral

	data pooled across conditions
Type of analysis	Complex
Statistical details	Correlations between activity in 15 ROIs and lesion extent were compared between patients with frontal and temporal lesions. There was no correction for multiple comparisons across the 15 ROIs.
Findings	None
Findings notes	—

Complex analysis 10

First level contrast	Listening to normal sentences and making a plausibility judgment (paradigm 1) or listening to normal sentences (paradigm 2) vs listening to reversed speech
Analysis class	Longitudinal between two groups with aphasia
Group(s)	(Aphasia frontal (n = 17) T2 vs T1) vs (temporo-parietal (n = 17) T2 vs T1)
Covariate	—
Is the second level contrast valid in terms of the group(s), time point(s), and measures involved?	Yes
Is accuracy matched across the second level contrast?	Unknown, not reported
Is reaction time matched across the second level contrast?	Unknown, not reported
Behavioral data notes	No differences in proportion of expected button presses by group or time, but behavioral data pooled across conditions
Type of analysis	Complex
Statistical details	Correlations between changes in activity in 15 ROIs and lesion extent were compared between patients with frontal and temporal lesions. There was no correction for multiple comparisons across the 15 ROIs.
Findings	None
Findings notes	—

Complex analysis 11

First level contrast	Listening to normal sentences and making a plausibility judgment (paradigm 1) or listening to normal sentences (paradigm 2) vs listening to reversed speech
Analysis class	Longitudinal between two groups with aphasia
Group(s)	(Aphasia frontal (n = 17) T3 vs T1) vs (temporo-parietal (n = 17) T3 vs T1)
Covariate	—
Is the second level contrast valid in terms of the group(s), time point(s), and measures involved?	Yes
Is accuracy matched across the second level contrast?	Unknown, not reported
Is reaction time matched across the second level contrast?	Unknown, not reported
Behavioral data notes	No differences in proportion of expected button presses by group or time, but behavioral data pooled across conditions
Type of analysis	Complex
Statistical details	Correlations between changes in activity in 15 ROIs and lesion extent were compared between patients with frontal and temporal lesions. There was no correction for multiple comparisons across the 15 ROIs.
Findings	None
Findings notes	—

Complex analysis 12

First level contrast	Listening to normal sentences and making a plausibility judgment (paradigm 1) or listening to normal sentences (paradigm 2) vs listening to reversed speech
Analysis class	Longitudinal between two groups with aphasia
Group(s)	(Aphasia frontal (n = 17) T3 vs T2) vs (temporo-parietal (n = 17) T3 vs T2)
Covariate	—

Is the second level contrast valid in terms of the group(s), time point(s), and measures involved?	Yes
Is accuracy matched across the second level contrast?	Unknown, not reported
Is reaction time matched across the second level contrast?	Unknown, not reported
Behavioral data notes	No differences in proportion of expected button presses by group or time, but behavioral data pooled across conditions
Type of analysis	Complex
Statistical details	Correlations between changes in activity in 15 ROIs and lesion extent were compared between patients with frontal and temporal lesions. There was no correction for multiple comparisons across the 15 ROIs.
Findings	None
Findings notes	—

Complex analysis 13

First level contrast	Listening to normal sentences and making a plausibility judgment (paradigm 1) or listening to normal sentences (paradigm 2) vs listening to reversed speech
Analysis class	Cross-sectional correlation with language or other measure
Group(s)	Aphasia T1
Covariate	Interaction of comprehension composite by lesion size
Is the second level contrast valid in terms of the group(s), time point(s), and measures involved?	Yes
Is accuracy matched across the second level contrast?	Unknown, not reported
Is reaction time matched across the second level contrast?	Unknown, not reported
Behavioral data notes	No differences in proportion of expected button presses by group or time, but behavioral data pooled across conditions
Type of analysis	Complex
Statistical details	To investigate why some activation-behavior relationships did not remain significant when lesion extent was included as a covariate, models were constructed looking at the relationship between activation and behavior in patients with larger and smaller lesions.
Findings	Other
Findings notes	The three regions where this applied at T1, namely perilesional cortex, L IFG op, and L IFG orb, all showed positive correlations between activation and LRScomp in patients with larger lesions, but no correlations in patients with smaller lesions.

Complex analysis 14

First level contrast	Listening to normal sentences and making a plausibility judgment (paradigm 1) or listening to normal sentences (paradigm 2) vs listening to reversed speech
Analysis class	Longitudinal correlation with language or other measure
Group(s)	Aphasia T2 vs T1
Covariate	Interaction of Δ comprehension composite by lesion size
Is the second level contrast valid in terms of the group(s), time point(s), and measures involved?	Yes
Is accuracy matched across the second level contrast?	Unknown, not reported
Is reaction time matched across the second level contrast?	Unknown, not reported
Behavioral data notes	No differences in proportion of expected button presses by group or time, but behavioral data pooled across conditions
Type of analysis	Complex
Statistical details	To investigate why some activation-behavior relationships did not remain significant when lesion extent was included as a covariate, models were constructed looking at the relationship between activation and behavior in patients with larger and smaller lesions.
Findings	Other

Findings notes	This applied to the R DLPFC in the T2 vs T1 analysis. This region showed a positive correlation between activation and LRScomp in patients with larger lesions, but no correlation in patients with smaller lesions.

Notes

Excluded analyses	ROI analyses 27-32 and 45-50 were carried out with and without lesion extent as a covariate, but are coded only once, with notes as to which regions did not remain significant when the covariate was included
